# Supplementary material for: Prediction of Influenza Complications: Development and Validation of a Machine Learning Prediction Model to Improve and Expand the Identification of Vaccine-Hesitant Patients at Risk of Severe Influenza Complications
Source: J Clin Med. 2022 Jul 26;11(15):4342. doi: 10.3390/jcm11154342 (PMC9332321; doi:10.3390/jcm11154342)
Supplement: Supplementary file 1 [file jcm-11-04342-s001.zip › jcm-1700795-supplementary.pdf]

Supplementary Table S1

Time windows	Data sources	Aggregation functions
Last 3 years	All numeric lab data sources (not PCR or Urine which has categorical results) + vital signs + BMI + Weight	first value, last value, minimal value, maximal value , std of the values, average value
Between 3 years to 5 years	All numeric lab data sources (not PCR or Urine which has categorical results) + vital signs + BMI + Weight	first value, last value, minimal value, maximal value , std of the values, average value
Last 3 years	All numeric lab data sources (not PCR or Urine which has categorical results) + vital signs + BMI + Weight	slope - the slope of fitted linear line on the historical values
Last year	Influenza diagnosis, flu vaccination, complication event	number of events
Between 1 year to 3 years	Influenza diagnosis, flu vaccination, complication event	number of events
Last year	Categorical laboratory test: Flu RT-PCR and antigen tests, Urine tests	first value, last value, minimal value, maximal value
Between 1 year to 3 years	Categorical laboratory test: Flu RT-PCR and antigen tests, Urine tests	first value, last value, minimal value, maximal value
Between 3 years to 5 years	Categorical laboratory test: Flu RT-PCR and Antigentests, Urine tests	first value, last value, minimal value, maximal value
All history	Smoking	smoking years, pack years, time since quit smoking, smoking inteseting, current smoker?
No time	Race, Ethnicity, Age, Gender	
All history	Diabetes, Asthma, 4 levels of Asthma by severity, Chronic hear disease, COPD, Cancer, Immunosuppression, Chronic_Kidney, Chronic_Liver, Neurological_Diseas	number of events
Last year	Pregnancy	Boolean
Last 3 years	Immunosuppression_Drugs, BloodPressureDrugs, Asthma_Drugs, Asthma_Severe_Drugs, positive_pcr_flu_test	number of events
Last year	Admission length, membership coverage	number of days in this time window
Last 3 years	Admission length, membership coverage	number of days in this time window
Last 5 years	Admission length, membership coverage	number of days in this time window
Between 1 year to 3 years	Admission length, membership coverage	number of days in this time window
Between 3 years to 5 years	Admission length, membership coverage	number of days in this time window
Last year	Diagnosis ICD10 codes, Medication - ATC codes	number of events
Last 5 years	Diagnosis ICD10 codes, Medication - ATC codes	number of events
Last year	Admission type, outpatient visit type, hospital transfer department, billing code, procedure, admission reason	number of events
Last 5 years	Admission type, outpatient visit type, hospital transfer department, billing code, procedure, admission reason	number of events

Numeric Laboratory data sources + vital signs	Categorical laboratory data sources
Weight	Ethanol
SpO2	Microcytosis
Temperature	Tobramycin
Acetaminophen	Urine_Bacteria
Albumin	Urine_Bilirubin
ALKP	Urine_Blood
ALT	Urine_Glucose
Amylase	Urine_Ketone
aPTT	Urine_Leukocyte_Esterase
AST	Urine_Nitrite
B12	Urine_RBC
Bands#	Urine_Total_Protein
Bands%	Urine_Urobilinogen
Base_excess	Urine_WBC
Basophils#	Vancomycin
Basophils%	
Bicarbonate	
Bilirubin	
Bilirubin_Direct	
BLASTS	
Blasts%	
BNP	
BUN	
C3	
C4	
Ca	
CA125	
CA199	
CEA	
Chloride	
Cholesterol	
Cholesterol_over_HDL	

CK
CK_MB_INDEX
Cl
CO2
Cortisol
Creatine_Kinase_MB
Creatinine
CRP
D_DIMER
Digoxin
eGFR
Eosinophils#
Eosinophils%
ESR
Ferritin
Fibrinogen
FiO2
freeCalcium
FreeT3
FreeT4
FSH
Gentamicin
GFR
GGT
Globulin
Glucose
Haptoglobin
HbA1C
HDL
Hematocrit
Hemoglobin
IgA
IgG
IgM
Immature_Granulocytes#
Immature_Granulocytes%
INR
Iron_Binding_Capacity
Iron_Fe
Lactate
LDH
LDL
Lipase
Lithium
LuteinisingHormone
Lymphocytes#
Lymphocytes%
MCH
MCHC
MCV
Metamyelocytes#
Metamyelocytes%
Mg
Monocytes#
Monocytes%
MPV
Myelocytes#
Myelocytes%
Na
Neutrophils#
Neutrophils%

NonHDLCholesterol
NRBC%
Osmolality
PCO2
pH
Phenytoin
Phosphore
Platelets
PO2
Potassium
Progesterone
Prolactin
Protein_Total
PSA
PTH
RBC
RDW
Reticulocytes#
Reticulocytes%
SaO2
SerumAnionGap
T4
Tacrolimus
Testosterone
Transferrin
Transferrin_Saturation_Index
Triglycerides
TroponinI
TroponinT
TSH
Uric_Acid
Urine_Creatinine
Urine_Microalbumin
Urine_Osmolality
Urine_PH
Urine_Protein_Over_Creatinine
Urine_Spec_Gravity
UrineAlbumin_over_Creatinine
VitaminD_25
WBC

Supplementary Table S2 (Registry 1, RT-PCR Results)

flu Lab value	MAP	Filter applied for only "POSITIVE" as a influenza diagnos
POSITIVE	FLU_LAB_RESULT::POSITIVE	
INFLUENZA A RNA DETECTED BY PCR (AMPLIFIED PROBE)	FLU_LAB_RESULT::POSITIVE	
INFLUENZA B RNA DETECTED BY PCR (AMPLIFIED PROBE)	FLU_LAB_RESULT::POSITIVE	
POSITIVE FOR INFLUENZA A VIRUS, SUBTYPE H3. TEST RESULTS REPORTED TO PA DEPT OF HEALTH.	FLU_LAB_RESULT::POSITIVE	
POSITIVE FOR INFLUENZA A VIRUS, SUBTYPE 2009 H1. TEST RESULTS REPORTED TO PA DEPT OF HEALTH.	FLU_LAB_RESULT::POSITIVE	
POSITIVE FOR INFLUENZA A VIRUS BY PCR (AMPLIFIED PROBE) TEST RESULTS REPORTED TO PA DEPT OF HEALTH.	FLU_LAB_RESULT::POSITIVE	
POSITIVE FOR INFLUENZA B VIRUS. TEST RESULTS REPORTED TO PA DEPT OF HEALTH.	FLU_LAB_RESULT::POSITIVE	
POSITIVE FOR INFLUENZA B VIRUS BY PCR (AMPLIFIED PROBE) TEST RESULTS REPORTED TO PA DEPT OF HEALTH.	FLU_LAB_RESULT::POSITIVE	
POSITIVE FOR INFLUENZA A VIRUS SUBTYPE H3. TEST RESULTS REPORTED TO PA DEPT OF HEALTH.	FLU_LAB_RESULT::POSITIVE	
POSITIVE FOR INFLUENZA A VIRUS, NON-TYPEABLE. TEST RESULTS REPORTED TO PA DEPT OF HEALTH.	FLU_LAB_RESULT::POSITIVE	
POSITIVE, NON SUBTYPEABLE	FLU_LAB_RESULT::POSITIVE	
pos	FLU_LAB_RESULT::POSITIVE	
POSITIVE FOR INFLUENZA A VIRUS, NON-SUBTYPEABLE.	FLU_LAB_RESULT::POSITIVE	
POSITIVE FOR INFLUENZA A VIRUS, NON-SUBTYPEABLE. TEST RESULTS REPORTED TO PA DEPT OF HEALTH.	FLU_LAB_RESULT::POSITIVE	
POSITIVE FOR INFLUENZA A VIRUS, SUBTYPE H3.	FLU_LAB_RESULT::POSITIVE	
POSITIVE FOR INFLUENZA A VIRUS, PROBABLE NOVEL H1N1 STRAIN TEST RESULTS REPORTED TO PA DEPT OF HEALTH.	FLU_LAB_RESULT::POSITIVE	
POSITIVE FOR INFLUENZA A VIRUS, SUBTYPE H3. Negative for all other analytes tested. This assay identifies the following analytes: Influenza	FLU_LAB_RESULT::POSITIVE	
POSITIVE FOR INFLUENZA A VIRUS, SUBTYPE 2009 H1.	FLU_LAB_RESULT::POSITIVE	
POSITIVE FOR INFLUENZA A VIRUS, SUBTYPE H3. TEST RESULTS REPORTED TO PA DEPT OF HEALTH. Negative for all other analytes tested. Th	FLU_LAB_RESULT::POSITIVE	
POSITIVE FOR INFLUENZA B VIRUS.	FLU_LAB_RESULT::POSITIVE	
POSITIVE FOR INFLUENZA A VIRUS, SUBTYPE H1. TEST RESULTS REPORTED TO PA DEPT OF HEALTH.	FLU_LAB_RESULT::POSITIVE	
POSITIVE FOR INFLUENZA A VIRUS SUBTYPE H3.	FLU_LAB_RESULT::POSITIVE	
RESULT=POSITIVE	FLU_LAB_RESULT::POSITIVE	
POSITIVE FOR INFLUENZA A VIRUS, SUBTYPE H3. Negative for all other analytes tested. This assay identifies the following analytes: Influenza	FLU_LAB_RESULT::POSITIVE	
POSITIVE FOR INFLUENZA A VIRUS, SUBTYPE H3. TEST RESULTS REPORTED TO PA DEPT OF HEALTH. Negative for all other analytes tested. Th	FLU_LAB_RESULT::POSITIVE	
POSITIVE FOR INFLUENZA B VIRUS. TEST RESULTS REPORTED TO PA DEPT OF HEALTH. Negative for all other analytes tested. This assay ident	FLU_LAB_RESULT::POSITIVE	
POSITIVE FOR INFLUENZA A VIRUS, SUBTYPE 2009 H1. Negative for all other analytes tested. This assay identifies the following analytes: Infl	FLU_LAB_RESULT::POSITIVE	
RESULT=POSITIVECOMMENT:This test can not distinguish infections caused by	FLU_LAB_RESULT::POSITIVE	
CORRECTED REPORT, PREVIOUSLY REPORTED AS POSITIVE FOR INFLUENZA A VIRUS, SUBTYPE H3.	FLU_LAB_RESULT::POSITIVE	
POSITIVE FOR INFLUENZA A VIRUS, SUBTYPE 2009 H1. TEST RESULTS REPORTED TO PA DEPT OF HEALTH. CORRECTED REPORT, PREVIOUSLY I	FLU_LAB_RESULT::POSITIVE	
CORRECTED REPORT, PREVIOUSLY REPORTED AS POSITIVE FOR INFLUENZA A VIRUS, SUBTYPE H1.	FLU_LAB_RESULT::POSITIVE	
INFLUENZA A RNA DETECTED BY PCR (AMPLIFIED PROBE) TEST RESULTS REPORTED TO PA DEPT OF HEALTH. CORRECTED REPORT, PREVIOUS	FLU_LAB_RESULT::POSITIVE	
POSITIVE FOR INFLUENZA B VIRUS BY PCR (AMPLIFIED PROBE) TEST RESULTS REPORTED TO PA DEPT OF HEALTH. CORRECTED REPORT, PREV	FLU_LAB_RESULT::POSITIVE	
postitive	FLU_LAB_RESULT::POSITIVE	
POSITIVE FOR INFLUENZA A VIRUS, NON-TYPEABLE.	FLU_LAB_RESULT::POSITIVE	
POSITIVE FOR INFLUENZA A VIRUS, SUBTYPE H3. EQUIVOCAL RESULTS FOR INFLUENZA A VIRUS.	FLU_LAB_RESULT::POSITIVE	
POSITIVE FOR INFLUENZA A VIRUS, SUBTYPE H3. Negative for all other analytes tested. This assay identifies the following analytes: Influenza	FLU_LAB_RESULT::POSITIVE	
POSITIVE FOR INFLUENZA B VIRUS. Negative for all other analytes tested. This assay identifies the following analytes: Influenza A virus (H1,H	FLU_LAB_RESULT::POSITIVE	
POSITIVE FOR INFLUENZA B VIRUS. TEST RESULTS REPORTED TO PA DEPT OF HEALTH. CORRECTED REPORT, PREVIOUSLY REPORTED AS POSI	FLU_LAB_RESULT::POSITIVE	
POSITIVE FOR INFLUENZA B VIRUS. TEST RESULTS REPORTED TO PA DEPT OF HEALTH. Negative for all other analytes tested. This assay ident	FLU_LAB_RESULT::POSITIVE	
POSITIVE FOR INFLUENZA A VIRUS, SUBTYPE 2009 H1. TEST RESULTS REPORTED TO PA DEPT OF HEALTH. CORRECTED REPORT, PREVIOUSLY I	FLU_LAB_RESULT::POSITIVE	
INFLUENZA VIRUS TYPE A ANTIGEN DETECTED	FLU_LAB_RESULT::POSITIVE	
CORRECTED REPORT, PREVIOUSLY REPORTED AS POSITIVE FOR INFLUENZA A VIRUS, NON-SUBTYPEABLE.	FLU_LAB_RESULT::POSITIVE	
+	FLU_LAB_RESULT::POSITIVE	
POSITIVE FOR INFLUENZA A VIRUS, SUBTYPE 2009 H1. CORRECTED REPORT, PREVIOUSLY REPORTED AS POSITIVE FOR INFLUENZA A VIRUS, S	FLU_LAB_RESULT::POSITIVE	
POSITIVE FOR INFLUENZA A VIRUS, NON-TYPEABLE. TEST RESULTS REPORTED TO PA DEPT OF HEALTH. CORRECTED REPORT, PREVIOUSLY RE	FLU_LAB_RESULT::POSITIVE	
POSITIVE FOR PARAINFLUENZA VIRUS TYPE 3. CORRECTED REPORT, PREVIOUSLY REPORTED AS POSITIVE FOR INFLUENZA A VIRUS, SUBTYPE	FLU_LAB_RESULT::POSITIVE	
CORRECTED REPORT, PREVIOUSLY REPORTED AS POSITIVE FOR INFLUENZA B VIRUS.	FLU_LAB_RESULT::POSITIVE	
CORRECTED REPORT, PREVIOUSLY REPORTED AS POSITIVE FOR INFLUENZA A VIRUS, SUBTYPE H1. TEST RESULTS REPORTED TO PA DEPT OF I	FLU_LAB_RESULT::POSITIVE	
POSITIVE FOR INFLUENZA B VIRUS BY PCR (AMPLIFIED PROBE) TEST RESULTS REPORTED TO PA DEPT OF HEALTH. The validation of this speci	FLU_LAB_RESULT::POSITIVE	
POSITIVE FOR INFLUENZA A VIRUS, PROBABLE NOVEL H1N1 STRAIN	FLU_LAB_RESULT::POSITIVE	
POSITIVE FOR INFLUENZA A VIRUS, SUBTYPE 2009 H1. TEST RESULTS REPORTED TO PA DEPT OF HEALTH. CORRECTED REPORT, PREVIOUSLY I	FLU_LAB_RESULT::POSITIVE	
positve	FLU_LAB_RESULT::POSITIVE	

Supplementary Table S2 (Registry 1, EDG Codes)
EDG:1005:OTHER_SPECIFIED_VIRAL_INFECTION_IN_CONDITIONS_CLASSIFIED_ELSEWHERE_AND_OF_UNSPECIFIED_SITE
EDG:1008:UNSPECIFIED_VIRAL_INFECTION_IN_CONDITIONS_CLASSIFIED_ELSEWHERE_AND_OF_UNSPECIFIED_SITE
EDG:102682:TRACHEITIS_ACUTE_WITH_OBSTRUCTION
EDG:102882:URTI_(ACUTE_UPPER_RESPIRATORY_INFECTION)
EDG:103525:ACUTE_INFECTIVE_RHINITIS
EDG:1039558:PNEUMONIA_INVOLVING_LEFT_LUNG_UNSPECIFIED_PART_OF_LUNG
EDG:1039644:PNEUMONIA_INVOLVING_RIGHT_LUNG_UNSPECIFIED_PART_OF_LUNG
EDG:1039729:ACUTE_BRONCHITIS_UNSPECIFIED_ORGANISM
EDG:104630:BILATERAL_BRONCHOPNEUMONIA
EDG:105768:COLD_VIRUS
EDG:1090222:PNEUMONIA_UNSPECIFIED_LATERALITY_UNSPECIFIED_PART_OF_LUNG
EDG:1090236:PNEUMONIA_OF_LOWER_LOBE_OF_LUNG_UNSPECIFIED_LATERALITY
EDG:1097529:FEVER_UNSPECIFIED_FEVER_CAUSE
EDG:1097628:ACUTE_PHARYNGITIS_UNSPECIFIED_PHARYNGITIS_TYPE
EDG:1098093:VIRAL_INFECTION_IN_CONDITIONS_CLASSIFIED_ELSEWHERE_AND_OF_UNSPECIFIED_SITE
EDG:110069:INFECTION_OF_THE_UPPER_RESPIRATORY_TRACT
EDG:110153:INFLUENZA_WITH_BRONCHOPNEUMONIA
EDG:110154:INFLUENZA_WITH_GASTROINTESTINAL_TRACT_INVOLVEMENT
EDG:110156:INFLUENZA_WITH_PHARYNGITIS
EDG:110157:INFLUENZA_BRONCHOPNEUMONIA
EDG:1101940:COPD_WITH_ACUTE_LOWER_RESPIRATORY_INFECTION_(HCC)
EDG:1102104:FEVER_DUE_TO_UNSPECIFIED_CONDITION
EDG:1107855:PNEUMONIA_OF_BOTH_LOWER_LOBES_(HCC)
EDG:1107857:PNEUMONIA_OF_BOTH_UPPER_LOBES_DUE_TO_INFLUENZA_A_VIRUS
EDG:1107869:PNEUMONIA_OF_LOWER_LOBE_DUE_TO_INFECTIOUS_ORGANISM_(HCC)
EDG:1107895:PNEUMONIA_OF_RIGHT_UPPER_LOBE_DUE_TO_INFECTIOUS_ORGANISM_(HCC)
EDG:1107903:PNEUMONIA_OF_LEFT_UPPER_LOBE_DUE_TO_INFLUENZA_A_VIRUS
EDG:1107925:PNEUMONIA_OF_LEFT_LOWER_LOBE_DUE_TO_INFECTIOUS_ORGANISM_(HCC)
EDG:1107926:PNEUMONIA_OF_RIGHT_MIDDLE_LOBE_DUE_TO_INFLUENZA_A_VIRUS
EDG:1107933:PNEUMONIA_OF_LEFT_LOWER_LOBE_DUE_TO_INFLUENZA_A_VIRUS
EDG:1107937:PNEUMONIA_OF_BOTH_LUNGS_DUE_TO_INFECTIOUS_ORGANISM
EDG:1107944:PNEUMONIA_OF_BOTH_LOWER_LOBES_DUE_TO_INFECTIOUS_ORGANISM_(HCC)
EDG:1107950:PNEUMONIA_OF_RIGHT_MIDDLE_LOBE_DUE_TO_INFECTIOUS_ORGANISM_(HCC)
EDG:1107973:PNEUMONIA_OF_RIGHT_LUNG_DUE_TO_INFECTIOUS_ORGANISM
EDG:1107988:PNEUMONIA_OF_RIGHT_LOWER_LOBE_DUE_TO_INFLUENZA_A_VIRUS
EDG:1107993:PNEUMONIA_OF_RIGHT_LOWER_LOBE_DUE_TO_INFECTIOUS_ORGANISM_(HCC)
EDG:1107994:PNEUMONIA_OF_BOTH_UPPER_LOBES_DUE_TO_INFECTIOUS_ORGANISM_(HCC)
EDG:1108022:PNEUMONIA_OF_BOTH_LOWER_LOBES_DUE_TO_INFLUENZA_A_VIRUS
EDG:1108023:PNEUMONIA_OF_LEFT_UPPER_LOBE_DUE_TO_INFECTIOUS_ORGANISM_(HCC)
EDG:110803:LIP_(LYMPHOID_INTERSTITIAL_PNEUMONITIS)_(HCC)
EDG:110924:LOWER_RESP._TRACT_INFECTION
EDG:110925:LOWER_RESPIRATORY_TRACT_INFECTIOUS_DISEASE
EDG:110929:LRTI_(LOWER_RESPIRATORY_TRACT_INFECTION)
EDG:111003:LYMPHOID_INTERSTITIAL_PNEUMONITIS_(HCC)
EDG:1111201:ACUTE_TRACHEITIS_WITH_AIRWAY_OBSTRUCTION
EDG:1111229:SUPRAGLOTTITIS_WITHOUT_AIRWAY_OBSTRUCTION
EDG:1111238:ACUTE_TRACHEITIS_WITHOUT_AIRWAY_OBSTRUCTION
EDG:1111267:ACUTE_PHARYNGITIS_DUE_TO_INFECTIOUS_MONONUCLEOSIS
EDG:1113767:INFLAMMATION_OF_LUNG
EDG:1114047:ACUTE_RESPIRATORY_INFECTION_DUE_TO_INFLUENZA_A_SUBTYPE_H1N1_VIRUS
EDG:1114342:OTITIS_MEDIA_DUE_TO_INFLUENZA
EDG:1114377:OTITIS_MEDIA_DUE_TO_INFLUENZA_A_VIRUS
EDG:1114529:UPPER_RESPIRATORY_TRACT_INFECTION_DUE_TO_INFLUENZA
EDG:1114593:BRONCHOPNEUMONIA_DUE_TO_VIRUS
EDG:1114806:INTERSTITIAL_LUNG_DISEASE_DUE_TO_CONNECTIVE_TISSUE_DISEASE_(HCC)
EDG:1114809:UPPER_RESPIRATORY_TRACT_INFECTION_DUE_TO_INFLUENZA_A_VIRUS
EDG:1114973:PNEUMONIA_DUE_TO_INFLUENZA
EDG:1115007:BRONCHIOLITIS_DUE_TO_INFLUENZA_VIRUS
EDG:111838:NASOPHARYNGITIS_ACUTE
EDG:111839:NASOPHARYNGITIS_INFECTIVE
EDG:1123998:URI_DUE_TO_INFLUENZA_A_VIRUS
EDG:1124154:INFLUENZA_DUE_TO_AVIAN_INFLUENZA_A_VIRUS_SUBTYPE_H3N2
EDG:1124947:INFLUENZA_WITH_RESPIRATORY_SYMPTOMS
EDG:1127838:PHARYNGITIS_DUE_TO_INFECTIOUS_MONONUCLEOSIS
EDG:1132717:PAIN_AGGRAVATED_BY_COUGHING
EDG:1134919:PNEUMONIA_DUE_TO_INFECTIOUS_ORGANISM
EDG:1135225:INFLUENZA_WITH_RESPIRATORY_MANIFESTATION
EDG:1137446:VIRAL_INFECTION_UNSPECIFIED
EDG:1137623:ACUTE_PHARYNGITIS_UNSPECIFIED
EDG:1137741:ACUTE_BRONCHITIS_UNSPECIFIED
EDG:1137823:INTERSTITIAL_PULMONARY_DISEASE_UNSPECIFIED_(HCC)
EDG:1137866:ACUTE_NASOPHARYNGITIS_(COMMON_COLD)
EDG:1137935:ACUTE_UPPER_RESPIRATORY_INFECTION_UNSPECIFIED
EDG:114086:RTI_(RESPIRATORY_TRACT_INFECTION)
EDG:1141237:DISEASES_OF_THE_RESPIRATORY_SYSTEM_COMPLICATING_PREGNANCY_SECOND_TRIMESTER
EDG:115877:UPPER_RESPIRATORY_TRACT_INFECTION
EDG:115929:URTI_(INFECTION_OF_THE_UPPER_RESPIRATORY_TRACT)
EDG:1176858:BRONCHOPNEUMONIA_UNSPECIFIED_ORGANISM
EDG:1176952:UNSPECIFIED_ACUTE_LOWER_RESPIRATORY_INFECTION
EDG:1176981:OTHER_VIRAL_INFECTIONS_OF_UNSPECIFIED_SITE

Supplementary Table S2 (Registry 2, ICD Codes) ICD10_CODE:J020
ICD10_CODE:J028
ICD10_CODE:J06
ICD10_CODE:J09
ICD10_CODE:J10
ICD10_CODE:J11
ICD10_CODE:J129
ICD10_CODE:J180
ICD10_CODE:J181
ICD10_CODE:J188
ICD10_CODE:J209
ICD9_CODE:487
ICD9_CODE:488
ICD9_CODE:51632
ICD9_CODE:51633
ICD9_CODE:51634

Supplementary Table S2 (Registry 2, Prescriptions)

Oseltamivir_30_MG_Oral_Capsule
Oseltamivir_30_MG_Oral_Capsule_[Tamiflu]
Oseltamivir_45_MG_Oral_Capsule
Oseltamivir_45_MG_Oral_Capsule_[Tamiflu]
Oseltamivir_6_MG/ML_Oral_Suspension
Oseltamivir_6_MG/ML_Oral_Suspension_[Tamiflu]
Oseltamivir_75_MG_Oral_Capsule
Oseltamivir_75_MG_Oral_Capsule_[Tamiflu]
MED51527:TAMIFLU_30_MG_PO_CAPS
MED65577:OSELTAMIVIR_PHOSPHATE_30_MG_PO_CAPS
MED58484:OSELTAMIVIR_PHOSPHATE_45_MG_PO_CAPS
MED52536:TAMIFLU_45_MG_PO_CAPS
MED33769:OSELTAMIVIR_PHOSPHATE_6_MG/ML_PO_SUSR
MED60604:TAMIFLU_6_MG/ML_PO_SUSR
MED55342:OSELTAMIVIR_PHOSPHATE_75_MG_PO_CAPS
MED16824:TAMIFLU_75_MG_PO_CAPS

Supplementary Table S3 (ICD EDG and CPT codes defined)

Chart category	2021 ICD-10-CM Codes	DX_ID	DX_LEXICAL_NM	CODE_SYSTEM	MAP_PRIORITY	CS_CD	POST_COOR_EXP	CONCEPT_NM	AUTHOR	STEWARD	CAT_TYPE	REC_STATE	CLINICALI	EFF_DT	EXP_DT	MAP_DT
Diagnosis: ICD10 Code, F00-F99 Define	F01-F99 Mental, Behavioral and Neurodevelopmental disorders	963754	CADASIL dementia, without behavioral disturbance	ICD10CM	1	F01.50		Vascular dementia without behavioral disturbance	IMO	GHS-PACDC	IMO_2015_R	ACTIVE	N	1900-01-01	9999-12-31	2015-07-10
Diagnosis: ICD10 Code, F00-F99 Define	F01-F99 Mental, Behavioral and Neurodevelopmental disorders	1280874	Severe possible major vascular neurocognitive disorder	ICD10CM	1	F01.50		Vascular dementia without behavioral disturbance	IMO	GHS-PACDC	IMO_2017_R	ACTIVE	N	1900-01-01	9999-12-31	2016-09-30
Diagnosis: ICD10 Code, F00-F99 Define	F01-F99 Mental, Behavioral and Neurodevelopmental disorders	1274196	Multi-infarct dementia due to atherosclerosis (HCC)	ICD10CM	1	F01.50	F01.50,I70.209	Vascular dementia without behavioral disturbance	IMO	GHS-PACDC	IMO_2017_R	ACTIVE	N	1900-01-01	9999-12-31	2016-09-30
Diagnosis: ICD10 Code, F00-F99 Define	F01-F99 Mental, Behavioral and Neurodevelopmental disorders	162601	Vascular dementia of acute onset	ICD10CM	1	F01.50		Vascular dementia without behavioral disturbance	IMO	GHS-PACDC	DX_LEGACY	ACTIVE	N	1900-01-01	9999-12-31	2015-07-10
Diagnosis: ICD10 Code, F00-F99 Define	F01-F99 Mental, Behavioral and Neurodevelopmental disorders	30053	Vascular dementia	ICD10CM	1	F01.50		Vascular dementia without behavioral disturbance	IMO	GHS-PACDC	DX_LEGACY	ACTIVE	N	1900-01-01	9999-12-31	2015-07-10
Diagnosis: ICD10 Code, F00-F99 Define	F01-F99 Mental, Behavioral and Neurodevelopmental disorders	262949	Dementia in human immunodeficiency virus (HIV) disease (HCC)	ICD10CM	2	F02.80	B20,F02.80	Dementia in other diseases classified elsewhere without	IMO	GHS-PACDC	DX_LEGACY	ACTIVE	N	2016-04-10	9999-12-31	2015-07-10
Diagnosis: ICD10 Code, F00-F99 Define	F01-F99 Mental, Behavioral and Neurodevelopmental disorders	1091190	Mild major neurocognitive disorder as late effect of traumatic brain injury with behavioral distu	ICD10CM	3	F02.80	S06.9X0S,F02.81,F02.80	Dementia in other diseases classified elsewhere without	IMO	GHS-PACDC	IMO_2015_R	DELETED	Y	1900-01-01	2017-10-14	2015-07-10
Diagnosis: ICD10 Code, F00-F99 Define	F01-F99 Mental, Behavioral and Neurodevelopmental disorders	375644	Dementia due to Parkinson's disease without behavioral disturbance (HCC)	ICD10CM	2	F02.80	G20,F02.80	Dementia in other diseases classified elsewhere without	IMO	GHS-PACDC	DX_LEGACY	ACTIVE	N	1900-01-01	9999-12-31	2015-07-10
Diagnosis: ICD10 Code, F00-F99 Define	F01-F99 Mental, Behavioral and Neurodevelopmental disorders	1200706	Major neurocognitive disorder due to traumatic brain injury without behavioral disturbance, wi	ICD10CM	2	F02.80	S06.9X0A,F02.80	Dementia in other diseases classified elsewhere without	IMO	GHS-PACDC	IMO_2016_R	DELETED	Y	1900-01-01	2016-10-14	2016-06-02
Diagnosis: ICD10 Code, F00-F99 Define	F01-F99 Mental, Behavioral and Neurodevelopmental disorders	611288	Dementia in other diseases classified elsewhere without behavioral disturbance	ICD10CM	1	F02.80		Dementia in other diseases classified elsewhere without	IMO	GHS-PACDC	IMO_2015_R	ACTIVE	Y	1900-01-01	9999-12-31	2015-07-10
Diagnosis: ICD10 Code, F00-F99 Define	F01-F99 Mental, Behavioral and Neurodevelopmental disorders	486681	Severe major neurocognitive disorder due to prion disease without behavioral disturbance	ICD10CM	2	F02.80	A81.9,F02.80	Dementia in other diseases classified elsewhere without	IMO	GHS-PACDC	IMO_2015_R	ACTIVE	N	1900-01-01	9999-12-31	2015-07-10
Diagnosis: ICD10 Code, F00-F99 Define	F01-F99 Mental, Behavioral and Neurodevelopmental disorders	190878	Psychosis associated with new variant Jakob-Creutzfeldt disease	ICD10CM	2	F02.80	A81.01,F02.80	Dementia in other diseases classified elsewhere without	IMO	GHS-PACDC	DX_LEGACY	ACTIVE	N	1900-01-01	9999-12-31	2015-07-10
Diagnosis: ICD10 Code, F00-F99 Define	F01-F99 Mental, Behavioral and Neurodevelopmental disorders	1205897	Major neurocognitive disorder due to traumatic brain injury without behavioral disturbance, wi	ICD10CM	2	F02.80	S06.9X4S,F02.80	Dementia in other diseases classified elsewhere without	IMO	GHS-PACDC	IMO_2016_R	DELETED	Y	1900-01-01	2016-10-14	2016-06-02
Diagnosis: ICD10 Code, F00-F99 Define	F01-F99 Mental, Behavioral and Neurodevelopmental disorders	1201778	Major neurocognitive disorder due to traumatic brain injury, without loss of consciousness, sub	ICD10CM	2	F02.80	S06.9X0D,F02.80	Dementia in other diseases classified elsewhere without	IMO	GHS-PACDC	IMO_2016_R	DELETED	Y	1900-01-01	2016-10-14	2016-06-02
Diagnosis: ICD10 Code, F00-F99 Define	F01-F99 Mental, Behavioral and Neurodevelopmental disorders	147964	Dementing neurological disease or syndrome	ICD10CM	2	F02.80	G98.8,F02.80	Dementia in other diseases classified elsewhere without	IMO	GHS-PACDC	DX_LEGACY	ACTIVE	N	1900-01-01	9999-12-31	2015-07-10
Diagnosis: ICD10 Code, F00-F99 Define	F01-F99 Mental, Behavioral and Neurodevelopmental disorders	185696	Dementia due to Jakob's disease without behavioral disturbance	ICD10CM	2	F02.80	A81.00,F02.80	Dementia in other diseases classified elsewhere without	IMO	GHS-PACDC	DX_LEGACY	ACTIVE	N	1900-01-01	9999-12-31	2015-07-10
Diagnosis: ICD10 Code, F00-F99 Define	F01-F99 Mental, Behavioral and Neurodevelopmental disorders	190862	Psychosis due to Creutzfeldt-Jakob disease, no behavioral disturbance	ICD10CM	2	F02.80	A81.00,F02.80	Dementia in other diseases classified elsewhere without	IMO	GHS-PACDC	DX_LEGACY	ACTIVE	N	1900-01-01	9999-12-31	2015-07-10
Diagnosis: ICD10 Code, F00-F99 Define	F01-F99 Mental, Behavioral and Neurodevelopmental disorders	137055	Semantic dementia	ICD10CM	2	F02.80	G31.09,F02.80	Dementia in other diseases classified elsewhere without	IMO	GHS-PACDC	DX_LEGACY	ACTIVE	N	2017-03-31	9999-12-31	2017-04-14
Diagnosis: ICD10 Code, F00-F99 Define	F01-F99 Mental, Behavioral and Neurodevelopmental disorders	1101866	Alzheimer's disease with Pick's disease	ICD10CM	3	F02.80	G30.9,G31.01,F02.80	Dementia in other diseases classified elsewhere without	IMO	GHS-PACDC	IMO_2016_R	ACTIVE	N	2016-09-17	9999-12-31	2016-09-30
Diagnosis: ICD10 Code, F00-F99 Define	F01-F99 Mental, Behavioral and Neurodevelopmental disorders	429039	Mild frontotemporal neurocognitive disorder	ICD10CM	2	F02.80	G31.09,F02.80	Dementia in other diseases classified elsewhere without	IMO	GHS-PACDC	IMO_2015_R	ACTIVE	N	2016-09-17	9999-12-31	2016-09-30
Diagnosis: ICD10 Code, F00-F99 Define	F01-F99 Mental, Behavioral and Neurodevelopmental disorders	429339	Major neurocognitive disorder due to Parkinson's disease, possible (HCC)	ICD10CM	2	F02.80	G20,F02.80	Dementia in other diseases classified elsewhere without	IMO	GHS-PACDC	IMO_2015_R	ACTIVE	N	2016-09-17	9999-12-31	2016-09-30
Diagnosis: ICD10 Code, F00-F99 Define	F01-F99 Mental, Behavioral and Neurodevelopmental disorders	42582	DAT (dementia of Alzheimer type)	ICD10CM	2	F02.80	G30.9,F02.80	Dementia in other diseases classified elsewhere without	IMO	GHS-PACDC	DX_LEGACY	ACTIVE	N	1900-01-01	2016-05-07	2015-07-10
Diagnosis: ICD10 Code, F00-F99 Define	F01-F99 Mental, Behavioral and Neurodevelopmental disorders	715876	Dementia due to head trauma without behavioral disturbance, sequela	ICD10CM	2	F02.80	S09.90XS,F02.80,F02.81	Dementia in other diseases classified elsewhere without	IMO	GHS-PACDC	IMO_2015_R	ACTIVE	N	1900-01-01	2016-05-07	2015-07-10
Diagnosis: ICD10 Code, F00-F99 Define	F01-F99 Mental, Behavioral and Neurodevelopmental disorders	137066	Dementia in pick's disease	ICD10CM	2	F02.80	G31.01,F02.80	Dementia in other diseases classified elsewhere without	IMO	GHS-PACDC	DX_LEGACY	ACTIVE	N	2016-09-17	9999-12-31	2016-09-30
Diagnosis: ICD10 Code, F00-F99 Define	F01-F99 Mental, Behavioral and Neurodevelopmental disorders	716145	Dementia due to head trauma without behavioral disturbance, subsequent encounter	ICD10CM	2	F02.80	S09.90XD,F02.80,F02.81	Dementia in other diseases classified elsewhere without	IMO	GHS-PACDC	IMO_2015_R	ACTIVE	N	1900-01-01	2016-05-07	2015-07-10
Diagnosis: ICD10 Code, F00-F99 Define	F01-F99 Mental, Behavioral and Neurodevelopmental disorders	485837	Moderate major neurocognitive disorder due to multiple etiologies without behavioral disturba	ICD10CM	1	F02.80		Dementia in other diseases classified elsewhere without	IMO	GHS-PACDC	IMO_2015_R	ACTIVE	N	1900-01-01	2016-10-14	2015-07-10
Diagnosis: ICD10 Code, F00-F99 Define	F01-F99 Mental, Behavioral and Neurodevelopmental disorders	299875	Dementia due to medical condition	ICD10CM	1	F02.80		Dementia in other diseases classified elsewhere without	IMO	GHS-PACDC	DX_LEGACY	ACTIVE	N	1900-01-01	9999-12-31	2015-07-10
Diagnosis: ICD10 Code, F00-F99 Define	F01-F99 Mental, Behavioral and Neurodevelopmental disorders	486680	Moderate major neurocognitive disorder due to prion disease without behavioral disturbance	ICD10CM	2	F02.80	A81.9,F02.80	Dementia in other diseases classified elsewhere without	IMO	GHS-PACDC	IMO_2015_R	ACTIVE	N	2016-09-17	9999-12-31	2016-09-30
Diagnosis: ICD10 Code, F00-F99 Define	F01-F99 Mental, Behavioral and Neurodevelopmental disorders	262152	Frontal lobe dementia	ICD10CM	2	F02.80	G31.09,F02.80	Dementia in other diseases classified elsewhere without	IMO	GHS-PACDC	DX_LEGACY	ACTIVE	N	2016-04-10	9999-12-31	2015-07-10
Diagnosis: ICD10 Code, F00-F99 Define	F01-F99 Mental, Behavioral and Neurodevelopmental disorders	520661	Waisman syndrome	ICD10CM	2	F02.80	G31.83,F02.80	Dementia in other diseases classified elsewhere without	IMO	GHS-PACDC	IMO_2015_R	ACTIVE	N	1900-01-01	9999-12-31	2015-07-10
Diagnosis: ICD10 Code, F00-F99 Define	F01-F99 Mental, Behavioral and Neurodevelopmental disorders	203430	Dementia in Huntington's disease (HCC)	ICD10CM	2	F02.80	G10,F02.80	Dementia in other diseases classified elsewhere without	IMO	GHS-PACDC	DX_LEGACY	ACTIVE	N	2017-09-17	9999-12-31	2017-10-30
Diagnosis: ICD10 Code, F00-F99 Define	F01-F99 Mental, Behavioral and Neurodevelopmental disorders	314178	Parkinson's disease dementia (HCC)	ICD10CM	2	F02.80	G20,F02.80	Dementia in other diseases classified elsewhere without	IMO	GHS-PACDC	DX_LEGACY	ACTIVE	N	1900-01-01	9999-12-31	2015-07-10
Diagnosis: ICD10 Code, F00-F99 Define	F01-F99 Mental, Behavioral and Neurodevelopmental disorders	411766	Epileptic dementia (HCC)	ICD10CM	2	F02.80	G40.909,F02.80	Dementia in other diseases classified elsewhere without	IMO	GHS-PACDC	DX_LEGACY	ACTIVE	N	1900-01-01	9999-12-31	2015-07-10
Diagnosis: ICD10 Code, F00-F99 Define	F01-F99 Mental, Behavioral and Neurodevelopmental disorders	264359	AIDS with dementia (HCC)	ICD10CM	2	F02.80	B20,F02.80	Dementia in other diseases classified elsewhere without	IMO	GHS-PACDC	DX_LEGACY	ACTIVE	N	2016-04-10	9999-12-31	2015-07-10
Diagnosis: ICD10 Code, F00-F99 Define	F01-F99 Mental, Behavioral and Neurodevelopmental disorders	486993	Moderate major neurocognitive disorder due to another medical condition without behavioral	ICD10CM	1	F02.80		Dementia in other diseases classified elsewhere without	IMO	GHS-PACDC	IMO_2015_R	ACTIVE	N	1900-01-01	9999-12-31	2015-07-10
Diagnosis: ICD10 Code, F00-F99 Define	F01-F99 Mental, Behavioral and Neurodevelopmental disorders	313376	Dementia in conditions classified elsewhere with aggressive behavior	ICD10CM	1	F02.81		Dementia in other diseases classified elsewhere with be	IMO	GHS-PACDC	DX_LEGACY	ACTIVE	N	1900-01-01	9999-12-31	2015-07-10
Diagnosis: ICD10 Code, F00-F99 Define	F01-F99 Mental, Behavioral and Neurodevelopmental disorders	1209319	Alzheimer's disease of other onset with behavioral disturbance	ICD10CM	2	F02.81	G30.8,F02.81	Dementia in other diseases classified elsewhere with be	IMO	GHS-PACDC	IMO_2016_R	DELETED	Y	1900-01-01	9999-12-31	2016-06-02
Diagnosis: ICD10 Code, F00-F99 Define	F01-F99 Mental, Behavioral and Neurodevelopmental disorders	717815	Dementia due to head trauma with behavioral disturbance, initial encounter	ICD10CM	2	F02.81	S09.90XA,F02.81	Dementia in other diseases classified elsewhere with be	IMO	GHS-PACDC	IMO_2015_R	ACTIVE	N	2016-04-10	9999-12-31	2015-07-10
Diagnosis: ICD10 Code, F00-F99 Define	F01-F99 Mental, Behavioral and Neurodevelopmental disorders	488567	Major neurocognitive disorder, due to hiv infection, with behavioral disturbance, severe (HCC)	ICD10CM	2	F02.81	B20,F02.81	Dementia in other diseases classified elsewhere with be	IMO	GHS-PACDC	IMO_2015_R	ACTIVE	N	1900-01-01	9999-12-31	2015-07-10
Diagnosis: ICD10 Code, F00-F99 Define	F01-F99 Mental, Behavioral and Neurodevelopmental disorders	1096346	Major neurocognitive disorder due to traumatic brain injury with behavioral disturbance, initial	ICD10CM	2	F02.81	S06.9X9A,F02.81	Dementia in other diseases classified elsewhere with be	IMO	GHS-PACDC	IMO_2015_R	DELETED	Y	2017-09-17	9999-12-31	2017-10-30
Diagnosis: ICD10 Code, F00-F99 Define	F01-F99 Mental, Behavioral and Neurodevelopmental disorders	485972	Severe major neurocognitive disorder due to HIV infection with behavioral disturbance (HCC)	ICD10CM	2	F02.81	B20,F02.81	Dementia in other diseases classified elsewhere with be	IMO	GHS-PACDC	IMO_2015_R	ACTIVE	N	1900-01-01	9999-12-31	2015-07-10
Diagnosis: ICD10 Code, F00-F99 Define	F01-F99 Mental, Behavioral and Neurodevelopmental disorders	954195	Dementia in Alzheimer's disease with early onset, with behavioral disturbance	ICD10CM	1	F02.81		Dementia in other diseases classified elsewhere with be	IMO	GHS-PACDC	IMO_2015_R	DELETED	Y	1900-01-01	9999-12-31	2015-07-10
Diagnosis: ICD10 Code, F00-F99 Define	F01-F99 Mental, Behavioral and Neurodevelopmental disorders	717610	Dementia due to head trauma with behavioral disturbance, subsequent encounter	ICD10CM	2	F02.81	S09.90XD,F02.81	Dementia in other diseases classified elsewhere with be	IMO	GHS-PACDC	IMO_2015_R	ACTIVE	N	2016-04-10	9999-12-31	2015-07-10
Diagnosis: ICD10 Code, F00-F99 Define	F01-F99 Mental, Behavioral and Neurodevelopmental disorders	185693	Dementia due to Jakob-Creutzfeldt disease with behavioral disturbance	ICD10CM	2	F02.81	A81.00,F02.81	Dementia in other diseases classified elsewhere with be	IMO	GHS-PACDC	DX_LEGACY	ACTIVE	N	1900-01-01	9999-12-31	2015-07-10
Diagnosis: ICD10 Code, F00-F99 Define	F01-F99 Mental, Behavioral and Neurodevelopmental disorders	429303	Major frontotemporal neurocognitive disorder, probable, with behavioral disturbance	ICD10CM	2	F02.81	G31.09,F02.81	Dementia in other diseases classified elsewhere with be	IMO	GHS-PACDC	IMO_2015_R	ACTIVE	N	1900-01-01	9999-12-31	2015-07-10
Diagnosis: ICD10 Code, F00-F99 Define	F01-F99 Mental, Behavioral and Neurodevelopmental disorders	251428	Dementia, Alzheimer's, with behavior disturbance	ICD10CM	2	F02.81	G30.8,F02.81	Dementia in other diseases classified elsewhere with be	IMO	GHS-PACDC	DX_LEGACY	ACTIVE	N	2016-04-10	9999-12-31	2015-07-10
Diagnosis: ICD10 Code, F00-F99 Define	F01-F99 Mental, Behavioral and Neurodevelopmental disorders	1209336	Other frontotemporal dementia with behavioral disturbance	ICD10CM	2	F02.81	G31.09,F02.81	Dementia in other diseases classified elsewhere with be	IMO	GHS-PACDC	IMO_2016_R	DELETED	Y	1900-01-01	9999-12-31	2016-06-02
Diagnosis: ICD10 Code, F00-F99 Define	F01-F99 Mental, Behavioral and Neurodevelopmental disorders	488552	Major neurocognitive disorder, due to multiple etiologies, with behavioral disturbance, mild	ICD10CM	1	F02.81		Dementia in other diseases classified elsewhere with be	IMO	GHS-PACDC	IMO_2015_R	ACTIVE	N	1900-01-01	2016-10-14	2015-07-10
Diagnosis: ICD10 Code, F00-F99 Define	F01-F99 Mental, Behavioral and Neurodevelopmental disorders	1202132	Mild major neurocognitive disorder as late effect of traumatic brain injury with behavioral distu	ICD10CM	2	F02.81	S06.9X3D,F02.81	Dementia in other diseases classified elsewhere with be	IMO	GHS-PACDC	IMO_2016_R	DELETED	Y	1900-01-01	2016-10-14	2016-06-02
Diagnosis: ICD10 Code, F00-F99 Define	F01-F99 Mental, Behavioral and Neurodevelopmental disorders	488765	Major neurocognitive disorder, due to frontotemporal lobar degeneration, with behavioral dist	ICD10CM	2	F02.81	G31.09,F02.81	Dementia in other diseases classified elsewhere with be	IMO	GHS-PACDC	IMO_2015_R	ACTIVE	N	2016-09-17	9999-12-31	2016-09-30
Diagnosis: ICD10 Code, F00-F99 Define	F01-F99 Mental, Behavioral and Neurodevelopmental disorders	140777	Senile and presenile organic psychotic conditions	ICD10CM	1	F03.90		Unspecified dementia without behavioral disturbance	IMO	GHS-PACDC	DX_LEGACY	ACTIVE	N	1900-01-01	9999-12-31	2015-07-10
Diagnosis: ICD10 Code, F00-F99 Define	F01-F99 Mental, Behavioral and Neurodevelopmental disorders	958800	Dementia, old age, without behavioral disturbance	ICD10CM	1	F03.90		Unspecified dementia without behavioral disturbance	IMO	GHS-PACDC	IMO_2015_R	ACTIVE	N	1900-01-01	9999-12-31	2015-07-10
Diagnosis: ICD10 Code, F00-F99 Define	F01-F99 Mental, Behavioral and Neurodevelopmental disorders	962302	Senile and presenile organic psychotic conditions, without behavioral disturbance	ICD10CM	1	F03.90		Unspecified dementia without behavioral disturbance	IMO	GHS-PACDC	IMO_2015_R	ACTIVE	N	1900-01-01	9999-12-31	2015-07-10
Diagnosis: ICD10 Code, F00-F99 Define	F01-F99 Mental, Behavioral and Neurodevelopmental disorders	957607	Dementia, primary degenerative, senile, without behavioral disturbance	ICD10CM	1	F03.90		Unspecified dementia without behavioral disturbance	IMO	GHS-PACDC	IMO_2015_R	ACTIVE	N	1900-01-01	9999-12-31	2015-07-10
Diagnosis: ICD10 Code, F00-F99 Define	F01-F99 Mental, Behavioral and Neurodevelopmental disorders	424346	Dementia, unspecified	ICD10CM	1	F03.90		Unspecified dementia without behavioral disturbance	IMO	GHS-PACDC	IMO_2015_R	DELETED	Y	1900-01-01	9999-12-31	2015-07-10
Diagnosis: ICD10 Code, F00-F99 Define	F01-F99 Mental, Behavioral and Neurodevelopmental disorders	955540	Psychosis in elderly, without behavioral disturbance	ICD10CM	1	F03.90		Unspecified dementia without behavioral disturbance	IMO	GHS-PACDC	IMO_2015_R	ACTIVE	N	1900-01-01	9999-12-31	2015-07-10
Diagnosis: ICD10 Code, F00-F99 Define	F01-F99 Mental, Behavioral and Neurodevelopmental disorders	97752	Dementia, in, senility	ICD10CM	1	F03.90		Unspecified dementia without behavioral disturbance	IMO	GHS-PACDC	DX_LEGACY	ACTIVE	N	1900-01-01	9999-12-31	2015-07-10
Diagnosis: ICD10 Code, F00-F99 Define	F01-F99 Mental, Behavioral and Neurodevelopmental disorders	254958	Geriatric psychosis	ICD10CM	1	F03.90		Unspecified dementia without behavioral disturbance	IMO	GHS-PACDC	DX_LEGACY	ACTIVE	N	1900-01-01	9999-12-31	2015-07-10
Diagnosis: ICD10 Code, F00-F99 Define	F01-F99 Mental, Behavioral and Neurodevelopmental disorders	962280	Mixed vascular and neurodegenerative dementia, without behavioral disturbance	ICD10CM	2	F03.90	F01.50,F03.90	Unspecified dementia without behavioral disturbance	IMO	GHS-PACDC	IMO_2015_R	ACTIVE	N	2016-04-10	2017-10-14	2015-07-10
Diagnosis: ICD10 Code, F00-F99 Define	F01-F99 Mental, Behavioral and Neurodevelopmental disorders	959150	Senile psychosis, without behavioral disturbance	ICD10CM	1	F03.90		Unspecified dementia without behavioral disturbance	IMO	GHS-PACDC	IMO_2015_R	ACTIVE	N	1900-01-01	9999-12-31	2015-07-10
Diagnosis: ICD10 Code, F00-F99 Define	F01-F99 Mental, Behavioral and Neurodevelopmental disorders	1070063	Presenile dementia with delusions, without behavioral disturbance	ICD10CM	1	F03.90		Unspecified dementia without behavioral disturbance	IMO	GHS-PACDC	IMO_2015_R	ACTIVE	N	1900-01-01	9999-12-31	2015-07-10
Diagnosis: ICD10 Code, F00-F99 Define	F01-F99 Mental, Behavioral and Neurodevelopmental disorders	961560	Presenile depression, without behavioral disturbance	ICD10CM	1	F03.90		Unspecified dementia without behavioral disturbance	IMO	GHS-PACDC	IMO_2015_R	ACTIVE	N	1900-01-01	9999-12-31	2015-07-10
Diagnosis: ICD10 Code, F00-F99 Define	F01-F99 Mental, Behavioral and Neurodevelopmental disorders	962817	Senile dementia, delirium, with behavioral disturbance	ICD10CM	1	F03.91		Unspecified dementia with behavioral disturbance	IMO	GHS-PACDC	IMO_2015_R	ACTIVE	N	1900-01-01	2017-10-14	2015-07-10
Diagnosis: ICD10 Code, F00-F99 Define	F01-F99 Mental, Behavioral and Neurodevelopmental disorders	452949	Senile dementia with delirium with behavioral disturbance	ICD10CM	1	F03.91	F03.91,F05	Unspecified dementia with behavioral disturbance	IMO	GHS-PACDC	IMO_2015_R	ACTIVE	N	2017-09-17	9999-12-31	2017-10-30
Diagnosis: ICD10 Code, F00-F99 Define	F01-F99 Mental, Behavioral and Neurodevelopmental disorders	514594	Dementia due to axis III etiology with behavioral disturbance	ICD10CM	1	F03.91		Unspecified dementia with behavioral disturbance	IMO	GHS-PACDC	IMO_2015_R	ACTIVE	N	1900-01-01	9999-12-31	2015-07-10
Diagnosis: ICD10 Code, F00-F99 Define	F01-F99 Mental, Behavioral and Neurodevelopmental disorders	465179	Presenile dementia with paranoia with behavioral disturbance	ICD10CM	1	F03.91		Unspecified dementia with behavioral disturbance	IMO	GHS-PACDC	IMO_2015_R	ACTIVE	N	1900-01-01	9999-12-31	2015-07-10
Diagnosis: ICD10 Code, F00-F99 Define	F01-F99 Mental, Behavioral and Neurodevelopmental disorders	456254	Presenile dementia with behavioral disturbance	ICD10CM	1	F03.91		Unspecified dementia with behavioral disturbance	IMO	GHS-PACDC	IMO_2015_R	ACTIVE	N	1900-01-01	9999-12-31	2015-07-10
Diagnosis: ICD10 Code, F00-F99 Define	F01-F99 Mental, Behavioral and Neurodevelopmental disorders	956235	Presenile depression, with behavioral disturbance	ICD10CM	1	F03.91		Unspecified dementia with behavioral disturbance	IMO	GHS-PACDC	IMO_2015_R	ACTIVE	N	1900-01-01	9999-12-31	2015-07-10
Diagnosis: ICD10 Code, F00-F99 Define	F01-F99 Mental, Behavioral and Neurodevelopmental disorders	420228	Dementia with psychosis	ICD10CM	1	F03.91		Unspecified dementia with behavioral disturbance	IMO	GHS-PACDC	DX_LEGACY	ACTIVE	N	1900-01-01	9999-12-31	2015-07-10
Diagnosis: ICD10 Code, F00-F99 Define	F01-F99 Mental, Behavioral and Neurodevelopmental disorders	452996	Senile psychosis with behavioral disturbance	ICD10CM	1	F03.91		Unspecified dementia with behavioral disturbance	IMO	GHS-PACDC	IMO_2015_R	ACTIVE	N	1900-01-01	9999-12-31	2015-07-10
Diagnosis: ICD10 Code, F00-F99 Define	F01-F99 Mental, Behavioral and Neurodevelopmental disorders	448700	Senile dementia with delusional features with behavioral disturbance (HCC)	ICD10CM	1	F03.91	F03.91,F22	Unspecified dementia with behavioral disturbance	IMO	GHS-PACDC	IMO_2015_R	ACTIVE	N	1900-01-01	9999-12-31	2015-07-10
Diagnosis: ICD10 Code, F00-F99 Define	F01-F99 Mental, Behavioral and Neurodevelopmental disorders	77854	Korsakoff's psychosis	ICD10CM	1	F04		Amnestic disorder due to known physiological condition	IMO	GHS-PACDC	DX_LEGACY	ACTIVE	N	1900-01-01	9999-12-31	2015-07-10
Diagnosis: ICD10 Code, F00-F99 Define	F01-F99 Mental, Behavioral and Neurodevelopmental disorders	489462	Mixed level of activity delirium due to multiple etiologies, persistent	ICD10CM	1	F05		Delirium due to known physiological condition	IMO	GHS-PACDC	IMO_2015_R	ACTIVE	N	1900-01-01	9999-12-31	2015-07-10
Diagnosis: ICD10 Code, F00-F99 Define	F01-F99 Mental, Behavioral and Neurodevelopmental disorders	489214	Delirium due to multiple etiologies, acute, mixed level of activity	ICD10CM	1	F05		Delirium due to known physiological condition	IMO	GHS-PACDC	IMO_2015_R	ACTIVE	N	1900-01-01	9999-12-31	2015-07-10
Diagnosis: ICD10 Code, F00-F99 Define	F01-F99 Mental, Behavioral and Neurodevelopmental disorders	661824	Acute post-traumatic delirium, subsequent encounter	ICD10CM	2	F05	T79.8XXD,F05	Delirium due to known physiological condition	IMO	GHS-PACDC	IMO_2015_R	ACTIVE	N	1900-01-01	9999-12-31	2015-07-10
Diagnosis: ICD10 Code, F00-F99 Define	F01-F99 Mental, Behavioral and Neurodevelopmental disorders	408534	Subacute confusional state of metabolic origin	ICD10CM	1	F05		Delirium due to known physiological condition	IMO	GHS-PACDC	DX_LEGACY	ACTIVE	N	1900-01-01	9999-12-31	2015-07-10
Diagnosis: ICD10 Code, F00-F99 Define	F01-F99 Mental, Behavioral and Neurodevelopmental disorders	962312	Senile dementia with delirium, without behavioral disturbance	ICD10CM	2	F05	F03.90,F05	Delirium due to known physiological condition	IMO	GHS-PACDC	IMO_2015_R	ACTIVE	N	2016-04-10	9999-12-31	2015-07-10
Diagnosis: ICD10 Code, F00-F99 Define	F01-F99 Mental, Behavioral and Neurodevelopmental disorders	593056	Catatonic disorder due to known physiological condition	ICD10CM	1	F06.1		Catatonic disorder due to known physiological condition	IMO	GHS-PACDC	IMO_2015_R	ACTIVE	Y	1900-01-01	9999-12-31	2015-07-10
Diagnosis: ICD10 Code, F00-F99 Define	F01-F99 Mental, Behavioral and Neurodevelopmental disorders	227008	Catatonic state	ICD10CM	1	F06.1		Catatonic disorder due to known physiological condition	IMO	GHS-PACDC	DX_LEGACY	ACTIVE	N	2016-09-17	9999-12-31	2016-09-30
Diagnosis: ICD10 Code, F00-F99 Define	F01-F99 Mental, Behavioral and Neurodevelopmental disorders	488888	Bipolar I disorder, severe, current or most recent episode depressed, in full remission, with cata	ICD10CM	2	F06.1	F31.76,F06.1	Catatonic disorder due to known physiological condition	IMO	GHS-PACDC	IMO_2015_R	ACTIVE	N	2016-09-17	9999-12-31	2016-09-30
Diagnosis: ICD10 Code, F00-F99 Define	F01-F99 Mental, Behavioral and Neurodevelopmental disorders	486251	Bipolar I disorder, most recent episode (or current) manic, moderate with catatonia (HCC)	ICD10CM	2	F06.1	F31.12,F06.1	Catatonic disorder due to known physiological condition	IMO	GHS-PACDC	IMO_2015_R	ACTIVE	N	2016-09-17	9999-12-31	2016-09-30
Diagnosis: ICD10 Code, F00-F99 Define	F01-F99 Mental, Behavioral and Neurodevelopmental disorders	489567	Bipolar I disorder, mild, current or most recent episode manic, with psychotic features, with cat	ICD10CM	2	F06.1	F31.2,F06.1	Catatonic disorder due to known physiological condition	IMO	GHS-PACDC	IMO_2015_R	ACTIVE	N	2016-09-17	9999-12-31	2016-09-30
Diagnosis: ICD10 Code, F00-F99 Define	F01-F99 Mental, Behavioral and Neurodevelopmental disorders	140795	Psychotic disorder with delusions in conditions classified elsewhere	ICD10CM	1	F06.2		Psychotic disorder with delusions due to known physiolo	IMO	GHS-PACDC	DX_LEGACY	ACTIVE	N	1900-01-01	9999-12-31	2015-07-10
Diagnosis: ICD10 Code, F00-F99 Define	F01-F99 Mental, Behavioral and Neurodevelopmental disorders	602514	Mood disorder due to known physiological condition, unspecified	ICD10CM	1	F06.30		Mood disorder due to known physiological condition, ur	IMO	GHS-PACDC	IMO_2015_R	ACTIVE	Y	1900-01-01	9999-12-31	2015-07-10
Diagnosis: ICD10 Code, F00-F99 Define	F01-F99 Mental, Behavioral and Neurodevelopmental disorders	254165	Mood disorder secondary to multiple medical problems	ICD10CM	1	F06.30		Mood disorder due to known physiological condition, ur	IMO	GHS-PACDC	DX_LEGACY	ACTIVE	N	1900-01-01	9999-12-31	2015-07-10
Diagnosis: ICD10 Code, F00-F99 Define	F01-F99 Mental, Behavioral and Neurodevelopmental disorders	1366379	Mood disord d/t physiol cond w major depressive-like epsd	ICD10CM	1	F06.32		Mood disorder due to known physiological condition wi	IMO	GHS-PACDC	IMO_2018_R	ACTIVE	N	1900-01-01	9999-12-31	2017-10-30
Diagnosis: ICD10 Code, F00-F99 Define	F01-F99 Mental, Behavioral and Neurodevelopmental disorders	30043	Organic mood disorder of manic type	ICD10CM	1	F06.33		Mood disorder due to known physiological condition wi	IMO	GHS-PACDC	DX_LEGACY	ACTIVE	N	1900-01-01	9999-12-31	2015-07-10
Diagnosis: ICD10 Code, F00-F99 Define	F01-F99 Mental, Behavioral and Neurodevelopmental disorders	156160	Mental disorder due to general medical condition	ICD10CM	1	F06.8		Other specified mental disorders due to known physiolo	IMO	GHS-PACDC	DX_LEGACY	ACTIVE	N	1900-01-01	9999-12-31	2015-07-10
Diagnosis: ICD10 Code, F00-F99 Define	F01-F99 Mental, Behavioral and Neurodevelopmental disorders	157126	Mental disorder, organic	ICD10CM	1	F06.8		Other specified mental disorders due to known physiolo	IMO	GHS-PACDC	DX_LEGACY	ACTIVE	N	1900-01-01	9999-12-31	2015-07-10
Diagnosis: ICD10 Code, F00-F99 Define	F01-F99 Mental, Behavioral and Neurodevelopmental disorders	299876	Mental disorder following injury	ICD10CM	1	F06.8		Other specified mental disorders due to known physiolo	IMO	GHS-PACDC	DX_LEGACY	ACTIVE	N	1900-01-01	9999-12-31	2015-07-10
Diagnosis: ICD10 Code, F00-F99 Define	F01-F99 Mental, Behavioral and Neurodevelopmental disorders	413007	Organic dementia associated with acquired immunodeficiency syndrome (AIDS) (HCC)	ICD10CM	2	F06.8	B20,F06.8	Other specified mental disorders due to known physiolo	IMO	GHS-PACDC	DX_LEGACY	ACTIVE	N	1900-01-01	9999-12-31	2015-07-10
Diagnosis: ICD10 Code, F00-F99 Define	F01-F99 Mental, Behavioral and Neurodevelopmental disorders	156252	Amnestic disorder in conditions classified elsewhere	ICD10CM	1	F06.8		Other specified mental disorders due to known physiolo	IMO	GHS-PACDC	DX_LEGACY	ACTIVE	N	1900-01-01	9999-12-31	2015-07-10
Diagnosis: ICD10 Code, F00-F99 Define	F01-F99 Mental, Behavioral and Neurodevelopmental disorders	343725	PANDAS (pediatric autoimmune neuropsychiatric disorders associated with streptococcal infect	ICD10CM	1	F06.8	F06.8,B94.9	Other specified mental disorders due to known physiolo	IMO	GHS-PACDC	DX_LEGACY	ACTIVE	N	1900-01-01	2017-10-14	2015-07-10

Diagnosis: ICD10 Code, F00-F99 Define	F01-F99 Mental, Behavioral and Neurodevelopmental disorders	615096	Personality and behavioral disorders due to known physiological condition	ICD10CM	1	F07		Personality and behavioral disorders due to known phys	IMO	GHS-PACDC	IMO_2015_R	ACTIVE	Y	1900-01-01	9999-12-31	2015-07-10
Diagnosis: ICD10 Code, F00-F99 Define	F01-F99 Mental, Behavioral and Neurodevelopmental disorders	156169	Personality change due to physical condition	ICD10CM	1	F07.0		Personality change due to known physiological conditior	IMO	GHS-PACDC	DX_LEGACY	ACTIVE	N	1900-01-01	9999-12-31	2015-07-10
Diagnosis: ICD10 Code, F00-F99 Define	F01-F99 Mental, Behavioral and Neurodevelopmental disorders	409246	Organic pseudopsychopathic personality	ICD10CM	1	F07.0		Personality change due to known physiological conditior	IMO	GHS-PACDC	DX_LEGACY	ACTIVE	N	1900-01-01	9999-12-31	2015-07-10
Diagnosis: ICD10 Code, F00-F99 Define	F01-F99 Mental, Behavioral and Neurodevelopmental disorders	73099	Presbyophrenia	ICD10CM	1	F07.0		Personality change due to known physiological conditior	IMO	GHS-PACDC	DX_LEGACY	ACTIVE	N	1900-01-01	9999-12-31	2015-07-10
Diagnosis: ICD10 Code, F00-F99 Define	F01-F99 Mental, Behavioral and Neurodevelopmental disorders	3298	Organic personality disorder	ICD10CM	1	F07.0		Personality change due to known physiological conditior	IMO	GHS-PACDC	DX_LEGACY	ACTIVE	N	1900-01-01	9999-12-31	2015-07-10
Diagnosis: ICD10 Code, F00-F99 Define	F01-F99 Mental, Behavioral and Neurodevelopmental disorders	263865	Change in personality	ICD10CM	1	F07.0		Personality change due to known physiological conditior	IMO	GHS-PACDC	DX_LEGACY	ACTIVE	N	1900-01-01	2016-05-07	2015-07-10
Diagnosis: ICD10 Code, F00-F99 Define	F01-F99 Mental, Behavioral and Neurodevelopmental disorders	128772	Post concussive encephalopathy	ICD10CM	1	F07.81		Postconcussional syndrome	IMO	GHS-PACDC	DX_LEGACY	ACTIVE	N	1900-01-01	9999-12-31	2015-07-10
Diagnosis: ICD10 Code, F00-F99 Define	F01-F99 Mental, Behavioral and Neurodevelopmental disorders	409245	Postconcussional personality disorder	ICD10CM	2	F07.81	F60.9,F07.81	Postconcussional syndrome	IMO	GHS-PACDC	DX_LEGACY	ACTIVE	N	1900-01-01	9999-12-31	2015-07-10
Diagnosis: ICD10 Code, F00-F99 Define	F01-F99 Mental, Behavioral and Neurodevelopmental disorders	125550	Chronic traumatic encephalopathy	ICD10CM	1	F07.81		Postconcussional syndrome	IMO	GHS-PACDC	DX_LEGACY	ACTIVE	N	1900-01-01	9999-12-31	2015-07-10
Diagnosis: ICD10 Code, F00-F99 Define	F01-F99 Mental, Behavioral and Neurodevelopmental disorders	30683	Post-traumatic brain syndrome	ICD10CM	1	F07.81		Postconcussional syndrome	IMO	GHS-PACDC	DX_LEGACY	ACTIVE	N	1900-01-01	9999-12-31	2015-07-10
Diagnosis: ICD10 Code, F00-F99 Define	F01-F99 Mental, Behavioral and Neurodevelopmental disorders	59726	Postconcussive syndrome	ICD10CM	1	F07.81		Postconcussional syndrome	IMO	GHS-PACDC	DX_LEGACY	ACTIVE	N	1900-01-01	9999-12-31	2015-07-10
Diagnosis: ICD10 Code, F00-F99 Define	F01-F99 Mental, Behavioral and Neurodevelopmental disorders	101550	Postcontusional encephalopathy	ICD10CM	1	F07.81		Postconcussional syndrome	IMO	GHS-PACDC	DX_LEGACY	ACTIVE	N	1900-01-01	9999-12-31	2015-07-10
Diagnosis: ICD10 Code, F00-F99 Define	F01-F99 Mental, Behavioral and Neurodevelopmental disorders	499969	Personality and behavioral disorders due to brain disease, damage, and dysfunction	ICD10CM	2	F07.89	G93.89,F07.89	Other personality and behavioral disorders due to know	IMO	GHS-PACDC	IMO_2015_R	ACTIVE	N	1900-01-01	9999-12-31	2015-07-10
Diagnosis: ICD10 Code, F00-F99 Define	F01-F99 Mental, Behavioral and Neurodevelopmental disorders	1092518	Severe major neurocognitive disorder due to traumatic brain injury without behavioral disturba	ICD10CM	2	F09	S06.9X0D,F09	Unspecified mental disorder due to known physiological	IMO	GHS-PACDC	IMO_2015_R	DELETED	Y	1900-01-01	2017-10-14	2015-07-10
Diagnosis: ICD10 Code, F00-F99 Define	F01-F99 Mental, Behavioral and Neurodevelopmental disorders	251865	Acquired cognitive dysfunction	ICD10CM	1	F09		Unspecified mental disorder due to known physiological	IMO	GHS-PACDC	DX_LEGACY	ACTIVE	N	1900-01-01	9999-12-31	2015-07-10
Diagnosis: ICD10 Code, F00-F99 Define	F01-F99 Mental, Behavioral and Neurodevelopmental disorders	488818	Possible major vascular neurocognitive disorder with behavioral disturbance	ICD10CM	1	F09		Unspecified mental disorder due to known physiological	IMO	GHS-PACDC	IMO_2015_R	ACTIVE	N	1900-01-01	2016-10-14	2015-07-10
Diagnosis: ICD10 Code, F00-F99 Define	F01-F99 Mental, Behavioral and Neurodevelopmental disorders	489065	Possible major neurocognitive disorder due to frontotemporal lobar degeneration	ICD10CM	2	F09	G31.9,F09	Unspecified mental disorder due to known physiological	IMO	GHS-PACDC	IMO_2015_R	ACTIVE	N	1900-01-01	2016-10-14	2015-07-10
Diagnosis: ICD10 Code, F00-F99 Define	F01-F99 Mental, Behavioral and Neurodevelopmental disorders	327602	Cognitive deficit secondary to MS (HCC)	ICD10CM	2	F09	G35,F09	Unspecified mental disorder due to known physiological	IMO	GHS-PACDC	DX_LEGACY	ACTIVE	N	1900-01-01	9999-12-31	2015-07-10
Diagnosis: ICD10 Code, F00-F99 Define	F01-F99 Mental, Behavioral and Neurodevelopmental disorders	296968	Psychological trauma	ICD10CM	1	F09		Unspecified mental disorder due to known physiological	IMO	GHS-PACDC	DX_LEGACY	ACTIVE	N	1900-01-01	9999-12-31	2015-07-10
Diagnosis: ICD10 Code, F00-F99 Define	F01-F99 Mental, Behavioral and Neurodevelopmental disorders	423634	Persistent mental disorders due to conditions classified elsewhere	ICD10CM	1	F09		Unspecified mental disorder due to known physiological	IMO	GHS-PACDC	IMO_2015_R	DELETED	Y	2016-04-10	9999-12-31	2015-07-10
Diagnosis: ICD10 Code, F00-F99 Define	F01-F99 Mental, Behavioral and Neurodevelopmental disorders	256921	Cognitive deficit due to multiple subcortical infarcts (HCC)	ICD10CM	1	F09		Unspecified mental disorder due to known physiological	IMO	GHS-PACDC	DX_LEGACY	ACTIVE	N	1900-01-01	2016-05-07	2015-07-10
Diagnosis: ICD10 Code, F00-F99 Define	F01-F99 Mental, Behavioral and Neurodevelopmental disorders	488763	Major neurocognitive disorder, due to alzheimer's disease, without behavioral disturbance, mo	ICD10CM	2	F09	G30.9,F09	Unspecified mental disorder due to known physiological	IMO	GHS-PACDC	IMO_2015_R	ACTIVE	N	1900-01-01	2016-10-14	2015-07-10
Diagnosis: ICD10 Code, F00-F99 Define	F01-F99 Mental, Behavioral and Neurodevelopmental disorders	206612	History of alcohol abuse	ICD10CM	1	F10.10		Alcohol abuse, uncomplicated	IMO	GHS-PACDC	DX_LEGACY	ACTIVE	N	1900-01-01	2016-05-07	2015-07-10
Diagnosis: ICD10 Code, F00-F99 Define	F01-F99 Mental, Behavioral and Neurodevelopmental disorders	39255	Alcohol abuse	ICD10CM	1	F10.10		Alcohol abuse, uncomplicated	IMO	GHS-PACDC	DX_LEGACY	ACTIVE	N	1900-01-01	9999-12-31	2015-07-10
Diagnosis: ICD10 Code, F00-F99 Define	F01-F99 Mental, Behavioral and Neurodevelopmental disorders	76622	Nondependent alcohol abuse, in remission	ICD10CM	1	F10.10		Alcohol abuse, uncomplicated	IMO	GHS-PACDC	DX_LEGACY	ACTIVE	N	1900-01-01	2017-10-14	2015-07-10
Diagnosis: ICD10 Code, F00-F99 Define	F01-F99 Mental, Behavioral and Neurodevelopmental disorders	182991	Alcohol abuse complicating pregnancy	ICD10CM	2	F10.10	O99.310,F10.10	Alcohol abuse, uncomplicated	IMO	GHS-PACDC	DX_LEGACY	ACTIVE	N	1900-01-01	9999-12-31	2015-07-10
Diagnosis: ICD10 Code, F00-F99 Define	F01-F99 Mental, Behavioral and Neurodevelopmental disorders	526828	Alcohol abuse affecting pregnancy in first trimester	ICD10CM	2	F10.10	O99.311,F10.10	Alcohol abuse, uncomplicated	IMO	GHS-PACDC	IMO_2015_R	ACTIVE	N	1900-01-01	9999-12-31	2015-07-10
Diagnosis: ICD10 Code, F00-F99 Define	F01-F99 Mental, Behavioral and Neurodevelopmental disorders	3174	Alcohol abuse, episodic	ICD10CM	1	F10.10		Alcohol abuse, uncomplicated	IMO	GHS-PACDC	DX_LEGACY	ACTIVE	N	1900-01-01	9999-12-31	2015-07-10
Diagnosis: ICD10 Code, F00-F99 Define	F01-F99 Mental, Behavioral and Neurodevelopmental disorders	488575	Alcohol use disorder, mild, in early remission	ICD10CM	1	F10.10		Alcohol abuse, uncomplicated	IMO	GHS-PACDC	IMO_2015_R	ACTIVE	N	2016-09-17	9999-12-31	2016-09-30
Diagnosis: ICD10 Code, F00-F99 Define	F01-F99 Mental, Behavioral and Neurodevelopmental disorders	508104	Alcohol use disorder, mild, in early remission, in controlled environment, abuse	ICD10CM	1	F10.11		Alcohol abuse, in remission	IMO	GHS-PACDC	IMO_2015_R	ACTIVE	N	2017-09-17	9999-12-31	2017-10-30
Diagnosis: ICD10 Code, F00-F99 Define	F01-F99 Mental, Behavioral and Neurodevelopmental disorders	955515	Alcohol intoxication, uncomplicated (HCC)	ICD10CM	1	F10.120		Alcohol abuse with intoxication, uncomplicated	IMO	GHS-PACDC	IMO_2015_R	ACTIVE	N	1900-01-01	2017-10-14	2015-07-10
Diagnosis: ICD10 Code, F00-F99 Define	F01-F99 Mental, Behavioral and Neurodevelopmental disorders	982907	Very severe alcohol intoxication with complication, uncomplicated	ICD10CM	1	F10.120		Alcohol abuse with intoxication, uncomplicated	IMO	GHS-PACDC	IMO_2015_R	DELETED	Y	1900-01-01	9999-12-31	2015-07-10
Diagnosis: ICD10 Code, F00-F99 Define	F01-F99 Mental, Behavioral and Neurodevelopmental disorders	460130	Hangover without complication (HCC)	ICD10CM	1	F10.120		Alcohol abuse with intoxication, uncomplicated	IMO	GHS-PACDC	IMO_2015_R	ACTIVE	N	1900-01-01	9999-12-31	2015-07-10
Diagnosis: ICD10 Code, F00-F99 Define	F01-F99 Mental, Behavioral and Neurodevelopmental disorders	978284	Hangover with complication, with delirium	ICD10CM	1	F10.121		Alcohol abuse with intoxication delirium	IMO	GHS-PACDC	IMO_2015_R	DELETED	Y	1900-01-01	9999-12-31	2015-07-10
Diagnosis: ICD10 Code, F00-F99 Define	F01-F99 Mental, Behavioral and Neurodevelopmental disorders	955841	Alcohol intoxication, with delirium (HCC)	ICD10CM	1	F10.121		Alcohol abuse with intoxication delirium	IMO	GHS-PACDC	IMO_2015_R	ACTIVE	N	1900-01-01	2017-10-14	2015-07-10
Diagnosis: ICD10 Code, F00-F99 Define	F01-F99 Mental, Behavioral and Neurodevelopmental disorders	957610	The hangs, with delirium (HCC)	ICD10CM	1	F10.121		Alcohol abuse with intoxication delirium	IMO	GHS-PACDC	IMO_2015_R	ACTIVE	N	1900-01-01	9999-12-31	2015-07-10
Diagnosis: ICD10 Code, F00-F99 Define	F01-F99 Mental, Behavioral and Neurodevelopmental disorders	427208	Episodic acute alcoholic intoxication, with delirium (HCC)	ICD10CM	1	F10.121		Alcohol abuse with intoxication delirium	IMO	GHS-PACDC	IMO_2015_R	ACTIVE	N	1900-01-01	9999-12-31	2015-07-10
Diagnosis: ICD10 Code, F00-F99 Define	F01-F99 Mental, Behavioral and Neurodevelopmental disorders	962909	Acute alcoholic intoxication, episodic drinking behavior, with delirium (HCC)	ICD10CM	1	F10.121		Alcohol abuse with intoxication delirium	IMO	GHS-PACDC	IMO_2015_R	ACTIVE	N	1900-01-01	9999-12-31	2015-07-10
Diagnosis: ICD10 Code, F00-F99 Define	F01-F99 Mental, Behavioral and Neurodevelopmental disorders	959223	Acute alcoholic intoxication, with delirium (HCC)	ICD10CM	1	F10.121		Alcohol abuse with intoxication delirium	IMO	GHS-PACDC	IMO_2015_R	ACTIVE	N	1900-01-01	2017-10-14	2015-07-10
Diagnosis: ICD10 Code, F00-F99 Define	F01-F99 Mental, Behavioral and Neurodevelopmental disorders	1088650	Alcohol intoxication with mild use disorder with complication, with unspecified complication	ICD10CM	1	F10.129		Alcohol abuse with intoxication, unspecified	IMO	GHS-PACDC	IMO_2015_R	DELETED	Y	1900-01-01	9999-12-31	2015-07-10
Diagnosis: ICD10 Code, F00-F99 Define	F01-F99 Mental, Behavioral and Neurodevelopmental disorders	485888	Alcohol-induced anxiety disorder with mild use disorder with onset during intoxication (HCC)	ICD10CM	2	F10.129	F10.180,F10.129	Alcohol abuse with intoxication, unspecified	IMO	GHS-PACDC	IMO_2015_R	ACTIVE	N	2016-09-17	9999-12-31	2016-09-30
Diagnosis: ICD10 Code, F00-F99 Define	F01-F99 Mental, Behavioral and Neurodevelopmental disorders	1089583	Alcohol use with uncomplicated intoxication without use disorder, with unspecified complicatio	ICD10CM	1	F10.129		Alcohol abuse with intoxication, unspecified	IMO	GHS-PACDC	IMO_2015_R	DELETED	Y	1900-01-01	9999-12-31	2015-07-10
Diagnosis: ICD10 Code, F00-F99 Define	F01-F99 Mental, Behavioral and Neurodevelopmental disorders	957251	Alcoholic intoxication, chronic, with unspecified complication (HCC)	ICD10CM	1	F10.129		Alcohol abuse with intoxication, unspecified	IMO	GHS-PACDC	IMO_2015_R	ACTIVE	N	1900-01-01	9999-12-31	2015-07-10
Diagnosis: ICD10 Code, F00-F99 Define	F01-F99 Mental, Behavioral and Neurodevelopmental disorders	201789	Very severe alcohol intoxication (HCC)	ICD10CM	1	F10.129		Alcohol abuse with intoxication, unspecified	IMO	GHS-PACDC	DX_LEGACY	ACTIVE	N	1900-01-01	9999-12-31	2015-07-10
Diagnosis: ICD10 Code, F00-F99 Define	F01-F99 Mental, Behavioral and Neurodevelopmental disorders	429106	Onset of alcohol-induced psychotic disorder with hallucinations during intoxication (HCC)	ICD10CM	2	F10.129	F10.151,F10.129	Alcohol abuse with intoxication, unspecified	IMO	GHS-PACDC	IMO_2015_R	ACTIVE	N	1900-01-01	2017-04-27	2015-07-10
Diagnosis: ICD10 Code, F00-F99 Define	F01-F99 Mental, Behavioral and Neurodevelopmental disorders	1087504	Alcohol-induced psychotic disorder with mild use disorder with onset during intoxication with c	ICD10CM	1	F10.150	F10.150,F10.129	Alcohol abuse with alcohol-induced psychotic disorder v	IMO	GHS-PACDC	IMO_2015_R	ACTIVE	N	2016-09-17	9999-12-31	2016-09-30
Diagnosis: ICD10 Code, F00-F99 Define	F01-F99 Mental, Behavioral and Neurodevelopmental disorders	1184278	Alcohol abuse with alcohol-induced psychotic disorder with hallucinations (HCC)	ICD10CM	1	F10.151		Alcohol abuse with alcohol-induced psychotic disorder v	IMO	GHS-PACDC	IMO_2016_R	ACTIVE	N	1900-01-01	9999-12-31	2016-06-02
Diagnosis: ICD10 Code, F00-F99 Define	F01-F99 Mental, Behavioral and Neurodevelopmental disorders	270270	Alcohol abuse with alcohol-induced psychotic disorder (HCC)	ICD10CM	1	F10.159		Alcohol abuse with alcohol-induced psychotic disorder, u	IMO	GHS-PACDC	DX_LEGACY	ACTIVE	N	1900-01-01	9999-12-31	2015-07-10
Diagnosis: ICD10 Code, F00-F99 Define	F01-F99 Mental, Behavioral and Neurodevelopmental disorders	486442	Alcohol-induced psychotic disorder with mild use disorder with onset during intoxication (HCC)	ICD10CM	1	F10.159	F10.159,F10.129	Alcohol abuse with alcohol-induced psychotic disorder, u	IMO	GHS-PACDC	IMO_2015_R	ACTIVE	N	2016-09-17	9999-12-31	2016-09-30
Diagnosis: ICD10 Code, F00-F99 Define	F01-F99 Mental, Behavioral and Neurodevelopmental disorders	632993	Alcohol abuse with other alcohol-induced disorder (HCC)	ICD10CM	1	F10.188		Alcohol abuse with other alcohol-induced disorder	IMO	GHS-PACDC	IMO_2015_R	ACTIVE	N	1900-01-01	9999-12-31	2015-07-10
Diagnosis: ICD10 Code, F00-F99 Define	F01-F99 Mental, Behavioral and Neurodevelopmental disorders	493048	Cerebellar ataxia due to alcohol (HCC)	ICD10CM	1	F10.20	F10.20,G32.81	Alcohol dependence, uncomplicated	IMO	GHS-PACDC	IMO_2015_R	ACTIVE	N	1900-01-01	9999-12-31	2015-07-10
Diagnosis: ICD10 Code, F00-F99 Define	F01-F99 Mental, Behavioral and Neurodevelopmental disorders	317449	Alcohol dependence, binge pattern (HCC)	ICD10CM	1	F10.20		Alcohol dependence, uncomplicated	IMO	GHS-PACDC	DX_LEGACY	ACTIVE	N	1900-01-01	9999-12-31	2015-07-10
Diagnosis: ICD10 Code, F00-F99 Define	F01-F99 Mental, Behavioral and Neurodevelopmental disorders	595296	Alcohol dependence, uncomplicated	ICD10CM	1	F10.20		Alcohol dependence, uncomplicated	IMO	GHS-PACDC	IMO_2015_R	ACTIVE	Y	1900-01-01	9999-12-31	2015-07-10
Diagnosis: ICD10 Code, F00-F99 Define	F01-F99 Mental, Behavioral and Neurodevelopmental disorders	421829	Other and unspecified alcohol dependence	ICD10CM	1	F10.20		Alcohol dependence, uncomplicated	IMO	GHS-PACDC	IMO_2015_R	DELETED	Y	1900-01-01	9999-12-31	2015-07-10
Diagnosis: ICD10 Code, F00-F99 Define	F01-F99 Mental, Behavioral and Neurodevelopmental disorders	488861	Moderate alcohol use disorder in controlled environment (HCC)	ICD10CM	1	F10.20		Alcohol dependence, uncomplicated	IMO	GHS-PACDC	IMO_2015_R	ACTIVE	N	2016-09-17	9999-12-31	2016-09-30
Diagnosis: ICD10 Code, F00-F99 Define	F01-F99 Mental, Behavioral and Neurodevelopmental disorders	489099	Severe alcohol use disorder in controlled environment (HCC)	ICD10CM	1	F10.20		Alcohol dependence, uncomplicated	IMO	GHS-PACDC	IMO_2015_R	ACTIVE	N	2016-09-17	9999-12-31	2016-09-30
Diagnosis: ICD10 Code, F00-F99 Define	F01-F99 Mental, Behavioral and Neurodevelopmental disorders	174771	Alcohol dependency (HCC)	ICD10CM	1	F10.20		Alcohol dependence, uncomplicated	IMO	GHS-PACDC	DX_LEGACY	ACTIVE	N	1900-01-01	9999-12-31	2015-07-10
Diagnosis: ICD10 Code, F00-F99 Define	F01-F99 Mental, Behavioral and Neurodevelopmental disorders	508351	Alcohol use disorder, severe, in sustained remission, in controlled environment, dependence (H	ICD10CM	1	F10.21		Alcohol dependence, in remission	IMO	GHS-PACDC	IMO_2015_R	ACTIVE	N	1900-01-01	9999-12-31	2015-07-10
Diagnosis: ICD10 Code, F00-F99 Define	F01-F99 Mental, Behavioral and Neurodevelopmental disorders	1106056	Alcohol-induced psychotic disorder with onset during intoxication without complication (HCC)	ICD10CM	1	F10.220	F10.220,F10.159	Alcohol dependence with intoxication, uncomplicated	IMO	GHS-PACDC	IMO_2016_R	ACTIVE	N	1900-01-01	9999-12-31	2016-06-02
Diagnosis: ICD10 Code, F00-F99 Define	F01-F99 Mental, Behavioral and Neurodevelopmental disorders	1025564	Acute alcoholic intoxication in alcoholism with blood level over 0.3 without complication, uncor	ICD10CM	1	F10.220		Alcohol dependence with intoxication, uncomplicated	IMO	GHS-PACDC	IMO_2015_R	DELETED	Y	1900-01-01	9999-12-31	2015-07-10
Diagnosis: ICD10 Code, F00-F99 Define	F01-F99 Mental, Behavioral and Neurodevelopmental disorders	1307884	Alcohol dependence with intoxication and delirium (HCC)	ICD10CM	1	F10.221		Alcohol dependence with intoxication delirium	IMO	GHS-PACDC	IMO_2017_R	ACTIVE	N	1900-01-01	9999-12-31	2017-04-14
Diagnosis: ICD10 Code, F00-F99 Define	F01-F99 Mental, Behavioral and Neurodevelopmental disorders	1008685	Acute alcoholic intoxication in alcoholism with complication, with delirium	ICD10CM	1	F10.221		Alcohol dependence with intoxication delirium	IMO	GHS-PACDC	IMO_2015_R	DELETED	Y	1900-01-01	9999-12-31	2015-07-10
Diagnosis: ICD10 Code, F00-F99 Define	F01-F99 Mental, Behavioral and Neurodevelopmental disorders	969778	Acute alcohol intoxication in patient with alcoholism with blood alcohol level 0.08 to 0.29, with	ICD10CM	1	F10.229		Alcohol dependence with intoxication, unspecified	IMO	GHS-PACDC	IMO_2015_R	ACTIVE	N	1900-01-01	9999-12-31	2015-07-10
Diagnosis: ICD10 Code, F00-F99 Define	F01-F99 Mental, Behavioral and Neurodevelopmental disorders	245439	Acute alcohol intoxication with alcoholism (HCC)	ICD10CM	1	F10.229		Alcohol dependence with intoxication, unspecified	IMO	GHS-PACDC	DX_LEGACY	ACTIVE	N	1900-01-01	9999-12-31	2015-07-10
Diagnosis: ICD10 Code, F00-F99 Define	F01-F99 Mental, Behavioral and Neurodevelopmental disorders	455078	Acute alcoholic intoxication in alcoholism with complication (HCC)	ICD10CM	1	F10.229		Alcohol dependence with intoxication, unspecified	IMO	GHS-PACDC	IMO_2015_R	ACTIVE	N	1900-01-01	9999-12-31	2015-07-10
Diagnosis: ICD10 Code, F00-F99 Define	F01-F99 Mental, Behavioral and Neurodevelopmental disorders	1368450	Alcohol-induced psychotic disorder with moderate or severe use disorder with onset during into	ICD10CM	2	F10.229	F10.250,F10.229	Alcohol dependence with intoxication, unspecified	IMO	GHS-PACDC	IMO_2018_R	ACTIVE	N	1900-01-01	9999-12-31	2017-10-30
Diagnosis: ICD10 Code, F00-F99 Define	F01-F99 Mental, Behavioral and Neurodevelopmental disorders	517611	Onset of alcohol-induced anxiety disorder during withdrawal with delirium (HCC)	ICD10CM	1	F10.231		Alcohol dependence with withdrawal delirium	IMO	GHS-PACDC	IMO_2015_R	ACTIVE	N	1900-01-01	2016-10-14	2015-07-10
Diagnosis: ICD10 Code, F00-F99 Define	F01-F99 Mental, Behavioral and Neurodevelopmental disorders	956675	Alcohol withdrawal, with delirium (HCC)	ICD10CM	1	F10.231		Alcohol dependence with withdrawal delirium	IMO	GHS-PACDC	IMO_2015_R	ACTIVE	N	1900-01-01	9999-12-31	2015-07-10
Diagnosis: ICD10 Code, F00-F99 Define	F01-F99 Mental, Behavioral and Neurodevelopmental disorders	1106049	Alcohol-induced psychotic disorder without use disorder with onset during withdrawal with del	ICD10CM	1	F10.231	F10.231,F10.250	Alcohol dependence with withdrawal delirium	IMO	GHS-PACDC	IMO_2016_R	ACTIVE	N	1900-01-01	9999-12-31	2016-06-02
Diagnosis: ICD10 Code, F00-F99 Define	F01-F99 Mental, Behavioral and Neurodevelopmental disorders	990333	Alcohol withdrawal with complication with inpatient treatment, with delirium (HCC)	ICD10CM	1	F10.231		Alcohol dependence with withdrawal delirium	IMO	GHS-PACDC	IMO_2015_R	ACTIVE	N	1900-01-01	9999-12-31	2015-07-10
Diagnosis: ICD10 Code, F00-F99 Define	F01-F99 Mental, Behavioral and Neurodevelopmental disorders	1081516	Alcohol-induced psychotic disorder with onset during withdrawal with complication, with delusi	ICD10CM	5	F10.231	F10.950,F10.230,F10.232,F10.239	Alcohol dependence with withdrawal delirium	IMO	GHS-PACDC	IMO_2015_R	DELETED	Y	1900-01-01	2016-05-07	2015-07-10
Diagnosis: ICD10 Code, F00-F99 Define	F01-F99 Mental, Behavioral and Neurodevelopmental disorders	989214	Alcohol withdrawal seizure with complication, with perceptual disturbance (HCC)	ICD10CM	1	F10.232		Alcohol dependence with withdrawal with perceptual di	IMO	GHS-PACDC	IMO_2015_R	ACTIVE	N	1900-01-01	9999-12-31	2015-07-10
Diagnosis: ICD10 Code, F00-F99 Define	F01-F99 Mental, Behavioral and Neurodevelopmental disorders	966304	Onset of alcohol-induced anxiety disorder during withdrawal, with perceptual disturbance (HCC	ICD10CM	1	F10.232		Alcohol dependence with withdrawal with perceptual di	IMO	GHS-PACDC	IMO_2015_R	ACTIVE	N	2016-04-10	9999-12-31	2015-07-10
Diagnosis: ICD10 Code, F00-F99 Define	F01-F99 Mental, Behavioral and Neurodevelopmental disorders	1104539	Alcohol-induced psychotic disorder with onset during withdrawal with hallucinations (HCC)	ICD10CM	2	F10.239	F10.251,F10.239	Alcohol dependence with withdrawal, unspecified	IMO	GHS-PACDC	IMO_2016_R	ACTIVE	N	1900-01-01	9999-12-31	2016-06-02
Diagnosis: ICD10 Code, F00-F99 Define	F01-F99 Mental, Behavioral and Neurodevelopmental disorders	486740	Alcohol-induced psychotic disorder without use disorder with onset during withdrawal (HCC)	ICD10CM	1	F10.239	F10.239,F10.259	Alcohol dependence with withdrawal, unspecified	IMO	GHS-PACDC	IMO_2015_R	ACTIVE	N	2016-04-10	9999-12-31	2015-07-10
Diagnosis: ICD10 Code, F00-F99 Define	F01-F99 Mental, Behavioral and Neurodevelopmental disorders	976186	Onset of alcohol-induced psychotic disorder during withdrawal, with delusions (HCC)	ICD10CM	2	F10.239	F10.950,F10.239	Alcohol dependence with withdrawal, unspecified	IMO	GHS-PACDC	IMO_2015_R	ACTIVE	N	2016-04-10	9999-12-31	2015-07-10
Diagnosis: ICD10 Code, F00-F99 Define	F01-F99 Mental, Behavioral and Neurodevelopmental disorders	973295	Onset of alcohol-induced sleep disorder, insomnia type, during discontinuation or withdrawal, v	ICD10CM	1	F10.239		Alcohol dependence with withdrawal, unspecified	IMO	GHS-PACDC	IMO_2015_R	ACTIVE	N	1900-01-01	9999-12-31	2015-07-10
Diagnosis: ICD10 Code, F00-F99 Define	F01-F99 Mental, Behavioral and Neurodevelopmental disorders	517926	Alcohol-induced psychotic disorder with onset during withdrawal with complication (HCC)	ICD10CM	2	F10.239	F10.959,F10.239	Alcohol dependence with withdrawal, unspecified	IMO	GHS-PACDC	IMO_2015_R	ACTIVE	N	1900-01-01	2016-10-14	2015-07-10
Diagnosis: ICD10 Code, F00-F99 Define	F01-F99 Mental, Behavioral and Neurodevelopmental disorders	465047	Autonomic hyperactivity due to alcohol withdrawal with complication (HCC)	ICD10CM	1	F10.239		Alcohol dependence with withdrawal, unspecified	IMO	GHS-PACDC	IMO_2015_R	ACTIVE	N	1900-01-01	2016-10-14	2015-07-10
Diagnosis: ICD10 Code, F00-F99 Define	F01-F99 Mental, Behavioral and Neurodevelopmental disorders	429103	Onset of alcohol-induced mood disorder during withdrawal (HCC)	ICD10CM	1	F10.24	F10.24,F10.239	Alcohol dependence with alcohol-induced mood disorde	IMO	GHS-PACDC	IMO_2015_R	ACTIVE	N	1900-01-01	9999-12-31	2015-07-10
Diagnosis: ICD10 Code, F00-F99 Define	F01-F99 Mental, Behavioral and Neurodevelopmental disorders	516494	Alcohol-induced psychotic disorder without use disorder with delusions (HCC)	ICD10CM	2	F10.250	F10.959,F10.250	Alcohol dependence with alcohol-induced psychotic disc	IMO	GHS-PACDC	IMO_2015_R	ACTIVE	N	1900-01-01	2016-05-07	2015-07-10
Diagnosis: ICD10 Code, F00-F99 Define	F01-F99 Mental, Behavioral and Neurodevelopmental disorders	1207327	Alcohol-induced psychotic disorder with mild use disorder with onset during withdrawal with de	ICD10CM	2	F10.250	F10.230,F10.250	Alcohol dependence with alcohol-induced psychotic disc	IMO	GHS-PACDC	IMO_2016_R	ACTIVE	N	1900-01-01	9999-12-31	2016-06-02
Diagnosis: ICD10 Code, F00-F99 Define	F01-F99 Mental, Behavioral and Neurodevelopmental disorders	1085425	Alcohol-induced psychotic disorder with mild use disorder with onset during withdrawal with de	ICD10CM	2	F10.250	F10.239,F10.250	Alcohol dependence with alcohol-induced psychotic disc	IMO	GHS-PACDC	IMO_2015_R	ACTIVE	N	2016-04-10	9999-12-31	2015-07-10
Diagnosis: ICD10 Code, F00-F99 Define	F01-F99 Mental, Behavioral and Neurodevelopmental disorders	429105	Onset of alcohol-induced psychotic disorder with delusions during withdrawal (HCC)	ICD10CM	2	F10.250	F10.239,F10.250	Alcohol dependence with alcohol-induced psychotic disc	IMO	GHS-PACDC	IMO_2015_R	ACTIVE	N	2017-09-17	9999-12-31	2017-10-30
Diagnosis: ICD10 Code, F00-F99 Define	F01-F99 Mental, Behavioral and Neurodevelopmental disorders	516658	Chronic alcoholism with psychosis with hallucinations (HCC)	ICD10CM	1	F10.251		Alcohol dependence with alcohol-induced psychotic disc	IMO	GHS-PACDC	IMO_2015_R	ACTIVE	N	1900-01-01	2017-10-14	2015-07-10
Diagnosis: ICD10 Code, F00-F99 Define	F01-F99 Mental, Behavioral and Neurodevelopmental disorders	1089253	Alcohol-induced psychotic disorder without use disorder with onset during intoxication with de	ICD10CM	4	F10.251	F10.950,F10.250,F10.259,F10.251	Alcohol dependence with alcohol-induced psychotic disc	IMO	GHS-PACDC	IMO_2015_R	DELETED	Y	1900-01-01	9999-12-31	2015-07-10
Diagnosis: ICD10 Code, F00-F99 Define	F01-F99 Mental, Behavioral and Neurodevelopmental disorders	501321	Alcoholism with psychosis (HCC)	ICD10CM	1	F10.259		Alcohol dependence with alcohol-induced psychotic disc	IMO	GHS-PACDC	IMO_2015_R	ACTIVE	N	1900-01-01	2016-10-14	2015-07-10
Diagnosis: ICD10 Code, F00-F99 Define	F01-F99 Mental, Behavioral and Neurodevelopmental disorders	1106060	Alcohol-induced psychotic disorder with mild use disorder with onset during withdrawal withou	ICD10CM	2	F10.259	F10.230,F10.259	Alcohol dependence with alcohol-induced psychotic disc	IMO	GHS-PACDC	IMO_2016_R	ACTIVE	N	1900-01-01	9999-12-31	2016-06-02
Diagnosis: ICD10 Code, F00-F99 Define	F01-F99 Mental, Behavioral and Neurodevelopmental disorders	519313	Alcoholism with psychosis with complication (HCC)	ICD10CM	1	F10.259		Alcohol dependence with alcohol-induced psychotic disc	IMO	GHS-PACDC	IMO_2015_R	ACTIVE	N	1900-01-01	2016-10-14	2015-07-10
Diagnosis: ICD10 Code, F00-F99 Define	F01-F99 Mental, Behavioral and Neurodevelopmental disorders	973010	Alcohol-induced psychotic disorder with moderate or severe use disorder with onset during wit	ICD10CM	2	F10.259	F10.239,F10.259	Alcohol dependence with alcohol-induced psychotic disc	IMO	GHS-PACDC	IMO_2015_R	ACTIVE	N	2016-09-17	9999-12-31	2016-09-30
Diagnosis: ICD10 Code, F00-F99 Define	F01-F99 Mental, Behavioral and Neurodevelopmental disorders	1047415	Alcoholism with psychosis, with unspecified complication (HCC)	ICD10CM	1	F10.259		Alcohol dependence with alcohol-induced psychotic disc	IMO	GHS-PACDC	IMO_2015_R	ACTIVE	N	1900-01-01	2016-10-14	2015-07-10
Diagnosis: ICD10 Code, F00-F99 Define	F01-F99 Mental, Behavioral and Neurodevelopmental disorders	1365376	Alcohol dependence w alcoh-induce psychotic disorder, unsp (HCC)	ICD10CM	1	F10.259		Alcohol dependence with alcohol-induced psychotic disc	IMO	GHS-PACDC	IMO_2018_R	ACTIVE	N	1900-01-01	9999-12-31	2017-10-30
Diagnosis: ICD10 Code, F00-F99 Define	F01-F99 Mental, Behavioral and Neurodevelopmental disorders	1085970	Alcohol-induced psychotic disorder without use disorder with onset during intoxication with de	ICD10CM	3	F10.259	F10.959,F10.251,F10.259,F10.250	Alcohol dependence with alcohol-induced psychotic disc	IMO	GHS-PACDC	IMO_2015_R	DELETED	Y	1900-01-01	2016-05-07	2015-07-10
Diagnosis: ICD10 Code, F00-F99 Define	F01-F99 Mental, Behavioral and Neurodevelopmental disorders	486773	Mild alcohol-induced major neurocognitive disorder, nonamnestic confabulatory type, with mo	ICD10CM	1	F10.27		Alcohol dependence with alcohol-induced persisting der	IMO	GHS-PACDC	IMO_2015_R	ACTIVE	N	2016-09-17	9999-12-31	2016-09-30
Diagnosis: ICD10 Code, F00-F99 Define	F01-F99 Mental, Behavioral and Neurodevelopmental disorders	592572	Alcohol dependence with alcohol-induced persisting dementia	ICD10CM	1	F10.27		Alcohol dependence with alcohol-induced persisting der	IMO	GHS-PACDC	IMO_2015_R	ACTIVE	Y	1900-01-01	9999-12-31	2015-07-10

Diagnosis: ICD10 Code, F00-F99 Define	F01-F99 Mental, Behavioral and Neurodevelopmental disorders	2825	Dementia due to alcohol (HCC)	ICD10CM	1	F10.27		Alcohol dependence with alcohol-induced persisting der	IMO	GHS-PACDC	DX_LEGACY	ACTIVE	N	1900-01-01	9999-12-31	2015-07-10
Diagnosis: ICD10 Code, F00-F99 Define	F01-F99 Mental, Behavioral and Neurodevelopmental disorders	517664	Onset of alcohol-induced anxiety disorder during withdrawal without complication (HCC)	ICD10CM	1	F10.28(	F10.280,F10.230	Alcohol dependence with alcohol-induced anxiety disor(	IMO	GHS-PACDC	IMO_2015_R	ACTIVE	N	2017-09-17	9999-12-31	2017-10-30
Diagnosis: ICD10 Code, F00-F99 Define	F01-F99 Mental, Behavioral and Neurodevelopmental disorders	591936	Alcohol dependence with alcohol-induced anxiety disorder	ICD10CM	1	F10.280		Alcohol dependence with alcohol-induced anxiety disor(	IMO	GHS-PACDC	IMO_2015_R	ACTIVE	Y	1900-01-01	9999-12-31	2015-07-10
Diagnosis: ICD10 Code, F00-F99 Define	F01-F99 Mental, Behavioral and Neurodevelopmental disorders	429109	Onset of alcohol-induced sexual dysfunction during withdrawal (HCC)	ICD10CM	1	F10.281	F10.281,F10.239	Alcohol dependence with alcohol-induced sexual dysfun	IMO	GHS-PACDC	IMO_2015_R	ACTIVE	N	1900-01-01	9999-12-31	2015-07-10
Diagnosis: ICD10 Code, F00-F99 Define	F01-F99 Mental, Behavioral and Neurodevelopmental disorders	485829	Alcohol-induced sleep disorder with moderate or severe use disorder, mixed type (HCC)	ICD10CM	1	F10.282		Alcohol dependence with alcohol-induced sleep disorde	IMO	GHS-PACDC	IMO_2015_R	ACTIVE	N	2016-09-17	9999-12-31	2016-09-30
Diagnosis: ICD10 Code, F00-F99 Define	F01-F99 Mental, Behavioral and Neurodevelopmental disorders	633167	Alcohol dependence with other alcohol-induced disorder (HCC)	ICD10CM	1	F10.288		Alcohol dependence with other alcohol-induced disorde	IMO	GHS-PACDC	IMO_2015_R	ACTIVE	N	1900-01-01	9999-12-31	2015-07-10
Diagnosis: ICD10 Code, F00-F99 Define	F01-F99 Mental, Behavioral and Neurodevelopmental disorders	209578	Anemia due to alcoholism (HCC)	ICD10CM	1	F10.28:	F10.288,D64.89	Alcohol dependence with other alcohol-induced disorde	IMO	GHS-PACDC	DX_LEGACY	ACTIVE	N	2016-04-10	9999-12-31	2015-07-10
Diagnosis: ICD10 Code, F00-F99 Define	F01-F99 Mental, Behavioral and Neurodevelopmental disorders	516660	Idiosyncratic drug intoxication without complication (HCC)	ICD10CM	1	F10.920		Alcohol use, unspecified with intoxication, uncomplicate	IMO	GHS-PACDC	IMO_2015_R	ACTIVE	N	2016-04-10	9999-12-31	2015-07-10
Diagnosis: ICD10 Code, F00-F99 Define	F01-F99 Mental, Behavioral and Neurodevelopmental disorders	1103831	Alcohol-induced psychotic disorder with onset during intoxication with delusions and hallucinat	ICD10CM	3	F10.92(	F10.950,F10.951,F10.920	Alcohol use, unspecified with intoxication, uncomplicate	IMO	GHS-PACDC	IMO_2016_R	ACTIVE	N	1900-01-01	9999-12-31	2016-06-02
Diagnosis: ICD10 Code, F00-F99 Define	F01-F99 Mental, Behavioral and Neurodevelopmental disorders	961232	Alcohol intoxication, pathological, uncomplicated (HCC)	ICD10CM	1	F10.920		Alcohol use, unspecified with intoxication, uncomplicate	IMO	GHS-PACDC	IMO_2015_R	ACTIVE	N	1900-01-01	9999-12-31	2015-07-10
Diagnosis: ICD10 Code, F00-F99 Define	F01-F99 Mental, Behavioral and Neurodevelopmental disorders	1299129	Alcohol use with intoxication, with delirium (HCC)	ICD10CM	1	F10.921		Alcohol use, unspecified with intoxication delirium	IMO	GHS-PACDC	IMO_2017_R	ACTIVE	N	1900-01-01	9999-12-31	2017-04-14
Diagnosis: ICD10 Code, F00-F99 Define	F01-F99 Mental, Behavioral and Neurodevelopmental disorders	1007266	Pathological alcohol intoxication with complication, with delirium	ICD10CM	1	F10.921		Alcohol use, unspecified with intoxication delirium	IMO	GHS-PACDC	IMO_2015_R	DELETED	Y	1900-01-01	9999-12-31	2015-07-10
Diagnosis: ICD10 Code, F00-F99 Define	F01-F99 Mental, Behavioral and Neurodevelopmental disorders	486951	Acute, mixed level of activity, alcohol intoxication delirium with moderate or severe use disorde	ICD10CM	1	F10.921		Alcohol use, unspecified with intoxication delirium	IMO	GHS-PACDC	IMO_2015_R	ACTIVE	N	1900-01-01	2016-10-14	2015-07-10
Diagnosis: ICD10 Code, F00-F99 Define	F01-F99 Mental, Behavioral and Neurodevelopmental disorders	287470	Alcohol use with intoxication delirium (HCC)	ICD10CM	1	F10.921		Alcohol use, unspecified with intoxication delirium	IMO	GHS-PACDC	DX_LEGACY	ACTIVE	N	1900-01-01	9999-12-31	2015-07-10
Diagnosis: ICD10 Code, F00-F99 Define	F01-F99 Mental, Behavioral and Neurodevelopmental disorders	957797	Alcohol intoxication, pathological, with delirium (HCC)	ICD10CM	1	F10.921		Alcohol use, unspecified with intoxication delirium	IMO	GHS-PACDC	IMO_2015_R	ACTIVE	N	1900-01-01	9999-12-31	2015-07-10
Diagnosis: ICD10 Code, F00-F99 Define	F01-F99 Mental, Behavioral and Neurodevelopmental disorders	962917	Drug intoxication, idiosyncratic, with delirium (HCC)	ICD10CM	1	F10.921		Alcohol use, unspecified with intoxication delirium	IMO	GHS-PACDC	IMO_2015_R	ACTIVE	N	2017-09-17	9999-12-31	2017-10-30
Diagnosis: ICD10 Code, F00-F99 Define	F01-F99 Mental, Behavioral and Neurodevelopmental disorders	489396	Alcohol intoxication delirium, persistent, hypoactive (HCC)	ICD10CM	1	F10.921		Alcohol use, unspecified with intoxication delirium	IMO	GHS-PACDC	IMO_2015_R	ACTIVE	N	1900-01-01	9999-12-31	2015-07-10
Diagnosis: ICD10 Code, F00-F99 Define	F01-F99 Mental, Behavioral and Neurodevelopmental disorders	182839	Acute alcoholic intoxication (HCC)	ICD10CM	1	F10.929		Alcohol use, unspecified with intoxication, unspecified	IMO	GHS-PACDC	DX_LEGACY	ACTIVE	N	2017-09-17	9999-12-31	2017-10-30
Diagnosis: ICD10 Code, F00-F99 Define	F01-F99 Mental, Behavioral and Neurodevelopmental disorders	960723	Drug intoxication, idiosyncratic, with unspecified complication (HCC)	ICD10CM	1	F10.929		Alcohol use, unspecified with intoxication, unspecified	IMO	GHS-PACDC	IMO_2015_R	ACTIVE	N	2017-09-17	9999-12-31	2017-10-30
Diagnosis: ICD10 Code, F00-F99 Define	F01-F99 Mental, Behavioral and Neurodevelopmental disorders	516697	Idiosyncratic drug intoxication with perceptual disturbance (HCC)	ICD10CM	1	F10.929		Alcohol use, unspecified with intoxication, unspecified	IMO	GHS-PACDC	IMO_2015_R	ACTIVE	N	2017-09-17	9999-12-31	2017-10-30
Diagnosis: ICD10 Code, F00-F99 Define	F01-F99 Mental, Behavioral and Neurodevelopmental disorders	960754	Pathologic alcohol intoxication, with unspecified complication (HCC)	ICD10CM	1	F10.929		Alcohol use, unspecified with intoxication, unspecified	IMO	GHS-PACDC	IMO_2015_R	ACTIVE	N	1900-01-01	9999-12-31	2015-07-10
Diagnosis: ICD10 Code, F00-F99 Define	F01-F99 Mental, Behavioral and Neurodevelopmental disorders	516948	Onset of alcohol-induced psychotic disorder without use disorder during intoxication with hallu	ICD10CM	2	F10.92!	F10.951,F10.929	Alcohol use, unspecified with intoxication, unspecified	IMO	GHS-PACDC	IMO_2015_R	ACTIVE	N	2016-09-17	9999-12-31	2016-09-30
Diagnosis: ICD10 Code, F00-F99 Define	F01-F99 Mental, Behavioral and Neurodevelopmental disorders	42470	Alcohol idiosyncratic intoxication	ICD10CM	1	F10.929		Alcohol use, unspecified with intoxication, unspecified	IMO	GHS-PACDC	DX_LEGACY	DELETED	Y	1900-01-01	9999-12-31	2015-07-10
Diagnosis: ICD10 Code, F00-F99 Define	F01-F99 Mental, Behavioral and Neurodevelopmental disorders	971931	Onset of alcohol-induced psychotic disorder during intoxication, with delusions (HCC)	ICD10CM	2	F10.92!	F10.950,F10.929	Alcohol use, unspecified with intoxication, unspecified	IMO	GHS-PACDC	IMO_2015_R	ACTIVE	N	2017-03-31	9999-12-31	2017-04-14
Diagnosis: ICD10 Code, F00-F99 Define	F01-F99 Mental, Behavioral and Neurodevelopmental disorders	973641	Onset of alcohol-induced psychotic disorder during intoxication, with hallucinations (HCC)	ICD10CM	2	F10.92!	F10.951,F10.929	Alcohol use, unspecified with intoxication, unspecified	IMO	GHS-PACDC	IMO_2015_R	ACTIVE	N	2017-03-31	9999-12-31	2017-04-14
Diagnosis: ICD10 Code, F00-F99 Define	F01-F99 Mental, Behavioral and Neurodevelopmental disorders	30081	Extreme sensitivity to alcohol syndrome (HCC)	ICD10CM	1	F10.929		Alcohol use, unspecified with intoxication, unspecified	IMO	GHS-PACDC	DX_LEGACY	ACTIVE	N	1900-01-01	9999-12-31	2015-07-10
Diagnosis: ICD10 Code, F00-F99 Define	F01-F99 Mental, Behavioral and Neurodevelopmental disorders	961945	Acute alcoholic intoxication, episodic drinking behavior, with unspecified complication (HCC)	ICD10CM	1	F10.929		Alcohol use, unspecified with intoxication, unspecified	IMO	GHS-PACDC	IMO_2015_R	ACTIVE	N	2017-09-17	9999-12-31	2017-10-30
Diagnosis: ICD10 Code, F00-F99 Define	F01-F99 Mental, Behavioral and Neurodevelopmental disorders	955780	Acute alcohol overingestion, with unspecified complication (HCC)	ICD10CM	1	F10.929		Alcohol use, unspecified with intoxication, unspecified	IMO	GHS-PACDC	IMO_2015_R	ACTIVE	N	2017-09-17	9999-12-31	2017-10-30
Diagnosis: ICD10 Code, F00-F99 Define	F01-F99 Mental, Behavioral and Neurodevelopmental disorders	516973	Alcohol-induced psychotic disorder with onset during intoxication with complication (HCC)	ICD10CM	1	F10.92!	F10.929,F10.959	Alcohol use, unspecified with intoxication, unspecified	IMO	GHS-PACDC	IMO_2015_R	ACTIVE	N	2016-04-10	9999-12-31	2015-07-10
Diagnosis: ICD10 Code, F00-F99 Define	F01-F99 Mental, Behavioral and Neurodevelopmental disorders	963949	Episodic acute alcoholic intoxication, with unspecified complication (HCC)	ICD10CM	1	F10.929		Alcohol use, unspecified with intoxication, unspecified	IMO	GHS-PACDC	IMO_2015_R	ACTIVE	N	2017-09-17	9999-12-31	2017-10-30
Diagnosis: ICD10 Code, F00-F99 Define	F01-F99 Mental, Behavioral and Neurodevelopmental disorders	30082	Pathological alcohol intoxication (HCC)	ICD10CM	1	F10.929		Alcohol use, unspecified with intoxication, unspecified	IMO	GHS-PACDC	DX_LEGACY	ACTIVE	N	1900-01-01	9999-12-31	2015-07-10
Diagnosis: ICD10 Code, F00-F99 Define	F01-F99 Mental, Behavioral and Neurodevelopmental disorders	1023089	Pathological alcohol intoxication with complication, with unspecified complication	ICD10CM	1	F10.929		Alcohol use, unspecified with intoxication, unspecified	IMO	GHS-PACDC	IMO_2015_R	DELETED	Y	1900-01-01	9999-12-31	2015-07-10
Diagnosis: ICD10 Code, F00-F99 Define	F01-F99 Mental, Behavioral and Neurodevelopmental disorders	1103866	Alcohol-induced psychotic disorder without use disorder with onset during intoxication with ha	ICD10CM	2	F10.92!	F10.951,F10.929	Alcohol use, unspecified with intoxication, unspecified	IMO	GHS-PACDC	IMO_2016_R	ACTIVE	N	1900-01-01	9999-12-31	2016-06-02
Diagnosis: ICD10 Code, F00-F99 Define	F01-F99 Mental, Behavioral and Neurodevelopmental disorders	489104	Alcohol-induced bipolar disorder with onset during intoxication (HCC)	ICD10CM	1	F10.94		Alcohol use, unspecified with alcohol-induced mood dis(	IMO	GHS-PACDC	IMO_2015_R	ACTIVE	N	1900-01-01	9999-12-31	2015-07-10
Diagnosis: ICD10 Code, F00-F99 Define	F01-F99 Mental, Behavioral and Neurodevelopmental disorders	428804	Alcohol-induced bipolar and related disorder with moderate or severe use disorder (HCC)	ICD10CM	1	F10.94		Alcohol use, unspecified with alcohol-induced mood dis(	IMO	GHS-PACDC	IMO_2015_R	ACTIVE	N	2016-04-10	2016-10-14	2015-07-10
Diagnosis: ICD10 Code, F00-F99 Define	F01-F99 Mental, Behavioral and Neurodevelopmental disorders	428808	Alcohol-induced depressive disorder without use disorder (HCC)	ICD10CM	1	F10.94		Alcohol use, unspecified with alcohol-induced mood dis(	IMO	GHS-PACDC	IMO_2015_R	ACTIVE	N	1900-01-01	9999-12-31	2015-07-10
Diagnosis: ICD10 Code, F00-F99 Define	F01-F99 Mental, Behavioral and Neurodevelopmental disorders	963558	Alcohol use with alcohol-induced psychotic disorder, with delusions (HCC)	ICD10CM	1	F10.950		Alcohol use, unspecified with alcohol-induced psychotic	IMO	GHS-PACDC	IMO_2015_R	ACTIVE	N	1900-01-01	9999-12-31	2015-07-10
Diagnosis: ICD10 Code, F00-F99 Define	F01-F99 Mental, Behavioral and Neurodevelopmental disorders	1103831	Alcohol-induced psychotic disorder with onset during intoxication with delusions and hallucinat	ICD10CM	1	F10.95(	F10.950,F10.951,F10.920	Alcohol use, unspecified with alcohol-induced psychotic	IMO	GHS-PACDC	IMO_2016_R	ACTIVE	N	1900-01-01	9999-12-31	2016-06-02
Diagnosis: ICD10 Code, F00-F99 Define	F01-F99 Mental, Behavioral and Neurodevelopmental disorders	1089767	Alcohol-induced psychotic disorder with mild use disorder with complication, with delusions (H(	ICD10CM	1	F10.950		Alcohol use, unspecified with alcohol-induced psychotic	IMO	GHS-PACDC	IMO_2015_R	ACTIVE	N	1900-01-01	2016-10-14	2015-07-10
Diagnosis: ICD10 Code, F00-F99 Define	F01-F99 Mental, Behavioral and Neurodevelopmental disorders	978769	Alcohol-induced psychosis with complication, with delusions (HCC)	ICD10CM	1	F10.950		Alcohol use, unspecified with alcohol-induced psychotic	IMO	GHS-PACDC	IMO_2015_R	ACTIVE	N	1900-01-01	9999-12-31	2015-07-10
Diagnosis: ICD10 Code, F00-F99 Define	F01-F99 Mental, Behavioral and Neurodevelopmental disorders	1087504	Alcohol-induced psychotic disorder with mild use disorder with onset during intoxication with c	ICD10CM	1	F10.950		Alcohol use, unspecified with alcohol-induced psychotic	IMO	GHS-PACDC	IMO_2015_R	ACTIVE	N	1900-01-01	2016-10-14	2015-07-10
Diagnosis: ICD10 Code, F00-F99 Define	F01-F99 Mental, Behavioral and Neurodevelopmental disorders	974880	Alcohol-induced psychotic disorder with mild use disorder with onset during intoxication, with (	ICD10CM	1	F10.950		Alcohol use, unspecified with alcohol-induced psychotic	IMO	GHS-PACDC	IMO_2015_R	ACTIVE	N	1900-01-01	2016-10-14	2015-07-10
Diagnosis: ICD10 Code, F00-F99 Define	F01-F99 Mental, Behavioral and Neurodevelopmental disorders	960190	Chronic alcoholism with psychosis, with delusions (HCC)	ICD10CM	1	F10.950		Alcohol use, unspecified with alcohol-induced psychotic	IMO	GHS-PACDC	IMO_2015_R	ACTIVE	N	1900-01-01	9999-12-31	2015-07-10
Diagnosis: ICD10 Code, F00-F99 Define	F01-F99 Mental, Behavioral and Neurodevelopmental disorders	972081	Alcohol-induced psychotic disorder without use disorder with onset during intoxication, with ha	ICD10CM	1	F10.951	F10.951,F10.929	Alcohol use, unspecified with alcohol-induced psychotic	IMO	GHS-PACDC	IMO_2015_R	ACTIVE	N	2016-09-17	9999-12-31	2016-09-30
Diagnosis: ICD10 Code, F00-F99 Define	F01-F99 Mental, Behavioral and Neurodevelopmental disorders	1082067	Alcoholism with psychosis with complication, with hallucinations (HCC)	ICD10CM	1	F10.951		Alcohol use, unspecified with alcohol-induced psychotic	IMO	GHS-PACDC	IMO_2015_R	ACTIVE	N	2016-09-17	9999-12-31	2016-09-30
Diagnosis: ICD10 Code, F00-F99 Define	F01-F99 Mental, Behavioral and Neurodevelopmental disorders	963158	Alcohol use with alcohol-induced psychotic disorder, with hallucinations (HCC)	ICD10CM	1	F10.951		Alcohol use, unspecified with alcohol-induced psychotic	IMO	GHS-PACDC	IMO_2015_R	ACTIVE	N	1900-01-01	9999-12-31	2015-07-10
Diagnosis: ICD10 Code, F00-F99 Define	F01-F99 Mental, Behavioral and Neurodevelopmental disorders	975801	Alcohol-induced psychotic disorder with onset during intoxication, with hallucinations (HCC)	ICD10CM	1	F10.951		Alcohol use, unspecified with alcohol-induced psychotic	IMO	GHS-PACDC	IMO_2015_R	ACTIVE	N	1900-01-01	2017-04-27	2015-07-10
Diagnosis: ICD10 Code, F00-F99 Define	F01-F99 Mental, Behavioral and Neurodevelopmental disorders	975167	Alcohol-induced psychotic disorder without use disorder with onset during withdrawal, with un	ICD10CM	1	F10.959		Alcohol use, unspecified with alcohol-induced psychotic	IMO	GHS-PACDC	IMO_2015_R	ACTIVE	N	1900-01-01	2016-05-07	2015-07-10
Diagnosis: ICD10 Code, F00-F99 Define	F01-F99 Mental, Behavioral and Neurodevelopmental disorders	1082925	Alcohol-induced psychotic disorder without use disorder with hallucinations, with unspecified c	ICD10CM	1	F10.95!	F10.959,F10.150,F10.159,F10.151	Alcohol use, unspecified with alcohol-induced psychotic	IMO	GHS-PACDC	IMO_2015_R	DELETED	Y	1900-01-01	9999-12-31	2015-07-10
Diagnosis: ICD10 Code, F00-F99 Define	F01-F99 Mental, Behavioral and Neurodevelopmental disorders	1088533	Alcohol-induced psychotic disorder with mild use disorder with onset during withdrawal with c(	ICD10CM	1	F10.959		Alcohol use, unspecified with alcohol-induced psychotic	IMO	GHS-PACDC	IMO_2015_R	ACTIVE	N	1900-01-01	2016-05-07	2015-07-10
Diagnosis: ICD10 Code, F00-F99 Define	F01-F99 Mental, Behavioral and Neurodevelopmental disorders	487081	Alcohol-induced psychotic disorder without use disorder with onset during intoxication (HCC)	ICD10CM	2	F10.95!	F10.929,F10.959	Alcohol use, unspecified with alcohol-induced psychotic	IMO	GHS-PACDC	IMO_2015_R	ACTIVE	N	2016-04-10	2016-10-14	2015-07-10
Diagnosis: ICD10 Code, F00-F99 Define	F01-F99 Mental, Behavioral and Neurodevelopmental disorders	958878	Alcohol use with alcohol-induced psychotic disorder, with unspecified complication (HCC)	ICD10CM	1	F10.959		Alcohol use, unspecified with alcohol-induced psychotic	IMO	GHS-PACDC	IMO_2015_R	ACTIVE	N	1900-01-01	9999-12-31	2015-07-10
Diagnosis: ICD10 Code, F00-F99 Define	F01-F99 Mental, Behavioral and Neurodevelopmental disorders	488601	Persistent alcohol-induced major neurocognitive disorder, amnestic-confabulatory type (HCC)	ICD10CM	1	F10.96		Alcohol use, unspecified with alcohol-induced persisting	IMO	GHS-PACDC	IMO_2015_R	ACTIVE	N	1900-01-01	9999-12-31	2015-07-10
Diagnosis: ICD10 Code, F00-F99 Define	F01-F99 Mental, Behavioral and Neurodevelopmental disorders	27945	Amnestic syndrome of Wernicke's disease (HCC)	ICD10CM	1	F10.96		Alcohol use, unspecified with alcohol-induced persisting	IMO	GHS-PACDC	DX_LEGACY	ACTIVE	N	1900-01-01	9999-12-31	2015-07-10
Diagnosis: ICD10 Code, F00-F99 Define	F01-F99 Mental, Behavioral and Neurodevelopmental disorders	1109489	Dementia associated with alcoholism with behavioral disturbance (HCC)	ICD10CM	2	F10.97	F03.91,F10.97	Alcohol use, unspecified with alcohol-induced persisting	IMO	GHS-PACDC	IMO_2016_R	ACTIVE	N	1900-01-01	9999-12-31	2016-06-02
Diagnosis: ICD10 Code, F00-F99 Define	F01-F99 Mental, Behavioral and Neurodevelopmental disorders	614912	Alcohol use, unspecified with other alcohol-induced disorders	ICD10CM	1	F10.98		Alcohol use, unspecified with other alcohol-induced disc	IMO	GHS-PACDC	IMO_2015_R	ACTIVE	Y	1900-01-01	9999-12-31	2015-07-10
Diagnosis: ICD10 Code, F00-F99 Define	F01-F99 Mental, Behavioral and Neurodevelopmental disorders	485853	Alcohol-induced anxiety disorder with moderate or severe use disorder with onset during withc	ICD10CM	1	F10.980		Alcohol use, unspecified with alcohol-induced anxiety di	IMO	GHS-PACDC	IMO_2015_R	ACTIVE	N	1900-01-01	2016-10-14	2015-07-10
Diagnosis: ICD10 Code, F00-F99 Define	F01-F99 Mental, Behavioral and Neurodevelopmental disorders	486683	Alcohol-induced sexual dysfunction without use disorder with onset during withdrawal (HCC)	ICD10CM	1	F10.981		Alcohol use, unspecified with alcohol-induced sexual dy:	IMO	GHS-PACDC	IMO_2015_R	ACTIVE	N	1900-01-01	2016-10-14	2015-07-10
Diagnosis: ICD10 Code, F00-F99 Define	F01-F99 Mental, Behavioral and Neurodevelopmental disorders	258988	Parasomnia due to alcohol (HCC)	ICD10CM	1	F10.982		Alcohol use, unspecified with alcohol-induced sleep disc	IMO	GHS-PACDC	DX_LEGACY	ACTIVE	N	1900-01-01	9999-12-31	2015-07-10
Diagnosis: ICD10 Code, F00-F99 Define	F01-F99 Mental, Behavioral and Neurodevelopmental disorders	144235	Alcohol induced hypersomnia (HCC)	ICD10CM	1	F10.982		Alcohol use, unspecified with alcohol-induced sleep disc	IMO	GHS-PACDC	DX_LEGACY	ACTIVE	N	1900-01-01	9999-12-31	2015-07-10
Diagnosis: ICD10 Code, F00-F99 Define	F01-F99 Mental, Behavioral and Neurodevelopmental disorders	488874	Alcohol-induced sleep disorder, mixed type, with onset during discontinuation/withdrawal (HC(	ICD10CM	1	F10.982		Alcohol use, unspecified with alcohol-induced sleep disc	IMO	GHS-PACDC	IMO_2015_R	ACTIVE	N	1900-01-01	9999-12-31	2015-07-10
Diagnosis: ICD10 Code, F00-F99 Define	F01-F99 Mental, Behavioral and Neurodevelopmental disorders	259570	Unspecified alcohol induced mental disorders	ICD10CM	1	F10.988		Alcohol use, unspecified with other alcohol-induced disc	IMO	GHS-PACDC	DX_LEGACY	ACTIVE	Y	2016-04-10	9999-12-31	2015-07-10
Diagnosis: ICD10 Code, F00-F99 Define	F01-F99 Mental, Behavioral and Neurodevelopmental disorders	140786	Other specified alcohol-induced mental disorders	ICD10CM	1	F10.988		Alcohol use, unspecified with other alcohol-induced disc	IMO	GHS-PACDC	DX_LEGACY	ACTIVE	Y	1900-01-01	9999-12-31	2017-04-14
Diagnosis: ICD10 Code, F00-F99 Define	F01-F99 Mental, Behavioral and Neurodevelopmental disorders	517728	Alcohol abuse affecting pregnancy, antepartum	ICD10CM	2	F10.99	O99.310,F10.99	Alcohol use, unspecified with unspecified alcohol-induce	IMO	GHS-PACDC	IMO_2015_R	ACTIVE	N	1900-01-01	2016-10-14	2015-07-10
Diagnosis: ICD10 Code, F00-F99 Define	F01-F99 Mental, Behavioral and Neurodevelopmental disorders	1101947	Alcohol use disorder (HCC)	ICD10CM	1	F10.99		Alcohol use, unspecified with unspecified alcohol-induce	IMO	GHS-PACDC	IMO_2016_R	ACTIVE	N	1900-01-01	9999-12-31	2016-06-02
Diagnosis: ICD10 Code, F00-F99 Define	F01-F99 Mental, Behavioral and Neurodevelopmental disorders	485196	Significant use of alcohol	ICD10CM	1	F10.99		Alcohol use, unspecified with unspecified alcohol-induce	IMO	GHS-PACDC	IMO_2015_R	ACTIVE	N	1900-01-01	2016-05-07	2015-07-10
Diagnosis: ICD10 Code, F00-F99 Define	F01-F99 Mental, Behavioral and Neurodevelopmental disorders	423771	Current drinker	ICD10CM	1	F10.99		Alcohol use, unspecified with unspecified alcohol-induce	IMO	GHS-PACDC	IMO_2015_R	ACTIVE	N	1900-01-01	2016-05-07	2015-07-10
Diagnosis: ICD10 Code, F00-F99 Define	F01-F99 Mental, Behavioral and Neurodevelopmental disorders	492164	Lomotil use disorder, mild, in controlled environment	ICD10CM	1	F11.10		Opioid abuse, uncomplicated	IMO	GHS-PACDC	IMO_2015_R	ACTIVE	N	2016-09-17	9999-12-31	2016-09-30
Diagnosis: ICD10 Code, F00-F99 Define	F01-F99 Mental, Behavioral and Neurodevelopmental disorders	510024	Darvocet use disorder, mild, in sustained remission, abuse	ICD10CM	1	F11.10		Opioid abuse, uncomplicated	IMO	GHS-PACDC	IMO_2015_R	ACTIVE	N	1900-01-01	2017-10-14	2015-07-10
Diagnosis: ICD10 Code, F00-F99 Define	F01-F99 Mental, Behavioral and Neurodevelopmental disorders	484981	Mild butorphanol tartrate use disorder on maintenance therapy	ICD10CM	1	F11.10		Opioid abuse, uncomplicated	IMO	GHS-PACDC	IMO_2015_R	ACTIVE	N	2016-09-17	9999-12-31	2016-09-30
Diagnosis: ICD10 Code, F00-F99 Define	F01-F99 Mental, Behavioral and Neurodevelopmental disorders	507573	Methadone use disorder, mild, in sustained remission, abuse	ICD10CM	1	F11.10		Opioid abuse, uncomplicated	IMO	GHS-PACDC	IMO_2015_R	ACTIVE	N	1900-01-01	2017-10-14	2015-07-10
Diagnosis: ICD10 Code, F00-F99 Define	F01-F99 Mental, Behavioral and Neurodevelopmental disorders	484903	Mild codeine use disorder in controlled environment	ICD10CM	1	F11.10		Opioid abuse, uncomplicated	IMO	GHS-PACDC	IMO_2015_R	ACTIVE	N	2016-09-17	9999-12-31	2016-09-30
Diagnosis: ICD10 Code, F00-F99 Define	F01-F99 Mental, Behavioral and Neurodevelopmental disorders	486339	Mild opioid use disorder, in sustained remission	ICD10CM	1	F11.10		Opioid abuse, uncomplicated	IMO	GHS-PACDC	IMO_2015_R	ACTIVE	N	2016-09-17	9999-12-31	2016-09-30
Diagnosis: ICD10 Code, F00-F99 Define	F01-F99 Mental, Behavioral and Neurodevelopmental disorders	484650	Mild nalbuphine hydrochloride use disorder in early remission	ICD10CM	1	F11.10		Opioid abuse, uncomplicated	IMO	GHS-PACDC	IMO_2015_R	ACTIVE	N	2016-09-17	9999-12-31	2016-09-30
Diagnosis: ICD10 Code, F00-F99 Define	F01-F99 Mental, Behavioral and Neurodevelopmental disorders	484831	Mild heroin use disorder in early remission	ICD10CM	1	F11.10		Opioid abuse, uncomplicated	IMO	GHS-PACDC	IMO_2015_R	ACTIVE	N	2016-09-17	9999-12-31	2016-09-30
Diagnosis: ICD10 Code, F00-F99 Define	F01-F99 Mental, Behavioral and Neurodevelopmental disorders	507867	Vicodin use disorder, mild, in controlled environment, abuse	ICD10CM	1	F11.10		Opioid abuse, uncomplicated	IMO	GHS-PACDC	IMO_2015_R	ACTIVE	N	1900-01-01	9999-12-31	2015-07-10
Diagnosis: ICD10 Code, F00-F99 Define	F01-F99 Mental, Behavioral and Neurodevelopmental disorders	483942	Mild propoxyphene use disorder	ICD10CM	1	F11.10		Opioid abuse, uncomplicated	IMO	GHS-PACDC	IMO_2015_R	ACTIVE	N	2016-09-17	9999-12-31	2016-09-30
Diagnosis: ICD10 Code, F00-F99 Define	F01-F99 Mental, Behavioral and Neurodevelopmental disorders	507355	Mild tramadol abuse in controlled environment	ICD10CM	1	F11.10		Opioid abuse, uncomplicated	IMO	GHS-PACDC	IMO_2015_R	ACTIVE	N	1900-01-01	9999-12-31	2015-07-10
Diagnosis: ICD10 Code, F00-F99 Define	F01-F99 Mental, Behavioral and Neurodevelopmental disorders	492464	Talwin use disorder, mild, on maintenance therapy	ICD10CM	1	F11.10		Opioid abuse, uncomplicated	IMO	GHS-PACDC	IMO_2015_R	ACTIVE	N	2016-09-17	9999-12-31	2016-09-30
Diagnosis: ICD10 Code, F00-F99 Define	F01-F99 Mental, Behavioral and Neurodevelopmental disorders	508933	Mild opium abuse in early remission in controlled environment	ICD10CM	1	F11.10		Opioid abuse, uncomplicated	IMO	GHS-PACDC	IMO_2015_R	ACTIVE	N	1900-01-01	2017-10-14	2015-07-10
Diagnosis: ICD10 Code, F00-F99 Define	F01-F99 Mental, Behavioral and Neurodevelopmental disorders	509856	Mild tramadol abuse in sustained remission in controlled environment	ICD10CM	1	F11.10		Opioid abuse, uncomplicated	IMO	GHS-PACDC	IMO_2015_R	ACTIVE	N	1900-01-01	2017-10-14	2015-07-10
Diagnosis: ICD10 Code, F00-F99 Define	F01-F99 Mental, Behavioral and Neurodevelopmental disorders	507563	Mild tramadol abuse in sustained remission on maintenance therapy	ICD10CM	1	F11.10		Opioid abuse, uncomplicated	IMO	GHS-PACDC	IMO_2015_R	ACTIVE	N	1900-01-01	2017-10-14	2015-07-10
Diagnosis: ICD10 Code, F00-F99 Define	F01-F99 Mental, Behavioral and Neurodevelopmental disorders	484389	Severe tramadol use disorder on maintenance therapy (HCC)	ICD10CM	1	F11.10		Opioid abuse, uncomplicated	IMO	GHS-PACDC	IMO_2015_R	ACTIVE	N	1900-01-01	2016-10-14	2015-07-10
Diagnosis: ICD10 Code, F00-F99 Define	F01-F99 Mental, Behavioral and Neurodevelopmental disorders	509628	Mild heroin abuse	ICD10CM	1	F11.10		Opioid abuse, uncomplicated	IMO	GHS-PACDC	IMO_2015_R	ACTIVE	N	1900-01-01	9999-12-31	2015-07-10
Diagnosis: ICD10 Code, F00-F99 Define	F01-F99 Mental, Behavioral and Neurodevelopmental disorders	509141	Nubain use disorder, mild, abuse	ICD10CM	1	F11.10		Opioid abuse, uncomplicated	IMO	GHS-PACDC	IMO_2015_R	ACTIVE	N	1900-01-01	9999-12-31	2015-07-10
Diagnosis: ICD10 Code, F00-F99 Define	F01-F99 Mental, Behavioral and Neurodevelopmental disorders	507812	Mild continuous-release oxycodone abuse in early remission in controlled environment	ICD10CM	1	F11.10		Opioid abuse, uncomplicated	IMO	GHS-PACDC	IMO_2015_R	ACTIVE	N	1900-01-01	2017-10-14	2015-07-10
Diagnosis: ICD10 Code, F00-F99 Define	F01-F99 Mental, Behavioral and Neurodevelopmental disorders	507143	Morphine use disorder, mild, on maintenance therapy, abuse	ICD10CM	1	F11.10		Opioid abuse, uncomplicated	IMO	GHS-PACDC	IMO_2015_R	ACTIVE	N	1900-01-01	9999-12-31	2015-07-10
Diagnosis: ICD10 Code, F00-F99 Define	F01-F99 Mental, Behavioral and Neurodevelopmental disorders	509997	Darvocet use disorder, mild, in controlled environment, abuse	ICD10CM	1	F11.10		Opioid abuse, uncomplicated	IMO	GHS-PACDC	IMO_2015_R	ACTIVE	N	1900-01-01	9999-12-31	2015-07-10
Diagnosis: ICD10 Code, F00-F99 Define	F01-F99 Mental, Behavioral and Neurodevelopmental disorders	507452	Talwin use disorder, mild, in sustained remission, on maintenance therapy, abuse	ICD10CM	1	F11.10		Opioid abuse, uncomplicated	IMO	GHS-PACDC	IMO_2015_R	ACTIVE	N	1900-01-01	2017-10-14	2015-07-10
Diagnosis: ICD10 Code, F00-F99 Define	F01-F99 Mental, Behavioral and Neurodevelopmental disorders	508831	Dextromethorphan use disorder, mild, in sustained remission, abuse	ICD10CM	1	F11.10		Opioid abuse, uncomplicated	IMO	GHS-PACDC	IMO_2015_R	ACTIVE	N	1900-01-01	2016-10-14	2015-07-10
Diagnosis: ICD10 Code, F00-F99 Define	F01-F99 Mental, Behavioral and Neurodevelopmental disorders	509531	Mild acetaminophen-hydrocodone abuse in early remission	ICD10CM	1	F11.10		Opioid abuse, uncomplicated	IMO	GHS-PACDC	IMO_2015_R	ACTIVE	N	1900-01-01	2017-10-14	2015-07-10
Diagnosis: ICD10 Code, F00-F99 Define	F01-F99 Mental, Behavioral and Neurodevelopmental disorders	484365	Mild propoxyphene use disorder on maintenance therapy	ICD10CM	1	F11.10		Opioid abuse, uncomplicated	IMO	GHS-PACDC	IMO_2015_R	ACTIVE	N	2016-09-17	9999-12-31	2016-09-30
Diagnosis: ICD10 Code, F00-F99 Define	F01-F99 Mental, Behavioral and Neurodevelopmental disorders	508069	Morphine use disorder, mild, in controlled environment, abuse	ICD10CM	1	F11.10		Opioid abuse, uncomplicated	IMO	GHS-PACDC	IMO_2015_R	ACTIVE	N	1900-01-01	9999-12-31	2015-07-10
Diagnosis: ICD10 Code, F00-F99 Define	F01-F99 Mental, Behavioral and Neurodevelopmental disorders	509801	Opioid use disorder, mild, on maintenance therapy, abuse	ICD10CM	1	F11.10		Opioid abuse, uncomplicated	IMO	GHS-PACDC	IMO_2015_R	ACTIVE	N	1900-01-01	9999-12-31	2015-07-10
Diagnosis: ICD10 Code, F00-F99 Define	F01-F99 Mental, Behavioral and Neurodevelopmental disorders	509700	Mild methadone abuse on maintenance therapy	ICD10CM	1	F11.10		Opioid abuse, uncomplicated	IMO	GHS-PACDC	IMO_2015_R	ACTIVE	N	1900-01-01	9999-12-31	2015-07-10

Diagnosis: ICD10 Code, F00-F99 Define	F01-F99 Mental, Behavioral and Neurodevelopmental disorders	483945	Heroin use disorder, mild	ICD10CM	1	F11.10		Opioid abuse, uncomplicated	IMO	GHS-PACDC	IMO_2015_R	ACTIVE	N	2016-09-17	9999-12-31	2016-09-30
Diagnosis: ICD10 Code, F00-F99 Define	F01-F99 Mental, Behavioral and Neurodevelopmental disorders	509642	Darvon use disorder, mild, in controlled environment, abuse	ICD10CM	1	F11.10		Opioid abuse, uncomplicated	IMO	GHS-PACDC	IMO_2015_R	ACTIVE	N	1900-01-01	9999-12-31	2015-07-10
Diagnosis: ICD10 Code, F00-F99 Define	F01-F99 Mental, Behavioral and Neurodevelopmental disorders	492424	Dilaudid use disorder, mild, on maintenance therapy	ICD10CM	1	F11.10		Opioid abuse, uncomplicated	IMO	GHS-PACDC	IMO_2015_R	ACTIVE	N	2016-09-17	9999-12-31	2016-09-30
Diagnosis: ICD10 Code, F00-F99 Define	F01-F99 Mental, Behavioral and Neurodevelopmental disorders	509957	Opium use disorder, mild, in early remission, in controlled environment, abuse	ICD10CM	1	F11.10		Opioid abuse, uncomplicated	IMO	GHS-PACDC	IMO_2015_R	ACTIVE	N	1900-01-01	2017-10-14	2015-07-10
Diagnosis: ICD10 Code, F00-F99 Define	F01-F99 Mental, Behavioral and Neurodevelopmental disorders	39266	Opioid abuse, episodic use	ICD10CM	1	F11.10		Opioid abuse, uncomplicated	IMO	GHS-PACDC	DX_LEGACY	ACTIVE	N	1900-01-01	9999-12-31	2015-07-10
Diagnosis: ICD10 Code, F00-F99 Define	F01-F99 Mental, Behavioral and Neurodevelopmental disorders	484211	Mild oxycodone-acetaminophen use disorder on maintenance therapy	ICD10CM	1	F11.10		Opioid abuse, uncomplicated	IMO	GHS-PACDC	IMO_2015_R	ACTIVE	N	2016-09-17	9999-12-31	2016-09-30
Diagnosis: ICD10 Code, F00-F99 Define	F01-F99 Mental, Behavioral and Neurodevelopmental disorders	509116	Mild oxycodone abuse in early remission in controlled environment	ICD10CM	1	F11.10		Opioid abuse, uncomplicated	IMO	GHS-PACDC	IMO_2015_R	ACTIVE	N	1900-01-01	2017-10-14	2015-07-10
Diagnosis: ICD10 Code, F00-F99 Define	F01-F99 Mental, Behavioral and Neurodevelopmental disorders	484938	Mild nalbuphine hydrochloride use disorder on maintenance therapy	ICD10CM	1	F11.10		Opioid abuse, uncomplicated	IMO	GHS-PACDC	IMO_2015_R	ACTIVE	N	2016-09-17	9999-12-31	2016-09-30
Diagnosis: ICD10 Code, F00-F99 Define	F01-F99 Mental, Behavioral and Neurodevelopmental disorders	507870	Opium use disorder, mild, in sustained remission, in controlled environment, abuse	ICD10CM	1	F11.10		Opioid abuse, uncomplicated	IMO	GHS-PACDC	IMO_2015_R	ACTIVE	N	1900-01-01	2017-10-14	2015-07-10
Diagnosis: ICD10 Code, F00-F99 Define	F01-F99 Mental, Behavioral and Neurodevelopmental disorders	508055	Oxycontin use disorder, mild, in early remission, abuse	ICD10CM	1	F11.11		Opioid abuse, in remission	IMO	GHS-PACDC	IMO_2015_R	ACTIVE	N	2017-09-17	9999-12-31	2017-10-30
Diagnosis: ICD10 Code, F00-F99 Define	F01-F99 Mental, Behavioral and Neurodevelopmental disorders	509856	Mild tramadol abuse in sustained remission in controlled environment	ICD10CM	1	F11.11		Opioid abuse, in remission	IMO	GHS-PACDC	IMO_2015_R	ACTIVE	N	2017-09-17	9999-12-31	2017-10-30
Diagnosis: ICD10 Code, F00-F99 Define	F01-F99 Mental, Behavioral and Neurodevelopmental disorders	508922	Vicodin use disorder, mild, in early remission, in controlled environment, abuse	ICD10CM	1	F11.11		Opioid abuse, in remission	IMO	GHS-PACDC	IMO_2015_R	ACTIVE	N	2017-09-17	9999-12-31	2017-10-30
Diagnosis: ICD10 Code, F00-F99 Define	F01-F99 Mental, Behavioral and Neurodevelopmental disorders	507474	Lortab use disorder, mild, in sustained remission, in controlled environment, abuse	ICD10CM	1	F11.11		Opioid abuse, in remission	IMO	GHS-PACDC	IMO_2015_R	ACTIVE	N	2017-09-17	9999-12-31	2017-10-30
Diagnosis: ICD10 Code, F00-F99 Define	F01-F99 Mental, Behavioral and Neurodevelopmental disorders	508523	Mild hydromorphone abuse in sustained remission	ICD10CM	1	F11.11		Opioid abuse, in remission	IMO	GHS-PACDC	IMO_2015_R	ACTIVE	N	2017-09-17	9999-12-31	2017-10-30
Diagnosis: ICD10 Code, F00-F99 Define	F01-F99 Mental, Behavioral and Neurodevelopmental disorders	507734	Mild nalbuphine hydrochloride abuse in early remission on maintenance therapy	ICD10CM	1	F11.11		Opioid abuse, in remission	IMO	GHS-PACDC	IMO_2015_R	ACTIVE	N	2017-09-17	9999-12-31	2017-10-30
Diagnosis: ICD10 Code, F00-F99 Define	F01-F99 Mental, Behavioral and Neurodevelopmental disorders	507812	Mild continuous-release oxycodone abuse in early remission in controlled environment	ICD10CM	1	F11.11		Opioid abuse, in remission	IMO	GHS-PACDC	IMO_2015_R	ACTIVE	N	2017-09-17	9999-12-31	2017-10-30
Diagnosis: ICD10 Code, F00-F99 Define	F01-F99 Mental, Behavioral and Neurodevelopmental disorders	508148	Mild tramadol abuse in early remission	ICD10CM	1	F11.11		Opioid abuse, in remission	IMO	GHS-PACDC	IMO_2015_R	ACTIVE	N	2017-09-17	9999-12-31	2017-10-30
Diagnosis: ICD10 Code, F00-F99 Define	F01-F99 Mental, Behavioral and Neurodevelopmental disorders	508389	Mild oxycodone-acetaminophen abuse in sustained remission	ICD10CM	1	F11.11		Opioid abuse, in remission	IMO	GHS-PACDC	IMO_2015_R	ACTIVE	N	2017-09-17	9999-12-31	2017-10-30
Diagnosis: ICD10 Code, F00-F99 Define	F01-F99 Mental, Behavioral and Neurodevelopmental disorders	508929	Mild opioid abuse in sustained remission on maintenance therapy	ICD10CM	1	F11.11		Opioid abuse, in remission	IMO	GHS-PACDC	IMO_2015_R	ACTIVE	N	2017-09-17	9999-12-31	2017-10-30
Diagnosis: ICD10 Code, F00-F99 Define	F01-F99 Mental, Behavioral and Neurodevelopmental disorders	509860	Heroin use disorder, mild, in sustained remission, on maintenance therapy, abuse	ICD10CM	1	F11.11		Opioid abuse, in remission	IMO	GHS-PACDC	IMO_2015_R	ACTIVE	N	2017-09-17	9999-12-31	2017-10-30
Diagnosis: ICD10 Code, F00-F99 Define	F01-F99 Mental, Behavioral and Neurodevelopmental disorders	507620	Lomotil use disorder, mild, in sustained remission, in controlled environment, abuse	ICD10CM	1	F11.11		Opioid abuse, in remission	IMO	GHS-PACDC	IMO_2015_R	ACTIVE	N	2017-09-17	9999-12-31	2017-10-30
Diagnosis: ICD10 Code, F00-F99 Define	F01-F99 Mental, Behavioral and Neurodevelopmental disorders	509848	Vicodin use disorder, mild, in sustained remission, on maintenance therapy, abuse	ICD10CM	1	F11.11		Opioid abuse, in remission	IMO	GHS-PACDC	IMO_2015_R	ACTIVE	N	2017-09-17	9999-12-31	2017-10-30
Diagnosis: ICD10 Code, F00-F99 Define	F01-F99 Mental, Behavioral and Neurodevelopmental disorders	508320	Mild oxycodone-acetaminophen abuse in early remission in controlled environment	ICD10CM	1	F11.11		Opioid abuse, in remission	IMO	GHS-PACDC	IMO_2015_R	ACTIVE	N	2017-09-17	9999-12-31	2017-10-30
Diagnosis: ICD10 Code, F00-F99 Define	F01-F99 Mental, Behavioral and Neurodevelopmental disorders	507464	Opium use disorder, mild, in early remission, on maintenance therapy, abuse	ICD10CM	1	F11.11		Opioid abuse, in remission	IMO	GHS-PACDC	IMO_2015_R	ACTIVE	N	2017-09-17	9999-12-31	2017-10-30
Diagnosis: ICD10 Code, F00-F99 Define	F01-F99 Mental, Behavioral and Neurodevelopmental disorders	507542	Darvocet use disorder, mild, in sustained remission, in controlled environment, abuse	ICD10CM	1	F11.11		Opioid abuse, in remission	IMO	GHS-PACDC	IMO_2015_R	ACTIVE	N	2017-09-17	9999-12-31	2017-10-30
Diagnosis: ICD10 Code, F00-F99 Define	F01-F99 Mental, Behavioral and Neurodevelopmental disorders	509901	Mild heroin abuse in early remission on maintenance therapy	ICD10CM	1	F11.11		Opioid abuse, in remission	IMO	GHS-PACDC	IMO_2015_R	ACTIVE	N	2017-09-17	9999-12-31	2017-10-30
Diagnosis: ICD10 Code, F00-F99 Define	F01-F99 Mental, Behavioral and Neurodevelopmental disorders	509340	Oxycontin use disorder, mild, in early remission, in controlled environment, abuse	ICD10CM	1	F11.11		Opioid abuse, in remission	IMO	GHS-PACDC	IMO_2015_R	ACTIVE	N	2017-09-17	9999-12-31	2017-10-30
Diagnosis: ICD10 Code, F00-F99 Define	F01-F99 Mental, Behavioral and Neurodevelopmental disorders	508192	Mild fentanyl abuse in sustained remission in controlled environment	ICD10CM	1	F11.11		Opioid abuse, in remission	IMO	GHS-PACDC	IMO_2015_R	ACTIVE	N	2017-09-17	9999-12-31	2017-10-30
Diagnosis: ICD10 Code, F00-F99 Define	F01-F99 Mental, Behavioral and Neurodevelopmental disorders	508207	Mild codeine abuse in early remission on maintenance therapy	ICD10CM	1	F11.11		Opioid abuse, in remission	IMO	GHS-PACDC	IMO_2015_R	ACTIVE	N	2017-09-17	9999-12-31	2017-10-30
Diagnosis: ICD10 Code, F00-F99 Define	F01-F99 Mental, Behavioral and Neurodevelopmental disorders	597033	Opioid abuse with intoxication, uncomplicated	ICD10CM	1	F11.120		Opioid abuse with intoxication, uncomplicated	IMO	GHS-PACDC	IMO_2015_R	ACTIVE	Y	1900-01-01	9999-12-31	2015-07-10
Diagnosis: ICD10 Code, F00-F99 Define	F01-F99 Mental, Behavioral and Neurodevelopmental disorders	965391	Onset of opioid-induced mood disorder during intoxication, uncomplicated (HCC)	ICD10CM	1	F11.120	F11.120,F11.920	Opioid abuse with intoxication, uncomplicated	IMO	GHS-PACDC	IMO_2015_R	ACTIVE	N	2016-04-10	2017-10-14	2015-07-10
Diagnosis: ICD10 Code, F00-F99 Define	F01-F99 Mental, Behavioral and Neurodevelopmental disorders	1023093	Opioid intoxication with complication, uncomplicated	ICD10CM	1	F11.120		Opioid abuse with intoxication, uncomplicated	IMO	GHS-PACDC	IMO_2015_R	DELETED	Y	1900-01-01	9999-12-31	2015-07-10
Diagnosis: ICD10 Code, F00-F99 Define	F01-F99 Mental, Behavioral and Neurodevelopmental disorders	429161	Onset of opioid-induced sexual dysfunction during intoxication (HCC)	ICD10CM	1	F11.120	F11.120,F11.181	Opioid abuse with intoxication, uncomplicated	IMO	GHS-PACDC	IMO_2015_R	ACTIVE	N	1900-01-01	9999-12-31	2015-07-10
Diagnosis: ICD10 Code, F00-F99 Define	F01-F99 Mental, Behavioral and Neurodevelopmental disorders	1001833	Opioid intoxication with complication, with delirium (HCC)	ICD10CM	1	F11.121		Opioid abuse with intoxication delirium	IMO	GHS-PACDC	IMO_2015_R	ACTIVE	N	1900-01-01	2016-10-14	2015-07-10
Diagnosis: ICD10 Code, F00-F99 Define	F01-F99 Mental, Behavioral and Neurodevelopmental disorders	486312	Acute, mixed level of activity, opioid intoxication delirium with mild use disorder (HCC)	ICD10CM	1	F11.121		Opioid abuse with intoxication delirium	IMO	GHS-PACDC	IMO_2015_R	ACTIVE	N	2016-09-17	9999-12-31	2016-09-30
Diagnosis: ICD10 Code, F00-F99 Define	F01-F99 Mental, Behavioral and Neurodevelopmental disorders	965949	Onset of opioid-induced mood disorder during intoxication, with delirium (HCC)	ICD10CM	1	F11.121	F11.121,F11.14	Opioid abuse with intoxication delirium	IMO	GHS-PACDC	IMO_2015_R	ACTIVE	N	1900-01-01	2016-05-07	2015-07-10
Diagnosis: ICD10 Code, F00-F99 Define	F01-F99 Mental, Behavioral and Neurodevelopmental disorders	968245	Onset of opioid-induced mood disorder during intoxication, with perceptual disturbance (HCC)	ICD10CM	1	F11.122	F11.122,F11.922	Opioid abuse with intoxication with perceptual disturba	IMO	GHS-PACDC	IMO_2015_R	ACTIVE	N	2016-04-10	2017-10-14	2015-07-10
Diagnosis: ICD10 Code, F00-F99 Define	F01-F99 Mental, Behavioral and Neurodevelopmental disorders	1004302	Opioid intoxication with complication, with unspecified complication (HCC)	ICD10CM	1	F11.129		Opioid abuse with intoxication, unspecified	IMO	GHS-PACDC	IMO_2015_R	ACTIVE	N	1900-01-01	2016-10-14	2015-07-10
Diagnosis: ICD10 Code, F00-F99 Define	F01-F99 Mental, Behavioral and Neurodevelopmental disorders	484315	Moderate diphenoxylate-atropine use disorder on maintenance therapy (HCC)	ICD10CM	1	F11.20		Opioid dependence, uncomplicated	IMO	GHS-PACDC	IMO_2015_R	ACTIVE	N	2016-09-17	9999-12-31	2016-09-30
Diagnosis: ICD10 Code, F00-F99 Define	F01-F99 Mental, Behavioral and Neurodevelopmental disorders	507286	Severe diphenoxylate-atropine dependence in early remission on maintenance therapy (HCC)	ICD10CM	1	F11.20		Opioid dependence, uncomplicated	IMO	GHS-PACDC	IMO_2015_R	ACTIVE	N	1900-01-01	2016-10-14	2015-07-10
Diagnosis: ICD10 Code, F00-F99 Define	F01-F99 Mental, Behavioral and Neurodevelopmental disorders	509767	Severe meperidine dependence (HCC)	ICD10CM	1	F11.20		Opioid dependence, uncomplicated	IMO	GHS-PACDC	IMO_2015_R	ACTIVE	N	1900-01-01	9999-12-31	2015-07-10
Diagnosis: ICD10 Code, F00-F99 Define	F01-F99 Mental, Behavioral and Neurodevelopmental disorders	507336	Talwin use disorder, moderate, on maintenance therapy, dependence (HCC)	ICD10CM	1	F11.20		Opioid dependence, uncomplicated	IMO	GHS-PACDC	IMO_2015_R	ACTIVE	N	1900-01-01	9999-12-31	2015-07-10
Diagnosis: ICD10 Code, F00-F99 Define	F01-F99 Mental, Behavioral and Neurodevelopmental disorders	508688	Percocet use disorder, severe, dependence (HCC)	ICD10CM	1	F11.20		Opioid dependence, uncomplicated	IMO	GHS-PACDC	IMO_2015_R	ACTIVE	N	1900-01-01	9999-12-31	2015-07-10
Diagnosis: ICD10 Code, F00-F99 Define	F01-F99 Mental, Behavioral and Neurodevelopmental disorders	508963	Percocet use disorder, severe, on maintenance therapy, dependence (HCC)	ICD10CM	1	F11.20		Opioid dependence, uncomplicated	IMO	GHS-PACDC	IMO_2015_R	ACTIVE	N	1900-01-01	9999-12-31	2015-07-10
Diagnosis: ICD10 Code, F00-F99 Define	F01-F99 Mental, Behavioral and Neurodevelopmental disorders	484515	Severe acetaminophen-propoxyphene disorder in controlled environment (HCC)	ICD10CM	1	F11.20		Opioid dependence, uncomplicated	IMO	GHS-PACDC	IMO_2015_R	ACTIVE	N	1900-01-01	9999-12-31	2015-07-10
Diagnosis: ICD10 Code, F00-F99 Define	F01-F99 Mental, Behavioral and Neurodevelopmental disorders	508730	Moderate fentanyl dependence in controlled environment (HCC)	ICD10CM	1	F11.20		Opioid dependence, uncomplicated	IMO	GHS-PACDC	IMO_2015_R	ACTIVE	N	1900-01-01	9999-12-31	2015-07-10
Diagnosis: ICD10 Code, F00-F99 Define	F01-F99 Mental, Behavioral and Neurodevelopmental disorders	507319	Dextromethorphan use disorder, severe, dependence (HCC)	ICD10CM	1	F11.20		Opioid dependence, uncomplicated	IMO	GHS-PACDC	IMO_2015_R	ACTIVE	N	1900-01-01	2016-10-14	2015-07-10
Diagnosis: ICD10 Code, F00-F99 Define	F01-F99 Mental, Behavioral and Neurodevelopmental disorders	492442	Demerol use disorder, severe, on maintenance therapy (HCC)	ICD10CM	1	F11.20		Opioid dependence, uncomplicated	IMO	GHS-PACDC	IMO_2015_R	ACTIVE	N	2016-09-17	9999-12-31	2016-09-30
Diagnosis: ICD10 Code, F00-F99 Define	F01-F99 Mental, Behavioral and Neurodevelopmental disorders	479948	Moderate oxycodone-aspirin use disorder (HCC)	ICD10CM	1	F11.20		Opioid dependence, uncomplicated	IMO	GHS-PACDC	IMO_2015_R	ACTIVE	N	2016-09-17	9999-12-31	2016-09-30
Diagnosis: ICD10 Code, F00-F99 Define	F01-F99 Mental, Behavioral and Neurodevelopmental disorders	508086	Moderate pentazocine dependence on maintenance therapy (HCC)	ICD10CM	1	F11.20		Opioid dependence, uncomplicated	IMO	GHS-PACDC	IMO_2015_R	ACTIVE	N	1900-01-01	9999-12-31	2015-07-10
Diagnosis: ICD10 Code, F00-F99 Define	F01-F99 Mental, Behavioral and Neurodevelopmental disorders	488792	Moderate opioid use disorder in controlled environment (HCC)	ICD10CM	1	F11.20		Opioid dependence, uncomplicated	IMO	GHS-PACDC	IMO_2015_R	ACTIVE	N	2016-09-17	9999-12-31	2016-09-30
Diagnosis: ICD10 Code, F00-F99 Define	F01-F99 Mental, Behavioral and Neurodevelopmental disorders	507845	Moderate morphine dependence on maintenance therapy (HCC)	ICD10CM	1	F11.20		Opioid dependence, uncomplicated	IMO	GHS-PACDC	IMO_2015_R	ACTIVE	N	1900-01-01	9999-12-31	2015-07-10
Diagnosis: ICD10 Code, F00-F99 Define	F01-F99 Mental, Behavioral and Neurodevelopmental disorders	484546	Ultram use disorder, severe, in controlled environment (HCC)	ICD10CM	1	F11.20		Opioid dependence, uncomplicated	IMO	GHS-PACDC	IMO_2015_R	ACTIVE	N	2016-09-17	9999-12-31	2016-09-30
Diagnosis: ICD10 Code, F00-F99 Define	F01-F99 Mental, Behavioral and Neurodevelopmental disorders	507184	Nubain use disorder, severe, in early remission, on maintenance therapy, dependence (HCC)	ICD10CM	1	F11.20		Opioid dependence, uncomplicated	IMO	GHS-PACDC	IMO_2015_R	ACTIVE	N	1900-01-01	2016-10-14	2015-07-10
Diagnosis: ICD10 Code, F00-F99 Define	F01-F99 Mental, Behavioral and Neurodevelopmental disorders	492122	Percocet use disorder, severe, in controlled environment (HCC)	ICD10CM	1	F11.20		Opioid dependence, uncomplicated	IMO	GHS-PACDC	IMO_2015_R	ACTIVE	N	2016-09-17	9999-12-31	2016-09-30
Diagnosis: ICD10 Code, F00-F99 Define	F01-F99 Mental, Behavioral and Neurodevelopmental disorders	509752	Severe heroin dependence in early remission in controlled environment (HCC)	ICD10CM	1	F11.20		Opioid dependence, uncomplicated	IMO	GHS-PACDC	IMO_2015_R	ACTIVE	N	1900-01-01	2016-10-14	2015-07-10
Diagnosis: ICD10 Code, F00-F99 Define	F01-F99 Mental, Behavioral and Neurodevelopmental disorders	509353	Moderate dextromethorphan dependence in early remission in controlled environment (HCC)	ICD10CM	1	F11.20		Opioid dependence, uncomplicated	IMO	GHS-PACDC	IMO_2015_R	ACTIVE	N	1900-01-01	2016-10-14	2015-07-10
Diagnosis: ICD10 Code, F00-F99 Define	F01-F99 Mental, Behavioral and Neurodevelopmental disorders	489473	Opioid use disorder, severe, in controlled environment (HCC)	ICD10CM	1	F11.20		Opioid dependence, uncomplicated	IMO	GHS-PACDC	IMO_2015_R	ACTIVE	N	2016-09-17	9999-12-31	2016-09-30
Diagnosis: ICD10 Code, F00-F99 Define	F01-F99 Mental, Behavioral and Neurodevelopmental disorders	884983	Suboxone maintenance treatment complicating pregnancy, antepartum, second trimester (HCC	ICD10CM	2	F11.20	O99.322,F11.20	Opioid dependence, uncomplicated	IMO	GHS-PACDC	IMO_2015_R	ACTIVE	N	2016-04-10	9999-12-31	2015-07-10
Diagnosis: ICD10 Code, F00-F99 Define	F01-F99 Mental, Behavioral and Neurodevelopmental disorders	484816	Severe morphine use disorder on maintenance therapy (HCC)	ICD10CM	1	F11.20		Opioid dependence, uncomplicated	IMO	GHS-PACDC	IMO_2015_R	ACTIVE	N	2016-09-17	9999-12-31	2016-09-30
Diagnosis: ICD10 Code, F00-F99 Define	F01-F99 Mental, Behavioral and Neurodevelopmental disorders	479881	Methadone use disorder, moderate (HCC)	ICD10CM	1	F11.20		Opioid dependence, uncomplicated	IMO	GHS-PACDC	IMO_2015_R	ACTIVE	N	2016-09-17	9999-12-31	2016-09-30
Diagnosis: ICD10 Code, F00-F99 Define	F01-F99 Mental, Behavioral and Neurodevelopmental disorders	509815	Vicodin use disorder, severe, dependence (HCC)	ICD10CM	1	F11.20		Opioid dependence, uncomplicated	IMO	GHS-PACDC	IMO_2015_R	ACTIVE	N	1900-01-01	9999-12-31	2015-07-10
Diagnosis: ICD10 Code, F00-F99 Define	F01-F99 Mental, Behavioral and Neurodevelopmental disorders	519120	Buprenorphine maintenance treatment affecting pregnancy in third trimester, antepartum	ICD10CM	2	F11.20	O99.323,F11.20,Z79.891	Opioid dependence, uncomplicated	IMO	GHS-PACDC	IMO_2015_R	DELETED	Y	2016-04-10	9999-12-31	2015-07-10
Diagnosis: ICD10 Code, F00-F99 Define	F01-F99 Mental, Behavioral and Neurodevelopmental disorders	508074	Morphine use disorder, severe, in controlled environment, dependence (HCC)	ICD10CM	1	F11.20		Opioid dependence, uncomplicated	IMO	GHS-PACDC	IMO_2015_R	ACTIVE	N	1900-01-01	9999-12-31	2015-07-10
Diagnosis: ICD10 Code, F00-F99 Define	F01-F99 Mental, Behavioral and Neurodevelopmental disorders	485017	Moderate Percodan use disorder in controlled environment (HCC)	ICD10CM	1	F11.20		Opioid dependence, uncomplicated	IMO	GHS-PACDC	IMO_2015_R	ACTIVE	N	2016-09-17	9999-12-31	2016-09-30
Diagnosis: ICD10 Code, F00-F99 Define	F01-F99 Mental, Behavioral and Neurodevelopmental disorders	492449	Fentanyl use disorder, moderate, on maintenance therapy (HCC)	ICD10CM	1	F11.20		Opioid dependence, uncomplicated	IMO	GHS-PACDC	IMO_2015_R	ACTIVE	N	2016-09-17	9999-12-31	2016-09-30
Diagnosis: ICD10 Code, F00-F99 Define	F01-F99 Mental, Behavioral and Neurodevelopmental disorders	484323	Severe heroin use disorder in controlled environment (HCC)	ICD10CM	1	F11.20		Opioid dependence, uncomplicated	IMO	GHS-PACDC	IMO_2015_R	ACTIVE	N	2016-09-17	9999-12-31	2016-09-30
Diagnosis: ICD10 Code, F00-F99 Define	F01-F99 Mental, Behavioral and Neurodevelopmental disorders	509717	Ultram use disorder, severe, in controlled environment, dependence (HCC)	ICD10CM	1	F11.20		Opioid dependence, uncomplicated	IMO	GHS-PACDC	IMO_2015_R	ACTIVE	N	1900-01-01	9999-12-31	2015-07-10
Diagnosis: ICD10 Code, F00-F99 Define	F01-F99 Mental, Behavioral and Neurodevelopmental disorders	747123	Suboxone maintenance treatment complicating pregnancy, antepartum, first trimester (HCC)	ICD10CM	2	F11.20	O99.321,F11.20	Opioid dependence, uncomplicated	IMO	GHS-PACDC	IMO_2015_R	ACTIVE	N	2016-04-10	9999-12-31	2015-07-10
Diagnosis: ICD10 Code, F00-F99 Define	F01-F99 Mental, Behavioral and Neurodevelopmental disorders	491909	Talwin use disorder, severe, in controlled environment (HCC)	ICD10CM	1	F11.20		Opioid dependence, uncomplicated	IMO	GHS-PACDC	IMO_2015_R	ACTIVE	N	2016-09-17	9999-12-31	2016-09-30
Diagnosis: ICD10 Code, F00-F99 Define	F01-F99 Mental, Behavioral and Neurodevelopmental disorders	484135	Severe acetaminophen-propoxyphene disorder (HCC)	ICD10CM	1	F11.20		Opioid dependence, uncomplicated	IMO	GHS-PACDC	IMO_2015_R	ACTIVE	N	1900-01-01	9999-12-31	2015-07-10
Diagnosis: ICD10 Code, F00-F99 Define	F01-F99 Mental, Behavioral and Neurodevelopmental disorders	479908	Severe tramadol use disorder (HCC)	ICD10CM	1	F11.20		Opioid dependence, uncomplicated	IMO	GHS-PACDC	IMO_2015_R	ACTIVE	N	2016-09-17	9999-12-31	2016-09-30
Diagnosis: ICD10 Code, F00-F99 Define	F01-F99 Mental, Behavioral and Neurodevelopmental disorders	492472	Hydrocodone use disorder, severe, on maintenance therapy (HCC)	ICD10CM	1	F11.20		Opioid dependence, uncomplicated	IMO	GHS-PACDC	IMO_2015_R	ACTIVE	N	2016-09-17	9999-12-31	2016-09-30
Diagnosis: ICD10 Code, F00-F99 Define	F01-F99 Mental, Behavioral and Neurodevelopmental disorders	492341	Darvocet use disorder, severe, in controlled environment (HCC)	ICD10CM	1	F11.20		Opioid dependence, uncomplicated	IMO	GHS-PACDC	IMO_2015_R	ACTIVE	N	1900-01-01	9999-12-31	2015-07-10
Diagnosis: ICD10 Code, F00-F99 Define	F01-F99 Mental, Behavioral and Neurodevelopmental disorders	484316	Severe morphine use disorder in controlled environment (HCC)	ICD10CM	1	F11.20		Opioid dependence, uncomplicated	IMO	GHS-PACDC	IMO_2015_R	ACTIVE	N	2016-09-17	9999-12-31	2016-09-30
Diagnosis: ICD10 Code, F00-F99 Define	F01-F99 Mental, Behavioral and Neurodevelopmental disorders	507726	Opium use disorder, moderate, in controlled environment, dependence (HCC)	ICD10CM	1	F11.20		Opioid dependence, uncomplicated	IMO	GHS-PACDC	IMO_2015_R	ACTIVE	N	1900-01-01	9999-12-31	2015-07-10
Diagnosis: ICD10 Code, F00-F99 Define	F01-F99 Mental, Behavioral and Neurodevelopmental disorders	507868	Heroin use disorder, moderate, in controlled environment, dependence (HCC)	ICD10CM	1	F11.20		Opioid dependence, uncomplicated	IMO	GHS-PACDC	IMO_2015_R	ACTIVE	N	1900-01-01	9999-12-31	2015-07-10
Diagnosis: ICD10 Code, F00-F99 Define	F01-F99 Mental, Behavioral and Neurodevelopmental disorders	508331	Methadone use disorder, severe, in early remission, on maintenance therapy, dependence (HC	ICD10CM	1	F11.20		Opioid dependence, uncomplicated	IMO	GHS-PACDC	IMO_2015_R	ACTIVE	N	1900-01-01	2016-10-14	2015-07-10
Diagnosis: ICD10 Code, F00-F99 Define	F01-F99 Mental, Behavioral and Neurodevelopmental disorders	508876	Methadone use disorder, severe, on maintenance therapy, dependence (HCC)	ICD10CM	1	F11.20		Opioid dependence, uncomplicated	IMO	GHS-PACDC	IMO_2015_R	ACTIVE	N	1900-01-01	9999-12-31	2015-07-10
Diagnosis: ICD10 Code, F00-F99 Define	F01-F99 Mental, Behavioral and Neurodevelopmental disorders	797911	Methadone maintenance treatment complicating pregnancy, antepartum, second trimester (HC	ICD10CM	2	F11.20	O99.322,F11.20,F11.21	Opioid dependence, uncomplicated	IMO	GHS-PACDC	IMO_2015_R	ACTIVE	N	1900-01-01	2016-05-07	2015-07-10
Diagnosis: ICD10 Code, F00-F99 Define	F01-F99 Mental, Behavioral and Neurodevelopmental disorders	783202	Pregnancy complicated by Suboxone maintenance, antepartum, first trimester (HCC)	ICD10CM	2	F11.20	O99.321,F11.20	Opioid dependence, uncomplicated	IMO	GHS-PACDC	IMO_2015_R	ACTIVE	N	2016-04-10	9999-12-31	2015-07-10
Diagnosis: ICD10 Code, F00-F99 Define	F01-F99 Mental, Behavioral and Neurodevelopmental disorders	479929	Vicodin use disorder, moderate (HCC)	ICD10CM	1	F11.20		Opioid dependence, uncomplicated	IMO	GHS-PACDC	IMO_2015_R	ACTIVE	N	2016-09-17	9999-12-31	2016-09-30
Diagnosis: ICD10 Code, F00-F99 Define	F01-F99 Mental, Behavioral and Neurodevelopmental disorders	484728	Moderate heroin use disorder on maintenance therapy (HCC)	ICD10CM	1	F11.20		Opioid dependence, uncomplicated	IMO	GHS-PACDC	IMO_2015_R	ACTIVE	N	2016-09-17	9999-12-31	2016-09-30
Diagnosis: ICD10 Code, F00-F99 Define	F01-F99 Mental, Behavioral and Neurodevelopmental disorders	484566	Severe acetaminophen-hydrocodone use disorder in controlled environment (HCC)	ICD10CM	1	F11.20		Opioid dependence, uncomplicated	IMO	GHS-PACDC	IMO_2015_R	ACTIVE	N	2016-09-17	9999-12-31	2016-09-30
Diagnosis: ICD10 Code, F00-F99 Define	F01-F99 Mental, Behavioral and Neurodevelopmental disorders	156593	Narcotic dependence, episodic use (HCC)	ICD10CM	1	F11.20		Opioid dependence, uncomplicated	IMO	GHS-PACDC	DX_LEGACY	ACTIVE	N	2016-04-10	9999-12-31	2015-07-10
Diagnosis: ICD10 Code, F00-F99 Define	F01-F99 Mental, Behavioral and Neurodevelopmental disorders	517920	Buprenorphine maintenance treatment affecting pregnancy, antepartum (HCC)	ICD10CM	2	F11.20	O99.320,F11.20,Z79.891	Opioid dependence, uncomplicated	IMO	GHS-PACDC	IMO_2015_R	ACTIVE	N	1900-01-01	9999-12-31	2015-07-10
Diagnosis: ICD10 Code, F00-F99 Define	F01-F99 Mental, Behavioral and Neurodevelopmental disorders	430968	Buprenorphine and naloxone maintenance treatment complicating pregnancy in third trimester	ICD10CM	2	F11.20	O99.323,F11.20	Opioid dependence, uncomplicated	IMO	GHS-PACDC	IMO_2015_R	ACTIVE	N	1900-01-01	9999-12-31	2015-07-10
Diagnosis: ICD10 Code, F00-F99 Define	F01-F99 Mental, Behavioral and Neurodevelopmental disorders	492445	Stadol use disorder, moderate, on maintenance therapy (HCC)	ICD10CM	1	F11.20		Opioid dependence, uncomplicated	IMO	GHS-PACDC	IMO_2015_R	ACTIVE	N	2016-09-17	9999-12-31	2016-09-30
Diagnosis: ICD10 Code, F00-F99 Define	F01-F99 Mental, Behavioral and Neurodevelopmental disorders	508449	Heroin use disorder, moderate, dependence (HCC)	ICD10CM	1	F11.20		Opioid dependence, uncomplicated	IMO	GHS-PACDC	IMO_2015_R	ACTIVE	N	1900-01-01	9999-12-31	2015-07-10
Diagnosis: ICD10 Code, F00-F99 Define	F01-F99 Mental, Behavioral and Neurodevelopmental disorders	507280	Severe meperidine dependence in early remission in controlled environment (HCC)	ICD10CM	1	F11.20		Opioid dependence, uncomplicated	IMO	GHS-PACDC	IMO_2015_R	ACTIVE	N	1900-01-01	2016-10-14	2015-07-10
Diagnosis: ICD10 Code, F00-F99 Define	F01-F99 Mental, Behavioral and Neurodevelopmental disorders	507088	Demerol use disorder, moderate, in early remission, on maintenance therapy, dependence (HC	ICD10CM	1	F11.20		Opioid dependence, uncomplicated	IMO	GHS-PACDC	IMO_2015_R	ACTIVE	N	1900-01-01	2016-10-14	2015-07-10
Diagnosis: ICD10 Code, F00-F99 Define	F01-F99 Mental, Behavioral and Neurodevelopmental disorders	30208	Narcotism (HCC)	ICD10CM	1	F11.20		Opioid dependence, uncomplicated	IMO	GHS-PACDC	DX_LEGACY	ACTIVE	N	2016-04-10	9999-12-31	2015-07-10
Diagnosis: ICD10 Code, F00-F99 Define	F01-F99 Mental, Behavioral and Neurodevelopmental disorders	491902	Methadone use disorder, moderate, in controlled environment (HCC)	ICD10CM	1	F11.20		Opioid dependence, uncomplicated	IMO	GHS-PACDC	IMO_2015_R	ACTIVE	N	2016-09-17	9999-12-31	2016-09-30
Diagnosis: ICD10 Code, F00-F99 Define	F01-F99 Mental, Behavioral and Neurodevelopmental disorders	507799	Moderate acetaminophen-propoxyphene dependence in early remission on maintenance thera	ICD10CM	1	F11.20		Opioid dependence, uncomplicated	IMO	GHS-PACDC	IMO_2015_R	ACTIVE	N	1900-01-01	2016-10-14	2015-07-10
Diagnosis: ICD10 Code, F00-F99 Define	F01-F99 Mental, Behavioral and Neurodevelopmental disorders	509953	Codeine use disorder, severe, in controlled environment, dependence (HCC)	ICD10CM	1	F11.20		Opioid dependence, uncomplicated	IMO	GHS-PACDC	IMO_2015_R	ACTIVE	N	1900-01-01	9999-12-31	2015-07-10

Diagnosis: ICD10 Code, F00-F99 Define	F01-F99 Mental, Behavioral and Neurodevelopmental disorders	509546	Oxycodone use disorder, severe, in controlled environment, dependence (HCC)	ICD10CM	1	F11.20		Opioid dependence, uncomplicated	IMO	GHS-PACDC	IMO_2015_R	ACTIVE	N	1900-01-01	9999-12-31	2015-07-10
Diagnosis: ICD10 Code, F00-F99 Define	F01-F99 Mental, Behavioral and Neurodevelopmental disorders	508367	Morphine use disorder, moderate, in early remission, on maintenance therapy, dependence (H	ICD10CM	1	F11.20		Opioid dependence, uncomplicated	IMO	GHS-PACDC	IMO_2015_R	ACTIVE	N	1900-01-01	2016-10-14	2015-07-10
Diagnosis: ICD10 Code, F00-F99 Define	F01-F99 Mental, Behavioral and Neurodevelopmental disorders	428930	Moderate opioid use disorder (HCC)	ICD10CM	1	F11.20		Opioid dependence, uncomplicated	IMO	GHS-PACDC	IMO_2015_R	ACTIVE	N	2016-09-17	9999-12-31	2016-09-30
Diagnosis: ICD10 Code, F00-F99 Define	F01-F99 Mental, Behavioral and Neurodevelopmental disorders	508301	Moderate hydromorphone dependence on maintenance therapy (HCC)	ICD10CM	1	F11.20		Opioid dependence, uncomplicated	IMO	GHS-PACDC	IMO_2015_R	ACTIVE	N	1900-01-01	9999-12-31	2015-07-10
Diagnosis: ICD10 Code, F00-F99 Define	F01-F99 Mental, Behavioral and Neurodevelopmental disorders	484000	Severe meperidine use disorder (HCC)	ICD10CM	1	F11.20		Opioid dependence, uncomplicated	IMO	GHS-PACDC	IMO_2015_R	ACTIVE	N	2016-09-17	9999-12-31	2016-09-30
Diagnosis: ICD10 Code, F00-F99 Define	F01-F99 Mental, Behavioral and Neurodevelopmental disorders	507773	Severe oxycodone-aspirin dependence in early remission in controlled environment (HCC)	ICD10CM	1	F11.21		Opioid dependence, in remission	IMO	GHS-PACDC	IMO_2015_R	ACTIVE	N	2016-09-17	9999-12-31	2016-09-30
Diagnosis: ICD10 Code, F00-F99 Define	F01-F99 Mental, Behavioral and Neurodevelopmental disorders	492241	Oxycontin use disorder, severe, in sustained remission (HCC)	ICD10CM	1	F11.21		Opioid dependence, in remission	IMO	GHS-PACDC	IMO_2015_R	ACTIVE	N	2016-09-17	9999-12-31	2016-09-30
Diagnosis: ICD10 Code, F00-F99 Define	F01-F99 Mental, Behavioral and Neurodevelopmental disorders	507197	Heroin use disorder, moderate, in sustained remission, on maintenance therapy, dependence (H	ICD10CM	1	F11.21		Opioid dependence, in remission	IMO	GHS-PACDC	IMO_2015_R	ACTIVE	N	1900-01-01	9999-12-31	2015-07-10
Diagnosis: ICD10 Code, F00-F99 Define	F01-F99 Mental, Behavioral and Neurodevelopmental disorders	484409	Moderate pentazocine use disorder in sustained remission (HCC)	ICD10CM	1	F11.21		Opioid dependence, in remission	IMO	GHS-PACDC	IMO_2015_R	ACTIVE	N	2016-09-17	9999-12-31	2016-09-30
Diagnosis: ICD10 Code, F00-F99 Define	F01-F99 Mental, Behavioral and Neurodevelopmental disorders	3159	Combinations of opioid type drug with any other drug dependence, in remission	ICD10CM	1	F11.21		Opioid dependence, in remission	IMO	GHS-PACDC	DX_LEGACY	ACTIVE	Y	1900-01-01	9999-12-31	2015-07-10
Diagnosis: ICD10 Code, F00-F99 Define	F01-F99 Mental, Behavioral and Neurodevelopmental disorders	507460	Dilaudid use disorder, severe, in early remission, on maintenance therapy, dependence (HCC)	ICD10CM	1	F11.21		Opioid dependence, in remission	IMO	GHS-PACDC	IMO_2015_R	ACTIVE	N	2016-09-17	9999-12-31	2016-09-30
Diagnosis: ICD10 Code, F00-F99 Define	F01-F99 Mental, Behavioral and Neurodevelopmental disorders	508624	Percodan use disorder, moderate, in sustained remission, dependence (HCC)	ICD10CM	1	F11.21		Opioid dependence, in remission	IMO	GHS-PACDC	IMO_2015_R	ACTIVE	N	1900-01-01	9999-12-31	2015-07-10
Diagnosis: ICD10 Code, F00-F99 Define	F01-F99 Mental, Behavioral and Neurodevelopmental disorders	507823	Dextromethorphan use disorder, severe, in sustained remission, dependence (HCC)	ICD10CM	1	F11.21		Opioid dependence, in remission	IMO	GHS-PACDC	IMO_2015_R	ACTIVE	N	1900-01-01	2016-10-14	2015-07-10
Diagnosis: ICD10 Code, F00-F99 Define	F01-F99 Mental, Behavioral and Neurodevelopmental disorders	509286	Ultram use disorder, moderate, in early remission, dependence (HCC)	ICD10CM	1	F11.21		Opioid dependence, in remission	IMO	GHS-PACDC	IMO_2015_R	ACTIVE	N	1900-01-01	9999-12-31	2015-07-10
Diagnosis: ICD10 Code, F00-F99 Define	F01-F99 Mental, Behavioral and Neurodevelopmental disorders	509082	Hydrocodone use disorder, moderate, in sustained remission, dependence (HCC)	ICD10CM	1	F11.21		Opioid dependence, in remission	IMO	GHS-PACDC	IMO_2015_R	ACTIVE	N	1900-01-01	9999-12-31	2015-07-10
Diagnosis: ICD10 Code, F00-F99 Define	F01-F99 Mental, Behavioral and Neurodevelopmental disorders	508609	Moderate acetaminophen-hydrocodone dependence in early remission (HCC)	ICD10CM	1	F11.21		Opioid dependence, in remission	IMO	GHS-PACDC	IMO_2015_R	ACTIVE	N	1900-01-01	9999-12-31	2015-07-10
Diagnosis: ICD10 Code, F00-F99 Define	F01-F99 Mental, Behavioral and Neurodevelopmental disorders	509903	Moderate tramadol dependence in early remission (HCC)	ICD10CM	1	F11.21		Opioid dependence, in remission	IMO	GHS-PACDC	IMO_2015_R	ACTIVE	N	1900-01-01	9999-12-31	2015-07-10
Diagnosis: ICD10 Code, F00-F99 Define	F01-F99 Mental, Behavioral and Neurodevelopmental disorders	509778	Moderate opioid dependence in early remission in controlled environment (HCC)	ICD10CM	1	F11.21		Opioid dependence, in remission	IMO	GHS-PACDC	IMO_2015_R	ACTIVE	N	1900-01-01	9999-12-31	2015-07-10
Diagnosis: ICD10 Code, F00-F99 Define	F01-F99 Mental, Behavioral and Neurodevelopmental disorders	507926	Vicodin use disorder, moderate, in sustained remission, dependence (HCC)	ICD10CM	1	F11.21		Opioid dependence, in remission	IMO	GHS-PACDC	IMO_2015_R	ACTIVE	N	1900-01-01	9999-12-31	2015-07-10
Diagnosis: ICD10 Code, F00-F99 Define	F01-F99 Mental, Behavioral and Neurodevelopmental disorders	506993	Moderate hydrocodone dependence in sustained remission (HCC)	ICD10CM	1	F11.21		Opioid dependence, in remission	IMO	GHS-PACDC	IMO_2015_R	ACTIVE	N	1900-01-01	9999-12-31	2015-07-10
Diagnosis: ICD10 Code, F00-F99 Define	F01-F99 Mental, Behavioral and Neurodevelopmental disorders	507505	Severe codeine dependence in early remission (HCC)	ICD10CM	1	F11.21		Opioid dependence, in remission	IMO	GHS-PACDC	IMO_2015_R	ACTIVE	N	1900-01-01	9999-12-31	2015-07-10
Diagnosis: ICD10 Code, F00-F99 Define	F01-F99 Mental, Behavioral and Neurodevelopmental disorders	509369	Moderate morphine dependence in early remission in controlled environment (HCC)	ICD10CM	1	F11.21		Opioid dependence, in remission	IMO	GHS-PACDC	IMO_2015_R	ACTIVE	N	2016-09-17	9999-12-31	2016-09-30
Diagnosis: ICD10 Code, F00-F99 Define	F01-F99 Mental, Behavioral and Neurodevelopmental disorders	509392	Nubain use disorder, moderate, in sustained remission, in controlled environment, dependence	ICD10CM	1	F11.21		Opioid dependence, in remission	IMO	GHS-PACDC	IMO_2015_R	ACTIVE	N	1900-01-01	9999-12-31	2015-07-10
Diagnosis: ICD10 Code, F00-F99 Define	F01-F99 Mental, Behavioral and Neurodevelopmental disorders	492100	Nubain use disorder, moderate, in sustained remission (HCC)	ICD10CM	1	F11.21		Opioid dependence, in remission	IMO	GHS-PACDC	IMO_2015_R	ACTIVE	N	2016-09-17	9999-12-31	2016-09-30
Diagnosis: ICD10 Code, F00-F99 Define	F01-F99 Mental, Behavioral and Neurodevelopmental disorders	507770	Fentanyl use disorder, moderate, in early remission, on maintenance therapy, dependence (HC	ICD10CM	1	F11.21		Opioid dependence, in remission	IMO	GHS-PACDC	IMO_2015_R	ACTIVE	N	2016-09-17	9999-12-31	2016-09-30
Diagnosis: ICD10 Code, F00-F99 Define	F01-F99 Mental, Behavioral and Neurodevelopmental disorders	507822	Opium use disorder, severe, in sustained remission, on maintenance therapy, dependence (HCC	ICD10CM	1	F11.21		Opioid dependence, in remission	IMO	GHS-PACDC	IMO_2015_R	ACTIVE	N	1900-01-01	9999-12-31	2015-07-10
Diagnosis: ICD10 Code, F00-F99 Define	F01-F99 Mental, Behavioral and Neurodevelopmental disorders	505182	Opioid use disorder, moderate, in early remission, on maintenance therapy (HCC)	ICD10CM	1	F11.21		Opioid dependence, in remission	IMO	GHS-PACDC	IMO_2015_R	ACTIVE	N	2016-09-17	9999-12-31	2016-09-30
Diagnosis: ICD10 Code, F00-F99 Define	F01-F99 Mental, Behavioral and Neurodevelopmental disorders	507388	Talwin use disorder, moderate, in early remission, on maintenance therapy, dependence (HCC)	ICD10CM	1	F11.21		Opioid dependence, in remission	IMO	GHS-PACDC	IMO_2015_R	ACTIVE	N	2016-09-17	9999-12-31	2016-09-30
Diagnosis: ICD10 Code, F00-F99 Define	F01-F99 Mental, Behavioral and Neurodevelopmental disorders	507878	Severe continuous-release oxycodone dependence in early remission on maintenance therapy (	ICD10CM	1	F11.21		Opioid dependence, in remission	IMO	GHS-PACDC	IMO_2015_R	ACTIVE	N	2016-09-17	9999-12-31	2016-09-30
Diagnosis: ICD10 Code, F00-F99 Define	F01-F99 Mental, Behavioral and Neurodevelopmental disorders	509842	Severe heroin dependence in sustained remission in controlled environment (HCC)	ICD10CM	1	F11.21		Opioid dependence, in remission	IMO	GHS-PACDC	IMO_2015_R	ACTIVE	N	1900-01-01	9999-12-31	2015-07-10
Diagnosis: ICD10 Code, F00-F99 Define	F01-F99 Mental, Behavioral and Neurodevelopmental disorders	509177	Severe acetaminophen-hydrocodone dependence in sustained remission on maintenance thera	ICD10CM	1	F11.21		Opioid dependence, in remission	IMO	GHS-PACDC	IMO_2015_R	ACTIVE	N	1900-01-01	9999-12-31	2015-07-10
Diagnosis: ICD10 Code, F00-F99 Define	F01-F99 Mental, Behavioral and Neurodevelopmental disorders	484937	Severe meperidine use disorder in early remission (HCC)	ICD10CM	1	F11.21		Opioid dependence, in remission	IMO	GHS-PACDC	IMO_2015_R	ACTIVE	N	2016-09-17	9999-12-31	2016-09-30
Diagnosis: ICD10 Code, F00-F99 Define	F01-F99 Mental, Behavioral and Neurodevelopmental disorders	673931	Methadone maintenance treatment complicating pregnancy, third trimester (HCC)	ICD10CM	3	F11.21	O99.323,F11.20,F11.21	Opioid dependence, in remission	IMO	GHS-PACDC	IMO_2015_R	ACTIVE	N	1900-01-01	2016-05-07	2015-07-10
Diagnosis: ICD10 Code, F00-F99 Define	F01-F99 Mental, Behavioral and Neurodevelopmental disorders	508324	Severe oxycodone-acetaminophen dependence in early remission (HCC)	ICD10CM	1	F11.21		Opioid dependence, in remission	IMO	GHS-PACDC	IMO_2015_R	ACTIVE	N	1900-01-01	9999-12-31	2015-07-10
Diagnosis: ICD10 Code, F00-F99 Define	F01-F99 Mental, Behavioral and Neurodevelopmental disorders	507643	Moderate propoxyphene dependence in early remission on maintenance therapy (HCC)	ICD10CM	1	F11.21		Opioid dependence, in remission	IMO	GHS-PACDC	IMO_2015_R	ACTIVE	N	2016-09-17	9999-12-31	2016-09-30
Diagnosis: ICD10 Code, F00-F99 Define	F01-F99 Mental, Behavioral and Neurodevelopmental disorders	509654	Opium use disorder, moderate, in sustained remission, dependence (HCC)	ICD10CM	1	F11.21		Opioid dependence, in remission	IMO	GHS-PACDC	IMO_2015_R	ACTIVE	N	1900-01-01	9999-12-31	2015-07-10
Diagnosis: ICD10 Code, F00-F99 Define	F01-F99 Mental, Behavioral and Neurodevelopmental disorders	492012	Percocet use disorder, severe, in early remission (HCC)	ICD10CM	1	F11.21		Opioid dependence, in remission	IMO	GHS-PACDC	IMO_2015_R	ACTIVE	N	2016-09-17	9999-12-31	2016-09-30
Diagnosis: ICD10 Code, F00-F99 Define	F01-F99 Mental, Behavioral and Neurodevelopmental disorders	506930	Opioid use disorder, moderate, in early remission, in controlled environment (HCC)	ICD10CM	1	F11.21		Opioid dependence, in remission	IMO	GHS-PACDC	IMO_2015_R	ACTIVE	N	2016-09-17	9999-12-31	2016-09-30
Diagnosis: ICD10 Code, F00-F99 Define	F01-F99 Mental, Behavioral and Neurodevelopmental disorders	509763	Severe morphine dependence in early remission (HCC)	ICD10CM	1	F11.21		Opioid dependence, in remission	IMO	GHS-PACDC	IMO_2015_R	ACTIVE	N	1900-01-01	9999-12-31	2015-07-10
Diagnosis: ICD10 Code, F00-F99 Define	F01-F99 Mental, Behavioral and Neurodevelopmental disorders	507263	Moderate opioid dependence in sustained remission (HCC)	ICD10CM	1	F11.21		Opioid dependence, in remission	IMO	GHS-PACDC	IMO_2015_R	ACTIVE	N	1900-01-01	9999-12-31	2015-07-10
Diagnosis: ICD10 Code, F00-F99 Define	F01-F99 Mental, Behavioral and Neurodevelopmental disorders	508616	Severe pentazocine dependence in sustained remission in controlled environment (HCC)	ICD10CM	1	F11.21		Opioid dependence, in remission	IMO	GHS-PACDC	IMO_2015_R	ACTIVE	N	1900-01-01	9999-12-31	2015-07-10
Diagnosis: ICD10 Code, F00-F99 Define	F01-F99 Mental, Behavioral and Neurodevelopmental disorders	506935	Opioid use disorder, moderate, in sustained remission, in controlled environment (HCC)	ICD10CM	1	F11.21		Opioid dependence, in remission	IMO	GHS-PACDC	IMO_2015_R	ACTIVE	N	2016-09-17	9999-12-31	2016-09-30
Diagnosis: ICD10 Code, F00-F99 Define	F01-F99 Mental, Behavioral and Neurodevelopmental disorders	509492	Severe opioid dependence in early remission in controlled environment (HCC)	ICD10CM	1	F11.21		Opioid dependence, in remission	IMO	GHS-PACDC	IMO_2015_R	ACTIVE	N	1900-01-01	9999-12-31	2015-07-10
Diagnosis: ICD10 Code, F00-F99 Define	F01-F99 Mental, Behavioral and Neurodevelopmental disorders	507693	Severe opium dependence in sustained remission (HCC)	ICD10CM	1	F11.21		Opioid dependence, in remission	IMO	GHS-PACDC	IMO_2015_R	ACTIVE	N	1900-01-01	9999-12-31	2015-07-10
Diagnosis: ICD10 Code, F00-F99 Define	F01-F99 Mental, Behavioral and Neurodevelopmental disorders	509111	Severe propoxyphene dependence in early remission (HCC)	ICD10CM	1	F11.21		Opioid dependence, in remission	IMO	GHS-PACDC	IMO_2015_R	ACTIVE	N	1900-01-01	9999-12-31	2015-07-10
Diagnosis: ICD10 Code, F00-F99 Define	F01-F99 Mental, Behavioral and Neurodevelopmental disorders	484219	Severe opium use disorder in early remission (HCC)	ICD10CM	1	F11.21		Opioid dependence, in remission	IMO	GHS-PACDC	IMO_2015_R	ACTIVE	N	2016-09-17	9999-12-31	2016-09-30
Diagnosis: ICD10 Code, F00-F99 Define	F01-F99 Mental, Behavioral and Neurodevelopmental disorders	509421	Talwin use disorder, moderate, in sustained remission, dependence (HCC)	ICD10CM	1	F11.21		Opioid dependence, in remission	IMO	GHS-PACDC	IMO_2015_R	ACTIVE	N	1900-01-01	9999-12-31	2015-07-10
Diagnosis: ICD10 Code, F00-F99 Define	F01-F99 Mental, Behavioral and Neurodevelopmental disorders	484956	Severe Percodan use disorder in early remission (HCC)	ICD10CM	1	F11.21		Opioid dependence, in remission	IMO	GHS-PACDC	IMO_2015_R	ACTIVE	N	2016-09-17	9999-12-31	2016-09-30
Diagnosis: ICD10 Code, F00-F99 Define	F01-F99 Mental, Behavioral and Neurodevelopmental disorders	492471	Darvocet use disorder, severe, on maintenance therapy (HCC)	ICD10CM	1	F11.21		Opioid dependence, in remission	IMO	GHS-PACDC	IMO_2015_R	ACTIVE	N	1900-01-01	9999-12-31	2015-07-10
Diagnosis: ICD10 Code, F00-F99 Define	F01-F99 Mental, Behavioral and Neurodevelopmental disorders	484759	Severe morphine use disorder in early remission (HCC)	ICD10CM	1	F11.21		Opioid dependence, in remission	IMO	GHS-PACDC	IMO_2015_R	ACTIVE	N	2016-09-17	9999-12-31	2016-09-30
Diagnosis: ICD10 Code, F00-F99 Define	F01-F99 Mental, Behavioral and Neurodevelopmental disorders	509596	Severe methadone dependence in sustained remission on maintenance therapy (HCC)	ICD10CM	1	F11.21		Opioid dependence, in remission	IMO	GHS-PACDC	IMO_2015_R	ACTIVE	N	1900-01-01	9999-12-31	2015-07-10
Diagnosis: ICD10 Code, F00-F99 Define	F01-F99 Mental, Behavioral and Neurodevelopmental disorders	507634	Moderate oxycodone dependence in sustained remission in controlled environment (HCC)	ICD10CM	1	F11.21		Opioid dependence, in remission	IMO	GHS-PACDC	IMO_2015_R	ACTIVE	N	1900-01-01	9999-12-31	2015-07-10
Diagnosis: ICD10 Code, F00-F99 Define	F01-F99 Mental, Behavioral and Neurodevelopmental disorders	509868	Demerol use disorder, severe, in sustained remission, dependence (HCC)	ICD10CM	1	F11.21		Opioid dependence, in remission	IMO	GHS-PACDC	IMO_2015_R	ACTIVE	N	1900-01-01	9999-12-31	2015-07-10
Diagnosis: ICD10 Code, F00-F99 Define	F01-F99 Mental, Behavioral and Neurodevelopmental disorders	509730	Opium use disorder, severe, in early remission, on maintenance therapy, dependence (HCC)	ICD10CM	1	F11.21		Opioid dependence, in remission	IMO	GHS-PACDC	IMO_2015_R	ACTIVE	N	2016-09-17	9999-12-31	2016-09-30
Diagnosis: ICD10 Code, F00-F99 Define	F01-F99 Mental, Behavioral and Neurodevelopmental disorders	507555	Moderate opioid dependence in sustained remission on maintenance therapy (HCC)	ICD10CM	1	F11.21		Opioid dependence, in remission	IMO	GHS-PACDC	IMO_2015_R	ACTIVE	N	1900-01-01	9999-12-31	2015-07-10
Diagnosis: ICD10 Code, F00-F99 Define	F01-F99 Mental, Behavioral and Neurodevelopmental disorders	508950	Severe hydromorphone dependence in sustained remission on maintenance therapy (HCC)	ICD10CM	1	F11.21		Opioid dependence, in remission	IMO	GHS-PACDC	IMO_2015_R	ACTIVE	N	1900-01-01	9999-12-31	2015-07-10
Diagnosis: ICD10 Code, F00-F99 Define	F01-F99 Mental, Behavioral and Neurodevelopmental disorders	485834	Moderate opioid use disorder, in early remission (HCC)	ICD10CM	1	F11.21		Opioid dependence, in remission	IMO	GHS-PACDC	IMO_2015_R	ACTIVE	N	2016-09-17	9999-12-31	2016-09-30
Diagnosis: ICD10 Code, F00-F99 Define	F01-F99 Mental, Behavioral and Neurodevelopmental disorders	507640	Talwin use disorder, moderate, in sustained remission, in controlled environment, dependence	ICD10CM	1	F11.21		Opioid dependence, in remission	IMO	GHS-PACDC	IMO_2015_R	ACTIVE	N	1900-01-01	9999-12-31	2015-07-10
Diagnosis: ICD10 Code, F00-F99 Define	F01-F99 Mental, Behavioral and Neurodevelopmental disorders	484465	Severe codeine use disorder in early remission (HCC)	ICD10CM	1	F11.21		Opioid dependence, in remission	IMO	GHS-PACDC	IMO_2015_R	ACTIVE	N	2016-09-17	9999-12-31	2016-09-30
Diagnosis: ICD10 Code, F00-F99 Define	F01-F99 Mental, Behavioral and Neurodevelopmental disorders	508961	Methadone use disorder, moderate, in early remission, dependence (HCC)	ICD10CM	1	F11.21		Opioid dependence, in remission	IMO	GHS-PACDC	IMO_2015_R	ACTIVE	N	1900-01-01	9999-12-31	2015-07-10
Diagnosis: ICD10 Code, F00-F99 Define	F01-F99 Mental, Behavioral and Neurodevelopmental disorders	509382	Moderate oxycodone-aspirin dependence in early remission on maintenance therapy (HCC)	ICD10CM	1	F11.21		Opioid dependence, in remission	IMO	GHS-PACDC	IMO_2015_R	ACTIVE	N	2016-09-17	9999-12-31	2016-09-30
Diagnosis: ICD10 Code, F00-F99 Define	F01-F99 Mental, Behavioral and Neurodevelopmental disorders	507132	Percodan use disorder, moderate, in early remission, dependence (HCC)	ICD10CM	1	F11.21		Opioid dependence, in remission	IMO	GHS-PACDC	IMO_2015_R	ACTIVE	N	1900-01-01	9999-12-31	2015-07-10
Diagnosis: ICD10 Code, F00-F99 Define	F01-F99 Mental, Behavioral and Neurodevelopmental disorders	484851	Moderate heroin use disorder in sustained remission (HCC)	ICD10CM	1	F11.21		Opioid dependence, in remission	IMO	GHS-PACDC	IMO_2015_R	ACTIVE	N	2016-09-17	9999-12-31	2016-09-30
Diagnosis: ICD10 Code, F00-F99 Define	F01-F99 Mental, Behavioral and Neurodevelopmental disorders	505186	Opioid use disorder, severe, in sustained remission, on maintenance therapy (HCC)	ICD10CM	1	F11.21		Opioid dependence, in remission	IMO	GHS-PACDC	IMO_2015_R	ACTIVE	N	2016-09-17	9999-12-31	2016-09-30
Diagnosis: ICD10 Code, F00-F99 Define	F01-F99 Mental, Behavioral and Neurodevelopmental disorders	484368	Severe oxycodone-aspirin use disorder in sustained remission (HCC)	ICD10CM	1	F11.21		Opioid dependence, in remission	IMO	GHS-PACDC	IMO_2015_R	ACTIVE	N	2016-09-17	9999-12-31	2016-09-30
Diagnosis: ICD10 Code, F00-F99 Define	F01-F99 Mental, Behavioral and Neurodevelopmental disorders	960262	Polydrug dependence including opioid type drug, continuous use, with delirium	ICD10CM	1	F11.221		Opioid dependence with intoxication delirium	IMO	GHS-PACDC	IMO_2015_R	DELETED	Y	1900-01-01	9999-12-31	2015-07-10
Diagnosis: ICD10 Code, F00-F99 Define	F01-F99 Mental, Behavioral and Neurodevelopmental disorders	429055	Opioid intoxication without perceptual disturbances with moderate or severe use disorder (HC	ICD10CM	1	F11.229		Opioid dependence with intoxication, unspecified	IMO	GHS-PACDC	IMO_2015_R	ACTIVE	N	2016-09-17	9999-12-31	2016-09-30
Diagnosis: ICD10 Code, F00-F99 Define	F01-F99 Mental, Behavioral and Neurodevelopmental disorders	489246	Onset of opioid-induced sleep disorder, insomnia type, during discontinuation or withdrawal (H	ICD10CM	1	F11.23	F11.23,F11.282	Opioid dependence with withdrawal	IMO	GHS-PACDC	IMO_2015_R	ACTIVE	N	1900-01-01	9999-12-31	2015-07-10
Diagnosis: ICD10 Code, F00-F99 Define	F01-F99 Mental, Behavioral and Neurodevelopmental disorders	487084	Opioid-induced anxiety disorder without use disorder with onset during withdrawal (HCC)	ICD10CM	3	F11.23	F11.288,F41.8,F11.23	Opioid dependence with withdrawal	IMO	GHS-PACDC	IMO_2015_R	ACTIVE	N	2016-09-17	9999-12-31	2016-09-30
Diagnosis: ICD10 Code, F00-F99 Define	F01-F99 Mental, Behavioral and Neurodevelopmental disorders	488809	Opioid-induced sleep disorder, parasomnia type, with onset during discontinuation/withdrawal	ICD10CM	1	F11.23	F11.23,F11.282	Opioid dependence with withdrawal	IMO	GHS-PACDC	IMO_2015_R	ACTIVE	N	1900-01-01	9999-12-31	2015-07-10
Diagnosis: ICD10 Code, F00-F99 Define	F01-F99 Mental, Behavioral and Neurodevelopmental disorders	269019	Opioid dependence with opioid-induced mood disorder (HCC)	ICD10CM	1	F11.24		Opioid dependence with opioid-induced mood disorder	IMO	GHS-PACDC	DX_LEGACY	ACTIVE	N	1900-01-01	9999-12-31	2015-07-10
Diagnosis: ICD10 Code, F00-F99 Define	F01-F99 Mental, Behavioral and Neurodevelopmental disorders	487148	Opioid-induced sexual dysfunction without use disorder with onset during withdrawal (HCC)	ICD10CM	1	F11.281	F11.281,F11.23	Opioid dependence with opioid-induced sexual dysfunct	IMO	GHS-PACDC	IMO_2015_R	ACTIVE	N	2016-09-17	9999-12-31	2016-09-30
Diagnosis: ICD10 Code, F00-F99 Define	F01-F99 Mental, Behavioral and Neurodevelopmental disorders	487212	Opioid-induced sleep disorder with moderate or severe use disorder, mixed type (HCC)	ICD10CM	1	F11.282		Opioid dependence with opioid-induced sleep disorder	IMO	GHS-PACDC	IMO_2015_R	ACTIVE	N	2016-09-17	9999-12-31	2016-09-30
Diagnosis: ICD10 Code, F00-F99 Define	F01-F99 Mental, Behavioral and Neurodevelopmental disorders	487082	Opioid-induced sleep disorder with moderate or severe use disorder, insomnia type (HCC)	ICD10CM	1	F11.282		Opioid dependence with opioid-induced sleep disorder	IMO	GHS-PACDC	IMO_2015_R	ACTIVE	N	2016-09-17	9999-12-31	2016-09-30
Diagnosis: ICD10 Code, F00-F99 Define	F01-F99 Mental, Behavioral and Neurodevelopmental disorders	429070	Opioid-induced sleep disorder with moderate or severe use disorder (HCC)	ICD10CM	1	F11.282		Opioid dependence with opioid-induced sleep disorder	IMO	GHS-PACDC	IMO_2015_R	ACTIVE	N	2016-09-17	9999-12-31	2016-09-30
Diagnosis: ICD10 Code, F00-F99 Define	F01-F99 Mental, Behavioral and Neurodevelopmental disorders	489249	Opioid-induced sleep disorder, mixed type, with onset during discontinuation/withdrawal (HCC	ICD10CM	2	F11.282	F11.23,F11.282	Opioid dependence with opioid-induced sleep disorder	IMO	GHS-PACDC	IMO_2015_R	ACTIVE	N	1900-01-01	9999-12-31	2015-07-10
Diagnosis: ICD10 Code, F00-F99 Define	F01-F99 Mental, Behavioral and Neurodevelopmental disorders	486732	Opioid-induced anxiety disorder with moderate or severe use disorder with onset during withd	ICD10CM	1	F11.288	F11.288,F41.8,F11.23	Opioid dependence with other opioid-induced disorder	IMO	GHS-PACDC	IMO_2015_R	ACTIVE	N	2016-09-17	9999-12-31	2016-09-30
Diagnosis: ICD10 Code, F00-F99 Define	F01-F99 Mental, Behavioral and Neurodevelopmental disorders	484362	Moderate diphenoxylate-atropine use disorder in controlled environment (HCC)	ICD10CM	1	F11.90		Opioid use, unspecified, uncomplicated	IMO	GHS-PACDC	IMO_2015_R	ACTIVE	N	1900-01-01	2016-10-14	2015-07-10
Diagnosis: ICD10 Code, F00-F99 Define	F01-F99 Mental, Behavioral and Neurodevelopmental disorders	484386	Severe hydromorphone use disorder in early remission (HCC)	ICD10CM	1	F11.90		Opioid use, unspecified, uncomplicated	IMO	GHS-PACDC	IMO_2015_R	ACTIVE	N	1900-01-01	2016-10-14	2015-07-10
Diagnosis: ICD10 Code, F00-F99 Define	F01-F99 Mental, Behavioral and Neurodevelopmental disorders	484092	Mild tramadol use disorder	ICD10CM	1	F11.90		Opioid use, unspecified, uncomplicated	IMO	GHS-PACDC	IMO_2015_R	ACTIVE	N	1900-01-01	2016-10-14	2015-07-10
Diagnosis: ICD10 Code, F00-F99 Define	F01-F99 Mental, Behavioral and Neurodevelopmental disorders	489047	Severe opioid use disorder on maintenance therapy (HCC)	ICD10CM	1	F11.90		Opioid use, unspecified, uncomplicated	IMO	GHS-PACDC	IMO_2015_R	ACTIVE	N	1900-01-01	2016-10-14	2015-07-10
Diagnosis: ICD10 Code, F00-F99 Define	F01-F99 Mental, Behavioral and Neurodevelopmental disorders	484404	Moderate tramadol use disorder in sustained remission (HCC)	ICD10CM	1	F11.90		Opioid use, unspecified, uncomplicated	IMO	GHS-PACDC	IMO_2015_R	ACTIVE	N	1900-01-01	2016-10-14	2015-07-10
Diagnosis: ICD10 Code, F00-F99 Define	F01-F99 Mental, Behavioral and Neurodevelopmental disorders	484728	Moderate heroin use disorder on maintenance therapy (HCC)	ICD10CM	1	F11.90		Opioid use, unspecified, uncomplicated	IMO	GHS-PACDC	IMO_2015_R	ACTIVE	N	1900-01-01	2016-10-14	2015-07-10
Diagnosis: ICD10 Code, F00-F99 Define	F01-F99 Mental, Behavioral and Neurodevelopmental disorders	484607	Moderate morphine use disorder in controlled environment (HCC)	ICD10CM	1	F11.90		Opioid use, unspecified, uncomplicated	IMO	GHS-PACDC	IMO_2015_R	ACTIVE	N	1900-01-01	2016-10-14	2015-07-10
Diagnosis: ICD10 Code, F00-F99 Define	F01-F99 Mental, Behavioral and Neurodevelopmental disorders	491889	Darvon use disorder, mild, in early remission	ICD10CM	1	F11.90		Opioid use, unspecified, uncomplicated	IMO	GHS-PACDC	IMO_2015_R	ACTIVE	N	1900-01-01	2016-10-14	2015-07-10
Diagnosis: ICD10 Code, F00-F99 Define	F01-F99 Mental, Behavioral and Neurodevelopmental disorders	484521	Mild continuous-release oxycodone use disorder in controlled environment	ICD10CM	1	F11.90		Opioid use, unspecified, uncomplicated	IMO	GHS-PACDC	IMO_2015_R	ACTIVE	N	1900-01-01	2016-10-14	2015-07-10
Diagnosis: ICD10 Code, F00-F99 Define	F01-F99 Mental, Behavioral and Neurodevelopmental disorders	484163	Talwin use disorder, mild	ICD10CM	1	F11.90		Opioid use, unspecified, uncomplicated	IMO	GHS-PACDC	IMO_2015_R	ACTIVE	N	1900-01-01	2016-10-14	2015-07-10
Diagnosis: ICD10 Code, F00-F99 Define	F01-F99 Mental, Behavioral and Neurodevelopmental disorders	484802	Severe dextromethorphan use disorder in controlled environment (HCC)	ICD10CM	1	F11.90		Opioid use, unspecified, uncomplicated	IMO	GHS-PACDC	IMO_2015_R	ACTIVE	N	1900-01-01	2016-10-14	2015-07-10
Diagnosis: ICD10 Code, F00-F99 Define	F01-F99 Mental, Behavioral and Neurodevelopmental disorders	484779	Ultram use disorder, moderate, in early remission (HCC)	ICD10CM	1	F11.90		Opioid use, unspecified, uncomplicated	IMO	GHS-PACDC	IMO_2015_R	ACTIVE	N	1900-01-01	2016-10-14	2015-07-10
Diagnosis: ICD10 Code, F00-F99 Define	F01-F99 Mental, Behavioral and Neurodevelopmental disorders	484522	Severe propoxyphene use disorder on maintenance therapy (HCC)	ICD10CM	1	F11.90		Opioid use, unspecified, uncomplicated	IMO	GHS-PACDC	IMO_2015_R	ACTIVE	N	1900-01-01	2016-10-14	2015-07-10
Diagnosis: ICD10 Code, F00-F99 Define	F01-F99 Mental, Behavioral and Neurodevelopmental disorders	484132	Lortab use disorder, mild	ICD10CM	1	F11.90		Opioid use, unspecified, uncomplicated	IMO	GHS-PACDC	IMO_2015_R	ACTIVE	N	1900-01-01	2016-10-14	2015-07-10
Diagnosis: ICD10 Code, F00-F99 Define	F01-F99 Mental, Behavioral and Neurodevelopmental disorders	491789	Dilaudid use disorder, severe, in controlled environment (HCC)	ICD10CM	1	F11.90		Opioid use, unspecified, uncomplicated	IMO	GHS-PACDC	IMO_2015_R	ACTIVE	N	1900-01-01	2016-10-14	2015-07-10
Diagnosis: ICD10 Code, F00-F99 Define	F01-F99 Mental, Behavioral and Neurodevelopmental disorders	484419	Mild oxycodone-acetaminophen use disorder in sustained remission	ICD10CM	1	F11.90		Opioid use, unspecified, uncomplicated	IMO	GHS-PACDC	IMO_2015_R	ACTIVE	N	1900-01-01	2016-10-14	2015-07-10
Diagnosis: ICD10 Code, F00-F99 Define	F01-F99 Mental, Behavioral and Neurodevelopmental disorders	491844	Fentanyl use disorder, moderate, in controlled environment (HCC)	ICD10CM	1	F11.90		Opioid use, unspecified, uncomplicated	IMO	GHS-PACDC	IMO_2015_R	ACTIVE	N	1900-01-01	2016-10-14	2015-07-10
Diagnosis: ICD10 Code, F00-F99 Define	F01-F99 Mental, Behavioral and Neurodevelopmental disorders	484493	Vicodin use disorder, severe, in controlled environment (HCC)	ICD10CM	1	F11.90		Opioid use, unspecified, uncomplicated	IMO	GHS-PACDC	IMO_2015_R	ACTIVE	N	1900-01-01	2016-10-14	2015-07-10

Diagnosis: ICD10 Code, F00-F99 Define	F01-F99 Mental, Behavioral and Neurodevelopmental disorders	492116	Percocet use disorder, moderate, in controlled environment (HCC)	ICD10CM	1	F11.90		Opioid use, unspecified, uncomplicated	IMO	GHS-PACDC	IMO_2015_R	ACTIVE	N	1900-01-01	2016-10-14	2015-07-10
Diagnosis: ICD10 Code, F00-F99 Define	F01-F99 Mental, Behavioral and Neurodevelopmental disorders	491956	Lortab use disorder, mild, in controlled environment	ICD10CM	1	F11.90		Opioid use, unspecified, uncomplicated	IMO	GHS-PACDC	IMO_2015_R	ACTIVE	N	1900-01-01	2016-10-14	2015-07-10
Diagnosis: ICD10 Code, F00-F99 Define	F01-F99 Mental, Behavioral and Neurodevelopmental disorders	484315	Moderate diphenoxylate-atropine use disorder on maintenance therapy (HCC)	ICD10CM	1	F11.90		Opioid use, unspecified, uncomplicated	IMO	GHS-PACDC	IMO_2015_R	ACTIVE	N	1900-01-01	2016-10-14	2015-07-10
Diagnosis: ICD10 Code, F00-F99 Define	F01-F99 Mental, Behavioral and Neurodevelopmental disorders	492283	Percodan use disorder, moderate, in early remission (HCC)	ICD10CM	1	F11.90		Opioid use, unspecified, uncomplicated	IMO	GHS-PACDC	IMO_2015_R	ACTIVE	N	1900-01-01	2016-10-14	2015-07-10
Diagnosis: ICD10 Code, F00-F99 Define	F01-F99 Mental, Behavioral and Neurodevelopmental disorders	484219	Severe opium use disorder in early remission (HCC)	ICD10CM	1	F11.90		Opioid use, unspecified, uncomplicated	IMO	GHS-PACDC	IMO_2015_R	ACTIVE	N	1900-01-01	2016-10-14	2015-07-10
Diagnosis: ICD10 Code, F00-F99 Define	F01-F99 Mental, Behavioral and Neurodevelopmental disorders	492466	Stadol use disorder, mild, on maintenance therapy	ICD10CM	1	F11.90		Opioid use, unspecified, uncomplicated	IMO	GHS-PACDC	IMO_2015_R	ACTIVE	N	1900-01-01	2016-10-14	2015-07-10
Diagnosis: ICD10 Code, F00-F99 Define	F01-F99 Mental, Behavioral and Neurodevelopmental disorders	484838	Severe meperidine use disorder in sustained remission (HCC)	ICD10CM	1	F11.90		Opioid use, unspecified, uncomplicated	IMO	GHS-PACDC	IMO_2015_R	ACTIVE	N	1900-01-01	2016-10-14	2015-07-10
Diagnosis: ICD10 Code, F00-F99 Define	F01-F99 Mental, Behavioral and Neurodevelopmental disorders	492457	Heroin use disorder, severe, on maintenance therapy (HCC)	ICD10CM	1	F11.90		Opioid use, unspecified, uncomplicated	IMO	GHS-PACDC	IMO_2015_R	ACTIVE	N	1900-01-01	2016-10-14	2015-07-10
Diagnosis: ICD10 Code, F00-F99 Define	F01-F99 Mental, Behavioral and Neurodevelopmental disorders	485009	Mild diphenoxylate-atropine use disorder on maintenance therapy	ICD10CM	1	F11.90		Opioid use, unspecified, uncomplicated	IMO	GHS-PACDC	IMO_2015_R	ACTIVE	N	1900-01-01	2016-10-14	2015-07-10
Diagnosis: ICD10 Code, F00-F99 Define	F01-F99 Mental, Behavioral and Neurodevelopmental disorders	491818	Tylox use disorder, severe, in controlled environment (HCC)	ICD10CM	1	F11.90		Opioid use, unspecified, uncomplicated	IMO	GHS-PACDC	IMO_2015_R	ACTIVE	N	1900-01-01	2016-10-14	2015-07-10
Diagnosis: ICD10 Code, F00-F99 Define	F01-F99 Mental, Behavioral and Neurodevelopmental disorders	492475	Percocet use disorder, moderate, on maintenance therapy (HCC)	ICD10CM	1	F11.90		Opioid use, unspecified, uncomplicated	IMO	GHS-PACDC	IMO_2015_R	ACTIVE	N	1900-01-01	2016-10-14	2015-07-10
Diagnosis: ICD10 Code, F00-F99 Define	F01-F99 Mental, Behavioral and Neurodevelopmental disorders	484981	Mild butorphanol tartrate use disorder on maintenance therapy	ICD10CM	1	F11.90		Opioid use, unspecified, uncomplicated	IMO	GHS-PACDC	IMO_2015_R	ACTIVE	N	1900-01-01	2016-10-14	2015-07-10
Diagnosis: ICD10 Code, F00-F99 Define	F01-F99 Mental, Behavioral and Neurodevelopmental disorders	484096	Severe nalbuphine hydrochloride use disorder (HCC)	ICD10CM	1	F11.90		Opioid use, unspecified, uncomplicated	IMO	GHS-PACDC	IMO_2015_R	ACTIVE	N	1900-01-01	2016-10-14	2015-07-10
Diagnosis: ICD10 Code, F00-F99 Define	F01-F99 Mental, Behavioral and Neurodevelopmental disorders	484250	Moderate opium use disorder in early remission (HCC)	ICD10CM	1	F11.90		Opioid use, unspecified, uncomplicated	IMO	GHS-PACDC	IMO_2015_R	ACTIVE	N	1900-01-01	2016-10-14	2015-07-10
Diagnosis: ICD10 Code, F00-F99 Define	F01-F99 Mental, Behavioral and Neurodevelopmental disorders	492326	Nubain use disorder, mild, in sustained remission	ICD10CM	1	F11.90		Opioid use, unspecified, uncomplicated	IMO	GHS-PACDC	IMO_2015_R	ACTIVE	N	1900-01-01	2016-10-14	2015-07-10
Diagnosis: ICD10 Code, F00-F99 Define	F01-F99 Mental, Behavioral and Neurodevelopmental disorders	492154	Stadol use disorder, severe, in controlled environment (HCC)	ICD10CM	1	F11.90		Opioid use, unspecified, uncomplicated	IMO	GHS-PACDC	IMO_2015_R	ACTIVE	N	1900-01-01	2016-10-14	2015-07-10
Diagnosis: ICD10 Code, F00-F99 Define	F01-F99 Mental, Behavioral and Neurodevelopmental disorders	486702	Mild opioid use disorder, in early remission	ICD10CM	1	F11.90		Opioid use, unspecified, uncomplicated	IMO	GHS-PACDC	IMO_2015_R	ACTIVE	N	1900-01-01	2016-10-14	2015-07-10
Diagnosis: ICD10 Code, F00-F99 Define	F01-F99 Mental, Behavioral and Neurodevelopmental disorders	479930	Hydrocodone use disorder, moderate (HCC)	ICD10CM	1	F11.90		Opioid use, unspecified, uncomplicated	IMO	GHS-PACDC	IMO_2015_R	ACTIVE	N	1900-01-01	2016-10-14	2015-07-10
Diagnosis: ICD10 Code, F00-F99 Define	F01-F99 Mental, Behavioral and Neurodevelopmental disorders	484859	Moderate propoxyphene use disorder in sustained remission (HCC)	ICD10CM	1	F11.90		Opioid use, unspecified, uncomplicated	IMO	GHS-PACDC	IMO_2015_R	ACTIVE	N	1900-01-01	2016-10-14	2015-07-10
Diagnosis: ICD10 Code, F00-F99 Define	F01-F99 Mental, Behavioral and Neurodevelopmental disorders	492003	Methadone use disorder, mild, in sustained remission	ICD10CM	1	F11.90		Opioid use, unspecified, uncomplicated	IMO	GHS-PACDC	IMO_2015_R	ACTIVE	N	1900-01-01	2016-10-14	2015-07-10
Diagnosis: ICD10 Code, F00-F99 Define	F01-F99 Mental, Behavioral and Neurodevelopmental disorders	484229	Moderate triazolam use disorder in controlled environment (HCC)	ICD10CM	1	F11.90		Opioid use, unspecified, uncomplicated	IMO	GHS-PACDC	IMO_2015_R	ACTIVE	N	1900-01-01	2016-10-14	2015-07-10
Diagnosis: ICD10 Code, F00-F99 Define	F01-F99 Mental, Behavioral and Neurodevelopmental disorders	484193	Moderate dextromethorphan use disorder in controlled environment (HCC)	ICD10CM	1	F11.90		Opioid use, unspecified, uncomplicated	IMO	GHS-PACDC	IMO_2015_R	ACTIVE	N	1900-01-01	2016-10-14	2015-07-10
Diagnosis: ICD10 Code, F00-F99 Define	F01-F99 Mental, Behavioral and Neurodevelopmental disorders	484795	Severe oxycodone use disorder on maintenance therapy (HCC)	ICD10CM	1	F11.90		Opioid use, unspecified, uncomplicated	IMO	GHS-PACDC	IMO_2015_R	ACTIVE	N	1900-01-01	2016-10-14	2015-07-10
Diagnosis: ICD10 Code, F00-F99 Define	F01-F99 Mental, Behavioral and Neurodevelopmental disorders	484221	Mild dextromethorphan use disorder in sustained remission	ICD10CM	1	F11.90		Opioid use, unspecified, uncomplicated	IMO	GHS-PACDC	IMO_2015_R	ACTIVE	N	1900-01-01	2016-10-14	2015-07-10
Diagnosis: ICD10 Code, F00-F99 Define	F01-F99 Mental, Behavioral and Neurodevelopmental disorders	492449	Fentanyl use disorder, moderate, on maintenance therapy (HCC)	ICD10CM	1	F11.90		Opioid use, unspecified, uncomplicated	IMO	GHS-PACDC	IMO_2015_R	ACTIVE	N	1900-01-01	2016-10-14	2015-07-10
Diagnosis: ICD10 Code, F00-F99 Define	F01-F99 Mental, Behavioral and Neurodevelopmental disorders	479933	Oxycontin use disorder, moderate (HCC)	ICD10CM	1	F11.90		Opioid use, unspecified, uncomplicated	IMO	GHS-PACDC	IMO_2015_R	ACTIVE	N	1900-01-01	2016-10-14	2015-07-10
Diagnosis: ICD10 Code, F00-F99 Define	F01-F99 Mental, Behavioral and Neurodevelopmental disorders	485835	Moderate opioid use disorder, in early remission, in controlled environment (HCC)	ICD10CM	1	F11.90		Opioid use, unspecified, uncomplicated	IMO	GHS-PACDC	IMO_2015_R	ACTIVE	N	1900-01-01	2016-10-14	2015-07-10
Diagnosis: ICD10 Code, F00-F99 Define	F01-F99 Mental, Behavioral and Neurodevelopmental disorders	492462	Morphine use disorder, mild, on maintenance therapy	ICD10CM	1	F11.90		Opioid use, unspecified, uncomplicated	IMO	GHS-PACDC	IMO_2015_R	ACTIVE	N	1900-01-01	2016-10-14	2015-07-10
Diagnosis: ICD10 Code, F00-F99 Define	F01-F99 Mental, Behavioral and Neurodevelopmental disorders	452089	Acute narcotic intoxication without complication (HCC)	ICD10CM	1	F11.920		Opioid use, unspecified with intoxication, uncomplicatec	IMO	GHS-PACDC	IMO_2015_R	ACTIVE	N	2017-03-31	9999-12-31	2017-04-14
Diagnosis: ICD10 Code, F00-F99 Define	F01-F99 Mental, Behavioral and Neurodevelopmental disorders	516897	Opioid intoxication without perceptual disturbances without use disorder without complication	ICD10CM	1	F11.920		Opioid use, unspecified with intoxication, uncomplicatec	IMO	GHS-PACDC	IMO_2015_R	ACTIVE	N	1900-01-01	9999-12-31	2015-07-10
Diagnosis: ICD10 Code, F00-F99 Define	F01-F99 Mental, Behavioral and Neurodevelopmental disorders	968303	Opioid intoxication without perceptual disturbances with moderate or severe use disorder, unc	ICD10CM	1	F11.920		Opioid use, unspecified with intoxication, uncomplicatec	IMO	GHS-PACDC	IMO_2015_R	ACTIVE	N	1900-01-01	2016-10-14	2015-07-10
Diagnosis: ICD10 Code, F00-F99 Define	F01-F99 Mental, Behavioral and Neurodevelopmental disorders	967445	Opioid intoxication without perceptual disturbances without use disorder, uncomplicated (HCC)	ICD10CM	1	F11.920		Opioid use, unspecified with intoxication, uncomplicatec	IMO	GHS-PACDC	IMO_2015_R	ACTIVE	N	1900-01-01	9999-12-31	2015-07-10
Diagnosis: ICD10 Code, F00-F99 Define	F01-F99 Mental, Behavioral and Neurodevelopmental disorders	517260	Opioid intoxication without perceptual disturbances without use disorder with delirium (HCC)	ICD10CM	1	F11.921		Opioid use, unspecified with intoxication delirium	IMO	GHS-PACDC	IMO_2015_R	ACTIVE	N	1900-01-01	9999-12-31	2015-07-10
Diagnosis: ICD10 Code, F00-F99 Define	F01-F99 Mental, Behavioral and Neurodevelopmental disorders	486318	Acute, mixed level of activity, opioid intoxication delirium without use disorder (HCC)	ICD10CM	1	F11.921		Opioid use, unspecified with intoxication delirium	IMO	GHS-PACDC	IMO_2015_R	ACTIVE	N	1900-01-01	9999-12-31	2015-07-10
Diagnosis: ICD10 Code, F00-F99 Define	F01-F99 Mental, Behavioral and Neurodevelopmental disorders	429054	Opioid intoxication without perceptual disturbances with mild use disorder (HCC)	ICD10CM	1	F11.929		Opioid use, unspecified with intoxication, unspecified	IMO	GHS-PACDC	IMO_2015_R	ACTIVE	N	1900-01-01	2016-10-14	2015-07-10
Diagnosis: ICD10 Code, F00-F99 Define	F01-F99 Mental, Behavioral and Neurodevelopmental disorders	518509	Opioid-induced mood disorder with onset during intoxication without complication (HCC)	ICD10CM	1	F11.94	F11.94,F11.920	Opioid use, unspecified with opioid-induced mood disor	IMO	GHS-PACDC	IMO_2015_R	ACTIVE	N	1900-01-01	2017-10-14	2015-07-10
Diagnosis: ICD10 Code, F00-F99 Define	F01-F99 Mental, Behavioral and Neurodevelopmental disorders	518360	Opioid-induced mood disorder with onset during intoxication with complication (HCC)	ICD10CM	2	F11.94	F11.929,F11.94	Opioid use, unspecified with opioid-induced mood disor	IMO	GHS-PACDC	IMO_2015_R	ACTIVE	N	2017-09-17	9999-12-31	2017-10-30
Diagnosis: ICD10 Code, F00-F99 Define	F01-F99 Mental, Behavioral and Neurodevelopmental disorders	966753	Onset of opioid-induced mood disorder during intoxication, with unspecified complication (HCC	ICD10CM	2	F11.94	F11.929,F11.94	Opioid use, unspecified with opioid-induced mood disor	IMO	GHS-PACDC	IMO_2015_R	ACTIVE	N	2017-09-17	9999-12-31	2017-10-30
Diagnosis: ICD10 Code, F00-F99 Define	F01-F99 Mental, Behavioral and Neurodevelopmental disorders	30163	Opioid-induced sexual dysfunction (HCC)	ICD10CM	1	F11.981		Opioid use, unspecified with opioid-induced sexual dysfi	IMO	GHS-PACDC	DX_LEGACY	ACTIVE	N	1900-01-01	9999-12-31	2015-07-10
Diagnosis: ICD10 Code, F00-F99 Define	F01-F99 Mental, Behavioral and Neurodevelopmental disorders	489541	Opioid-induced sexual dysfunction, with onset during intoxication, mild (HCC)	ICD10CM	1	F11.981		Opioid use, unspecified with opioid-induced sexual dysfi	IMO	GHS-PACDC	IMO_2015_R	ACTIVE	N	1900-01-01	9999-12-31	2015-07-10
Diagnosis: ICD10 Code, F00-F99 Define	F01-F99 Mental, Behavioral and Neurodevelopmental disorders	489057	Onset of opioid-induced sleep disorder, insomnia type, during intoxication (HCC)	ICD10CM	1	F11.982		Opioid use, unspecified with opioid-induced sleep disor	IMO	GHS-PACDC	IMO_2015_R	ACTIVE	N	1900-01-01	9999-12-31	2015-07-10
Diagnosis: ICD10 Code, F00-F99 Define	F01-F99 Mental, Behavioral and Neurodevelopmental disorders	487083	Opioid-induced sleep disorder with moderate or severe use disorder, parasomnia type (HCC)	ICD10CM	1	F11.982		Opioid use, unspecified with opioid-induced sleep disor	IMO	GHS-PACDC	IMO_2015_R	ACTIVE	N	1900-01-01	2016-10-14	2015-07-10
Diagnosis: ICD10 Code, F00-F99 Define	F01-F99 Mental, Behavioral and Neurodevelopmental disorders	487041	Opioid-induced sleep disorder without use disorder, insomnia type (HCC)	ICD10CM	1	F11.982		Opioid use, unspecified with opioid-induced sleep disor	IMO	GHS-PACDC	IMO_2015_R	ACTIVE	N	1900-01-01	9999-12-31	2015-07-10
Diagnosis: ICD10 Code, F00-F99 Define	F01-F99 Mental, Behavioral and Neurodevelopmental disorders	487161	Opioid-induced sleep disorder with moderate or severe use disorder, daytime sleepiness type (I	ICD10CM	1	F11.982		Opioid use, unspecified with opioid-induced sleep disor	IMO	GHS-PACDC	IMO_2015_R	ACTIVE	N	1900-01-01	2016-10-14	2015-07-10
Diagnosis: ICD10 Code, F00-F99 Define	F01-F99 Mental, Behavioral and Neurodevelopmental disorders	429069	Opioid-induced sleep disorder with mild use disorder (HCC)	ICD10CM	1	F11.982		Opioid use, unspecified with opioid-induced sleep disor	IMO	GHS-PACDC	IMO_2015_R	ACTIVE	N	1900-01-01	2016-10-14	2015-07-10
Diagnosis: ICD10 Code, F00-F99 Define	F01-F99 Mental, Behavioral and Neurodevelopmental disorders	485875	Opioid-induced anxiety disorder with moderate or severe use disorder with onset during intoxi	ICD10CM	1	F11.988	F11.988,F11.929	Opioid use, unspecified with other opioid-induced disor	IMO	GHS-PACDC	IMO_2015_R	ACTIVE	N	1900-01-01	2016-10-14	2015-07-10
Diagnosis: ICD10 Code, F00-F99 Define	F01-F99 Mental, Behavioral and Neurodevelopmental disorders	486453	Opioid-induced anxiety disorder with mild use disorder with onset during intoxication (HCC)	ICD10CM	1	F11.988	F11.988,F11.929	Opioid use, unspecified with other opioid-induced disor	IMO	GHS-PACDC	IMO_2015_R	ACTIVE	N	1900-01-01	2016-10-14	2015-07-10
Diagnosis: ICD10 Code, F00-F99 Define	F01-F99 Mental, Behavioral and Neurodevelopmental disorders	510005	Tetrahydrocannabinol (THC) use disorder, mild, in early remission, in controlled environment, a	ICD10CM	1	F12.10		Cannabis abuse, uncomplicated	IMO	GHS-PACDC	IMO_2015_R	ACTIVE	N	1900-01-01	2017-10-14	2015-07-10
Diagnosis: ICD10 Code, F00-F99 Define	F01-F99 Mental, Behavioral and Neurodevelopmental disorders	78221	Cannabis abuse	ICD10CM	1	F12.10		Cannabis abuse, uncomplicated	IMO	GHS-PACDC	DX_LEGACY	ACTIVE	N	1900-01-01	9999-12-31	2015-07-10
Diagnosis: ICD10 Code, F00-F99 Define	F01-F99 Mental, Behavioral and Neurodevelopmental disorders	83745	Cannabis abuse, episodic	ICD10CM	1	F12.10		Cannabis abuse, uncomplicated	IMO	GHS-PACDC	DX_LEGACY	ACTIVE	N	1900-01-01	9999-12-31	2015-07-10
Diagnosis: ICD10 Code, F00-F99 Define	F01-F99 Mental, Behavioral and Neurodevelopmental disorders	507749	Cannabis use disorder, mild, in early remission, in controlled environment, abuse	ICD10CM	1	F12.10		Cannabis abuse, uncomplicated	IMO	GHS-PACDC	IMO_2015_R	ACTIVE	N	1900-01-01	2017-10-14	2015-07-10
Diagnosis: ICD10 Code, F00-F99 Define	F01-F99 Mental, Behavioral and Neurodevelopmental disorders	3183	Nondependent cannabis abuse, continuous	ICD10CM	1	F12.10		Cannabis abuse, uncomplicated	IMO	GHS-PACDC	DX_LEGACY	ACTIVE	N	1900-01-01	9999-12-31	2015-07-10
Diagnosis: ICD10 Code, F00-F99 Define	F01-F99 Mental, Behavioral and Neurodevelopmental disorders	508102	Mild dronabinol abuse in early remission	ICD10CM	1	F12.11		Cannabis abuse, in remission	IMO	GHS-PACDC	IMO_2015_R	ACTIVE	N	2017-09-17	9999-12-31	2017-10-30
Diagnosis: ICD10 Code, F00-F99 Define	F01-F99 Mental, Behavioral and Neurodevelopmental disorders	508415	Tetrahydrocannabinol (THC) use disorder, mild, in sustained remission, in controlled environme	ICD10CM	1	F12.11		Cannabis abuse, in remission	IMO	GHS-PACDC	IMO_2015_R	ACTIVE	N	2017-09-17	9999-12-31	2017-10-30
Diagnosis: ICD10 Code, F00-F99 Define	F01-F99 Mental, Behavioral and Neurodevelopmental disorders	490353	Marijuana abuse in remission	ICD10CM	1	F12.11		Cannabis abuse, in remission	IMO	GHS-PACDC	IMO_2015_R	ACTIVE	N	2017-09-17	9999-12-31	2017-10-30
Diagnosis: ICD10 Code, F00-F99 Define	F01-F99 Mental, Behavioral and Neurodevelopmental disorders	1185541	Cannabis abuse with intoxication, uncomplicated (HCC)	ICD10CM	1	F12.120		Cannabis abuse with intoxication, uncomplicated	IMO	GHS-PACDC	IMO_2016_R	ACTIVE	N	1900-01-01	9999-12-31	2016-06-02
Diagnosis: ICD10 Code, F00-F99 Define	F01-F99 Mental, Behavioral and Neurodevelopmental disorders	486736	Acute cannabis intoxication delirium with mild use disorder (HCC)	ICD10CM	1	F12.121		Cannabis abuse with intoxication delirium	IMO	GHS-PACDC	IMO_2015_R	ACTIVE	N	1900-01-01	9999-12-31	2015-07-10
Diagnosis: ICD10 Code, F00-F99 Define	F01-F99 Mental, Behavioral and Neurodevelopmental disorders	517936	Cannabis intoxication without perceptual disturbances with mild use disorder with delirium (HC	ICD10CM	1	F12.121		Cannabis abuse with intoxication delirium	IMO	GHS-PACDC	IMO_2015_R	ACTIVE	N	2016-09-17	9999-12-31	2016-09-30
Diagnosis: ICD10 Code, F00-F99 Define	F01-F99 Mental, Behavioral and Neurodevelopmental disorders	429217	Onset of cannabis-induced psychotic disorder with hallucinations during intoxication (HCC)	ICD10CM	1	F12.129	F12.129,F12.151	Cannabis abuse with intoxication, unspecified	IMO	GHS-PACDC	IMO_2015_R	ACTIVE	N	1900-01-01	9999-12-31	2015-07-10
Diagnosis: ICD10 Code, F00-F99 Define	F01-F99 Mental, Behavioral and Neurodevelopmental disorders	458908	Cannabis abuse with psychotic disorder with complication (HCC)	ICD10CM	1	F12.159		Cannabis abuse with psychotic disorder, unspecified	IMO	GHS-PACDC	IMO_2015_R	ACTIVE	N	1900-01-01	9999-12-31	2015-07-10
Diagnosis: ICD10 Code, F00-F99 Define	F01-F99 Mental, Behavioral and Neurodevelopmental disorders	428848	Cannabis dependence with or without physiological dependence (HCC)	ICD10CM	1	F12.20		Cannabis dependence, uncomplicated	IMO	GHS-PACDC	IMO_2015_R	ACTIVE	N	1900-01-01	9999-12-31	2015-07-10
Diagnosis: ICD10 Code, F00-F99 Define	F01-F99 Mental, Behavioral and Neurodevelopmental disorders	212588	Amphetamine dependence, continuous (HCC)	ICD10CM	1	F12.20		Cannabis dependence, uncomplicated	IMO	GHS-PACDC	DX_LEGACY	ACTIVE	N	1900-01-01	2017-10-14	2015-07-10
Diagnosis: ICD10 Code, F00-F99 Define	F01-F99 Mental, Behavioral and Neurodevelopmental disorders	106705	Dependence, hashish (HCC)	ICD10CM	1	F12.20		Cannabis dependence, uncomplicated	IMO	GHS-PACDC	DX_LEGACY	ACTIVE	N	1900-01-01	9999-12-31	2015-07-10
Diagnosis: ICD10 Code, F00-F99 Define	F01-F99 Mental, Behavioral and Neurodevelopmental disorders	489193	Moderate cannabis use disorder in controlled environment (HCC)	ICD10CM	1	F12.20		Cannabis dependence, uncomplicated	IMO	GHS-PACDC	IMO_2015_R	ACTIVE	N	2016-09-17	9999-12-31	2016-09-30
Diagnosis: ICD10 Code, F00-F99 Define	F01-F99 Mental, Behavioral and Neurodevelopmental disorders	486315	Moderate cannabis use disorder, in early remission (HCC)	ICD10CM	1	F12.21		Cannabis dependence, in remission	IMO	GHS-PACDC	IMO_2015_R	ACTIVE	N	2016-09-17	9999-12-31	2016-09-30
Diagnosis: ICD10 Code, F00-F99 Define	F01-F99 Mental, Behavioral and Neurodevelopmental disorders	509608	Moderate tetrahydrocannabinol (THC) dependence in sustained remission (HCC)	ICD10CM	1	F12.21		Cannabis dependence, in remission	IMO	GHS-PACDC	IMO_2015_R	ACTIVE	N	1900-01-01	9999-12-31	2015-07-10
Diagnosis: ICD10 Code, F00-F99 Define	F01-F99 Mental, Behavioral and Neurodevelopmental disorders	954314	Cannabis dependence with psychotic disorder, with delusions (HCC)	ICD10CM	1	F12.250		Cannabis dependence with psychotic disorder with delu	IMO	GHS-PACDC	IMO_2015_R	ACTIVE	N	1900-01-01	9999-12-31	2015-07-10
Diagnosis: ICD10 Code, F00-F99 Define	F01-F99 Mental, Behavioral and Neurodevelopmental disorders	270385	Cannabis dependence with psychotic disorder (HCC)	ICD10CM	1	F12.259		Cannabis dependence with psychotic disorder, unspecifi	IMO	GHS-PACDC	DX_LEGACY	ACTIVE	N	1900-01-01	9999-12-31	2015-07-10
Diagnosis: ICD10 Code, F00-F99 Define	F01-F99 Mental, Behavioral and Neurodevelopmental disorders	519581	Cannabis-induced psychotic disorder with mild use disorder with onset during withdrawal with	ICD10CM	1	F12.259	F12.259,F19.239	Cannabis dependence with psychotic disorder, unspecifi	IMO	GHS-PACDC	IMO_2015_R	ACTIVE	N	1900-01-01	2016-10-14	2015-07-10
Diagnosis: ICD10 Code, F00-F99 Define	F01-F99 Mental, Behavioral and Neurodevelopmental disorders	428855	Cannabis-induced anxiety disorder with moderate or severe use disorder (HCC)	ICD10CM	1	F12.280		Cannabis dependence with cannabis-induced anxiety dis	IMO	GHS-PACDC	IMO_2015_R	ACTIVE	N	2016-09-17	9999-12-31	2016-09-30
Diagnosis: ICD10 Code, F00-F99 Define	F01-F99 Mental, Behavioral and Neurodevelopmental disorders	486463	Cannabis-induced anxiety disorder without use disorder with onset during withdrawal (HCC)	ICD10CM	1	F12.280	F12.280,F12.288	Cannabis dependence with cannabis-induced anxiety dis	IMO	GHS-PACDC	IMO_2015_R	ACTIVE	N	2016-09-17	9999-12-31	2016-09-30
Diagnosis: ICD10 Code, F00-F99 Define	F01-F99 Mental, Behavioral and Neurodevelopmental disorders	524715	Cannabis-induced psychotic disorder with mild use disorder with onset during withdrawal with	ICD10CM	2	F12.288	F12.251,F12.288	Cannabis dependence with other cannabis-induced diso	IMO	GHS-PACDC	IMO_2015_R	ACTIVE	N	2016-09-17	9999-12-31	2016-09-30
Diagnosis: ICD10 Code, F00-F99 Define	F01-F99 Mental, Behavioral and Neurodevelopmental disorders	293179	Cannabis use, uncomplicated	ICD10CM	1	F12.90		Cannabis use, unspecified, uncomplicated	IMO	GHS-PACDC	DX_LEGACY	ACTIVE	N	1900-01-01	9999-12-31	2015-07-10
Diagnosis: ICD10 Code, F00-F99 Define	F01-F99 Mental, Behavioral and Neurodevelopmental disorders	487053	Moderate cannabis use disorder, in sustained remission, in controlled environment (HCC)	ICD10CM	1	F12.90		Cannabis use, unspecified, uncomplicated	IMO	GHS-PACDC	IMO_2015_R	ACTIVE	N	1900-01-01	2016-10-14	2015-07-10
Diagnosis: ICD10 Code, F00-F99 Define	F01-F99 Mental, Behavioral and Neurodevelopmental disorders	485808	Moderate cannabis use disorder, in early remission, in controlled environment (HCC)	ICD10CM	1	F12.90		Cannabis use, unspecified, uncomplicated	IMO	GHS-PACDC	IMO_2015_R	ACTIVE	N	1900-01-01	2016-10-14	2015-07-10
Diagnosis: ICD10 Code, F00-F99 Define	F01-F99 Mental, Behavioral and Neurodevelopmental disorders	414860	Uses marijuana	ICD10CM	1	F12.90		Cannabis use, unspecified, uncomplicated	IMO	GHS-PACDC	DX_LEGACY	ACTIVE	N	1900-01-01	9999-12-31	2015-07-10
Diagnosis: ICD10 Code, F00-F99 Define	F01-F99 Mental, Behavioral and Neurodevelopmental disorders	612343	Cannabis use, unspecified with intoxication	ICD10CM	1	F12.92		Cannabis use, unspecified with intoxication	IMO	GHS-PACDC	IMO_2015_R	ACTIVE	Y	1900-01-01	9999-12-31	2015-07-10
Diagnosis: ICD10 Code, F00-F99 Define	F01-F99 Mental, Behavioral and Neurodevelopmental disorders	1298321	Cannabis intoxication with delirium (HCC)	ICD10CM	1	F12.921		Cannabis use, unspecified with intoxication delirium	IMO	GHS-PACDC	IMO_2017_R	ACTIVE	N	1900-01-01	9999-12-31	2017-04-14
Diagnosis: ICD10 Code, F00-F99 Define	F01-F99 Mental, Behavioral and Neurodevelopmental disorders	485877	Persistent, mixed level of activity, cannabis intoxication delirium without use disorder (HCC)	ICD10CM	1	F12.921		Cannabis use, unspecified with intoxication delirium	IMO	GHS-PACDC	IMO_2015_R	ACTIVE	N	2016-09-17	9999-12-31	2016-09-30
Diagnosis: ICD10 Code, F00-F99 Define	F01-F99 Mental, Behavioral and Neurodevelopmental disorders	524792	Cannabis-induced psychotic disorder with mild use disorder with delusions with perceptual dist	ICD10CM	2	F12.922	F12.950,F12.922	Cannabis use, unspecified with intoxication with percept	IMO	GHS-PACDC	IMO_2015_R	ACTIVE	N	1900-01-01	2016-10-14	2015-07-10
Diagnosis: ICD10 Code, F00-F99 Define	F01-F99 Mental, Behavioral and Neurodevelopmental disorders	524650	Cannabis-induced psychotic disorder without use disorder with onset during withdrawal with ha	ICD10CM	2	F12.922	F12.951,F12.922	Cannabis use, unspecified with intoxication with percept	IMO	GHS-PACDC	IMO_2015_R	ACTIVE	N	1900-01-01	2016-10-14	2015-07-10
Diagnosis: ICD10 Code, F00-F99 Define	F01-F99 Mental, Behavioral and Neurodevelopmental disorders	1179736	Cannabis use, unspecified with psychotic disorder with hallucinations (HCC)	ICD10CM	1	F12.951		Cannabis use, unspecified with psychotic disorder with h	IMO	GHS-PACDC	IMO_2016_R	ACTIVE	N	1900-01-01	9999-12-31	2016-06-02
Diagnosis: ICD10 Code, F00-F99 Define	F01-F99 Mental, Behavioral and Neurodevelopmental disorders	533033	Cannabis-induced psychotic disorder with mild use disorder with hallucinations and delirium (H	ICD10CM	1	F12.951	F12.951,F12.921	Cannabis use, unspecified with psychotic disorder with h	IMO	GHS-PACDC	IMO_2015_R	ACTIVE	N	1900-01-01	2016-10-14	2015-07-10
Diagnosis: ICD10 Code, F00-F99 Define	F01-F99 Mental, Behavioral and Neurodevelopmental disorders	524631	Cannabis-induced psychotic disorder without use disorder with onset during withdrawal with ha	ICD10CM	1	F12.951	F12.951,F12.921	Cannabis use, unspecified with psychotic disorder with h	IMO	GHS-PACDC	IMO_2015_R	ACTIVE	N	1900-01-01	2016-10-14	2015-07-10
Diagnosis: ICD10 Code, F00-F99 Define	F01-F99 Mental, Behavioral and Neurodevelopmental disorders	1183618	Cannabis use, unspecified with psychotic disorder, unspecified (HCC)	ICD10CM	1	F12.959		Cannabis use, unspecified with psychotic disorder, unsp	IMO	GHS-PACDC	IMO_2016_R	ACTIVE	N	1900-01-01	9999-12-31	2016-06-02
Diagnosis: ICD10 Code, F00-F99 Define	F01-F99 Mental, Behavioral and Neurodevelopmental disorders	488724	Onset of cannabis-induced psychotic disorder during intoxication (HCC)	ICD10CM	1	F12.959	F12.959,F12.929	Cannabis use, unspecified with psychotic disorder, unsp	IMO	GHS-PACDC	IMO_2015_R	ACTIVE	N	1900-01-01	9999-12-31	2015-07-10
Diagnosis: ICD10 Code, F00-F99 Define	F01-F99 Mental, Behavioral and Neurodevelopmental disorders	485979	Cannabis-induced sleep disorder without use disorder, parasomnia type (HCC)	ICD10CM	1	F12.988		Cannabis use, unspecified with other cannabis-induced c	IMO	GHS-PACDC	IMO_2015_R	ACTIVE	N	1900-01-01	9999-12-31	2015-07-10
Diagnosis: ICD10 Code, F00-F99 Define	F01-F99 Mental, Behavioral and Neurodevelopmental disorders	487182	Cannabis-induced sleep disorder without use disorder, insomnia type (HCC)	ICD10CM	1	F12.988		Cannabis use, unspecified with other cannabis-induced c	IMO	GHS-PACDC	IMO_2015_R	ACTIVE	N	1900-01-01	9999-12-31	2015-07-10
Diagnosis: ICD10 Code, F00-F99 Define	F01-F99 Mental, Behavioral and Neurodevelopmental disorders	486791	Cannabis-induced sleep disorder with mild use disorder, daytime sleepiness type (HCC)	ICD10CM	1	F12.988		Cannabis use, unspecified with other cannabis-induced c	IMO	GHS-PACDC	IMO_2015_R	ACTIVE	N	1900-01-01	2016-10-14	2015-07-10
Diagnosis: ICD10 Code, F00-F99 Define	F01-F99 Mental, Behavioral and Neurodevelopmental disorders	489350	Onset of cannabis-induced sleep disorder, mixed type, during intoxication (HCC)	ICD10CM	1	F12.988		Cannabis use, unspecified with other cannabis-induced c	IMO	GHS-PACDC	IMO_2015_R	ACTIVE	N	1900-01-01	9999-12-31	2015-07-10
Diagnosis: ICD10 Code, F00-F99 Define	F01-F99 Mental, Behavioral and Neurodevelopmental disorders	487067	Cannabis-induced sleep disorder with moderate or severe use disorder, daytime sleepiness type	ICD10CM	1	F12.988		Cannabis use, unspecified with other cannabis-induced c	IMO	GHS-PACDC	IMO_2015_R	ACTIVE	N	1900-01-01	2016-10-14	2015-07-10
Diagnosis: ICD10 Code, F00-F99 Define	F01-F99 Mental, Behavioral and Neurodevelopmental disorders	508894	Halcion use disorder, mild, in early remission, abuse	ICD10CM	1	F13.10		Sedative, hypnotic or anxiolytic abuse, uncomplicated	IMO	GHS-PACDC	IMO_2015_R	ACTIVE	N	1900-01-01	2017-10-14	2015-07-10
Diagnosis: ICD10 Code, F00-F99 Define	F01-F99 Mental, Behavioral and Neurodevelopmental disorders	510019	Mild pentobarbital abuse in sustained remission in controlled environment	ICD10CM	1	F13.10		Sedative, hypnotic or anxiolytic abuse, uncomplicated	IMO	GHS-PACDC	IMO_2015_R	ACTIVE	N	1900-01-01	2017-10-14	2015-07-10
Diagnosis: ICD10 Code, F00-F99 Define	F01-F99 Mental, Behavioral and Neurodevelopmental disorders	484581	Mild barbiturate use disorder in sustained remission	ICD10CM	1	F13.10		Sedative, hypnotic or anxiolytic abuse, uncomplicated	IMO	GHS-PACDC	IMO_2015_R	ACTIVE	N	1900-01-01	9999-12-31	2015-07-10
Diagnosis: ICD10 Code, F00-F99 Define	F01-F99 Mental, Behavioral and Neurodevelopmental disorders	484786	Mild temazepam use disorder in controlled environment	ICD10CM	1	F13.10		Sedative, hypnotic or anxiolytic abuse, uncomplicated	IMO	GHS-PACDC	IMO_2015_R	ACTIVE	N	2016-09-17	9999-12-31	2016-09-30

Diagnosis: ICD10 Code, F00-F99 Define	F01-F99 Mental, Behavioral and Neurodevelopmental disorders	507925	Mild lorazepam abuse in sustained remission	ICD10CM	1	F13.10		Sedative, hypnotic or anxiolytic abuse, uncomplicated	IMO	GHS-PACDC	IMO_2015_R	ACTIVE	N	1900-01-01	2017-10-14	2015-07-10
Diagnosis: ICD10 Code, F00-F99 Define	F01-F99 Mental, Behavioral and Neurodevelopmental disorders	485003	Mild gamma-hydroxybutyrate use disorder in controlled environment	ICD10CM	1	F13.10		Sedative, hypnotic or anxiolytic abuse, uncomplicated	IMO	GHS-PACDC	IMO_2015_R	ACTIVE	N	2016-09-17	9999-12-31	2016-09-30
Diagnosis: ICD10 Code, F00-F99 Define	F01-F99 Mental, Behavioral and Neurodevelopmental disorders	484286	Moderate clonazepam use disorder in sustained remission (HCC)	ICD10CM	1	F13.10		Sedative, hypnotic or anxiolytic abuse, uncomplicated	IMO	GHS-PACDC	IMO_2015_R	ACTIVE	N	1900-01-01	2016-10-14	2015-07-10
Diagnosis: ICD10 Code, F00-F99 Define	F01-F99 Mental, Behavioral and Neurodevelopmental disorders	484799	Mild lorazepam use disorder in sustained remission	ICD10CM	1	F13.10		Sedative, hypnotic or anxiolytic abuse, uncomplicated	IMO	GHS-PACDC	IMO_2015_R	ACTIVE	N	2016-09-17	9999-12-31	2016-09-30
Diagnosis: ICD10 Code, F00-F99 Define	F01-F99 Mental, Behavioral and Neurodevelopmental disorders	408270	Benzodiazepine misuse	ICD10CM	1	F13.10		Sedative, hypnotic or anxiolytic abuse, uncomplicated	IMO	GHS-PACDC	DX_LEGACY	ACTIVE	N	1900-01-01	9999-12-31	2015-07-10
Diagnosis: ICD10 Code, F00-F99 Define	F01-F99 Mental, Behavioral and Neurodevelopmental disorders	509738	Mild secobarbital sodium abuse in sustained remission	ICD10CM	1	F13.10		Sedative, hypnotic or anxiolytic abuse, uncomplicated	IMO	GHS-PACDC	IMO_2015_R	ACTIVE	N	1900-01-01	2017-10-14	2015-07-10
Diagnosis: ICD10 Code, F00-F99 Define	F01-F99 Mental, Behavioral and Neurodevelopmental disorders	484467	Moderate clonazepam use disorder in early remission (HCC)	ICD10CM	1	F13.10		Sedative, hypnotic or anxiolytic abuse, uncomplicated	IMO	GHS-PACDC	IMO_2015_R	ACTIVE	N	1900-01-01	2016-10-14	2015-07-10
Diagnosis: ICD10 Code, F00-F99 Define	F01-F99 Mental, Behavioral and Neurodevelopmental disorders	509034	Lunesta use disorder, mild, in sustained remission, abuse	ICD10CM	1	F13.10		Sedative, hypnotic or anxiolytic abuse, uncomplicated	IMO	GHS-PACDC	IMO_2015_R	ACTIVE	N	1900-01-01	2017-10-14	2015-07-10
Diagnosis: ICD10 Code, F00-F99 Define	F01-F99 Mental, Behavioral and Neurodevelopmental disorders	508794	Mild triazolam abuse in controlled environment	ICD10CM	1	F13.10		Sedative, hypnotic or anxiolytic abuse, uncomplicated	IMO	GHS-PACDC	IMO_2015_R	ACTIVE	N	1900-01-01	9999-12-31	2015-07-10
Diagnosis: ICD10 Code, F00-F99 Define	F01-F99 Mental, Behavioral and Neurodevelopmental disorders	508383	Mild eszopiclone abuse in controlled environment	ICD10CM	1	F13.10		Sedative, hypnotic or anxiolytic abuse, uncomplicated	IMO	GHS-PACDC	IMO_2015_R	ACTIVE	N	1900-01-01	9999-12-31	2015-07-10
Diagnosis: ICD10 Code, F00-F99 Define	F01-F99 Mental, Behavioral and Neurodevelopmental disorders	509699	Tranxene use disorder, mild, abuse	ICD10CM	1	F13.10		Sedative, hypnotic or anxiolytic abuse, uncomplicated	IMO	GHS-PACDC	IMO_2015_R	ACTIVE	N	1900-01-01	9999-12-31	2015-07-10
Diagnosis: ICD10 Code, F00-F99 Define	F01-F99 Mental, Behavioral and Neurodevelopmental disorders	509784	Propofol use disorder, mild, in early remission, in controlled environment, abuse	ICD10CM	1	F13.10		Sedative, hypnotic or anxiolytic abuse, uncomplicated	IMO	GHS-PACDC	IMO_2015_R	ACTIVE	N	1900-01-01	2016-10-14	2015-07-10
Diagnosis: ICD10 Code, F00-F99 Define	F01-F99 Mental, Behavioral and Neurodevelopmental disorders	507779	Mild oxazepam abuse in early remission in controlled environment	ICD10CM	1	F13.10		Sedative, hypnotic or anxiolytic abuse, uncomplicated	IMO	GHS-PACDC	IMO_2015_R	ACTIVE	N	1900-01-01	2017-10-14	2015-07-10
Diagnosis: ICD10 Code, F00-F99 Define	F01-F99 Mental, Behavioral and Neurodevelopmental disorders	483963	Mild oxazepam use disorder	ICD10CM	1	F13.10		Sedative, hypnotic or anxiolytic abuse, uncomplicated	IMO	GHS-PACDC	IMO_2015_R	ACTIVE	N	2016-09-17	9999-12-31	2016-09-30
Diagnosis: ICD10 Code, F00-F99 Define	F01-F99 Mental, Behavioral and Neurodevelopmental disorders	507043	Mild lorazepam abuse in early remission in controlled environment	ICD10CM	1	F13.10		Sedative, hypnotic or anxiolytic abuse, uncomplicated	IMO	GHS-PACDC	IMO_2015_R	ACTIVE	N	1900-01-01	2017-10-14	2015-07-10
Diagnosis: ICD10 Code, F00-F99 Define	F01-F99 Mental, Behavioral and Neurodevelopmental disorders	509812	Mild oxazepam abuse	ICD10CM	1	F13.10		Sedative, hypnotic or anxiolytic abuse, uncomplicated	IMO	GHS-PACDC	IMO_2015_R	ACTIVE	N	1900-01-01	9999-12-31	2015-07-10
Diagnosis: ICD10 Code, F00-F99 Define	F01-F99 Mental, Behavioral and Neurodevelopmental disorders	484455	Mild gamma-hydroxybutyrate use disorder in sustained remission	ICD10CM	1	F13.10		Sedative, hypnotic or anxiolytic abuse, uncomplicated	IMO	GHS-PACDC	IMO_2015_R	ACTIVE	N	2016-09-17	9999-12-31	2016-09-30
Diagnosis: ICD10 Code, F00-F99 Define	F01-F99 Mental, Behavioral and Neurodevelopmental disorders	508749	Ambien use disorder, mild, in controlled environment, abuse	ICD10CM	1	F13.10		Sedative, hypnotic or anxiolytic abuse, uncomplicated	IMO	GHS-PACDC	IMO_2015_R	ACTIVE	N	1900-01-01	9999-12-31	2015-07-10
Diagnosis: ICD10 Code, F00-F99 Define	F01-F99 Mental, Behavioral and Neurodevelopmental disorders	508926	Sedative, hypnotic or anxiolytic use disorder, mild, in controlled environment, abuse	ICD10CM	1	F13.10		Sedative, hypnotic or anxiolytic abuse, uncomplicated	IMO	GHS-PACDC	IMO_2015_R	ACTIVE	N	1900-01-01	9999-12-31	2015-07-10
Diagnosis: ICD10 Code, F00-F99 Define	F01-F99 Mental, Behavioral and Neurodevelopmental disorders	507910	Mild butalbital-acetaminophen-caffeine abuse in early remission in controlled environment	ICD10CM	1	F13.10		Sedative, hypnotic or anxiolytic abuse, uncomplicated	IMO	GHS-PACDC	IMO_2015_R	ACTIVE	N	1900-01-01	2017-10-14	2015-07-10
Diagnosis: ICD10 Code, F00-F99 Define	F01-F99 Mental, Behavioral and Neurodevelopmental disorders	484167	Mild butalbital-acetaminophen-caffeine use disorder	ICD10CM	1	F13.10		Sedative, hypnotic or anxiolytic abuse, uncomplicated	IMO	GHS-PACDC	IMO_2015_R	ACTIVE	N	1900-01-01	9999-12-31	2015-07-10
Diagnosis: ICD10 Code, F00-F99 Define	F01-F99 Mental, Behavioral and Neurodevelopmental disorders	372912	Benzodiazepine abuse, episodic	ICD10CM	1	F13.10		Sedative, hypnotic or anxiolytic abuse, uncomplicated	IMO	GHS-PACDC	DX_LEGACY	ACTIVE	N	1900-01-01	9999-12-31	2015-07-10
Diagnosis: ICD10 Code, F00-F99 Define	F01-F99 Mental, Behavioral and Neurodevelopmental disorders	507371	Sodium amytal use disorder, mild, in sustained remission, abuse	ICD10CM	1	F13.10		Sedative, hypnotic or anxiolytic abuse, uncomplicated	IMO	GHS-PACDC	IMO_2015_R	ACTIVE	N	1900-01-01	2017-10-14	2015-07-10
Diagnosis: ICD10 Code, F00-F99 Define	F01-F99 Mental, Behavioral and Neurodevelopmental disorders	509258	Mild propofol abuse in sustained remission in controlled environment	ICD10CM	1	F13.10		Sedative, hypnotic or anxiolytic abuse, uncomplicated	IMO	GHS-PACDC	IMO_2015_R	ACTIVE	N	1900-01-01	2016-10-14	2015-07-10
Diagnosis: ICD10 Code, F00-F99 Define	F01-F99 Mental, Behavioral and Neurodevelopmental disorders	484446	Librium use disorder, moderate, in early remission (HCC)	ICD10CM	1	F13.10		Sedative, hypnotic or anxiolytic abuse, uncomplicated	IMO	GHS-PACDC	IMO_2015_R	ACTIVE	N	1900-01-01	2016-10-14	2015-07-10
Diagnosis: ICD10 Code, F00-F99 Define	F01-F99 Mental, Behavioral and Neurodevelopmental disorders	508508	Libritabs use disorder, mild, in sustained remission, abuse	ICD10CM	1	F13.10		Sedative, hypnotic or anxiolytic abuse, uncomplicated	IMO	GHS-PACDC	IMO_2015_R	ACTIVE	N	1900-01-01	2017-10-14	2015-07-10
Diagnosis: ICD10 Code, F00-F99 Define	F01-F99 Mental, Behavioral and Neurodevelopmental disorders	508976	Ativan use disorder, mild, in early remission, abuse	ICD10CM	1	F13.10		Sedative, hypnotic or anxiolytic abuse, uncomplicated	IMO	GHS-PACDC	IMO_2015_R	ACTIVE	N	1900-01-01	2017-10-14	2015-07-10
Diagnosis: ICD10 Code, F00-F99 Define	F01-F99 Mental, Behavioral and Neurodevelopmental disorders	506914	Quaalude use disorder, mild	ICD10CM	1	F13.10		Sedative, hypnotic or anxiolytic abuse, uncomplicated	IMO	GHS-PACDC	IMO_2015_R	ACTIVE	N	1900-01-01	9999-12-31	2015-07-10
Diagnosis: ICD10 Code, F00-F99 Define	F01-F99 Mental, Behavioral and Neurodevelopmental disorders	509301	Mild eszopiclone abuse in early remission	ICD10CM	1	F13.10		Sedative, hypnotic or anxiolytic abuse, uncomplicated	IMO	GHS-PACDC	IMO_2015_R	ACTIVE	N	1900-01-01	2017-10-14	2015-07-10
Diagnosis: ICD10 Code, F00-F99 Define	F01-F99 Mental, Behavioral and Neurodevelopmental disorders	492084	Fioricet use disorder, mild, in controlled environment	ICD10CM	1	F13.10		Sedative, hypnotic or anxiolytic abuse, uncomplicated	IMO	GHS-PACDC	IMO_2015_R	ACTIVE	N	1900-01-01	9999-12-31	2015-07-10
Diagnosis: ICD10 Code, F00-F99 Define	F01-F99 Mental, Behavioral and Neurodevelopmental disorders	507760	Mild clonazepam abuse in early remission	ICD10CM	1	F13.10		Sedative, hypnotic or anxiolytic abuse, uncomplicated	IMO	GHS-PACDC	IMO_2015_R	ACTIVE	N	1900-01-01	2017-10-14	2015-07-10
Diagnosis: ICD10 Code, F00-F99 Define	F01-F99 Mental, Behavioral and Neurodevelopmental disorders	489259	Sedative, hypnotic or anxiolytic use disorder, mild, in controlled environment	ICD10CM	1	F13.10		Sedative, hypnotic or anxiolytic abuse, uncomplicated	IMO	GHS-PACDC	IMO_2015_R	ACTIVE	N	2016-09-17	9999-12-31	2016-09-30
Diagnosis: ICD10 Code, F00-F99 Define	F01-F99 Mental, Behavioral and Neurodevelopmental disorders	491934	Rohypnol use disorder, mild, in controlled environment	ICD10CM	1	F13.10		Sedative, hypnotic or anxiolytic abuse, uncomplicated	IMO	GHS-PACDC	IMO_2015_R	ACTIVE	N	2016-09-17	9999-12-31	2016-09-30
Diagnosis: ICD10 Code, F00-F99 Define	F01-F99 Mental, Behavioral and Neurodevelopmental disorders	508144	Mild sedative hypnotic or anxiolytic abuse in sustained remission	ICD10CM	1	F13.11		Sedative, hypnotic or anxiolytic abuse, in remission	IMO	GHS-PACDC	IMO_2015_R	ACTIVE	N	2017-09-17	9999-12-31	2017-10-30
Diagnosis: ICD10 Code, F00-F99 Define	F01-F99 Mental, Behavioral and Neurodevelopmental disorders	509652	Lunesta use disorder, mild, in early remission, in controlled environment, abuse	ICD10CM	1	F13.11		Sedative, hypnotic or anxiolytic abuse, in remission	IMO	GHS-PACDC	IMO_2015_R	ACTIVE	N	2017-09-17	9999-12-31	2017-10-30
Diagnosis: ICD10 Code, F00-F99 Define	F01-F99 Mental, Behavioral and Neurodevelopmental disorders	507240	Mild secobarbital sodium abuse in early remission	ICD10CM	1	F13.11		Sedative, hypnotic or anxiolytic abuse, in remission	IMO	GHS-PACDC	IMO_2015_R	ACTIVE	N	2017-09-17	9999-12-31	2017-10-30
Diagnosis: ICD10 Code, F00-F99 Define	F01-F99 Mental, Behavioral and Neurodevelopmental disorders	509180	Mild oxazepam abuse in sustained remission	ICD10CM	1	F13.11		Sedative, hypnotic or anxiolytic abuse, in remission	IMO	GHS-PACDC	IMO_2015_R	ACTIVE	N	2017-09-17	9999-12-31	2017-10-30
Diagnosis: ICD10 Code, F00-F99 Define	F01-F99 Mental, Behavioral and Neurodevelopmental disorders	508287	Gammahydroxy butarate (GHB) use disorder, mild, in sustained remission, in controlled environ	ICD10CM	1	F13.11		Sedative, hypnotic or anxiolytic abuse, in remission	IMO	GHS-PACDC	IMO_2015_R	ACTIVE	N	2017-09-17	9999-12-31	2017-10-30
Diagnosis: ICD10 Code, F00-F99 Define	F01-F99 Mental, Behavioral and Neurodevelopmental disorders	507458	Mild lorazepam abuse in early remission	ICD10CM	1	F13.11		Sedative, hypnotic or anxiolytic abuse, in remission	IMO	GHS-PACDC	IMO_2015_R	ACTIVE	N	2017-09-17	9999-12-31	2017-10-30
Diagnosis: ICD10 Code, F00-F99 Define	F01-F99 Mental, Behavioral and Neurodevelopmental disorders	508269	Mild clorazepate dipotassium abuse in early remission	ICD10CM	1	F13.11		Sedative, hypnotic or anxiolytic abuse, in remission	IMO	GHS-PACDC	IMO_2015_R	ACTIVE	N	2017-09-17	9999-12-31	2017-10-30
Diagnosis: ICD10 Code, F00-F99 Define	F01-F99 Mental, Behavioral and Neurodevelopmental disorders	509150	Serax use disorder, mild, in early remission, abuse	ICD10CM	1	F13.11		Sedative, hypnotic or anxiolytic abuse, in remission	IMO	GHS-PACDC	IMO_2015_R	ACTIVE	N	2017-09-17	9999-12-31	2017-10-30
Diagnosis: ICD10 Code, F00-F99 Define	F01-F99 Mental, Behavioral and Neurodevelopmental disorders	509318	Mild gamma-hydroxybutyrate (GHB) abuse in early remission in controlled environment	ICD10CM	1	F13.11		Sedative, hypnotic or anxiolytic abuse, in remission	IMO	GHS-PACDC	IMO_2015_R	ACTIVE	N	2017-09-17	9999-12-31	2017-10-30
Diagnosis: ICD10 Code, F00-F99 Define	F01-F99 Mental, Behavioral and Neurodevelopmental disorders	507041	Serax use disorder, mild, in sustained remission, in controlled environment, abuse	ICD10CM	1	F13.11		Sedative, hypnotic or anxiolytic abuse, in remission	IMO	GHS-PACDC	IMO_2015_R	ACTIVE	N	2017-09-17	9999-12-31	2017-10-30
Diagnosis: ICD10 Code, F00-F99 Define	F01-F99 Mental, Behavioral and Neurodevelopmental disorders	507941	Mild chlordiazepoxide abuse in sustained remission	ICD10CM	1	F13.11		Sedative, hypnotic or anxiolytic abuse, in remission	IMO	GHS-PACDC	IMO_2015_R	ACTIVE	N	2017-09-17	9999-12-31	2017-10-30
Diagnosis: ICD10 Code, F00-F99 Define	F01-F99 Mental, Behavioral and Neurodevelopmental disorders	508538	Mild alprazolam abuse in early remission in controlled environment	ICD10CM	1	F13.11		Sedative, hypnotic or anxiolytic abuse, in remission	IMO	GHS-PACDC	IMO_2015_R	ACTIVE	N	2017-09-17	9999-12-31	2017-10-30
Diagnosis: ICD10 Code, F00-F99 Define	F01-F99 Mental, Behavioral and Neurodevelopmental disorders	509898	Mild secobarbital-amobarbital abuse in sustained remission in controlled environment	ICD10CM	1	F13.11		Sedative, hypnotic or anxiolytic abuse, in remission	IMO	GHS-PACDC	IMO_2015_R	ACTIVE	N	2017-09-17	9999-12-31	2017-10-30
Diagnosis: ICD10 Code, F00-F99 Define	F01-F99 Mental, Behavioral and Neurodevelopmental disorders	509886	Mild amobarbital abuse in sustained remission	ICD10CM	1	F13.11		Sedative, hypnotic or anxiolytic abuse, in remission	IMO	GHS-PACDC	IMO_2015_R	ACTIVE	N	2017-09-17	9999-12-31	2017-10-30
Diagnosis: ICD10 Code, F00-F99 Define	F01-F99 Mental, Behavioral and Neurodevelopmental disorders	508748	Placidyl use disorder, mild, in early remission, in controlled environment, abuse	ICD10CM	1	F13.11		Sedative, hypnotic or anxiolytic abuse, in remission	IMO	GHS-PACDC	IMO_2015_R	ACTIVE	N	2017-09-17	9999-12-31	2017-10-30
Diagnosis: ICD10 Code, F00-F99 Define	F01-F99 Mental, Behavioral and Neurodevelopmental disorders	601874	Sedative, hypnotic or anxiolytic abuse with intoxication delirium	ICD10CM	1	F13.121		Sedative, hypnotic or anxiolytic abuse with intoxication (	IMO	GHS-PACDC	IMO_2015_R	ACTIVE	Y	1900-01-01	9999-12-31	2015-07-10
Diagnosis: ICD10 Code, F00-F99 Define	F01-F99 Mental, Behavioral and Neurodevelopmental disorders	974077	Onset of sedative, hypnotic, or anxiolytic-induced psychotic disorder during intoxication, with h	ICD10CM	1	F13.129	F13.129,F13.151,F13.150	Sedative, hypnotic or anxiolytic abuse with intoxication,	IMO	GHS-PACDC	IMO_2015_R	DELETED	Y	2016-09-17	2017-04-27	2016-09-30
Diagnosis: ICD10 Code, F00-F99 Define	F01-F99 Mental, Behavioral and Neurodevelopmental disorders	1038022	Hypnotic intoxication with complication, with unspecified complication	ICD10CM	1	F13.129		Sedative, hypnotic or anxiolytic abuse with intoxication,	IMO	GHS-PACDC	IMO_2015_R	DELETED	Y	1900-01-01	9999-12-31	2015-07-10
Diagnosis: ICD10 Code, F00-F99 Define	F01-F99 Mental, Behavioral and Neurodevelopmental disorders	954028	Acute lorazepam intoxication, with unspecified complication (HCC)	ICD10CM	1	F13.129		Sedative, hypnotic or anxiolytic abuse with intoxication,	IMO	GHS-PACDC	IMO_2015_R	ACTIVE	N	1900-01-01	2016-10-14	2015-07-10
Diagnosis: ICD10 Code, F00-F99 Define	F01-F99 Mental, Behavioral and Neurodevelopmental disorders	969468	Sedative, hypnotic, or anxiolytic intoxication with moderate or severe use disorder, with unspec	ICD10CM	1	F13.129		Sedative, hypnotic or anxiolytic abuse with intoxication,	IMO	GHS-PACDC	IMO_2015_R	ACTIVE	N	1900-01-01	2016-10-14	2015-07-10
Diagnosis: ICD10 Code, F00-F99 Define	F01-F99 Mental, Behavioral and Neurodevelopmental disorders	975576	Sedative, hypnotic, or anxiolytic-induced psychotic disorder with mild use disorder with onset d	ICD10CM	2	F13.129	F13.151,F13.129	Sedative, hypnotic or anxiolytic abuse with intoxication,	IMO	GHS-PACDC	IMO_2015_R	ACTIVE	N	2016-09-17	9999-12-31	2016-09-30
Diagnosis: ICD10 Code, F00-F99 Define	F01-F99 Mental, Behavioral and Neurodevelopmental disorders	429185	Onset of sedative, hypnotic or anxiolytic-induced psychotic disorder with hallucinations during i	ICD10CM	1	F13.129	F13.129,F13.151,F13.150	Sedative, hypnotic or anxiolytic abuse with intoxication,	IMO	GHS-PACDC	IMO_2015_R	DELETED	Y	2016-09-17	2017-04-27	2016-09-30
Diagnosis: ICD10 Code, F00-F99 Define	F01-F99 Mental, Behavioral and Neurodevelopmental disorders	968608	Onset of sedative, hypnotic or anxiolytic-induced mood disorder during intoxication, uncomplic	ICD10CM	2	F13.14	F13.120,F13.14	Sedative, hypnotic or anxiolytic abuse with sedative, hyp	IMO	GHS-PACDC	IMO_2015_R	ACTIVE	N	1900-01-01	2016-05-07	2015-07-10
Diagnosis: ICD10 Code, F00-F99 Define	F01-F99 Mental, Behavioral and Neurodevelopmental disorders	429126	Sedative, hypnotic, or anxiolytic-induced depressive disorder with mild use disorder (HCC)	ICD10CM	1	F13.14	F13.14,F32.89	Sedative, hypnotic or anxiolytic abuse with sedative, hyp	IMO	GHS-PACDC	IMO_2015_R	ACTIVE	N	2016-09-17	9999-12-31	2016-09-30
Diagnosis: ICD10 Code, F00-F99 Define	F01-F99 Mental, Behavioral and Neurodevelopmental disorders	429185	Onset of sedative, hypnotic or anxiolytic-induced psychotic disorder with hallucinations during i	ICD10CM	2	F13.151	F13.129,F13.151,F13.150	Sedative, hypnotic or anxiolytic abuse with sedative, hyp	IMO	GHS-PACDC	IMO_2015_R	DELETED	Y	2016-09-17	2017-04-27	2016-09-30
Diagnosis: ICD10 Code, F00-F99 Define	F01-F99 Mental, Behavioral and Neurodevelopmental disorders	965996	Sedative, hypnotic, or anxiolytic-induced psychotic disorder with mild use disorder, with unspec	ICD10CM	1	F13.159		Sedative, hypnotic or anxiolytic abuse with sedative, hyp	IMO	GHS-PACDC	IMO_2015_R	ACTIVE	N	1900-01-01	9999-12-31	2015-07-10
Diagnosis: ICD10 Code, F00-F99 Define	F01-F99 Mental, Behavioral and Neurodevelopmental disorders	1368507	Sedative, hypnotic, or anxiolytic-induced psychotic disorder with mild use disorder with complic	ICD10CM	1	F13.159		Sedative, hypnotic or anxiolytic abuse with sedative, hyp	IMO	GHS-PACDC	IMO_2018_R	ACTIVE	N	1900-01-01	9999-12-31	2017-10-30
Diagnosis: ICD10 Code, F00-F99 Define	F01-F99 Mental, Behavioral and Neurodevelopmental disorders	602644	Sedative, hypnotic or anxiolytic abuse with sedative, hypnotic or anxiolytic-induced anxiety diso	ICD10CM	1	F13.180		Sedative, hypnotic or anxiolytic abuse with sedative, hyp	IMO	GHS-PACDC	IMO_2015_R	ACTIVE	Y	1900-01-01	9999-12-31	2015-07-10
Diagnosis: ICD10 Code, F00-F99 Define	F01-F99 Mental, Behavioral and Neurodevelopmental disorders	429187	Onset of sedative, hypnotic or anxiolytic-induced sleep disorder during intoxication (HCC)	ICD10CM	2	F13.182	F13.129,F13.182	Sedative, hypnotic or anxiolytic abuse with sedative, hyp	IMO	GHS-PACDC	IMO_2015_R	ACTIVE	N	1900-01-01	2017-10-14	2015-07-10
Diagnosis: ICD10 Code, F00-F99 Define	F01-F99 Mental, Behavioral and Neurodevelopmental disorders	508551	Valium use disorder, moderate, in controlled environment, dependence (HCC)	ICD10CM	1	F13.20		Sedative, hypnotic or anxiolytic dependence, uncomplica	IMO	GHS-PACDC	IMO_2015_R	ACTIVE	N	1900-01-01	9999-12-31	2015-07-10
Diagnosis: ICD10 Code, F00-F99 Define	F01-F99 Mental, Behavioral and Neurodevelopmental disorders	507762	Ativan use disorder, severe, dependence (HCC)	ICD10CM	1	F13.20		Sedative, hypnotic or anxiolytic dependence, uncomplica	IMO	GHS-PACDC	IMO_2015_R	ACTIVE	N	1900-01-01	9999-12-31	2015-07-10
Diagnosis: ICD10 Code, F00-F99 Define	F01-F99 Mental, Behavioral and Neurodevelopmental disorders	484674	Moderate ethchlorvynol use disorder in controlled environment (HCC)	ICD10CM	1	F13.20		Sedative, hypnotic or anxiolytic dependence, uncomplica	IMO	GHS-PACDC	IMO_2015_R	ACTIVE	N	2016-09-17	9999-12-31	2016-09-30
Diagnosis: ICD10 Code, F00-F99 Define	F01-F99 Mental, Behavioral and Neurodevelopmental disorders	479918	Severe clonazepam use disorder (HCC)	ICD10CM	1	F13.20		Sedative, hypnotic or anxiolytic dependence, uncomplica	IMO	GHS-PACDC	IMO_2015_R	ACTIVE	N	2016-09-17	9999-12-31	2016-09-30
Diagnosis: ICD10 Code, F00-F99 Define	F01-F99 Mental, Behavioral and Neurodevelopmental disorders	491742	Phenobarbital use disorder, moderate (HCC)	ICD10CM	1	F13.20		Sedative, hypnotic or anxiolytic dependence, uncomplica	IMO	GHS-PACDC	IMO_2015_R	ACTIVE	N	2016-09-17	9999-12-31	2016-09-30
Diagnosis: ICD10 Code, F00-F99 Define	F01-F99 Mental, Behavioral and Neurodevelopmental disorders	484187	Nembutyl use disorder, moderate (HCC)	ICD10CM	1	F13.20		Sedative, hypnotic or anxiolytic dependence, uncomplica	IMO	GHS-PACDC	IMO_2015_R	ACTIVE	N	1900-01-01	9999-12-31	2015-07-10
Diagnosis: ICD10 Code, F00-F99 Define	F01-F99 Mental, Behavioral and Neurodevelopmental disorders	479826	Severe oxazepam use disorder (HCC)	ICD10CM	1	F13.20		Sedative, hypnotic or anxiolytic dependence, uncomplica	IMO	GHS-PACDC	IMO_2015_R	ACTIVE	N	2016-09-17	9999-12-31	2016-09-30
Diagnosis: ICD10 Code, F00-F99 Define	F01-F99 Mental, Behavioral and Neurodevelopmental disorders	509349	Librium use disorder, severe, in early remission, in controlled environment, dependence (HCC)	ICD10CM	1	F13.20		Sedative, hypnotic or anxiolytic dependence, uncomplica	IMO	GHS-PACDC	IMO_2015_R	ACTIVE	N	1900-01-01	9999-12-31	2015-07-10
Diagnosis: ICD10 Code, F00-F99 Define	F01-F99 Mental, Behavioral and Neurodevelopmental disorders	484148	Ativan use disorder, moderate (HCC)	ICD10CM	1	F13.20		Sedative, hypnotic or anxiolytic dependence, uncomplica	IMO	GHS-PACDC	IMO_2015_R	ACTIVE	N	2016-09-17	9999-12-31	2016-09-30
Diagnosis: ICD10 Code, F00-F99 Define	F01-F99 Mental, Behavioral and Neurodevelopmental disorders	508136	Lunesta use disorder, moderate, dependence (HCC)	ICD10CM	1	F13.20		Sedative, hypnotic or anxiolytic dependence, uncomplica	IMO	GHS-PACDC	IMO_2015_R	ACTIVE	N	1900-01-01	9999-12-31	2015-07-10
Diagnosis: ICD10 Code, F00-F99 Define	F01-F99 Mental, Behavioral and Neurodevelopmental disorders	508106	Propofol use disorder, moderate, in controlled environment, dependence (HCC)	ICD10CM	1	F13.20		Sedative, hypnotic or anxiolytic dependence, uncomplica	IMO	GHS-PACDC	IMO_2015_R	ACTIVE	N	1900-01-01	2016-10-14	2015-07-10
Diagnosis: ICD10 Code, F00-F99 Define	F01-F99 Mental, Behavioral and Neurodevelopmental disorders	484010	Moderate flurazepam use disorder (HCC)	ICD10CM	1	F13.20		Sedative, hypnotic or anxiolytic dependence, uncomplica	IMO	GHS-PACDC	IMO_2015_R	ACTIVE	N	2016-09-17	9999-12-31	2016-09-30
Diagnosis: ICD10 Code, F00-F99 Define	F01-F99 Mental, Behavioral and Neurodevelopmental disorders	492297	Fioricet use disorder, severe, in controlled environment (HCC)	ICD10CM	1	F13.20		Sedative, hypnotic or anxiolytic dependence, uncomplica	IMO	GHS-PACDC	IMO_2015_R	ACTIVE	N	1900-01-01	9999-12-31	2015-07-10
Diagnosis: ICD10 Code, F00-F99 Define	F01-F99 Mental, Behavioral and Neurodevelopmental disorders	507790	Tuinal use disorder, severe, in early remission, dependence (HCC)	ICD10CM	1	F13.20		Sedative, hypnotic or anxiolytic dependence, uncomplica	IMO	GHS-PACDC	IMO_2015_R	ACTIVE	N	1900-01-01	9999-12-31	2015-07-10
Diagnosis: ICD10 Code, F00-F99 Define	F01-F99 Mental, Behavioral and Neurodevelopmental disorders	492280	Phenobarbital use disorder, severe, in controlled environment (HCC)	ICD10CM	1	F13.20		Sedative, hypnotic or anxiolytic dependence, uncomplica	IMO	GHS-PACDC	IMO_2015_R	ACTIVE	N	2016-09-17	9999-12-31	2016-09-30
Diagnosis: ICD10 Code, F00-F99 Define	F01-F99 Mental, Behavioral and Neurodevelopmental disorders	508544	Severe secobarbital sodium dependence in early remission in controlled environment (HCC)	ICD10CM	1	F13.20		Sedative, hypnotic or anxiolytic dependence, uncomplica	IMO	GHS-PACDC	IMO_2015_R	ACTIVE	N	1900-01-01	9999-12-31	2015-07-10
Diagnosis: ICD10 Code, F00-F99 Define	F01-F99 Mental, Behavioral and Neurodevelopmental disorders	484430	Severe alprazolam use disorder in controlled environment (HCC)	ICD10CM	1	F13.20		Sedative, hypnotic or anxiolytic dependence, uncomplica	IMO	GHS-PACDC	IMO_2015_R	ACTIVE	N	2016-09-17	9999-12-31	2016-09-30
Diagnosis: ICD10 Code, F00-F99 Define	F01-F99 Mental, Behavioral and Neurodevelopmental disorders	484693	Gamma-hydroxybutyrate (GHB) use disorder, moderate, in controlled environment (HCC)	ICD10CM	1	F13.20		Sedative, hypnotic or anxiolytic dependence, uncomplica	IMO	GHS-PACDC	IMO_2015_R	ACTIVE	N	2016-09-17	9999-12-31	2016-09-30
Diagnosis: ICD10 Code, F00-F99 Define	F01-F99 Mental, Behavioral and Neurodevelopmental disorders	507788	Serax use disorder, severe, in early remission, in controlled environment, dependence (HCC)	ICD10CM	1	F13.20		Sedative, hypnotic or anxiolytic dependence, uncomplica	IMO	GHS-PACDC	IMO_2015_R	ACTIVE	N	1900-01-01	9999-12-31	2015-07-10
Diagnosis: ICD10 Code, F00-F99 Define	F01-F99 Mental, Behavioral and Neurodevelopmental disorders	508720	Moderate methaqualone dependence in controlled environment (HCC)	ICD10CM	1	F13.20		Sedative, hypnotic or anxiolytic dependence, uncomplica	IMO	GHS-PACDC	IMO_2015_R	ACTIVE	N	1900-01-01	9999-12-31	2015-07-10
Diagnosis: ICD10 Code, F00-F99 Define	F01-F99 Mental, Behavioral and Neurodevelopmental disorders	508379	Libritabs use disorder, severe, in early remission, in controlled environment, dependence (HCC)	ICD10CM	1	F13.20		Sedative, hypnotic or anxiolytic dependence, uncomplica	IMO	GHS-PACDC	IMO_2015_R	ACTIVE	N	1900-01-01	9999-12-31	2015-07-10
Diagnosis: ICD10 Code, F00-F99 Define	F01-F99 Mental, Behavioral and Neurodevelopmental disorders	508641	Moderate amobarbital dependence (HCC)	ICD10CM	1	F13.20		Sedative, hypnotic or anxiolytic dependence, uncomplica	IMO	GHS-PACDC	IMO_2015_R	ACTIVE	N	1900-01-01	9999-12-31	2015-07-10
Diagnosis: ICD10 Code, F00-F99 Define	F01-F99 Mental, Behavioral and Neurodevelopmental disorders	334005	Barbiturate and similarly acting sedative or hypnotic dependence, continuous use	ICD10CM	1	F13.20		Sedative, hypnotic or anxiolytic dependence, uncomplica	IMO	GHS-PACDC	DX_LEGACY	DELETED	Y	1900-01-01	9999-12-31	2015-07-10
Diagnosis: ICD10 Code, F00-F99 Define	F01-F99 Mental, Behavioral and Neurodevelopmental disorders	509455	Moderate propofol dependence (HCC)	ICD10CM	1	F13.20		Sedative, hypnotic or anxiolytic dependence, uncomplica	IMO	GHS-PACDC	IMO_2015_R	ACTIVE	N	1900-01-01	2016-10-14	2015-07-10
Diagnosis: ICD10 Code, F00-F99 Define	F01-F99 Mental, Behavioral and Neurodevelopmental disorders	508517	Severe propofol dependence in controlled environment (HCC)	ICD10CM	1	F13.20		Sedative, hypnotic or anxiolytic dependence, uncomplica	IMO	GHS-PACDC	IMO_2015_R	ACTIVE	N	1900-01-01	2016-10-14	2015-07-10
Diagnosis: ICD10 Code, F00-F99 Define	F01-F99 Mental, Behavioral and Neurodevelopmental disorders	508008	Moderate diazepam dependence in controlled environment (HCC)	ICD10CM	1	F13.20		Sedative, hypnotic or anxiolytic dependence, uncomplica	IMO	GHS-PACDC	IMO_2015_R	ACTIVE	N	1900-01-01	9999-12-31	2015-07-10
Diagnosis: ICD10 Code, F00-F99 Define	F01-F99 Mental, Behavioral and Neurodevelopmental disorders	509890	Moderate eszopiclone dependence (HCC)	ICD10CM	1	F13.20		Sedative, hypnotic or anxiolytic dependence, uncomplica	IMO	GHS-PACDC	IMO_2015_R	ACTIVE	N	1900-01-01	9999-12-31	2015-07-10
Diagnosis: ICD10 Code, F00-F99 Define	F01-F99 Mental, Behavioral and Neurodevelopmental disorders	508231	Severe eszopiclone dependence in early remission in controlled environment (HCC)	ICD10CM	1	F13.20		Sedative, hypnotic or anxiolytic dependence, uncomplica	IMO	GHS-PACDC	IMO_2015_R	ACTIVE	N	1900-01-01	9999-12-31	2015-07-10
Diagnosis: ICD10 Code, F00-F99 Define	F01-F99 Mental, Behavioral and Neurodevelopmental disorders	485002	Severe secobarbital sodium use disorder in controlled environment (HCC)	ICD10CM	1	F13.20		Sedative, hypnotic or anxiolytic dependence, uncomplica	IMO	GHS-PACDC	IMO_2015_R	ACTIVE	N	2016-09-17	9999-12-31	2016-09-30
Diagnosis: ICD10 Code, F00-F99 Define	F01-F99 Mental, Behavioral and Neurodevelopmental disorders	484013	Placidyl use disorder, moderate (HCC)	ICD10CM	1	F13.20		Sedative, hypnotic or anxiolytic dependence, uncomplica	IMO	GHS-PACDC	IMO_2015_R	ACTIVE	N	2016-09-17	9999-12-31	2016-09-30
Diagnosis: ICD10 Code, F00-F99 Define	F01-F99 Mental, Behavioral and Neurodevelopmental disorders	479839	Fiorinal use disorder, moderate (HCC)	ICD10CM	1	F13.20		Sedative, hypnotic or anxiolytic dependence, uncomplica	IMO	GHS-PACDC	IMO_2015_R	ACTIVE	N	2016-09-17	9999-12-31	2016-09-30
Diagnosis: ICD10 Code, F00-F99 Define	F01-F99 Mental, Behavioral and Neurodevelopmental disorders	484112	Valium use disorder, severe (HCC)	ICD10CM	1	F13.20		Sedative, hypnotic or anxiolytic dependence, uncomplica	IMO	GHS-PACDC	IMO_2015_R	ACTIVE	N	2016-09-17	9999-12-31	2016-09-30
Diagnosis: ICD10 Code, F00-F99 Define	F01-F99 Mental, Behavioral and Neurodevelopmental disorders	510003	Severe pentobarbital dependence (HCC)	ICD10CM	1	F13.20		Sedative, hypnotic or anxiolytic dependence, uncomplica	IMO	GHS-PACDC	IMO_2015_R	ACTIVE	N	1900-01-01	9999-12-31	2015-07-10

Diagnosis: ICD10 Code, F00-F99 Define	F01-F99 Mental, Behavioral and Neurodevelopmental disorders	508573	Moderate flurazepam dependence (HCC)	ICD10CM	1	F13.20		Sedative, hypnotic or anxiolytic dependence, uncomplica	IMO	GHS-PACDC	IMO_2015_R	ACTIVE	N	1900-01-01	9999-12-31	2015-07-10
Diagnosis: ICD10 Code, F00-F99 Define	F01-F99 Mental, Behavioral and Neurodevelopmental disorders	509467	Severe cyclobenzaprine dependence (HCC)	ICD10CM	1	F13.20		Sedative, hypnotic or anxiolytic dependence, uncomplica	IMO	GHS-PACDC	IMO_2015_R	ACTIVE	N	1900-01-01	2016-10-14	2015-07-10
Diagnosis: ICD10 Code, F00-F99 Define	F01-F99 Mental, Behavioral and Neurodevelopmental disorders	337377	Barbiturate and similarly acting sedative or hypnotic dependence, abuse (HCC)	ICD10CM	1	F13.20		Sedative, hypnotic or anxiolytic dependence, uncomplica	IMO	GHS-PACDC	DX_LEGACY	ACTIVE	N	1900-01-01	9999-12-31	2015-07-10
Diagnosis: ICD10 Code, F00-F99 Define	F01-F99 Mental, Behavioral and Neurodevelopmental disorders	509585	Klonopin use disorder, severe, in early remission, in controlled environment, dependence (HCC)	ICD10CM	1	F13.20		Sedative, hypnotic or anxiolytic dependence, uncomplica	IMO	GHS-PACDC	IMO_2015_R	ACTIVE	N	1900-01-01	9999-12-31	2015-07-10
Diagnosis: ICD10 Code, F00-F99 Define	F01-F99 Mental, Behavioral and Neurodevelopmental disorders	335824	Barbiturate and similarly acting sedative or hypnotic dependence, episodic abuse	ICD10CM	1	F13.20		Sedative, hypnotic or anxiolytic dependence, uncomplica	IMO	GHS-PACDC	DX_LEGACY	DELETED	Y	1900-01-01	9999-12-31	2015-07-10
Diagnosis: ICD10 Code, F00-F99 Define	F01-F99 Mental, Behavioral and Neurodevelopmental disorders	491851	Severe Fiorinal use disorder, in controlled environment (HCC)	ICD10CM	1	F13.20		Sedative, hypnotic or anxiolytic dependence, uncomplica	IMO	GHS-PACDC	IMO_2015_R	ACTIVE	N	2016-09-17	9999-12-31	2016-09-30
Diagnosis: ICD10 Code, F00-F99 Define	F01-F99 Mental, Behavioral and Neurodevelopmental disorders	507531	Gamma-hydroxybutyrate (GHB) use disorder, severe, in controlled environment, dependence (H	ICD10CM	1	F13.20		Sedative, hypnotic or anxiolytic dependence, uncomplica	IMO	GHS-PACDC	IMO_2015_R	ACTIVE	N	1900-01-01	9999-12-31	2015-07-10
Diagnosis: ICD10 Code, F00-F99 Define	F01-F99 Mental, Behavioral and Neurodevelopmental disorders	508280	Tranxene use disorder, severe, in early remission, in controlled environment, dependence (HCC	ICD10CM	1	F13.20		Sedative, hypnotic or anxiolytic dependence, uncomplica	IMO	GHS-PACDC	IMO_2015_R	ACTIVE	N	1900-01-01	9999-12-31	2015-07-10
Diagnosis: ICD10 Code, F00-F99 Define	F01-F99 Mental, Behavioral and Neurodevelopmental disorders	509551	Dalmane use disorder, severe, in controlled environment, dependence (HCC)	ICD10CM	1	F13.20		Sedative, hypnotic or anxiolytic dependence, uncomplica	IMO	GHS-PACDC	IMO_2015_R	ACTIVE	N	1900-01-01	9999-12-31	2015-07-10
Diagnosis: ICD10 Code, F00-F99 Define	F01-F99 Mental, Behavioral and Neurodevelopmental disorders	90522	Veronal dependence (HCC)	ICD10CM	1	F13.20		Sedative, hypnotic or anxiolytic dependence, uncomplica	IMO	GHS-PACDC	DX_LEGACY	ACTIVE	N	1900-01-01	9999-12-31	2015-07-10
Diagnosis: ICD10 Code, F00-F99 Define	F01-F99 Mental, Behavioral and Neurodevelopmental disorders	479825	Severe phenobarbital use disorder (HCC)	ICD10CM	1	F13.20		Sedative, hypnotic or anxiolytic dependence, uncomplica	IMO	GHS-PACDC	IMO_2015_R	ACTIVE	N	2016-09-17	9999-12-31	2016-09-30
Diagnosis: ICD10 Code, F00-F99 Define	F01-F99 Mental, Behavioral and Neurodevelopmental disorders	491746	Barbiturate use disorder, severe (HCC)	ICD10CM	1	F13.20		Sedative, hypnotic or anxiolytic dependence, uncomplica	IMO	GHS-PACDC	IMO_2015_R	ACTIVE	N	2016-09-17	9999-12-31	2016-09-30
Diagnosis: ICD10 Code, F00-F99 Define	F01-F99 Mental, Behavioral and Neurodevelopmental disorders	507389	Moderate secobarbital sodium dependence in sustained remission (HCC)	ICD10CM	1	F13.21		Sedative, hypnotic or anxiolytic dependence, in remissio	IMO	GHS-PACDC	IMO_2015_R	ACTIVE	N	1900-01-01	9999-12-31	2015-07-10
Diagnosis: ICD10 Code, F00-F99 Define	F01-F99 Mental, Behavioral and Neurodevelopmental disorders	508065	Sodium amytal use disorder, moderate, in sustained remission, dependence (HCC)	ICD10CM	1	F13.21		Sedative, hypnotic or anxiolytic dependence, in remissio	IMO	GHS-PACDC	IMO_2015_R	ACTIVE	N	1900-01-01	9999-12-31	2015-07-10
Diagnosis: ICD10 Code, F00-F99 Define	F01-F99 Mental, Behavioral and Neurodevelopmental disorders	508526	Severe gamma-hydroxybutyrate (GHB) dependence in sustained remission in controlled enviror	ICD10CM	1	F13.21		Sedative, hypnotic or anxiolytic dependence, in remissio	IMO	GHS-PACDC	IMO_2015_R	ACTIVE	N	1900-01-01	9999-12-31	2015-07-10
Diagnosis: ICD10 Code, F00-F99 Define	F01-F99 Mental, Behavioral and Neurodevelopmental disorders	508560	Dalmane use disorder, severe, in early remission, dependence (HCC)	ICD10CM	1	F13.21		Sedative, hypnotic or anxiolytic dependence, in remissio	IMO	GHS-PACDC	IMO_2015_R	ACTIVE	N	1900-01-01	9999-12-31	2015-07-10
Diagnosis: ICD10 Code, F00-F99 Define	F01-F99 Mental, Behavioral and Neurodevelopmental disorders	508738	Severe gamma-hydroxybutyrate dependence in early remission (HCC)	ICD10CM	1	F13.21		Sedative, hypnotic or anxiolytic dependence, in remissio	IMO	GHS-PACDC	IMO_2015_R	ACTIVE	N	1900-01-01	9999-12-31	2015-07-10
Diagnosis: ICD10 Code, F00-F99 Define	F01-F99 Mental, Behavioral and Neurodevelopmental disorders	507260	Serax use disorder, severe, in sustained remission, in controlled environment, dependence (HC(	ICD10CM	1	F13.21		Sedative, hypnotic or anxiolytic dependence, in remissio	IMO	GHS-PACDC	IMO_2015_R	ACTIVE	N	1900-01-01	9999-12-31	2015-07-10
Diagnosis: ICD10 Code, F00-F99 Define	F01-F99 Mental, Behavioral and Neurodevelopmental disorders	509563	Ambien use disorder, severe, in early remission, dependence (HCC)	ICD10CM	1	F13.21		Sedative, hypnotic or anxiolytic dependence, in remissio	IMO	GHS-PACDC	IMO_2015_R	ACTIVE	N	1900-01-01	9999-12-31	2015-07-10
Diagnosis: ICD10 Code, F00-F99 Define	F01-F99 Mental, Behavioral and Neurodevelopmental disorders	509218	Gamma-hydroxybutyrate (GHB) use disorder, severe, in sustained remission, dependence (HCC)	ICD10CM	1	F13.21		Sedative, hypnotic or anxiolytic dependence, in remissio	IMO	GHS-PACDC	IMO_2015_R	ACTIVE	N	1900-01-01	9999-12-31	2015-07-10
Diagnosis: ICD10 Code, F00-F99 Define	F01-F99 Mental, Behavioral and Neurodevelopmental disorders	484885	Severe flunitrazepam use disorder in sustained remission (HCC)	ICD10CM	1	F13.21		Sedative, hypnotic or anxiolytic dependence, in remissio	IMO	GHS-PACDC	IMO_2015_R	ACTIVE	N	2016-09-17	9999-12-31	2016-09-30
Diagnosis: ICD10 Code, F00-F99 Define	F01-F99 Mental, Behavioral and Neurodevelopmental disorders	509470	Lunesta use disorder, severe, in sustained remission, dependence (HCC)	ICD10CM	1	F13.21		Sedative, hypnotic or anxiolytic dependence, in remissio	IMO	GHS-PACDC	IMO_2015_R	ACTIVE	N	1900-01-01	9999-12-31	2015-07-10
Diagnosis: ICD10 Code, F00-F99 Define	F01-F99 Mental, Behavioral and Neurodevelopmental disorders	522240	Equanil use disorder, moderate, in early remission, dependence (HCC)	ICD10CM	1	F13.21		Sedative, hypnotic or anxiolytic dependence, in remissio	IMO	GHS-PACDC	IMO_2015_R	ACTIVE	N	1900-01-01	9999-12-31	2015-07-10
Diagnosis: ICD10 Code, F00-F99 Define	F01-F99 Mental, Behavioral and Neurodevelopmental disorders	492201	Phenobarbital use disorder, severe, in sustained remission (HCC)	ICD10CM	1	F13.21		Sedative, hypnotic or anxiolytic dependence, in remissio	IMO	GHS-PACDC	IMO_2015_R	ACTIVE	N	2016-09-17	9999-12-31	2016-09-30
Diagnosis: ICD10 Code, F00-F99 Define	F01-F99 Mental, Behavioral and Neurodevelopmental disorders	492316	Fiorinal use disorder, moderate, in early remission (HCC)	ICD10CM	1	F13.21		Sedative, hypnotic or anxiolytic dependence, in remissio	IMO	GHS-PACDC	IMO_2015_R	ACTIVE	N	2016-09-17	9999-12-31	2016-09-30
Diagnosis: ICD10 Code, F00-F99 Define	F01-F99 Mental, Behavioral and Neurodevelopmental disorders	491966	Seconal use disorder, moderate, in sustained remission (HCC)	ICD10CM	1	F13.21		Sedative, hypnotic or anxiolytic dependence, in remissio	IMO	GHS-PACDC	IMO_2015_R	ACTIVE	N	2016-09-17	9999-12-31	2016-09-30
Diagnosis: ICD10 Code, F00-F99 Define	F01-F99 Mental, Behavioral and Neurodevelopmental disorders	508347	Librium use disorder, moderate, in early remission, dependence (HCC)	ICD10CM	1	F13.21		Sedative, hypnotic or anxiolytic dependence, in remissio	IMO	GHS-PACDC	IMO_2015_R	ACTIVE	N	1900-01-01	9999-12-31	2015-07-10
Diagnosis: ICD10 Code, F00-F99 Define	F01-F99 Mental, Behavioral and Neurodevelopmental disorders	508856	Ativan use disorder, severe, in sustained remission, dependence (HCC)	ICD10CM	1	F13.21		Sedative, hypnotic or anxiolytic dependence, in remissio	IMO	GHS-PACDC	IMO_2015_R	ACTIVE	N	1900-01-01	9999-12-31	2015-07-10
Diagnosis: ICD10 Code, F00-F99 Define	F01-F99 Mental, Behavioral and Neurodevelopmental disorders	491832	Libritabs use disorder, severe, in sustained remission (HCC)	ICD10CM	1	F13.21		Sedative, hypnotic or anxiolytic dependence, in remissio	IMO	GHS-PACDC	IMO_2015_R	ACTIVE	N	2016-09-17	9999-12-31	2016-09-30
Diagnosis: ICD10 Code, F00-F99 Define	F01-F99 Mental, Behavioral and Neurodevelopmental disorders	507746	Restoril use disorder, moderate, in early remission, dependence (HCC)	ICD10CM	1	F13.21		Sedative, hypnotic or anxiolytic dependence, in remissio	IMO	GHS-PACDC	IMO_2015_R	ACTIVE	N	1900-01-01	9999-12-31	2015-07-10
Diagnosis: ICD10 Code, F00-F99 Define	F01-F99 Mental, Behavioral and Neurodevelopmental disorders	507974	Severe methaqualone dependence in sustained remission in controlled environment (HCC)	ICD10CM	1	F13.21		Sedative, hypnotic or anxiolytic dependence, in remissio	IMO	GHS-PACDC	IMO_2015_R	ACTIVE	N	1900-01-01	9999-12-31	2015-07-10
Diagnosis: ICD10 Code, F00-F99 Define	F01-F99 Mental, Behavioral and Neurodevelopmental disorders	484965	Klonopin use disorder, severe, in sustained remission (HCC)	ICD10CM	1	F13.21		Sedative, hypnotic or anxiolytic dependence, in remissio	IMO	GHS-PACDC	IMO_2015_R	ACTIVE	N	2016-09-17	9999-12-31	2016-09-30
Diagnosis: ICD10 Code, F00-F99 Define	F01-F99 Mental, Behavioral and Neurodevelopmental disorders	509951	Sedative, hypnotic or anxiolytic use disorder, severe, in sustained remission, dependence (HCC)	ICD10CM	1	F13.21		Sedative, hypnotic or anxiolytic dependence, in remissio	IMO	GHS-PACDC	IMO_2015_R	ACTIVE	N	1900-01-01	9999-12-31	2015-07-10
Diagnosis: ICD10 Code, F00-F99 Define	F01-F99 Mental, Behavioral and Neurodevelopmental disorders	508448	Flexeril use disorder, moderate, in early remission, dependence (HCC)	ICD10CM	1	F13.21		Sedative, hypnotic or anxiolytic dependence, in remissio	IMO	GHS-PACDC	IMO_2015_R	ACTIVE	N	1900-01-01	2016-10-14	2015-07-10
Diagnosis: ICD10 Code, F00-F99 Define	F01-F99 Mental, Behavioral and Neurodevelopmental disorders	508734	Lunesta use disorder, severe, in sustained remission, in controlled environment, dependence (H	ICD10CM	1	F13.21		Sedative, hypnotic or anxiolytic dependence, in remissio	IMO	GHS-PACDC	IMO_2015_R	ACTIVE	N	1900-01-01	9999-12-31	2015-07-10
Diagnosis: ICD10 Code, F00-F99 Define	F01-F99 Mental, Behavioral and Neurodevelopmental disorders	509508	Halcion use disorder, moderate, in sustained remission, in controlled environment, dependence	ICD10CM	1	F13.21		Sedative, hypnotic or anxiolytic dependence, in remissio	IMO	GHS-PACDC	IMO_2015_R	ACTIVE	N	1900-01-01	9999-12-31	2015-07-10
Diagnosis: ICD10 Code, F00-F99 Define	F01-F99 Mental, Behavioral and Neurodevelopmental disorders	508457	Tranxene use disorder, moderate, in sustained remission, in controlled environment, dependen	ICD10CM	1	F13.21		Sedative, hypnotic or anxiolytic dependence, in remissio	IMO	GHS-PACDC	IMO_2015_R	ACTIVE	N	1900-01-01	9999-12-31	2015-07-10
Diagnosis: ICD10 Code, F00-F99 Define	F01-F99 Mental, Behavioral and Neurodevelopmental disorders	492024	Valium use disorder, severe, in sustained remission (HCC)	ICD10CM	1	F13.21		Sedative, hypnotic or anxiolytic dependence, in remissio	IMO	GHS-PACDC	IMO_2015_R	ACTIVE	N	2016-09-17	9999-12-31	2016-09-30
Diagnosis: ICD10 Code, F00-F99 Define	F01-F99 Mental, Behavioral and Neurodevelopmental disorders	509666	Severe propofol dependence in sustained remission in controlled environment (HCC)	ICD10CM	1	F13.21		Sedative, hypnotic or anxiolytic dependence, in remissio	IMO	GHS-PACDC	IMO_2015_R	ACTIVE	N	1900-01-01	2016-10-14	2015-07-10
Diagnosis: ICD10 Code, F00-F99 Define	F01-F99 Mental, Behavioral and Neurodevelopmental disorders	488521	Sedative, hypnotic or anxiolytic use disorder, severe, in early remission (HCC)	ICD10CM	1	F13.21		Sedative, hypnotic or anxiolytic dependence, in remissio	IMO	GHS-PACDC	IMO_2015_R	ACTIVE	N	2016-09-17	9999-12-31	2016-09-30
Diagnosis: ICD10 Code, F00-F99 Define	F01-F99 Mental, Behavioral and Neurodevelopmental disorders	507161	Libritabs use disorder, severe, in sustained remission, in controlled environment, dependence (I	ICD10CM	1	F13.21		Sedative, hypnotic or anxiolytic dependence, in remissio	IMO	GHS-PACDC	IMO_2015_R	ACTIVE	N	1900-01-01	9999-12-31	2015-07-10
Diagnosis: ICD10 Code, F00-F99 Define	F01-F99 Mental, Behavioral and Neurodevelopmental disorders	509927	Valium use disorder, severe, in early remission, dependence (HCC)	ICD10CM	1	F13.21		Sedative, hypnotic or anxiolytic dependence, in remissio	IMO	GHS-PACDC	IMO_2015_R	ACTIVE	N	1900-01-01	9999-12-31	2015-07-10
Diagnosis: ICD10 Code, F00-F99 Define	F01-F99 Mental, Behavioral and Neurodevelopmental disorders	486697	Acute sedative, hypnotic, or anxiolytic intoxication delirium with moderate or severe use disord	ICD10CM	1	F13.221		Sedative, hypnotic or anxiolytic dependence with intoxic	IMO	GHS-PACDC	IMO_2015_R	ACTIVE	N	2016-09-17	9999-12-31	2016-09-30
Diagnosis: ICD10 Code, F00-F99 Define	F01-F99 Mental, Behavioral and Neurodevelopmental disorders	487120	Sedative, hypnotic, or anxiolytic-induced sexual dysfunction with moderate or severe use disorc	ICD10CM	2	F13.229	F13.281,F13.229	Sedative, hypnotic or anxiolytic dependence with intoxic	IMO	GHS-PACDC	IMO_2015_R	DELETED	Y	2016-09-17	9999-12-31	2016-09-30
Diagnosis: ICD10 Code, F00-F99 Define	F01-F99 Mental, Behavioral and Neurodevelopmental disorders	968852	Onset of sedative, hypnotic or anxiolytic-induced sleep disorder during withdrawal, uncomplica	ICD10CM	1	F13.230		Sedative, hypnotic or anxiolytic dependence with withdr	IMO	GHS-PACDC	IMO_2015_R	ACTIVE	N	2016-04-10	9999-12-31	2015-07-10
Diagnosis: ICD10 Code, F00-F99 Define	F01-F99 Mental, Behavioral and Neurodevelopmental disorders	1368490	Sedative, hypnotic, or anxiolytic withdrawal without perceptual disturbances without complicat	ICD10CM	1	F13.230		Sedative, hypnotic or anxiolytic dependence with withdr	IMO	GHS-PACDC	IMO_2018_R	ACTIVE	N	1900-01-01	9999-12-31	2017-10-30
Diagnosis: ICD10 Code, F00-F99 Define	F01-F99 Mental, Behavioral and Neurodevelopmental disorders	961238	Inhalant withdrawal, uncomplicated	ICD10CM	1	F13.230		Sedative, hypnotic or anxiolytic dependence with withdr	IMO	GHS-PACDC	IMO_2015_R	DELETED	Y	1900-01-01	9999-12-31	2015-07-10
Diagnosis: ICD10 Code, F00-F99 Define	F01-F99 Mental, Behavioral and Neurodevelopmental disorders	969028	Onset of sedative, hypnotic or anxiolytic-induced anxiety disorder during withdrawal, uncompli(	ICD10CM	1	F13.230	F13.230,F19.239	Sedative, hypnotic or anxiolytic dependence with withdr	IMO	GHS-PACDC	IMO_2015_R	DELETED	Y	2016-04-10	2016-10-14	2015-07-10
Diagnosis: ICD10 Code, F00-F99 Define	F01-F99 Mental, Behavioral and Neurodevelopmental disorders	473550	Inhalant withdrawal with delirium (HCC)	ICD10CM	1	F13.231		Sedative, hypnotic or anxiolytic dependence with withdr	IMO	GHS-PACDC	IMO_2015_R	ACTIVE	N	1900-01-01	9999-12-31	2015-07-10
Diagnosis: ICD10 Code, F00-F99 Define	F01-F99 Mental, Behavioral and Neurodevelopmental disorders	429180	Onset of sedative, hypnotic or anxiolytic-induced psychotic disorder with delusions during with(	ICD10CM	2	F13.239	F13.250,F13.239	Sedative, hypnotic or anxiolytic dependence with withdr	IMO	GHS-PACDC	IMO_2015_R	ACTIVE	N	2017-09-17	9999-12-31	2017-10-30
Diagnosis: ICD10 Code, F00-F99 Define	F01-F99 Mental, Behavioral and Neurodevelopmental disorders	523834	Withdrawal from sedative drug (HCC)	ICD10CM	1	F13.239		Sedative, hypnotic or anxiolytic dependence with withdr	IMO	GHS-PACDC	IMO_2015_R	ACTIVE	N	1900-01-01	9999-12-31	2015-07-10
Diagnosis: ICD10 Code, F00-F99 Define	F01-F99 Mental, Behavioral and Neurodevelopmental disorders	1368467	Sedative, hypnotic, or anxiolytic-induced psychotic disorder with moderate or severe use disord	ICD10CM	1	F13.250	F13.250,F13.229	Sedative, hypnotic or anxiolytic dependence with sedati	IMO	GHS-PACDC	IMO_2018_R	ACTIVE	N	1900-01-01	9999-12-31	2017-10-30
Diagnosis: ICD10 Code, F00-F99 Define	F01-F99 Mental, Behavioral and Neurodevelopmental disorders	1368460	Sedative, hypnotic, or anxiolytic-induced psychotic disorder with moderate or severe use disord	ICD10CM	1	F13.250		Sedative, hypnotic or anxiolytic dependence with sedati	IMO	GHS-PACDC	IMO_2018_R	ACTIVE	N	1900-01-01	9999-12-31	2017-10-30
Diagnosis: ICD10 Code, F00-F99 Define	F01-F99 Mental, Behavioral and Neurodevelopmental disorders	1368492	Sedative, hypnotic, or anxiolytic-induced psychotic disorder with moderate or severe use disord	ICD10CM	2	F13.250	F13.232,F13.250	Sedative, hypnotic or anxiolytic dependence with sedati	IMO	GHS-PACDC	IMO_2018_R	ACTIVE	N	1900-01-01	9999-12-31	2017-10-30
Diagnosis: ICD10 Code, F00-F99 Define	F01-F99 Mental, Behavioral and Neurodevelopmental disorders	1368477	Sedative, hypnotic, or anxiolytic-induced psychotic disorder with moderate or severe use disord	ICD10CM	1	F13.251	F13.251,F13.232	Sedative, hypnotic or anxiolytic dependence with sedati	IMO	GHS-PACDC	IMO_2018_R	ACTIVE	N	1900-01-01	9999-12-31	2017-10-30
Diagnosis: ICD10 Code, F00-F99 Define	F01-F99 Mental, Behavioral and Neurodevelopmental disorders	1366302	Sedatv/hyp/anxiolytc depend w psychotic disorder w hallucin (HCC)	ICD10CM	1	F13.251		Sedative, hypnotic or anxiolytic dependence with sedati	IMO	GHS-PACDC	IMO_2018_R	ACTIVE	N	1900-01-01	9999-12-31	2017-10-30
Diagnosis: ICD10 Code, F00-F99 Define	F01-F99 Mental, Behavioral and Neurodevelopmental disorders	486910	Sedative, hypnotic, or anxiolytic-induced psychotic disorder with mild use disorder with onset d	ICD10CM	1	F13.259	F13.259,F13.239	Sedative, hypnotic or anxiolytic dependence with sedati	IMO	GHS-PACDC	IMO_2015_R	ACTIVE	N	2016-09-17	9999-12-31	2016-09-30
Diagnosis: ICD10 Code, F00-F99 Define	F01-F99 Mental, Behavioral and Neurodevelopmental disorders	595269	Sedative, hypnotic or anxiolytic dependence with sedative, hypnotic or anxiolytic-induced persi:	ICD10CM	1	F13.26		Sedative, hypnotic or anxiolytic dependence with sedati	IMO	GHS-PACDC	IMO_2015_R	ACTIVE	Y	1900-01-01	9999-12-31	2015-07-10
Diagnosis: ICD10 Code, F00-F99 Define	F01-F99 Mental, Behavioral and Neurodevelopmental disorders	486915	Moderate sedative, hypnotic, or anxiolytic-induced major neurocognitive disorder with modera	ICD10CM	1	F13.27		Sedative, hypnotic or anxiolytic dependence with sedati	IMO	GHS-PACDC	IMO_2015_R	ACTIVE	N	2016-09-17	9999-12-31	2016-09-30
Diagnosis: ICD10 Code, F00-F99 Define	F01-F99 Mental, Behavioral and Neurodevelopmental disorders	486970	Sedative, hypnotic, or anxiolytic-induced anxiety disorder without use disorder with onset durir	ICD10CM	1	F13.280	F13.280,F13.239	Sedative, hypnotic or anxiolytic dependence with sedati	IMO	GHS-PACDC	IMO_2015_R	ACTIVE	N	2016-09-17	9999-12-31	2016-09-30
Diagnosis: ICD10 Code, F00-F99 Define	F01-F99 Mental, Behavioral and Neurodevelopmental disorders	429093	Sedative, hypnotic, or anxiolytic-induced anxiety disorder with moderate or severe use disorder	ICD10CM	1	F13.280		Sedative, hypnotic or anxiolytic dependence with sedati	IMO	GHS-PACDC	IMO_2015_R	ACTIVE	N	1900-01-01	9999-12-31	2015-07-10
Diagnosis: ICD10 Code, F00-F99 Define	F01-F99 Mental, Behavioral and Neurodevelopmental disorders	488846	Sedative, hypnotic, or anxiolytic-induced sleep disorder, insomnia type, with onset during disco	ICD10CM	2	F13.282	F13.239,F13.282	Sedative, hypnotic or anxiolytic dependence with sedati	IMO	GHS-PACDC	IMO_2015_R	DELETED	Y	1900-01-01	2016-05-07	2015-07-10
Diagnosis: ICD10 Code, F00-F99 Define	F01-F99 Mental, Behavioral and Neurodevelopmental disorders	429100	Sedative, hypnotic, or anxiolytic-induced sleep disorder with moderate or severe use disorder (I	ICD10CM	1	F13.282		Sedative, hypnotic or anxiolytic dependence with sedati	IMO	GHS-PACDC	IMO_2015_R	ACTIVE	N	1900-01-01	9999-12-31	2015-07-10
Diagnosis: ICD10 Code, F00-F99 Define	F01-F99 Mental, Behavioral and Neurodevelopmental disorders	488844	Sedative, hypnotic, or anxiolytic-induced sleep disorder, daytime sleepiness type, with onset du	ICD10CM	2	F13.282	F13.239,F13.282	Sedative, hypnotic or anxiolytic dependence with sedati	IMO	GHS-PACDC	IMO_2015_R	DELETED	Y	1900-01-01	2016-05-07	2015-07-10
Diagnosis: ICD10 Code, F00-F99 Define	F01-F99 Mental, Behavioral and Neurodevelopmental disorders	429097	Sedative, hypnotic, or anxiolytic-induced mild neurocognitive disorder with moderate or severe	ICD10CM	1	F13.288		Sedative, hypnotic or anxiolytic dependence with other	IMO	GHS-PACDC	IMO_2015_R	ACTIVE	N	1900-01-01	9999-12-31	2015-07-10
Diagnosis: ICD10 Code, F00-F99 Define	F01-F99 Mental, Behavioral and Neurodevelopmental disorders	491981	Barbiturate use disorder, severe, in controlled environment (HCC)	ICD10CM	1	F13.90		Sedative, hypnotic, or anxiolytic use, unspecified, uncom	IMO	GHS-PACDC	IMO_2015_R	ACTIVE	N	1900-01-01	2016-10-14	2015-07-10
Diagnosis: ICD10 Code, F00-F99 Define	F01-F99 Mental, Behavioral and Neurodevelopmental disorders	484176	Severe propofol use disorder (HCC)	ICD10CM	1	F13.90		Sedative, hypnotic, or anxiolytic use, unspecified, uncom	IMO	GHS-PACDC	IMO_2015_R	ACTIVE	N	1900-01-01	2016-10-14	2015-07-10
Diagnosis: ICD10 Code, F00-F99 Define	F01-F99 Mental, Behavioral and Neurodevelopmental disorders	491838	Librium use disorder, mild, in sustained remission	ICD10CM	1	F13.90		Sedative, hypnotic, or anxiolytic use, unspecified, uncom	IMO	GHS-PACDC	IMO_2015_R	ACTIVE	N	1900-01-01	2016-10-14	2015-07-10
Diagnosis: ICD10 Code, F00-F99 Define	F01-F99 Mental, Behavioral and Neurodevelopmental disorders	428933	Moderate sedative, hypnotic, or anxiolytic use disorder	ICD10CM	1	F13.90		Sedative, hypnotic, or anxiolytic use, unspecified, uncom	IMO	GHS-PACDC	IMO_2015_R	ACTIVE	N	1900-01-01	9999-12-31	2015-07-10
Diagnosis: ICD10 Code, F00-F99 Define	F01-F99 Mental, Behavioral and Neurodevelopmental disorders	488572	Sedative, hypnotic or anxiolytic use disorder, moderate, in sustained remission	ICD10CM	1	F13.90		Sedative, hypnotic, or anxiolytic use, unspecified, uncom	IMO	GHS-PACDC	IMO_2015_R	ACTIVE	N	1900-01-01	9999-12-31	2015-07-10
Diagnosis: ICD10 Code, F00-F99 Define	F01-F99 Mental, Behavioral and Neurodevelopmental disorders	479897	Severe Fiorinal use disorder (HCC)	ICD10CM	1	F13.90		Sedative, hypnotic, or anxiolytic use, unspecified, uncom	IMO	GHS-PACDC	IMO_2015_R	ACTIVE	N	1900-01-01	2016-10-14	2015-07-10
Diagnosis: ICD10 Code, F00-F99 Define	F01-F99 Mental, Behavioral and Neurodevelopmental disorders	484862	Moderate flurazepam use disorder in sustained remission (HCC)	ICD10CM	1	F13.90		Sedative, hypnotic, or anxiolytic use, unspecified, uncom	IMO	GHS-PACDC	IMO_2015_R	ACTIVE	N	1900-01-01	2016-10-14	2015-07-10
Diagnosis: ICD10 Code, F00-F99 Define	F01-F99 Mental, Behavioral and Neurodevelopmental disorders	492300	Flexeril use disorder, moderate, in controlled environment (HCC)	ICD10CM	1	F13.90		Sedative, hypnotic, or anxiolytic use, unspecified, uncom	IMO	GHS-PACDC	IMO_2015_R	ACTIVE	N	1900-01-01	2016-10-14	2015-07-10
Diagnosis: ICD10 Code, F00-F99 Define	F01-F99 Mental, Behavioral and Neurodevelopmental disorders	492157	Placidyl use disorder, mild, in early remission	ICD10CM	1	F13.90		Sedative, hypnotic, or anxiolytic use, unspecified, uncom	IMO	GHS-PACDC	IMO_2015_R	ACTIVE	N	1900-01-01	2016-10-14	2015-07-10
Diagnosis: ICD10 Code, F00-F99 Define	F01-F99 Mental, Behavioral and Neurodevelopmental disorders	484914	Severe barbiturate use disorder in sustained remission (HCC)	ICD10CM	1	F13.90		Sedative, hypnotic, or anxiolytic use, unspecified, uncom	IMO	GHS-PACDC	IMO_2015_R	ACTIVE	N	1900-01-01	2016-10-14	2015-07-10
Diagnosis: ICD10 Code, F00-F99 Define	F01-F99 Mental, Behavioral and Neurodevelopmental disorders	479951	Severe butalbital-aspirin-caffeine use disorder (HCC)	ICD10CM	1	F13.90		Sedative, hypnotic, or anxiolytic use, unspecified, uncom	IMO	GHS-PACDC	IMO_2015_R	ACTIVE	N	1900-01-01	2016-10-14	2015-07-10
Diagnosis: ICD10 Code, F00-F99 Define	F01-F99 Mental, Behavioral and Neurodevelopmental disorders	484786	Mild temazepam use disorder in controlled environment	ICD10CM	1	F13.90		Sedative, hypnotic, or anxiolytic use, unspecified, uncom	IMO	GHS-PACDC	IMO_2015_R	ACTIVE	N	1900-01-01	2016-10-14	2015-07-10
Diagnosis: ICD10 Code, F00-F99 Define	F01-F99 Mental, Behavioral and Neurodevelopmental disorders	491809	Placidyl use disorder, moderate, in controlled environment (HCC)	ICD10CM	1	F13.90		Sedative, hypnotic, or anxiolytic use, unspecified, uncom	IMO	GHS-PACDC	IMO_2015_R	ACTIVE	N	1900-01-01	2016-10-14	2015-07-10
Diagnosis: ICD10 Code, F00-F99 Define	F01-F99 Mental, Behavioral and Neurodevelopmental disorders	484842	Moderate flunitrazepam use disorder in controlled environment (HCC)	ICD10CM	1	F13.90		Sedative, hypnotic, or anxiolytic use, unspecified, uncom	IMO	GHS-PACDC	IMO_2015_R	ACTIVE	N	1900-01-01	2016-10-14	2015-07-10
Diagnosis: ICD10 Code, F00-F99 Define	F01-F99 Mental, Behavioral and Neurodevelopmental disorders	484966	Mild temazepam use disorder in early remission	ICD10CM	1	F13.90		Sedative, hypnotic, or anxiolytic use, unspecified, uncom	IMO	GHS-PACDC	IMO_2015_R	ACTIVE	N	1900-01-01	2016-10-14	2015-07-10
Diagnosis: ICD10 Code, F00-F99 Define	F01-F99 Mental, Behavioral and Neurodevelopmental disorders	484245	Moderate temazepam use disorder in early remission (HCC)	ICD10CM	1	F13.90		Sedative, hypnotic, or anxiolytic use, unspecified, uncom	IMO	GHS-PACDC	IMO_2015_R	ACTIVE	N	1900-01-01	2016-10-14	2015-07-10
Diagnosis: ICD10 Code, F00-F99 Define	F01-F99 Mental, Behavioral and Neurodevelopmental disorders	484975	Severe butalbital-aspirin-caffeine use disorder in sustained remission (HCC)	ICD10CM	1	F13.90		Sedative, hypnotic, or anxiolytic use, unspecified, uncom	IMO	GHS-PACDC	IMO_2015_R	ACTIVE	N	1900-01-01	2016-10-14	2015-07-10
Diagnosis: ICD10 Code, F00-F99 Define	F01-F99 Mental, Behavioral and Neurodevelopmental disorders	1181386	Sedative, hypnotic, or anxiolytic use, unspecified, uncomplicated	ICD10CM	1	F13.90		Sedative, hypnotic, or anxiolytic use, unspecified, uncom	IMO	GHS-PACDC	IMO_2016_R	ACTIVE	N	1900-01-01	9999-12-31	2016-06-02
Diagnosis: ICD10 Code, F00-F99 Define	F01-F99 Mental, Behavioral and Neurodevelopmental disorders	484177	Propofol use disorder, severe (HCC)	ICD10CM	1	F13.90		Sedative, hypnotic, or anxiolytic use, unspecified, uncom	IMO	GHS-PACDC	IMO_2015_R	ACTIVE	N	1900-01-01	2016-10-14	2015-07-10
Diagnosis: ICD10 Code, F00-F99 Define	F01-F99 Mental, Behavioral and Neurodevelopmental disorders	484942	Mild flunitrazepam use disorder in early remission	ICD10CM	1	F13.90		Sedative, hypnotic, or anxiolytic use, unspecified, uncom	IMO	GHS-PACDC	IMO_2015_R	ACTIVE	N	1900-01-01	2016-10-14	2015-07-10
Diagnosis: ICD10 Code, F00-F99 Define	F01-F99 Mental, Behavioral and Neurodevelopmental disorders	492271	Restoril use disorder, mild, in early remission	ICD10CM	1	F13.90		Sedative, hypnotic, or anxiolytic use, unspecified, uncom	IMO	GHS-PACDC	IMO_2015_R	ACTIVE	N	1900-01-01	2016-10-14	2015-07-10
Diagnosis: ICD10 Code, F00-F99 Define	F01-F99 Mental, Behavioral and Neurodevelopmental disorders	484953	Moderate oxazepam use disorder in sustained remission (HCC)	ICD10CM	1	F13.90		Sedative, hypnotic, or anxiolytic use, unspecified, uncom	IMO	GHS-PACDC	IMO_2015_R	ACTIVE	N	1900-01-01	2016-10-14	2015-07-10
Diagnosis: ICD10 Code, F00-F99 Define	F01-F99 Mental, Behavioral and Neurodevelopmental disorders	413518	Employs barbiturates as base powder during the smoking of heroin	ICD10CM	2	F13.90	F11.90,F13.90	Sedative, hypnotic, or anxiolytic use, unspecified, uncom	IMO	GHS-PACDC	DX_LEGACY	ACTIVE	N	1900-01-01	9999-12-31	2015-07-10
Diagnosis: ICD10 Code, F00-F99 Define	F01-F99 Mental, Behavioral and Neurodevelopmental disorders	479852	Moderate cyclobenzaprine use disorder (HCC)	ICD10CM	1	F13.90		Sedative, hypnotic, or anxiolytic use, unspecified, uncom	IMO	GHS-PACDC	IMO_2015_R	ACTIVE	N	1900-01-01	2016-10-14	2015-07-10
Diagnosis: ICD10 Code, F00-F99 Define	F01-F99 Mental, Behavioral and Neurodevelopmental disorders	492124	Fiorinal use disorder, severe, in sustained remission (HCC)	ICD10CM	1	F13.90		Sedative, hypnotic, or anxiolytic use, unspecified, uncom	IMO	GHS-PACDC	IMO_2015_R	ACTIVE	N	1900-01-01	2016-10-14	2015-07-10
Diagnosis: ICD10 Code, F00-F99 Define	F01-F99 Mental, Behavioral and Neurodevelopmental disorders	492328	Halcion use disorder, mild, in controlled environment	ICD10CM	1	F13.90		Sedative, hypnotic, or anxiolytic use, unspecified, uncom	IMO	GHS-PACDC	IMO_2015_R	ACTIVE	N	1900-01-01	2016-10-14	2015-07-10
Diagnosis: ICD10 Code, F00-F99 Define	F01-F99 Mental, Behavioral and Neurodevelopmental disorders	486757	Severe sedative, hypnotic, or anxiolytic use disorder, in sustained remission (HCC)	ICD10CM	1	F13.90		Sedative, hypnotic, or anxiolytic use, unspecified, uncom	IMO	GHS-PACDC	IMO_2015_R	ACTIVE	N	1900-01-01	2016-10-14	2015-07-10
Diagnosis: ICD10 Code, F00-F99 Define	F01-F99 Mental, Behavioral and Neurodevelopmental disorders	969318	Sedative, hypnotic, or anxiolytic intoxication without use disorder, uncomplicated (HCC)	ICD10CM	1	F13.920		Sedative, hypnotic or anxiolytic use, unspecified with int	IMO	GHS-PACDC	IMO_2015_R	ACTIVE	N	2016-09-17	9999-12-31	2016-09-30
Diagnosis: ICD10 Code, F00-F99 Define	F01-F99 Mental, Behavioral and Neurodevelopmental disorders	974077	Onset of sedative, hypnotic, or anxiolytic-induced psychotic disorder during intoxication, with h	ICD10CM	2	F13.920	F13.951,F13.920	Sedative, hypnotic or anxiolytic use, unspecified with int	IMO	GHS-PACDC	IMO_2015_R	DELETED	Y	2017-03-31	9999-12-31	2017-04-14
Diagnosis: ICD10 Code, F00-F99 Define	F01-F99 Mental, Behavioral and Neurodevelopmental disorders	966424	Onset of sedative, hypnotic or anxiolytic-induced mood disorder during intoxication, with deliri	ICD10CM	2	F13.921	F13.121,F13.921	Sedative, hypnotic or anxiolytic use, unspecified with int	IMO	GHS-PACDC	IMO_2015_R	ACTIVE	N	2016-04-10	9999-12-31	2015-07-10

Diagnosis: ICD10 Code, F00-F99 Define	F01-F99 Mental, Behavioral and Neurodevelopmental disorders	486912	Persistent sedative, hypnotic, or anxiolytic intoxication delirium with moderate or severe use di	ICD10CM	1	F13.921		Sedative, hypnotic or anxiolytic use, unspecified with int	IMO	GHS-PACDC	IMO_2015_R	ACTIVE	N	1900-01-01	2016-10-14	2015-07-10
Diagnosis: ICD10 Code, F00-F99 Define	F01-F99 Mental, Behavioral and Neurodevelopmental disorders	114326	Sedative, hypnotic, or anxiolytic intoxication delirium (HCC)	ICD10CM	1	F13.921		Sedative, hypnotic or anxiolytic use, unspecified with int	IMO	GHS-PACDC	DX_LEGACY	ACTIVE	N	1900-01-01	9999-12-31	2015-07-10
Diagnosis: ICD10 Code, F00-F99 Define	F01-F99 Mental, Behavioral and Neurodevelopmental disorders	30184	Anxiolytic intoxication (HCC)	ICD10CM	1	F13.929		Sedative, hypnotic or anxiolytic use, unspecified with int	IMO	GHS-PACDC	DX_LEGACY	ACTIVE	N	2016-09-17	9999-12-31	2016-09-30
Diagnosis: ICD10 Code, F00-F99 Define	F01-F99 Mental, Behavioral and Neurodevelopmental disorders	453020	Acute lorazepam intoxication with complication (HCC)	ICD10CM	1	F13.929		Sedative, hypnotic or anxiolytic use, unspecified with int	IMO	GHS-PACDC	IMO_2015_R	ACTIVE	N	2016-09-17	9999-12-31	2016-09-30
Diagnosis: ICD10 Code, F00-F99 Define	F01-F99 Mental, Behavioral and Neurodevelopmental disorders	974697	Sedative, hypnotic, or anxiolytic-induced psychotic disorder with moderate or severe use disord	ICD10CM	2	F13.929	F13.959,F13.929	Sedative, hypnotic or anxiolytic use, unspecified with int	IMO	GHS-PACDC	IMO_2015_R	ACTIVE	N	2016-04-10	2016-10-14	2015-07-10
Diagnosis: ICD10 Code, F00-F99 Define	F01-F99 Mental, Behavioral and Neurodevelopmental disorders	601014	Sedative, hypnotic or anxiolytic use, unspecified with withdrawal, uncomplicated	ICD10CM	1	F13.930		Sedative, hypnotic or anxiolytic use, unspecified with wi	IMO	GHS-PACDC	IMO_2015_R	ACTIVE	Y	1900-01-01	9999-12-31	2015-07-10
Diagnosis: ICD10 Code, F00-F99 Define	F01-F99 Mental, Behavioral and Neurodevelopmental disorders	488830	Sedative, hypnotic, or anxiolytic withdrawal delirium, persistent, hypoactive (HCC)	ICD10CM	1	F13.931		Sedative, hypnotic or anxiolytic use, unspecified with wi	IMO	GHS-PACDC	IMO_2015_R	ACTIVE	N	1900-01-01	9999-12-31	2015-07-10
Diagnosis: ICD10 Code, F00-F99 Define	F01-F99 Mental, Behavioral and Neurodevelopmental disorders	488854	Substance withdrawal delirium, sedative, hypnotic, or anxiolytic, acute (HCC)	ICD10CM	1	F13.931		Sedative, hypnotic or anxiolytic use, unspecified with wi	IMO	GHS-PACDC	IMO_2015_R	ACTIVE	N	1900-01-01	9999-12-31	2015-07-10
Diagnosis: ICD10 Code, F00-F99 Define	F01-F99 Mental, Behavioral and Neurodevelopmental disorders	488829	Persistent hypoactive sedative, hypnotic, or anxiolytic withdrawal delirium (HCC)	ICD10CM	1	F13.931		Sedative, hypnotic or anxiolytic use, unspecified with wi	IMO	GHS-PACDC	IMO_2015_R	ACTIVE	N	1900-01-01	9999-12-31	2015-07-10
Diagnosis: ICD10 Code, F00-F99 Define	F01-F99 Mental, Behavioral and Neurodevelopmental disorders	975901	Sedative, hypnotic, or anxiolytic-induced psychotic disorder with moderate or severe use disord	ICD10CM	2	F13.932	F13.951,F13.932	Sedative, hypnotic or anxiolytic use, unspecified with wi	IMO	GHS-PACDC	IMO_2015_R	ACTIVE	N	2016-04-10	2016-10-14	2015-07-10
Diagnosis: ICD10 Code, F00-F99 Define	F01-F99 Mental, Behavioral and Neurodevelopmental disorders	429125	Sedative, hypnotic, or anxiolytic-induced bipolar and related disorder without use disorder (HC	ICD10CM	1	F13.94		Sedative, hypnotic or anxiolytic use, unspecified with se	IMO	GHS-PACDC	IMO_2015_R	ACTIVE	N	1900-01-01	9999-12-31	2015-07-10
Diagnosis: ICD10 Code, F00-F99 Define	F01-F99 Mental, Behavioral and Neurodevelopmental disorders	486744	Sedative, hypnotic, or anxiolytic-induced depressive disorder with moderate or severe use disor	ICD10CM	1	F13.94		Sedative, hypnotic or anxiolytic use, unspecified with se	IMO	GHS-PACDC	IMO_2015_R	ACTIVE	N	1900-01-01	2016-10-14	2015-07-10
Diagnosis: ICD10 Code, F00-F99 Define	F01-F99 Mental, Behavioral and Neurodevelopmental disorders	613621	Sedative, hypnotic or anxiolytic use, unspecified with sedative, hypnotic or anxiolytic-induced p	ICD10CM	1	F13.95		Sedative, hypnotic or anxiolytic use, unspecified with se	IMO	GHS-PACDC	IMO_2015_R	ACTIVE	Y	1900-01-01	9999-12-31	2015-07-10
Diagnosis: ICD10 Code, F00-F99 Define	F01-F99 Mental, Behavioral and Neurodevelopmental disorders	975100	Onset of sedative, hypnotic, or anxiolytic-induced psychotic disorder during withdrawal, with de	ICD10CM	1	F13.950		Sedative, hypnotic or anxiolytic use, unspecified with se	IMO	GHS-PACDC	IMO_2015_R	ACTIVE	N	1900-01-01	2016-05-07	2015-07-10
Diagnosis: ICD10 Code, F00-F99 Define	F01-F99 Mental, Behavioral and Neurodevelopmental disorders	428695	Benzodiazepine-induced psychosis, with delusions (HCC)	ICD10CM	1	F13.950		Sedative, hypnotic or anxiolytic use, unspecified with se	IMO	GHS-PACDC	IMO_2015_R	ACTIVE	N	1900-01-01	9999-12-31	2015-07-10
Diagnosis: ICD10 Code, F00-F99 Define	F01-F99 Mental, Behavioral and Neurodevelopmental disorders	429142	Sedative, hypnotic, or anxiolytic-induced major neurocognitive disorder without use disorder (H	ICD10CM	1	F13.97		Sedative, hypnotic or anxiolytic use, unspecified with se	IMO	GHS-PACDC	IMO_2015_R	ACTIVE	N	1900-01-01	9999-12-31	2015-07-10
Diagnosis: ICD10 Code, F00-F99 Define	F01-F99 Mental, Behavioral and Neurodevelopmental disorders	429119	Sedative, hypnotic, or anxiolytic-induced anxiety disorder without use disorder (HCC)	ICD10CM	1	F13.980		Sedative, hypnotic or anxiolytic use, unspecified with se	IMO	GHS-PACDC	IMO_2015_R	ACTIVE	N	1900-01-01	9999-12-31	2015-07-10
Diagnosis: ICD10 Code, F00-F99 Define	F01-F99 Mental, Behavioral and Neurodevelopmental disorders	597791	Sedative, hypnotic or anxiolytic use, unspecified with sedative, hypnotic or anxiolytic-induced a	ICD10CM	1	F13.980		Sedative, hypnotic or anxiolytic use, unspecified with se	IMO	GHS-PACDC	IMO_2015_R	ACTIVE	Y	1900-01-01	9999-12-31	2015-07-10
Diagnosis: ICD10 Code, F00-F99 Define	F01-F99 Mental, Behavioral and Neurodevelopmental disorders	489269	Sedative, hypnotic, or anxiolytic-induced sexual dysfunction, with onset after medication use, m	ICD10CM	1	F13.981		Sedative, hypnotic or anxiolytic use, unspecified with se	IMO	GHS-PACDC	IMO_2015_R	ACTIVE	N	1900-01-01	9999-12-31	2015-07-10
Diagnosis: ICD10 Code, F00-F99 Define	F01-F99 Mental, Behavioral and Neurodevelopmental disorders	599101	Sedative, hypnotic or anxiolytic use, unspecified with sedative, hypnotic or anxiolytic-induced se	ICD10CM	1	F13.981		Sedative, hypnotic or anxiolytic use, unspecified with se	IMO	GHS-PACDC	IMO_2015_R	ACTIVE	Y	1900-01-01	9999-12-31	2015-07-10
Diagnosis: ICD10 Code, F00-F99 Define	F01-F99 Mental, Behavioral and Neurodevelopmental disorders	429112	Sedative, hypnotic, or anxiolytic-induced sleep disorder without use disorder (HCC)	ICD10CM	1	F13.982		Sedative, hypnotic or anxiolytic use, unspecified with se	IMO	GHS-PACDC	IMO_2015_R	ACTIVE	N	1900-01-01	9999-12-31	2015-07-10
Diagnosis: ICD10 Code, F00-F99 Define	F01-F99 Mental, Behavioral and Neurodevelopmental disorders	488848	Sedative, hypnotic, or anxiolytic-induced sleep disorder, insomnia type, with onset during intoxi	ICD10CM	1	F13.982		Sedative, hypnotic or anxiolytic use, unspecified with se	IMO	GHS-PACDC	IMO_2015_R	ACTIVE	N	1900-01-01	9999-12-31	2015-07-10
Diagnosis: ICD10 Code, F00-F99 Define	F01-F99 Mental, Behavioral and Neurodevelopmental disorders	488838	Sedative, hypnotic, or anxiolytic-induced mild neurocognitive disorder, persistent (HCC)	ICD10CM	1	F13.988		Sedative, hypnotic or anxiolytic use, unspecified with otl	IMO	GHS-PACDC	IMO_2015_R	ACTIVE	N	2016-09-17	9999-12-31	2016-09-30
Diagnosis: ICD10 Code, F00-F99 Define	F01-F99 Mental, Behavioral and Neurodevelopmental disorders	1184158	Sedative, hypnotic or anxiolytic use, unspecified with unspecified sedative, hypnotic or anxiolyti	ICD10CM	1	F13.99		Sedative, hypnotic or anxiolytic use, unspecified with un	IMO	GHS-PACDC	IMO_2016_R	ACTIVE	N	1900-01-01	9999-12-31	2016-06-02
Diagnosis: ICD10 Code, F00-F99 Define	F01-F99 Mental, Behavioral and Neurodevelopmental disorders	537622	Cocaine related disorders	ICD10CM	1	F14		Cocaine related disorders	IMO	GHS-PACDC	IMO_2015_R	ACTIVE	Y	1900-01-01	9999-12-31	2015-07-10
Diagnosis: ICD10 Code, F00-F99 Define	F01-F99 Mental, Behavioral and Neurodevelopmental disorders	509173	Cocaine use disorder, mild, in early remission, abuse	ICD10CM	1	F14.10		Cocaine abuse, uncomplicated	IMO	GHS-PACDC	IMO_2015_R	ACTIVE	N	1900-01-01	2017-10-14	2015-07-10
Diagnosis: ICD10 Code, F00-F99 Define	F01-F99 Mental, Behavioral and Neurodevelopmental disorders	522225	Cocaine use disorder, mild, abuse	ICD10CM	1	F14.10		Cocaine abuse, uncomplicated	IMO	GHS-PACDC	IMO_2015_R	ACTIVE	N	1900-01-01	9999-12-31	2015-07-10
Diagnosis: ICD10 Code, F00-F99 Define	F01-F99 Mental, Behavioral and Neurodevelopmental disorders	489456	Mild cocaine use disorder in controlled environment	ICD10CM	1	F14.10		Cocaine abuse, uncomplicated	IMO	GHS-PACDC	IMO_2015_R	ACTIVE	N	2016-09-17	9999-12-31	2016-09-30
Diagnosis: ICD10 Code, F00-F99 Define	F01-F99 Mental, Behavioral and Neurodevelopmental disorders	508554	Mild cocaine abuse in sustained remission in controlled environment	ICD10CM	1	F14.10		Cocaine abuse, uncomplicated	IMO	GHS-PACDC	IMO_2015_R	ACTIVE	N	1900-01-01	2017-10-14	2015-07-10
Diagnosis: ICD10 Code, F00-F99 Define	F01-F99 Mental, Behavioral and Neurodevelopmental disorders	592067	Cocaine abuse, uncomplicated	ICD10CM	1	F14.10		Cocaine abuse, uncomplicated	IMO	GHS-PACDC	IMO_2015_R	ACTIVE	Y	1900-01-01	9999-12-31	2015-07-10
Diagnosis: ICD10 Code, F00-F99 Define	F01-F99 Mental, Behavioral and Neurodevelopmental disorders	507067	Cocaine use disorder, mild, in early remission, in controlled environment, abuse	ICD10CM	1	F14.10		Cocaine abuse, uncomplicated	IMO	GHS-PACDC	IMO_2015_R	ACTIVE	N	1900-01-01	2017-10-14	2015-07-10
Diagnosis: ICD10 Code, F00-F99 Define	F01-F99 Mental, Behavioral and Neurodevelopmental disorders	964670	Cocaine intoxication without perceptual disturbances without use disorder, uncomplicated (HC	ICD10CM	1	F14.120		Cocaine abuse with intoxication, uncomplicated	IMO	GHS-PACDC	IMO_2015_R	ACTIVE	N	1900-01-01	2016-05-07	2015-07-10
Diagnosis: ICD10 Code, F00-F99 Define	F01-F99 Mental, Behavioral and Neurodevelopmental disorders	965342	Onset of cocaine-induced anxiety disorder during intoxication, uncomplicated	ICD10CM	1	F14.120	F14.120,F14.180	Cocaine abuse with intoxication, uncomplicated	IMO	GHS-PACDC	IMO_2015_R	DELETED	Y	1900-01-01	2016-05-07	2015-07-10
Diagnosis: ICD10 Code, F00-F99 Define	F01-F99 Mental, Behavioral and Neurodevelopmental disorders	1086224	Cocaine-induced psychotic disorder with onset during intoxication with complication, with delu	ICD10CM	3	F14.120	F14.150,F14.122,F14.120,F14.12	Cocaine abuse with intoxication, uncomplicated	IMO	GHS-PACDC	IMO_2015_R	DELETED	Y	1900-01-01	2016-05-07	2015-07-10
Diagnosis: ICD10 Code, F00-F99 Define	F01-F99 Mental, Behavioral and Neurodevelopmental disorders	965666	Cocaine intoxication without perceptual disturbances with moderate or severe use disorder, wi	ICD10CM	1	F14.121		Cocaine abuse with intoxication with delirium	IMO	GHS-PACDC	IMO_2015_R	ACTIVE	N	1900-01-01	2016-05-07	2015-07-10
Diagnosis: ICD10 Code, F00-F99 Define	F01-F99 Mental, Behavioral and Neurodevelopmental disorders	518419	Cocaine-induced sexual dysfunction with onset during intoxication with delirium (HCC)	ICD10CM	2	F14.121	F14.181,F14.121	Cocaine abuse with intoxication with delirium	IMO	GHS-PACDC	IMO_2015_R	ACTIVE	N	1900-01-01	2017-10-14	2015-07-10
Diagnosis: ICD10 Code, F00-F99 Define	F01-F99 Mental, Behavioral and Neurodevelopmental disorders	518397	Cocaine-induced sleep disorder with onset during intoxication with delirium (HCC)	ICD10CM	2	F14.121	F14.182,F14.121	Cocaine abuse with intoxication with delirium	IMO	GHS-PACDC	IMO_2015_R	ACTIVE	N	1900-01-01	2017-10-14	2015-07-10
Diagnosis: ICD10 Code, F00-F99 Define	F01-F99 Mental, Behavioral and Neurodevelopmental disorders	1089697	Cocaine intoxication without perceptual disturbances with moderate or severe use disorder wit	ICD10CM	1	F14.122		Cocaine abuse with intoxication with perceptual disturb	IMO	GHS-PACDC	IMO_2015_R	DELETED	Y	1900-01-01	9999-12-31	2015-07-10
Diagnosis: ICD10 Code, F00-F99 Define	F01-F99 Mental, Behavioral and Neurodevelopmental disorders	1086224	Cocaine-induced psychotic disorder with onset during intoxication with complication, with delu	ICD10CM	2	F14.122	F14.150,F14.122,F14.120,F14.12	Cocaine abuse with intoxication with perceptual disturb	IMO	GHS-PACDC	IMO_2015_R	DELETED	Y	1900-01-01	2016-05-07	2015-07-10
Diagnosis: ICD10 Code, F00-F99 Define	F01-F99 Mental, Behavioral and Neurodevelopmental disorders	965398	Onset of cocaine-induced mood disorder during intoxication, with unspecified complication (HC	ICD10CM	1	F14.129		Cocaine abuse with intoxication, unspecified	IMO	GHS-PACDC	IMO_2015_R	ACTIVE	N	2016-04-10	9999-12-31	2015-07-10
Diagnosis: ICD10 Code, F00-F99 Define	F01-F99 Mental, Behavioral and Neurodevelopmental disorders	518551	Cocaine-induced sleep disorder with onset during intoxication with complication (HCC)	ICD10CM	2	F14.129	F14.182,F14.129	Cocaine abuse with intoxication, unspecified	IMO	GHS-PACDC	IMO_2015_R	ACTIVE	N	1900-01-01	2017-10-14	2015-07-10
Diagnosis: ICD10 Code, F00-F99 Define	F01-F99 Mental, Behavioral and Neurodevelopmental disorders	518534	Cocaine-induced sexual dysfunction with onset during intoxication with complication (HCC)	ICD10CM	2	F14.129	F14.181,F14.129	Cocaine abuse with intoxication, unspecified	IMO	GHS-PACDC	IMO_2015_R	ACTIVE	N	1900-01-01	2017-10-14	2015-07-10
Diagnosis: ICD10 Code, F00-F99 Define	F01-F99 Mental, Behavioral and Neurodevelopmental disorders	1088144	Cocaine-induced psychotic disorder with mild use disorder with onset during intoxication with c	ICD10CM	4	F14.129	F14.151,F14.120,F14.122,F14.12	Cocaine abuse with intoxication, unspecified	IMO	GHS-PACDC	IMO_2015_R	ACTIVE	N	1900-01-01	2016-05-07	2015-07-10
Diagnosis: ICD10 Code, F00-F99 Define	F01-F99 Mental, Behavioral and Neurodevelopmental disorders	516537	Cocaine-induced psychotic disorder without use disorder with onset during intoxication with de	ICD10CM	2	F14.129	F14.150,F14.129	Cocaine abuse with intoxication, unspecified	IMO	GHS-PACDC	IMO_2015_R	ACTIVE	N	1900-01-01	2016-10-14	2015-07-10
Diagnosis: ICD10 Code, F00-F99 Define	F01-F99 Mental, Behavioral and Neurodevelopmental disorders	1088937	Cocaine intoxication without perceptual disturbances with moderate or severe use disorder wit	ICD10CM	1	F14.129		Cocaine abuse with intoxication, unspecified	IMO	GHS-PACDC	IMO_2015_R	DELETED	Y	1900-01-01	9999-12-31	2015-07-10
Diagnosis: ICD10 Code, F00-F99 Define	F01-F99 Mental, Behavioral and Neurodevelopmental disorders	974277	Cocaine-induced psychotic disorder with moderate or severe use disorder with onset during int	ICD10CM	2	F14.129	F14.951,F14.129	Cocaine abuse with intoxication, unspecified	IMO	GHS-PACDC	IMO_2015_R	ACTIVE	N	2016-04-10	2016-10-14	2015-07-10
Diagnosis: ICD10 Code, F00-F99 Define	F01-F99 Mental, Behavioral and Neurodevelopmental disorders	517438	Cocaine-induced mood disorder with onset during intoxication with complication (HCC)	ICD10CM	1	F14.14	F14.14,F14.129	Cocaine abuse with cocaine-induced mood disorder	IMO	GHS-PACDC	IMO_2015_R	ACTIVE	N	1900-01-01	2017-10-14	2015-07-10
Diagnosis: ICD10 Code, F00-F99 Define	F01-F99 Mental, Behavioral and Neurodevelopmental disorders	592660	Cocaine abuse with cocaine-induced psychotic disorder	ICD10CM	1	F14.15		Cocaine abuse with cocaine-induced psychotic disorder	IMO	GHS-PACDC	IMO_2015_R	ACTIVE	Y	1900-01-01	9999-12-31	2015-07-10
Diagnosis: ICD10 Code, F00-F99 Define	F01-F99 Mental, Behavioral and Neurodevelopmental disorders	966945	Cocaine-induced psychotic disorder with mild use disorder, with delusions (HCC)	ICD10CM	1	F14.150		Cocaine abuse with cocaine-induced psychotic disorder	IMO	GHS-PACDC	IMO_2015_R	ACTIVE	N	2016-09-17	9999-12-31	2016-09-30
Diagnosis: ICD10 Code, F00-F99 Define	F01-F99 Mental, Behavioral and Neurodevelopmental disorders	517641	Cocaine-induced psychotic disorder without use disorder with delusions (HCC)	ICD10CM	1	F14.150		Cocaine abuse with cocaine-induced psychotic disorder	IMO	GHS-PACDC	IMO_2015_R	ACTIVE	N	1900-01-01	2017-10-14	2015-07-10
Diagnosis: ICD10 Code, F00-F99 Define	F01-F99 Mental, Behavioral and Neurodevelopmental disorders	517716	Cocaine-induced psychotic disorder with moderate or severe use disorder with onset during int	ICD10CM	1	F14.151	F14.151,F14.129	Cocaine abuse with cocaine-induced psychotic disorder	IMO	GHS-PACDC	IMO_2015_R	DELETED	Y	1900-01-01	2016-10-14	2015-07-10
Diagnosis: ICD10 Code, F00-F99 Define	F01-F99 Mental, Behavioral and Neurodevelopmental disorders	961693	Cocaine abuse with cocaine-induced psychotic disorder, with unspecified complication (HCC)	ICD10CM	1	F14.159		Cocaine abuse with cocaine-induced psychotic disorder,	IMO	GHS-PACDC	IMO_2015_R	ACTIVE	N	1900-01-01	9999-12-31	2015-07-10
Diagnosis: ICD10 Code, F00-F99 Define	F01-F99 Mental, Behavioral and Neurodevelopmental disorders	975769	Cocaine-induced psychotic disorder with mild use disorder with onset during intoxication, with	ICD10CM	1	F14.159	F14.159,F14.129	Cocaine abuse with cocaine-induced psychotic disorder,	IMO	GHS-PACDC	IMO_2015_R	ACTIVE	N	2016-09-17	9999-12-31	2016-09-30
Diagnosis: ICD10 Code, F00-F99 Define	F01-F99 Mental, Behavioral and Neurodevelopmental disorders	428953	Cocaine-induced anxiety disorder with mild use disorder (HCC)	ICD10CM	1	F14.180		Cocaine abuse with cocaine-induced anxiety disorder	IMO	GHS-PACDC	IMO_2015_R	ACTIVE	N	1900-01-01	9999-12-31	2015-07-10
Diagnosis: ICD10 Code, F00-F99 Define	F01-F99 Mental, Behavioral and Neurodevelopmental disorders	489623	Cocaine-induced anxiety disorder with onset during intoxication, uncomplicated (HCC)	ICD10CM	1	F14.180	F14.180,F14.129	Cocaine abuse with cocaine-induced anxiety disorder	IMO	GHS-PACDC	IMO_2015_R	ACTIVE	N	1900-01-01	9999-12-31	2015-07-10
Diagnosis: ICD10 Code, F00-F99 Define	F01-F99 Mental, Behavioral and Neurodevelopmental disorders	518534	Cocaine-induced sexual dysfunction with onset during intoxication with complication (HCC)	ICD10CM	1	F14.181	F14.181,F14.129	Cocaine abuse with cocaine-induced sexual dysfunction	IMO	GHS-PACDC	IMO_2015_R	ACTIVE	N	1900-01-01	2017-10-14	2015-07-10
Diagnosis: ICD10 Code, F00-F99 Define	F01-F99 Mental, Behavioral and Neurodevelopmental disorders	428944	Cocaine dependence in controlled environment (HCC)	ICD10CM	1	F14.20		Cocaine dependence, uncomplicated	IMO	GHS-PACDC	IMO_2015_R	ACTIVE	N	1900-01-01	9999-12-31	2015-07-10
Diagnosis: ICD10 Code, F00-F99 Define	F01-F99 Mental, Behavioral and Neurodevelopmental disorders	317483	Cocaine dependence, daily use (HCC)	ICD10CM	1	F14.20		Cocaine dependence, uncomplicated	IMO	GHS-PACDC	DX_LEGACY	ACTIVE	N	1900-01-01	9999-12-31	2015-07-10
Diagnosis: ICD10 Code, F00-F99 Define	F01-F99 Mental, Behavioral and Neurodevelopmental disorders	488999	Severe cocaine use disorder in controlled environment (HCC)	ICD10CM	1	F14.20		Cocaine dependence, uncomplicated	IMO	GHS-PACDC	IMO_2015_R	ACTIVE	N	2016-09-17	9999-12-31	2016-09-30
Diagnosis: ICD10 Code, F00-F99 Define	F01-F99 Mental, Behavioral and Neurodevelopmental disorders	128616	Cocaine addiction (HCC)	ICD10CM	1	F14.20		Cocaine dependence, uncomplicated	IMO	GHS-PACDC	DX_LEGACY	ACTIVE	N	1900-01-01	9999-12-31	2015-07-10
Diagnosis: ICD10 Code, F00-F99 Define	F01-F99 Mental, Behavioral and Neurodevelopmental disorders	268169	Cocaine dependence, uncomplicated (HCC)	ICD10CM	1	F14.20		Cocaine dependence, uncomplicated	IMO	GHS-PACDC	DX_LEGACY	ACTIVE	N	1900-01-01	9999-12-31	2015-07-10
Diagnosis: ICD10 Code, F00-F99 Define	F01-F99 Mental, Behavioral and Neurodevelopmental disorders	508307	Severe cocaine dependence in early remission (HCC)	ICD10CM	1	F14.21		Cocaine dependence, in remission	IMO	GHS-PACDC	IMO_2015_R	ACTIVE	N	1900-01-01	9999-12-31	2015-07-10
Diagnosis: ICD10 Code, F00-F99 Define	F01-F99 Mental, Behavioral and Neurodevelopmental disorders	486917	Severe cocaine use disorder, in sustained remission (HCC)	ICD10CM	1	F14.21		Cocaine dependence, in remission	IMO	GHS-PACDC	IMO_2015_R	ACTIVE	N	2016-09-17	9999-12-31	2016-09-30
Diagnosis: ICD10 Code, F00-F99 Define	F01-F99 Mental, Behavioral and Neurodevelopmental disorders	965666	Cocaine intoxication without perceptual disturbances with moderate or severe use disorder, wi	ICD10CM	1	F14.221		Cocaine dependence with intoxication delirium	IMO	GHS-PACDC	IMO_2015_R	ACTIVE	N	2016-09-17	9999-12-31	2016-09-30
Diagnosis: ICD10 Code, F00-F99 Define	F01-F99 Mental, Behavioral and Neurodevelopmental disorders	486405	Persistent hyperactive cocaine intoxication delirium with moderate or severe use disorder (HCC	ICD10CM	1	F14.221		Cocaine dependence with intoxication delirium	IMO	GHS-PACDC	IMO_2015_R	ACTIVE	N	2016-09-17	9999-12-31	2016-09-30
Diagnosis: ICD10 Code, F00-F99 Define	F01-F99 Mental, Behavioral and Neurodevelopmental disorders	969019	Cocaine intoxication without perceptual disturbances with moderate or severe use disorder, wi	ICD10CM	1	F14.222		Cocaine dependence with intoxication with perceptual c	IMO	GHS-PACDC	IMO_2015_R	ACTIVE	N	2016-09-17	9999-12-31	2016-09-30
Diagnosis: ICD10 Code, F00-F99 Define	F01-F99 Mental, Behavioral and Neurodevelopmental disorders	486455	Cocaine-induced sexual dysfunction with mild use disorder with onset during withdrawal (HCC)	ICD10CM	2	F14.23	F14.281,F14.23	Cocaine dependence with withdrawal	IMO	GHS-PACDC	IMO_2015_R	ACTIVE	N	2016-09-17	9999-12-31	2016-09-30
Diagnosis: ICD10 Code, F00-F99 Define	F01-F99 Mental, Behavioral and Neurodevelopmental disorders	588442	Cocaine dependence with withdrawal	ICD10CM	1	F14.23		Cocaine dependence with withdrawal	IMO	GHS-PACDC	IMO_2015_R	ACTIVE	Y	1900-01-01	9999-12-31	2015-07-10
Diagnosis: ICD10 Code, F00-F99 Define	F01-F99 Mental, Behavioral and Neurodevelopmental disorders	1087823	Cocaine-induced psychotic disorder without use disorder with onset during withdrawal with co	ICD10CM	2	F14.23	F14.259,F14.23	Cocaine dependence with withdrawal	IMO	GHS-PACDC	IMO_2015_R	DELETED	Y	1900-01-01	9999-12-31	2015-07-10
Diagnosis: ICD10 Code, F00-F99 Define	F01-F99 Mental, Behavioral and Neurodevelopmental disorders	488750	Onset of cocaine-induced sleep disorder, mixed type, during discontinuation or withdrawal (HC	ICD10CM	1	F14.23	F14.23,F14.282	Cocaine dependence with withdrawal	IMO	GHS-PACDC	IMO_2015_R	ACTIVE	N	1900-01-01	9999-12-31	2015-07-10
Diagnosis: ICD10 Code, F00-F99 Define	F01-F99 Mental, Behavioral and Neurodevelopmental disorders	486460	Cocaine-induced depressive disorder with moderate or severe use disorder with onset during w	ICD10CM	2	F14.23	F14.24,F14.23	Cocaine dependence with withdrawal	IMO	GHS-PACDC	IMO_2015_R	ACTIVE	N	1900-01-01	2016-10-14	2015-07-10
Diagnosis: ICD10 Code, F00-F99 Define	F01-F99 Mental, Behavioral and Neurodevelopmental disorders	517732	Cocaine-induced psychotic disorder without use disorder with onset during withdrawal with co	ICD10CM	2	F14.23	F14.259,F14.23	Cocaine dependence with withdrawal	IMO	GHS-PACDC	IMO_2015_R	ACTIVE	N	1900-01-01	9999-12-31	2015-07-10
Diagnosis: ICD10 Code, F00-F99 Define	F01-F99 Mental, Behavioral and Neurodevelopmental disorders	485915	Cocaine-induced bipolar and related disorder with moderate or severe use disorder with onset	ICD10CM	1	F14.24	F14.24,F14.23,F31.9	Cocaine dependence with cocaine-induced mood disord	IMO	GHS-PACDC	IMO_2015_R	ACTIVE	N	1900-01-01	2016-10-14	2015-07-10
Diagnosis: ICD10 Code, F00-F99 Define	F01-F99 Mental, Behavioral and Neurodevelopmental disorders	976184	Cocaine-induced psychotic disorder with moderate or severe use disorder with onset during int	ICD10CM	1	F14.250	F14.250,F14.229	Cocaine dependence with cocaine-induced psychotic dis	IMO	GHS-PACDC	IMO_2015_R	ACTIVE	N	2016-09-17	2017-10-14	2016-09-30
Diagnosis: ICD10 Code, F00-F99 Define	F01-F99 Mental, Behavioral and Neurodevelopmental disorders	344486	Cocaine dependence with cocaine-induced psychotic disorder with hallucinations (HCC)	ICD10CM	1	F14.251		Cocaine dependence with cocaine-induced psychotic dis	IMO	GHS-PACDC	DX_LEGACY	ACTIVE	N	1900-01-01	9999-12-31	2015-07-10
Diagnosis: ICD10 Code, F00-F99 Define	F01-F99 Mental, Behavioral and Neurodevelopmental disorders	517696	Cocaine-induced psychotic disorder with onset during withdrawal with complication (HCC)	ICD10CM	1	F14.259	F14.259,F14.23	Cocaine dependence with cocaine-induced psychotic dis	IMO	GHS-PACDC	IMO_2015_R	ACTIVE	N	1900-01-01	2016-10-14	2015-07-10
Diagnosis: ICD10 Code, F00-F99 Define	F01-F99 Mental, Behavioral and Neurodevelopmental disorders	485855	Cocaine-induced sleep disorder with moderate or severe use disorder, parasomnia type (HCC)	ICD10CM	1	F14.282		Cocaine dependence with cocaine-induced sleep disord	IMO	GHS-PACDC	IMO_2015_R	ACTIVE	N	2016-09-17	9999-12-31	2016-09-30
Diagnosis: ICD10 Code, F00-F99 Define	F01-F99 Mental, Behavioral and Neurodevelopmental disorders	488750	Onset of cocaine-induced sleep disorder, mixed type, during discontinuation or withdrawal (HC	ICD10CM	2	F14.282	F14.23,F14.282	Cocaine dependence with cocaine-induced sleep disord	IMO	GHS-PACDC	IMO_2015_R	ACTIVE	N	1900-01-01	9999-12-31	2015-07-10
Diagnosis: ICD10 Code, F00-F99 Define	F01-F99 Mental, Behavioral and Neurodevelopmental disorders	488735	Cocaine induced obsessive-compulsive disorder with onset during withdrawal (HCC)	ICD10CM	1	F14.288	F14.288,F42.9,F14.23	Cocaine dependence with other cocaine-induced disord	IMO	GHS-PACDC	IMO_2015_R	ACTIVE	N	2016-09-17	9999-12-31	2016-09-30
Diagnosis: ICD10 Code, F00-F99 Define	F01-F99 Mental, Behavioral and Neurodevelopmental disorders	485951	Cocaine-induced obsessive-compulsive and related disorder with moderate or severe use disor	ICD10CM	1	F14.288	F14.288,F14.23	Cocaine dependence with other cocaine-induced disord	IMO	GHS-PACDC	IMO_2015_R	ACTIVE	N	2016-09-17	9999-12-31	2016-09-30
Diagnosis: ICD10 Code, F00-F99 Define	F01-F99 Mental, Behavioral and Neurodevelopmental disorders	613386	Cocaine use, unspecified	ICD10CM	1	F14.9		Cocaine use, unspecified	IMO	GHS-PACDC	IMO_2015_R	ACTIVE	Y	1900-01-01	9999-12-31	2015-07-10
Diagnosis: ICD10 Code, F00-F99 Define	F01-F99 Mental, Behavioral and Neurodevelopmental disorders	486711	Moderate cocaine use disorder, in early remission (HCC)	ICD10CM	1	F14.90		Cocaine use, unspecified, uncomplicated	IMO	GHS-PACDC	IMO_2015_R	ACTIVE	N	1900-01-01	2016-10-14	2015-07-10
Diagnosis: ICD10 Code, F00-F99 Define	F01-F99 Mental, Behavioral and Neurodevelopmental disorders	486333	Severe cocaine use disorder, in early remission (HCC)	ICD10CM	1	F14.90		Cocaine use, unspecified, uncomplicated	IMO	GHS-PACDC	IMO_2015_R	ACTIVE	N	1900-01-01	2016-10-14	2015-07-10
Diagnosis: ICD10 Code, F00-F99 Define	F01-F99 Mental, Behavioral and Neurodevelopmental disorders	975919	Cocaine-induced psychotic disorder without use disorder with onset during intoxication, with u	ICD10CM	3	F14.920	F14.959,F14.929,F14.920,F14.92	Cocaine use, unspecified with intoxication, uncomplicate	IMO	GHS-PACDC	IMO_2015_R	ACTIVE	N	1900-01-01	2016-05-07	2015-07-10
Diagnosis: ICD10 Code, F00-F99 Define	F01-F99 Mental, Behavioral and Neurodevelopmental disorders	486958	Persistent hypoactive cocaine intoxication delirium with moderate or severe use disorder (HCC)	ICD10CM	1	F14.921		Cocaine use, unspecified with intoxication delirium	IMO	GHS-PACDC	IMO_2015_R	ACTIVE	N	1900-01-01	2016-10-14	2015-07-10
Diagnosis: ICD10 Code, F00-F99 Define	F01-F99 Mental, Behavioral and Neurodevelopmental disorders	487157	Acute cocaine intoxication delirium without use disorder (HCC)	ICD10CM	1	F14.921		Cocaine use, unspecified with intoxication delirium	IMO	GHS-PACDC	IMO_2015_R	ACTIVE	N	1900-01-01	9999-12-31	2015-07-10
Diagnosis: ICD10 Code, F00-F99 Define	F01-F99 Mental, Behavioral and Neurodevelopmental disorders	30124	Cocaine intoxication delirium (HCC)	ICD10CM	1	F14.921		Cocaine use, unspecified with intoxication delirium	IMO	GHS-PACDC	DX_LEGACY	ACTIVE	N	1900-01-01	9999-12-31	2015-07-10
Diagnosis: ICD10 Code, F00-F99 Define	F01-F99 Mental, Behavioral and Neurodevelopmental disorders	428943	Cocaine intoxication delirium without use disorder (HCC)	ICD10CM	1	F14.921		Cocaine use, unspecified with intoxication delirium	IMO	GHS-PACDC	IMO_2015_R	ACTIVE	N	2016-09-17	9999-12-31	2016-09-30
Diagnosis: ICD10 Code, F00-F99 Define	F01-F99 Mental, Behavioral and Neurodevelopmental disorders	518397	Cocaine-induced sleep disorder with onset during intoxication with delirium (HCC)	ICD10CM	2	F14.921	F14.982,F14.921	Cocaine use, unspecified with intoxication delirium	IMO	GHS-PACDC	IMO_2015_R	ACTIVE	N	2017-09-17	9999-12-31	2017-10-30
Diagnosis: ICD10 Code, F00-F99 Define	F01-F99 Mental, Behavioral and Neurodevelopmental disorders	1088191	Cocaine-induced psychotic disorder without use disorder with onset during intoxication with co	ICD10CM	1	F14.922	F14.922,F14.950	Cocaine use, unspecified with intoxication with percept	IMO	GHS-PACDC	IMO_2015_R	ACTIVE	N	2017-09-17	9999-12-31	2017-10-30
Diagnosis: ICD10 Code, F00-F99 Define	F01-F99 Mental, Behavioral and Neurodevelopmental disorders	518527	Cocaine-induced sexual dysfunction with onset during intoxication with perceptual disturbance	ICD10CM	2	F14.922	F14.981,F14.922	Cocaine use, unspecified with intoxication with percept	IMO	GHS-PACDC	IMO_2015_R	ACTIVE	N	2017-09-17	9999-12-31	2017-10-30
Diagnosis: ICD10 Code, F00-F99 Define	F01-F99 Mental, Behavioral and Neurodevelopmental disorders	279285	Cocaine use with intoxication with perceptual disturbance (HCC)	ICD10CM	1	F14.922		Cocaine use, unspecified with intoxication with percept	IMO	GHS-PACDC	DX_LEGACY	ACTIVE	N	1900-01-01	9999-12-31	2015-07-10
Diagnosis: ICD10 Code, F00-F99 Define	F01-F99 Mental, Behavioral and Neurodevelopmental disorders	976398	Cocaine-induced psychotic disorder with mild use disorder with onset during intoxication, with	ICD10CM	4	F14.922	F14.950,F14.921,F14.929,F14.92	Cocaine use, unspecified with intoxication with percept	IMO	GHS-PACDC	IMO_2015_R	ACTIVE	N	1900-01-01	2016-05-07	2015-07-10
Diagnosis: ICD10 Code, F00-F99 Define	F01-F99 Mental, Behavioral and Neurodevelopmental disorders	517898	Cocaine intoxication without perceptual disturbances with mild use disorder with complication	ICD10CM	1	F14.929		Cocaine use, unspecified with intoxication, unspecified	IMO	GHS-PACDC	IMO_2015_R	ACTIVE	N	2016-09-17	2017-10-14	2016-09-30
Diagnosis: ICD10 Code, F00-F99 Define	F01-F99 Mental, Behavioral and Neurodevelopmental disorders	975919	Cocaine-induced psychotic disorder without use disorder with onset during intoxication, with u	ICD10CM	2	F14.929	F14.959,F14.929	Cocaine use, unspecified with intoxication, unspecified	IMO	GHS-PACDC	IMO_2015_R	ACTIVE	N	2016-04-10	9999-12-31	2015-07-10

Diagnosis: ICD10 Code, F00-F99 Define	F01-F99 Mental, Behavioral and Neurodevelopmental disorders	486984	Cocaine-induced bipolar and related disorder without use disorder with onset during intoxicatic	ICD10CM	2	F14.92	F14.94,F14.929	Cocaine use, unspecified with intoxication, unspecified	IMO	GHS-PACDC	IMO_2015_R	ACTIVE	N	2016-09-17	9999-12-31	2016-09-30
Diagnosis: ICD10 Code, F00-F99 Define	F01-F99 Mental, Behavioral and Neurodevelopmental disorders	485949	Cocaine-induced depressive disorder with moderate or severe use disorder with onset during ir	ICD10CM	1	F14.94	F14.94,F14.929	Cocaine use, unspecified with cocaine-induced mood dis	IMO	GHS-PACDC	IMO_2015_R	ACTIVE	N	1900-01-01	2016-10-14	2015-07-10
Diagnosis: ICD10 Code, F00-F99 Define	F01-F99 Mental, Behavioral and Neurodevelopmental disorders	518170	Cocaine-induced mood disorder with onset during intoxication with delirium (HCC)	ICD10CM	1	F14.94	F14.94,F14.921	Cocaine use, unspecified with cocaine-induced mood dis	IMO	GHS-PACDC	IMO_2015_R	ACTIVE	N	2017-09-17	9999-12-31	2017-10-30
Diagnosis: ICD10 Code, F00-F99 Define	F01-F99 Mental, Behavioral and Neurodevelopmental disorders	966883	Cocaine-induced psychotic disorder with moderate or severe use disorder, with delusions (HCC)	ICD10CM	1	F14.950		Cocaine use, unspecified with cocaine-induced psychotic	IMO	GHS-PACDC	IMO_2015_R	ACTIVE	N	1900-01-01	2016-10-14	2015-07-10
Diagnosis: ICD10 Code, F00-F99 Define	F01-F99 Mental, Behavioral and Neurodevelopmental disorders	976203	Onset of cocaine-induced psychotic disorder during withdrawal, with hallucinations (HCC)	ICD10CM	1	F14.951		Cocaine use, unspecified with cocaine-induced psychotic	IMO	GHS-PACDC	IMO_2015_R	ACTIVE	N	1900-01-01	2016-05-07	2015-07-10
Diagnosis: ICD10 Code, F00-F99 Define	F01-F99 Mental, Behavioral and Neurodevelopmental disorders	374508	Cocaine use w/cocaine-induced psychotic disorder w/hallucinations (HCC)	ICD10CM	1	F14.951		Cocaine use, unspecified with cocaine-induced psychotic	IMO	GHS-PACDC	DX_LEGACY	ACTIVE	N	1900-01-01	9999-12-31	2015-07-10
Diagnosis: ICD10 Code, F00-F99 Define	F01-F99 Mental, Behavioral and Neurodevelopmental disorders	350553	Cocaine use with cocaine-induced psychotic disorder with hallucinations (HCC)	ICD10CM	1	F14.951		Cocaine use, unspecified with cocaine-induced psychotic	IMO	GHS-PACDC	DX_LEGACY	ACTIVE	N	1900-01-01	9999-12-31	2015-07-10
Diagnosis: ICD10 Code, F00-F99 Define	F01-F99 Mental, Behavioral and Neurodevelopmental disorders	429221	Onset of cocaine-induced psychotic disorder with hallucinations during intoxication (HCC)	ICD10CM	1	F14.951	F14.951,F14.922	Cocaine use, unspecified with cocaine-induced psychotic	IMO	GHS-PACDC	IMO_2015_R	ACTIVE	N	2017-03-31	9999-12-31	2017-04-14
Diagnosis: ICD10 Code, F00-F99 Define	F01-F99 Mental, Behavioral and Neurodevelopmental disorders	971860	Cocaine-induced psychotic disorder with onset during withdrawal, with unspecified complicatio	ICD10CM	1	F14.959		Cocaine use, unspecified with cocaine-induced psychotic	IMO	GHS-PACDC	IMO_2015_R	DELETED	Y	1900-01-01	9999-12-31	2015-07-10
Diagnosis: ICD10 Code, F00-F99 Define	F01-F99 Mental, Behavioral and Neurodevelopmental disorders	1265483	Cocaine-induced psychotic disorder without use disorder with onset during intoxication, with pe	ICD10CM	1	F14.95	F14.959,F14.922	Cocaine use, unspecified with cocaine-induced psychotic	IMO	GHS-PACDC	IMO_2017_R	ACTIVE	N	1900-01-01	9999-12-31	2016-09-30
Diagnosis: ICD10 Code, F00-F99 Define	F01-F99 Mental, Behavioral and Neurodevelopmental disorders	974108	Cocaine-induced psychotic disorder with onset during intoxication, with unspecified complicatic	ICD10CM	2	F14.95	F14.929,F14.959	Cocaine use, unspecified with cocaine-induced psychotic	IMO	GHS-PACDC	IMO_2015_R	ACTIVE	N	2017-09-17	9999-12-31	2017-10-30
Diagnosis: ICD10 Code, F00-F99 Define	F01-F99 Mental, Behavioral and Neurodevelopmental disorders	289028	Cocaine use with cocaine-induced anxiety disorder (HCC)	ICD10CM	1	F14.980		Cocaine use, unspecified with cocaine-induced anxiety d	IMO	GHS-PACDC	DX_LEGACY	ACTIVE	N	1900-01-01	9999-12-31	2015-07-10
Diagnosis: ICD10 Code, F00-F99 Define	F01-F99 Mental, Behavioral and Neurodevelopmental disorders	489621	Cocaine-induced anxiety disorder with onset during intoxication, with delirium (HCC)	ICD10CM	1	F14.98	F14.980,F14.921	Cocaine use, unspecified with cocaine-induced anxiety d	IMO	GHS-PACDC	IMO_2015_R	ACTIVE	N	2016-09-17	9999-12-31	2016-09-30
Diagnosis: ICD10 Code, F00-F99 Define	F01-F99 Mental, Behavioral and Neurodevelopmental disorders	1182868	Cocaine use, unspecified with cocaine-induced sexual dysfunction (HCC)	ICD10CM	1	F14.981		Cocaine use, unspecified with cocaine-induced sexual dy	IMO	GHS-PACDC	IMO_2016_R	ACTIVE	N	1900-01-01	9999-12-31	2016-06-02
Diagnosis: ICD10 Code, F00-F99 Define	F01-F99 Mental, Behavioral and Neurodevelopmental disorders	486731	Cocaine-induced sleep disorder with mild use disorder, daytime sleepiness type (HCC)	ICD10CM	1	F14.982		Cocaine use, unspecified with cocaine-induced sleep dis	IMO	GHS-PACDC	IMO_2015_R	ACTIVE	N	1900-01-01	2016-10-14	2015-07-10
Diagnosis: ICD10 Code, F00-F99 Define	F01-F99 Mental, Behavioral and Neurodevelopmental disorders	488752	Onset of cocaine-induced sleep disorder, mixed type, during intoxication (HCC)	ICD10CM	1	F14.982		Cocaine use, unspecified with cocaine-induced sleep dis	IMO	GHS-PACDC	IMO_2015_R	ACTIVE	N	1900-01-01	9999-12-31	2015-07-10
Diagnosis: ICD10 Code, F00-F99 Define	F01-F99 Mental, Behavioral and Neurodevelopmental disorders	488733	Cocaine induced obsessive-compulsive disorder with onset after medication use (HCC)	ICD10CM	1	F14.98	F14.988,F42.9	Cocaine use, unspecified with other cocaine-induced dis	IMO	GHS-PACDC	IMO_2015_R	ACTIVE	N	2016-09-17	9999-12-31	2016-09-30
Diagnosis: ICD10 Code, F00-F99 Define	F01-F99 Mental, Behavioral and Neurodevelopmental disorders	489454	Onset of cocaine induced obsessive-compulsive disorder during intoxication (HCC)	ICD10CM	1	F14.98	F14.988,F42.9,F14.929	Cocaine use, unspecified with other cocaine-induced dis	IMO	GHS-PACDC	IMO_2015_R	ACTIVE	N	2016-09-17	9999-12-31	2016-09-30
Diagnosis: ICD10 Code, F00-F99 Define	F01-F99 Mental, Behavioral and Neurodevelopmental disorders	507855	Methylenedioxymethyamphetamine (MDMA) use disorder, mild, in controlled environment, ab	ICD10CM	1	F15.10		Other stimulant abuse, uncomplicated	IMO	GHS-PACDC	IMO_2015_R	ACTIVE	N	1900-01-01	9999-12-31	2015-07-10
Diagnosis: ICD10 Code, F00-F99 Define	F01-F99 Mental, Behavioral and Neurodevelopmental disorders	484377	Mild methamphetamine use disorder in controlled environment	ICD10CM	1	F15.10		Other stimulant abuse, uncomplicated	IMO	GHS-PACDC	IMO_2015_R	ACTIVE	N	2016-09-17	9999-12-31	2016-09-30
Diagnosis: ICD10 Code, F00-F99 Define	F01-F99 Mental, Behavioral and Neurodevelopmental disorders	484039	Mild Benzedrine use disorder	ICD10CM	1	F15.10		Other stimulant abuse, uncomplicated	IMO	GHS-PACDC	IMO_2015_R	ACTIVE	N	2016-09-17	9999-12-31	2016-09-30
Diagnosis: ICD10 Code, F00-F99 Define	F01-F99 Mental, Behavioral and Neurodevelopmental disorders	509475	Mild racemic amphetamine abuse in sustained remission in controlled environment	ICD10CM	1	F15.10		Other stimulant abuse, uncomplicated	IMO	GHS-PACDC	IMO_2015_R	ACTIVE	N	1900-01-01	2017-10-14	2015-07-10
Diagnosis: ICD10 Code, F00-F99 Define	F01-F99 Mental, Behavioral and Neurodevelopmental disorders	315876	Amphetamine and psychostimulant abuse, daily use	ICD10CM	1	F15.10		Other stimulant abuse, uncomplicated	IMO	GHS-PACDC	DX_LEGACY	ACTIVE	N	1900-01-01	9999-12-31	2015-07-10
Diagnosis: ICD10 Code, F00-F99 Define	F01-F99 Mental, Behavioral and Neurodevelopmental disorders	491993	Methylenedioxyamphetamine (MDA) use disorder, mild, in early remission	ICD10CM	1	F15.10		Other stimulant abuse, uncomplicated	IMO	GHS-PACDC	IMO_2015_R	ACTIVE	N	2016-09-17	9999-12-31	2016-09-30
Diagnosis: ICD10 Code, F00-F99 Define	F01-F99 Mental, Behavioral and Neurodevelopmental disorders	491876	Methylenedioxymethyamphetamine (MDMA) use disorder, mild, in early remission	ICD10CM	1	F15.10		Other stimulant abuse, uncomplicated	IMO	GHS-PACDC	IMO_2015_R	ACTIVE	N	2016-09-17	9999-12-31	2016-09-30
Diagnosis: ICD10 Code, F00-F99 Define	F01-F99 Mental, Behavioral and Neurodevelopmental disorders	507306	Mild methylenedioxymethamphetamine abuse in controlled environment	ICD10CM	1	F15.10		Other stimulant abuse, uncomplicated	IMO	GHS-PACDC	IMO_2015_R	ACTIVE	N	1900-01-01	9999-12-31	2015-07-10
Diagnosis: ICD10 Code, F00-F99 Define	F01-F99 Mental, Behavioral and Neurodevelopmental disorders	484300	Mild dextroamphetamine use disorder in sustained remission	ICD10CM	1	F15.10		Other stimulant abuse, uncomplicated	IMO	GHS-PACDC	IMO_2015_R	ACTIVE	N	2016-09-17	9999-12-31	2016-09-30
Diagnosis: ICD10 Code, F00-F99 Define	F01-F99 Mental, Behavioral and Neurodevelopmental disorders	505525	Amphetamine-type substance use disorder, mild, in early remission, in controlled enviroment	ICD10CM	1	F15.10		Other stimulant abuse, uncomplicated	IMO	GHS-PACDC	IMO_2015_R	ACTIVE	N	2016-09-17	9999-12-31	2016-09-30
Diagnosis: ICD10 Code, F00-F99 Define	F01-F99 Mental, Behavioral and Neurodevelopmental disorders	508051	Mild lisdexamfetamine dimesylate abuse in early remission in controlled environment	ICD10CM	1	F15.10		Other stimulant abuse, uncomplicated	IMO	GHS-PACDC	IMO_2015_R	ACTIVE	N	1900-01-01	2017-10-14	2015-07-10
Diagnosis: ICD10 Code, F00-F99 Define	F01-F99 Mental, Behavioral and Neurodevelopmental disorders	508721	Mild methylenedioxymethamphetamine (MDMA) abuse in sustained remission in controlled en	ICD10CM	1	F15.10		Other stimulant abuse, uncomplicated	IMO	GHS-PACDC	IMO_2015_R	ACTIVE	N	1900-01-01	2017-10-14	2015-07-10
Diagnosis: ICD10 Code, F00-F99 Define	F01-F99 Mental, Behavioral and Neurodevelopmental disorders	492013	Tenuate use disorder, mild, in early remission	ICD10CM	1	F15.10		Other stimulant abuse, uncomplicated	IMO	GHS-PACDC	IMO_2015_R	ACTIVE	N	2016-09-17	9999-12-31	2016-09-30
Diagnosis: ICD10 Code, F00-F99 Define	F01-F99 Mental, Behavioral and Neurodevelopmental disorders	488614	Amphetamine-type substance use disorder, mild, in controlled environment	ICD10CM	1	F15.10		Other stimulant abuse, uncomplicated	IMO	GHS-PACDC	IMO_2015_R	ACTIVE	N	2016-09-17	9999-12-31	2016-09-30
Diagnosis: ICD10 Code, F00-F99 Define	F01-F99 Mental, Behavioral and Neurodevelopmental disorders	484033	Mild methylenedioxymethamphetamine use disorder	ICD10CM	1	F15.10		Other stimulant abuse, uncomplicated	IMO	GHS-PACDC	IMO_2015_R	ACTIVE	N	2016-09-17	9999-12-31	2016-09-30
Diagnosis: ICD10 Code, F00-F99 Define	F01-F99 Mental, Behavioral and Neurodevelopmental disorders	491807	Adderall use disorder, mild, in sustained remission	ICD10CM	1	F15.10		Other stimulant abuse, uncomplicated	IMO	GHS-PACDC	IMO_2015_R	ACTIVE	N	2016-09-17	9999-12-31	2016-09-30
Diagnosis: ICD10 Code, F00-F99 Define	F01-F99 Mental, Behavioral and Neurodevelopmental disorders	507416	Khat use disorder, mild, in early remission, abuse	ICD10CM	1	F15.10		Other stimulant abuse, uncomplicated	IMO	GHS-PACDC	IMO_2015_R	ACTIVE	N	1900-01-01	2017-10-14	2015-07-10
Diagnosis: ICD10 Code, F00-F99 Define	F01-F99 Mental, Behavioral and Neurodevelopmental disorders	509883	Methamphetamine use disorder, mild, in controlled environment, abuse	ICD10CM	1	F15.10		Other stimulant abuse, uncomplicated	IMO	GHS-PACDC	IMO_2015_R	ACTIVE	N	1900-01-01	9999-12-31	2015-07-10
Diagnosis: ICD10 Code, F00-F99 Define	F01-F99 Mental, Behavioral and Neurodevelopmental disorders	426734	Intravenous amphetamine abuse without dependence	ICD10CM	1	F15.10		Other stimulant abuse, uncomplicated	IMO	GHS-PACDC	IMO_2015_R	ACTIVE	N	1900-01-01	9999-12-31	2015-07-10
Diagnosis: ICD10 Code, F00-F99 Define	F01-F99 Mental, Behavioral and Neurodevelopmental disorders	484511	Mild methylenedioxymethamphetamine use disorder in early remission	ICD10CM	1	F15.10		Other stimulant abuse, uncomplicated	IMO	GHS-PACDC	IMO_2015_R	ACTIVE	N	2016-09-17	9999-12-31	2016-09-30
Diagnosis: ICD10 Code, F00-F99 Define	F01-F99 Mental, Behavioral and Neurodevelopmental disorders	508854	Tenuate use disorder, mild, in sustained remission, in controlled environment, abuse	ICD10CM	1	F15.10		Other stimulant abuse, uncomplicated	IMO	GHS-PACDC	IMO_2015_R	ACTIVE	N	1900-01-01	2017-10-14	2015-07-10
Diagnosis: ICD10 Code, F00-F99 Define	F01-F99 Mental, Behavioral and Neurodevelopmental disorders	507013	Mild methedrine abuse in sustained remission	ICD10CM	1	F15.10		Other stimulant abuse, uncomplicated	IMO	GHS-PACDC	IMO_2015_R	ACTIVE	N	1900-01-01	2017-10-14	2015-07-10
Diagnosis: ICD10 Code, F00-F99 Define	F01-F99 Mental, Behavioral and Neurodevelopmental disorders	508698	Mild diethylpropion abuse	ICD10CM	1	F15.10		Other stimulant abuse, uncomplicated	IMO	GHS-PACDC	IMO_2015_R	ACTIVE	N	1900-01-01	9999-12-31	2015-07-10
Diagnosis: ICD10 Code, F00-F99 Define	F01-F99 Mental, Behavioral and Neurodevelopmental disorders	484208	Mild catha edulis use disorder in early remission	ICD10CM	1	F15.10		Other stimulant abuse, uncomplicated	IMO	GHS-PACDC	IMO_2015_R	ACTIVE	N	2016-09-17	9999-12-31	2016-09-30
Diagnosis: ICD10 Code, F00-F99 Define	F01-F99 Mental, Behavioral and Neurodevelopmental disorders	484919	Mild methylenedioxyamphetamine use disorder in early remission	ICD10CM	1	F15.10		Other stimulant abuse, uncomplicated	IMO	GHS-PACDC	IMO_2015_R	ACTIVE	N	2016-09-17	9999-12-31	2016-09-30
Diagnosis: ICD10 Code, F00-F99 Define	F01-F99 Mental, Behavioral and Neurodevelopmental disorders	508012	Methylenedioxymethyamphetamine (MDMA) use disorder, mild, in early remission, in controlle	ICD10CM	1	F15.11		Other stimulant abuse, in remission	IMO	GHS-PACDC	IMO_2015_R	ACTIVE	N	2017-09-17	9999-12-31	2017-10-30
Diagnosis: ICD10 Code, F00-F99 Define	F01-F99 Mental, Behavioral and Neurodevelopmental disorders	507617	Methylenedioxymethyamphetamine (MDMA) use disorder, mild, in sustained remission, in cont	ICD10CM	1	F15.11		Other stimulant abuse, in remission	IMO	GHS-PACDC	IMO_2015_R	ACTIVE	N	2017-09-17	9999-12-31	2017-10-30
Diagnosis: ICD10 Code, F00-F99 Define	F01-F99 Mental, Behavioral and Neurodevelopmental disorders	508282	Mild mixed dextroamphetamine-amphetamine abuse in sustained remission in controlled envir	ICD10CM	1	F15.11		Other stimulant abuse, in remission	IMO	GHS-PACDC	IMO_2015_R	ACTIVE	N	2017-09-17	9999-12-31	2017-10-30
Diagnosis: ICD10 Code, F00-F99 Define	F01-F99 Mental, Behavioral and Neurodevelopmental disorders	508466	Dexedrine use disorder, mild, in sustained remission, abuse	ICD10CM	1	F15.11		Other stimulant abuse, in remission	IMO	GHS-PACDC	IMO_2015_R	ACTIVE	N	2017-09-17	9999-12-31	2017-10-30
Diagnosis: ICD10 Code, F00-F99 Define	F01-F99 Mental, Behavioral and Neurodevelopmental disorders	509072	Adderall use disorder, mild, in early remission, in controlled environment, abuse	ICD10CM	1	F15.11		Other stimulant abuse, in remission	IMO	GHS-PACDC	IMO_2015_R	ACTIVE	N	2017-09-17	9999-12-31	2017-10-30
Diagnosis: ICD10 Code, F00-F99 Define	F01-F99 Mental, Behavioral and Neurodevelopmental disorders	1356129	Other stimulant abuse, in remission	ICD10CM	1	F15.11		Other stimulant abuse, in remission	IMO	GHS-PACDC	IMO_2018_R	ACTIVE	Y	1900-01-01	9999-12-31	2017-10-30
Diagnosis: ICD10 Code, F00-F99 Define	F01-F99 Mental, Behavioral and Neurodevelopmental disorders	509561	Mild methylenedioxymethamphetamine (MDMA) abuse in early remission in controlled enviror	ICD10CM	1	F15.11		Other stimulant abuse, in remission	IMO	GHS-PACDC	IMO_2015_R	ACTIVE	N	2017-09-17	9999-12-31	2017-10-30
Diagnosis: ICD10 Code, F00-F99 Define	F01-F99 Mental, Behavioral and Neurodevelopmental disorders	507113	Vyvanse use disorder, mild, in sustained remission, in controlled environment, abuse	ICD10CM	1	F15.11		Other stimulant abuse, in remission	IMO	GHS-PACDC	IMO_2015_R	ACTIVE	N	2017-09-17	9999-12-31	2017-10-30
Diagnosis: ICD10 Code, F00-F99 Define	F01-F99 Mental, Behavioral and Neurodevelopmental disorders	507623	Adderall use disorder, mild, in early remission, abuse	ICD10CM	1	F15.11		Other stimulant abuse, in remission	IMO	GHS-PACDC	IMO_2015_R	ACTIVE	N	2017-09-17	9999-12-31	2017-10-30
Diagnosis: ICD10 Code, F00-F99 Define	F01-F99 Mental, Behavioral and Neurodevelopmental disorders	508613	Mild methylphenidate abuse in sustained remission	ICD10CM	1	F15.11		Other stimulant abuse, in remission	IMO	GHS-PACDC	IMO_2015_R	ACTIVE	N	2017-09-17	9999-12-31	2017-10-30
Diagnosis: ICD10 Code, F00-F99 Define	F01-F99 Mental, Behavioral and Neurodevelopmental disorders	509066	Khat use disorder, mild, in sustained remission, in controlled environment, abuse	ICD10CM	1	F15.11		Other stimulant abuse, in remission	IMO	GHS-PACDC	IMO_2015_R	ACTIVE	N	2017-09-17	9999-12-31	2017-10-30
Diagnosis: ICD10 Code, F00-F99 Define	F01-F99 Mental, Behavioral and Neurodevelopmental disorders	966646	Onset of caffeine-induced sleep disorder during intoxication, uncomplicated (HCC)	ICD10CM	1	F15.12	F15.120,F15.182	Other stimulant abuse with intoxication, uncomplicated	IMO	GHS-PACDC	IMO_2015_R	ACTIVE	N	1900-01-01	2017-10-14	2015-07-10
Diagnosis: ICD10 Code, F00-F99 Define	F01-F99 Mental, Behavioral and Neurodevelopmental disorders	1028295	Continuous caffeine intoxication with complication, uncomplicated	ICD10CM	1	F15.120		Other stimulant abuse with intoxication, uncomplicated	IMO	GHS-PACDC	IMO_2015_R	DELETED	Y	1900-01-01	9999-12-31	2015-07-10
Diagnosis: ICD10 Code, F00-F99 Define	F01-F99 Mental, Behavioral and Neurodevelopmental disorders	976283	Caffeine-induced anxiety disorder with onset during intoxication, uncomplicated (HCC)	ICD10CM	1	F15.12	F15.120,F15.90	Other stimulant abuse with intoxication, uncomplicated	IMO	GHS-PACDC	IMO_2015_R	ACTIVE	N	2016-04-10	9999-12-31	2015-07-10
Diagnosis: ICD10 Code, F00-F99 Define	F01-F99 Mental, Behavioral and Neurodevelopmental disorders	516855	Onset of amphetamine-induced sleep disorder during intoxication without complication (HCC)	ICD10CM	2	F15.12	F15.182,F15.120	Other stimulant abuse with intoxication, uncomplicated	IMO	GHS-PACDC	IMO_2015_R	ACTIVE	N	1900-01-01	2017-10-14	2015-07-10
Diagnosis: ICD10 Code, F00-F99 Define	F01-F99 Mental, Behavioral and Neurodevelopmental disorders	476379	Episodic caffeine intoxication with perceptual disturbance (HCC)	ICD10CM	1	F15.122		Other stimulant abuse with intoxication with perceptual	IMO	GHS-PACDC	IMO_2015_R	ACTIVE	N	1900-01-01	2017-10-14	2015-07-10
Diagnosis: ICD10 Code, F00-F99 Define	F01-F99 Mental, Behavioral and Neurodevelopmental disorders	963688	Continuous caffeine intoxication, with perceptual disturbance (HCC)	ICD10CM	1	F15.122		Other stimulant abuse with intoxication with perceptual	IMO	GHS-PACDC	IMO_2015_R	ACTIVE	N	1900-01-01	2017-10-14	2015-07-10
Diagnosis: ICD10 Code, F00-F99 Define	F01-F99 Mental, Behavioral and Neurodevelopmental disorders	974413	Caffeine-induced anxiety disorder with onset during intoxication, with unspecified complication	ICD10CM	1	F15.12	F15.129,F15.929	Other stimulant abuse with intoxication, unspecified	IMO	GHS-PACDC	IMO_2015_R	ACTIVE	N	2016-04-10	9999-12-31	2015-07-10
Diagnosis: ICD10 Code, F00-F99 Define	F01-F99 Mental, Behavioral and Neurodevelopmental disorders	508783	Tenuate use disorder, severe, dependence (HCC)	ICD10CM	1	F15.20		Other stimulant dependence, uncomplicated	IMO	GHS-PACDC	IMO_2015_R	ACTIVE	N	1900-01-01	9999-12-31	2015-07-10
Diagnosis: ICD10 Code, F00-F99 Define	F01-F99 Mental, Behavioral and Neurodevelopmental disorders	484043	Severe diethylpropion use disorder (HCC)	ICD10CM	1	F15.20		Other stimulant dependence, uncomplicated	IMO	GHS-PACDC	IMO_2015_R	ACTIVE	N	2016-09-17	9999-12-31	2016-09-30
Diagnosis: ICD10 Code, F00-F99 Define	F01-F99 Mental, Behavioral and Neurodevelopmental disorders	505535	Amphetamine-type substance use disorder, moderate, in sustained remission, in controlled env	ICD10CM	1	F15.20		Other stimulant dependence, uncomplicated	IMO	GHS-PACDC	IMO_2015_R	ACTIVE	N	2016-09-17	9999-12-31	2016-09-30
Diagnosis: ICD10 Code, F00-F99 Define	F01-F99 Mental, Behavioral and Neurodevelopmental disorders	484736	Moderate amphetamine substance use disorder in early remission (HCC)	ICD10CM	1	F15.20		Other stimulant dependence, uncomplicated	IMO	GHS-PACDC	IMO_2015_R	ACTIVE	N	2016-09-17	9999-12-31	2016-09-30
Diagnosis: ICD10 Code, F00-F99 Define	F01-F99 Mental, Behavioral and Neurodevelopmental disorders	492039	Amphetamine use disorder, moderate, in controlled environment (HCC)	ICD10CM	1	F15.20		Other stimulant dependence, uncomplicated	IMO	GHS-PACDC	IMO_2015_R	ACTIVE	N	2016-09-17	9999-12-31	2016-09-30
Diagnosis: ICD10 Code, F00-F99 Define	F01-F99 Mental, Behavioral and Neurodevelopmental disorders	492192	Moderate Benzedrine use disorder, in controlled environment (HCC)	ICD10CM	1	F15.20		Other stimulant dependence, uncomplicated	IMO	GHS-PACDC	IMO_2015_R	ACTIVE	N	2016-09-17	9999-12-31	2016-09-30
Diagnosis: ICD10 Code, F00-F99 Define	F01-F99 Mental, Behavioral and Neurodevelopmental disorders	507003	Ritalin use disorder, moderate, dependence (HCC)	ICD10CM	1	F15.20		Other stimulant dependence, uncomplicated	IMO	GHS-PACDC	IMO_2015_R	ACTIVE	N	1900-01-01	9999-12-31	2015-07-10
Diagnosis: ICD10 Code, F00-F99 Define	F01-F99 Mental, Behavioral and Neurodevelopmental disorders	509088	Khat use disorder, severe, dependence (HCC)	ICD10CM	1	F15.20		Other stimulant dependence, uncomplicated	IMO	GHS-PACDC	IMO_2015_R	ACTIVE	N	1900-01-01	9999-12-31	2015-07-10
Diagnosis: ICD10 Code, F00-F99 Define	F01-F99 Mental, Behavioral and Neurodevelopmental disorders	212588	Amphetamine dependence, continuous (HCC)	ICD10CM	1	F15.20		Other stimulant dependence, uncomplicated	IMO	GHS-PACDC	DX_LEGACY	ACTIVE	N	2017-09-17	9999-12-31	2017-10-30
Diagnosis: ICD10 Code, F00-F99 Define	F01-F99 Mental, Behavioral and Neurodevelopmental disorders	428826	Amphetamine dependence with or without physiological dependence (HCC)	ICD10CM	1	F15.20		Other stimulant dependence, uncomplicated	IMO	GHS-PACDC	IMO_2015_R	ACTIVE	N	1900-01-01	9999-12-31	2015-07-10
Diagnosis: ICD10 Code, F00-F99 Define	F01-F99 Mental, Behavioral and Neurodevelopmental disorders	479887	Severe methylenedioxyamphetamine use disorder (HCC)	ICD10CM	1	F15.20		Other stimulant dependence, uncomplicated	IMO	GHS-PACDC	IMO_2015_R	ACTIVE	N	2016-09-17	9999-12-31	2016-09-30
Diagnosis: ICD10 Code, F00-F99 Define	F01-F99 Mental, Behavioral and Neurodevelopmental disorders	507834	Dexedrine use disorder, severe, dependence (HCC)	ICD10CM	1	F15.20		Other stimulant dependence, uncomplicated	IMO	GHS-PACDC	IMO_2015_R	ACTIVE	N	1900-01-01	9999-12-31	2015-07-10
Diagnosis: ICD10 Code, F00-F99 Define	F01-F99 Mental, Behavioral and Neurodevelopmental disorders	1178418	Other stimulant dependence, uncomplicated (HCC)	ICD10CM	1	F15.20		Other stimulant dependence, uncomplicated	IMO	GHS-PACDC	IMO_2016_R	ACTIVE	N	1900-01-01	9999-12-31	2016-06-02
Diagnosis: ICD10 Code, F00-F99 Define	F01-F99 Mental, Behavioral and Neurodevelopmental disorders	508322	Amphetamine use disorder, moderate, in controlled environment, dependence (HCC)	ICD10CM	1	F15.20		Other stimulant dependence, uncomplicated	IMO	GHS-PACDC	IMO_2015_R	ACTIVE	N	1900-01-01	9999-12-31	2015-07-10
Diagnosis: ICD10 Code, F00-F99 Define	F01-F99 Mental, Behavioral and Neurodevelopmental disorders	510006	Moderate mixed dextroamphetamine-amphetamine dependence (HCC)	ICD10CM	1	F15.20		Other stimulant dependence, uncomplicated	IMO	GHS-PACDC	IMO_2015_R	ACTIVE	N	1900-01-01	9999-12-31	2015-07-10
Diagnosis: ICD10 Code, F00-F99 Define	F01-F99 Mental, Behavioral and Neurodevelopmental disorders	484539	Moderate racemic amphetamine use disorder in controlled environment (HCC)	ICD10CM	1	F15.20		Other stimulant dependence, uncomplicated	IMO	GHS-PACDC	IMO_2015_R	ACTIVE	N	2016-09-17	9999-12-31	2016-09-30
Diagnosis: ICD10 Code, F00-F99 Define	F01-F99 Mental, Behavioral and Neurodevelopmental disorders	484278	Moderate dextroamphetamine use disorder in controlled environment (HCC)	ICD10CM	1	F15.20		Other stimulant dependence, uncomplicated	IMO	GHS-PACDC	IMO_2015_R	ACTIVE	N	2016-09-17	9999-12-31	2016-09-30
Diagnosis: ICD10 Code, F00-F99 Define	F01-F99 Mental, Behavioral and Neurodevelopmental disorders	212310	Continuous amphetamine dependence (HCC)	ICD10CM	1	F15.20		Other stimulant dependence, uncomplicated	IMO	GHS-PACDC	DX_LEGACY	ACTIVE	N	2017-09-17	9999-12-31	2017-10-30
Diagnosis: ICD10 Code, F00-F99 Define	F01-F99 Mental, Behavioral and Neurodevelopmental disorders	507587	Benzedrine use disorder, moderate, in early remission, in controlled environment, dependence	ICD10CM	1	F15.20		Other stimulant dependence, uncomplicated	IMO	GHS-PACDC	IMO_2015_R	ACTIVE	N	1900-01-01	9999-12-31	2015-07-10
Diagnosis: ICD10 Code, F00-F99 Define	F01-F99 Mental, Behavioral and Neurodevelopmental disorders	484908	Moderate methylphenidate use disorder in controlled environment (HCC)	ICD10CM	1	F15.20		Other stimulant dependence, uncomplicated	IMO	GHS-PACDC	IMO_2015_R	ACTIVE	N	2016-09-17	9999-12-31	2016-09-30
Diagnosis: ICD10 Code, F00-F99 Define	F01-F99 Mental, Behavioral and Neurodevelopmental disorders	484426	Severe methylenedioxyamphetamine use disorder in early remission (HCC)	ICD10CM	1	F15.21		Other stimulant dependence, in remission	IMO	GHS-PACDC	IMO_2015_R	ACTIVE	N	2016-09-17	9999-12-31	2016-09-30
Diagnosis: ICD10 Code, F00-F99 Define	F01-F99 Mental, Behavioral and Neurodevelopmental disorders	484705	Severe diethylpropion use disorder in sustained remission (HCC)	ICD10CM	1	F15.21		Other stimulant dependence, in remission	IMO	GHS-PACDC	IMO_2015_R	ACTIVE	N	2016-09-17	9999-12-31	2016-09-30
Diagnosis: ICD10 Code, F00-F99 Define	F01-F99 Mental, Behavioral and Neurodevelopmental disorders	508605	Severe methylphenidate dependence in sustained remission (HCC)	ICD10CM	1	F15.21		Other stimulant dependence, in remission	IMO	GHS-PACDC	IMO_2015_R	ACTIVE	N	1900-01-01	9999-12-31	2015-07-10
Diagnosis: ICD10 Code, F00-F99 Define	F01-F99 Mental, Behavioral and Neurodevelopmental disorders	484847	Moderate methylphenidate use disorder in early remission (HCC)	ICD10CM	1	F15.21		Other stimulant dependence, in remission	IMO	GHS-PACDC	IMO_2015_R	ACTIVE	N	2016-09-17	9999-12-31	2016-09-30
Diagnosis: ICD10 Code, F00-F99 Define	F01-F99 Mental, Behavioral and Neurodevelopmental disorders	509544	Tenuate use disorder, moderate, in sustained remission, in controlled environment, dependenc	ICD10CM	1	F15.21		Other stimulant dependence, in remission	IMO	GHS-PACDC	IMO_2015_R	ACTIVE	N	1900-01-01	9999-12-31	2015-07-10
Diagnosis: ICD10 Code, F00-F99 Define	F01-F99 Mental, Behavioral and Neurodevelopmental disorders	484974	Ritalin use disorder, severe, in sustained remission (HCC)	ICD10CM	1	F15.21		Other stimulant dependence, in remission	IMO	GHS-PACDC	IMO_2015_R	ACTIVE	N	2016-09-17	9999-12-31	2016-09-30
Diagnosis: ICD10 Code, F00-F99 Define	F01-F99 Mental, Behavioral and Neurodevelopmental disorders	508732	Moderate dextroamphetamine-amphetamine dependence in sustained remission in controlled	ICD10CM	1	F15.21		Other stimulant dependence, in remission	IMO	GHS-PACDC	IMO_2015_R	ACTIVE	N	1900-01-01	9999-12-31	2015-07-10
Diagnosis: ICD10 Code, F00-F99 Define	F01-F99 Mental, Behavioral and Neurodevelopmental disorders	507189	Moderate lisdexamfetamine dimesylate dependence in sustained remission (HCC)	ICD10CM	1	F15.21		Other stimulant dependence, in remission	IMO	GHS-PACDC	IMO_2015_R	ACTIVE	N	1900-01-01	9999-12-31	2015-07-10
Diagnosis: ICD10 Code, F00-F99 Define	F01-F99 Mental, Behavioral and Neurodevelopmental disorders	507512	Severe racemic amphetamine dependence in sustained remission in controlled environment (H	ICD10CM	1	F15.21		Other stimulant dependence, in remission	IMO	GHS-PACDC	IMO_2015_R	ACTIVE	N	1900-01-01	9999-12-31	2015-07-10
Diagnosis: ICD10 Code, F00-F99 Define	F01-F99 Mental, Behavioral and Neurodevelopmental disorders	484550	Severe methylenedioxyamphetamine use disorder in sustained remission (HCC)	ICD10CM	1	F15.21		Other stimulant dependence, in remission	IMO	GHS-PACDC	IMO_2015_R	ACTIVE	N	2016-09-17	9999-12-31	2016-09-30
Diagnosis: ICD10 Code, F00-F99 Define	F01-F99 Mental, Behavioral and Neurodevelopmental disorders	509584	Severe diethylpropion dependence in early remission (HCC)	ICD10CM	1	F15.21		Other stimulant dependence, in remission	IMO	GHS-PACDC	IMO_2015_R	ACTIVE	N	1900-01-01	9999-12-31	2015-07-10
Diagnosis: ICD10 Code, F00-F99 Define	F01-F99 Mental, Behavioral and Neurodevelopmental disorders	509315	Severe diethylpropion dependence in sustained remission (HCC)	ICD10CM	1	F15.21		Other stimulant dependence, in remission	IMO	GHS-PACDC	IMO_2015_R	ACTIVE	N	1900-01-01	9999-12-31	2015-07-10
Diagnosis: ICD10 Code, F00-F99 Define	F01-F99 Mental, Behavioral and Neurodevelopmental disorders	507004	Moderate racemic amphetamine dependence in sustained remission in controlled environment	ICD10CM	1	F15.21		Other stimulant dependence, in remission	IMO	GHS-PACDC	IMO_2015_R	ACTIVE	N	1900-01-01	9999-12-31	2015-07-10
Diagnosis: ICD10 Code, F00-F99 Define	F01-F99 Mental, Behavioral and Neurodevelopmental disorders	509972	Moderate amphetamine substance dependence in early remission in controlled environment (H	ICD10CM	1	F15.21		Other stimulant dependence, in remission	IMO	GHS-PACDC	IMO_2015_R	ACTIVE	N	1900-01-01	9999-12-31	2015-07-10

Diagnosis: ICD10 Code, F00-F99 Define	F01-F99 Mental, Behavioral and Neurodevelopmental disorders	484424	Severe khat use disorder in sustained remission (HCC)	ICD10CM	1	F15.21		Other stimulant dependence, in remission	IMO	GHS-PACDC	IMO_2015_R	ACTIVE	N	2016-09-17	9999-12-31	2016-09-30
Diagnosis: ICD10 Code, F00-F99 Define	F01-F99 Mental, Behavioral and Neurodevelopmental disorders	484945	Severe lisdexamfetamine dimesylate use disorder in early remission (HCC)	ICD10CM	1	F15.21		Other stimulant dependence, in remission	IMO	GHS-PACDC	IMO_2015_R	ACTIVE	N	2016-09-17	9999-12-31	2016-09-30
Diagnosis: ICD10 Code, F00-F99 Define	F01-F99 Mental, Behavioral and Neurodevelopmental disorders	484828	Ecstasy use disorder, severe, in sustained remission (HCC)	ICD10CM	1	F15.21		Other stimulant dependence, in remission	IMO	GHS-PACDC	IMO_2015_R	ACTIVE	N	2016-09-17	9999-12-31	2016-09-30
Diagnosis: ICD10 Code, F00-F99 Define	F01-F99 Mental, Behavioral and Neurodevelopmental disorders	507732	Severe mixed dextroamphetamine-amphetamine dependence in early remission (HCC)	ICD10CM	1	F15.21		Other stimulant dependence, in remission	IMO	GHS-PACDC	IMO_2015_R	ACTIVE	N	1900-01-01	9999-12-31	2015-07-10
Diagnosis: ICD10 Code, F00-F99 Define	F01-F99 Mental, Behavioral and Neurodevelopmental disorders	507317	Severe methamphetamine dependence in sustained remission in controlled environment (HCC)	ICD10CM	1	F15.21		Other stimulant dependence, in remission	IMO	GHS-PACDC	IMO_2015_R	ACTIVE	N	1900-01-01	9999-12-31	2015-07-10
Diagnosis: ICD10 Code, F00-F99 Define	F01-F99 Mental, Behavioral and Neurodevelopmental disorders	508060	Moderate methylenedioxyamphetamine dependence in sustained remission (HCC)	ICD10CM	1	F15.21		Other stimulant dependence, in remission	IMO	GHS-PACDC	IMO_2015_R	ACTIVE	N	1900-01-01	9999-12-31	2015-07-10
Diagnosis: ICD10 Code, F00-F99 Define	F01-F99 Mental, Behavioral and Neurodevelopmental disorders	633312	Other stimulant dependence with intoxication, unspecified (HCC)	ICD10CM	1	F15.229		Other stimulant dependence with intoxication, unspecifi	IMO	GHS-PACDC	IMO_2015_R	ACTIVE	N	1900-01-01	9999-12-31	2015-07-10
Diagnosis: ICD10 Code, F00-F99 Define	F01-F99 Mental, Behavioral and Neurodevelopmental disorders	516459	Onset of amphetamine-induced mood disorder during withdrawal with delirium (HCC)	ICD10CM	2	F15.23	F15.24,F15.23,F15.288	Other stimulant dependence with withdrawal	IMO	GHS-PACDC	IMO_2015_R	ACTIVE	N	1900-01-01	2017-10-14	2015-07-10
Diagnosis: ICD10 Code, F00-F99 Define	F01-F99 Mental, Behavioral and Neurodevelopmental disorders	1178419	Other stimulant dependence with stimulant-induced sleep disorder (HCC)	ICD10CM	1	F15.282		Other stimulant dependence with stimulant-induced sle	IMO	GHS-PACDC	IMO_2016_R	ACTIVE	N	1900-01-01	9999-12-31	2016-06-02
Diagnosis: ICD10 Code, F00-F99 Define	F01-F99 Mental, Behavioral and Neurodevelopmental disorders	484114	Moderate khat use disorder (HCC)	ICD10CM	1	F15.29		Other stimulant dependence with unspecified stimulant	IMO	GHS-PACDC	IMO_2015_R	ACTIVE	N	1900-01-01	2016-10-14	2015-07-10
Diagnosis: ICD10 Code, F00-F99 Define	F01-F99 Mental, Behavioral and Neurodevelopmental disorders	483926	Khat use disorder, severe (HCC)	ICD10CM	1	F15.29		Other stimulant dependence with unspecified stimulant	IMO	GHS-PACDC	IMO_2015_R	ACTIVE	N	1900-01-01	2016-10-14	2015-07-10
Diagnosis: ICD10 Code, F00-F99 Define	F01-F99 Mental, Behavioral and Neurodevelopmental disorders	484832	Moderate dextroamphetamine use disorder in sustained remission (HCC)	ICD10CM	1	F15.29		Other stimulant dependence with unspecified stimulant	IMO	GHS-PACDC	IMO_2015_R	ACTIVE	N	1900-01-01	2016-10-14	2015-07-10
Diagnosis: ICD10 Code, F00-F99 Define	F01-F99 Mental, Behavioral and Neurodevelopmental disorders	483918	Methadrine use disorder, mild	ICD10CM	1	F15.90		Other stimulant use, unspecified, uncomplicated	IMO	GHS-PACDC	IMO_2015_R	ACTIVE	N	1900-01-01	2016-10-14	2015-07-10
Diagnosis: ICD10 Code, F00-F99 Define	F01-F99 Mental, Behavioral and Neurodevelopmental disorders	491757	Mild Benzedrine use disorder, in controlled environment	ICD10CM	1	F15.90		Other stimulant use, unspecified, uncomplicated	IMO	GHS-PACDC	IMO_2015_R	ACTIVE	N	1900-01-01	2016-10-14	2015-07-10
Diagnosis: ICD10 Code, F00-F99 Define	F01-F99 Mental, Behavioral and Neurodevelopmental disorders	488478	Amphetamine substance use disorder, severe, in controlled environment (HCC)	ICD10CM	1	F15.90		Other stimulant use, unspecified, uncomplicated	IMO	GHS-PACDC	IMO_2015_R	ACTIVE	N	1900-01-01	2016-10-14	2015-07-10
Diagnosis: ICD10 Code, F00-F99 Define	F01-F99 Mental, Behavioral and Neurodevelopmental disorders	505526	Amphetamine-type substance use disorder, moderate, in early remission, in controlled envirom	ICD10CM	1	F15.90		Other stimulant use, unspecified, uncomplicated	IMO	GHS-PACDC	IMO_2015_R	ACTIVE	N	1900-01-01	2016-10-14	2015-07-10
Diagnosis: ICD10 Code, F00-F99 Define	F01-F99 Mental, Behavioral and Neurodevelopmental disorders	429203	Stimulant use disorder	ICD10CM	1	F15.90		Other stimulant use, unspecified, uncomplicated	IMO	GHS-PACDC	IMO_2015_R	ACTIVE	N	2016-09-17	9999-12-31	2016-09-30
Diagnosis: ICD10 Code, F00-F99 Define	F01-F99 Mental, Behavioral and Neurodevelopmental disorders	484746	Severe methedrine use disorder in early remission (HCC)	ICD10CM	1	F15.90		Other stimulant use, unspecified, uncomplicated	IMO	GHS-PACDC	IMO_2015_R	ACTIVE	N	1900-01-01	2016-10-14	2015-07-10
Diagnosis: ICD10 Code, F00-F99 Define	F01-F99 Mental, Behavioral and Neurodevelopmental disorders	484421	Mild methylphenidate use disorder in controlled environment	ICD10CM	1	F15.90		Other stimulant use, unspecified, uncomplicated	IMO	GHS-PACDC	IMO_2015_R	ACTIVE	N	1900-01-01	2016-10-14	2015-07-10
Diagnosis: ICD10 Code, F00-F99 Define	F01-F99 Mental, Behavioral and Neurodevelopmental disorders	491775	Amphetamine-type substance use disorder, severe, in early remission (HCC)	ICD10CM	1	F15.90		Other stimulant use, unspecified, uncomplicated	IMO	GHS-PACDC	IMO_2015_R	ACTIVE	N	1900-01-01	2016-10-14	2015-07-10
Diagnosis: ICD10 Code, F00-F99 Define	F01-F99 Mental, Behavioral and Neurodevelopmental disorders	484852	Severe amphetamine substance use disorder in sustained remission (HCC)	ICD10CM	1	F15.90		Other stimulant use, unspecified, uncomplicated	IMO	GHS-PACDC	IMO_2015_R	ACTIVE	N	1900-01-01	2016-10-14	2015-07-10
Diagnosis: ICD10 Code, F00-F99 Define	F01-F99 Mental, Behavioral and Neurodevelopmental disorders	505535	Amphetamine-type substance use disorder, moderate, in sustained remission, in controlled env	ICD10CM	1	F15.90		Other stimulant use, unspecified, uncomplicated	IMO	GHS-PACDC	IMO_2015_R	ACTIVE	N	1900-01-01	2016-10-14	2015-07-10
Diagnosis: ICD10 Code, F00-F99 Define	F01-F99 Mental, Behavioral and Neurodevelopmental disorders	488546	Amphetamine-type substance use disorder, mild, in early remission	ICD10CM	1	F15.90		Other stimulant use, unspecified, uncomplicated	IMO	GHS-PACDC	IMO_2015_R	ACTIVE	N	1900-01-01	2016-10-14	2015-07-10
Diagnosis: ICD10 Code, F00-F99 Define	F01-F99 Mental, Behavioral and Neurodevelopmental disorders	484717	Mild lisdexamfetamine dimesylate use disorder in sustained remission	ICD10CM	1	F15.90		Other stimulant use, unspecified, uncomplicated	IMO	GHS-PACDC	IMO_2015_R	ACTIVE	N	1900-01-01	2016-10-14	2015-07-10
Diagnosis: ICD10 Code, F00-F99 Define	F01-F99 Mental, Behavioral and Neurodevelopmental disorders	483984	Moderate racemic amphetamine use disorder (HCC)	ICD10CM	1	F15.90		Other stimulant use, unspecified, uncomplicated	IMO	GHS-PACDC	IMO_2015_R	ACTIVE	N	1900-01-01	2016-10-14	2015-07-10
Diagnosis: ICD10 Code, F00-F99 Define	F01-F99 Mental, Behavioral and Neurodevelopmental disorders	484997	Ecstasy use disorder, mild, in sustained remission	ICD10CM	1	F15.90		Other stimulant use, unspecified, uncomplicated	IMO	GHS-PACDC	IMO_2015_R	ACTIVE	N	1900-01-01	2016-10-14	2015-07-10
Diagnosis: ICD10 Code, F00-F99 Define	F01-F99 Mental, Behavioral and Neurodevelopmental disorders	492172	Mild Benzedrine use disorder, in early remission	ICD10CM	1	F15.90		Other stimulant use, unspecified, uncomplicated	IMO	GHS-PACDC	IMO_2015_R	ACTIVE	N	1900-01-01	2016-10-14	2015-07-10
Diagnosis: ICD10 Code, F00-F99 Define	F01-F99 Mental, Behavioral and Neurodevelopmental disorders	492035	Methedrine use disorder, moderate, in sustained remission (HCC)	ICD10CM	1	F15.90		Other stimulant use, unspecified, uncomplicated	IMO	GHS-PACDC	IMO_2015_R	ACTIVE	N	1900-01-01	2016-10-14	2015-07-10
Diagnosis: ICD10 Code, F00-F99 Define	F01-F99 Mental, Behavioral and Neurodevelopmental disorders	484300	Mild dextroamphetamine use disorder in sustained remission	ICD10CM	1	F15.90		Other stimulant use, unspecified, uncomplicated	IMO	GHS-PACDC	IMO_2015_R	ACTIVE	N	1900-01-01	2016-10-14	2015-07-10
Diagnosis: ICD10 Code, F00-F99 Define	F01-F99 Mental, Behavioral and Neurodevelopmental disorders	484391	Mild methedrine use disorder in sustained remission	ICD10CM	1	F15.90		Other stimulant use, unspecified, uncomplicated	IMO	GHS-PACDC	IMO_2015_R	ACTIVE	N	1900-01-01	2016-10-14	2015-07-10
Diagnosis: ICD10 Code, F00-F99 Define	F01-F99 Mental, Behavioral and Neurodevelopmental disorders	484620	Moderate mixed dextroamphetamine-amphetamine use disorder in sustained remission (HCC)	ICD10CM	1	F15.90		Other stimulant use, unspecified, uncomplicated	IMO	GHS-PACDC	IMO_2015_R	ACTIVE	N	1900-01-01	2016-10-14	2015-07-10
Diagnosis: ICD10 Code, F00-F99 Define	F01-F99 Mental, Behavioral and Neurodevelopmental disorders	484223	Methylenedioxyamphetamine (MDA) use disorder, moderate, in early remission (HCC)	ICD10CM	1	F15.90		Other stimulant use, unspecified, uncomplicated	IMO	GHS-PACDC	IMO_2015_R	ACTIVE	N	1900-01-01	2016-10-14	2015-07-10
Diagnosis: ICD10 Code, F00-F99 Define	F01-F99 Mental, Behavioral and Neurodevelopmental disorders	612479	Other stimulant use, unspecified with intoxication	ICD10CM	1	F15.92		Other stimulant use, unspecified with intoxication	IMO	GHS-PACDC	IMO_2015_R	ACTIVE	Y	1900-01-01	9999-12-31	2015-07-10
Diagnosis: ICD10 Code, F00-F99 Define	F01-F99 Mental, Behavioral and Neurodevelopmental disorders	958547	Amphetamine intoxication, uncomplicated (HCC)	ICD10CM	1	F15.920		Other stimulant use, unspecified with intoxication, unco	IMO	GHS-PACDC	IMO_2015_R	ACTIVE	N	2017-09-17	9999-12-31	2017-10-30
Diagnosis: ICD10 Code, F00-F99 Define	F01-F99 Mental, Behavioral and Neurodevelopmental disorders	959448	Caffeine intoxication, continuous, uncomplicated (HCC)	ICD10CM	1	F15.920		Other stimulant use, unspecified with intoxication, unco	IMO	GHS-PACDC	IMO_2015_R	ACTIVE	N	2017-09-17	9999-12-31	2017-10-30
Diagnosis: ICD10 Code, F00-F99 Define	F01-F99 Mental, Behavioral and Neurodevelopmental disorders	967161	Onset of amphetamine-induced mood disorder during intoxication, with delirium (HCC)	ICD10CM	2	F15.921	F19.121,F15.921	Other stimulant use, unspecified with intoxication deliri	IMO	GHS-PACDC	IMO_2015_R	ACTIVE	N	2016-04-10	2017-10-14	2015-07-10
Diagnosis: ICD10 Code, F00-F99 Define	F01-F99 Mental, Behavioral and Neurodevelopmental disorders	517494	Caffeine-induced anxiety disorder with onset during intoxication with delirium (HCC)	ICD10CM	1	F15.921	F15.921,F15.980	Other stimulant use, unspecified with intoxication deliri	IMO	GHS-PACDC	IMO_2015_R	ACTIVE	N	2017-09-17	9999-12-31	2017-10-30
Diagnosis: ICD10 Code, F00-F99 Define	F01-F99 Mental, Behavioral and Neurodevelopmental disorders	980178	Continuous caffeine intoxication with complication, with perceptual disturbance (HCC)	ICD10CM	1	F15.922		Other stimulant use, unspecified with intoxication with p	IMO	GHS-PACDC	IMO_2015_R	ACTIVE	N	2016-09-17	9999-12-31	2016-09-30
Diagnosis: ICD10 Code, F00-F99 Define	F01-F99 Mental, Behavioral and Neurodevelopmental disorders	516915	Onset of amphetamine-induced sexual dysfunction during intoxication with perceptual disturba	ICD10CM	1	F15.922	F15.922,F15.981	Other stimulant use, unspecified with intoxication with p	IMO	GHS-PACDC	IMO_2015_R	ACTIVE	N	2017-09-17	9999-12-31	2017-10-30
Diagnosis: ICD10 Code, F00-F99 Define	F01-F99 Mental, Behavioral and Neurodevelopmental disorders	965414	Onset of amphetamine-induced sleep disorder during intoxication, with perceptual disturbance	ICD10CM	1	F15.922	F15.922,F15.982	Other stimulant use, unspecified with intoxication with p	IMO	GHS-PACDC	IMO_2015_R	ACTIVE	N	2017-09-17	9999-12-31	2017-10-30
Diagnosis: ICD10 Code, F00-F99 Define	F01-F99 Mental, Behavioral and Neurodevelopmental disorders	517908	Amphetamine-induced sleep disorder with onset during intoxication with perceptual disturbanc	ICD10CM	1	F15.922	F15.922,F15.982	Other stimulant use, unspecified with intoxication with p	IMO	GHS-PACDC	IMO_2015_R	ACTIVE	N	2017-09-17	9999-12-31	2017-10-30
Diagnosis: ICD10 Code, F00-F99 Define	F01-F99 Mental, Behavioral and Neurodevelopmental disorders	975815	Caffeine-induced anxiety disorder with onset during intoxication, with perceptual disturbance (I	ICD10CM	2	F15.922	F15.122,F15.922	Other stimulant use, unspecified with intoxication with p	IMO	GHS-PACDC	IMO_2015_R	ACTIVE	N	2016-04-10	9999-12-31	2015-07-10
Diagnosis: ICD10 Code, F00-F99 Define	F01-F99 Mental, Behavioral and Neurodevelopmental disorders	1030520	Continuous caffeine intoxication with complication, with unspecified complication (HCC)	ICD10CM	1	F15.929		Other stimulant use, unspecified with intoxication, unsp	IMO	GHS-PACDC	IMO_2015_R	ACTIVE	N	2016-09-17	9999-12-31	2016-09-30
Diagnosis: ICD10 Code, F00-F99 Define	F01-F99 Mental, Behavioral and Neurodevelopmental disorders	1003067	Episodic caffeine intoxication with complication, with unspecified complication (HCC)	ICD10CM	1	F15.929		Other stimulant use, unspecified with intoxication, unsp	IMO	GHS-PACDC	IMO_2015_R	ACTIVE	N	2016-09-17	9999-12-31	2016-09-30
Diagnosis: ICD10 Code, F00-F99 Define	F01-F99 Mental, Behavioral and Neurodevelopmental disorders	30109	Caffeine intoxication (HCC)	ICD10CM	1	F15.929		Other stimulant use, unspecified with intoxication, unsp	IMO	GHS-PACDC	DX_LEGACY	ACTIVE	N	1900-01-01	9999-12-31	2015-07-10
Diagnosis: ICD10 Code, F00-F99 Define	F01-F99 Mental, Behavioral and Neurodevelopmental disorders	517452	Onset of amphetamine-induced mood disorder during intoxication with complication (HCC)	ICD10CM	1	F15.929	F15.929,F15.94	Other stimulant use, unspecified with intoxication, unsp	IMO	GHS-PACDC	IMO_2015_R	ACTIVE	N	2017-09-17	9999-12-31	2017-10-30
Diagnosis: ICD10 Code, F00-F99 Define	F01-F99 Mental, Behavioral and Neurodevelopmental disorders	965428	Onset of amphetamine-induced sexual dysfunction during intoxication, with unspecified compli	ICD10CM	1	F15.929	F15.929,F15.981	Other stimulant use, unspecified with intoxication, unsp	IMO	GHS-PACDC	IMO_2015_R	ACTIVE	N	2017-09-17	9999-12-31	2017-10-30
Diagnosis: ICD10 Code, F00-F99 Define	F01-F99 Mental, Behavioral and Neurodevelopmental disorders	957620	Amphetamine and psychostimulant withdrawal, with delirium (HCC)	ICD10CM	2	F15.93	F19.931,F15.93	Other stimulant use, unspecified with withdrawal	IMO	GHS-PACDC	IMO_2015_R	ACTIVE	N	2016-04-10	9999-12-31	2015-07-10
Diagnosis: ICD10 Code, F00-F99 Define	F01-F99 Mental, Behavioral and Neurodevelopmental disorders	613926	Other stimulant use, unspecified with stimulant-induced psychotic disorder	ICD10CM	1	F15.95		Other stimulant use, unspecified with stimulant-induced	IMO	GHS-PACDC	IMO_2015_R	ACTIVE	Y	1900-01-01	9999-12-31	2015-07-10
Diagnosis: ICD10 Code, F00-F99 Define	F01-F99 Mental, Behavioral and Neurodevelopmental disorders	1183258	Other stimulant use, unspecified with stimulant-induced psychotic disorder with delusions (HCC	ICD10CM	1	F15.950		Other stimulant use, unspecified with stimulant-induced	IMO	GHS-PACDC	IMO_2016_R	ACTIVE	N	1900-01-01	9999-12-31	2016-06-02
Diagnosis: ICD10 Code, F00-F99 Define	F01-F99 Mental, Behavioral and Neurodevelopmental disorders	603366	Other stimulant use, unspecified with stimulant-induced psychotic disorder with hallucinations	ICD10CM	1	F15.951		Other stimulant use, unspecified with stimulant-induced	IMO	GHS-PACDC	IMO_2015_R	ACTIVE	Y	1900-01-01	9999-12-31	2015-07-10
Diagnosis: ICD10 Code, F00-F99 Define	F01-F99 Mental, Behavioral and Neurodevelopmental disorders	1186292	Other stimulant use, unspecified with stimulant-induced psychotic disorder with hallucinations	ICD10CM	1	F15.951		Other stimulant use, unspecified with stimulant-induced	IMO	GHS-PACDC	IMO_2016_R	ACTIVE	N	1900-01-01	9999-12-31	2016-06-02
Diagnosis: ICD10 Code, F00-F99 Define	F01-F99 Mental, Behavioral and Neurodevelopmental disorders	517046	Caffeine-induced anxiety disorder with onset during intoxication without complication (HCC)	ICD10CM	2	F15.980	F15.920,F15.980	Other stimulant use, unspecified with stimulant-induced	IMO	GHS-PACDC	IMO_2015_R	ACTIVE	N	2017-09-17	9999-12-31	2017-10-30
Diagnosis: ICD10 Code, F00-F99 Define	F01-F99 Mental, Behavioral and Neurodevelopmental disorders	317472	Amphetamine and psychostimulant-induced anxiety disorder (HCC)	ICD10CM	1	F15.980		Other stimulant use, unspecified with stimulant-induced	IMO	GHS-PACDC	DX_LEGACY	ACTIVE	N	1900-01-01	9999-12-31	2015-07-10
Diagnosis: ICD10 Code, F00-F99 Define	F01-F99 Mental, Behavioral and Neurodevelopmental disorders	487101	Caffeine-induced anxiety disorder without use disorder with onset during intoxication (HCC)	ICD10CM	1	F15.980	F15.980,F15.929	Other stimulant use, unspecified with stimulant-induced	IMO	GHS-PACDC	IMO_2015_R	ACTIVE	N	2016-09-17	9999-12-31	2016-09-30
Diagnosis: ICD10 Code, F00-F99 Define	F01-F99 Mental, Behavioral and Neurodevelopmental disorders	317474	Amphetamine and psychostimulant-induced sexual disorder (HCC)	ICD10CM	1	F15.981		Other stimulant use, unspecified with stimulant-induced	IMO	GHS-PACDC	DX_LEGACY	ACTIVE	N	1900-01-01	9999-12-31	2015-07-10
Diagnosis: ICD10 Code, F00-F99 Define	F01-F99 Mental, Behavioral and Neurodevelopmental disorders	489531	Caffeine-induced sleep disorder, parasomnia type, with onset during discontinuation/withdrawa	ICD10CM	1	F15.982		Other stimulant use, unspecified with stimulant-induced	IMO	GHS-PACDC	IMO_2015_R	ACTIVE	N	1900-01-01	9999-12-31	2015-07-10
Diagnosis: ICD10 Code, F00-F99 Define	F01-F99 Mental, Behavioral and Neurodevelopmental disorders	516472	Caffeine-induced sleep disorder with onset during intoxication with delirium (HCC)	ICD10CM	2	F15.982	F15.921,F15.982	Other stimulant use, unspecified with stimulant-induced	IMO	GHS-PACDC	IMO_2015_R	ACTIVE	N	2017-09-17	9999-12-31	2017-10-30
Diagnosis: ICD10 Code, F00-F99 Define	F01-F99 Mental, Behavioral and Neurodevelopmental disorders	144240	Caffeine-induced sleep disorder (HCC)	ICD10CM	1	F15.982		Other stimulant use, unspecified with stimulant-induced	IMO	GHS-PACDC	DX_LEGACY	ACTIVE	N	1900-01-01	9999-12-31	2015-07-10
Diagnosis: ICD10 Code, F00-F99 Define	F01-F99 Mental, Behavioral and Neurodevelopmental disorders	487156	Caffeine-induced sleep disorder without use disorder, daytime sleepiness type (HCC)	ICD10CM	1	F15.982		Other stimulant use, unspecified with stimulant-induced	IMO	GHS-PACDC	IMO_2015_R	ACTIVE	N	1900-01-01	9999-12-31	2015-07-10
Diagnosis: ICD10 Code, F00-F99 Define	F01-F99 Mental, Behavioral and Neurodevelopmental disorders	966646	Onset of caffeine-induced sleep disorder during intoxication, uncomplicated (HCC)	ICD10CM	2	F15.982	F15.920,F15.982	Other stimulant use, unspecified with stimulant-induced	IMO	GHS-PACDC	IMO_2015_R	ACTIVE	N	2017-09-17	9999-12-31	2017-10-30
Diagnosis: ICD10 Code, F00-F99 Define	F01-F99 Mental, Behavioral and Neurodevelopmental disorders	1181268	Other stimulant use, unspecified with other stimulant-induced disorder (HCC)	ICD10CM	1	F15.988		Other stimulant use, unspecified with other stimulant-in	IMO	GHS-PACDC	IMO_2016_R	ACTIVE	N	1900-01-01	9999-12-31	2016-06-02
Diagnosis: ICD10 Code, F00-F99 Define	F01-F99 Mental, Behavioral and Neurodevelopmental disorders	207378	Amphetamine-induced mental disorder (HCC)	ICD10CM	1	F15.988		Other stimulant use, unspecified with other stimulant-in	IMO	GHS-PACDC	DX_LEGACY	ACTIVE	N	1900-01-01	9999-12-31	2015-07-10
Diagnosis: ICD10 Code, F00-F99 Define	F01-F99 Mental, Behavioral and Neurodevelopmental disorders	491935	Mild khat use disorder, in controlled environment	ICD10CM	1	F15.99		Other stimulant use, unspecified with unspecified stimul	IMO	GHS-PACDC	IMO_2015_R	ACTIVE	N	1900-01-01	2016-10-14	2015-07-10
Diagnosis: ICD10 Code, F00-F99 Define	F01-F99 Mental, Behavioral and Neurodevelopmental disorders	491974	Vyvanse use disorder, mild, in controlled environment	ICD10CM	1	F15.99		Other stimulant use, unspecified with unspecified stimul	IMO	GHS-PACDC	IMO_2015_R	ACTIVE	N	1900-01-01	2016-10-14	2015-07-10
Diagnosis: ICD10 Code, F00-F99 Define	F01-F99 Mental, Behavioral and Neurodevelopmental disorders	484036	Mild amphetamine use disorder	ICD10CM	1	F15.99		Other stimulant use, unspecified with unspecified stimul	IMO	GHS-PACDC	IMO_2015_R	ACTIVE	N	1900-01-01	2016-10-14	2015-07-10
Diagnosis: ICD10 Code, F00-F99 Define	F01-F99 Mental, Behavioral and Neurodevelopmental disorders	507093	Ketamine use disorder, mild, in controlled environment, abuse	ICD10CM	1	F16.10		Hallucinogen abuse, uncomplicated	IMO	GHS-PACDC	IMO_2015_R	ACTIVE	N	1900-01-01	2016-10-14	2015-07-10
Diagnosis: ICD10 Code, F00-F99 Define	F01-F99 Mental, Behavioral and Neurodevelopmental disorders	484042	Psilocybin use disorder, mild	ICD10CM	1	F16.10		Hallucinogen abuse, uncomplicated	IMO	GHS-PACDC	IMO_2015_R	ACTIVE	N	2016-09-17	9999-12-31	2016-09-30
Diagnosis: ICD10 Code, F00-F99 Define	F01-F99 Mental, Behavioral and Neurodevelopmental disorders	484782	Mild 2,5-dimethoxy-4-methylamphetamine (DOM) use disorder in controlled environment	ICD10CM	1	F16.10		Hallucinogen abuse, uncomplicated	IMO	GHS-PACDC	IMO_2015_R	ACTIVE	N	2016-09-17	9999-12-31	2016-09-30
Diagnosis: ICD10 Code, F00-F99 Define	F01-F99 Mental, Behavioral and Neurodevelopmental disorders	491936	Peyote use disorder, mild, in controlled environment	ICD10CM	1	F16.10		Hallucinogen abuse, uncomplicated	IMO	GHS-PACDC	IMO_2015_R	ACTIVE	N	2016-09-17	9999-12-31	2016-09-30
Diagnosis: ICD10 Code, F00-F99 Define	F01-F99 Mental, Behavioral and Neurodevelopmental disorders	484714	Mild 2,5-dimethoxy-4-methylamphetamine (DOM) use disorder in early remission	ICD10CM	1	F16.10		Hallucinogen abuse, uncomplicated	IMO	GHS-PACDC	IMO_2015_R	ACTIVE	N	2016-09-17	9999-12-31	2016-09-30
Diagnosis: ICD10 Code, F00-F99 Define	F01-F99 Mental, Behavioral and Neurodevelopmental disorders	508274	Mild phencyclidine abuse in sustained remission	ICD10CM	1	F16.11		Hallucinogen abuse, in remission	IMO	GHS-PACDC	IMO_2015_R	ACTIVE	N	2017-09-17	9999-12-31	2017-10-30
Diagnosis: ICD10 Code, F00-F99 Define	F01-F99 Mental, Behavioral and Neurodevelopmental disorders	1083281	Hallucinogen-induced mood disorder with onset during intoxication without complication, unco	ICD10CM	1	F16.120	F16.120,F16.180	Hallucinogen abuse with intoxication, uncomplicated	IMO	GHS-PACDC	IMO_2015_R	DELETED	Y	1900-01-01	9999-12-31	2015-07-10
Diagnosis: ICD10 Code, F00-F99 Define	F01-F99 Mental, Behavioral and Neurodevelopmental disorders	968374	Phencyclidine intoxication with mild use disorder, uncomplicated (HCC)	ICD10CM	1	F16.120		Hallucinogen abuse with intoxication, uncomplicated	IMO	GHS-PACDC	IMO_2015_R	ACTIVE	N	1900-01-01	9999-12-31	2015-07-10
Diagnosis: ICD10 Code, F00-F99 Define	F01-F99 Mental, Behavioral and Neurodevelopmental disorders	485757	Acute phencyclidine intoxication delirium with mild use disorder (HCC)	ICD10CM	1	F16.121		Hallucinogen abuse with intoxication with delirium	IMO	GHS-PACDC	IMO_2015_R	ACTIVE	N	2016-09-17	9999-12-31	2016-09-30
Diagnosis: ICD10 Code, F00-F99 Define	F01-F99 Mental, Behavioral and Neurodevelopmental disorders	964746	Onset of hallucinogen-induced anxiety disorder during intoxication, with perceptual disturbanc	ICD10CM	1	F16.122	F16.122,F16.180	Hallucinogen abuse with intoxication with perceptual dis	IMO	GHS-PACDC	IMO_2015_R	DELETED	Y	1900-01-01	9999-12-31	2015-07-10
Diagnosis: ICD10 Code, F00-F99 Define	F01-F99 Mental, Behavioral and Neurodevelopmental disorders	973487	Phencyclidine-induced anxiety disorder with onset during intoxication, with perceptual disturba	ICD10CM	2	F16.122	F19.122,F16.122	Hallucinogen abuse with intoxication with perceptual dis	IMO	GHS-PACDC	IMO_2015_R	DELETED	Y	2016-04-10	9999-12-31	2015-07-10
Diagnosis: ICD10 Code, F00-F99 Define	F01-F99 Mental, Behavioral and Neurodevelopmental disorders	517136	Phencyclidine intoxication with mild use disorder with perceptual disturbance (HCC)	ICD10CM	1	F16.122		Hallucinogen abuse with intoxication with perceptual dis	IMO	GHS-PACDC	IMO_2015_R	ACTIVE	N	2016-09-17	9999-12-31	2016-09-30
Diagnosis: ICD10 Code, F00-F99 Define	F01-F99 Mental, Behavioral and Neurodevelopmental disorders	429241	Phencyclidine intoxication with mild use disorder (HCC)	ICD10CM	1	F16.129		Hallucinogen abuse with intoxication, unspecified	IMO	GHS-PACDC	IMO_2015_R	ACTIVE	N	1900-01-01	9999-12-31	2015-07-10
Diagnosis: ICD10 Code, F00-F99 Define	F01-F99 Mental, Behavioral and Neurodevelopmental disorders	1030248	Acute intoxication from hallucinogens with complication, with unspecified complication (HCC)	ICD10CM	1	F16.129		Hallucinogen abuse with intoxication, unspecified	IMO	GHS-PACDC	IMO_2015_R	ACTIVE	N	1900-01-01	2016-10-14	2015-07-10
Diagnosis: ICD10 Code, F00-F99 Define	F01-F99 Mental, Behavioral and Neurodevelopmental disorders	975783	Phencyclidine-induced psychotic disorder with mild use disorder with onset during intoxication,	ICD10CM	1	F16.150	F16.150,F16.129	Hallucinogen abuse with hallucinogen-induced psychoti	IMO	GHS-PACDC	IMO_2015_R	ACTIVE	N	2016-09-17	2017-10-14	2016-09-30
Diagnosis: ICD10 Code, F00-F99 Define	F01-F99 Mental, Behavioral and Neurodevelopmental disorders	955169	Hallucinogen abuse with hallucinogen-induced psychotic disorder, with delusions (HCC)	ICD10CM	1	F16.150		Hallucinogen abuse with hallucinogen-induced psychoti	IMO	GHS-PACDC	IMO_2015_R	ACTIVE	N	1900-01-01	9999-12-31	2015-07-10
Diagnosis: ICD10 Code, F00-F99 Define	F01-F99 Mental, Behavioral and Neurodevelopmental disorders	344638	Hallucinogen abuse with hallucinogen-induced psychotic disorder with delusions (HCC)	ICD10CM	1	F16.150		Hallucinogen abuse with hallucinogen-induced psychoti	IMO	GHS-PACDC	DX_LEGACY	ACTIVE	N	1900-01-01	9999-12-31	2015-07-10
Diagnosis: ICD10 Code, F00-F99 Define	F01-F99 Mental, Behavioral and Neurodevelopmental disorders	429227	Onset of hallucinogen-induced psychotic disorder with hallucinations during intoxication (HCC)	ICD10CM	2	F16.151	F16.129,F16.151	Hallucinogen abuse with hallucinogen-induced psychoti	IMO	GHS-PACDC	IMO_2015_R	ACTIVE	N	1900-01-01	9999-12-31	2015-07-10
Diagnosis: ICD10 Code, F00-F99 Define	F01-F99 Mental, Behavioral and Neurodevelopmental disorders	1366280	Hallucinogen abuse w psychotic disorder, unsp (HCC)	ICD10CM	1	F16.159		Hallucinogen abuse with hallucinogen-induced psychoti	IMO	GHS-PACDC	IMO_2018_R	ACTIVE	N	1900-01-01	9999-12-31	2017-10-30
Diagnosis: ICD10 Code, F00-F99 Define	F01-F99 Mental, Behavioral and Neurodevelopmental disorders	1088898	Hallucinogen-induced mood disorder with onset during intoxication with delirium, with deliriu	ICD10CM	2	F16.180	F16.121,F16.180	Hallucinogen abuse with hallucinogen-induced anxiety d	IMO	GHS-PACDC	IMO_2015_R	DELETED	Y	1900-01-01	9999-12-31	2015-07-10
Diagnosis: ICD10 Code, F00-F99 Define	F01-F99 Mental, Behavioral and Neurodevelopmental disorders	967625	Onset of hallucinogen-induced anxiety disorder during intoxication, with delirium (HCC)	ICD10CM	2	F16.180	F16.121,F16.180	Hallucinogen abuse with hallucinogen-induced anxiety d	IMO	GHS-PACDC	IMO_2015_R	ACTIVE	N	1900-01-01	2016-05-07	2015-07-10
Diagnosis: ICD10 Code, F00-F99 Define	F01-F99 Mental, Behavioral and Neurodevelopmental disorders	518485	Hallucinogen-induced mood disorder with onset during intoxication with delirium (HCC)	ICD10CM	2	F16.180	F16.129,F16.180	Hallucinogen abuse with hallucinogen-induced anxiety d	IMO	GHS-PACDC	IMO_2015_R	ACTIVE	N	1900-01-01	2016-05-07	2015-07-10
Diagnosis: ICD10 Code, F00-F99 Define	F01-F99 Mental, Behavioral and Neurodevelopmental disorders	1087898	Hallucinogen-induced anxiety disorder with onset during intoxication without complication, wit	ICD10CM	2	F16.180	F16.121,F16.180	Hallucinogen abuse with hallucinogen-induced anxiety d	IMO	GHS-PACDC	IMO_2015_R	DELETED	Y	1900-01-01	9999-12-31	2015-07-10
Diagnosis: ICD10 Code, F00-F99 Define	F01-F99 Mental, Behavioral and Neurodevelopmental disorders	518185	Hallucinogen-induced anxiety disorder with onset during intoxication without complication (HC	ICD10CM	2	F16.180	F16.129,F16.180	Hallucinogen abuse with hallucinogen-induced anxiety d	IMO	GHS-PACDC	IMO_2015_R	ACTIVE	N	1900-01-01	2016-05-07	2015-07-10
Diagnosis: ICD10 Code, F00-F99 Define	F01-F99 Mental, Behavioral and Neurodevelopmental disorders	592027	Hallucinogen abuse with hallucinogen-induced anxiety disorder	ICD10CM	1	F16.180		Hallucinogen abuse with hallucinogen-induced anxiety d	IMO	GHS-PACDC	IMO_2015_R	ACTIVE	Y	1900-01-01	9999-12-31	2015-07-10
Diagnosis: ICD10 Code, F00-F99 Define	F01-F99 Mental, Behavioral and Neurodevelopmental disorders	507628	Severe phencyclidine dependence (HCC)	ICD10CM	1	F16.20		Hallucinogen dependence, uncomplicated	IMO	GHS-PACDC	IMO_2015_R	ACTIVE	N	1900-01-01	9999-12-31	2015-07-10
Diagnosis: ICD10 Code, F00-F99 Define	F01-F99 Mental, Behavioral and Neurodevelopmental disorders	484298	Dimethyltryptamine (DMT) use disorder, moderate, in controlled environment (HCC)	ICD10CM	1	F16.20		Hallucinogen dependence, uncomplicated	IMO	GHS-PACDC	IMO_2015_R	ACTIVE	N	2016-09-17	9999-12-31	2016-09-30
Diagnosis: ICD10 Code, F00-F99 Define	F01-F99 Mental, Behavioral and Neurodevelopmental disorders	492128	Angel dust use disorder, severe, in controlled environment (HCC)	ICD10CM	1	F16.20		Hallucinogen dependence, uncomplicated	IMO	GHS-PACDC	IMO_2015_R	ACTIVE	N	2016-09-17	9999-12-31	2016-09-30
Diagnosis: ICD10 Code, F00-F99 Define	F01-F99 Mental, Behavioral and Neurodevelopmental disorders	508174	Phencyclidine use disorder, severe, in controlled environment, dependence (HCC)	ICD10CM	1	F16.20		Hallucinogen dependence, uncomplicated	IMO	GHS-PACDC	IMO_2015_R	ACTIVE	N	1900-01-01	9999-12-31	2015-07-10

Diagnosis: ICD10 Code, F00-F99 Define	F01-F99 Mental, Behavioral and Neurodevelopmental disorders	492138	Psilocybin mushrooms use disorder, moderate, in controlled environment (HCC)	ICD10CM	1	F16.20		Hallucinogen dependence, uncomplicated	IMO	GHS-PACDC	IMO_2015_R	ACTIVE	N	2016-09-17	9999-12-31	2016-09-30
Diagnosis: ICD10 Code, F00-F99 Define	F01-F99 Mental, Behavioral and Neurodevelopmental disorders	491996	Salvia use disorder, severe, in controlled environment (HCC)	ICD10CM	1	F16.20		Hallucinogen dependence, uncomplicated	IMO	GHS-PACDC	IMO_2015_R	ACTIVE	N	2016-09-17	9999-12-31	2016-09-30
Diagnosis: ICD10 Code, F00-F99 Define	F01-F99 Mental, Behavioral and Neurodevelopmental disorders	385094	Dimethyltryptamine dependence (HCC)	ICD10CM	1	F16.20		Hallucinogen dependence, uncomplicated	IMO	GHS-PACDC	DX_LEGACY	ACTIVE	N	1900-01-01	9999-12-31	2015-07-10
Diagnosis: ICD10 Code, F00-F99 Define	F01-F99 Mental, Behavioral and Neurodevelopmental disorders	484508	Moderate 2,5-dimethoxy-4-methylamphetamine (DOM) use disorder in controlled environment	ICD10CM	1	F16.20		Hallucinogen dependence, uncomplicated	IMO	GHS-PACDC	IMO_2015_R	ACTIVE	N	2016-09-17	9999-12-31	2016-09-30
Diagnosis: ICD10 Code, F00-F99 Define	F01-F99 Mental, Behavioral and Neurodevelopmental disorders	141022	Phencyclidine dependence (HCC)	ICD10CM	1	F16.20		Hallucinogen dependence, uncomplicated	IMO	GHS-PACDC	DX_LEGACY	ACTIVE	N	2016-09-17	9999-12-31	2016-09-30
Diagnosis: ICD10 Code, F00-F99 Define	F01-F99 Mental, Behavioral and Neurodevelopmental disorders	479938	Moderate lysergic acid diethylamide (LSD) use disorder (HCC)	ICD10CM	1	F16.20		Hallucinogen dependence, uncomplicated	IMO	GHS-PACDC	IMO_2015_R	ACTIVE	N	2016-09-17	9999-12-31	2016-09-30
Diagnosis: ICD10 Code, F00-F99 Define	F01-F99 Mental, Behavioral and Neurodevelopmental disorders	507406	Moderate ketamine dependence in early remission (HCC)	ICD10CM	1	F16.21		Hallucinogen dependence, in remission	IMO	GHS-PACDC	IMO_2015_R	ACTIVE	N	1900-01-01	2016-10-14	2015-07-10
Diagnosis: ICD10 Code, F00-F99 Define	F01-F99 Mental, Behavioral and Neurodevelopmental disorders	492028	Psilocin use disorder, moderate, in early remission (HCC)	ICD10CM	1	F16.21		Hallucinogen dependence, in remission	IMO	GHS-PACDC	IMO_2015_R	ACTIVE	N	2016-09-17	9999-12-31	2016-09-30
Diagnosis: ICD10 Code, F00-F99 Define	F01-F99 Mental, Behavioral and Neurodevelopmental disorders	507955	Moderate phencyclidine dependence in sustained remission (HCC)	ICD10CM	1	F16.21		Hallucinogen dependence, in remission	IMO	GHS-PACDC	IMO_2015_R	ACTIVE	N	1900-01-01	9999-12-31	2015-07-10
Diagnosis: ICD10 Code, F00-F99 Define	F01-F99 Mental, Behavioral and Neurodevelopmental disorders	492259	Serenity, Tranquility, and Peace (STP) use disorder, moderate, in sustained remission (HCC)	ICD10CM	1	F16.21		Hallucinogen dependence, in remission	IMO	GHS-PACDC	IMO_2015_R	ACTIVE	N	2016-09-17	9999-12-31	2016-09-30
Diagnosis: ICD10 Code, F00-F99 Define	F01-F99 Mental, Behavioral and Neurodevelopmental disorders	509208	Severe phencyclidine dependence in early remission in controlled environment (HCC)	ICD10CM	1	F16.21		Hallucinogen dependence, in remission	IMO	GHS-PACDC	IMO_2015_R	ACTIVE	N	1900-01-01	9999-12-31	2015-07-10
Diagnosis: ICD10 Code, F00-F99 Define	F01-F99 Mental, Behavioral and Neurodevelopmental disorders	492046	Serenity, Tranquility, and Peace (STP) use disorder, severe, in sustained remission (HCC)	ICD10CM	1	F16.21		Hallucinogen dependence, in remission	IMO	GHS-PACDC	IMO_2015_R	ACTIVE	N	1900-01-01	9999-12-31	2015-07-10
Diagnosis: ICD10 Code, F00-F99 Define	F01-F99 Mental, Behavioral and Neurodevelopmental disorders	507408	Severe phencyclidine dependence in sustained remission (HCC)	ICD10CM	1	F16.21		Hallucinogen dependence, in remission	IMO	GHS-PACDC	IMO_2015_R	ACTIVE	N	1900-01-01	9999-12-31	2015-07-10
Diagnosis: ICD10 Code, F00-F99 Define	F01-F99 Mental, Behavioral and Neurodevelopmental disorders	492342	Angel dust use disorder, moderate, in sustained remission (HCC)	ICD10CM	1	F16.21		Hallucinogen dependence, in remission	IMO	GHS-PACDC	IMO_2015_R	ACTIVE	N	2016-09-17	9999-12-31	2016-09-30
Diagnosis: ICD10 Code, F00-F99 Define	F01-F99 Mental, Behavioral and Neurodevelopmental disorders	487109	Moderate phencyclidine use disorder, in sustained remission (HCC)	ICD10CM	1	F16.21		Hallucinogen dependence, in remission	IMO	GHS-PACDC	IMO_2015_R	ACTIVE	N	2016-09-17	9999-12-31	2016-09-30
Diagnosis: ICD10 Code, F00-F99 Define	F01-F99 Mental, Behavioral and Neurodevelopmental disorders	484958	Severe psilocin use disorder in early remission (HCC)	ICD10CM	1	F16.21		Hallucinogen dependence, in remission	IMO	GHS-PACDC	IMO_2015_R	ACTIVE	N	2016-09-17	9999-12-31	2016-09-30
Diagnosis: ICD10 Code, F00-F99 Define	F01-F99 Mental, Behavioral and Neurodevelopmental disorders	492269	Salvia use disorder, severe, in early remission (HCC)	ICD10CM	1	F16.21		Hallucinogen dependence, in remission	IMO	GHS-PACDC	IMO_2015_R	ACTIVE	N	2016-09-17	9999-12-31	2016-09-30
Diagnosis: ICD10 Code, F00-F99 Define	F01-F99 Mental, Behavioral and Neurodevelopmental disorders	484359	Severe psilocybin use disorder in early remission (HCC)	ICD10CM	1	F16.21		Hallucinogen dependence, in remission	IMO	GHS-PACDC	IMO_2015_R	ACTIVE	N	2016-09-17	9999-12-31	2016-09-30
Diagnosis: ICD10 Code, F00-F99 Define	F01-F99 Mental, Behavioral and Neurodevelopmental disorders	484921	Severe salvia use disorder in sustained remission (HCC)	ICD10CM	1	F16.21		Hallucinogen dependence, in remission	IMO	GHS-PACDC	IMO_2015_R	ACTIVE	N	2016-09-17	9999-12-31	2016-09-30
Diagnosis: ICD10 Code, F00-F99 Define	F01-F99 Mental, Behavioral and Neurodevelopmental disorders	488543	Phencyclidine use disorder, severe, in early remission (HCC)	ICD10CM	1	F16.21		Hallucinogen dependence, in remission	IMO	GHS-PACDC	IMO_2015_R	ACTIVE	N	2016-09-17	9999-12-31	2016-09-30
Diagnosis: ICD10 Code, F00-F99 Define	F01-F99 Mental, Behavioral and Neurodevelopmental disorders	485770	Acute phencyclidine intoxication delirium with moderate or severe use disorder (HCC)	ICD10CM	1	F16.221		Hallucinogen dependence with intoxication with deliriur	IMO	GHS-PACDC	IMO_2015_R	ACTIVE	N	2016-09-17	9999-12-31	2016-09-30
Diagnosis: ICD10 Code, F00-F99 Define	F01-F99 Mental, Behavioral and Neurodevelopmental disorders	486294	Persistent, mixed level of activity, phencyclidine intoxication delirium with moderate or severe	ICD10CM	1	F16.221		Hallucinogen dependence with intoxication with deliriur	IMO	GHS-PACDC	IMO_2015_R	ACTIVE	N	2016-09-17	9999-12-31	2016-09-30
Diagnosis: ICD10 Code, F00-F99 Define	F01-F99 Mental, Behavioral and Neurodevelopmental disorders	596132	Hallucinogen dependence with intoxication, unspecified	ICD10CM	1	F16.229		Hallucinogen dependence with intoxication, unspecified	IMO	GHS-PACDC	IMO_2015_R	ACTIVE	Y	1900-01-01	9999-12-31	2015-07-10
Diagnosis: ICD10 Code, F00-F99 Define	F01-F99 Mental, Behavioral and Neurodevelopmental disorders	485908	Phencyclidine-induced bipolar and related disorder with moderate or severe use disorder with	ICD10CM	2	F16.229	F16.24,F16.229	Hallucinogen dependence with intoxication, unspecified	IMO	GHS-PACDC	IMO_2015_R	ACTIVE	N	2016-09-17	9999-12-31	2016-09-30
Diagnosis: ICD10 Code, F00-F99 Define	F01-F99 Mental, Behavioral and Neurodevelopmental disorders	1368484	Phencyclidine-induced psychotic disorder with moderate or severe use disorder with onset duri	ICD10CM	2	F16.229	F16.251,F16.229	Hallucinogen dependence with intoxication, unspecified	IMO	GHS-PACDC	IMO_2018_R	ACTIVE	N	1900-01-01	9999-12-31	2017-10-30
Diagnosis: ICD10 Code, F00-F99 Define	F01-F99 Mental, Behavioral and Neurodevelopmental disorders	974962	Phencyclidine-induced psychotic disorder with moderate or severe use disorder with onset duri	ICD10CM	2	F16.229	F16.259,F16.229	Hallucinogen dependence with intoxication, unspecified	IMO	GHS-PACDC	IMO_2015_R	ACTIVE	N	2016-09-17	9999-12-31	2016-09-30
Diagnosis: ICD10 Code, F00-F99 Define	F01-F99 Mental, Behavioral and Neurodevelopmental disorders	485928	Phencyclidine-induced depressive disorder without use disorder with onset during withdrawal (	ICD10CM	1	F16.24	F16.24,F32.89,F16.288	Hallucinogen dependence with hallucinogen-induced m	IMO	GHS-PACDC	IMO_2015_R	ACTIVE	N	2016-09-17	9999-12-31	2016-09-30
Diagnosis: ICD10 Code, F00-F99 Define	F01-F99 Mental, Behavioral and Neurodevelopmental disorders	1368449	Phencyclidine-induced psychotic disorder with moderate or severe use disorder with onset duri	ICD10CM	1	F16.250	F16.250,F16.229	Hallucinogen dependence with hallucinogen-induced ps	IMO	GHS-PACDC	IMO_2018_R	ACTIVE	N	1900-01-01	9999-12-31	2017-10-30
Diagnosis: ICD10 Code, F00-F99 Define	F01-F99 Mental, Behavioral and Neurodevelopmental disorders	517141	Phencyclidine-induced psychotic disorder with mild use disorder with onset during withdrawal	ICD10CM	2	F16.251	F16.99,F16.251	Hallucinogen dependence with hallucinogen-induced ps	IMO	GHS-PACDC	IMO_2015_R	ACTIVE	N	1900-01-01	2016-10-14	2015-07-10
Diagnosis: ICD10 Code, F00-F99 Define	F01-F99 Mental, Behavioral and Neurodevelopmental disorders	517007	Phencyclidine-induced psychotic disorder without use disorder with onset during withdrawal w	ICD10CM	1	F16.251	F16.251,F16.288	Hallucinogen dependence with hallucinogen-induced ps	IMO	GHS-PACDC	IMO_2015_R	ACTIVE	N	2016-09-17	9999-12-31	2016-09-30
Diagnosis: ICD10 Code, F00-F99 Define	F01-F99 Mental, Behavioral and Neurodevelopmental disorders	489475	Phencyclidine-induced psychotic disorder with onset during withdrawal (HCC)	ICD10CM	2	F16.259	F16.288,F16.259	Hallucinogen dependence with hallucinogen-induced ps	IMO	GHS-PACDC	IMO_2015_R	ACTIVE	N	2017-09-17	9999-12-31	2017-10-30
Diagnosis: ICD10 Code, F00-F99 Define	F01-F99 Mental, Behavioral and Neurodevelopmental disorders	1368461	Phencyclidine-induced psychotic disorder with moderate or severe use disorder with onset duri	ICD10CM	1	F16.259	F16.259,F16.229	Hallucinogen dependence with hallucinogen-induced ps	IMO	GHS-PACDC	IMO_2018_R	ACTIVE	N	1900-01-01	9999-12-31	2017-10-30
Diagnosis: ICD10 Code, F00-F99 Define	F01-F99 Mental, Behavioral and Neurodevelopmental disorders	517473	Phencyclidine-induced psychotic disorder without use disorder with onset during withdrawal w	ICD10CM	1	F16.259	F16.259,F19.239	Hallucinogen dependence with hallucinogen-induced ps	IMO	GHS-PACDC	IMO_2015_R	ACTIVE	N	1900-01-01	2016-10-14	2015-07-10
Diagnosis: ICD10 Code, F00-F99 Define	F01-F99 Mental, Behavioral and Neurodevelopmental disorders	1367090	Hallucinogen dependence w psychotic disorder, unsp (HCC)	ICD10CM	1	F16.259		Hallucinogen dependence with hallucinogen-induced ps	IMO	GHS-PACDC	IMO_2018_R	ACTIVE	N	1900-01-01	9999-12-31	2017-10-30
Diagnosis: ICD10 Code, F00-F99 Define	F01-F99 Mental, Behavioral and Neurodevelopmental disorders	489475	Phencyclidine-induced psychotic disorder with onset during withdrawal (HCC)	ICD10CM	1	F16.259		Hallucinogen dependence with hallucinogen-induced ps	IMO	GHS-PACDC	IMO_2015_R	ACTIVE	N	2016-04-10	2017-10-14	2015-07-10
Diagnosis: ICD10 Code, F00-F99 Define	F01-F99 Mental, Behavioral and Neurodevelopmental disorders	516832	Phencyclidine-induced psychotic disorder with onset during withdrawal with complication (HCC	ICD10CM	1	F16.259	F16.259,F19.239	Hallucinogen dependence with hallucinogen-induced ps	IMO	GHS-PACDC	IMO_2015_R	ACTIVE	N	1900-01-01	2016-10-14	2015-07-10
Diagnosis: ICD10 Code, F00-F99 Define	F01-F99 Mental, Behavioral and Neurodevelopmental disorders	974123	Phencyclidine-induced psychotic disorder with moderate or severe use disorder with onset duri	ICD10CM	2	F16.288	F16.259,F16.288	Hallucinogen dependence with other hallucinogen-indu	IMO	GHS-PACDC	IMO_2015_R	ACTIVE	N	2016-09-17	9999-12-31	2016-09-30
Diagnosis: ICD10 Code, F00-F99 Define	F01-F99 Mental, Behavioral and Neurodevelopmental disorders	517961	Onset of phencyclidine-induced psychotic disorder during withdrawal with complication (HCC)	ICD10CM	1	F16.288	F16.288,F16.259	Hallucinogen dependence with other hallucinogen-indu	IMO	GHS-PACDC	IMO_2015_R	ACTIVE	N	2017-09-17	9999-12-31	2017-10-30
Diagnosis: ICD10 Code, F00-F99 Define	F01-F99 Mental, Behavioral and Neurodevelopmental disorders	1368466	Phencyclidine-induced psychotic disorder with moderate or severe use disorder with onset duri	ICD10CM	2	F16.288	F16.250,F16.288	Hallucinogen dependence with other hallucinogen-indu	IMO	GHS-PACDC	IMO_2018_R	ACTIVE	N	1900-01-01	9999-12-31	2017-10-30
Diagnosis: ICD10 Code, F00-F99 Define	F01-F99 Mental, Behavioral and Neurodevelopmental disorders	614672	Hallucinogen use, unspecified	ICD10CM	1	F16.9		Hallucinogen use, unspecified	IMO	GHS-PACDC	IMO_2015_R	ACTIVE	Y	1900-01-01	9999-12-31	2015-07-10
Diagnosis: ICD10 Code, F00-F99 Define	F01-F99 Mental, Behavioral and Neurodevelopmental disorders	491975	Serenity, Tranquility, and Peace (STP) use disorder, mild, in sustained remission	ICD10CM	1	F16.90		Hallucinogen use, unspecified, uncomplicated	IMO	GHS-PACDC	IMO_2015_R	ACTIVE	N	1900-01-01	2016-10-14	2015-07-10
Diagnosis: ICD10 Code, F00-F99 Define	F01-F99 Mental, Behavioral and Neurodevelopmental disorders	484884	Moderate ketamine use disorder in early remission (HCC)	ICD10CM	1	F16.90		Hallucinogen use, unspecified, uncomplicated	IMO	GHS-PACDC	IMO_2015_R	ACTIVE	N	1900-01-01	2016-10-14	2015-07-10
Diagnosis: ICD10 Code, F00-F99 Define	F01-F99 Mental, Behavioral and Neurodevelopmental disorders	484308	Moderate psilocin use disorder in early remission (HCC)	ICD10CM	1	F16.90		Hallucinogen use, unspecified, uncomplicated	IMO	GHS-PACDC	IMO_2015_R	ACTIVE	N	1900-01-01	2016-10-14	2015-07-10
Diagnosis: ICD10 Code, F00-F99 Define	F01-F99 Mental, Behavioral and Neurodevelopmental disorders	492212	Ketamine use disorder, moderate, in controlled environment (HCC)	ICD10CM	1	F16.90		Hallucinogen use, unspecified, uncomplicated	IMO	GHS-PACDC	IMO_2015_R	ACTIVE	N	1900-01-01	2016-10-14	2015-07-10
Diagnosis: ICD10 Code, F00-F99 Define	F01-F99 Mental, Behavioral and Neurodevelopmental disorders	484597	Mild psilocin use disorder in early remission	ICD10CM	1	F16.90		Hallucinogen use, unspecified, uncomplicated	IMO	GHS-PACDC	IMO_2015_R	ACTIVE	N	1900-01-01	2016-10-14	2015-07-10
Diagnosis: ICD10 Code, F00-F99 Define	F01-F99 Mental, Behavioral and Neurodevelopmental disorders	484015	Psilocybin use disorder, severe (HCC)	ICD10CM	1	F16.90		Hallucinogen use, unspecified, uncomplicated	IMO	GHS-PACDC	IMO_2015_R	ACTIVE	N	1900-01-01	2016-10-14	2015-07-10
Diagnosis: ICD10 Code, F00-F99 Define	F01-F99 Mental, Behavioral and Neurodevelopmental disorders	492027	Serenity, Tranquility, and Peace (STP) use disorder, mild, in controlled environment	ICD10CM	1	F16.90		Hallucinogen use, unspecified, uncomplicated	IMO	GHS-PACDC	IMO_2015_R	ACTIVE	N	1900-01-01	2016-10-14	2015-07-10
Diagnosis: ICD10 Code, F00-F99 Define	F01-F99 Mental, Behavioral and Neurodevelopmental disorders	484565	Moderate lysergic acid diethylamide (LSD) use disorder in sustained remission (HCC)	ICD10CM	1	F16.90		Hallucinogen use, unspecified, uncomplicated	IMO	GHS-PACDC	IMO_2015_R	ACTIVE	N	1900-01-01	2016-10-14	2015-07-10
Diagnosis: ICD10 Code, F00-F99 Define	F01-F99 Mental, Behavioral and Neurodevelopmental disorders	492228	Mescaline use disorder, moderate, in early remission (HCC)	ICD10CM	1	F16.90		Hallucinogen use, unspecified, uncomplicated	IMO	GHS-PACDC	IMO_2015_R	ACTIVE	N	1900-01-01	2016-10-14	2015-07-10
Diagnosis: ICD10 Code, F00-F99 Define	F01-F99 Mental, Behavioral and Neurodevelopmental disorders	485005	Mild mescaline use disorder in controlled environment	ICD10CM	1	F16.90		Hallucinogen use, unspecified, uncomplicated	IMO	GHS-PACDC	IMO_2015_R	ACTIVE	N	1900-01-01	2016-10-14	2015-07-10
Diagnosis: ICD10 Code, F00-F99 Define	F01-F99 Mental, Behavioral and Neurodevelopmental disorders	484742	Severe lysergic acid diethylamide (LSD) use disorder in sustained remission (HCC)	ICD10CM	1	F16.90		Hallucinogen use, unspecified, uncomplicated	IMO	GHS-PACDC	IMO_2015_R	ACTIVE	N	1900-01-01	2016-10-14	2015-07-10
Diagnosis: ICD10 Code, F00-F99 Define	F01-F99 Mental, Behavioral and Neurodevelopmental disorders	492191	2,5-dimethoxy-4-methylamphetamine (DOM) use disorder, moderate, in sustained remission (H	ICD10CM	1	F16.90		Hallucinogen use, unspecified, uncomplicated	IMO	GHS-PACDC	IMO_2015_R	ACTIVE	N	1900-01-01	2016-10-14	2015-07-10
Diagnosis: ICD10 Code, F00-F99 Define	F01-F99 Mental, Behavioral and Neurodevelopmental disorders	484696	Moderate psilocybin use disorder in controlled environment (HCC)	ICD10CM	1	F16.90		Hallucinogen use, unspecified, uncomplicated	IMO	GHS-PACDC	IMO_2015_R	ACTIVE	N	1900-01-01	2016-10-14	2015-07-10
Diagnosis: ICD10 Code, F00-F99 Define	F01-F99 Mental, Behavioral and Neurodevelopmental disorders	488543	Phencyclidine use disorder, severe, in early remission (HCC)	ICD10CM	1	F16.90		Hallucinogen use, unspecified, uncomplicated	IMO	GHS-PACDC	IMO_2015_R	ACTIVE	N	1900-01-01	2016-10-14	2015-07-10
Diagnosis: ICD10 Code, F00-F99 Define	F01-F99 Mental, Behavioral and Neurodevelopmental disorders	484751	Severe mescaline use disorder in early remission (HCC)	ICD10CM	1	F16.90		Hallucinogen use, unspecified, uncomplicated	IMO	GHS-PACDC	IMO_2015_R	ACTIVE	N	1900-01-01	2016-10-14	2015-07-10
Diagnosis: ICD10 Code, F00-F99 Define	F01-F99 Mental, Behavioral and Neurodevelopmental disorders	277996	Hallucinogen use	ICD10CM	1	F16.90		Hallucinogen use, unspecified, uncomplicated	IMO	GHS-PACDC	DX_LEGACY	ACTIVE	N	1900-01-01	9999-12-31	2015-07-10
Diagnosis: ICD10 Code, F00-F99 Define	F01-F99 Mental, Behavioral and Neurodevelopmental disorders	484874	Severe mescaline use disorder in sustained remission (HCC)	ICD10CM	1	F16.90		Hallucinogen use, unspecified, uncomplicated	IMO	GHS-PACDC	IMO_2015_R	ACTIVE	N	1900-01-01	2016-10-14	2015-07-10
Diagnosis: ICD10 Code, F00-F99 Define	F01-F99 Mental, Behavioral and Neurodevelopmental disorders	491737	Psilocin use disorder, mild	ICD10CM	1	F16.90		Hallucinogen use, unspecified, uncomplicated	IMO	GHS-PACDC	IMO_2015_R	ACTIVE	N	1900-01-01	2016-10-14	2015-07-10
Diagnosis: ICD10 Code, F00-F99 Define	F01-F99 Mental, Behavioral and Neurodevelopmental disorders	472818	Acute intoxication from hallucinogens with delirium (HCC)	ICD10CM	1	F16.921		Hallucinogen use, unspecified with intoxication with deli	IMO	GHS-PACDC	IMO_2015_R	ACTIVE	N	2016-09-17	9999-12-31	2016-09-30
Diagnosis: ICD10 Code, F00-F99 Define	F01-F99 Mental, Behavioral and Neurodevelopmental disorders	261218	Hallucinogen intoxication with delirium (HCC)	ICD10CM	1	F16.921		Hallucinogen use, unspecified with intoxication with deli	IMO	GHS-PACDC	DX_LEGACY	ACTIVE	N	1900-01-01	9999-12-31	2015-07-10
Diagnosis: ICD10 Code, F00-F99 Define	F01-F99 Mental, Behavioral and Neurodevelopmental disorders	518209	Hallucinogen-induced anxiety disorder with onset during intoxication with delirium (HCC)	ICD10CM	1	F16.921	F16.921,F16.980	Hallucinogen use, unspecified with intoxication with deli	IMO	GHS-PACDC	IMO_2015_R	ACTIVE	N	2016-04-10	9999-12-31	2015-07-10
Diagnosis: ICD10 Code, F00-F99 Define	F01-F99 Mental, Behavioral and Neurodevelopmental disorders	223732	Acute intoxication from hallucinogens (HCC)	ICD10CM	1	F16.929		Hallucinogen use, unspecified with intoxication, unspeci	IMO	GHS-PACDC	DX_LEGACY	ACTIVE	N	2016-09-17	9999-12-31	2016-09-30
Diagnosis: ICD10 Code, F00-F99 Define	F01-F99 Mental, Behavioral and Neurodevelopmental disorders	486381	Phencyclidine-induced psychotic disorder without use disorder with onset during intoxication (H	ICD10CM	2	F16.929	F16.959,F16.929	Hallucinogen use, unspecified with intoxication, unspeci	IMO	GHS-PACDC	IMO_2015_R	ACTIVE	N	2016-09-17	2017-10-14	2016-09-30
Diagnosis: ICD10 Code, F00-F99 Define	F01-F99 Mental, Behavioral and Neurodevelopmental disorders	975783	Phencyclidine-induced psychotic disorder with mild use disorder with onset during intoxication,	ICD10CM	2	F16.929	F16.950,F16.929	Hallucinogen use, unspecified with intoxication, unspeci	IMO	GHS-PACDC	IMO_2015_R	ACTIVE	N	2016-04-10	2016-10-14	2015-07-10
Diagnosis: ICD10 Code, F00-F99 Define	F01-F99 Mental, Behavioral and Neurodevelopmental disorders	1037359	Phencyclidine (PCP) intoxication, with unspecified complication (HCC)	ICD10CM	1	F16.929		Hallucinogen use, unspecified with intoxication, unspeci	IMO	GHS-PACDC	IMO_2015_R	ACTIVE	N	2016-04-10	9999-12-31	2015-07-10
Diagnosis: ICD10 Code, F00-F99 Define	F01-F99 Mental, Behavioral and Neurodevelopmental disorders	449087	Acute intoxication from hallucinogens with complication (HCC)	ICD10CM	1	F16.929		Hallucinogen use, unspecified with intoxication, unspeci	IMO	GHS-PACDC	IMO_2015_R	ACTIVE	N	2016-09-17	9999-12-31	2016-09-30
Diagnosis: ICD10 Code, F00-F99 Define	F01-F99 Mental, Behavioral and Neurodevelopmental disorders	976376	Phencyclidine-induced psychotic disorder without use disorder with onset during intoxication, v	ICD10CM	2	F16.929	F16.950,F16.929	Hallucinogen use, unspecified with intoxication, unspeci	IMO	GHS-PACDC	IMO_2015_R	ACTIVE	N	2016-09-17	9999-12-31	2016-09-30
Diagnosis: ICD10 Code, F00-F99 Define	F01-F99 Mental, Behavioral and Neurodevelopmental disorders	965194	Onset of hallucinogen-induced mood disorder during intoxication, with delirium (HCC)	ICD10CM	2	F16.94	F16.921,F16.94	Hallucinogen use, unspecified with hallucinogen-induce	IMO	GHS-PACDC	IMO_2015_R	ACTIVE	N	2016-04-10	9999-12-31	2015-07-10
Diagnosis: ICD10 Code, F00-F99 Define	F01-F99 Mental, Behavioral and Neurodevelopmental disorders	487170	Phencyclidine-induced bipolar and related disorder without use disorder with onset during into	ICD10CM	1	F16.94		Hallucinogen use, unspecified with hallucinogen-induce	IMO	GHS-PACDC	IMO_2015_R	ACTIVE	N	1900-01-01	2016-10-14	2015-07-10
Diagnosis: ICD10 Code, F00-F99 Define	F01-F99 Mental, Behavioral and Neurodevelopmental disorders	30137	Hallucinogen mood disorder (HCC)	ICD10CM	1	F16.94		Hallucinogen use, unspecified with hallucinogen-induce	IMO	GHS-PACDC	DX_LEGACY	ACTIVE	N	1900-01-01	9999-12-31	2015-07-10
Diagnosis: ICD10 Code, F00-F99 Define	F01-F99 Mental, Behavioral and Neurodevelopmental disorders	487170	Phencyclidine-induced bipolar and related disorder without use disorder with onset during into	ICD10CM	1	F16.94	F16.94,F16.929	Hallucinogen use, unspecified with hallucinogen-induce	IMO	GHS-PACDC	IMO_2015_R	ACTIVE	N	2016-09-17	9999-12-31	2016-09-30
Diagnosis: ICD10 Code, F00-F99 Define	F01-F99 Mental, Behavioral and Neurodevelopmental disorders	487222	Phencyclidine-induced bipolar and related disorder with mild use disorder with onset during wi	ICD10CM	1	F16.94		Hallucinogen use, unspecified with hallucinogen-induce	IMO	GHS-PACDC	IMO_2015_R	ACTIVE	N	1900-01-01	2016-10-14	2015-07-10
Diagnosis: ICD10 Code, F00-F99 Define	F01-F99 Mental, Behavioral and Neurodevelopmental disorders	488815	Phencyclidine-induced bipolar disorder with onset during intoxication (HCC)	ICD10CM	1	F16.94		Hallucinogen use, unspecified with hallucinogen-induce	IMO	GHS-PACDC	IMO_2015_R	ACTIVE	N	1900-01-01	9999-12-31	2015-07-10
Diagnosis: ICD10 Code, F00-F99 Define	F01-F99 Mental, Behavioral and Neurodevelopmental disorders	489063	Onset of phencyclidine-induced bipolar disorder during withdrawal (HCC)	ICD10CM	1	F16.94		Hallucinogen use, unspecified with hallucinogen-induce	IMO	GHS-PACDC	IMO_2015_R	ACTIVE	N	1900-01-01	9999-12-31	2015-07-10
Diagnosis: ICD10 Code, F00-F99 Define	F01-F99 Mental, Behavioral and Neurodevelopmental disorders	968207	Onset of phencyclidine-induced mood disorder during intoxication, with delirium (HCC)	ICD10CM	2	F16.94	F16.921,F16.94	Hallucinogen use, unspecified with hallucinogen-induce	IMO	GHS-PACDC	IMO_2015_R	ACTIVE	N	2017-09-17	9999-12-31	2017-10-30
Diagnosis: ICD10 Code, F00-F99 Define	F01-F99 Mental, Behavioral and Neurodevelopmental disorders	488816	Onset of phencyclidine-induced depressive disorder during withdrawal (HCC)	ICD10CM	1	F16.94		Hallucinogen use, unspecified with hallucinogen-induce	IMO	GHS-PACDC	IMO_2015_R	ACTIVE	N	1900-01-01	9999-12-31	2015-07-10
Diagnosis: ICD10 Code, F00-F99 Define	F01-F99 Mental, Behavioral and Neurodevelopmental disorders	517119	Phencyclidine-induced psychotic disorder with mild use disorder with onset during intoxication	ICD10CM	1	F16.950	F16.950,F16.929	Hallucinogen use, unspecified with hallucinogen-induce	IMO	GHS-PACDC	IMO_2015_R	ACTIVE	N	1900-01-01	2016-10-14	2015-07-10
Diagnosis: ICD10 Code, F00-F99 Define	F01-F99 Mental, Behavioral and Neurodevelopmental disorders	517984	Phencyclidine-induced psychotic disorder without use disorder with onset during intoxication w	ICD10CM	2	F16.950	F16.920,F16.950	Hallucinogen use, unspecified with hallucinogen-induce	IMO	GHS-PACDC	IMO_2015_R	ACTIVE	N	1900-01-01	2016-10-14	2015-07-10
Diagnosis: ICD10 Code, F00-F99 Define	F01-F99 Mental, Behavioral and Neurodevelopmental disorders	973551	Phencyclidine-induced psychotic disorder with mild use disorder with onset during withdrawal,	ICD10CM	1	F16.951		Hallucinogen use, unspecified with hallucinogen-induce	IMO	GHS-PACDC	IMO_2015_R	ACTIVE	N	1900-01-01	2016-05-07	2015-07-10
Diagnosis: ICD10 Code, F00-F99 Define	F01-F99 Mental, Behavioral and Neurodevelopmental disorders	30133	Hallucinogen hallucinosis (HCC)	ICD10CM	1	F16.951		Hallucinogen use, unspecified with hallucinogen-induce	IMO	GHS-PACDC	DX_LEGACY	ACTIVE	N	1900-01-01	9999-12-31	2015-07-10
Diagnosis: ICD10 Code, F00-F99 Define	F01-F99 Mental, Behavioral and Neurodevelopmental disorders	1412259	Phencyclidine-induced psychotic disorder with onset during intoxication, with delirium (HCC)	ICD10CM	2	F16.959	F16.921,F16.959	Hallucinogen use, unspecified with hallucinogen-induce	IMO	GHS-PACDC	IMO_2018_R	ACTIVE	N	1900-01-01	9999-12-31	2017-10-30
Diagnosis: ICD10 Code, F00-F99 Define	F01-F99 Mental, Behavioral and Neurodevelopmental disorders	1103924	Hallucinogen-induced anxiety disorder with onset during intoxication with perceptual disturban	ICD10CM	3	F16.983	F16.921,F16.980,F16.983	Hallucinogen use, unspecified with hallucinogen persisti	IMO	GHS-PACDC	IMO_2016_R	ACTIVE	N	1900-01-01	9999-12-31	2016-06-02
Diagnosis: ICD10 Code, F00-F99 Define	F01-F99 Mental, Behavioral and Neurodevelopmental disorders	1087964	Hallucinogen-induced mood disorder with onset during intoxication with perceptual disturbanc	ICD10CM	3	F16.983	F16.929,F16.94,F16.983	Hallucinogen use, unspecified with hallucinogen persisti	IMO	GHS-PACDC	IMO_2015_R	ACTIVE	N	2016-04-10	9999-12-31	2015-07-10
Diagnosis: ICD10 Code, F00-F99 Define	F01-F99 Mental, Behavioral and Neurodevelopmental disorders	971942	Phencyclidine-induced psychotic disorder with onset during withdrawal, with delusions (HCC)	ICD10CM	2	F16.988	F16.250,F16.988	Hallucinogen use, unspecified with other hallucinogen-ir	IMO	GHS-PACDC	IMO_2015_R	ACTIVE	N	2016-09-17	9999-12-31	2016-09-30
Diagnosis: ICD10 Code, F00-F99 Define	F01-F99 Mental, Behavioral and Neurodevelopmental disorders	517703	Onset of phencyclidine-induced psychotic disorder during withdrawal with hallucinations (HCC)	ICD10CM	2	F16.988	F16.951,F16.988	Hallucinogen use, unspecified with other hallucinogen-ir	IMO	GHS-PACDC	IMO_2015_R	ACTIVE	N	2016-09-17	2017-10-14	2016-09-30
Diagnosis: ICD10 Code, F00-F99 Define	F01-F99 Mental, Behavioral and Neurodevelopmental disorders	30166	Arylcyclohexylamine-related disorder (HCC)	ICD10CM	1	F16.99		Hallucinogen use, unspecified with unspecified hallucinc	IMO	GHS-PACDC	DX_LEGACY	ACTIVE	N	2016-04-10	9999-12-31	2015-07-10
Diagnosis: ICD10 Code, F00-F99 Define	F01-F99 Mental, Behavioral and Neurodevelopmental disorders	375662	Current some day smoker	ICD10CM	1	F17.200		Nicotine dependence, unspecified, uncomplicated	IMO	GHS-PACDC	DX_LEGACY	ACTIVE	N	2017-03-31	9999-12-31	2017-04-14
Diagnosis: ICD10 Code, F00-F99 Define	F01-F99 Mental, Behavioral and Neurodevelopmental disorders	1103981	Tobacco use disorder affecting pregnancy in third trimester, antepartum	ICD10CM	2	F17.200	O99.333,F17.200	Nicotine dependence, unspecified, uncomplicated	IMO	GHS-PACDC	IMO_2016_R	ACTIVE	N	2017-09-17	9999-12-31	2017-10-30
Diagnosis: ICD10 Code, F00-F99 Define	F01-F99 Mental, Behavioral and Neurodevelopmental disorders	491395	Smokes 11 or more cigarettes per day	ICD10CM	1	F17.200		Nicotine dependence, unspecified, uncomplicated	IMO	GHS-PACDC	IMO_2015_R	ACTIVE	N	1900-01-01	2016-05-07	2015-07-10
Diagnosis: ICD10 Code, F00-F99 Define	F01-F99 Mental, Behavioral and Neurodevelopmental disorders	429080	Mild tobacco use disorder	ICD10CM	1	F17.200		Nicotine dependence, unspecified, uncomplicated	IMO	GHS-PACDC	IMO_2015_R	ACTIVE	N	2016-09-17	9999-12-31	2016-09-30
Diagnosis: ICD10 Code, F00-F99 Define	F01-F99 Mental, Behavioral and Neurodevelopmental disorders	375677	Smokes more than 4 packs of cigarettes per day	ICD10CM	1	F17.200		Nicotine dependence, unspecified, uncomplicated	IMO	GHS-PACDC	DX_LEGACY	ACTIVE	N	1900-01-01	2016-05-07	2015-07-10
Diagnosis: ICD10 Code, F00-F99 Define	F01-F99 Mental, Behavioral and Neurodevelopmental disorders	598348	Nicotine dependence, unspecified, uncomplicated	ICD10CM	1	F17.200		Nicotine dependence, unspecified, uncomplicated	IMO	GHS-PACDC	IMO_2015_R	ACTIVE	Y	1900-01-01	9999-12-31	2015-07-10
Diagnosis: ICD10 Code, F00-F99 Define	F01-F99 Mental, Behavioral and Neurodevelopmental disorders	1303674	Has been smoking tobacco for 28 years	ICD10CM	1	F17.200		Nicotine dependence, unspecified, uncomplicated	IMO	GHS-PACDC	IMO_2017_R	ACTIVE	N	1900-01-01	9999-12-31	2017-04-14
Diagnosis: ICD10 Code, F00-F99 Define	F01-F99 Mental, Behavioral and Neurodevelopmental disorders	595734	Nicotine dependence, unspecified, in remission	ICD10CM	1	F17.201		Nicotine dependence, unspecified, in remission	IMO	GHS-PACDC	IMO_2015_R	ACTIVE	Y	1900-01-01	9999-12-31	2015-07-10
Diagnosis: ICD10 Code, F00-F99 Define	F01-F99 Mental, Behavioral and Neurodevelopmental disorders	486691	Severe tobacco use disorder, in early remission	ICD10CM	1	F17.201		Nicotine dependence, unspecified, in remission	IMO	GHS-PACDC	IMO_2015_R	ACTIVE	N	2016-09-17	9999-12-31	2016-09-30

Diagnosis: ICD10 Code, F00-F99 Define	F01-F99 Mental, Behavioral and Neurodevelopmental disorders	509974	Tobacco use disorder, moderate, in sustained remission, dependence	ICD10CM	1	F17.201		Nicotine dependence, unspecified, in remission	IMO	GHS-PACDC	IMO_2015_R	ACTIVE	N	1900-01-01	9999-12-31	2015-07-10
Diagnosis: ICD10 Code, F00-F99 Define	F01-F99 Mental, Behavioral and Neurodevelopmental disorders	486983	Tobacco-induced sleep disorder with moderate or severe use disorder, insomnia type	ICD10CM	1	F17.208	F17.208,G47.8	Nicotine dependence, unspecified, with other nicotine-i	IMO	GHS-PACDC	IMO_2015_R	ACTIVE	N	2016-09-17	9999-12-31	2016-09-30
Diagnosis: ICD10 Code, F00-F99 Define	F01-F99 Mental, Behavioral and Neurodevelopmental disorders	1366031	Nicotine dependence, unsp, w unsp nicotine-induced disorders	ICD10CM	1	F17.209		Nicotine dependence, unspecified, with unspecified nicc	IMO	GHS-PACDC	IMO_2018_R	ACTIVE	N	1900-01-01	9999-12-31	2017-10-30
Diagnosis: ICD10 Code, F00-F99 Define	F01-F99 Mental, Behavioral and Neurodevelopmental disorders	329047	Smoking greater than 10 pack years	ICD10CM	1	F17.210		Nicotine dependence, cigarettes, uncomplicated	IMO	GHS-PACDC	DX_LEGACY	ACTIVE	N	1900-01-01	9999-12-31	2015-07-10
Diagnosis: ICD10 Code, F00-F99 Define	F01-F99 Mental, Behavioral and Neurodevelopmental disorders	331424	Smokes less than 2 packs a day with greater than 15 pack year history	ICD10CM	1	F17.210		Nicotine dependence, cigarettes, uncomplicated	IMO	GHS-PACDC	DX_LEGACY	ACTIVE	N	1900-01-01	9999-12-31	2015-07-10
Diagnosis: ICD10 Code, F00-F99 Define	F01-F99 Mental, Behavioral and Neurodevelopmental disorders	1105403	Dependence on nicotine from cigarettes	ICD10CM	1	F17.210		Nicotine dependence, cigarettes, uncomplicated	IMO	GHS-PACDC	IMO_2016_R	ACTIVE	N	2016-09-17	9999-12-31	2016-09-30
Diagnosis: ICD10 Code, F00-F99 Define	F01-F99 Mental, Behavioral and Neurodevelopmental disorders	471208	Smokes three packs per day or less	ICD10CM	1	F17.210		Nicotine dependence, cigarettes, uncomplicated	IMO	GHS-PACDC	IMO_2015_R	ACTIVE	N	2016-04-10	9999-12-31	2015-07-10
Diagnosis: ICD10 Code, F00-F99 Define	F01-F99 Mental, Behavioral and Neurodevelopmental disorders	331425	Smokes less than 2 packs a day with greater than 20 pack year history	ICD10CM	1	F17.210		Nicotine dependence, cigarettes, uncomplicated	IMO	GHS-PACDC	DX_LEGACY	ACTIVE	N	1900-01-01	9999-12-31	2015-07-10
Diagnosis: ICD10 Code, F00-F99 Define	F01-F99 Mental, Behavioral and Neurodevelopmental disorders	331421	Smokes less than 1 pack a day with greater than 30 pack year history	ICD10CM	1	F17.210		Nicotine dependence, cigarettes, uncomplicated	IMO	GHS-PACDC	DX_LEGACY	ACTIVE	N	1900-01-01	9999-12-31	2015-07-10
Diagnosis: ICD10 Code, F00-F99 Define	F01-F99 Mental, Behavioral and Neurodevelopmental disorders	293968	Chewing tobacco nicotine dependence without complication	ICD10CM	1	F17.220		Nicotine dependence, chewing tobacco, uncomplicated	IMO	GHS-PACDC	DX_LEGACY	ACTIVE	N	1900-01-01	9999-12-31	2015-07-10
Diagnosis: ICD10 Code, F00-F99 Define	F01-F99 Mental, Behavioral and Neurodevelopmental disorders	710591	Tobacco use complicating pregnancy, unspecified trimester	ICD10CM	2	F17.290	O99.330,F17.290	Nicotine dependence, other tobacco product, uncomplic	IMO	GHS-PACDC	IMO_2015_R	ACTIVE	N	2016-04-10	2017-10-14	2015-07-10
Diagnosis: ICD10 Code, F00-F99 Define	F01-F99 Mental, Behavioral and Neurodevelopmental disorders	326926	Pipe smoker unmotivated to quit	ICD10CM	1	F17.290		Nicotine dependence, other tobacco product, uncomplic	IMO	GHS-PACDC	DX_LEGACY	ACTIVE	N	1900-01-01	9999-12-31	2015-07-10
Diagnosis: ICD10 Code, F00-F99 Define	F01-F99 Mental, Behavioral and Neurodevelopmental disorders	1178499	Other tobacco product nicotine dependence, uncomplicated	ICD10CM	1	F17.290		Nicotine dependence, other tobacco product, uncomplic	IMO	GHS-PACDC	IMO_2016_R	DELETED	Y	1900-01-01	9999-12-31	2016-06-02
Diagnosis: ICD10 Code, F00-F99 Define	F01-F99 Mental, Behavioral and Neurodevelopmental disorders	315252	Episodic inhalant abuse	ICD10CM	1	F18.10		Inhalant abuse, uncomplicated	IMO	GHS-PACDC	DX_LEGACY	ACTIVE	N	1900-01-01	9999-12-31	2015-07-10
Diagnosis: ICD10 Code, F00-F99 Define	F01-F99 Mental, Behavioral and Neurodevelopmental disorders	486423	Mild aerosol use disorder	ICD10CM	1	F18.10		Inhalant abuse, uncomplicated	IMO	GHS-PACDC	IMO_2015_R	ACTIVE	N	2016-09-17	9999-12-31	2016-09-30
Diagnosis: ICD10 Code, F00-F99 Define	F01-F99 Mental, Behavioral and Neurodevelopmental disorders	268674	Inhalant abuse, uncomplicated	ICD10CM	1	F18.10		Inhalant abuse, uncomplicated	IMO	GHS-PACDC	DX_LEGACY	ACTIVE	N	1900-01-01	9999-12-31	2015-07-10
Diagnosis: ICD10 Code, F00-F99 Define	F01-F99 Mental, Behavioral and Neurodevelopmental disorders	967336	Onset of inhalant-induced mood disorder during intoxication, uncomplicated (HCC)	ICD10CM	1	F18.120	F18.120,F18.14	Inhalant abuse with intoxication, uncomplicated	IMO	GHS-PACDC	IMO_2015_R	ACTIVE	N	1900-01-01	2016-05-07	2015-07-10
Diagnosis: ICD10 Code, F00-F99 Define	F01-F99 Mental, Behavioral and Neurodevelopmental disorders	974304	Inhalant-induced anxiety disorder with onset during intoxication, with delirium (HCC)	ICD10CM	1	F18.121	F18.121,F18.180	Inhalant abuse with intoxication delirium	IMO	GHS-PACDC	IMO_2015_R	ACTIVE	N	1900-01-01	2016-05-07	2015-07-10
Diagnosis: ICD10 Code, F00-F99 Define	F01-F99 Mental, Behavioral and Neurodevelopmental disorders	518399	Inhalant-induced mood disorder with onset during intoxication with delirium (HCC)	ICD10CM	2	F18.121	F18.14,F18.121	Inhalant abuse with intoxication delirium	IMO	GHS-PACDC	IMO_2015_R	ACTIVE	N	1900-01-01	2017-10-14	2015-07-10
Diagnosis: ICD10 Code, F00-F99 Define	F01-F99 Mental, Behavioral and Neurodevelopmental disorders	517874	Inhalant-induced psychotic disorder with mild use disorder with onset during intoxication with I	ICD10CM	2	F18.129	F18.151,F18.129	Inhalant abuse with intoxication, unspecified	IMO	GHS-PACDC	IMO_2015_R	ACTIVE	N	2016-09-17	9999-12-31	2016-09-30
Diagnosis: ICD10 Code, F00-F99 Define	F01-F99 Mental, Behavioral and Neurodevelopmental disorders	486452	Inhalant-induced anxiety disorder with mild use disorder with onset during intoxication (HCC)	ICD10CM	2	F18.129	F18.180,F18.129	Inhalant abuse with intoxication, unspecified	IMO	GHS-PACDC	IMO_2015_R	ACTIVE	N	2016-09-17	9999-12-31	2016-09-30
Diagnosis: ICD10 Code, F00-F99 Define	F01-F99 Mental, Behavioral and Neurodevelopmental disorders	487071	Inhalant-induced depressive disorder with mild use disorder with onset during intoxication (HC	ICD10CM	3	F18.129	F18.14,F32.89,F18.129	Inhalant abuse with intoxication, unspecified	IMO	GHS-PACDC	IMO_2015_R	ACTIVE	N	2016-09-17	9999-12-31	2016-09-30
Diagnosis: ICD10 Code, F00-F99 Define	F01-F99 Mental, Behavioral and Neurodevelopmental disorders	998695	Inhalant abuse with inhalant-induced psychotic disorder with complication, with delusions (HCC	ICD10CM	1	F18.150		Inhalant abuse with inhalant-induced psychotic disorder	IMO	GHS-PACDC	IMO_2015_R	ACTIVE	N	1900-01-01	9999-12-31	2015-07-10
Diagnosis: ICD10 Code, F00-F99 Define	F01-F99 Mental, Behavioral and Neurodevelopmental disorders	270767	Inhalant abuse with inhalant-induced psychotic disorder (HCC)	ICD10CM	1	F18.159		Inhalant abuse with inhalant-induced psychotic disorder	IMO	GHS-PACDC	DX_LEGACY	ACTIVE	N	1900-01-01	9999-12-31	2015-07-10
Diagnosis: ICD10 Code, F00-F99 Define	F01-F99 Mental, Behavioral and Neurodevelopmental disorders	592650	Inhalant abuse with inhalant-induced dementia	ICD10CM	1	F18.17		Inhalant abuse with inhalant-induced dementia	IMO	GHS-PACDC	IMO_2015_R	ACTIVE	Y	1900-01-01	9999-12-31	2015-07-10
Diagnosis: ICD10 Code, F00-F99 Define	F01-F99 Mental, Behavioral and Neurodevelopmental disorders	968037	Onset of inhalant-induced anxiety disorder during intoxication, uncomplicated (HCC)	ICD10CM	2	F18.180	F18.120,F18.180	Inhalant abuse with inhalant-induced anxiety disorder	IMO	GHS-PACDC	IMO_2015_R	ACTIVE	N	1900-01-01	2016-05-07	2015-07-10
Diagnosis: ICD10 Code, F00-F99 Define	F01-F99 Mental, Behavioral and Neurodevelopmental disorders	508163	Severe inhalant dependence in controlled environment (HCC)	ICD10CM	1	F18.20		Inhalant dependence, uncomplicated	IMO	GHS-PACDC	IMO_2015_R	ACTIVE	N	1900-01-01	9999-12-31	2015-07-10
Diagnosis: ICD10 Code, F00-F99 Define	F01-F99 Mental, Behavioral and Neurodevelopmental disorders	508465	Inhalant use disorder, moderate, in sustained remission, dependence (HCC)	ICD10CM	1	F18.21		Inhalant dependence, in remission	IMO	GHS-PACDC	IMO_2015_R	ACTIVE	N	1900-01-01	9999-12-31	2015-07-10
Diagnosis: ICD10 Code, F00-F99 Define	F01-F99 Mental, Behavioral and Neurodevelopmental disorders	485790	Moderate or severe gas use disorder, in sustained remission, in controlled environment (HCC)	ICD10CM	1	F18.21		Inhalant dependence, in remission	IMO	GHS-PACDC	IMO_2015_R	ACTIVE	N	2016-09-17	9999-12-31	2016-09-30
Diagnosis: ICD10 Code, F00-F99 Define	F01-F99 Mental, Behavioral and Neurodevelopmental disorders	488758	Moderate inhalant use disorder in early remission (HCC)	ICD10CM	1	F18.21		Inhalant dependence, in remission	IMO	GHS-PACDC	IMO_2015_R	ACTIVE	N	2016-09-17	9999-12-31	2016-09-30
Diagnosis: ICD10 Code, F00-F99 Define	F01-F99 Mental, Behavioral and Neurodevelopmental disorders	141019	Inhalant dependence, in remission (HCC)	ICD10CM	1	F18.21		Inhalant dependence, in remission	IMO	GHS-PACDC	DX_LEGACY	ACTIVE	N	1900-01-01	9999-12-31	2015-07-10
Diagnosis: ICD10 Code, F00-F99 Define	F01-F99 Mental, Behavioral and Neurodevelopmental disorders	508505	Severe inhalant dependence in sustained remission in controlled environment (HCC)	ICD10CM	1	F18.21		Inhalant dependence, in remission	IMO	GHS-PACDC	IMO_2015_R	ACTIVE	N	1900-01-01	9999-12-31	2015-07-10
Diagnosis: ICD10 Code, F00-F99 Define	F01-F99 Mental, Behavioral and Neurodevelopmental disorders	268682	Inhalant dependence with intoxication, uncomplicated (HCC)	ICD10CM	1	F18.220		Inhalant dependence with intoxication, uncomplicated	IMO	GHS-PACDC	DX_LEGACY	ACTIVE	N	1900-01-01	9999-12-31	2015-07-10
Diagnosis: ICD10 Code, F00-F99 Define	F01-F99 Mental, Behavioral and Neurodevelopmental disorders	428997	Inhalant intoxication delirium with moderate or severe use disorder (HCC)	ICD10CM	1	F18.221		Inhalant dependence with intoxication delirium	IMO	GHS-PACDC	IMO_2015_R	ACTIVE	N	2016-09-17	9999-12-31	2016-09-30
Diagnosis: ICD10 Code, F00-F99 Define	F01-F99 Mental, Behavioral and Neurodevelopmental disorders	1299134	Inhalant dependence with intoxication, with delirium (HCC)	ICD10CM	1	F18.221		Inhalant dependence with intoxication delirium	IMO	GHS-PACDC	IMO_2017_R	ACTIVE	N	1900-01-01	9999-12-31	2017-04-14
Diagnosis: ICD10 Code, F00-F99 Define	F01-F99 Mental, Behavioral and Neurodevelopmental disorders	460123	Inhalant dependence with intoxication with complication (HCC)	ICD10CM	1	F18.229		Inhalant dependence with intoxication, unspecified	IMO	GHS-PACDC	IMO_2015_R	ACTIVE	N	1900-01-01	9999-12-31	2015-07-10
Diagnosis: ICD10 Code, F00-F99 Define	F01-F99 Mental, Behavioral and Neurodevelopmental disorders	485836	Inhalant-induced depressive disorder with moderate or severe use disorder with onset during v	ICD10CM	1	F18.24	F18.24,F32.89,F18.288	Inhalant dependence with inhalant-induced mood disor	IMO	GHS-PACDC	IMO_2015_R	ACTIVE	N	2016-09-17	9999-12-31	2016-09-30
Diagnosis: ICD10 Code, F00-F99 Define	F01-F99 Mental, Behavioral and Neurodevelopmental disorders	592652	Inhalant dependence with inhalant-induced psychotic disorder with delusions	ICD10CM	1	F18.250		Inhalant dependence with inhalant-induced psychotic di	IMO	GHS-PACDC	IMO_2015_R	ACTIVE	Y	1900-01-01	9999-12-31	2015-07-10
Diagnosis: ICD10 Code, F00-F99 Define	F01-F99 Mental, Behavioral and Neurodevelopmental disorders	344651	Inhalant dependence with inhalant-induced psychotic disorder with hallucinations (HCC)	ICD10CM	1	F18.251		Inhalant dependence with inhalant-induced psychotic di	IMO	GHS-PACDC	DX_LEGACY	ACTIVE	N	1900-01-01	9999-12-31	2015-07-10
Diagnosis: ICD10 Code, F00-F99 Define	F01-F99 Mental, Behavioral and Neurodevelopmental disorders	974034	Inhalant-induced psychotic disorder with moderate or severe use disorder with onset during int	ICD10CM	1	F18.251	F18.251,F18.229	Inhalant dependence with inhalant-induced psychotic di	IMO	GHS-PACDC	IMO_2015_R	ACTIVE	N	2016-09-17	9999-12-31	2016-09-30
Diagnosis: ICD10 Code, F00-F99 Define	F01-F99 Mental, Behavioral and Neurodevelopmental disorders	517369	Inhalant-induced psychotic disorder with onset during withdrawal with hallucinations (HCC)	ICD10CM	2	F18.251	F18.288,F18.251	Inhalant dependence with inhalant-induced psychotic di	IMO	GHS-PACDC	IMO_2015_R	ACTIVE	N	2017-09-17	9999-12-31	2017-10-30
Diagnosis: ICD10 Code, F00-F99 Define	F01-F99 Mental, Behavioral and Neurodevelopmental disorders	1180758	Inhalant dependence with inhalant-induced psychotic disorder, unspecified (HCC)	ICD10CM	1	F18.259		Inhalant dependence with inhalant-induced psychotic di	IMO	GHS-PACDC	IMO_2016_R	ACTIVE	N	1900-01-01	9999-12-31	2016-06-02
Diagnosis: ICD10 Code, F00-F99 Define	F01-F99 Mental, Behavioral and Neurodevelopmental disorders	458615	Inhalant dependence with inhalant-induced psychotic disorder with complication (HCC)	ICD10CM	1	F18.259		Inhalant dependence with inhalant-induced psychotic di	IMO	GHS-PACDC	IMO_2015_R	ACTIVE	N	1900-01-01	9999-12-31	2015-07-10
Diagnosis: ICD10 Code, F00-F99 Define	F01-F99 Mental, Behavioral and Neurodevelopmental disorders	486474	Inhalant-induced anxiety disorder with moderate or severe use disorder with onset during with	ICD10CM	1	F18.280		Inhalant dependence with inhalant-induced anxiety diso	IMO	GHS-PACDC	IMO_2015_R	ACTIVE	N	1900-01-01	2016-10-14	2015-07-10
Diagnosis: ICD10 Code, F00-F99 Define	F01-F99 Mental, Behavioral and Neurodevelopmental disorders	486474	Inhalant-induced anxiety disorder with moderate or severe use disorder with onset during with	ICD10CM	2	F18.288	F18.280,F18.288	Inhalant dependence with other inhalant-induced disor	IMO	GHS-PACDC	IMO_2015_R	ACTIVE	N	2016-09-17	9999-12-31	2016-09-30
Diagnosis: ICD10 Code, F00-F99 Define	F01-F99 Mental, Behavioral and Neurodevelopmental disorders	1368473	Inhalant-induced psychotic disorder with moderate or severe use disorder with onset during wi	ICD10CM	2	F18.288	F18.251,F18.288	Inhalant dependence with other inhalant-induced disor	IMO	GHS-PACDC	IMO_2018_R	ACTIVE	N	1900-01-01	9999-12-31	2017-10-30
Diagnosis: ICD10 Code, F00-F99 Define	F01-F99 Mental, Behavioral and Neurodevelopmental disorders	485793	Moderate or severe volatile solvent use disorder, in early remission, in controlled environment	ICD10CM	1	F18.90		Inhalant use, unspecified, uncomplicated	IMO	GHS-PACDC	IMO_2015_R	ACTIVE	N	1900-01-01	2016-10-14	2015-07-10
Diagnosis: ICD10 Code, F00-F99 Define	F01-F99 Mental, Behavioral and Neurodevelopmental disorders	505175	Inhalant use disorder, severe, in early remission, in controlled environment (HCC)	ICD10CM	1	F18.90		Inhalant use, unspecified, uncomplicated	IMO	GHS-PACDC	IMO_2015_R	ACTIVE	N	1900-01-01	2016-10-14	2015-07-10
Diagnosis: ICD10 Code, F00-F99 Define	F01-F99 Mental, Behavioral and Neurodevelopmental disorders	487096	Mild aerosol use disorder, in sustained remission	ICD10CM	1	F18.90		Inhalant use, unspecified, uncomplicated	IMO	GHS-PACDC	IMO_2015_R	ACTIVE	N	1900-01-01	2016-10-14	2015-07-10
Diagnosis: ICD10 Code, F00-F99 Define	F01-F99 Mental, Behavioral and Neurodevelopmental disorders	485912	Mild volatile solvent use disorder, in early remission	ICD10CM	1	F18.90		Inhalant use, unspecified, uncomplicated	IMO	GHS-PACDC	IMO_2015_R	ACTIVE	N	1900-01-01	2016-10-14	2015-07-10
Diagnosis: ICD10 Code, F00-F99 Define	F01-F99 Mental, Behavioral and Neurodevelopmental disorders	486428	Mild volatile solvent use disorder	ICD10CM	1	F18.90		Inhalant use, unspecified, uncomplicated	IMO	GHS-PACDC	IMO_2015_R	ACTIVE	N	1900-01-01	2016-10-14	2015-07-10
Diagnosis: ICD10 Code, F00-F99 Define	F01-F99 Mental, Behavioral and Neurodevelopmental disorders	486425	Mild gas use disorder, in early remission	ICD10CM	1	F18.90		Inhalant use, unspecified, uncomplicated	IMO	GHS-PACDC	IMO_2015_R	ACTIVE	N	1900-01-01	2016-10-14	2015-07-10
Diagnosis: ICD10 Code, F00-F99 Define	F01-F99 Mental, Behavioral and Neurodevelopmental disorders	517244	Inhalant-induced anxiety disorder with onset during intoxication without complication (HCC)	ICD10CM	2	F18.920	F18.980,F18.920	Inhalant use, unspecified with intoxication, uncomplicat	IMO	GHS-PACDC	IMO_2015_R	ACTIVE	N	2016-04-10	2017-10-14	2015-07-10
Diagnosis: ICD10 Code, F00-F99 Define	F01-F99 Mental, Behavioral and Neurodevelopmental disorders	485917	Persistent hyperactive inhalant intoxication delirium with moderate or severe use disorder (HC	ICD10CM	1	F18.921		Inhalant use, unspecified with intoxication with delirium	IMO	GHS-PACDC	IMO_2015_R	ACTIVE	N	1900-01-01	2016-10-14	2015-07-10
Diagnosis: ICD10 Code, F00-F99 Define	F01-F99 Mental, Behavioral and Neurodevelopmental disorders	485916	Acute hyperactive inhalant intoxication delirium with moderate or severe use disorder (HCC)	ICD10CM	1	F18.921		Inhalant use, unspecified with intoxication with delirium	IMO	GHS-PACDC	IMO_2015_R	ACTIVE	N	1900-01-01	2016-10-14	2015-07-10
Diagnosis: ICD10 Code, F00-F99 Define	F01-F99 Mental, Behavioral and Neurodevelopmental disorders	487048	Acute hyperactive inhalant intoxication delirium without use disorder (HCC)	ICD10CM	1	F18.921		Inhalant use, unspecified with intoxication with delirium	IMO	GHS-PACDC	IMO_2015_R	ACTIVE	N	1900-01-01	9999-12-31	2015-07-10
Diagnosis: ICD10 Code, F00-F99 Define	F01-F99 Mental, Behavioral and Neurodevelopmental disorders	485779	Persistent hyperactive inhalant intoxication delirium without use disorder (HCC)	ICD10CM	1	F18.921		Inhalant use, unspecified with intoxication with delirium	IMO	GHS-PACDC	IMO_2015_R	ACTIVE	N	1900-01-01	9999-12-31	2015-07-10
Diagnosis: ICD10 Code, F00-F99 Define	F01-F99 Mental, Behavioral and Neurodevelopmental disorders	517585	Inhalant-induced psychotic disorder with onset during intoxication with delusions (HCC)	ICD10CM	2	F18.929	F18.950,F18.929	Inhalant use, unspecified with intoxication, unspecified	IMO	GHS-PACDC	IMO_2015_R	ACTIVE	N	2016-09-17	9999-12-31	2016-09-30
Diagnosis: ICD10 Code, F00-F99 Define	F01-F99 Mental, Behavioral and Neurodevelopmental disorders	429000	Inhalant intoxication with moderate or severe use disorder (HCC)	ICD10CM	1	F18.929		Inhalant use, unspecified with intoxication, unspecified	IMO	GHS-PACDC	IMO_2015_R	ACTIVE	N	1900-01-01	2016-10-14	2015-07-10
Diagnosis: ICD10 Code, F00-F99 Define	F01-F99 Mental, Behavioral and Neurodevelopmental disorders	517818	Inhalant-induced psychotic disorder without use disorder with onset during intoxication with ha	ICD10CM	2	F18.929	F18.951,F18.929	Inhalant use, unspecified with intoxication, unspecified	IMO	GHS-PACDC	IMO_2015_R	ACTIVE	N	1900-01-01	9999-12-31	2015-07-10
Diagnosis: ICD10 Code, F00-F99 Define	F01-F99 Mental, Behavioral and Neurodevelopmental disorders	485932	Inhalant-induced depressive disorder without use disorder with onset during intoxication (HCC)	ICD10CM	2	F18.929	F18.94,F18.929	Inhalant use, unspecified with intoxication, unspecified	IMO	GHS-PACDC	IMO_2015_R	ACTIVE	N	1900-01-01	9999-12-31	2015-07-10
Diagnosis: ICD10 Code, F00-F99 Define	F01-F99 Mental, Behavioral and Neurodevelopmental disorders	598964	Inhalant use, unspecified with intoxication, unspecified	ICD10CM	1	F18.929		Inhalant use, unspecified with intoxication, unspecified	IMO	GHS-PACDC	IMO_2015_R	ACTIVE	Y	1900-01-01	9999-12-31	2015-07-10
Diagnosis: ICD10 Code, F00-F99 Define	F01-F99 Mental, Behavioral and Neurodevelopmental disorders	429229	Onset of inhalant-induced mood disorder during intoxication (HCC)	ICD10CM	2	F18.94	F18.929,F18.94	Inhalant use, unspecified with inhalant-induced mood di	IMO	GHS-PACDC	IMO_2015_R	ACTIVE	N	2017-09-17	9999-12-31	2017-10-30
Diagnosis: ICD10 Code, F00-F99 Define	F01-F99 Mental, Behavioral and Neurodevelopmental disorders	517778	Inhalant-induced psychotic disorder with onset during intoxication with hallucinations (HCC)	ICD10CM	1	F18.951	F18.951,F18.129	Inhalant use, unspecified with inhalant-induced psychoti	IMO	GHS-PACDC	IMO_2015_R	ACTIVE	N	1900-01-01	2016-10-14	2015-07-10
Diagnosis: ICD10 Code, F00-F99 Define	F01-F99 Mental, Behavioral and Neurodevelopmental disorders	972512	Inhalant-induced psychotic disorder with mild use disorder with onset during intoxication, with	ICD10CM	1	F18.959	F18.959,F18.929	Inhalant use, unspecified with inhalant-induced psychoti	IMO	GHS-PACDC	IMO_2015_R	ACTIVE	N	2016-04-10	2016-10-14	2015-07-10
Diagnosis: ICD10 Code, F00-F99 Define	F01-F99 Mental, Behavioral and Neurodevelopmental disorders	974161	Onset of inhalant-induced psychotic disorder during withdrawal, with unspecified complication	ICD10CM	1	F18.959	F18.959,F18.288	Inhalant use, unspecified with inhalant-induced psychoti	IMO	GHS-PACDC	IMO_2015_R	ACTIVE	N	2017-03-31	9999-12-31	2017-04-14
Diagnosis: ICD10 Code, F00-F99 Define	F01-F99 Mental, Behavioral and Neurodevelopmental disorders	489033	Onset of inhalant-induced psychotic disorder during intoxication (HCC)	ICD10CM	1	F18.959		Inhalant use, unspecified with inhalant-induced psychoti	IMO	GHS-PACDC	IMO_2015_R	ACTIVE	N	1900-01-01	2017-04-27	2015-07-10
Diagnosis: ICD10 Code, F00-F99 Define	F01-F99 Mental, Behavioral and Neurodevelopmental disorders	972431	Inhalant-induced psychotic disorder without use disorder with onset during intoxication, with u	ICD10CM	1	F18.959	F18.959,F18.920,F18.929,F18.921	Inhalant use, unspecified with inhalant-induced psychoti	IMO	GHS-PACDC	IMO_2015_R	ACTIVE	N	1900-01-01	2016-05-07	2015-07-10
Diagnosis: ICD10 Code, F00-F99 Define	F01-F99 Mental, Behavioral and Neurodevelopmental disorders	429008	Inhalant-induced major neurocognitive disorder with mild use disorder (HCC)	ICD10CM	1	F18.988		Inhalant use, unspecified with other inhalant-induced di	IMO	GHS-PACDC	IMO_2015_R	ACTIVE	N	1900-01-01	2016-10-14	2015-07-10
Diagnosis: ICD10 Code, F00-F99 Define	F01-F99 Mental, Behavioral and Neurodevelopmental disorders	486799	Severe inhalant-induced major neurocognitive disorder with mild use disorder (HCC)	ICD10CM	1	F18.988		Inhalant use, unspecified with other inhalant-induced di	IMO	GHS-PACDC	IMO_2015_R	ACTIVE	N	1900-01-01	2016-10-14	2015-07-10
Diagnosis: ICD10 Code, F00-F99 Define	F01-F99 Mental, Behavioral and Neurodevelopmental disorders	615130	Other psychoactive substance related disorders	ICD10CM	1	F19		Other psychoactive substance related disorders	IMO	GHS-PACDC	IMO_2015_R	ACTIVE	Y	1900-01-01	9999-12-31	2015-07-10
Diagnosis: ICD10 Code, F00-F99 Define	F01-F99 Mental, Behavioral and Neurodevelopmental disorders	507284	Mild cyclobenzaprine abuse	ICD10CM	1	F19.10		Other psychoactive substance abuse, uncomplicated	IMO	GHS-PACDC	IMO_2015_R	ACTIVE	N	2016-09-17	9999-12-31	2016-09-30
Diagnosis: ICD10 Code, F00-F99 Define	F01-F99 Mental, Behavioral and Neurodevelopmental disorders	507307	Mild dextromethorphan abuse in controlled environment	ICD10CM	1	F19.10		Other psychoactive substance abuse, uncomplicated	IMO	GHS-PACDC	IMO_2015_R	ACTIVE	N	2016-09-17	9999-12-31	2016-09-30
Diagnosis: ICD10 Code, F00-F99 Define	F01-F99 Mental, Behavioral and Neurodevelopmental disorders	320666	H/O mixed drug abuse	ICD10CM	1	F19.10		Other psychoactive substance abuse, uncomplicated	IMO	GHS-PACDC	DX_LEGACY	ACTIVE	N	1900-01-01	2016-05-07	2015-07-10
Diagnosis: ICD10 Code, F00-F99 Define	F01-F99 Mental, Behavioral and Neurodevelopmental disorders	507916	Mild dextromethorphan abuse in sustained remission	ICD10CM	1	F19.10		Other psychoactive substance abuse, uncomplicated	IMO	GHS-PACDC	IMO_2015_R	ACTIVE	N	2016-09-17	2017-10-14	2016-09-30
Diagnosis: ICD10 Code, F00-F99 Define	F01-F99 Mental, Behavioral and Neurodevelopmental disorders	215304	Drug abuse, nondependent	ICD10CM	1	F19.10		Other psychoactive substance abuse, uncomplicated	IMO	GHS-PACDC	DX_LEGACY	ACTIVE	N	1900-01-01	9999-12-31	2015-07-10
Diagnosis: ICD10 Code, F00-F99 Define	F01-F99 Mental, Behavioral and Neurodevelopmental disorders	484227	Mild dextromethorphan use disorder in early remission	ICD10CM	1	F19.10		Other psychoactive substance abuse, uncomplicated	IMO	GHS-PACDC	IMO_2015_R	ACTIVE	N	2016-09-17	9999-12-31	2016-09-30
Diagnosis: ICD10 Code, F00-F99 Define	F01-F99 Mental, Behavioral and Neurodevelopmental disorders	141033	PCP abuse, continuous	ICD10CM	1	F19.10		Other psychoactive substance abuse, uncomplicated	IMO	GHS-PACDC	DX_LEGACY	ACTIVE	N	1900-01-01	2016-05-07	2015-07-10
Diagnosis: ICD10 Code, F00-F99 Define	F01-F99 Mental, Behavioral and Neurodevelopmental disorders	706221	Drug abuse of mother, second trimester	ICD10CM	2	F19.10	O99.322,F19.10	Other psychoactive substance abuse, uncomplicated	IMO	GHS-PACDC	IMO_2015_R	ACTIVE	N	2017-09-17	9999-12-31	2017-10-30
Diagnosis: ICD10 Code, F00-F99 Define	F01-F99 Mental, Behavioral and Neurodevelopmental disorders	263688	Nicotine abuse	ICD10CM	1	F19.10		Other psychoactive substance abuse, uncomplicated	IMO	GHS-PACDC	DX_LEGACY	ACTIVE	N	1900-01-01	2016-05-07	2015-07-10
Diagnosis: ICD10 Code, F00-F99 Define	F01-F99 Mental, Behavioral and Neurodevelopmental disorders	507203	Mild propofol abuse	ICD10CM	1	F19.10		Other psychoactive substance abuse, uncomplicated	IMO	GHS-PACDC	IMO_2015_R	ACTIVE	N	2016-09-17	9999-12-31	2016-09-30
Diagnosis: ICD10 Code, F00-F99 Define	F01-F99 Mental, Behavioral and Neurodevelopmental disorders	508303	Dextromethorphan use disorder, mild, abuse	ICD10CM	1	F19.10		Other psychoactive substance abuse, uncomplicated	IMO	GHS-PACDC	IMO_2015_R	ACTIVE	N	2016-09-17	9999-12-31	2016-09-30
Diagnosis: ICD10 Code, F00-F99 Define	F01-F99 Mental, Behavioral and Neurodevelopmental disorders	39279	Antidepressant type abuse, episodic use	ICD10CM	1	F19.10		Other psychoactive substance abuse, uncomplicated	IMO	GHS-PACDC	DX_LEGACY	ACTIVE	N	1900-01-01	9999-12-31	2015-07-10
Diagnosis: ICD10 Code, F00-F99 Define	F01-F99 Mental, Behavioral and Neurodevelopmental disorders	484849	Mild cyclobenzaprine use disorder in sustained remission	ICD10CM	1	F19.10		Other psychoactive substance abuse, uncomplicated	IMO	GHS-PACDC	IMO_2015_R	ACTIVE	N	2016-09-17	9999-12-31	2016-09-30
Diagnosis: ICD10 Code, F00-F99 Define	F01-F99 Mental, Behavioral and Neurodevelopmental disorders	484518	Mild dextromethorphan use disorder on maintenance therapy	ICD10CM	1	F19.10		Other psychoactive substance abuse, uncomplicated	IMO	GHS-PACDC	IMO_2015_R	ACTIVE	N	2016-09-17	9999-12-31	2016-09-30
Diagnosis: ICD10 Code, F00-F99 Define	F01-F99 Mental, Behavioral and Neurodevelopmental disorders	3212	Nondependent antidepressant type abuse	ICD10CM	1	F19.10		Other psychoactive substance abuse, uncomplicated	IMO	GHS-PACDC	DX_LEGACY	ACTIVE	N	1900-01-01	9999-12-31	2015-07-10
Diagnosis: ICD10 Code, F00-F99 Define	F01-F99 Mental, Behavioral and Neurodevelopmental disorders	507172	Mild ketamine abuse in sustained remission in controlled environment	ICD10CM	1	F19.11		Other psychoactive substance abuse, in remission	IMO	GHS-PACDC	IMO_2015_R	ACTIVE	N	2017-09-17	9999-12-31	2017-10-30
Diagnosis: ICD10 Code, F00-F99 Define	F01-F99 Mental, Behavioral and Neurodevelopmental disorders	509694	Mild ketamine abuse in early remission in controlled environment	ICD10CM	1	F19.11		Other psychoactive substance abuse, in remission	IMO	GHS-PACDC	IMO_2015_R	ACTIVE	N	2017-09-17	9999-12-31	2017-10-30
Diagnosis: ICD10 Code, F00-F99 Define	F01-F99 Mental, Behavioral and Neurodevelopmental disorders	3220	Mixed, or nondependent drug abuse, in remission	ICD10CM	1	F19.11		Other psychoactive substance abuse, in remission	IMO	GHS-PACDC	DX_LEGACY	ACTIVE	N	2017-09-17	9999-12-31	2017-10-30
Diagnosis: ICD10 Code, F00-F99 Define	F01-F99 Mental, Behavioral and Neurodevelopmental disorders	508404	Mild propofol abuse in early remission in controlled environment	ICD10CM	1	F19.11		Other psychoactive substance abuse, in remission	IMO	GHS-PACDC	IMO_2015_R	ACTIVE	N	2017-09-17	9999-12-31	2017-10-30
Diagnosis: ICD10 Code, F00-F99 Define	F01-F99 Mental, Behavioral and Neurodevelopmental disorders	509506	Dextromethorphan use disorder, mild, in early remission, abuse	ICD10CM	1	F19.11		Other psychoactive substance abuse, in remission	IMO	GHS-PACDC	IMO_2015_R	ACTIVE	N	2017-09-17	9999-12-31	2017-10-30
Diagnosis: ICD10 Code, F00-F99 Define	F01-F99 Mental, Behavioral and Neurodevelopmental disorders	964814	Onset of amphetamine-induced sexual dysfunction during intoxication, uncomplicated (HCC)	ICD10CM	1	F19.120	F19.120,F15.120	Other psychoactive substance abuse with intoxication, u	IMO	GHS-PACDC	IMO_2015_R	ACTIVE	N	2016-04-10	2017-10-14	2015-07-10
Diagnosis: ICD10 Code, F00-F99 Define	F01-F99 Mental, Behavioral and Neurodevelopmental disorders	957631	Pathological drug intoxication, uncomplicated	ICD10CM	1	F19.120		Other psychoactive substance abuse with intoxication, u	IMO	GHS-PACDC	IMO_2015_R	ACTIVE	N	1900-01-01	2017-10-14	2015-07-10
Diagnosis: ICD10 Code, F00-F99 Define	F01-F99 Mental, Behavioral and Neurodevelopmental disorders	958366	PCP intoxication, uncomplicated (HCC)	ICD10CM	1	F19.120		Other psychoactive substance abuse with intoxication, u	IMO	GHS-PACDC	IMO_2015_R	ACTIVE	N	1900-01-01	2016-05-07	2015-07-10
Diagnosis: ICD10 Code, F00-F99 Define	F01-F99 Mental, Behavioral and Neurodevelopmental disorders	964574	Onset of phencyclidine-induced mood disorder during intoxication, uncomplicated	ICD10CM	1	F19.120		Other psychoactive substance abuse with intoxication, u	IMO	GHS-PACDC	IMO_2015_R	ACTIVE	N	2016-04-10	2017-10-14	2015-07-10

Diagnosis: ICD10 Code, F00-F99 Define	F01-F99 Mental, Behavioral and Neurodevelopmental disorders	1178239	Other psychoactive substance abuse with intoxication, uncomplicated	ICD10CM	1	F19.120		Other psychoactive substance abuse with intoxication, u	IMO	GHS-PACDC	IMO_2016_R	DELETED	Y	1900-01-01	9999-12-31	2016-06-02
Diagnosis: ICD10 Code, F00-F99 Define	F01-F99 Mental, Behavioral and Neurodevelopmental disorders	974779	Onset of unknown substance-induced anxiety disorder during intoxication, with delirium	ICD10CM	1	F19.121		Other psychoactive substance abuse with intoxication de	IMO	GHS-PACDC	IMO_2015_R	DELETED	Y	2016-04-10	9999-12-31	2015-07-10
Diagnosis: ICD10 Code, F00-F99 Define	F01-F99 Mental, Behavioral and Neurodevelopmental disorders	972278	Phencyclidine-induced anxiety disorder with onset during intoxication, with delirium	ICD10CM	1	F19.121	F19.121,F16.921	Other psychoactive substance abuse with intoxication de	IMO	GHS-PACDC	IMO_2015_R	DELETED	Y	2016-04-10	9999-12-31	2015-07-10
Diagnosis: ICD10 Code, F00-F99 Define	F01-F99 Mental, Behavioral and Neurodevelopmental disorders	976490	Unknown substance-induced anxiety disorder with onset during intoxication, with perceptual di	ICD10CM	1	F19.122		Other psychoactive substance abuse with intoxication w	IMO	GHS-PACDC	IMO_2015_R	DELETED	Y	2016-04-10	9999-12-31	2015-07-10
Diagnosis: ICD10 Code, F00-F99 Define	F01-F99 Mental, Behavioral and Neurodevelopmental disorders	965258	Onset of phencyclidine-induced anxiety disorder during intoxication, with perceptual disturbanc	ICD10CM	1	F19.122	F19.122,F19.180	Other psychoactive substance abuse with intoxication w	IMO	GHS-PACDC	IMO_2015_R	DELETED	Y	1900-01-01	2016-05-07	2015-07-10
Diagnosis: ICD10 Code, F00-F99 Define	F01-F99 Mental, Behavioral and Neurodevelopmental disorders	968953	Onset of amphetamine-induced sleep disorder during intoxication, with unspecified complicatic	ICD10CM	1	F19.129	F19.129,F15.129	Other psychoactive substance abuse with intoxication, u	IMO	GHS-PACDC	IMO_2015_R	ACTIVE	N	2016-04-10	2017-10-14	2015-07-10
Diagnosis: ICD10 Code, F00-F99 Define	F01-F99 Mental, Behavioral and Neurodevelopmental disorders	993056	Phencyclidine (PCP) intoxication with complication, with unspecified complication (HCC)	ICD10CM	1	F19.129		Other psychoactive substance abuse with intoxication, u	IMO	GHS-PACDC	IMO_2015_R	ACTIVE	N	1900-01-01	2016-10-14	2015-07-10
Diagnosis: ICD10 Code, F00-F99 Define	F01-F99 Mental, Behavioral and Neurodevelopmental disorders	35618	Pathological drug poisoning (HCC)	ICD10CM	1	F19.129		Other psychoactive substance abuse with intoxication, u	IMO	GHS-PACDC	DX_LEGACY	ACTIVE	N	1900-01-01	2017-10-14	2015-07-10
Diagnosis: ICD10 Code, F00-F99 Define	F01-F99 Mental, Behavioral and Neurodevelopmental disorders	1035254	Substance intoxication with complication, with unspecified complication (HCC)	ICD10CM	1	F19.129		Other psychoactive substance abuse with intoxication, u	IMO	GHS-PACDC	IMO_2015_R	ACTIVE	N	1900-01-01	2016-10-14	2015-07-10
Diagnosis: ICD10 Code, F00-F99 Define	F01-F99 Mental, Behavioral and Neurodevelopmental disorders	965203	Onset of amphetamine-induced mood disorder during intoxication, with unspecified complicati	ICD10CM	1	F19.129	F19.129,F19.14	Other psychoactive substance abuse with intoxication, u	IMO	GHS-PACDC	IMO_2015_R	ACTIVE	N	1900-01-01	2016-05-07	2015-07-10
Diagnosis: ICD10 Code, F00-F99 Define	F01-F99 Mental, Behavioral and Neurodevelopmental disorders	956248	Drug intoxication, with unspecified complication (HCC)	ICD10CM	1	F19.129		Other psychoactive substance abuse with intoxication, u	IMO	GHS-PACDC	IMO_2015_R	ACTIVE	N	1900-01-01	2016-05-07	2015-07-10
Diagnosis: ICD10 Code, F00-F99 Define	F01-F99 Mental, Behavioral and Neurodevelopmental disorders	454816	Acute narcotic intoxication with complication (HCC)	ICD10CM	1	F19.129		Other psychoactive substance abuse with intoxication, u	IMO	GHS-PACDC	IMO_2015_R	ACTIVE	N	1900-01-01	2016-10-14	2015-07-10
Diagnosis: ICD10 Code, F00-F99 Define	F01-F99 Mental, Behavioral and Neurodevelopmental disorders	428495	Pathologic drug reaction resulting in brief psychotic states, with delusions (HCC)	ICD10CM	1	F19.150		Other psychoactive substance abuse with psychoactive s	IMO	GHS-PACDC	IMO_2015_R	ACTIVE	N	1900-01-01	2017-10-14	2015-07-10
Diagnosis: ICD10 Code, F00-F99 Define	F01-F99 Mental, Behavioral and Neurodevelopmental disorders	592068	Other psychoactive substance abuse with psychoactive substance-induced psychotic disorder w	ICD10CM	1	F19.151		Other psychoactive substance abuse with psychoactive s	IMO	GHS-PACDC	IMO_2015_R	ACTIVE	Y	1900-01-01	9999-12-31	2015-07-10
Diagnosis: ICD10 Code, F00-F99 Define	F01-F99 Mental, Behavioral and Neurodevelopmental disorders	427214	Psychoactive substance-induced organic intoxication, with hallucinations (HCC)	ICD10CM	1	F19.151		Other psychoactive substance abuse with psychoactive s	IMO	GHS-PACDC	IMO_2015_R	ACTIVE	N	1900-01-01	9999-12-31	2015-07-10
Diagnosis: ICD10 Code, F00-F99 Define	F01-F99 Mental, Behavioral and Neurodevelopmental disorders	611794	Other psychoactive substance abuse with other psychoactive substance-induced disorders	ICD10CM	1	F19.18		Other psychoactive substance abuse with other psychoa	IMO	GHS-PACDC	IMO_2015_R	ACTIVE	Y	1900-01-01	9999-12-31	2015-07-10
Diagnosis: ICD10 Code, F00-F99 Define	F01-F99 Mental, Behavioral and Neurodevelopmental disorders	972278	Phencyclidine-induced anxiety disorder with onset during intoxication, with delirium	ICD10CM	2	F19.180	F19.121,F19.180	Other psychoactive substance abuse with psychoactive s	IMO	GHS-PACDC	IMO_2015_R	DELETED	Y	1900-01-01	2016-05-07	2015-07-10
Diagnosis: ICD10 Code, F00-F99 Define	F01-F99 Mental, Behavioral and Neurodevelopmental disorders	974534	Onset of unknown substance-induced anxiety disorder during intoxication, with unspecified cor	ICD10CM	2	F19.180	F19.129,F19.180	Other psychoactive substance abuse with psychoactive s	IMO	GHS-PACDC	IMO_2015_R	DELETED	Y	1900-01-01	2016-05-07	2015-07-10
Diagnosis: ICD10 Code, F00-F99 Define	F01-F99 Mental, Behavioral and Neurodevelopmental disorders	593619	Other psychoactive substance abuse with psychoactive substance-induced sexual dysfunction	ICD10CM	1	F19.181		Other psychoactive substance abuse with psychoactive s	IMO	GHS-PACDC	IMO_2015_R	ACTIVE	Y	1900-01-01	9999-12-31	2015-07-10
Diagnosis: ICD10 Code, F00-F99 Define	F01-F99 Mental, Behavioral and Neurodevelopmental disorders	500600	Skin lesion due to intravenous drug abuse (HCC)	ICD10CM	2	F19.19	L98.9,F19.19	Other psychoactive substance abuse with unspecified ps	IMO	GHS-PACDC	IMO_2015_R	ACTIVE	N	1900-01-01	9999-12-31	2015-07-10
Diagnosis: ICD10 Code, F00-F99 Define	F01-F99 Mental, Behavioral and Neurodevelopmental disorders	595587	Other psychoactive substance abuse with unspecified psychoactive substance-induced disorder	ICD10CM	1	F19.19		Other psychoactive substance abuse with unspecified ps	IMO	GHS-PACDC	IMO_2015_R	ACTIVE	Y	1900-01-01	9999-12-31	2015-07-10
Diagnosis: ICD10 Code, F00-F99 Define	F01-F99 Mental, Behavioral and Neurodevelopmental disorders	508474	Flexeril use disorder, severe, dependence (HCC)	ICD10CM	1	F19.20		Other psychoactive substance dependence, uncomplicat	IMO	GHS-PACDC	IMO_2015_R	ACTIVE	N	2016-09-17	9999-12-31	2016-09-30
Diagnosis: ICD10 Code, F00-F99 Define	F01-F99 Mental, Behavioral and Neurodevelopmental disorders	508517	Severe propofol dependence in controlled environment (HCC)	ICD10CM	1	F19.20		Other psychoactive substance dependence, uncomplicat	IMO	GHS-PACDC	IMO_2015_R	ACTIVE	N	2016-09-17	9999-12-31	2016-09-30
Diagnosis: ICD10 Code, F00-F99 Define	F01-F99 Mental, Behavioral and Neurodevelopmental disorders	1103732	Drug dependence during puerperium, delivered (HCC)	ICD10CM	2	F19.20	O99.325,F19.20	Other psychoactive substance dependence, uncomplicat	IMO	GHS-PACDC	IMO_2016_R	ACTIVE	N	1900-01-01	9999-12-31	2016-06-02
Diagnosis: ICD10 Code, F00-F99 Define	F01-F99 Mental, Behavioral and Neurodevelopmental disorders	1098313	Other specified drug dependence, unspecified abuse	ICD10CM	1	F19.20		Other psychoactive substance dependence, uncomplicat	IMO	GHS-PACDC	IMO_2016_R	DELETED	Y	1900-01-01	9999-12-31	2016-06-02
Diagnosis: ICD10 Code, F00-F99 Define	F01-F99 Mental, Behavioral and Neurodevelopmental disorders	422081	Drug dependence of mother, with delivery, with mention of postpartum complication	ICD10CM	2	F19.20	O99.325,F19.20	Other psychoactive substance dependence, uncomplicat	IMO	GHS-PACDC	IMO_2015_R	DELETED	Y	1900-01-01	9999-12-31	2015-07-10
Diagnosis: ICD10 Code, F00-F99 Define	F01-F99 Mental, Behavioral and Neurodevelopmental disorders	216305	Polysubstance dependence, non-opioid, continuous (HCC)	ICD10CM	1	F19.20		Other psychoactive substance dependence, uncomplicat	IMO	GHS-PACDC	DX_LEGACY	ACTIVE	N	1900-01-01	9999-12-31	2015-07-10
Diagnosis: ICD10 Code, F00-F99 Define	F01-F99 Mental, Behavioral and Neurodevelopmental disorders	509103	Propofol use disorder, severe, in controlled environment, dependence (HCC)	ICD10CM	1	F19.20		Other psychoactive substance dependence, uncomplicat	IMO	GHS-PACDC	IMO_2015_R	ACTIVE	N	2016-09-17	9999-12-31	2016-09-30
Diagnosis: ICD10 Code, F00-F99 Define	F01-F99 Mental, Behavioral and Neurodevelopmental disorders	3166	Unspecified drug dependence, unspecified	ICD10CM	1	F19.20		Other psychoactive substance dependence, uncomplicat	IMO	GHS-PACDC	DX_LEGACY	ACTIVE	Y	1900-01-01	9999-12-31	2015-07-10
Diagnosis: ICD10 Code, F00-F99 Define	F01-F99 Mental, Behavioral and Neurodevelopmental disorders	479854	Severe cyclobenzaprine use disorder (HCC)	ICD10CM	1	F19.20		Other psychoactive substance dependence, uncomplicat	IMO	GHS-PACDC	IMO_2015_R	ACTIVE	N	2016-09-17	9999-12-31	2016-09-30
Diagnosis: ICD10 Code, F00-F99 Define	F01-F99 Mental, Behavioral and Neurodevelopmental disorders	359646	Polysubstance (excluding opioids) dependence without physiological dependence (HCC)	ICD10CM	1	F19.20		Other psychoactive substance dependence, uncomplicat	IMO	GHS-PACDC	DX_LEGACY	ACTIVE	N	1900-01-01	9999-12-31	2015-07-10
Diagnosis: ICD10 Code, F00-F99 Define	F01-F99 Mental, Behavioral and Neurodevelopmental disorders	683703	Chemical dependency complicating pregnancy, antepartum, first trimester (HCC)	ICD10CM	2	F19.20	O99.321,F19.20	Other psychoactive substance dependence, uncomplicat	IMO	GHS-PACDC	IMO_2015_R	ACTIVE	N	2016-04-10	9999-12-31	2015-07-10
Diagnosis: ICD10 Code, F00-F99 Define	F01-F99 Mental, Behavioral and Neurodevelopmental disorders	509079	Ketamine use disorder, moderate, dependence (HCC)	ICD10CM	1	F19.20		Other psychoactive substance dependence, uncomplicat	IMO	GHS-PACDC	IMO_2015_R	ACTIVE	N	2016-09-17	9999-12-31	2016-09-30
Diagnosis: ICD10 Code, F00-F99 Define	F01-F99 Mental, Behavioral and Neurodevelopmental disorders	1347620	Drug dependence of mother, with delivery, with mention of postpartum complication	ICD10CM	2	F19.20	O99.325,F19.20	Other psychoactive substance dependence, uncomplicat	IMO	GHS-PACDC	IMO_2018_R	DELETED	Y	1900-01-01	9999-12-31	2017-10-30
Diagnosis: ICD10 Code, F00-F99 Define	F01-F99 Mental, Behavioral and Neurodevelopmental disorders	674298	Drug dependence, antepartum, first trimester (HCC)	ICD10CM	2	F19.20	O99.321,F19.20,F19.21	Other psychoactive substance dependence, uncomplicat	IMO	GHS-PACDC	IMO_2015_R	ACTIVE	N	1900-01-01	2016-05-07	2015-07-10
Diagnosis: ICD10 Code, F00-F99 Define	F01-F99 Mental, Behavioral and Neurodevelopmental disorders	484193	Moderate dextromethorphan use disorder in controlled environment (HCC)	ICD10CM	1	F19.20		Other psychoactive substance dependence, uncomplicat	IMO	GHS-PACDC	IMO_2015_R	ACTIVE	N	2016-09-17	9999-12-31	2016-09-30
Diagnosis: ICD10 Code, F00-F99 Define	F01-F99 Mental, Behavioral and Neurodevelopmental disorders	315888	Phencyclidine dependence, daily use (HCC)	ICD10CM	1	F19.20		Other psychoactive substance dependence, uncomplicat	IMO	GHS-PACDC	DX_LEGACY	ACTIVE	N	1900-01-01	9999-12-31	2015-07-10
Diagnosis: ICD10 Code, F00-F99 Define	F01-F99 Mental, Behavioral and Neurodevelopmental disorders	431080	Chemical dependency complicating pregnancy in third trimester, antepartum (HCC)	ICD10CM	2	F19.20	O99.323,F19.20	Other psychoactive substance dependence, uncomplicat	IMO	GHS-PACDC	IMO_2015_R	ACTIVE	N	1900-01-01	9999-12-31	2015-07-10
Diagnosis: ICD10 Code, F00-F99 Define	F01-F99 Mental, Behavioral and Neurodevelopmental disorders	317954	Unknown substance dependence without physiological dependence (HCC)	ICD10CM	1	F19.20		Other psychoactive substance dependence, uncomplicat	IMO	GHS-PACDC	DX_LEGACY	ACTIVE	N	1900-01-01	9999-12-31	2015-07-10
Diagnosis: ICD10 Code, F00-F99 Define	F01-F99 Mental, Behavioral and Neurodevelopmental disorders	165285	Nose drop addiction (HCC)	ICD10CM	1	F19.20		Other psychoactive substance dependence, uncomplicat	IMO	GHS-PACDC	DX_LEGACY	ACTIVE	N	1900-01-01	9999-12-31	2015-07-10
Diagnosis: ICD10 Code, F00-F99 Define	F01-F99 Mental, Behavioral and Neurodevelopmental disorders	484819	Severe propofol use disorder in controlled environment (HCC)	ICD10CM	1	F19.20		Other psychoactive substance dependence, uncomplicat	IMO	GHS-PACDC	IMO_2015_R	ACTIVE	N	2016-09-17	9999-12-31	2016-09-30
Diagnosis: ICD10 Code, F00-F99 Define	F01-F99 Mental, Behavioral and Neurodevelopmental disorders	479912	Severe ketamine use disorder (HCC)	ICD10CM	1	F19.20		Other psychoactive substance dependence, uncomplicat	IMO	GHS-PACDC	IMO_2015_R	ACTIVE	N	2016-09-17	9999-12-31	2016-09-30
Diagnosis: ICD10 Code, F00-F99 Define	F01-F99 Mental, Behavioral and Neurodevelopmental disorders	518815	Drug dependence during pregnancy, antepartum (HCC)	ICD10CM	2	F19.20	O99.320,F19.20	Other psychoactive substance dependence, uncomplicat	IMO	GHS-PACDC	IMO_2015_R	ACTIVE	N	1900-01-01	9999-12-31	2015-07-10
Diagnosis: ICD10 Code, F00-F99 Define	F01-F99 Mental, Behavioral and Neurodevelopmental disorders	951908	Drug dependence of mother in pregnancy, first trimester (HCC)	ICD10CM	2	F19.20	O99.321,F19.20	Other psychoactive substance dependence, uncomplicat	IMO	GHS-PACDC	IMO_2015_R	ACTIVE	N	2016-04-10	9999-12-31	2015-07-10
Diagnosis: ICD10 Code, F00-F99 Define	F01-F99 Mental, Behavioral and Neurodevelopmental disorders	507397	Severe cyclobenzaprine dependence in controlled environment (HCC)	ICD10CM	1	F19.20		Other psychoactive substance dependence, uncomplicat	IMO	GHS-PACDC	IMO_2015_R	ACTIVE	N	2016-09-17	9999-12-31	2016-09-30
Diagnosis: ICD10 Code, F00-F99 Define	F01-F99 Mental, Behavioral and Neurodevelopmental disorders	951908	Drug dependence of mother in pregnancy, first trimester (HCC)	ICD10CM	2	F19.20	O99.321,F19.20,F19.21	Other psychoactive substance dependence, uncomplicat	IMO	GHS-PACDC	IMO_2015_R	ACTIVE	N	1900-01-01	2016-05-07	2015-07-10
Diagnosis: ICD10 Code, F00-F99 Define	F01-F99 Mental, Behavioral and Neurodevelopmental disorders	380514	Active drug dependence (HCC)	ICD10CM	1	F19.20		Other psychoactive substance dependence, uncomplicat	IMO	GHS-PACDC	DX_LEGACY	ACTIVE	N	1900-01-01	9999-12-31	2015-07-10
Diagnosis: ICD10 Code, F00-F99 Define	F01-F99 Mental, Behavioral and Neurodevelopmental disorders	135384	Absinthe addiction (HCC)	ICD10CM	1	F19.20		Other psychoactive substance dependence, uncomplicat	IMO	GHS-PACDC	DX_LEGACY	ACTIVE	N	1900-01-01	9999-12-31	2015-07-10
Diagnosis: ICD10 Code, F00-F99 Define	F01-F99 Mental, Behavioral and Neurodevelopmental disorders	374177	Polysubstance (including opioids) dependence w/o physiol dependence (HCC)	ICD10CM	1	F19.20		Other psychoactive substance dependence, uncomplicat	IMO	GHS-PACDC	DX_LEGACY	ACTIVE	N	1900-01-01	9999-12-31	2015-07-10
Diagnosis: ICD10 Code, F00-F99 Define	F01-F99 Mental, Behavioral and Neurodevelopmental disorders	334012	Combinations of drug dependence excluding opioid type drug, episodic use (HCC)	ICD10CM	1	F19.20		Other psychoactive substance dependence, uncomplicat	IMO	GHS-PACDC	DX_LEGACY	ACTIVE	N	1900-01-01	9999-12-31	2015-07-10
Diagnosis: ICD10 Code, F00-F99 Define	F01-F99 Mental, Behavioral and Neurodevelopmental disorders	317514	Polysubstance dependence including opioid type drug, continuous use (HCC)	ICD10CM	2	F19.20	F11.20,F19.20	Other psychoactive substance dependence, uncomplicat	IMO	GHS-PACDC	DX_LEGACY	ACTIVE	N	2016-04-10	9999-12-31	2015-07-10
Diagnosis: ICD10 Code, F00-F99 Define	F01-F99 Mental, Behavioral and Neurodevelopmental disorders	526550	Combined opioid and non-opioid drug dependence, in remission (HCC)	ICD10CM	2	F19.21	F11.21,F19.21	Other psychoactive substance dependence, in remission	IMO	GHS-PACDC	IMO_2015_R	ACTIVE	N	1900-01-01	9999-12-31	2015-07-10
Diagnosis: ICD10 Code, F00-F99 Define	F01-F99 Mental, Behavioral and Neurodevelopmental disorders	507487	Severe dextromethorphan dependence in sustained remission on maintenance therapy (HCC)	ICD10CM	1	F19.21		Other psychoactive substance dependence, in remission	IMO	GHS-PACDC	IMO_2015_R	ACTIVE	N	2016-09-17	9999-12-31	2016-09-30
Diagnosis: ICD10 Code, F00-F99 Define	F01-F99 Mental, Behavioral and Neurodevelopmental disorders	484725	Moderate cyclobenzaprine use disorder in sustained remission (HCC)	ICD10CM	1	F19.21		Other psychoactive substance dependence, in remission	IMO	GHS-PACDC	IMO_2015_R	ACTIVE	N	2016-09-17	9999-12-31	2016-09-30
Diagnosis: ICD10 Code, F00-F99 Define	F01-F99 Mental, Behavioral and Neurodevelopmental disorders	509174	Propofol use disorder, moderate, in sustained remission, dependence (HCC)	ICD10CM	1	F19.21		Other psychoactive substance dependence, in remission	IMO	GHS-PACDC	IMO_2015_R	ACTIVE	N	2016-09-17	9999-12-31	2016-09-30
Diagnosis: ICD10 Code, F00-F99 Define	F01-F99 Mental, Behavioral and Neurodevelopmental disorders	676987	Drug dependence of mother in pregnancy, antepartum, third trimester (HCC)	ICD10CM	3	F19.21	O99.323,F19.20,F19.21	Other psychoactive substance dependence, in remission	IMO	GHS-PACDC	IMO_2015_R	ACTIVE	N	1900-01-01	2016-05-07	2015-07-10
Diagnosis: ICD10 Code, F00-F99 Define	F01-F99 Mental, Behavioral and Neurodevelopmental disorders	702580	Drug dependence of mother in pregnancy, antepartum, second trimester (HCC)	ICD10CM	3	F19.21	O99.322,F19.20,F19.21	Other psychoactive substance dependence, in remission	IMO	GHS-PACDC	IMO_2015_R	ACTIVE	N	1900-01-01	2016-05-07	2015-07-10
Diagnosis: ICD10 Code, F00-F99 Define	F01-F99 Mental, Behavioral and Neurodevelopmental disorders	507270	Moderate propofol dependence in sustained remission (HCC)	ICD10CM	1	F19.21		Other psychoactive substance dependence, in remission	IMO	GHS-PACDC	IMO_2015_R	ACTIVE	N	2016-09-17	9999-12-31	2016-09-30
Diagnosis: ICD10 Code, F00-F99 Define	F01-F99 Mental, Behavioral and Neurodevelopmental disorders	944200	Drug dependence of mother in pregnancy, second trimester (HCC)	ICD10CM	3	F19.21	O99.322,F19.20,F19.21	Other psychoactive substance dependence, in remission	IMO	GHS-PACDC	IMO_2015_R	ACTIVE	N	1900-01-01	2016-05-07	2015-07-10
Diagnosis: ICD10 Code, F00-F99 Define	F01-F99 Mental, Behavioral and Neurodevelopmental disorders	507521	Moderate ketamine dependence in sustained remission (HCC)	ICD10CM	1	F19.21		Other psychoactive substance dependence, in remission	IMO	GHS-PACDC	IMO_2015_R	ACTIVE	N	2016-09-17	9999-12-31	2016-09-30
Diagnosis: ICD10 Code, F00-F99 Define	F01-F99 Mental, Behavioral and Neurodevelopmental disorders	509353	Moderate dextromethorphan dependence in early remission in controlled environment (HCC)	ICD10CM	1	F19.21		Other psychoactive substance dependence, in remission	IMO	GHS-PACDC	IMO_2015_R	ACTIVE	N	2016-09-17	9999-12-31	2016-09-30
Diagnosis: ICD10 Code, F00-F99 Define	F01-F99 Mental, Behavioral and Neurodevelopmental disorders	641889	Oth psychoactive substance dependence w intoxication, uncomp	ICD10CM	1	F19.220		Other psychoactive substance dependence with intoxica	IMO	GHS-PACDC	IMO_2015_R	DELETED	Y	1900-01-01	9999-12-31	2015-07-10
Diagnosis: ICD10 Code, F00-F99 Define	F01-F99 Mental, Behavioral and Neurodevelopmental disorders	461788	Psychoactive substance-induced organic withdrawal without complication (HCC)	ICD10CM	1	F19.230		Other psychoactive substance dependence with withdra	IMO	GHS-PACDC	IMO_2015_R	ACTIVE	N	2017-09-17	9999-12-31	2017-10-30
Diagnosis: ICD10 Code, F00-F99 Define	F01-F99 Mental, Behavioral and Neurodevelopmental disorders	962053	Steroid withdrawal syndrome, uncomplicated (HCC)	ICD10CM	1	F19.230		Other psychoactive substance dependence with withdra	IMO	GHS-PACDC	IMO_2015_R	ACTIVE	N	1900-01-01	9999-12-31	2015-07-10
Diagnosis: ICD10 Code, F00-F99 Define	F01-F99 Mental, Behavioral and Neurodevelopmental disorders	458966	Acute narcotic withdrawal without complication (HCC)	ICD10CM	1	F19.230		Other psychoactive substance dependence with withdra	IMO	GHS-PACDC	IMO_2015_R	ACTIVE	N	1900-01-01	9999-12-31	2015-07-10
Diagnosis: ICD10 Code, F00-F99 Define	F01-F99 Mental, Behavioral and Neurodevelopmental disorders	452157	Paroxetine withdrawal syndrome without complication (HCC)	ICD10CM	1	F19.230		Other psychoactive substance dependence with withdra	IMO	GHS-PACDC	IMO_2015_R	ACTIVE	N	1900-01-01	9999-12-31	2015-07-10
Diagnosis: ICD10 Code, F00-F99 Define	F01-F99 Mental, Behavioral and Neurodevelopmental disorders	1083623	Cannabis-induced psychotic disorder with mild use disorder with onset during withdrawal with	ICD10CM	3	F19.230	F12.250,F19.239,F19.230,F19.232	Other psychoactive substance dependence with withdra	IMO	GHS-PACDC	IMO_2015_R	ACTIVE	N	1900-01-01	2016-05-07	2015-07-10
Diagnosis: ICD10 Code, F00-F99 Define	F01-F99 Mental, Behavioral and Neurodevelopmental disorders	593062	Other psychoactive substance dependence with withdrawal delirium	ICD10CM	1	F19.231		Other psychoactive substance dependence with withdra	IMO	GHS-PACDC	IMO_2015_R	ACTIVE	Y	1900-01-01	9999-12-31	2015-07-10
Diagnosis: ICD10 Code, F00-F99 Define	F01-F99 Mental, Behavioral and Neurodevelopmental disorders	1082033	Cannabis-induced psychotic disorder without use disorder with onset during withdrawal with cc	ICD10CM	5	F19.231	F19.250,F19.232,F19.230,F19.239	Other psychoactive substance dependence with withdra	IMO	GHS-PACDC	IMO_2015_R	DELETED	Y	1900-01-01	2016-05-07	2015-07-10
Diagnosis: ICD10 Code, F00-F99 Define	F01-F99 Mental, Behavioral and Neurodevelopmental disorders	956138	Drug withdrawal headache, with delirium (HCC)	ICD10CM	1	F19.231		Other psychoactive substance dependence with withdra	IMO	GHS-PACDC	IMO_2015_R	ACTIVE	N	1900-01-01	9999-12-31	2015-07-10
Diagnosis: ICD10 Code, F00-F99 Define	F01-F99 Mental, Behavioral and Neurodevelopmental disorders	955777	Paxil withdrawal syndrome (properly prescribed and administered), with delirium (HCC)	ICD10CM	1	F19.231		Other psychoactive substance dependence with withdra	IMO	GHS-PACDC	IMO_2015_R	ACTIVE	N	1900-01-01	2016-05-07	2015-07-10
Diagnosis: ICD10 Code, F00-F99 Define	F01-F99 Mental, Behavioral and Neurodevelopmental disorders	1082482	Cannabis-induced psychotic disorder without use disorder with onset during withdrawal with cc	ICD10CM	2	F19.231	F19.251,F19.231,F19.239,F19.232	Other psychoactive substance dependence with withdra	IMO	GHS-PACDC	IMO_2015_R	DELETED	Y	1900-01-01	2016-05-07	2015-07-10
Diagnosis: ICD10 Code, F00-F99 Define	F01-F99 Mental, Behavioral and Neurodevelopmental disorders	981682	Psychoactive substance-induced organic withdrawal with complication, with delirium (HCC)	ICD10CM	1	F19.231		Other psychoactive substance dependence with withdra	IMO	GHS-PACDC	IMO_2015_R	ACTIVE	N	2016-09-17	9999-12-31	2016-09-30
Diagnosis: ICD10 Code, F00-F99 Define	F01-F99 Mental, Behavioral and Neurodevelopmental disorders	600230	Other psychoactive substance dependence with withdrawal with perceptual disturbance	ICD10CM	1	F19.232		Other psychoactive substance dependence with withdra	IMO	GHS-PACDC	IMO_2015_R	ACTIVE	Y	1900-01-01	9999-12-31	2015-07-10
Diagnosis: ICD10 Code, F00-F99 Define	F01-F99 Mental, Behavioral and Neurodevelopmental disorders	1083357	Cannabis-induced psychotic disorder without use disorder with onset during withdrawal with cc	ICD10CM	4	F19.232	F19.259,F19.239,F19.231,F19.232	Other psychoactive substance dependence with withdra	IMO	GHS-PACDC	IMO_2015_R	DELETED	Y	1900-01-01	2016-05-07	2015-07-10
Diagnosis: ICD10 Code, F00-F99 Define	F01-F99 Mental, Behavioral and Neurodevelopmental disorders	953868	Paroxetine withdrawal syndrome (properly prescribed and administered), with perceptual distu	ICD10CM	1	F19.232	F19.232,T43.225A	Other psychoactive substance dependence with withdra	IMO	GHS-PACDC	IMO_2015_R	ACTIVE	N	2016-04-10	2016-10-14	2015-07-10
Diagnosis: ICD10 Code, F00-F99 Define	F01-F99 Mental, Behavioral and Neurodevelopmental disorders	517447	Moderate cannabis withdrawal with perceptual disturbance (HCC)	ICD10CM	1	F19.232		Other psychoactive substance dependence with withdra	IMO	GHS-PACDC	IMO_2015_R	ACTIVE	N	1900-01-01	9999-12-31	2015-07-10
Diagnosis: ICD10 Code, F00-F99 Define	F01-F99 Mental, Behavioral and Neurodevelopmental disorders	959731	Drug withdrawal headache, with perceptual disturbance (HCC)	ICD10CM	1	F19.232		Other psychoactive substance dependence with withdra	IMO	GHS-PACDC	IMO_2015_R	ACTIVE	N	1900-01-01	9999-12-31	2015-07-10
Diagnosis: ICD10 Code, F00-F99 Define	F01-F99 Mental, Behavioral and Neurodevelopmental disorders	960319	Drug withdrawal seizure, with perceptual disturbance (HCC)	ICD10CM	1	F19.232		Other psychoactive substance dependence with withdra	IMO	GHS-PACDC	IMO_2015_R	ACTIVE	N	1900-01-01	9999-12-31	2015-07-10
Diagnosis: ICD10 Code, F00-F99 Define	F01-F99 Mental, Behavioral and Neurodevelopmental disorders	428006	Substance abuse withdrawal, with perceptual disturbance (HCC)	ICD10CM	1	F19.232		Other psychoactive substance dependence with withdra	IMO	GHS-PACDC	IMO_2015_R	ACTIVE	N	1900-01-01	9999-12-31	2015-07-10
Diagnosis: ICD10 Code, F00-F99 Define	F01-F99 Mental, Behavioral and Neurodevelopmental disorders	1020387	Drug withdrawal headache with complication, with perceptual disturbance (HCC)	ICD10CM	1	F19.232		Other psychoactive substance dependence with withdra	IMO	GHS-PACDC	IMO_2015_R	ACTIVE	N	1900-01-01	9999-12-31	2015-07-10
Diagnosis: ICD10 Code, F00-F99 Define	F01-F99 Mental, Behavioral and Neurodevelopmental disorders	1027036	Acute narcotic withdrawal with complication, with perceptual disturbance (HCC)	ICD10CM	1	F19.232		Other psychoactive substance dependence with withdra	IMO	GHS-PACDC	IMO_2015_R	ACTIVE	N	1900-01-01	9999-12-31	2015-07-10
Diagnosis: ICD10 Code, F00-F99 Define	F01-F99 Mental, Behavioral and Neurodevelopmental disorders	1083623	Cannabis-induced psychotic disorder with mild use disorder with onset during withdrawal with	ICD10CM	2	F19.239	F12.250,F19.239,F19.230,F19.232	Other psychoactive substance dependence with withdra	IMO	GHS-PACDC	IMO_2015_R	ACTIVE	N	1900-01-01	2016-05-07	2015-07-10
Diagnosis: ICD10 Code, F00-F99 Define	F01-F99 Mental, Behavioral and Neurodevelopmental disorders	448140	Steroid withdrawal syndrome with complication (HCC)	ICD10CM	1	F19.239		Other psychoactive substance dependence with withdra	IMO	GHS-PACDC	IMO_2015_R	ACTIVE	N	1900-01-01	9999-12-31	2015-07-10
Diagnosis: ICD10 Code, F00-F99 Define	F01-F99 Mental, Behavioral and Neurodevelopmental disorders	960330	Paroxetine withdrawal syndrome (properly prescribed and administered), with unspecified com	ICD10CM	1	F19.239		Other psychoactive substance dependence with withdra	IMO	GHS-PACDC	IMO_2015_R	ACTIVE	N	1900-01-01	2016-05-07	2015-07-10
Diagnosis: ICD10 Code, F00-F99 Define	F01-F99 Mental, Behavioral and Neurodevelopmental disorders	982234	Acute drug withdrawal syndrome with complication, with unspecified complication (HCC)	ICD10CM	1	F19.239		Other psychoactive substance dependence with withdra	IMO	GHS-PACDC	IMO_2015_R	ACTIVE	N	1900-01-01	9999-12-31	2015-07-10
Diagnosis: ICD10 Code, F00-F99 Define	F01-F99 Mental, Behavioral and Neurodevelopmental disorders	165279	Paxil withdrawal syndrome (properly prescribed and administered) (HCC)	ICD10CM	1	F19.239		Other psychoactive substance dependence with withdra	IMO	GHS-PACDC	DX_LEGACY	ACTIVE	N	1900-01-01	9999-12-31	2015-07-10
Diagnosis: ICD10 Code, F00-F99 Define	F01-F99 Mental, Behavioral and Neurodevelopmental disorders	1082033	Cannabis-induced psychotic disorder without use disorder with onset during withdrawal with cc	ICD10CM	4	F19.239	F19.250,F19.232,F19.230,F19.239	Other psychoactive substance dependence with withdra	IMO	GHS-PACDC	IMO_2015_R	DELETED	Y	1900-01-01	2016-05-07	2015-07-10
Diagnosis: ICD10 Code, F00-F99 Define	F01-F99 Mental, Behavioral and Neurodevelopmental disorders	429115	Onset of amphetamine-induced mood disorder during withdrawal (HCC)	ICD10CM	1	F19.239	F19.239,F19.24	Other psychoactive substance dependence with withdra	IMO	GHS-PACDC	IMO_2015_R	ACTIVE	N	1900-01-01	2017-10-14	2015-07-10
Diagnosis: ICD10 Code, F00-F99 Define	F01-F99 Mental, Behavioral and Neurodevelopmental disorders	1356920	Other psychoactive substance dependence with withdrawal (HCC)	ICD10CM	1	F19.239		Other psychoactive substance dependence with withdra	IMO	GHS-PACDC	IMO_2018_R	ACTIVE	N	1900-01-01	9999-12-31	2017-10-30
Diagnosis: ICD10 Code, F00-F99 Define	F01-F99 Mental, Behavioral and Neurodevelopmental disorders	324409	Drug withdrawal seizure (HCC)	ICD10CM	1	F19.239		Other psychoactive substance dependence with withdra	IMO	GHS-PACDC	DX_LEGACY	ACTIVE	N	1900-01-01	2016-05-07	2015-07-10
Diagnosis: ICD10 Code, F00-F99 Define	F01-F99 Mental, Behavioral and Neurodevelopmental disorders	1367031	Oth psychoactive substance dependence w mood disorder (HCC)	ICD10CM	1	F19.24		Other psychoactive substance dependence with psychoa	IMO	GHS-PACDC	IMO_2018_R	ACTIVE	N	1900-01-01	9999-12-31	2017-10-30
Diagnosis: ICD10 Code, F00-F99 Define	F01-F99 Mental, Behavioral and Neurodevelopmental disorders	1351976	Unknown substance-induced psychotic disorder with onset during withdrawal with perceptual c	ICD10CM	2	F19.259	F19.232,F19.259	Other psychoactive substance dependence with psychoa	IMO	GHS-PACDC	IMO_2018_R	ACTIVE	N	1900-01-01	9999-12-31	2017-10-30
Diagnosis: ICD10 Code, F00-F99 Define	F01-F99 Mental, Behavioral and Neurodevelopmental disorders	973916	Unknown substance-induced psychotic disorder with onset during withdrawal, with unspecified	ICD10CM	2	F19.259	F19.239,F19.259	Other psychoactive substance dependence with psychoa	IMO	GHS-PACDC	IMO_2015_R	ACTIVE	N	2017-09-17	9999-12-31	2017-10-30
Diagnosis: ICD10 Code, F00-F99 Define	F01-F99 Mental, Behavioral and Neurodevelopmental disorders	1356921	Other psychoactive substance dependence with psychoactive substance-induced psychotic disor	ICD10CM	1	F19.259		Other psychoactive substance dependence with psychoa	IMO	GHS-PACDC	IMO_2018_R	ACTIVE	N	1900-01-01	9999-12-31	2017-10-30

Diagnosis: ICD10 Code, F00-F99 Define	F01-F99 Mental, Behavioral and Neurodevelopmental disorders	1178437	Other psychoactive substance dependence with psychoactive substance-induced persisting dem	ICD10CM	1	F19.27		Other psychoactive substance dependence with psychoa	IMO	GHS-PACDC	IMO_2016_R	ACTIVE	N	1900-01-01	9999-12-31	2016-06-02
Diagnosis: ICD10 Code, F00-F99 Define	F01-F99 Mental, Behavioral and Neurodevelopmental disorders	641922	Oth psychoactive substance dependence w oth disorder	ICD10CM	1	F19.288		Other psychoactive substance dependence with other ps	IMO	GHS-PACDC	IMO_2015_R	DELETED	Y	1900-01-01	9999-12-31	2015-07-10
Diagnosis: ICD10 Code, F00-F99 Define	F01-F99 Mental, Behavioral and Neurodevelopmental disorders	320717	H/O drug abuse	ICD10CM	1	F19.90		Other psychoactive substance use, unspecified, uncomp	IMO	GHS-PACDC	DX_LEGACY	ACTIVE	N	1900-01-01	2016-05-07	2015-07-10
Diagnosis: ICD10 Code, F00-F99 Define	F01-F99 Mental, Behavioral and Neurodevelopmental disorders	484611	Mild dextromethorphan use disorder in controlled environment	ICD10CM	1	F19.90		Other psychoactive substance use, unspecified, uncomp	IMO	GHS-PACDC	IMO_2015_R	ACTIVE	N	1900-01-01	2016-10-14	2015-07-10
Diagnosis: ICD10 Code, F00-F99 Define	F01-F99 Mental, Behavioral and Neurodevelopmental disorders	1079765	Intravenous drug use during pregnancy, antepartum, second trimester	ICD10CM	2	F19.90	O99.322,F19.90	Other psychoactive substance use, unspecified, uncomp	IMO	GHS-PACDC	IMO_2015_R	ACTIVE	N	1900-01-01	9999-12-31	2015-07-10
Diagnosis: ICD10 Code, F00-F99 Define	F01-F99 Mental, Behavioral and Neurodevelopmental disorders	1298328	Uses drugs daily	ICD10CM	1	F19.90		Other psychoactive substance use, unspecified, uncomp	IMO	GHS-PACDC	IMO_2017_R	ACTIVE	N	1900-01-01	9999-12-31	2017-04-14
Diagnosis: ICD10 Code, F00-F99 Define	F01-F99 Mental, Behavioral and Neurodevelopmental disorders	520112	Harmful use of non-dependence producing substance	ICD10CM	1	F19.90		Other psychoactive substance use, unspecified, uncomp	IMO	GHS-PACDC	IMO_2015_R	ACTIVE	N	1900-01-01	9999-12-31	2015-07-10
Diagnosis: ICD10 Code, F00-F99 Define	F01-F99 Mental, Behavioral and Neurodevelopmental disorders	165230	Psychoactive substance use disorder	ICD10CM	1	F19.90		Other psychoactive substance use, unspecified, uncomp	IMO	GHS-PACDC	DX_LEGACY	ACTIVE	N	2016-09-17	9999-12-31	2016-09-30
Diagnosis: ICD10 Code, F00-F99 Define	F01-F99 Mental, Behavioral and Neurodevelopmental disorders	596152	Other psychoactive substance use, unspecified with intoxication with delirium	ICD10CM	1	F19.921		Other psychoactive substance use, unspecified with into	IMO	GHS-PACDC	IMO_2015_R	ACTIVE	Y	1900-01-01	9999-12-31	2015-07-10
Diagnosis: ICD10 Code, F00-F99 Define	F01-F99 Mental, Behavioral and Neurodevelopmental disorders	488779	Medication-induced delirium, acute, mixed level of activity (HCC)	ICD10CM	1	F19.921		Other psychoactive substance use, unspecified with into	IMO	GHS-PACDC	IMO_2015_R	ACTIVE	N	1900-01-01	9999-12-31	2015-07-10
Diagnosis: ICD10 Code, F00-F99 Define	F01-F99 Mental, Behavioral and Neurodevelopmental disorders	2836	Pathological drug intoxication (HCC)	ICD10CM	1	F19.929		Other psychoactive substance use, unspecified with into	IMO	GHS-PACDC	DX_LEGACY	ACTIVE	N	2017-09-17	9999-12-31	2017-10-30
Diagnosis: ICD10 Code, F00-F99 Define	F01-F99 Mental, Behavioral and Neurodevelopmental disorders	957798	Psychoactive substance-induced organic intoxication, with unspecified complication (HCC)	ICD10CM	1	F19.929		Other psychoactive substance use, unspecified with into	IMO	GHS-PACDC	IMO_2015_R	ACTIVE	N	2017-09-17	9999-12-31	2017-10-30
Diagnosis: ICD10 Code, F00-F99 Define	F01-F99 Mental, Behavioral and Neurodevelopmental disorders	1030775	Pathological drug intoxication with complication, with unspecified complication (HCC)	ICD10CM	1	F19.929		Other psychoactive substance use, unspecified with into	IMO	GHS-PACDC	IMO_2015_R	ACTIVE	N	2016-09-17	9999-12-31	2016-09-30
Diagnosis: ICD10 Code, F00-F99 Define	F01-F99 Mental, Behavioral and Neurodevelopmental disorders	972100	Unknown substance-induced anxiety disorder with onset during intoxication, with unspecified c	ICD10CM	2	F19.929	F19.129,F19.929	Other psychoactive substance use, unspecified with into	IMO	GHS-PACDC	IMO_2015_R	DELETED	Y	2016-04-10	9999-12-31	2015-07-10
Diagnosis: ICD10 Code, F00-F99 Define	F01-F99 Mental, Behavioral and Neurodevelopmental disorders	461788	Psychoactive substance-induced organic withdrawal without complication (HCC)	ICD10CM	1	F19.930		Other psychoactive substance use, unspecified with with	IMO	GHS-PACDC	IMO_2015_R	ACTIVE	N	1900-01-01	2017-10-14	2015-07-10
Diagnosis: ICD10 Code, F00-F99 Define	F01-F99 Mental, Behavioral and Neurodevelopmental disorders	955342	Multiple substance withdrawal, with delirium (HCC)	ICD10CM	1	F19.931		Other psychoactive substance use, unspecified with with	IMO	GHS-PACDC	IMO_2015_R	ACTIVE	N	1900-01-01	2017-10-14	2015-07-10
Diagnosis: ICD10 Code, F00-F99 Define	F01-F99 Mental, Behavioral and Neurodevelopmental disorders	956589	Severe drug withdrawal syndrome, with delirium (HCC)	ICD10CM	1	F19.931		Other psychoactive substance use, unspecified with with	IMO	GHS-PACDC	IMO_2015_R	ACTIVE	N	1900-01-01	2017-10-14	2015-07-10
Diagnosis: ICD10 Code, F00-F99 Define	F01-F99 Mental, Behavioral and Neurodevelopmental disorders	1006315	Drug withdrawal syndrome with complication, with perceptual disturbance (HCC)	ICD10CM	1	F19.932		Other psychoactive substance use, unspecified with with	IMO	GHS-PACDC	IMO_2015_R	ACTIVE	N	1900-01-01	9999-12-31	2015-07-10
Diagnosis: ICD10 Code, F00-F99 Define	F01-F99 Mental, Behavioral and Neurodevelopmental disorders	1014178	Irritability due to drug withdrawal syndrome with complication, with unspecified complication (	ICD10CM	1	F19.939		Other psychoactive substance use, unspecified with with	IMO	GHS-PACDC	IMO_2015_R	ACTIVE	N	1900-01-01	2016-10-14	2015-07-10
Diagnosis: ICD10 Code, F00-F99 Define	F01-F99 Mental, Behavioral and Neurodevelopmental disorders	961434	Psychoactive substance-induced organic withdrawal, with unspecified complication (HCC)	ICD10CM	1	F19.939		Other psychoactive substance use, unspecified with with	IMO	GHS-PACDC	IMO_2015_R	ACTIVE	N	1900-01-01	2017-10-14	2015-07-10
Diagnosis: ICD10 Code, F00-F99 Define	F01-F99 Mental, Behavioral and Neurodevelopmental disorders	60410	Drug withdrawal syndrome (HCC)	ICD10CM	1	F19.939		Other psychoactive substance use, unspecified with with	IMO	GHS-PACDC	DX_LEGACY	ACTIVE	N	1900-01-01	9999-12-31	2015-07-10
Diagnosis: ICD10 Code, F00-F99 Define	F01-F99 Mental, Behavioral and Neurodevelopmental disorders	1114784	Abstinent from drug misuse (HCC)	ICD10CM	1	F19.939		Other psychoactive substance use, unspecified with with	IMO	GHS-PACDC	IMO_2016_R	ACTIVE	N	1900-01-01	9999-12-31	2016-06-02
Diagnosis: ICD10 Code, F00-F99 Define	F01-F99 Mental, Behavioral and Neurodevelopmental disorders	971817	Other psychoactive substance-induced mood disorder (HCC)	ICD10CM	1	F19.94		Other psychoactive substance use, unspecified with psyc	IMO	GHS-PACDC	IMO_2015_R	DELETED	Y	1900-01-01	9999-12-31	2015-07-10
Diagnosis: ICD10 Code, F00-F99 Define	F01-F99 Mental, Behavioral and Neurodevelopmental disorders	315878	Amphetamine and psychostimulant-induced mood disorder (HCC)	ICD10CM	1	F19.94		Other psychoactive substance use, unspecified with psyc	IMO	GHS-PACDC	DX_LEGACY	ACTIVE	N	1900-01-01	9999-12-31	2015-07-10
Diagnosis: ICD10 Code, F00-F99 Define	F01-F99 Mental, Behavioral and Neurodevelopmental disorders	975207	Onset of unknown substance-induced psychotic disorder during withdrawal, with delusions (HC	ICD10CM	1	F19.950	F19.950,F19.239	Other psychoactive substance use, unspecified with psyc	IMO	GHS-PACDC	IMO_2015_R	ACTIVE	N	2016-04-10	2016-10-14	2015-07-10
Diagnosis: ICD10 Code, F00-F99 Define	F01-F99 Mental, Behavioral and Neurodevelopmental disorders	957636	Organic brain syndrome associated with consumption of drugs, with delusions	ICD10CM	1	F19.950		Other psychoactive substance use, unspecified with psyc	IMO	GHS-PACDC	IMO_2015_R	DELETED	Y	1900-01-01	9999-12-31	2015-07-10
Diagnosis: ICD10 Code, F00-F99 Define	F01-F99 Mental, Behavioral and Neurodevelopmental disorders	1038459	Steroid-induced psychosis with complication, with delusions (HCC)	ICD10CM	1	F19.950		Other psychoactive substance use, unspecified with psyc	IMO	GHS-PACDC	IMO_2015_R	ACTIVE	N	1900-01-01	9999-12-31	2015-07-10
Diagnosis: ICD10 Code, F00-F99 Define	F01-F99 Mental, Behavioral and Neurodevelopmental disorders	972244	Unknown substance-induced psychotic disorder with onset during withdrawal, with delusions (H	ICD10CM	1	F19.950	F19.950,F19.239	Other psychoactive substance use, unspecified with psyc	IMO	GHS-PACDC	IMO_2015_R	ACTIVE	N	2016-04-10	2016-10-14	2015-07-10
Diagnosis: ICD10 Code, F00-F99 Define	F01-F99 Mental, Behavioral and Neurodevelopmental disorders	1031740	Steroid-induced psychosis with complication, with hallucinations (HCC)	ICD10CM	1	F19.951		Other psychoactive substance use, unspecified with psyc	IMO	GHS-PACDC	IMO_2015_R	ACTIVE	N	1900-01-01	9999-12-31	2015-07-10
Diagnosis: ICD10 Code, F00-F99 Define	F01-F99 Mental, Behavioral and Neurodevelopmental disorders	955699	Pathologic drug reaction resulting in brief psychotic states, with hallucinations (HCC)	ICD10CM	1	F19.951		Other psychoactive substance use, unspecified with psyc	IMO	GHS-PACDC	IMO_2015_R	ACTIVE	N	2017-09-17	9999-12-31	2017-10-30
Diagnosis: ICD10 Code, F00-F99 Define	F01-F99 Mental, Behavioral and Neurodevelopmental disorders	83533	Drug hallucinosis (HCC)	ICD10CM	1	F19.951		Other psychoactive substance use, unspecified with psyc	IMO	GHS-PACDC	DX_LEGACY	ACTIVE	N	1900-01-01	9999-12-31	2015-07-10
Diagnosis: ICD10 Code, F00-F99 Define	F01-F99 Mental, Behavioral and Neurodevelopmental disorders	1366285	Oth psychoactv substance use, unsp w psych disorder, unsp (HCC)	ICD10CM	1	F19.959		Other psychoactive substance use, unspecified with psyc	IMO	GHS-PACDC	IMO_2018_R	ACTIVE	N	1900-01-01	9999-12-31	2017-10-30
Diagnosis: ICD10 Code, F00-F99 Define	F01-F99 Mental, Behavioral and Neurodevelopmental disorders	974461	Substance or medication-induced psychotic disorder with onset during intoxication, with unspe	ICD10CM	2	F19.959	F19.929,F19.959	Other psychoactive substance use, unspecified with psyc	IMO	GHS-PACDC	IMO_2015_R	ACTIVE	N	2017-09-17	9999-12-31	2017-10-30
Diagnosis: ICD10 Code, F00-F99 Define	F01-F99 Mental, Behavioral and Neurodevelopmental disorders	423631	Drug-induced persisting amnestic disorder (HCC)	ICD10CM	1	F19.96		Other psychoactive substance use, unspecified with psyc	IMO	GHS-PACDC	IMO_2015_R	ACTIVE	N	1900-01-01	9999-12-31	2015-07-10
Diagnosis: ICD10 Code, F00-F99 Define	F01-F99 Mental, Behavioral and Neurodevelopmental disorders	61272	Drug-induced amnestic syndrome (HCC)	ICD10CM	1	F19.96		Other psychoactive substance use, unspecified with psyc	IMO	GHS-PACDC	DX_LEGACY	ACTIVE	N	1900-01-01	9999-12-31	2015-07-10
Diagnosis: ICD10 Code, F00-F99 Define	F01-F99 Mental, Behavioral and Neurodevelopmental disorders	61270	Drug-induced dementia (HCC)	ICD10CM	1	F19.97		Other psychoactive substance use, unspecified with psyc	IMO	GHS-PACDC	DX_LEGACY	ACTIVE	N	1900-01-01	9999-12-31	2015-07-10
Diagnosis: ICD10 Code, F00-F99 Define	F01-F99 Mental, Behavioral and Neurodevelopmental disorders	518272	Unknown substance-induced anxiety disorder with onset during intoxication with complication	ICD10CM	1	F19.980	F19.980,F19.929	Other psychoactive substance use, unspecified with psyc	IMO	GHS-PACDC	IMO_2015_R	ACTIVE	N	1900-01-01	9999-12-31	2015-07-10
Diagnosis: ICD10 Code, F00-F99 Define	F01-F99 Mental, Behavioral and Neurodevelopmental disorders	387116	Phencyclidine (PCP)-induced anxiety disorder (HCC)	ICD10CM	1	F19.980		Other psychoactive substance use, unspecified with psyc	IMO	GHS-PACDC	DX_LEGACY	ACTIVE	N	1900-01-01	2016-05-07	2015-07-10
Diagnosis: ICD10 Code, F00-F99 Define	F01-F99 Mental, Behavioral and Neurodevelopmental disorders	332816	Substance-induced sexual dysfunction (HCC)	ICD10CM	1	F19.981		Other psychoactive substance use, unspecified with psyc	IMO	GHS-PACDC	DX_LEGACY	ACTIVE	N	1900-01-01	9999-12-31	2015-07-10
Diagnosis: ICD10 Code, F00-F99 Define	F01-F99 Mental, Behavioral and Neurodevelopmental disorders	16948	Drug induced sleep disorder (HCC)	ICD10CM	1	F19.982		Other psychoactive substance use, unspecified with psyc	IMO	GHS-PACDC	DX_LEGACY	ACTIVE	N	1900-01-01	9999-12-31	2015-07-10
Diagnosis: ICD10 Code, F00-F99 Define	F01-F99 Mental, Behavioral and Neurodevelopmental disorders	30074	Psychoactive substance-induced organic personality disorder (HCC)	ICD10CM	1	F19.988		Other psychoactive substance use, unspecified with othe	IMO	GHS-PACDC	DX_LEGACY	ACTIVE	N	1900-01-01	9999-12-31	2015-07-10
Diagnosis: ICD10 Code, F00-F99 Define	F01-F99 Mental, Behavioral and Neurodevelopmental disorders	1181124	Other psychoactive substance use, unspecified with unspecified psychoactive substance-induce	ICD10CM	1	F19.99		Other psychoactive substance use, unspecified with unsp	IMO	GHS-PACDC	IMO_2016_R	ACTIVE	N	1900-01-01	9999-12-31	2016-06-02
Diagnosis: ICD10 Code, F00-F99 Define	F01-F99 Mental, Behavioral and Neurodevelopmental disorders	536196	Schizophrenia	ICD10CM	1	F20		Schizophrenia	IMO	GHS-PACDC	IMO_2015_R	ACTIVE	Y	1900-01-01	9999-12-31	2015-07-10
Diagnosis: ICD10 Code, F00-F99 Define	F01-F99 Mental, Behavioral and Neurodevelopmental disorders	216227	Schizophrenia, paranoid, chronic (HCC)	ICD10CM	1	F20.0		Paranoid schizophrenia	IMO	GHS-PACDC	DX_LEGACY	ACTIVE	N	1900-01-01	9999-12-31	2015-07-10
Diagnosis: ICD10 Code, F00-F99 Define	F01-F99 Mental, Behavioral and Neurodevelopmental disorders	408536	Subchronic hebephrenic schizophrenia with acute exacerbation (HCC)	ICD10CM	1	F20.1		Disorganized schizophrenia	IMO	GHS-PACDC	DX_LEGACY	ACTIVE	N	1900-01-01	9999-12-31	2015-07-10
Diagnosis: ICD10 Code, F00-F99 Define	F01-F99 Mental, Behavioral and Neurodevelopmental disorders	61282	Disorganized type schizophrenia, chronic state (HCC)	ICD10CM	1	F20.1		Disorganized schizophrenia	IMO	GHS-PACDC	DX_LEGACY	ACTIVE	N	1900-01-01	9999-12-31	2015-07-10
Diagnosis: ICD10 Code, F00-F99 Define	F01-F99 Mental, Behavioral and Neurodevelopmental disorders	144048	Schizophrenic catatonia (HCC)	ICD10CM	1	F20.2		Catatonic schizophrenia	IMO	GHS-PACDC	DX_LEGACY	ACTIVE	N	1900-01-01	9999-12-31	2015-07-10
Diagnosis: ICD10 Code, F00-F99 Define	F01-F99 Mental, Behavioral and Neurodevelopmental disorders	537122	Catatonic schizophrenia	ICD10CM	1	F20.2		Catatonic schizophrenia	IMO	GHS-PACDC	IMO_2015_R	ACTIVE	Y	1900-01-01	9999-12-31	2015-07-10
Diagnosis: ICD10 Code, F00-F99 Define	F01-F99 Mental, Behavioral and Neurodevelopmental disorders	30233	Subchronic undifferentiated schizophrenia (HCC)	ICD10CM	1	F20.3		Undifferentiated schizophrenia	IMO	GHS-PACDC	DX_LEGACY	ACTIVE	N	2016-04-10	9999-12-31	2015-07-10
Diagnosis: ICD10 Code, F00-F99 Define	F01-F99 Mental, Behavioral and Neurodevelopmental disorders	151147	Undifferentiated schizophrenia (HCC)	ICD10CM	1	F20.3		Undifferentiated schizophrenia	IMO	GHS-PACDC	DX_LEGACY	ACTIVE	N	1900-01-01	9999-12-31	2015-07-10
Diagnosis: ICD10 Code, F00-F99 Define	F01-F99 Mental, Behavioral and Neurodevelopmental disorders	2902	Residual type schizophrenic disorder, unspecified condition	ICD10CM	1	F20.5		Residual schizophrenia	IMO	GHS-PACDC	DX_LEGACY	ACTIVE	Y	1900-01-01	9999-12-31	2015-07-10
Diagnosis: ICD10 Code, F00-F99 Define	F01-F99 Mental, Behavioral and Neurodevelopmental disorders	30241	Residual schizophrenia in remission (HCC)	ICD10CM	1	F20.5		Residual schizophrenia	IMO	GHS-PACDC	DX_LEGACY	ACTIVE	N	1900-01-01	9999-12-31	2015-07-10
Diagnosis: ICD10 Code, F00-F99 Define	F01-F99 Mental, Behavioral and Neurodevelopmental disorders	140937	Chronic residual schizophrenia, with acute exacerbation (HCC)	ICD10CM	1	F20.5		Residual schizophrenia	IMO	GHS-PACDC	DX_LEGACY	ACTIVE	N	1900-01-01	9999-12-31	2015-07-10
Diagnosis: ICD10 Code, F00-F99 Define	F01-F99 Mental, Behavioral and Neurodevelopmental disorders	1100593	Schizophrenifrm dis, subchr/exac	ICD10CM	1	F20.81		Schizophreniform disorder	IMO	GHS-PACDC	IMO_2016_R	DELETED	Y	1900-01-01	9999-12-31	2016-06-02
Diagnosis: ICD10 Code, F00-F99 Define	F01-F99 Mental, Behavioral and Neurodevelopmental disorders	521201	Confusional type of schizophreniform psychosis (HCC)	ICD10CM	1	F20.81		Schizophreniform disorder	IMO	GHS-PACDC	IMO_2015_R	ACTIVE	N	1900-01-01	9999-12-31	2015-07-10
Diagnosis: ICD10 Code, F00-F99 Define	F01-F99 Mental, Behavioral and Neurodevelopmental disorders	1098106	Other specified types of schizophrenia, unspecified state	ICD10CM	1	F20.89		Other schizophrenia	IMO	GHS-PACDC	IMO_2016_R	DELETED	Y	1900-01-01	9999-12-31	2016-06-02
Diagnosis: ICD10 Code, F00-F99 Define	F01-F99 Mental, Behavioral and Neurodevelopmental disorders	1134752	Schizophrenia spectrum disorder with psychotic disorder type not yet determined	ICD10CM	1	F20.89	F20.89,F29	Other schizophrenia	IMO	GHS-PACDC	IMO_2016_R	ACTIVE	N	1900-01-01	2017-04-27	2016-06-02
Diagnosis: ICD10 Code, F00-F99 Define	F01-F99 Mental, Behavioral and Neurodevelopmental disorders	608875	Other schizophrenia	ICD10CM	1	F20.89		Other schizophrenia	IMO	GHS-PACDC	IMO_2015_R	ACTIVE	Y	1900-01-01	9999-12-31	2015-07-10
Diagnosis: ICD10 Code, F00-F99 Define	F01-F99 Mental, Behavioral and Neurodevelopmental disorders	2921	Other specified types of schizophrenia, in remission	ICD10CM	1	F20.89		Other schizophrenia	IMO	GHS-PACDC	DX_LEGACY	ACTIVE	Y	1900-01-01	9999-12-31	2015-07-10
Diagnosis: ICD10 Code, F00-F99 Define	F01-F99 Mental, Behavioral and Neurodevelopmental disorders	2917	Other specified types of schizophrenia, subchronic condition	ICD10CM	1	F20.89		Other schizophrenia	IMO	GHS-PACDC	DX_LEGACY	ACTIVE	Y	1900-01-01	9999-12-31	2015-07-10
Diagnosis: ICD10 Code, F00-F99 Define	F01-F99 Mental, Behavioral and Neurodevelopmental disorders	641285	Other schizophrenia (HCC)	ICD10CM	1	F20.89		Other schizophrenia	IMO	GHS-PACDC	IMO_2015_R	ACTIVE	N	1900-01-01	9999-12-31	2015-07-10
Diagnosis: ICD10 Code, F00-F99 Define	F01-F99 Mental, Behavioral and Neurodevelopmental disorders	147461	Cenesthopathic schizophrenia, subchronic with acute exacerbation (HCC)	ICD10CM	1	F20.89		Other schizophrenia	IMO	GHS-PACDC	DX_LEGACY	ACTIVE	N	1900-01-01	9999-12-31	2015-07-10
Diagnosis: ICD10 Code, F00-F99 Define	F01-F99 Mental, Behavioral and Neurodevelopmental disorders	2864	Simple schizophrenia, chronic condition with acute exacerbation (HCC)	ICD10CM	1	F20.89		Other schizophrenia	IMO	GHS-PACDC	DX_LEGACY	ACTIVE	N	1900-01-01	9999-12-31	2015-07-10
Diagnosis: ICD10 Code, F00-F99 Define	F01-F99 Mental, Behavioral and Neurodevelopmental disorders	30216	Chronic schizophrenia (HCC)	ICD10CM	1	F20.9		Schizophrenia, unspecified	IMO	GHS-PACDC	DX_LEGACY	ACTIVE	N	1900-01-01	9999-12-31	2015-07-10
Diagnosis: ICD10 Code, F00-F99 Define	F01-F99 Mental, Behavioral and Neurodevelopmental disorders	140950	Subchronic schizophrenia with acute exacerbation (HCC)	ICD10CM	1	F20.9		Schizophrenia, unspecified	IMO	GHS-PACDC	DX_LEGACY	ACTIVE	N	1900-01-01	9999-12-31	2015-07-10
Diagnosis: ICD10 Code, F00-F99 Define	F01-F99 Mental, Behavioral and Neurodevelopmental disorders	487919	Delusional disorder, persecutory type (HCC)	ICD10CM	1	F22		Delusional disorders	IMO	GHS-PACDC	IMO_2015_R	ACTIVE	N	1900-01-01	9999-12-31	2015-07-10
Diagnosis: ICD10 Code, F00-F99 Define	F01-F99 Mental, Behavioral and Neurodevelopmental disorders	366778	Capgras' syndrome (HCC)	ICD10CM	1	F22		Delusional disorders	IMO	GHS-PACDC	DX_LEGACY	ACTIVE	N	1900-01-01	9999-12-31	2015-07-10
Diagnosis: ICD10 Code, F00-F99 Define	F01-F99 Mental, Behavioral and Neurodevelopmental disorders	408563	Pinocchio syndrome (HCC)	ICD10CM	1	F22		Delusional disorders	IMO	GHS-PACDC	DX_LEGACY	ACTIVE	N	1900-01-01	9999-12-31	2015-07-10
Diagnosis: ICD10 Code, F00-F99 Define	F01-F99 Mental, Behavioral and Neurodevelopmental disorders	488366	Delusional disorder, somatic type, continuous (HCC)	ICD10CM	1	F22		Delusional disorders	IMO	GHS-PACDC	IMO_2015_R	ACTIVE	N	1900-01-01	9999-12-31	2015-07-10
Diagnosis: ICD10 Code, F00-F99 Define	F01-F99 Mental, Behavioral and Neurodevelopmental disorders	408570	Delusion of hyperhidrosis (HCC)	ICD10CM	1	F22		Delusional disorders	IMO	GHS-PACDC	DX_LEGACY	ACTIVE	N	1900-01-01	9999-12-31	2015-07-10
Diagnosis: ICD10 Code, F00-F99 Define	F01-F99 Mental, Behavioral and Neurodevelopmental disorders	3001	Paranoid state (HCC)	ICD10CM	1	F22		Delusional disorders	IMO	GHS-PACDC	DX_LEGACY	ACTIVE	N	1900-01-01	9999-12-31	2015-07-10
Diagnosis: ICD10 Code, F00-F99 Define	F01-F99 Mental, Behavioral and Neurodevelopmental disorders	376971	Paranoid behavior (HCC)	ICD10CM	1	F22		Delusional disorders	IMO	GHS-PACDC	DX_LEGACY	ACTIVE	N	1900-01-01	9999-12-31	2015-07-10
Diagnosis: ICD10 Code, F00-F99 Define	F01-F99 Mental, Behavioral and Neurodevelopmental disorders	488397	Delusional disorder, jealous type, continuous (HCC)	ICD10CM	1	F22		Delusional disorders	IMO	GHS-PACDC	IMO_2015_R	ACTIVE	N	1900-01-01	9999-12-31	2015-07-10
Diagnosis: ICD10 Code, F00-F99 Define	F01-F99 Mental, Behavioral and Neurodevelopmental disorders	53967	Paranoid psychosis (HCC)	ICD10CM	1	F22		Delusional disorders	IMO	GHS-PACDC	DX_LEGACY	ACTIVE	N	1900-01-01	9999-12-31	2015-07-10
Diagnosis: ICD10 Code, F00-F99 Define	F01-F99 Mental, Behavioral and Neurodevelopmental disorders	488145	Delusional disorder, somatic type, multiple episodes, currently in partial remission (HCC)	ICD10CM	1	F22		Delusional disorders	IMO	GHS-PACDC	IMO_2015_R	ACTIVE	N	1900-01-01	9999-12-31	2015-07-10
Diagnosis: ICD10 Code, F00-F99 Define	F01-F99 Mental, Behavioral and Neurodevelopmental disorders	488435	Delusional disorder, persecutory type, with bizarre content, continuous (HCC)	ICD10CM	1	F22		Delusional disorders	IMO	GHS-PACDC	IMO_2015_R	ACTIVE	N	1900-01-01	9999-12-31	2015-07-10
Diagnosis: ICD10 Code, F00-F99 Define	F01-F99 Mental, Behavioral and Neurodevelopmental disorders	448700	Senile dementia with delusional features with behavioral disturbance (HCC)	ICD10CM	2	F22	F03.91,F22	Delusional disorders	IMO	GHS-PACDC	IMO_2015_R	ACTIVE	N	1900-01-01	9999-12-31	2015-07-10
Diagnosis: ICD10 Code, F00-F99 Define	F01-F99 Mental, Behavioral and Neurodevelopmental disorders	485266	Mixed type delusional disorder, with bizarre content, first episode, currently in partial remissior	ICD10CM	1	F22		Delusional disorders	IMO	GHS-PACDC	IMO_2015_R	ACTIVE	N	1900-01-01	9999-12-31	2015-07-10
Diagnosis: ICD10 Code, F00-F99 Define	F01-F99 Mental, Behavioral and Neurodevelopmental disorders	50337	Involution, paraphrenia (HCC)	ICD10CM	1	F22		Delusional disorders	IMO	GHS-PACDC	DX_LEGACY	ACTIVE	N	1900-01-01	9999-12-31	2015-07-10
Diagnosis: ICD10 Code, F00-F99 Define	F01-F99 Mental, Behavioral and Neurodevelopmental disorders	488367	Delusional disorder, persecutory type, continuous (HCC)	ICD10CM	1	F22		Delusional disorders	IMO	GHS-PACDC	IMO_2015_R	ACTIVE	N	1900-01-01	9999-12-31	2015-07-10
Diagnosis: ICD10 Code, F00-F99 Define	F01-F99 Mental, Behavioral and Neurodevelopmental disorders	488432	Continuous grandiose type delusional disorder, with bizarre content (HCC)	ICD10CM	1	F22		Delusional disorders	IMO	GHS-PACDC	IMO_2015_R	ACTIVE	N	1900-01-01	9999-12-31	2015-07-10
Diagnosis: ICD10 Code, F00-F99 Define	F01-F99 Mental, Behavioral and Neurodevelopmental disorders	366777	Capgras syndrome (HCC)	ICD10CM	1	F22		Delusional disorders	IMO	GHS-PACDC	DX_LEGACY	ACTIVE	N	1900-01-01	9999-12-31	2015-07-10
Diagnosis: ICD10 Code, F00-F99 Define	F01-F99 Mental, Behavioral and Neurodevelopmental disorders	45764	Paraphrenia, involutional (HCC)	ICD10CM	1	F22		Delusional disorders	IMO	GHS-PACDC	DX_LEGACY	ACTIVE	N	1900-01-01	9999-12-31	2015-07-10
Diagnosis: ICD10 Code, F00-F99 Define	F01-F99 Mental, Behavioral and Neurodevelopmental disorders	486515	Jealous type delusional disorder, with bizarre content, multiple episodes currently in full remiss	ICD10CM	1	F22		Delusional disorders	IMO	GHS-PACDC	IMO_2015_R	ACTIVE	N	1900-01-01	9999-12-31	2015-07-10
Diagnosis: ICD10 Code, F00-F99 Define	F01-F99 Mental, Behavioral and Neurodevelopmental disorders	30364	Brief psychotic disorder	ICD10CM	1	F23		Brief psychotic disorder	IMO	GHS-PACDC	DX_LEGACY	ACTIVE	N	1900-01-01	9999-12-31	2015-07-10
Diagnosis: ICD10 Code, F00-F99 Define	F01-F99 Mental, Behavioral and Neurodevelopmental disorders	1111003	Schizophrenia, first episode, currently acute	ICD10CM	1	F23		Brief psychotic disorder	IMO	GHS-PACDC	IMO_2016_R	ACTIVE	N	1900-01-01	9999-12-31	2016-06-02
Diagnosis: ICD10 Code, F00-F99 Define	F01-F99 Mental, Behavioral and Neurodevelopmental disorders	317209	Acute and transient psychotic disorder	ICD10CM	1	F23		Brief psychotic disorder	IMO	GHS-PACDC	DX_LEGACY	ACTIVE	N	1900-01-01	9999-12-31	2015-07-10
Diagnosis: ICD10 Code, F00-F99 Define	F01-F99 Mental, Behavioral and Neurodevelopmental disorders	486804	Brief psychotic disorder with catatonia	ICD10CM	1	F23	F23,F06.1	Brief psychotic disorder	IMO	GHS-PACDC	IMO_2015_R	ACTIVE	N	2016-09-17	9999-12-31	2016-09-30
Diagnosis: ICD10 Code, F00-F99 Define	F01-F99 Mental, Behavioral and Neurodevelopmental disorders	30365	Bouffee delirante	ICD10CM	1	F23		Brief psychotic disorder	IMO	GHS-PACDC	DX_LEGACY	ACTIVE	N	2016-09-17	9999-12-31	2016-09-30
Diagnosis: ICD10 Code, F00-F99 Define	F01-F99 Mental, Behavioral and Neurodevelopmental disorders	2999	Shared paranoid disorder (HCC)	ICD10CM	1	F24		Shared psychotic disorder	IMO	GHS-PACDC	DX_LEGACY	ACTIVE	N	1900-01-01	9999-12-31	2015-07-10
Diagnosis: ICD10 Code, F00-F99 Define	F01-F99 Mental, Behavioral and Neurodevelopmental disorders	114229	Schizoaffective-type schizophrenia (HCC)	ICD10CM	1	F25.0		Schizoaffective disorder, bipolar type	IMO	GHS-PACDC	DX_LEGACY	ACTIVE	N	1900-01-01	9999-12-31	2015-07-10
Diagnosis: ICD10 Code, F00-F99 Define	F01-F99 Mental, Behavioral and Neurodevelopmental disorders	486540	Schizoaffective disorder, depressive type without good prognostic features (HCC)	ICD10CM	1	F25.1		Schizoaffective disorder, depressive type	IMO	GHS-PACDC	IMO_2015_R	ACTIVE	N	1900-01-01	9999-12-31	2015-07-10
Diagnosis: ICD10 Code, F00-F99 Define	F01-F99 Mental, Behavioral and Neurodevelopmental disorders	219750	Schizophrenia, schizoaffective, subchronic with acute exacerbation (HCC)	ICD10CM	1	F25.8		Other schizoaffective disorders	IMO	GHS-PACDC	DX_LEGACY	ACTIVE	N	1900-01-01	9999-12-31	2015-07-10
Diagnosis: ICD10 Code, F00-F99 Define	F01-F99 Mental, Behavioral and Neurodevelopmental disorders	2910	Schizoaffective disorder, subchronic condition (HCC)	ICD10CM	1	F25.8		Other schizoaffective disorders	IMO	GHS-PACDC	DX_LEGACY	ACTIVE	N	1900-01-01	9999-12-31	2015-07-10
Diagnosis: ICD10 Code, F00-F99 Define	F01-F99 Mental, Behavioral and Neurodevelopmental disorders	2933	Bipolar I disorder, single manic episode, moderate (HCC)	ICD10CM	1	F30.12		Manic episode without psychotic symptoms, moderate	IMO	GHS-PACDC	DX_LEGACY	ACTIVE	N	1900-01-01	9999-12-31	2015-07-10

Diagnosis: ICD10 Code, F00-F99 Define	F01-F99 Mental, Behavioral and Neurodevelopmental disorders	335055	Manic disorder, single episode, severe degree, without mention of psychotic behavior	ICD10CM	1	F30.13		Manic episode, severe, without psychotic symptoms	IMO	GHS-PACDC	DX_LEGACY	DELETED	Y	1900-01-01	9999-12-31	2015-07-10
Diagnosis: ICD10 Code, F00-F99 Define	F01-F99 Mental, Behavioral and Neurodevelopmental disorders	260781	Bipolar I disorder, single manic episode, severe, specified as with psychotic behavior	ICD10CM	1	F30.2		Manic episode, severe with psychotic symptoms	IMO	GHS-PACDC	DX_LEGACY	ACTIVE	Y	1900-01-01	9999-12-31	2015-07-10
Diagnosis: ICD10 Code, F00-F99 Define	F01-F99 Mental, Behavioral and Neurodevelopmental disorders	333374	Severe bipolar I disorder, single manic episode with psychotic features, mood-congruent (HCC)	ICD10CM	1	F30.2		Manic episode, severe with psychotic symptoms	IMO	GHS-PACDC	DX_LEGACY	ACTIVE	N	1900-01-01	9999-12-31	2015-07-10
Diagnosis: ICD10 Code, F00-F99 Define	F01-F99 Mental, Behavioral and Neurodevelopmental disorders	268865	Manic episode in partial remission (HCC)	ICD10CM	1	F30.3		Manic episode in partial remission	IMO	GHS-PACDC	DX_LEGACY	ACTIVE	N	1900-01-01	9999-12-31	2015-07-10
Diagnosis: ICD10 Code, F00-F99 Define	F01-F99 Mental, Behavioral and Neurodevelopmental disorders	304610	Bipolar I disorder, single manic episode, in partial or unspecified remission	ICD10CM	1	F30.3		Manic episode in partial remission	IMO	GHS-PACDC	DX_LEGACY	ACTIVE	Y	2016-04-10	9999-12-31	2015-07-10
Diagnosis: ICD10 Code, F00-F99 Define	F01-F99 Mental, Behavioral and Neurodevelopmental disorders	39212	Manic affective disorder, recurrent episode, moderate degree (HCC)	ICD10CM	1	F30.8		Other manic episodes	IMO	GHS-PACDC	DX_LEGACY	ACTIVE	N	1900-01-01	2016-05-07	2015-07-10
Diagnosis: ICD10 Code, F00-F99 Define	F01-F99 Mental, Behavioral and Neurodevelopmental disorders	487283	Severe manic disorder, recurrent episode (HCC)	ICD10CM	1	F30.8		Other manic episodes	IMO	GHS-PACDC	IMO_2015_R	ACTIVE	N	1900-01-01	9999-12-31	2015-07-10
Diagnosis: ICD10 Code, F00-F99 Define	F01-F99 Mental, Behavioral and Neurodevelopmental disorders	30282	Bipolar I disorder, single manic episode, in remission (HCC)	ICD10CM	1	F30.8		Other manic episodes	IMO	GHS-PACDC	DX_LEGACY	ACTIVE	N	1900-01-01	2016-05-07	2015-07-10
Diagnosis: ICD10 Code, F00-F99 Define	F01-F99 Mental, Behavioral and Neurodevelopmental disorders	83617	Manic disorder, recurrent episode, severe, specified as with psychotic behavior	ICD10CM	1	F30.8		Other manic episodes	IMO	GHS-PACDC	DX_LEGACY	ACTIVE	Y	1900-01-01	2016-05-07	2015-07-10
Diagnosis: ICD10 Code, F00-F99 Define	F01-F99 Mental, Behavioral and Neurodevelopmental disorders	61311	Manic disorder, recurrent episode, in partial or unspecified remission	ICD10CM	1	F30.8		Other manic episodes	IMO	GHS-PACDC	DX_LEGACY	ACTIVE	Y	1900-01-01	9999-12-31	2015-07-10
Diagnosis: ICD10 Code, F00-F99 Define	F01-F99 Mental, Behavioral and Neurodevelopmental disorders	1138002	Manic episode, unspecified (HCC)	ICD10CM	1	F30.9		Manic episode, unspecified	IMO	GHS-PACDC	IMO_2016_R	ACTIVE	N	1900-01-01	9999-12-31	2016-06-02
Diagnosis: ICD10 Code, F00-F99 Define	F01-F99 Mental, Behavioral and Neurodevelopmental disorders	428980	Bipolar I disorder, most recent episode hypomanic with seasonal pattern (HCC)	ICD10CM	1	F31.0		Bipolar disorder, current episode hypomanic	IMO	GHS-PACDC	IMO_2015_R	ACTIVE	N	1900-01-01	9999-12-31	2015-07-10
Diagnosis: ICD10 Code, F00-F99 Define	F01-F99 Mental, Behavioral and Neurodevelopmental disorders	307995	Bipolar disorder, current episode hypomanic (HCC)	ICD10CM	1	F31.0		Bipolar disorder, current episode hypomanic	IMO	GHS-PACDC	DX_LEGACY	ACTIVE	N	1900-01-01	9999-12-31	2015-07-10
Diagnosis: ICD10 Code, F00-F99 Define	F01-F99 Mental, Behavioral and Neurodevelopmental disorders	307994	Bipolar affective disorder, current episode hypomanic (HCC)	ICD10CM	1	F31.0		Bipolar disorder, current episode hypomanic	IMO	GHS-PACDC	DX_LEGACY	ACTIVE	N	1900-01-01	9999-12-31	2015-07-10
Diagnosis: ICD10 Code, F00-F99 Define	F01-F99 Mental, Behavioral and Neurodevelopmental disorders	331831	Bipolar I disorder with mania (HCC)	ICD10CM	1	F31.10		Bipolar disorder, current episode manic without psychot	IMO	GHS-PACDC	DX_LEGACY	ACTIVE	N	1900-01-01	9999-12-31	2015-07-10
Diagnosis: ICD10 Code, F00-F99 Define	F01-F99 Mental, Behavioral and Neurodevelopmental disorders	971809	Bipolar affective disorder, current episode manic without psychotic symptoms, unspecified seve	ICD10CM	1	F31.10		Bipolar disorder, current episode manic without psychot	IMO	GHS-PACDC	IMO_2015_R	DELETED	Y	1900-01-01	9999-12-31	2015-07-10
Diagnosis: ICD10 Code, F00-F99 Define	F01-F99 Mental, Behavioral and Neurodevelopmental disorders	485415	Bipolar I disorder, most recent episode (or current) manic with peripartum onset (HCC)	ICD10CM	1	F31.10		Bipolar disorder, current episode manic without psychot	IMO	GHS-PACDC	IMO_2015_R	ACTIVE	N	1900-01-01	9999-12-31	2015-07-10
Diagnosis: ICD10 Code, F00-F99 Define	F01-F99 Mental, Behavioral and Neurodevelopmental disorders	486878	Bipolar I disorder, most recent episode (or current) manic, moderate with rapid cycling (HCC)	ICD10CM	1	F31.12		Bipolar disorder, current episode manic without psychot	IMO	GHS-PACDC	IMO_2015_R	ACTIVE	N	1900-01-01	9999-12-31	2015-07-10
Diagnosis: ICD10 Code, F00-F99 Define	F01-F99 Mental, Behavioral and Neurodevelopmental disorders	1102456	Moderate bipolar I disorder with mania as current episode (HCC)	ICD10CM	1	F31.12		Bipolar disorder, current episode manic without psychot	IMO	GHS-PACDC	IMO_2016_R	ACTIVE	N	1900-01-01	9999-12-31	2016-06-02
Diagnosis: ICD10 Code, F00-F99 Define	F01-F99 Mental, Behavioral and Neurodevelopmental disorders	140963	Bipolar I disorder, most recent episode (or current) manic, moderate (HCC)	ICD10CM	1	F31.12		Bipolar disorder, current episode manic without psychot	IMO	GHS-PACDC	DX_LEGACY	ACTIVE	N	1900-01-01	9999-12-31	2015-07-10
Diagnosis: ICD10 Code, F00-F99 Define	F01-F99 Mental, Behavioral and Neurodevelopmental disorders	489440	Severe bipolar I disorder, current or most recent episode manic, with rapid cycling (HCC)	ICD10CM	1	F31.13		Bipolar disorder, current episode manic without psychot	IMO	GHS-PACDC	IMO_2015_R	ACTIVE	N	1900-01-01	9999-12-31	2015-07-10
Diagnosis: ICD10 Code, F00-F99 Define	F01-F99 Mental, Behavioral and Neurodevelopmental disorders	488963	Severe bipolar I disorder, current or most recent episode manic, with melancholic features (HC	ICD10CM	1	F31.13		Bipolar disorder, current episode manic without psychot	IMO	GHS-PACDC	IMO_2015_R	ACTIVE	N	1900-01-01	9999-12-31	2015-07-10
Diagnosis: ICD10 Code, F00-F99 Define	F01-F99 Mental, Behavioral and Neurodevelopmental disorders	489183	Moderate bipolar I disorder, current or most recent episode manic, with psychotic features, wit	ICD10CM	1	F31.2		Bipolar disorder, current episode manic severe with psy	IMO	GHS-PACDC	IMO_2015_R	ACTIVE	N	1900-01-01	9999-12-31	2015-07-10
Diagnosis: ICD10 Code, F00-F99 Define	F01-F99 Mental, Behavioral and Neurodevelopmental disorders	489443	Bipolar I disorder, moderate, current or most recent episode manic, with psychotic features (HC	ICD10CM	1	F31.2		Bipolar disorder, current episode manic severe with psy	IMO	GHS-PACDC	IMO_2015_R	ACTIVE	N	1900-01-01	9999-12-31	2015-07-10
Diagnosis: ICD10 Code, F00-F99 Define	F01-F99 Mental, Behavioral and Neurodevelopmental disorders	489447	Bipolar I disorder, moderate, current or most recent episode manic, with psychotic features, wi	ICD10CM	1	F31.2		Bipolar disorder, current episode manic severe with psy	IMO	GHS-PACDC	IMO_2015_R	ACTIVE	N	1900-01-01	9999-12-31	2015-07-10
Diagnosis: ICD10 Code, F00-F99 Define	F01-F99 Mental, Behavioral and Neurodevelopmental disorders	2967	Bipolar I, most recent episode manic, severe with psychotic behavior (HCC)	ICD10CM	1	F31.2		Bipolar disorder, current episode manic severe with psy	IMO	GHS-PACDC	DX_LEGACY	ACTIVE	N	1900-01-01	9999-12-31	2015-07-10
Diagnosis: ICD10 Code, F00-F99 Define	F01-F99 Mental, Behavioral and Neurodevelopmental disorders	487100	Bipolar I disorder, current or most recent episode manic, with psychotic features with mixed fe	ICD10CM	1	F31.2		Bipolar disorder, current episode manic severe with psy	IMO	GHS-PACDC	IMO_2015_R	ACTIVE	N	1900-01-01	9999-12-31	2015-07-10
Diagnosis: ICD10 Code, F00-F99 Define	F01-F99 Mental, Behavioral and Neurodevelopmental disorders	488971	Mild bipolar I disorder, current or most recent episode manic, with psychotic features, with per	ICD10CM	1	F31.2		Bipolar disorder, current episode manic severe with psy	IMO	GHS-PACDC	IMO_2015_R	ACTIVE	N	1900-01-01	9999-12-31	2015-07-10
Diagnosis: ICD10 Code, F00-F99 Define	F01-F99 Mental, Behavioral and Neurodevelopmental disorders	83638	Bipolar I disorder, most recent episode (or current) manic, severe, specified as with psychotic b	ICD10CM	1	F31.2		Bipolar disorder, current episode manic severe with psy	IMO	GHS-PACDC	DX_LEGACY	ACTIVE	Y	1900-01-01	9999-12-31	2015-07-10
Diagnosis: ICD10 Code, F00-F99 Define	F01-F99 Mental, Behavioral and Neurodevelopmental disorders	333383	Bipolar I disorder, most recent episode depressed with melancholic features (HCC)	ICD10CM	1	F31.30		Bipolar disorder, current episode depressed, mild or mo	IMO	GHS-PACDC	DX_LEGACY	ACTIVE	N	1900-01-01	9999-12-31	2015-07-10
Diagnosis: ICD10 Code, F00-F99 Define	F01-F99 Mental, Behavioral and Neurodevelopmental disorders	315713	Mild depressed bipolar 1 disorder (HCC)	ICD10CM	1	F31.31		Bipolar disorder, current episode depressed, mild	IMO	GHS-PACDC	DX_LEGACY	ACTIVE	N	1900-01-01	9999-12-31	2015-07-10
Diagnosis: ICD10 Code, F00-F99 Define	F01-F99 Mental, Behavioral and Neurodevelopmental disorders	30286	Mild bipolar I disorder, most recent episode depressed (HCC)	ICD10CM	1	F31.31		Bipolar disorder, current episode depressed, mild	IMO	GHS-PACDC	DX_LEGACY	ACTIVE	N	1900-01-01	9999-12-31	2015-07-10
Diagnosis: ICD10 Code, F00-F99 Define	F01-F99 Mental, Behavioral and Neurodevelopmental disorders	485327	Mild depressed bipolar I disorder with catatonia (HCC)	ICD10CM	1	F31.31		Bipolar disorder, current episode depressed, mild	IMO	GHS-PACDC	IMO_2015_R	ACTIVE	N	1900-01-01	2016-10-14	2015-07-10
Diagnosis: ICD10 Code, F00-F99 Define	F01-F99 Mental, Behavioral and Neurodevelopmental disorders	488914	Bipolar I disorder, mild, current or most recent episode depressed, with psychotic features, with	ICD10CM	1	F31.31		Bipolar disorder, current episode depressed, mild	IMO	GHS-PACDC	IMO_2015_R	ACTIVE	N	1900-01-01	9999-12-31	2015-07-10
Diagnosis: ICD10 Code, F00-F99 Define	F01-F99 Mental, Behavioral and Neurodevelopmental disorders	485335	Moderate depressed bipolar I disorder with peripartum onset (HCC)	ICD10CM	1	F31.32		Bipolar disorder, current episode depressed, moderate	IMO	GHS-PACDC	IMO_2015_R	ACTIVE	N	1900-01-01	9999-12-31	2015-07-10
Diagnosis: ICD10 Code, F00-F99 Define	F01-F99 Mental, Behavioral and Neurodevelopmental disorders	163458	Bipolar affective disorder, currently depressed, moderate (HCC)	ICD10CM	1	F31.32		Bipolar disorder, current episode depressed, moderate	IMO	GHS-PACDC	DX_LEGACY	ACTIVE	N	1900-01-01	9999-12-31	2015-07-10
Diagnosis: ICD10 Code, F00-F99 Define	F01-F99 Mental, Behavioral and Neurodevelopmental disorders	486054	Moderate depressed bipolar I disorder with mood-incongruent psychotic features (HCC)	ICD10CM	1	F31.32		Bipolar disorder, current episode depressed, moderate	IMO	GHS-PACDC	IMO_2015_R	ACTIVE	N	1900-01-01	9999-12-31	2015-07-10
Diagnosis: ICD10 Code, F00-F99 Define	F01-F99 Mental, Behavioral and Neurodevelopmental disorders	75930	Bipolar affective disorder, depressed, moderate (HCC)	ICD10CM	1	F31.32		Bipolar disorder, current episode depressed, moderate	IMO	GHS-PACDC	DX_LEGACY	ACTIVE	N	1900-01-01	9999-12-31	2015-07-10
Diagnosis: ICD10 Code, F00-F99 Define	F01-F99 Mental, Behavioral and Neurodevelopmental disorders	488901	Moderate bipolar I disorder, current or most recent episode depressed, with anxious distress (H	ICD10CM	1	F31.32		Bipolar disorder, current episode depressed, moderate	IMO	GHS-PACDC	IMO_2015_R	ACTIVE	N	1900-01-01	9999-12-31	2015-07-10
Diagnosis: ICD10 Code, F00-F99 Define	F01-F99 Mental, Behavioral and Neurodevelopmental disorders	2974	Bipolar I, most recent episode depressed, severe (HCC)	ICD10CM	1	F31.4		Bipolar disorder, current episode depressed, severe, wit	IMO	GHS-PACDC	DX_LEGACY	ACTIVE	N	1900-01-01	9999-12-31	2015-07-10
Diagnosis: ICD10 Code, F00-F99 Define	F01-F99 Mental, Behavioral and Neurodevelopmental disorders	488636	Bipolar I disorder, severe, current or most recent episode depressed, with melancholic features	ICD10CM	1	F31.4		Bipolar disorder, current episode depressed, severe, wit	IMO	GHS-PACDC	IMO_2015_R	ACTIVE	N	1900-01-01	9999-12-31	2015-07-10
Diagnosis: ICD10 Code, F00-F99 Define	F01-F99 Mental, Behavioral and Neurodevelopmental disorders	488635	Severe bipolar I disorder, current or most recent episode depressed, with melancholic features	ICD10CM	1	F31.4		Bipolar disorder, current episode depressed, severe, wit	IMO	GHS-PACDC	IMO_2015_R	ACTIVE	N	1900-01-01	9999-12-31	2015-07-10
Diagnosis: ICD10 Code, F00-F99 Define	F01-F99 Mental, Behavioral and Neurodevelopmental disorders	487221	Bipolar I disorder, current or most recent episode depressed, with psychotic features with anxic	ICD10CM	1	F31.5		Bipolar disorder, current episode depressed, severe, wit	IMO	GHS-PACDC	IMO_2015_R	ACTIVE	N	1900-01-01	9999-12-31	2015-07-10
Diagnosis: ICD10 Code, F00-F99 Define	F01-F99 Mental, Behavioral and Neurodevelopmental disorders	489146	Severe bipolar I disorder, current or most recent episode depressed, with psychotic features, wi	ICD10CM	1	F31.5		Bipolar disorder, current episode depressed, severe, wit	IMO	GHS-PACDC	IMO_2015_R	ACTIVE	N	1900-01-01	9999-12-31	2015-07-10
Diagnosis: ICD10 Code, F00-F99 Define	F01-F99 Mental, Behavioral and Neurodevelopmental disorders	485505	Bipolar I disorder, most recent episode depressed with mood-congruent psychotic features (HC	ICD10CM	1	F31.5		Bipolar disorder, current episode depressed, severe, wit	IMO	GHS-PACDC	IMO_2015_R	ACTIVE	N	1900-01-01	9999-12-31	2015-07-10
Diagnosis: ICD10 Code, F00-F99 Define	F01-F99 Mental, Behavioral and Neurodevelopmental disorders	30259	Bipolar I disorder, most recent episode mixed with catatonic features (HCC)	ICD10CM	1	F31.60	F31.60,F06.1	Bipolar disorder, current episode mixed, unspecified	IMO	GHS-PACDC	DX_LEGACY	ACTIVE	N	2016-09-17	9999-12-31	2016-09-30
Diagnosis: ICD10 Code, F00-F99 Define	F01-F99 Mental, Behavioral and Neurodevelopmental disorders	147240	Bipolar affective, mixed (HCC)	ICD10CM	1	F31.60		Bipolar disorder, current episode mixed, unspecified	IMO	GHS-PACDC	DX_LEGACY	ACTIVE	N	1900-01-01	9999-12-31	2015-07-10
Diagnosis: ICD10 Code, F00-F99 Define	F01-F99 Mental, Behavioral and Neurodevelopmental disorders	30256	Moderate bipolar I disorder, most recent episode mixed (HCC)	ICD10CM	1	F31.62		Bipolar disorder, current episode mixed, moderate	IMO	GHS-PACDC	DX_LEGACY	ACTIVE	N	1900-01-01	9999-12-31	2015-07-10
Diagnosis: ICD10 Code, F00-F99 Define	F01-F99 Mental, Behavioral and Neurodevelopmental disorders	335071	Bipolar affective disorder, mixed, severe degree, without mention of psychotic behavior	ICD10CM	1	F31.63		Bipolar disorder, current episode mixed, severe, without	IMO	GHS-PACDC	DX_LEGACY	DELETED	Y	1900-01-01	9999-12-31	2015-07-10
Diagnosis: ICD10 Code, F00-F99 Define	F01-F99 Mental, Behavioral and Neurodevelopmental disorders	483719	Severe bipolar I disorder, current or most recent episode mixed (HCC)	ICD10CM	1	F31.63		Bipolar disorder, current episode mixed, severe, without	IMO	GHS-PACDC	IMO_2015_R	ACTIVE	N	1900-01-01	9999-12-31	2015-07-10
Diagnosis: ICD10 Code, F00-F99 Define	F01-F99 Mental, Behavioral and Neurodevelopmental disorders	329250	History of depressed bipolar disorder (HCC)	ICD10CM	1	F31.70		Bipolar disorder, currently in remission, most recent epi	IMO	GHS-PACDC	DX_LEGACY	ACTIVE	N	2016-04-10	9999-12-31	2015-07-10
Diagnosis: ICD10 Code, F00-F99 Define	F01-F99 Mental, Behavioral and Neurodevelopmental disorders	30262	Bipolar I disorder, most recent episode mixed, in remission (HCC)	ICD10CM	1	F31.70		Bipolar disorder, currently in remission, most recent epi	IMO	GHS-PACDC	DX_LEGACY	ACTIVE	N	2016-04-10	9999-12-31	2015-07-10
Diagnosis: ICD10 Code, F00-F99 Define	F01-F99 Mental, Behavioral and Neurodevelopmental disorders	268015	Bipolar disorder, in full remission, most recent episode hypomanic (HCC)	ICD10CM	1	F31.72		Bipolar disorder, in full remission, most recent episode h	IMO	GHS-PACDC	DX_LEGACY	ACTIVE	N	1900-01-01	9999-12-31	2015-07-10
Diagnosis: ICD10 Code, F00-F99 Define	F01-F99 Mental, Behavioral and Neurodevelopmental disorders	485766	Bipolar I disorder, current or most recent episode hypomanic, in partial remission with seasona	ICD10CM	1	F31.73		Bipolar disorder, in partial remission, most recent episo	IMO	GHS-PACDC	IMO_2015_R	ACTIVE	N	1900-01-01	9999-12-31	2015-07-10
Diagnosis: ICD10 Code, F00-F99 Define	F01-F99 Mental, Behavioral and Neurodevelopmental disorders	485765	Bipolar I disorder, current or most recent episode hypomanic, in partial remission with peripart	ICD10CM	1	F31.73		Bipolar disorder, in partial remission, most recent episo	IMO	GHS-PACDC	IMO_2015_R	ACTIVE	N	1900-01-01	9999-12-31	2015-07-10
Diagnosis: ICD10 Code, F00-F99 Define	F01-F99 Mental, Behavioral and Neurodevelopmental disorders	489521	Bipolar I disorder, moderate, current or most recent episode manic, in partial remission, with m	ICD10CM	1	F31.73		Bipolar disorder, in partial remission, most recent episo	IMO	GHS-PACDC	IMO_2015_R	ACTIVE	N	1900-01-01	9999-12-31	2015-07-10
Diagnosis: ICD10 Code, F00-F99 Define	F01-F99 Mental, Behavioral and Neurodevelopmental disorders	489328	Mild bipolar I disorder, current or most recent episode manic, in partial remission, with peripart	ICD10CM	1	F31.73		Bipolar disorder, in partial remission, most recent episo	IMO	GHS-PACDC	IMO_2015_R	ACTIVE	N	1900-01-01	9999-12-31	2015-07-10
Diagnosis: ICD10 Code, F00-F99 Define	F01-F99 Mental, Behavioral and Neurodevelopmental disorders	489523	Bipolar I disorder, severe, current or most recent episode manic, in partial remission, with anxic	ICD10CM	1	F31.73		Bipolar disorder, in partial remission, most recent episo	IMO	GHS-PACDC	IMO_2015_R	ACTIVE	N	1900-01-01	9999-12-31	2015-07-10
Diagnosis: ICD10 Code, F00-F99 Define	F01-F99 Mental, Behavioral and Neurodevelopmental disorders	488677	Bipolar I disorder, mild, current or most recent episode manic, in partial remission, with catator	ICD10CM	1	F31.73		Bipolar disorder, in partial remission, most recent episo	IMO	GHS-PACDC	IMO_2015_R	ACTIVE	N	1900-01-01	2016-10-14	2015-07-10
Diagnosis: ICD10 Code, F00-F99 Define	F01-F99 Mental, Behavioral and Neurodevelopmental disorders	487135	Bipolar I disorder, current or most recent episode hypomanic, in partial remission with mood-in	ICD10CM	1	F31.73		Bipolar disorder, in partial remission, most recent episo	IMO	GHS-PACDC	IMO_2015_R	ACTIVE	N	1900-01-01	9999-12-31	2015-07-10
Diagnosis: ICD10 Code, F00-F99 Define	F01-F99 Mental, Behavioral and Neurodevelopmental disorders	486977	Bipolar I disorder, current or most recent episode manic, in partial remission with mood-incong	ICD10CM	1	F31.73		Bipolar disorder, in partial remission, most recent episo	IMO	GHS-PACDC	IMO_2015_R	ACTIVE	N	1900-01-01	9999-12-31	2015-07-10
Diagnosis: ICD10 Code, F00-F99 Define	F01-F99 Mental, Behavioral and Neurodevelopmental disorders	428972	Bipolar I disorder, current or most recent episode manic, in partial remission (HCC)	ICD10CM	1	F31.73		Bipolar disorder, in partial remission, most recent episo	IMO	GHS-PACDC	IMO_2015_R	ACTIVE	N	1900-01-01	9999-12-31	2015-07-10
Diagnosis: ICD10 Code, F00-F99 Define	F01-F99 Mental, Behavioral and Neurodevelopmental disorders	488658	Bipolar I disorder, mild, current or most recent episode manic, in full remission, with melanchol	ICD10CM	1	F31.74		Bipolar disorder, in full remission, most recent episode r	IMO	GHS-PACDC	IMO_2015_R	ACTIVE	N	1900-01-01	9999-12-31	2015-07-10
Diagnosis: ICD10 Code, F00-F99 Define	F01-F99 Mental, Behavioral and Neurodevelopmental disorders	489512	Mild bipolar I disorder, current or most recent episode manic, in full remission (HCC)	ICD10CM	1	F31.74		Bipolar disorder, in full remission, most recent episode r	IMO	GHS-PACDC	IMO_2015_R	ACTIVE	N	1900-01-01	9999-12-31	2015-07-10
Diagnosis: ICD10 Code, F00-F99 Define	F01-F99 Mental, Behavioral and Neurodevelopmental disorders	486370	Bipolar I disorder, current or most recent episode hypomanic, in full remission with catatonia (H	ICD10CM	1	F31.74		Bipolar disorder, in full remission, most recent episode r	IMO	GHS-PACDC	IMO_2015_R	ACTIVE	N	1900-01-01	2016-10-14	2015-07-10
Diagnosis: ICD10 Code, F00-F99 Define	F01-F99 Mental, Behavioral and Neurodevelopmental disorders	489156	Mild bipolar I disorder, current or most recent episode manic, in full remission, with peripartum	ICD10CM	1	F31.74		Bipolar disorder, in full remission, most recent episode r	IMO	GHS-PACDC	IMO_2015_R	ACTIVE	N	1900-01-01	9999-12-31	2015-07-10
Diagnosis: ICD10 Code, F00-F99 Define	F01-F99 Mental, Behavioral and Neurodevelopmental disorders	268018	Bipolar disorder, in partial remission, most recent episode depressed (HCC)	ICD10CM	1	F31.75		Bipolar disorder, in partial remission, most recent episo	IMO	GHS-PACDC	DX_LEGACY	ACTIVE	N	1900-01-01	9999-12-31	2015-07-10
Diagnosis: ICD10 Code, F00-F99 Define	F01-F99 Mental, Behavioral and Neurodevelopmental disorders	489592	Severe bipolar I disorder, current or most recent episode depressed, in partial remission, with c	ICD10CM	1	F31.75		Bipolar disorder, in partial remission, most recent episo	IMO	GHS-PACDC	IMO_2015_R	ACTIVE	N	1900-01-01	2016-10-14	2015-07-10
Diagnosis: ICD10 Code, F00-F99 Define	F01-F99 Mental, Behavioral and Neurodevelopmental disorders	488630	Bipolar I disorder, severe, current or most recent episode depressed, in partial remission, with r	ICD10CM	1	F31.75		Bipolar disorder, in partial remission, most recent episo	IMO	GHS-PACDC	IMO_2015_R	ACTIVE	N	1900-01-01	9999-12-31	2015-07-10
Diagnosis: ICD10 Code, F00-F99 Define	F01-F99 Mental, Behavioral and Neurodevelopmental disorders	489118	Mild bipolar I disorder, current or most recent episode depressed, in full remission, with catato	ICD10CM	1	F31.76	F31.76,F06.1	Bipolar disorder, in full remission, most recent episode c	IMO	GHS-PACDC	IMO_2015_R	ACTIVE	N	2016-09-17	9999-12-31	2016-09-30
Diagnosis: ICD10 Code, F00-F99 Define	F01-F99 Mental, Behavioral and Neurodevelopmental disorders	488892	Bipolar I disorder, severe, current or most recent episode depressed, in full remission, with peri	ICD10CM	1	F31.76		Bipolar disorder, in full remission, most recent episode c	IMO	GHS-PACDC	IMO_2015_R	ACTIVE	N	1900-01-01	9999-12-31	2015-07-10
Diagnosis: ICD10 Code, F00-F99 Define	F01-F99 Mental, Behavioral and Neurodevelopmental disorders	61328	Bipolar affective disorder, depressed, in full remission (HCC)	ICD10CM	1	F31.76		Bipolar disorder, in full remission, most recent episode c	IMO	GHS-PACDC	DX_LEGACY	ACTIVE	N	1900-01-01	9999-12-31	2015-07-10
Diagnosis: ICD10 Code, F00-F99 Define	F01-F99 Mental, Behavioral and Neurodevelopmental disorders	489401	Moderate bipolar I disorder, current or most recent episode depressed, in full remission, with n	ICD10CM	1	F31.76		Bipolar disorder, in full remission, most recent episode c	IMO	GHS-PACDC	IMO_2015_R	ACTIVE	N	1900-01-01	9999-12-31	2015-07-10
Diagnosis: ICD10 Code, F00-F99 Define	F01-F99 Mental, Behavioral and Neurodevelopmental disorders	2985	Bipolar I disorder, most recent episode mixed, in full remission (HCC)	ICD10CM	1	F31.78		Bipolar disorder, in full remission, most recent episode r	IMO	GHS-PACDC	DX_LEGACY	ACTIVE	N	1900-01-01	9999-12-31	2015-07-10
Diagnosis: ICD10 Code, F00-F99 Define	F01-F99 Mental, Behavioral and Neurodevelopmental disorders	30264	Mixed bipolar I disorder in full remission (HCC)	ICD10CM	1	F31.78		Bipolar disorder, in full remission, most recent episode r	IMO	GHS-PACDC	DX_LEGACY	ACTIVE	N	1900-01-01	9999-12-31	2015-07-10
Diagnosis: ICD10 Code, F00-F99 Define	F01-F99 Mental, Behavioral and Neurodevelopmental disorders	487022	Severe bipolar II disorder, hypomanic episode, in full remission, with seasonal pattern (HCC)	ICD10CM	1	F31.81		Bipolar II disorder	IMO	GHS-PACDC	IMO_2015_R	ACTIVE	N	1900-01-01	9999-12-31	2015-07-10
Diagnosis: ICD10 Code, F00-F99 Define	F01-F99 Mental, Behavioral and Neurodevelopmental disorders	488030	Bipolar II disorder, moderate, depressed, with mood-congruent psychotic features, in partial re	ICD10CM	1	F31.81		Bipolar II disorder	IMO	GHS-PACDC	IMO_2015_R	ACTIVE	N	1900-01-01	9999-12-31	2015-07-10
Diagnosis: ICD10 Code, F00-F99 Define	F01-F99 Mental, Behavioral and Neurodevelopmental disorders	486847	Moderate bipolar II disorder, major depressive episode, in partial remission, with mood-incongr	ICD10CM	1	F31.81		Bipolar II disorder	IMO	GHS-PACDC	IMO_2015_R	ACTIVE	N	1900-01-01	9999-12-31	2015-07-10
Diagnosis: ICD10 Code, F00-F99 Define	F01-F99 Mental, Behavioral and Neurodevelopmental disorders	486115	Mild bipolar II disorder, hypomanic episode, in full remission, with seasonal pattern (HCC)	ICD10CM	1	F31.81		Bipolar II disorder	IMO	GHS-PACDC	IMO_2015_R	ACTIVE	N	1900-01-01	9999-12-31	2015-07-10
Diagnosis: ICD10 Code, F00-F99 Define	F01-F99 Mental, Behavioral and Neurodevelopmental disorders	485448	Moderate bipolar II disorder, depressed, in partial remission, with catatonia (HCC)	ICD10CM	1	F31.81		Bipolar II disorder	IMO	GHS-PACDC	IMO_2015_R	ACTIVE	N	1900-01-01	2016-10-14	2015-07-10
Diagnosis: ICD10 Code, F00-F99 Define	F01-F99 Mental, Behavioral and Neurodevelopmental disorders	485471	Severe bipolar II disorder, depressed, in full remission, with atypical features (HCC)	ICD10CM	1	F31.81		Bipolar II disorder	IMO	GHS-PACDC	IMO_2015_R	ACTIVE	N	1900-01-01	9999-12-31	2015-07-10
Diagnosis: ICD10 Code, F00-F99 Define	F01-F99 Mental, Behavioral and Neurodevelopmental disorders	488224	Bipolar II disorder, moderate, hypomanic, with catatonia, in partial remission (HCC)	ICD10CM	1	F31.81	F31.81,F06.1	Bipolar II disorder	IMO	GHS-PACDC	IMO_2015_R	ACTIVE	N	2016-09-17	9999-12-31	2016-09-30
Diagnosis: ICD10 Code, F00-F99 Define	F01-F99 Mental, Behavioral and Neurodevelopmental disorders	488292	Bipolar II disorder, moderate, hypomanic, with seasonal pattern, in partial remission (HCC)	ICD10CM	1	F31.81		Bipolar II disorder	IMO	GHS-PACDC	IMO_2015_R	ACTIVE	N	1900-01-01	9999-12-31	2015-07-10
Diagnosis: ICD10 Code, F00-F99 Define	F01-F99 Mental, Behavioral and Neurodevelopmental disorders	485465	Moderate bipolar II disorder, major depressive episode, in full remission, with mood-congruent	ICD10CM	1	F31.81		Bipolar II disorder	IMO	GHS-PACDC	IMO_2015_R	ACTIVE	N	1900-01-01	9999-12-31	2015-07-10
Diagnosis: ICD10 Code, F00-F99 Define	F01-F99 Mental, Behavioral and Neurodevelopmental disorders	485493	Severe bipolar II disorder, major depressive episode, in full remission, with catatonia (HCC)	ICD10CM	1	F31.81	F31.81,F06.1	Bipolar II disorder	IMO	GHS-PACDC	IMO_2015_R	ACTIVE	N	2016-09-17	9999-12-31	2016-09-30
Diagnosis: ICD10 Code, F00-F99 Define	F01-F99 Mental, Behavioral and Neurodevelopmental disorders	488178	Bipolar II disorder, severe, depressed, with rapid cycling, in full remission (HCC)	ICD10CM	1	F31.81		Bipolar II disorder	IMO	GHS-PACDC	IMO_2015_R	ACTIVE	N	1900-01-01	9999-12-31	2015-07-10
Diagnosis: ICD10 Code, F00-F99 Define	F01-F99 Mental, Behavioral and Neurodevelopmental disorders	488009	Bipolar II disorder, moderate, depressed, with rapid cycling, in partial remission (HCC)	ICD10CM	1	F31.81		Bipolar II disorder	IMO	GHS-PACDC	IMO_2015_R	ACTIVE	N	1900-01-01	9999-12-31	2015-07-10
Diagnosis: ICD10 Code, F00-F99 Define	F01-F99 Mental, Behavioral and Neurodevelopmental disorders	488015	Bipolar II disorder, mild, hypomanic, with catatonia, in full remission (HCC)	ICD10CM	1	F31.81	F31.81,F06.1	Bipolar II disorder	IMO	GHS-PACDC	IMO_2015_R	ACTIVE	N	2016-09-17	9999-12-31	2016-09-30
Diagnosis: ICD10 Code, F00-F99 Define	F01-F99 Mental, Behavioral and Neurodevelopmental disorders	488276	Bipolar II disorder, severe, depressed, with mixed features, in partial remission (HCC)	ICD10CM	1	F31.81		Bipolar II disorder	IMO	GHS-PACDC	IMO_2015_R	ACTIVE	N	1900-01-01	9999-12-31	2015-07-10
Diagnosis: ICD10 Code, F00-F99 Define	F01-F99 Mental, Behavioral and Neurodevelopmental disorders	486140	Severe bipolar II disorder, hypomanic episode, in partial remission, with catatonia (HCC)	ICD10CM	1	F31.81		Bipolar II disorder	IMO	GHS-PACDC	IMO_2015_R	ACTIVE	N	1900-01-01	2016-10-14	2015-07-10
Diagnosis: ICD10 Code, F00-F99 Define	F01-F99 Mental, Behavioral and Neurodevelopmental disorders	254140	Mild mixed bipolar II disorder (HCC)	ICD10CM	1	F31.81		Bipolar II disorder	IMO	GHS-PACDC	DX_LEGACY	ACTIVE	N	1900-01-01	9999-12-31	2015-07-10
Diagnosis: ICD10 Code, F00-F99 Define	F01-F99 Mental, Behavioral and Neurodevelopmental disorders	487962	Bipolar II disorder, mild, hypomanic, with mood-incongruent psychotic features, in full remissio	ICD10CM	1	F31.81		Bipolar II disorder	IMO	GHS-PACDC	IMO_2015_R	ACTIVE	N	1900-01-01	9999-12-31	2015-07-10
Diagnosis: ICD10 Code, F00-F99 Define	F01-F99 Mental, Behavioral and Neurodevelopmental disorders	490994	Bipolar II, mixed, severe with psychotic behavior (HCC)	ICD10CM	1	F31.81		Bipolar II disorder	IMO	GHS-PACDC	IMO_2015_R	ACTIVE	N	1900-01-01	9999-12-31	2015-07-10
Diagnosis: ICD10 Code, F00-F99 Define	F01-F99 Mental, Behavioral and Neurodevelopmental disorders	485435	Mild bipolar II disorder, major depressive episode, in full remission, with catatonia (HCC)	ICD10CM	1	F31.81		Bipolar II disorder	IMO	GHS-PACDC	IMO_2015_R	ACTIVE	N	1900-01-01	2016-10-14	2015-07-10
Diagnosis: ICD10 Code, F00-F99 Define	F01-F99 Mental, Behavioral and Neurodevelopmental disorders	167749	Sev bipolar II disordr, recnt episode majr depress no psychotc feature (HCC)	ICD10CM	1	F31.81		Bipolar II disorder	IMO	GHS-PACDC	DX_LEGACY	ACTIVE	N	1900-01-01	9999-12-31	2015-07-10

Diagnosis: ICD10 Code, F00-F99 Define	F01-F99 Mental, Behavioral and Neurodevelopmental disorders	485478	Severe bipolar II disorder, depressed, in partial remission, with mixed features (HCC)	ICD10CM	1	F31.81		Bipolar II disorder	IMO	GHS-PACDC	IMO_2015_R	ACTIVE	N	1900-01-01	9999-12-31	2015-07-10
Diagnosis: ICD10 Code, F00-F99 Define	F01-F99 Mental, Behavioral and Neurodevelopmental disorders	2939	Manic disorder, recurrent episode (HCC)	ICD10CM	1	F31.89		Other bipolar disorder	IMO	GHS-PACDC	DX_LEGACY	ACTIVE	N	1900-01-01	9999-12-31	2015-07-10
Diagnosis: ICD10 Code, F00-F99 Define	F01-F99 Mental, Behavioral and Neurodevelopmental disorders	15912	Bipolar disorder (HCC)	ICD10CM	1	F31.9		Bipolar disorder, unspecified	IMO	GHS-PACDC	DX_LEGACY	ACTIVE	N	1900-01-01	9999-12-31	2015-07-10
Diagnosis: ICD10 Code, F00-F99 Define	F01-F99 Mental, Behavioral and Neurodevelopmental disorders	1083230	Bipolar disease during pregnancy, unspecified trimester (HCC)	ICD10CM	2	F31.9	O99.340,F31.9	Bipolar disorder, unspecified	IMO	GHS-PACDC	IMO_2015_R	ACTIVE	N	1900-01-01	9999-12-31	2015-07-10
Diagnosis: ICD10 Code, F00-F99 Define	F01-F99 Mental, Behavioral and Neurodevelopmental disorders	161569	Pediatric bipolar disorder (HCC)	ICD10CM	1	F31.9		Bipolar disorder, unspecified	IMO	GHS-PACDC	DX_LEGACY	ACTIVE	N	1900-01-01	9999-12-31	2015-07-10
Diagnosis: ICD10 Code, F00-F99 Define	F01-F99 Mental, Behavioral and Neurodevelopmental disorders	43714	Manic depressive psychosis (HCC)	ICD10CM	1	F31.9		Bipolar disorder, unspecified	IMO	GHS-PACDC	DX_LEGACY	ACTIVE	N	1900-01-01	9999-12-31	2015-07-10
Diagnosis: ICD10 Code, F00-F99 Define	F01-F99 Mental, Behavioral and Neurodevelopmental disorders	538026	Bipolar disorder, unspecified	ICD10CM	1	F31.9		Bipolar disorder, unspecified	IMO	GHS-PACDC	IMO_2015_R	ACTIVE	Y	1900-01-01	9999-12-31	2015-07-10
Diagnosis: ICD10 Code, F00-F99 Define	F01-F99 Mental, Behavioral and Neurodevelopmental disorders	43708	Bipolar affective (HCC)	ICD10CM	1	F31.9		Bipolar disorder, unspecified	IMO	GHS-PACDC	DX_LEGACY	ACTIVE	N	1900-01-01	9999-12-31	2015-07-10
Diagnosis: ICD10 Code, F00-F99 Define	F01-F99 Mental, Behavioral and Neurodevelopmental disorders	486660	Bipolar I disorder with mood-congruent psychotic features (HCC)	ICD10CM	1	F31.9		Bipolar disorder, unspecified	IMO	GHS-PACDC	IMO_2015_R	ACTIVE	N	1900-01-01	9999-12-31	2015-07-10
Diagnosis: ICD10 Code, F00-F99 Define	F01-F99 Mental, Behavioral and Neurodevelopmental disorders	485382	Major depressive disorder, single episode, mild with atypical features (HCC)	ICD10CM	1	F32.0		Major depressive disorder, single episode, mild	IMO	GHS-PACDC	IMO_2015_R	ACTIVE	N	1900-01-01	9999-12-31	2015-07-10
Diagnosis: ICD10 Code, F00-F99 Define	F01-F99 Mental, Behavioral and Neurodevelopmental disorders	485336	Major depressive disorder, single episode, moderate with anxious distress (HCC)	ICD10CM	1	F32.1		Major depressive disorder, single episode, moderate	IMO	GHS-PACDC	IMO_2015_R	ACTIVE	N	1900-01-01	9999-12-31	2015-07-10
Diagnosis: ICD10 Code, F00-F99 Define	F01-F99 Mental, Behavioral and Neurodevelopmental disorders	485339	Major depressive disorder, single episode, moderate with mood-incongruent psychotic features	ICD10CM	1	F32.1		Major depressive disorder, single episode, moderate	IMO	GHS-PACDC	IMO_2015_R	ACTIVE	N	1900-01-01	9999-12-31	2015-07-10
Diagnosis: ICD10 Code, F00-F99 Define	F01-F99 Mental, Behavioral and Neurodevelopmental disorders	2949	Major depressive disorder, single episode, moderate (HCC)	ICD10CM	1	F32.1		Major depressive disorder, single episode, moderate	IMO	GHS-PACDC	DX_LEGACY	ACTIVE	N	1900-01-01	9999-12-31	2015-07-10
Diagnosis: ICD10 Code, F00-F99 Define	F01-F99 Mental, Behavioral and Neurodevelopmental disorders	318593	MDD (major depressive disorder), severe (HCC)	ICD10CM	1	F32.2		Major depressive disorder, single episode, severe withou	IMO	GHS-PACDC	DX_LEGACY	ACTIVE	N	1900-01-01	9999-12-31	2015-07-10
Diagnosis: ICD10 Code, F00-F99 Define	F01-F99 Mental, Behavioral and Neurodevelopmental disorders	373201	Chronic major depressive disorder	ICD10CM	1	F32.2		Major depressive disorder, single episode, severe withou	IMO	GHS-PACDC	DX_LEGACY	ACTIVE	N	1900-01-01	2016-05-07	2015-07-10
Diagnosis: ICD10 Code, F00-F99 Define	F01-F99 Mental, Behavioral and Neurodevelopmental disorders	161592	Unipolar depression (HCC)	ICD10CM	1	F32.2		Major depressive disorder, single episode, severe withou	IMO	GHS-PACDC	DX_LEGACY	ACTIVE	N	1900-01-01	2016-05-07	2015-07-10
Diagnosis: ICD10 Code, F00-F99 Define	F01-F99 Mental, Behavioral and Neurodevelopmental disorders	147028	Major depression	ICD10CM	1	F32.2		Major depressive disorder, single episode, severe withou	IMO	GHS-PACDC	DX_LEGACY	ACTIVE	N	1900-01-01	2016-05-07	2015-07-10
Diagnosis: ICD10 Code, F00-F99 Define	F01-F99 Mental, Behavioral and Neurodevelopmental disorders	327950	Severe depressive psychosis (HCC)	ICD10CM	1	F32.3		Major depressive disorder, single episode, severe with p	IMO	GHS-PACDC	DX_LEGACY	ACTIVE	N	1900-01-01	9999-12-31	2015-07-10
Diagnosis: ICD10 Code, F00-F99 Define	F01-F99 Mental, Behavioral and Neurodevelopmental disorders	333397	Severe major depression, single episode, with psychotic features, mood-congruent (HCC)	ICD10CM	1	F32.3		Major depressive disorder, single episode, severe with p	IMO	GHS-PACDC	DX_LEGACY	ACTIVE	N	1900-01-01	9999-12-31	2015-07-10
Diagnosis: ICD10 Code, F00-F99 Define	F01-F99 Mental, Behavioral and Neurodevelopmental disorders	30309	Severe major depression with psychotic features, mood-incongruent (HCC)	ICD10CM	1	F32.3		Major depressive disorder, single episode, severe with p	IMO	GHS-PACDC	DX_LEGACY	ACTIVE	N	1900-01-01	9999-12-31	2015-07-10
Diagnosis: ICD10 Code, F00-F99 Define	F01-F99 Mental, Behavioral and Neurodevelopmental disorders	486274	Major depressive disorder, single episode, in partial remission with seasonal pattern (HCC)	ICD10CM	1	F32.4		Major depressive disorder, single episode, in partial rem	IMO	GHS-PACDC	IMO_2015_R	ACTIVE	N	1900-01-01	9999-12-31	2015-07-10
Diagnosis: ICD10 Code, F00-F99 Define	F01-F99 Mental, Behavioral and Neurodevelopmental disorders	163453	Single major depressive episode, in partial or unspecified remission	ICD10CM	1	F32.4		Major depressive disorder, single episode, in partial rem	IMO	GHS-PACDC	DX_LEGACY	DELETED	Y	1900-01-01	2016-05-07	2015-07-10
Diagnosis: ICD10 Code, F00-F99 Define	F01-F99 Mental, Behavioral and Neurodevelopmental disorders	30319	Major depression single episode, in partial remission (HCC)	ICD10CM	1	F32.4		Major depressive disorder, single episode, in partial rem	IMO	GHS-PACDC	DX_LEGACY	ACTIVE	N	1900-01-01	9999-12-31	2015-07-10
Diagnosis: ICD10 Code, F00-F99 Define	F01-F99 Mental, Behavioral and Neurodevelopmental disorders	1125035	Depression of infancy to early childhood, major depression, single episode, in full remission (HC	ICD10CM	1	F32.5		Major depressive disorder, single episode, in full remissi	IMO	GHS-PACDC	IMO_2016_R	ACTIVE	N	1900-01-01	9999-12-31	2016-06-02
Diagnosis: ICD10 Code, F00-F99 Define	F01-F99 Mental, Behavioral and Neurodevelopmental disorders	494111	Depressive psychosis in full remission (HCC)	ICD10CM	1	F32.5		Major depressive disorder, single episode, in full remissi	IMO	GHS-PACDC	IMO_2015_R	ACTIVE	N	1900-01-01	9999-12-31	2015-07-10
Diagnosis: ICD10 Code, F00-F99 Define	F01-F99 Mental, Behavioral and Neurodevelopmental disorders	486827	Major depressive disorder, single episode, in full remission with mood-congruent psychotic feat	ICD10CM	1	F32.5		Major depressive disorder, single episode, in full remissi	IMO	GHS-PACDC	IMO_2015_R	ACTIVE	N	1900-01-01	9999-12-31	2015-07-10
Diagnosis: ICD10 Code, F00-F99 Define	F01-F99 Mental, Behavioral and Neurodevelopmental disorders	1125071	Depression of infancy to early childhood, type I, single episode, in full remission (HCC)	ICD10CM	1	F32.5		Major depressive disorder, single episode, in full remissi	IMO	GHS-PACDC	IMO_2016_R	ACTIVE	N	1900-01-01	9999-12-31	2016-06-02
Diagnosis: ICD10 Code, F00-F99 Define	F01-F99 Mental, Behavioral and Neurodevelopmental disorders	486083	Major depressive disorder, single episode, in full remission with catatonia (HCC)	ICD10CM	1	F32.5		Major depressive disorder, single episode, in full remissi	IMO	GHS-PACDC	IMO_2015_R	ACTIVE	N	1900-01-01	2016-10-14	2015-07-10
Diagnosis: ICD10 Code, F00-F99 Define	F01-F99 Mental, Behavioral and Neurodevelopmental disorders	1125088	Depression of infancy to early childhood, type II	ICD10CM	1	F32.8		Other depressive episodes	IMO	GHS-PACDC	IMO_2016_R	ACTIVE	N	1900-01-01	2016-10-14	2016-06-02
Diagnosis: ICD10 Code, F00-F99 Define	F01-F99 Mental, Behavioral and Neurodevelopmental disorders	359727	MDD (major depressive disorder), single episode with melancholic features	ICD10CM	1	F32.8		Other depressive episodes	IMO	GHS-PACDC	DX_LEGACY	ACTIVE	N	1900-01-01	2016-10-14	2015-07-10
Diagnosis: ICD10 Code, F00-F99 Define	F01-F99 Mental, Behavioral and Neurodevelopmental disorders	158889	Involutional depression	ICD10CM	1	F32.8		Other depressive episodes	IMO	GHS-PACDC	DX_LEGACY	ACTIVE	N	1900-01-01	2016-10-14	2015-07-10
Diagnosis: ICD10 Code, F00-F99 Define	F01-F99 Mental, Behavioral and Neurodevelopmental disorders	30303	Menopausal depression	ICD10CM	1	F32.8		Other depressive episodes	IMO	GHS-PACDC	DX_LEGACY	ACTIVE	N	2016-04-10	2016-10-14	2015-07-10
Diagnosis: ICD10 Code, F00-F99 Define	F01-F99 Mental, Behavioral and Neurodevelopmental disorders	30312	Chronic major depressive disorder, single episode	ICD10CM	1	F32.8		Other depressive episodes	IMO	GHS-PACDC	DX_LEGACY	ACTIVE	N	1900-01-01	2016-05-07	2015-07-10
Diagnosis: ICD10 Code, F00-F99 Define	F01-F99 Mental, Behavioral and Neurodevelopmental disorders	413733	Premenstrual dysphoric disorder in remission	ICD10CM	1	F32.81		Premenstrual dysphoric disorder	IMO	GHS-PACDC	DX_LEGACY	ACTIVE	N	2016-09-17	9999-12-31	2016-09-30
Diagnosis: ICD10 Code, F00-F99 Define	F01-F99 Mental, Behavioral and Neurodevelopmental disorders	485836	Inhalant-induced depressive disorder with moderate or severe use disorder with onset during v	ICD10CM	2	F32.89	F18.24,F32.89,F18.288	Other specified depressive episodes	IMO	GHS-PACDC	IMO_2015_R	ACTIVE	N	2016-09-17	9999-12-31	2016-09-30
Diagnosis: ICD10 Code, F00-F99 Define	F01-F99 Mental, Behavioral and Neurodevelopmental disorders	486362	Inhalant-induced depressive disorder with mild use disorder with onset during withdrawal (HCC	ICD10CM	2	F32.89	F18.24,F32.89,F18.288	Other specified depressive episodes	IMO	GHS-PACDC	IMO_2015_R	ACTIVE	N	2016-09-17	9999-12-31	2016-09-30
Diagnosis: ICD10 Code, F00-F99 Define	F01-F99 Mental, Behavioral and Neurodevelopmental disorders	485948	Cocaine-induced depressive disorder with mild use disorder with onset during withdrawal (HCC	ICD10CM	2	F32.89	F14.24,F32.89,F14.23	Other specified depressive episodes	IMO	GHS-PACDC	IMO_2015_R	ACTIVE	N	2016-09-17	9999-12-31	2016-09-30
Diagnosis: ICD10 Code, F00-F99 Define	F01-F99 Mental, Behavioral and Neurodevelopmental disorders	487071	Inhalant-induced depressive disorder with mild use disorder with onset during intoxication (HC	ICD10CM	2	F32.89	F18.14,F32.89,F18.129	Other specified depressive episodes	IMO	GHS-PACDC	IMO_2015_R	ACTIVE	N	2016-09-17	9999-12-31	2016-09-30
Diagnosis: ICD10 Code, F00-F99 Define	F01-F99 Mental, Behavioral and Neurodevelopmental disorders	718492	Depression complicating pregnancy, antepartum, third trimester	ICD10CM	2	F32.9	O99.343,F32.9	Major depressive disorder, single episode, unspecified	IMO	GHS-PACDC	IMO_2015_R	ACTIVE	N	1900-01-01	9999-12-31	2015-07-10
Diagnosis: ICD10 Code, F00-F99 Define	F01-F99 Mental, Behavioral and Neurodevelopmental disorders	325647	Feeling hopeless	ICD10CM	1	F32.9		Major depressive disorder, single episode, unspecified	IMO	GHS-PACDC	DX_LEGACY	ACTIVE	N	1900-01-01	2016-05-07	2015-07-10
Diagnosis: ICD10 Code, F00-F99 Define	F01-F99 Mental, Behavioral and Neurodevelopmental disorders	140782	Vascular dementia with depressed mood	ICD10CM	2	F32.9	F01.51,F32.9	Major depressive disorder, single episode, unspecified	IMO	GHS-PACDC	DX_LEGACY	ACTIVE	N	1900-01-01	9999-12-31	2015-07-10
Diagnosis: ICD10 Code, F00-F99 Define	F01-F99 Mental, Behavioral and Neurodevelopmental disorders	361098	Dementia in Alzheimer's disease with depression	ICD10CM	3	F32.9	G30.9,F02.80,F32.9	Major depressive disorder, single episode, unspecified	IMO	GHS-PACDC	DX_LEGACY	ACTIVE	N	1900-01-01	9999-12-31	2015-07-10
Diagnosis: ICD10 Code, F00-F99 Define	F01-F99 Mental, Behavioral and Neurodevelopmental disorders	708082	Perinatal depression, third trimester	ICD10CM	2	F32.9	O99.343,F32.9	Major depressive disorder, single episode, unspecified	IMO	GHS-PACDC	IMO_2015_R	ACTIVE	N	1900-01-01	9999-12-31	2015-07-10
Diagnosis: ICD10 Code, F00-F99 Define	F01-F99 Mental, Behavioral and Neurodevelopmental disorders	524518	Depression during pregnancy in third trimester	ICD10CM	2	F32.9	O99.343,F32.9	Major depressive disorder, single episode, unspecified	IMO	GHS-PACDC	IMO_2015_R	ACTIVE	N	2016-04-10	9999-12-31	2015-07-10
Diagnosis: ICD10 Code, F00-F99 Define	F01-F99 Mental, Behavioral and Neurodevelopmental disorders	359727	MDD (major depressive disorder), single episode with melancholic features	ICD10CM	1	F32.9		Major depressive disorder, single episode, unspecified	IMO	GHS-PACDC	DX_LEGACY	ACTIVE	N	2016-09-17	9999-12-31	2016-09-30
Diagnosis: ICD10 Code, F00-F99 Define	F01-F99 Mental, Behavioral and Neurodevelopmental disorders	431974	Perinatal depression in second trimester	ICD10CM	2	F32.9	O99.342,F32.9	Major depressive disorder, single episode, unspecified	IMO	GHS-PACDC	IMO_2015_R	ACTIVE	N	1900-01-01	9999-12-31	2015-07-10
Diagnosis: ICD10 Code, F00-F99 Define	F01-F99 Mental, Behavioral and Neurodevelopmental disorders	1128308	Major depressive disorder with single episode	ICD10CM	1	F32.9		Major depressive disorder, single episode, unspecified	IMO	GHS-PACDC	IMO_2016_R	ACTIVE	N	1900-01-01	9999-12-31	2016-06-02
Diagnosis: ICD10 Code, F00-F99 Define	F01-F99 Mental, Behavioral and Neurodevelopmental disorders	210147	Dementia, vascular, with depression	ICD10CM	2	F32.9	F01.51,F32.9	Major depressive disorder, single episode, unspecified	IMO	GHS-PACDC	DX_LEGACY	ACTIVE	N	1900-01-01	2016-05-07	2015-07-10
Diagnosis: ICD10 Code, F00-F99 Define	F01-F99 Mental, Behavioral and Neurodevelopmental disorders	487014	Major depressive disorder, single episode with seasonal pattern	ICD10CM	1	F32.9		Major depressive disorder, single episode, unspecified	IMO	GHS-PACDC	IMO_2015_R	ACTIVE	N	1900-01-01	9999-12-31	2015-07-10
Diagnosis: ICD10 Code, F00-F99 Define	F01-F99 Mental, Behavioral and Neurodevelopmental disorders	66435	Despondency	ICD10CM	1	F32.9		Major depressive disorder, single episode, unspecified	IMO	GHS-PACDC	DX_LEGACY	ACTIVE	N	1900-01-01	9999-12-31	2015-07-10
Diagnosis: ICD10 Code, F00-F99 Define	F01-F99 Mental, Behavioral and Neurodevelopmental disorders	359726	MDD (major depressive disorder), single episode with catatonic features	ICD10CM	1	F32.9	F32.9,F06.1	Major depressive disorder, single episode, unspecified	IMO	GHS-PACDC	DX_LEGACY	ACTIVE	N	2016-09-17	9999-12-31	2016-09-30
Diagnosis: ICD10 Code, F00-F99 Define	F01-F99 Mental, Behavioral and Neurodevelopmental disorders	489599	Cocaine-induced depressive disorder with onset during withdrawal (HCC)	ICD10CM	2	F32.9	F14.94,F32.9,F19.939	Major depressive disorder, single episode, unspecified	IMO	GHS-PACDC	IMO_2015_R	ACTIVE	N	1900-01-01	9999-12-31	2015-07-10
Diagnosis: ICD10 Code, F00-F99 Define	F01-F99 Mental, Behavioral and Neurodevelopmental disorders	526685	Post-viral psychological depression	ICD10CM	1	F32.9		Major depressive disorder, single episode, unspecified	IMO	GHS-PACDC	IMO_2015_R	ACTIVE	N	1900-01-01	9999-12-31	2015-07-10
Diagnosis: ICD10 Code, F00-F99 Define	F01-F99 Mental, Behavioral and Neurodevelopmental disorders	1125070	Depression of infancy to early childhood, type I, single episode	ICD10CM	1	F32.9		Major depressive disorder, single episode, unspecified	IMO	GHS-PACDC	IMO_2016_R	ACTIVE	N	1900-01-01	9999-12-31	2016-06-02
Diagnosis: ICD10 Code, F00-F99 Define	F01-F99 Mental, Behavioral and Neurodevelopmental disorders	1125088	Depression of infancy to early childhood, type II	ICD10CM	1	F32.9		Major depressive disorder, single episode, unspecified	IMO	GHS-PACDC	IMO_2016_R	ACTIVE	N	2016-09-17	9999-12-31	2016-09-30
Diagnosis: ICD10 Code, F00-F99 Define	F01-F99 Mental, Behavioral and Neurodevelopmental disorders	1125030	Depression of infancy to early childhood, major depression, single episode	ICD10CM	1	F32.9		Major depressive disorder, single episode, unspecified	IMO	GHS-PACDC	IMO_2016_R	ACTIVE	N	1900-01-01	9999-12-31	2016-06-02
Diagnosis: ICD10 Code, F00-F99 Define	F01-F99 Mental, Behavioral and Neurodevelopmental disorders	432258	Depression, controlled	ICD10CM	1	F32.9		Major depressive disorder, single episode, unspecified	IMO	GHS-PACDC	IMO_2015_R	ACTIVE	N	1900-01-01	9999-12-31	2015-07-10
Diagnosis: ICD10 Code, F00-F99 Define	F01-F99 Mental, Behavioral and Neurodevelopmental disorders	30303	Menopausal depression	ICD10CM	1	F32.9		Major depressive disorder, single episode, unspecified	IMO	GHS-PACDC	DX_LEGACY	ACTIVE	N	1900-01-01	2016-05-07	2015-07-10
Diagnosis: ICD10 Code, F00-F99 Define	F01-F99 Mental, Behavioral and Neurodevelopmental disorders	1348714	Depressed mood with feeling of loneliness	ICD10CM	1	F32.9		Major depressive disorder, single episode, unspecified	IMO	GHS-PACDC	IMO_2018_R	ACTIVE	N	1900-01-01	9999-12-31	2017-10-30
Diagnosis: ICD10 Code, F00-F99 Define	F01-F99 Mental, Behavioral and Neurodevelopmental disorders	486075	Major depressive disorder, recurrent episode, moderate with peripartum onset (HCC)	ICD10CM	1	F33.1		Major depressive disorder, recurrent, moderate	IMO	GHS-PACDC	IMO_2015_R	ACTIVE	N	1900-01-01	9999-12-31	2015-07-10
Diagnosis: ICD10 Code, F00-F99 Define	F01-F99 Mental, Behavioral and Neurodevelopmental disorders	408674	Recurrent endogenous depression (HCC)	ICD10CM	1	F33.2		Major depressive disorder, recurrent severe without psy	IMO	GHS-PACDC	DX_LEGACY	ACTIVE	N	1900-01-01	9999-12-31	2015-07-10
Diagnosis: ICD10 Code, F00-F99 Define	F01-F99 Mental, Behavioral and Neurodevelopmental disorders	414524	Severe recurrent major depressive disorder with psychotic features (HCC)	ICD10CM	1	F33.3		Major depressive disorder, recurrent, severe with psych	IMO	GHS-PACDC	DX_LEGACY	ACTIVE	N	1900-01-01	9999-12-31	2015-07-10
Diagnosis: ICD10 Code, F00-F99 Define	F01-F99 Mental, Behavioral and Neurodevelopmental disorders	333400	Severe recurrent major depression with psychotic features, mood-incongruent (HCC)	ICD10CM	1	F33.3		Major depressive disorder, recurrent, severe with psych	IMO	GHS-PACDC	DX_LEGACY	ACTIVE	N	1900-01-01	9999-12-31	2015-07-10
Diagnosis: ICD10 Code, F00-F99 Define	F01-F99 Mental, Behavioral and Neurodevelopmental disorders	337376	Major depressive disorder, recurrent episode, severe, with psychotic behavior (HCC)	ICD10CM	1	F33.3		Major depressive disorder, recurrent, severe with psych	IMO	GHS-PACDC	DX_LEGACY	ACTIVE	N	1900-01-01	9999-12-31	2015-07-10
Diagnosis: ICD10 Code, F00-F99 Define	F01-F99 Mental, Behavioral and Neurodevelopmental disorders	486080	Severe recurrent major depressive disorder with psychotic features with mixed features (HCC)	ICD10CM	1	F33.3		Major depressive disorder, recurrent, severe with psych	IMO	GHS-PACDC	IMO_2015_R	ACTIVE	N	1900-01-01	9999-12-31	2015-07-10
Diagnosis: ICD10 Code, F00-F99 Define	F01-F99 Mental, Behavioral and Neurodevelopmental disorders	166834	Severe recurrent major depression w/psychotic features, mood-congruent (HCC)	ICD10CM	1	F33.3		Major depressive disorder, recurrent, severe with psych	IMO	GHS-PACDC	DX_LEGACY	ACTIVE	N	1900-01-01	9999-12-31	2015-07-10
Diagnosis: ICD10 Code, F00-F99 Define	F01-F99 Mental, Behavioral and Neurodevelopmental disorders	1128318	Severe episode of recurrent major depressive disorder, with psychotic features (HCC)	ICD10CM	1	F33.3		Major depressive disorder, recurrent, severe with psych	IMO	GHS-PACDC	IMO_2016_R	ACTIVE	N	1900-01-01	9999-12-31	2016-06-02
Diagnosis: ICD10 Code, F00-F99 Define	F01-F99 Mental, Behavioral and Neurodevelopmental disorders	1185790	Major depressive disorder, recurrent, in remission, unspecified (HCC)	ICD10CM	1	F33.40		Major depressive disorder, recurrent, in remission, unsp	IMO	GHS-PACDC	IMO_2016_R	ACTIVE	N	1900-01-01	9999-12-31	2016-06-02
Diagnosis: ICD10 Code, F00-F99 Define	F01-F99 Mental, Behavioral and Neurodevelopmental disorders	264145	Recurrent major depressive disorder in remission (HCC)	ICD10CM	1	F33.40		Major depressive disorder, recurrent, in remission, unsp	IMO	GHS-PACDC	DX_LEGACY	ACTIVE	N	1900-01-01	9999-12-31	2015-07-10
Diagnosis: ICD10 Code, F00-F99 Define	F01-F99 Mental, Behavioral and Neurodevelopmental disorders	1098557	Recur major depre, part remis	ICD10CM	1	F33.41		Major depressive disorder, recurrent, in partial remissio	IMO	GHS-PACDC	IMO_2016_R	DELETED	Y	1900-01-01	9999-12-31	2016-06-02
Diagnosis: ICD10 Code, F00-F99 Define	F01-F99 Mental, Behavioral and Neurodevelopmental disorders	485739	Major depressive disorder, recurrent episode, in partial remission with catatonia (HCC)	ICD10CM	1	F33.41		Major depressive disorder, recurrent, in partial remissio	IMO	GHS-PACDC	IMO_2015_R	ACTIVE	N	1900-01-01	2016-10-14	2015-07-10
Diagnosis: ICD10 Code, F00-F99 Define	F01-F99 Mental, Behavioral and Neurodevelopmental disorders	485700	Major depressive disorder, recurrent episode with catatonia (HCC)	ICD10CM	1	F33.9	F33.9,F06.1	Major depressive disorder, recurrent, unspecified	IMO	GHS-PACDC	IMO_2015_R	ACTIVE	N	2016-09-17	9999-12-31	2016-09-30
Diagnosis: ICD10 Code, F00-F99 Define	F01-F99 Mental, Behavioral and Neurodevelopmental disorders	318613	MDD (major depressive disorder), recurrent, with melancholic features (HCC)	ICD10CM	1	F33.9		Major depressive disorder, recurrent, unspecified	IMO	GHS-PACDC	DX_LEGACY	ACTIVE	N	1900-01-01	9999-12-31	2015-07-10
Diagnosis: ICD10 Code, F00-F99 Define	F01-F99 Mental, Behavioral and Neurodevelopmental disorders	299361	Depression, major, recurrent (HCC)	ICD10CM	1	F33.9		Major depressive disorder, recurrent, unspecified	IMO	GHS-PACDC	DX_LEGACY	ACTIVE	N	1900-01-01	9999-12-31	2015-07-10
Diagnosis: ICD10 Code, F00-F99 Define	F01-F99 Mental, Behavioral and Neurodevelopmental disorders	486581	Mild early onset dysthymic disorder, in full remission, with anxious distress, with pure dysthymi	ICD10CM	1	F34.1		Dysthymic disorder	IMO	GHS-PACDC	IMO_2015_R	ACTIVE	N	1900-01-01	9999-12-31	2015-07-10
Diagnosis: ICD10 Code, F00-F99 Define	F01-F99 Mental, Behavioral and Neurodevelopmental disorders	113862	Reactive depression neurotic	ICD10CM	1	F34.1		Dysthymic disorder	IMO	GHS-PACDC	DX_LEGACY	ACTIVE	N	1900-01-01	2016-05-07	2015-07-10
Diagnosis: ICD10 Code, F00-F99 Define	F01-F99 Mental, Behavioral and Neurodevelopmental disorders	485556	Mild late onset dysthymic disorder, in partial remission, with mixed features, with pure dysthym	ICD10CM	1	F34.1		Dysthymic disorder	IMO	GHS-PACDC	IMO_2015_R	ACTIVE	N	1900-01-01	9999-12-31	2015-07-10
Diagnosis: ICD10 Code, F00-F99 Define	F01-F99 Mental, Behavioral and Neurodevelopmental disorders	486229	Severe late onset dysthymic disorder, in partial remission, with atypical features, with intermitte	ICD10CM	1	F34.1		Dysthymic disorder	IMO	GHS-PACDC	IMO_2015_R	ACTIVE	N	1900-01-01	9999-12-31	2015-07-10
Diagnosis: ICD10 Code, F00-F99 Define	F01-F99 Mental, Behavioral and Neurodevelopmental disorders	486863	Moderate early onset dysthymic disorder, in partial remission, with mood-incongruent psychoti	ICD10CM	1	F34.1		Dysthymic disorder	IMO	GHS-PACDC	IMO_2015_R	ACTIVE	N	1900-01-01	9999-12-31	2015-07-10
Diagnosis: ICD10 Code, F00-F99 Define	F01-F99 Mental, Behavioral and Neurodevelopmental disorders	30338	Early onset dysthymia	ICD10CM	1	F34.1		Dysthymic disorder	IMO	GHS-PACDC	DX_LEGACY	ACTIVE	N	1900-01-01	9999-12-31	2015-07-10
Diagnosis: ICD10 Code, F00-F99 Define	F01-F99 Mental, Behavioral and Neurodevelopmental disorders	486195	Moderate late onset dysthymic disorder, in full remission, with mixed features, with pure dysth	ICD10CM	1	F34.1		Dysthymic disorder	IMO	GHS-PACDC	IMO_2015_R	ACTIVE	N	1900-01-01	9999-12-31	2015-07-10
Diagnosis: ICD10 Code, F00-F99 Define	F01-F99 Mental, Behavioral and Neurodevelopmental disorders	486617	Moderate late onset dysthymic disorder, in partial remission, with melancholic features, with pu	ICD10CM	1	F34.1		Dysthymic disorder	IMO	GHS-PACDC	IMO_2015_R	ACTIVE	N	1900-01-01	9999-12-31	2015-07-10
Diagnosis: ICD10 Code, F00-F99 Define	F01-F99 Mental, Behavioral and Neurodevelopmental disorders	485634	Severe early onset dysthymic disorder, in partial remission, with melancholic features, with pur	ICD10CM	1	F34.1		Dysthymic disorder	IMO	GHS-PACDC	IMO_2015_R	ACTIVE	N	1900-01-01	9999-12-31	2015-07-10
Diagnosis: ICD10 Code, F00-F99 Define	F01-F99 Mental, Behavioral and Neurodevelopmental disorders	486632	Severe late onset dysthymic disorder, in full remission, with mood-congruent psychotic features	ICD10CM	1	F34.1		Dysthymic disorder	IMO	GHS-PACDC	IMO_2015_R	ACTIVE	N	1900-01-01	2017-10-14	2015-07-10
Diagnosis: ICD10 Code, F00-F99 Define	F01-F99 Mental, Behavioral and Neurodevelopmental disorders	485626	Severe early onset dysthymic disorder, in full remission, with peripartum onset, with intermitte	ICD10CM	1	F34.1		Dysthymic disorder	IMO	GHS-PACDC	IMO_2015_R	ACTIVE	N	1900-01-01	9999-12-31	2015-07-10
Diagnosis: ICD10 Code, F00-F99 Define	F01-F99 Mental, Behavioral and Neurodevelopmental disorders	486197	Moderate late onset dysthymic disorder, in full remission, with mood-incongruent psychotic fea	ICD10CM	1	F34.1		Dysthymic disorder	IMO	GHS-PACDC	IMO_2015_R	ACTIVE	N	1900-01-01	9999-12-31	2015-07-10
Diagnosis: ICD10 Code, F00-F99 Define	F01-F99 Mental, Behavioral and Neurodevelopmental disorders	485519	Mild early onset dysthymic disorder, in full remission, with mood-incongruent psychotic feature	ICD10CM	1	F34.1		Dysthymic disorder	IMO	GHS-PACDC	IMO_2015_R	ACTIVE	N	1900-01-01	2017-10-14	2015-07-10
Diagnosis: ICD10 Code, F00-F99 Define	F01-F99 Mental, Behavioral and Neurodevelopmental disorders	486592	Mild early onset dysthymic disorder, in partial remission, with mixed features, with pure dysthy	ICD10CM	1	F34.1		Dysthymic disorder	IMO	GHS-PACDC	IMO_2015_R	ACTIVE	N	1900-01-01	9999-12-31	2015-07-10
Diagnosis: ICD10 Code, F00-F99 Define	F01-F99 Mental, Behavioral and Neurodevelopmental disorders	486603	Moderate early onset dysthymic disorder, in full remission, with mixed features, with intermitte	ICD10CM	1	F34.1		Dysthymic disorder	IMO	GHS-PACDC	IMO_2015_R	ACTIVE	N	1900-01-01	9999-12-31	2015-07-10
Diagnosis: ICD10 Code, F00-F99 Define	F01-F99 Mental, Behavioral and Neurodevelopmental disorders	72999	Neurotic depressive state	ICD10CM	1	F34.1		Dysthymic disorder	IMO	GHS-PACDC	DX_LEGACY	ACTIVE	N	1900-01-01	9999-12-31	2015-07-10
Diagnosis: ICD10 Code, F00-F99 Define	F01-F99 Mental, Behavioral and Neurodevelopmental disorders	485621	Severe early onset dysthymic disorder, in full remission, with mood-congruent psychotic feature	ICD10CM	1	F34.1		Dysthymic disorder	IMO	GHS-PACDC	IMO_2015_R	ACTIVE	N	1900-01-01	2017-10-14	2015-07-10
Diagnosis: ICD10 Code, F00-F99 Define	F01-F99 Mental, Behavioral and Neurodevelopmental disorders	485549	Mild late onset dysthymic disorder, in partial remission, with anxious distress, with pure dysthy	ICD10CM	1	F34.1		Dysthymic disorder	IMO	GHS-PACDC	IMO_2015_R	ACTIVE	N	1900-01-01	9999-12-31	2015-07-10
Diagnosis: ICD10 Code, F00-F99 Define	F01-F99 Mental, Behavioral and Neurodevelopmental disorders	486183	Mild late onset dysthymic disorder, in partial remission, with peripartum onset, with persistent	ICD10CM	1	F34.1		Dysthymic disorder	IMO	GHS-PACDC	IMO_2015_R	ACTIVE	N	1900-01-01	9999-12-31	2015-07-10
Diagnosis: ICD10 Code, F00-F99 Define	F01-F99 Mental, Behavioral and Neurodevelopmental disorders	485552	Mild late onset dysthymic disorder, in partial remission, with atypical features, with pure dysthy	ICD10CM	1	F34.1		Dysthymic disorder	IMO	GHS-PACDC	IMO_2015_R	ACTIVE	N	1900-01-01	9999-12-31	2015-07-10
Diagnosis: ICD10 Code, F00-F99 Define	F01-F99 Mental, Behavioral and Neurodevelopmental disorders	485661	Severe late onset dysthymic disorder, in partial remission, with mixed features, with intermitter	ICD10CM	1	F34.1		Dysthymic disorder	IMO	GHS-PACDC	IMO_2015_R	ACTIVE	N	1900-01-01	9999-12-31	2015-07-10

Diagnosis: ICD10 Code, F00-F99 Define	F01-F99 Mental, Behavioral and Neurodevelopmental disorders	485523	Mild early onset dysthymic disorder, in partial remission, with atypical features, with intermitte	ICD10CM	1	F34.1		Dysthymic disorder	IMO	GHS-PACDC	IMO_2015_R	ACTIVE	N	1900-01-01	9999-12-31	2015-07-10
Diagnosis: ICD10 Code, F00-F99 Define	F01-F99 Mental, Behavioral and Neurodevelopmental disorders	486626	Severe early onset dysthymic disorder, in partial remission, with mixed features, with pure dystl	ICD10CM	1	F34.1		Dysthymic disorder	IMO	GHS-PACDC	IMO_2015_R	ACTIVE	N	1900-01-01	9999-12-31	2015-07-10
Diagnosis: ICD10 Code, F00-F99 Define	F01-F99 Mental, Behavioral and Neurodevelopmental disorders	30344	Secondary dysthymia early onset	ICD10CM	1	F34.1		Dysthymic disorder	IMO	GHS-PACDC	DX_LEGACY	ACTIVE	N	1900-01-01	9999-12-31	2015-07-10
Diagnosis: ICD10 Code, F00-F99 Define	F01-F99 Mental, Behavioral and Neurodevelopmental disorders	486874	Severe late onset dysthymic disorder, in partial remission, with mood-congruent psychotic featu	ICD10CM	1	F34.1		Dysthymic disorder	IMO	GHS-PACDC	IMO_2015_R	ACTIVE	N	1900-01-01	9999-12-31	2015-07-10
Diagnosis: ICD10 Code, F00-F99 Define	F01-F99 Mental, Behavioral and Neurodevelopmental disorders	486219	Severe early onset dysthymic disorder, in partial remission, with mixed features, with intermitte	ICD10CM	1	F34.1		Dysthymic disorder	IMO	GHS-PACDC	IMO_2015_R	ACTIVE	N	1900-01-01	9999-12-31	2015-07-10
Diagnosis: ICD10 Code, F00-F99 Define	F01-F99 Mental, Behavioral and Neurodevelopmental disorders	1128991	Persistent depressive disorder with mood-incongruent psychotic features, currently severe	ICD10CM	1	F34.1		Dysthymic disorder	IMO	GHS-PACDC	IMO_2016_R	ACTIVE	N	1900-01-01	9999-12-31	2016-06-02
Diagnosis: ICD10 Code, F00-F99 Define	F01-F99 Mental, Behavioral and Neurodevelopmental disorders	485580	Moderate early onset dysthymic disorder, in partial remission, with mixed features, with interm	ICD10CM	1	F34.1		Dysthymic disorder	IMO	GHS-PACDC	IMO_2015_R	ACTIVE	N	1900-01-01	9999-12-31	2015-07-10
Diagnosis: ICD10 Code, F00-F99 Define	F01-F99 Mental, Behavioral and Neurodevelopmental disorders	133700	Mood disorder with psychosis (HCC)	ICD10CM	1	F39		Unspecified mood [affective] disorder	IMO	GHS-PACDC	DX_LEGACY	ACTIVE	N	1900-01-01	9999-12-31	2015-07-10
Diagnosis: ICD10 Code, F00-F99 Define	F01-F99 Mental, Behavioral and Neurodevelopmental disorders	30249	Mood disorder due to a general medical condition	ICD10CM	1	F39		Unspecified mood [affective] disorder	IMO	GHS-PACDC	DX_LEGACY	ACTIVE	N	1900-01-01	2016-05-07	2015-07-10
Diagnosis: ICD10 Code, F00-F99 Define	F01-F99 Mental, Behavioral and Neurodevelopmental disorders	333423	Panic disorder with agoraphobia, severe agoraphobic avoidance and panic attacks in full remissi	ICD10CM	1	F40.01		Agoraphobia with panic disorder	IMO	GHS-PACDC	DX_LEGACY	ACTIVE	N	1900-01-01	9999-12-31	2015-07-10
Diagnosis: ICD10 Code, F00-F99 Define	F01-F99 Mental, Behavioral and Neurodevelopmental disorders	333407	Panic disorder with agoraphobia, moderate agoraphobic avoidance and moderate panic attacks	ICD10CM	1	F40.01		Agoraphobia with panic disorder	IMO	GHS-PACDC	DX_LEGACY	ACTIVE	N	1900-01-01	9999-12-31	2015-07-10
Diagnosis: ICD10 Code, F00-F99 Define	F01-F99 Mental, Behavioral and Neurodevelopmental disorders	333404	Panic disorder with agoraphobia, agoraphobic avoidance in partial remission and mild panic att	ICD10CM	1	F40.01		Agoraphobia with panic disorder	IMO	GHS-PACDC	DX_LEGACY	ACTIVE	N	1900-01-01	9999-12-31	2015-07-10
Diagnosis: ICD10 Code, F00-F99 Define	F01-F99 Mental, Behavioral and Neurodevelopmental disorders	166583	Panic disorder/agoraphobia, sev agoraphobic avoidance/sev panic attack	ICD10CM	1	F40.01		Agoraphobia with panic disorder	IMO	GHS-PACDC	DX_LEGACY	ACTIVE	N	1900-01-01	9999-12-31	2015-07-10
Diagnosis: ICD10 Code, F00-F99 Define	F01-F99 Mental, Behavioral and Neurodevelopmental disorders	340604	Sensitivity, shyness, and social withdrawal disorder specific to childhood and adolescence	ICD10CM	1	F40.10		Social phobia, unspecified	IMO	GHS-PACDC	DX_LEGACY	ACTIVE	N	2016-09-17	9999-12-31	2016-09-30
Diagnosis: ICD10 Code, F00-F99 Define	F01-F99 Mental, Behavioral and Neurodevelopmental disorders	326892	Social fears	ICD10CM	1	F40.10		Social phobia, unspecified	IMO	GHS-PACDC	DX_LEGACY	ACTIVE	N	1900-01-01	9999-12-31	2015-07-10
Diagnosis: ICD10 Code, F00-F99 Define	F01-F99 Mental, Behavioral and Neurodevelopmental disorders	1316403	Fear of school	ICD10CM	1	F40.10		Social phobia, unspecified	IMO	GHS-PACDC	IMO_2017_R	ACTIVE	N	1900-01-01	9999-12-31	2017-04-14
Diagnosis: ICD10 Code, F00-F99 Define	F01-F99 Mental, Behavioral and Neurodevelopmental disorders	66492	Fear of animals	ICD10CM	1	F40.218		Other animal type phobia	IMO	GHS-PACDC	DX_LEGACY	ACTIVE	N	1900-01-01	9999-12-31	2015-07-10
Diagnosis: ICD10 Code, F00-F99 Define	F01-F99 Mental, Behavioral and Neurodevelopmental disorders	263318	Fear of darkness	ICD10CM	1	F40.228		Other natural environment type phobia	IMO	GHS-PACDC	DX_LEGACY	ACTIVE	N	2016-04-10	9999-12-31	2015-07-10
Diagnosis: ICD10 Code, F00-F99 Define	F01-F99 Mental, Behavioral and Neurodevelopmental disorders	1316429	Fear of hospitals	ICD10CM	1	F40.248		Other situational type phobia	IMO	GHS-PACDC	IMO_2017_R	ACTIVE	N	1900-01-01	9999-12-31	2017-04-14
Diagnosis: ICD10 Code, F00-F99 Define	F01-F99 Mental, Behavioral and Neurodevelopmental disorders	1316416	Fear of fainting	ICD10CM	1	F40.248		Other situational type phobia	IMO	GHS-PACDC	IMO_2017_R	ACTIVE	N	1900-01-01	9999-12-31	2017-04-14
Diagnosis: ICD10 Code, F00-F99 Define	F01-F99 Mental, Behavioral and Neurodevelopmental disorders	254757	Phobia of natural environment	ICD10CM	1	F40.298		Other specified phobia	IMO	GHS-PACDC	DX_LEGACY	ACTIVE	N	1900-01-01	9999-12-31	2015-07-10
Diagnosis: ICD10 Code, F00-F99 Define	F01-F99 Mental, Behavioral and Neurodevelopmental disorders	1350772	Fear of developing malignant neoplasm of endocrine system	ICD10CM	1	F40.298		Other specified phobia	IMO	GHS-PACDC	IMO_2018_R	ACTIVE	N	1900-01-01	9999-12-31	2017-10-30
Diagnosis: ICD10 Code, F00-F99 Define	F01-F99 Mental, Behavioral and Neurodevelopmental disorders	263322	Vaccination phobia	ICD10CM	1	F40.298		Other specified phobia	IMO	GHS-PACDC	DX_LEGACY	ACTIVE	N	1900-01-01	9999-12-31	2015-07-10
Diagnosis: ICD10 Code, F00-F99 Define	F01-F99 Mental, Behavioral and Neurodevelopmental disorders	256789	Cancer phobia	ICD10CM	1	F40.298		Other specified phobia	IMO	GHS-PACDC	DX_LEGACY	ACTIVE	N	2016-04-10	9999-12-31	2015-07-10
Diagnosis: ICD10 Code, F00-F99 Define	F01-F99 Mental, Behavioral and Neurodevelopmental disorders	501266	Fear associated with illness and body function	ICD10CM	1	F40.9		Phobic anxiety disorder, unspecified	IMO	GHS-PACDC	IMO_2015_R	ACTIVE	N	2017-09-17	9999-12-31	2017-10-30
Diagnosis: ICD10 Code, F00-F99 Define	F01-F99 Mental, Behavioral and Neurodevelopmental disorders	69733	Abnormal fear	ICD10CM	1	F40.9		Phobic anxiety disorder, unspecified	IMO	GHS-PACDC	DX_LEGACY	ACTIVE	N	1900-01-01	9999-12-31	2015-07-10
Diagnosis: ICD10 Code, F00-F99 Define	F01-F99 Mental, Behavioral and Neurodevelopmental disorders	333427	Panic disorder without agoraphobia with panic attacks in full remission	ICD10CM	1	F41.0		Panic disorder [episodic paroxysmal anxiety]	IMO	GHS-PACDC	DX_LEGACY	ACTIVE	N	1900-01-01	9999-12-31	2015-07-10
Diagnosis: ICD10 Code, F00-F99 Define	F01-F99 Mental, Behavioral and Neurodevelopmental disorders	166584	Panic disorder without agoraphobia with panic attacks full remission	ICD10CM	1	F41.0		Panic disorder [episodic paroxysmal anxiety]	IMO	GHS-PACDC	DX_LEGACY	ACTIVE	N	1900-01-01	9999-12-31	2015-07-10
Diagnosis: ICD10 Code, F00-F99 Define	F01-F99 Mental, Behavioral and Neurodevelopmental disorders	611565	Panic disorder (episodic paroxysmal anxiety) without agoraphobia	ICD10CM	1	F41.0		Panic disorder [episodic paroxysmal anxiety]	IMO	GHS-PACDC	IMO_2015_R	ACTIVE	Y	1900-01-01	9999-12-31	2015-07-10
Diagnosis: ICD10 Code, F00-F99 Define	F01-F99 Mental, Behavioral and Neurodevelopmental disorders	98343	Episodic paroxysmal anxiety disorder	ICD10CM	1	F41.0		Panic disorder [episodic paroxysmal anxiety]	IMO	GHS-PACDC	DX_LEGACY	ACTIVE	N	1900-01-01	9999-12-31	2015-07-10
Diagnosis: ICD10 Code, F00-F99 Define	F01-F99 Mental, Behavioral and Neurodevelopmental disorders	1195093	Panic disorder (episodic paroxysmal anxiety) without agoraphobia	ICD10CM	1	F41.0		Panic disorder [episodic paroxysmal anxiety]	IMO	GHS-PACDC	IMO_2016_R	ACTIVE	N	1900-01-01	9999-12-31	2016-06-02
Diagnosis: ICD10 Code, F00-F99 Define	F01-F99 Mental, Behavioral and Neurodevelopmental disorders	237347	Anxiety in acute stress reaction	ICD10CM	1	F41.1	F41.1,F43.0	Generalized anxiety disorder	IMO	GHS-PACDC	DX_LEGACY	ACTIVE	N	2016-04-10	9999-12-31	2015-07-10
Diagnosis: ICD10 Code, F00-F99 Define	F01-F99 Mental, Behavioral and Neurodevelopmental disorders	608745	Other mixed anxiety disorders	ICD10CM	1	F41.3		Other mixed anxiety disorders	IMO	GHS-PACDC	IMO_2015_R	ACTIVE	Y	1900-01-01	9999-12-31	2015-07-10
Diagnosis: ICD10 Code, F00-F99 Define	F01-F99 Mental, Behavioral and Neurodevelopmental disorders	252140	Anxiety with somatic features	ICD10CM	1	F41.8		Other specified anxiety disorders	IMO	GHS-PACDC	DX_LEGACY	ACTIVE	N	2016-04-10	9999-12-31	2015-07-10
Diagnosis: ICD10 Code, F00-F99 Define	F01-F99 Mental, Behavioral and Neurodevelopmental disorders	419163	Parental anxiety due to jaundice in newborn	ICD10CM	1	F41.8		Other specified anxiety disorders	IMO	GHS-PACDC	DX_LEGACY	ACTIVE	N	1900-01-01	9999-12-31	2015-07-10
Diagnosis: ICD10 Code, F00-F99 Define	F01-F99 Mental, Behavioral and Neurodevelopmental disorders	329294	Situational anxiety	ICD10CM	1	F41.8		Other specified anxiety disorders	IMO	GHS-PACDC	DX_LEGACY	ACTIVE	N	1900-01-01	9999-12-31	2015-07-10
Diagnosis: ICD10 Code, F00-F99 Define	F01-F99 Mental, Behavioral and Neurodevelopmental disorders	1111043	Anxiety disorder with care-avoidant behavior	ICD10CM	1	F41.8		Other specified anxiety disorders	IMO	GHS-PACDC	IMO_2016_R	ACTIVE	N	1900-01-01	9999-12-31	2016-06-02
Diagnosis: ICD10 Code, F00-F99 Define	F01-F99 Mental, Behavioral and Neurodevelopmental disorders	66663	Anxious mood	ICD10CM	1	F41.9		Anxiety disorder, unspecified	IMO	GHS-PACDC	DX_LEGACY	ACTIVE	N	1900-01-01	9999-12-31	2015-07-10
Diagnosis: ICD10 Code, F00-F99 Define	F01-F99 Mental, Behavioral and Neurodevelopmental disorders	181918	Anxiety and depression	ICD10CM	1	F41.9	F41.9,F32.9	Anxiety disorder, unspecified	IMO	GHS-PACDC	DX_LEGACY	ACTIVE	N	2016-04-10	2017-10-14	2015-07-10
Diagnosis: ICD10 Code, F00-F99 Define	F01-F99 Mental, Behavioral and Neurodevelopmental disorders	470969	Anxiety-related barrier to education	ICD10CM	2	F41.9	Z78.9,F41.9	Anxiety disorder, unspecified	IMO	GHS-PACDC	IMO_2015_R	ACTIVE	N	2017-09-17	9999-12-31	2017-10-30
Diagnosis: ICD10 Code, F00-F99 Define	F01-F99 Mental, Behavioral and Neurodevelopmental disorders	1216846	Anxiety disorder, unspecified type	ICD10CM	1	F41.9		Anxiety disorder, unspecified	IMO	GHS-PACDC	IMO_2016_R	ACTIVE	N	1900-01-01	9999-12-31	2016-06-02
Diagnosis: ICD10 Code, F00-F99 Define	F01-F99 Mental, Behavioral and Neurodevelopmental disorders	521155	Anxiety during pregnancy, antepartum	ICD10CM	2	F41.9	O99.340,F41.9	Anxiety disorder, unspecified	IMO	GHS-PACDC	IMO_2015_R	ACTIVE	N	1900-01-01	9999-12-31	2015-07-10
Diagnosis: ICD10 Code, F00-F99 Define	F01-F99 Mental, Behavioral and Neurodevelopmental disorders	488249	Obsessive-compulsive disorder with poor insight, tic-related	ICD10CM	1	F42	F42,F95.9	Obsessive-compulsive disorder	IMO	GHS-PACDC	IMO_2015_R	ACTIVE	N	1900-01-01	2016-10-14	2015-07-10
Diagnosis: ICD10 Code, F00-F99 Define	F01-F99 Mental, Behavioral and Neurodevelopmental disorders	415163	Obsessive neurosis	ICD10CM	1	F42		Obsessive-compulsive disorder	IMO	GHS-PACDC	DX_LEGACY	ACTIVE	N	1900-01-01	2016-10-14	2015-07-10
Diagnosis: ICD10 Code, F00-F99 Define	F01-F99 Mental, Behavioral and Neurodevelopmental disorders	163815	Obsessional thoughts	ICD10CM	1	F42		Obsessive-compulsive disorder	IMO	GHS-PACDC	DX_LEGACY	ACTIVE	N	1900-01-01	2016-10-14	2015-07-10
Diagnosis: ICD10 Code, F00-F99 Define	F01-F99 Mental, Behavioral and Neurodevelopmental disorders	485342	Tic-related obsessive-compulsive disorder with absent insight or delusional beliefs	ICD10CM	1	F42	F42,F95.9	Obsessive-compulsive disorder	IMO	GHS-PACDC	IMO_2015_R	ACTIVE	N	1900-01-01	2016-10-14	2015-07-10
Diagnosis: ICD10 Code, F00-F99 Define	F01-F99 Mental, Behavioral and Neurodevelopmental disorders	429157	Hoarding disorder	ICD10CM	1	F42		Obsessive-compulsive disorder	IMO	GHS-PACDC	IMO_2015_R	ACTIVE	N	1900-01-01	2016-10-14	2015-07-10
Diagnosis: ICD10 Code, F00-F99 Define	F01-F99 Mental, Behavioral and Neurodevelopmental disorders	1274752	Mixed obsessional thoughts and acts	ICD10CM	1	F42.2		Mixed obsessional thoughts and acts	IMO	GHS-PACDC	IMO_2017_R	ACTIVE	Y	2016-09-17	9999-12-31	2016-09-30
Diagnosis: ICD10 Code, F00-F99 Define	F01-F99 Mental, Behavioral and Neurodevelopmental disorders	429855	Skin-picking disorder	ICD10CM	1	F42.4		Excoriation (skin-picking) disorder	IMO	GHS-PACDC	IMO_2015_R	ACTIVE	N	2016-09-17	9999-12-31	2016-09-30
Diagnosis: ICD10 Code, F00-F99 Define	F01-F99 Mental, Behavioral and Neurodevelopmental disorders	415163	Obsessive neurosis	ICD10CM	1	F42.8		Other obsessive-compulsive disorder	IMO	GHS-PACDC	DX_LEGACY	ACTIVE	N	2016-09-17	9999-12-31	2016-09-30
Diagnosis: ICD10 Code, F00-F99 Define	F01-F99 Mental, Behavioral and Neurodevelopmental disorders	489095	Onset of unknown substance-induced obsessive-compulsive disorder after medication use (HCC	ICD10CM	2	F42.9	F19.988,F42.9	Obsessive-compulsive disorder, unspecified	IMO	GHS-PACDC	IMO_2015_R	ACTIVE	N	2016-09-17	9999-12-31	2016-09-30
Diagnosis: ICD10 Code, F00-F99 Define	F01-F99 Mental, Behavioral and Neurodevelopmental disorders	53262	Obsessive compulsive disorder	ICD10CM	1	F42.9		Obsessive-compulsive disorder, unspecified	IMO	GHS-PACDC	DX_LEGACY	ACTIVE	N	2016-09-17	9999-12-31	2016-09-30
Diagnosis: ICD10 Code, F00-F99 Define	F01-F99 Mental, Behavioral and Neurodevelopmental disorders	1329209	Obsessive-compulsive disorder, unspecified type	ICD10CM	1	F42.9		Obsessive-compulsive disorder, unspecified	IMO	GHS-PACDC	IMO_2017_R	ACTIVE	N	1900-01-01	9999-12-31	2017-04-14
Diagnosis: ICD10 Code, F00-F99 Define	F01-F99 Mental, Behavioral and Neurodevelopmental disorders	3275	Other acute reactions to stress	ICD10CM	1	F43.0		Acute stress reaction	IMO	GHS-PACDC	DX_LEGACY	ACTIVE	N	1900-01-01	2016-05-07	2015-07-10
Diagnosis: ICD10 Code, F00-F99 Define	F01-F99 Mental, Behavioral and Neurodevelopmental disorders	98937	Gross stress reaction (acute)	ICD10CM	1	F43.0		Acute stress reaction	IMO	GHS-PACDC	DX_LEGACY	ACTIVE	N	1900-01-01	9999-12-31	2015-07-10
Diagnosis: ICD10 Code, F00-F99 Define	F01-F99 Mental, Behavioral and Neurodevelopmental disorders	73083	Emotional crisis as acute reaction to exceptional (gross) stress	ICD10CM	1	F43.0		Acute stress reaction	IMO	GHS-PACDC	DX_LEGACY	ACTIVE	N	1900-01-01	9999-12-31	2015-07-10
Diagnosis: ICD10 Code, F00-F99 Define	F01-F99 Mental, Behavioral and Neurodevelopmental disorders	141051	Neurosis, posttraumatic	ICD10CM	1	F43.10		Post-traumatic stress disorder, unspecified	IMO	GHS-PACDC	DX_LEGACY	ACTIVE	N	1900-01-01	9999-12-31	2015-07-10
Diagnosis: ICD10 Code, F00-F99 Define	F01-F99 Mental, Behavioral and Neurodevelopmental disorders	536666	Adjustment disorders	ICD10CM	1	F43.2		Adjustment disorders	IMO	GHS-PACDC	IMO_2015_R	ACTIVE	Y	1900-01-01	9999-12-31	2015-07-10
Diagnosis: ICD10 Code, F00-F99 Define	F01-F99 Mental, Behavioral and Neurodevelopmental disorders	57876	Unspecified adjustment reaction	ICD10CM	1	F43.20		Adjustment disorder, unspecified	IMO	GHS-PACDC	DX_LEGACY	ACTIVE	N	1900-01-01	9999-12-31	2015-07-10
Diagnosis: ICD10 Code, F00-F99 Define	F01-F99 Mental, Behavioral and Neurodevelopmental disorders	73096	Hospitalism (in children)	ICD10CM	1	F43.20		Adjustment disorder, unspecified	IMO	GHS-PACDC	DX_LEGACY	ACTIVE	N	2016-04-10	9999-12-31	2015-07-10
Diagnosis: ICD10 Code, F00-F99 Define	F01-F99 Mental, Behavioral and Neurodevelopmental disorders	48746	Grief reaction	ICD10CM	1	F43.20		Adjustment disorder, unspecified	IMO	GHS-PACDC	DX_LEGACY	ACTIVE	N	2016-04-10	9999-12-31	2015-07-10
Diagnosis: ICD10 Code, F00-F99 Define	F01-F99 Mental, Behavioral and Neurodevelopmental disorders	408268	Specific work inhibition	ICD10CM	1	F43.20		Adjustment disorder, unspecified	IMO	GHS-PACDC	DX_LEGACY	ACTIVE	N	2017-03-31	9999-12-31	2017-04-14
Diagnosis: ICD10 Code, F00-F99 Define	F01-F99 Mental, Behavioral and Neurodevelopmental disorders	96810	Brief depressive adjustment reaction	ICD10CM	1	F43.21		Adjustment disorder with depressed mood	IMO	GHS-PACDC	DX_LEGACY	ACTIVE	N	1900-01-01	9999-12-31	2015-07-10
Diagnosis: ICD10 Code, F00-F99 Define	F01-F99 Mental, Behavioral and Neurodevelopmental disorders	48745	Grief	ICD10CM	1	F43.21		Adjustment disorder with depressed mood	IMO	GHS-PACDC	DX_LEGACY	ACTIVE	N	1900-01-01	2016-05-07	2015-07-10
Diagnosis: ICD10 Code, F00-F99 Define	F01-F99 Mental, Behavioral and Neurodevelopmental disorders	42309	Adjustment reaction with brief depressive reaction	ICD10CM	1	F43.21		Adjustment disorder with depressed mood	IMO	GHS-PACDC	DX_LEGACY	ACTIVE	N	1900-01-01	9999-12-31	2015-07-10
Diagnosis: ICD10 Code, F00-F99 Define	F01-F99 Mental, Behavioral and Neurodevelopmental disorders	424423	Situational depression	ICD10CM	1	F43.21		Adjustment disorder with depressed mood	IMO	GHS-PACDC	IMO_2015_R	ACTIVE	N	1900-01-01	9999-12-31	2015-07-10
Diagnosis: ICD10 Code, F00-F99 Define	F01-F99 Mental, Behavioral and Neurodevelopmental disorders	328233	Adjustment reaction with anxiety	ICD10CM	1	F43.22		Adjustment disorder with anxiety	IMO	GHS-PACDC	DX_LEGACY	ACTIVE	N	1900-01-01	9999-12-31	2015-07-10
Diagnosis: ICD10 Code, F00-F99 Define	F01-F99 Mental, Behavioral and Neurodevelopmental disorders	1313169	Acute adjustment disorder with anxiety	ICD10CM	1	F43.22		Adjustment disorder with anxiety	IMO	GHS-PACDC	IMO_2017_R	ACTIVE	N	1900-01-01	9999-12-31	2017-04-14
Diagnosis: ICD10 Code, F00-F99 Define	F01-F99 Mental, Behavioral and Neurodevelopmental disorders	255196	Situational mixed anxiety and depressive disorder	ICD10CM	1	F43.23		Adjustment disorder with mixed anxiety and depressed	IMO	GHS-PACDC	DX_LEGACY	ACTIVE	N	1900-01-01	9999-12-31	2015-07-10
Diagnosis: ICD10 Code, F00-F99 Define	F01-F99 Mental, Behavioral and Neurodevelopmental disorders	30530	Imu	ICD10CM	1	F43.29		Adjustment disorder with other symptoms	IMO	GHS-PACDC	DX_LEGACY	ACTIVE	N	1900-01-01	2016-05-07	2015-07-10
Diagnosis: ICD10 Code, F00-F99 Define	F01-F99 Mental, Behavioral and Neurodevelopmental disorders	30524	Latah	ICD10CM	1	F43.29		Adjustment disorder with other symptoms	IMO	GHS-PACDC	DX_LEGACY	ACTIVE	N	1900-01-01	2016-05-07	2015-07-10
Diagnosis: ICD10 Code, F00-F99 Define	F01-F99 Mental, Behavioral and Neurodevelopmental disorders	30525	Amurakh	ICD10CM	1	F43.29		Adjustment disorder with other symptoms	IMO	GHS-PACDC	DX_LEGACY	ACTIVE	N	1900-01-01	2016-05-07	2015-07-10
Diagnosis: ICD10 Code, F00-F99 Define	F01-F99 Mental, Behavioral and Neurodevelopmental disorders	30566	Perdida del alma	ICD10CM	1	F43.29		Adjustment disorder with other symptoms	IMO	GHS-PACDC	DX_LEGACY	ACTIVE	N	1900-01-01	9999-12-31	2015-07-10
Diagnosis: ICD10 Code, F00-F99 Define	F01-F99 Mental, Behavioral and Neurodevelopmental disorders	30538	Witigo psychosis	ICD10CM	1	F43.29		Adjustment disorder with other symptoms	IMO	GHS-PACDC	DX_LEGACY	ACTIVE	N	1900-01-01	9999-12-31	2015-07-10
Diagnosis: ICD10 Code, F00-F99 Define	F01-F99 Mental, Behavioral and Neurodevelopmental disorders	30554	Nervios	ICD10CM	1	F43.29		Adjustment disorder with other symptoms	IMO	GHS-PACDC	DX_LEGACY	ACTIVE	N	1900-01-01	9999-12-31	2015-07-10
Diagnosis: ICD10 Code, F00-F99 Define	F01-F99 Mental, Behavioral and Neurodevelopmental disorders	148871	Post-trauma response	ICD10CM	1	F43.29		Adjustment disorder with other symptoms	IMO	GHS-PACDC	DX_LEGACY	ACTIVE	N	1900-01-01	9999-12-31	2015-07-10
Diagnosis: ICD10 Code, F00-F99 Define	F01-F99 Mental, Behavioral and Neurodevelopmental disorders	30564	Espanto	ICD10CM	1	F43.29		Adjustment disorder with other symptoms	IMO	GHS-PACDC	DX_LEGACY	ACTIVE	N	1900-01-01	9999-12-31	2015-07-10
Diagnosis: ICD10 Code, F00-F99 Define	F01-F99 Mental, Behavioral and Neurodevelopmental disorders	417621	Reaction to internal stress	ICD10CM	1	F43.8		Other reactions to severe stress	IMO	GHS-PACDC	DX_LEGACY	ACTIVE	N	1900-01-01	9999-12-31	2015-07-10
Diagnosis: ICD10 Code, F00-F99 Define	F01-F99 Mental, Behavioral and Neurodevelopmental disorders	3032	Psychogenic amnesia	ICD10CM	1	F44.0		Dissociative amnesia	IMO	GHS-PACDC	DX_LEGACY	ACTIVE	N	1900-01-01	9999-12-31	2015-07-10
Diagnosis: ICD10 Code, F00-F99 Define	F01-F99 Mental, Behavioral and Neurodevelopmental disorders	30489	Dissociative fugue	ICD10CM	1	F44.1		Dissociative fugue	IMO	GHS-PACDC	DX_LEGACY	ACTIVE	N	1900-01-01	9999-12-31	2015-07-10
Diagnosis: ICD10 Code, F00-F99 Define	F01-F99 Mental, Behavioral and Neurodevelopmental disorders	485813	Conversion disorder with swallowing symptoms, persistent, without psychological stressor	ICD10CM	1	F44.4		Conversion disorder with motor symptom or deficit	IMO	GHS-PACDC	IMO_2015_R	ACTIVE	N	1900-01-01	9999-12-31	2015-07-10
Diagnosis: ICD10 Code, F00-F99 Define	F01-F99 Mental, Behavioral and Neurodevelopmental disorders	485811	Conversion disorder with weakness or paralysis, acute episode, with psychological stressor	ICD10CM	1	F44.4		Conversion disorder with motor symptom or deficit	IMO	GHS-PACDC	IMO_2015_R	ACTIVE	N	1900-01-01	9999-12-31	2015-07-10
Diagnosis: ICD10 Code, F00-F99 Define	F01-F99 Mental, Behavioral and Neurodevelopmental disorders	487205	Conversion disorder with attacks or seizures, acute episode, with psychological stressor	ICD10CM	1	F44.5		Conversion disorder with seizures or convulsions	IMO	GHS-PACDC	IMO_2015_R	ACTIVE	N	1900-01-01	9999-12-31	2015-07-10
Diagnosis: ICD10 Code, F00-F99 Define	F01-F99 Mental, Behavioral and Neurodevelopmental disorders	489017	Conversion disorder with attacks or seizures, acute episode	ICD10CM	1	F44.5		Conversion disorder with seizures or convulsions	IMO	GHS-PACDC	IMO_2015_R	ACTIVE	N	1900-01-01	9999-12-31	2015-07-10
Diagnosis: ICD10 Code, F00-F99 Define	F01-F99 Mental, Behavioral and Neurodevelopmental disorders	489359	Conversion disorder, acute episode, with mixed symptoms	ICD10CM	1	F44.7		Conversion disorder with mixed symptom presentation	IMO	GHS-PACDC	IMO_2015_R	ACTIVE	N	1900-01-01	9999-12-31	2015-07-10
Diagnosis: ICD10 Code, F00-F99 Define	F01-F99 Mental, Behavioral and Neurodevelopmental disorders	487219	Conversion disorder with mixed symptoms, persistent, with psychological stressor	ICD10CM	1	F44.7		Conversion disorder with mixed symptom presentation	IMO	GHS-PACDC	IMO_2015_R	ACTIVE	N	1900-01-01	9999-12-31	2015-07-10
Diagnosis: ICD10 Code, F00-F99 Define	F01-F99 Mental, Behavioral and Neurodevelopmental disorders	46200	Dual personality	ICD10CM	1	F44.81		Dissociative identity disorder	IMO	GHS-PACDC	DX_LEGACY	ACTIVE	N	1900-01-01	9999-12-31	2015-07-10
Diagnosis: ICD10 Code, F00-F99 Define	F01-F99 Mental, Behavioral and Neurodevelopmental disorders	52646	Multiple personality	ICD10CM	1	F44.81		Dissociative identity disorder	IMO	GHS-PACDC	DX_LEGACY	ACTIVE	N	1900-01-01	9999-12-31	2015-07-10
Diagnosis: ICD10 Code, F00-F99 Define	F01-F99 Mental, Behavioral and Neurodevelopmental disorders	45123	Confusion state	ICD10CM	1	F44.89		Other dissociative and conversion disorders	IMO	GHS-PACDC	DX_LEGACY	ACTIVE	N	1900-01-01	9999-12-31	2015-07-10
Diagnosis: ICD10 Code, F00-F99 Define	F01-F99 Mental, Behavioral and Neurodevelopmental disorders	30510	Ganser syndrome	ICD10CM	1	F44.89		Other dissociative and conversion disorders	IMO	GHS-PACDC	DX_LEGACY	ACTIVE	N	1900-01-01	9999-12-31	2015-07-10
Diagnosis: ICD10 Code, F00-F99 Define	F01-F99 Mental, Behavioral and Neurodevelopmental disorders	258795	Sleep-related dissociative disorder	ICD10CM	1	F44.9		Dissociative and conversion disorder, unspecified	IMO	GHS-PACDC	DX_LEGACY	ACTIVE	N	1900-01-01	9999-12-31	2015-07-10
Diagnosis: ICD10 Code, F00-F99 Define	F01-F99 Mental, Behavioral and Neurodevelopmental disorders	424479	Atypical dissociative disorder	ICD10CM	1	F44.9		Dissociative and conversion disorder, unspecified	IMO	GHS-PACDC	IMO_2015_R	ACTIVE	N	1900-01-01	9999-12-31	2015-07-10
Diagnosis: ICD10 Code, F00-F99 Define	F01-F99 Mental, Behavioral and Neurodevelopmental disorders	361346	Anxiety with somatization	ICD10CM	2	F45.0	F41.9,F45.0	Somatization disorder	IMO	GHS-PACDC	DX_LEGACY	ACTIVE	N	1900-01-01	9999-12-31	2015-07-10
Diagnosis: ICD10 Code, F00-F99 Define	F01-F99 Mental, Behavioral and Neurodevelopmental disorders	15019	Undifferentiated somatoform disorder	ICD10CM	1	F45.1		Undifferentiated somatoform disorder	IMO	GHS-PACDC	DX_LEGACY	ACTIVE	N	1900-01-01	9999-12-31	2015-07-10
Diagnosis: ICD10 Code, F00-F99 Define	F01-F99 Mental, Behavioral and Neurodevelopmental disorders	488406	Persistent moderate somatic symptom disorder	ICD10CM	1	F45.1		Undifferentiated somatoform disorder	IMO	GHS-PACDC	IMO_2015_R	ACTIVE	N	2016-09-17	9999-12-31	2016-09-30

Diagnosis: ICD10 Code, F00-F99 Define	F01-F99 Mental, Behavioral and Neurodevelopmental disorders	487225	Persistent mild somatic symptom disorder with predominant pain	ICD10CM	1	F45.1		Undifferentiated somatoform disorder	IMO	GHS-PACDC	IMO_2015_R	ACTIVE	N	2016-09-17	9999-12-31	2016-09-30
Diagnosis: ICD10 Code, F00-F99 Define	F01-F99 Mental, Behavioral and Neurodevelopmental disorders	30485	Hypochondriacal neurosis	ICD10CM	1	F45.21		Hypochondriasis	IMO	GHS-PACDC	DX_LEGACY	ACTIVE	N	1900-01-01	9999-12-31	2015-07-10
Diagnosis: ICD10 Code, F00-F99 Define	F01-F99 Mental, Behavioral and Neurodevelopmental disorders	633973	Other hypochondriacal disorders	ICD10CM	1	F45.29		Other hypochondriacal disorders	IMO	GHS-PACDC	IMO_2015_R	ACTIVE	N	1900-01-01	9999-12-31	2015-07-10
Diagnosis: ICD10 Code, F00-F99 Define	F01-F99 Mental, Behavioral and Neurodevelopmental disorders	264571	Persistent somatoform pain disorder	ICD10CM	1	F45.41		Pain disorder exclusively related to psychological factors	IMO	GHS-PACDC	DX_LEGACY	ACTIVE	N	1900-01-01	9999-12-31	2015-07-10
Diagnosis: ICD10 Code, F00-F99 Define	F01-F99 Mental, Behavioral and Neurodevelopmental disorders	60505	Pain, psychogenic	ICD10CM	1	F45.41		Pain disorder exclusively related to psychological factors	IMO	GHS-PACDC	DX_LEGACY	ACTIVE	N	1900-01-01	9999-12-31	2015-07-10
Diagnosis: ICD10 Code, F00-F99 Define	F01-F99 Mental, Behavioral and Neurodevelopmental disorders	3233	Endocrine disorder arising from mental factors	ICD10CM	1	F45.8		Other somatoform disorders	IMO	GHS-PACDC	DX_LEGACY	ACTIVE	N	1900-01-01	9999-12-31	2015-07-10
Diagnosis: ICD10 Code, F00-F99 Define	F01-F99 Mental, Behavioral and Neurodevelopmental disorders	147344	Genitourinary malfunctions arising from mental factors	ICD10CM	1	F45.8		Other somatoform disorders	IMO	GHS-PACDC	DX_LEGACY	ACTIVE	N	1900-01-01	9999-12-31	2015-07-10
Diagnosis: ICD10 Code, F00-F99 Define	F01-F99 Mental, Behavioral and Neurodevelopmental disorders	66478	Megalomania	ICD10CM	1	F45.8		Other somatoform disorders	IMO	GHS-PACDC	DX_LEGACY	ACTIVE	N	1900-01-01	9999-12-31	2015-07-10
Diagnosis: ICD10 Code, F00-F99 Define	F01-F99 Mental, Behavioral and Neurodevelopmental disorders	330649	Psychogenic diabetes insipidus	ICD10CM	1	F45.8		Other somatoform disorders	IMO	GHS-PACDC	DX_LEGACY	ACTIVE	N	1900-01-01	9999-12-31	2015-07-10
Diagnosis: ICD10 Code, F00-F99 Define	F01-F99 Mental, Behavioral and Neurodevelopmental disorders	1105363	Dizziness, psychogenic	ICD10CM	1	F45.8		Other somatoform disorders	IMO	GHS-PACDC	IMO_2016_R	ACTIVE	N	1900-01-01	9999-12-31	2016-06-02
Diagnosis: ICD10 Code, F00-F99 Define	F01-F99 Mental, Behavioral and Neurodevelopmental disorders	113628	Pruritic, psychogenic	ICD10CM	1	F45.8		Other somatoform disorders	IMO	GHS-PACDC	DX_LEGACY	ACTIVE	N	1900-01-01	9999-12-31	2015-07-10
Diagnosis: ICD10 Code, F00-F99 Define	F01-F99 Mental, Behavioral and Neurodevelopmental disorders	96984	Cardioneurosis	ICD10CM	1	F45.8		Other somatoform disorders	IMO	GHS-PACDC	DX_LEGACY	ACTIVE	N	1900-01-01	9999-12-31	2015-07-10
Diagnosis: ICD10 Code, F00-F99 Define	F01-F99 Mental, Behavioral and Neurodevelopmental disorders	109133	Hearing loss, nonorganic	ICD10CM	1	F45.8		Other somatoform disorders	IMO	GHS-PACDC	DX_LEGACY	ACTIVE	N	1900-01-01	2016-05-07	2015-07-10
Diagnosis: ICD10 Code, F00-F99 Define	F01-F99 Mental, Behavioral and Neurodevelopmental disorders	488428	Somatic symptom disorder, persistent, mild	ICD10CM	1	F45.8		Other somatoform disorders	IMO	GHS-PACDC	IMO_2015_R	ACTIVE	N	1900-01-01	2016-10-14	2015-07-10
Diagnosis: ICD10 Code, F00-F99 Define	F01-F99 Mental, Behavioral and Neurodevelopmental disorders	73062	Diarrhea, psychogenic	ICD10CM	1	F45.8		Other somatoform disorders	IMO	GHS-PACDC	DX_LEGACY	ACTIVE	N	1900-01-01	9999-12-31	2015-07-10
Diagnosis: ICD10 Code, F00-F99 Define	F01-F99 Mental, Behavioral and Neurodevelopmental disorders	488205	Somatic symptom disorder, persistent, mild, with predominant pain	ICD10CM	1	F45.8		Other somatoform disorders	IMO	GHS-PACDC	IMO_2015_R	ACTIVE	N	1900-01-01	2016-10-14	2015-07-10
Diagnosis: ICD10 Code, F00-F99 Define	F01-F99 Mental, Behavioral and Neurodevelopmental disorders	147877	Shy bladder syndrome	ICD10CM	1	F45.8		Other somatoform disorders	IMO	GHS-PACDC	DX_LEGACY	ACTIVE	N	1900-01-01	9999-12-31	2015-07-10
Diagnosis: ICD10 Code, F00-F99 Define	F01-F99 Mental, Behavioral and Neurodevelopmental disorders	488394	Somatic symptom disorder, mild	ICD10CM	1	F45.8		Other somatoform disorders	IMO	GHS-PACDC	IMO_2015_R	ACTIVE	N	1900-01-01	2016-10-14	2015-07-10
Diagnosis: ICD10 Code, F00-F99 Define	F01-F99 Mental, Behavioral and Neurodevelopmental disorders	245679	Psychogenic respiratory malfunction	ICD10CM	1	F45.8		Other somatoform disorders	IMO	GHS-PACDC	DX_LEGACY	ACTIVE	N	1900-01-01	9999-12-31	2015-07-10
Diagnosis: ICD10 Code, F00-F99 Define	F01-F99 Mental, Behavioral and Neurodevelopmental disorders	156569	Somatoform disorder	ICD10CM	1	F45.9		Somatoform disorder, unspecified	IMO	GHS-PACDC	DX_LEGACY	ACTIVE	N	1900-01-01	9999-12-31	2015-07-10
Diagnosis: ICD10 Code, F00-F99 Define	F01-F99 Mental, Behavioral and Neurodevelopmental disorders	90912	Psychophysiological malfunction	ICD10CM	1	F45.9		Somatoform disorder, unspecified	IMO	GHS-PACDC	DX_LEGACY	ACTIVE	N	1900-01-01	9999-12-31	2015-07-10
Diagnosis: ICD10 Code, F00-F99 Define	F01-F99 Mental, Behavioral and Neurodevelopmental disorders	54940	Psychophysiological disorder	ICD10CM	1	F45.9		Somatoform disorder, unspecified	IMO	GHS-PACDC	DX_LEGACY	ACTIVE	N	1900-01-01	9999-12-31	2015-07-10
Diagnosis: ICD10 Code, F00-F99 Define	F01-F99 Mental, Behavioral and Neurodevelopmental disorders	612283	Other nonpsychotic mental disorders	ICD10CM	1	F48		Other nonpsychotic mental disorders	IMO	GHS-PACDC	IMO_2015_R	ACTIVE	Y	1900-01-01	9999-12-31	2015-07-10
Diagnosis: ICD10 Code, F00-F99 Define	F01-F99 Mental, Behavioral and Neurodevelopmental disorders	100866	Neurotic derealization	ICD10CM	1	F48.1		Depersonalization-derealization syndrome	IMO	GHS-PACDC	DX_LEGACY	ACTIVE	N	1900-01-01	9999-12-31	2015-07-10
Diagnosis: ICD10 Code, F00-F99 Define	F01-F99 Mental, Behavioral and Neurodevelopmental disorders	30494	Depersonalization neurosis	ICD10CM	1	F48.1		Depersonalization-derealization syndrome	IMO	GHS-PACDC	DX_LEGACY	ACTIVE	N	1900-01-01	9999-12-31	2015-07-10
Diagnosis: ICD10 Code, F00-F99 Define	F01-F99 Mental, Behavioral and Neurodevelopmental disorders	1180761	Other specified nonpsychotic mental disorders	ICD10CM	1	F48.8		Other specified nonpsychotic mental disorders	IMO	GHS-PACDC	IMO_2016_R	ACTIVE	N	1900-01-01	9999-12-31	2016-06-02
Diagnosis: ICD10 Code, F00-F99 Define	F01-F99 Mental, Behavioral and Neurodevelopmental disorders	73007	Psychasthenia	ICD10CM	1	F48.8		Other specified nonpsychotic mental disorders	IMO	GHS-PACDC	DX_LEGACY	ACTIVE	N	1900-01-01	9999-12-31	2015-07-10
Diagnosis: ICD10 Code, F00-F99 Define	F01-F99 Mental, Behavioral and Neurodevelopmental disorders	66061	Occupational neurosis	ICD10CM	1	F48.8		Other specified nonpsychotic mental disorders	IMO	GHS-PACDC	DX_LEGACY	ACTIVE	N	1900-01-01	9999-12-31	2015-07-10
Diagnosis: ICD10 Code, F00-F99 Define	F01-F99 Mental, Behavioral and Neurodevelopmental disorders	125569	Combat neurosis	ICD10CM	1	F48.8		Other specified nonpsychotic mental disorders	IMO	GHS-PACDC	DX_LEGACY	ACTIVE	N	2016-04-10	9999-12-31	2015-07-10
Diagnosis: ICD10 Code, F00-F99 Define	F01-F99 Mental, Behavioral and Neurodevelopmental disorders	66056	Nervous breakdown	ICD10CM	1	F48.8		Other specified nonpsychotic mental disorders	IMO	GHS-PACDC	DX_LEGACY	ACTIVE	N	1900-01-01	9999-12-31	2015-07-10
Diagnosis: ICD10 Code, F00-F99 Define	F01-F99 Mental, Behavioral and Neurodevelopmental disorders	29931	Postpartum neurosis	ICD10CM	1	F48.9		Nonpsychotic mental disorder, unspecified	IMO	GHS-PACDC	DX_LEGACY	ACTIVE	N	1900-01-01	9999-12-31	2015-07-10
Diagnosis: ICD10 Code, F00-F99 Define	F01-F99 Mental, Behavioral and Neurodevelopmental disorders	328990	Self-esteem disturbance	ICD10CM	1	F48.9		Nonpsychotic mental disorder, unspecified	IMO	GHS-PACDC	DX_LEGACY	ACTIVE	N	1900-01-01	9999-12-31	2015-07-10
Diagnosis: ICD10 Code, F00-F99 Define	F01-F99 Mental, Behavioral and Neurodevelopmental disorders	156586	Psychogenic disorder	ICD10CM	1	F48.9		Nonpsychotic mental disorder, unspecified	IMO	GHS-PACDC	DX_LEGACY	ACTIVE	N	1900-01-01	9999-12-31	2015-07-10
Diagnosis: ICD10 Code, F00-F99 Define	F01-F99 Mental, Behavioral and Neurodevelopmental disorders	53193	Psychoneurosis	ICD10CM	1	F48.9		Nonpsychotic mental disorder, unspecified	IMO	GHS-PACDC	DX_LEGACY	ACTIVE	N	1900-01-01	9999-12-31	2015-07-10
Diagnosis: ICD10 Code, F00-F99 Define	F01-F99 Mental, Behavioral and Neurodevelopmental disorders	331597	Powerlessness	ICD10CM	1	F48.9		Nonpsychotic mental disorder, unspecified	IMO	GHS-PACDC	DX_LEGACY	ACTIVE	N	1900-01-01	9999-12-31	2015-07-10
Diagnosis: ICD10 Code, F00-F99 Define	F01-F99 Mental, Behavioral and Neurodevelopmental disorders	500451	Disorder of form of thought	ICD10CM	1	F48.9		Nonpsychotic mental disorder, unspecified	IMO	GHS-PACDC	IMO_2015_R	ACTIVE	N	1900-01-01	9999-12-31	2015-07-10
Diagnosis: ICD10 Code, F00-F99 Define	F01-F99 Mental, Behavioral and Neurodevelopmental disorders	423642	Dissociative, conversion and factitious disorders	ICD10CM	1	F48.9		Nonpsychotic mental disorder, unspecified	IMO	GHS-PACDC	IMO_2015_R	DELETED	Y	1900-01-01	9999-12-31	2015-07-10
Diagnosis: ICD10 Code, F00-F99 Define	F01-F99 Mental, Behavioral and Neurodevelopmental disorders	212251	Suicide threat or attempt	ICD10CM	1	F48.9		Nonpsychotic mental disorder, unspecified	IMO	GHS-PACDC	DX_LEGACY	DELETED	Y	1900-01-01	2016-05-07	2015-07-10
Diagnosis: ICD10 Code, F00-F99 Define	F01-F99 Mental, Behavioral and Neurodevelopmental disorders	485228	Moderate anorexia nervosa, restricting type, in partial remission	ICD10CM	1	F50.01		Anorexia nervosa, restricting type	IMO	GHS-PACDC	IMO_2015_R	ACTIVE	N	1900-01-01	9999-12-31	2015-07-10
Diagnosis: ICD10 Code, F00-F99 Define	F01-F99 Mental, Behavioral and Neurodevelopmental disorders	488220	Binge-eating disorder, mild	ICD10CM	1	F50.02		Anorexia nervosa, binge eating/purging type	IMO	GHS-PACDC	IMO_2015_R	ACTIVE	N	1900-01-01	2016-05-07	2015-07-10
Diagnosis: ICD10 Code, F00-F99 Define	F01-F99 Mental, Behavioral and Neurodevelopmental disorders	1113901	Severe binge-eating purging type anorexia nervosa	ICD10CM	1	F50.02		Anorexia nervosa, binge eating/purging type	IMO	GHS-PACDC	IMO_2016_R	ACTIVE	N	1900-01-01	9999-12-31	2016-06-02
Diagnosis: ICD10 Code, F00-F99 Define	F01-F99 Mental, Behavioral and Neurodevelopmental disorders	488103	Bulimia nervosa, in partial remission, moderate	ICD10CM	1	F50.2		Bulimia nervosa	IMO	GHS-PACDC	IMO_2015_R	ACTIVE	N	1900-01-01	9999-12-31	2015-07-10
Diagnosis: ICD10 Code, F00-F99 Define	F01-F99 Mental, Behavioral and Neurodevelopmental disorders	536969	Bulimia nervosa	ICD10CM	1	F50.2		Bulimia nervosa	IMO	GHS-PACDC	IMO_2015_R	ACTIVE	Y	1900-01-01	9999-12-31	2015-07-10
Diagnosis: ICD10 Code, F00-F99 Define	F01-F99 Mental, Behavioral and Neurodevelopmental disorders	3260	Pica	ICD10CM	1	F50.8		Other eating disorders	IMO	GHS-PACDC	DX_LEGACY	ACTIVE	N	1900-01-01	2016-10-14	2015-07-10
Diagnosis: ICD10 Code, F00-F99 Define	F01-F99 Mental, Behavioral and Neurodevelopmental disorders	66499	Hysterical vomiting	ICD10CM	1	F50.8		Other eating disorders	IMO	GHS-PACDC	DX_LEGACY	ACTIVE	N	1900-01-01	2016-10-14	2015-07-10
Diagnosis: ICD10 Code, F00-F99 Define	F01-F99 Mental, Behavioral and Neurodevelopmental disorders	428966	Avoidant or restrictive food intake disorder	ICD10CM	1	F50.8		Other eating disorders	IMO	GHS-PACDC	IMO_2015_R	ACTIVE	N	1900-01-01	2016-10-14	2015-07-10
Diagnosis: ICD10 Code, F00-F99 Define	F01-F99 Mental, Behavioral and Neurodevelopmental disorders	145841	Feeding problem in infant, nonorganic	ICD10CM	1	F50.8		Other eating disorders	IMO	GHS-PACDC	DX_LEGACY	ACTIVE	N	1900-01-01	2016-10-14	2015-07-10
Diagnosis: ICD10 Code, F00-F99 Define	F01-F99 Mental, Behavioral and Neurodevelopmental disorders	985299	Dog bite of left eyelid with infection, subsequent encounter	ICD10CM	2	F50.8	S01.152D,L08.9,W54.0XXD	Local infection of the skin and subcutaneous tissue, unsp	IMO	GHS-PACDC	IMO_2015_R	ACTIVE	N	2016-09-17	9999-12-31	2016-09-30
Diagnosis: ICD10 Code, F00-F99 Define	F01-F99 Mental, Behavioral and Neurodevelopmental disorders	488133	Binge-eating disorder, in full remission, mild	ICD10CM	1	F50.8		Other eating disorders	IMO	GHS-PACDC	IMO_2015_R	ACTIVE	N	2016-04-10	2016-10-14	2015-07-10
Diagnosis: ICD10 Code, F00-F99 Define	F01-F99 Mental, Behavioral and Neurodevelopmental disorders	485238	Moderate binge-eating disorder, in partial remission	ICD10CM	1	F50.8		Other eating disorders	IMO	GHS-PACDC	IMO_2015_R	ACTIVE	N	2016-04-10	2016-10-14	2015-07-10
Diagnosis: ICD10 Code, F00-F99 Define	F01-F99 Mental, Behavioral and Neurodevelopmental disorders	486494	Mild binge-eating disorder, in full remission	ICD10CM	1	F50.81		Binge eating disorder	IMO	GHS-PACDC	IMO_2015_R	ACTIVE	N	2016-09-17	9999-12-31	2016-09-30
Diagnosis: ICD10 Code, F00-F99 Define	F01-F99 Mental, Behavioral and Neurodevelopmental disorders	485235	Extreme binge-eating disorder, in partial remission	ICD10CM	1	F50.81		Binge eating disorder	IMO	GHS-PACDC	IMO_2015_R	ACTIVE	N	2016-09-17	9999-12-31	2016-09-30
Diagnosis: ICD10 Code, F00-F99 Define	F01-F99 Mental, Behavioral and Neurodevelopmental disorders	252684	Compulsive overeater	ICD10CM	1	F50.89		Other specified eating disorder	IMO	GHS-PACDC	DX_LEGACY	ACTIVE	N	2016-09-17	9999-12-31	2016-09-30
Diagnosis: ICD10 Code, F00-F99 Define	F01-F99 Mental, Behavioral and Neurodevelopmental disorders	263050	Pica in adults	ICD10CM	1	F50.89		Other specified eating disorder	IMO	GHS-PACDC	DX_LEGACY	ACTIVE	N	2016-09-17	9999-12-31	2016-09-30
Diagnosis: ICD10 Code, F00-F99 Define	F01-F99 Mental, Behavioral and Neurodevelopmental disorders	387035	Female athletic triad syndrome	ICD10CM	1	F50.9	F50.9,M81.0,N91.2	Eating disorder, unspecified	IMO	GHS-PACDC	DX_LEGACY	ACTIVE	N	1900-01-01	2016-05-07	2015-07-10
Diagnosis: ICD10 Code, F00-F99 Define	F01-F99 Mental, Behavioral and Neurodevelopmental disorders	175957	Pagophagia	ICD10CM	1	F50.9		Eating disorder, unspecified	IMO	GHS-PACDC	DX_LEGACY	ACTIVE	N	1900-01-01	2016-05-07	2015-07-10
Diagnosis: ICD10 Code, F00-F99 Define	F01-F99 Mental, Behavioral and Neurodevelopmental disorders	534232	Primary insomnia	ICD10CM	1	F51.01		Primary insomnia	IMO	GHS-PACDC	IMO_2015_R	ACTIVE	Y	1900-01-01	9999-12-31	2015-07-10
Diagnosis: ICD10 Code, F00-F99 Define	F01-F99 Mental, Behavioral and Neurodevelopmental disorders	255696	Persistent insomnia, nonorganic	ICD10CM	1	F51.01		Primary insomnia	IMO	GHS-PACDC	DX_LEGACY	ACTIVE	N	1900-01-01	9999-12-31	2015-07-10
Diagnosis: ICD10 Code, F00-F99 Define	F01-F99 Mental, Behavioral and Neurodevelopmental disorders	335740	Hyposomnia, insomnia, or sleeplessness associated with conditioned arousal	ICD10CM	1	F51.03		Paradoxical insomnia	IMO	GHS-PACDC	DX_LEGACY	ACTIVE	N	1900-01-01	9999-12-31	2015-07-10
Diagnosis: ICD10 Code, F00-F99 Define	F01-F99 Mental, Behavioral and Neurodevelopmental disorders	144279	Insufficient sleep syndrome	ICD10CM	1	F51.12		Insufficient sleep syndrome	IMO	GHS-PACDC	DX_LEGACY	ACTIVE	N	1900-01-01	9999-12-31	2015-07-10
Diagnosis: ICD10 Code, F00-F99 Define	F01-F99 Mental, Behavioral and Neurodevelopmental disorders	538108	Insufficient sleep syndrome	ICD10CM	1	F51.12		Insufficient sleep syndrome	IMO	GHS-PACDC	IMO_2015_R	ACTIVE	Y	1900-01-01	9999-12-31	2015-07-10
Diagnosis: ICD10 Code, F00-F99 Define	F01-F99 Mental, Behavioral and Neurodevelopmental disorders	126958	Night terrors, primary	ICD10CM	1	F51.4		Sleep terrors [night terrors]	IMO	GHS-PACDC	DX_LEGACY	ACTIVE	N	1900-01-01	9999-12-31	2015-07-10
Diagnosis: ICD10 Code, F00-F99 Define	F01-F99 Mental, Behavioral and Neurodevelopmental disorders	124994	Adult night terrors	ICD10CM	1	F51.4		Sleep terrors [night terrors]	IMO	GHS-PACDC	DX_LEGACY	ACTIVE	N	1900-01-01	9999-12-31	2015-07-10
Diagnosis: ICD10 Code, F00-F99 Define	F01-F99 Mental, Behavioral and Neurodevelopmental disorders	30441	Pavor diurnus	ICD10CM	1	F51.4		Sleep terrors [night terrors]	IMO	GHS-PACDC	DX_LEGACY	ACTIVE	N	1900-01-01	9999-12-31	2015-07-10
Diagnosis: ICD10 Code, F00-F99 Define	F01-F99 Mental, Behavioral and Neurodevelopmental disorders	73069	Nightmares REM-sleep type	ICD10CM	1	F51.5		Nightmare disorder	IMO	GHS-PACDC	DX_LEGACY	ACTIVE	N	1900-01-01	9999-12-31	2015-07-10
Diagnosis: ICD10 Code, F00-F99 Define	F01-F99 Mental, Behavioral and Neurodevelopmental disorders	258790	Circadian rhythm sleep disorder, nonorganic	ICD10CM	2	F51.8	G47.20,F51.8	Other sleep disorders not due to a substance or known	IMO	GHS-PACDC	DX_LEGACY	ACTIVE	N	2016-04-10	9999-12-31	2015-07-10
Diagnosis: ICD10 Code, F00-F99 Define	F01-F99 Mental, Behavioral and Neurodevelopmental disorders	420856	Abnormal Dreams	ICD10CM	1	F51.8		Other sleep disorders not due to a substance or known	IMO	GHS-PACDC	DX_LEGACY	ACTIVE	N	1900-01-01	9999-12-31	2015-07-10
Diagnosis: ICD10 Code, F00-F99 Define	F01-F99 Mental, Behavioral and Neurodevelopmental disorders	421833	Specific disorders of sleep of nonorganic origin	ICD10CM	1	F51.9		Sleep disorder not due to a substance or known physiol	IMO	GHS-PACDC	IMO_2015_R	DELETED	Y	1900-01-01	9999-12-31	2015-07-10
Diagnosis: ICD10 Code, F00-F99 Define	F01-F99 Mental, Behavioral and Neurodevelopmental disorders	486891	Situational mild acquired male hypoactive sexual desire disorder	ICD10CM	1	F52.0		Hypoactive sexual desire disorder	IMO	GHS-PACDC	IMO_2015_R	ACTIVE	N	1900-01-01	9999-12-31	2015-07-10
Diagnosis: ICD10 Code, F00-F99 Define	F01-F99 Mental, Behavioral and Neurodevelopmental disorders	488170	Male hypoactive sexual desire disorder, lifelong, generalized, mild	ICD10CM	1	F52.0		Hypoactive sexual desire disorder	IMO	GHS-PACDC	IMO_2015_R	ACTIVE	N	1900-01-01	9999-12-31	2015-07-10
Diagnosis: ICD10 Code, F00-F99 Define	F01-F99 Mental, Behavioral and Neurodevelopmental disorders	486286	Situational moderate lifelong male hypoactive sexual desire disorder	ICD10CM	1	F52.0		Hypoactive sexual desire disorder	IMO	GHS-PACDC	IMO_2015_R	ACTIVE	N	1900-01-01	9999-12-31	2015-07-10
Diagnosis: ICD10 Code, F00-F99 Define	F01-F99 Mental, Behavioral and Neurodevelopmental disorders	485290	Generalized mild lifelong female sexual interest disorder	ICD10CM	1	F52.22		Female sexual arousal disorder	IMO	GHS-PACDC	IMO_2015_R	ACTIVE	N	1900-01-01	9999-12-31	2015-07-10
Diagnosis: ICD10 Code, F00-F99 Define	F01-F99 Mental, Behavioral and Neurodevelopmental disorders	486030	Situational severe acquired female sexual arousal disorder	ICD10CM	1	F52.22		Female sexual arousal disorder	IMO	GHS-PACDC	IMO_2015_R	ACTIVE	N	1900-01-01	9999-12-31	2015-07-10
Diagnosis: ICD10 Code, F00-F99 Define	F01-F99 Mental, Behavioral and Neurodevelopmental disorders	488294	Female sexual interest/arousal disorder, lifelong, generalized, moderate	ICD10CM	1	F52.22		Female sexual arousal disorder	IMO	GHS-PACDC	IMO_2015_R	ACTIVE	N	1900-01-01	9999-12-31	2015-07-10
Diagnosis: ICD10 Code, F00-F99 Define	F01-F99 Mental, Behavioral and Neurodevelopmental disorders	485287	Generalized mild acquired female sexual arousal disorder	ICD10CM	1	F52.22		Female sexual arousal disorder	IMO	GHS-PACDC	IMO_2015_R	ACTIVE	N	1900-01-01	9999-12-31	2015-07-10
Diagnosis: ICD10 Code, F00-F99 Define	F01-F99 Mental, Behavioral and Neurodevelopmental disorders	486035	Generalized moderate lifelong female orgasmic disorder	ICD10CM	1	F52.31		Female orgasmic disorder	IMO	GHS-PACDC	IMO_2015_R	ACTIVE	N	1900-01-01	9999-12-31	2015-07-10
Diagnosis: ICD10 Code, F00-F99 Define	F01-F99 Mental, Behavioral and Neurodevelopmental disorders	485734	Situational mild lifelong delayed ejaculation	ICD10CM	1	F52.32		Male orgasmic disorder	IMO	GHS-PACDC	IMO_2015_R	ACTIVE	N	2016-09-17	9999-12-31	2016-09-30
Diagnosis: ICD10 Code, F00-F99 Define	F01-F99 Mental, Behavioral and Neurodevelopmental disorders	485353	Generalized moderate lifelong premature ejaculation	ICD10CM	1	F52.4		Premature ejaculation	IMO	GHS-PACDC	IMO_2015_R	ACTIVE	N	1900-01-01	9999-12-31	2015-07-10
Diagnosis: ICD10 Code, F00-F99 Define	F01-F99 Mental, Behavioral and Neurodevelopmental disorders	30406	Ecoutage	ICD10CM	1	F52.8		Other sexual dysfunction not due to a substance or kno	IMO	GHS-PACDC	DX_LEGACY	ACTIVE	N	1900-01-01	9999-12-31	2015-07-10
Diagnosis: ICD10 Code, F00-F99 Define	F01-F99 Mental, Behavioral and Neurodevelopmental disorders	423406	Other specified psychosexual disorders	ICD10CM	1	F52.8		Other sexual dysfunction not due to a substance or kno	IMO	GHS-PACDC	IMO_2015_R	DELETED	Y	1900-01-01	9999-12-31	2015-07-10
Diagnosis: ICD10 Code, F00-F99 Define	F01-F99 Mental, Behavioral and Neurodevelopmental disorders	30376	Coprophilia	ICD10CM	1	F52.8		Other sexual dysfunction not due to a substance or kno	IMO	GHS-PACDC	DX_LEGACY	ACTIVE	N	1900-01-01	9999-12-31	2015-07-10
Diagnosis: ICD10 Code, F00-F99 Define	F01-F99 Mental, Behavioral and Neurodevelopmental disorders	3106	Other specified psychosexual disorder	ICD10CM	1	F52.8		Other sexual dysfunction not due to a substance or kno	IMO	GHS-PACDC	DX_LEGACY	ACTIVE	N	1900-01-01	9999-12-31	2015-07-10
Diagnosis: ICD10 Code, F00-F99 Define	F01-F99 Mental, Behavioral and Neurodevelopmental disorders	255184	Sexual behavior disorder	ICD10CM	1	F52.9		Unspecified sexual dysfunction not due to a substance o	IMO	GHS-PACDC	DX_LEGACY	ACTIVE	N	1900-01-01	9999-12-31	2015-07-10
Diagnosis: ICD10 Code, F00-F99 Define	F01-F99 Mental, Behavioral and Neurodevelopmental disorders	519698	Severe depressive episode with psychotic symptoms in postpartum period	ICD10CM	1	F53		Puerperal psychosis	IMO	GHS-PACDC	IMO_2015_R	ACTIVE	N	1900-01-01	9999-12-31	2015-07-10
Diagnosis: ICD10 Code, F00-F99 Define	F01-F99 Mental, Behavioral and Neurodevelopmental disorders	95693	Post-partum depression	ICD10CM	1	F53		Puerperal psychosis	IMO	GHS-PACDC	DX_LEGACY	ACTIVE	N	1900-01-01	9999-12-31	2015-07-10
Diagnosis: ICD10 Code, F00-F99 Define	F01-F99 Mental, Behavioral and Neurodevelopmental disorders	220768	Depression, postpartum, postpartum condition	ICD10CM	1	F53		Puerperal psychosis	IMO	GHS-PACDC	DX_LEGACY	ACTIVE	N	2016-09-17	9999-12-31	2016-09-30
Diagnosis: ICD10 Code, F00-F99 Define	F01-F99 Mental, Behavioral and Neurodevelopmental disorders	1305327	Postpartum depression associated with fifth pregnancy	ICD10CM	1	F53		Puerperal psychosis	IMO	GHS-PACDC	IMO_2017_R	ACTIVE	N	1900-01-01	9999-12-31	2017-04-14
Diagnosis: ICD10 Code, F00-F99 Define	F01-F99 Mental, Behavioral and Neurodevelopmental disorders	230303	Postpartum psychosis	ICD10CM	2	F53	O99.345,F53	Puerperal psychosis	IMO	GHS-PACDC	DX_LEGACY	ACTIVE	N	2016-04-10	9999-12-31	2015-07-10
Diagnosis: ICD10 Code, F00-F99 Define	F01-F99 Mental, Behavioral and Neurodevelopmental disorders	379200	Psychological factors affecting morbid obesity (HCC)	ICD10CM	2	F54	E66.01,F54	Psychological and behavioral factors associated with dis	IMO	GHS-PACDC	DX_LEGACY	ACTIVE	N	1900-01-01	9999-12-31	2015-07-10
Diagnosis: ICD10 Code, F00-F99 Define	F01-F99 Mental, Behavioral and Neurodevelopmental disorders	262667	Abuse of dietary supplements	ICD10CM	1	F55.1		Abuse of herbal or folk remedies	IMO	GHS-PACDC	DX_LEGACY	ACTIVE	N	1900-01-01	2016-05-07	2015-07-10
Diagnosis: ICD10 Code, F00-F99 Define	F01-F99 Mental, Behavioral and Neurodevelopmental disorders	262670	Abuse of non-prescription analgesics	ICD10CM	1	F55.8		Abuse of other non-psychoactive substances	IMO	GHS-PACDC	DX_LEGACY	ACTIVE	N	1900-01-01	9999-12-31	2015-07-10
Diagnosis: ICD10 Code, F00-F99 Define	F01-F99 Mental, Behavioral and Neurodevelopmental disorders	379807	Excessive use of non-steroidal anti-inflammatory drugs, continuous	ICD10CM	1	F55.8		Abuse of other non-psychoactive substances	IMO	GHS-PACDC	DX_LEGACY	ACTIVE	N	1900-01-01	9999-12-31	2015-07-10
Diagnosis: ICD10 Code, F00-F99 Define	F01-F99 Mental, Behavioral and Neurodevelopmental disorders	262668	Abuse of nutritional supplements	ICD10CM	1	F55.8		Abuse of other non-psychoactive substances	IMO	GHS-PACDC	DX_LEGACY	ACTIVE	N	2016-04-10	9999-12-31	2015-07-10
Diagnosis: ICD10 Code, F00-F99 Define	F01-F99 Mental, Behavioral and Neurodevelopmental disorders	1191580	Unspecified behavioral syndromes associated with physiological disturbances and physical facto	ICD10CM	1	F59		Unspecified behavioral syndromes associated with physi	IMO	GHS-PACDC	IMO_2016_R	ACTIVE	N	1900-01-01	9999-12-31	2016-06-02
Diagnosis: ICD10 Code, F00-F99 Define	F01-F99 Mental, Behavioral and Neurodevelopmental disorders	98111	Dissocial personality disorder	ICD10CM	1	F60.2		Antisocial personality disorder	IMO	GHS-PACDC	DX_LEGACY	ACTIVE	N	1900-01-01	9999-12-31	2015-07-10

Diagnosis: ICD10 Code, F00-F99 Define	F01-F99 Mental, Behavioral and Neurodevelopmental disorders	43011	Psychopathic personality	ICD10CM	1	F60.2		Antisocial personality disorder	IMO	GHS-PACDC	DX_LEGACY	ACTIVE	N	1900-01-01	9999-12-31	2015-07-10
Diagnosis: ICD10 Code, F00-F99 Define	F01-F99 Mental, Behavioral and Neurodevelopmental disorders	1258149	Emotionally unstable borderline personality disorder in adolescent	ICD10CM	1	F60.3		Borderline personality disorder	IMO	GHS-PACDC	IMO_2017_R	ACTIVE	N	1900-01-01	9999-12-31	2016-09-30
Diagnosis: ICD10 Code, F00-F99 Define	F01-F99 Mental, Behavioral and Neurodevelopmental disorders	50810	Labile personality	ICD10CM	1	F60.4		Histrionic personality disorder	IMO	GHS-PACDC	DX_LEGACY	ACTIVE	N	1900-01-01	9999-12-31	2015-07-10
Diagnosis: ICD10 Code, F00-F99 Define	F01-F99 Mental, Behavioral and Neurodevelopmental disorders	97313	Compulsive behavior disorder	ICD10CM	1	F60.5		Obsessive-compulsive personality disorder	IMO	GHS-PACDC	DX_LEGACY	ACTIVE	N	1900-01-01	9999-12-31	2015-07-10
Diagnosis: ICD10 Code, F00-F99 Define	F01-F99 Mental, Behavioral and Neurodevelopmental disorders	540059	Other specific personality disorders	ICD10CM	1	F60.89		Other specific personality disorders	IMO	GHS-PACDC	IMO_2015_R	ACTIVE	Y	1900-01-01	9999-12-31	2015-07-10
Diagnosis: ICD10 Code, F00-F99 Define	F01-F99 Mental, Behavioral and Neurodevelopmental disorders	420414	Inability to maintain self-control	ICD10CM	1	F60.89		Other specific personality disorders	IMO	GHS-PACDC	DX_LEGACY	ACTIVE	N	1900-01-01	2016-10-14	2015-07-10
Diagnosis: ICD10 Code, F00-F99 Define	F01-F99 Mental, Behavioral and Neurodevelopmental disorders	163781	Hypomanic personality trait	ICD10CM	1	F60.89		Other specific personality disorders	IMO	GHS-PACDC	DX_LEGACY	ACTIVE	N	1900-01-01	9999-12-31	2015-07-10
Diagnosis: ICD10 Code, F00-F99 Define	F01-F99 Mental, Behavioral and Neurodevelopmental disorders	64797	Personality, pathologic NEC	ICD10CM	1	F60.89		Other specific personality disorders	IMO	GHS-PACDC	DX_LEGACY	DELETED	Y	2016-09-17	9999-12-31	2016-09-30
Diagnosis: ICD10 Code, F00-F99 Define	F01-F99 Mental, Behavioral and Neurodevelopmental disorders	1258196	Cluster B personality disorder in adult	ICD10CM	1	F60.9		Personality disorder, unspecified	IMO	GHS-PACDC	IMO_2017_R	ACTIVE	N	1900-01-01	9999-12-31	2016-09-30
Diagnosis: ICD10 Code, F00-F99 Define	F01-F99 Mental, Behavioral and Neurodevelopmental disorders	1258200	Cluster A personality disorder in adolescent	ICD10CM	1	F60.9		Personality disorder, unspecified	IMO	GHS-PACDC	IMO_2017_R	ACTIVE	N	1900-01-01	9999-12-31	2016-09-30
Diagnosis: ICD10 Code, F00-F99 Define	F01-F99 Mental, Behavioral and Neurodevelopmental disorders	261960	Uncontrolled gambling or betting	ICD10CM	1	F63.0		Pathological gambling	IMO	GHS-PACDC	DX_LEGACY	ACTIVE	N	1900-01-01	9999-12-31	2015-07-10
Diagnosis: ICD10 Code, F00-F99 Define	F01-F99 Mental, Behavioral and Neurodevelopmental disorders	488336	Gambling disorder, episodic, in early remission, severe	ICD10CM	1	F63.0		Pathological gambling	IMO	GHS-PACDC	IMO_2015_R	ACTIVE	N	1900-01-01	9999-12-31	2015-07-10
Diagnosis: ICD10 Code, F00-F99 Define	F01-F99 Mental, Behavioral and Neurodevelopmental disorders	488031	Gambling disorder, persistent, mild	ICD10CM	1	F63.0		Pathological gambling	IMO	GHS-PACDC	IMO_2015_R	ACTIVE	N	1900-01-01	9999-12-31	2015-07-10
Diagnosis: ICD10 Code, F00-F99 Define	F01-F99 Mental, Behavioral and Neurodevelopmental disorders	485254	Severe persistent gambling disorder, in sustained remission	ICD10CM	1	F63.0		Pathological gambling	IMO	GHS-PACDC	IMO_2015_R	ACTIVE	N	1900-01-01	9999-12-31	2015-07-10
Diagnosis: ICD10 Code, F00-F99 Define	F01-F99 Mental, Behavioral and Neurodevelopmental disorders	488392	Gambling disorder, episodic	ICD10CM	1	F63.0		Pathological gambling	IMO	GHS-PACDC	IMO_2015_R	ACTIVE	N	1900-01-01	9999-12-31	2015-07-10
Diagnosis: ICD10 Code, F00-F99 Define	F01-F99 Mental, Behavioral and Neurodevelopmental disorders	1258210	Pyromania in adult	ICD10CM	1	F63.1		Pyromania	IMO	GHS-PACDC	IMO_2017_R	ACTIVE	N	1900-01-01	9999-12-31	2016-09-30
Diagnosis: ICD10 Code, F00-F99 Define	F01-F99 Mental, Behavioral and Neurodevelopmental disorders	1258024	Kleptomania in adolescent	ICD10CM	1	F63.2		Kleptomania	IMO	GHS-PACDC	IMO_2017_R	ACTIVE	N	1900-01-01	9999-12-31	2016-09-30
Diagnosis: ICD10 Code, F00-F99 Define	F01-F99 Mental, Behavioral and Neurodevelopmental disorders	156966	Hair pulling	ICD10CM	1	F63.3		Trichotillomania	IMO	GHS-PACDC	DX_LEGACY	ACTIVE	N	1900-01-01	9999-12-31	2015-07-10
Diagnosis: ICD10 Code, F00-F99 Define	F01-F99 Mental, Behavioral and Neurodevelopmental disorders	1258198	Internet addiction in adult	ICD10CM	1	F63.89		Other impulse disorders	IMO	GHS-PACDC	IMO_2017_R	ACTIVE	N	1900-01-01	9999-12-31	2016-09-30
Diagnosis: ICD10 Code, F00-F99 Define	F01-F99 Mental, Behavioral and Neurodevelopmental disorders	3326	Other disorder of impulse control	ICD10CM	1	F63.89		Other impulse disorders	IMO	GHS-PACDC	DX_LEGACY	ACTIVE	N	1900-01-01	9999-12-31	2015-07-10
Diagnosis: ICD10 Code, F00-F99 Define	F01-F99 Mental, Behavioral and Neurodevelopmental disorders	76391	Impulse control disorder, unspecified	ICD10CM	1	F63.9		Impulse disorder, unspecified	IMO	GHS-PACDC	DX_LEGACY	ACTIVE	N	1900-01-01	9999-12-31	2015-07-10
Diagnosis: ICD10 Code, F00-F99 Define	F01-F99 Mental, Behavioral and Neurodevelopmental disorders	201261	Disorder of impulse control	ICD10CM	1	F63.9		Impulse disorder, unspecified	IMO	GHS-PACDC	DX_LEGACY	ACTIVE	N	1900-01-01	9999-12-31	2015-07-10
Diagnosis: ICD10 Code, F00-F99 Define	F01-F99 Mental, Behavioral and Neurodevelopmental disorders	1258167	Gender dysphoria in adult	ICD10CM	1	F64.0		Transsexualism	IMO	GHS-PACDC	IMO_2017_R	ACTIVE	N	1900-01-01	9999-12-31	2016-09-30
Diagnosis: ICD10 Code, F00-F99 Define	F01-F99 Mental, Behavioral and Neurodevelopmental disorders	168888	Gender identity disorder of adulthood, s/p gender reassignment surgery	ICD10CM	1	F64.0	F64.0,Z87.890	Transsexualism	IMO	GHS-PACDC	DX_LEGACY	ACTIVE	N	2016-09-17	9999-12-31	2016-09-30
Diagnosis: ICD10 Code, F00-F99 Define	F01-F99 Mental, Behavioral and Neurodevelopmental disorders	417770	Female to male transsexuality	ICD10CM	1	F64.0		Transsexualism	IMO	GHS-PACDC	DX_LEGACY	ACTIVE	N	2016-09-17	9999-12-31	2016-09-30
Diagnosis: ICD10 Code, F00-F99 Define	F01-F99 Mental, Behavioral and Neurodevelopmental disorders	1108827	Female-to-male transgender person	ICD10CM	1	F64.1		Dual role transvestism	IMO	GHS-PACDC	IMO_2016_R	ACTIVE	N	1900-01-01	2016-10-14	2016-06-02
Diagnosis: ICD10 Code, F00-F99 Define	F01-F99 Mental, Behavioral and Neurodevelopmental disorders	102690	Trans-sexuality with asexual history	ICD10CM	1	F64.1		Dual role transvestism	IMO	GHS-PACDC	DX_LEGACY	ACTIVE	N	1900-01-01	2016-10-14	2015-07-10
Diagnosis: ICD10 Code, F00-F99 Define	F01-F99 Mental, Behavioral and Neurodevelopmental disorders	57557	Transvestitism	ICD10CM	1	F64.1		Dual role transvestism	IMO	GHS-PACDC	DX_LEGACY	ACTIVE	N	1900-01-01	9999-12-31	2015-07-10
Diagnosis: ICD10 Code, F00-F99 Define	F01-F99 Mental, Behavioral and Neurodevelopmental disorders	490459	Transgendered	ICD10CM	1	F64.1		Dual role transvestism	IMO	GHS-PACDC	IMO_2015_R	DELETED	Y	1900-01-01	2016-10-14	2015-07-10
Diagnosis: ICD10 Code, F00-F99 Define	F01-F99 Mental, Behavioral and Neurodevelopmental disorders	90882	Trans-sexualism with sexual history	ICD10CM	1	F64.1		Dual role transvestism	IMO	GHS-PACDC	DX_LEGACY	ACTIVE	N	1900-01-01	2016-10-14	2015-07-10
Diagnosis: ICD10 Code, F00-F99 Define	F01-F99 Mental, Behavioral and Neurodevelopmental disorders	417294	Female to male transsexual	ICD10CM	1	F64.1		Dual role transvestism	IMO	GHS-PACDC	DX_LEGACY	ACTIVE	N	1900-01-01	2016-10-14	2015-07-10
Diagnosis: ICD10 Code, F00-F99 Define	F01-F99 Mental, Behavioral and Neurodevelopmental disorders	253339	Gender identity disorder in adult	ICD10CM	1	F64.1		Dual role transvestism	IMO	GHS-PACDC	DX_LEGACY	ACTIVE	N	1900-01-01	2016-10-14	2015-07-10
Diagnosis: ICD10 Code, F00-F99 Define	F01-F99 Mental, Behavioral and Neurodevelopmental disorders	3090	Gender identity disorder of childhood	ICD10CM	1	F64.2		Gender identity disorder of childhood	IMO	GHS-PACDC	DX_LEGACY	ACTIVE	N	1900-01-01	9999-12-31	2015-07-10
Diagnosis: ICD10 Code, F00-F99 Define	F01-F99 Mental, Behavioral and Neurodevelopmental disorders	168886	Gender identity disorder of adolescence, s/p gender reassignment	ICD10CM	1	F64.8		Other gender identity disorders	IMO	GHS-PACDC	DX_LEGACY	ACTIVE	N	1900-01-01	2016-05-07	2015-07-10
Diagnosis: ICD10 Code, F00-F99 Define	F01-F99 Mental, Behavioral and Neurodevelopmental disorders	98848	Gender role disorder	ICD10CM	1	F64.9		Gender identity disorder, unspecified	IMO	GHS-PACDC	DX_LEGACY	ACTIVE	N	1900-01-01	9999-12-31	2015-07-10
Diagnosis: ICD10 Code, F00-F99 Define	F01-F99 Mental, Behavioral and Neurodevelopmental disorders	1123948	Transvestic disorder in controlled environment	ICD10CM	1	F65.1		Transvestic fetishism	IMO	GHS-PACDC	IMO_2016_R	ACTIVE	N	1900-01-01	9999-12-31	2016-06-02
Diagnosis: ICD10 Code, F00-F99 Define	F01-F99 Mental, Behavioral and Neurodevelopmental disorders	1123943	Transvestic disorder with fetishism in full remission	ICD10CM	1	F65.1		Transvestic fetishism	IMO	GHS-PACDC	IMO_2016_R	ACTIVE	N	1900-01-01	9999-12-31	2016-06-02
Diagnosis: ICD10 Code, F00-F99 Define	F01-F99 Mental, Behavioral and Neurodevelopmental disorders	30379	Compulsive exhibitionism	ICD10CM	1	F65.2		Exhibitionism	IMO	GHS-PACDC	DX_LEGACY	ACTIVE	N	1900-01-01	9999-12-31	2015-07-10
Diagnosis: ICD10 Code, F00-F99 Define	F01-F99 Mental, Behavioral and Neurodevelopmental disorders	1123944	Sexual masochism disorder with asphyxiophilia in controlled environment	ICD10CM	1	F65.51		Sexual masochism	IMO	GHS-PACDC	IMO_2016_R	ACTIVE	N	1900-01-01	9999-12-31	2016-06-02
Diagnosis: ICD10 Code, F00-F99 Define	F01-F99 Mental, Behavioral and Neurodevelopmental disorders	55802	Sadism	ICD10CM	1	F65.52		Sexual sadism	IMO	GHS-PACDC	DX_LEGACY	ACTIVE	N	1900-01-01	9999-12-31	2015-07-10
Diagnosis: ICD10 Code, F00-F99 Define	F01-F99 Mental, Behavioral and Neurodevelopmental disorders	30384	Frottage	ICD10CM	1	F65.81		Frotteurism	IMO	GHS-PACDC	DX_LEGACY	ACTIVE	N	1900-01-01	9999-12-31	2015-07-10
Diagnosis: ICD10 Code, F00-F99 Define	F01-F99 Mental, Behavioral and Neurodevelopmental disorders	3081	Zoophilia	ICD10CM	1	F65.89		Other paraphilias	IMO	GHS-PACDC	DX_LEGACY	ACTIVE	N	1900-01-01	9999-12-31	2015-07-10
Diagnosis: ICD10 Code, F00-F99 Define	F01-F99 Mental, Behavioral and Neurodevelopmental disorders	30374	Necrophilia	ICD10CM	1	F65.89		Other paraphilias	IMO	GHS-PACDC	DX_LEGACY	ACTIVE	N	1900-01-01	9999-12-31	2015-07-10
Diagnosis: ICD10 Code, F00-F99 Define	F01-F99 Mental, Behavioral and Neurodevelopmental disorders	1178529	Other paraphilias	ICD10CM	1	F65.89		Other paraphilias	IMO	GHS-PACDC	IMO_2016_R	DELETED	Y	1900-01-01	9999-12-31	2016-06-02
Diagnosis: ICD10 Code, F00-F99 Define	F01-F99 Mental, Behavioral and Neurodevelopmental disorders	53992	Paraphilia	ICD10CM	1	F65.9		Paraphilia, unspecified	IMO	GHS-PACDC	DX_LEGACY	ACTIVE	N	1900-01-01	9999-12-31	2015-07-10
Diagnosis: ICD10 Code, F00-F99 Define	F01-F99 Mental, Behavioral and Neurodevelopmental disorders	1258006	Sexual maturation disorder of sexual identity in pediatric patient	ICD10CM	1	F66		Other sexual disorders	IMO	GHS-PACDC	IMO_2017_R	ACTIVE	N	1900-01-01	9999-12-31	2016-09-30
Diagnosis: ICD10 Code, F00-F99 Define	F01-F99 Mental, Behavioral and Neurodevelopmental disorders	1258148	Gender identity uncertainty in adult	ICD10CM	1	F66		Other sexual disorders	IMO	GHS-PACDC	IMO_2017_R	ACTIVE	N	1900-01-01	9999-12-31	2016-09-30
Diagnosis: ICD10 Code, F00-F99 Define	F01-F99 Mental, Behavioral and Neurodevelopmental disorders	1258042	Sexual maturation disorder of sexual orientation in pediatric patient	ICD10CM	1	F66		Other sexual disorders	IMO	GHS-PACDC	IMO_2017_R	ACTIVE	N	1900-01-01	9999-12-31	2016-09-30
Diagnosis: ICD10 Code, F00-F99 Define	F01-F99 Mental, Behavioral and Neurodevelopmental disorders	61340	Other and unspecified factitious illness	ICD10CM	1	F68.10		Factitious disorder, unspecified	IMO	GHS-PACDC	DX_LEGACY	ACTIVE	N	1900-01-01	9999-12-31	2015-07-10
Diagnosis: ICD10 Code, F00-F99 Define	F01-F99 Mental, Behavioral and Neurodevelopmental disorders	415825	Pseudoparasitic dysesthesia (HCC)	ICD10CM	1	F68.11		Factitious disorder with predominantly psychological sig	IMO	GHS-PACDC	DX_LEGACY	ACTIVE	N	1900-01-01	2016-10-14	2015-07-10
Diagnosis: ICD10 Code, F00-F99 Define	F01-F99 Mental, Behavioral and Neurodevelopmental disorders	1258179	Chronic factitious illness with physical symptoms in adult	ICD10CM	1	F68.12		Factitious disorder with predominantly physical signs an	IMO	GHS-PACDC	IMO_2017_R	ACTIVE	N	1900-01-01	9999-12-31	2016-09-30
Diagnosis: ICD10 Code, F00-F99 Define	F01-F99 Mental, Behavioral and Neurodevelopmental disorders	1258145	Atypical factitious disorder with physical symptoms in adult	ICD10CM	1	F68.12		Factitious disorder with predominantly physical signs an	IMO	GHS-PACDC	IMO_2017_R	ACTIVE	N	1900-01-01	9999-12-31	2016-09-30
Diagnosis: ICD10 Code, F00-F99 Define	F01-F99 Mental, Behavioral and Neurodevelopmental disorders	1258180	Personality change in adult	ICD10CM	1	F68.8		Other specified disorders of adult personality and behav	IMO	GHS-PACDC	IMO_2017_R	ACTIVE	N	1900-01-01	9999-12-31	2016-09-30
Diagnosis: ICD10 Code, F00-F99 Define	F01-F99 Mental, Behavioral and Neurodevelopmental disorders	1309039	Psychiatric complaint	ICD10CM	1	F69		Unspecified disorder of adult personality and behavior	IMO	GHS-PACDC	IMO_2017_R	ACTIVE	N	1900-01-01	9999-12-31	2017-04-14
Diagnosis: ICD10 Code, F00-F99 Define	F01-F99 Mental, Behavioral and Neurodevelopmental disorders	111505	Mild mental retardation (I.Q. 50-70)	ICD10CM	1	F70		Mild intellectual disabilities	IMO	GHS-PACDC	DX_LEGACY	ACTIVE	N	1900-01-01	9999-12-31	2015-07-10
Diagnosis: ICD10 Code, F00-F99 Define	F01-F99 Mental, Behavioral and Neurodevelopmental disorders	201731	Moderate intellectual disability	ICD10CM	1	F71		Moderate intellectual disabilities	IMO	GHS-PACDC	DX_LEGACY	ACTIVE	N	1900-01-01	9999-12-31	2015-07-10
Diagnosis: ICD10 Code, F00-F99 Define	F01-F99 Mental, Behavioral and Neurodevelopmental disorders	102135	Severe mental retardation, IQ in range 20-34	ICD10CM	1	F72		Severe intellectual disabilities	IMO	GHS-PACDC	DX_LEGACY	ACTIVE	N	1900-01-01	9999-12-31	2015-07-10
Diagnosis: ICD10 Code, F00-F99 Define	F01-F99 Mental, Behavioral and Neurodevelopmental disorders	1264289	Profound intellectual disability in adult	ICD10CM	1	F73		Profound intellectual disabilities	IMO	GHS-PACDC	IMO_2017_R	ACTIVE	N	1900-01-01	9999-12-31	2016-09-30
Diagnosis: ICD10 Code, F00-F99 Define	F01-F99 Mental, Behavioral and Neurodevelopmental disorders	527868	Autosomal dominant mental retardation type 9	ICD10CM	1	F78		Other intellectual disabilities	IMO	GHS-PACDC	IMO_2015_R	ACTIVE	N	1900-01-01	9999-12-31	2015-07-10
Diagnosis: ICD10 Code, F00-F99 Define	F01-F99 Mental, Behavioral and Neurodevelopmental disorders	528964	X-linked intellectual disability associated with mutation in SYP gene	ICD10CM	1	F78		Other intellectual disabilities	IMO	GHS-PACDC	IMO_2015_R	ACTIVE	N	1900-01-01	9999-12-31	2015-07-10
Diagnosis: ICD10 Code, F00-F99 Define	F01-F99 Mental, Behavioral and Neurodevelopmental disorders	527860	Autosomal dominant mental retardation type 27	ICD10CM	1	F78		Other intellectual disabilities	IMO	GHS-PACDC	IMO_2015_R	ACTIVE	N	1900-01-01	9999-12-31	2015-07-10
Diagnosis: ICD10 Code, F00-F99 Define	F01-F99 Mental, Behavioral and Neurodevelopmental disorders	528954	X-linked mental retardation type 91	ICD10CM	1	F78		Other intellectual disabilities	IMO	GHS-PACDC	IMO_2015_R	ACTIVE	N	1900-01-01	9999-12-31	2015-07-10
Diagnosis: ICD10 Code, F00-F99 Define	F01-F99 Mental, Behavioral and Neurodevelopmental disorders	521722	Autosomal dominant intellectual disability type 9	ICD10CM	1	F78		Other intellectual disabilities	IMO	GHS-PACDC	IMO_2015_R	ACTIVE	N	1900-01-01	9999-12-31	2015-07-10
Diagnosis: ICD10 Code, F00-F99 Define	F01-F99 Mental, Behavioral and Neurodevelopmental disorders	528931	X-linked intellectual disability associated with mutation in TSPAN7 gene	ICD10CM	1	F78		Other intellectual disabilities	IMO	GHS-PACDC	IMO_2015_R	ACTIVE	N	1900-01-01	9999-12-31	2015-07-10
Diagnosis: ICD10 Code, F00-F99 Define	F01-F99 Mental, Behavioral and Neurodevelopmental disorders	1194736	Other intellectual disabilities	ICD10CM	1	F78		Other intellectual disabilities	IMO	GHS-PACDC	IMO_2016_R	DELETED	Y	1900-01-01	9999-12-31	2016-06-02
Diagnosis: ICD10 Code, F00-F99 Define	F01-F99 Mental, Behavioral and Neurodevelopmental disorders	421453	X-linked mental retardation	ICD10CM	1	F78		Other intellectual disabilities	IMO	GHS-PACDC	DX_LEGACY	ACTIVE	N	1900-01-01	9999-12-31	2015-07-10
Diagnosis: ICD10 Code, F00-F99 Define	F01-F99 Mental, Behavioral and Neurodevelopmental disorders	27509	Hyperphosphatasemia with mental retardation	ICD10CM	2	F79	E83.39,F79	Unspecified intellectual disabilities	IMO	GHS-PACDC	DX_LEGACY	ACTIVE	N	1900-01-01	9999-12-31	2015-07-10
Diagnosis: ICD10 Code, F00-F99 Define	F01-F99 Mental, Behavioral and Neurodevelopmental disorders	514772	Alopecia-contractures-dwarfism mental retardation syndrome	ICD10CM	3	F79	L65.9,E34.3,F79	Unspecified intellectual disabilities	IMO	GHS-PACDC	IMO_2015_R	ACTIVE	N	1900-01-01	2017-10-14	2015-07-10
Diagnosis: ICD10 Code, F00-F99 Define	F01-F99 Mental, Behavioral and Neurodevelopmental disorders	1257925	Disorder of intellectual development	ICD10CM	1	F79		Unspecified intellectual disabilities	IMO	GHS-PACDC	IMO_2017_R	ACTIVE	N	1900-01-01	9999-12-31	2016-09-30
Diagnosis: ICD10 Code, F00-F99 Define	F01-F99 Mental, Behavioral and Neurodevelopmental disorders	527645	Alopecia, epilepsy, pyorrhea, and intellectual disability syndrome (HCC)	ICD10CM	3	F79	Q87.89,Q84.0,F79,K05.30,G40.40	Unspecified intellectual disabilities	IMO	GHS-PACDC	IMO_2015_R	ACTIVE	N	2017-09-17	9999-12-31	2017-10-30
Diagnosis: ICD10 Code, F00-F99 Define	F01-F99 Mental, Behavioral and Neurodevelopmental disorders	1348428	X-linked intellectual disability syndrome with hypogammaglobulinemia and progressive neurolc	ICD10CM	3	F79	Q87.89,D80.0,F79,R29.818	Unspecified intellectual disabilities	IMO	GHS-PACDC	IMO_2018_R	ACTIVE	N	1900-01-01	9999-12-31	2017-10-30
Diagnosis: ICD10 Code, F00-F99 Define	F01-F99 Mental, Behavioral and Neurodevelopmental disorders	533233	Mental retardation, optic atrophy, deafness, and seizures syndrome (HCC)	ICD10CM	2	F79	Q87.89,F79,H47.20,R56.9,H90.5	Unspecified intellectual disabilities	IMO	GHS-PACDC	IMO_2015_R	ACTIVE	N	2017-09-17	9999-12-31	2017-10-30
Diagnosis: ICD10 Code, F00-F99 Define	F01-F99 Mental, Behavioral and Neurodevelopmental disorders	1112376	Autosomal dominant mental retardation type 29	ICD10CM	1	F79		Unspecified intellectual disabilities	IMO	GHS-PACDC	IMO_2016_R	ACTIVE	N	1900-01-01	9999-12-31	2016-06-02
Diagnosis: ICD10 Code, F00-F99 Define	F01-F99 Mental, Behavioral and Neurodevelopmental disorders	159902	Mental impairment	ICD10CM	1	F79		Unspecified intellectual disabilities	IMO	GHS-PACDC	DX_LEGACY	ACTIVE	N	1900-01-01	9999-12-31	2015-07-10
Diagnosis: ICD10 Code, F00-F99 Define	F01-F99 Mental, Behavioral and Neurodevelopmental disorders	1350265	X-linked intellectual disability and cerebellar hypoplasia syndrome (HCC)	ICD10CM	2	F79	Q87.89,F79,Q04.3	Unspecified intellectual disabilities	IMO	GHS-PACDC	IMO_2018_R	ACTIVE	N	1900-01-01	9999-12-31	2017-10-30
Diagnosis: ICD10 Code, F00-F99 Define	F01-F99 Mental, Behavioral and Neurodevelopmental disorders	528563	Alopecia intellectual disability syndrome type 3	ICD10CM	2	F79	L65.9,F79	Unspecified intellectual disabilities	IMO	GHS-PACDC	IMO_2015_R	ACTIVE	N	1900-01-01	2017-10-14	2015-07-10
Diagnosis: ICD10 Code, F00-F99 Define	F01-F99 Mental, Behavioral and Neurodevelopmental disorders	521636	Nonprogressive cerebellar ataxia with mental retardation (HCC)	ICD10CM	3	F79	Q87.89,G11.0,F79	Unspecified intellectual disabilities	IMO	GHS-PACDC	IMO_2015_R	ACTIVE	N	1900-01-01	9999-12-31	2015-07-10
Diagnosis: ICD10 Code, F00-F99 Define	F01-F99 Mental, Behavioral and Neurodevelopmental disorders	1354452	Microcephaly with seizures, intellectual disability, and cardiac disorder (HCC)	ICD10CM	4	F79	Q02,R56.9,I51.9,F79	Unspecified intellectual disabilities	IMO	GHS-PACDC	IMO_2018_R	ACTIVE	N	1900-01-01	9999-12-31	2017-10-30
Diagnosis: ICD10 Code, F00-F99 Define	F01-F99 Mental, Behavioral and Neurodevelopmental disorders	1348385	Cerebellar ataxia, intellectual disability, and dysequilibrium syndrome associated with mutation	ICD10CM	4	F79	Q87.89,F79,G11.9,R42,F79	Unspecified intellectual disabilities	IMO	GHS-PACDC	IMO_2018_R	ACTIVE	N	1900-01-01	9999-12-31	2017-10-30
Diagnosis: ICD10 Code, F00-F99 Define	F01-F99 Mental, Behavioral and Neurodevelopmental disorders	527553	Hyperphosphatasia with intellectual disability	ICD10CM	2	F79	E83.39,F79	Unspecified intellectual disabilities	IMO	GHS-PACDC	IMO_2015_R	ACTIVE	N	1900-01-01	9999-12-31	2015-07-10
Diagnosis: ICD10 Code, F00-F99 Define	F01-F99 Mental, Behavioral and Neurodevelopmental disorders	1351241	Aural atresia, multiple congenital anomalies, and intellectual disability syndrome	ICD10CM	3	F79	Q87.89,Q16.1,F79	Unspecified intellectual disabilities	IMO	GHS-PACDC	IMO_2018_R	ACTIVE	N	1900-01-01	9999-12-31	2017-10-30
Diagnosis: ICD10 Code, F00-F99 Define	F01-F99 Mental, Behavioral and Neurodevelopmental disorders	1354128	Finnish type progressive epilepsy with intellectual disability (HCC)	ICD10CM	2	F79	E75.4,F79,G40.909	Unspecified intellectual disabilities	IMO	GHS-PACDC	IMO_2018_R	ACTIVE	N	1900-01-01	9999-12-31	2017-10-30
Diagnosis: ICD10 Code, F00-F99 Define	F01-F99 Mental, Behavioral and Neurodevelopmental disorders	1351092	X-linked intellectual disability syndrome, Armfield type	ICD10CM	2	F79	Q87.89,F79	Unspecified intellectual disabilities	IMO	GHS-PACDC	IMO_2018_R	ACTIVE	N	1900-01-01	9999-12-31	2017-10-30
Diagnosis: ICD10 Code, F00-F99 Define	F01-F99 Mental, Behavioral and Neurodevelopmental disorders	514710	Alopecia-mental retardation syndrome 2	ICD10CM	3	F79	Q87.89,Q84.0,F79	Unspecified intellectual disabilities	IMO	GHS-PACDC	IMO_2015_R	ACTIVE	N	2017-09-17	9999-12-31	2017-10-30
Diagnosis: ICD10 Code, F00-F99 Define	F01-F99 Mental, Behavioral and Neurodevelopmental disorders	533263	Intellectual disability with anterior maxillary protrusion and strabismus	ICD10CM	2	F79	Q89.8,F79,H50.9,M26.19	Unspecified intellectual disabilities	IMO	GHS-PACDC	IMO_2015_R	ACTIVE	N	1900-01-01	9999-12-31	2015-07-10
Diagnosis: ICD10 Code, F00-F99 Define	F01-F99 Mental, Behavioral and Neurodevelopmental disorders	526368	Neuhauser syndrome	ICD10CM	2	F79	Q87.89,F79,Q15.8	Unspecified intellectual disabilities	IMO	GHS-PACDC	IMO_2015_R	ACTIVE	N	2016-04-10	9999-12-31	2015-07-10
Diagnosis: ICD10 Code, F00-F99 Define	F01-F99 Mental, Behavioral and Neurodevelopmental disorders	526368	Neuhauser syndrome	ICD10CM	1	F79	F79,Q15.8	Unspecified intellectual disabilities	IMO	GHS-PACDC	IMO_2015_R	ACTIVE	N	1900-01-01	2016-05-07	2015-07-10
Diagnosis: ICD10 Code, F00-F99 Define	F01-F99 Mental, Behavioral and Neurodevelopmental disorders	1352718	Aortic arch anomaly with peculiar facies and intellectual disability	ICD10CM	4	F79	Q87.89,Q25.40,Q18.9,F79	Unspecified intellectual disabilities	IMO	GHS-PACDC	IMO_2018_R	ACTIVE	N	1900-01-01	9999-12-31	2017-10-30
Diagnosis: ICD10 Code, F00-F99 Define	F01-F99 Mental, Behavioral and Neurodevelopmental disorders	260697	Deafness, onychodystrophy, osteodystrophy,and intellectual disability	ICD10CM	2	F79	Q87.89,F79,H90.5,Q78.9,Q84.6	Unspecified intellectual disabilities	IMO	GHS-PACDC	DX_LEGACY	ACTIVE	N	2017-09-17	9999-12-31	2017-10-30
Diagnosis: ICD10 Code, F00-F99 Define	F01-F99 Mental, Behavioral and Neurodevelopmental disorders	1351130	Skeletal dysplasia and intellectual disability syndrome	ICD10CM	2	F79	Q87.89,F79	Unspecified intellectual disabilities	IMO	GHS-PACDC	IMO_2018_R	ACTIVE	N	1900-01-01	9999-12-31	2017-10-30
Diagnosis: ICD10 Code, F00-F99 Define	F01-F99 Mental, Behavioral and Neurodevelopmental disorders	1354438	Metaphyseal dysostosis with intellectual disability and conductive deafness	ICD10CM	2	F79	Q78.8,F79,H90.2	Unspecified intellectual disabilities	IMO	GHS-PACDC	IMO_2018_R	ACTIVE	N	1900-01-01	9999-12-31	2017-10-30
Diagnosis: ICD10 Code, F00-F99 Define	F01-F99 Mental, Behavioral and Neurodevelopmental disorders	527587	X-linked intellectual disability syndrome type 12	ICD10CM	1	F79	F79,Q99.8	Unspecified intellectual disabilities	IMO	GHS-PACDC	IMO_2015_R	ACTIVE	N	1900-01-01	9999-12-31	2015-07-10
Diagnosis: ICD10 Code, F00-F99 Define	F01-F99 Mental, Behavioral and Neurodevelopmental disorders	1349870	Nishimura type spondyloepiphyseal dysplasia	ICD10CM	5	F79	Q87.89,Q77.7,Q12.0,Q35.9,F79	Unspecified intellectual disabilities	IMO	GHS-PACDC	IMO_2018_R	ACTIVE	N	1900-01-01	9999-12-31	2017-10-30
Diagnosis: ICD10 Code, F00-F99 Define	F01-F99 Mental, Behavioral and Neurodevelopmental disorders	527853	Autosomal dominant mental retardation type 21	ICD10CM	1	F79	F79,Q99.8	Unspecified intellectual disabilities	IMO	GHS-PACDC	IMO_2015_R	ACTIVE	N	1900-01-01	9999-12-31	2015-07-10
Diagnosis: ICD10 Code, F00-F99 Define	F01-F99 Mental, Behavioral and Neurodevelopmental disorders	1355234	Intellectual disability with macrocephaly, coarse facies, and hypotonia	ICD10CM	1	F79	F79,Q75.3,Q18.8,R29.898	Unspecified intellectual disabilities	IMO	GHS-PACDC	IMO_2018_R	ACTIVE	N	1900-01-01	9999-12-31	2017-10-30

Diagnosis: ICD10 Code, F00-F99 Define	F01-F99 Mental, Behavioral and Neurodevelopmental disorders	1264283	Intellectual disability associated with Fragile X syndrome	ICD10CM	1	F79	F79,Q99.2	Unspecified intellectual disabilities	IMO	GHS-PACDC	IMO_2017_R	ACTIVE	N	1900-01-01	9999-12-31	2016-09-30
Diagnosis: ICD10 Code, F00-F99 Define	F01-F99 Mental, Behavioral and Neurodevelopmental disorders	527854	Autosomal dominant intellectual disability type 20	ICD10CM	1	F79	F79,Q99.8	Unspecified intellectual disabilities	IMO	GHS-PACDC	IMO_2015_R	ACTIVE	N	1900-01-01	9999-12-31	2015-07-10
Diagnosis: ICD10 Code, F00-F99 Define	F01-F99 Mental, Behavioral and Neurodevelopmental disorders	505131	Mild expressive language delay	ICD10CM	1	F80.1		Expressive language disorder	IMO	GHS-PACDC	IMO_2015_R	ACTIVE	N	1900-01-01	9999-12-31	2015-07-10
Diagnosis: ICD10 Code, F00-F99 Define	F01-F99 Mental, Behavioral and Neurodevelopmental disorders	1258266	Acquired impairment of language comprehension	ICD10CM	1	F80.2		Mixed receptive-expressive language disorder	IMO	GHS-PACDC	IMO_2017_R	ACTIVE	N	1900-01-01	9999-12-31	2016-09-30
Diagnosis: ICD10 Code, F00-F99 Define	F01-F99 Mental, Behavioral and Neurodevelopmental disorders	408676	Restricted receptive language development	ICD10CM	1	F80.2		Mixed receptive-expressive language disorder	IMO	GHS-PACDC	DX_LEGACY	ACTIVE	N	1900-01-01	9999-12-31	2015-07-10
Diagnosis: ICD10 Code, F00-F99 Define	F01-F99 Mental, Behavioral and Neurodevelopmental disorders	90456	Other speech and language deficits	ICD10CM	1	F80.9	F80.9,R47.9	Developmental disorder of speech and language, unspec	IMO	GHS-PACDC	DX_LEGACY	DELETED	Y	1900-01-01	9999-12-31	2015-07-10
Diagnosis: ICD10 Code, F00-F99 Define	F01-F99 Mental, Behavioral and Neurodevelopmental disorders	223957	Speech or language delay	ICD10CM	1	F80.9		Developmental disorder of speech and language, unspec	IMO	GHS-PACDC	DX_LEGACY	ACTIVE	N	1900-01-01	9999-12-31	2015-07-10
Diagnosis: ICD10 Code, F00-F99 Define	F01-F99 Mental, Behavioral and Neurodevelopmental disorders	259182	Disorder of speech or language development	ICD10CM	1	F80.9		Developmental disorder of speech and language, unspec	IMO	GHS-PACDC	DX_LEGACY	ACTIVE	N	1900-01-01	9999-12-31	2015-07-10
Diagnosis: ICD10 Code, F00-F99 Define	F01-F99 Mental, Behavioral and Neurodevelopmental disorders	30032	Developmental speech disorder	ICD10CM	1	F80.9		Developmental disorder of speech and language, unspec	IMO	GHS-PACDC	DX_LEGACY	ACTIVE	N	1900-01-01	9999-12-31	2015-07-10
Diagnosis: ICD10 Code, F00-F99 Define	F01-F99 Mental, Behavioral and Neurodevelopmental disorders	163456	Finding of cerebral dominance	ICD10CM	1	F81.0		Specific reading disorder	IMO	GHS-PACDC	DX_LEGACY	ACTIVE	N	1900-01-01	9999-12-31	2015-07-10
Diagnosis: ICD10 Code, F00-F99 Define	F01-F99 Mental, Behavioral and Neurodevelopmental disorders	102278	Specific reading difficulty	ICD10CM	1	F81.0		Specific reading disorder	IMO	GHS-PACDC	DX_LEGACY	ACTIVE	N	1900-01-01	9999-12-31	2015-07-10
Diagnosis: ICD10 Code, F00-F99 Define	F01-F99 Mental, Behavioral and Neurodevelopmental disorders	488256	Specific learning disorder, with impairment in mathematics, moderate	ICD10CM	1	F81.2		Mathematics disorder	IMO	GHS-PACDC	IMO_2015_R	ACTIVE	N	1900-01-01	9999-12-31	2015-07-10
Diagnosis: ICD10 Code, F00-F99 Define	F01-F99 Mental, Behavioral and Neurodevelopmental disorders	48138	Gerstmanns syndrome	ICD10CM	1	F81.2		Mathematics disorder	IMO	GHS-PACDC	DX_LEGACY	ACTIVE	N	1900-01-01	9999-12-31	2015-07-10
Diagnosis: ICD10 Code, F00-F99 Define	F01-F99 Mental, Behavioral and Neurodevelopmental disorders	608503	Other developmental disorders of scholastic skills	ICD10CM	1	F81.8		Other developmental disorders of scholastic skills	IMO	GHS-PACDC	IMO_2015_R	ACTIVE	Y	1900-01-01	9999-12-31	2015-07-10
Diagnosis: ICD10 Code, F00-F99 Define	F01-F99 Mental, Behavioral and Neurodevelopmental disorders	251438	Writing learning disorder	ICD10CM	1	F81.81		Disorder of written expression	IMO	GHS-PACDC	DX_LEGACY	ACTIVE	N	1900-01-01	9999-12-31	2015-07-10
Diagnosis: ICD10 Code, F00-F99 Define	F01-F99 Mental, Behavioral and Neurodevelopmental disorders	252985	Non-verbal learning disorder	ICD10CM	1	F81.89		Other developmental disorders of scholastic skills	IMO	GHS-PACDC	DX_LEGACY	ACTIVE	N	1900-01-01	9999-12-31	2015-07-10
Diagnosis: ICD10 Code, F00-F99 Define	F01-F99 Mental, Behavioral and Neurodevelopmental disorders	156777	General learning disability	ICD10CM	1	F81.9		Developmental disorder of scholastic skills, unspecified	IMO	GHS-PACDC	DX_LEGACY	ACTIVE	N	1900-01-01	9999-12-31	2015-07-10
Diagnosis: ICD10 Code, F00-F99 Define	F01-F99 Mental, Behavioral and Neurodevelopmental disorders	156778	Developmental disorder of scholastic skill	ICD10CM	1	F81.9		Developmental disorder of scholastic skills, unspecified	IMO	GHS-PACDC	DX_LEGACY	ACTIVE	N	1900-01-01	9999-12-31	2015-07-10
Diagnosis: ICD10 Code, F00-F99 Define	F01-F99 Mental, Behavioral and Neurodevelopmental disorders	325269	Immature motor skills	ICD10CM	1	F82		Specific developmental disorder of motor function	IMO	GHS-PACDC	DX_LEGACY	ACTIVE	N	1900-01-01	9999-12-31	2015-07-10
Diagnosis: ICD10 Code, F00-F99 Define	F01-F99 Mental, Behavioral and Neurodevelopmental disorders	514650	Delay of motor development	ICD10CM	1	F82		Specific developmental disorder of motor function	IMO	GHS-PACDC	IMO_2015_R	ACTIVE	N	1900-01-01	9999-12-31	2015-07-10
Diagnosis: ICD10 Code, F00-F99 Define	F01-F99 Mental, Behavioral and Neurodevelopmental disorders	73124	Specific motor development disorder	ICD10CM	1	F82		Specific developmental disorder of motor function	IMO	GHS-PACDC	DX_LEGACY	ACTIVE	N	1900-01-01	9999-12-31	2015-07-10
Diagnosis: ICD10 Code, F00-F99 Define	F01-F99 Mental, Behavioral and Neurodevelopmental disorders	526393	MECP2-related autism	ICD10CM	1	F84.0		Autistic disorder	IMO	GHS-PACDC	IMO_2015_R	ACTIVE	N	1900-01-01	9999-12-31	2015-07-10
Diagnosis: ICD10 Code, F00-F99 Define	F01-F99 Mental, Behavioral and Neurodevelopmental disorders	1199340	Autism associated with mutation in SHANK2 gene	ICD10CM	1	F84.0		Autistic disorder	IMO	GHS-PACDC	IMO_2016_R	ACTIVE	N	1900-01-01	9999-12-31	2016-06-02
Diagnosis: ICD10 Code, F00-F99 Define	F01-F99 Mental, Behavioral and Neurodevelopmental disorders	1127410	Autism spectrum disorder, requiring substantial support, without accompanying language impa	ICD10CM	1	F84.0		Autistic disorder	IMO	GHS-PACDC	IMO_2016_R	ACTIVE	N	1900-01-01	9999-12-31	2016-06-02
Diagnosis: ICD10 Code, F00-F99 Define	F01-F99 Mental, Behavioral and Neurodevelopmental disorders	1199272	KCNQ3-associated autism	ICD10CM	1	F84.0		Autistic disorder	IMO	GHS-PACDC	IMO_2016_R	ACTIVE	N	1900-01-01	9999-12-31	2016-06-02
Diagnosis: ICD10 Code, F00-F99 Define	F01-F99 Mental, Behavioral and Neurodevelopmental disorders	488422	Autism spectrum disorder, requiring substantial support, with accompanying intellectual impair	ICD10CM	1	F84.0		Autistic disorder	IMO	GHS-PACDC	IMO_2015_R	ACTIVE	N	1900-01-01	9999-12-31	2015-07-10
Diagnosis: ICD10 Code, F00-F99 Define	F01-F99 Mental, Behavioral and Neurodevelopmental disorders	3019	Other specified pervasive developmental disorders, residual state	ICD10CM	1	F84.8		Other pervasive developmental disorders	IMO	GHS-PACDC	DX_LEGACY	ACTIVE	N	2016-04-10	9999-12-31	2015-07-10
Diagnosis: ICD10 Code, F00-F99 Define	F01-F99 Mental, Behavioral and Neurodevelopmental disorders	526699	Atypical pervasive developmental disorder	ICD10CM	1	F84.8		Other pervasive developmental disorders	IMO	GHS-PACDC	IMO_2015_R	ACTIVE	N	1900-01-01	9999-12-31	2015-07-10
Diagnosis: ICD10 Code, F00-F99 Define	F01-F99 Mental, Behavioral and Neurodevelopmental disorders	55977	Schizophrenia, childhood	ICD10CM	1	F84.9		Pervasive developmental disorder, unspecified	IMO	GHS-PACDC	DX_LEGACY	ACTIVE	N	1900-01-01	9999-12-31	2015-07-10
Diagnosis: ICD10 Code, F00-F99 Define	F01-F99 Mental, Behavioral and Neurodevelopmental disorders	3022	Pervasive developmental disorder, residual	ICD10CM	1	F84.9		Pervasive developmental disorder, unspecified	IMO	GHS-PACDC	DX_LEGACY	ACTIVE	N	1900-01-01	9999-12-31	2015-07-10
Diagnosis: ICD10 Code, F00-F99 Define	F01-F99 Mental, Behavioral and Neurodevelopmental disorders	3021	Pervasive developmental disorder, active	ICD10CM	1	F84.9		Pervasive developmental disorder, unspecified	IMO	GHS-PACDC	DX_LEGACY	ACTIVE	N	1900-01-01	9999-12-31	2015-07-10
Diagnosis: ICD10 Code, F00-F99 Define	F01-F99 Mental, Behavioral and Neurodevelopmental disorders	1125598	Hypersensitive sensory processing disorder, type A	ICD10CM	1	F88		Other disorders of psychological development	IMO	GHS-PACDC	IMO_2016_R	ACTIVE	N	1900-01-01	9999-12-31	2016-06-02
Diagnosis: ICD10 Code, F00-F99 Define	F01-F99 Mental, Behavioral and Neurodevelopmental disorders	1125067	Hypersensitive sensory processing disorder, fearful or cautious	ICD10CM	1	F88		Other disorders of psychological development	IMO	GHS-PACDC	IMO_2016_R	ACTIVE	N	1900-01-01	9999-12-31	2016-06-02
Diagnosis: ICD10 Code, F00-F99 Define	F01-F99 Mental, Behavioral and Neurodevelopmental disorders	1125042	Hypersensitive sensory processing disorder, generalized, fearful or cautious	ICD10CM	1	F88		Other disorders of psychological development	IMO	GHS-PACDC	IMO_2016_R	ACTIVE	N	1900-01-01	9999-12-31	2016-06-02
Diagnosis: ICD10 Code, F00-F99 Define	F01-F99 Mental, Behavioral and Neurodevelopmental disorders	1351230	Anomaly of ossification and psychomotor developmental delay syndrome	ICD10CM	2	F88	Q87.89,F88	Other disorders of psychological development	IMO	GHS-PACDC	IMO_2018_R	ACTIVE	N	1900-01-01	9999-12-31	2017-10-30
Diagnosis: ICD10 Code, F00-F99 Define	F01-F99 Mental, Behavioral and Neurodevelopmental disorders	1125034	Hypersensitive sensory processing disorder, negative or defiant	ICD10CM	1	F88		Other disorders of psychological development	IMO	GHS-PACDC	IMO_2016_R	ACTIVE	N	1900-01-01	9999-12-31	2016-06-02
Diagnosis: ICD10 Code, F00-F99 Define	F01-F99 Mental, Behavioral and Neurodevelopmental disorders	429207	Neurodevelopmental disorder	ICD10CM	1	F89		Unspecified disorder of psychological development	IMO	GHS-PACDC	IMO_2015_R	ACTIVE	N	1900-01-01	9999-12-31	2015-07-10
Diagnosis: ICD10 Code, F00-F99 Define	F01-F99 Mental, Behavioral and Neurodevelopmental disorders	209834	Developmental deviation/disorder	ICD10CM	1	F89		Unspecified disorder of psychological development	IMO	GHS-PACDC	DX_LEGACY	ACTIVE	N	1900-01-01	9999-12-31	2015-07-10
Diagnosis: ICD10 Code, F00-F99 Define	F01-F99 Mental, Behavioral and Neurodevelopmental disorders	1125008	Multisystem developmental disorder with communication disorder in patient younger than 2 ye	ICD10CM	1	F89	F89,F80.9	Unspecified disorder of psychological development	IMO	GHS-PACDC	IMO_2016_R	ACTIVE	N	1900-01-01	9999-12-31	2016-06-02
Diagnosis: ICD10 Code, F00-F99 Define	F01-F99 Mental, Behavioral and Neurodevelopmental disorders	73117	Simple disturbance of attention with overactivity	ICD10CM	1	F90.0		Attention-deficit hyperactivity disorder, predominantly i	IMO	GHS-PACDC	DX_LEGACY	ACTIVE	N	1900-01-01	2017-04-27	2015-07-10
Diagnosis: ICD10 Code, F00-F99 Define	F01-F99 Mental, Behavioral and Neurodevelopmental disorders	421619	Attention deficit hyperactivity disorder, predominantly inattentive type	ICD10CM	1	F90.0		Attention-deficit hyperactivity disorder, predominantly i	IMO	GHS-PACDC	IMO_2015_R	ACTIVE	N	1900-01-01	9999-12-31	2015-07-10
Diagnosis: ICD10 Code, F00-F99 Define	F01-F99 Mental, Behavioral and Neurodevelopmental disorders	496452	Impulsive type attention deficit hyperactivity disorder (ADHD)	ICD10CM	1	F90.1		Attention-deficit hyperactivity disorder, predominantly h	IMO	GHS-PACDC	IMO_2015_R	ACTIVE	N	1900-01-01	2016-05-07	2015-07-10
Diagnosis: ICD10 Code, F00-F99 Define	F01-F99 Mental, Behavioral and Neurodevelopmental disorders	342619	ADHD (attention deficit hyperactivity disorder), predominantly hyperactive impulsive type	ICD10CM	1	F90.1		Attention-deficit hyperactivity disorder, predominantly h	IMO	GHS-PACDC	DX_LEGACY	ACTIVE	N	1900-01-01	9999-12-31	2015-07-10
Diagnosis: ICD10 Code, F00-F99 Define	F01-F99 Mental, Behavioral and Neurodevelopmental disorders	253513	ADHD, impulsive type	ICD10CM	1	F90.1		Attention-deficit hyperactivity disorder, predominantly h	IMO	GHS-PACDC	DX_LEGACY	ACTIVE	N	1900-01-01	2016-05-07	2015-07-10
Diagnosis: ICD10 Code, F00-F99 Define	F01-F99 Mental, Behavioral and Neurodevelopmental disorders	496449	Attention deficit hyperactivity disorder (ADHD), predominantly hyperactive-impulsive or combir	ICD10CM	1	F90.2		Attention-deficit hyperactivity disorder, combined type	IMO	GHS-PACDC	IMO_2015_R	ACTIVE	N	1900-01-01	9999-12-31	2015-07-10
Diagnosis: ICD10 Code, F00-F99 Define	F01-F99 Mental, Behavioral and Neurodevelopmental disorders	147037	MBD (minimal brain dysfunction)	ICD10CM	1	F90.9		Attention-deficit hyperactivity disorder, unspecified type	IMO	GHS-PACDC	DX_LEGACY	ACTIVE	N	1900-01-01	9999-12-31	2015-07-10
Diagnosis: ICD10 Code, F00-F99 Define	F01-F99 Mental, Behavioral and Neurodevelopmental disorders	49842	Hyperkinesis	ICD10CM	1	F90.9		Attention-deficit hyperactivity disorder, unspecified type	IMO	GHS-PACDC	DX_LEGACY	ACTIVE	N	1900-01-01	9999-12-31	2015-07-10
Diagnosis: ICD10 Code, F00-F99 Define	F01-F99 Mental, Behavioral and Neurodevelopmental disorders	61386	Attention deficit disorder of childhood with hyperactivity	ICD10CM	1	F90.9		Attention-deficit hyperactivity disorder, unspecified type	IMO	GHS-PACDC	DX_LEGACY	ACTIVE	N	2016-09-17	9999-12-31	2016-09-30
Diagnosis: ICD10 Code, F00-F99 Define	F01-F99 Mental, Behavioral and Neurodevelopmental disorders	237021	Attention deficit disorder (ADD), child, with hyperactivity	ICD10CM	1	F90.9		Attention-deficit hyperactivity disorder, unspecified type	IMO	GHS-PACDC	DX_LEGACY	ACTIVE	N	2016-09-17	9999-12-31	2016-09-30
Diagnosis: ICD10 Code, F00-F99 Define	F01-F99 Mental, Behavioral and Neurodevelopmental disorders	147038	MBD syndrome	ICD10CM	1	F90.9		Attention-deficit hyperactivity disorder, unspecified type	IMO	GHS-PACDC	DX_LEGACY	ACTIVE	N	1900-01-01	9999-12-31	2015-07-10
Diagnosis: ICD10 Code, F00-F99 Define	F01-F99 Mental, Behavioral and Neurodevelopmental disorders	39299	Undersocialized conduct disorder, aggressive type, moderate degree	ICD10CM	1	F91.1		Conduct disorder, childhood-onset type	IMO	GHS-PACDC	DX_LEGACY	ACTIVE	N	1900-01-01	9999-12-31	2015-07-10
Diagnosis: ICD10 Code, F00-F99 Define	F01-F99 Mental, Behavioral and Neurodevelopmental disorders	488383	Conduct disorder, childhood-onset type, mild	ICD10CM	1	F91.1		Conduct disorder, childhood-onset type	IMO	GHS-PACDC	IMO_2015_R	ACTIVE	N	1900-01-01	9999-12-31	2015-07-10
Diagnosis: ICD10 Code, F00-F99 Define	F01-F99 Mental, Behavioral and Neurodevelopmental disorders	212067	Conduct disorder, unaggressive type, mild	ICD10CM	1	F91.1		Conduct disorder, childhood-onset type	IMO	GHS-PACDC	DX_LEGACY	ACTIVE	N	1900-01-01	9999-12-31	2015-07-10
Diagnosis: ICD10 Code, F00-F99 Define	F01-F99 Mental, Behavioral and Neurodevelopmental disorders	29973	Conduct disorder, solitary aggressive type	ICD10CM	1	F91.1		Conduct disorder, childhood-onset type	IMO	GHS-PACDC	DX_LEGACY	ACTIVE	N	1900-01-01	9999-12-31	2015-07-10
Diagnosis: ICD10 Code, F00-F99 Define	F01-F99 Mental, Behavioral and Neurodevelopmental disorders	488404	Moderate conduct disorder, adolescent-onset type	ICD10CM	1	F91.2		Conduct disorder, adolescent-onset type	IMO	GHS-PACDC	IMO_2015_R	ACTIVE	N	1900-01-01	9999-12-31	2015-07-10
Diagnosis: ICD10 Code, F00-F99 Define	F01-F99 Mental, Behavioral and Neurodevelopmental disorders	485255	Adolescent-onset type conduct disorder with deceitfulness or theft	ICD10CM	1	F91.2		Conduct disorder, adolescent-onset type	IMO	GHS-PACDC	IMO_2015_R	ACTIVE	N	1900-01-01	9999-12-31	2015-07-10
Diagnosis: ICD10 Code, F00-F99 Define	F01-F99 Mental, Behavioral and Neurodevelopmental disorders	485256	Adolescent-onset type conduct disorder with destruction of property	ICD10CM	1	F91.2		Conduct disorder, adolescent-onset type	IMO	GHS-PACDC	IMO_2015_R	ACTIVE	N	1900-01-01	9999-12-31	2015-07-10
Diagnosis: ICD10 Code, F00-F99 Define	F01-F99 Mental, Behavioral and Neurodevelopmental disorders	1101492	Other conduct disorders	ICD10CM	1	F91.8		Other conduct disorders	IMO	GHS-PACDC	IMO_2016_R	DELETED	Y	1900-01-01	9999-12-31	2016-06-02
Diagnosis: ICD10 Code, F00-F99 Define	F01-F99 Mental, Behavioral and Neurodevelopmental disorders	327545	Excessive self-criticism	ICD10CM	1	F91.8		Other conduct disorders	IMO	GHS-PACDC	DX_LEGACY	ACTIVE	N	1900-01-01	9999-12-31	2015-07-10
Diagnosis: ICD10 Code, F00-F99 Define	F01-F99 Mental, Behavioral and Neurodevelopmental disorders	304999	Disorganized behavior	ICD10CM	1	F91.8		Other conduct disorders	IMO	GHS-PACDC	DX_LEGACY	ACTIVE	N	1900-01-01	2016-05-07	2015-07-10
Diagnosis: ICD10 Code, F00-F99 Define	F01-F99 Mental, Behavioral and Neurodevelopmental disorders	66451	Temper tantrums	ICD10CM	1	F91.8		Other conduct disorders	IMO	GHS-PACDC	DX_LEGACY	ACTIVE	N	2016-04-10	9999-12-31	2015-07-10
Diagnosis: ICD10 Code, F00-F99 Define	F01-F99 Mental, Behavioral and Neurodevelopmental disorders	523777	Conduct disorder of unspecified onset with aggression to people and animals	ICD10CM	1	F91.8		Other conduct disorders	IMO	GHS-PACDC	IMO_2015_R	DELETED	Y	1900-01-01	9999-12-31	2015-07-10
Diagnosis: ICD10 Code, F00-F99 Define	F01-F99 Mental, Behavioral and Neurodevelopmental disorders	61378	Conduct disturbance	ICD10CM	1	F91.9		Conduct disorder, unspecified	IMO	GHS-PACDC	DX_LEGACY	ACTIVE	N	1900-01-01	9999-12-31	2015-07-10
Diagnosis: ICD10 Code, F00-F99 Define	F01-F99 Mental, Behavioral and Neurodevelopmental disorders	488364	Conduct disorder, with limited prosocial emotions, mild	ICD10CM	1	F91.9		Conduct disorder, unspecified	IMO	GHS-PACDC	IMO_2015_R	ACTIVE	N	1900-01-01	9999-12-31	2015-07-10
Diagnosis: ICD10 Code, F00-F99 Define	F01-F99 Mental, Behavioral and Neurodevelopmental disorders	488375	Conduct disorder, with limited prosocial emotions, severe	ICD10CM	1	F91.9		Conduct disorder, unspecified	IMO	GHS-PACDC	IMO_2015_R	ACTIVE	N	1900-01-01	9999-12-31	2015-07-10
Diagnosis: ICD10 Code, F00-F99 Define	F01-F99 Mental, Behavioral and Neurodevelopmental disorders	260686	Anxiety disorder of childhood or adolescence	ICD10CM	1	F93.8		Other childhood emotional disorders	IMO	GHS-PACDC	DX_LEGACY	ACTIVE	N	1900-01-01	9999-12-31	2015-07-10
Diagnosis: ICD10 Code, F00-F99 Define	F01-F99 Mental, Behavioral and Neurodevelopmental disorders	29984	Avoidant disorder of childhood or adolescence	ICD10CM	1	F93.8		Other childhood emotional disorders	IMO	GHS-PACDC	DX_LEGACY	ACTIVE	N	1900-01-01	9999-12-31	2015-07-10
Diagnosis: ICD10 Code, F00-F99 Define	F01-F99 Mental, Behavioral and Neurodevelopmental disorders	1112953	Behavioral and emotional disorder with onset in childhood	ICD10CM	2	F93.9	F91.9,F93.9	Childhood emotional disorder, unspecified	IMO	GHS-PACDC	IMO_2016_R	ACTIVE	N	1900-01-01	9999-12-31	2016-06-02
Diagnosis: ICD10 Code, F00-F99 Define	F01-F99 Mental, Behavioral and Neurodevelopmental disorders	210465	Adjustment reaction with elective mutism	ICD10CM	1	F94.0		Selective mutism	IMO	GHS-PACDC	DX_LEGACY	ACTIVE	N	1900-01-01	9999-12-31	2015-07-10
Diagnosis: ICD10 Code, F00-F99 Define	F01-F99 Mental, Behavioral and Neurodevelopmental disorders	3345	Reactive attachment disorder of infancy	ICD10CM	1	F94.1		Reactive attachment disorder of childhood	IMO	GHS-PACDC	DX_LEGACY	ACTIVE	N	1900-01-01	9999-12-31	2015-07-10
Diagnosis: ICD10 Code, F00-F99 Define	F01-F99 Mental, Behavioral and Neurodevelopmental disorders	29965	Reactive attachment disorder of early childhood	ICD10CM	1	F94.1		Reactive attachment disorder of childhood	IMO	GHS-PACDC	DX_LEGACY	ACTIVE	N	1900-01-01	9999-12-31	2015-07-10
Diagnosis: ICD10 Code, F00-F99 Define	F01-F99 Mental, Behavioral and Neurodevelopmental disorders	429140	Disinhibited social engagement disorder	ICD10CM	1	F94.2		Disinhibited attachment disorder of childhood	IMO	GHS-PACDC	IMO_2015_R	ACTIVE	N	1900-01-01	9999-12-31	2015-07-10
Diagnosis: ICD10 Code, F00-F99 Define	F01-F99 Mental, Behavioral and Neurodevelopmental disorders	169859	Childhood and adolescent sensitivity, shyness, and social withdrawal	ICD10CM	1	F94.8		Other childhood disorders of social functioning	IMO	GHS-PACDC	DX_LEGACY	ACTIVE	N	1900-01-01	2016-10-14	2015-07-10
Diagnosis: ICD10 Code, F00-F99 Define	F01-F99 Mental, Behavioral and Neurodevelopmental disorders	3242	Transient tic disorder	ICD10CM	1	F95.0		Transient tic disorder	IMO	GHS-PACDC	DX_LEGACY	ACTIVE	N	1900-01-01	9999-12-31	2015-07-10
Diagnosis: ICD10 Code, F00-F99 Define	F01-F99 Mental, Behavioral and Neurodevelopmental disorders	486524	Tic-related obsessive-compulsive disorder with good or fair insight	ICD10CM	1	F95.9	F95.9,F42.9	Tic disorder, unspecified	IMO	GHS-PACDC	IMO_2015_R	ACTIVE	N	2016-09-17	9999-12-31	2016-09-30
Diagnosis: ICD10 Code, F00-F99 Define	F01-F99 Mental, Behavioral and Neurodevelopmental disorders	408169	Habit tic affecting hair	ICD10CM	1	F95.9		Tic disorder, unspecified	IMO	GHS-PACDC	DX_LEGACY	ACTIVE	N	1900-01-01	9999-12-31	2015-07-10
Diagnosis: ICD10 Code, F00-F99 Define	F01-F99 Mental, Behavioral and Neurodevelopmental disorders	204557	Functional enuresis	ICD10CM	1	F98.0		Enuresis not due to a substance or known physiological	IMO	GHS-PACDC	DX_LEGACY	ACTIVE	N	1900-01-01	9999-12-31	2015-07-10
Diagnosis: ICD10 Code, F00-F99 Define	F01-F99 Mental, Behavioral and Neurodevelopmental disorders	1111118	Combined nocturnal and diurnal nonorganic enuresis	ICD10CM	1	F98.0		Enuresis not due to a substance or known physiological	IMO	GHS-PACDC	IMO_2016_R	ACTIVE	N	1900-01-01	9999-12-31	2016-06-02
Diagnosis: ICD10 Code, F00-F99 Define	F01-F99 Mental, Behavioral and Neurodevelopmental disorders	1111119	Nonorganic enuresis, combined nocturnal and diurnal	ICD10CM	1	F98.0		Enuresis not due to a substance or known physiological	IMO	GHS-PACDC	IMO_2016_R	ACTIVE	N	1900-01-01	9999-12-31	2016-06-02
Diagnosis: ICD10 Code, F00-F99 Define	F01-F99 Mental, Behavioral and Neurodevelopmental disorders	1299746	Non-organic continuous encopresis	ICD10CM	1	F98.1		Encopresis not due to a substance or known physiologic	IMO	GHS-PACDC	IMO_2017_R	ACTIVE	N	1900-01-01	9999-12-31	2017-04-14
Diagnosis: ICD10 Code, F00-F99 Define	F01-F99 Mental, Behavioral and Neurodevelopmental disorders	30017	Primary functional encopresis	ICD10CM	1	F98.1		Encopresis not due to a substance or known physiologic	IMO	GHS-PACDC	DX_LEGACY	ACTIVE	N	1900-01-01	9999-12-31	2015-07-10
Diagnosis: ICD10 Code, F00-F99 Define	F01-F99 Mental, Behavioral and Neurodevelopmental disorders	264572	Pica of infancy and childhood	ICD10CM	1	F98.3		Pica of infancy and childhood	IMO	GHS-PACDC	DX_LEGACY	ACTIVE	N	1900-01-01	9999-12-31	2015-07-10
Diagnosis: ICD10 Code, F00-F99 Define	F01-F99 Mental, Behavioral and Neurodevelopmental disorders	485399	Mild stereotypic movement disorder associated with known medical condition, without self-inju	ICD10CM	1	F98.4		Stereotyped movement disorders	IMO	GHS-PACDC	IMO_2015_R	ACTIVE	N	1900-01-01	9999-12-31	2015-07-10
Diagnosis: ICD10 Code, F00-F99 Define	F01-F99 Mental, Behavioral and Neurodevelopmental disorders	488023	Stereotypic movement disorder, with self-injurious behavior, associated with known medical or	ICD10CM	1	F98.4		Stereotyped movement disorders	IMO	GHS-PACDC	IMO_2015_R	ACTIVE	N	1900-01-01	9999-12-31	2015-07-10
Diagnosis: ICD10 Code, F00-F99 Define	F01-F99 Mental, Behavioral and Neurodevelopmental disorders	488003	Stereotypic movement disorder, without self-injurious behavior, associated with known medica	ICD10CM	1	F98.4		Stereotyped movement disorders	IMO	GHS-PACDC	IMO_2015_R	ACTIVE	N	1900-01-01	9999-12-31	2015-07-10
Diagnosis: ICD10 Code, F00-F99 Define	F01-F99 Mental, Behavioral and Neurodevelopmental disorders	486547	Mild stereotypic movement disorder associated with known neurodevelopmental disorder, with	ICD10CM	1	F98.4		Stereotyped movement disorders	IMO	GHS-PACDC	IMO_2015_R	ACTIVE	N	1900-01-01	9999-12-31	2015-07-10
Diagnosis: ICD10 Code, F00-F99 Define	F01-F99 Mental, Behavioral and Neurodevelopmental disorders	163083	Repetitive rocking movements	ICD10CM	1	F98.4		Stereotyped movement disorders	IMO	GHS-PACDC	DX_LEGACY	ACTIVE	N	1900-01-01	9999-12-31	2015-07-10
Diagnosis: ICD10 Code, F00-F99 Define	F01-F99 Mental, Behavioral and Neurodevelopmental disorders	493613	Sleep-related head banging	ICD10CM	2	F98.4	F51.8,F98.4	Stereotyped movement disorders	IMO	GHS-PACDC	IMO_2015_R	ACTIVE	N	1900-01-01	9999-12-31	2015-07-10
Diagnosis: ICD10 Code, F00-F99 Define	F01-F99 Mental, Behavioral and Neurodevelopmental disorders	1409313	Attention deficit disorder, unspecified hyperactivity presence	ICD10CM	1	F98.8		Other specified behavioral and emotional disorders with	IMO	GHS-PACDC	IMO_2018_R	ACTIVE	N	1900-01-01	9999-12-31	2017-10-30
Diagnosis: ICD10 Code, F00-F99 Define	F01-F99 Mental, Behavioral and Neurodevelopmental disorders	163044	Digit sucking	ICD10CM	1	F98.8		Other specified behavioral and emotional disorders with	IMO	GHS-PACDC	DX_LEGACY	DELETED	Y	1900-01-01	2016-05-07	2015-07-10
Diagnosis: ICD10 Code, F00-F99 Define	F01-F99 Mental, Behavioral and Neurodevelopmental disorders	115857	Undifferentiated attention-deficit disorder	ICD10CM	1	F98.8		Other specified behavioral and emotional disorders with	IMO	GHS-PACDC	DX_LEGACY	ACTIVE	N	2016-09-17	9999-12-31	2016-09-30
Diagnosis: ICD10 Code, F00-F99 Define	F01-F99 Mental, Behavioral and Neurodevelopmental disorders	1114450	Habitual sucking of finger	ICD10CM	1	F98.8		Other specified behavioral and emotional disorders with	IMO	GHS-PACDC	IMO_2016_R	ACTIVE	N	1900-01-01	9999-12-31	2016-06-02
Diagnosis: ICD10 Code, F00-F99 Define	F01-F99 Mental, Behavioral and Neurodevelopmental disorders	52898	Onychophagy	ICD10CM	1	F98.8		Other specified behavioral and emotional disorders with	IMO	GHS-PACDC	DX_LEGACY	ACTIVE	N	1900-01-01	9999-12-31	2015-07-10
Diagnosis: ICD10 Code, F00-F99 Define	F01-F99 Mental, Behavioral and Neurodevelopmental disorders	3349	Attention deficit disorder without hyperactivity	ICD10CM	1	F98.8		Other specified behavioral and emotional disorders with	IMO	GHS-PACDC	DX_LEGACY	ACTIVE	N	2017-03-31	9999-12-31	2017-04-14
Diagnosis: ICD10 Code, F00-F99 Define	F01-F99 Mental, Behavioral and Neurodevelopmental disorders	332552	Co-occurrence of multiple mental disorders	ICD10CM	1	F99		Mental disorder, not otherwise specified	IMO	GHS-PACDC	DX_LEGACY	ACTIVE	N	1900-01-01	9999-12-31	2015-07-10

Diagnosis: ICD10 Code, F00-F99 Define	F01-F99 Mental, Behavioral and Neurodevelopmental disorders	486264	Mild neurocognitive disorder	ICD10CM	1	F99		Mental disorder, not otherwise specified	IMO	GHS-PACDC	IMO_2015_R	ACTIVE	N	1900-01-01	2016-10-14	2015-07-10
Diagnosis: ICD10 Code, F00-F99 Define	F01-F99 Mental, Behavioral and Neurodevelopmental disorders	226052	Steroid euphoria	ICD10CM	1	F99		Mental disorder, not otherwise specified	IMO	GHS-PACDC	DX_LEGACY	ACTIVE	N	1900-01-01	9999-12-31	2015-07-10
Diagnosis: ICD10 Code, F00-F99 Define	F01-F99 Mental, Behavioral and Neurodevelopmental disorders	328332	Socially inappropriate behavior	ICD10CM	1	F99		Mental disorder, not otherwise specified	IMO	GHS-PACDC	DX_LEGACY	ACTIVE	N	1900-01-01	9999-12-31	2015-07-10
Diagnosis: ICD10 Code, F00-F99 Define	F01-F99 Mental, Behavioral and Neurodevelopmental disorders	245662	Mental derangement	ICD10CM	1	F99		Mental disorder, not otherwise specified	IMO	GHS-PACDC	DX_LEGACY	ACTIVE	N	1900-01-01	9999-12-31	2015-07-10
Diagnosis: ICD10 Code, F00-F99 Define	F01-F99 Mental, Behavioral and Neurodevelopmental disorders	95212	Breakdown	ICD10CM	1	F99		Mental disorder, not otherwise specified	IMO	GHS-PACDC	DX_LEGACY	DELETED	Y	1900-01-01	2017-04-27	2015-07-10
Diagnosis: ICD10 Code, F00-F99 Define	F01-F99 Mental, Behavioral and Neurodevelopmental disorders	1138004	Mental disorder, not otherwise specified	ICD10CM	1	F99		Mental disorder, not otherwise specified	IMO	GHS-PACDC	IMO_2016_R	DELETED	Y	1900-01-01	9999-12-31	2016-06-02
Diagnosis: ICD10 Code, F00-F99 Define	F01-F99 Mental, Behavioral and Neurodevelopmental disorders	127307	Psychiatric diagnosis	ICD10CM	1	F99		Mental disorder, not otherwise specified	IMO	GHS-PACDC	DX_LEGACY	ACTIVE	N	1900-01-01	9999-12-31	2015-07-10
Diagnosis: ICD10 Code, F00-F99 Define	F01-F99 Mental, Behavioral and Neurodevelopmental disorders	328739	Unresolved independence-dependence conflict	ICD10CM	1	F99		Mental disorder, not otherwise specified	IMO	GHS-PACDC	DX_LEGACY	ACTIVE	N	1900-01-01	2017-10-14	2015-07-10
Diagnosis: ICD10 Code, I00-I99, Define	I00-I99 Diseases of the circulatory system	73332	Rheumatism, articular, acute or subacute	ICD10CM	1	I00		Rheumatic fever without heart involvement	IMO	GHS-PACDC	DX_LEGACY	ACTIVE	N	1900-01-01	9999-12-31	2015-07-10
Diagnosis: ICD10 Code, I00-I99, Define	I00-I99 Diseases of the circulatory system	218317	Endocarditis, acute rheumatic	ICD10CM	1	I01.1		Acute rheumatic endocarditis	IMO	GHS-PACDC	DX_LEGACY	ACTIVE	N	1900-01-01	9999-12-31	2015-07-10
Diagnosis: ICD10 Code, I00-I99, Define	I00-I99 Diseases of the circulatory system	4859	Acute rheumatic heart disease	ICD10CM	1	I01.9		Acute rheumatic heart disease, unspecified	IMO	GHS-PACDC	DX_LEGACY	ACTIVE	N	1900-01-01	9999-12-31	2015-07-10
Diagnosis: ICD10 Code, I00-I99, Define	I00-I99 Diseases of the circulatory system	535546	Rheumatic chorea	ICD10CM	1	I02		Rheumatic chorea	IMO	GHS-PACDC	IMO_2015_R	ACTIVE	Y	1900-01-01	9999-12-31	2015-07-10
Diagnosis: ICD10 Code, I00-I99, Define	I00-I99 Diseases of the circulatory system	325504	Mild mitral stenosis	ICD10CM	1	I05.0		Rheumatic mitral stenosis	IMO	GHS-PACDC	DX_LEGACY	ACTIVE	N	1900-01-01	9999-12-31	2015-07-10
Diagnosis: ICD10 Code, I00-I99, Define	I00-I99 Diseases of the circulatory system	702533	Maternal mitral stenosis, third trimester	ICD10CM	2	I05.0	O99.413,I05.0	Rheumatic mitral stenosis	IMO	GHS-PACDC	IMO_2015_R	DELETED	Y	1900-01-01	9999-12-31	2015-07-10
Diagnosis: ICD10 Code, I00-I99, Define	I00-I99 Diseases of the circulatory system	250458	Severe mitral stenosis by prior echocardiogram	ICD10CM	1	I05.0		Rheumatic mitral stenosis	IMO	GHS-PACDC	DX_LEGACY	ACTIVE	N	1900-01-01	9999-12-31	2015-07-10
Diagnosis: ICD10 Code, I00-I99, Define	I00-I99 Diseases of the circulatory system	21733	Rheumatic mitral insufficiency	ICD10CM	1	I05.1		Rheumatic mitral insufficiency	IMO	GHS-PACDC	DX_LEGACY	ACTIVE	N	1900-01-01	9999-12-31	2015-07-10
Diagnosis: ICD10 Code, I00-I99, Define	I00-I99 Diseases of the circulatory system	228906	Mitral annular calcification	ICD10CM	1	I05.9		Rheumatic mitral valve disease, unspecified	IMO	GHS-PACDC	DX_LEGACY	ACTIVE	N	1900-01-01	9999-12-31	2015-07-10
Diagnosis: ICD10 Code, I00-I99, Define	I00-I99 Diseases of the circulatory system	521055	Mitral valve disorder during pregnancy in first trimester	ICD10CM	2	I05.9	O99.411,I05.9	Rheumatic mitral valve disease, unspecified	IMO	GHS-PACDC	IMO_2015_R	ACTIVE	N	1900-01-01	9999-12-31	2015-07-10
Diagnosis: ICD10 Code, I00-I99, Define	I00-I99 Diseases of the circulatory system	303111	Rheumatic mitral valve annular calcification	ICD10CM	1	I05.9		Rheumatic mitral valve disease, unspecified	IMO	GHS-PACDC	DX_LEGACY	ACTIVE	N	1900-01-01	9999-12-31	2015-07-10
Diagnosis: ICD10 Code, I00-I99, Define	I00-I99 Diseases of the circulatory system	211591	Aortic valve disease, rheumatic	ICD10CM	1	I06.9		Rheumatic aortic valve disease, unspecified	IMO	GHS-PACDC	DX_LEGACY	ACTIVE	N	1900-01-01	9999-12-31	2015-07-10
Diagnosis: ICD10 Code, I00-I99, Define	I00-I99 Diseases of the circulatory system	1135073	Moderate tricuspid stenosis by prior echocardiography	ICD10CM	1	I07.0		Rheumatic tricuspid stenosis	IMO	GHS-PACDC	IMO_2016_R	ACTIVE	N	1900-01-01	9999-12-31	2016-06-02
Diagnosis: ICD10 Code, I00-I99, Define	I00-I99 Diseases of the circulatory system	250477	Trace tricuspid regurgitation by prior echocardiogram	ICD10CM	1	I07.1		Rheumatic tricuspid insufficiency	IMO	GHS-PACDC	DX_LEGACY	ACTIVE	N	1900-01-01	9999-12-31	2015-07-10
Diagnosis: ICD10 Code, I00-I99, Define	I00-I99 Diseases of the circulatory system	114024	Rheumatic tricuspid insufficiency	ICD10CM	1	I07.1		Rheumatic tricuspid insufficiency	IMO	GHS-PACDC	DX_LEGACY	ACTIVE	N	1900-01-01	9999-12-31	2015-07-10
Diagnosis: ICD10 Code, I00-I99, Define	I00-I99 Diseases of the circulatory system	57623	Tricuspid regurgitation	ICD10CM	1	I07.1		Rheumatic tricuspid insufficiency	IMO	GHS-PACDC	DX_LEGACY	ACTIVE	N	1900-01-01	9999-12-31	2015-07-10
Diagnosis: ICD10 Code, I00-I99, Define	I00-I99 Diseases of the circulatory system	21890	Tricuspid valve disorder	ICD10CM	1	I07.9		Rheumatic tricuspid valve disease, unspecified	IMO	GHS-PACDC	DX_LEGACY	ACTIVE	N	1900-01-01	9999-12-31	2015-07-10
Diagnosis: ICD10 Code, I00-I99, Define	I00-I99 Diseases of the circulatory system	21748	Rheumatic mitral and aortic valve regurgitation	ICD10CM	1	I08.0		Rheumatic disorders of both mitral and aortic valves	IMO	GHS-PACDC	DX_LEGACY	ACTIVE	N	1900-01-01	9999-12-31	2015-07-10
Diagnosis: ICD10 Code, I00-I99, Define	I00-I99 Diseases of the circulatory system	236169	Stenosis of aortic and mitral valves	ICD10CM	1	I08.0		Rheumatic disorders of both mitral and aortic valves	IMO	GHS-PACDC	DX_LEGACY	ACTIVE	N	1900-01-01	9999-12-31	2015-07-10
Diagnosis: ICD10 Code, I00-I99, Define	I00-I99 Diseases of the circulatory system	21718	Rheumatic heart valve stenosis	ICD10CM	1	I09.1		Rheumatic diseases of endocardium, valve unspecified	IMO	GHS-PACDC	DX_LEGACY	ACTIVE	N	1900-01-01	9999-12-31	2015-07-10
Diagnosis: ICD10 Code, I00-I99, Define	I00-I99 Diseases of the circulatory system	61981	Rheumatic diseases of endocardium, valve unspecified	ICD10CM	1	I09.1		Rheumatic diseases of endocardium, valve unspecified	IMO	GHS-PACDC	DX_LEGACY	ACTIVE	N	1900-01-01	9999-12-31	2015-07-10
Diagnosis: ICD10 Code, I00-I99, Define	I00-I99 Diseases of the circulatory system	81121	Pericarditis, chronic, rheumatic	ICD10CM	1	I09.2		Chronic rheumatic pericarditis	IMO	GHS-PACDC	DX_LEGACY	ACTIVE	N	1900-01-01	9999-12-31	2015-07-10
Diagnosis: ICD10 Code, I00-I99, Define	I00-I99 Diseases of the circulatory system	517795	Acute diastolic congestive heart failure due to valvular disease (HCC)	ICD10CM	1	I09.81	I09.81,I50.31	Rheumatic heart failure	IMO	GHS-PACDC	IMO_2015_R	ACTIVE	N	1900-01-01	9999-12-31	2015-07-10
Diagnosis: ICD10 Code, I00-I99, Define	I00-I99 Diseases of the circulatory system	234864	Rheumatic heart valve failure (HCC)	ICD10CM	1	I09.81	I09.81,I50.9	Rheumatic heart failure	IMO	GHS-PACDC	DX_LEGACY	ACTIVE	N	1900-01-01	2017-10-14	2015-07-10
Diagnosis: ICD10 Code, I00-I99, Define	I00-I99 Diseases of the circulatory system	21761	Rheumatic pulmonary valve disease	ICD10CM	1	I09.89		Other specified rheumatic heart diseases	IMO	GHS-PACDC	DX_LEGACY	ACTIVE	N	1900-01-01	9999-12-31	2015-07-10
Diagnosis: ICD10 Code, I00-I99, Define	I00-I99 Diseases of the circulatory system	80301	Rheumatoid carditis	ICD10CM	1	I09.9		Rheumatic heart disease, unspecified	IMO	GHS-PACDC	DX_LEGACY	ACTIVE	N	1900-01-01	9999-12-31	2015-07-10
Diagnosis: ICD10 Code, I00-I99, Define	I00-I99 Diseases of the circulatory system	1092539	Hypertension as manifestation of blood transfusion reaction, subsequent encounter	ICD10CM	2	I10	T80.89XD,I10	Essential (primary) hypertension	IMO	GHS-PACDC	IMO_2015_R	ACTIVE	N	1900-01-01	2017-04-27	2015-07-10
Diagnosis: ICD10 Code, I00-I99, Define	I00-I99 Diseases of the circulatory system	384266	Malignant essential hypertension with congestive heart failure, NYHA class 4 (HCC)	ICD10CM	1	I10	I10,I50.9	Essential (primary) hypertension	IMO	GHS-PACDC	DX_LEGACY	ACTIVE	N	1900-01-01	2016-10-14	2015-07-10
Diagnosis: ICD10 Code, I00-I99, Define	I00-I99 Diseases of the circulatory system	531851	Essential hypertension associated with mutation in PTGIS gene	ICD10CM	1	I10		Essential (primary) hypertension	IMO	GHS-PACDC	IMO_2015_R	ACTIVE	N	1900-01-01	9999-12-31	2015-07-10
Diagnosis: ICD10 Code, I00-I99, Define	I00-I99 Diseases of the circulatory system	1303826	Elevated heart rate with elevated blood pressure and diagnosis of hypertension	ICD10CM	1	I10	I10,R00.9	Essential (primary) hypertension	IMO	GHS-PACDC	IMO_2017_R	ACTIVE	N	1900-01-01	9999-12-31	2017-04-14
Diagnosis: ICD10 Code, I00-I99, Define	I00-I99 Diseases of the circulatory system	144798	HTN (hypertension), malignant	ICD10CM	1	I10		Essential (primary) hypertension	IMO	GHS-PACDC	DX_LEGACY	ACTIVE	N	1900-01-01	9999-12-31	2015-07-10
Diagnosis: ICD10 Code, I00-I99, Define	I00-I99 Diseases of the circulatory system	342305	Intracranial hemorrhage, spontaneous intraparenchymal, associated with hypertension, in utero	ICD10CM	2	I10	P54.8,I10	Essential (primary) hypertension	IMO	GHS-PACDC	DX_LEGACY	ACTIVE	N	1900-01-01	9999-12-31	2015-07-10
Diagnosis: ICD10 Code, I00-I99, Define	I00-I99 Diseases of the circulatory system	106810	Dia hypertension	ICD10CM	1	I10		Essential (primary) hypertension	IMO	GHS-PACDC	DX_LEGACY	ACTIVE	N	1900-01-01	9999-12-31	2015-07-10
Diagnosis: ICD10 Code, I00-I99, Define	I00-I99 Diseases of the circulatory system	523534	Systolic hypertension in pediatric patient	ICD10CM	1	I10		Essential (primary) hypertension	IMO	GHS-PACDC	IMO_2015_R	ACTIVE	N	1900-01-01	9999-12-31	2015-07-10
Diagnosis: ICD10 Code, I00-I99, Define	I00-I99 Diseases of the circulatory system	21493	Accelerated essential hypertension	ICD10CM	1	I10		Essential (primary) hypertension	IMO	GHS-PACDC	DX_LEGACY	ACTIVE	N	1900-01-01	9999-12-31	2015-07-10
Diagnosis: ICD10 Code, I00-I99, Define	I00-I99 Diseases of the circulatory system	342091	Spontaneous perinatal intraventricular hemorrhage associated with hypertension	ICD10CM	2	I10	P52.3,I10	Essential (primary) hypertension	IMO	GHS-PACDC	DX_LEGACY	ACTIVE	N	1900-01-01	9999-12-31	2015-07-10
Diagnosis: ICD10 Code, I00-I99, Define	I00-I99 Diseases of the circulatory system	380341	Hypertension not at goal	ICD10CM	1	I10		Essential (primary) hypertension	IMO	GHS-PACDC	DX_LEGACY	ACTIVE	N	1900-01-01	9999-12-31	2015-07-10
Diagnosis: ICD10 Code, I00-I99, Define	I00-I99 Diseases of the circulatory system	262621	Malignant hypertension requiring acute intensive management	ICD10CM	1	I10		Essential (primary) hypertension	IMO	GHS-PACDC	DX_LEGACY	ACTIVE	N	1900-01-01	9999-12-31	2015-07-10
Diagnosis: ICD10 Code, I00-I99, Define	I00-I99 Diseases of the circulatory system	254062	Malignant systolic hypertension without heart disease	ICD10CM	1	I10		Essential (primary) hypertension	IMO	GHS-PACDC	DX_LEGACY	ACTIVE	N	1900-01-01	9999-12-31	2015-07-10
Diagnosis: ICD10 Code, I00-I99, Define	I00-I99 Diseases of the circulatory system	253471	Hypertensive response to exercise	ICD10CM	1	I10		Essential (primary) hypertension	IMO	GHS-PACDC	DX_LEGACY	ACTIVE	N	1900-01-01	9999-12-31	2015-07-10
Diagnosis: ICD10 Code, I00-I99, Define	I00-I99 Diseases of the circulatory system	202581	Acute spontaneous intraventricular hemorrhage assoc w/ hypertension (HCC)	ICD10CM	2	I10	I61.5,I10	Essential (primary) hypertension	IMO	GHS-PACDC	DX_LEGACY	ACTIVE	N	1900-01-01	9999-12-31	2015-07-10
Diagnosis: ICD10 Code, I00-I99, Define	I00-I99 Diseases of the circulatory system	341812	Intracranial hemorrhage, spontaneous subarachnoid, associated with hypertension, chronic (HC	ICD10CM	2	I10	I60.9,I10	Essential (primary) hypertension	IMO	GHS-PACDC	DX_LEGACY	ACTIVE	N	1900-01-01	9999-12-31	2015-07-10
Diagnosis: ICD10 Code, I00-I99, Define	I00-I99 Diseases of the circulatory system	696444	Malignant hypertension complicating pregnancy, second trimester	ICD10CM	2	I10	O10.912,I10	Essential (primary) hypertension	IMO	GHS-PACDC	IMO_2015_R	ACTIVE	N	1900-01-01	2016-05-07	2015-07-10
Diagnosis: ICD10 Code, I00-I99, Define	I00-I99 Diseases of the circulatory system	384455	Malignant essential hypertension with CHF, NYHA class 3 (HCC)	ICD10CM	1	I10	I10,I50.9	Essential (primary) hypertension	IMO	GHS-PACDC	DX_LEGACY	ACTIVE	N	1900-01-01	2016-10-14	2015-07-10
Diagnosis: ICD10 Code, I00-I99, Define	I00-I99 Diseases of the circulatory system	1131596	Hypertension with goal blood pressure less than 130/85	ICD10CM	1	I10		Essential (primary) hypertension	IMO	GHS-PACDC	IMO_2016_R	ACTIVE	N	1900-01-01	9999-12-31	2016-06-02
Diagnosis: ICD10 Code, I00-I99, Define	I00-I99 Diseases of the circulatory system	115129	Sys hypertension	ICD10CM	1	I10		Essential (primary) hypertension	IMO	GHS-PACDC	DX_LEGACY	ACTIVE	N	1900-01-01	9999-12-31	2015-07-10
Diagnosis: ICD10 Code, I00-I99, Define	I00-I99 Diseases of the circulatory system	499856	Benign essential hypertension with target blood pressure below 140/90	ICD10CM	1	I10		Essential (primary) hypertension	IMO	GHS-PACDC	IMO_2015_R	ACTIVE	N	1900-01-01	9999-12-31	2015-07-10
Diagnosis: ICD10 Code, I00-I99, Define	I00-I99 Diseases of the circulatory system	645909	Maternal chronic hypertension, unspecified trimester	ICD10CM	2	I10	O10.919,I10	Essential (primary) hypertension	IMO	GHS-PACDC	IMO_2015_R	ACTIVE	N	1900-01-01	2016-05-07	2015-07-10
Diagnosis: ICD10 Code, I00-I99, Define	I00-I99 Diseases of the circulatory system	134800	Situational hypertension	ICD10CM	1	I10		Essential (primary) hypertension	IMO	GHS-PACDC	DX_LEGACY	ACTIVE	N	2016-04-10	2017-04-27	2015-07-10
Diagnosis: ICD10 Code, I00-I99, Define	I00-I99 Diseases of the circulatory system	228444	Hypertension associated with diabetes (HCC)	ICD10CM	2	I10	E11.59,I10	Essential (primary) hypertension	IMO	GHS-PACDC	DX_LEGACY	ACTIVE	N	2016-04-10	9999-12-31	2015-07-10
Diagnosis: ICD10 Code, I00-I99, Define	I00-I99 Diseases of the circulatory system	299584	Hypertension, uncontrolled	ICD10CM	1	I10		Essential (primary) hypertension	IMO	GHS-PACDC	DX_LEGACY	ACTIVE	N	1900-01-01	9999-12-31	2015-07-10
Diagnosis: ICD10 Code, I00-I99, Define	I00-I99 Diseases of the circulatory system	59067	Primary hypertension	ICD10CM	1	I10		Essential (primary) hypertension	IMO	GHS-PACDC	DX_LEGACY	ACTIVE	N	1900-01-01	9999-12-31	2015-07-10
Diagnosis: ICD10 Code, I00-I99, Define	I00-I99 Diseases of the circulatory system	143059	Blood pressure elevated	ICD10CM	1	I10		Essential (primary) hypertension	IMO	GHS-PACDC	DX_LEGACY	DELETED	Y	2016-04-10	2017-04-27	2015-07-10
Diagnosis: ICD10 Code, I00-I99, Define	I00-I99 Diseases of the circulatory system	229448	Poor hypertension control	ICD10CM	1	I10		Essential (primary) hypertension	IMO	GHS-PACDC	DX_LEGACY	ACTIVE	N	1900-01-01	9999-12-31	2015-07-10
Diagnosis: ICD10 Code, I00-I99, Define	I00-I99 Diseases of the circulatory system	301833	Hypertension in child age 0-18	ICD10CM	1	I10		Essential (primary) hypertension	IMO	GHS-PACDC	DX_LEGACY	ACTIVE	N	1900-01-01	9999-12-31	2015-07-10
Diagnosis: ICD10 Code, I00-I99, Define	I00-I99 Diseases of the circulatory system	21486	Idiopathic hypertension	ICD10CM	1	I10		Essential (primary) hypertension	IMO	GHS-PACDC	DX_LEGACY	ACTIVE	N	1900-01-01	9999-12-31	2015-07-10
Diagnosis: ICD10 Code, I00-I99, Define	I00-I99 Diseases of the circulatory system	59068	Unspecified essential hypertension	ICD10CM	1	I10		Essential (primary) hypertension	IMO	GHS-PACDC	DX_LEGACY	ACTIVE	N	1900-01-01	9999-12-31	2015-07-10
Diagnosis: ICD10 Code, I00-I99, Define	I00-I99 Diseases of the circulatory system	383677	Benign hypertensive heart disease with congestive heart failure with reduced left ventricular fu	ICD10CM	1	I11.0	I11.0,I50.9	Hypertensive heart disease with heart failure	IMO	GHS-PACDC	DX_LEGACY	ACTIVE	N	1900-01-01	9999-12-31	2015-07-10
Diagnosis: ICD10 Code, I00-I99, Define	I00-I99 Diseases of the circulatory system	384192	Benign hypertensive heart disease with CHF, NYHA class 1 (HCC)	ICD10CM	1	I11.0	I11.0,I50.9	Hypertensive heart disease with heart failure	IMO	GHS-PACDC	DX_LEGACY	ACTIVE	N	1900-01-01	9999-12-31	2015-07-10
Diagnosis: ICD10 Code, I00-I99, Define	I00-I99 Diseases of the circulatory system	159163	Heart failure due to high blood pressure (HCC)	ICD10CM	1	I11.0		Hypertensive heart disease with heart failure	IMO	GHS-PACDC	DX_LEGACY	ACTIVE	N	1900-01-01	9999-12-31	2015-07-10
Diagnosis: ICD10 Code, I00-I99, Define	I00-I99 Diseases of the circulatory system	384404	Malignant syst hypertension w/congestive heart failure, NYHA class 1 (HCC)	ICD10CM	1	I11.0		Hypertensive heart disease with heart failure	IMO	GHS-PACDC	DX_LEGACY	ACTIVE	N	2016-09-17	9999-12-31	2016-09-30
Diagnosis: ICD10 Code, I00-I99, Define	I00-I99 Diseases of the circulatory system	383326	Hypertensive heart disease with congestive heart failure and with combined systolic and diastol	ICD10CM	1	I11.0	I11.0,I50.40	Hypertensive heart disease with heart failure	IMO	GHS-PACDC	DX_LEGACY	ACTIVE	N	1900-01-01	9999-12-31	2015-07-10
Diagnosis: ICD10 Code, I00-I99, Define	I00-I99 Diseases of the circulatory system	633130	Pre-existing hypertensive heart disease complicating pregnancy, first trimester	ICD10CM	3	I11.0	O10.111,I11.9,I11.0	Hypertensive heart disease with heart failure	IMO	GHS-PACDC	IMO_2015_R	ACTIVE	N	1900-01-01	2016-05-07	2015-07-10
Diagnosis: ICD10 Code, I00-I99, Define	I00-I99 Diseases of the circulatory system	960510	Hypertensive left ventricular hypertrophy, with heart failure (HCC)	ICD10CM	1	I11.0		Hypertensive heart disease with heart failure	IMO	GHS-PACDC	IMO_2015_R	ACTIVE	N	1900-01-01	9999-12-31	2015-07-10
Diagnosis: ICD10 Code, I00-I99, Define	I00-I99 Diseases of the circulatory system	222479	Hypertensive CHF (congestive heart failure) (HCC)	ICD10CM	1	I11.0	I11.0,I50.9	Hypertensive heart disease with heart failure	IMO	GHS-PACDC	DX_LEGACY	ACTIVE	N	1900-01-01	2016-05-07	2015-07-10
Diagnosis: ICD10 Code, I00-I99, Define	I00-I99 Diseases of the circulatory system	384348	Malignant diastolic hypertension with CHF, NYHA class 3 (HCC)	ICD10CM	1	I11.0		Hypertensive heart disease with heart failure	IMO	GHS-PACDC	DX_LEGACY	ACTIVE	N	2016-09-17	9999-12-31	2016-09-30
Diagnosis: ICD10 Code, I00-I99, Define	I00-I99 Diseases of the circulatory system	505373	Hypertension, accelerated, with diastolic congestive heart failure, NYHA class 1 (HCC)	ICD10CM	1	I11.0	I11.0,I50.30	Hypertensive heart disease with heart failure	IMO	GHS-PACDC	IMO_2015_R	ACTIVE	N	2016-09-17	9999-12-31	2016-09-30
Diagnosis: ICD10 Code, I00-I99, Define	I00-I99 Diseases of the circulatory system	384497	Malignant essential hypertension with congestive heart failure without renal disease (HCC)	ICD10CM	1	I11.0		Hypertensive heart disease with heart failure	IMO	GHS-PACDC	DX_LEGACY	ACTIVE	N	2016-09-17	9999-12-31	2016-09-30
Diagnosis: ICD10 Code, I00-I99, Define	I00-I99 Diseases of the circulatory system	427584	Cardiomyopathy due to hypertension, with heart failure (HCC)	ICD10CM	1	I11.0	I11.0,I42.9	Hypertensive heart disease with heart failure	IMO	GHS-PACDC	IMO_2015_R	ACTIVE	N	1900-01-01	2016-05-07	2015-07-10
Diagnosis: ICD10 Code, I00-I99, Define	I00-I99 Diseases of the circulatory system	1210654	Pre-existing hypertensive heart disease affecting pregnancy, first trimester	ICD10CM	2	I11.9	O10.111,I11.9	Hypertensive heart disease without heart failure	IMO	GHS-PACDC	IMO_2016_R	ACTIVE	N	1900-01-01	9999-12-31	2016-06-02
Diagnosis: ICD10 Code, I00-I99, Define	I00-I99 Diseases of the circulatory system	29254	Hypertensive heart disease in obstetric context	ICD10CM	2	I11.9	O10.119,I11.9	Hypertensive heart disease without heart failure	IMO	GHS-PACDC	DX_LEGACY	ACTIVE	N	1900-01-01	2017-04-27	2015-07-10
Diagnosis: ICD10 Code, I00-I99, Define	I00-I99 Diseases of the circulatory system	165376	Hypertensive heart disease complicating pregnancy	ICD10CM	2	I11.9	O10.119,I11.9	Hypertensive heart disease without heart failure	IMO	GHS-PACDC	DX_LEGACY	ACTIVE	N	1900-01-01	9999-12-31	2015-07-10
Diagnosis: ICD10 Code, I00-I99, Define	I00-I99 Diseases of the circulatory system	222491	Malignant hypertensive heart disease without HF (heart failure)	ICD10CM	1	I11.9		Hypertensive heart disease without heart failure	IMO	GHS-PACDC	DX_LEGACY	ACTIVE	N	1900-01-01	9999-12-31	2015-07-10
Diagnosis: ICD10 Code, I00-I99, Define	I00-I99 Diseases of the circulatory system	252280	Cardiomyopathy, hypertensive, benign	ICD10CM	1	I11.9	I11.9,I43	Hypertensive heart disease without heart failure	IMO	GHS-PACDC	DX_LEGACY	ACTIVE	N	2017-09-17	9999-12-31	2017-10-30
Diagnosis: ICD10 Code, I00-I99, Define	I00-I99 Diseases of the circulatory system	1041841	Hypertensive heart disease in obstetric context, first trimester	ICD10CM	2	I11.9	O10.111,I11.9	Hypertensive heart disease without heart failure	IMO	GHS-PACDC	IMO_2015_R	ACTIVE	N	2016-04-10	2017-04-27	2015-07-10
Diagnosis: ICD10 Code, I00-I99, Define	I00-I99 Diseases of the circulatory system	222456	Hypertensive heart and kidney disease without HF or CKD	ICD10CM	1	I11.9	I11.9,N28.9	Hypertensive heart disease without heart failure	IMO	GHS-PACDC	DX_LEGACY	ACTIVE	N	1900-01-01	2016-10-14	2015-07-10
Diagnosis: ICD10 Code, I00-I99, Define	I00-I99 Diseases of the circulatory system	21511	Hypertensive cardiopathy	ICD10CM	1	I11.9		Hypertensive heart disease without heart failure	IMO	GHS-PACDC	DX_LEGACY	ACTIVE	N	1900-01-01	9999-12-31	2015-07-10
Diagnosis: ICD10 Code, I00-I99, Define	I00-I99 Diseases of the circulatory system	136028	Malignant hypertensive heart disease without heart failure	ICD10CM	1	I11.9		Hypertensive heart disease without heart failure	IMO	GHS-PACDC	DX_LEGACY	ACTIVE	N	1900-01-01	9999-12-31	2015-07-10
Diagnosis: ICD10 Code, I00-I99, Define	I00-I99 Diseases of the circulatory system	963640	Hypertensive renal disease, benign, stage 5 chronic kidney disease or end stage renal disease (H	ICD10CM	1	I12.0		Hypertensive chronic kidney disease with stage 5 chroni	IMO	GHS-PACDC	IMO_2015_R	ACTIVE	N	2016-09-17	9999-12-31	2016-09-30
Diagnosis: ICD10 Code, I00-I99, Define	I00-I99 Diseases of the circulatory system	359736	Malignant hypertension with heart failure and chronic kidney disease stage V (HCC)	ICD10CM	1	I12.0	I12.0,I50.9,N18.5	Hypertensive chronic kidney disease with stage 5 chroni	IMO	GHS-PACDC	DX_LEGACY	ACTIVE	N	1900-01-01	2016-10-14	2015-07-10
Diagnosis: ICD10 Code, I00-I99, Define	I00-I99 Diseases of the circulatory system	1123986	Stage 5 chronic kidney disease due to benign hypertension (HCC)	ICD10CM	1	I12.0	I12.0,N18.5	Hypertensive chronic kidney disease with stage 5 chroni	IMO	GHS-PACDC	IMO_2016_R	ACTIVE	N	1900-01-01	9999-12-31	2016-06-02
Diagnosis: ICD10 Code, I00-I99, Define	I00-I99 Diseases of the circulatory system	153660	Hypertensive kidney disease with end-stage renal disease (HCC)	ICD10CM	1	I12.0	I12.0,N18.6	Hypertensive chronic kidney disease with stage 5 chroni	IMO	GHS-PACDC	DX_LEGACY	ACTIVE	N	1900-01-01	9999-12-31	2015-07-10
Diagnosis: ICD10 Code, I00-I99, Define	I00-I99 Diseases of the circulatory system	894492	Antepartum renal hypertension, unspecified trimester	ICD10CM	3	I12.0	O10.219,I12.9,I12.0	Hypertensive chronic kidney disease with stage 5 chroni	IMO	GHS-PACDC	IMO_2015_R	ACTIVE	N	1900-01-01	2016-05-07	2015-07-10
Diagnosis: ICD10 Code, I00-I99, Define	I00-I99 Diseases of the circulatory system	222576	Benign hypertension with CKD (chronic kidney disease) stage V (HCC)	ICD10CM	1	I12.0	I12.0,N18.5	Hypertensive chronic kidney disease with stage 5 chroni	IMO	GHS-PACDC	DX_LEGACY	ACTIVE	N	1900-01-01	9999-12-31	2015-07-10
Diagnosis: ICD10 Code, I00-I99, Define	I00-I99 Diseases of the circulatory system	1105198	Hypertension due to end stage renal disease on dialysis (HCC)	ICD10CM	1	I12.0	I12.0,N18.6,Z99.2	Hypertensive chronic kidney disease with stage 5 chroni	IMO	GHS-PACDC	IMO_2016_R	ACTIVE	N	1900-01-01	9999-12-31	2016-06-02
Diagnosis: ICD10 Code, I00-I99, Define	I00-I99 Diseases of the circulatory system	682824	Hypertensive CKD (chronic kidney disease) complicating pregnancy, first trimester	ICD10CM	3	I12.0	O10.211,I12.9,I12.0	Hypertensive chronic kidney disease with stage 5 chroni	IMO	GHS-PACDC	IMO_2015_R	DELETED	Y	1900-01-01	9999-12-31	2015-07-10
Diagnosis: ICD10 Code, I00-I99, Define	I00-I99 Diseases of the circulatory system	1083634	Pre-existing hypertensive chronic kidney disease affecting pregnancy, antepartum, first trimeste	ICD10CM	2	I12.0	O10.211,I12.0,I12.9	Hypertensive chronic kidney disease with stage 5 chroni	IMO	GHS-PACDC	IMO_2015_R	ACTIVE	N	1900-01-01	2016-05-07	2015-07-10

Diagnosis: ICD10 Code, I00-I99, Define	I00-I99	Diseases of the circulatory system	1114868	Type 1 DM with hypertension and ESRD on dialysis (HCC)	ICD10CM	2	I12.0	E10.22,I12.0,Z99.2,N18.6	Hypertensive chronic kidney disease with stage 5 chroni	IMO	GHS-PACDC	IMO_2016_R	ACTIVE	N	1900-01-01	9999-12-31	2016-06-02
Diagnosis: ICD10 Code, I00-I99, Define	I00-I99	Diseases of the circulatory system	703954	Hypertension in pregnancy, renal, unspecified trimester	ICD10CM	2	I12.0	O10.219,I12.0,I12.9	Hypertensive chronic kidney disease with stage 5 chroni	IMO	GHS-PACDC	IMO_2015_R	ACTIVE	N	1900-01-01	2016-05-07	2015-07-10
Diagnosis: ICD10 Code, I00-I99, Define	I00-I99	Diseases of the circulatory system	956549	High blood pressure with chronic kidney disease, stage 5 chronic kidney disease or end stage re	ICD10CM	1	I12.0	I12.0,N03.9,N18.5	Hypertensive chronic kidney disease with stage 5 chroni	IMO	GHS-PACDC	IMO_2015_R	ACTIVE	N	1900-01-01	2016-05-07	2015-07-10
Diagnosis: ICD10 Code, I00-I99, Define	I00-I99	Diseases of the circulatory system	661087	Hypertensive CKD (chronic kidney disease) complicating pregnancy, unspecified trimester	ICD10CM	3	I12.0	O10.219,I12.9,I12.0	Hypertensive chronic kidney disease with stage 5 chroni	IMO	GHS-PACDC	IMO_2015_R	DELETED	Y	1900-01-01	9999-12-31	2015-07-10
Diagnosis: ICD10 Code, I00-I99, Define	I00-I99	Diseases of the circulatory system	954187	Arteriolonephrosclerosis, stage 5 chronic kidney disease or end stage renal disease (HCC)	ICD10CM	1	I12.0		Hypertensive chronic kidney disease with stage 5 chroni	IMO	GHS-PACDC	IMO_2015_R	ACTIVE	N	1900-01-01	9999-12-31	2015-07-10
Diagnosis: ICD10 Code, I00-I99, Define	I00-I99	Diseases of the circulatory system	670096	Hypertensive CKD (chronic kidney disease) complicating pregnancy, second trimester	ICD10CM	3	I12.0	O10.212,I12.9,I12.0	Hypertensive chronic kidney disease with stage 5 chroni	IMO	GHS-PACDC	IMO_2015_R	DELETED	Y	1900-01-01	9999-12-31	2015-07-10
Diagnosis: ICD10 Code, I00-I99, Define	I00-I99	Diseases of the circulatory system	153677	Malignant hypertension with heart failure and stage V renal dis (HCC)	ICD10CM	1	I12.0	I12.0,I50.9,N18.5	Hypertensive chronic kidney disease with stage 5 chroni	IMO	GHS-PACDC	DX_LEGACY	ACTIVE	N	1900-01-01	2016-10-14	2015-07-10
Diagnosis: ICD10 Code, I00-I99, Define	I00-I99	Diseases of the circulatory system	339701	Malignant hypertensive kidney disease with chronic kidney disease stage V (HCC)	ICD10CM	1	I12.0		Hypertensive chronic kidney disease with stage 5 chroni	IMO	GHS-PACDC	DX_LEGACY	ACTIVE	N	1900-01-01	2016-05-07	2015-07-10
Diagnosis: ICD10 Code, I00-I99, Define	I00-I99	Diseases of the circulatory system	960466	Benign hypertensive CKD, stage 5 chronic kidney disease or end stage renal disease (HCC)	ICD10CM	1	I12.0		Hypertensive chronic kidney disease with stage 5 chroni	IMO	GHS-PACDC	IMO_2015_R	ACTIVE	N	2016-04-10	9999-12-31	2015-07-10
Diagnosis: ICD10 Code, I00-I99, Define	I00-I99	Diseases of the circulatory system	963640	Hypertensive renal disease, benign, stage 5 chronic kidney disease or end stage renal disease (H	ICD10CM	1	I12.0	I12.0,N18.4,N18.5,N18.3,N18.1,N	Hypertensive chronic kidney disease with stage 5 chroni	IMO	GHS-PACDC	IMO_2015_R	ACTIVE	N	1900-01-01	2016-05-07	2015-07-10
Diagnosis: ICD10 Code, I00-I99, Define	I00-I99	Diseases of the circulatory system	710907	Renal hypertension complicating pregnancy, unspecified trimester	ICD10CM	3	I12.0	O10.219,I12.9,I12.0	Hypertensive chronic kidney disease with stage 5 chroni	IMO	GHS-PACDC	IMO_2015_R	ACTIVE	N	1900-01-01	2016-05-07	2015-07-10
Diagnosis: ICD10 Code, I00-I99, Define	I00-I99	Diseases of the circulatory system	962577	Hypertensive nephrosclerosis, stage 5 chronic kidney disease or end stage renal disease (HCC)	ICD10CM	1	I12.0		Hypertensive chronic kidney disease with stage 5 chroni	IMO	GHS-PACDC	IMO_2015_R	ACTIVE	N	1900-01-01	9999-12-31	2015-07-10
Diagnosis: ICD10 Code, I00-I99, Define	I00-I99	Diseases of the circulatory system	960704	Malignant systolic hypertension with CHF and chronic kidney disease, stage 5 chronic kidney dis	ICD10CM	1	I12.0	I12.0,N18.9	Hypertensive chronic kidney disease with stage 5 chroni	IMO	GHS-PACDC	IMO_2015_R	ACTIVE	N	2016-04-10	2016-10-14	2015-07-10
Diagnosis: ICD10 Code, I00-I99, Define	I00-I99	Diseases of the circulatory system	955809	Hypertensive kidney disease, benign, stage 5 chronic kidney disease or end stage renal disease (	ICD10CM	1	I12.0		Hypertensive chronic kidney disease with stage 5 chroni	IMO	GHS-PACDC	IMO_2015_R	ACTIVE	N	2016-09-17	9999-12-31	2016-09-30
Diagnosis: ICD10 Code, I00-I99, Define	I00-I99	Diseases of the circulatory system	960460	Chronic kidney disease with malignant hypertension, stage 5 chronic kidney disease or end stag	ICD10CM	1	I12.0	I12.0,N03.9	Hypertensive chronic kidney disease with stage 5 chroni	IMO	GHS-PACDC	IMO_2015_R	ACTIVE	N	1900-01-01	2016-05-07	2015-07-10
Diagnosis: ICD10 Code, I00-I99, Define	I00-I99	Diseases of the circulatory system	1367833	Parenchymal renal hypertension, stage 1 through stage 4 or unspecified chronic kidney disease	ICD10CM	1	I12.9		Hypertensive chronic kidney disease with stage 1 throug	IMO	GHS-PACDC	IMO_2018_R	ACTIVE	N	1900-01-01	9999-12-31	2017-10-30
Diagnosis: ICD10 Code, I00-I99, Define	I00-I99	Diseases of the circulatory system	971033	Arteriolar sclerosis of renal arterioles, stage 1-4 or unspecified chronic kidney disease	ICD10CM	1	I12.9		Hypertensive chronic kidney disease with stage 1 throug	IMO	GHS-PACDC	IMO_2015_R	ACTIVE	N	1900-01-01	2016-05-07	2015-07-10
Diagnosis: ICD10 Code, I00-I99, Define	I00-I99	Diseases of the circulatory system	1367881	Ischemic nephropathy with hypertensive nephrosclerosis, stage 1 through stage 4 or unspecifie	ICD10CM	1	I12.9		Hypertensive chronic kidney disease with stage 1 throug	IMO	GHS-PACDC	IMO_2018_R	ACTIVE	N	1900-01-01	9999-12-31	2017-10-30
Diagnosis: ICD10 Code, I00-I99, Define	I00-I99	Diseases of the circulatory system	345010	Pre-existing hypertensive chronic kidney disease complicating pregnancy	ICD10CM	2	I12.9	O10.219,I12.9,N18.9	Hypertensive chronic kidney disease with stage 1 throug	IMO	GHS-PACDC	DX_LEGACY	ACTIVE	N	1900-01-01	9999-12-31	2015-07-10
Diagnosis: ICD10 Code, I00-I99, Define	I00-I99	Diseases of the circulatory system	1114905	Chronic kidney disease due to benign hypertension	ICD10CM	1	I12.9	I12.9,N18.9	Hypertensive chronic kidney disease with stage 1 throug	IMO	GHS-PACDC	IMO_2016_R	ACTIVE	N	1900-01-01	9999-12-31	2016-06-02
Diagnosis: ICD10 Code, I00-I99, Define	I00-I99	Diseases of the circulatory system	333274	Hypertensive renal disease complicating or reason for care during childbirth	ICD10CM	2	I12.9	O10.22,I12.9	Hypertensive chronic kidney disease with stage 1 throug	IMO	GHS-PACDC	DX_LEGACY	ACTIVE	N	1900-01-01	9999-12-31	2015-07-10
Diagnosis: ICD10 Code, I00-I99, Define	I00-I99	Diseases of the circulatory system	647809	Hypertensive renal disease complicating pregnancy, unspecified trimester	ICD10CM	2	I12.9	O10.219,I12.9,I12.0	Hypertensive chronic kidney disease with stage 1 throug	IMO	GHS-PACDC	IMO_2015_R	ACTIVE	N	1900-01-01	2016-05-07	2015-07-10
Diagnosis: ICD10 Code, I00-I99, Define	I00-I99	Diseases of the circulatory system	1366837	Pre-exist hyp chronic kidney disease comp preg, second tri	ICD10CM	2	I12.9	O10.212,I12.9,N18.9	Hypertensive chronic kidney disease with stage 1 throug	IMO	GHS-PACDC	IMO_2018_R	ACTIVE	N	1900-01-01	9999-12-31	2017-10-30
Diagnosis: ICD10 Code, I00-I99, Define	I00-I99	Diseases of the circulatory system	1365498	Pre-existing hyp chronic kidney disease comp the puerperium	ICD10CM	2	I12.9	O10.23,I12.9	Hypertensive chronic kidney disease with stage 1 throug	IMO	GHS-PACDC	IMO_2018_R	ACTIVE	N	1900-01-01	9999-12-31	2017-10-30
Diagnosis: ICD10 Code, I00-I99, Define	I00-I99	Diseases of the circulatory system	971756	Hypertensive renal disease with renal failure, stage 1-4 or unspecified chronic kidney disease	ICD10CM	1	I12.9		Hypertensive chronic kidney disease with stage 1 throug	IMO	GHS-PACDC	IMO_2015_R	ACTIVE	N	2016-04-10	9999-12-31	2015-07-10
Diagnosis: ICD10 Code, I00-I99, Define	I00-I99	Diseases of the circulatory system	970925	Hypertensive CKD (chronic kidney disease), stage 1-4 or unspecified chronic kidney disease	ICD10CM	1	I12.9		Hypertensive chronic kidney disease with stage 1 throug	IMO	GHS-PACDC	IMO_2015_R	DELETED	Y	2016-09-17	9999-12-31	2016-09-30
Diagnosis: ICD10 Code, I00-I99, Define	I00-I99	Diseases of the circulatory system	219033	Renal disease, hypertensive benign	ICD10CM	1	I12.9		Hypertensive chronic kidney disease with stage 1 throug	IMO	GHS-PACDC	DX_LEGACY	ACTIVE	N	2016-09-17	9999-12-31	2016-09-30
Diagnosis: ICD10 Code, I00-I99, Define	I00-I99	Diseases of the circulatory system	1304103	Hypertensive kidney disease complicating or reason for care during pregnancy in third trimeste	ICD10CM	2	I12.9	O10.213,I12.9	Hypertensive chronic kidney disease with stage 1 throug	IMO	GHS-PACDC	IMO_2017_R	ACTIVE	N	1900-01-01	9999-12-31	2017-04-14
Diagnosis: ICD10 Code, I00-I99, Define	I00-I99	Diseases of the circulatory system	21529	Interstitial arteriosclerotic nephritis	ICD10CM	1	I12.9		Hypertensive chronic kidney disease with stage 1 throug	IMO	GHS-PACDC	DX_LEGACY	ACTIVE	N	1900-01-01	9999-12-31	2015-07-10
Diagnosis: ICD10 Code, I00-I99, Define	I00-I99	Diseases of the circulatory system	302984	Benign hypertensive kidney disease, stage II	ICD10CM	1	I12.9	I12.9,N18.2	Hypertensive chronic kidney disease with stage 1 throug	IMO	GHS-PACDC	DX_LEGACY	ACTIVE	N	1900-01-01	9999-12-31	2015-07-10
Diagnosis: ICD10 Code, I00-I99, Define	I00-I99	Diseases of the circulatory system	68850	Nephrosclerosis arteriolar	ICD10CM	1	I12.9		Hypertensive chronic kidney disease with stage 1 throug	IMO	GHS-PACDC	DX_LEGACY	ACTIVE	N	1900-01-01	9999-12-31	2015-07-10
Diagnosis: ICD10 Code, I00-I99, Define	I00-I99	Diseases of the circulatory system	895029	Antepartum renal hypertension, first trimester	ICD10CM	2	I12.9	O10.211,I12.9,I12.0	Hypertensive chronic kidney disease with stage 1 throug	IMO	GHS-PACDC	IMO_2015_R	ACTIVE	N	1900-01-01	2016-05-07	2015-07-10
Diagnosis: ICD10 Code, I00-I99, Define	I00-I99	Diseases of the circulatory system	971102	Ischemic nephropathy with hypertensive nephrosclerosis, stage 1-4 or unspecified chronic kidn	ICD10CM	1	I12.9		Hypertensive chronic kidney disease with stage 1 throug	IMO	GHS-PACDC	IMO_2015_R	ACTIVE	N	1900-01-01	9999-12-31	2015-07-10
Diagnosis: ICD10 Code, I00-I99, Define	I00-I99	Diseases of the circulatory system	970692	Malignant hypertension (arteriolar nephrosclerosis), stage 1-4 or unspecified chronic kidney dis	ICD10CM	1	I12.9		Hypertensive chronic kidney disease with stage 1 throug	IMO	GHS-PACDC	IMO_2015_R	ACTIVE	N	1900-01-01	9999-12-31	2015-07-10
Diagnosis: ICD10 Code, I00-I99, Define	I00-I99	Diseases of the circulatory system	1210553	Pre-existing hypertensive chronic kidney disease affecting pregnancy, second trimester	ICD10CM	2	I12.9	O10.212,I12.9,N18.9	Hypertensive chronic kidney disease with stage 1 throug	IMO	GHS-PACDC	IMO_2016_R	ACTIVE	N	2017-09-17	9999-12-31	2017-10-30
Diagnosis: ICD10 Code, I00-I99, Define	I00-I99	Diseases of the circulatory system	346797	Pre-existing hypertensive chronic kidney disease complicating pregnancy in first trimester	ICD10CM	2	I12.9	O10.211,I12.9,N18.9	Hypertensive chronic kidney disease with stage 1 throug	IMO	GHS-PACDC	DX_LEGACY	ACTIVE	N	1900-01-01	9999-12-31	2015-07-10
Diagnosis: ICD10 Code, I00-I99, Define	I00-I99	Diseases of the circulatory system	600710	Hypertensive chronic kidney disease with stage 1 through stage 4 chronic kidney disease, or uns	ICD10CM	1	I12.9		Hypertensive chronic kidney disease with stage 1 throug	IMO	GHS-PACDC	IMO_2015_R	ACTIVE	Y	1900-01-01	9999-12-31	2015-07-10
Diagnosis: ICD10 Code, I00-I99, Define	I00-I99	Diseases of the circulatory system	970810	Parenchymal renal hypertension, stage 1-4 or unspecified chronic kidney disease	ICD10CM	1	I12.9		Hypertensive chronic kidney disease with stage 1 throug	IMO	GHS-PACDC	IMO_2015_R	ACTIVE	N	1900-01-01	9999-12-31	2015-07-10
Diagnosis: ICD10 Code, I00-I99, Define	I00-I99	Diseases of the circulatory system	1078893	Pre-existing hypertensive heart and renal disease during pregnancy, antepartum, second trimes	ICD10CM	2	I12.9	O10.312,I12.9	Hypertensive chronic kidney disease with stage 1 throug	IMO	GHS-PACDC	IMO_2015_R	ACTIVE	N	2016-04-10	2017-04-27	2015-07-10
Diagnosis: ICD10 Code, I00-I99, Define	I00-I99	Diseases of the circulatory system	109687	Hypertensive renovascular disease	ICD10CM	1	I12.9	I12.9,N18.9	Hypertensive chronic kidney disease with stage 1 throug	IMO	GHS-PACDC	DX_LEGACY	ACTIVE	N	1900-01-01	2016-10-14	2015-07-10
Diagnosis: ICD10 Code, I00-I99, Define	I00-I99	Diseases of the circulatory system	970563	Hardening of the arteries of the kidney, stage 1-4 or unspecified chronic kidney disease	ICD10CM	1	I12.9		Hypertensive chronic kidney disease with stage 1 throug	IMO	GHS-PACDC	IMO_2015_R	ACTIVE	N	2016-09-17	9999-12-31	2016-09-30
Diagnosis: ICD10 Code, I00-I99, Define	I00-I99	Diseases of the circulatory system	971135	Malignant hypertensive renal disease with renal failure, stage 1-4 or unspecified chronic kidney	ICD10CM	1	I12.9		Hypertensive chronic kidney disease with stage 1 throug	IMO	GHS-PACDC	IMO_2015_R	ACTIVE	N	2016-04-10	9999-12-31	2015-07-10
Diagnosis: ICD10 Code, I00-I99, Define	I00-I99	Diseases of the circulatory system	1367896	Malignant nephrosclerosis, stage 1 through stage 4 or unspecified chronic kidney disease	ICD10CM	1	I12.9		Hypertensive chronic kidney disease with stage 1 throug	IMO	GHS-PACDC	IMO_2018_R	ACTIVE	N	1900-01-01	9999-12-31	2017-10-30
Diagnosis: ICD10 Code, I00-I99, Define	I00-I99	Diseases of the circulatory system	28158	Malignant arteriolar nephrosclerosis	ICD10CM	1	I12.9		Hypertensive chronic kidney disease with stage 1 throug	IMO	GHS-PACDC	DX_LEGACY	ACTIVE	N	1900-01-01	9999-12-31	2015-07-10
Diagnosis: ICD10 Code, I00-I99, Define	I00-I99	Diseases of the circulatory system	1078893	Pre-existing hypertensive heart and renal disease during pregnancy, antepartum, second trimes	ICD10CM	2	I12.9	O10.312,I12.9,I12.0	Hypertensive chronic kidney disease with stage 1 throug	IMO	GHS-PACDC	IMO_2015_R	ACTIVE	N	1900-01-01	2016-05-07	2015-07-10
Diagnosis: ICD10 Code, I00-I99, Define	I00-I99	Diseases of the circulatory system	971589	Benign hypertensive kidney disease with CKD (chronic kidney disease), stage 1-4 or unspecified	ICD10CM	1	I12.9		Hypertensive chronic kidney disease with stage 1 throug	IMO	GHS-PACDC	IMO_2015_R	ACTIVE	N	2016-04-10	9999-12-31	2015-07-10
Diagnosis: ICD10 Code, I00-I99, Define	I00-I99	Diseases of the circulatory system	971589	Benign hypertensive kidney disease with CKD (chronic kidney disease), stage 1-4 or unspecified	ICD10CM	1	I12.9	I12.9,N18.3,N18.2,N18.9,N18.4,N	Hypertensive chronic kidney disease with stage 1 throug	IMO	GHS-PACDC	IMO_2015_R	ACTIVE	N	1900-01-01	2016-05-07	2015-07-10
Diagnosis: ICD10 Code, I00-I99, Define	I00-I99	Diseases of the circulatory system	970827	Renal disease due to hypertension, stage 1-4 or unspecified chronic kidney disease	ICD10CM	1	I12.9	I12.9,N18.2,N18.1,N18.5,N18.4,N	Hypertensive chronic kidney disease with stage 1 throug	IMO	GHS-PACDC	IMO_2015_R	ACTIVE	N	1900-01-01	2016-05-07	2015-07-10
Diagnosis: ICD10 Code, I00-I99, Define	I00-I99	Diseases of the circulatory system	971579	Malignant hypertension with renal failure and congestive heart failure, stage 1-4 or unspecified	ICD10CM	1	I12.9	I12.9,I50.9	Hypertensive chronic kidney disease with stage 1 throug	IMO	GHS-PACDC	IMO_2015_R	DELETED	Y	1900-01-01	9999-12-31	2015-07-10
Diagnosis: ICD10 Code, I00-I99, Define	I00-I99	Diseases of the circulatory system	703954	Hypertension in pregnancy, renal, unspecified trimester	ICD10CM	3	I12.9	O10.219,I12.0,I12.9	Hypertensive chronic kidney disease with stage 1 throug	IMO	GHS-PACDC	IMO_2015_R	ACTIVE	N	1900-01-01	2016-05-07	2015-07-10
Diagnosis: ICD10 Code, I00-I99, Define	I00-I99	Diseases of the circulatory system	39528	Hypertension secondary to renal disease, postpartum	ICD10CM	2	I12.9	O10.23,I12.9,N18.9	Hypertensive chronic kidney disease with stage 1 throug	IMO	GHS-PACDC	DX_LEGACY	ACTIVE	N	1900-01-01	9999-12-31	2015-07-10
Diagnosis: ICD10 Code, I00-I99, Define	I00-I99	Diseases of the circulatory system	212837	High blood pressure with chronic kidney disease	ICD10CM	1	I12.9		Hypertensive chronic kidney disease with stage 1 throug	IMO	GHS-PACDC	DX_LEGACY	ACTIVE	N	2016-04-10	9999-12-31	2015-07-10
Diagnosis: ICD10 Code, I00-I99, Define	I00-I99	Diseases of the circulatory system	971173	Hypertensive renal disease, stage 1-4 or unspecified chronic kidney disease	ICD10CM	1	I12.9	I12.9,N18.9	Hypertensive chronic kidney disease with stage 1 throug	IMO	GHS-PACDC	IMO_2015_R	ACTIVE	N	2016-04-10	2016-10-14	2015-07-10
Diagnosis: ICD10 Code, I00-I99, Define	I00-I99	Diseases of the circulatory system	689790	Hypertensive renal disease complicating or reason for care during pregnancy, first trimester	ICD10CM	2	I12.9	O10.211,I12.9,I12.0	Hypertensive chronic kidney disease with stage 1 throug	IMO	GHS-PACDC	IMO_2015_R	ACTIVE	N	1900-01-01	2016-05-07	2015-07-10
Diagnosis: ICD10 Code, I00-I99, Define	I00-I99	Diseases of the circulatory system	1354974	Hypertensive arterionephrosclerosis of transplanted kidney	ICD10CM	2	I12.9	T86.19,I12.9	Hypertensive chronic kidney disease with stage 1 throug	IMO	GHS-PACDC	IMO_2018_R	ACTIVE	N	1900-01-01	9999-12-31	2017-10-30
Diagnosis: ICD10 Code, I00-I99, Define	I00-I99	Diseases of the circulatory system	970947	Renal disease, hypertensive benign, stage 1-4 or unspecified chronic kidney disease	ICD10CM	1	I12.9	I12.9,N18.9	Hypertensive chronic kidney disease with stage 1 throug	IMO	GHS-PACDC	IMO_2015_R	ACTIVE	N	2016-04-10	2016-10-14	2015-07-10
Diagnosis: ICD10 Code, I00-I99, Define	I00-I99	Diseases of the circulatory system	302991	Hypertensive kidney disease, stage IV (HCC)	ICD10CM	1	I12.9	I12.9,N18.4	Hypertensive chronic kidney disease with stage 1 throug	IMO	GHS-PACDC	DX_LEGACY	ACTIVE	N	1900-01-01	9999-12-31	2015-07-10
Diagnosis: ICD10 Code, I00-I99, Define	I00-I99	Diseases of the circulatory system	69787	ANS (arteriolar nephrosclerosis)	ICD10CM	1	I12.9		Hypertensive chronic kidney disease with stage 1 throug	IMO	GHS-PACDC	DX_LEGACY	ACTIVE	N	1900-01-01	9999-12-31	2015-07-10
Diagnosis: ICD10 Code, I00-I99, Define	I00-I99	Diseases of the circulatory system	971296	Arteriolar nephrosclerosis, benign, stage 1-4 or unspecified chronic kidney disease	ICD10CM	1	I12.9		Hypertensive chronic kidney disease with stage 1 throug	IMO	GHS-PACDC	IMO_2015_R	ACTIVE	N	1900-01-01	9999-12-31	2015-07-10
Diagnosis: ICD10 Code, I00-I99, Define	I00-I99	Diseases of the circulatory system	971043	Malignant hypertensive chronic kidney disease, stage 1-4 or unspecified chronic kidney disease	ICD10CM	1	I12.9		Hypertensive chronic kidney disease with stage 1 throug	IMO	GHS-PACDC	IMO_2015_R	ACTIVE	N	2016-04-10	9999-12-31	2015-07-10
Diagnosis: ICD10 Code, I00-I99, Define	I00-I99	Diseases of the circulatory system	1367838	Secondary hypertension due to glomerulosclerosis, stage 1 through stage 4 or unspecified chro	ICD10CM	1	I12.9		Hypertensive chronic kidney disease with stage 1 throug	IMO	GHS-PACDC	IMO_2018_R	ACTIVE	N	1900-01-01	9999-12-31	2017-10-30
Diagnosis: ICD10 Code, I00-I99, Define	I00-I99	Diseases of the circulatory system	387878	Malignant systolic hypertension with CHF and chronic kidney disease (HCC)	ICD10CM	1	I13.0	I13.0,I50.9	Hypertensive heart and chronic kidney disease with hea	IMO	GHS-PACDC	DX_LEGACY	ACTIVE	N	2016-09-17	2017-10-14	2016-09-30
Diagnosis: ICD10 Code, I00-I99, Define	I00-I99	Diseases of the circulatory system	222547	Malignant hypertensive heart and renal disease with HF and CKD stage I (HCC)	ICD10CM	1	I13.0	I13.0,N18.1,I50.9	Hypertensive heart and chronic kidney disease with hea	IMO	GHS-PACDC	DX_LEGACY	ACTIVE	N	1900-01-01	2016-10-14	2015-07-10
Diagnosis: ICD10 Code, I00-I99, Define	I00-I99	Diseases of the circulatory system	339560	Hypertensive heart and kidney disease with heart failure and with chronic kidney disease stage	ICD10CM	1	I13.0	I13.0,N18.1,I50.9	Hypertensive heart and chronic kidney disease with hea	IMO	GHS-PACDC	DX_LEGACY	ACTIVE	N	1900-01-01	2016-10-14	2015-07-10
Diagnosis: ICD10 Code, I00-I99, Define	I00-I99	Diseases of the circulatory system	357302	Hypertensive heart disease with congestive heart failure and stage 2 kidney disease (HCC)	ICD10CM	1	I13.0	I13.0,N18.2,I50.9	Hypertensive heart and chronic kidney disease with hea	IMO	GHS-PACDC	DX_LEGACY	ACTIVE	N	1900-01-01	9999-12-31	2015-07-10
Diagnosis: ICD10 Code, I00-I99, Define	I00-I99	Diseases of the circulatory system	971444	Hypertensive cardiovascular-renal disease, stage 1-4 or unspecified chronic kidney disease, with	ICD10CM	1	I13.0		Hypertensive heart and chronic kidney disease with hea	IMO	GHS-PACDC	IMO_2015_R	ACTIVE	N	2017-09-17	9999-12-31	2017-10-30
Diagnosis: ICD10 Code, I00-I99, Define	I00-I99	Diseases of the circulatory system	344188	Malignant hypertensive heart failure with stage 1-4 chronic kidney disease requiring acute man	ICD10CM	1	I13.0	I13.0,N18.9,I50.9	Hypertensive heart and chronic kidney disease with hea	IMO	GHS-PACDC	DX_LEGACY	DELETED	Y	1900-01-01	2017-10-14	2015-07-10
Diagnosis: ICD10 Code, I00-I99, Define	I00-I99	Diseases of the circulatory system	599676	Hypertensive heart and chronic kidney disease with heart failure and stage 1 through stage 4 ch	ICD10CM	1	I13.0		Hypertensive heart and chronic kidney disease with hea	IMO	GHS-PACDC	IMO_2015_R	ACTIVE	Y	1900-01-01	9999-12-31	2015-07-10
Diagnosis: ICD10 Code, I00-I99, Define	I00-I99	Diseases of the circulatory system	343341	Malignant hypertensive heart and renal disease with heart failure and chronic kidney disease st	ICD10CM	1	I13.0	I13.0,N18.3	Hypertensive heart and chronic kidney disease with hea	IMO	GHS-PACDC	DX_LEGACY	ACTIVE	N	2017-09-17	9999-12-31	2017-10-30
Diagnosis: ICD10 Code, I00-I99, Define	I00-I99	Diseases of the circulatory system	501900	Benign hypertensive heart and kidney disease with heart failure (HCC)	ICD10CM	1	I13.0	I13.0,I50.9,N18.9	Hypertensive heart and chronic kidney disease with hea	IMO	GHS-PACDC	IMO_2015_R	ACTIVE	N	1900-01-01	2016-10-14	2015-07-10
Diagnosis: ICD10 Code, I00-I99, Define	I00-I99	Diseases of the circulatory system	387875	Malignant systolic hypertension with congestive heart failure and chronic kidney disease (HCC)	ICD10CM	1	I13.0		Hypertensive heart and chronic kidney disease with hea	IMO	GHS-PACDC	DX_LEGACY	ACTIVE	N	2017-09-17	9999-12-31	2017-10-30
Diagnosis: ICD10 Code, I00-I99, Define	I00-I99	Diseases of the circulatory system	971480	Heart disease and kidney failure due to high blood pressure, stage 1-4 or unspecified chronic ki	ICD10CM	1	I13.0	I13.0,N18.3,N18.5,N18.4,N18.2,N	Hypertensive heart and chronic kidney disease with hea	IMO	GHS-PACDC	IMO_2015_R	ACTIVE	N	1900-01-01	2016-05-07	2015-07-10
Diagnosis: ICD10 Code, I00-I99, Define	I00-I99	Diseases of the circulatory system	1041247	Benign hypertensive heart and kidney disease with chronic kidney disease, stage I, with heart fa	ICD10CM	1	I13.0	I13.0,N18.1	Hypertensive heart and chronic kidney disease with hea	IMO	GHS-PACDC	IMO_2015_R	ACTIVE	N	2016-04-10	9999-12-31	2015-07-10
Diagnosis: ICD10 Code, I00-I99, Define	I00-I99	Diseases of the circulatory system	970599	Malignant hypertensive heart and kidney disease, stage 1-4 or unspecified chronic kidney disea	ICD10CM	1	I13.0		Hypertensive heart and chronic kidney disease with hea	IMO	GHS-PACDC	IMO_2015_R	ACTIVE	N	2016-09-17	9999-12-31	2016-09-30
Diagnosis: ICD10 Code, I00-I99, Define	I00-I99	Diseases of the circulatory system	387875	Malignant systolic hypertension with congestive heart failure and chronic kidney disease (HCC)	ICD10CM	1	I13.0	I13.0,I50.9	Hypertensive heart and chronic kidney disease with hea	IMO	GHS-PACDC	DX_LEGACY	ACTIVE	N	2016-09-17	2017-10-14	2016-09-30
Diagnosis: ICD10 Code, I00-I99, Define	I00-I99	Diseases of the circulatory system	491439	Benign hypertensive heart and kidney disease with systolic congestive heart failure, NYHA class	ICD10CM	1	I13.0	I13.0,I50.20,N18.3	Hypertensive heart and chronic kidney disease with hea	IMO	GHS-PACDC	IMO_2015_R	ACTIVE	N	1900-01-01	9999-12-31	2015-07-10
Diagnosis: ICD10 Code, I00-I99, Define	I00-I99	Diseases of the circulatory system	970540	Heart & renal disease, hypertensive malignant, stage 1-4 or unspecified chronic kidney disease,	ICD10CM	1	I13.0	I13.0,I50.9,N18.9	Hypertensive heart and chronic kidney disease with hea	IMO	GHS-PACDC	IMO_2015_R	ACTIVE	N	2016-04-10	2016-10-14	2015-07-10
Diagnosis: ICD10 Code, I00-I99, Define	I00-I99	Diseases of the circulatory system	342956	Benign hypertensive heart and kidney disease with HF and CKD stage I-IV	ICD10CM	1	I13.0		Hypertensive heart and chronic kidney disease with hea	IMO	GHS-PACDC	DX_LEGACY	DELETED	Y	2016-09-17	9999-12-31	2016-09-30
Diagnosis: ICD10 Code, I00-I99, Define	I00-I99	Diseases of the circulatory system	4923	Hypertensive heart failure, benign, with CKD stage 1-4	ICD10CM	1	I13.0		Hypertensive heart and chronic kidney disease with hea	IMO	GHS-PACDC	DX_LEGACY	DELETED	Y	2016-09-17	9999-12-31	2016-09-30
Diagnosis: ICD10 Code, I00-I99, Define	I00-I99	Diseases of the circulatory system	249493	Benign hypertensive heart and kidney disease with HF and CKD (HCC)	ICD10CM	1	I13.0		Hypertensive heart and chronic kidney disease with hea	IMO	GHS-PACDC	DX_LEGACY	ACTIVE	N	2017-09-17	9999-12-31	2017-10-30
Diagnosis: ICD10 Code, I00-I99, Define	I00-I99	Diseases of the circulatory system	971042	Hypertensive heart and kidney disease, malignant, stage 1-4 or unspecified chronic kidney disea	ICD10CM	1	I13.0		Hypertensive heart and chronic kidney disease with hea	IMO	GHS-PACDC	IMO_2015_R	ACTIVE	N	1900-01-01	2016-05-07	2015-07-10
Diagnosis: ICD10 Code, I00-I99, Define	I00-I99	Diseases of the circulatory system	262609	Hypertensive heart failure with chronic kidney disease, stage 1-4	ICD10CM	1	I13.0		Hypertensive heart and chronic kidney disease with hea	IMO	GHS-PACDC	DX_LEGACY	DELETED	Y	2016-09-17	9999-12-31	2016-09-30
Diagnosis: ICD10 Code, I00-I99, Define	I00-I99	Diseases of the circulatory system	1132656	Benign hypertensive heart and kidney disease with NYHA class 3 systolic congestive heart failur	ICD10CM	1	I13.0	I13.0,I50.20,N18.3	Hypertensive heart and chronic kidney disease with hea	IMO	GHS-PACDC	IMO_2016_R	ACTIVE	N	1900-01-01	9999-12-31	2016-06-02
Diagnosis: ICD10 Code, I00-I99, Define	I00-I99	Diseases of the circulatory system	971444	Hypertensive cardiovascular-renal disease, stage 1-4 or unspecified chronic kidney disease, with	ICD10CM	1	I13.0	I13.0,I50.9	Hypertensive heart and chronic kidney disease with hea	IMO	GHS-PACDC	IMO_2015_R	ACTIVE	N	2016-04-10	2017-10-14	2015-07-10
Diagnosis: ICD10 Code, I00-I99, Define	I00-I99	Diseases of the circulatory system	419814	Malignant hypertensive heart and kidney disease with CHF, NYHA class II and CKD stage IV (HCC	ICD10CM	1	I13.0	I13.0,I50.9,N18.4	Hypertensive heart and chronic kidney disease with hea	IMO	GHS-PACDC	DX_LEGACY	ACTIVE	N	1900-01-01	9999-12-31	2015-07-10
Diagnosis: ICD10 Code, I00-I99, Define	I00-I99	Diseases of the circulatory system	384317	Malignant hypertensive kidney and heart disease with CHF, stage IV (HCC)	ICD10CM	1	I13.0	I13.0,N18.4,I50.9	Hypertensive heart and chronic kidney disease with hea	IMO	GHS-PACDC	DX_LEGACY	ACTIVE	N	1900-01-01	2016-10-14	2015-07-10
Diagnosis: ICD10 Code, I00-I99, Define	I00-I99	Diseases of the circulatory system	632690	Pre-existing hypertensive heart and chronic kidney disease complicating pregnancy, first trimest	ICD10CM	2	I13.10	O10.311,I13.10,N18.9	Hypertensive heart and chronic kidney disease without h	IMO	GHS-PACDC	IMO_2015_R	ACTIVE	N	2016-04-10	2017-04-27	2015-07-10
Diagnosis: ICD10 Code, I00-I99, Define	I00-I99	Diseases of the circulatory system	1041642	Hypertensive heart and renal disease complicating or reason for care during pregnancy, unspec	ICD10CM	2	I13.10	O10.319,I13.10,I13.11	Hypertensive heart and chronic kidney disease without h	IMO	GHS-PACDC	IMO_2015_R	ACTIVE	N	1900-01-01	2016-05-07	2015-07-10
Diagnosis: ICD10 Code, I00-I99, Define	I00-I99	Diseases of the circulatory system	361050	Benign hypertensive kidney and heart disease without congestive heart failure, stage III	ICD10CM	1	I13.10	I13.10,N18.3	Hypertensive heart and chronic kidney disease without h	IMO	GHS-PACDC	DX_LEGACY	DELETED	Y	1900-01-01	9999-12-31	2015-07-10
Diagnosis: ICD10 Code, I00-I99, Define	I00-I99	Diseases of the circulatory system	222611	Malignant hypertensive heart and CKD (chronic kidney disease) stage I	ICD10CM	1	I13.10	I13.10,N18.1	Hypertensive heart and chronic kidney disease without h	IMO	GHS-PACDC	DX_LEGACY	ACTIVE	N	1900-01-01	9999-12-31	2015-07-10
Diagnosis: ICD10 Code, I00-I99, Define	I00-I99	Diseases of the circulatory system	1041655	Hypertensive heart and renal disease complicating or reason for care during pregnancy, third tr	ICD10CM	2	I13.10	O10.313,I13.10	Hypertensive heart and chronic kidney disease without h	IMO	GHS-PACDC	IMO_2015_R	ACTIVE	N	2016-04-10	2016-10-14	2015-07-10
Diagnosis: ICD10 Code, I00-I99, Define	I00-I99	Diseases of the circulatory system	1367827	Benign hypertensive heart and kidney disease with chronic kidney disease, stage 1 through stag	ICD10CM	1	I13.10		Hypertensive heart and chronic kidney disease without h	IMO	GHS-PACDC	IMO_2018_R	ACTIVE	N	1900-01-01	9999-12-31	2017-10-30
Diagnosis: ICD10 Code, I00-I99, Define	I00-I99	Diseases of the circulatory system	372796	Malignant hypertensive kidney and heart disease without CHF, stage IV (HCC)	ICD10CM	1	I13.10	I13.10,N18.4	Hypertensive heart and chronic kidney disease without h	IMO	GHS-PACDC	DX_LEGACY	ACTIVE	N	1900-01-01	9999-12-31	2015-07-10
Diagnosis: ICD10 Code, I00-I99, Define	I00-I99	Diseases of the circulatory system	222613	Malignant hypertensive heart and CKD (chronic kidney disease) stage II	ICD10CM	1	I13.10	I13.10,N18.2	Hypertensive heart and chronic kidney disease without h	IMO	GHS-PACDC	DX_LEGACY	ACTIVE	N	1900-01-01	9999-12-31	2015-07-10
Diagnosis: ICD10 Code, I00-I99, Define	I00-I99	Diseases of the circulatory system	1367858	Heart and kidney disease due to severe high blood pressure, stage 1 through stage 4 or unspeci	ICD10CM	1	I13.10		Hypertensive heart and chronic kidney disease without h	IMO	GHS-PACDC	IMO_2018_R	ACTIVE	N	1900-01-01	9999-12-31	2017-10-30

Diagnosis: ICD10 Code, I00-I99, Define	I00-I99	Diseases of the circulatory system	153662	Hypertensive heart and chronic kidney disease	ICD10CM	1	I13.10		Hypertensive heart and chronic kidney disease without h	IMO	GHS-PACDC	DX_LEGACY	ACTIVE	N	1900-01-01	9999-12-31	2015-07-10
Diagnosis: ICD10 Code, I00-I99, Define	I00-I99	Diseases of the circulatory system	632639	Pre-existing hypertensive heart and chronic kidney disease complicating pregnancy, unspecified	ICD10CM	2	I13.10	O10.319,I13.10,N18.9	Hypertensive heart and chronic kidney disease without h	IMO	GHS-PACDC	IMO_2015_R	ACTIVE	N	2016-04-10	2016-10-14	2015-07-10
Diagnosis: ICD10 Code, I00-I99, Define	I00-I99	Diseases of the circulatory system	338934	Malignant hypertensive heart and renal disease without mention of heart failure or renal failure	ICD10CM	1	I13.10		Hypertensive heart and chronic kidney disease without h	IMO	GHS-PACDC	DX_LEGACY	DELETED	Y	1900-01-01	9999-12-31	2015-07-10
Diagnosis: ICD10 Code, I00-I99, Define	I00-I99	Diseases of the circulatory system	1428404	Hypertensive heart and chronic kidney disease without heart failure, with stage 1 through stage	ICD10CM	1	I13.10		Hypertensive heart and chronic kidney disease without h	IMO	GHS-PACDC	IMO_2018_R	ACTIVE	N	1900-01-01	9999-12-31	2017-10-30
Diagnosis: ICD10 Code, I00-I99, Define	I00-I99	Diseases of the circulatory system	531019	Pre-existing hypertensive heart and kidney disease in third trimester, antepartum	ICD10CM	2	I13.10	O10.313,I13.10,N18.9	Hypertensive heart and chronic kidney disease without h	IMO	GHS-PACDC	IMO_2015_R	ACTIVE	N	1900-01-01	2017-04-27	2015-07-10
Diagnosis: ICD10 Code, I00-I99, Define	I00-I99	Diseases of the circulatory system	153678	Benign hypertensive heart and kidney disease without heart failure and with chronic kidney dise	ICD10CM	1	I13.10		Hypertensive heart and chronic kidney disease without h	IMO	GHS-PACDC	DX_LEGACY	ACTIVE	Y	1900-01-01	9999-12-31	2015-07-10
Diagnosis: ICD10 Code, I00-I99, Define	I00-I99	Diseases of the circulatory system	339708	Hypertensive heart and kidney disease without heart failure and with chronic kidney disease sta	ICD10CM	1	I13.10	I13.10,N18.3	Hypertensive heart and chronic kidney disease without h	IMO	GHS-PACDC	DX_LEGACY	ACTIVE	N	1900-01-01	9999-12-31	2015-07-10
Diagnosis: ICD10 Code, I00-I99, Define	I00-I99	Diseases of the circulatory system	165369	Hypertensive heart/kidney disease w/chronic kidney disease stage IV (HCC)	ICD10CM	1	I13.10	I13.10,N18.4	Hypertensive heart and chronic kidney disease without h	IMO	GHS-PACDC	DX_LEGACY	ACTIVE	N	1900-01-01	9999-12-31	2015-07-10
Diagnosis: ICD10 Code, I00-I99, Define	I00-I99	Diseases of the circulatory system	689683	Hypertensive heart and renal disease in obstetric context, second trimester	ICD10CM	2	I13.10	O10.312,I13.10,I13.11	Hypertensive heart and chronic kidney disease without h	IMO	GHS-PACDC	IMO_2015_R	ACTIVE	N	1900-01-01	2016-05-07	2015-07-10
Diagnosis: ICD10 Code, I00-I99, Define	I00-I99	Diseases of the circulatory system	21516	Cardiorenal disease	ICD10CM	1	I13.10		Hypertensive heart and chronic kidney disease without h	IMO	GHS-PACDC	DX_LEGACY	ACTIVE	N	1900-01-01	9999-12-31	2015-07-10
Diagnosis: ICD10 Code, I00-I99, Define	I00-I99	Diseases of the circulatory system	677211	Hypertensive heart and renal disease in obstetric context, third trimester	ICD10CM	2	I13.10	O10.313,I13.10	Hypertensive heart and chronic kidney disease without h	IMO	GHS-PACDC	IMO_2015_R	ACTIVE	N	2016-04-10	2016-10-14	2015-07-10
Diagnosis: ICD10 Code, I00-I99, Define	I00-I99	Diseases of the circulatory system	674744	Hypertensive heart and renal disease in obstetric context, first trimester	ICD10CM	3	I13.11	O10.311,I13.10,I13.11	Hypertensive heart and chronic kidney disease without h	IMO	GHS-PACDC	IMO_2015_R	ACTIVE	N	1900-01-01	2016-05-07	2015-07-10
Diagnosis: ICD10 Code, I00-I99, Define	I00-I99	Diseases of the circulatory system	266073	Hypertensive heart disease, benign, with end stage renal disease (HCC)	ICD10CM	1	I13.11	I13.11,N18.6	Hypertensive heart and chronic kidney disease without h	IMO	GHS-PACDC	DX_LEGACY	ACTIVE	N	1900-01-01	9999-12-31	2015-07-10
Diagnosis: ICD10 Code, I00-I99, Define	I00-I99	Diseases of the circulatory system	1367819	Malignant hypertensive heart and renal disease with renal failure, stage 5 chronic kidney diseas	ICD10CM	1	I13.11		Hypertensive heart and chronic kidney disease without h	IMO	GHS-PACDC	IMO_2018_R	ACTIVE	N	1900-01-01	9999-12-31	2017-10-30
Diagnosis: ICD10 Code, I00-I99, Define	I00-I99	Diseases of the circulatory system	423731	Malignant hypertensive heart and kidney disease without heart failure and with chronic kidney	ICD10CM	1	I13.11	I13.11,N18.5	Hypertensive heart and chronic kidney disease without h	IMO	GHS-PACDC	IMO_2015_R	DELETED	Y	1900-01-01	9999-12-31	2015-07-10
Diagnosis: ICD10 Code, I00-I99, Define	I00-I99	Diseases of the circulatory system	954749	Hypertensive heart and kidney disease, malignant, stage 5 chronic kidney disease or end stage r	ICD10CM	1	I13.11		Hypertensive heart and chronic kidney disease without h	IMO	GHS-PACDC	IMO_2015_R	ACTIVE	N	1900-01-01	9999-12-31	2015-07-10
Diagnosis: ICD10 Code, I00-I99, Define	I00-I99	Diseases of the circulatory system	76518	Malignant hypertensive heart and renal disease with renal failure (HCC)	ICD10CM	1	I13.11	I13.11,N19	Hypertensive heart and chronic kidney disease without h	IMO	GHS-PACDC	DX_LEGACY	ACTIVE	N	1900-01-01	2016-10-14	2015-07-10
Diagnosis: ICD10 Code, I00-I99, Define	I00-I99	Diseases of the circulatory system	423922	Hypertensive heart and renal disease with both (congestive) heart failure and renal failure (HCC	ICD10CM	1	I13.2	I13.2,N19,I50.9	Hypertensive heart and chronic kidney disease with hea	IMO	GHS-PACDC	IMO_2015_R	ACTIVE	N	1900-01-01	9999-12-31	2015-07-10
Diagnosis: ICD10 Code, I00-I99, Define	I00-I99	Diseases of the circulatory system	1041219	Hypertensive heart and renal disease, malignant, with renal failure, with heart failure (HCC)	ICD10CM	1	I13.2		Hypertensive heart and chronic kidney disease with hea	IMO	GHS-PACDC	IMO_2015_R	ACTIVE	N	2016-04-10	9999-12-31	2015-07-10
Diagnosis: ICD10 Code, I00-I99, Define	I00-I99	Diseases of the circulatory system	4930	Hypertensive heart failure with ESRD (HCC)	ICD10CM	1	I13.2	I13.2,N18.6	Hypertensive heart and chronic kidney disease with hea	IMO	GHS-PACDC	DX_LEGACY	ACTIVE	N	2017-09-17	9999-12-31	2017-10-30
Diagnosis: ICD10 Code, I00-I99, Define	I00-I99	Diseases of the circulatory system	4925	Hypertensive heart failure with end-stage renal disease (ESRD) (HCC)	ICD10CM	1	I13.2	I13.2,N18.6	Hypertensive heart and chronic kidney disease with hea	IMO	GHS-PACDC	DX_LEGACY	ACTIVE	N	2017-09-17	9999-12-31	2017-10-30
Diagnosis: ICD10 Code, I00-I99, Define	I00-I99	Diseases of the circulatory system	963668	Hypertensive heart and renal disease, stage 5 chronic kidney disease or end stage renal disease,	ICD10CM	1	I13.2	I13.2,I50.9,N18.5	Hypertensive heart and chronic kidney disease with hea	IMO	GHS-PACDC	IMO_2015_R	ACTIVE	N	2016-04-10	2016-10-14	2015-07-10
Diagnosis: ICD10 Code, I00-I99, Define	I00-I99	Diseases of the circulatory system	357319	Malignant hypertensive heart and end-stage renal disease with congestive heart failure (HCC)	ICD10CM	1	I13.2	I13.2,N18.6,I50.9	Hypertensive heart and chronic kidney disease with hea	IMO	GHS-PACDC	DX_LEGACY	ACTIVE	N	1900-01-01	9999-12-31	2015-07-10
Diagnosis: ICD10 Code, I00-I99, Define	I00-I99	Diseases of the circulatory system	4936	Hypertension, benign renovascular	ICD10CM	1	I15.0		Renovascular hypertension	IMO	GHS-PACDC	DX_LEGACY	ACTIVE	N	1900-01-01	9999-12-31	2015-07-10
Diagnosis: ICD10 Code, I00-I99, Define	I00-I99	Diseases of the circulatory system	109687	Hypertensive renovascular disease	ICD10CM	1	I15.0		Renovascular hypertension	IMO	GHS-PACDC	DX_LEGACY	ACTIVE	N	2016-09-17	9999-12-31	2016-09-30
Diagnosis: ICD10 Code, I00-I99, Define	I00-I99	Diseases of the circulatory system	216722	Hypertension in pregnancy, renal, delivered with postpartum condition	ICD10CM	2	I15.1	O10.43,I15.1	Hypertension secondary to other renal disorders	IMO	GHS-PACDC	DX_LEGACY	ACTIVE	N	1900-01-01	9999-12-31	2015-07-10
Diagnosis: ICD10 Code, I00-I99, Define	I00-I99	Diseases of the circulatory system	684077	Maternal hypertension secondary to renal disease, antepartum, third trimester	ICD10CM	2	I15.1	O10.413,I15.1	Hypertension secondary to other renal disorders	IMO	GHS-PACDC	IMO_2015_R	ACTIVE	N	1900-01-01	9999-12-31	2015-07-10
Diagnosis: ICD10 Code, I00-I99, Define	I00-I99	Diseases of the circulatory system	333277	Hypertension secondary to renal disease complicating or reason for care during childbirth	ICD10CM	2	I15.1	O10.22,I15.1	Hypertension secondary to other renal disorders	IMO	GHS-PACDC	DX_LEGACY	DELETED	Y	1900-01-01	9999-12-31	2015-07-10
Diagnosis: ICD10 Code, I00-I99, Define	I00-I99	Diseases of the circulatory system	264034	Hypertension secondary to renal disease in pregnancy, deliver/postpart	ICD10CM	2	I15.1	O10.43,I15.1	Hypertension secondary to other renal disorders	IMO	GHS-PACDC	DX_LEGACY	ACTIVE	N	1900-01-01	9999-12-31	2015-07-10
Diagnosis: ICD10 Code, I00-I99, Define	I00-I99	Diseases of the circulatory system	372503	Malignant secondary hypertension due to renal disease	ICD10CM	1	I15.1		Hypertension secondary to other renal disorders	IMO	GHS-PACDC	DX_LEGACY	ACTIVE	N	2016-09-17	9999-12-31	2016-09-30
Diagnosis: ICD10 Code, I00-I99, Define	I00-I99	Diseases of the circulatory system	607581	Hypertension secondary to other renal disorders	ICD10CM	1	I15.1		Hypertension secondary to other renal disorders	IMO	GHS-PACDC	IMO_2015_R	ACTIVE	Y	1900-01-01	9999-12-31	2015-07-10
Diagnosis: ICD10 Code, I00-I99, Define	I00-I99	Diseases of the circulatory system	333276	Hypertension secondary to renal disease complicating or reason for care during pregnancy	ICD10CM	2	I15.1	O10.419,I15.1	Hypertension secondary to other renal disorders	IMO	GHS-PACDC	DX_LEGACY	DELETED	Y	1900-01-01	9999-12-31	2015-07-10
Diagnosis: ICD10 Code, I00-I99, Define	I00-I99	Diseases of the circulatory system	1084346	Hypertension secondary to renal disease during pregnancy, antepartum, third trimester	ICD10CM	2	I15.1	O10.413,I15.1	Hypertension secondary to other renal disorders	IMO	GHS-PACDC	IMO_2015_R	ACTIVE	N	1900-01-01	9999-12-31	2015-07-10
Diagnosis: ICD10 Code, I00-I99, Define	I00-I99	Diseases of the circulatory system	233739	High blood pressure due to drug	ICD10CM	2	I15.8	T50.901A,I15.8	Other secondary hypertension	IMO	GHS-PACDC	DX_LEGACY	ACTIVE	N	2016-04-10	2017-10-14	2015-07-10
Diagnosis: ICD10 Code, I00-I99, Define	I00-I99	Diseases of the circulatory system	526886	Pre-existing secondary hypertension affecting pregnancy in third trimester	ICD10CM	2	I15.9	O10.413,I15.9	Secondary hypertension, unspecified	IMO	GHS-PACDC	IMO_2015_R	ACTIVE	N	1900-01-01	2016-05-07	2015-07-10
Diagnosis: ICD10 Code, I00-I99, Define	I00-I99	Diseases of the circulatory system	499851	Benign hypertension secondary to endocrine disorder	ICD10CM	1	I15.9		Secondary hypertension, unspecified	IMO	GHS-PACDC	IMO_2015_R	ACTIVE	N	1900-01-01	2016-10-14	2015-07-10
Diagnosis: ICD10 Code, I00-I99, Define	I00-I99	Diseases of the circulatory system	1131362	Secondary hypertension with goal blood pressure less than 130/85	ICD10CM	1	I15.9		Secondary hypertension, unspecified	IMO	GHS-PACDC	IMO_2016_R	ACTIVE	N	1900-01-01	9999-12-31	2016-06-02
Diagnosis: ICD10 Code, I00-I99, Define	I00-I99	Diseases of the circulatory system	1110430	Pre-existing secondary hypertension during childbirth	ICD10CM	2	I15.9	O10.42,I15.9	Secondary hypertension, unspecified	IMO	GHS-PACDC	IMO_2016_R	ACTIVE	N	1900-01-01	9999-12-31	2016-06-02
Diagnosis: ICD10 Code, I00-I99, Define	I00-I99	Diseases of the circulatory system	255129	Secondary hypertension in child	ICD10CM	1	I15.9		Secondary hypertension, unspecified	IMO	GHS-PACDC	DX_LEGACY	ACTIVE	N	1900-01-01	9999-12-31	2015-07-10
Diagnosis: ICD10 Code, I00-I99, Define	I00-I99	Diseases of the circulatory system	418990	Secondary malignant non-renal hypertension	ICD10CM	1	I15.9		Secondary hypertension, unspecified	IMO	GHS-PACDC	DX_LEGACY	ACTIVE	N	1900-01-01	9999-12-31	2015-07-10
Diagnosis: ICD10 Code, I00-I99, Define	I00-I99	Diseases of the circulatory system	93154	Secondary hypertension, benign	ICD10CM	1	I15.9		Secondary hypertension, unspecified	IMO	GHS-PACDC	DX_LEGACY	ACTIVE	N	1900-01-01	9999-12-31	2015-07-10
Diagnosis: ICD10 Code, I00-I99, Define	I00-I99	Diseases of the circulatory system	351143	Pre-existing secondary hypertension complicating pregnancy in third trimester	ICD10CM	2	I15.9	O10.413,I15.9	Secondary hypertension, unspecified	IMO	GHS-PACDC	DX_LEGACY	ACTIVE	N	1900-01-01	2016-05-07	2015-07-10
Diagnosis: ICD10 Code, I00-I99, Define	I00-I99	Diseases of the circulatory system	490997	Asymptomatic hypertensive urgency	ICD10CM	1	I16.0		Hypertensive urgency	IMO	GHS-PACDC	IMO_2015_R	ACTIVE	N	2016-09-17	9999-12-31	2016-09-30
Diagnosis: ICD10 Code, I00-I99, Define	I00-I99	Diseases of the circulatory system	96290	Angina pectoris, crescendo (HCC)	ICD10CM	1	I20.0		Unstable angina	IMO	GHS-PACDC	DX_LEGACY	ACTIVE	N	1900-01-01	9999-12-31	2015-07-10
Diagnosis: ICD10 Code, I00-I99, Define	I00-I99	Diseases of the circulatory system	412209	Recurrent angina status post coronary stent placement (HCC)	ICD10CM	1	I20.9	I20.9,Z95.1	Angina pectoris, unspecified	IMO	GHS-PACDC	DX_LEGACY	ACTIVE	N	1900-01-01	2017-04-27	2015-07-10
Diagnosis: ICD10 Code, I00-I99, Define	I00-I99	Diseases of the circulatory system	408790	Recurrent angina status post directional coronary atherectomy (HCC)	ICD10CM	1	I20.9	I20.9,Z98.890	Angina pectoris, unspecified	IMO	GHS-PACDC	DX_LEGACY	ACTIVE	N	2016-09-17	9999-12-31	2016-09-30
Diagnosis: ICD10 Code, I00-I99, Define	I00-I99	Diseases of the circulatory system	159469	Class IV angina, pre-operative cardiovascular examination (HCC)	ICD10CM	2	I20.9	Z01.810,I20.9	Angina pectoris, unspecified	IMO	GHS-PACDC	DX_LEGACY	ACTIVE	N	2016-04-10	9999-12-31	2015-07-10
Diagnosis: ICD10 Code, I00-I99, Define	I00-I99	Diseases of the circulatory system	1365591	STEMI involving left main coronary artery (HCC)	ICD10CM	1	I21.01		ST elevation (STEMI) myocardial infarction involving left	IMO	GHS-PACDC	IMO_2018_R	ACTIVE	N	1900-01-01	9999-12-31	2017-10-30
Diagnosis: ICD10 Code, I00-I99, Define	I00-I99	Diseases of the circulatory system	496655	Acute myocardial infarction involving left main coronary artery (HCC)	ICD10CM	1	I21.01		ST elevation (STEMI) myocardial infarction involving left	IMO	GHS-PACDC	IMO_2015_R	ACTIVE	N	1900-01-01	9999-12-31	2015-07-10
Diagnosis: ICD10 Code, I00-I99, Define	I00-I99	Diseases of the circulatory system	275082	ST elevation myocardial infarction involving left main coronary artery (HCC)	ICD10CM	1	I21.01		ST elevation (STEMI) myocardial infarction involving left	IMO	GHS-PACDC	DX_LEGACY	ACTIVE	N	1900-01-01	9999-12-31	2015-07-10
Diagnosis: ICD10 Code, I00-I99, Define	I00-I99	Diseases of the circulatory system	496669	Acute myocardial infarction involving left anterior descending (LAD) coronary artery (HCC)	ICD10CM	1	I21.02		ST elevation (STEMI) myocardial infarction involving left	IMO	GHS-PACDC	IMO_2015_R	ACTIVE	N	1900-01-01	9999-12-31	2015-07-10
Diagnosis: ICD10 Code, I00-I99, Define	I00-I99	Diseases of the circulatory system	1102088	Acute myocardial infarction of other anterior wall, subsequent to initial episode of care	ICD10CM	1	I21.09		ST elevation (STEMI) myocardial infarction involving oth	IMO	GHS-PACDC	IMO_2016_R	DELETED	Y	1900-01-01	9999-12-31	2016-06-02
Diagnosis: ICD10 Code, I00-I99, Define	I00-I99	Diseases of the circulatory system	1365793	STEMI involving oth coronary artery of anterior wall (HCC)	ICD10CM	1	I21.09		ST elevation (STEMI) myocardial infarction involving oth	IMO	GHS-PACDC	IMO_2018_R	ACTIVE	N	1900-01-01	9999-12-31	2017-10-30
Diagnosis: ICD10 Code, I00-I99, Define	I00-I99	Diseases of the circulatory system	257884	Anterolateral myocardial infarction (HCC)	ICD10CM	1	I21.09		ST elevation (STEMI) myocardial infarction involving oth	IMO	GHS-PACDC	DX_LEGACY	ACTIVE	N	1900-01-01	9999-12-31	2015-07-10
Diagnosis: ICD10 Code, I00-I99, Define	I00-I99	Diseases of the circulatory system	202800	Acute transmural anterior wall MI (HCC)	ICD10CM	1	I21.09		ST elevation (STEMI) myocardial infarction involving oth	IMO	GHS-PACDC	DX_LEGACY	ACTIVE	N	1900-01-01	9999-12-31	2015-07-10
Diagnosis: ICD10 Code, I00-I99, Define	I00-I99	Diseases of the circulatory system	423040	Acute myocardial infarction of other anterior wall	ICD10CM	1	I21.09		ST elevation (STEMI) myocardial infarction involving oth	IMO	GHS-PACDC	IMO_2015_R	DELETED	Y	1900-01-01	9999-12-31	2015-07-10
Diagnosis: ICD10 Code, I00-I99, Define	I00-I99	Diseases of the circulatory system	1104380	Anterior ST segment elevation (HCC)	ICD10CM	1	I21.09		ST elevation (STEMI) myocardial infarction involving oth	IMO	GHS-PACDC	IMO_2016_R	ACTIVE	N	1900-01-01	9999-12-31	2016-06-02
Diagnosis: ICD10 Code, I00-I99, Define	I00-I99	Diseases of the circulatory system	377779	Transmural anteroseptal myocardial infarction, initial hospitalization (HCC)	ICD10CM	1	I21.09		ST elevation (STEMI) myocardial infarction involving oth	IMO	GHS-PACDC	DX_LEGACY	ACTIVE	N	1900-01-01	9999-12-31	2015-07-10
Diagnosis: ICD10 Code, I00-I99, Define	I00-I99	Diseases of the circulatory system	15929	Acute anterolateral myocardial infarction (HCC)	ICD10CM	1	I21.09		ST elevation (STEMI) myocardial infarction involving oth	IMO	GHS-PACDC	DX_LEGACY	ACTIVE	N	1900-01-01	9999-12-31	2015-07-10
Diagnosis: ICD10 Code, I00-I99, Define	I00-I99	Diseases of the circulatory system	133082	Additional heart attack (anterolateral wall) (HCC)	ICD10CM	1	I21.09		ST elevation (STEMI) myocardial infarction involving oth	IMO	GHS-PACDC	DX_LEGACY	ACTIVE	N	1900-01-01	9999-12-31	2015-07-10
Diagnosis: ICD10 Code, I00-I99, Define	I00-I99	Diseases of the circulatory system	487402	Acute Q wave myocardial infarction involving right coronary artery (HCC)	ICD10CM	1	I21.11		ST elevation (STEMI) myocardial infarction involving righ	IMO	GHS-PACDC	IMO_2015_R	ACTIVE	N	1900-01-01	9999-12-31	2015-07-10
Diagnosis: ICD10 Code, I00-I99, Define	I00-I99	Diseases of the circulatory system	15934	F/u of acute inferoposterior myocardial infarction (HCC)	ICD10CM	1	I21.11		ST elevation (STEMI) myocardial infarction involving righ	IMO	GHS-PACDC	DX_LEGACY	ACTIVE	N	1900-01-01	2016-05-07	2015-07-10
Diagnosis: ICD10 Code, I00-I99, Define	I00-I99	Diseases of the circulatory system	165965	RCA occlusion (HCC)	ICD10CM	1	I21.11		ST elevation (STEMI) myocardial infarction involving righ	IMO	GHS-PACDC	DX_LEGACY	ACTIVE	N	1900-01-01	9999-12-31	2015-07-10
Diagnosis: ICD10 Code, I00-I99, Define	I00-I99	Diseases of the circulatory system	480145	ST segment elevation myocardial infarction (STEMI) of inferoposterior wall, subsequent to initia	ICD10CM	1	I21.19		ST elevation (STEMI) myocardial infarction involving oth	IMO	GHS-PACDC	IMO_2015_R	ACTIVE	N	2016-09-17	9999-12-31	2016-09-30
Diagnosis: ICD10 Code, I00-I99, Define	I00-I99	Diseases of the circulatory system	479960	Subsequent myocardial infarction of inferior wall within 4 weeks of initial infarction (HCC)	ICD10CM	2	I21.19	I22.1,I21.19	ST elevation (STEMI) myocardial infarction involving oth	IMO	GHS-PACDC	IMO_2015_R	ACTIVE	N	1900-01-01	2016-05-07	2015-07-10
Diagnosis: ICD10 Code, I00-I99, Define	I00-I99	Diseases of the circulatory system	21614	Acute myocardial infarction of inferior wall (HCC)	ICD10CM	1	I21.19		ST elevation (STEMI) myocardial infarction involving oth	IMO	GHS-PACDC	DX_LEGACY	ACTIVE	N	1900-01-01	9999-12-31	2015-07-10
Diagnosis: ICD10 Code, I00-I99, Define	I00-I99	Diseases of the circulatory system	255552	Transmural acute inferoposterior myocardial infarction (HCC)	ICD10CM	1	I21.19		ST elevation (STEMI) myocardial infarction involving oth	IMO	GHS-PACDC	DX_LEGACY	ACTIVE	N	2016-09-17	9999-12-31	2016-09-30
Diagnosis: ICD10 Code, I00-I99, Define	I00-I99	Diseases of the circulatory system	137912	Inferior MI (HCC)	ICD10CM	1	I21.19		ST elevation (STEMI) myocardial infarction involving oth	IMO	GHS-PACDC	DX_LEGACY	ACTIVE	N	1900-01-01	9999-12-31	2015-07-10
Diagnosis: ICD10 Code, I00-I99, Define	I00-I99	Diseases of the circulatory system	4951	Acute MI, inferolateral wall (HCC)	ICD10CM	1	I21.19		ST elevation (STEMI) myocardial infarction involving oth	IMO	GHS-PACDC	DX_LEGACY	ACTIVE	N	1900-01-01	9999-12-31	2015-07-10
Diagnosis: ICD10 Code, I00-I99, Define	I00-I99	Diseases of the circulatory system	343971	Transmural acute inferior myocardial infarction, initial hospitalization (HCC)	ICD10CM	1	I21.19		ST elevation (STEMI) myocardial infarction involving oth	IMO	GHS-PACDC	DX_LEGACY	ACTIVE	N	1900-01-01	9999-12-31	2015-07-10
Diagnosis: ICD10 Code, I00-I99, Define	I00-I99	Diseases of the circulatory system	1185575	ST elevation (STEMI) myocardial infarction involving other coronary artery of inferior wall (HCC)	ICD10CM	1	I21.19		ST elevation (STEMI) myocardial infarction involving oth	IMO	GHS-PACDC	IMO_2016_R	ACTIVE	N	1900-01-01	9999-12-31	2016-06-02
Diagnosis: ICD10 Code, I00-I99, Define	I00-I99	Diseases of the circulatory system	216905	Myocardial infarction, inferoposterior wall, initial care (HCC)	ICD10CM	1	I21.19		ST elevation (STEMI) myocardial infarction involving oth	IMO	GHS-PACDC	DX_LEGACY	ACTIVE	N	2016-04-10	9999-12-31	2015-07-10
Diagnosis: ICD10 Code, I00-I99, Define	I00-I99	Diseases of the circulatory system	613099	St elevation (stemi) myocardial infarction of other sites	ICD10CM	1	I21.2		ST elevation (STEMI) myocardial infarction of other sites	IMO	GHS-PACDC	IMO_2015_R	ACTIVE	Y	1900-01-01	9999-12-31	2015-07-10
Diagnosis: ICD10 Code, I00-I99, Define	I00-I99	Diseases of the circulatory system	1366403	STEMI involving left circumflex coronary artery (HCC)	ICD10CM	1	I21.21		ST elevation (STEMI) myocardial infarction involving left	IMO	GHS-PACDC	IMO_2018_R	ACTIVE	N	1900-01-01	9999-12-31	2017-10-30
Diagnosis: ICD10 Code, I00-I99, Define	I00-I99	Diseases of the circulatory system	487589	Silent ST elevation myocardial infarction (STEMI) involving left circumflex coronary artery (HCC)	ICD10CM	1	I21.21		ST elevation (STEMI) myocardial infarction involving left	IMO	GHS-PACDC	IMO_2015_R	ACTIVE	N	1900-01-01	9999-12-31	2015-07-10
Diagnosis: ICD10 Code, I00-I99, Define	I00-I99	Diseases of the circulatory system	15680	F/u of myocardial infarction of right ventricle (HCC)	ICD10CM	1	I21.29		ST elevation (STEMI) myocardial infarction involving oth	IMO	GHS-PACDC	DX_LEGACY	ACTIVE	N	1900-01-01	9999-12-31	2015-07-10
Diagnosis: ICD10 Code, I00-I99, Define	I00-I99	Diseases of the circulatory system	235626	Myocardial infarction, posterobasal (HCC)	ICD10CM	1	I21.29		ST elevation (STEMI) myocardial infarction involving oth	IMO	GHS-PACDC	DX_LEGACY	ACTIVE	N	1900-01-01	9999-12-31	2015-07-10
Diagnosis: ICD10 Code, I00-I99, Define	I00-I99	Diseases of the circulatory system	405792	RV (right ventricular) mural thrombus with acute MI (HCC)	ICD10CM	1	I21.29		ST elevation (STEMI) myocardial infarction involving oth	IMO	GHS-PACDC	DX_LEGACY	ACTIVE	N	1900-01-01	9999-12-31	2015-07-10
Diagnosis: ICD10 Code, I00-I99, Define	I00-I99	Diseases of the circulatory system	405795	Mural thrombus of right ventricle apex with acute MI (HCC)	ICD10CM	1	I21.29		ST elevation (STEMI) myocardial infarction involving oth	IMO	GHS-PACDC	DX_LEGACY	ACTIVE	N	1900-01-01	9999-12-31	2015-07-10
Diagnosis: ICD10 Code, I00-I99, Define	I00-I99	Diseases of the circulatory system	149358	High lateral infarction (HCC)	ICD10CM	1	I21.29		ST elevation (STEMI) myocardial infarction involving oth	IMO	GHS-PACDC	DX_LEGACY	ACTIVE	N	1900-01-01	9999-12-31	2015-07-10
Diagnosis: ICD10 Code, I00-I99, Define	I00-I99	Diseases of the circulatory system	332053	Myocardial infarction of posterolateral wall (HCC)	ICD10CM	1	I21.29		ST elevation (STEMI) myocardial infarction involving oth	IMO	GHS-PACDC	DX_LEGACY	ACTIVE	N	1900-01-01	9999-12-31	2015-07-10
Diagnosis: ICD10 Code, I00-I99, Define	I00-I99	Diseases of the circulatory system	1127210	Encounter for follow-up of myocardial infarction of right ventricle (HCC)	ICD10CM	1	I21.29		ST elevation (STEMI) myocardial infarction involving oth	IMO	GHS-PACDC	IMO_2016_R	ACTIVE	N	1900-01-01	9999-12-31	2016-06-02
Diagnosis: ICD10 Code, I00-I99, Define	I00-I99	Diseases of the circulatory system	339150	ST elevation myocardial infarction (STEMI) of true posterior wall, subsequent episode of care (H	ICD10CM	1	I21.29		ST elevation (STEMI) myocardial infarction involving oth	IMO	GHS-PACDC	DX_LEGACY	ACTIVE	N	1900-01-01	9999-12-31	2015-07-10
Diagnosis: ICD10 Code, I00-I99, Define	I00-I99	Diseases of the circulatory system	263989	Acute MI, lateral wall (HCC)	ICD10CM	1	I21.29		ST elevation (STEMI) myocardial infarction involving oth	IMO	GHS-PACDC	DX_LEGACY	ACTIVE	N	1900-01-01	9999-12-31	2015-07-10
Diagnosis: ICD10 Code, I00-I99, Define	I00-I99	Diseases of the circulatory system	405745	Mural thrombus of left ventricle following MI (HCC)	ICD10CM	1	I21.29	I21.29,I23.6	ST elevation (STEMI) myocardial infarction involving oth	IMO	GHS-PACDC	DX_LEGACY	ACTIVE	N	2017-09-17	9999-12-31	2017-10-30
Diagnosis: ICD10 Code, I00-I99, Define	I00-I99	Diseases of the circulatory system	1350727	Mural thrombus of left ventricle following acute myocardial infarction (HCC)	ICD10CM	1	I21.29	I21.29,I23.6	ST elevation (STEMI) myocardial infarction involving oth	IMO	GHS-PACDC	IMO_2018_R	ACTIVE	N	1900-01-01	9999-12-31	2017-10-30
Diagnosis: ICD10 Code, I00-I99, Define	I00-I99	Diseases of the circulatory system	15672	Acute lateral myocardial infarction (HCC)	ICD10CM	1	I21.29		ST elevation (STEMI) myocardial infarction involving oth	IMO	GHS-PACDC	DX_LEGACY	ACTIVE	N	1900-01-01	9999-12-31	2015-07-10
Diagnosis: ICD10 Code, I00-I99, Define	I00-I99	Diseases of the circulatory system	405789	Thrombus of right ventricle with acute MI (HCC)	ICD10CM	1	I21.29		ST elevation (STEMI) myocardial infarction involving oth	IMO	GHS-PACDC	DX_LEGACY	ACTIVE	N	1900-01-01	9999-12-31	2015-07-10
Diagnosis: ICD10 Code, I00-I99, Define	I00-I99	Diseases of the circulatory system	1114594	Acute ST elevation myocardial infarction (STEMI) of posterior wall (HCC)	ICD10CM	1	I21.29		ST elevation (STEMI) myocardial infarction involving oth	IMO	GHS-PACDC	IMO_2016_R	ACTIVE	N	1900-01-01	9999-12-31	2016-06-02
Diagnosis: ICD10 Code, I00-I99, Define	I00-I99	Diseases of the circulatory system	405784	Left ventricular thrombosis with acute MI (HCC)	ICD10CM	1	I21.29		ST elevation (STEMI) myocardial infarction involving oth	IMO	GHS-PACDC	DX_LEGACY	ACTIVE	N	1900-01-01	9999-12-31	2015-07-10
Diagnosis: ICD10 Code, I00-I99, Define	I00-I99	Diseases of the circulatory system	405390	Left ventricular apical thrombus following myocardial infarction (HCC)	ICD10CM	1	I21.29	I21.29,I23.6	ST elevation (STEMI) myocardial infarction involving oth	IMO	GHS-PACDC	DX_LEGACY	ACTIVE	N	2017-03-31	9999-12-31	2017-04-14
Diagnosis: ICD10 Code, I00-I99, Define	I00-I99	Diseases of the circulatory system	480135	Acute myocardial infarction of lateral wall, subsequent to initial episode of care (HCC)	ICD10CM	1	I21.29		ST elevation (STEMI) myocardial infarction involving oth	IMO	GHS-PACDC	IMO_2015_R	ACTIVE	N	1900-01-01	9999-12-31	2015-07-10
Diagnosis: ICD10 Code, I00-I99, Define	I00-I99	Diseases of the circulatory system	4967	Acute MI, true posterior wall (HCC)	ICD10CM	1	I21.29		ST elevation (STEMI) myocardial infarction involving oth	IMO	GHS-PACDC	DX_LEGACY	ACTIVE	N	1900-01-01	9999-12-31	2015-07-10
Diagnosis: ICD10 Code, I00-I99, Define	I00-I99	Diseases of the circulatory system	134920	Atrial thrombus following MI (HCC)	ICD10CM	2	I21.3	I23.6,I21.3	ST elevation (STEMI) myocardial infarction of unspecifie	IMO	GHS-PACDC	DX_LEGACY	ACTIVE	N	2016-09-17	9999-12-31	2016-09-30
Diagnosis: ICD10 Code, I00-I99, Define	I00-I99	Diseases of the circulatory system	106366	CT (coronary thrombosis) (HCC)	ICD10CM	1	I21.3		ST elevation (STEMI) myocardial infarction of unspecifie	IMO	GHS-PACDC	DX_LEGACY	ACTIVE	N	1900-01-01	2017-10-14	2015-07-10

Diagnosis: ICD10 Code, I00-I99, Define	I00-I99	Diseases of the circulatory system	1114808	Acute ST elevation myocardial infarction (STEMI) due to occlusion of right coronary artery (HCC	ICD10CM	1	I21.3		ST elevation (STEMI) myocardial infarction of unspecifie	IMO	GHS-PACDC	IMO_2016_R	ACTIVE	N	1900-01-01	9999-12-31	2016-06-02
Diagnosis: ICD10 Code, I00-I99, Define	I00-I99	Diseases of the circulatory system	1352271	Coronary artery embolism with myocardial infarction (HCC)	ICD10CM	1	I21.3		ST elevation (STEMI) myocardial infarction of unspecifie	IMO	GHS-PACDC	IMO_2018_R	ACTIVE	N	1900-01-01	9999-12-31	2017-10-30
Diagnosis: ICD10 Code, I00-I99, Define	I00-I99	Diseases of the circulatory system	91048	Acute myocardial infarction, subsequent episode of care (HCC)	ICD10CM	1	I21.3		ST elevation (STEMI) myocardial infarction of unspecifie	IMO	GHS-PACDC	DX_LEGACY	ACTIVE	N	1900-01-01	2017-10-14	2015-07-10
Diagnosis: ICD10 Code, I00-I99, Define	I00-I99	Diseases of the circulatory system	159322	Pericarditis secondary to acute myocardial infarction (HCC)	ICD10CM	1	I21.3	I21.3,I32	ST elevation (STEMI) myocardial infarction of unspecifie	IMO	GHS-PACDC	DX_LEGACY	ACTIVE	N	1900-01-01	2017-10-14	2015-07-10
Diagnosis: ICD10 Code, I00-I99, Define	I00-I99	Diseases of the circulatory system	175668	LV (left ventricular) mural thrombus following MI (HCC)	ICD10CM	2	I21.3	I51.3,I21.3	ST elevation (STEMI) myocardial infarction of unspecifie	IMO	GHS-PACDC	DX_LEGACY	ACTIVE	N	1900-01-01	2017-10-14	2015-07-10
Diagnosis: ICD10 Code, I00-I99, Define	I00-I99	Diseases of the circulatory system	405391	Left ventricular apical thrombus following MI (HCC)	ICD10CM	2	I21.3	I51.3,I21.3	ST elevation (STEMI) myocardial infarction of unspecifie	IMO	GHS-PACDC	DX_LEGACY	ACTIVE	N	1900-01-01	2017-04-27	2015-07-10
Diagnosis: ICD10 Code, I00-I99, Define	I00-I99	Diseases of the circulatory system	340767	Acute myocardial infarction of right ventricle, subsequent episode of care	ICD10CM	1	I21.3		ST elevation (STEMI) myocardial infarction of unspecifie	IMO	GHS-PACDC	DX_LEGACY	DELETED	Y	2016-09-17	2017-10-14	2016-09-30
Diagnosis: ICD10 Code, I00-I99, Define	I00-I99	Diseases of the circulatory system	405770	Right ventricular apical thrombus following myocardial infarction (HCC)	ICD10CM	2	I21.3	I51.3,I21.3	ST elevation (STEMI) myocardial infarction of unspecifie	IMO	GHS-PACDC	DX_LEGACY	ACTIVE	N	1900-01-01	9999-12-31	2015-07-10
Diagnosis: ICD10 Code, I00-I99, Define	I00-I99	Diseases of the circulatory system	134919	Atrial thrombus	ICD10CM	1	I21.3		ST elevation (STEMI) myocardial infarction of unspecifie	IMO	GHS-PACDC	DX_LEGACY	DELETED	Y	1900-01-01	2017-04-27	2015-07-10
Diagnosis: ICD10 Code, I00-I99, Define	I00-I99	Diseases of the circulatory system	4980	Acute MI, initial	ICD10CM	1	I21.3		ST elevation (STEMI) myocardial infarction of unspecifie	IMO	GHS-PACDC	DX_LEGACY	DELETED	Y	1900-01-01	2017-10-14	2015-07-10
Diagnosis: ICD10 Code, I00-I99, Define	I00-I99	Diseases of the circulatory system	328420	Mural thrombus of left ventricular apex	ICD10CM	1	I21.3		ST elevation (STEMI) myocardial infarction of unspecifie	IMO	GHS-PACDC	DX_LEGACY	DELETED	Y	1900-01-01	2017-04-27	2015-07-10
Diagnosis: ICD10 Code, I00-I99, Define	I00-I99	Diseases of the circulatory system	327560	Left ventricular apical thrombus	ICD10CM	1	I21.3		ST elevation (STEMI) myocardial infarction of unspecifie	IMO	GHS-PACDC	DX_LEGACY	DELETED	Y	1900-01-01	2017-04-27	2015-07-10
Diagnosis: ICD10 Code, I00-I99, Define	I00-I99	Diseases of the circulatory system	266111	Follow-up of acute heart attack (HCC)	ICD10CM	1	I21.3		ST elevation (STEMI) myocardial infarction of unspecifie	IMO	GHS-PACDC	DX_LEGACY	ACTIVE	N	1900-01-01	2017-10-14	2015-07-10
Diagnosis: ICD10 Code, I00-I99, Define	I00-I99	Diseases of the circulatory system	159478	Acute non-ST-elevation MI following previous MI (HCC)	ICD10CM	2	I21.3	I22.9,I21.3	ST elevation (STEMI) myocardial infarction of unspecifie	IMO	GHS-PACDC	DX_LEGACY	ACTIVE	N	1900-01-01	2016-05-07	2015-07-10
Diagnosis: ICD10 Code, I00-I99, Define	I00-I99	Diseases of the circulatory system	200275	Acute subendocardial myocardial infarction of inferior wall (HCC)	ICD10CM	1	I21.4		Non-ST elevation (NSTEMI) myocardial infarction	IMO	GHS-PACDC	DX_LEGACY	ACTIVE	N	1900-01-01	9999-12-31	2015-07-10
Diagnosis: ICD10 Code, I00-I99, Define	I00-I99	Diseases of the circulatory system	379325	Post-hospitalization followup for acute nontransmural myocardial infarction of other site in pric	ICD10CM	1	I21.4		Non-ST elevation (NSTEMI) myocardial infarction	IMO	GHS-PACDC	DX_LEGACY	DELETED	Y	1900-01-01	2016-05-07	2015-07-10
Diagnosis: ICD10 Code, I00-I99, Define	I00-I99	Diseases of the circulatory system	1132422	Acute non-ST elevation myocardial infarction (NSTEMI) (HCC)	ICD10CM	1	I21.4		Non-ST elevation (NSTEMI) myocardial infarction	IMO	GHS-PACDC	IMO_2016_R	ACTIVE	N	1900-01-01	9999-12-31	2016-06-02
Diagnosis: ICD10 Code, I00-I99, Define	I00-I99	Diseases of the circulatory system	325516	Non-Q wave myocardial infarction of inferolateral wall (HCC)	ICD10CM	1	I21.4		Non-ST elevation (NSTEMI) myocardial infarction	IMO	GHS-PACDC	DX_LEGACY	ACTIVE	N	1900-01-01	9999-12-31	2015-07-10
Diagnosis: ICD10 Code, I00-I99, Define	I00-I99	Diseases of the circulatory system	206318	Acute non Q wave MI (myocardial infarction), initial episode of care (HCC)	ICD10CM	1	I21.4		Non-ST elevation (NSTEMI) myocardial infarction	IMO	GHS-PACDC	DX_LEGACY	ACTIVE	N	1900-01-01	9999-12-31	2015-07-10
Diagnosis: ICD10 Code, I00-I99, Define	I00-I99	Diseases of the circulatory system	144000	Acute non-ST-elevation myocardial infarction (HCC)	ICD10CM	1	I21.4		Non-ST elevation (NSTEMI) myocardial infarction	IMO	GHS-PACDC	DX_LEGACY	ACTIVE	N	1900-01-01	9999-12-31	2015-07-10
Diagnosis: ICD10 Code, I00-I99, Define	I00-I99	Diseases of the circulatory system	377678	Nontransmural anteroapical myocardial infarction, initial hospitalization (HCC)	ICD10CM	1	I21.4		Non-ST elevation (NSTEMI) myocardial infarction	IMO	GHS-PACDC	DX_LEGACY	ACTIVE	N	1900-01-01	9999-12-31	2015-07-10
Diagnosis: ICD10 Code, I00-I99, Define	I00-I99	Diseases of the circulatory system	408841	Acute non-Q wave myocardial infarction of lateral wall (HCC)	ICD10CM	1	I21.4		Non-ST elevation (NSTEMI) myocardial infarction	IMO	GHS-PACDC	DX_LEGACY	ACTIVE	N	1900-01-01	9999-12-31	2015-07-10
Diagnosis: ICD10 Code, I00-I99, Define	I00-I99	Diseases of the circulatory system	254522	Nontransmural myocardial infarction, initial hospitalization (HCC)	ICD10CM	1	I21.4		Non-ST elevation (NSTEMI) myocardial infarction	IMO	GHS-PACDC	DX_LEGACY	ACTIVE	N	1900-01-01	9999-12-31	2015-07-10
Diagnosis: ICD10 Code, I00-I99, Define	I00-I99	Diseases of the circulatory system	234627	Demand myocardial infarction (HCC)	ICD10CM	1	I21.9		Acute myocardial infarction, unspecified	IMO	GHS-PACDC	DX_LEGACY	ACTIVE	N	2017-09-17	9999-12-31	2017-10-30
Diagnosis: ICD10 Code, I00-I99, Define	I00-I99	Diseases of the circulatory system	340767	Acute myocardial infarction of right ventricle, subsequent episode of care	ICD10CM	1	I21.9		Acute myocardial infarction, unspecified	IMO	GHS-PACDC	DX_LEGACY	DELETED	Y	2017-09-17	9999-12-31	2017-10-30
Diagnosis: ICD10 Code, I00-I99, Define	I00-I99	Diseases of the circulatory system	479962	Subsequent ST elevation (STEMI) myocardial infarction of anterior wall within 4 weeks of initial	ICD10CM	1	I22.0		Subsequent ST elevation (STEMI) myocardial infarction c	IMO	GHS-PACDC	IMO_2015_R	ACTIVE	N	2016-09-17	9999-12-31	2016-09-30
Diagnosis: ICD10 Code, I00-I99, Define	I00-I99	Diseases of the circulatory system	499447	Acute Q wave myocardial infarction involving left coronary artery (HCC)	ICD10CM	1	I22.0	I22.0,I21.11	Subsequent ST elevation (STEMI) myocardial infarction c	IMO	GHS-PACDC	IMO_2015_R	ACTIVE	N	1900-01-01	2016-05-07	2015-07-10
Diagnosis: ICD10 Code, I00-I99, Define	I00-I99	Diseases of the circulatory system	267192	Subsequent ST elevation (STEMI) myocardial infarction of inferior wall (HCC)	ICD10CM	1	I22.1		Subsequent ST elevation (STEMI) myocardial infarction c	IMO	GHS-PACDC	DX_LEGACY	ACTIVE	N	2016-04-10	9999-12-31	2015-07-10
Diagnosis: ICD10 Code, I00-I99, Define	I00-I99	Diseases of the circulatory system	1192898	Subsequent st elevation (stemi) myocardial infarction of other sites (HCC)	ICD10CM	1	I22.8		Subsequent ST elevation (STEMI) myocardial infarction c	IMO	GHS-PACDC	IMO_2016_R	ACTIVE	N	1900-01-01	9999-12-31	2016-06-02
Diagnosis: ICD10 Code, I00-I99, Define	I00-I99	Diseases of the circulatory system	200356	Additional heart attack (subendocardial) (HCC)	ICD10CM	1	I22.8		Subsequent ST elevation (STEMI) myocardial infarction c	IMO	GHS-PACDC	DX_LEGACY	ACTIVE	N	1900-01-01	9999-12-31	2015-07-10
Diagnosis: ICD10 Code, I00-I99, Define	I00-I99	Diseases of the circulatory system	487595	Subsequent ST elevation myocardial infarction (STEMI) involving left anterior descending (LAD)	ICD10CM	1	I22.9	I22.9,I21.02	Subsequent ST elevation (STEMI) myocardial infarction c	IMO	GHS-PACDC	IMO_2015_R	ACTIVE	N	1900-01-01	2016-05-07	2015-07-10
Diagnosis: ICD10 Code, I00-I99, Define	I00-I99	Diseases of the circulatory system	379352	Acute nontransmural myocardial infarction in prior 8 weeks, subsequent admission after initial	ICD10CM	1	I22.9	I22.9,I21.4	Subsequent ST elevation (STEMI) myocardial infarction c	IMO	GHS-PACDC	DX_LEGACY	DELETED	Y	1900-01-01	2016-05-07	2015-07-10
Diagnosis: ICD10 Code, I00-I99, Define	I00-I99	Diseases of the circulatory system	1365997	Hemopericardium as current complication following AMI (HCC)	ICD10CM	1	I23.0		Hemopericardium as current complication following acu	IMO	GHS-PACDC	IMO_2018_R	ACTIVE	N	1900-01-01	9999-12-31	2017-10-30
Diagnosis: ICD10 Code, I00-I99, Define	I00-I99	Diseases of the circulatory system	1233611	Thrombosis of atrium, auricular appendage, and ventricle as current complications following ac	ICD10CM	1	I23.6		Thrombosis of atrium, auricular appendage, and ventricl	IMO	GHS-PACDC	IMO_2016_R	ACTIVE	N	1900-01-01	9999-12-31	2016-06-02
Diagnosis: ICD10 Code, I00-I99, Define	I00-I99	Diseases of the circulatory system	1365332	Thombos of atrium/auric append/ventr as current comp fol AMI (HCC)	ICD10CM	1	I23.6	I23.6,I21.3	Thrombosis of atrium, auricular appendage, and ventricl	IMO	GHS-PACDC	IMO_2018_R	ACTIVE	N	1900-01-01	9999-12-31	2017-10-30
Diagnosis: ICD10 Code, I00-I99, Define	I00-I99	Diseases of the circulatory system	499682	Post-infarction thrombus of right ventricle (HCC)	ICD10CM	1	I23.6		Thrombosis of atrium, auricular appendage, and ventricl	IMO	GHS-PACDC	IMO_2015_R	ACTIVE	N	1900-01-01	9999-12-31	2015-07-10
Diagnosis: ICD10 Code, I00-I99, Define	I00-I99	Diseases of the circulatory system	1350727	Mural thrombus of left ventricle following acute myocardial infarction (HCC)	ICD10CM	2	I23.6	I21.29,I23.6	Thrombosis of atrium, auricular appendage, and ventricl	IMO	GHS-PACDC	IMO_2018_R	ACTIVE	N	1900-01-01	9999-12-31	2017-10-30
Diagnosis: ICD10 Code, I00-I99, Define	I00-I99	Diseases of the circulatory system	588947	Postinfarction angina	ICD10CM	1	I23.7		Postinfarction angina	IMO	GHS-PACDC	IMO_2015_R	ACTIVE	Y	1900-01-01	9999-12-31	2015-07-10
Diagnosis: ICD10 Code, I00-I99, Define	I00-I99	Diseases of the circulatory system	326595	Myocardial infarction complications (HCC)	ICD10CM	1	I23.8		Other current complications following acute myocardial	IMO	GHS-PACDC	DX_LEGACY	ACTIVE	N	1900-01-01	9999-12-31	2015-07-10
Diagnosis: ICD10 Code, I00-I99, Define	I00-I99	Diseases of the circulatory system	335756	Coronary artery embolism without or not resulting in myocardial infarction (HCC)	ICD10CM	1	I24.0		Acute coronary thrombosis not resulting in myocardial ii	IMO	GHS-PACDC	DX_LEGACY	ACTIVE	N	1900-01-01	9999-12-31	2015-07-10
Diagnosis: ICD10 Code, I00-I99, Define	I00-I99	Diseases of the circulatory system	405763	Ventricular mural thrombus without MI	ICD10CM	1	I24.0		Acute coronary thrombosis not resulting in myocardial ii	IMO	GHS-PACDC	DX_LEGACY	ACTIVE	N	1900-01-01	2016-10-14	2015-07-10
Diagnosis: ICD10 Code, I00-I99, Define	I00-I99	Diseases of the circulatory system	163592	Post-infarction pericarditis (HCC)	ICD10CM	1	I24.1		Dressler's syndrome	IMO	GHS-PACDC	DX_LEGACY	ACTIVE	N	1900-01-01	9999-12-31	2015-07-10
Diagnosis: ICD10 Code, I00-I99, Define	I00-I99	Diseases of the circulatory system	491378	Dressler's syndrome post-CABG (HCC)	ICD10CM	1	I24.1	I24.1,I25.810	Dressler's syndrome	IMO	GHS-PACDC	IMO_2015_R	ACTIVE	N	1900-01-01	9999-12-31	2015-07-10
Diagnosis: ICD10 Code, I00-I99, Define	I00-I99	Diseases of the circulatory system	4987	Other acute and subacute form of ischemic heart disease	ICD10CM	1	I24.8		Other forms of acute ischemic heart disease	IMO	GHS-PACDC	DX_LEGACY	ACTIVE	Y	1900-01-01	9999-12-31	2015-07-10
Diagnosis: ICD10 Code, I00-I99, Define	I00-I99	Diseases of the circulatory system	21609	Microinfarct of heart (HCC)	ICD10CM	1	I24.8		Other forms of acute ischemic heart disease	IMO	GHS-PACDC	DX_LEGACY	ACTIVE	N	1900-01-01	9999-12-31	2015-07-10
Diagnosis: ICD10 Code, I00-I99, Define	I00-I99	Diseases of the circulatory system	1185181	Acute ischemic heart disease, unspecified (HCC)	ICD10CM	1	I24.9		Acute ischemic heart disease, unspecified	IMO	GHS-PACDC	IMO_2016_R	ACTIVE	N	1900-01-01	9999-12-31	2016-06-02
Diagnosis: ICD10 Code, I00-I99, Define	I00-I99	Diseases of the circulatory system	39607	Other cardiovascular diseases of mother, postpartum	ICD10CM	2	I25.10	O99.43,I25.10	Atherosclerotic heart disease of native coronary artery v	IMO	GHS-PACDC	DX_LEGACY	ACTIVE	N	1900-01-01	9999-12-31	2015-07-10
Diagnosis: ICD10 Code, I00-I99, Define	I00-I99	Diseases of the circulatory system	763716	Maternal acquired cardiovascular disease complicating pregnancy, antepartum, third trimester	ICD10CM	2	I25.10	O99.413,I25.10	Atherosclerotic heart disease of native coronary artery v	IMO	GHS-PACDC	IMO_2015_R	ACTIVE	N	1900-01-01	2016-05-07	2015-07-10
Diagnosis: ICD10 Code, I00-I99, Define	I00-I99	Diseases of the circulatory system	39606	Other cardiovascular diseases of mother, antepartum	ICD10CM	2	I25.10	O99.419,I25.10	Atherosclerotic heart disease of native coronary artery v	IMO	GHS-PACDC	DX_LEGACY	ACTIVE	N	1900-01-01	9999-12-31	2015-07-10
Diagnosis: ICD10 Code, I00-I99, Define	I00-I99	Diseases of the circulatory system	962924	Diastolic heart failure secondary to coronary artery disease, acute (HCC)	ICD10CM	2	I25.10	I50.31,I25.10	Atherosclerotic heart disease of native coronary artery v	IMO	GHS-PACDC	IMO_2015_R	ACTIVE	N	1900-01-01	9999-12-31	2015-07-10
Diagnosis: ICD10 Code, I00-I99, Define	I00-I99	Diseases of the circulatory system	716463	Maternal cardiovascular disease complicating pregnancy, antepartum, third trimester	ICD10CM	2	I25.10	O99.413,I25.10	Atherosclerotic heart disease of native coronary artery v	IMO	GHS-PACDC	IMO_2015_R	ACTIVE	N	1900-01-01	2016-05-07	2015-07-10
Diagnosis: ICD10 Code, I00-I99, Define	I00-I99	Diseases of the circulatory system	1079266	Cardiovascular disorder affecting pregnancy, unspecified trimester	ICD10CM	2	I25.10	O99.419,I25.10	Atherosclerotic heart disease of native coronary artery v	IMO	GHS-PACDC	IMO_2015_R	DELETED	Y	1900-01-01	9999-12-31	2015-07-10
Diagnosis: ICD10 Code, I00-I99, Define	I00-I99	Diseases of the circulatory system	357253	Recurrent coronary arteriosclerosis after percutaneous transluminal coronary angioplasty	ICD10CM	1	I25.10	I25.10,Z98.61	Atherosclerotic heart disease of native coronary artery v	IMO	GHS-PACDC	DX_LEGACY	ACTIVE	N	1900-01-01	9999-12-31	2015-07-10
Diagnosis: ICD10 Code, I00-I99, Define	I00-I99	Diseases of the circulatory system	21585	Coronary artery disease	ICD10CM	1	I25.10		Atherosclerotic heart disease of native coronary artery v	IMO	GHS-PACDC	DX_LEGACY	ACTIVE	N	1900-01-01	9999-12-31	2015-07-10
Diagnosis: ICD10 Code, I00-I99, Define	I00-I99	Diseases of the circulatory system	379412	Maternal acquired cardiovascular disease complicating pregnancy, antepartum	ICD10CM	2	I25.10	O99.419,I25.10	Atherosclerotic heart disease of native coronary artery v	IMO	GHS-PACDC	DX_LEGACY	ACTIVE	N	1900-01-01	9999-12-31	2015-07-10
Diagnosis: ICD10 Code, I00-I99, Define	I00-I99	Diseases of the circulatory system	961867	Diastolic heart failure secondary to coronary artery disease, acute on chronic (HCC)	ICD10CM	2	I25.10	I50.33,I25.10	Atherosclerotic heart disease of native coronary artery v	IMO	GHS-PACDC	IMO_2015_R	ACTIVE	N	1900-01-01	9999-12-31	2015-07-10
Diagnosis: ICD10 Code, I00-I99, Define	I00-I99	Diseases of the circulatory system	249768	3-vessel CAD	ICD10CM	1	I25.10		Atherosclerotic heart disease of native coronary artery v	IMO	GHS-PACDC	DX_LEGACY	ACTIVE	N	1900-01-01	9999-12-31	2015-07-10
Diagnosis: ICD10 Code, I00-I99, Define	I00-I99	Diseases of the circulatory system	677782	Cardiovascular disease of mother, antepartum, third trimester	ICD10CM	2	I25.10	O99.413,I25.10	Atherosclerotic heart disease of native coronary artery v	IMO	GHS-PACDC	IMO_2015_R	ACTIVE	N	1900-01-01	9999-12-31	2015-07-10
Diagnosis: ICD10 Code, I00-I99, Define	I00-I99	Diseases of the circulatory system	151444	Coronary artery arteriosclerosis	ICD10CM	1	I25.10		Atherosclerotic heart disease of native coronary artery v	IMO	GHS-PACDC	DX_LEGACY	ACTIVE	N	1900-01-01	9999-12-31	2015-07-10
Diagnosis: ICD10 Code, I00-I99, Define	I00-I99	Diseases of the circulatory system	17932	Heart failure, diastolic, due to CAD (HCC)	ICD10CM	2	I25.10	I50.30,I25.10	Atherosclerotic heart disease of native coronary artery v	IMO	GHS-PACDC	DX_LEGACY	ACTIVE	N	1900-01-01	9999-12-31	2015-07-10
Diagnosis: ICD10 Code, I00-I99, Define	I00-I99	Diseases of the circulatory system	690553	Cardiovascular disease of mother in pregnancy, antepartum, unspecified trimester	ICD10CM	2	I25.10	O99.419,I25.10	Atherosclerotic heart disease of native coronary artery v	IMO	GHS-PACDC	IMO_2015_R	ACTIVE	N	1900-01-01	9999-12-31	2015-07-10
Diagnosis: ICD10 Code, I00-I99, Define	I00-I99	Diseases of the circulatory system	259981	Systolic heart failure secondary to coronary artery disease (HCC)	ICD10CM	2	I25.10	I50.9,I25.10	Atherosclerotic heart disease of native coronary artery v	IMO	GHS-PACDC	DX_LEGACY	ACTIVE	N	1900-01-01	2016-05-07	2015-07-10
Diagnosis: ICD10 Code, I00-I99, Define	I00-I99	Diseases of the circulatory system	104182	Arteriosclerotic heart disease	ICD10CM	1	I25.10		Atherosclerotic heart disease of native coronary artery v	IMO	GHS-PACDC	DX_LEGACY	ACTIVE	N	1900-01-01	9999-12-31	2015-07-10
Diagnosis: ICD10 Code, I00-I99, Define	I00-I99	Diseases of the circulatory system	303031	CAD, multiple vessel	ICD10CM	1	I25.10		Atherosclerotic heart disease of native coronary artery v	IMO	GHS-PACDC	DX_LEGACY	ACTIVE	N	1900-01-01	9999-12-31	2015-07-10
Diagnosis: ICD10 Code, I00-I99, Define	I00-I99	Diseases of the circulatory system	954181	Heart failure, diastolic, due to CAD, unspecified failure chronicity (HCC)	ICD10CM	2	I25.10	I50.30,I25.10	Atherosclerotic heart disease of native coronary artery v	IMO	GHS-PACDC	IMO_2015_R	ACTIVE	N	1900-01-01	9999-12-31	2015-07-10
Diagnosis: ICD10 Code, I00-I99, Define	I00-I99	Diseases of the circulatory system	1239634	Coronary artery disease involving native coronary artery without angina pectoris, unspecified w	ICD10CM	1	I25.10		Atherosclerotic heart disease of native coronary artery v	IMO	GHS-PACDC	IMO_2016_R	ACTIVE	N	1900-01-01	9999-12-31	2016-06-02
Diagnosis: ICD10 Code, I00-I99, Define	I00-I99	Diseases of the circulatory system	357298	Coronary artery disease with history of myocardial infarction without history of CABG	ICD10CM	1	I25.10		Atherosclerotic heart disease of native coronary artery v	IMO	GHS-PACDC	DX_LEGACY	ACTIVE	N	1900-01-01	2016-05-07	2015-07-10
Diagnosis: ICD10 Code, I00-I99, Define	I00-I99	Diseases of the circulatory system	344460	Atherosclerotic heart disease of native coronary artery with unstable angina pectoris (HCC)	ICD10CM	1	I25.110		Atherosclerotic heart disease of native coronary artery v	IMO	GHS-PACDC	DX_LEGACY	ACTIVE	N	1900-01-01	9999-12-31	2015-07-10
Diagnosis: ICD10 Code, I00-I99, Define	I00-I99	Diseases of the circulatory system	1252950	Atherosclerosis of coronary artery with angina pectoris and documented spasm, unspecified ves	ICD10CM	1	I25.111		Atherosclerotic heart disease of native coronary artery v	IMO	GHS-PACDC	IMO_2016_R	ACTIVE	N	1900-01-01	9999-12-31	2016-06-02
Diagnosis: ICD10 Code, I00-I99, Define	I00-I99	Diseases of the circulatory system	592448	Atherosclerotic heart disease of native coronary artery with angina pectoris with documented s	ICD10CM	1	I25.111		Atherosclerotic heart disease of native coronary artery v	IMO	GHS-PACDC	IMO_2015_R	ACTIVE	Y	1900-01-01	9999-12-31	2015-07-10
Diagnosis: ICD10 Code, I00-I99, Define	I00-I99	Diseases of the circulatory system	1236849	Coronary artery disease involving native coronary artery with angina pectoris with documented	ICD10CM	1	I25.111		Atherosclerotic heart disease of native coronary artery v	IMO	GHS-PACDC	IMO_2016_R	ACTIVE	N	1900-01-01	9999-12-31	2016-06-02
Diagnosis: ICD10 Code, I00-I99, Define	I00-I99	Diseases of the circulatory system	1237083	Coronary artery disease involving native coronary artery with other form of angina pectoris, uns	ICD10CM	1	I25.118		Atherosclerotic heart disease of native coronary artery v	IMO	GHS-PACDC	IMO_2016_R	ACTIVE	N	1900-01-01	9999-12-31	2016-06-02
Diagnosis: ICD10 Code, I00-I99, Define	I00-I99	Diseases of the circulatory system	1237285	Coronary artery disease involving native coronary artery with angina pectoris, unspecified whet	ICD10CM	1	I25.119		Atherosclerotic heart disease of native coronary artery v	IMO	GHS-PACDC	IMO_2016_R	ACTIVE	N	1900-01-01	9999-12-31	2016-06-02
Diagnosis: ICD10 Code, I00-I99, Define	I00-I99	Diseases of the circulatory system	1102413	Coronary artery disease with angina pectoris (HCC)	ICD10CM	1	I25.119		Atherosclerotic heart disease of native coronary artery v	IMO	GHS-PACDC	IMO_2016_R	ACTIVE	N	1900-01-01	9999-12-31	2016-06-02
Diagnosis: ICD10 Code, I00-I99, Define	I00-I99	Diseases of the circulatory system	1252363	Atherosclerosis of coronary artery of native heart with angina pectoris, unspecified vessel or les	ICD10CM	1	I25.119		Atherosclerotic heart disease of native coronary artery v	IMO	GHS-PACDC	IMO_2016_R	ACTIVE	N	1900-01-01	9999-12-31	2016-06-02
Diagnosis: ICD10 Code, I00-I99, Define	I00-I99	Diseases of the circulatory system	1221321	Coronary artery disease of native heart with stable angina pectoris, unspecified vessel or lesion	ICD10CM	1	I25.119		Atherosclerotic heart disease of native coronary artery v	IMO	GHS-PACDC	IMO_2016_R	ACTIVE	N	1900-01-01	2016-10-14	2016-06-02
Diagnosis: ICD10 Code, I00-I99, Define	I00-I99	Diseases of the circulatory system	1250880	Atherosclerosis of coronary artery with angina pectoris, unspecified vessel or lesion type, unspe	ICD10CM	1	I25.119		Atherosclerotic heart disease of native coronary artery v	IMO	GHS-PACDC	IMO_2016_R	ACTIVE	N	1900-01-01	9999-12-31	2016-06-02
Diagnosis: ICD10 Code, I00-I99, Define	I00-I99	Diseases of the circulatory system	1126121	Myocardial infarction greater than 8 weeks ago	ICD10CM	1	I25.2		Old myocardial infarction	IMO	GHS-PACDC	IMO_2016_R	ACTIVE	N	1900-01-01	9999-12-31	2016-06-02
Diagnosis: ICD10 Code, I00-I99, Define	I00-I99	Diseases of the circulatory system	490777	History of anteroapical myocardial infarction	ICD10CM	1	I25.2		Old myocardial infarction	IMO	GHS-PACDC	IMO_2015_R	ACTIVE	N	1900-01-01	9999-12-31	2015-07-10
Diagnosis: ICD10 Code, I00-I99, Define	I00-I99	Diseases of the circulatory system	254567	Previous inferior myocardial infarction older than 8 weeks	ICD10CM	1	I25.2		Old myocardial infarction	IMO	GHS-PACDC	DX_LEGACY	ACTIVE	N	1900-01-01	9999-12-31	2015-07-10
Diagnosis: ICD10 Code, I00-I99, Define	I00-I99	Diseases of the circulatory system	480651	Acute myocardial infarct greater than 3 months ago	ICD10CM	1	I25.2		Old myocardial infarction	IMO	GHS-PACDC	IMO_2015_R	ACTIVE	N	1900-01-01	9999-12-31	2015-07-10
Diagnosis: ICD10 Code, I00-I99, Define	I00-I99	Diseases of the circulatory system	323574	H/O acute myocardial infarction of septum	ICD10CM	1	I25.2		Old myocardial infarction	IMO	GHS-PACDC	DX_LEGACY	ACTIVE	N	1900-01-01	9999-12-31	2015-07-10
Diagnosis: ICD10 Code, I00-I99, Define	I00-I99	Diseases of the circulatory system	107399	ECG: old myocardial infarction	ICD10CM	1	I25.2		Old myocardial infarction	IMO	GHS-PACDC	DX_LEGACY	ACTIVE	N	1900-01-01	9999-12-31	2015-07-10
Diagnosis: ICD10 Code, I00-I99, Define	I00-I99	Diseases of the circulatory system	296353	History of acute myocardial infarction of anterolateral wall	ICD10CM	1	I25.2		Old myocardial infarction	IMO	GHS-PACDC	DX_LEGACY	ACTIVE	N	1900-01-01	9999-12-31	2015-07-10
Diagnosis: ICD10 Code, I00-I99, Define	I00-I99	Diseases of the circulatory system	303150	History of ST elevation myocardial infarction	ICD10CM	1	I25.2		Old myocardial infarction	IMO	GHS-PACDC	DX_LEGACY	ACTIVE	N	1900-01-01	9999-12-31	2015-07-10
Diagnosis: ICD10 Code, I00-I99, Define	I00-I99	Diseases of the circulatory system	52667	Mural aneurysm	ICD10CM	1	I25.3		Aneurysm of heart	IMO	GHS-PACDC	DX_LEGACY	ACTIVE	N	1900-01-01	9999-12-31	2015-07-10
Diagnosis: ICD10 Code, I00-I99, Define	I00-I99	Diseases of the circulatory system	1107219	Aneurysm of coronary artery	ICD10CM	1	I25.41		Coronary artery aneurysm	IMO	GHS-PACDC	IMO_2016_R	ACTIVE	N	1900-01-01	9999-12-31	2016-06-02
Diagnosis: ICD10 Code, I00-I99, Define	I00-I99	Diseases of the circulatory system	314183	Coronary artery fistula to right atrium	ICD10CM	1	I25.41		Coronary artery aneurysm	IMO	GHS-PACDC	DX_LEGACY	ACTIVE	N	1900-01-01	9999-12-31	2015-07-10
Diagnosis: ICD10 Code, I00-I99, Define	I00-I99	Diseases of the circulatory system	136052	Coronary artery dissection	ICD10CM	1	I25.42		Coronary artery dissection	IMO	GHS-PACDC	DX_LEGACY	ACTIVE	N	1900-01-01	9999-12-31	2015-07-10
Diagnosis: ICD10 Code, I00-I99, Define	I00-I99	Diseases of the circulatory system	163596	Dissecting aneurysm of coronary artery	ICD10CM	1	I25.42		Coronary artery dissection	IMO	GHS-PACDC	DX_LEGACY	ACTIVE	N	1900-01-01	9999-12-31	2015-07-10
Diagnosis: ICD10 Code, I00-I99, Define	I00-I99	Diseases of the circulatory system	502848	Dissecting aneurysm of left main coronary artery	ICD10CM	1	I25.42		Coronary artery dissection	IMO	GHS-PACDC	IMO_2015_R	ACTIVE	N	1900-01-01	9999-12-31	2015-07-10
Diagnosis: ICD10 Code, I00-I99, Define	I00-I99	Diseases of the circulatory system	1107804	Atherosclerosis of coronary artery bypass graft of native heart with angina pectoris (HCC)	ICD10CM	1	I25.709		Atherosclerosis of coronary artery bypass graft(s), unspe	IMO	GHS-PACDC	IMO_2016_R	ACTIVE	N	1900-01-01	9999-12-31	2016-06-02
Diagnosis: ICD10 Code, I00-I99, Define	I00-I99	Diseases of the circulatory system	1102416	Coronary artery disease involving coronary bypass graft with angina pectoris (HCC)	ICD10CM	1	I25.709		Atherosclerosis of coronary artery bypass graft(s), unspe	IMO	GHS-PACDC	IMO_2016_R	ACTIVE	N	1900-01-01	9999-12-31	2016-06-02
Diagnosis: ICD10 Code, I00-I99, Define	I00-I99	Diseases of the circulatory system	1366525	Athscl autologous vein CABG w unstable angina pectoris (HCC)	ICD10CM	1	I25.710		Atherosclerosis of autologous vein coronary artery bypa	IMO	GHS-PACDC	IMO_2018_R	ACTIVE	N	1900-01-01	9999-12-31	2017-10-30
Diagnosis: ICD10 Code, I00-I99, Define	I00-I99	Diseases of the circulatory system	1102412	Coronary artery disease involving autologous vein coronary bypass graft with angina pectoris (H	ICD10CM	1	I25.719		Atherosclerosis of autologous vein coronary artery bypa	IMO	GHS-PACDC	IMO_2016_R	ACTIVE	N	1900-01-01	9999-12-31	2016-06-02

Diagnosis: ICD10 Code, I00-I99, Define	I00-I99	Diseases of the circulatory system	346153	Atherosclerosis of autologous artery coronary artery bypass graft with unstable angina pectoris	ICD10CM	1	I25.720		Atherosclerosis of autologous artery coronary artery byp	IMO	GHS-PACDC	DX_LEGACY	ACTIVE	N	1900-01-01	9999-12-31	2015-07-10
Diagnosis: ICD10 Code, I00-I99, Define	I00-I99	Diseases of the circulatory system	501873	Coronary artery disease involving autologous artery coronary artery bypass graft with angina pectoris	ICD10CM	1	I25.721		Atherosclerosis of autologous artery coronary artery byp	IMO	GHS-PACDC	IMO_2015_R	ACTIVE	N	1900-01-01	9999-12-31	2015-07-10
Diagnosis: ICD10 Code, I00-I99, Define	I00-I99	Diseases of the circulatory system	356004	Atherosclerosis of autologous artery coronary artery bypass graft with angina pectoris (HCC)	ICD10CM	1	I25.729		Atherosclerosis of autologous artery coronary artery byp	IMO	GHS-PACDC	DX_LEGACY	ACTIVE	N	1900-01-01	9999-12-31	2015-07-10
Diagnosis: ICD10 Code, I00-I99, Define	I00-I99	Diseases of the circulatory system	596629	Atherosclerosis of nonautologous biological coronary artery bypass graft(s) with other forms of	ICD10CM	1	I25.738		Atherosclerosis of nonautologous biological coronary ar	IMO	GHS-PACDC	IMO_2015_R	ACTIVE	Y	1900-01-01	9999-12-31	2015-07-10
Diagnosis: ICD10 Code, I00-I99, Define	I00-I99	Diseases of the circulatory system	352987	Atherosclerosis of nonautologous biological coronary artery bypass graft with angina pectoris (H	ICD10CM	1	I25.739		Atherosclerosis of nonautologous biological coronary ar	IMO	GHS-PACDC	DX_LEGACY	ACTIVE	N	1900-01-01	9999-12-31	2015-07-10
Diagnosis: ICD10 Code, I00-I99, Define	I00-I99	Diseases of the circulatory system	1102410	Coronary artery disease involving nonautologous biological coronary artery bypass graft with angina p	ICD10CM	1	I25.739		Atherosclerosis of nonautologous biological coronary ar	IMO	GHS-PACDC	IMO_2016_R	ACTIVE	N	1900-01-01	9999-12-31	2016-06-02
Diagnosis: ICD10 Code, I00-I99, Define	I00-I99	Diseases of the circulatory system	501882	Coronary artery disease involving native artery of transplanted heart with angina pectoris with (	ICD10CM	1	I25.751		Atherosclerosis of native coronary artery of transplantec	IMO	GHS-PACDC	IMO_2015_R	ACTIVE	N	1900-01-01	9999-12-31	2015-07-10
Diagnosis: ICD10 Code, I00-I99, Define	I00-I99	Diseases of the circulatory system	1041999	Coronary artery disease involving bypass graft of transplanted heart with unspecified angina pe	ICD10CM	1	I25.769		Atherosclerosis of bypass graft of coronary artery of tran	IMO	GHS-PACDC	IMO_2015_R	DELETED	Y	2016-04-10	9999-12-31	2015-07-10
Diagnosis: ICD10 Code, I00-I99, Define	I00-I99	Diseases of the circulatory system	633064	Atherosclerosis of other coronary artery bypass graft with angina pectoris (HCC)	ICD10CM	1	I25.799		Atherosclerosis of other coronary artery bypass graft(s) \	IMO	GHS-PACDC	IMO_2015_R	DELETED	Y	1900-01-01	9999-12-31	2015-07-10
Diagnosis: ICD10 Code, I00-I99, Define	I00-I99	Diseases of the circulatory system	501559	Coronary artery disease involving autologous vein bypass graft	ICD10CM	1	I25.810		Atherosclerosis of coronary artery bypass graft(s) withou	IMO	GHS-PACDC	IMO_2015_R	ACTIVE	N	1900-01-01	9999-12-31	2015-07-10
Diagnosis: ICD10 Code, I00-I99, Define	I00-I99	Diseases of the circulatory system	415705	Arteriosclerosis of coronary artery bypass graft	ICD10CM	1	I25.810		Atherosclerosis of coronary artery bypass graft(s) withou	IMO	GHS-PACDC	DX_LEGACY	ACTIVE	N	1900-01-01	9999-12-31	2015-07-10
Diagnosis: ICD10 Code, I00-I99, Define	I00-I99	Diseases of the circulatory system	600879	Atherosclerosis of coronary artery bypass graft(s) without angina pectoris	ICD10CM	1	I25.810		Atherosclerosis of coronary artery bypass graft(s) withou	IMO	GHS-PACDC	IMO_2015_R	ACTIVE	Y	1900-01-01	9999-12-31	2015-07-10
Diagnosis: ICD10 Code, I00-I99, Define	I00-I99	Diseases of the circulatory system	15283	Coronary atherosclerosis of bypass graft	ICD10CM	1	I25.810		Atherosclerosis of coronary artery bypass graft(s) withou	IMO	GHS-PACDC	DX_LEGACY	ACTIVE	N	1900-01-01	9999-12-31	2015-07-10
Diagnosis: ICD10 Code, I00-I99, Define	I00-I99	Diseases of the circulatory system	1104447	Atherosclerosis of native coronary artery of transplanted heart	ICD10CM	1	I25.811		Atherosclerosis of native coronary artery of transplantec	IMO	GHS-PACDC	IMO_2016_R	ACTIVE	N	1900-01-01	9999-12-31	2016-06-02
Diagnosis: ICD10 Code, I00-I99, Define	I00-I99	Diseases of the circulatory system	1253548	Coronary artery disease involving transplanted heart without angina pectoris, unspecified vesse	ICD10CM	1	I25.811		Atherosclerosis of native coronary artery of transplantec	IMO	GHS-PACDC	IMO_2016_R	ACTIVE	N	1900-01-01	9999-12-31	2016-06-02
Diagnosis: ICD10 Code, I00-I99, Define	I00-I99	Diseases of the circulatory system	17692	Coronary atherosclerosis due to lipid rich plaque	ICD10CM	2	I25.83	I25.10,I25.83	Coronary atherosclerosis due to lipid rich plaque	IMO	GHS-PACDC	DX_LEGACY	ACTIVE	N	2016-04-10	9999-12-31	2015-07-10
Diagnosis: ICD10 Code, I00-I99, Define	I00-I99	Diseases of the circulatory system	419752	Coronary artery calcification	ICD10CM	2	I25.84	I25.10,I25.84	Coronary atherosclerosis due to calcified coronary lesior	IMO	GHS-PACDC	DX_LEGACY	ACTIVE	N	1900-01-01	9999-12-31	2015-07-10
Diagnosis: ICD10 Code, I00-I99, Define	I00-I99	Diseases of the circulatory system	1428183	Other forms of chronic ischemic heart disease (CODE)	ICD10CM	1	I25.89		Other forms of chronic ischemic heart disease	IMO	GHS-PACDC	IMO_2018_R	ACTIVE	N	1900-01-01	9999-12-31	2017-10-30
Diagnosis: ICD10 Code, I00-I99, Define	I00-I99	Diseases of the circulatory system	236667	Ischemic heart disease, chronic	ICD10CM	1	I25.9		Chronic ischemic heart disease, unspecified	IMO	GHS-PACDC	DX_LEGACY	ACTIVE	N	1900-01-01	9999-12-31	2015-07-10
Diagnosis: ICD10 Code, I00-I99, Define	I00-I99	Diseases of the circulatory system	159453	Pre-operative cardiovascular examination, myocardial ischemia	ICD10CM	2	I25.9	Z01.810,I25.9	Chronic ischemic heart disease, unspecified	IMO	GHS-PACDC	DX_LEGACY	ACTIVE	N	2017-09-17	9999-12-31	2017-10-30
Diagnosis: ICD10 Code, I00-I99, Define	I00-I99	Diseases of the circulatory system	487580	Mixed myocardial ischemia and non-ST elevation myocardial infarction (HCC)	ICD10CM	1	I25.9	I25.9,I21.4	Chronic ischemic heart disease, unspecified	IMO	GHS-PACDC	IMO_2015_R	ACTIVE	N	1900-01-01	9999-12-31	2015-07-10
Diagnosis: ICD10 Code, I00-I99, Define	I00-I99	Diseases of the circulatory system	249570	Supply ischemia of myocardium	ICD10CM	1	I25.9		Chronic ischemic heart disease, unspecified	IMO	GHS-PACDC	DX_LEGACY	ACTIVE	N	1900-01-01	9999-12-31	2015-07-10
Diagnosis: ICD10 Code, I00-I99, Define	I00-I99	Diseases of the circulatory system	1107316	Acute septic pulmonary embolus without acute cor pulmonale (HCC)	ICD10CM	1	I26.90		Septic pulmonary embolism without acute cor pulmonal	IMO	GHS-PACDC	IMO_2016_R	ACTIVE	N	1900-01-01	9999-12-31	2016-06-02
Diagnosis: ICD10 Code, I00-I99, Define	I00-I99	Diseases of the circulatory system	610795	Saddle embolus of pulmonary artery without acute cor pulmonale	ICD10CM	1	I26.92		Saddle embolus of pulmonary artery without acute cor p	IMO	GHS-PACDC	IMO_2015_R	ACTIVE	Y	1900-01-01	9999-12-31	2015-07-10
Diagnosis: ICD10 Code, I00-I99, Define	I00-I99	Diseases of the circulatory system	1277297	Iatrogenic pulmonary embolism and infarction, subsequent encounter	ICD10CM	2	I26.99	T81.718D,I26.99	Other pulmonary embolism without acute cor pulmonal	IMO	GHS-PACDC	IMO_2017_R	ACTIVE	N	2017-09-17	9999-12-31	2017-10-30
Diagnosis: ICD10 Code, I00-I99, Define	I00-I99	Diseases of the circulatory system	93237	Pulmonary embolism (HCC)	ICD10CM	1	I26.99		Other pulmonary embolism without acute cor pulmonal	IMO	GHS-PACDC	DX_LEGACY	ACTIVE	N	1900-01-01	9999-12-31	2015-07-10
Diagnosis: ICD10 Code, I00-I99, Define	I00-I99	Diseases of the circulatory system	218882	Pulmonary thromboembolism (HCC)	ICD10CM	1	I26.99		Other pulmonary embolism without acute cor pulmonal	IMO	GHS-PACDC	DX_LEGACY	ACTIVE	N	1900-01-01	9999-12-31	2015-07-10
Diagnosis: ICD10 Code, I00-I99, Define	I00-I99	Diseases of the circulatory system	1259940	Pulmonary embolism during treatment with long-term anticoagulation therapy (HCC)	ICD10CM	1	I26.99	I26.99,Z79.01	Other pulmonary embolism without acute cor pulmonal	IMO	GHS-PACDC	IMO_2017_R	ACTIVE	N	1900-01-01	9999-12-31	2016-09-30
Diagnosis: ICD10 Code, I00-I99, Define	I00-I99	Diseases of the circulatory system	93241	Pulmonary embolus and infarction (HCC)	ICD10CM	1	I26.99		Other pulmonary embolism without acute cor pulmonal	IMO	GHS-PACDC	DX_LEGACY	ACTIVE	N	1900-01-01	9999-12-31	2015-07-10
Diagnosis: ICD10 Code, I00-I99, Define	I00-I99	Diseases of the circulatory system	204200	Recurrent pulmonary embolism (HCC)	ICD10CM	1	I26.99		Other pulmonary embolism without acute cor pulmonal	IMO	GHS-PACDC	DX_LEGACY	ACTIVE	N	1900-01-01	9999-12-31	2015-07-10
Diagnosis: ICD10 Code, I00-I99, Define	I00-I99	Diseases of the circulatory system	21989	Pulmonary apoplexy (HCC)	ICD10CM	1	I26.99		Other pulmonary embolism without acute cor pulmonal	IMO	GHS-PACDC	DX_LEGACY	ACTIVE	N	1900-01-01	9999-12-31	2015-07-10
Diagnosis: ICD10 Code, I00-I99, Define	I00-I99	Diseases of the circulatory system	316270	Right pulmonary artery occlusion (HCC)	ICD10CM	1	I26.99		Other pulmonary embolism without acute cor pulmonal	IMO	GHS-PACDC	DX_LEGACY	ACTIVE	N	1900-01-01	9999-12-31	2015-07-10
Diagnosis: ICD10 Code, I00-I99, Define	I00-I99	Diseases of the circulatory system	229713	Pulmonary embolus, left (HCC)	ICD10CM	1	I26.99		Other pulmonary embolism without acute cor pulmonal	IMO	GHS-PACDC	DX_LEGACY	ACTIVE	N	1900-01-01	9999-12-31	2015-07-10
Diagnosis: ICD10 Code, I00-I99, Define	I00-I99	Diseases of the circulatory system	499746	Occlusion of left pulmonary artery (HCC)	ICD10CM	1	I26.99		Other pulmonary embolism without acute cor pulmonal	IMO	GHS-PACDC	IMO_2015_R	ACTIVE	N	1900-01-01	9999-12-31	2015-07-10
Diagnosis: ICD10 Code, I00-I99, Define	I00-I99	Diseases of the circulatory system	326995	Pulmonary venous thrombosis (HCC)	ICD10CM	1	I26.99		Other pulmonary embolism without acute cor pulmonal	IMO	GHS-PACDC	DX_LEGACY	ACTIVE	N	1900-01-01	9999-12-31	2015-07-10
Diagnosis: ICD10 Code, I00-I99, Define	I00-I99	Diseases of the circulatory system	1259799	Pulmonary embolism on long-term anticoagulation therapy (HCC)	ICD10CM	1	I26.99	I26.99,Z79.01	Other pulmonary embolism without acute cor pulmonal	IMO	GHS-PACDC	IMO_2017_R	ACTIVE	N	1900-01-01	9999-12-31	2016-09-30
Diagnosis: ICD10 Code, I00-I99, Define	I00-I99	Diseases of the circulatory system	506205	BMPR1B-related primary pulmonary hypertension (HCC)	ICD10CM	1	I27.0		Primary pulmonary hypertension	IMO	GHS-PACDC	IMO_2015_R	ACTIVE	N	1900-01-01	9999-12-31	2015-07-10
Diagnosis: ICD10 Code, I00-I99, Define	I00-I99	Diseases of the circulatory system	531972	Primary pulmonary hypertension associated with mutation in BMPR1B gene (HCC)	ICD10CM	1	I27.0		Primary pulmonary hypertension	IMO	GHS-PACDC	IMO_2015_R	ACTIVE	N	1900-01-01	9999-12-31	2015-07-10
Diagnosis: ICD10 Code, I00-I99, Define	I00-I99	Diseases of the circulatory system	533692	Primary pulmonary hypertension	ICD10CM	1	I27.0		Primary pulmonary hypertension	IMO	GHS-PACDC	IMO_2015_R	ACTIVE	Y	1900-01-01	9999-12-31	2015-07-10
Diagnosis: ICD10 Code, I00-I99, Define	I00-I99	Diseases of the circulatory system	5011	Primary pulmonary hypertension (HCC)	ICD10CM	1	I27.0		Primary pulmonary hypertension	IMO	GHS-PACDC	DX_LEGACY	ACTIVE	N	1900-01-01	9999-12-31	2015-07-10
Diagnosis: ICD10 Code, I00-I99, Define	I00-I99	Diseases of the circulatory system	21994	Pulmonary hypertensive venous disease (HCC)	ICD10CM	1	I27.0		Primary pulmonary hypertension	IMO	GHS-PACDC	DX_LEGACY	ACTIVE	N	1900-01-01	2017-10-14	2015-07-10
Diagnosis: ICD10 Code, I00-I99, Define	I00-I99	Diseases of the circulatory system	329733	Persistent pulmonary hypertension (HCC)	ICD10CM	1	I27.2		Other secondary pulmonary hypertension	IMO	GHS-PACDC	DX_LEGACY	ACTIVE	N	2016-04-10	2017-10-14	2015-07-10
Diagnosis: ICD10 Code, I00-I99, Define	I00-I99	Diseases of the circulatory system	302252	Pulmonary hypertension after splenectomy (HCC)	ICD10CM	2	I27.2	I97.89,I27.2	Other secondary pulmonary hypertension	IMO	GHS-PACDC	DX_LEGACY	ACTIVE	N	1900-01-01	2017-10-14	2015-07-10
Diagnosis: ICD10 Code, I00-I99, Define	I00-I99	Diseases of the circulatory system	302263	Pulmonary hypertension associated with mediastinal fibrosis (HCC)	ICD10CM	1	I27.2	I27.2,J98.5	Other secondary pulmonary hypertension	IMO	GHS-PACDC	DX_LEGACY	ACTIVE	N	1900-01-01	2016-10-14	2015-07-10
Diagnosis: ICD10 Code, I00-I99, Define	I00-I99	Diseases of the circulatory system	1348452	Hyperuricemia, pulmonary hypertension, renal failure, and alkalosis (HURPA) syndrome	ICD10CM	3	I27.20	Q87.89,E79.0,I27.20,E87.3,N19	Pulmonary hypertension, unspecified	IMO	GHS-PACDC	IMO_2018_R	ACTIVE	N	1900-01-01	9999-12-31	2017-10-30
Diagnosis: ICD10 Code, I00-I99, Define	I00-I99	Diseases of the circulatory system	159180	PAH (pulmonary artery hypertension) (HCC)	ICD10CM	1	I27.21		Secondary pulmonary arterial hypertension	IMO	GHS-PACDC	DX_LEGACY	ACTIVE	N	2017-09-17	9999-12-31	2017-10-30
Diagnosis: ICD10 Code, I00-I99, Define	I00-I99	Diseases of the circulatory system	1355962	Pulmonary hypertension due to left heart disease	ICD10CM	1	I27.22		Pulmonary hypertension due to left heart disease	IMO	GHS-PACDC	IMO_2018_R	ACTIVE	Y	1900-01-01	9999-12-31	2017-10-30
Diagnosis: ICD10 Code, I00-I99, Define	I00-I99	Diseases of the circulatory system	302659	Pulmonary hypertension due to COPD (HCC)	ICD10CM	1	I27.23	I27.23,J44.9	Pulmonary hypertension due to lung diseases and hypox	IMO	GHS-PACDC	DX_LEGACY	ACTIVE	N	2017-09-17	9999-12-31	2017-10-30
Diagnosis: ICD10 Code, I00-I99, Define	I00-I99	Diseases of the circulatory system	302279	Pulmonary hypertension associated with tumoral obstruction (HCC)	ICD10CM	1	I27.29	I27.29,D49.9	Other secondary pulmonary hypertension	IMO	GHS-PACDC	DX_LEGACY	ACTIVE	N	2017-09-17	9999-12-31	2017-10-30
Diagnosis: ICD10 Code, I00-I99, Define	I00-I99	Diseases of the circulatory system	80199	Cor pulmonale (HCC)	ICD10CM	1	I27.81		Cor pulmonale (chronic)	IMO	GHS-PACDC	DX_LEGACY	ACTIVE	N	2016-04-10	9999-12-31	2015-07-10
Diagnosis: ICD10 Code, I00-I99, Define	I00-I99	Diseases of the circulatory system	1209058	Other chronic pulmonary embolism without acute cor pulmonale (HCC)	ICD10CM	1	I27.82		Chronic pulmonary embolism	IMO	GHS-PACDC	IMO_2016_R	DELETED	Y	1900-01-01	9999-12-31	2016-06-02
Diagnosis: ICD10 Code, I00-I99, Define	I00-I99	Diseases of the circulatory system	23425	Eisenmenger's complex (HCC)	ICD10CM	1	I27.83		Eisenmenger's syndrome	IMO	GHS-PACDC	DX_LEGACY	ACTIVE	N	2017-09-17	9999-12-31	2017-10-30
Diagnosis: ICD10 Code, I00-I99, Define	I00-I99	Diseases of the circulatory system	15792	Eisenmenger's syndrome (HCC)	ICD10CM	1	I27.83		Eisenmenger's syndrome	IMO	GHS-PACDC	DX_LEGACY	ACTIVE	N	2017-09-17	9999-12-31	2017-10-30
Diagnosis: ICD10 Code, I00-I99, Define	I00-I99	Diseases of the circulatory system	159359	Pulmonary hypertension secondary to scleroderma (HCC)	ICD10CM	1	I27.9		Pulmonary heart disease, unspecified	IMO	GHS-PACDC	DX_LEGACY	ACTIVE	N	1900-01-01	9999-12-31	2015-07-10
Diagnosis: ICD10 Code, I00-I99, Define	I00-I99	Diseases of the circulatory system	357183	Pulmonary hypertensive arterial disease associated with schistosomiasis (HCC)	ICD10CM	1	I27.9		Pulmonary heart disease, unspecified	IMO	GHS-PACDC	DX_LEGACY	ACTIVE	N	1900-01-01	9999-12-31	2015-07-10
Diagnosis: ICD10 Code, I00-I99, Define	I00-I99	Diseases of the circulatory system	183601	Pulmonary hypertension secondary to increased PVR (HCC)	ICD10CM	1	I27.9		Pulmonary heart disease, unspecified	IMO	GHS-PACDC	DX_LEGACY	ACTIVE	N	1900-01-01	2016-05-07	2015-07-10
Diagnosis: ICD10 Code, I00-I99, Define	I00-I99	Diseases of the circulatory system	301411	Pulmonary hypertensive arterial disease associated with HIV infection (HCC)	ICD10CM	1	I27.9		Pulmonary heart disease, unspecified	IMO	GHS-PACDC	DX_LEGACY	ACTIVE	N	1900-01-01	9999-12-31	2015-07-10
Diagnosis: ICD10 Code, I00-I99, Define	I00-I99	Diseases of the circulatory system	327697	Left to right cardiac shunt (HCC)	ICD10CM	1	I28.0		Arteriovenous fistula of pulmonary vessels	IMO	GHS-PACDC	DX_LEGACY	ACTIVE	N	1900-01-01	9999-12-31	2015-07-10
Diagnosis: ICD10 Code, I00-I99, Define	I00-I99	Diseases of the circulatory system	19002	Pulmonary arteriovenous malformation	ICD10CM	1	I28.0		Arteriovenous fistula of pulmonary vessels	IMO	GHS-PACDC	DX_LEGACY	ACTIVE	N	1900-01-01	2016-05-07	2015-07-10
Diagnosis: ICD10 Code, I00-I99, Define	I00-I99	Diseases of the circulatory system	113737	Pulmonary vessel stricture (HCC)	ICD10CM	1	I28.8		Other diseases of pulmonary vessels	IMO	GHS-PACDC	DX_LEGACY	ACTIVE	N	1900-01-01	9999-12-31	2015-07-10
Diagnosis: ICD10 Code, I00-I99, Define	I00-I99	Diseases of the circulatory system	91049	Acute/subacute bacterial endocarditis	ICD10CM	1	I28.8		Other diseases of pulmonary vessels	IMO	GHS-PACDC	DX_LEGACY	ACTIVE	N	1900-01-01	2016-05-07	2015-07-10
Diagnosis: ICD10 Code, I00-I99, Define	I00-I99	Diseases of the circulatory system	1216881	Chronic constrictive infectious pericarditis, unspecified infectious etiology	ICD10CM	1	I31.1		Chronic constrictive pericarditis	IMO	GHS-PACDC	IMO_2016_R	ACTIVE	N	1900-01-01	9999-12-31	2016-06-02
Diagnosis: ICD10 Code, I00-I99, Define	I00-I99	Diseases of the circulatory system	494440	Fetal pericardial effusion	ICD10CM	1	I31.3		Pericardial effusion (noninflammatory)	IMO	GHS-PACDC	IMO_2015_R	DELETED	Y	1900-01-01	2016-05-07	2015-07-10
Diagnosis: ICD10 Code, I00-I99, Define	I00-I99	Diseases of the circulatory system	22331	Obliterative pericarditis	ICD10CM	1	I31.8		Other specified diseases of pericardium	IMO	GHS-PACDC	DX_LEGACY	ACTIVE	N	1900-01-01	9999-12-31	2015-07-10
Diagnosis: ICD10 Code, I00-I99, Define	I00-I99	Diseases of the circulatory system	151256	Granular pericarditis	ICD10CM	1	I31.8		Other specified diseases of pericardium	IMO	GHS-PACDC	DX_LEGACY	ACTIVE	N	1900-01-01	9999-12-31	2015-07-10
Diagnosis: ICD10 Code, I00-I99, Define	I00-I99	Diseases of the circulatory system	112866	Pericardial calcification	ICD10CM	1	I31.8		Other specified diseases of pericardium	IMO	GHS-PACDC	DX_LEGACY	ACTIVE	N	1900-01-01	9999-12-31	2015-07-10
Diagnosis: ICD10 Code, I00-I99, Define	I00-I99	Diseases of the circulatory system	303535	Pericardiomyopathy	ICD10CM	1	I31.9		Disease of pericardium, unspecified	IMO	GHS-PACDC	DX_LEGACY	ACTIVE	N	1900-01-01	9999-12-31	2015-07-10
Diagnosis: ICD10 Code, I00-I99, Define	I00-I99	Diseases of the circulatory system	251118	Pericardial mass	ICD10CM	1	I31.9		Disease of pericardium, unspecified	IMO	GHS-PACDC	DX_LEGACY	ACTIVE	N	1900-01-01	9999-12-31	2015-07-10
Diagnosis: ICD10 Code, I00-I99, Define	I00-I99	Diseases of the circulatory system	1080139	Maternal pericarditis affecting pregnancy, first trimester	ICD10CM	2	I31.9	O99.411,I31.9	Disease of pericardium, unspecified	IMO	GHS-PACDC	IMO_2015_R	ACTIVE	N	1900-01-01	9999-12-31	2015-07-10
Diagnosis: ICD10 Code, I00-I99, Define	I00-I99	Diseases of the circulatory system	505665	Maternal pericarditis affecting pregnancy	ICD10CM	2	I31.9	O99.419,I31.9	Disease of pericardium, unspecified	IMO	GHS-PACDC	IMO_2015_R	ACTIVE	N	1900-01-01	9999-12-31	2015-07-10
Diagnosis: ICD10 Code, I00-I99, Define	I00-I99	Diseases of the circulatory system	159232	Pericarditis associated with severe chronic anemia	ICD10CM	2	I32	D64.9,I32	Pericarditis in diseases classified elsewhere	IMO	GHS-PACDC	DX_LEGACY	ACTIVE	N	2016-04-10	9999-12-31	2015-07-10
Diagnosis: ICD10 Code, I00-I99, Define	I00-I99	Diseases of the circulatory system	73381	Lenta endocarditis	ICD10CM	1	I33.0		Acute and subacute infective endocarditis	IMO	GHS-PACDC	DX_LEGACY	ACTIVE	N	2016-04-10	9999-12-31	2015-07-10
Diagnosis: ICD10 Code, I00-I99, Define	I00-I99	Diseases of the circulatory system	405423	Endocarditis due to Staphylococcus epidermidis	ICD10CM	1	I33.0	I33.0,B95.7	Acute and subacute infective endocarditis	IMO	GHS-PACDC	DX_LEGACY	ACTIVE	N	1900-01-01	9999-12-31	2015-07-10
Diagnosis: ICD10 Code, I00-I99, Define	I00-I99	Diseases of the circulatory system	73382	Infective aneurysm (HCC)	ICD10CM	1	I33.0		Acute and subacute infective endocarditis	IMO	GHS-PACDC	DX_LEGACY	ACTIVE	N	1900-01-01	2016-05-07	2015-07-10
Diagnosis: ICD10 Code, I00-I99, Define	I00-I99	Diseases of the circulatory system	67066	Endocarditis parietalis fibroplastica with eosinophilia	ICD10CM	1	I33.0		Acute and subacute infective endocarditis	IMO	GHS-PACDC	DX_LEGACY	ACTIVE	N	1900-01-01	9999-12-31	2015-07-10
Diagnosis: ICD10 Code, I00-I99, Define	I00-I99	Diseases of the circulatory system	262504	Acute endocarditis, non-rheumatic	ICD10CM	1	I33.9		Acute and subacute endocarditis, unspecified	IMO	GHS-PACDC	DX_LEGACY	ACTIVE	N	1900-01-01	9999-12-31	2015-07-10
Diagnosis: ICD10 Code, I00-I99, Define	I00-I99	Diseases of the circulatory system	493586	Abscess of not morphologically mitral left atrioventricular valve	ICD10CM	1	I33.9		Acute and subacute endocarditis, unspecified	IMO	GHS-PACDC	IMO_2015_R	ACTIVE	N	1900-01-01	9999-12-31	2015-07-10
Diagnosis: ICD10 Code, I00-I99, Define	I00-I99	Diseases of the circulatory system	62021	Acute endocarditis	ICD10CM	1	I33.9		Acute and subacute endocarditis, unspecified	IMO	GHS-PACDC	DX_LEGACY	ACTIVE	N	1900-01-01	9999-12-31	2015-07-10
Diagnosis: ICD10 Code, I00-I99, Define	I00-I99	Diseases of the circulatory system	191784	Mitral insufficiency, acute	ICD10CM	1	I34.0		Nonrheumatic mitral (valve) insufficiency	IMO	GHS-PACDC	DX_LEGACY	ACTIVE	N	1900-01-01	9999-12-31	2015-07-10
Diagnosis: ICD10 Code, I00-I99, Define	I00-I99	Diseases of the circulatory system	651101	Maternal mitral regurgitation, third trimester	ICD10CM	2	I34.0	O99.413,I34.0	Nonrheumatic mitral (valve) insufficiency	IMO	GHS-PACDC	IMO_2015_R	DELETED	Y	1900-01-01	9999-12-31	2015-07-10
Diagnosis: ICD10 Code, I00-I99, Define	I00-I99	Diseases of the circulatory system	409199	Acute mitral regurgitation from chordal dysfunction	ICD10CM	1	I34.0		Nonrheumatic mitral (valve) insufficiency	IMO	GHS-PACDC	DX_LEGACY	ACTIVE	N	1900-01-01	9999-12-31	2015-07-10
Diagnosis: ICD10 Code, I00-I99, Define	I00-I99	Diseases of the circulatory system	493796	Cardiac murmur due to mitral valve disorder	ICD10CM	1	I34.0		Nonrheumatic mitral (valve) insufficiency	IMO	GHS-PACDC	IMO_2015_R	ACTIVE	N	1900-01-01	9999-12-31	2015-07-10
Diagnosis: ICD10 Code, I00-I99, Define	I00-I99	Diseases of the circulatory system	123650	Acute mitral regurgitation	ICD10CM	1	I34.0		Nonrheumatic mitral (valve) insufficiency	IMO	GHS-PACDC	DX_LEGACY	ACTIVE	N	1900-01-01	9999-12-31	2015-07-10
Diagnosis: ICD10 Code, I00-I99, Define	I00-I99	Diseases of the circulatory system	191782	Mitral regurgitation, acute	ICD10CM	1	I34.0		Nonrheumatic mitral (valve) insufficiency	IMO	GHS-PACDC	DX_LEGACY	ACTIVE	N	1900-01-01	9999-12-31	2015-07-10
Diagnosis: ICD10 Code, I00-I99, Define	I00-I99	Diseases of the circulatory system	409185	Deficiency of mitral leaflet	ICD10CM	1	I34.0		Nonrheumatic mitral (valve) insufficiency	IMO	GHS-PACDC	DX_LEGACY	ACTIVE	N	1900-01-01	9999-12-31	2015-07-10
Diagnosis: ICD10 Code, I00-I99, Define	I00-I99	Diseases of the circulatory system	324861	Mild mitral insufficiency	ICD10CM	1	I34.0		Nonrheumatic mitral (valve) insufficiency	IMO	GHS-PACDC	DX_LEGACY	ACTIVE	N	1900-01-01	9999-12-31	2015-07-10
Diagnosis: ICD10 Code, I00-I99, Define	I00-I99	Diseases of the circulatory system	430884	Maternal mitral regurgitation complicating pregnancy in second trimester	ICD10CM	2	I34.0	O99.412,I34.0	Nonrheumatic mitral (valve) insufficiency	IMO	GHS-PACDC	IMO_2015_R	ACTIVE	N	1900-01-01	9999-12-31	2015-07-10
Diagnosis: ICD10 Code, I00-I99, Define	I00-I99	Diseases of the circulatory system	697402	Maternal mitral regurgitation complicating pregnancy, second trimester	ICD10CM	2	I34.0	O99.412,I34.0	Nonrheumatic mitral (valve) insufficiency	IMO	GHS-PACDC	IMO_2015_R	ACTIVE	N	1900-01-01	9999-12-31	2015-07-10
Diagnosis: ICD10 Code, I00-I99, Define	I00-I99	Diseases of the circulatory system	1184446	Nonrheumatic mitral (valve) insufficiency	ICD10CM	1	I34.0		Nonrheumatic mitral (valve) insufficiency	IMO	GHS-PACDC	IMO_2016_R	ACTIVE	N	1900-01-01	9999-12-31	2016-06-02
Diagnosis: ICD10 Code, I00-I99, Define	I00-I99	Diseases of the circulatory system	75285	Mitral valvular prolapse syndrome	ICD10CM	1	I34.1		Nonrheumatic mitral (valve) prolapse	IMO	GHS-PACDC	DX_LEGACY	ACTIVE	N	1900-01-01	9999-12-31	2015-07-10
Diagnosis: ICD10 Code, I00-I99, Define	I00-I99	Diseases of the circulatory system	176276	Maternal mitral valve prolapse	ICD10CM	2	I34.1	O99.419,I34.1	Nonrheumatic mitral (valve) prolapse	IMO	GHS-PACDC	DX_LEGACY	DELETED	Y	1900-01-01	2016-10-14	2015-07-10
Diagnosis: ICD10 Code, I00-I99, Define	I00-I99	Diseases of the circulatory system	524868	Mitral valve prolapse in mother affecting pregnancy in second trimester, antepartum	ICD10CM	2	I34.1	O99.412,I34.1	Nonrheumatic mitral (valve) prolapse	IMO	GHS-PACDC	IMO_2015_R	ACTIVE	N	1900-01-01	9999-12-31	2015-07-10
Diagnosis: ICD10 Code, I00-I99, Define	I00-I99	Diseases of the circulatory system	540175	Nonrheumatic mitral (valve) stenosis	ICD10CM	1	I34.2		Nonrheumatic mitral (valve) stenosis	IMO	GHS-PACDC	IMO_2015_R	ACTIVE	Y	1900-01-01	9999-12-31	2015-07-10
Diagnosis: ICD10 Code, I00-I99, Define	I00-I99	Diseases of the circulatory system	408927	Masses on mitral apparatus	ICD10CM	1	I34.8		Other nonrheumatic mitral valve disorders	IMO	GHS-PACDC	DX_LEGACY	ACTIVE	N	1900-01-01	9999-12-31	2015-07-10
Diagnosis: ICD10 Code, I00-I99, Define	I00-I99	Diseases of the circulatory system	408931	Torn mitral leaflet	ICD10CM	1	I34.8		Other nonrheumatic mitral valve disorders	IMO	GHS-PACDC	DX_LEGACY	ACTIVE	N	1900-01-01	9999-12-31	2015-07-10

Diagnosis: ICD10 Code, I00-I99, Define	I00-I99	Diseases of the circulatory system	615164	Nonrheumatic aortic valve disorders	ICD10CM	1	I35		Nonrheumatic aortic valve disorders	IMO	GHS-PACDC	IMO_2015_R	ACTIVE	Y	1900-01-01	9999-12-31	2015-07-10
Diagnosis: ICD10 Code, I00-I99, Define	I00-I99	Diseases of the circulatory system	491566	Aortic stenosis due to bicuspid aortic valve	ICD10CM	2	I35.0	Q23.1,I35.0	Nonrheumatic aortic (valve) stenosis	IMO	GHS-PACDC	IMO_2015_R	ACTIVE	N	1900-01-01	2017-04-27	2015-07-10
Diagnosis: ICD10 Code, I00-I99, Define	I00-I99	Diseases of the circulatory system	249039	Residual aortic stenosis	ICD10CM	1	I35.0		Nonrheumatic aortic (valve) stenosis	IMO	GHS-PACDC	DX_LEGACY	ACTIVE	N	1900-01-01	9999-12-31	2015-07-10
Diagnosis: ICD10 Code, I00-I99, Define	I00-I99	Diseases of the circulatory system	409871	Calcific aortic stenosis of bicuspid valve	ICD10CM	1	I35.0		Nonrheumatic aortic (valve) stenosis	IMO	GHS-PACDC	DX_LEGACY	ACTIVE	N	1900-01-01	9999-12-31	2015-07-10
Diagnosis: ICD10 Code, I00-I99, Define	I00-I99	Diseases of the circulatory system	21889	Nodular calcific aortic valve stenosis	ICD10CM	1	I35.0		Nonrheumatic aortic (valve) stenosis	IMO	GHS-PACDC	DX_LEGACY	ACTIVE	N	1900-01-01	9999-12-31	2015-07-10
Diagnosis: ICD10 Code, I00-I99, Define	I00-I99	Diseases of the circulatory system	505684	Maternal aortic regurgitation affecting pregnancy, antepartum	ICD10CM	2	I35.1	O99.419,I35.1	Nonrheumatic aortic (valve) insufficiency	IMO	GHS-PACDC	IMO_2015_R	ACTIVE	N	1900-01-01	9999-12-31	2015-07-10
Diagnosis: ICD10 Code, I00-I99, Define	I00-I99	Diseases of the circulatory system	597085	Nonrheumatic aortic (valve) insufficiency	ICD10CM	1	I35.1		Nonrheumatic aortic (valve) insufficiency	IMO	GHS-PACDC	IMO_2015_R	ACTIVE	Y	1900-01-01	9999-12-31	2015-07-10
Diagnosis: ICD10 Code, I00-I99, Define	I00-I99	Diseases of the circulatory system	426876	Aortic diastolic murmur on examination	ICD10CM	1	I35.1		Nonrheumatic aortic (valve) insufficiency	IMO	GHS-PACDC	IMO_2015_R	ACTIVE	N	1900-01-01	2016-10-14	2015-07-10
Diagnosis: ICD10 Code, I00-I99, Define	I00-I99	Diseases of the circulatory system	170335	Aortic insufficiency and aortic stenosis	ICD10CM	1	I35.2		Nonrheumatic aortic (valve) stenosis with insufficiency	IMO	GHS-PACDC	DX_LEGACY	DELETED	Y	1900-01-01	9999-12-31	2015-07-10
Diagnosis: ICD10 Code, I00-I99, Define	I00-I99	Diseases of the circulatory system	15724	Aortic valve mass	ICD10CM	1	I35.9		Nonrheumatic aortic valve disorder, unspecified	IMO	GHS-PACDC	DX_LEGACY	ACTIVE	N	1900-01-01	9999-12-31	2015-07-10
Diagnosis: ICD10 Code, I00-I99, Define	I00-I99	Diseases of the circulatory system	1135067	Mild pulmonic stenosis by prior echocardiography	ICD10CM	1	I37.0		Nonrheumatic pulmonary valve stenosis	IMO	GHS-PACDC	IMO_2016_R	ACTIVE	N	1900-01-01	9999-12-31	2016-06-02
Diagnosis: ICD10 Code, I00-I99, Define	I00-I99	Diseases of the circulatory system	75309	Pulmonary valvular stenosis	ICD10CM	1	I37.0		Nonrheumatic pulmonary valve stenosis	IMO	GHS-PACDC	DX_LEGACY	ACTIVE	N	1900-01-01	9999-12-31	2015-07-10
Diagnosis: ICD10 Code, I00-I99, Define	I00-I99	Diseases of the circulatory system	361653	Residual pulmonary stenosis	ICD10CM	1	I37.0		Nonrheumatic pulmonary valve stenosis	IMO	GHS-PACDC	DX_LEGACY	ACTIVE	N	1900-01-01	9999-12-31	2015-07-10
Diagnosis: ICD10 Code, I00-I99, Define	I00-I99	Diseases of the circulatory system	410092	Pulmonary valve stenosis with narrow jet	ICD10CM	1	I37.0		Nonrheumatic pulmonary valve stenosis	IMO	GHS-PACDC	DX_LEGACY	ACTIVE	N	2016-04-10	9999-12-31	2015-07-10
Diagnosis: ICD10 Code, I00-I99, Define	I00-I99	Diseases of the circulatory system	259895	Pulmonary valve insufficiency due to infection	ICD10CM	1	I37.1		Nonrheumatic pulmonary valve insufficiency	IMO	GHS-PACDC	DX_LEGACY	ACTIVE	N	1900-01-01	9999-12-31	2015-07-10
Diagnosis: ICD10 Code, I00-I99, Define	I00-I99	Diseases of the circulatory system	1348592	Physiologic regurgitation of pulmonary valve	ICD10CM	1	I37.1		Nonrheumatic pulmonary valve insufficiency	IMO	GHS-PACDC	IMO_2018_R	ACTIVE	N	1900-01-01	9999-12-31	2017-10-30
Diagnosis: ICD10 Code, I00-I99, Define	I00-I99	Diseases of the circulatory system	301120	Secondary pulmonary valve insufficiency	ICD10CM	1	I37.1		Nonrheumatic pulmonary valve insufficiency	IMO	GHS-PACDC	DX_LEGACY	ACTIVE	N	1900-01-01	9999-12-31	2015-07-10
Diagnosis: ICD10 Code, I00-I99, Define	I00-I99	Diseases of the circulatory system	254142	Mild pulmonary valve insufficiency	ICD10CM	1	I37.1		Nonrheumatic pulmonary valve insufficiency	IMO	GHS-PACDC	DX_LEGACY	ACTIVE	N	1900-01-01	9999-12-31	2015-07-10
Diagnosis: ICD10 Code, I00-I99, Define	I00-I99	Diseases of the circulatory system	1216780	Pulmonary valve stenosis with insufficiency, unspecified etiology	ICD10CM	1	I37.2		Nonrheumatic pulmonary valve stenosis with insufficien	IMO	GHS-PACDC	IMO_2016_R	ACTIVE	N	1900-01-01	9999-12-31	2016-06-02
Diagnosis: ICD10 Code, I00-I99, Define	I00-I99	Diseases of the circulatory system	587908	Nonrheumatic pulmonary valve stenosis with insufficiency	ICD10CM	1	I37.2		Nonrheumatic pulmonary valve stenosis with insufficien	IMO	GHS-PACDC	IMO_2015_R	ACTIVE	Y	1900-01-01	9999-12-31	2015-07-10
Diagnosis: ICD10 Code, I00-I99, Define	I00-I99	Diseases of the circulatory system	194158	Pulmonary valve stenosis with insufficiency	ICD10CM	1	I37.2		Nonrheumatic pulmonary valve stenosis with insufficien	IMO	GHS-PACDC	DX_LEGACY	ACTIVE	N	1900-01-01	9999-12-31	2015-07-10
Diagnosis: ICD10 Code, I00-I99, Define	I00-I99	Diseases of the circulatory system	408846	Pulmonary valve prolapse	ICD10CM	1	I37.8		Other nonrheumatic pulmonary valve disorders	IMO	GHS-PACDC	DX_LEGACY	ACTIVE	N	1900-01-01	9999-12-31	2015-07-10
Diagnosis: ICD10 Code, I00-I99, Define	I00-I99	Diseases of the circulatory system	249042	Unicommissural pulmonic valve	ICD10CM	1	I37.8		Other nonrheumatic pulmonary valve disorders	IMO	GHS-PACDC	DX_LEGACY	ACTIVE	N	1900-01-01	9999-12-31	2015-07-10
Diagnosis: ICD10 Code, I00-I99, Define	I00-I99	Diseases of the circulatory system	270118	Nonrheumatic pulmonary valve disorder	ICD10CM	1	I37.9		Nonrheumatic pulmonary valve disorder, unspecified	IMO	GHS-PACDC	DX_LEGACY	ACTIVE	N	1900-01-01	9999-12-31	2015-07-10
Diagnosis: ICD10 Code, I00-I99, Define	I00-I99	Diseases of the circulatory system	1079851	Maternal endocarditis affecting pregnancy, antepartum, first trimester	ICD10CM	2	I38	O99.411,I38	Endocarditis, valve unspecified	IMO	GHS-PACDC	IMO_2015_R	ACTIVE	N	1900-01-01	9999-12-31	2015-07-10
Diagnosis: ICD10 Code, I00-I99, Define	I00-I99	Diseases of the circulatory system	704221	Maternal endocarditis, unspecified trimester	ICD10CM	2	I38	O99.419,I38	Endocarditis, valve unspecified	IMO	GHS-PACDC	IMO_2015_R	ACTIVE	N	1900-01-01	9999-12-31	2015-07-10
Diagnosis: ICD10 Code, I00-I99, Define	I00-I99	Diseases of the circulatory system	408853	Disorder of endocardium and heart valve	ICD10CM	1	I38		Endocarditis, valve unspecified	IMO	GHS-PACDC	DX_LEGACY	ACTIVE	N	1900-01-01	9999-12-31	2015-07-10
Diagnosis: ICD10 Code, I00-I99, Define	I00-I99	Diseases of the circulatory system	962005	CHF due to valvular disease, unspecified failure chronicity, combined	ICD10CM	2	I38	I50.40,I38	Endocarditis, valve unspecified	IMO	GHS-PACDC	IMO_2015_R	DELETED	Y	1900-01-01	9999-12-31	2015-07-10
Diagnosis: ICD10 Code, I00-I99, Define	I00-I99	Diseases of the circulatory system	58102	Valvular regurgitation	ICD10CM	1	I38		Endocarditis, valve unspecified	IMO	GHS-PACDC	DX_LEGACY	ACTIVE	N	1900-01-01	9999-12-31	2015-07-10
Diagnosis: ICD10 Code, I00-I99, Define	I00-I99	Diseases of the circulatory system	46694	Endocarditis	ICD10CM	1	I38		Endocarditis, valve unspecified	IMO	GHS-PACDC	DX_LEGACY	ACTIVE	N	1900-01-01	9999-12-31	2015-07-10
Diagnosis: ICD10 Code, I00-I99, Define	I00-I99	Diseases of the circulatory system	955192	Heart failure due to valvular disease, unspecified failure chronicity, systolic (HCC)	ICD10CM	2	I38	I50.20,I38	Endocarditis, valve unspecified	IMO	GHS-PACDC	IMO_2015_R	ACTIVE	N	1900-01-01	9999-12-31	2015-07-10
Diagnosis: ICD10 Code, I00-I99, Define	I00-I99	Diseases of the circulatory system	519604	Acute on chronic diastolic congestive heart failure due to valvular disease (HCC)	ICD10CM	2	I38	I50.33,I38	Endocarditis, valve unspecified	IMO	GHS-PACDC	IMO_2015_R	ACTIVE	N	1900-01-01	9999-12-31	2015-07-10
Diagnosis: ICD10 Code, I00-I99, Define	I00-I99	Diseases of the circulatory system	431321	Maternal endocarditis complicating pregnancy in third trimester	ICD10CM	2	I38	O99.413,I38	Endocarditis, valve unspecified	IMO	GHS-PACDC	IMO_2015_R	ACTIVE	N	1900-01-01	9999-12-31	2015-07-10
Diagnosis: ICD10 Code, I00-I99, Define	I00-I99	Diseases of the circulatory system	364774	Common atrioventricular valve insufficiency	ICD10CM	1	I38		Endocarditis, valve unspecified	IMO	GHS-PACDC	DX_LEGACY	ACTIVE	N	1900-01-01	9999-12-31	2015-07-10
Diagnosis: ICD10 Code, I00-I99, Define	I00-I99	Diseases of the circulatory system	99193	Histoplasma capsulatum with endocarditis	ICD10CM	2	I39	B39.4,I39	Endocarditis and heart valve disorders in diseases classif	IMO	GHS-PACDC	DX_LEGACY	ACTIVE	N	2016-04-10	9999-12-31	2015-07-10
Diagnosis: ICD10 Code, I00-I99, Define	I00-I99	Diseases of the circulatory system	193067	Septic myocarditis due to Streptococcus pneumoniae	ICD10CM	1	I40.0	I40.0,B95.3	Infective myocarditis	IMO	GHS-PACDC	DX_LEGACY	ACTIVE	N	1900-01-01	9999-12-31	2015-07-10
Diagnosis: ICD10 Code, I00-I99, Define	I00-I99	Diseases of the circulatory system	35811	Bacterial myocarditis	ICD10CM	1	I40.0		Infective myocarditis	IMO	GHS-PACDC	DX_LEGACY	ACTIVE	N	1900-01-01	9999-12-31	2015-07-10
Diagnosis: ICD10 Code, I00-I99, Define	I00-I99	Diseases of the circulatory system	262505	Acute infective myocarditis	ICD10CM	1	I40.0		Infective myocarditis	IMO	GHS-PACDC	DX_LEGACY	ACTIVE	N	1900-01-01	9999-12-31	2015-07-10
Diagnosis: ICD10 Code, I00-I99, Define	I00-I99	Diseases of the circulatory system	193065	Septic myocarditis due to Staphylococcus	ICD10CM	1	I40.0	I40.0,B95.8	Infective myocarditis	IMO	GHS-PACDC	DX_LEGACY	ACTIVE	N	1900-01-01	9999-12-31	2015-07-10
Diagnosis: ICD10 Code, I00-I99, Define	I00-I99	Diseases of the circulatory system	1111067	Acute idiopathic myocarditis	ICD10CM	1	I40.1		Isolated myocarditis	IMO	GHS-PACDC	IMO_2016_R	ACTIVE	N	1900-01-01	9999-12-31	2016-06-02
Diagnosis: ICD10 Code, I00-I99, Define	I00-I99	Diseases of the circulatory system	73387	Acute or subacute (interstitial) myocarditis	ICD10CM	1	I40.9		Acute myocarditis, unspecified	IMO	GHS-PACDC	DX_LEGACY	ACTIVE	N	1900-01-01	9999-12-31	2015-07-10
Diagnosis: ICD10 Code, I00-I99, Define	I00-I99	Diseases of the circulatory system	524782	X-linked dilated cardiomyopathy type 3B (HCC)	ICD10CM	1	I42.0		Dilated cardiomyopathy	IMO	GHS-PACDC	IMO_2015_R	ACTIVE	N	1900-01-01	9999-12-31	2015-07-10
Diagnosis: ICD10 Code, I00-I99, Define	I00-I99	Diseases of the circulatory system	531145	Autosomal dominant dilated cardiomyopathy associated with mutation in TMPO gene (HCC)	ICD10CM	1	I42.0	I42.0,Z15.89	Dilated cardiomyopathy	IMO	GHS-PACDC	IMO_2015_R	ACTIVE	N	1900-01-01	9999-12-31	2015-07-10
Diagnosis: ICD10 Code, I00-I99, Define	I00-I99	Diseases of the circulatory system	106147	Congestive dilated cardiomyopathy (HCC)	ICD10CM	1	I42.0		Dilated cardiomyopathy	IMO	GHS-PACDC	DX_LEGACY	ACTIVE	N	1900-01-01	9999-12-31	2015-07-10
Diagnosis: ICD10 Code, I00-I99, Define	I00-I99	Diseases of the circulatory system	44349	Cardiomyopathy, dilated (HCC)	ICD10CM	1	I42.0		Dilated cardiomyopathy	IMO	GHS-PACDC	DX_LEGACY	ACTIVE	N	1900-01-01	9999-12-31	2015-07-10
Diagnosis: ICD10 Code, I00-I99, Define	I00-I99	Diseases of the circulatory system	21787	Primary idiopathic dilated cardiomyopathy (HCC)	ICD10CM	1	I42.0		Dilated cardiomyopathy	IMO	GHS-PACDC	DX_LEGACY	ACTIVE	N	1900-01-01	9999-12-31	2015-07-10
Diagnosis: ICD10 Code, I00-I99, Define	I00-I99	Diseases of the circulatory system	524509	Autosomal recessive dilated cardiomyopathy associated with mutation in TNNI3 gene (HCC)	ICD10CM	1	I42.0		Dilated cardiomyopathy	IMO	GHS-PACDC	IMO_2015_R	ACTIVE	N	1900-01-01	9999-12-31	2015-07-10
Diagnosis: ICD10 Code, I00-I99, Define	I00-I99	Diseases of the circulatory system	505819	Dilated cardiomyopathy-1I associated with mutation in DES gene (HCC)	ICD10CM	1	I42.0	I42.0,Z15.89	Dilated cardiomyopathy	IMO	GHS-PACDC	IMO_2015_R	ACTIVE	N	1900-01-01	9999-12-31	2015-07-10
Diagnosis: ICD10 Code, I00-I99, Define	I00-I99	Diseases of the circulatory system	510102	Autosomal dominant dilated cardiomyopathy type 1R associated with mutation in ACTC1 gene (	ICD10CM	1	I42.0	I42.0,Z15.89	Dilated cardiomyopathy	IMO	GHS-PACDC	IMO_2015_R	ACTIVE	N	1900-01-01	9999-12-31	2015-07-10
Diagnosis: ICD10 Code, I00-I99, Define	I00-I99	Diseases of the circulatory system	344109	Heart failure, diastolic, due to hypertrophic obstructive cardiomyopathy (HCC)	ICD10CM	2	I42.1	I50.30,I42.1	Obstructive hypertrophic cardiomyopathy	IMO	GHS-PACDC	DX_LEGACY	ACTIVE	N	1900-01-01	9999-12-31	2015-07-10
Diagnosis: ICD10 Code, I00-I99, Define	I00-I99	Diseases of the circulatory system	306539	Muscular subaortic stenosis (HCC)	ICD10CM	1	I42.1		Obstructive hypertrophic cardiomyopathy	IMO	GHS-PACDC	DX_LEGACY	ACTIVE	N	1900-01-01	9999-12-31	2015-07-10
Diagnosis: ICD10 Code, I00-I99, Define	I00-I99	Diseases of the circulatory system	21841	Primary familial hypertrophic cardiomyopathy (HCC)	ICD10CM	1	I42.2		Other hypertrophic cardiomyopathy	IMO	GHS-PACDC	DX_LEGACY	ACTIVE	N	1900-01-01	9999-12-31	2015-07-10
Diagnosis: ICD10 Code, I00-I99, Define	I00-I99	Diseases of the circulatory system	266136	Apical variant hypertrophic cardiomyopathy (HCC)	ICD10CM	1	I42.2		Other hypertrophic cardiomyopathy	IMO	GHS-PACDC	DX_LEGACY	ACTIVE	N	1900-01-01	9999-12-31	2015-07-10
Diagnosis: ICD10 Code, I00-I99, Define	I00-I99	Diseases of the circulatory system	427199	Hypertensive hypertrophic cardiomyopathy, with heart failure (HCC)	ICD10CM	2	I42.2	I11.0,I42.2	Other hypertrophic cardiomyopathy	IMO	GHS-PACDC	IMO_2015_R	ACTIVE	N	1900-01-01	9999-12-31	2015-07-10
Diagnosis: ICD10 Code, I00-I99, Define	I00-I99	Diseases of the circulatory system	259980	Diastolic heart failure secondary to hypertrophic cardiomyopathy (HCC)	ICD10CM	2	I42.2	I50.30,I42.2	Other hypertrophic cardiomyopathy	IMO	GHS-PACDC	DX_LEGACY	ACTIVE	N	2016-04-10	9999-12-31	2015-07-10
Diagnosis: ICD10 Code, I00-I99, Define	I00-I99	Diseases of the circulatory system	259998	Systolic heart failure secondary to hypertrophic cardiomyopathy (HCC)	ICD10CM	2	I42.2	I50.20,I42.2	Other hypertrophic cardiomyopathy	IMO	GHS-PACDC	DX_LEGACY	ACTIVE	N	2016-04-10	9999-12-31	2015-07-10
Diagnosis: ICD10 Code, I00-I99, Define	I00-I99	Diseases of the circulatory system	312903	Endocardial fibroelastosis of right ventricle (HCC)	ICD10CM	1	I42.4		Endocardial fibroelastosis	IMO	GHS-PACDC	DX_LEGACY	ACTIVE	N	1900-01-01	9999-12-31	2015-07-10
Diagnosis: ICD10 Code, I00-I99, Define	I00-I99	Diseases of the circulatory system	531210	Left ventricular noncompaction type 5 associated with mutation in MYH7 gene (HCC)	ICD10CM	1	I42.4		Endocardial fibroelastosis	IMO	GHS-PACDC	IMO_2015_R	ACTIVE	N	1900-01-01	9999-12-31	2015-07-10
Diagnosis: ICD10 Code, I00-I99, Define	I00-I99	Diseases of the circulatory system	533682	Endocardial fibroelastosis	ICD10CM	1	I42.4		Endocardial fibroelastosis	IMO	GHS-PACDC	IMO_2015_R	ACTIVE	Y	1900-01-01	9999-12-31	2015-07-10
Diagnosis: ICD10 Code, I00-I99, Define	I00-I99	Diseases of the circulatory system	531221	Left ventricular noncompaction cardiomyopathy type 9 associated with mutation in TPM1 gene	ICD10CM	1	I42.4		Endocardial fibroelastosis	IMO	GHS-PACDC	IMO_2015_R	ACTIVE	N	1900-01-01	9999-12-31	2015-07-10
Diagnosis: ICD10 Code, I00-I99, Define	I00-I99	Diseases of the circulatory system	450569	Acute on chronic diastolic heart failure secondary to restrictive cardiomyopathy (HCC)	ICD10CM	2	I42.5	I50.33,I42.5	Other restrictive cardiomyopathy	IMO	GHS-PACDC	IMO_2015_R	ACTIVE	N	2016-04-10	9999-12-31	2015-07-10
Diagnosis: ICD10 Code, I00-I99, Define	I00-I99	Diseases of the circulatory system	524689	Restrictive cardiomyopathy associated with mutation in ACTC1 gene (HCC)	ICD10CM	1	I42.5		Other restrictive cardiomyopathy	IMO	GHS-PACDC	IMO_2015_R	ACTIVE	N	1900-01-01	9999-12-31	2015-07-10
Diagnosis: ICD10 Code, I00-I99, Define	I00-I99	Diseases of the circulatory system	15739	Restrictive cardiomyopathy (HCC)	ICD10CM	1	I42.5		Other restrictive cardiomyopathy	IMO	GHS-PACDC	DX_LEGACY	ACTIVE	N	1900-01-01	9999-12-31	2015-07-10
Diagnosis: ICD10 Code, I00-I99, Define	I00-I99	Diseases of the circulatory system	260000	Diastolic heart failure secondary to restrictive cardiomyopathy (HCC)	ICD10CM	2	I42.5	I50.30,I42.5	Other restrictive cardiomyopathy	IMO	GHS-PACDC	DX_LEGACY	ACTIVE	N	2016-04-10	9999-12-31	2015-07-10
Diagnosis: ICD10 Code, I00-I99, Define	I00-I99	Diseases of the circulatory system	959850	Heart failure, diastolic, due to restrictive cardiomyopathy, acute on chronic (HCC)	ICD10CM	2	I42.5	I50.33,I42.5	Other restrictive cardiomyopathy	IMO	GHS-PACDC	IMO_2015_R	ACTIVE	N	2016-04-10	9999-12-31	2015-07-10
Diagnosis: ICD10 Code, I00-I99, Define	I00-I99	Diseases of the circulatory system	15734	Drug-induced cardiomyopathy (HCC)	ICD10CM	1	I42.7		Cardiomyopathy due to drug and external agent	IMO	GHS-PACDC	DX_LEGACY	ACTIVE	N	1900-01-01	9999-12-31	2015-07-10
Diagnosis: ICD10 Code, I00-I99, Define	I00-I99	Diseases of the circulatory system	175882	Radiation-induced cardiomyopathy (HCC)	ICD10CM	1	I42.7		Cardiomyopathy due to drug and external agent	IMO	GHS-PACDC	DX_LEGACY	ACTIVE	N	2017-09-17	9999-12-31	2017-10-30
Diagnosis: ICD10 Code, I00-I99, Define	I00-I99	Diseases of the circulatory system	234893	Chemotherapy induced cardiomyopathy (HCC)	ICD10CM	1	I42.7	I42.7,T45.1X5A	Cardiomyopathy due to drug and external agent	IMO	GHS-PACDC	DX_LEGACY	ACTIVE	N	1900-01-01	9999-12-31	2015-07-10
Diagnosis: ICD10 Code, I00-I99, Define	I00-I99	Diseases of the circulatory system	373454	Toxic cardiomyopathy (HCC)	ICD10CM	1	I42.7		Cardiomyopathy due to drug and external agent	IMO	GHS-PACDC	DX_LEGACY	ACTIVE	N	1900-01-01	9999-12-31	2015-07-10
Diagnosis: ICD10 Code, I00-I99, Define	I00-I99	Diseases of the circulatory system	17937	Heart failure, diastolic, due to restrictive cardiomyopathy (HCC)	ICD10CM	2	I42.8	I50.30,I42.8	Other cardiomyopathies	IMO	GHS-PACDC	DX_LEGACY	ACTIVE	N	1900-01-01	2016-05-07	2015-07-10
Diagnosis: ICD10 Code, I00-I99, Define	I00-I99	Diseases of the circulatory system	962756	Heart failure, diastolic, due to idiopathic cardiomyopathy, chronic (HCC)	ICD10CM	2	I42.8	I50.32,I42.8	Other cardiomyopathies	IMO	GHS-PACDC	IMO_2015_R	ACTIVE	N	1900-01-01	2016-10-14	2015-07-10
Diagnosis: ICD10 Code, I00-I99, Define	I00-I99	Diseases of the circulatory system	609812	Other cardiomyopathies	ICD10CM	1	I42.8		Other cardiomyopathies	IMO	GHS-PACDC	IMO_2015_R	ACTIVE	Y	1900-01-01	9999-12-31	2015-07-10
Diagnosis: ICD10 Code, I00-I99, Define	I00-I99	Diseases of the circulatory system	157050	Obstructive cardiomyopathy (HCC)	ICD10CM	1	I42.8		Other cardiomyopathies	IMO	GHS-PACDC	DX_LEGACY	ACTIVE	N	1900-01-01	9999-12-31	2015-07-10
Diagnosis: ICD10 Code, I00-I99, Define	I00-I99	Diseases of the circulatory system	133041	Cardiomyopathy, nonischemic (HCC)	ICD10CM	1	I42.8		Other cardiomyopathies	IMO	GHS-PACDC	DX_LEGACY	ACTIVE	N	2017-03-31	9999-12-31	2017-04-14
Diagnosis: ICD10 Code, I00-I99, Define	I00-I99	Diseases of the circulatory system	963583	Diastolic heart failure secondary to idiopathic cardiomyopathy, acute on chronic (HCC)	ICD10CM	2	I42.9	I50.33,I42.9	Cardiomyopathy, unspecified	IMO	GHS-PACDC	IMO_2015_R	ACTIVE	N	2016-09-17	9999-12-31	2016-09-30
Diagnosis: ICD10 Code, I00-I99, Define	I00-I99	Diseases of the circulatory system	430461	Maternal cardiomyopathy complicating pregnancy in third trimester (HCC)	ICD10CM	2	I42.9	O99.413,I42.9	Cardiomyopathy, unspecified	IMO	GHS-PACDC	IMO_2015_R	ACTIVE	N	1900-01-01	9999-12-31	2015-07-10
Diagnosis: ICD10 Code, I00-I99, Define	I00-I99	Diseases of the circulatory system	531853	Familial hypertrophic cardiomyopathy associated with mutation in CALR3 gene (HCC)	ICD10CM	1	I42.9		Cardiomyopathy, unspecified	IMO	GHS-PACDC	IMO_2015_R	ACTIVE	N	1900-01-01	9999-12-31	2015-07-10
Diagnosis: ICD10 Code, I00-I99, Define	I00-I99	Diseases of the circulatory system	528619	Familial hypertrophic cardiomyopathy type 6 (HCC)	ICD10CM	1	I42.9		Cardiomyopathy, unspecified	IMO	GHS-PACDC	IMO_2015_R	ACTIVE	N	1900-01-01	9999-12-31	2015-07-10
Diagnosis: ICD10 Code, I00-I99, Define	I00-I99	Diseases of the circulatory system	671169	Maternal cardiomyopathy complicating pregnancy, unspecified trimester (HCC)	ICD10CM	2	I42.9	O99.419,I42.9	Cardiomyopathy, unspecified	IMO	GHS-PACDC	IMO_2015_R	ACTIVE	N	1900-01-01	9999-12-31	2015-07-10
Diagnosis: ICD10 Code, I00-I99, Define	I00-I99	Diseases of the circulatory system	191712	Non-ischemic cardiomyopathy (HCC)	ICD10CM	1	I42.9		Cardiomyopathy, unspecified	IMO	GHS-PACDC	DX_LEGACY	ACTIVE	N	1900-01-01	2017-04-27	2015-07-10
Diagnosis: ICD10 Code, I00-I99, Define	I00-I99	Diseases of the circulatory system	528587	Familial hypertrophic cardiomyopathy type 12 (HCC)	ICD10CM	1	I42.9		Cardiomyopathy, unspecified	IMO	GHS-PACDC	IMO_2015_R	ACTIVE	N	1900-01-01	9999-12-31	2015-07-10
Diagnosis: ICD10 Code, I00-I99, Define	I00-I99	Diseases of the circulatory system	60361	Cardiomyopathy, secondary (HCC)	ICD10CM	1	I42.9		Cardiomyopathy, unspecified	IMO	GHS-PACDC	DX_LEGACY	ACTIVE	N	1900-01-01	9999-12-31	2015-07-10
Diagnosis: ICD10 Code, I00-I99, Define	I00-I99	Diseases of the circulatory system	5062	Secondary cardiomyopathy (HCC)	ICD10CM	1	I42.9		Cardiomyopathy, unspecified	IMO	GHS-PACDC	DX_LEGACY	ACTIVE	N	1900-01-01	9999-12-31	2015-07-10
Diagnosis: ICD10 Code, I00-I99, Define	I00-I99	Diseases of the circulatory system	304600	Cardiomyopathy, familial (HCC)	ICD10CM	1	I42.9		Cardiomyopathy, unspecified	IMO	GHS-PACDC	DX_LEGACY	ACTIVE	N	2016-04-10	9999-12-31	2015-07-10
Diagnosis: ICD10 Code, I00-I99, Define	I00-I99	Diseases of the circulatory system	230369	Restrictive cardiomyopathy secondary to glycogen storage disease (HCC)	ICD10CM	2	I42.9	E74.00,I42.9	Cardiomyopathy, unspecified	IMO	GHS-PACDC	DX_LEGACY	ACTIVE	N	1900-01-01	9999-12-31	2015-07-10
Diagnosis: ICD10 Code, I00-I99, Define	I00-I99	Diseases of the circulatory system	528622	Familial hypertrophic cardiomyopathy with mutation of MYL3 gene (HCC)	ICD10CM	1	I42.9		Cardiomyopathy, unspecified	IMO	GHS-PACDC	IMO_2015_R	ACTIVE	N	1900-01-01	9999-12-31	2015-07-10
Diagnosis: ICD10 Code, I00-I99, Define	I00-I99	Diseases of the circulatory system	506872	Cardiomyopathy associated with type 1 diabetes mellitus (HCC)	ICD10CM	2	I43	E10.59,I43	Cardiomyopathy in diseases classified elsewhere	IMO	GHS-PACDC	IMO_2015_R	ACTIVE	N	2016-04-10	9999-12-31	2015-07-10
Diagnosis: ICD10 Code, I00-I99, Define	I00-I99	Diseases of the circulatory system	21817	Dilated cardiomyopathy secondary to Friedreich's ataxia (HCC)	ICD10CM	2	I43	G11.1,I43	Cardiomyopathy in diseases classified elsewhere	IMO	GHS-PACDC	DX_LEGACY	ACTIVE	N	1900-01-01	9999-12-31	2015-07-10
Diagnosis: ICD10 Code, I00-I99, Define	I00-I99	Diseases of the circulatory system	21808	Dilated cardiomyopathy secondary to dermatomyositis (HCC)	ICD10CM	2	I43	M33.92,I43	Cardiomyopathy in diseases classified elsewhere	IMO	GHS-PACDC	DX_LEGACY	ACTIVE	N	1900-01-01	9999-12-31	2015-07-10
Diagnosis: ICD10 Code, I00-I99, Define	I00-I99	Diseases of the circulatory system	152730	Cardiac amyloidosis (HCC)	ICD10CM	2	I43	E85.4,I43	Cardiomyopathy in diseases classified elsewhere	IMO	GHS-PACDC	DX_LEGACY	ACTIVE	N	1900-01-01	9999-12-31	2015-07-10
Diagnosis: ICD10 Code, I00-I99, Define	I00-I99	Diseases of the circulatory system	598528	Cardiomyopathy in diseases classified elsewhere	ICD10CM	1	I43		Cardiomyopathy in diseases classified elsewhere	IMO	GHS-PACDC	IMO_2015_R	ACTIVE	Y	1900-01-01	9999-12-31	2015-07-10
Diagnosis: ICD10 Code, I00-I99, Define	I00-I99	Diseases of the circulatory system	248779	Myocardiopathy due to influenza virus (HCC)	ICD10CM	2	I43	J11.89,I43	Cardiomyopathy in diseases classified elsewhere	IMO	GHS-PACDC	DX_LEGACY	ACTIVE	N	1900-01-01	9999-12-31	2015-07-10
Diagnosis: ICD10 Code, I00-I99, Define	I00-I99	Diseases of the circulatory system	956998	Hypertensive cardiomyopathy, with heart failure (HCC)	ICD10CM	2	I43	I11.0,I43	Cardiomyopathy in diseases classified elsewhere	IMO	GHS-PACDC	IMO_2015_R	ACTIVE	N	2017-09-17	9999-12-31	2017-10-30
Diagnosis: ICD10 Code, I00-I99, Define	I00-I99	Diseases of the circulatory system	298392	Heart block, first degree	ICD10CM	1	I44.0		Atrioventricular block, first degree	IMO	GHS-PACDC	DX_LEGACY	ACTIVE	N	1900-01-01	9999-12-31	2015-07-10
Diagnosis: ICD10 Code, I00-I99, Define	I00-I99	Diseases of the circulatory system	1135092	Second degree heart block by electrocardiography	ICD10CM	1	I44.1		Atrioventricular block, second degree	IMO	GHS-PACDC	IMO_2016_R	ACTIVE	N	1900-01-01	9999-12-31	2016-06-02

Diagnosis: ICD10 Code, I00-I99, Define	I00-I99	Diseases of the circulatory system	100692	Mobitz type 2 second degree atrioventricular block	ICD10CM	1	I44.1		Atrioventricular block, second degree	IMO	GHS-PACDC	DX_LEGACY	ACTIVE	N	1900-01-01	9999-12-31	2015-07-10
Diagnosis: ICD10 Code, I00-I99, Define	I00-I99	Diseases of the circulatory system	104321	Atrioventricular block, second degree	ICD10CM	1	I44.1		Atrioventricular block, second degree	IMO	GHS-PACDC	DX_LEGACY	ACTIVE	N	1900-01-01	9999-12-31	2015-07-10
Diagnosis: ICD10 Code, I00-I99, Define	I00-I99	Diseases of the circulatory system	111541	Mobitz I	ICD10CM	1	I44.1		Atrioventricular block, second degree	IMO	GHS-PACDC	DX_LEGACY	ACTIVE	N	1900-01-01	9999-12-31	2015-07-10
Diagnosis: ICD10 Code, I00-I99, Define	I00-I99	Diseases of the circulatory system	234899	AV block, Mobitz 2	ICD10CM	1	I44.1		Atrioventricular block, second degree	IMO	GHS-PACDC	DX_LEGACY	ACTIVE	N	1900-01-01	9999-12-31	2015-07-10
Diagnosis: ICD10 Code, I00-I99, Define	I00-I99	Diseases of the circulatory system	15742	Second degree Mobitz I AV block	ICD10CM	1	I44.1		Atrioventricular block, second degree	IMO	GHS-PACDC	DX_LEGACY	ACTIVE	N	1900-01-01	9999-12-31	2015-07-10
Diagnosis: ICD10 Code, I00-I99, Define	I00-I99	Diseases of the circulatory system	167657	Complete heart block, post-surgical (HCC)	ICD10CM	1	I44.2	I44.2,Z98.89	Atrioventricular block, complete	IMO	GHS-PACDC	DX_LEGACY	ACTIVE	N	2016-04-10	2016-10-14	2015-07-10
Diagnosis: ICD10 Code, I00-I99, Define	I00-I99	Diseases of the circulatory system	5066	Atrioventricular block	ICD10CM	1	I44.30		Unspecified atrioventricular block	IMO	GHS-PACDC	DX_LEGACY	ACTIVE	N	1900-01-01	9999-12-31	2015-07-10
Diagnosis: ICD10 Code, I00-I99, Define	I00-I99	Diseases of the circulatory system	539393	Left bundle-branch block, unspecified	ICD10CM	1	I44.7		Left bundle-branch block, unspecified	IMO	GHS-PACDC	IMO_2015_R	ACTIVE	Y	1900-01-01	9999-12-31	2015-07-10
Diagnosis: ICD10 Code, I00-I99, Define	I00-I99	Diseases of the circulatory system	528243	Complete left bundle branch block (LBBB)	ICD10CM	1	I44.7		Left bundle-branch block, unspecified	IMO	GHS-PACDC	IMO_2015_R	ACTIVE	N	1900-01-01	9999-12-31	2015-07-10
Diagnosis: ICD10 Code, I00-I99, Define	I00-I99	Diseases of the circulatory system	1132542	New onset left bundle branch block (LBBB)	ICD10CM	1	I44.7		Left bundle-branch block, unspecified	IMO	GHS-PACDC	IMO_2016_R	ACTIVE	N	1900-01-01	9999-12-31	2016-06-02
Diagnosis: ICD10 Code, I00-I99, Define	I00-I99	Diseases of the circulatory system	528222	Incomplete right bundle branch block (RBBB)	ICD10CM	1	I45.0		Right fascicular block	IMO	GHS-PACDC	IMO_2015_R	ACTIVE	N	1900-01-01	2016-10-14	2015-07-10
Diagnosis: ICD10 Code, I00-I99, Define	I00-I99	Diseases of the circulatory system	493781	Right bundle branch block (RBBB) on electrocardiogram (ECG)	ICD10CM	1	I45.10		Unspecified right bundle-branch block	IMO	GHS-PACDC	IMO_2015_R	ACTIVE	N	1900-01-01	9999-12-31	2015-07-10
Diagnosis: ICD10 Code, I00-I99, Define	I00-I99	Diseases of the circulatory system	424339	Right bundle branch block (RBBB)	ICD10CM	1	I45.10		Unspecified right bundle-branch block	IMO	GHS-PACDC	IMO_2015_R	ACTIVE	N	1900-01-01	9999-12-31	2015-07-10
Diagnosis: ICD10 Code, I00-I99, Define	I00-I99	Diseases of the circulatory system	1132543	New onset right bundle branch block (RBBB)	ICD10CM	1	I45.10		Unspecified right bundle-branch block	IMO	GHS-PACDC	IMO_2016_R	ACTIVE	N	1900-01-01	9999-12-31	2016-06-02
Diagnosis: ICD10 Code, I00-I99, Define	I00-I99	Diseases of the circulatory system	592630	Other right bundle-branch block	ICD10CM	1	I45.19		Other right bundle-branch block	IMO	GHS-PACDC	IMO_2015_R	ACTIVE	Y	1900-01-01	9999-12-31	2015-07-10
Diagnosis: ICD10 Code, I00-I99, Define	I00-I99	Diseases of the circulatory system	528231	Right bundle branch block (RBBB) and left anterior fascicular block	ICD10CM	1	I45.2		Bifascicular block	IMO	GHS-PACDC	IMO_2015_R	DELETED	Y	1900-01-01	9999-12-31	2015-07-10
Diagnosis: ICD10 Code, I00-I99, Define	I00-I99	Diseases of the circulatory system	536629	Bifascicular block	ICD10CM	1	I45.2		Bifascicular block	IMO	GHS-PACDC	IMO_2015_R	ACTIVE	Y	1900-01-01	9999-12-31	2015-07-10
Diagnosis: ICD10 Code, I00-I99, Define	I00-I99	Diseases of the circulatory system	68556	Block, bifascicular	ICD10CM	1	I45.2		Bifascicular block	IMO	GHS-PACDC	DX_LEGACY	ACTIVE	N	1900-01-01	9999-12-31	2015-07-10
Diagnosis: ICD10 Code, I00-I99, Define	I00-I99	Diseases of the circulatory system	84734	Right BBB/left post fasc block	ICD10CM	1	I45.2		Bifascicular block	IMO	GHS-PACDC	DX_LEGACY	ACTIVE	N	1900-01-01	9999-12-31	2015-07-10
Diagnosis: ICD10 Code, I00-I99, Define	I00-I99	Diseases of the circulatory system	62042	Block, trifascicular	ICD10CM	1	I45.3		Trifascicular block	IMO	GHS-PACDC	DX_LEGACY	ACTIVE	N	1900-01-01	9999-12-31	2015-07-10
Diagnosis: ICD10 Code, I00-I99, Define	I00-I99	Diseases of the circulatory system	1353618	Progressive familial heart block type 1B	ICD10CM	1	I45.5		Other specified heart block	IMO	GHS-PACDC	IMO_2018_R	ACTIVE	N	1900-01-01	9999-12-31	2017-10-30
Diagnosis: ICD10 Code, I00-I99, Define	I00-I99	Diseases of the circulatory system	21969	Myofibrillar intraventricular block	ICD10CM	1	I45.5		Other specified heart block	IMO	GHS-PACDC	DX_LEGACY	ACTIVE	N	1900-01-01	9999-12-31	2015-07-10
Diagnosis: ICD10 Code, I00-I99, Define	I00-I99	Diseases of the circulatory system	247510	Concealed WPW (Wolff-Parkinson-White) syndrome	ICD10CM	1	I45.6		Pre-excitation syndrome	IMO	GHS-PACDC	DX_LEGACY	ACTIVE	N	1900-01-01	9999-12-31	2015-07-10
Diagnosis: ICD10 Code, I00-I99, Define	I00-I99	Diseases of the circulatory system	15743	Wolff-Parkinson-White syndrome	ICD10CM	1	I45.6		Pre-excitation syndrome	IMO	GHS-PACDC	DX_LEGACY	ACTIVE	N	1900-01-01	9999-12-31	2015-07-10
Diagnosis: ICD10 Code, I00-I99, Define	I00-I99	Diseases of the circulatory system	312679	Posterior septal accessory conduction	ICD10CM	1	I45.6		Pre-excitation syndrome	IMO	GHS-PACDC	DX_LEGACY	ACTIVE	N	1900-01-01	9999-12-31	2015-07-10
Diagnosis: ICD10 Code, I00-I99, Define	I00-I99	Diseases of the circulatory system	51666	Short PR-normal QRS complex syndrome	ICD10CM	1	I45.6		Pre-excitation syndrome	IMO	GHS-PACDC	DX_LEGACY	ACTIVE	N	1900-01-01	9999-12-31	2015-07-10
Diagnosis: ICD10 Code, I00-I99, Define	I00-I99	Diseases of the circulatory system	1112275	Long QT syndrome type 15	ICD10CM	1	I45.81		Long QT syndrome	IMO	GHS-PACDC	IMO_2016_R	ACTIVE	N	1900-01-01	9999-12-31	2016-06-02
Diagnosis: ICD10 Code, I00-I99, Define	I00-I99	Diseases of the circulatory system	532122	Long QT syndrome associated with mutation in SNTA1 gene	ICD10CM	1	I45.81	I45.81,Z15.89	Long QT syndrome	IMO	GHS-PACDC	IMO_2015_R	ACTIVE	N	1900-01-01	9999-12-31	2015-07-10
Diagnosis: ICD10 Code, I00-I99, Define	I00-I99	Diseases of the circulatory system	1135027	Congenital QT prolongation on electrocardiography	ICD10CM	1	I45.81		Long QT syndrome	IMO	GHS-PACDC	IMO_2016_R	ACTIVE	N	1900-01-01	9999-12-31	2016-06-02
Diagnosis: ICD10 Code, I00-I99, Define	I00-I99	Diseases of the circulatory system	532126	Long QT syndrome associated with mutation in KCNH2 gene	ICD10CM	1	I45.81	I45.81,Z15.89	Long QT syndrome	IMO	GHS-PACDC	IMO_2015_R	ACTIVE	N	1900-01-01	9999-12-31	2015-07-10
Diagnosis: ICD10 Code, I00-I99, Define	I00-I99	Diseases of the circulatory system	420977	Long QT interval and deafness	ICD10CM	1	I45.81	I45.81,H91.90	Long QT syndrome	IMO	GHS-PACDC	DX_LEGACY	ACTIVE	N	1900-01-01	9999-12-31	2015-07-10
Diagnosis: ICD10 Code, I00-I99, Define	I00-I99	Diseases of the circulatory system	299805	Isorhythmic atrioventricular dissociation	ICD10CM	1	I45.89		Other specified conduction disorders	IMO	GHS-PACDC	DX_LEGACY	ACTIVE	N	1900-01-01	9999-12-31	2015-07-10
Diagnosis: ICD10 Code, I00-I99, Define	I00-I99	Diseases of the circulatory system	421936	Other specified conduction disorders	ICD10CM	1	I45.89		Other specified conduction disorders	IMO	GHS-PACDC	IMO_2015_R	DELETED	Y	1900-01-01	9999-12-31	2015-07-10
Diagnosis: ICD10 Code, I00-I99, Define	I00-I99	Diseases of the circulatory system	528529	Atrial standstill associated with mutations of both SCN5A and GJA5 genes	ICD10CM	1	I45.89		Other specified conduction disorders	IMO	GHS-PACDC	IMO_2015_R	ACTIVE	N	1900-01-01	9999-12-31	2015-07-10
Diagnosis: ICD10 Code, I00-I99, Define	I00-I99	Diseases of the circulatory system	239285	Conduction block	ICD10CM	1	I45.9		Conduction disorder, unspecified	IMO	GHS-PACDC	DX_LEGACY	ACTIVE	N	1900-01-01	9999-12-31	2015-07-10
Diagnosis: ICD10 Code, I00-I99, Define	I00-I99	Diseases of the circulatory system	261093	Cardiac conduction disorder	ICD10CM	1	I45.9		Conduction disorder, unspecified	IMO	GHS-PACDC	DX_LEGACY	ACTIVE	N	1900-01-01	9999-12-31	2015-07-10
Diagnosis: ICD10 Code, I00-I99, Define	I00-I99	Diseases of the circulatory system	312695	Intra-ventricular conduction delay	ICD10CM	1	I45.9		Conduction disorder, unspecified	IMO	GHS-PACDC	DX_LEGACY	ACTIVE	N	2016-09-17	9999-12-31	2016-09-30
Diagnosis: ICD10 Code, I00-I99, Define	I00-I99	Diseases of the circulatory system	192824	Conduction disorder, unspecified	ICD10CM	1	I45.9		Conduction disorder, unspecified	IMO	GHS-PACDC	DX_LEGACY	ACTIVE	N	1900-01-01	9999-12-31	2015-07-10
Diagnosis: ICD10 Code, I00-I99, Define	I00-I99	Diseases of the circulatory system	1309877	Abnormal saltatory conduction	ICD10CM	1	I45.9		Conduction disorder, unspecified	IMO	GHS-PACDC	IMO_2017_R	ACTIVE	N	1900-01-01	9999-12-31	2017-04-14
Diagnosis: ICD10 Code, I00-I99, Define	I00-I99	Diseases of the circulatory system	1180403	Cardiac arrest, cause unspecified (HCC)	ICD10CM	1	I46.9		Cardiac arrest, cause unspecified	IMO	GHS-PACDC	IMO_2016_R	ACTIVE	N	1900-01-01	9999-12-31	2016-06-02
Diagnosis: ICD10 Code, I00-I99, Define	I00-I99	Diseases of the circulatory system	102952	Ventricular asystolia (HCC)	ICD10CM	1	I46.9		Cardiac arrest, cause unspecified	IMO	GHS-PACDC	DX_LEGACY	ACTIVE	N	1900-01-01	9999-12-31	2015-07-10
Diagnosis: ICD10 Code, I00-I99, Define	I00-I99	Diseases of the circulatory system	487876	Death due to cardiac arrest (HCC)	ICD10CM	1	I46.9		Cardiac arrest, cause unspecified	IMO	GHS-PACDC	IMO_2015_R	ACTIVE	N	1900-01-01	9999-12-31	2015-07-10
Diagnosis: ICD10 Code, I00-I99, Define	I00-I99	Diseases of the circulatory system	520362	Supraventricular tachycardia determined by electrocardiogram (HCC)	ICD10CM	1	I47.1		Supraventricular tachycardia	IMO	GHS-PACDC	IMO_2015_R	ACTIVE	N	1900-01-01	9999-12-31	2015-07-10
Diagnosis: ICD10 Code, I00-I99, Define	I00-I99	Diseases of the circulatory system	21945	Paroxysmal supraventricular tachycardia (HCC)	ICD10CM	1	I47.1		Supraventricular tachycardia	IMO	GHS-PACDC	DX_LEGACY	ACTIVE	N	1900-01-01	9999-12-31	2015-07-10
Diagnosis: ICD10 Code, I00-I99, Define	I00-I99	Diseases of the circulatory system	302980	Postoperative junctional ectopic tachycardia (HCC)	ICD10CM	2	I47.1	I97.89,I47.1	Supraventricular tachycardia	IMO	GHS-PACDC	DX_LEGACY	ACTIVE	N	1900-01-01	9999-12-31	2015-07-10
Diagnosis: ICD10 Code, I00-I99, Define	I00-I99	Diseases of the circulatory system	1304547	Supraventricular tachycardia during pregnancy (HCC)	ICD10CM	2	I47.1	O99.419,I47.1	Supraventricular tachycardia	IMO	GHS-PACDC	IMO_2017_R	ACTIVE	N	1900-01-01	9999-12-31	2017-04-14
Diagnosis: ICD10 Code, I00-I99, Define	I00-I99	Diseases of the circulatory system	408242	Sinoatrial nodal reentrant tachycardia (HCC)	ICD10CM	1	I47.1		Supraventricular tachycardia	IMO	GHS-PACDC	DX_LEGACY	ACTIVE	N	1900-01-01	9999-12-31	2015-07-10
Diagnosis: ICD10 Code, I00-I99, Define	I00-I99	Diseases of the circulatory system	1135087	Paroxysmal atrial tachycardia by electrocardiography (HCC)	ICD10CM	1	I47.1		Supraventricular tachycardia	IMO	GHS-PACDC	IMO_2016_R	ACTIVE	N	1900-01-01	9999-12-31	2016-06-02
Diagnosis: ICD10 Code, I00-I99, Define	I00-I99	Diseases of the circulatory system	182785	Catecholaminergic polymorphic ventricular tachycardia (HCC)	ICD10CM	1	I47.2		Ventricular tachycardia	IMO	GHS-PACDC	DX_LEGACY	ACTIVE	N	1900-01-01	9999-12-31	2015-07-10
Diagnosis: ICD10 Code, I00-I99, Define	I00-I99	Diseases of the circulatory system	252372	Bradycardia dependent ventricular tachycardia (HCC)	ICD10CM	1	I47.2		Ventricular tachycardia	IMO	GHS-PACDC	DX_LEGACY	ACTIVE	N	1900-01-01	9999-12-31	2015-07-10
Diagnosis: ICD10 Code, I00-I99, Define	I00-I99	Diseases of the circulatory system	303016	Ventricular tachycardia, monomorphic (HCC)	ICD10CM	1	I47.2		Ventricular tachycardia	IMO	GHS-PACDC	DX_LEGACY	ACTIVE	N	1900-01-01	9999-12-31	2015-07-10
Diagnosis: ICD10 Code, I00-I99, Define	I00-I99	Diseases of the circulatory system	247518	Bidirectional ventricular tachycardia (HCC)	ICD10CM	1	I47.2		Ventricular tachycardia	IMO	GHS-PACDC	DX_LEGACY	ACTIVE	N	1900-01-01	9999-12-31	2015-07-10
Diagnosis: ICD10 Code, I00-I99, Define	I00-I99	Diseases of the circulatory system	15745	Sustained VT (ventricular tachycardia) (HCC)	ICD10CM	1	I47.2		Ventricular tachycardia	IMO	GHS-PACDC	DX_LEGACY	ACTIVE	N	1900-01-01	9999-12-31	2015-07-10
Diagnosis: ICD10 Code, I00-I99, Define	I00-I99	Diseases of the circulatory system	162659	Persistent atrial fibrillation (HCC)	ICD10CM	1	I48.1		Persistent atrial fibrillation	IMO	GHS-PACDC	DX_LEGACY	ACTIVE	N	1900-01-01	9999-12-31	2015-07-10
Diagnosis: ICD10 Code, I00-I99, Define	I00-I99	Diseases of the circulatory system	1264404	Permanent atrial fibrillation with rapid ventricular response (HCC)	ICD10CM	1	I48.2		Chronic atrial fibrillation	IMO	GHS-PACDC	IMO_2017_R	ACTIVE	N	1900-01-01	9999-12-31	2016-09-30
Diagnosis: ICD10 Code, I00-I99, Define	I00-I99	Diseases of the circulatory system	161344	Chronic a-fib (HCC)	ICD10CM	1	I48.2		Chronic atrial fibrillation	IMO	GHS-PACDC	DX_LEGACY	ACTIVE	N	1900-01-01	9999-12-31	2015-07-10
Diagnosis: ICD10 Code, I00-I99, Define	I00-I99	Diseases of the circulatory system	325945	Typical atrial flutter (HCC)	ICD10CM	1	I48.3		Typical atrial flutter	IMO	GHS-PACDC	DX_LEGACY	ACTIVE	N	1900-01-01	9999-12-31	2015-07-10
Diagnosis: ICD10 Code, I00-I99, Define	I00-I99	Diseases of the circulatory system	613323	Unspecified atrial fibrillation and atrial flutter	ICD10CM	1	I48.9		Unspecified atrial fibrillation and atrial flutter	IMO	GHS-PACDC	IMO_2015_R	ACTIVE	Y	1900-01-01	9999-12-31	2015-07-10
Diagnosis: ICD10 Code, I00-I99, Define	I00-I99	Diseases of the circulatory system	529109	Familial atrial fibrillation associated with mutation in NPPA gene (HCC)	ICD10CM	1	I48.91	I48.91,Z15.89	Unspecified atrial fibrillation	IMO	GHS-PACDC	IMO_2015_R	ACTIVE	N	1900-01-01	2017-04-27	2015-07-10
Diagnosis: ICD10 Code, I00-I99, Define	I00-I99	Diseases of the circulatory system	412927	Atrial fibrillation by electrocardiogram (HCC)	ICD10CM	1	I48.91		Unspecified atrial fibrillation	IMO	GHS-PACDC	DX_LEGACY	ACTIVE	N	1900-01-01	9999-12-31	2015-07-10
Diagnosis: ICD10 Code, I00-I99, Define	I00-I99	Diseases of the circulatory system	1258397	First detected episode of atrial fibrillation (HCC)	ICD10CM	1	I48.91		Unspecified atrial fibrillation	IMO	GHS-PACDC	IMO_2017_R	ACTIVE	N	1900-01-01	9999-12-31	2016-09-30
Diagnosis: ICD10 Code, I00-I99, Define	I00-I99	Diseases of the circulatory system	529112	Familial atrial fibrillation type 7 (HCC)	ICD10CM	1	I48.91	I48.91,Z15.89	Unspecified atrial fibrillation	IMO	GHS-PACDC	IMO_2015_R	ACTIVE	N	1900-01-01	2017-04-27	2015-07-10
Diagnosis: ICD10 Code, I00-I99, Define	I00-I99	Diseases of the circulatory system	102958	VF (ventricular fibrillation) (HCC)	ICD10CM	1	I49.01		Ventricular fibrillation	IMO	GHS-PACDC	DX_LEGACY	ACTIVE	N	1900-01-01	9999-12-31	2015-07-10
Diagnosis: ICD10 Code, I00-I99, Define	I00-I99	Diseases of the circulatory system	510293	Paroxysmal familial ventricular fibrillation type 1 (HCC)	ICD10CM	1	I49.01		Ventricular fibrillation	IMO	GHS-PACDC	IMO_2015_R	ACTIVE	N	1900-01-01	9999-12-31	2015-07-10
Diagnosis: ICD10 Code, I00-I99, Define	I00-I99	Diseases of the circulatory system	330514	Periodic heart flutter	ICD10CM	1	I49.02		Ventricular flutter	IMO	GHS-PACDC	DX_LEGACY	ACTIVE	N	1900-01-01	2016-05-07	2015-07-10
Diagnosis: ICD10 Code, I00-I99, Define	I00-I99	Diseases of the circulatory system	231966	Ventricular fibrillation and flutter (HCC)	ICD10CM	2	I49.02	I49.01,I49.02	Ventricular flutter	IMO	GHS-PACDC	DX_LEGACY	ACTIVE	N	1900-01-01	9999-12-31	2015-07-10
Diagnosis: ICD10 Code, I00-I99, Define	I00-I99	Diseases of the circulatory system	21946	Supraventricular premature beats	ICD10CM	1	I49.1		Atrial premature depolarization	IMO	GHS-PACDC	DX_LEGACY	ACTIVE	N	1900-01-01	9999-12-31	2015-07-10
Diagnosis: ICD10 Code, I00-I99, Define	I00-I99	Diseases of the circulatory system	408258	Run of atrial premature complexes	ICD10CM	1	I49.1		Atrial premature depolarization	IMO	GHS-PACDC	DX_LEGACY	ACTIVE	N	1900-01-01	9999-12-31	2015-07-10
Diagnosis: ICD10 Code, I00-I99, Define	I00-I99	Diseases of the circulatory system	248969	Junctional premature beats (HCC)	ICD10CM	1	I49.2		Junctional premature depolarization	IMO	GHS-PACDC	DX_LEGACY	ACTIVE	N	1900-01-01	9999-12-31	2015-07-10
Diagnosis: ICD10 Code, I00-I99, Define	I00-I99	Diseases of the circulatory system	248972	Ectopic junctional beats (HCC)	ICD10CM	1	I49.2		Junctional premature depolarization	IMO	GHS-PACDC	DX_LEGACY	ACTIVE	N	1900-01-01	9999-12-31	2015-07-10
Diagnosis: ICD10 Code, I00-I99, Define	I00-I99	Diseases of the circulatory system	250251	Isolated premature ventricular contractions	ICD10CM	1	I49.3		Ventricular premature depolarization	IMO	GHS-PACDC	DX_LEGACY	ACTIVE	N	1900-01-01	9999-12-31	2015-07-10
Diagnosis: ICD10 Code, I00-I99, Define	I00-I99	Diseases of the circulatory system	60014	Ventricular premature complex	ICD10CM	1	I49.3		Ventricular premature depolarization	IMO	GHS-PACDC	DX_LEGACY	ACTIVE	N	1900-01-01	9999-12-31	2015-07-10
Diagnosis: ICD10 Code, I00-I99, Define	I00-I99	Diseases of the circulatory system	161582	Ventricular ectopy/arrhythmia, pre-operative cardiovascular exam	ICD10CM	2	I49.3	Z01.810,I49.3	Ventricular premature depolarization	IMO	GHS-PACDC	DX_LEGACY	ACTIVE	N	2017-09-17	9999-12-31	2017-10-30
Diagnosis: ICD10 Code, I00-I99, Define	I00-I99	Diseases of the circulatory system	1135080	Premature ventricular contraction on electrocardiography	ICD10CM	1	I49.3		Ventricular premature depolarization	IMO	GHS-PACDC	IMO_2016_R	ACTIVE	N	1900-01-01	9999-12-31	2016-06-02
Diagnosis: ICD10 Code, I00-I99, Define	I00-I99	Diseases of the circulatory system	496653	Cardiac arrhythmia due to premature depolarization	ICD10CM	1	I49.40		Unspecified premature depolarization	IMO	GHS-PACDC	IMO_2015_R	ACTIVE	N	1900-01-01	9999-12-31	2015-07-10
Diagnosis: ICD10 Code, I00-I99, Define	I00-I99	Diseases of the circulatory system	47221	Cardiac premature complexes	ICD10CM	1	I49.40		Unspecified premature depolarization	IMO	GHS-PACDC	DX_LEGACY	ACTIVE	N	1900-01-01	9999-12-31	2015-07-10
Diagnosis: ICD10 Code, I00-I99, Define	I00-I99	Diseases of the circulatory system	47231	Premature cardiac complex	ICD10CM	1	I49.40		Unspecified premature depolarization	IMO	GHS-PACDC	DX_LEGACY	ACTIVE	N	1900-01-01	9999-12-31	2015-07-10
Diagnosis: ICD10 Code, I00-I99, Define	I00-I99	Diseases of the circulatory system	124333	Premature contraction	ICD10CM	1	I49.49		Other premature depolarization	IMO	GHS-PACDC	DX_LEGACY	ACTIVE	N	1900-01-01	9999-12-31	2015-07-10
Diagnosis: ICD10 Code, I00-I99, Define	I00-I99	Diseases of the circulatory system	244731	Sick sinus syndrome with tachycardia (HCC)	ICD10CM	1	I49.5		Sick sinus syndrome	IMO	GHS-PACDC	DX_LEGACY	ACTIVE	N	1900-01-01	9999-12-31	2015-07-10
Diagnosis: ICD10 Code, I00-I99, Define	I00-I99	Diseases of the circulatory system	515532	MYH6-related sick sinus syndrome, autosomal dominant (HCC)	ICD10CM	1	I49.5	I49.5,Z15.89	Sick sinus syndrome	IMO	GHS-PACDC	IMO_2015_R	ACTIVE	N	1900-01-01	9999-12-31	2015-07-10
Diagnosis: ICD10 Code, I00-I99, Define	I00-I99	Diseases of the circulatory system	510149	Brugada syndrome type 7 associated with mutation in SCN3B gene	ICD10CM	1	I49.8		Other specified cardiac arrhythmias	IMO	GHS-PACDC	IMO_2015_R	ACTIVE	N	2016-09-17	9999-12-31	2016-09-30
Diagnosis: ICD10 Code, I00-I99, Define	I00-I99	Diseases of the circulatory system	408243	Withdrawal arrhythmia (HCC)	ICD10CM	1	I49.8	I49.8,F19.939	Other specified cardiac arrhythmias	IMO	GHS-PACDC	DX_LEGACY	ACTIVE	N	1900-01-01	9999-12-31	2015-07-10
Diagnosis: ICD10 Code, I00-I99, Define	I00-I99	Diseases of the circulatory system	505786	RANGRF-related Brugada syndrome	ICD10CM	1	I49.8		Other specified cardiac arrhythmias	IMO	GHS-PACDC	IMO_2015_R	ACTIVE	N	2016-09-17	9999-12-31	2016-09-30
Diagnosis: ICD10 Code, I00-I99, Define	I00-I99	Diseases of the circulatory system	414166	Ectopic cardiac beats	ICD10CM	1	I49.8		Other specified cardiac arrhythmias	IMO	GHS-PACDC	DX_LEGACY	ACTIVE	N	1900-01-01	2016-05-07	2015-07-10
Diagnosis: ICD10 Code, I00-I99, Define	I00-I99	Diseases of the circulatory system	110455	Junctional rhythm	ICD10CM	1	I49.8		Other specified cardiac arrhythmias	IMO	GHS-PACDC	DX_LEGACY	ACTIVE	N	1900-01-01	9999-12-31	2015-07-10
Diagnosis: ICD10 Code, I00-I99, Define	I00-I99	Diseases of the circulatory system	246814	Pulsus trigeminus	ICD10CM	1	I49.8		Other specified cardiac arrhythmias	IMO	GHS-PACDC	DX_LEGACY	ACTIVE	N	1900-01-01	2016-05-07	2015-07-10
Diagnosis: ICD10 Code, I00-I99, Define	I00-I99	Diseases of the circulatory system	505882	Brugada syndrome associated with mutation in SCN2B gene	ICD10CM	1	I49.8		Other specified cardiac arrhythmias	IMO	GHS-PACDC	IMO_2015_R	ACTIVE	N	2016-09-17	9999-12-31	2016-09-30
Diagnosis: ICD10 Code, I00-I99, Define	I00-I99	Diseases of the circulatory system	58545	Accelerated idioventricular rhythm	ICD10CM	1	I49.8		Other specified cardiac arrhythmias	IMO	GHS-PACDC	DX_LEGACY	ACTIVE	N	2016-04-10	9999-12-31	2015-07-10
Diagnosis: ICD10 Code, I00-I99, Define	I00-I99	Diseases of the circulatory system	506556	SCN3B-related Brugada syndrome 7	ICD10CM	1	I49.8		Other specified cardiac arrhythmias	IMO	GHS-PACDC	IMO_2015_R	ACTIVE	N	2016-09-17	9999-12-31	2016-09-30
Diagnosis: ICD10 Code, I00-I99, Define	I00-I99	Diseases of the circulatory system	430451	Maternal arrhythmia complicating pregnancy in second trimester	ICD10CM	2	I49.9	O99.412,I49.9	Cardiac arrhythmia, unspecified	IMO	GHS-PACDC	IMO_2015_R	ACTIVE	N	1900-01-01	9999-12-31	2015-07-10
Diagnosis: ICD10 Code, I00-I99, Define	I00-I99	Diseases of the circulatory system	1134503	Pulse regularly irregular	ICD10CM	1	I49.9		Cardiac arrhythmia, unspecified	IMO	GHS-PACDC	IMO_2016_R	ACTIVE	N	1900-01-01	9999-12-31	2016-06-02
Diagnosis: ICD10 Code, I00-I99, Define	I00-I99	Diseases of the circulatory system	1132631	Regularly irregular pulse rhythym	ICD10CM	1	I49.9		Cardiac arrhythmia, unspecified	IMO	GHS-PACDC	IMO_2016_R	ACTIVE	N	1900-01-01	9999-12-31	2016-06-02
Diagnosis: ICD10 Code, I00-I99, Define	I00-I99	Diseases of the circulatory system	258476	Supraventricular arrhythmia	ICD10CM	1	I49.9		Cardiac arrhythmia, unspecified	IMO	GHS-PACDC	DX_LEGACY	ACTIVE	N	1900-01-01	9999-12-31	2015-07-10
Diagnosis: ICD10 Code, I00-I99, Define	I00-I99	Diseases of the circulatory system	160883	Atrial bigeminy	ICD10CM	1	I49.9		Cardiac arrhythmia, unspecified	IMO	GHS-PACDC	DX_LEGACY	ACTIVE	N	1900-01-01	2016-05-07	2015-07-10
Diagnosis: ICD10 Code, I00-I99, Define	I00-I99	Diseases of the circulatory system	367033	Pulmonary edema with congestive heart failure with reduced left ventricular function (HCC)	ICD10CM	1	I50.1		Left ventricular failure, unspecified	IMO	GHS-PACDC	DX_LEGACY	ACTIVE	N	1900-01-01	9999-12-31	2015-07-10

Diagnosis: ICD10 Code, I00-I99, Define	I00-I99	Diseases of the circulatory system	491298	Pulmonary edema with diastolic CHF, NYHA class 3 (HCC)	ICD10CM	1	I50.1	I50.1,I50.30	Left ventricular failure, unspecified	IMO	GHS-PACDC	IMO_2015_R	ACTIVE	N	1900-01-01	2017-10-14	2015-07-10
Diagnosis: ICD10 Code, I00-I99, Define	I00-I99	Diseases of the circulatory system	384757	Left heart failure with preserved LV function (HCC)	ICD10CM	1	I50.1		Left ventricular failure, unspecified	IMO	GHS-PACDC	DX_LEGACY	ACTIVE	N	1900-01-01	9999-12-31	2015-07-10
Diagnosis: ICD10 Code, I00-I99, Define	I00-I99	Diseases of the circulatory system	491199	Pulmonary edema with NYHA class 3 systolic heart failure (HCC)	ICD10CM	1	I50.1		Left ventricular failure, unspecified	IMO	GHS-PACDC	IMO_2015_R	ACTIVE	N	2016-09-17	9999-12-31	2016-09-30
Diagnosis: ICD10 Code, I00-I99, Define	I00-I99	Diseases of the circulatory system	109141	Heart failure, acute, left-sided (HCC)	ICD10CM	1	I50.1		Left ventricular failure, unspecified	IMO	GHS-PACDC	DX_LEGACY	ACTIVE	N	1900-01-01	9999-12-31	2015-07-10
Diagnosis: ICD10 Code, I00-I99, Define	I00-I99	Diseases of the circulatory system	50916	Cardiac failure left (HCC)	ICD10CM	1	I50.1		Left ventricular failure, unspecified	IMO	GHS-PACDC	DX_LEGACY	ACTIVE	N	1900-01-01	9999-12-31	2015-07-10
Diagnosis: ICD10 Code, I00-I99, Define	I00-I99	Diseases of the circulatory system	490574	Heart failure with reduced ejection fraction, NYHA class III (HCC)	ICD10CM	1	I50.20		Unspecified systolic (congestive) heart failure	IMO	GHS-PACDC	IMO_2015_R	ACTIVE	N	2017-09-17	9999-12-31	2017-10-30
Diagnosis: ICD10 Code, I00-I99, Define	I00-I99	Diseases of the circulatory system	961778	Class I congestive heart failure, unspecified failure chronicity, systolic (HCC)	ICD10CM	1	I50.20		Unspecified systolic (congestive) heart failure	IMO	GHS-PACDC	IMO_2015_R	ACTIVE	N	1900-01-01	9999-12-31	2015-07-10
Diagnosis: ICD10 Code, I00-I99, Define	I00-I99	Diseases of the circulatory system	958055	CHF NYHA class III (symptoms with mildly strenuous activities), unspecified failure chronicity, sy	ICD10CM	1	I50.20		Unspecified systolic (congestive) heart failure	IMO	GHS-PACDC	IMO_2015_R	ACTIVE	N	1900-01-01	9999-12-31	2015-07-10
Diagnosis: ICD10 Code, I00-I99, Define	I00-I99	Diseases of the circulatory system	962104	CHF (NYHA class III, ACC/AHA stage C), unspecified failure chronicity, systolic	ICD10CM	1	I50.20		Unspecified systolic (congestive) heart failure	IMO	GHS-PACDC	IMO_2015_R	DELETED	Y	1900-01-01	9999-12-31	2015-07-10
Diagnosis: ICD10 Code, I00-I99, Define	I00-I99	Diseases of the circulatory system	260599	Systolic heart failure secondary to idiopathic cardiomyopathy (HCC)	ICD10CM	1	I50.20	I50.20,I42.9	Unspecified systolic (congestive) heart failure	IMO	GHS-PACDC	DX_LEGACY	ACTIVE	N	2016-09-17	9999-12-31	2016-09-30
Diagnosis: ICD10 Code, I00-I99, Define	I00-I99	Diseases of the circulatory system	136055	Systolic heart failure (HCC)	ICD10CM	1	I50.20		Unspecified systolic (congestive) heart failure	IMO	GHS-PACDC	DX_LEGACY	ACTIVE	N	1900-01-01	9999-12-31	2015-07-10
Diagnosis: ICD10 Code, I00-I99, Define	I00-I99	Diseases of the circulatory system	1416993	Systolic congestive heart failure, NYHA class 1, unspecified congestive heart failure chronicity (H	ICD10CM	1	I50.20		Unspecified systolic (congestive) heart failure	IMO	GHS-PACDC	IMO_2018_R	ACTIVE	N	1900-01-01	9999-12-31	2017-10-30
Diagnosis: ICD10 Code, I00-I99, Define	I00-I99	Diseases of the circulatory system	16338	Heart failure, systolic (HCC)	ICD10CM	1	I50.20		Unspecified systolic (congestive) heart failure	IMO	GHS-PACDC	DX_LEGACY	ACTIVE	N	1900-01-01	9999-12-31	2015-07-10
Diagnosis: ICD10 Code, I00-I99, Define	I00-I99	Diseases of the circulatory system	1132658	Benign hypertensive heart and kidney disease with NYHA class 2 systolic congestive heart failure	ICD10CM	2	I50.20	I13.0,I50.20,N18.2	Unspecified systolic (congestive) heart failure	IMO	GHS-PACDC	IMO_2016_R	ACTIVE	N	1900-01-01	9999-12-31	2016-06-02
Diagnosis: ICD10 Code, I00-I99, Define	I00-I99	Diseases of the circulatory system	491283	Benign hypertensive heart and kidney disease with systolic congestive heart failure, NYHA class	ICD10CM	2	I50.20	I13.0,I50.20,N18.4	Unspecified systolic (congestive) heart failure	IMO	GHS-PACDC	IMO_2015_R	ACTIVE	N	1900-01-01	9999-12-31	2015-07-10
Diagnosis: ICD10 Code, I00-I99, Define	I00-I99	Diseases of the circulatory system	958384	CHF NYHA class IV, unspecified failure chronicity, systolic (HCC)	ICD10CM	1	I50.20		Unspecified systolic (congestive) heart failure	IMO	GHS-PACDC	IMO_2015_R	ACTIVE	N	1900-01-01	9999-12-31	2015-07-10
Diagnosis: ICD10 Code, I00-I99, Define	I00-I99	Diseases of the circulatory system	499448	Congestive heart failure with right ventricular systolic dysfunction (HCC)	ICD10CM	1	I50.20		Unspecified systolic (congestive) heart failure	IMO	GHS-PACDC	IMO_2015_R	ACTIVE	N	1900-01-01	2017-10-14	2015-07-10
Diagnosis: ICD10 Code, I00-I99, Define	I00-I99	Diseases of the circulatory system	1378539	End-stage systolic heart failure, unspecified heart failure chronicity (HCC)	ICD10CM	1	I50.20		Unspecified systolic (congestive) heart failure	IMO	GHS-PACDC	IMO_2018_R	ACTIVE	N	1900-01-01	9999-12-31	2017-10-30
Diagnosis: ICD10 Code, I00-I99, Define	I00-I99	Diseases of the circulatory system	490436	NYHA class 1 heart failure with reduced ejection fraction (HCC)	ICD10CM	1	I50.20		Unspecified systolic (congestive) heart failure	IMO	GHS-PACDC	IMO_2015_R	ACTIVE	N	2017-09-17	9999-12-31	2017-10-30
Diagnosis: ICD10 Code, I00-I99, Define	I00-I99	Diseases of the circulatory system	1353400	Hypertensive heart disease with acute systolic congestive heart failure (HCC)	ICD10CM	2	I50.21	I11.0,I50.21	Acute systolic (congestive) heart failure	IMO	GHS-PACDC	IMO_2018_R	ACTIVE	N	1900-01-01	9999-12-31	2017-10-30
Diagnosis: ICD10 Code, I00-I99, Define	I00-I99	Diseases of the circulatory system	387148	Acute systolic CHF (congestive heart failure), NYHA class 1 (HCC)	ICD10CM	1	I50.21		Acute systolic (congestive) heart failure	IMO	GHS-PACDC	DX_LEGACY	ACTIVE	N	1900-01-01	9999-12-31	2015-07-10
Diagnosis: ICD10 Code, I00-I99, Define	I00-I99	Diseases of the circulatory system	960107	Class I congestive heart failure, acute, systolic (HCC)	ICD10CM	1	I50.21		Acute systolic (congestive) heart failure	IMO	GHS-PACDC	IMO_2015_R	ACTIVE	N	1900-01-01	9999-12-31	2015-07-10
Diagnosis: ICD10 Code, I00-I99, Define	I00-I99	Diseases of the circulatory system	953974	Congestive heart failure, NYHA class 1, acute, systolic (HCC)	ICD10CM	1	I50.21		Acute systolic (congestive) heart failure	IMO	GHS-PACDC	IMO_2015_R	ACTIVE	N	1900-01-01	9999-12-31	2015-07-10
Diagnosis: ICD10 Code, I00-I99, Define	I00-I99	Diseases of the circulatory system	1353401	Hypertensive heart disease with acute on chronic systolic congestive heart failure (HCC)	ICD10CM	2	I50.23	I11.0,I50.23	Acute on chronic systolic (congestive) heart failure	IMO	GHS-PACDC	IMO_2018_R	ACTIVE	N	1900-01-01	9999-12-31	2017-10-30
Diagnosis: ICD10 Code, I00-I99, Define	I00-I99	Diseases of the circulatory system	960402	Clinical systolic heart failure, acute on chronic (HCC)	ICD10CM	1	I50.23		Acute on chronic systolic (congestive) heart failure	IMO	GHS-PACDC	IMO_2015_R	ACTIVE	N	1900-01-01	9999-12-31	2015-07-10
Diagnosis: ICD10 Code, I00-I99, Define	I00-I99	Diseases of the circulatory system	499327	Acute on chronic systolic heart failure, NYHA class 2 (HCC)	ICD10CM	1	I50.23		Acute on chronic systolic (congestive) heart failure	IMO	GHS-PACDC	IMO_2015_R	ACTIVE	N	1900-01-01	9999-12-31	2015-07-10
Diagnosis: ICD10 Code, I00-I99, Define	I00-I99	Diseases of the circulatory system	532319	Diastolic CHF due to valvular disease (HCC)	ICD10CM	2	I50.30	I38,I50.30	Unspecified diastolic (congestive) heart failure	IMO	GHS-PACDC	IMO_2015_R	ACTIVE	N	1900-01-01	9999-12-31	2015-07-10
Diagnosis: ICD10 Code, I00-I99, Define	I00-I99	Diseases of the circulatory system	260616	Hypertrophic obstructive cardiomyopathy with diastolic heart failure (HCC)	ICD10CM	1	I50.30	I50.30,I42.1	Unspecified diastolic (congestive) heart failure	IMO	GHS-PACDC	DX_LEGACY	ACTIVE	N	1900-01-01	9999-12-31	2015-07-10
Diagnosis: ICD10 Code, I00-I99, Define	I00-I99	Diseases of the circulatory system	490569	NYHA class 3 heart failure with borderline preserved ejection fraction (HCC)	ICD10CM	1	I50.30		Unspecified diastolic (congestive) heart failure	IMO	GHS-PACDC	IMO_2015_R	ACTIVE	N	2017-09-17	9999-12-31	2017-10-30
Diagnosis: ICD10 Code, I00-I99, Define	I00-I99	Diseases of the circulatory system	519902	Diastolic congestive heart failure due to valvular disease (HCC)	ICD10CM	2	I50.30	I38,I50.30	Unspecified diastolic (congestive) heart failure	IMO	GHS-PACDC	IMO_2015_R	ACTIVE	N	1900-01-01	9999-12-31	2015-07-10
Diagnosis: ICD10 Code, I00-I99, Define	I00-I99	Diseases of the circulatory system	379278	Diastolic congestive heart failure with preserved left ventricular function, NYHA class 4 (HCC)	ICD10CM	1	I50.30		Unspecified diastolic (congestive) heart failure	IMO	GHS-PACDC	DX_LEGACY	ACTIVE	N	1900-01-01	9999-12-31	2015-07-10
Diagnosis: ICD10 Code, I00-I99, Define	I00-I99	Diseases of the circulatory system	956547	CHF NYHA class II (symptoms with moderately strenuous activities), unspecified failure chronicit	ICD10CM	1	I50.30		Unspecified diastolic (congestive) heart failure	IMO	GHS-PACDC	IMO_2015_R	ACTIVE	N	1900-01-01	9999-12-31	2015-07-10
Diagnosis: ICD10 Code, I00-I99, Define	I00-I99	Diseases of the circulatory system	490575	NYHA class 4 heart failure with borderline preserved ejection fraction (HCC)	ICD10CM	1	I50.30		Unspecified diastolic (congestive) heart failure	IMO	GHS-PACDC	IMO_2015_R	ACTIVE	N	2017-09-17	9999-12-31	2017-10-30
Diagnosis: ICD10 Code, I00-I99, Define	I00-I99	Diseases of the circulatory system	1416995	Diastolic congestive heart failure, NYHA class 2, unspecified congestive heart failure chronicity (	ICD10CM	1	I50.30		Unspecified diastolic (congestive) heart failure	IMO	GHS-PACDC	IMO_2018_R	ACTIVE	N	1900-01-01	9999-12-31	2017-10-30
Diagnosis: ICD10 Code, I00-I99, Define	I00-I99	Diseases of the circulatory system	499612	Severe right ventricular systolic dysfunction (HCC)	ICD10CM	1	I50.30		Unspecified diastolic (congestive) heart failure	IMO	GHS-PACDC	IMO_2015_R	ACTIVE	N	1900-01-01	9999-12-31	2015-07-10
Diagnosis: ICD10 Code, I00-I99, Define	I00-I99	Diseases of the circulatory system	259979	Diastolic heart failure secondary to hypertension (HCC)	ICD10CM	2	I50.30	I11.0,I50.30	Unspecified diastolic (congestive) heart failure	IMO	GHS-PACDC	DX_LEGACY	ACTIVE	N	1900-01-01	9999-12-31	2015-07-10
Diagnosis: ICD10 Code, I00-I99, Define	I00-I99	Diseases of the circulatory system	419835	Benign hypertensive heart disease with diastolic congestive heart failure, NYHA class 2 (HCC)	ICD10CM	2	I50.30	I11.0,I50.30	Unspecified diastolic (congestive) heart failure	IMO	GHS-PACDC	DX_LEGACY	ACTIVE	N	1900-01-01	9999-12-31	2015-07-10
Diagnosis: ICD10 Code, I00-I99, Define	I00-I99	Diseases of the circulatory system	490724	Benign hypertensive heart and kidney disease with diastolic CHF, NYHA class 3 and CKD stage 3	ICD10CM	2	I50.30	I13.0,I50.30,N18.3	Unspecified diastolic (congestive) heart failure	IMO	GHS-PACDC	IMO_2015_R	ACTIVE	N	1900-01-01	9999-12-31	2015-07-10
Diagnosis: ICD10 Code, I00-I99, Define	I00-I99	Diseases of the circulatory system	387604	Diastolic congestive heart failure, NYHA class 2 (HCC)	ICD10CM	1	I50.30		Unspecified diastolic (congestive) heart failure	IMO	GHS-PACDC	DX_LEGACY	ACTIVE	N	1900-01-01	9999-12-31	2015-07-10
Diagnosis: ICD10 Code, I00-I99, Define	I00-I99	Diseases of the circulatory system	961293	Congestive heart failure due to valvular disease, unspecified failure chronicity, diastolic	ICD10CM	1	I50.30		Unspecified diastolic (congestive) heart failure	IMO	GHS-PACDC	IMO_2015_R	DELETED	Y	2016-04-10	9999-12-31	2015-07-10
Diagnosis: ICD10 Code, I00-I99, Define	I00-I99	Diseases of the circulatory system	964168	Congestive heart failure, NYHA class 4, acute, diastolic (HCC)	ICD10CM	1	I50.31		Acute diastolic (congestive) heart failure	IMO	GHS-PACDC	IMO_2015_R	ACTIVE	N	1900-01-01	9999-12-31	2015-07-10
Diagnosis: ICD10 Code, I00-I99, Define	I00-I99	Diseases of the circulatory system	958270	Diastolic heart failure secondary to idiopathic cardiomyopathy, acute (HCC)	ICD10CM	1	I50.31	I50.31,I42.9	Acute diastolic (congestive) heart failure	IMO	GHS-PACDC	IMO_2015_R	ACTIVE	N	2016-09-17	9999-12-31	2016-09-30
Diagnosis: ICD10 Code, I00-I99, Define	I00-I99	Diseases of the circulatory system	959291	Heart failure, diastolic, due to hypertrophic obstructive cardiomyopathy, acute (HCC)	ICD10CM	1	I50.31	I50.31,I42.1	Acute diastolic (congestive) heart failure	IMO	GHS-PACDC	IMO_2015_R	ACTIVE	N	1900-01-01	9999-12-31	2015-07-10
Diagnosis: ICD10 Code, I00-I99, Define	I00-I99	Diseases of the circulatory system	955692	Diastolic heart failure secondary to restrictive cardiomyopathy, acute (HCC)	ICD10CM	1	I50.31	I50.31,I42.8	Acute diastolic (congestive) heart failure	IMO	GHS-PACDC	IMO_2015_R	ACTIVE	N	1900-01-01	2016-05-07	2015-07-10
Diagnosis: ICD10 Code, I00-I99, Define	I00-I99	Diseases of the circulatory system	958929	Congestive heart failure, NYHA class II, acute, diastolic (HCC)	ICD10CM	1	I50.31		Acute diastolic (congestive) heart failure	IMO	GHS-PACDC	IMO_2015_R	ACTIVE	N	1900-01-01	9999-12-31	2015-07-10
Diagnosis: ICD10 Code, I00-I99, Define	I00-I99	Diseases of the circulatory system	954385	CHF NYHA class I, acute, diastolic (HCC)	ICD10CM	1	I50.31		Acute diastolic (congestive) heart failure	IMO	GHS-PACDC	IMO_2015_R	ACTIVE	N	1900-01-01	9999-12-31	2015-07-10
Diagnosis: ICD10 Code, I00-I99, Define	I00-I99	Diseases of the circulatory system	962396	CHF (NYHA class III, ACC/AHA stage C), acute, diastolic	ICD10CM	1	I50.31		Acute diastolic (congestive) heart failure	IMO	GHS-PACDC	IMO_2015_R	DELETED	Y	1900-01-01	9999-12-31	2015-07-10
Diagnosis: ICD10 Code, I00-I99, Define	I00-I99	Diseases of the circulatory system	387184	Acute diastolic HF (heart failure), NYHA class 1 (HCC)	ICD10CM	1	I50.31		Acute diastolic (congestive) heart failure	IMO	GHS-PACDC	DX_LEGACY	ACTIVE	N	1900-01-01	9999-12-31	2015-07-10
Diagnosis: ICD10 Code, I00-I99, Define	I00-I99	Diseases of the circulatory system	461583	Chronic diastolic heart failure secondary to hypertrophic cardiomyopathy (HCC)	ICD10CM	1	I50.32	I50.32,I42.1	Chronic diastolic (congestive) heart failure	IMO	GHS-PACDC	IMO_2015_R	ACTIVE	N	1900-01-01	2016-05-07	2015-07-10
Diagnosis: ICD10 Code, I00-I99, Define	I00-I99	Diseases of the circulatory system	234392	Diastolic CHF, chronic (HCC)	ICD10CM	1	I50.32		Chronic diastolic (congestive) heart failure	IMO	GHS-PACDC	DX_LEGACY	ACTIVE	N	1900-01-01	9999-12-31	2015-07-10
Diagnosis: ICD10 Code, I00-I99, Define	I00-I99	Diseases of the circulatory system	960350	CHF (NYHA class IV, ACC/AHA stage D), chronic, diastolic	ICD10CM	1	I50.32		Chronic diastolic (congestive) heart failure	IMO	GHS-PACDC	IMO_2015_R	DELETED	Y	1900-01-01	9999-12-31	2015-07-10
Diagnosis: ICD10 Code, I00-I99, Define	I00-I99	Diseases of the circulatory system	451955	Chronic diastolic heart failure secondary to restrictive cardiomyopathy (HCC)	ICD10CM	1	I50.32	I50.32,I42.8	Chronic diastolic (congestive) heart failure	IMO	GHS-PACDC	IMO_2015_R	ACTIVE	N	1900-01-01	2016-05-07	2015-07-10
Diagnosis: ICD10 Code, I00-I99, Define	I00-I99	Diseases of the circulatory system	955778	Diastolic heart failure secondary to restrictive cardiomyopathy, chronic (HCC)	ICD10CM	1	I50.32	I50.32,I42.8	Chronic diastolic (congestive) heart failure	IMO	GHS-PACDC	IMO_2015_R	ACTIVE	N	1900-01-01	2016-05-07	2015-07-10
Diagnosis: ICD10 Code, I00-I99, Define	I00-I99	Diseases of the circulatory system	451955	Chronic diastolic heart failure secondary to restrictive cardiomyopathy (HCC)	ICD10CM	1	I50.32	I50.32,I42.5	Chronic diastolic (congestive) heart failure	IMO	GHS-PACDC	IMO_2015_R	ACTIVE	N	2016-04-10	9999-12-31	2015-07-10
Diagnosis: ICD10 Code, I00-I99, Define	I00-I99	Diseases of the circulatory system	587153	Chronic diastolic (congestive) heart failure	ICD10CM	1	I50.32		Chronic diastolic (congestive) heart failure	IMO	GHS-PACDC	IMO_2015_R	ACTIVE	Y	1900-01-01	9999-12-31	2015-07-10
Diagnosis: ICD10 Code, I00-I99, Define	I00-I99	Diseases of the circulatory system	957527	Heart failure due to valvular disease, acute on chronic, diastolic (HCC)	ICD10CM	1	I50.33	I50.33,I38	Acute on chronic diastolic (congestive) heart failure	IMO	GHS-PACDC	IMO_2015_R	ACTIVE	N	1900-01-01	9999-12-31	2015-07-10
Diagnosis: ICD10 Code, I00-I99, Define	I00-I99	Diseases of the circulatory system	956490	Congestive heart failure, NYHA class 4, acute on chronic, diastolic (HCC)	ICD10CM	1	I50.33		Acute on chronic diastolic (congestive) heart failure	IMO	GHS-PACDC	IMO_2015_R	ACTIVE	N	1900-01-01	9999-12-31	2015-07-10
Diagnosis: ICD10 Code, I00-I99, Define	I00-I99	Diseases of the circulatory system	136065	Acute on chronic diastolic heart failure (HCC)	ICD10CM	1	I50.33		Acute on chronic diastolic (congestive) heart failure	IMO	GHS-PACDC	DX_LEGACY	ACTIVE	N	1900-01-01	9999-12-31	2015-07-10
Diagnosis: ICD10 Code, I00-I99, Define	I00-I99	Diseases of the circulatory system	499498	Acute on chronic congestive heart failure with right ventricular diastolic dysfunction (HCC)	ICD10CM	1	I50.33		Acute on chronic diastolic (congestive) heart failure	IMO	GHS-PACDC	IMO_2015_R	ACTIVE	N	1900-01-01	2017-10-14	2015-07-10
Diagnosis: ICD10 Code, I00-I99, Define	I00-I99	Diseases of the circulatory system	956185	Stage C diastolic heart failure, acute on chronic (HCC)	ICD10CM	1	I50.33		Acute on chronic diastolic (congestive) heart failure	IMO	GHS-PACDC	IMO_2015_R	ACTIVE	N	1900-01-01	9999-12-31	2015-07-10
Diagnosis: ICD10 Code, I00-I99, Define	I00-I99	Diseases of the circulatory system	519604	Acute on chronic diastolic congestive heart failure due to valvular disease (HCC)	ICD10CM	1	I50.33	I50.33,I38	Acute on chronic diastolic (congestive) heart failure	IMO	GHS-PACDC	IMO_2015_R	ACTIVE	N	1900-01-01	9999-12-31	2015-07-10
Diagnosis: ICD10 Code, I00-I99, Define	I00-I99	Diseases of the circulatory system	958945	Congestive heart failure, NYHA class IV, acute on chronic, diastolic (HCC)	ICD10CM	1	I50.33		Acute on chronic diastolic (congestive) heart failure	IMO	GHS-PACDC	IMO_2015_R	ACTIVE	N	1900-01-01	9999-12-31	2015-07-10
Diagnosis: ICD10 Code, I00-I99, Define	I00-I99	Diseases of the circulatory system	383688	Benign hypertensive kidney and heart disease with congestive heart failure with combined syste	ICD10CM	2	I50.40	I13.0,I50.40,N18.9	Unspecified combined systolic (congestive) and diastolic	IMO	GHS-PACDC	DX_LEGACY	ACTIVE	N	1900-01-01	9999-12-31	2015-07-10
Diagnosis: ICD10 Code, I00-I99, Define	I00-I99	Diseases of the circulatory system	266814	Combined congestive systolic and diastolic heart failure (HCC)	ICD10CM	1	I50.40		Unspecified combined systolic (congestive) and diastolic	IMO	GHS-PACDC	DX_LEGACY	ACTIVE	N	1900-01-01	9999-12-31	2015-07-10
Diagnosis: ICD10 Code, I00-I99, Define	I00-I99	Diseases of the circulatory system	491053	Malignant essential hypertension with combined systolic and diastolic NYHA class 2 congestive I	ICD10CM	2	I50.40	I10,I50.40	Unspecified combined systolic (congestive) and diastolic	IMO	GHS-PACDC	IMO_2015_R	ACTIVE	N	1900-01-01	2016-10-14	2015-07-10
Diagnosis: ICD10 Code, I00-I99, Define	I00-I99	Diseases of the circulatory system	1423863	Combined systolic and diastolic heart failure due to valvular disease, unspecified heart failure cl	ICD10CM	1	I50.40	I50.40,I38	Unspecified combined systolic (congestive) and diastolic	IMO	GHS-PACDC	IMO_2018_R	ACTIVE	N	1900-01-01	9999-12-31	2017-10-30
Diagnosis: ICD10 Code, I00-I99, Define	I00-I99	Diseases of the circulatory system	1417000	Combined systolic and diastolic congestive heart failure, NYHA class 4, unspecified congestive h	ICD10CM	1	I50.40		Unspecified combined systolic (congestive) and diastolic	IMO	GHS-PACDC	IMO_2018_R	ACTIVE	N	1900-01-01	9999-12-31	2017-10-30
Diagnosis: ICD10 Code, I00-I99, Define	I00-I99	Diseases of the circulatory system	1416994	Combined systolic and diastolic congestive heart failure, NYHA class 2, unspecified congestive h	ICD10CM	1	I50.40		Unspecified combined systolic (congestive) and diastolic	IMO	GHS-PACDC	IMO_2018_R	ACTIVE	N	1900-01-01	9999-12-31	2017-10-30
Diagnosis: ICD10 Code, I00-I99, Define	I00-I99	Diseases of the circulatory system	958797	CHF NYHA class III, unspecified failure chronicity, combined (HCC)	ICD10CM	1	I50.40		Unspecified combined systolic (congestive) and diastolic	IMO	GHS-PACDC	IMO_2015_R	ACTIVE	N	1900-01-01	9999-12-31	2015-07-10
Diagnosis: ICD10 Code, I00-I99, Define	I00-I99	Diseases of the circulatory system	517651	Severe combined systolic and diastolic congestive heart failure (HCC)	ICD10CM	1	I50.40		Unspecified combined systolic (congestive) and diastolic	IMO	GHS-PACDC	IMO_2015_R	ACTIVE	N	1900-01-01	9999-12-31	2015-07-10
Diagnosis: ICD10 Code, I00-I99, Define	I00-I99	Diseases of the circulatory system	1365578	Unsp combined systolic and diastolic (congestive) hrt fail (HCC)	ICD10CM	1	I50.40		Unspecified combined systolic (congestive) and diastolic	IMO	GHS-PACDC	IMO_2018_R	ACTIVE	N	1900-01-01	9999-12-31	2017-10-30
Diagnosis: ICD10 Code, I00-I99, Define	I00-I99	Diseases of the circulatory system	954828	CHF NYHA class II, acute, combined (HCC)	ICD10CM	1	I50.41		Acute combined systolic (congestive) and diastolic (cong	IMO	GHS-PACDC	IMO_2015_R	ACTIVE	N	1900-01-01	9999-12-31	2015-07-10
Diagnosis: ICD10 Code, I00-I99, Define	I00-I99	Diseases of the circulatory system	963969	CHF NYHA class I, acute, combined (HCC)	ICD10CM	1	I50.41		Acute combined systolic (congestive) and diastolic (cong	IMO	GHS-PACDC	IMO_2015_R	ACTIVE	N	1900-01-01	9999-12-31	2015-07-10
Diagnosis: ICD10 Code, I00-I99, Define	I00-I99	Diseases of the circulatory system	385288	Acute combined systolic and diastolic congestive heart failure, NYHA class 2 (HCC)	ICD10CM	1	I50.41		Acute combined systolic (congestive) and diastolic (cong	IMO	GHS-PACDC	DX_LEGACY	ACTIVE	N	1900-01-01	9999-12-31	2015-07-10
Diagnosis: ICD10 Code, I00-I99, Define	I00-I99	Diseases of the circulatory system	386695	Acute combined systolic and diastolic CHF, NYHA class 2 (HCC)	ICD10CM	1	I50.41		Acute combined systolic (congestive) and diastolic (cong	IMO	GHS-PACDC	DX_LEGACY	ACTIVE	N	1900-01-01	9999-12-31	2015-07-10
Diagnosis: ICD10 Code, I00-I99, Define	I00-I99	Diseases of the circulatory system	960334	Congestive heart failure, NYHA class 1, acute, combined (HCC)	ICD10CM	1	I50.41		Acute combined systolic (congestive) and diastolic (cong	IMO	GHS-PACDC	IMO_2015_R	ACTIVE	N	1900-01-01	9999-12-31	2015-07-10
Diagnosis: ICD10 Code, I00-I99, Define	I00-I99	Diseases of the circulatory system	963121	CHF (NYHA class II, ACC/AHA stage C), acute, combined	ICD10CM	1	I50.41		Acute combined systolic (congestive) and diastolic (cong	IMO	GHS-PACDC	IMO_2015_R	DELETED	Y	1900-01-01	9999-12-31	2015-07-10
Diagnosis: ICD10 Code, I00-I99, Define	I00-I99	Diseases of the circulatory system	955613	CHF (congestive heart failure), NYHA class I, acute, combined (HCC)	ICD10CM	1	I50.41		Acute combined systolic (congestive) and diastolic (cong	IMO	GHS-PACDC	IMO_2015_R	ACTIVE	N	1900-01-01	9999-12-31	2015-07-10
Diagnosis: ICD10 Code, I00-I99, Define	I00-I99	Diseases of the circulatory system	1365635	Acute combined systolic and diastolic (congestive) hrt fail (HCC)	ICD10CM	1	I50.41		Acute combined systolic (congestive) and diastolic (cong	IMO	GHS-PACDC	IMO_2018_R	ACTIVE	N	1900-01-01	9999-12-31	2017-10-30
Diagnosis: ICD10 Code, I00-I99, Define	I00-I99	Diseases of the circulatory system	385315	Chronic combined systolic and diastolic congestive heart failure, NYHA class 4 (HCC)	ICD10CM	1	I50.42		Chronic combined systolic (congestive) and diastolic (cor	IMO	GHS-PACDC	DX_LEGACY	ACTIVE	N	1900-01-01	9999-12-31	2015-07-10
Diagnosis: ICD10 Code, I00-I99, Define	I00-I99	Diseases of the circulatory system	1265696	Chronic combined systolic and diastolic CHF, NYHA class 2 and ACA/AHA stage C (HCC)	ICD10CM	1	I50.42		Chronic combined systolic (congestive) and diastolic (cor	IMO	GHS-PACDC	IMO_2017_R	ACTIVE	N	1900-01-01	9999-12-31	2016-09-30
Diagnosis: ICD10 Code, I00-I99, Define	I00-I99	Diseases of the circulatory system	960068	Class 2 congestive heart failure, chronic, combined (HCC)	ICD10CM	1	I50.42		Chronic combined systolic (congestive) and diastolic (cor	IMO	GHS-PACDC	IMO_2015_R	ACTIVE	N	1900-01-01	9999-12-31	2015-07-10
Diagnosis: ICD10 Code, I00-I99, Define	I00-I99	Diseases of the circulatory system	954057	Class 4 congestive heart failure, acute on chronic, combined (HCC)	ICD10CM	1	I50.43		Acute on chronic combined systolic (congestive) and dia	IMO	GHS-PACDC	IMO_2015_R	ACTIVE	N	1900-01-01	9999-12-31	2015-07-10
Diagnosis: ICD10 Code, I00-I99, Define	I00-I99	Diseases of the circulatory system	956514	CHF (NYHA class II, ACC/AHA stage C), acute on chronic, combined	ICD10CM	1	I50.43		Acute on chronic combined systolic (congestive) and dia	IMO	GHS-PACDC	IMO_2015_R	DELETED	Y	1900-01-01	9999-12-31	2015-07-10
Diagnosis: ICD10 Code, I00-I99, Define	I00-I99	Diseases of the circulatory system	385318	Acute on chronic combined systolic and diastolic congestive heart failure, NYHA class 1 (HCC)	ICD10CM	1	I50.43		Acute on chronic combined systolic (congestive) and dia	IMO	GHS-PACDC	DX_LEGACY	ACTIVE	N	1900-01-01	9999-12-31	2015-07-10
Diagnosis: ICD10 Code, I00-I99, Define	I00-I99	Diseases of the circulatory system	385332	Acute on chronic combined systolic and diastolic heart failure, NYHA class 4 (HCC)	ICD10CM	1	I50.43		Acute on chronic combined systolic (congestive) and dia	IMO	GHS-PACDC	DX_LEGACY	ACTIVE	N	1900-01-01	9999-12-31	2015-07-10
Diagnosis: ICD10 Code, I00-I99, Define	I00-I99	Diseases of the circulatory system	385321	Acute on chronic combined systolic and diastolic heart failure, NYHA class 1 (HCC)	ICD10CM	1	I50.43		Acute on chronic combined systolic (congestive) and dia	IMO	GHS-PACDC	DX_LEGACY	ACTIVE	N	1900-01-01	9999-12-31	2015-07-10
Diagnosis: ICD10 Code, I00-I99, Define	I00-I99	Diseases of the circulatory system	954792	Congestive heart failure, NYHA class IV, acute on chronic, combined (HCC)	ICD10CM	1	I50.43		Acute on chronic combined systolic (congestive) and dia	IMO	GHS-PACDC	IMO_2015_R	ACTIVE	N	1900-01-01	9999-12-31	2015-07-10
Diagnosis: ICD10 Code, I00-I99, Define	I00-I99	Diseases of the circulatory system	958452	CHF due to valvular disease, acute on chronic, combined	ICD10CM	1	I50.43	I50.43,I38	Acute on chronic combined systolic (congestive) and dia	IMO	GHS-PACDC	IMO_2015_R	DELETED	Y	1900-01-01	9999-12-31	2015-07-10
Diagnosis: ICD10 Code, I00-I99, Define	I00-I99	Diseases of the circulatory system	955367	Congestive heart failure, NYHA class I, acute on chronic, combined (HCC)	ICD10CM	1	I50.43		Acute on chronic combined systolic (congestive) and dia	IMO	GHS-PACDC	IMO_2015_R	ACTIVE	N	1900-01-01	9999-12-31	2015-07-10
Diagnosis: ICD10 Code, I00-I99, Define	I00-I99	Diseases of the circulatory system	21655	Bernheim's syndrome (HCC)	ICD10CM	1	I50.810		Right heart failure, unspecified	IMO	GHS-PACDC	DX_LEGACY	ACTIVE	N	2017-09-17	9999-12-31	2017-10-30
Diagnosis: ICD10 Code, I00-I99, Define	I00-I99	Diseases of the circulatory system	205626	Right-sided heart failure (HCC)	ICD10CM	1	I50.810		Right heart failure, unspecified	IMO	GHS-PACDC	DX_LEGACY	ACTIVE	N	2017-09-17	9999-12-31	2017-10-30
Diagnosis: ICD10 Code, I00-I99, Define	I00-I99	Diseases of the circulatory system	222457	Biventricular HF (heart failure) (HCC)	ICD10CM	1	I50.82		Biventricular heart failure	IMO	GHS-PACDC	DX_LEGACY	ACTIVE	N	2017-09-17	9999-12-31	2017-10-30
Diagnosis: ICD10 Code, I00-I99, Define	I00-I99	Diseases of the circulatory system	4930	Hypertensive heart failure with ESRD (HCC)	ICD10CM	3	I50.9	I13.2,N18.6,I50.9	Heart failure, unspecified	IMO	GHS-PACDC	DX_LEGACY	ACTIVE	N	1900-01-01	2017-10-14	2015-07-10
Diagnosis: ICD10 Code, I00-I99, Define	I00-I99	Diseases of the circulatory system	249492	Heart & renal disease, hypertensive benign, heart fail/chr renal dis (HCC)	ICD10CM	2	I50.9	I13.0,I50.9,N18.9	Heart failure, unspecified	IMO	GHS-PACDC	DX_LEGACY	ACTIVE	N	2016-09-17	2017-10-14	2016-09-30

Diagnosis: ICD10 Code, I00-I99, Define	I00-I99	Diseases of the circulatory system	971579	Malignant hypertension with renal failure and congestive heart failure, stage 1-4 or unspecified	ICD10CM	2	I50.9	I12.9,I50.9	Heart failure, unspecified	IMO	GHS-PACDC	IMO_2015_R	DELETED	Y	1900-01-01	9999-12-31	2015-07-10
Diagnosis: ICD10 Code, I00-I99, Define	I00-I99	Diseases of the circulatory system	384211	Hypertensive kidney and heart disease with CHF, stage I (HCC)	ICD10CM	2	I50.9	I13.0,I50.9,N18.1	Heart failure, unspecified	IMO	GHS-PACDC	DX_LEGACY	ACTIVE	N	1900-01-01	9999-12-31	2015-07-10
Diagnosis: ICD10 Code, I00-I99, Define	I00-I99	Diseases of the circulatory system	105194	Cardiac failure right (HCC)	ICD10CM	1	I50.9		Heart failure, unspecified	IMO	GHS-PACDC	DX_LEGACY	ACTIVE	N	1900-01-01	2017-10-14	2015-07-10
Diagnosis: ICD10 Code, I00-I99, Define	I00-I99	Diseases of the circulatory system	360002	Hypertensive heart and chronic kidney disease with heart failure and stage 1 through stage 4 ch	ICD10CM	2	I50.9	I13.0,I50.9,N18.9	Heart failure, unspecified	IMO	GHS-PACDC	DX_LEGACY	ACTIVE	N	1900-01-01	2017-10-14	2015-07-10
Diagnosis: ICD10 Code, I00-I99, Define	I00-I99	Diseases of the circulatory system	222428	Benign hypertensive heart disease with CHF (congestive heart failure) (HCC)	ICD10CM	2	I50.9	I11.0,I50.9	Heart failure, unspecified	IMO	GHS-PACDC	DX_LEGACY	ACTIVE	N	1900-01-01	9999-12-31	2015-07-10
Diagnosis: ICD10 Code, I00-I99, Define	I00-I99	Diseases of the circulatory system	339562	Hypertensive heart and kidney disease with heart failure and with chronic kidney disease stage	ICD10CM	2	I50.9	I13.0,I50.9,N18.3	Heart failure, unspecified	IMO	GHS-PACDC	DX_LEGACY	ACTIVE	N	1900-01-01	2016-10-14	2015-07-10
Diagnosis: ICD10 Code, I00-I99, Define	I00-I99	Diseases of the circulatory system	423922	Hypertensive heart and renal disease with both (congestive) heart failure and renal failure (HCC	ICD10CM	3	I50.9	I13.2,N19,I50.9	Heart failure, unspecified	IMO	GHS-PACDC	IMO_2015_R	ACTIVE	N	1900-01-01	9999-12-31	2015-07-10
Diagnosis: ICD10 Code, I00-I99, Define	I00-I99	Diseases of the circulatory system	344189	Hypertensive heart failure, malignant, with stage 1-4 CKD, acute management	ICD10CM	3	I50.9	I13.0,N18.9,I50.9	Heart failure, unspecified	IMO	GHS-PACDC	DX_LEGACY	DELETED	Y	1900-01-01	2017-10-14	2015-07-10
Diagnosis: ICD10 Code, I00-I99, Define	I00-I99	Diseases of the circulatory system	222442	Hypertensive heart and kidney disease with HF and with CKD stage I (HCC)	ICD10CM	3	I50.9	I13.0,N18.1,I50.9	Heart failure, unspecified	IMO	GHS-PACDC	DX_LEGACY	ACTIVE	N	1900-01-01	2016-10-14	2015-07-10
Diagnosis: ICD10 Code, I00-I99, Define	I00-I99	Diseases of the circulatory system	384453	Malignant essential hypertension with CHF, NYHA class 2 (HCC)	ICD10CM	2	I50.9	I10,I50.9	Heart failure, unspecified	IMO	GHS-PACDC	DX_LEGACY	ACTIVE	N	1900-01-01	2016-10-14	2015-07-10
Diagnosis: ICD10 Code, I00-I99, Define	I00-I99	Diseases of the circulatory system	1423866	Heart failure due to valvular disease, unspecified heart failure type (HCC)	ICD10CM	1	I50.9	I50.9,I38	Heart failure, unspecified	IMO	GHS-PACDC	IMO_2018_R	ACTIVE	N	1900-01-01	9999-12-31	2017-10-30
Diagnosis: ICD10 Code, I00-I99, Define	I00-I99	Diseases of the circulatory system	343341	Malignant hypertensive heart and renal disease with heart failure and chronic kidney disease st	ICD10CM	3	I50.9	I13.0,N18.3,I50.9	Heart failure, unspecified	IMO	GHS-PACDC	DX_LEGACY	ACTIVE	N	1900-01-01	2017-10-14	2015-07-10
Diagnosis: ICD10 Code, I00-I99, Define	I00-I99	Diseases of the circulatory system	418861	Hypertensive heart and kidney disease with heart failure (HCC)	ICD10CM	2	I50.9	I30.0,I50.9,N18.9	Heart failure, unspecified	IMO	GHS-PACDC	DX_LEGACY	ACTIVE	N	1900-01-01	2016-05-07	2015-07-10
Diagnosis: ICD10 Code, I00-I99, Define	I00-I99	Diseases of the circulatory system	343310	Malignant hypertensive heart and renal disease with congestive heart failure and renal failure (I	ICD10CM	2	I50.9	I13.0,I50.9,N19	Heart failure, unspecified	IMO	GHS-PACDC	DX_LEGACY	ACTIVE	N	1900-01-01	9999-12-31	2015-07-10
Diagnosis: ICD10 Code, I00-I99, Define	I00-I99	Diseases of the circulatory system	134160	Congestive heart failure, NYHA class III (HCC)	ICD10CM	1	I50.9		Heart failure, unspecified	IMO	GHS-PACDC	DX_LEGACY	ACTIVE	N	1900-01-01	9999-12-31	2015-07-10
Diagnosis: ICD10 Code, I00-I99, Define	I00-I99	Diseases of the circulatory system	424254	Hyp heart and kidney disease w/ failure (HCC)	ICD10CM	2	I50.9	I13.0,I50.9,N18.9	Heart failure, unspecified	IMO	GHS-PACDC	IMO_2015_R	ACTIVE	N	1900-01-01	2017-10-14	2015-07-10
Diagnosis: ICD10 Code, I00-I99, Define	I00-I99	Diseases of the circulatory system	298896	CHF, chronic (HCC)	ICD10CM	1	I50.9		Heart failure, unspecified	IMO	GHS-PACDC	DX_LEGACY	ACTIVE	N	1900-01-01	9999-12-31	2015-07-10
Diagnosis: ICD10 Code, I00-I99, Define	I00-I99	Diseases of the circulatory system	971075	Cardiovascular renal disease, stage 1-4 or unspecified chronic kidney disease, with heart failure	ICD10CM	2	I50.9	I13.0,I50.9	Heart failure, unspecified	IMO	GHS-PACDC	IMO_2015_R	ACTIVE	N	2016-04-10	2016-10-14	2015-07-10
Diagnosis: ICD10 Code, I00-I99, Define	I00-I99	Diseases of the circulatory system	490435	NYHA class 1 heart failure with preserved ejection fraction (HCC)	ICD10CM	1	I50.9		Heart failure, unspecified	IMO	GHS-PACDC	IMO_2015_R	ACTIVE	N	1900-01-01	2017-10-14	2015-07-10
Diagnosis: ICD10 Code, I00-I99, Define	I00-I99	Diseases of the circulatory system	17923	Heart failure due to valvular disease (HCC)	ICD10CM	1	I50.9	I50.9,I38	Heart failure, unspecified	IMO	GHS-PACDC	DX_LEGACY	ACTIVE	N	1900-01-01	9999-12-31	2015-07-10
Diagnosis: ICD10 Code, I00-I99, Define	I00-I99	Diseases of the circulatory system	153702	Unspecified hypertensive heart and kidney disease with heart failure and with chronic kidney di	ICD10CM	2	I50.9	I13.0,I50.9,N18.9	Heart failure, unspecified	IMO	GHS-PACDC	DX_LEGACY	ACTIVE	Y	1900-01-01	9999-12-31	2017-04-14
Diagnosis: ICD10 Code, I00-I99, Define	I00-I99	Diseases of the circulatory system	958468	CHF (congestive heart failure), NYHA class III, unspecified failure chronicity, unspecified type (H	ICD10CM	1	I50.9		Heart failure, unspecified	IMO	GHS-PACDC	IMO_2015_R	ACTIVE	N	1900-01-01	9999-12-31	2015-07-10
Diagnosis: ICD10 Code, I00-I99, Define	I00-I99	Diseases of the circulatory system	428518	Malignant hypertensive heart disease requiring acute intensive management, with heart failure	ICD10CM	2	I50.9	I11.0,I50.9	Heart failure, unspecified	IMO	GHS-PACDC	IMO_2015_R	ACTIVE	N	1900-01-01	2017-10-14	2015-07-10
Diagnosis: ICD10 Code, I00-I99, Define	I00-I99	Diseases of the circulatory system	384552	Malignant essential hypertension with congestive heart failure (HCC)	ICD10CM	2	I50.9	I10,I50.9	Heart failure, unspecified	IMO	GHS-PACDC	DX_LEGACY	ACTIVE	N	1900-01-01	2016-10-14	2015-07-10
Diagnosis: ICD10 Code, I00-I99, Define	I00-I99	Diseases of the circulatory system	487899	Death due to congestive heart failure (HCC)	ICD10CM	1	I50.9		Heart failure, unspecified	IMO	GHS-PACDC	IMO_2015_R	ACTIVE	N	1900-01-01	9999-12-31	2015-07-10
Diagnosis: ICD10 Code, I00-I99, Define	I00-I99	Diseases of the circulatory system	491508	Right-sided congestive heart failure (HCC)	ICD10CM	1	I50.9		Heart failure, unspecified	IMO	GHS-PACDC	IMO_2015_R	ACTIVE	N	1900-01-01	2017-10-14	2015-07-10
Diagnosis: ICD10 Code, I00-I99, Define	I00-I99	Diseases of the circulatory system	958866	Heart and kidney disease due to severe high blood pressure, stage 5 chronic kidney disease or e	ICD10CM	3	I50.9	I13.2,N18.9,I50.9	Heart failure, unspecified	IMO	GHS-PACDC	IMO_2015_R	ACTIVE	N	1900-01-01	2016-10-14	2015-07-10
Diagnosis: ICD10 Code, I00-I99, Define	I00-I99	Diseases of the circulatory system	338938	Hypertensive heart and renal disease with heart failure and renal failure (HCC)	ICD10CM	3	I50.9	I13.2,N19,I50.9	Heart failure, unspecified	IMO	GHS-PACDC	DX_LEGACY	ACTIVE	N	1900-01-01	9999-12-31	2015-07-10
Diagnosis: ICD10 Code, I00-I99, Define	I00-I99	Diseases of the circulatory system	205532	Acute exacerbation of CHF (congestive heart failure) (HCC)	ICD10CM	1	I50.9		Heart failure, unspecified	IMO	GHS-PACDC	DX_LEGACY	ACTIVE	N	1900-01-01	9999-12-31	2015-07-10
Diagnosis: ICD10 Code, I00-I99, Define	I00-I99	Diseases of the circulatory system	344096	Systolic heart failure secondary to hypertrophic obstructive cardiomyopathy (HCC)	ICD10CM	1	I50.9	I50.9,I42.1	Heart failure, unspecified	IMO	GHS-PACDC	DX_LEGACY	ACTIVE	N	1900-01-01	2016-10-14	2015-07-10
Diagnosis: ICD10 Code, I00-I99, Define	I00-I99	Diseases of the circulatory system	384196	Benign hypertensive heart disease with CHF, NYHA class 3 (HCC)	ICD10CM	2	I50.9	I11.0,I50.9	Heart failure, unspecified	IMO	GHS-PACDC	DX_LEGACY	ACTIVE	N	1900-01-01	9999-12-31	2015-07-10
Diagnosis: ICD10 Code, I00-I99, Define	I00-I99	Diseases of the circulatory system	505859	Idiopathic heart failure (HCC)	ICD10CM	1	I50.9		Heart failure, unspecified	IMO	GHS-PACDC	IMO_2015_R	ACTIVE	N	1900-01-01	9999-12-31	2015-07-10
Diagnosis: ICD10 Code, I00-I99, Define	I00-I99	Diseases of the circulatory system	490578	Heart failure with preserved ejection fraction, NYHA class IV (HCC)	ICD10CM	1	I50.9		Heart failure, unspecified	IMO	GHS-PACDC	IMO_2015_R	ACTIVE	N	1900-01-01	2017-10-14	2015-07-10
Diagnosis: ICD10 Code, I00-I99, Define	I00-I99	Diseases of the circulatory system	231112	Malignant hypertensive heart and kidney disease with HF and CKD (HCC)	ICD10CM	3	I50.9	I13.0,N18.9,I50.9	Heart failure, unspecified	IMO	GHS-PACDC	DX_LEGACY	ACTIVE	N	1900-01-01	2017-10-14	2015-07-10
Diagnosis: ICD10 Code, I00-I99, Define	I00-I99	Diseases of the circulatory system	153675	Malignant hypertensive heart and kidney disease with heart failure and chronic kidney disease s	ICD10CM	2	I50.9	I13.2,I50.9	Heart failure, unspecified	IMO	GHS-PACDC	DX_LEGACY	ACTIVE	Y	1900-01-01	9999-12-31	2017-04-14
Diagnosis: ICD10 Code, I00-I99, Define	I00-I99	Diseases of the circulatory system	384867	Benign hypertensive kidney and heart disease with congestive heart failure, NYHA class 1 (HCC)	ICD10CM	2	I50.9	I13.0,I50.9,N18.9	Heart failure, unspecified	IMO	GHS-PACDC	DX_LEGACY	ACTIVE	N	1900-01-01	9999-12-31	2015-07-10
Diagnosis: ICD10 Code, I00-I99, Define	I00-I99	Diseases of the circulatory system	159228	Pre-operative cardiovascular examination, symptomatic CHF (HCC)	ICD10CM	2	I50.9	Z01.810,I50.9	Heart failure, unspecified	IMO	GHS-PACDC	DX_LEGACY	ACTIVE	N	2017-09-17	9999-12-31	2017-10-30
Diagnosis: ICD10 Code, I00-I99, Define	I00-I99	Diseases of the circulatory system	537614	Cardiac septal defect, acquired	ICD10CM	1	I51.0		Cardiac septal defect, acquired	IMO	GHS-PACDC	IMO_2015_R	ACTIVE	Y	1900-01-01	9999-12-31	2015-07-10
Diagnosis: ICD10 Code, I00-I99, Define	I00-I99	Diseases of the circulatory system	1304194	Thrombosis of atrium without antecedent myocardial infarction	ICD10CM	1	I51.3		Intracardiac thrombosis, not elsewhere classified	IMO	GHS-PACDC	IMO_2017_R	ACTIVE	N	1900-01-01	9999-12-31	2017-04-14
Diagnosis: ICD10 Code, I00-I99, Define	I00-I99	Diseases of the circulatory system	405747	Mural thrombus of left ventricular apex following MI (HCC)	ICD10CM	1	I51.3	I51.3,I21.3	Intracardiac thrombosis, not elsewhere classified	IMO	GHS-PACDC	DX_LEGACY	ACTIVE	N	1900-01-01	2017-04-27	2015-07-10
Diagnosis: ICD10 Code, I00-I99, Define	I00-I99	Diseases of the circulatory system	405745	Mural thrombus of left ventricle following MI (HCC)	ICD10CM	1	I51.3	I51.3,I21.3	Intracardiac thrombosis, not elsewhere classified	IMO	GHS-PACDC	DX_LEGACY	ACTIVE	N	1900-01-01	2017-10-14	2015-07-10
Diagnosis: ICD10 Code, I00-I99, Define	I00-I99	Diseases of the circulatory system	405763	Ventricular mural thrombus without MI	ICD10CM	1	I51.3		Intracardiac thrombosis, not elsewhere classified	IMO	GHS-PACDC	DX_LEGACY	ACTIVE	N	2016-09-17	9999-12-31	2016-09-30
Diagnosis: ICD10 Code, I00-I99, Define	I00-I99	Diseases of the circulatory system	1304204	Thrombosis of left atrium without antecedent myocardial infarction	ICD10CM	1	I51.3		Intracardiac thrombosis, not elsewhere classified	IMO	GHS-PACDC	IMO_2017_R	ACTIVE	N	1900-01-01	9999-12-31	2017-04-14
Diagnosis: ICD10 Code, I00-I99, Define	I00-I99	Diseases of the circulatory system	405395	Left ventricular mural thrombus without myocardial infarction	ICD10CM	1	I51.3		Intracardiac thrombosis, not elsewhere classified	IMO	GHS-PACDC	DX_LEGACY	ACTIVE	N	2016-09-17	9999-12-31	2016-09-30
Diagnosis: ICD10 Code, I00-I99, Define	I00-I99	Diseases of the circulatory system	1252853	Chronic myocarditis, unspecified myocarditis type (HCC)	ICD10CM	1	I51.4		Myocarditis, unspecified	IMO	GHS-PACDC	IMO_2016_R	ACTIVE	N	1900-01-01	9999-12-31	2016-06-02
Diagnosis: ICD10 Code, I00-I99, Define	I00-I99	Diseases of the circulatory system	1111070	Chronic infective myocarditis (HCC)	ICD10CM	1	I51.4		Myocarditis, unspecified	IMO	GHS-PACDC	IMO_2016_R	ACTIVE	N	1900-01-01	9999-12-31	2016-06-02
Diagnosis: ICD10 Code, I00-I99, Define	I00-I99	Diseases of the circulatory system	1268862	Myocarditis of mother during pregnancy (HCC)	ICD10CM	2	I51.4	O99.419,I51.4	Myocarditis, unspecified	IMO	GHS-PACDC	IMO_2017_R	ACTIVE	N	1900-01-01	9999-12-31	2016-09-30
Diagnosis: ICD10 Code, I00-I99, Define	I00-I99	Diseases of the circulatory system	48951	Heart size increased	ICD10CM	1	I51.7		Cardiomegaly	IMO	GHS-PACDC	DX_LEGACY	ACTIVE	N	1900-01-01	9999-12-31	2015-07-10
Diagnosis: ICD10 Code, I00-I99, Define	I00-I99	Diseases of the circulatory system	165808	Enlarged LV	ICD10CM	1	I51.7		Cardiomegaly	IMO	GHS-PACDC	DX_LEGACY	ACTIVE	N	1900-01-01	9999-12-31	2015-07-10
Diagnosis: ICD10 Code, I00-I99, Define	I00-I99	Diseases of the circulatory system	302713	Ventricular enlargement, right	ICD10CM	1	I51.7		Cardiomegaly	IMO	GHS-PACDC	DX_LEGACY	ACTIVE	N	1900-01-01	9999-12-31	2015-07-10
Diagnosis: ICD10 Code, I00-I99, Define	I00-I99	Diseases of the circulatory system	499630	Mild concentric right ventricular hypertrophy (RVH)	ICD10CM	1	I51.7		Cardiomegaly	IMO	GHS-PACDC	IMO_2015_R	ACTIVE	N	1900-01-01	9999-12-31	2015-07-10
Diagnosis: ICD10 Code, I00-I99, Define	I00-I99	Diseases of the circulatory system	1274634	Familial idiopathic dilation of right atrium	ICD10CM	1	I51.7		Cardiomegaly	IMO	GHS-PACDC	IMO_2017_R	ACTIVE	N	1900-01-01	9999-12-31	2016-09-30
Diagnosis: ICD10 Code, I00-I99, Define	I00-I99	Diseases of the circulatory system	374121	Mild pulmonic regurgitation and RV dilation by prior echocardiogram	ICD10CM	2	I51.7	I37.1,I51.7	Cardiomegaly	IMO	GHS-PACDC	DX_LEGACY	ACTIVE	N	1900-01-01	9999-12-31	2015-07-10
Diagnosis: ICD10 Code, I00-I99, Define	I00-I99	Diseases of the circulatory system	253078	Enlargement of right atrium	ICD10CM	1	I51.7		Cardiomegaly	IMO	GHS-PACDC	DX_LEGACY	ACTIVE	N	1900-01-01	9999-12-31	2015-07-10
Diagnosis: ICD10 Code, I00-I99, Define	I00-I99	Diseases of the circulatory system	159013	Bulging of heart wall	ICD10CM	1	I51.89		Other ill-defined heart diseases	IMO	GHS-PACDC	DX_LEGACY	ACTIVE	N	1900-01-01	9999-12-31	2015-07-10
Diagnosis: ICD10 Code, I00-I99, Define	I00-I99	Diseases of the circulatory system	59803	Papillary muscle dysfunction	ICD10CM	1	I51.89		Other ill-defined heart diseases	IMO	GHS-PACDC	DX_LEGACY	ACTIVE	N	1900-01-01	9999-12-31	2015-07-10
Diagnosis: ICD10 Code, I00-I99, Define	I00-I99	Diseases of the circulatory system	493100	Abscess of wall of right ventricle	ICD10CM	1	I51.89		Other ill-defined heart diseases	IMO	GHS-PACDC	IMO_2015_R	ACTIVE	N	1900-01-01	9999-12-31	2015-07-10
Diagnosis: ICD10 Code, I00-I99, Define	I00-I99	Diseases of the circulatory system	493201	Abscess of common atrioventricular valve	ICD10CM	1	I51.89	I51.89,Q21.2	Other ill-defined heart diseases	IMO	GHS-PACDC	IMO_2015_R	ACTIVE	N	1900-01-01	9999-12-31	2015-07-10
Diagnosis: ICD10 Code, I00-I99, Define	I00-I99	Diseases of the circulatory system	1094902	Abscess of heart at site of prosthetic valve, subsequent encounter	ICD10CM	2	I51.89	T82.6XXD,I51.89	Other ill-defined heart diseases	IMO	GHS-PACDC	IMO_2015_R	ACTIVE	N	1900-01-01	9999-12-31	2015-07-10
Diagnosis: ICD10 Code, I00-I99, Define	I00-I99	Diseases of the circulatory system	499599	Dynamic right ventricular outflow obstruction	ICD10CM	1	I51.89		Other ill-defined heart diseases	IMO	GHS-PACDC	IMO_2015_R	ACTIVE	N	1900-01-01	9999-12-31	2015-07-10
Diagnosis: ICD10 Code, I00-I99, Define	I00-I99	Diseases of the circulatory system	1137418	Right ventricular (RV) hypertension	ICD10CM	1	I51.89		Other ill-defined heart diseases	IMO	GHS-PACDC	IMO_2016_R	DELETED	Y	1900-01-01	9999-12-31	2016-06-02
Diagnosis: ICD10 Code, I00-I99, Define	I00-I99	Diseases of the circulatory system	249614	Pre-operative cardiovascular examination, LVEF < 35%	ICD10CM	1	I51.89	I51.89,Z01.810	Other ill-defined heart diseases	IMO	GHS-PACDC	DX_LEGACY	ACTIVE	N	1900-01-01	2017-10-14	2015-07-10
Diagnosis: ICD10 Code, I00-I99, Define	I00-I99	Diseases of the circulatory system	73432	Papillary muscle atrophy	ICD10CM	1	I51.89		Other ill-defined heart diseases	IMO	GHS-PACDC	DX_LEGACY	ACTIVE	N	1900-01-01	9999-12-31	2015-07-10
Diagnosis: ICD10 Code, I00-I99, Define	I00-I99	Diseases of the circulatory system	430659	Maternal acquired cardiovascular disease complicating pregnancy in first trimester, antepartum	ICD10CM	2	I51.9	O99.411,I51.9	Heart disease, unspecified	IMO	GHS-PACDC	IMO_2015_R	ACTIVE	N	1900-01-01	2017-10-14	2015-07-10
Diagnosis: ICD10 Code, I00-I99, Define	I00-I99	Diseases of the circulatory system	225177	Radiation induced cardiotoxicity	ICD10CM	1	I51.9		Heart disease, unspecified	IMO	GHS-PACDC	DX_LEGACY	ACTIVE	N	1900-01-01	2016-05-07	2015-07-10
Diagnosis: ICD10 Code, I00-I99, Define	I00-I99	Diseases of the circulatory system	408220	Structural disorder of heart	ICD10CM	1	I51.9		Heart disease, unspecified	IMO	GHS-PACDC	DX_LEGACY	ACTIVE	N	1900-01-01	9999-12-31	2015-07-10
Diagnosis: ICD10 Code, I00-I99, Define	I00-I99	Diseases of the circulatory system	1351449	Cleft lip-cleft palate-intestinal malrotation-cardiopathy syndrome	ICD10CM	2	I51.9	Q37.9,I51.9,Q43.3	Heart disease, unspecified	IMO	GHS-PACDC	IMO_2018_R	ACTIVE	N	1900-01-01	9999-12-31	2017-10-30
Diagnosis: ICD10 Code, I00-I99, Define	I00-I99	Diseases of the circulatory system	35163	Heart failure during or resulting from a procedure	ICD10CM	1	I51.9		Heart disease, unspecified	IMO	GHS-PACDC	DX_LEGACY	DELETED	Y	1900-01-01	2016-05-07	2015-07-10
Diagnosis: ICD10 Code, I00-I99, Define	I00-I99	Diseases of the circulatory system	517918	Heart disease in mother affecting pregnancy in third trimester	ICD10CM	2	I51.9	O99.413,I51.9	Heart disease, unspecified	IMO	GHS-PACDC	IMO_2015_R	ACTIVE	N	1900-01-01	9999-12-31	2015-07-10
Diagnosis: ICD10 Code, I00-I99, Define	I00-I99	Diseases of the circulatory system	64632	Cardiac complications	ICD10CM	1	I51.9		Heart disease, unspecified	IMO	GHS-PACDC	DX_LEGACY	ACTIVE	N	1900-01-01	9999-12-31	2015-07-10
Diagnosis: ICD10 Code, I00-I99, Define	I00-I99	Diseases of the circulatory system	500579	Suspected heart disease	ICD10CM	1	I51.9		Heart disease, unspecified	IMO	GHS-PACDC	IMO_2015_R	ACTIVE	N	1900-01-01	9999-12-31	2015-07-10
Diagnosis: ICD10 Code, I00-I99, Define	I00-I99	Diseases of the circulatory system	333288	Heart disease, in mother complicating pregnancy, childbirth or puerperium	ICD10CM	2	I51.9	O99.419,I51.9	Heart disease, unspecified	IMO	GHS-PACDC	DX_LEGACY	DELETED	Y	1900-01-01	9999-12-31	2015-07-10
Diagnosis: ICD10 Code, I00-I99, Define	I00-I99	Diseases of the circulatory system	66762	Myocardial decompensation	ICD10CM	1	I51.9		Heart disease, unspecified	IMO	GHS-PACDC	DX_LEGACY	ACTIVE	N	1900-01-01	9999-12-31	2015-07-10
Diagnosis: ICD10 Code, I00-I99, Define	I00-I99	Diseases of the circulatory system	145695	Systolic dysfunction	ICD10CM	1	I51.9		Heart disease, unspecified	IMO	GHS-PACDC	DX_LEGACY	ACTIVE	N	1900-01-01	9999-12-31	2015-07-10
Diagnosis: ICD10 Code, I00-I99, Define	I00-I99	Diseases of the circulatory system	634017	Other heart disorders in diseases classified elsewhere	ICD10CM	1	I52		Other heart disorders in diseases classified elsewhere	IMO	GHS-PACDC	IMO_2015_R	ACTIVE	N	1900-01-01	9999-12-31	2015-07-10
Diagnosis: ICD10 Code, I00-I99, Define	I00-I99	Diseases of the circulatory system	970187	Subarachnoid hemorrhage from carotid artery aneurysm, left (HCC)	ICD10CM	1	I60.02		Nontraumatic subarachnoid hemorrhage from left carot	IMO	GHS-PACDC	IMO_2015_R	ACTIVE	N	1900-01-01	9999-12-31	2015-07-10
Diagnosis: ICD10 Code, I00-I99, Define	I00-I99	Diseases of the circulatory system	592262	Nontraumatic subarachnoid hemorrhage from left middle cerebral artery	ICD10CM	1	I60.12		Nontraumatic subarachnoid hemorrhage from left midd	IMO	GHS-PACDC	IMO_2015_R	ACTIVE	Y	1900-01-01	9999-12-31	2015-07-10
Diagnosis: ICD10 Code, I00-I99, Define	I00-I99	Diseases of the circulatory system	344699	Nontraumatic subarachnoid hemorrhage from left anterior communicating artery (HCC)	ICD10CM	1	I60.2		Nontraumatic subarachnoid hemorrhage from anterior (	IMO	GHS-PACDC	DX_LEGACY	ACTIVE	N	2016-09-17	9999-12-31	2016-09-30
Diagnosis: ICD10 Code, I00-I99, Define	I00-I99	Diseases of the circulatory system	408214	Subarachnoid hemorrhage from anterior communicating artery aneurysm (HCC)	ICD10CM	1	I60.2		Nontraumatic subarachnoid hemorrhage from anterior (	IMO	GHS-PACDC	DX_LEGACY	ACTIVE	N	2016-09-17	9999-12-31	2016-09-30
Diagnosis: ICD10 Code, I00-I99, Define	I00-I99	Diseases of the circulatory system	1181275	Nontraumatic subarachnoid hemorrhage from unspecified anterior communicating artery	ICD10CM	1	I60.20		ICD CONCEPT NAME NOT AVAILABLE	IMO	GHS-PACDC	IMO_2016_R	DELETED	Y	1900-01-01	9999-12-31	2016-06-02
Diagnosis: ICD10 Code, I00-I99, Define	I00-I99	Diseases of the circulatory system	475452	Subarachnoid hemorrhage from right anterior communicating artery (HCC)	ICD10CM	1	I60.21		ICD CONCEPT NAME NOT AVAILABLE	IMO	GHS-PACDC	IMO_2015_R	ACTIVE	N	1900-01-01	2016-10-14	2015-07-10
Diagnosis: ICD10 Code, I00-I99, Define	I00-I99	Diseases of the circulatory system	589560	Nontraumatic subarachnoid hemorrhage from right anterior communicating artery	ICD10CM	1	I60.21		ICD CONCEPT NAME NOT AVAILABLE	IMO	GHS-PACDC	IMO_2015_R	ACTIVE	Y	1900-01-01	9999-12-31	2015-07-10
Diagnosis: ICD10 Code, I00-I99, Define	I00-I99	Diseases of the circulatory system	876150	Nontraumatic subarachnoid bleed from anterior communicating artery, left	ICD10CM	1	I60.22		ICD CONCEPT NAME NOT AVAILABLE	IMO	GHS-PACDC	IMO_2015_R	DELETED	Y	1900-01-01	9999-12-31	2015-07-10
Diagnosis: ICD10 Code, I00-I99, Define	I00-I99	Diseases of the circulatory system	476790	Subarachnoid hemorrhage from aneurysm of right posterior communicating artery (HCC)	ICD10CM	1	I60.31		Nontraumatic subarachnoid hemorrhage from right post	IMO	GHS-PACDC	IMO_2015_R	ACTIVE	N	1900-01-01	9999-12-31	2015-07-10
Diagnosis: ICD10 Code, I00-I99, Define	I00-I99	Diseases of the circulatory system	1365612	Ntrm subarach hemor from left posterior communicating artery (HCC)	ICD10CM	1	I60.32		Nontraumatic subarachnoid hemorrhage from left poste	IMO	GHS-PACDC	IMO_2018_R	ACTIVE	N	1900-01-01	9999-12-31	2017-10-30
Diagnosis: ICD10 Code, I00-I99, Define	I00-I99	Diseases of the circulatory system	845501	Nontraumatic subarachnoid hemorrhage from vertebral artery, unspecified laterality (HCC)	ICD10CM	1	I60.50		Nontraumatic subarachnoid hemorrhage from unspecifi	IMO	GHS-PACDC	IMO_2015_R	ACTIVE	N	1900-01-01	9999-12-31	2015-07-10
Diagnosis: ICD10 Code, I00-I99, Define	I00-I99	Diseases of the circulatory system	268981	Nontraumatic subarachnoid hemorrhage from right vertebral artery (HCC)	ICD10CM	1	I60.51		Nontraumatic subarachnoid hemorrhage from right vert	IMO	GHS-PACDC	DX_LEGACY	ACTIVE	N	1900-01-01	9999-12-31	2015-07-10
Diagnosis: ICD10 Code, I00-I99, Define	I00-I99	Diseases of the circulatory system	1192016	Nontraumatic subarachnoid hemorrhage from unspecified intracranial artery (HCC)	ICD10CM	1	I60.7		Nontraumatic subarachnoid hemorrhage from unspecifi	IMO	GHS-PACDC	IMO_2016_R	ACTIVE	N	1900-01-01	9999-12-31	2016-06-02
Diagnosis: ICD10 Code, I00-I99, Define	I00-I99	Diseases of the circulatory system	264698	Ruptured congenital berry aneurysm (HCC)	ICD10CM	1	I60.7		Nontraumatic subarachnoid hemorrhage from unspecifi	IMO	GHS-PACDC	DX_LEGACY	ACTIVE	N	1900-01-01	9999-12-31	2015-07-10
Diagnosis: ICD10 Code, I00-I99, Define	I00-I99	Diseases of the circulatory system	130553	Spontaneous subarachnoid hemorrhage (HCC)	ICD10CM	1	I60.8		Other nontraumatic subarachnoid hemorrhage	IMO	GHS-PACDC	DX_LEGACY	ACTIVE	N	1900-01-01	2016-05-07	2015-07-10
Diagnosis: ICD10 Code, I00-I99, Define	I00-I99	Diseases of the circulatory system	343317	Spontaneous presumed perinatal subarachnoid intracranial hemorrhage due to cerebral arterio	ICD10CM	1	I60.9	I60.9,Q28.2	Nontraumatic subarachnoid hemorrhage, unspecified	IMO	GHS-PACDC	DX_LEGACY	ACTIVE	N	2016-09-17	9999-12-31	2016-09-30
Diagnosis: ICD10 Code, I00-I99, Define	I00-I99	Diseases of the circulatory system	225803	Spontaneous presumed perinatal subarachnoid intracranial hemorrhage (HCC)	ICD10CM	2	I60.9	P54.8,I60.9	Nontraumatic subarachnoid hemorrhage, unspecified	IMO	GHS-PACDC	DX_LEGACY	ACTIVE	N	2016-09-17	2017-10-14	2016-09-30
Diagnosis: ICD10 Code, I00-I99, Define	I00-I99	Diseases of the circulatory system	231091	Acute spont subarachnoid intracranial hemorrh d/t cerebral cavernoma (HCC)	ICD10CM	1	I60.9	I60.9,Q28.3	Nontraumatic subarachnoid hemorrhage, unspecified	IMO	GHS-PACDC	DX_LEGACY	ACTIVE	N	1900-01-01	9999-12-31	2015-07-10
Diagnosis: ICD10 Code, I00-I99, Define	I00-I99	Diseases of the circulatory system	1316182	Flaccid hemiplegia due to nontraumatic subarachnoid hemorrhage (HCC)	ICD10CM	2	I60.9	G81.00,I60.9	Nontraumatic subarachnoid hemorrhage, unspecified	IMO	GHS-PACDC	IMO_2017_R	ACTIVE	N	1900-01-01	9999-12-31	2017-04-14
Diagnosis: ICD10 Code, I00-I99, Define	I00-I99	Diseases of the circulatory system	341808	Intracranial hemorrhage, spontaneous subarachnoid, presumed choroid plexus, chronic (HCC)	ICD10CM	1	I60.9		Nontraumatic subarachnoid hemorrhage, unspecified	IMO	GHS-PACDC	DX_LEGACY	ACTIVE	N	1900-01-01	9999-12-31	2015-07-10

Diagnosis: ICD10 Code, I00-I99, Define	I00-I99	Diseases of the circulatory system	426267	Hypertensive hemorrhage of putamen (HCC)	ICD10CM	1	I61.0		Nontraumatic intracerebral hemorrhage in hemisphere,	IMO	GHS-PACDC	IMO_2015_R	ACTIVE	N	1900-01-01	9999-12-31	2015-07-10
Diagnosis: ICD10 Code, I00-I99, Define	I00-I99	Diseases of the circulatory system	1285342	Nontraumatic subcortical hemorrhage of cerebral hemisphere, unspecified laterality (HCC)	ICD10CM	1	I61.0		Nontraumatic intracerebral hemorrhage in hemisphere,	IMO	GHS-PACDC	IMO_2017_R	ACTIVE	N	1900-01-01	9999-12-31	2016-09-30
Diagnosis: ICD10 Code, I00-I99, Define	I00-I99	Diseases of the circulatory system	1191886	Nontraumatic intracerebral hemorrhage in hemisphere, subcortical (HCC)	ICD10CM	1	I61.0		Nontraumatic intracerebral hemorrhage in hemisphere,	IMO	GHS-PACDC	IMO_2016_R	ACTIVE	N	1900-01-01	9999-12-31	2016-06-02
Diagnosis: ICD10 Code, I00-I99, Define	I00-I99	Diseases of the circulatory system	318429	Brain stem hemorrhage (HCC)	ICD10CM	1	I61.3		Nontraumatic intracerebral hemorrhage in brain stem	IMO	GHS-PACDC	DX_LEGACY	ACTIVE	N	1900-01-01	9999-12-31	2015-07-10
Diagnosis: ICD10 Code, I00-I99, Define	I00-I99	Diseases of the circulatory system	1132279	Right-sided nontraumatic intracerebral hemorrhage of cerebellum (HCC)	ICD10CM	1	I61.4		Nontraumatic intracerebral hemorrhage in cerebellum	IMO	GHS-PACDC	IMO_2016_R	ACTIVE	N	1900-01-01	9999-12-31	2016-06-02
Diagnosis: ICD10 Code, I00-I99, Define	I00-I99	Diseases of the circulatory system	341732	Spontaneous presumed perinatal intraventricular hemorrhage due to cerebral AVM (HCC)	ICD10CM	1	I61.5	I61.5,Q28.3	Nontraumatic intracerebral hemorrhage, intraventricula	IMO	GHS-PACDC	DX_LEGACY	ACTIVE	N	1900-01-01	2016-10-14	2015-07-10
Diagnosis: ICD10 Code, I00-I99, Define	I00-I99	Diseases of the circulatory system	342067	Intracranial hemorrhage, spontaneous intraparenchymal, presumed to involve the germinal ma	ICD10CM	2	I61.5	P96.89,I61.5	Nontraumatic intracerebral hemorrhage, intraventricula	IMO	GHS-PACDC	DX_LEGACY	ACTIVE	N	1900-01-01	9999-12-31	2015-07-10
Diagnosis: ICD10 Code, I00-I99, Define	I00-I99	Diseases of the circulatory system	135400	Intraventricular hemorrhage, unspecified grade	ICD10CM	1	I61.5		Nontraumatic intracerebral hemorrhage, intraventricula	IMO	GHS-PACDC	DX_LEGACY	ACTIVE	Y	1900-01-01	9999-12-31	2017-04-14
Diagnosis: ICD10 Code, I00-I99, Define	I00-I99	Diseases of the circulatory system	341745	Intracranial hemorrhage, spontaneous intraventricular, associated with hypertension, acute (HC	ICD10CM	1	I61.5	I61.5,I10	Nontraumatic intracerebral hemorrhage, intraventricula	IMO	GHS-PACDC	DX_LEGACY	ACTIVE	N	1900-01-01	9999-12-31	2015-07-10
Diagnosis: ICD10 Code, I00-I99, Define	I00-I99	Diseases of the circulatory system	202091	Chronic idiopathic spontaneous intraventricular hemorrhage (HCC)	ICD10CM	1	I61.5		Nontraumatic intracerebral hemorrhage, intraventricula	IMO	GHS-PACDC	DX_LEGACY	ACTIVE	N	1900-01-01	9999-12-31	2015-07-10
Diagnosis: ICD10 Code, I00-I99, Define	I00-I99	Diseases of the circulatory system	202663	Spontaneous intraventricular intracranial hemorrhage, acute (HCC)	ICD10CM	1	I61.5		Nontraumatic intracerebral hemorrhage, intraventricula	IMO	GHS-PACDC	DX_LEGACY	ACTIVE	N	1900-01-01	9999-12-31	2015-07-10
Diagnosis: ICD10 Code, I00-I99, Define	I00-I99	Diseases of the circulatory system	315012	Nontraumatic multiple localized intracerebral hemorrhages (HCC)	ICD10CM	1	I61.6		Nontraumatic intracerebral hemorrhage, multiple locali:	IMO	GHS-PACDC	DX_LEGACY	ACTIVE	N	1900-01-01	9999-12-31	2015-07-10
Diagnosis: ICD10 Code, I00-I99, Define	I00-I99	Diseases of the circulatory system	1113948	Cerebrovascular accident (CVA) with intracranial hemorrhage (HCC)	ICD10CM	1	I61.9		Nontraumatic intracerebral hemorrhage, unspecified	IMO	GHS-PACDC	IMO_2016_R	ACTIVE	N	1900-01-01	9999-12-31	2016-06-02
Diagnosis: ICD10 Code, I00-I99, Define	I00-I99	Diseases of the circulatory system	365112	Chronic intracerebral hemorrhage (HCC)	ICD10CM	1	I61.9		Nontraumatic intracerebral hemorrhage, unspecified	IMO	GHS-PACDC	DX_LEGACY	ACTIVE	N	1900-01-01	9999-12-31	2015-07-10
Diagnosis: ICD10 Code, I00-I99, Define	I00-I99	Diseases of the circulatory system	499476	Hemorrhage of right temporal lobe (HCC)	ICD10CM	1	I61.9		Nontraumatic intracerebral hemorrhage, unspecified	IMO	GHS-PACDC	IMO_2015_R	ACTIVE	N	1900-01-01	9999-12-31	2015-07-10
Diagnosis: ICD10 Code, I00-I99, Define	I00-I99	Diseases of the circulatory system	496680	Left-sided intracerebral hemorrhage (HCC)	ICD10CM	1	I61.9		Nontraumatic intracerebral hemorrhage, unspecified	IMO	GHS-PACDC	IMO_2015_R	ACTIVE	N	1900-01-01	2016-05-07	2015-07-10
Diagnosis: ICD10 Code, I00-I99, Define	I00-I99	Diseases of the circulatory system	1321259	Spastic hemiparesis of right nondominant side due to nontraumatic intracerebral hemorrhage (	ICD10CM	1	I61.9	I61.9,G81.13	Nontraumatic intracerebral hemorrhage, unspecified	IMO	GHS-PACDC	IMO_2017_R	ACTIVE	N	2017-09-17	9999-12-31	2017-10-30
Diagnosis: ICD10 Code, I00-I99, Define	I00-I99	Diseases of the circulatory system	109836	ICH (intracerebral hemorrhage) (HCC)	ICD10CM	1	I61.9		Nontraumatic intracerebral hemorrhage, unspecified	IMO	GHS-PACDC	DX_LEGACY	ACTIVE	N	1900-01-01	9999-12-31	2015-07-10
Diagnosis: ICD10 Code, I00-I99, Define	I00-I99	Diseases of the circulatory system	426264	Hypertensive intracerebral hemorrhage (HCC)	ICD10CM	1	I61.9		Nontraumatic intracerebral hemorrhage, unspecified	IMO	GHS-PACDC	IMO_2015_R	ACTIVE	N	1900-01-01	9999-12-31	2015-07-10
Diagnosis: ICD10 Code, I00-I99, Define	I00-I99	Diseases of the circulatory system	330955	Choroid plexus hemorrhage (HCC)	ICD10CM	1	I61.9		Nontraumatic intracerebral hemorrhage, unspecified	IMO	GHS-PACDC	DX_LEGACY	ACTIVE	N	1900-01-01	9999-12-31	2015-07-10
Diagnosis: ICD10 Code, I00-I99, Define	I00-I99	Diseases of the circulatory system	1114581	Compression of brain due to spontaneous cerebral hemorrhage (HCC)	ICD10CM	2	I61.9	G93.5,I61.9	Nontraumatic intracerebral hemorrhage, unspecified	IMO	GHS-PACDC	IMO_2016_R	ACTIVE	N	1900-01-01	9999-12-31	2016-06-02
Diagnosis: ICD10 Code, I00-I99, Define	I00-I99	Diseases of the circulatory system	597697	Nontraumatic subdural hemorrhage, unspecified	ICD10CM	1	I62.00		Nontraumatic subdural hemorrhage, unspecified	IMO	GHS-PACDC	IMO_2015_R	ACTIVE	Y	1900-01-01	9999-12-31	2015-07-10
Diagnosis: ICD10 Code, I00-I99, Define	I00-I99	Diseases of the circulatory system	22251	Nontraumatic subdural hemorrhage (HCC)	ICD10CM	1	I62.00		Nontraumatic subdural hemorrhage, unspecified	IMO	GHS-PACDC	DX_LEGACY	ACTIVE	N	1900-01-01	9999-12-31	2015-07-10
Diagnosis: ICD10 Code, I00-I99, Define	I00-I99	Diseases of the circulatory system	608050	Nontraumatic acute subdural hemorrhage	ICD10CM	1	I62.01		Nontraumatic acute subdural hemorrhage	IMO	GHS-PACDC	IMO_2015_R	ACTIVE	Y	1900-01-01	9999-12-31	2015-07-10
Diagnosis: ICD10 Code, I00-I99, Define	I00-I99	Diseases of the circulatory system	22246	Extradural hemorrhage (HCC)	ICD10CM	1	I62.1		Nontraumatic extradural hemorrhage	IMO	GHS-PACDC	DX_LEGACY	ACTIVE	N	1900-01-01	2016-05-07	2015-07-10
Diagnosis: ICD10 Code, I00-I99, Define	I00-I99	Diseases of the circulatory system	200386	Non-traumatic intracranial hemorrhage (HCC)	ICD10CM	1	I62.9		Nontraumatic intracranial hemorrhage, unspecified	IMO	GHS-PACDC	DX_LEGACY	ACTIVE	N	1900-01-01	9999-12-31	2015-07-10
Diagnosis: ICD10 Code, I00-I99, Define	I00-I99	Diseases of the circulatory system	252506	Cerebral hemorrhage with hemiparesis (HCC)	ICD10CM	1	I62.9	I62.9,G81.90	Nontraumatic intracranial hemorrhage, unspecified	IMO	GHS-PACDC	DX_LEGACY	ACTIVE	N	2016-04-10	9999-12-31	2015-07-10
Diagnosis: ICD10 Code, I00-I99, Define	I00-I99	Diseases of the circulatory system	202680	Chronic spontaneous intraparenchymal intracranial hemorrhage (HCC)	ICD10CM	1	I62.9		Nontraumatic intracranial hemorrhage, unspecified	IMO	GHS-PACDC	DX_LEGACY	ACTIVE	N	1900-01-01	9999-12-31	2015-07-10
Diagnosis: ICD10 Code, I00-I99, Define	I00-I99	Diseases of the circulatory system	205685	Bleeding, intracranial (HCC)	ICD10CM	1	I62.9		Nontraumatic intracranial hemorrhage, unspecified	IMO	GHS-PACDC	DX_LEGACY	ACTIVE	N	1900-01-01	9999-12-31	2015-07-10
Diagnosis: ICD10 Code, I00-I99, Define	I00-I99	Diseases of the circulatory system	342218	Spontaneous perinatal intraparenchymal intracranial hemorrhage due to cerebral cavernoma (H	ICD10CM	2	I62.9	P54.8,I62.9,Q28.3	Nontraumatic intracranial hemorrhage, unspecified	IMO	GHS-PACDC	DX_LEGACY	ACTIVE	N	1900-01-01	9999-12-31	2015-07-10
Diagnosis: ICD10 Code, I00-I99, Define	I00-I99	Diseases of the circulatory system	341691	Chronic idiopathic spontaneous intraparenchymal intracranial hemorrhage (HCC)	ICD10CM	1	I62.9		Nontraumatic intracranial hemorrhage, unspecified	IMO	GHS-PACDC	DX_LEGACY	ACTIVE	N	1900-01-01	9999-12-31	2015-07-10
Diagnosis: ICD10 Code, I00-I99, Define	I00-I99	Diseases of the circulatory system	1321210	Hemiparesis of left nondominant side due to other nontraumatic intracranial hemorrhage (HCC	ICD10CM	1	I62.9	I62.9,G81.94	Nontraumatic intracranial hemorrhage, unspecified	IMO	GHS-PACDC	IMO_2017_R	ACTIVE	N	2017-09-17	9999-12-31	2017-10-30
Diagnosis: ICD10 Code, I00-I99, Define	I00-I99	Diseases of the circulatory system	202936	Spont perinatal intraparenchymal hemorrhage d/t cerebral aneurysm (HCC)	ICD10CM	2	I62.9	P54.8,I62.9,I67.1	Nontraumatic intracranial hemorrhage, unspecified	IMO	GHS-PACDC	DX_LEGACY	ACTIVE	N	1900-01-01	9999-12-31	2015-07-10
Diagnosis: ICD10 Code, I00-I99, Define	I00-I99	Diseases of the circulatory system	342253	Spont in utero intraparenchymal hemorrhage assoc w/ arterial infarction (HCC)	ICD10CM	2	I62.9	P54.8,I62.9,I63.9	Nontraumatic intracranial hemorrhage, unspecified	IMO	GHS-PACDC	DX_LEGACY	ACTIVE	N	1900-01-01	2016-05-07	2015-07-10
Diagnosis: ICD10 Code, I00-I99, Define	I00-I99	Diseases of the circulatory system	1113721	Stroke due to thrombosis of precerebral artery (HCC)	ICD10CM	1	I63.00		Cerebral infarction due to thrombosis of unspecified pre	IMO	GHS-PACDC	IMO_2016_R	ACTIVE	N	1900-01-01	9999-12-31	2016-06-02
Diagnosis: ICD10 Code, I00-I99, Define	I00-I99	Diseases of the circulatory system	1113566	Cerebrovascular accident (CVA) due to thrombosis of right vertebral artery (HCC)	ICD10CM	1	I63.011		Cerebral infarction due to thrombosis of right vertebral	IMO	GHS-PACDC	IMO_2016_R	ACTIVE	N	1900-01-01	9999-12-31	2016-06-02
Diagnosis: ICD10 Code, I00-I99, Define	I00-I99	Diseases of the circulatory system	599980	Cerebral infarction due to thrombosis of unspecified vertebral artery	ICD10CM	1	I63.019		Cerebral infarction due to thrombosis of unspecified ver	IMO	GHS-PACDC	IMO_2015_R	ACTIVE	Y	1900-01-01	9999-12-31	2015-07-10
Diagnosis: ICD10 Code, I00-I99, Define	I00-I99	Diseases of the circulatory system	270403	Cerebral infarction due to thrombosis of carotid artery (HCC)	ICD10CM	1	I63.039		Cerebral infarction due to thrombosis of unspecified car	IMO	GHS-PACDC	DX_LEGACY	ACTIVE	N	1900-01-01	9999-12-31	2015-07-10
Diagnosis: ICD10 Code, I00-I99, Define	I00-I99	Diseases of the circulatory system	1113642	Stroke due to thrombosis of carotid artery (HCC)	ICD10CM	1	I63.039		Cerebral infarction due to thrombosis of unspecified car	IMO	GHS-PACDC	IMO_2016_R	ACTIVE	N	1900-01-01	9999-12-31	2016-06-02
Diagnosis: ICD10 Code, I00-I99, Define	I00-I99	Diseases of the circulatory system	304965	Thrombotic stroke involving carotid artery (HCC)	ICD10CM	1	I63.039		Cerebral infarction due to thrombosis of unspecified car	IMO	GHS-PACDC	DX_LEGACY	ACTIVE	N	1900-01-01	9999-12-31	2015-07-10
Diagnosis: ICD10 Code, I00-I99, Define	I00-I99	Diseases of the circulatory system	1113591	Cerebrovascular accident (CVA) due to embolism of vertebral artery (HCC)	ICD10CM	1	I63.119		Cerebral infarction due to embolism of unspecified verte	IMO	GHS-PACDC	IMO_2016_R	ACTIVE	N	1900-01-01	9999-12-31	2016-06-02
Diagnosis: ICD10 Code, I00-I99, Define	I00-I99	Diseases of the circulatory system	268083	Cerebral infarction due to embolism of right carotid artery (HCC)	ICD10CM	1	I63.131		Cerebral infarction due to embolism of right carotid arte	IMO	GHS-PACDC	DX_LEGACY	ACTIVE	N	1900-01-01	9999-12-31	2015-07-10
Diagnosis: ICD10 Code, I00-I99, Define	I00-I99	Diseases of the circulatory system	1272882	Cerebral infarction due to bilateral embolism of carotid arteries (HCC)	ICD10CM	1	I63.133		Cerebral infarction due to embolism of bilateral carotid	IMO	GHS-PACDC	IMO_2017_R	ACTIVE	N	1900-01-01	9999-12-31	2016-09-30
Diagnosis: ICD10 Code, I00-I99, Define	I00-I99	Diseases of the circulatory system	1274838	Cerebral infarction due to embolism of bilateral carotid arteries	ICD10CM	1	I63.133		Cerebral infarction due to embolism of bilateral carotid	IMO	GHS-PACDC	IMO_2017_R	ACTIVE	Y	1900-01-01	9999-12-31	2017-04-14
Diagnosis: ICD10 Code, I00-I99, Define	I00-I99	Diseases of the circulatory system	203008	Perinatal ischemic vertebrobasilar artery cerebellar stroke (HCC)	ICD10CM	2	I63.20	P91.0,I63.20	Cerebral infarction due to unspecified occlusion or stenc	IMO	GHS-PACDC	DX_LEGACY	ACTIVE	N	2016-09-17	9999-12-31	2016-09-30
Diagnosis: ICD10 Code, I00-I99, Define	I00-I99	Diseases of the circulatory system	342071	Arterial ischemic stroke, vertebrobasilar, cerebellar, presumed perinatal (HCC)	ICD10CM	2	I63.20	P91.0,I63.20	Cerebral infarction due to unspecified occlusion or stenc	IMO	GHS-PACDC	DX_LEGACY	ACTIVE	N	2016-09-17	9999-12-31	2016-09-30
Diagnosis: ICD10 Code, I00-I99, Define	I00-I99	Diseases of the circulatory system	598417	Cerebral infarction due to unspecified occlusion or stenosis of unspecified precerebral arteries	ICD10CM	1	I63.20		Cerebral infarction due to unspecified occlusion or stenc	IMO	GHS-PACDC	IMO_2015_R	ACTIVE	Y	1900-01-01	9999-12-31	2015-07-10
Diagnosis: ICD10 Code, I00-I99, Define	I00-I99	Diseases of the circulatory system	1098640	Precerebral occl w/ infarct	ICD10CM	1	I63.20		Cerebral infarction due to unspecified occlusion or stenc	IMO	GHS-PACDC	IMO_2016_R	DELETED	Y	1900-01-01	9999-12-31	2016-06-02
Diagnosis: ICD10 Code, I00-I99, Define	I00-I99	Diseases of the circulatory system	957452	Acute ischemic VBA thalamic stroke, right (HCC)	ICD10CM	1	I63.211	I63.211,I63.22	Cerebral infarction due to unspecified occlusion or stenc	IMO	GHS-PACDC	IMO_2015_R	ACTIVE	N	2016-04-10	9999-12-31	2015-07-10
Diagnosis: ICD10 Code, I00-I99, Define	I00-I99	Diseases of the circulatory system	962376	Acute arterial ischemic stroke, vertebrobasilar, brainstem, left (HCC)	ICD10CM	1	I63.212		Cerebral infarction due to unspecified occlusion or stenc	IMO	GHS-PACDC	IMO_2015_R	ACTIVE	N	1900-01-01	2016-05-07	2015-07-10
Diagnosis: ICD10 Code, I00-I99, Define	I00-I99	Diseases of the circulatory system	602980	Cerebral infarction due to unspecified occlusion or stenosis of unspecified vertebral arteries	ICD10CM	1	I63.219		Cerebral infarction due to unspecified occlusion or stenc	IMO	GHS-PACDC	IMO_2015_R	ACTIVE	Y	1900-01-01	9999-12-31	2015-07-10
Diagnosis: ICD10 Code, I00-I99, Define	I00-I99	Diseases of the circulatory system	957452	Acute ischemic VBA thalamic stroke, right (HCC)	ICD10CM	2	I63.22	I63.211,I63.22	Cerebral infarction due to unspecified occlusion or stenc	IMO	GHS-PACDC	IMO_2015_R	ACTIVE	N	2016-04-10	9999-12-31	2015-07-10
Diagnosis: ICD10 Code, I00-I99, Define	I00-I99	Diseases of the circulatory system	202251	Acute ischemic vertebrobasilar artery thalamic stroke (HCC)	ICD10CM	2	I63.22	I63.219,I63.22	Cerebral infarction due to unspecified occlusion or stenc	IMO	GHS-PACDC	DX_LEGACY	ACTIVE	N	1900-01-01	9999-12-31	2015-07-10
Diagnosis: ICD10 Code, I00-I99, Define	I00-I99	Diseases of the circulatory system	971826	Cerebral infarction due to occlusion or stenosis of basilar artery	ICD10CM	1	I63.22		Cerebral infarction due to unspecified occlusion or stenc	IMO	GHS-PACDC	IMO_2015_R	DELETED	Y	1900-01-01	9999-12-31	2015-07-10
Diagnosis: ICD10 Code, I00-I99, Define	I00-I99	Diseases of the circulatory system	958206	Acute ischemic vertebrobasilar artery thalamic stroke, right (HCC)	ICD10CM	2	I63.22	I63.211,I63.22	Cerebral infarction due to unspecified occlusion or stenc	IMO	GHS-PACDC	IMO_2015_R	ACTIVE	N	2016-04-10	9999-12-31	2015-07-10
Diagnosis: ICD10 Code, I00-I99, Define	I00-I99	Diseases of the circulatory system	1427289	Cerebral infarction due to unspecified occlusion or stenosis of basilar artery (CODE) (HCC)	ICD10CM	1	I63.22		Cerebral infarction due to unspecified occlusion or stenc	IMO	GHS-PACDC	IMO_2018_R	ACTIVE	N	1900-01-01	9999-12-31	2017-10-30
Diagnosis: ICD10 Code, I00-I99, Define	I00-I99	Diseases of the circulatory system	612257	Cerebral infarction due to unspecified occlusion or stenosis of carotid arteries	ICD10CM	1	I63.23		Cerebral infarction due to unspecified occlusion or stenc	IMO	GHS-PACDC	IMO_2015_R	ACTIVE	Y	1900-01-01	9999-12-31	2015-07-10
Diagnosis: ICD10 Code, I00-I99, Define	I00-I99	Diseases of the circulatory system	202990	Perinatal ischemic right ICA stroke (HCC)	ICD10CM	2	I63.231	P91.0,I63.231	Cerebral infarction due to unspecified occlusion or stenc	IMO	GHS-PACDC	DX_LEGACY	ACTIVE	N	2016-09-17	9999-12-31	2016-09-30
Diagnosis: ICD10 Code, I00-I99, Define	I00-I99	Diseases of the circulatory system	202990	Perinatal ischemic right ICA stroke (HCC)	ICD10CM	2	I63.231	P96.89,I63.231	Cerebral infarction due to unspecified occlusion or stenc	IMO	GHS-PACDC	DX_LEGACY	ACTIVE	N	1900-01-01	2016-10-14	2015-07-10
Diagnosis: ICD10 Code, I00-I99, Define	I00-I99	Diseases of the circulatory system	221143	Perinatal right ICA stroke (HCC)	ICD10CM	2	I63.231	P91.0,I63.231	Cerebral infarction due to unspecified occlusion or stenc	IMO	GHS-PACDC	DX_LEGACY	ACTIVE	N	2016-09-17	9999-12-31	2016-09-30
Diagnosis: ICD10 Code, I00-I99, Define	I00-I99	Diseases of the circulatory system	496643	Cerebral infarction involving right carotid artery (HCC)	ICD10CM	1	I63.231		Cerebral infarction due to unspecified occlusion or stenc	IMO	GHS-PACDC	IMO_2015_R	ACTIVE	N	1900-01-01	9999-12-31	2015-07-10
Diagnosis: ICD10 Code, I00-I99, Define	I00-I99	Diseases of the circulatory system	202987	Perinatal ischemic left ICA stroke (HCC)	ICD10CM	2	I63.232	P91.0,I63.232	Cerebral infarction due to unspecified occlusion or stenc	IMO	GHS-PACDC	DX_LEGACY	ACTIVE	N	2016-09-17	9999-12-31	2016-09-30
Diagnosis: ICD10 Code, I00-I99, Define	I00-I99	Diseases of the circulatory system	221138	Arterial ischemic stroke, ICA (internal carotid artery), left, perintl (HCC)	ICD10CM	2	I63.232	P96.89,I63.232	Cerebral infarction due to unspecified occlusion or stenc	IMO	GHS-PACDC	DX_LEGACY	ACTIVE	N	1900-01-01	2016-10-14	2015-07-10
Diagnosis: ICD10 Code, I00-I99, Define	I00-I99	Diseases of the circulatory system	385875	Perinatal ischemic left internal carotid artery (ICA) stroke (HCC)	ICD10CM	2	I63.232	P91.0,I63.232	Cerebral infarction due to unspecified occlusion or stenc	IMO	GHS-PACDC	DX_LEGACY	ACTIVE	N	2016-09-17	9999-12-31	2016-09-30
Diagnosis: ICD10 Code, I00-I99, Define	I00-I99	Diseases of the circulatory system	524243	Cerebral infarction due to stenosis of left carotid artery (HCC)	ICD10CM	1	I63.232		Cerebral infarction due to unspecified occlusion or stenc	IMO	GHS-PACDC	IMO_2015_R	ACTIVE	N	1900-01-01	9999-12-31	2015-07-10
Diagnosis: ICD10 Code, I00-I99, Define	I00-I99	Diseases of the circulatory system	1096527	Cerebral infarction due to vascular occlusion, left	ICD10CM	1	I63.232		Cerebral infarction due to unspecified occlusion or stenc	IMO	GHS-PACDC	IMO_2015_R	DELETED	Y	1900-01-01	9999-12-31	2015-07-10
Diagnosis: ICD10 Code, I00-I99, Define	I00-I99	Diseases of the circulatory system	950874	Cerebral infarction due to internal carotid artery occlusion, left (HCC)	ICD10CM	1	I63.232		Cerebral infarction due to unspecified occlusion or stenc	IMO	GHS-PACDC	IMO_2015_R	ACTIVE	N	1900-01-01	9999-12-31	2015-07-10
Diagnosis: ICD10 Code, I00-I99, Define	I00-I99	Diseases of the circulatory system	1093340	Cerebral infarction due to vascular occlusion, unspecified laterality	ICD10CM	1	I63.239		Cerebral infarction due to unspecified occlusion or stenc	IMO	GHS-PACDC	IMO_2015_R	DELETED	Y	1900-01-01	9999-12-31	2015-07-10
Diagnosis: ICD10 Code, I00-I99, Define	I00-I99	Diseases of the circulatory system	1295991	Cerebrovascular accident (CVA) due to stenosis of carotid artery, unspecified blood vessel latera	ICD10CM	1	I63.239		Cerebral infarction due to unspecified occlusion or stenc	IMO	GHS-PACDC	IMO_2017_R	ACTIVE	N	1900-01-01	9999-12-31	2016-09-30
Diagnosis: ICD10 Code, I00-I99, Define	I00-I99	Diseases of the circulatory system	606379	Cerebral infarction due to thrombosis of cerebral arteries	ICD10CM	1	I63.3		Cerebral infarction due to thrombosis of cerebral arteri	IMO	GHS-PACDC	IMO_2015_R	ACTIVE	Y	1900-01-01	9999-12-31	2015-07-10
Diagnosis: ICD10 Code, I00-I99, Define	I00-I99	Diseases of the circulatory system	343195	Cerebral venous infarction, associated with cerebral sinovenous thrombosis, acute (HCC)	ICD10CM	1	I63.30		Cerebral infarction due to thrombosis of unspecified cer	IMO	GHS-PACDC	DX_LEGACY	ACTIVE	N	1900-01-01	2016-05-07	2015-07-10
Diagnosis: ICD10 Code, I00-I99, Define	I00-I99	Diseases of the circulatory system	268098	Cerebral infarction due to thrombosis of right middle cerebral artery (HCC)	ICD10CM	1	I63.311		Cerebral infarction due to thrombosis of right middle ce	IMO	GHS-PACDC	DX_LEGACY	ACTIVE	N	1900-01-01	9999-12-31	2015-07-10
Diagnosis: ICD10 Code, I00-I99, Define	I00-I99	Diseases of the circulatory system	304973	Thrombotic stroke involving left middle cerebral artery (HCC)	ICD10CM	1	I63.312		Cerebral infarction due to thrombosis of left middle cere	IMO	GHS-PACDC	DX_LEGACY	ACTIVE	N	1900-01-01	9999-12-31	2015-07-10
Diagnosis: ICD10 Code, I00-I99, Define	I00-I99	Diseases of the circulatory system	344481	Cerebral infarction due to thrombosis of right anterior cerebral artery (HCC)	ICD10CM	1	I63.321		Cerebral infarction due to thrombosis of right anterior c	IMO	GHS-PACDC	DX_LEGACY	ACTIVE	N	1900-01-01	9999-12-31	2015-07-10
Diagnosis: ICD10 Code, I00-I99, Define	I00-I99	Diseases of the circulatory system	1275035	Cerebral infarction due to thrombosis of bilateral anterior arteries	ICD10CM	1	I63.323		Cerebral infarction due to thrombosis of bilateral anteri	IMO	GHS-PACDC	IMO_2017_R	ACTIVE	Y	2016-09-17	9999-12-31	2016-09-30
Diagnosis: ICD10 Code, I00-I99, Define	I00-I99	Diseases of the circulatory system	1113609	Stroke due to thrombosis of right posterior cerebral artery (HCC)	ICD10CM	1	I63.331		Cerebral infarction due to thrombosis of right posterior	IMO	GHS-PACDC	IMO_2016_R	ACTIVE	N	1900-01-01	9999-12-31	2016-06-02
Diagnosis: ICD10 Code, I00-I99, Define	I00-I99	Diseases of the circulatory system	1272912	Cerebrovascular accident (CVA) due to bilateral thrombosis of posterior cerebral arteries (HCC)	ICD10CM	1	I63.333		Cerebral infarction to thrombosis of bilateral posterior c	IMO	GHS-PACDC	IMO_2017_R	ACTIVE	N	1900-01-01	9999-12-31	2016-09-30
Diagnosis: ICD10 Code, I00-I99, Define	I00-I99	Diseases of the circulatory system	588308	Cerebral infarction due to thrombosis of cerebellar artery	ICD10CM	1	I63.34		Cerebral infarction due to thrombosis of cerebellar arter	IMO	GHS-PACDC	IMO_2015_R	ACTIVE	Y	1900-01-01	9999-12-31	2015-07-10
Diagnosis: ICD10 Code, I00-I99, Define	I00-I99	Diseases of the circulatory system	1291688	Cerebral infarction to thrombosis of bilateral cerebellar arteries (HCC)	ICD10CM	1	I63.343		Cerebral infarction to thrombosis of bilateral cerebellar	IMO	GHS-PACDC	IMO_2017_R	ACTIVE	N	1900-01-01	9999-12-31	2016-09-30
Diagnosis: ICD10 Code, I00-I99, Define	I00-I99	Diseases of the circulatory system	265097	Acute embolic stroke within last 8 weeks	ICD10CM	1	I63.40		Cerebral infarction due to embolism of unspecified cere	IMO	GHS-PACDC	DX_LEGACY	ACTIVE	N	1900-01-01	2016-05-07	2015-07-10
Diagnosis: ICD10 Code, I00-I99, Define	I00-I99	Diseases of the circulatory system	262597	Stroke, acute, embolic (HCC)	ICD10CM	1	I63.40		Cerebral infarction due to embolism of unspecified cere	IMO	GHS-PACDC	DX_LEGACY	ACTIVE	N	1900-01-01	2016-05-07	2015-07-10
Diagnosis: ICD10 Code, I00-I99, Define	I00-I99	Diseases of the circulatory system	1272906	Cerebrovascular accident (CVA) due to bilateral embolism of middle cerebral arteries (HCC)	ICD10CM	1	I63.413		Cerebral infarction due to embolism of bilateral middle (	IMO	GHS-PACDC	IMO_2017_R	ACTIVE	N	1900-01-01	9999-12-31	2016-09-30
Diagnosis: ICD10 Code, I00-I99, Define	I00-I99	Diseases of the circulatory system	304952	Embolic stroke involving middle cerebral artery (HCC)	ICD10CM	1	I63.419		Cerebral infarction due to embolism of unspecified midc	IMO	GHS-PACDC	DX_LEGACY	ACTIVE	N	1900-01-01	9999-12-31	2015-07-10
Diagnosis: ICD10 Code, I00-I99, Define	I00-I99	Diseases of the circulatory system	1295570	Cerebral infarction due to embolism of middle cerebral artery, unspecified blood vessel lateralit	ICD10CM	1	I63.419		Cerebral infarction due to embolism of unspecified midc	IMO	GHS-PACDC	IMO_2017_R	ACTIVE	N	1900-01-01	9999-12-31	2016-09-30
Diagnosis: ICD10 Code, I00-I99, Define	I00-I99	Diseases of the circulatory system	270399	Cerebral infarction due to embolism of middle cerebral artery (HCC)	ICD10CM	1	I63.419		Cerebral infarction due to embolism of unspecified midc	IMO	GHS-PACDC	DX_LEGACY	ACTIVE	N	1900-01-01	9999-12-31	2015-07-10
Diagnosis: ICD10 Code, I00-I99, Define	I00-I99	Diseases of the circulatory system	1113705	Stroke due to embolism of right anterior cerebral artery (HCC)	ICD10CM	1	I63.421		Cerebral infarction due to embolism of right anterior ce	IMO	GHS-PACDC	IMO_2016_R	ACTIVE	N	1900-01-01	9999-12-31	2016-06-02
Diagnosis: ICD10 Code, I00-I99, Define	I00-I99	Diseases of the circulatory system	588292	Cerebral infarction due to embolism of right posterior cerebral artery	ICD10CM	1	I63.431		Cerebral infarction due to embolism of right posterior c	IMO	GHS-PACDC	IMO_2015_R	ACTIVE	Y	1900-01-01	9999-12-31	2015-07-10
Diagnosis: ICD10 Code, I00-I99, Define	I00-I99	Diseases of the circulatory system	304948	Embolic stroke involving left cerebellar artery (HCC)	ICD10CM	1	I63.442		Cerebral infarction due to embolism of left cerebellar ar	IMO	GHS-PACDC	DX_LEGACY	ACTIVE	N	1900-01-01	9999-12-31	2015-07-10
Diagnosis: ICD10 Code, I00-I99, Define	I00-I99	Diseases of the circulatory system	342263	Intracranial hemorrhage, spontaneous intraparenchymal, associated with arterial infarction, pre	ICD10CM	2	I63.50	P52.4,I63.50,P91.0	Cerebral infarction due to unspecified occlusion or stenc	IMO	GHS-PACDC	DX_LEGACY	ACTIVE	N	2016-09-17	9999-12-31	2016-09-30
Diagnosis: ICD10 Code, I00-I99, Define	I00-I99	Diseases of the circulatory system	203034	Arterial ischemic stroke, vertebrobasilar, cerebellar, in utero (HCC)	ICD10CM	2	I63.50	P96.89,I63.50	Cerebral infarction due to unspecified occlusion or stenc	IMO	GHS-PACDC	DX_LEGACY	ACTIVE	N	1900-01-01	2016-10-14	2015-07-10
Diagnosis: ICD10 Code, I00-I99, Define	I00-I99	Diseases of the circulatory system	329979	Left temporal lobe infarction (HCC)	ICD10CM	1	I63.50		Cerebral infarction due to unspecified occlusion or stenc	IMO	GHS-PACDC	DX_LEGACY	ACTIVE	N	1900-01-01	9999-12-31	2015-07-10
Diagnosis: ICD10 Code, I00-I99, Define	I00-I99	Diseases of the circulatory system	203013	Perinatal ischemic multifocal anterior circulation stroke (HCC)	ICD10CM	2	I63.50	P96.89,I63.50	Cerebral infarction due to unspecified occlusion or stenc	IMO	GHS-PACDC	DX_LEGACY	ACTIVE	N	1900-01-01	2016-10-14	2015-07-10
Diagnosis: ICD10 Code, I00-I99, Define	I00-I99	Diseases of the circulatory system	202915	Ischemic multifocal multiple vascular territories stroke, in utero (HCC)	ICD10CM	2	I63.50	P96.89,I63.50	Cerebral infarction due to unspecified occlusion or stenc	IMO	GHS-PACDC	DX_LEGACY	ACTIVE	N	1900-01-01	2017-10-14	2015-07-10
Diagnosis: ICD10 Code, I00-I99, Define	I00-I99	Diseases of the circulatory system	1106480	Cerebral infarction due to stenosis of cerebral artery (HCC)	ICD10CM	1	I63.50		Cerebral infarction due to unspecified occlusion or stenc	IMO	GHS-PACDC	IMO_2016_R	ACTIVE	N	1900-01-01	9999-12-31	2016-06-02
Diagnosis: ICD10 Code, I00-I99, Define	I00-I99	Diseases of the circulatory system	202146	Presumed perinatal ischemic right MCA stroke (HCC)	ICD10CM	1	I63.50		Cerebral infarction due to unspecified occlusion or stenc	IMO	GHS-PACDC	DX_LEGACY	ACTIVE	N	1900-01-01	2016-10-14	2015-07-10

Diagnosis: ICD10 Code, I00-I99, Define	I00-I99	Diseases of the circulatory system	201723	Encephalomalacia with cerebral infarction (HCC)	ICD10CM	1	I63.50		Cerebral infarction due to unspecified occlusion or stenc	IMO	GHS-PACDC	DX_LEGACY	ACTIVE	N	1900-01-01	9999-12-31	2015-07-10
Diagnosis: ICD10 Code, I00-I99, Define	I00-I99	Diseases of the circulatory system	385859	Ischemic left internal carotid artery (ICA) stroke, in utero (HCC)	ICD10CM	2	I63.50	P96.89,I63.50	Cerebral infarction due to unspecified occlusion or stenc	IMO	GHS-PACDC	DX_LEGACY	ACTIVE	N	1900-01-01	2016-05-07	2015-07-10
Diagnosis: ICD10 Code, I00-I99, Define	I00-I99	Diseases of the circulatory system	1114464	Cerebrovascular accident (CVA) involving right middle cerebral artery territory (HCC)	ICD10CM	1	I63.511		Cerebral infarction due to unspecified occlusion or stenc	IMO	GHS-PACDC	IMO_2016_R	ACTIVE	N	2016-09-17	9999-12-31	2016-09-30
Diagnosis: ICD10 Code, I00-I99, Define	I00-I99	Diseases of the circulatory system	385869	Ischemic right middle cerebral artery (MCA) stroke, in utero (HCC)	ICD10CM	2	I63.511	P96.89,I63.511	Cerebral infarction due to unspecified occlusion or stenc	IMO	GHS-PACDC	DX_LEGACY	ACTIVE	N	1900-01-01	2016-10-14	2015-07-10
Diagnosis: ICD10 Code, I00-I99, Define	I00-I99	Diseases of the circulatory system	385885	Perinatal ischemic right middle cerebral artery (MCA) stroke (HCC)	ICD10CM	2	I63.511	P96.89,I63.511	Cerebral infarction due to unspecified occlusion or stenc	IMO	GHS-PACDC	DX_LEGACY	ACTIVE	N	1900-01-01	2016-10-14	2015-07-10
Diagnosis: ICD10 Code, I00-I99, Define	I00-I99	Diseases of the circulatory system	385885	Perinatal ischemic right middle cerebral artery (MCA) stroke (HCC)	ICD10CM	2	I63.511	P91.0,I63.511	Cerebral infarction due to unspecified occlusion or stenc	IMO	GHS-PACDC	DX_LEGACY	ACTIVE	N	2016-09-17	9999-12-31	2016-09-30
Diagnosis: ICD10 Code, I00-I99, Define	I00-I99	Diseases of the circulatory system	1113615	Cerebrovascular accident (CVA) due to occlusion of left middle cerebral artery (HCC)	ICD10CM	1	I63.512		Cerebral infarction due to unspecified occlusion or stenc	IMO	GHS-PACDC	IMO_2016_R	ACTIVE	N	1900-01-01	9999-12-31	2016-06-02
Diagnosis: ICD10 Code, I00-I99, Define	I00-I99	Diseases of the circulatory system	385877	Perinatal ischemic left middle cerebral artery (MCA) stroke (HCC)	ICD10CM	2	I63.512	P91.0,I63.512	Cerebral infarction due to unspecified occlusion or stenc	IMO	GHS-PACDC	DX_LEGACY	ACTIVE	N	2016-09-17	9999-12-31	2016-09-30
Diagnosis: ICD10 Code, I00-I99, Define	I00-I99	Diseases of the circulatory system	202995	Arterial ischemic stroke, MCA, left, perinatal (HCC)	ICD10CM	2	I63.512	P96.89,I63.512	Cerebral infarction due to unspecified occlusion or stenc	IMO	GHS-PACDC	DX_LEGACY	ACTIVE	N	1900-01-01	2016-10-14	2015-07-10
Diagnosis: ICD10 Code, I00-I99, Define	I00-I99	Diseases of the circulatory system	1365950	Cereb infrc d/t unsp occls or stenos of right ant cereb art (HCC)	ICD10CM	1	I63.521		Cerebral infarction due to unspecified occlusion or stenc	IMO	GHS-PACDC	IMO_2018_R	ACTIVE	N	1900-01-01	9999-12-31	2017-10-30
Diagnosis: ICD10 Code, I00-I99, Define	I00-I99	Diseases of the circulatory system	479801	Acute ischemic multifocal anterior circulation stroke involving right-sided vessel (HCC)	ICD10CM	1	I63.521		Cerebral infarction due to unspecified occlusion or stenc	IMO	GHS-PACDC	IMO_2015_R	ACTIVE	N	1900-01-01	9999-12-31	2015-07-10
Diagnosis: ICD10 Code, I00-I99, Define	I00-I99	Diseases of the circulatory system	209194	Perinatal left ACA stroke (HCC)	ICD10CM	2	I63.522	P96.89,I63.522	Cerebral infarction due to unspecified occlusion or stenc	IMO	GHS-PACDC	DX_LEGACY	ACTIVE	N	1900-01-01	2016-10-14	2015-07-10
Diagnosis: ICD10 Code, I00-I99, Define	I00-I99	Diseases of the circulatory system	202473	Arterial ischemic stroke, ACA, left, presumed perinatal (HCC)	ICD10CM	2	I63.522	P91.0,I63.522	Cerebral infarction due to unspecified occlusion or stenc	IMO	GHS-PACDC	DX_LEGACY	ACTIVE	N	2016-09-17	9999-12-31	2016-09-30
Diagnosis: ICD10 Code, I00-I99, Define	I00-I99	Diseases of the circulatory system	203030	Arterial ischemic stroke, ACA, left, in utero (HCC)	ICD10CM	2	I63.522	P91.88,I63.522	Cerebral infarction due to unspecified occlusion or stenc	IMO	GHS-PACDC	DX_LEGACY	ACTIVE	N	2017-09-17	9999-12-31	2017-10-30
Diagnosis: ICD10 Code, I00-I99, Define	I00-I99	Diseases of the circulatory system	209194	Perinatal left ACA stroke (HCC)	ICD10CM	2	I63.522	P91.0,I63.522	Cerebral infarction due to unspecified occlusion or stenc	IMO	GHS-PACDC	DX_LEGACY	ACTIVE	N	2016-09-17	9999-12-31	2016-09-30
Diagnosis: ICD10 Code, I00-I99, Define	I00-I99	Diseases of the circulatory system	948851	Anterior cerebral circulation infarction, unspecified laterality (HCC)	ICD10CM	1	I63.529		Cerebral infarction due to unspecified occlusion or stenc	IMO	GHS-PACDC	IMO_2015_R	ACTIVE	N	1900-01-01	9999-12-31	2015-07-10
Diagnosis: ICD10 Code, I00-I99, Define	I00-I99	Diseases of the circulatory system	1105807	Cerebrovascular accident of uncertain pathology involving anterior circulation (HCC)	ICD10CM	1	I63.529		Cerebral infarction due to unspecified occlusion or stenc	IMO	GHS-PACDC	IMO_2016_R	ACTIVE	N	1900-01-01	9999-12-31	2016-06-02
Diagnosis: ICD10 Code, I00-I99, Define	I00-I99	Diseases of the circulatory system	203000	Perinatal ischemic right PCA stroke (HCC)	ICD10CM	2	I63.531	P91.0,I63.531	Cerebral infarction due to unspecified occlusion or stenc	IMO	GHS-PACDC	DX_LEGACY	ACTIVE	N	2016-09-17	9999-12-31	2016-09-30
Diagnosis: ICD10 Code, I00-I99, Define	I00-I99	Diseases of the circulatory system	385887	Perinatal ischemic right posterior cerebral artery (PCA) stroke (HCC)	ICD10CM	2	I63.531	P91.0,I63.531	Cerebral infarction due to unspecified occlusion or stenc	IMO	GHS-PACDC	DX_LEGACY	ACTIVE	N	2016-09-17	9999-12-31	2016-09-30
Diagnosis: ICD10 Code, I00-I99, Define	I00-I99	Diseases of the circulatory system	1113706	Stroke due to occlusion of right posterior cerebral artery (HCC)	ICD10CM	1	I63.531		Cerebral infarction due to unspecified occlusion or stenc	IMO	GHS-PACDC	IMO_2016_R	ACTIVE	N	1900-01-01	9999-12-31	2016-06-02
Diagnosis: ICD10 Code, I00-I99, Define	I00-I99	Diseases of the circulatory system	201668	Left acute arterial ischemic stroke, PCA (posterior cerebral artery) (HCC)	ICD10CM	1	I63.532		Cerebral infarction due to unspecified occlusion or stenc	IMO	GHS-PACDC	DX_LEGACY	ACTIVE	N	1900-01-01	9999-12-31	2015-07-10
Diagnosis: ICD10 Code, I00-I99, Define	I00-I99	Diseases of the circulatory system	203003	Arterial ischemic stroke, PCA, left, perinatal (HCC)	ICD10CM	2	I63.532	P96.89,I63.532	Cerebral infarction due to unspecified occlusion or stenc	IMO	GHS-PACDC	DX_LEGACY	ACTIVE	N	1900-01-01	2016-10-14	2015-07-10
Diagnosis: ICD10 Code, I00-I99, Define	I00-I99	Diseases of the circulatory system	385887	Perinatal ischemic right posterior cerebral artery (PCA) stroke (HCC)	ICD10CM	2	I63.532	P96.89,I63.532	Cerebral infarction due to unspecified occlusion or stenc	IMO	GHS-PACDC	DX_LEGACY	ACTIVE	N	1900-01-01	2016-10-14	2015-07-10
Diagnosis: ICD10 Code, I00-I99, Define	I00-I99	Diseases of the circulatory system	1426633	Cerebral infarction due to unspecified occlusion or stenosis of bilateral posterior cerebral arteri	ICD10CM	1	I63.533		Cerebral infarction due to unspecified occlusion or stenc	IMO	GHS-PACDC	IMO_2018_R	ACTIVE	N	1900-01-01	9999-12-31	2017-10-30
Diagnosis: ICD10 Code, I00-I99, Define	I00-I99	Diseases of the circulatory system	780305	Arterial ischemic stroke, multifocal, posterior circulation, acute, unspecified laterality (HCC)	ICD10CM	1	I63.539		Cerebral infarction due to unspecified occlusion or stenc	IMO	GHS-PACDC	IMO_2015_R	ACTIVE	N	1900-01-01	9999-12-31	2015-07-10
Diagnosis: ICD10 Code, I00-I99, Define	I00-I99	Diseases of the circulatory system	1106463	Cerebral infarction due to stenosis of right cerebellar artery (HCC)	ICD10CM	1	I63.541		Cerebral infarction due to unspecified occlusion or stenc	IMO	GHS-PACDC	IMO_2016_R	ACTIVE	N	1900-01-01	9999-12-31	2016-06-02
Diagnosis: ICD10 Code, I00-I99, Define	I00-I99	Diseases of the circulatory system	1113557	Stroke due to occlusion of right cerebellar artery (HCC)	ICD10CM	1	I63.541		Cerebral infarction due to unspecified occlusion or stenc	IMO	GHS-PACDC	IMO_2016_R	ACTIVE	N	1900-01-01	9999-12-31	2016-06-02
Diagnosis: ICD10 Code, I00-I99, Define	I00-I99	Diseases of the circulatory system	796856	Stroke in child due to aneurysm, right	ICD10CM	1	I63.541		Cerebral infarction due to unspecified occlusion or stenc	IMO	GHS-PACDC	IMO_2015_R	DELETED	Y	1900-01-01	9999-12-31	2015-07-10
Diagnosis: ICD10 Code, I00-I99, Define	I00-I99	Diseases of the circulatory system	781609	Stroke in child due to aneurysm, left	ICD10CM	1	I63.542		Cerebral infarction due to unspecified occlusion or stenc	IMO	GHS-PACDC	IMO_2015_R	DELETED	Y	1900-01-01	9999-12-31	2015-07-10
Diagnosis: ICD10 Code, I00-I99, Define	I00-I99	Diseases of the circulatory system	1113586	Stroke due to occlusion of cerebellar artery (HCC)	ICD10CM	1	I63.549		Cerebral infarction due to unspecified occlusion or stenc	IMO	GHS-PACDC	IMO_2016_R	ACTIVE	N	1900-01-01	9999-12-31	2016-06-02
Diagnosis: ICD10 Code, I00-I99, Define	I00-I99	Diseases of the circulatory system	487373	Occlusion of posterior inferior cerebellar artery with infarction (HCC)	ICD10CM	1	I63.549		Cerebral infarction due to unspecified occlusion or stenc	IMO	GHS-PACDC	IMO_2015_R	ACTIVE	N	1900-01-01	9999-12-31	2015-07-10
Diagnosis: ICD10 Code, I00-I99, Define	I00-I99	Diseases of the circulatory system	357167	Stenosis of multiple extra-cranial arteries with cerebral infarction within last 8 weeks	ICD10CM	1	I63.59		Cerebral infarction due to unspecified occlusion or stenc	IMO	GHS-PACDC	DX_LEGACY	ACTIVE	N	1900-01-01	2016-05-07	2015-07-10
Diagnosis: ICD10 Code, I00-I99, Define	I00-I99	Diseases of the circulatory system	607056	Cerebral infarction due to cerebral venous thrombosis, nonpyogenic	ICD10CM	1	I63.6		Cerebral infarction due to cerebral venous thrombosis, r	IMO	GHS-PACDC	IMO_2015_R	ACTIVE	Y	1900-01-01	9999-12-31	2015-07-10
Diagnosis: ICD10 Code, I00-I99, Define	I00-I99	Diseases of the circulatory system	202944	Cerebral venous infarction, idiopathic, perinatal (HCC)	ICD10CM	2	I63.6	P91.0,I63.6	Cerebral infarction due to cerebral venous thrombosis, r	IMO	GHS-PACDC	DX_LEGACY	ACTIVE	N	2016-09-17	9999-12-31	2016-09-30
Diagnosis: ICD10 Code, I00-I99, Define	I00-I99	Diseases of the circulatory system	225909	Acute cerebral venous infarction associated with CSVT (HCC)	ICD10CM	1	I63.6		Cerebral infarction due to cerebral venous thrombosis, r	IMO	GHS-PACDC	DX_LEGACY	ACTIVE	N	2016-04-10	9999-12-31	2015-07-10
Diagnosis: ICD10 Code, I00-I99, Define	I00-I99	Diseases of the circulatory system	202692	Acute cerebral venous infarction (HCC)	ICD10CM	1	I63.8		Other cerebral infarction	IMO	GHS-PACDC	DX_LEGACY	ACTIVE	N	1900-01-01	9999-12-31	2015-07-10
Diagnosis: ICD10 Code, I00-I99, Define	I00-I99	Diseases of the circulatory system	202187	Cerebral infarction, global diffuse, acute (HCC)	ICD10CM	1	I63.8		Other cerebral infarction	IMO	GHS-PACDC	DX_LEGACY	ACTIVE	N	1900-01-01	9999-12-31	2015-07-10
Diagnosis: ICD10 Code, I00-I99, Define	I00-I99	Diseases of the circulatory system	105307	Cerebral artery occlusion syndrome	ICD10CM	1	I63.9		Cerebral infarction, unspecified	IMO	GHS-PACDC	DX_LEGACY	ACTIVE	N	1900-01-01	2016-05-07	2015-07-10
Diagnosis: ICD10 Code, I00-I99, Define	I00-I99	Diseases of the circulatory system	22295	Cerebral artery occlusion	ICD10CM	1	I63.9		Cerebral infarction, unspecified	IMO	GHS-PACDC	DX_LEGACY	ACTIVE	N	1900-01-01	2016-05-07	2015-07-10
Diagnosis: ICD10 Code, I00-I99, Define	I00-I99	Diseases of the circulatory system	1113727	Cerebrovascular accident (CVA) due to thrombosis (HCC)	ICD10CM	1	I63.9		Cerebral infarction, unspecified	IMO	GHS-PACDC	IMO_2016_R	ACTIVE	N	1900-01-01	9999-12-31	2016-06-02
Diagnosis: ICD10 Code, I00-I99, Define	I00-I99	Diseases of the circulatory system	1330113	Hemiplegia of left nondominant side due to infarction of brain, unspecified hemiplegia type (HC	ICD10CM	2	I63.9	G81.94,I63.9	Cerebral infarction, unspecified	IMO	GHS-PACDC	IMO_2017_R	ACTIVE	N	2017-09-17	9999-12-31	2017-10-30
Diagnosis: ICD10 Code, I00-I99, Define	I00-I99	Diseases of the circulatory system	1348197	Persistent migraine aura with cerebral infarction, without mention of intractable migraine with	ICD10CM	2	I63.9	G43.609,I63.9	Cerebral infarction, unspecified	IMO	GHS-PACDC	IMO_2018_R	DELETED	Y	1900-01-01	9999-12-31	2017-10-30
Diagnosis: ICD10 Code, I00-I99, Define	I00-I99	Diseases of the circulatory system	203209	Cerebral infarction, watershed distribution, bilateral, perinatal (HCC)	ICD10CM	2	I63.9	P96.89,I63.9	Cerebral infarction, unspecified	IMO	GHS-PACDC	DX_LEGACY	ACTIVE	N	1900-01-01	2016-10-14	2015-07-10
Diagnosis: ICD10 Code, I00-I99, Define	I00-I99	Diseases of the circulatory system	1113592	Cerebrovascular accident (CVA) due to embolism (HCC)	ICD10CM	1	I63.9		Cerebral infarction, unspecified	IMO	GHS-PACDC	IMO_2016_R	ACTIVE	N	1900-01-01	9999-12-31	2016-06-02
Diagnosis: ICD10 Code, I00-I99, Define	I00-I99	Diseases of the circulatory system	1316155	Spastic hemiplegia of left dominant side due to infarction of brain (HCC)	ICD10CM	2	I63.9	G81.12,I63.9	Cerebral infarction, unspecified	IMO	GHS-PACDC	IMO_2017_R	ACTIVE	N	1900-01-01	9999-12-31	2017-04-14
Diagnosis: ICD10 Code, I00-I99, Define	I00-I99	Diseases of the circulatory system	1125592	Unknown when suspected stroke patient was last well	ICD10CM	1	I63.9		Cerebral infarction, unspecified	IMO	GHS-PACDC	IMO_2016_R	ACTIVE	N	2017-09-17	9999-12-31	2017-10-30
Diagnosis: ICD10 Code, I00-I99, Define	I00-I99	Diseases of the circulatory system	185727	Persistent migraine aura with cerebral infarction, with intractable migraine, so stated, with statu	ICD10CM	2	I63.9	G43.611,I63.9	Cerebral infarction, unspecified	IMO	GHS-PACDC	DX_LEGACY	ACTIVE	Y	1900-01-01	9999-12-31	2017-04-14
Diagnosis: ICD10 Code, I00-I99, Define	I00-I99	Diseases of the circulatory system	264007	Aphasia due to stroke (HCC)	ICD10CM	1	I63.9	I63.9,R47.01	Cerebral infarction, unspecified	IMO	GHS-PACDC	DX_LEGACY	ACTIVE	N	2016-04-10	9999-12-31	2015-07-10
Diagnosis: ICD10 Code, I00-I99, Define	I00-I99	Diseases of the circulatory system	163194	Embolic stroke (HCC)	ICD10CM	1	I63.9		Cerebral infarction, unspecified	IMO	GHS-PACDC	DX_LEGACY	ACTIVE	N	2016-04-10	9999-12-31	2015-07-10
Diagnosis: ICD10 Code, I00-I99, Define	I00-I99	Diseases of the circulatory system	342355	Cerebral infarction, associated with systemic hypoxia or ischemia, in utero (HCC)	ICD10CM	2	I63.9	P91.8,I63.9	Cerebral infarction, unspecified	IMO	GHS-PACDC	DX_LEGACY	ACTIVE	N	2016-04-10	2017-10-14	2015-07-10
Diagnosis: ICD10 Code, I00-I99, Define	I00-I99	Diseases of the circulatory system	262549	Migraine aura, persistent, with cerebral infarct, status migrainosus (HCC)	ICD10CM	2	I63.9	G43.601,I63.9	Cerebral infarction, unspecified	IMO	GHS-PACDC	DX_LEGACY	ACTIVE	N	1900-01-01	9999-12-31	2015-07-10
Diagnosis: ICD10 Code, I00-I99, Define	I00-I99	Diseases of the circulatory system	202962	Cerebral infarction in utero (HCC)	ICD10CM	2	I63.9	P96.89,I63.9	Cerebral infarction, unspecified	IMO	GHS-PACDC	DX_LEGACY	ACTIVE	N	1900-01-01	2016-10-14	2015-07-10
Diagnosis: ICD10 Code, I00-I99, Define	I00-I99	Diseases of the circulatory system	331363	Nondominant hemiplegia as complication of stroke (HCC)	ICD10CM	1	I63.9	I63.9,G81.90	Cerebral infarction, unspecified	IMO	GHS-PACDC	DX_LEGACY	ACTIVE	N	2016-04-10	9999-12-31	2015-07-10
Diagnosis: ICD10 Code, I00-I99, Define	I00-I99	Diseases of the circulatory system	342369	Cerebral infarction, watershed distribution, unilateral, presumed perinatal (HCC)	ICD10CM	2	I63.9	P96.89,I63.9	Cerebral infarction, unspecified	IMO	GHS-PACDC	DX_LEGACY	ACTIVE	N	1900-01-01	2016-10-14	2015-07-10
Diagnosis: ICD10 Code, I00-I99, Define	I00-I99	Diseases of the circulatory system	342355	Cerebral infarction, associated with systemic hypoxia or ischemia, in utero (HCC)	ICD10CM	2	I63.9	P91.88,I63.9	Cerebral infarction, unspecified	IMO	GHS-PACDC	DX_LEGACY	ACTIVE	N	2017-09-17	9999-12-31	2017-10-30
Diagnosis: ICD10 Code, I00-I99, Define	I00-I99	Diseases of the circulatory system	342580	Chronic cerebral infarction associated with systemic hypoxia or ischemia (HCC)	ICD10CM	1	I63.9		Cerebral infarction, unspecified	IMO	GHS-PACDC	DX_LEGACY	ACTIVE	N	1900-01-01	9999-12-31	2015-07-10
Diagnosis: ICD10 Code, I00-I99, Define	I00-I99	Diseases of the circulatory system	1113940	Acute cerebrovascular accident (CVA) within last 8 weeks (HCC)	ICD10CM	1	I63.9		Cerebral infarction, unspecified	IMO	GHS-PACDC	IMO_2016_R	ACTIVE	N	1900-01-01	2017-10-14	2016-06-02
Diagnosis: ICD10 Code, I00-I99, Define	I00-I99	Diseases of the circulatory system	202945	Cerebral venous infarction, perinatal (HCC)	ICD10CM	2	I63.9	P96.89,I63.9	Cerebral infarction, unspecified	IMO	GHS-PACDC	DX_LEGACY	ACTIVE	N	1900-01-01	2016-10-14	2015-07-10
Diagnosis: ICD10 Code, I00-I99, Define	I00-I99	Diseases of the circulatory system	342253	Spont in utero intraparenchymal hemorrhage assoc w/ arterial infarction (HCC)	ICD10CM	3	I63.9	P54.8,I62.9,I63.9	Cerebral infarction, unspecified	IMO	GHS-PACDC	DX_LEGACY	ACTIVE	N	1900-01-01	2016-05-07	2015-07-10
Diagnosis: ICD10 Code, I00-I99, Define	I00-I99	Diseases of the circulatory system	256524	Acute occipital temporal infarction (HCC)	ICD10CM	1	I63.9		Cerebral infarction, unspecified	IMO	GHS-PACDC	DX_LEGACY	ACTIVE	N	1900-01-01	9999-12-31	2015-07-10
Diagnosis: ICD10 Code, I00-I99, Define	I00-I99	Diseases of the circulatory system	487911	Death due to stroke (HCC)	ICD10CM	1	I63.9		Cerebral infarction, unspecified	IMO	GHS-PACDC	IMO_2015_R	ACTIVE	N	1900-01-01	9999-12-31	2015-07-10
Diagnosis: ICD10 Code, I00-I99, Define	I00-I99	Diseases of the circulatory system	342251	Intracranial hemorrhage, spontaneous intraparenchymal, associated with arterial infarction, per	ICD10CM	3	I63.9	P54.8,I62.9,I63.9	Cerebral infarction, unspecified	IMO	GHS-PACDC	DX_LEGACY	ACTIVE	N	1900-01-01	2016-10-14	2015-07-10
Diagnosis: ICD10 Code, I00-I99, Define	I00-I99	Diseases of the circulatory system	342236	Spontaneous in utero intraparenchymal intracranial hemorrhage associated with venous infarct	ICD10CM	3	I63.9	P54.8,I62.9,I63.9	Cerebral infarction, unspecified	IMO	GHS-PACDC	DX_LEGACY	ACTIVE	N	1900-01-01	2016-10-14	2015-07-10
Diagnosis: ICD10 Code, I00-I99, Define	I00-I99	Diseases of the circulatory system	237018	Carotid artery occlusion with infarction (HCC)	ICD10CM	1	I63.9		Cerebral infarction, unspecified	IMO	GHS-PACDC	DX_LEGACY	ACTIVE	N	1900-01-01	2016-05-07	2015-07-10
Diagnosis: ICD10 Code, I00-I99, Define	I00-I99	Diseases of the circulatory system	721389	Vertebral artery narrowing, right	ICD10CM	1	I65.01		Occlusion and stenosis of right vertebral artery	IMO	GHS-PACDC	IMO_2015_R	ACTIVE	N	1900-01-01	9999-12-31	2015-07-10
Diagnosis: ICD10 Code, I00-I99, Define	I00-I99	Diseases of the circulatory system	474592	Embolism of right vertebral artery	ICD10CM	1	I65.01		Occlusion and stenosis of right vertebral artery	IMO	GHS-PACDC	IMO_2015_R	ACTIVE	N	1900-01-01	9999-12-31	2015-07-10
Diagnosis: ICD10 Code, I00-I99, Define	I00-I99	Diseases of the circulatory system	740480	Vertebral artery thrombosis, right	ICD10CM	1	I65.01		Occlusion and stenosis of right vertebral artery	IMO	GHS-PACDC	IMO_2015_R	ACTIVE	N	1900-01-01	9999-12-31	2015-07-10
Diagnosis: ICD10 Code, I00-I99, Define	I00-I99	Diseases of the circulatory system	473866	Stenosis of right vertebral artery	ICD10CM	1	I65.01		Occlusion and stenosis of right vertebral artery	IMO	GHS-PACDC	IMO_2015_R	ACTIVE	N	1900-01-01	9999-12-31	2015-07-10
Diagnosis: ICD10 Code, I00-I99, Define	I00-I99	Diseases of the circulatory system	961239	Occlusion of vertebral artery without cerebral infarction, right	ICD10CM	1	I65.01		Occlusion and stenosis of right vertebral artery	IMO	GHS-PACDC	IMO_2015_R	ACTIVE	N	1900-01-01	9999-12-31	2015-07-10
Diagnosis: ICD10 Code, I00-I99, Define	I00-I99	Diseases of the circulatory system	472643	Occlusion of right vertebral artery	ICD10CM	1	I65.01		Occlusion and stenosis of right vertebral artery	IMO	GHS-PACDC	IMO_2015_R	ACTIVE	N	1900-01-01	9999-12-31	2015-07-10
Diagnosis: ICD10 Code, I00-I99, Define	I00-I99	Diseases of the circulatory system	944687	Occlusion of vertebral artery, right	ICD10CM	1	I65.01		Occlusion and stenosis of right vertebral artery	IMO	GHS-PACDC	IMO_2015_R	ACTIVE	N	1900-01-01	9999-12-31	2015-07-10
Diagnosis: ICD10 Code, I00-I99, Define	I00-I99	Diseases of the circulatory system	589303	Occlusion and stenosis of left vertebral artery	ICD10CM	1	I65.02		Occlusion and stenosis of left vertebral artery	IMO	GHS-PACDC	IMO_2015_R	ACTIVE	Y	1900-01-01	9999-12-31	2015-07-10
Diagnosis: ICD10 Code, I00-I99, Define	I00-I99	Diseases of the circulatory system	947782	Vertebral artery occlusion, bilateral	ICD10CM	1	I65.03		Occlusion and stenosis of bilateral vertebral arteries	IMO	GHS-PACDC	IMO_2015_R	ACTIVE	N	1900-01-01	9999-12-31	2015-07-10
Diagnosis: ICD10 Code, I00-I99, Define	I00-I99	Diseases of the circulatory system	949836	Occlusion of vertebral artery, bilateral	ICD10CM	1	I65.03		Occlusion and stenosis of bilateral vertebral arteries	IMO	GHS-PACDC	IMO_2015_R	ACTIVE	N	1900-01-01	9999-12-31	2015-07-10
Diagnosis: ICD10 Code, I00-I99, Define	I00-I99	Diseases of the circulatory system	928814	Vertebral artery stenosis, bilateral	ICD10CM	1	I65.03		Occlusion and stenosis of bilateral vertebral arteries	IMO	GHS-PACDC	IMO_2015_R	ACTIVE	N	1900-01-01	9999-12-31	2015-07-10
Diagnosis: ICD10 Code, I00-I99, Define	I00-I99	Diseases of the circulatory system	473712	Occlusion of both vertebral arteries	ICD10CM	1	I65.03		Occlusion and stenosis of bilateral vertebral arteries	IMO	GHS-PACDC	IMO_2015_R	ACTIVE	N	1900-01-01	9999-12-31	2015-07-10
Diagnosis: ICD10 Code, I00-I99, Define	I00-I99	Diseases of the circulatory system	75365	Vertebral artery embolus	ICD10CM	1	I65.09		Occlusion and stenosis of unspecified vertebral artery	IMO	GHS-PACDC	DX_LEGACY	ACTIVE	N	1900-01-01	9999-12-31	2015-07-10
Diagnosis: ICD10 Code, I00-I99, Define	I00-I99	Diseases of the circulatory system	22290	Vertebral artery narrowing	ICD10CM	1	I65.09		Occlusion and stenosis of unspecified vertebral artery	IMO	GHS-PACDC	DX_LEGACY	ACTIVE	N	1900-01-01	9999-12-31	2015-07-10
Diagnosis: ICD10 Code, I00-I99, Define	I00-I99	Diseases of the circulatory system	262570	Symptomatic basilar artery stenosis without infarction	ICD10CM	1	I65.1		Occlusion and stenosis of basilar artery	IMO	GHS-PACDC	DX_LEGACY	ACTIVE	N	1900-01-01	9999-12-31	2015-07-10
Diagnosis: ICD10 Code, I00-I99, Define	I00-I99	Diseases of the circulatory system	844077	Internal carotid artery thrombosis, right	ICD10CM	1	I65.21		Occlusion and stenosis of right carotid artery	IMO	GHS-PACDC	IMO_2015_R	ACTIVE	N	1900-01-01	9999-12-31	2015-07-10
Diagnosis: ICD10 Code, I00-I99, Define	I00-I99	Diseases of the circulatory system	732299	Carotid artery embolism, right	ICD10CM	1	I65.21		Occlusion and stenosis of right carotid artery	IMO	GHS-PACDC	IMO_2015_R	ACTIVE	N	1900-01-01	9999-12-31	2015-07-10
Diagnosis: ICD10 Code, I00-I99, Define	I00-I99	Diseases of the circulatory system	475299	Asymptomatic stenosis of right carotid artery without infarction	ICD10CM	1	I65.21		Occlusion and stenosis of right carotid artery	IMO	GHS-PACDC	IMO_2015_R	ACTIVE	N	1900-01-01	9999-12-31	2015-07-10
Diagnosis: ICD10 Code, I00-I99, Define	I00-I99	Diseases of the circulatory system	425368	Intracranial carotid stenosis, right	ICD10CM	1	I65.21		Occlusion and stenosis of right carotid artery	IMO	GHS-PACDC	IMO_2015_R	ACTIVE	N	1900-01-01	9999-12-31	2015-07-10
Diagnosis: ICD10 Code, I00-I99, Define	I00-I99	Diseases of the circulatory system	822263	Asymptomatic carotid artery stenosis without infarction, right	ICD10CM	1	I65.21		Occlusion and stenosis of right carotid artery	IMO	GHS-PACDC	IMO_2015_R	ACTIVE	N	1900-01-01	9999-12-31	2015-07-10
Diagnosis: ICD10 Code, I00-I99, Define	I00-I99	Diseases of the circulatory system	742859	Unilateral carotid artery stenosis, right	ICD10CM	1	I65.21		Occlusion and stenosis of right carotid artery	IMO	GHS-PACDC	IMO_2015_R	ACTIVE	N	1900-01-01	9999-12-31	2015-07-10
Diagnosis: ICD10 Code, I00-I99, Define	I00-I99	Diseases of the circulatory system	739949	Carotid artery stenosis, asymptomatic, right	ICD10CM	1	I65.21		Occlusion and stenosis of right carotid artery	IMO	GHS-PACDC	IMO_2015_R	ACTIVE	N	1900-01-01	9999-12-31	2015-07-10
Diagnosis: ICD10 Code, I00-I99, Define	I00-I99	Diseases of the circulatory system	517421	Obstruction of right carotid artery	ICD10CM	1	I65.21		Occlusion and stenosis of right carotid artery	IMO	GHS-PACDC	IMO_2015_R	ACTIVE	N	1900-01-01	9999-12-31	2015-07-10
Diagnosis: ICD10 Code, I00-I99, Define	I00-I99	Diseases of the circulatory system	477026	Stenosis of left internal carotid artery	ICD10CM	1	I65.22		Occlusion and stenosis of left carotid artery	IMO	GHS-PACDC	IMO_2015_R	ACTIVE	N	1900-01-01	9999-12-31	2015-07-10
Diagnosis: ICD10 Code, I00-I99, Define	I00-I99	Diseases of the circulatory system	768791	ICAO (internal carotid artery occlusion), left	ICD10CM	1	I65.22		Occlusion and stenosis of left carotid artery	IMO	GHS-PACDC	IMO_2015_R	ACTIVE	N	1900-01-01	9999-12-31	2015-07-10
Diagnosis: ICD10 Code, I00-I99, Define	I00-I99	Diseases of the circulatory system	475131	Embolism of left carotid artery	ICD10CM	1	I65.22		Occlusion and stenosis of left carotid artery	IMO	GHS-PACDC	IMO_2015_R	ACTIVE	N	1900-01-01	9999-12-31	2015-07-10
Diagnosis: ICD10 Code, I00-I99, Define	I00-I99	Diseases of the circulatory system	746408	Symptomatic carotid artery stenosis without infarction, left	ICD10CM	1	I65.22		Occlusion and stenosis of left carotid artery	IMO	GHS-PACDC	IMO_2015_R	ACTIVE	N	1900-01-01	9999-12-31	2015-07-10
Diagnosis: ICD10 Code, I00-I99, Define	I00-I99	Diseases of the circulatory system	752646	Thrombosis of internal carotid, left	ICD10CM	1	I65.22		Occlusion and stenosis of left carotid artery	IMO	GHS-PACDC	IMO_2015_R	ACTIVE	N	1900-01-01	9999-12-31	2015-07-10
Diagnosis: ICD10 Code, I00-I99, Define	I00-I99	Diseases of the circulatory system	800843	Stenosis of carotid artery, left	ICD10CM	1	I65.22		Occlusion and stenosis of left carotid artery	IMO	GHS-PACDC	IMO_2015_R	DELETED	Y	1900-01-01	9999-12-31	2015-07-10
Diagnosis: ICD10 Code, I00-I99, Define	I00-I99	Diseases of the circulatory system	745723	Carotid artery stenosis, unilateral, left	ICD10CM	1	I65.22		Occlusion and stenosis of left carotid artery	IMO	GHS-PACDC	IMO_2015_R	ACTIVE	N	1900-01-01	9999-12-31	2015-07-10
Diagnosis: ICD10 Code, I00-I99, Define	I00-I99	Diseases of the circulatory system	1096749	Occlusion of carotid artery without cerebral infarction, left	ICD10CM	1	I65.22		Occlusion and stenosis of left carotid artery	IMO	GHS-PACDC	IMO_2015_R	ACTIVE	N	1900-01-01	9999-12-31	2015-07-10

Diagnosis: ICD10 Code, I00-I99, Define	I00-I99	Diseases of the circulatory system	929104	Carotid artery embolism, bilateral	ICD10CM	1	I65.23		Occlusion and stenosis of bilateral carotid arteries	IMO	GHS-PACDC	IMO_2015_R	ACTIVE	N	1900-01-01	9999-12-31	2015-07-10
Diagnosis: ICD10 Code, I00-I99, Define	I00-I99	Diseases of the circulatory system	482457	Stenosis of both carotid arteries without infarction	ICD10CM	1	I65.23		Occlusion and stenosis of bilateral carotid arteries	IMO	GHS-PACDC	IMO_2015_R	ACTIVE	N	1900-01-01	9999-12-31	2015-07-10
Diagnosis: ICD10 Code, I00-I99, Define	I00-I99	Diseases of the circulatory system	935192	Carotid ulcer, bilateral	ICD10CM	1	I65.23		Occlusion and stenosis of bilateral carotid arteries	IMO	GHS-PACDC	IMO_2015_R	ACTIVE	N	1900-01-01	9999-12-31	2015-07-10
Diagnosis: ICD10 Code, I00-I99, Define	I00-I99	Diseases of the circulatory system	938310	Thrombosis of external carotid, bilateral	ICD10CM	1	I65.23		Occlusion and stenosis of bilateral carotid arteries	IMO	GHS-PACDC	IMO_2015_R	ACTIVE	N	1900-01-01	9999-12-31	2015-07-10
Diagnosis: ICD10 Code, I00-I99, Define	I00-I99	Diseases of the circulatory system	932600	Thrombosis of internal carotid, bilateral	ICD10CM	1	I65.23		Occlusion and stenosis of bilateral carotid arteries	IMO	GHS-PACDC	IMO_2015_R	ACTIVE	N	1900-01-01	9999-12-31	2015-07-10
Diagnosis: ICD10 Code, I00-I99, Define	I00-I99	Diseases of the circulatory system	477046	Asymptomatic stenosis of both carotid arteries without infarction	ICD10CM	1	I65.23		Occlusion and stenosis of bilateral carotid arteries	IMO	GHS-PACDC	IMO_2015_R	ACTIVE	N	1900-01-01	9999-12-31	2015-07-10
Diagnosis: ICD10 Code, I00-I99, Define	I00-I99	Diseases of the circulatory system	938007	Neck artery obstruction, bilateral	ICD10CM	1	I65.23		Occlusion and stenosis of bilateral carotid arteries	IMO	GHS-PACDC	IMO_2015_R	ACTIVE	N	1900-01-01	9999-12-31	2015-07-10
Diagnosis: ICD10 Code, I00-I99, Define	I00-I99	Diseases of the circulatory system	471691	Thrombosis of both internal carotid arteries	ICD10CM	1	I65.23		Occlusion and stenosis of bilateral carotid arteries	IMO	GHS-PACDC	IMO_2015_R	ACTIVE	N	1900-01-01	9999-12-31	2015-07-10
Diagnosis: ICD10 Code, I00-I99, Define	I00-I99	Diseases of the circulatory system	596181	Occlusion and stenosis of unspecified carotid artery	ICD10CM	1	I65.29		Occlusion and stenosis of unspecified carotid artery	IMO	GHS-PACDC	IMO_2015_R	ACTIVE	Y	1900-01-01	9999-12-31	2015-07-10
Diagnosis: ICD10 Code, I00-I99, Define	I00-I99	Diseases of the circulatory system	860064	Common carotid artery thrombosis, unspecified laterality	ICD10CM	1	I65.29		Occlusion and stenosis of unspecified carotid artery	IMO	GHS-PACDC	IMO_2015_R	ACTIVE	N	1900-01-01	9999-12-31	2015-07-10
Diagnosis: ICD10 Code, I00-I99, Define	I00-I99	Diseases of the circulatory system	888610	External carotid artery stenosis, unspecified laterality	ICD10CM	1	I65.29		Occlusion and stenosis of unspecified carotid artery	IMO	GHS-PACDC	IMO_2015_R	DELETED	Y	1900-01-01	9999-12-31	2015-07-10
Diagnosis: ICD10 Code, I00-I99, Define	I00-I99	Diseases of the circulatory system	767913	Carotid stenosis, unspecified laterality	ICD10CM	1	I65.29		Occlusion and stenosis of unspecified carotid artery	IMO	GHS-PACDC	IMO_2015_R	DELETED	Y	1900-01-01	9999-12-31	2015-07-10
Diagnosis: ICD10 Code, I00-I99, Define	I00-I99	Diseases of the circulatory system	762082	Carotid artery calcification, unspecified laterality	ICD10CM	1	I65.29		Occlusion and stenosis of unspecified carotid artery	IMO	GHS-PACDC	IMO_2015_R	ACTIVE	N	1900-01-01	9999-12-31	2015-07-10
Diagnosis: ICD10 Code, I00-I99, Define	I00-I99	Diseases of the circulatory system	877635	Carotid artery occlusion, unspecified laterality	ICD10CM	1	I65.29		Occlusion and stenosis of unspecified carotid artery	IMO	GHS-PACDC	IMO_2015_R	DELETED	Y	1900-01-01	9999-12-31	2015-07-10
Diagnosis: ICD10 Code, I00-I99, Define	I00-I99	Diseases of the circulatory system	735745	Carotid artery occlusion without infarction, unspecified laterality	ICD10CM	1	I65.29		Occlusion and stenosis of unspecified carotid artery	IMO	GHS-PACDC	IMO_2015_R	ACTIVE	N	1900-01-01	9999-12-31	2015-07-10
Diagnosis: ICD10 Code, I00-I99, Define	I00-I99	Diseases of the circulatory system	813727	Carotid artery obstruction, unspecified laterality	ICD10CM	1	I65.29		Occlusion and stenosis of unspecified carotid artery	IMO	GHS-PACDC	IMO_2015_R	ACTIVE	N	1900-01-01	9999-12-31	2015-07-10
Diagnosis: ICD10 Code, I00-I99, Define	I00-I99	Diseases of the circulatory system	874053	Internal carotid artery thrombosis, unspecified laterality	ICD10CM	1	I65.29		Occlusion and stenosis of unspecified carotid artery	IMO	GHS-PACDC	IMO_2015_R	ACTIVE	N	1900-01-01	9999-12-31	2015-07-10
Diagnosis: ICD10 Code, I00-I99, Define	I00-I99	Diseases of the circulatory system	1093770	Occlusion of extracranial carotid artery, unspecified laterality	ICD10CM	1	I65.29		Occlusion and stenosis of unspecified carotid artery	IMO	GHS-PACDC	IMO_2015_R	ACTIVE	N	1900-01-01	9999-12-31	2015-07-10
Diagnosis: ICD10 Code, I00-I99, Define	I00-I99	Diseases of the circulatory system	1094462	Occlusion of carotid artery without cerebral infarction, unspecified laterality	ICD10CM	1	I65.29		Occlusion and stenosis of unspecified carotid artery	IMO	GHS-PACDC	IMO_2015_R	ACTIVE	N	1900-01-01	9999-12-31	2015-07-10
Diagnosis: ICD10 Code, I00-I99, Define	I00-I99	Diseases of the circulatory system	118354	Carotid ulcer	ICD10CM	1	I65.29		Occlusion and stenosis of unspecified carotid artery	IMO	GHS-PACDC	DX_LEGACY	ACTIVE	N	1900-01-01	9999-12-31	2015-07-10
Diagnosis: ICD10 Code, I00-I99, Define	I00-I99	Diseases of the circulatory system	420358	Pontine Artery Occlusion	ICD10CM	1	I65.8		Occlusion and stenosis of other precerebral arteries	IMO	GHS-PACDC	DX_LEGACY	ACTIVE	N	1900-01-01	2016-10-14	2015-07-10
Diagnosis: ICD10 Code, I00-I99, Define	I00-I99	Diseases of the circulatory system	84764	Occlusion and stenosis of multiple and bilateral precerebral arteries without mention of cerebra	ICD10CM	1	I65.8		Occlusion and stenosis of other precerebral arteries	IMO	GHS-PACDC	DX_LEGACY	ACTIVE	Y	1900-01-01	9999-12-31	2015-07-10
Diagnosis: ICD10 Code, I00-I99, Define	I00-I99	Diseases of the circulatory system	160822	Occlusion and stenosis of other specified precerebral artery without mention of cerebral infarct	ICD10CM	1	I65.8		Occlusion and stenosis of other precerebral arteries	IMO	GHS-PACDC	DX_LEGACY	ACTIVE	Y	1900-01-01	9999-12-31	2015-07-10
Diagnosis: ICD10 Code, I00-I99, Define	I00-I99	Diseases of the circulatory system	421944	Occlusion and stenosis of unspecified precerebral artery	ICD10CM	1	I65.9		Occlusion and stenosis of unspecified precerebral artery	IMO	GHS-PACDC	IMO_2015_R	DELETED	Y	1900-01-01	9999-12-31	2015-07-10
Diagnosis: ICD10 Code, I00-I99, Define	I00-I99	Diseases of the circulatory system	22270	Occlusion of precerebral artery	ICD10CM	1	I65.9		Occlusion and stenosis of unspecified precerebral artery	IMO	GHS-PACDC	DX_LEGACY	ACTIVE	N	1900-01-01	9999-12-31	2015-07-10
Diagnosis: ICD10 Code, I00-I99, Define	I00-I99	Diseases of the circulatory system	91056	Occlusion and stenosis of precerebral artery	ICD10CM	1	I65.9		Occlusion and stenosis of unspecified precerebral artery	IMO	GHS-PACDC	DX_LEGACY	ACTIVE	N	1900-01-01	9999-12-31	2015-07-10
Diagnosis: ICD10 Code, I00-I99, Define	I00-I99	Diseases of the circulatory system	157740	Occlusion and stenosis of unspecified precerebral artery without mention of cerebral infarction	ICD10CM	1	I65.9		Occlusion and stenosis of unspecified precerebral artery	IMO	GHS-PACDC	DX_LEGACY	ACTIVE	Y	1900-01-01	9999-12-31	2015-07-10
Diagnosis: ICD10 Code, I00-I99, Define	I00-I99	Diseases of the circulatory system	475821	Embolism of both middle cerebral arteries	ICD10CM	1	I66.03		Occlusion and stenosis of bilateral middle cerebral arter	IMO	GHS-PACDC	IMO_2015_R	ACTIVE	N	1900-01-01	9999-12-31	2015-07-10
Diagnosis: ICD10 Code, I00-I99, Define	I00-I99	Diseases of the circulatory system	938797	Middle cerebral artery embolism, bilateral	ICD10CM	1	I66.03		Occlusion and stenosis of bilateral middle cerebral arter	IMO	GHS-PACDC	IMO_2015_R	ACTIVE	N	1900-01-01	9999-12-31	2015-07-10
Diagnosis: ICD10 Code, I00-I99, Define	I00-I99	Diseases of the circulatory system	775244	Middle cerebral artery stenosis, unspecified laterality	ICD10CM	1	I66.09		Occlusion and stenosis of unspecified middle cerebral ar	IMO	GHS-PACDC	IMO_2015_R	ACTIVE	N	1900-01-01	9999-12-31	2015-07-10
Diagnosis: ICD10 Code, I00-I99, Define	I00-I99	Diseases of the circulatory system	589526	Occlusion and stenosis of right anterior cerebral artery	ICD10CM	1	I66.11		Occlusion and stenosis of right anterior cerebral artery	IMO	GHS-PACDC	IMO_2015_R	ACTIVE	Y	1900-01-01	9999-12-31	2015-07-10
Diagnosis: ICD10 Code, I00-I99, Define	I00-I99	Diseases of the circulatory system	162259	Occlusion and stenosis of anterior cerebral artery	ICD10CM	1	I66.19		Occlusion and stenosis of unspecified anterior cerebral a	IMO	GHS-PACDC	DX_LEGACY	DELETED	Y	1900-01-01	9999-12-31	2015-07-10
Diagnosis: ICD10 Code, I00-I99, Define	I00-I99	Diseases of the circulatory system	475875	Embolism of both posterior cerebral arteries	ICD10CM	1	I66.23		Occlusion and stenosis of bilateral posterior cerebral art	IMO	GHS-PACDC	IMO_2015_R	ACTIVE	N	1900-01-01	9999-12-31	2015-07-10
Diagnosis: ICD10 Code, I00-I99, Define	I00-I99	Diseases of the circulatory system	277465	Occlusion and stenosis of both posterior cerebral arteries	ICD10CM	1	I66.23		Occlusion and stenosis of bilateral posterior cerebral art	IMO	GHS-PACDC	DX_LEGACY	DELETED	Y	1900-01-01	9999-12-31	2015-07-10
Diagnosis: ICD10 Code, I00-I99, Define	I00-I99	Diseases of the circulatory system	386219	Cerebellar artery embolism	ICD10CM	1	I66.3		Occlusion and stenosis of cerebellar arteries	IMO	GHS-PACDC	DX_LEGACY	ACTIVE	N	1900-01-01	9999-12-31	2015-07-10
Diagnosis: ICD10 Code, I00-I99, Define	I00-I99	Diseases of the circulatory system	93609	Cerebral thrombosis	ICD10CM	1	I66.9		Occlusion and stenosis of unspecified cerebral artery	IMO	GHS-PACDC	DX_LEGACY	ACTIVE	N	1900-01-01	9999-12-31	2015-07-10
Diagnosis: ICD10 Code, I00-I99, Define	I00-I99	Diseases of the circulatory system	386025	Dissecting aneurysm of basilar artery (HCC)	ICD10CM	1	I67.1		Cerebral aneurysm, nonruptured	IMO	GHS-PACDC	DX_LEGACY	ACTIVE	N	1900-01-01	2016-10-14	2015-07-10
Diagnosis: ICD10 Code, I00-I99, Define	I00-I99	Diseases of the circulatory system	331559	Aneurysm of right internal carotid artery	ICD10CM	1	I67.1		Cerebral aneurysm, nonruptured	IMO	GHS-PACDC	DX_LEGACY	ACTIVE	N	1900-01-01	9999-12-31	2015-07-10
Diagnosis: ICD10 Code, I00-I99, Define	I00-I99	Diseases of the circulatory system	313653	Nonruptured berry aneurysm	ICD10CM	1	I67.1		Cerebral aneurysm, nonruptured	IMO	GHS-PACDC	DX_LEGACY	ACTIVE	N	1900-01-01	9999-12-31	2015-07-10
Diagnosis: ICD10 Code, I00-I99, Define	I00-I99	Diseases of the circulatory system	120072	Basilar artery aneurysm (HCC)	ICD10CM	1	I67.1		Cerebral aneurysm, nonruptured	IMO	GHS-PACDC	DX_LEGACY	ACTIVE	N	1900-01-01	2017-04-27	2015-07-10
Diagnosis: ICD10 Code, I00-I99, Define	I00-I99	Diseases of the circulatory system	335762	Nonruptured cerebral aneurysm, internal carotid artery, intracranial portion	ICD10CM	1	I67.1		Cerebral aneurysm, nonruptured	IMO	GHS-PACDC	DX_LEGACY	ACTIVE	N	1900-01-01	9999-12-31	2015-07-10
Diagnosis: ICD10 Code, I00-I99, Define	I00-I99	Diseases of the circulatory system	22313	Atheroma of cerebral arteries	ICD10CM	1	I67.2		Cerebral atherosclerosis	IMO	GHS-PACDC	DX_LEGACY	ACTIVE	N	1900-01-01	9999-12-31	2015-07-10
Diagnosis: ICD10 Code, I00-I99, Define	I00-I99	Diseases of the circulatory system	17915	Cerebrovascular disease, arteriosclerotic, post-stroke	ICD10CM	1	I67.2	I67.2,Z86.73	Cerebral atherosclerosis	IMO	GHS-PACDC	DX_LEGACY	ACTIVE	N	1900-01-01	9999-12-31	2015-07-10
Diagnosis: ICD10 Code, I00-I99, Define	I00-I99	Diseases of the circulatory system	117823	Subcortical arteriosclerotic encephalopathy	ICD10CM	1	I67.3		Progressive vascular leukoencephalopathy	IMO	GHS-PACDC	DX_LEGACY	ACTIVE	N	1900-01-01	9999-12-31	2015-07-10
Diagnosis: ICD10 Code, I00-I99, Define	I00-I99	Diseases of the circulatory system	115323	Thrombosis of cerebral veins	ICD10CM	1	I67.6		Nonpyogenic thrombosis of intracranial venous system	IMO	GHS-PACDC	DX_LEGACY	ACTIVE	N	1900-01-01	2016-05-07	2015-07-10
Diagnosis: ICD10 Code, I00-I99, Define	I00-I99	Diseases of the circulatory system	1088157	Cerebral venous thrombosis during pregnancy, second trimester	ICD10CM	2	I67.6	O22.52,I67.6	Nonpyogenic thrombosis of intracranial venous system	IMO	GHS-PACDC	IMO_2015_R	ACTIVE	N	1900-01-01	2016-05-07	2015-07-10
Diagnosis: ICD10 Code, I00-I99, Define	I00-I99	Diseases of the circulatory system	329928	Cerebral vasculitis induced by radiation	ICD10CM	1	I67.7		Cerebral arteritis, not elsewhere classified	IMO	GHS-PACDC	DX_LEGACY	ACTIVE	N	1900-01-01	9999-12-31	2015-07-10
Diagnosis: ICD10 Code, I00-I99, Define	I00-I99	Diseases of the circulatory system	233332	Reversible posterior leukoencephalopathy syndrome	ICD10CM	1	I67.83		Posterior reversible encephalopathy syndrome	IMO	GHS-PACDC	DX_LEGACY	ACTIVE	N	1900-01-01	9999-12-31	2015-07-10
Diagnosis: ICD10 Code, I00-I99, Define	I00-I99	Diseases of the circulatory system	233331	Posterior reversible encephalopathy syndrome	ICD10CM	1	I67.83		Posterior reversible encephalopathy syndrome	IMO	GHS-PACDC	DX_LEGACY	ACTIVE	N	1900-01-01	9999-12-31	2015-07-10
Diagnosis: ICD10 Code, I00-I99, Define	I00-I99	Diseases of the circulatory system	150900	Ischemic encephalopathy	ICD10CM	1	I67.89		Other cerebrovascular disease	IMO	GHS-PACDC	DX_LEGACY	ACTIVE	N	1900-01-01	9999-12-31	2015-07-10
Diagnosis: ICD10 Code, I00-I99, Define	I00-I99	Diseases of the circulatory system	215446	Leukoariosis	ICD10CM	1	I67.89		Other cerebrovascular disease	IMO	GHS-PACDC	DX_LEGACY	ACTIVE	N	1900-01-01	2016-05-07	2015-07-10
Diagnosis: ICD10 Code, I00-I99, Define	I00-I99	Diseases of the circulatory system	343115	CADASIL (cerebral autosomal dominant arteriopathy with subcortical infarcts and leukoencepha	ICD10CM	2	I67.89	I63.9,I67.89,G93.49	Other cerebrovascular disease	IMO	GHS-PACDC	DX_LEGACY	ACTIVE	N	1900-01-01	9999-12-31	2015-07-10
Diagnosis: ICD10 Code, I00-I99, Define	I00-I99	Diseases of the circulatory system	246832	Insufficiency, arterial, cerebral	ICD10CM	1	I67.89		Other cerebrovascular disease	IMO	GHS-PACDC	DX_LEGACY	ACTIVE	N	1900-01-01	2016-05-07	2015-07-10
Diagnosis: ICD10 Code, I00-I99, Define	I00-I99	Diseases of the circulatory system	1321203	Hemiparesis of left dominant side due to cerebrovascular disease (HCC)	ICD10CM	1	I67.9	I67.9,G81.92	Cerebrovascular disease, unspecified	IMO	GHS-PACDC	IMO_2017_R	ACTIVE	N	2017-09-17	9999-12-31	2017-10-30
Diagnosis: ICD10 Code, I00-I99, Define	I00-I99	Diseases of the circulatory system	1329957	Flaccid hemiplegia of right nondominant side due to cerebrovascular disease, unspecified cereb	ICD10CM	2	I67.9	G81.03,I67.9	Cerebrovascular disease, unspecified	IMO	GHS-PACDC	IMO_2017_R	ACTIVE	N	1900-01-01	9999-12-31	2017-04-14
Diagnosis: ICD10 Code, I00-I99, Define	I00-I99	Diseases of the circulatory system	44520	Cerebrovascular disease	ICD10CM	1	I67.9		Cerebrovascular disease, unspecified	IMO	GHS-PACDC	DX_LEGACY	ACTIVE	N	1900-01-01	9999-12-31	2015-07-10
Diagnosis: ICD10 Code, I00-I99, Define	I00-I99	Diseases of the circulatory system	1321198	Spastic hemiparesis of left nondominant side due to cerebrovascular disease (HCC)	ICD10CM	1	I67.9	I67.9,G81.14	Cerebrovascular disease, unspecified	IMO	GHS-PACDC	IMO_2017_R	ACTIVE	N	2017-09-17	9999-12-31	2017-10-30
Diagnosis: ICD10 Code, I00-I99, Define	I00-I99	Diseases of the circulatory system	1316192	Hemiplegia due to cerebrovascular disease (HCC)	ICD10CM	2	I67.9	G81.90,I67.9	Cerebrovascular disease, unspecified	IMO	GHS-PACDC	IMO_2017_R	ACTIVE	N	1900-01-01	9999-12-31	2017-04-14
Diagnosis: ICD10 Code, I00-I99, Define	I00-I99	Diseases of the circulatory system	1316170	Spastic hemiplegia of left nondominant side due to cerebrovascular disease (HCC)	ICD10CM	2	I67.9	G81.14,I67.9	Cerebrovascular disease, unspecified	IMO	GHS-PACDC	IMO_2017_R	ACTIVE	N	1900-01-01	9999-12-31	2017-04-14
Diagnosis: ICD10 Code, I00-I99, Define	I00-I99	Diseases of the circulatory system	652891	Cerebrovascular disorder, obstetric, antepartum, first trimester	ICD10CM	2	I67.9	O99.411,I67.9	Cerebrovascular disease, unspecified	IMO	GHS-PACDC	IMO_2015_R	ACTIVE	N	1900-01-01	9999-12-31	2015-07-10
Diagnosis: ICD10 Code, I00-I99, Define	I00-I99	Diseases of the circulatory system	418521	Cerebrovascular disease consultation	ICD10CM	2	I67.9	Z71.89,I67.9	Cerebrovascular disease, unspecified	IMO	GHS-PACDC	DX_LEGACY	ACTIVE	N	1900-01-01	9999-12-31	2015-07-10
Diagnosis: ICD10 Code, I00-I99, Define	I00-I99	Diseases of the circulatory system	5176	Ill-defined cerebrovascular disease	ICD10CM	1	I67.9		Cerebrovascular disease, unspecified	IMO	GHS-PACDC	DX_LEGACY	ACTIVE	N	2016-04-10	9999-12-31	2015-07-10
Diagnosis: ICD10 Code, I00-I99, Define	I00-I99	Diseases of the circulatory system	1321192	Spastic hemiparesis of left dominant side due to cerebrovascular disease (HCC)	ICD10CM	1	I67.9	I67.9,G81.12	Cerebrovascular disease, unspecified	IMO	GHS-PACDC	IMO_2017_R	ACTIVE	N	2017-09-17	9999-12-31	2017-10-30
Diagnosis: ICD10 Code, I00-I99, Define	I00-I99	Diseases of the circulatory system	208909	Disease of blood vessels in brain after chickenpox	ICD10CM	2	I67.9	B01.89,I67.9	Cerebrovascular disease, unspecified	IMO	GHS-PACDC	DX_LEGACY	ACTIVE	N	1900-01-01	9999-12-31	2015-07-10
Diagnosis: ICD10 Code, I00-I99, Define	I00-I99	Diseases of the circulatory system	1330123	Spastic hemiplegia of left nondominant side due to cerebrovascular disease, unspecified cerebr	ICD10CM	2	I67.9	G81.14,I67.9	Cerebrovascular disease, unspecified	IMO	GHS-PACDC	IMO_2017_R	ACTIVE	N	1900-01-01	9999-12-31	2017-04-14
Diagnosis: ICD10 Code, I00-I99, Define	I00-I99	Diseases of the circulatory system	1099085	Puerp cerebvas dis-deliv	ICD10CM	2	I67.9	O99.42,I67.9	Cerebrovascular disease, unspecified	IMO	GHS-PACDC	IMO_2016_R	ACTIVE	N	1900-01-01	9999-12-31	2016-06-02
Diagnosis: ICD10 Code, I00-I99, Define	I00-I99	Diseases of the circulatory system	539038	Cerebrovascular disease, unspecified	ICD10CM	1	I67.9		Cerebrovascular disease, unspecified	IMO	GHS-PACDC	IMO_2015_R	ACTIVE	Y	1900-01-01	9999-12-31	2015-07-10
Diagnosis: ICD10 Code, I00-I99, Define	I00-I99	Diseases of the circulatory system	68184	Cerebral vascular insufficiency	ICD10CM	1	I67.9		Cerebrovascular disease, unspecified	IMO	GHS-PACDC	DX_LEGACY	ACTIVE	N	1900-01-01	9999-12-31	2015-07-10
Diagnosis: ICD10 Code, I00-I99, Define	I00-I99	Diseases of the circulatory system	537838	Cerebral amyloid angiopathy	ICD10CM	1	I68.0		Cerebral amyloid angiopathy	IMO	GHS-PACDC	IMO_2015_R	ACTIVE	Y	1900-01-01	9999-12-31	2015-07-10
Diagnosis: ICD10 Code, I00-I99, Define	I00-I99	Diseases of the circulatory system	152722	Amyloidosis VI (HCC)	ICD10CM	2	I68.0	E85.4,I68.0	Cerebral amyloid angiopathy	IMO	GHS-PACDC	DX_LEGACY	ACTIVE	N	2016-04-10	9999-12-31	2015-07-10
Diagnosis: ICD10 Code, I00-I99, Define	I00-I99	Diseases of the circulatory system	597567	Unspecified sequelae of nontraumatic subarachnoid hemorrhage	ICD10CM	1	I69.00		Unspecified sequelae of nontraumatic subarachnoid her	IMO	GHS-PACDC	IMO_2015_R	ACTIVE	Y	1900-01-01	9999-12-31	2015-07-10
Diagnosis: ICD10 Code, I00-I99, Define	I00-I99	Diseases of the circulatory system	1275437	Other symptoms and signs involving cognitive functions following nontraumatic subarachnoid h	ICD10CM	1	I69.018		Other symptoms and signs involving cognitive functions	IMO	GHS-PACDC	IMO_2017_R	ACTIVE	Y	2016-09-17	9999-12-31	2016-09-30
Diagnosis: ICD10 Code, I00-I99, Define	I00-I99	Diseases of the circulatory system	591869	Dysphasia following nontraumatic subarachnoid hemorrhage	ICD10CM	1	I69.021		Dysphasia following nontraumatic subarachnoid hemorr	IMO	GHS-PACDC	IMO_2015_R	ACTIVE	Y	1900-01-01	9999-12-31	2015-07-10
Diagnosis: ICD10 Code, I00-I99, Define	I00-I99	Diseases of the circulatory system	597116	Monoplegia of lower limb following nontraumatic subarachnoid hemorrhage affecting unspecifi	ICD10CM	1	I69.049		Monoplegia of lower limb following nontraumatic subar	IMO	GHS-PACDC	IMO_2015_R	ACTIVE	Y	1900-01-01	9999-12-31	2015-07-10
Diagnosis: ICD10 Code, I00-I99, Define	I00-I99	Diseases of the circulatory system	1366823	Monoplg low lmb following ntrm subarach hemor aff unsp side (HCC)	ICD10CM	1	I69.049		Monoplegia of lower limb following nontraumatic subar	IMO	GHS-PACDC	IMO_2018_R	ACTIVE	N	1900-01-01	9999-12-31	2017-10-30
Diagnosis: ICD10 Code, I00-I99, Define	I00-I99	Diseases of the circulatory system	1316162	Hemiplegia of left dominant side due to nontraumatic subarachnoid hemorrhage (HCC)	ICD10CM	1	I69.052		Hemiplegia and hemiparesis following nontraumatic sub	IMO	GHS-PACDC	IMO_2017_R	ACTIVE	N	1900-01-01	9999-12-31	2017-04-14
Diagnosis: ICD10 Code, I00-I99, Define	I00-I99	Diseases of the circulatory system	1321222	Hemiparesis of left nondominant side due to nontraumatic subarachnoid hemorrhage (HCC)	ICD10CM	1	I69.054		Hemiplegia and hemiparesis following nontraumatic sub	IMO	GHS-PACDC	IMO_2017_R	ACTIVE	N	1900-01-01	2017-10-14	2017-04-14
Diagnosis: ICD10 Code, I00-I99, Define	I00-I99	Diseases of the circulatory system	592202	Cognitive deficits following nontraumatic intracerebral hemorrhage	ICD10CM	1	I69.11		Cognitive deficits following nontraumatic intracerebral h	IMO	GHS-PACDC	IMO_2015_R	ACTIVE	Y	1900-01-01	9999-12-31	2015-07-10
Diagnosis: ICD10 Code, I00-I99, Define	I00-I99	Diseases of the circulatory system	1272876	Visuospatial deficit and spatial neglect after nontraumatic intracerebral hemorrhage	ICD10CM	1	I69.112		Visuospatial deficit and spatial neglect following nontrau	IMO	GHS-PACDC	IMO_2017_R	ACTIVE	N	1900-01-01	9999-12-31	2016-09-30
Diagnosis: ICD10 Code, I00-I99, Define	I00-I99	Diseases of the circulatory system	1291658	Psychomotor deficit following nontraumatic intracerebral hemorrhage	ICD10CM	1	I69.113		Psychomotor deficit following nontraumatic intracerebra	IMO	GHS-PACDC	IMO_2017_R	ACTIVE	N	1900-01-01	9999-12-31	2016-09-30
Diagnosis: ICD10 Code, I00-I99, Define	I00-I99	Diseases of the circulatory system	1366905	Monoplg upr lmb fol ntrm intcrbl hemor aff left nondom side (HCC)	ICD10CM	1	I69.134		Monoplegia of upper limb following nontraumatic intrac	IMO	GHS-PACDC	IMO_2018_R	ACTIVE	N	1900-01-01	9999-12-31	2017-10-30
Diagnosis: ICD10 Code, I00-I99, Define	I00-I99	Diseases of the circulatory system	1366365	Monoplg upr lmb following ntrm intcrbl hemor aff unsp side (HCC)	ICD10CM	1	I69.139		Monoplegia of upper limb following nontraumatic intrac	IMO	GHS-PACDC	IMO_2018_R	ACTIVE	N	1900-01-01	9999-12-31	2017-10-30
Diagnosis: ICD10 Code, I00-I99, Define	I00-I99	Diseases of the circulatory system	355314	Monoplegia of lower extremity following nontraumatic intracerebral hemorrhage affecting righ	ICD10CM	1	I69.141		Monoplegia of lower limb following nontraumatic intrac	IMO	GHS-PACDC	DX_LEGACY	ACTIVE	N	1900-01-01	9999-12-31	2015-07-10
Diagnosis: ICD10 Code, I00-I99, Define	I00-I99	Diseases of the circulatory system	1365683	Monoplg low lmb fol ntrm intcrbl hemor aff left dom side (HCC)	ICD10CM	1	I69.142		Monoplegia of lower limb following nontraumatic intrac	IMO	GHS-PACDC	IMO_2018_R	ACTIVE	N	1900-01-01	9999-12-31	2017-10-30
Diagnosis: ICD10 Code, I00-I99, Define	I00-I99	Diseases of the circulatory system	349493	Monoplegia of lower extremity following nontraumatic intracerebral hemorrhage affecting righ	ICD10CM	1	I69.143		Monoplegia of lower limb following nontraumatic intrac	IMO	GHS-PACDC	DX_LEGACY	ACTIVE	N	1900-01-01	9999-12-31	2015-07-10
Diagnosis: ICD10 Code, I00-I99, Define	I00-I99	Diseases of the circulatory system	1180277	Monoplegia of lower limb following nontraumatic intracerebral hemorrhage affecting unspecifi	ICD10CM	1	I69.149		Monoplegia of lower limb following nontraumatic intrac	IMO	GHS-PACDC	IMO_2016_R	ACTIVE	N	1900-01-01	9999-12-31	2016-06-02
Diagnosis: ICD10 Code, I00-I99, Define	I00-I99	Diseases of the circulatory system	1182397	Hemiplegia and hemiparesis following nontraumatic intracerebral hemorrhage affecting right n	ICD10CM	1	I69.153		Hemiplegia and hemiparesis following nontraumatic intr	IMO	GHS-PACDC	IMO_2016_R	ACTIVE	N	1900-01-01	9999-12-31	2016-06-02
Diagnosis: ICD10 Code, I00-I99, Define	I00-I99	Diseases of the circulatory system	1182398	Hemiplegia and hemiparesis following nontraumatic intracerebral hemorrhage affecting unspec	ICD10CM	1	I69.159		Hemiplegia and hemiparesis following nontraumatic intr	IMO	GHS-PACDC	IMO_2016_R	ACTIVE	N	1900-01-01	9999-12-31	2016-06-02
Diagnosis: ICD10 Code, I00-I99, Define	I00-I99	Diseases of the circulatory system	361315	Hemiparesis due to old intracerebral hemorrhage (HCC)	ICD10CM	1	I69.159		Hemiplegia and hemiparesis following nontraumatic intr	IMO	GHS-PACDC	DX_LEGACY	ACTIVE	N	1900-01-01	9999-12-31	2015-07-10
Diagnosis: ICD10 Code, I00-I99, Define	I00-I99	Diseases of the circulatory system	1178151	Other paralytic syndrome following nontraumatic intracerebral hemorrhage affecting right dom	ICD10CM	1	I69.161		Other paralytic syndrome following nontraumatic intrac	IMO	GHS-PACDC	IMO_2016_R	ACTIVE	N	1900-01-01	9999-12-31	2016-06-02
Diagnosis: ICD10 Code, I00-I99, Define	I00-I99	Diseases of the circulatory system	641765	Oth parlyt synd fol ntrm intcrbl hemor aff right nondom side	ICD10CM	1	I69.163		Other paralytic syndrome following nontraumatic intrac	IMO	GHS-PACDC	IMO_2015_R	DELETED	Y	1900-01-01	9999-12-31	2015-07-10
Diagnosis: ICD10 Code, I00-I99, Define	I00-I99	Diseases of the circulatory system	1356930	Other paralytic syndrome following nontraumatic intracerebral hemorrhage (HCC)	ICD10CM	1	I69.169		Other paralytic syndrome following nontraumatic intrac	IMO	GHS-PACDC	IMO_2018_R	ACTIVE	N	1900-01-01	9999-12-31	2017-10-30
Diagnosis: ICD10 Code, I00-I99, Define	I00-I99	Diseases of the circulatory system	641520	Oth paralytic syndrome fol ntrm intcrbl hemor aff unsp side	ICD10CM	1	I69.169		Other paralytic syndrome following nontraumatic intrac	IMO	GHS-PACDC	IMO_2015_R	DELETED	Y	1900-01-01	9999-12-31	2015-07-10
Diagnosis: ICD10 Code, I00-I99, Define	I00-I99	Diseases of the circulatory system	1291652	Memory deficit following other nontraumatic intracranial hemorrhage	ICD10CM	1	I69.211		Memory deficit following other nontraumatic intracrani	IMO	GHS-PACDC	IMO_2017_R	ACTIVE	N	1900-01-01	9999-12-31	2016-09-30
Diagnosis: ICD10 Code, I00-I99, Define	I00-I99	Diseases of the circulatory system	1275481	Memory deficit following other nontraumatic intracranial hemorrhage	ICD10CM	1	I69.211		Memory deficit following other nontraumatic intracrani	IMO	GHS-PACDC	IMO_2017_R	ACTIVE	Y	2016-09-17	9999-12-31	2016-09-30

Diagnosis: ICD10 Code, I00-I99, Define	I00-I99	Diseases of the circulatory system	1178211	Cognitive deficits following other nontraumatic intracranial hemorrhage	ICD10CM	1	I69.219		Unspecified symptoms and signs involving cognitive func	IMO	GHS-PACDC	IMO_2016_R	DELETED	Y	2016-09-17	9999-12-31	2016-09-30
Diagnosis: ICD10 Code, I00-I99, Define	I00-I99	Diseases of the circulatory system	1357076	Monoplegia of upper extremity following other nontraumatic intracranial hemorrhage affecting	ICD10CM	1	I69.232		Monoplegia of upper limb following other nontraumatic	IMO	GHS-PACDC	IMO_2018_R	ACTIVE	N	1900-01-01	9999-12-31	2017-10-30
Diagnosis: ICD10 Code, I00-I99, Define	I00-I99	Diseases of the circulatory system	1185451	Monoplegia of upper limb following other nontraumatic intracranial hemorrhage affecting left r	ICD10CM	1	I69.234		Monoplegia of upper limb following other nontraumatic	IMO	GHS-PACDC	IMO_2016_R	ACTIVE	N	1900-01-01	9999-12-31	2016-06-02
Diagnosis: ICD10 Code, I00-I99, Define	I00-I99	Diseases of the circulatory system	1185068	Monoplegia of lower limb following other nontraumatic intracranial hemorrhage affecting left c	ICD10CM	1	I69.242		Monoplegia of lower limb following other nontraumatic	IMO	GHS-PACDC	IMO_2016_R	ACTIVE	N	1900-01-01	9999-12-31	2016-06-02
Diagnosis: ICD10 Code, I00-I99, Define	I00-I99	Diseases of the circulatory system	1179157	Monoplegia of lower limb following other nontraumatic intracranial hemorrhage affecting unsp	ICD10CM	1	I69.249		Monoplegia of lower limb following other nontraumatic	IMO	GHS-PACDC	IMO_2016_R	ACTIVE	N	1900-01-01	9999-12-31	2016-06-02
Diagnosis: ICD10 Code, I00-I99, Define	I00-I99	Diseases of the circulatory system	1329540	Hemiplegia of right dominant side due to other nontraumatic intracranial hemorrhage (HCC)	ICD10CM	1	I69.251		Hemiplegia and hemiparesis following other nontrauma	IMO	GHS-PACDC	IMO_2017_R	DELETED	Y	1900-01-01	9999-12-31	2017-04-14
Diagnosis: ICD10 Code, I00-I99, Define	I00-I99	Diseases of the circulatory system	641538	Oth parlyt synd fol oth ntrm intcrn hemor aff left dom side	ICD10CM	1	I69.262		Other paralytic syndrome following other nontraumatic	IMO	GHS-PACDC	IMO_2015_R	DELETED	Y	1900-01-01	9999-12-31	2015-07-10
Diagnosis: ICD10 Code, I00-I99, Define	I00-I99	Diseases of the circulatory system	597247	Other paralytic syndrome following other nontraumatic intracranial hemorrhage, bilateral	ICD10CM	1	I69.265		Other paralytic syndrome following other nontraumatic	IMO	GHS-PACDC	IMO_2015_R	ACTIVE	Y	1900-01-01	9999-12-31	2015-07-10
Diagnosis: ICD10 Code, I00-I99, Define	I00-I99	Diseases of the circulatory system	202621	Chronic ischemic multifocal anterior circulation stroke	ICD10CM	1	I69.30		Unspecified sequelae of cerebral infarction	IMO	GHS-PACDC	DX_LEGACY	ACTIVE	N	2016-09-17	9999-12-31	2016-09-30
Diagnosis: ICD10 Code, I00-I99, Define	I00-I99	Diseases of the circulatory system	327881	Chronic ischemic left posterior cerebral artery (PCA) stroke	ICD10CM	1	I69.30		Unspecified sequelae of cerebral infarction	IMO	GHS-PACDC	DX_LEGACY	ACTIVE	N	2016-09-17	9999-12-31	2016-09-30
Diagnosis: ICD10 Code, I00-I99, Define	I00-I99	Diseases of the circulatory system	1114471	History of cerebrovascular accident (CVA) with residual deficit	ICD10CM	1	I69.30		Unspecified sequelae of cerebral infarction	IMO	GHS-PACDC	IMO_2016_R	ACTIVE	N	1900-01-01	9999-12-31	2016-06-02
Diagnosis: ICD10 Code, I00-I99, Define	I00-I99	Diseases of the circulatory system	204503	Arterial ischemic stroke, chronic	ICD10CM	1	I69.30		Unspecified sequelae of cerebral infarction	IMO	GHS-PACDC	DX_LEGACY	ACTIVE	N	2016-04-10	9999-12-31	2015-07-10
Diagnosis: ICD10 Code, I00-I99, Define	I00-I99	Diseases of the circulatory system	202605	Chronic left arterial ischemic stroke, PCA (posterior cerebral artery)	ICD10CM	1	I69.30		Unspecified sequelae of cerebral infarction	IMO	GHS-PACDC	DX_LEGACY	ACTIVE	N	2016-09-17	9999-12-31	2016-09-30
Diagnosis: ICD10 Code, I00-I99, Define	I00-I99	Diseases of the circulatory system	202269	Chronic bilateral cerebral infarction in watershed distribution	ICD10CM	1	I69.30		Unspecified sequelae of cerebral infarction	IMO	GHS-PACDC	DX_LEGACY	ACTIVE	N	2016-04-10	9999-12-31	2015-07-10
Diagnosis: ICD10 Code, I00-I99, Define	I00-I99	Diseases of the circulatory system	342014	Arterial ischemic stroke, ACA (anterior cerebral artery), left, chronic	ICD10CM	1	I69.30		Unspecified sequelae of cerebral infarction	IMO	GHS-PACDC	DX_LEGACY	ACTIVE	N	2016-04-10	9999-12-31	2015-07-10
Diagnosis: ICD10 Code, I00-I99, Define	I00-I99	Diseases of the circulatory system	202597	Chronic right arterial ischemic stroke, MCA (middle cerebral artery)	ICD10CM	1	I69.30		Unspecified sequelae of cerebral infarction	IMO	GHS-PACDC	DX_LEGACY	ACTIVE	N	2016-09-17	9999-12-31	2016-09-30
Diagnosis: ICD10 Code, I00-I99, Define	I00-I99	Diseases of the circulatory system	252613	Cognitive deficit due to old embolic stroke	ICD10CM	1	I69.31		Cognitive deficits following cerebral infarction	IMO	GHS-PACDC	DX_LEGACY	ACTIVE	N	2016-04-10	2016-10-14	2015-07-10
Diagnosis: ICD10 Code, I00-I99, Define	I00-I99	Diseases of the circulatory system	384507	Multiple old cerebral infarcts with cognitive deficit	ICD10CM	1	I69.31		Cognitive deficits following cerebral infarction	IMO	GHS-PACDC	DX_LEGACY	ACTIVE	N	2016-04-10	2016-10-14	2015-07-10
Diagnosis: ICD10 Code, I00-I99, Define	I00-I99	Diseases of the circulatory system	246406	CVA, old, aphasia	ICD10CM	1	I69.320		Aphasia following cerebral infarction	IMO	GHS-PACDC	DX_LEGACY	ACTIVE	N	2016-04-10	9999-12-31	2015-07-10
Diagnosis: ICD10 Code, I00-I99, Define	I00-I99	Diseases of the circulatory system	264006	Aphasia with stroke (HCC)	ICD10CM	1	I69.320		Aphasia following cerebral infarction	IMO	GHS-PACDC	DX_LEGACY	ACTIVE	N	1900-01-01	2016-05-07	2015-07-10
Diagnosis: ICD10 Code, I00-I99, Define	I00-I99	Diseases of the circulatory system	227471	Aphasia due to neonatal stroke	ICD10CM	2	I69.320	P29.89,I69.320	Aphasia following cerebral infarction	IMO	GHS-PACDC	DX_LEGACY	ACTIVE	N	2016-09-17	9999-12-31	2016-09-30
Diagnosis: ICD10 Code, I00-I99, Define	I00-I99	Diseases of the circulatory system	253035	Dysphasia due to old cerebrovascular accident	ICD10CM	1	I69.321		Dysphasia following cerebral infarction	IMO	GHS-PACDC	DX_LEGACY	ACTIVE	N	1900-01-01	9999-12-31	2015-07-10
Diagnosis: ICD10 Code, I00-I99, Define	I00-I99	Diseases of the circulatory system	589729	Dysarthria following cerebral infarction	ICD10CM	1	I69.322		Dysarthria following cerebral infarction	IMO	GHS-PACDC	IMO_2015_R	ACTIVE	Y	1900-01-01	9999-12-31	2015-07-10
Diagnosis: ICD10 Code, I00-I99, Define	I00-I99	Diseases of the circulatory system	383743	Dysarthria due to recent cerebrovascular accident	ICD10CM	1	I69.322		Dysarthria following cerebral infarction	IMO	GHS-PACDC	DX_LEGACY	ACTIVE	N	2016-04-10	9999-12-31	2015-07-10
Diagnosis: ICD10 Code, I00-I99, Define	I00-I99	Diseases of the circulatory system	379208	Hemiparesis, speech and language deficits, and cognitive deficits due to recent cerebral infarctio	ICD10CM	2	I69.328	I69.359,I69.328,I69.319	Other speech and language deficits following cerebral in	IMO	GHS-PACDC	DX_LEGACY	ACTIVE	N	2016-09-17	9999-12-31	2016-09-30
Diagnosis: ICD10 Code, I00-I99, Define	I00-I99	Diseases of the circulatory system	356092	Monoplegia of upper extremity following cerebral infarction affecting left dominant side (HCC)	ICD10CM	1	I69.332		Monoplegia of upper limb following cerebral infarction a	IMO	GHS-PACDC	DX_LEGACY	ACTIVE	N	1900-01-01	9999-12-31	2015-07-10
Diagnosis: ICD10 Code, I00-I99, Define	I00-I99	Diseases of the circulatory system	343255	Upper limb monoplegia of nondominant side status post cerebrovascular accident (HCC)	ICD10CM	1	I69.339		Monoplegia of upper limb following cerebral infarction a	IMO	GHS-PACDC	DX_LEGACY	ACTIVE	N	2016-04-10	9999-12-31	2015-07-10
Diagnosis: ICD10 Code, I00-I99, Define	I00-I99	Diseases of the circulatory system	254164	Monoplegia of upper limb due to old cerebral infarction (HCC)	ICD10CM	1	I69.339		Monoplegia of upper limb following cerebral infarction a	IMO	GHS-PACDC	DX_LEGACY	ACTIVE	N	2016-04-10	9999-12-31	2015-07-10
Diagnosis: ICD10 Code, I00-I99, Define	I00-I99	Diseases of the circulatory system	598996	Monoplegia of lower limb following cerebral infarction affecting right dominant side	ICD10CM	1	I69.341		Monoplegia of lower limb following cerebral infarction a	IMO	GHS-PACDC	IMO_2015_R	ACTIVE	Y	1900-01-01	9999-12-31	2015-07-10
Diagnosis: ICD10 Code, I00-I99, Define	I00-I99	Diseases of the circulatory system	600134	Monoplegia of lower limb following cerebral infarction affecting right non-dominant side	ICD10CM	1	I69.343		Monoplegia of lower limb following cerebral infarction a	IMO	GHS-PACDC	IMO_2015_R	ACTIVE	Y	1900-01-01	9999-12-31	2015-07-10
Diagnosis: ICD10 Code, I00-I99, Define	I00-I99	Diseases of the circulatory system	15355	Monoplegia, non-dominant lower extremity, post-stroke (HCC)	ICD10CM	1	I69.349		Monoplegia of lower limb following cerebral infarction a	IMO	GHS-PACDC	DX_LEGACY	ACTIVE	N	1900-01-01	2016-05-07	2015-07-10
Diagnosis: ICD10 Code, I00-I99, Define	I00-I99	Diseases of the circulatory system	624264	Hemiplegia and hemiparesis following cerebral infarction	ICD10CM	1	I69.35		Hemiplegia and hemiparesis following cerebral infarctio	IMO	GHS-PACDC	IMO_2015_R	ACTIVE	Y	1900-01-01	9999-12-31	2015-07-10
Diagnosis: ICD10 Code, I00-I99, Define	I00-I99	Diseases of the circulatory system	1321190	Hemiparesis of left dominant side due to cerebral infarction (HCC)	ICD10CM	1	I69.352		Hemiplegia and hemiparesis following cerebral infarctio	IMO	GHS-PACDC	IMO_2017_R	ACTIVE	N	1900-01-01	2017-10-14	2017-04-14
Diagnosis: ICD10 Code, I00-I99, Define	I00-I99	Diseases of the circulatory system	601507	Hemiplegia and hemiparesis following cerebral infarction affecting right non-dominant side	ICD10CM	1	I69.353		Hemiplegia and hemiparesis following cerebral infarctio	IMO	GHS-PACDC	IMO_2015_R	ACTIVE	Y	1900-01-01	9999-12-31	2015-07-10
Diagnosis: ICD10 Code, I00-I99, Define	I00-I99	Diseases of the circulatory system	1316194	Hemiplegia of right nondominant side due to infarction of brain (HCC)	ICD10CM	1	I69.353		Hemiplegia and hemiparesis following cerebral infarctio	IMO	GHS-PACDC	IMO_2017_R	ACTIVE	N	1900-01-01	9999-12-31	2017-04-14
Diagnosis: ICD10 Code, I00-I99, Define	I00-I99	Diseases of the circulatory system	1321250	Hemiparesis of left nondominant side due to cerebral infarction (HCC)	ICD10CM	1	I69.354		Hemiplegia and hemiparesis following cerebral infarctio	IMO	GHS-PACDC	IMO_2017_R	ACTIVE	N	1900-01-01	2017-10-14	2017-04-14
Diagnosis: ICD10 Code, I00-I99, Define	I00-I99	Diseases of the circulatory system	361313	Hemiparesis due to recent cerebrovascular accident (HCC)	ICD10CM	1	I69.359		Hemiplegia and hemiparesis following cerebral infarctio	IMO	GHS-PACDC	DX_LEGACY	ACTIVE	N	2016-04-10	9999-12-31	2015-07-10
Diagnosis: ICD10 Code, I00-I99, Define	I00-I99	Diseases of the circulatory system	1114479	Hemiparesis following cerebrovascular accident (CVA) (HCC)	ICD10CM	1	I69.359		Hemiplegia and hemiparesis following cerebral infarctio	IMO	GHS-PACDC	IMO_2016_R	ACTIVE	N	1900-01-01	9999-12-31	2016-06-02
Diagnosis: ICD10 Code, I00-I99, Define	I00-I99	Diseases of the circulatory system	426390	Hemiplegia due to recent cerebrovascular accident (HCC)	ICD10CM	1	I69.359		Hemiplegia and hemiparesis following cerebral infarctio	IMO	GHS-PACDC	IMO_2015_R	ACTIVE	N	1900-01-01	9999-12-31	2015-07-10
Diagnosis: ICD10 Code, I00-I99, Define	I00-I99	Diseases of the circulatory system	326959	Bilateral paralysis complicating stroke (HCC)	ICD10CM	1	I69.365		Other paralytic syndrome following cerebral infarction, I	IMO	GHS-PACDC	DX_LEGACY	ACTIVE	N	1900-01-01	2016-05-07	2015-07-10
Diagnosis: ICD10 Code, I00-I99, Define	I00-I99	Diseases of the circulatory system	331376	Paralysis as complication of stroke (HCC)	ICD10CM	1	I69.369		Other paralytic syndrome following cerebral infarction a	IMO	GHS-PACDC	DX_LEGACY	ACTIVE	N	1900-01-01	2016-05-07	2015-07-10
Diagnosis: ICD10 Code, I00-I99, Define	I00-I99	Diseases of the circulatory system	228289	Paralytic syndrome nondominant side S/P CVA (cerebrovascular accident) (HCC)	ICD10CM	1	I69.369		Other paralytic syndrome following cerebral infarction a	IMO	GHS-PACDC	DX_LEGACY	ACTIVE	N	1900-01-01	9999-12-31	2015-07-10
Diagnosis: ICD10 Code, I00-I99, Define	I00-I99	Diseases of the circulatory system	1114469	Dysphagia following cerebrovascular accident (CVA)	ICD10CM	1	I69.391		Dysphagia following cerebral infarction	IMO	GHS-PACDC	IMO_2016_R	ACTIVE	N	1900-01-01	9999-12-31	2016-06-02
Diagnosis: ICD10 Code, I00-I99, Define	I00-I99	Diseases of the circulatory system	262578	Dysphagia due to old stroke	ICD10CM	1	I69.391		Dysphagia following cerebral infarction	IMO	GHS-PACDC	DX_LEGACY	ACTIVE	N	1900-01-01	9999-12-31	2015-07-10
Diagnosis: ICD10 Code, I00-I99, Define	I00-I99	Diseases of the circulatory system	262576	CVA, old, dysphagia	ICD10CM	1	I69.391		Dysphagia following cerebral infarction	IMO	GHS-PACDC	DX_LEGACY	ACTIVE	N	2016-04-10	9999-12-31	2015-07-10
Diagnosis: ICD10 Code, I00-I99, Define	I00-I99	Diseases of the circulatory system	253456	Homonymous hemianopsia due to old cerebral infarction	ICD10CM	1	I69.398	I69.398,H53.469	Other sequelae of cerebral infarction	IMO	GHS-PACDC	DX_LEGACY	ACTIVE	N	2016-04-10	9999-12-31	2015-07-10
Diagnosis: ICD10 Code, I00-I99, Define	I00-I99	Diseases of the circulatory system	367157	Visual disturbance due to recent cerebral infarction	ICD10CM	1	I69.398	I69.398,H53.9	Other sequelae of cerebral infarction	IMO	GHS-PACDC	DX_LEGACY	ACTIVE	N	1900-01-01	9999-12-31	2015-07-10
Diagnosis: ICD10 Code, I00-I99, Define	I00-I99	Diseases of the circulatory system	16200	Visual field loss, post-stroke	ICD10CM	1	I69.398	I69.398,H54.7	Other sequelae of cerebral infarction	IMO	GHS-PACDC	DX_LEGACY	ACTIVE	N	1900-01-01	9999-12-31	2015-07-10
Diagnosis: ICD10 Code, I00-I99, Define	I00-I99	Diseases of the circulatory system	383594	Altered sensation due to old embolic stroke	ICD10CM	1	I69.398		Other sequelae of cerebral infarction	IMO	GHS-PACDC	DX_LEGACY	ACTIVE	N	1900-01-01	2016-05-07	2015-07-10
Diagnosis: ICD10 Code, I00-I99, Define	I00-I99	Diseases of the circulatory system	314546	Altered sensation due to stroke (HCC)	ICD10CM	1	I69.398	I69.398,R20.8	Other sequelae of cerebral infarction	IMO	GHS-PACDC	DX_LEGACY	ACTIVE	N	1900-01-01	2016-05-07	2015-07-10
Diagnosis: ICD10 Code, I00-I99, Define	I00-I99	Diseases of the circulatory system	501567	Alteration of sensation as late effect of stroke	ICD10CM	1	I69.398	I69.398,R20.9	Other sequelae of cerebral infarction	IMO	GHS-PACDC	IMO_2015_R	ACTIVE	N	2016-04-10	9999-12-31	2015-07-10
Diagnosis: ICD10 Code, I00-I99, Define	I00-I99	Diseases of the circulatory system	1275456	Visuospatial deficit and spatial neglect following other cerebrovascular disease	ICD10CM	1	I69.812		Visuospatial deficit and spatial neglect following other ce	IMO	GHS-PACDC	IMO_2017_R	ACTIVE	Y	2016-09-17	9999-12-31	2016-09-30
Diagnosis: ICD10 Code, I00-I99, Define	I00-I99	Diseases of the circulatory system	1275486	Psychomotor deficit following other cerebrovascular disease	ICD10CM	1	I69.813		Psychomotor deficit following other cerebrovascular dis	IMO	GHS-PACDC	IMO_2017_R	ACTIVE	Y	1900-01-01	9999-12-31	2017-04-14
Diagnosis: ICD10 Code, I00-I99, Define	I00-I99	Diseases of the circulatory system	1178173	Cognitive deficits following other cerebrovascular disease	ICD10CM	1	I69.819		Unspecified symptoms and signs involving cognitive func	IMO	GHS-PACDC	IMO_2016_R	DELETED	Y	2016-09-17	9999-12-31	2016-09-30
Diagnosis: ICD10 Code, I00-I99, Define	I00-I99	Diseases of the circulatory system	641615	Oth speech/lang deficits following oth cerebvasc disease	ICD10CM	1	I69.828		Other speech and language deficits following other cere	IMO	GHS-PACDC	IMO_2015_R	DELETED	Y	1900-01-01	9999-12-31	2015-07-10
Diagnosis: ICD10 Code, I00-I99, Define	I00-I99	Diseases of the circulatory system	641997	Monoplg low lmb fol oth cerebvasc dis aff right nondom side	ICD10CM	1	I69.843		Monoplegia of lower limb following other cerebrovascul	IMO	GHS-PACDC	IMO_2015_R	DELETED	Y	1900-01-01	9999-12-31	2015-07-10
Diagnosis: ICD10 Code, I00-I99, Define	I00-I99	Diseases of the circulatory system	641724	Monoplg low lmb fol oth cerebvasc dis aff left nondom side	ICD10CM	1	I69.844		Monoplegia of lower limb following other cerebrovascul	IMO	GHS-PACDC	IMO_2015_R	DELETED	Y	1900-01-01	9999-12-31	2015-07-10
Diagnosis: ICD10 Code, I00-I99, Define	I00-I99	Diseases of the circulatory system	1321261	Spastic hemiparesis of right dominant side due to other cerebrovascular disease (HCC)	ICD10CM	1	I69.851		Hemiplegia and hemiparesis following other cerebrovas	IMO	GHS-PACDC	IMO_2017_R	ACTIVE	N	1900-01-01	2017-10-14	2017-04-14
Diagnosis: ICD10 Code, I00-I99, Define	I00-I99	Diseases of the circulatory system	603015	Hemiplegia and hemiparesis following other cerebrovascular disease affecting left non-dominar	ICD10CM	1	I69.854		Hemiplegia and hemiparesis following other cerebrovas	IMO	GHS-PACDC	IMO_2015_R	ACTIVE	Y	1900-01-01	9999-12-31	2015-07-10
Diagnosis: ICD10 Code, I00-I99, Define	I00-I99	Diseases of the circulatory system	1329516	Spastic hemiplegia of left nondominant side due to other cerebrovascular disease (HCC)	ICD10CM	1	I69.854		Hemiplegia and hemiparesis following other cerebrovas	IMO	GHS-PACDC	IMO_2017_R	DELETED	Y	1900-01-01	9999-12-31	2017-04-14
Diagnosis: ICD10 Code, I00-I99, Define	I00-I99	Diseases of the circulatory system	375431	Hemiparesis affecting nondominant side as late effect of stroke (HCC)	ICD10CM	1	I69.859		Hemiplegia and hemiparesis following other cerebrovas	IMO	GHS-PACDC	DX_LEGACY	ACTIVE	N	1900-01-01	2016-05-07	2015-07-10
Diagnosis: ICD10 Code, I00-I99, Define	I00-I99	Diseases of the circulatory system	1178289	Other paralytic syndrome following other cerebrovascular disease affecting left non-dominant s	ICD10CM	1	I69.864		Other paralytic syndrome following other cerebrovascul	IMO	GHS-PACDC	IMO_2016_R	ACTIVE	N	1900-01-01	9999-12-31	2016-06-02
Diagnosis: ICD10 Code, I00-I99, Define	I00-I99	Diseases of the circulatory system	1180150	Other paralytic syndrome following other cerebrovascular disease, bilateral (HCC)	ICD10CM	1	I69.865		Other paralytic syndrome following other cerebrovascul	IMO	GHS-PACDC	IMO_2016_R	ACTIVE	N	1900-01-01	9999-12-31	2016-06-02
Diagnosis: ICD10 Code, I00-I99, Define	I00-I99	Diseases of the circulatory system	230094	Alterations of sensations following CVA (cerebrovascular accident)	ICD10CM	1	I69.898	I69.898,R20.8	Other sequelae of other cerebrovascular disease	IMO	GHS-PACDC	DX_LEGACY	ACTIVE	N	1900-01-01	2016-05-07	2015-07-10
Diagnosis: ICD10 Code, I00-I99, Define	I00-I99	Diseases of the circulatory system	142702	Cognitive deficits, late effect of cerebrovascular disease	ICD10CM	1	I69.91		Cognitive deficits following unspecified cerebrovascular	IMO	GHS-PACDC	DX_LEGACY	ACTIVE	Y	1900-01-01	2016-10-14	2015-07-10
Diagnosis: ICD10 Code, I00-I99, Define	I00-I99	Diseases of the circulatory system	595201	Cognitive deficits following unspecified cerebrovascular disease	ICD10CM	1	I69.91		Cognitive deficits following unspecified cerebrovascular	IMO	GHS-PACDC	IMO_2015_R	ACTIVE	Y	1900-01-01	9999-12-31	2015-07-10
Diagnosis: ICD10 Code, I00-I99, Define	I00-I99	Diseases of the circulatory system	1275381	Attention and concentration deficit following unspecified cerebrovascular disease	ICD10CM	1	I69.910		Attention and concentration deficit following unspecifie	IMO	GHS-PACDC	IMO_2017_R	ACTIVE	Y	2016-09-17	9999-12-31	2016-09-30
Diagnosis: ICD10 Code, I00-I99, Define	I00-I99	Diseases of the circulatory system	1275451	Visuospatial deficit and spatial neglect following unspecified cerebrovascular disease	ICD10CM	1	I69.912		Visuospatial deficit and spatial neglect following unspeci	IMO	GHS-PACDC	IMO_2017_R	ACTIVE	Y	1900-01-01	9999-12-31	2017-04-14
Diagnosis: ICD10 Code, I00-I99, Define	I00-I99	Diseases of the circulatory system	1275451	Visuospatial deficit and spatial neglect following unspecified cerebrovascular disease	ICD10CM	1	I69.912		Visuospatial deficit and spatial neglect following unspeci	IMO	GHS-PACDC	IMO_2017_R	ACTIVE	Y	2016-09-17	9999-12-31	2016-09-30
Diagnosis: ICD10 Code, I00-I99, Define	I00-I99	Diseases of the circulatory system	1275638	Unspecified symptoms and signs involving cognitive functions following unspecified cerebrovas	ICD10CM	1	I69.919		Unspecified symptoms and signs involving cognitive func	IMO	GHS-PACDC	IMO_2017_R	ACTIVE	Y	1900-01-01	9999-12-31	2017-04-14
Diagnosis: ICD10 Code, I00-I99, Define	I00-I99	Diseases of the circulatory system	213412	Fluency disorder as late effect of cerebrovascular disease	ICD10CM	1	I69.923		Fluency disorder following unspecified cerebrovascular (	IMO	GHS-PACDC	DX_LEGACY	ACTIVE	N	1900-01-01	9999-12-31	2015-07-10
Diagnosis: ICD10 Code, I00-I99, Define	I00-I99	Diseases of the circulatory system	213869	Stuttering as late effect of cerebrovascular disease	ICD10CM	1	I69.923		Fluency disorder following unspecified cerebrovascular (	IMO	GHS-PACDC	DX_LEGACY	ACTIVE	N	1900-01-01	9999-12-31	2015-07-10
Diagnosis: ICD10 Code, I00-I99, Define	I00-I99	Diseases of the circulatory system	1426972	Monoplegia of upper extremity following cerebrovascular disease affecting right non-dominant	ICD10CM	1	I69.933		Monoplegia of upper limb following unspecified cerebro	IMO	GHS-PACDC	IMO_2018_R	ACTIVE	N	1900-01-01	9999-12-31	2017-10-30
Diagnosis: ICD10 Code, I00-I99, Define	I00-I99	Diseases of the circulatory system	595363	Monoplegia of upper limb following unspecified cerebrovascular disease affecting left non-dom	ICD10CM	1	I69.934		Monoplegia of upper limb following unspecified cerebro	IMO	GHS-PACDC	IMO_2015_R	ACTIVE	Y	1900-01-01	9999-12-31	2015-07-10
Diagnosis: ICD10 Code, I00-I99, Define	I00-I99	Diseases of the circulatory system	343257	Monoplegia of lower limb dominant side status post cerebrovascular accident (HCC)	ICD10CM	1	I69.949		Monoplegia of lower limb following unspecified cerebro	IMO	GHS-PACDC	DX_LEGACY	ACTIVE	N	1900-01-01	2016-05-07	2015-07-10
Diagnosis: ICD10 Code, I00-I99, Define	I00-I99	Diseases of the circulatory system	523012	Monoplegia of lower extremity affecing dominant side following cerebrovascular accident (HCC	ICD10CM	1	I69.949		Monoplegia of lower limb following unspecified cerebro	IMO	GHS-PACDC	IMO_2015_R	ACTIVE	N	1900-01-01	2016-05-07	2015-07-10
Diagnosis: ICD10 Code, I00-I99, Define	I00-I99	Diseases of the circulatory system	374080	Monoplegia leg affect dominant side, late effect cerebrovasc disease (HCC)	ICD10CM	1	I69.949		Monoplegia of lower limb following unspecified cerebro	IMO	GHS-PACDC	DX_LEGACY	ACTIVE	N	1900-01-01	9999-12-31	2015-07-10
Diagnosis: ICD10 Code, I00-I99, Define	I00-I99	Diseases of the circulatory system	1184335	Hemiplegia and hemiparesis following unspecified cerebrovascular disease affecting right domi	ICD10CM	1	I69.951		Hemiplegia and hemiparesis following unspecified cereb	IMO	GHS-PACDC	IMO_2016_R	ACTIVE	N	1900-01-01	9999-12-31	2016-06-02
Diagnosis: ICD10 Code, I00-I99, Define	I00-I99	Diseases of the circulatory system	1330109	Hemiplegia of left dominant side due to cerebrovascular disease, unspecified cerebrovascular d	ICD10CM	1	I69.952		Hemiplegia and hemiparesis following unspecified cereb	IMO	GHS-PACDC	IMO_2017_R	ACTIVE	N	1900-01-01	9999-12-31	2017-04-14
Diagnosis: ICD10 Code, I00-I99, Define	I00-I99	Diseases of the circulatory system	93729	Hemiplegia affecting dominant side, late effect of cerebrovascular disease	ICD10CM	1	I69.959		Hemiplegia and hemiparesis following unspecified cereb	IMO	GHS-PACDC	DX_LEGACY	ACTIVE	Y	2016-04-10	9999-12-31	2015-07-10
Diagnosis: ICD10 Code, I00-I99, Define	I00-I99	Diseases of the circulatory system	357027	Hemiplegia of nondominant side as late effect of cerebrovascular disease (HCC)	ICD10CM	1	I69.959		Hemiplegia and hemiparesis following unspecified cereb	IMO	GHS-PACDC	DX_LEGACY	ACTIVE	N	1900-01-01	9999-12-31	2015-07-10
Diagnosis: ICD10 Code, I00-I99, Define	I00-I99	Diseases of the circulatory system	595034	Other paralytic syndrome following unspecified cerebrovascular disease affecting left dominant	ICD10CM	1	I69.962		Other paralytic syndrome following unspecified cerebro	IMO	GHS-PACDC	IMO_2015_R	ACTIVE	Y	1900-01-01	9999-12-31	2015-07-10
Diagnosis: ICD10 Code, I00-I99, Define	I00-I99	Diseases of the circulatory system	425440	Paralytic syndrome of nondominant side as late effect of stroke (HCC)	ICD10CM	1	I69.964		Other paralytic syndrome following unspecified cerebro	IMO	GHS-PACDC	IMO_2015_R	ACTIVE	N	1900-01-01	2016-05-07	2015-07-10
Diagnosis: ICD10 Code, I00-I99, Define	I00-I99	Diseases of the circulatory system	223633	Paralytic syndrome, late effect of cerebrovascular disease (HCC)	ICD10CM	1	I69.969		Other paralytic syndrome following unspecified cerebro	IMO	GHS-PACDC	DX_LEGACY	ACTIVE	N	1900-01-01	9999-12-31	2015-07-10
Diagnosis: ICD10 Code, I00-I99, Define	I00-I99	Diseases of the circulatory system	383233	Facial weakness due to recent cerebrovascular accident	ICD10CM	1	I69.992		Facial weakness following unspecified cerebrovascular d	IMO	GHS-PACDC	DX_LEGACY	ACTIVE	N	1900-01-01	2016-05-07	2015-07-10
Diagnosis: ICD10 Code, I00-I99, Define	I00-I99	Diseases of the circulatory system	136093	Facial weakness due to cerebrovascular disease	ICD10CM	1	I69.992		Facial weakness following unspecified cerebrovascular d	IMO	GHS-PACDC	DX_LEGACY	ACTIVE	Y	1900-01-01	2017-04-27	2015-07-10
Diagnosis: ICD10 Code, I00-I99, Define	I00-I99	Diseases of the circulatory system	598711	Ataxia following unspecified cerebrovascular disease	ICD10CM	1	I69.993		Ataxia following unspecified cerebrovascular disease	IMO	GHS-PACDC	IMO_2015_R	ACTIVE	Y	1900-01-01	9999-12-31	2015-07-10
Diagnosis: ICD10 Code, I00-I99, Define	I00-I99	Diseases of the circulatory system	156285	Disturbances of vision, late effect of cerebrovascular disease	ICD10CM	1	I69.998	I69.998,H53.9	Other sequelae following unspecified cerebrovascular di	IMO	GHS-PACDC	DX_LEGACY	ACTIVE	N	1900-01-01	9999-12-31	2015-07-10
Diagnosis: ICD10 Code, I00-I99, Define	I00-I99	Diseases of the circulatory system	384462	Neurobehavioral disorder following cerebrovascular accident	ICD10CM	1	I69.998	I69.998,F07.0	Other sequelae following unspecified cerebrovascular di	IMO	GHS-PACDC	DX_LEGACY	ACTIVE	N	1900-01-01	2016-05-07	2015-07-10
Diagnosis: ICD10 Code, I00-I99, Define	I00-I99	Diseases of the circulatory system	255889	Visual disturbance due to old intracerebral hemorrhage	ICD10CM	1	I69.998	I69.998,H53.9	Other sequelae following unspecified cerebrovascular di	IMO	GHS-PACDC	DX_LEGACY	ACTIVE	N	1900-01-01	2016-05-07	2015-07-10
Diagnosis: ICD10 Code, I00-I99, Define	I00-I99	Diseases of the circulatory system	183183	Abdominal aortic atherosclerosis (HCC)	ICD10CM	1	I70.0		Atherosclerosis of aorta	IMO	GHS-PACDC	DX_LEGACY	ACTIVE	N	1900-01-01	9999-12-31	2015-07-10
Diagnosis: ICD10 Code, I00-I99, Define	I00-I99	Diseases of the circulatory system	228934	Aortic arch atherosclerosis (HCC)	ICD10CM	1	I70.0		Atherosclerosis of aorta	IMO	GHS-PACDC	DX_LEGACY	ACTIVE	N	1900-01-01	9999-12-31	2015-07-10
Diagnosis: ICD10 Code, I00-I99, Define	I00-I99	Diseases of the circulatory system	175620	Bilateral atherosclerotic renal artery stenosis (HCC)	ICD10CM	1	I70.1		Atherosclerosis of renal artery	IMO	GHS-PACDC	DX_LEGACY	ACTIVE	N	1900-01-01	9999-12-31	2015-07-10
Diagnosis: ICD10 Code, I00-I99, Define	I00-I99	Diseases of the circulatory system	232031	Non-flow-limiting renal artery stenosis (HCC)	ICD10CM	1	I70.1		Atherosclerosis of renal artery	IMO	GHS-PACDC	DX_LEGACY	ACTIVE	N	1900-01-01	9999-12-31	2015-07-10
Diagnosis: ICD10 Code, I00-I99, Define	I00-I99	Diseases of the circulatory system	48631	Goldblatt hypertension (HCC)	ICD10CM	1	I70.1		Atherosclerosis of renal artery	IMO	GHS-PACDC	DX_LEGACY	ACTIVE	N	1900-01-01	9999-12-31	2015-07-10

Diagnosis: ICD10 Code, I00-I99, Define	I00-I99	Diseases of the circulatory system	232521	Native stenosis of both renal arteries (HCC)	ICD10CM	1	I70.1		Atherosclerosis of renal artery	IMO	GHS-PACDC	DX_LEGACY	ACTIVE	N	1900-01-01	9999-12-31	2015-07-10
Diagnosis: ICD10 Code, I00-I99, Define	I00-I99	Diseases of the circulatory system	69654	Renal artery arteriosclerosis (HCC)	ICD10CM	1	I70.1		Atherosclerosis of renal artery	IMO	GHS-PACDC	DX_LEGACY	ACTIVE	N	1900-01-01	9999-12-31	2015-07-10
Diagnosis: ICD10 Code, I00-I99, Define	I00-I99	Diseases of the circulatory system	229801	Peroneal artery stenosis, right (HCC)	ICD10CM	1	I70.201		Unspecified atherosclerosis of native arteries of extremi	IMO	GHS-PACDC	DX_LEGACY	ACTIVE	N	1900-01-01	9999-12-31	2015-07-10
Diagnosis: ICD10 Code, I00-I99, Define	I00-I99	Diseases of the circulatory system	230692	Femoral artery stenosis, left (HCC)	ICD10CM	1	I70.202		Unspecified atherosclerosis of native arteries of extremi	IMO	GHS-PACDC	DX_LEGACY	ACTIVE	N	1900-01-01	9999-12-31	2015-07-10
Diagnosis: ICD10 Code, I00-I99, Define	I00-I99	Diseases of the circulatory system	237647	Stenosis of left popliteal-tibial artery (HCC)	ICD10CM	1	I70.202		Unspecified atherosclerosis of native arteries of extremi	IMO	GHS-PACDC	DX_LEGACY	ACTIVE	N	1900-01-01	9999-12-31	2015-07-10
Diagnosis: ICD10 Code, I00-I99, Define	I00-I99	Diseases of the circulatory system	229719	Bilateral peroneal artery stenosis (HCC)	ICD10CM	1	I70.203		Unspecified atherosclerosis of native arteries of extremi	IMO	GHS-PACDC	DX_LEGACY	ACTIVE	N	1900-01-01	9999-12-31	2015-07-10
Diagnosis: ICD10 Code, I00-I99, Define	I00-I99	Diseases of the circulatory system	522482	Stenosis of artery of upper extremity (HCC)	ICD10CM	1	I70.208		Unspecified atherosclerosis of native arteries of extremi	IMO	GHS-PACDC	IMO_2015_R	ACTIVE	N	1900-01-01	9999-12-31	2015-07-10
Diagnosis: ICD10 Code, I00-I99, Define	I00-I99	Diseases of the circulatory system	229361	Femoral artery stenosis (HCC)	ICD10CM	1	I70.208		Unspecified atherosclerosis of native arteries of extremi	IMO	GHS-PACDC	DX_LEGACY	ACTIVE	N	1900-01-01	2016-05-07	2015-07-10
Diagnosis: ICD10 Code, I00-I99, Define	I00-I99	Diseases of the circulatory system	17442	Chronic total occlusion of artery of extremity (HCC)	ICD10CM	1	I70.209	I70.209,I70.92	Unspecified atherosclerosis of native arteries of extremi	IMO	GHS-PACDC	DX_LEGACY	ACTIVE	N	2016-04-10	2016-10-14	2015-07-10
Diagnosis: ICD10 Code, I00-I99, Define	I00-I99	Diseases of the circulatory system	301901	Diabetes type 1 with atherosclerosis of arteries of extremities (HCC)	ICD10CM	2	I70.209	E10.59,I70.209	Unspecified atherosclerosis of native arteries of extremi	IMO	GHS-PACDC	DX_LEGACY	ACTIVE	N	1900-01-01	2017-10-14	2015-07-10
Diagnosis: ICD10 Code, I00-I99, Define	I00-I99	Diseases of the circulatory system	301914	Uncontrolled diabetes type II with extremity artery atherosclerosis (HCC)	ICD10CM	2	I70.209	E11.59,I70.209	Unspecified atherosclerosis of native arteries of extremi	IMO	GHS-PACDC	DX_LEGACY	ACTIVE	N	1900-01-01	2016-05-07	2015-07-10
Diagnosis: ICD10 Code, I00-I99, Define	I00-I99	Diseases of the circulatory system	357198	Diabetes mellitus type 2 with atherosclerosis of arteries of extremities (HCC)	ICD10CM	2	I70.209	E11.59,I70.209	Unspecified atherosclerosis of native arteries of extremi	IMO	GHS-PACDC	DX_LEGACY	ACTIVE	N	1900-01-01	2017-10-14	2015-07-10
Diagnosis: ICD10 Code, I00-I99, Define	I00-I99	Diseases of the circulatory system	357200	Uncontrolled diabetes mellitus type 2 with atherosclerosis of arteries of extremities (HCC)	ICD10CM	2	I70.209	E11.51,I70.209,E11.65	Unspecified atherosclerosis of native arteries of extremi	IMO	GHS-PACDC	DX_LEGACY	ACTIVE	N	2017-09-17	9999-12-31	2017-10-30
Diagnosis: ICD10 Code, I00-I99, Define	I00-I99	Diseases of the circulatory system	328362	Atherosclerotic PVD with intermittent claudication (HCC)	ICD10CM	1	I70.219		Atherosclerosis of native arteries of extremities with int	IMO	GHS-PACDC	DX_LEGACY	ACTIVE	N	2017-09-17	9999-12-31	2017-10-30
Diagnosis: ICD10 Code, I00-I99, Define	I00-I99	Diseases of the circulatory system	1098642	Atheroscler-limb&claudic	ICD10CM	1	I70.219		Atherosclerosis of native arteries of extremities with int	IMO	GHS-PACDC	IMO_2016_R	DELETED	Y	1900-01-01	9999-12-31	2016-06-02
Diagnosis: ICD10 Code, I00-I99, Define	I00-I99	Diseases of the circulatory system	603843	Atherosclerosis of native arteries of extremities with intermittent claudication, unspecified extr	ICD10CM	1	I70.219		Atherosclerosis of native arteries of extremities with int	IMO	GHS-PACDC	IMO_2015_R	ACTIVE	Y	1900-01-01	9999-12-31	2015-07-10
Diagnosis: ICD10 Code, I00-I99, Define	I00-I99	Diseases of the circulatory system	503064	Atherosclerosis of lower extremity with intermittent claudication (HCC)	ICD10CM	1	I70.219		Atherosclerosis of native arteries of extremities with int	IMO	GHS-PACDC	IMO_2015_R	ACTIVE	N	2016-04-10	9999-12-31	2015-07-10
Diagnosis: ICD10 Code, I00-I99, Define	I00-I99	Diseases of the circulatory system	1209435	Atherosclerosis of other extremity with rest pain (HCC)	ICD10CM	1	I70.228		Atherosclerosis of native arteries of extremities with res	IMO	GHS-PACDC	IMO_2016_R	DELETED	Y	1900-01-01	9999-12-31	2016-06-02
Diagnosis: ICD10 Code, I00-I99, Define	I00-I99	Diseases of the circulatory system	212421	Extremity atherosclerosis with resting pain (HCC)	ICD10CM	1	I70.229		Atherosclerosis of native arteries of extremities with res	IMO	GHS-PACDC	DX_LEGACY	ACTIVE	N	1900-01-01	9999-12-31	2015-07-10
Diagnosis: ICD10 Code, I00-I99, Define	I00-I99	Diseases of the circulatory system	623886	Atherosclerosis of native arteries of right leg with ulceration	ICD10CM	1	I70.23		Atherosclerosis of native arteries of right leg with ulcera	IMO	GHS-PACDC	IMO_2015_R	ACTIVE	Y	1900-01-01	9999-12-31	2015-07-10
Diagnosis: ICD10 Code, I00-I99, Define	I00-I99	Diseases of the circulatory system	1109864	Atherosclerosis of right lower extremity with ulceration of ankle (HCC)	ICD10CM	1	I70.233		Atherosclerosis of native arteries of right leg with ulcera	IMO	GHS-PACDC	IMO_2016_R	ACTIVE	N	1900-01-01	9999-12-31	2016-06-02
Diagnosis: ICD10 Code, I00-I99, Define	I00-I99	Diseases of the circulatory system	1110256	Atherosclerosis of native artery of both lower extremities with bilateral ulceration of ankles (HC	ICD10CM	1	I70.233	I70.233,I70.243	Atherosclerosis of native arteries of right leg with ulcera	IMO	GHS-PACDC	IMO_2016_R	ACTIVE	N	1900-01-01	9999-12-31	2016-06-02
Diagnosis: ICD10 Code, I00-I99, Define	I00-I99	Diseases of the circulatory system	1366385	Athscl native arteries of right leg w ulceration of ankle (HCC)	ICD10CM	1	I70.233		Atherosclerosis of native arteries of right leg with ulcera	IMO	GHS-PACDC	IMO_2018_R	ACTIVE	N	1900-01-01	9999-12-31	2017-10-30
Diagnosis: ICD10 Code, I00-I99, Define	I00-I99	Diseases of the circulatory system	1110280	Atherosclerosis of both lower extremities with bilateral ulceration of heels (HCC)	ICD10CM	1	I70.234	I70.234,I70.244	Atherosclerosis of native arteries of right leg with ulcera	IMO	GHS-PACDC	IMO_2016_R	ACTIVE	N	1900-01-01	9999-12-31	2016-06-02
Diagnosis: ICD10 Code, I00-I99, Define	I00-I99	Diseases of the circulatory system	1209490	Atherosclerosis of native artery of both lower extremities with bilateral ulceration of other part	ICD10CM	1	I70.238	I70.238,I70.248	Atherosclerosis of native arteries of right leg with ulcera	IMO	GHS-PACDC	IMO_2016_R	DELETED	Y	1900-01-01	9999-12-31	2016-06-02
Diagnosis: ICD10 Code, I00-I99, Define	I00-I99	Diseases of the circulatory system	1109895	Atherosclerosis of left lower extremity with ulceration of ankle (HCC)	ICD10CM	1	I70.243		Atherosclerosis of native arteries of left leg with ulcerati	IMO	GHS-PACDC	IMO_2016_R	ACTIVE	N	1900-01-01	9999-12-31	2016-06-02
Diagnosis: ICD10 Code, I00-I99, Define	I00-I99	Diseases of the circulatory system	1109875	Atherosclerosis of native artery of left lower extremity with ulceration of midfoot (HCC)	ICD10CM	1	I70.244		Atherosclerosis of native arteries of left leg with ulcerati	IMO	GHS-PACDC	IMO_2016_R	ACTIVE	N	1900-01-01	9999-12-31	2016-06-02
Diagnosis: ICD10 Code, I00-I99, Define	I00-I99	Diseases of the circulatory system	1109886	Atherosclerosis of left lower extremity with ulceration of midfoot (HCC)	ICD10CM	1	I70.244		Atherosclerosis of native arteries of left leg with ulcerati	IMO	GHS-PACDC	IMO_2016_R	ACTIVE	N	1900-01-01	9999-12-31	2016-06-02
Diagnosis: ICD10 Code, I00-I99, Define	I00-I99	Diseases of the circulatory system	599148	Atherosclerosis of native arteries of left leg with ulceration of other part of foot	ICD10CM	1	I70.245		Atherosclerosis of native arteries of left leg with ulcerati	IMO	GHS-PACDC	IMO_2015_R	ACTIVE	Y	1900-01-01	9999-12-31	2015-07-10
Diagnosis: ICD10 Code, I00-I99, Define	I00-I99	Diseases of the circulatory system	641781	Athscl native art of left leg w ulcer oth prt lower left leg	ICD10CM	1	I70.248		Atherosclerosis of native arteries of left leg with ulcerati	IMO	GHS-PACDC	IMO_2015_R	DELETED	Y	1900-01-01	9999-12-31	2015-07-10
Diagnosis: ICD10 Code, I00-I99, Define	I00-I99	Diseases of the circulatory system	1109889	Atherosclerosis of both lower extremities with bilateral ulceration (HCC)	ICD10CM	2	I70.249	I70.239,I70.249	Atherosclerosis of native arteries of left leg with ulcerati	IMO	GHS-PACDC	IMO_2016_R	ACTIVE	N	1900-01-01	9999-12-31	2016-06-02
Diagnosis: ICD10 Code, I00-I99, Define	I00-I99	Diseases of the circulatory system	1109844	Atherosclerosis of native artery of lower extremity with ulceration (HCC)	ICD10CM	1	I70.25		Atherosclerosis of native arteries of other extremities wi	IMO	GHS-PACDC	IMO_2016_R	ACTIVE	N	1900-01-01	9999-12-31	2016-06-02
Diagnosis: ICD10 Code, I00-I99, Define	I00-I99	Diseases of the circulatory system	421945	Atherosclerosis of native arteries of the extremities with ulceration (HCC)	ICD10CM	1	I70.25		Atherosclerosis of native arteries of other extremities wi	IMO	GHS-PACDC	IMO_2015_R	ACTIVE	N	2016-04-10	9999-12-31	2015-07-10
Diagnosis: ICD10 Code, I00-I99, Define	I00-I99	Diseases of the circulatory system	357262	Atherosclerosis of native artery of lower extremity with ulceration of heel (HCC)	ICD10CM	1	I70.25	I70.25,L97.409	Atherosclerosis of native arteries of other extremities wi	IMO	GHS-PACDC	DX_LEGACY	ACTIVE	N	1900-01-01	2016-05-07	2015-07-10
Diagnosis: ICD10 Code, I00-I99, Define	I00-I99	Diseases of the circulatory system	1209415	Atherosclerosis of native artery of lower extremity with other clinical manifestation (HCC)	ICD10CM	1	I70.299		Other atherosclerosis of native arteries of extremities, u	IMO	GHS-PACDC	IMO_2016_R	DELETED	Y	1900-01-01	9999-12-31	2016-06-02
Diagnosis: ICD10 Code, I00-I99, Define	I00-I99	Diseases of the circulatory system	596497	Unspecified atherosclerosis of unspecified type of bypass graft(s) of the extremities, left leg	ICD10CM	1	I70.302		Unspecified atherosclerosis of unspecified type of bypas	IMO	GHS-PACDC	IMO_2015_R	ACTIVE	Y	1900-01-01	9999-12-31	2015-07-10
Diagnosis: ICD10 Code, I00-I99, Define	I00-I99	Diseases of the circulatory system	1244324	Atherosclerosis of bypass graft of left lower extremity, with unspecified presence of clinical mar	ICD10CM	1	I70.302		Unspecified atherosclerosis of unspecified type of bypas	IMO	GHS-PACDC	IMO_2016_R	ACTIVE	N	1900-01-01	9999-12-31	2016-06-02
Diagnosis: ICD10 Code, I00-I99, Define	I00-I99	Diseases of the circulatory system	1098110	Atherosclerosis of bypass graft of the extremities	ICD10CM	1	I70.309		Unspecified atherosclerosis of unspecified type of bypas	IMO	GHS-PACDC	IMO_2016_R	DELETED	Y	1900-01-01	9999-12-31	2016-06-02
Diagnosis: ICD10 Code, I00-I99, Define	I00-I99	Diseases of the circulatory system	623897	Atherosclerosis of unspecified type of bypass graft(s) of the extremities with intermittent claudi	ICD10CM	1	I70.31		Atherosclerosis of unspecified type of bypass graft(s) of	IMO	GHS-PACDC	IMO_2015_R	ACTIVE	Y	1900-01-01	9999-12-31	2015-07-10
Diagnosis: ICD10 Code, I00-I99, Define	I00-I99	Diseases of the circulatory system	374451	Atheroscler of bypass graft of right leg with intermit claudication (HCC)	ICD10CM	1	I70.311		Atherosclerosis of unspecified type of bypass graft(s) of	IMO	GHS-PACDC	DX_LEGACY	ACTIVE	N	1900-01-01	9999-12-31	2015-07-10
Diagnosis: ICD10 Code, I00-I99, Define	I00-I99	Diseases of the circulatory system	348689	Atherosclerosis of bypass graft of right lower extremity with intermittent claudication (HCC)	ICD10CM	1	I70.311		Atherosclerosis of unspecified type of bypass graft(s) of	IMO	GHS-PACDC	DX_LEGACY	ACTIVE	N	1900-01-01	9999-12-31	2015-07-10
Diagnosis: ICD10 Code, I00-I99, Define	I00-I99	Diseases of the circulatory system	600284	Atherosclerosis of unspecified type of bypass graft(s) of the extremities with intermittent claudi	ICD10CM	1	I70.312		Atherosclerosis of unspecified type of bypass graft(s) of	IMO	GHS-PACDC	IMO_2015_R	ACTIVE	Y	1900-01-01	9999-12-31	2015-07-10
Diagnosis: ICD10 Code, I00-I99, Define	I00-I99	Diseases of the circulatory system	1241548	Atherosclerosis of bypass graft of extremity with intermittent claudication, unspecified extremit	ICD10CM	1	I70.319		Atherosclerosis of unspecified type of bypass graft(s) of	IMO	GHS-PACDC	IMO_2016_R	ACTIVE	N	1900-01-01	9999-12-31	2016-06-02
Diagnosis: ICD10 Code, I00-I99, Define	I00-I99	Diseases of the circulatory system	1367036	Athscl unsp type bypass of the extrm w rest pain, bi legs (HCC)	ICD10CM	1	I70.323		Atherosclerosis of unspecified type of bypass graft(s) of	IMO	GHS-PACDC	IMO_2018_R	ACTIVE	N	1900-01-01	9999-12-31	2017-10-30
Diagnosis: ICD10 Code, I00-I99, Define	I00-I99	Diseases of the circulatory system	1241190	Atherosclerosis of bypass graft of extremity with rest pain, unspecified extremity (HCC)	ICD10CM	1	I70.329		Atherosclerosis of unspecified type of bypass graft(s) of	IMO	GHS-PACDC	IMO_2016_R	ACTIVE	N	1900-01-01	9999-12-31	2016-06-02
Diagnosis: ICD10 Code, I00-I99, Define	I00-I99	Diseases of the circulatory system	368467	Atherosclerosis of bypass graft of right leg with ulceration of calf (HCC)	ICD10CM	1	I70.332		Atherosclerosis of unspecified type of bypass graft(s) of	IMO	GHS-PACDC	DX_LEGACY	ACTIVE	N	1900-01-01	9999-12-31	2015-07-10
Diagnosis: ICD10 Code, I00-I99, Define	I00-I99	Diseases of the circulatory system	1185981	Atherosclerosis of unspecified type of bypass graft(s) of the right leg with ulceration of ankle (H	ICD10CM	1	I70.333		Atherosclerosis of unspecified type of bypass graft(s) of	IMO	GHS-PACDC	IMO_2016_R	ACTIVE	N	1900-01-01	9999-12-31	2016-06-02
Diagnosis: ICD10 Code, I00-I99, Define	I00-I99	Diseases of the circulatory system	595347	Atherosclerosis of unspecified type of bypass graft(s) of the right leg with ulceration of other pa	ICD10CM	1	I70.338		Atherosclerosis of unspecified type of bypass graft(s) of	IMO	GHS-PACDC	IMO_2015_R	ACTIVE	Y	1900-01-01	9999-12-31	2015-07-10
Diagnosis: ICD10 Code, I00-I99, Define	I00-I99	Diseases of the circulatory system	1366577	Athscl unsp type bypass of right leg w ulcer of unsp site (HCC)	ICD10CM	1	I70.339		Atherosclerosis of unspecified type of bypass graft(s) of	IMO	GHS-PACDC	IMO_2018_R	ACTIVE	N	1900-01-01	9999-12-31	2017-10-30
Diagnosis: ICD10 Code, I00-I99, Define	I00-I99	Diseases of the circulatory system	368463	Atherosclerosis of bypass graft of left leg with ulceration of thigh (HCC)	ICD10CM	1	I70.341		Atherosclerosis of unspecified type of bypass graft(s) of	IMO	GHS-PACDC	DX_LEGACY	ACTIVE	N	1900-01-01	9999-12-31	2015-07-10
Diagnosis: ICD10 Code, I00-I99, Define	I00-I99	Diseases of the circulatory system	1186449	Atherosclerosis of unspecified type of bypass graft(s) of the left leg with ulceration of ankle (HC	ICD10CM	1	I70.343		Atherosclerosis of unspecified type of bypass graft(s) of	IMO	GHS-PACDC	IMO_2016_R	ACTIVE	N	1900-01-01	9999-12-31	2016-06-02
Diagnosis: ICD10 Code, I00-I99, Define	I00-I99	Diseases of the circulatory system	1109924	Atherosclerosis of bypass graft of left lower extremity with ulceration of midfoot (HCC)	ICD10CM	1	I70.344		Atherosclerosis of unspecified type of bypass graft(s) of	IMO	GHS-PACDC	IMO_2016_R	ACTIVE	N	1900-01-01	9999-12-31	2016-06-02
Diagnosis: ICD10 Code, I00-I99, Define	I00-I99	Diseases of the circulatory system	1221429	Atherosclerosis of bypass graft of lower extremity with ulceration, unspecified laterality, unspec	ICD10CM	1	I70.35		Atherosclerosis of unspecified type of bypass graft(s) of	IMO	GHS-PACDC	IMO_2016_R	ACTIVE	N	1900-01-01	9999-12-31	2016-06-02
Diagnosis: ICD10 Code, I00-I99, Define	I00-I99	Diseases of the circulatory system	1222235	Atherosclerosis of bypass graft of lower extremity with ulceration of heel, unspecified laterality	ICD10CM	1	I70.35		Atherosclerosis of unspecified type of bypass graft(s) of	IMO	GHS-PACDC	IMO_2016_R	ACTIVE	N	1900-01-01	9999-12-31	2016-06-02
Diagnosis: ICD10 Code, I00-I99, Define	I00-I99	Diseases of the circulatory system	1242032	Atherosclerosis of bypass graft of extremity with gangrene, unspecified extremity (HCC)	ICD10CM	1	I70.369		Atherosclerosis of unspecified type of bypass graft(s) of	IMO	GHS-PACDC	IMO_2016_R	ACTIVE	N	1900-01-01	9999-12-31	2016-06-02
Diagnosis: ICD10 Code, I00-I99, Define	I00-I99	Diseases of the circulatory system	613512	Other atherosclerosis of unspecified type of bypass graft(s) of the extremities	ICD10CM	1	I70.39		Other atherosclerosis of unspecified type of bypass graft	IMO	GHS-PACDC	IMO_2015_R	ACTIVE	Y	1900-01-01	9999-12-31	2015-07-10
Diagnosis: ICD10 Code, I00-I99, Define	I00-I99	Diseases of the circulatory system	1366695	Athscl autol vein bypass of extrm w intrmt claud, right leg (HCC)	ICD10CM	1	I70.411		Atherosclerosis of autologous vein bypass graft(s) of the	IMO	GHS-PACDC	IMO_2018_R	ACTIVE	N	1900-01-01	9999-12-31	2017-10-30
Diagnosis: ICD10 Code, I00-I99, Define	I00-I99	Diseases of the circulatory system	368430	Atheroscler autologous vein bypass graft right leg w/ulceration thigh (HCC)	ICD10CM	1	I70.431		Atherosclerosis of autologous vein bypass graft(s) of the	IMO	GHS-PACDC	DX_LEGACY	ACTIVE	N	2016-04-10	9999-12-31	2015-07-10
Diagnosis: ICD10 Code, I00-I99, Define	I00-I99	Diseases of the circulatory system	600285	Atherosclerosis of autologous vein bypass graft(s) of the right leg with ulceration of calf	ICD10CM	1	I70.432		Atherosclerosis of autologous vein bypass graft(s) of the	IMO	GHS-PACDC	IMO_2015_R	ACTIVE	Y	1900-01-01	9999-12-31	2015-07-10
Diagnosis: ICD10 Code, I00-I99, Define	I00-I99	Diseases of the circulatory system	642159	Athscl autol vein bypass of right leg w ulcer oth prt foot	ICD10CM	1	I70.435		Atherosclerosis of autologous vein bypass graft(s) of the	IMO	GHS-PACDC	IMO_2015_R	DELETED	Y	1900-01-01	9999-12-31	2015-07-10
Diagnosis: ICD10 Code, I00-I99, Define	I00-I99	Diseases of the circulatory system	1365698	Athscl autol vein bypass of right leg w ulcer of unsp site (HCC)	ICD10CM	1	I70.439		Atherosclerosis of autologous vein bypass graft(s) of the	IMO	GHS-PACDC	IMO_2018_R	ACTIVE	N	1900-01-01	9999-12-31	2017-10-30
Diagnosis: ICD10 Code, I00-I99, Define	I00-I99	Diseases of the circulatory system	1110267	Atherosclerosis of autologous vein bypass graft of both lower extremities with bilateral ulcerati	ICD10CM	2	I70.442	I70.432,I70.442	Atherosclerosis of autologous vein bypass graft(s) of the	IMO	GHS-PACDC	IMO_2016_R	ACTIVE	N	1900-01-01	9999-12-31	2016-06-02
Diagnosis: ICD10 Code, I00-I99, Define	I00-I99	Diseases of the circulatory system	641588	Athscl autol vein bypass of left leg w ulc of heel and midft	ICD10CM	1	I70.444		Atherosclerosis of autologous vein bypass graft(s) of the	IMO	GHS-PACDC	IMO_2015_R	DELETED	Y	1900-01-01	9999-12-31	2015-07-10
Diagnosis: ICD10 Code, I00-I99, Define	I00-I99	Diseases of the circulatory system	1183929	Atherosclerosis of autologous vein bypass graft(s) of the left leg with ulceration of other part of	ICD10CM	1	I70.445		Atherosclerosis of autologous vein bypass graft(s) of the	IMO	GHS-PACDC	IMO_2016_R	ACTIVE	N	1900-01-01	9999-12-31	2016-06-02
Diagnosis: ICD10 Code, I00-I99, Define	I00-I99	Diseases of the circulatory system	598870	Atherosclerosis of autologous vein bypass graft(s) of the extremities with gangrene, unspecified	ICD10CM	1	I70.469		Atherosclerosis of autologous vein bypass graft(s) of the	IMO	GHS-PACDC	IMO_2015_R	ACTIVE	Y	1900-01-01	9999-12-31	2015-07-10
Diagnosis: ICD10 Code, I00-I99, Define	I00-I99	Diseases of the circulatory system	1182419	Atherosclerosis of autologous vein bypass graft(s) of the extremities with gangrene, unspecified	ICD10CM	1	I70.469		Atherosclerosis of autologous vein bypass graft(s) of the	IMO	GHS-PACDC	IMO_2016_R	ACTIVE	N	1900-01-01	9999-12-31	2016-06-02
Diagnosis: ICD10 Code, I00-I99, Define	I00-I99	Diseases of the circulatory system	597714	Other atherosclerosis of autologous vein bypass graft(s) of the extremities, bilateral legs	ICD10CM	1	I70.493		Other atherosclerosis of autologous vein bypass graft(s)	IMO	GHS-PACDC	IMO_2015_R	ACTIVE	Y	1900-01-01	9999-12-31	2015-07-10
Diagnosis: ICD10 Code, I00-I99, Define	I00-I99	Diseases of the circulatory system	1243085	Atherosclerosis of nonautologous biological bypass graft of right lower extremity, with unspecif	ICD10CM	1	I70.501		Unspecified atherosclerosis of nonautologous biological	IMO	GHS-PACDC	IMO_2016_R	ACTIVE	N	1900-01-01	9999-12-31	2016-06-02
Diagnosis: ICD10 Code, I00-I99, Define	I00-I99	Diseases of the circulatory system	600309	Atherosclerosis of nonautologous biological bypass graft(s) of the extremities with intermittent	ICD10CM	1	I70.513		Atherosclerosis of nonautologous biological bypass graft	IMO	GHS-PACDC	IMO_2015_R	ACTIVE	Y	1900-01-01	9999-12-31	2015-07-10
Diagnosis: ICD10 Code, I00-I99, Define	I00-I99	Diseases of the circulatory system	599445	Atherosclerosis of nonautologous biological bypass graft(s) of the extremities with rest pain, un	ICD10CM	1	I70.529		Atherosclerosis of nonautologous biological bypass graft	IMO	GHS-PACDC	IMO_2015_R	ACTIVE	Y	1900-01-01	9999-12-31	2015-07-10
Diagnosis: ICD10 Code, I00-I99, Define	I00-I99	Diseases of the circulatory system	1366887	Athscl nonaut bio bypass of extrm w rest pain, unsp extrm (HCC)	ICD10CM	1	I70.529		Atherosclerosis of nonautologous biological bypass graft	IMO	GHS-PACDC	IMO_2018_R	ACTIVE	N	1900-01-01	9999-12-31	2017-10-30
Diagnosis: ICD10 Code, I00-I99, Define	I00-I99	Diseases of the circulatory system	596674	Atherosclerosis of nonautologous biological bypass graft(s) of the right leg with ulceration of cal	ICD10CM	1	I70.532		Atherosclerosis of nonautologous biological bypass graft	IMO	GHS-PACDC	IMO_2015_R	ACTIVE	Y	1900-01-01	9999-12-31	2015-07-10
Diagnosis: ICD10 Code, I00-I99, Define	I00-I99	Diseases of the circulatory system	1180562	Atherosclerosis of nonautologous biological bypass graft(s) of the right leg with ulceration of cal	ICD10CM	1	I70.532		Atherosclerosis of nonautologous biological bypass graft	IMO	GHS-PACDC	IMO_2016_R	ACTIVE	N	1900-01-01	9999-12-31	2016-06-02
Diagnosis: ICD10 Code, I00-I99, Define	I00-I99	Diseases of the circulatory system	1183952	Atherosclerosis of nonautologous biological bypass graft(s) of the right leg with ulceration of he	ICD10CM	1	I70.534		Atherosclerosis of nonautologous biological bypass graft	IMO	GHS-PACDC	IMO_2016_R	ACTIVE	N	1900-01-01	9999-12-31	2016-06-02
Diagnosis: ICD10 Code, I00-I99, Define	I00-I99	Diseases of the circulatory system	347235	Atherosclerosis of nonautologous biological bypass graft of left lower extremity with ulceration	ICD10CM	1	I70.541		Atherosclerosis of nonautologous biological bypass graft	IMO	GHS-PACDC	DX_LEGACY	ACTIVE	N	1900-01-01	9999-12-31	2015-07-10
Diagnosis: ICD10 Code, I00-I99, Define	I00-I99	Diseases of the circulatory system	1110250	Atherosclerosis of nonautologous biological bypass graft of both lower extremities with bilatera	ICD10CM	2	I70.543	I70.533,I70.543	Atherosclerosis of nonautologous biological bypass graft	IMO	GHS-PACDC	IMO_2016_R	ACTIVE	N	1900-01-01	9999-12-31	2016-06-02
Diagnosis: ICD10 Code, I00-I99, Define	I00-I99	Diseases of the circulatory system	1221724	Atherosclerosis of nonautologous biological bypass graft of lower extremity with ulceration of o	ICD10CM	1	I70.55		Atherosclerosis of nonautologous biological bypass graft	IMO	GHS-PACDC	IMO_2016_R	ACTIVE	N	1900-01-01	9999-12-31	2016-06-02
Diagnosis: ICD10 Code, I00-I99, Define	I00-I99	Diseases of the circulatory system	1109834	Atherosclerosis of nonautologous biological bypass graft of lower extremity with ulceration of a	ICD10CM	1	I70.55		Atherosclerosis of nonautologous biological bypass graft	IMO	GHS-PACDC	IMO_2016_R	ACTIVE	N	1900-01-01	9999-12-31	2016-06-02
Diagnosis: ICD10 Code, I00-I99, Define	I00-I99	Diseases of the circulatory system	1109910	Atherosclerosis of nonautologous biological bypass graft of lower extremity with ulceration of n	ICD10CM	1	I70.55		Atherosclerosis of nonautologous biological bypass graft	IMO	GHS-PACDC	IMO_2016_R	ACTIVE	N	1900-01-01	9999-12-31	2016-06-02
Diagnosis: ICD10 Code, I00-I99, Define	I00-I99	Diseases of the circulatory system	612766	Atherosclerosis of nonautologous biological bypass graft(s) of the extremities with gangrene	ICD10CM	1	I70.56		Atherosclerosis of nonautologous biological bypass graft	IMO	GHS-PACDC	IMO_2015_R	ACTIVE	Y	1900-01-01	9999-12-31	2015-07-10
Diagnosis: ICD10 Code, I00-I99, Define	I00-I99	Diseases of the circulatory system	1366955	Athscl nonaut bio bypass of the extrm w gangrene, right leg (HCC)	ICD10CM	1	I70.561		Atherosclerosis of nonautologous biological bypass graft	IMO	GHS-PACDC	IMO_2018_R	ACTIVE	N	1900-01-01	9999-12-31	2017-10-30
Diagnosis: ICD10 Code, I00-I99, Define	I00-I99	Diseases of the circulatory system	352985	Atherosclerosis of nonautologous biological bypass graft of left lower extremity with gangrene (	ICD10CM	1	I70.562		Atherosclerosis of nonautologous biological bypass graft	IMO	GHS-PACDC	DX_LEGACY	ACTIVE	N	1900-01-01	9999-12-31	2015-07-10
Diagnosis: ICD10 Code, I00-I99, Define	I00-I99	Diseases of the circulatory system	601652	Atherosclerosis of nonautologous biological bypass graft(s) of the extremities with gangrene, ot	ICD10CM	1	I70.568		Atherosclerosis of nonautologous biological bypass graft	IMO	GHS-PACDC	IMO_2015_R	ACTIVE	Y	1900-01-01	9999-12-31	2015-07-10
Diagnosis: ICD10 Code, I00-I99, Define	I00-I99	Diseases of the circulatory system	374418	Atheroscler nonautolg biological bypass graft extremity w/gangrene (HCC)	ICD10CM	1	I70.569		Atherosclerosis of nonautologous biological bypass graft	IMO	GHS-PACDC	DX_LEGACY	ACTIVE	N	1900-01-01	9999-12-31	2015-07-10
Diagnosis: ICD10 Code, I00-I99, Define	I00-I99	Diseases of the circulatory system	1185073	Unspecified atherosclerosis of nonbiological bypass graft(s) of the extremities, right leg (HCC)	ICD10CM	1	I70.601		Unspecified atherosclerosis of nonbiological bypass graf	IMO	GHS-PACDC	IMO_2016_R	ACTIVE	N	1900-01-01	9999-12-31	2016-06-02
Diagnosis: ICD10 Code, I00-I99, Define	I00-I99	Diseases of the circulatory system	346869	Atherosclerosis of nonbiological bypass graft of right lower extremity with intermittent claudica	ICD10CM	1	I70.611		Atherosclerosis of nonbiological bypass graft(s) of the ex	IMO	GHS-PACDC	DX_LEGACY	ACTIVE	N	1900-01-01	9999-12-31	2015-07-10
Diagnosis: ICD10 Code, I00-I99, Define	I00-I99	Diseases of the circulatory system	1183806	Atherosclerosis of nonbiological bypass graft(s) of the extremities with intermittent claudicatior	ICD10CM	1	I70.613		Atherosclerosis of nonbiological bypass graft(s) of the ex	IMO	GHS-PACDC	IMO_2016_R	ACTIVE	N	1900-01-01	9999-12-31	2016-06-02
Diagnosis: ICD10 Code, I00-I99, Define	I00-I99	Diseases of the circulatory system	1184823	Atherosclerosis of nonbiological bypass graft(s) of the extremities with intermittent claudicatior	ICD10CM	1	I70.618		Atherosclerosis of nonbiological bypass graft(s) of the ex	IMO	GHS-PACDC	IMO_2016_R	ACTIVE	N	1900-01-01	9999-12-31	2016-06-02
Diagnosis: ICD10 Code, I00-I99, Define	I00-I99	Diseases of the circulatory system	368442	Atheroscler of nonbiologic bypass graft of both legs with rest pain (HCC)	ICD10CM	1	I70.623		Atherosclerosis of nonbiological bypass graft(s) of the ex	IMO	GHS-PACDC	DX_LEGACY	ACTIVE	N	1900-01-01	9999-12-31	2015-07-10
Diagnosis: ICD10 Code, I00-I99, Define	I00-I99	Diseases of the circulatory system	594772	Atherosclerosis of nonbiological bypass graft(s) of the extremities with rest pain, bilateral legs	ICD10CM	1	I70.623		Atherosclerosis of nonbiological bypass graft(s) of the ex	IMO	GHS-PACDC	IMO_2015_R	ACTIVE	Y	1900-01-01	9999-12-31	2015-07-10
Diagnosis: ICD10 Code, I00-I99, Define	I00-I99	Diseases of the circulatory system	1184331	Atherosclerosis of nonbiological bypass graft(s) of the right leg with ulceration of thigh (HCC)	ICD10CM	1	I70.631		Atherosclerosis of nonbiological bypass graft(s) of the rig	IMO	GHS-PACDC	IMO_2016_R	ACTIVE	N	1900-01-01	9999-12-31	2016-06-02
Diagnosis: ICD10 Code, I00-I99, Define	I00-I99	Diseases of the circulatory system	1181167	Atherosclerosis of nonbiological bypass graft(s) of the right leg with ulceration of calf (HCC)	ICD10CM	1	I70.632		Atherosclerosis of nonbiological bypass graft(s) of the rig	IMO	GHS-PACDC	IMO_2016_R	ACTIVE	N	1900-01-01	9999-12-31	2016-06-02
Diagnosis: ICD10 Code, I00-I99, Define	I00-I99	Diseases of the circulatory system	1110238	Atherosclerosis of nonbiological bypass graft of both lower extremities with bilateral ulceration	ICD10CM	2	I70.641	I70.631,I70.641	Atherosclerosis of nonbiological bypass graft(s) of the le	IMO	GHS-PACDC	IMO_2016_R	ACTIVE	N	1900-01-01	9999-12-31	2016-06-02
Diagnosis: ICD10 Code, I00-I99, Define	I00-I99	Diseases of the circulatory system	1180549	Atherosclerosis of nonbiological bypass graft(s) of the left leg with ulceration of unspecified site	ICD10CM	1	I70.649		Atherosclerosis of nonbiological bypass graft(s) of the le	IMO	GHS-PACDC	IMO_2016_R	ACTIVE	N	1900-01-01	9999-12-31	2016-06-02
Diagnosis: ICD10 Code, I00-I99, Define	I00-I99	Diseases of the circulatory system	354473	Atherosclerosis of nonbiological bypass graft of left lower extremity with ulceration (HCC)	ICD10CM	1	I70.649		Atherosclerosis of nonbiological bypass graft(s) of the le	IMO	GHS-PACDC	DX_LEGACY	ACTIVE	N	1900-01-01	9999-12-31	2015-07-10

Diagnosis: ICD10 Code, I00-I99, Define	I00-I99	Diseases of the circulatory system	1179903	Atherosclerosis of nonbiological bypass graft(s) of other extremity with ulceration (HCC)	ICD10CM	1	I70.65		Atherosclerosis of nonbiological bypass graft(s) of other	IMO	GHS-PACDC	IMO_2016_R	ACTIVE	N	1900-01-01	9999-12-31	2016-06-02
Diagnosis: ICD10 Code, I00-I99, Define	I00-I99	Diseases of the circulatory system	1244413	Atherosclerosis of nonbiological bypass graft of extremity with ulceration, unspecified extremit	ICD10CM	1	I70.65		Atherosclerosis of nonbiological bypass graft(s) of other	IMO	GHS-PACDC	IMO_2016_R	ACTIVE	N	1900-01-01	9999-12-31	2016-06-02
Diagnosis: ICD10 Code, I00-I99, Define	I00-I99	Diseases of the circulatory system	1109859	Atherosclerosis of nonbiological bypass graft of lower extremity with ulceration of thigh (HCC)	ICD10CM	1	I70.65		Atherosclerosis of nonbiological bypass graft(s) of other	IMO	GHS-PACDC	IMO_2016_R	ACTIVE	N	1900-01-01	9999-12-31	2016-06-02
Diagnosis: ICD10 Code, I00-I99, Define	I00-I99	Diseases of the circulatory system	623889	Atherosclerosis of nonbiological bypass graft(s) of the extremities with gangrene	ICD10CM	1	I70.66		Atherosclerosis of nonbiological bypass graft(s) of the ex	IMO	GHS-PACDC	IMO_2015_R	ACTIVE	Y	1900-01-01	9999-12-31	2015-07-10
Diagnosis: ICD10 Code, I00-I99, Define	I00-I99	Diseases of the circulatory system	1185843	Atherosclerosis of nonbiological bypass graft(s) of the extremities with gangrene, left leg (HCC)	ICD10CM	1	I70.662		Atherosclerosis of nonbiological bypass graft(s) of the ex	IMO	GHS-PACDC	IMO_2016_R	ACTIVE	N	1900-01-01	9999-12-31	2016-06-02
Diagnosis: ICD10 Code, I00-I99, Define	I00-I99	Diseases of the circulatory system	1209454	Atherosclerosis of nonbiological bypass graft of both lower extremities with other clinical manif	ICD10CM	1	I70.693		Other atherosclerosis of nonbiological bypass graft(s) of	IMO	GHS-PACDC	IMO_2016_R	DELETED	Y	1900-01-01	9999-12-31	2016-06-02
Diagnosis: ICD10 Code, I00-I99, Define	I00-I99	Diseases of the circulatory system	1245200	Atherosclerosis of other type of bypass graft of right lower extremity, with unspecified presence	ICD10CM	1	I70.701		Unspecified atherosclerosis of other type of bypass graft	IMO	GHS-PACDC	IMO_2016_R	ACTIVE	N	1900-01-01	9999-12-31	2016-06-02
Diagnosis: ICD10 Code, I00-I99, Define	I00-I99	Diseases of the circulatory system	642023	Unsp athscl type of bypass of the extremities, left leg	ICD10CM	1	I70.702		Unspecified atherosclerosis of other type of bypass graft	IMO	GHS-PACDC	IMO_2015_R	DELETED	Y	1900-01-01	9999-12-31	2015-07-10
Diagnosis: ICD10 Code, I00-I99, Define	I00-I99	Diseases of the circulatory system	596203	Atherosclerosis of other type of bypass graft(s) of the extremities with intermittent claudication	ICD10CM	1	I70.711		Atherosclerosis of other type of bypass graft(s) of the ex	IMO	GHS-PACDC	IMO_2015_R	ACTIVE	Y	1900-01-01	9999-12-31	2015-07-10
Diagnosis: ICD10 Code, I00-I99, Define	I00-I99	Diseases of the circulatory system	1180410	Atherosclerosis of other type of bypass graft(s) of the extremities with intermittent claudication	ICD10CM	1	I70.712		Atherosclerosis of other type of bypass graft(s) of the ex	IMO	GHS-PACDC	IMO_2016_R	ACTIVE	N	1900-01-01	9999-12-31	2016-06-02
Diagnosis: ICD10 Code, I00-I99, Define	I00-I99	Diseases of the circulatory system	642008	Athscl type of bypass of the extrm w rest pain, left leg	ICD10CM	1	I70.722		Atherosclerosis of other type of bypass graft(s) of the ex	IMO	GHS-PACDC	IMO_2015_R	DELETED	Y	1900-01-01	9999-12-31	2015-07-10
Diagnosis: ICD10 Code, I00-I99, Define	I00-I99	Diseases of the circulatory system	1178794	Atherosclerosis of other type of bypass graft(s) of the extremities with rest pain, unspecified ext	ICD10CM	1	I70.729		Atherosclerosis of other type of bypass graft(s) of the ex	IMO	GHS-PACDC	IMO_2016_R	ACTIVE	N	1900-01-01	9999-12-31	2016-06-02
Diagnosis: ICD10 Code, I00-I99, Define	I00-I99	Diseases of the circulatory system	611731	Atherosclerosis of other type of bypass graft(s) of the left leg with ulceration	ICD10CM	1	I70.74		Atherosclerosis of other type of bypass graft(s) of the lef	IMO	GHS-PACDC	IMO_2015_R	ACTIVE	Y	1900-01-01	9999-12-31	2015-07-10
Diagnosis: ICD10 Code, I00-I99, Define	I00-I99	Diseases of the circulatory system	641554	Athscl type of bypass of the left leg w ulceration of calf	ICD10CM	1	I70.742		Atherosclerosis of other type of bypass graft(s) of the lef	IMO	GHS-PACDC	IMO_2015_R	DELETED	Y	1900-01-01	9999-12-31	2015-07-10
Diagnosis: ICD10 Code, I00-I99, Define	I00-I99	Diseases of the circulatory system	1221544	Atherosclerosis of other bypass graft of lower extremity with ulceration, unspecified laterality, u	ICD10CM	1	I70.75		Atherosclerosis of other type of bypass graft(s) of other	IMO	GHS-PACDC	IMO_2016_R	ACTIVE	N	1900-01-01	9999-12-31	2016-06-02
Diagnosis: ICD10 Code, I00-I99, Define	I00-I99	Diseases of the circulatory system	1222216	Atherosclerosis of other bypass graft of lower extremity with ulceration of heel, unspecified late	ICD10CM	1	I70.75		Atherosclerosis of other type of bypass graft(s) of other	IMO	GHS-PACDC	IMO_2016_R	ACTIVE	N	1900-01-01	9999-12-31	2016-06-02
Diagnosis: ICD10 Code, I00-I99, Define	I00-I99	Diseases of the circulatory system	1177316	Atherosclerosis of other type of bypass graft of right lower extremity with gangrene (HCC)	ICD10CM	1	I70.761		Atherosclerosis of other type of bypass graft(s) of the ex	IMO	GHS-PACDC	IMO_2016_R	DELETED	Y	1900-01-01	9999-12-31	2016-06-02
Diagnosis: ICD10 Code, I00-I99, Define	I00-I99	Diseases of the circulatory system	1182042	Atherosclerosis of other type of bypass graft(s) of the extremities with gangrene, left leg (HCC)	ICD10CM	1	I70.762		Atherosclerosis of other type of bypass graft(s) of the ex	IMO	GHS-PACDC	IMO_2016_R	ACTIVE	N	1900-01-01	9999-12-31	2016-06-02
Diagnosis: ICD10 Code, I00-I99, Define	I00-I99	Diseases of the circulatory system	642067	Athscl type of bypass of the extrm w gangrene, left leg	ICD10CM	1	I70.762		Atherosclerosis of other type of bypass graft(s) of the ex	IMO	GHS-PACDC	IMO_2015_R	DELETED	Y	1900-01-01	9999-12-31	2015-07-10
Diagnosis: ICD10 Code, I00-I99, Define	I00-I99	Diseases of the circulatory system	599288	Atherosclerosis of other type of bypass graft(s) of the extremities with gangrene, unspecified ex	ICD10CM	1	I70.769		Atherosclerosis of other type of bypass graft(s) of the ex	IMO	GHS-PACDC	IMO_2015_R	ACTIVE	Y	1900-01-01	9999-12-31	2015-07-10
Diagnosis: ICD10 Code, I00-I99, Define	I00-I99	Diseases of the circulatory system	642137	Oth athscl type of bypass of the extremities, right leg	ICD10CM	1	I70.791		Other atherosclerosis of other type of bypass graft(s) of	IMO	GHS-PACDC	IMO_2015_R	DELETED	Y	1900-01-01	9999-12-31	2015-07-10
Diagnosis: ICD10 Code, I00-I99, Define	I00-I99	Diseases of the circulatory system	1180922	Other atherosclerosis of other type of bypass graft(s) of the extremities, right leg (HCC)	ICD10CM	1	I70.791		Other atherosclerosis of other type of bypass graft(s) of	IMO	GHS-PACDC	IMO_2016_R	ACTIVE	N	1900-01-01	9999-12-31	2016-06-02
Diagnosis: ICD10 Code, I00-I99, Define	I00-I99	Diseases of the circulatory system	641616	Oth athscl type of bypass of the extremities, bilateral legs	ICD10CM	1	I70.793		Other atherosclerosis of other type of bypass graft(s) of	IMO	GHS-PACDC	IMO_2015_R	DELETED	Y	1900-01-01	9999-12-31	2015-07-10
Diagnosis: ICD10 Code, I00-I99, Define	I00-I99	Diseases of the circulatory system	642016	Oth athscl type of bypass of the extremities, oth extremity	ICD10CM	1	I70.798		Other atherosclerosis of other type of bypass graft(s) of	IMO	GHS-PACDC	IMO_2015_R	DELETED	Y	1900-01-01	9999-12-31	2015-07-10
Diagnosis: ICD10 Code, I00-I99, Define	I00-I99	Diseases of the circulatory system	125178	Arterial fatty streaks	ICD10CM	1	I70.90		Unspecified atherosclerosis	IMO	GHS-PACDC	DX_LEGACY	ACTIVE	N	1900-01-01	9999-12-31	2015-07-10
Diagnosis: ICD10 Code, I00-I99, Define	I00-I99	Diseases of the circulatory system	104183	Arteriovascular disease	ICD10CM	1	I70.90		Unspecified atherosclerosis	IMO	GHS-PACDC	DX_LEGACY	ACTIVE	N	1900-01-01	9999-12-31	2015-07-10
Diagnosis: ICD10 Code, I00-I99, Define	I00-I99	Diseases of the circulatory system	146019	Arteriolosclerosis	ICD10CM	1	I70.90		Unspecified atherosclerosis	IMO	GHS-PACDC	DX_LEGACY	ACTIVE	N	1900-01-01	9999-12-31	2015-07-10
Diagnosis: ICD10 Code, I00-I99, Define	I00-I99	Diseases of the circulatory system	43229	Arteriosclerosis	ICD10CM	1	I70.90		Unspecified atherosclerosis	IMO	GHS-PACDC	DX_LEGACY	ACTIVE	N	1900-01-01	9999-12-31	2015-07-10
Diagnosis: ICD10 Code, I00-I99, Define	I00-I99	Diseases of the circulatory system	1353044	Aortic dissection with lentiginosis (HCC)	ICD10CM	1	I71.00	I71.00,L81.4	Dissection of unspecified site of aorta	IMO	GHS-PACDC	IMO_2018_R	ACTIVE	N	1900-01-01	9999-12-31	2017-10-30
Diagnosis: ICD10 Code, I00-I99, Define	I00-I99	Diseases of the circulatory system	258811	Type 1 dissection of ascending aorta (HCC)	ICD10CM	1	I71.01		Dissection of thoracic aorta	IMO	GHS-PACDC	DX_LEGACY	ACTIVE	N	1900-01-01	9999-12-31	2015-07-10
Diagnosis: ICD10 Code, I00-I99, Define	I00-I99	Diseases of the circulatory system	303156	Type 1 dissection of thoracic aorta (HCC)	ICD10CM	1	I71.01		Dissection of thoracic aorta	IMO	GHS-PACDC	DX_LEGACY	ACTIVE	N	1900-01-01	9999-12-31	2015-07-10
Diagnosis: ICD10 Code, I00-I99, Define	I00-I99	Diseases of the circulatory system	303294	Dissecting aneurysm of descending thoracic aorta (HCC)	ICD10CM	1	I71.01		Dissection of thoracic aorta	IMO	GHS-PACDC	DX_LEGACY	ACTIVE	N	1900-01-01	9999-12-31	2015-07-10
Diagnosis: ICD10 Code, I00-I99, Define	I00-I99	Diseases of the circulatory system	253016	Dissection of descending thoracic aorta (HCC)	ICD10CM	1	I71.01		Dissection of thoracic aorta	IMO	GHS-PACDC	DX_LEGACY	ACTIVE	N	1900-01-01	9999-12-31	2015-07-10
Diagnosis: ICD10 Code, I00-I99, Define	I00-I99	Diseases of the circulatory system	39400	Dissection of aorta, abdominal (HCC)	ICD10CM	1	I71.02		Dissection of abdominal aorta	IMO	GHS-PACDC	DX_LEGACY	ACTIVE	N	1900-01-01	9999-12-31	2015-07-10
Diagnosis: ICD10 Code, I00-I99, Define	I00-I99	Diseases of the circulatory system	76182	Dissecting aortic aneurysm (any part), thoracoabdominal (HCC)	ICD10CM	1	I71.03		Dissection of thoracoabdominal aorta	IMO	GHS-PACDC	DX_LEGACY	ACTIVE	N	1900-01-01	9999-12-31	2015-07-10
Diagnosis: ICD10 Code, I00-I99, Define	I00-I99	Diseases of the circulatory system	62055	Aneurysm, thoracic, ruptured (HCC)	ICD10CM	1	I71.1		Thoracic aortic aneurysm, ruptured	IMO	GHS-PACDC	DX_LEGACY	ACTIVE	N	1900-01-01	9999-12-31	2015-07-10
Diagnosis: ICD10 Code, I00-I99, Define	I00-I99	Diseases of the circulatory system	1307478	Abdominal aortic aneurysm (AAA) 3.0 cm to 5.0 cm in diameter in female (HCC)	ICD10CM	1	I71.4		Abdominal aortic aneurysm, without rupture	IMO	GHS-PACDC	IMO_2017_R	ACTIVE	N	1900-01-01	9999-12-31	2017-04-14
Diagnosis: ICD10 Code, I00-I99, Define	I00-I99	Diseases of the circulatory system	65099	Aortic aneurysm, abdominal (HCC)	ICD10CM	1	I71.4		Abdominal aortic aneurysm, without rupture	IMO	GHS-PACDC	DX_LEGACY	ACTIVE	N	1900-01-01	9999-12-31	2015-07-10
Diagnosis: ICD10 Code, I00-I99, Define	I00-I99	Diseases of the circulatory system	62062	Abdominal aneurysm without mention of rupture	ICD10CM	1	I71.4		Abdominal aortic aneurysm, without rupture	IMO	GHS-PACDC	DX_LEGACY	ACTIVE	Y	1900-01-01	9999-12-31	2015-07-10
Diagnosis: ICD10 Code, I00-I99, Define	I00-I99	Diseases of the circulatory system	1307479	Abdominal aortic aneurysm (AAA) greater than 5.5 cm in diameter in male (HCC)	ICD10CM	1	I71.4		Abdominal aortic aneurysm, without rupture	IMO	GHS-PACDC	IMO_2017_R	ACTIVE	N	1900-01-01	9999-12-31	2017-04-14
Diagnosis: ICD10 Code, I00-I99, Define	I00-I99	Diseases of the circulatory system	1257812	Abdominal aortic aneurysm (AAA) without rupture (HCC)	ICD10CM	1	I71.4		Abdominal aortic aneurysm, without rupture	IMO	GHS-PACDC	IMO_2017_R	ACTIVE	N	1900-01-01	9999-12-31	2016-09-30
Diagnosis: ICD10 Code, I00-I99, Define	I00-I99	Diseases of the circulatory system	535852	Aortic aneurysm of unspecified site, ruptured	ICD10CM	1	I71.8		Aortic aneurysm of unspecified site, ruptured	IMO	GHS-PACDC	IMO_2015_R	ACTIVE	Y	1900-01-01	9999-12-31	2015-07-10
Diagnosis: ICD10 Code, I00-I99, Define	I00-I99	Diseases of the circulatory system	234336	Descending aortic aneurysm (HCC)	ICD10CM	1	I71.9		Aortic aneurysm of unspecified site, without rupture	IMO	GHS-PACDC	DX_LEGACY	ACTIVE	N	1900-01-01	9999-12-31	2015-07-10
Diagnosis: ICD10 Code, I00-I99, Define	I00-I99	Diseases of the circulatory system	332548	Aneurysm of aortic root (HCC)	ICD10CM	1	I71.9		Aortic aneurysm of unspecified site, without rupture	IMO	GHS-PACDC	DX_LEGACY	ACTIVE	N	1900-01-01	9999-12-31	2015-07-10
Diagnosis: ICD10 Code, I00-I99, Define	I00-I99	Diseases of the circulatory system	1349074	Aneurysm of left renal artery (HCC)	ICD10CM	1	I72.2		Aneurysm of renal artery	IMO	GHS-PACDC	IMO_2018_R	ACTIVE	N	1900-01-01	9999-12-31	2017-10-30
Diagnosis: ICD10 Code, I00-I99, Define	I00-I99	Diseases of the circulatory system	303460	Mycotic aneurysm of kidney transplant (HCC)	ICD10CM	2	I72.2	T86.19,I72.2	Aneurysm of renal artery	IMO	GHS-PACDC	DX_LEGACY	ACTIVE	N	2016-04-10	9999-12-31	2015-07-10
Diagnosis: ICD10 Code, I00-I99, Define	I00-I99	Diseases of the circulatory system	317620	Renal artery pseudoaneurysm from second transplant (HCC)	ICD10CM	2	I72.2	T86.19,I72.2	Aneurysm of renal artery	IMO	GHS-PACDC	DX_LEGACY	ACTIVE	N	1900-01-01	9999-12-31	2015-07-10
Diagnosis: ICD10 Code, I00-I99, Define	I00-I99	Diseases of the circulatory system	303128	Ruptured aneurysm of internal iliac artery (HCC)	ICD10CM	1	I72.3		Aneurysm of iliac artery	IMO	GHS-PACDC	DX_LEGACY	ACTIVE	N	1900-01-01	9999-12-31	2015-07-10
Diagnosis: ICD10 Code, I00-I99, Define	I00-I99	Diseases of the circulatory system	229641	Bilateral pelvic artery aneurysm (HCC)	ICD10CM	1	I72.3		Aneurysm of iliac artery	IMO	GHS-PACDC	DX_LEGACY	ACTIVE	N	1900-01-01	9999-12-31	2015-07-10
Diagnosis: ICD10 Code, I00-I99, Define	I00-I99	Diseases of the circulatory system	103975	Aneurysm artery, popliteal (HCC)	ICD10CM	1	I72.4		Aneurysm of artery of lower extremity	IMO	GHS-PACDC	DX_LEGACY	ACTIVE	N	1900-01-01	9999-12-31	2015-07-10
Diagnosis: ICD10 Code, I00-I99, Define	I00-I99	Diseases of the circulatory system	15842	Femoral artery pseudoaneurysm complicating cardiac catheterization (HCC)	ICD10CM	2	I72.4	I97.88,I72.4	Aneurysm of artery of lower extremity	IMO	GHS-PACDC	DX_LEGACY	ACTIVE	N	2016-04-10	9999-12-31	2015-07-10
Diagnosis: ICD10 Code, I00-I99, Define	I00-I99	Diseases of the circulatory system	15842	Femoral artery pseudoaneurysm complicating cardiac catheterization (HCC)	ICD10CM	2	I72.4	T81.719A,I72.4	Aneurysm of artery of lower extremity	IMO	GHS-PACDC	DX_LEGACY	ACTIVE	N	1900-01-01	2016-05-07	2015-07-10
Diagnosis: ICD10 Code, I00-I99, Define	I00-I99	Diseases of the circulatory system	238578	Aneurysm of lower extremity, right (HCC)	ICD10CM	1	I72.4		Aneurysm of artery of lower extremity	IMO	GHS-PACDC	DX_LEGACY	ACTIVE	N	1900-01-01	9999-12-31	2015-07-10
Diagnosis: ICD10 Code, I00-I99, Define	I00-I99	Diseases of the circulatory system	331002	Vertebral artery aneurysm (HCC)	ICD10CM	1	I72.6		Aneurysm of vertebral artery	IMO	GHS-PACDC	DX_LEGACY	ACTIVE	N	2016-09-17	9999-12-31	2016-09-30
Diagnosis: ICD10 Code, I00-I99, Define	I00-I99	Diseases of the circulatory system	260993	Aneurysm of atrial appendage (HCC)	ICD10CM	1	I72.8		Aneurysm of other specified arteries	IMO	GHS-PACDC	DX_LEGACY	ACTIVE	N	1900-01-01	9999-12-31	2015-07-10
Diagnosis: ICD10 Code, I00-I99, Define	I00-I99	Diseases of the circulatory system	209371	Bulge of artery wall of digestive tract (HCC)	ICD10CM	1	I72.8		Aneurysm of other specified arteries	IMO	GHS-PACDC	DX_LEGACY	ACTIVE	N	1900-01-01	9999-12-31	2015-07-10
Diagnosis: ICD10 Code, I00-I99, Define	I00-I99	Diseases of the circulatory system	234212	Pseudoaneurysm of hypogastric artery (HCC)	ICD10CM	1	I72.8		Aneurysm of other specified arteries	IMO	GHS-PACDC	DX_LEGACY	ACTIVE	N	1900-01-01	9999-12-31	2015-07-10
Diagnosis: ICD10 Code, I00-I99, Define	I00-I99	Diseases of the circulatory system	1137878	Aneurysm of other specified arteries (HCC)	ICD10CM	1	I72.8		Aneurysm of other specified arteries	IMO	GHS-PACDC	IMO_2016_R	ACTIVE	N	1900-01-01	9999-12-31	2016-06-02
Diagnosis: ICD10 Code, I00-I99, Define	I00-I99	Diseases of the circulatory system	315901	Gastric artery aneurysm (HCC)	ICD10CM	1	I72.8		Aneurysm of other specified arteries	IMO	GHS-PACDC	DX_LEGACY	ACTIVE	N	1900-01-01	9999-12-31	2015-07-10
Diagnosis: ICD10 Code, I00-I99, Define	I00-I99	Diseases of the circulatory system	229684	Aneurysm of pancreatic artery (HCC)	ICD10CM	1	I72.8		Aneurysm of other specified arteries	IMO	GHS-PACDC	DX_LEGACY	ACTIVE	N	1900-01-01	9999-12-31	2015-07-10
Diagnosis: ICD10 Code, I00-I99, Define	I00-I99	Diseases of the circulatory system	1428356	Aneurysm of other specified arteries (CODE) (HCC)	ICD10CM	1	I72.8		Aneurysm of other specified arteries	IMO	GHS-PACDC	IMO_2018_R	ACTIVE	N	1900-01-01	9999-12-31	2017-10-30
Diagnosis: ICD10 Code, I00-I99, Define	I00-I99	Diseases of the circulatory system	201108	Aneurysm, splenic artery (HCC)	ICD10CM	1	I72.8		Aneurysm of other specified arteries	IMO	GHS-PACDC	DX_LEGACY	ACTIVE	N	1900-01-01	9999-12-31	2015-07-10
Diagnosis: ICD10 Code, I00-I99, Define	I00-I99	Diseases of the circulatory system	501674	Aneurysm of artery of trunk (HCC)	ICD10CM	1	I72.8		Aneurysm of other specified arteries	IMO	GHS-PACDC	IMO_2015_R	ACTIVE	N	2017-09-17	9999-12-31	2017-10-30
Diagnosis: ICD10 Code, I00-I99, Define	I00-I99	Diseases of the circulatory system	209372	Bulge of artery wall in liver (HCC)	ICD10CM	1	I72.8		Aneurysm of other specified arteries	IMO	GHS-PACDC	DX_LEGACY	ACTIVE	N	1900-01-01	9999-12-31	2015-07-10
Diagnosis: ICD10 Code, I00-I99, Define	I00-I99	Diseases of the circulatory system	22038	Aneurysmal varix of artery (HCC)	ICD10CM	1	I72.9		Aneurysm of unspecified site	IMO	GHS-PACDC	DX_LEGACY	ACTIVE	N	1900-01-01	9999-12-31	2015-07-10
Diagnosis: ICD10 Code, I00-I99, Define	I00-I99	Diseases of the circulatory system	22036	False aneurysm of artery (HCC)	ICD10CM	1	I72.9		Aneurysm of unspecified site	IMO	GHS-PACDC	DX_LEGACY	ACTIVE	N	1900-01-01	9999-12-31	2015-07-10
Diagnosis: ICD10 Code, I00-I99, Define	I00-I99	Diseases of the circulatory system	231954	False aneurysm of pancreatic artery (HCC)	ICD10CM	1	I72.9		Aneurysm of unspecified site	IMO	GHS-PACDC	DX_LEGACY	ACTIVE	N	1900-01-01	9999-12-31	2015-07-10
Diagnosis: ICD10 Code, I00-I99, Define	I00-I99	Diseases of the circulatory system	5220	Aneurysm (HCC)	ICD10CM	1	I72.9		Aneurysm of unspecified site	IMO	GHS-PACDC	DX_LEGACY	ACTIVE	N	1900-01-01	9999-12-31	2015-07-10
Diagnosis: ICD10 Code, I00-I99, Define	I00-I99	Diseases of the circulatory system	506195	Idiopathic aneurysm (HCC)	ICD10CM	1	I72.9		Aneurysm of unspecified site	IMO	GHS-PACDC	IMO_2015_R	ACTIVE	N	1900-01-01	9999-12-31	2015-07-10
Diagnosis: ICD10 Code, I00-I99, Define	I00-I99	Diseases of the circulatory system	326817	Aneurysm of arterial anastomosis (HCC)	ICD10CM	1	I72.9		Aneurysm of unspecified site	IMO	GHS-PACDC	DX_LEGACY	ACTIVE	N	1900-01-01	9999-12-31	2015-07-10
Diagnosis: ICD10 Code, I00-I99, Define	I00-I99	Diseases of the circulatory system	55326	Raynaud's phenomenon	ICD10CM	1	I73.00		Raynaud's syndrome without gangrene	IMO	GHS-PACDC	DX_LEGACY	ACTIVE	N	1900-01-01	9999-12-31	2015-07-10
Diagnosis: ICD10 Code, I00-I99, Define	I00-I99	Diseases of the circulatory system	22067	Presenile gangrene (HCC)	ICD10CM	1	I73.1		Thromboangiitis obliterans [Buerger's disease]	IMO	GHS-PACDC	DX_LEGACY	ACTIVE	N	1900-01-01	9999-12-31	2015-07-10
Diagnosis: ICD10 Code, I00-I99, Define	I00-I99	Diseases of the circulatory system	1302401	Neuropathy associated with thromboangiitis obliterans (HCC)	ICD10CM	1	I73.1	I73.1,G63	Thromboangiitis obliterans [Buerger's disease]	IMO	GHS-PACDC	IMO_2017_R	ACTIVE	N	1900-01-01	9999-12-31	2017-04-14
Diagnosis: ICD10 Code, I00-I99, Define	I00-I99	Diseases of the circulatory system	145207	Mitchell's disease (HCC)	ICD10CM	1	I73.81		Erythromelalgia	IMO	GHS-PACDC	DX_LEGACY	ACTIVE	N	1900-01-01	9999-12-31	2015-07-10
Diagnosis: ICD10 Code, I00-I99, Define	I00-I99	Diseases of the circulatory system	515424	Inherited erythromelalgia associated with mutation in SCN9A gene (HCC)	ICD10CM	1	I73.81		Erythromelalgia	IMO	GHS-PACDC	IMO_2015_R	ACTIVE	N	1900-01-01	9999-12-31	2015-07-10
Diagnosis: ICD10 Code, I00-I99, Define	I00-I99	Diseases of the circulatory system	1348546	Gangrene due to peripheral vascular disease (HCC)	ICD10CM	2	I73.9	I96,I73.9	Peripheral vascular disease, unspecified	IMO	GHS-PACDC	IMO_2018_R	ACTIVE	N	1900-01-01	9999-12-31	2017-10-30
Diagnosis: ICD10 Code, I00-I99, Define	I00-I99	Diseases of the circulatory system	539261	Peripheral vascular disease, unspecified	ICD10CM	1	I73.9		Peripheral vascular disease, unspecified	IMO	GHS-PACDC	IMO_2015_R	ACTIVE	Y	1900-01-01	9999-12-31	2015-07-10
Diagnosis: ICD10 Code, I00-I99, Define	I00-I99	Diseases of the circulatory system	331703	Poor perfusion of leg (HCC)	ICD10CM	1	I73.9		Peripheral vascular disease, unspecified	IMO	GHS-PACDC	DX_LEGACY	ACTIVE	N	1900-01-01	9999-12-31	2015-07-10
Diagnosis: ICD10 Code, I00-I99, Define	I00-I99	Diseases of the circulatory system	117464	Myasthenia angiosclerotica (HCC)	ICD10CM	1	I73.9		Peripheral vascular disease, unspecified	IMO	GHS-PACDC	DX_LEGACY	ACTIVE	N	1900-01-01	9999-12-31	2015-07-10
Diagnosis: ICD10 Code, I00-I99, Define	I00-I99	Diseases of the circulatory system	184109	Peripheral arterial disease (HCC)	ICD10CM	1	I73.9		Peripheral vascular disease, unspecified	IMO	GHS-PACDC	DX_LEGACY	ACTIVE	N	1900-01-01	9999-12-31	2015-07-10
Diagnosis: ICD10 Code, I00-I99, Define	I00-I99	Diseases of the circulatory system	18953	Saddle embolus of abdominal aorta (HCC)	ICD10CM	1	I74.01		Saddle embolus of abdominal aorta	IMO	GHS-PACDC	DX_LEGACY	ACTIVE	N	1900-01-01	9999-12-31	2015-07-10
Diagnosis: ICD10 Code, I00-I99, Define	I00-I99	Diseases of the circulatory system	306495	Embolism, aortic bifurcation (HCC)	ICD10CM	1	I74.09		Other arterial embolism and thrombosis of abdominal a	IMO	GHS-PACDC	DX_LEGACY	ACTIVE	N	1900-01-01	9999-12-31	2015-07-10
Diagnosis: ICD10 Code, I00-I99, Define	I00-I99	Diseases of the circulatory system	306492	Aortic bifurcation syndrome, chronic (HCC)	ICD10CM	1	I74.09		Other arterial embolism and thrombosis of abdominal a	IMO	GHS-PACDC	DX_LEGACY	ACTIVE	N	1900-01-01	9999-12-31	2015-07-10
Diagnosis: ICD10 Code, I00-I99, Define	I00-I99	Diseases of the circulatory system	235093	Thrombosis, upper extremity artery (HCC)	ICD10CM	1	I74.2		Embolism and thrombosis of arteries of the upper extre	IMO	GHS-PACDC	DX_LEGACY	ACTIVE	N	1900-01-01	9999-12-31	2015-07-10
Diagnosis: ICD10 Code, I00-I99, Define	I00-I99	Diseases of the circulatory system	229069	Brachial artery occlusion, left (HCC)	ICD10CM	1	I74.2		Embolism and thrombosis of arteries of the upper extre	IMO	GHS-PACDC	DX_LEGACY	ACTIVE	N	1900-01-01	9999-12-31	2015-07-10
Diagnosis: ICD10 Code, I00-I99, Define	I00-I99	Diseases of the circulatory system	600747	Embolism and thrombosis of arteries of the upper extremities	ICD10CM	1	I74.2		Embolism and thrombosis of arteries of the upper extre	IMO	GHS-PACDC	IMO_2015_R	ACTIVE	Y	1900-01-01	9999-12-31	2015-07-10
Diagnosis: ICD10 Code, I00-I99, Define	I00-I99	Diseases of the circulatory system	499745	Thrombus of left radial artery (HCC)	ICD10CM	1	I74.2		Embolism and thrombosis of arteries of the upper extre	IMO	GHS-PACDC	IMO_2015_R	ACTIVE	N	1900-01-01	9999-12-31	2015-07-10
Diagnosis: ICD10 Code, I00-I99, Define	I00-I99	Diseases of the circulatory system	255520	Ulnar artery thrombosis (HCC)	ICD10CM	1	I74.2		Embolism and thrombosis of arteries of the upper extre	IMO	GHS-PACDC	DX_LEGACY	ACTIVE	N	1900-01-01	9999-12-31	2015-07-10
Diagnosis: ICD10 Code, I00-I99, Define	I00-I99	Diseases of the circulatory system	409209	Embolism and thrombosis of femoral artery	ICD10CM	1	I74.3		Embolism and thrombosis of arteries of the lower extre	IMO	GHS-PACDC	DX_LEGACY	DELETED	Y	1900-01-01	9999-12-31	2015-07-10
Diagnosis: ICD10 Code, I00-I99, Define	I00-I99	Diseases of the circulatory system	230318	Embolus to lower extremity (HCC)	ICD10CM	1	I74.3		Embolism and thrombosis of arteries of the lower extre	IMO	GHS-PACDC	DX_LEGACY	ACTIVE	N	1900-01-01	9999-12-31	2015-07-10
Diagnosis: ICD10 Code, I00-I99, Define	I00-I99	Diseases of the circulatory system	412979	Embolus of femoral artery (HCC)	ICD10CM	1	I74.3		Embolism and thrombosis of arteries of the lower extre	IMO	GHS-PACDC	DX_LEGACY	ACTIVE	N	1900-01-01	9999-12-31	2015-07-10
Diagnosis: ICD10 Code, I00-I99, Define	I00-I99	Diseases of the circulatory system	230238	Occlusion of peroneal artery (HCC)	ICD10CM	1	I74.3		Embolism and thrombosis of arteries of the lower extre	IMO	GHS-PACDC	DX_LEGACY	ACTIVE	N	1900-01-01	9999-12-31	2015-07-10
Diagnosis: ICD10 Code, I00-I99, Define	I00-I99	Diseases of the circulatory system	228607	Femoral artery thrombosis, left (HCC)	ICD10CM	1	I74.3		Embolism and thrombosis of arteries of the lower extre	IMO	GHS-PACDC	DX_LEGACY	ACTIVE	N	1900-01-01	9999-12-31	2015-07-10
Diagnosis: ICD10 Code, I00-I99, Define	I00-I99	Diseases of the circulatory system	263290	Peripheral artery occlusion (HCC)	ICD10CM	1	I74.3		Embolism and thrombosis of arteries of the lower extre	IMO	GHS-PACDC	DX_LEGACY	ACTIVE	N	1900-01-01	2016-10-14	2015-07-10
Diagnosis: ICD10 Code, I00-I99, Define	I00-I99	Diseases of the circulatory system	329428	Occlusion of common femoral artery (HCC)	ICD10CM	1	I74.3		Embolism and thrombosis of arteries of the lower extre	IMO	GHS-PACDC	DX_LEGACY	ACTIVE	N	1900-01-01	9999-12-31	2015-07-10

Diagnosis: ICD10 Code, I00-I99, Define	I00-I99	Diseases of the circulatory system	229959	Occlusion of left peroneal artery (HCC)	ICD10CM	1	I74.3		Embolism and thrombosis of arteries of the lower extrer	IMO	GHS-PACDC	DX_LEGACY	ACTIVE	N	1900-01-01	9999-12-31	2015-07-10
Diagnosis: ICD10 Code, I00-I99, Define	I00-I99	Diseases of the circulatory system	237598	Femoral popliteal artery thrombus, right	ICD10CM	1	I74.3		Embolism and thrombosis of arteries of the lower extrer	IMO	GHS-PACDC	DX_LEGACY	DELETED	Y	1900-01-01	9999-12-31	2015-07-10
Diagnosis: ICD10 Code, I00-I99, Define	I00-I99	Diseases of the circulatory system	66755	Thrombosis, arteries, femoral (HCC)	ICD10CM	1	I74.3		Embolism and thrombosis of arteries of the lower extrer	IMO	GHS-PACDC	DX_LEGACY	ACTIVE	N	1900-01-01	9999-12-31	2015-07-10
Diagnosis: ICD10 Code, I00-I99, Define	I00-I99	Diseases of the circulatory system	229512	Thrombosis of left iliac artery (HCC)	ICD10CM	1	I74.5		Embolism and thrombosis of iliac artery	IMO	GHS-PACDC	DX_LEGACY	ACTIVE	N	1900-01-01	9999-12-31	2015-07-10
Diagnosis: ICD10 Code, I00-I99, Define	I00-I99	Diseases of the circulatory system	314618	Iliac artery thrombosis, right (HCC)	ICD10CM	1	I74.5		Embolism and thrombosis of iliac artery	IMO	GHS-PACDC	DX_LEGACY	ACTIVE	N	1900-01-01	9999-12-31	2015-07-10
Diagnosis: ICD10 Code, I00-I99, Define	I00-I99	Diseases of the circulatory system	5235	Iliac artery embolism (HCC)	ICD10CM	1	I74.5		Embolism and thrombosis of iliac artery	IMO	GHS-PACDC	DX_LEGACY	ACTIVE	N	1900-01-01	9999-12-31	2015-07-10
Diagnosis: ICD10 Code, I00-I99, Define	I00-I99	Diseases of the circulatory system	230879	Iliac artery occlusion (HCC)	ICD10CM	1	I74.5		Embolism and thrombosis of iliac artery	IMO	GHS-PACDC	DX_LEGACY	ACTIVE	N	1900-01-01	9999-12-31	2015-07-10
Diagnosis: ICD10 Code, I00-I99, Define	I00-I99	Diseases of the circulatory system	229841	Bilateral pelvic artery occlusion (HCC)	ICD10CM	1	I74.5		Embolism and thrombosis of iliac artery	IMO	GHS-PACDC	DX_LEGACY	ACTIVE	N	1900-01-01	9999-12-31	2015-07-10
Diagnosis: ICD10 Code, I00-I99, Define	I00-I99	Diseases of the circulatory system	304911	Hepatic artery thrombosis, right, transplanted liver (HCC)	ICD10CM	2	I74.8	T86.49,I74.8	Embolism and thrombosis of other arteries	IMO	GHS-PACDC	DX_LEGACY	ACTIVE	N	2016-04-10	9999-12-31	2015-07-10
Diagnosis: ICD10 Code, I00-I99, Define	I00-I99	Diseases of the circulatory system	246286	Celiac axis occlusion with collateral formation (HCC)	ICD10CM	1	I74.8		Embolism and thrombosis of other arteries	IMO	GHS-PACDC	DX_LEGACY	ACTIVE	N	1900-01-01	9999-12-31	2015-07-10
Diagnosis: ICD10 Code, I00-I99, Define	I00-I99	Diseases of the circulatory system	304084	Thrombosis of right hepatic artery of transplanted liver (HCC)	ICD10CM	2	I74.8	T86.49,I74.8	Embolism and thrombosis of other arteries	IMO	GHS-PACDC	DX_LEGACY	ACTIVE	N	2016-04-10	9999-12-31	2015-07-10
Diagnosis: ICD10 Code, I00-I99, Define	I00-I99	Diseases of the circulatory system	499477	Occlusion of left hepatic artery with collateral development (HCC)	ICD10CM	1	I74.8		Embolism and thrombosis of other arteries	IMO	GHS-PACDC	IMO_2015_R	ACTIVE	N	1900-01-01	9999-12-31	2015-07-10
Diagnosis: ICD10 Code, I00-I99, Define	I00-I99	Diseases of the circulatory system	330393	Occlusion of right subclavian artery (HCC)	ICD10CM	1	I74.8		Embolism and thrombosis of other arteries	IMO	GHS-PACDC	DX_LEGACY	ACTIVE	N	1900-01-01	9999-12-31	2015-07-10
Diagnosis: ICD10 Code, I00-I99, Define	I00-I99	Diseases of the circulatory system	539214	Embolism and thrombosis of other arteries	ICD10CM	1	I74.8		Embolism and thrombosis of other arteries	IMO	GHS-PACDC	IMO_2015_R	ACTIVE	Y	1900-01-01	9999-12-31	2015-07-10
Diagnosis: ICD10 Code, I00-I99, Define	I00-I99	Diseases of the circulatory system	252587	Chronic thromboembolic disease (HCC)	ICD10CM	1	I74.9		Embolism and thrombosis of unspecified artery	IMO	GHS-PACDC	DX_LEGACY	ACTIVE	N	1900-01-01	9999-12-31	2015-07-10
Diagnosis: ICD10 Code, I00-I99, Define	I00-I99	Diseases of the circulatory system	304911	Hepatic artery thrombosis, right, transplanted liver (HCC)	ICD10CM	2	I74.9	T86.49,I74.9	Embolism and thrombosis of unspecified artery	IMO	GHS-PACDC	DX_LEGACY	ACTIVE	N	1900-01-01	2016-05-07	2015-07-10
Diagnosis: ICD10 Code, I00-I99, Define	I00-I99	Diseases of the circulatory system	782665	Lower extremity atheroembolism, right (HCC)	ICD10CM	1	I75.021		Atheroembolism of right lower extremity	IMO	GHS-PACDC	IMO_2015_R	ACTIVE	N	1900-01-01	9999-12-31	2015-07-10
Diagnosis: ICD10 Code, I00-I99, Define	I00-I99	Diseases of the circulatory system	822609	Atheroembolism of toe, right (HCC)	ICD10CM	1	I75.021		Atheroembolism of right lower extremity	IMO	GHS-PACDC	IMO_2015_R	ACTIVE	N	1900-01-01	9999-12-31	2015-07-10
Diagnosis: ICD10 Code, I00-I99, Define	I00-I99	Diseases of the circulatory system	743216	Atherothrombotic microembolism of lower extremity, left (HCC)	ICD10CM	1	I75.022		Atheroembolism of left lower extremity	IMO	GHS-PACDC	IMO_2015_R	ACTIVE	N	1900-01-01	9999-12-31	2015-07-10
Diagnosis: ICD10 Code, I00-I99, Define	I00-I99	Diseases of the circulatory system	367141	Trash foot (HCC)	ICD10CM	2	I75.029	I96,I75.029	Atheroembolism of unspecified lower extremity	IMO	GHS-PACDC	DX_LEGACY	ACTIVE	N	2017-09-17	9999-12-31	2017-10-30
Diagnosis: ICD10 Code, I00-I99, Define	I00-I99	Diseases of the circulatory system	136137	Cholesterol embolism of leg (HCC)	ICD10CM	1	I75.029		Atheroembolism of unspecified lower extremity	IMO	GHS-PACDC	DX_LEGACY	ACTIVE	N	1900-01-01	9999-12-31	2015-07-10
Diagnosis: ICD10 Code, I00-I99, Define	I00-I99	Diseases of the circulatory system	228690	Hepatic AV fistula (HCC)	ICD10CM	1	I77.0		Arteriovenous fistula, acquired	IMO	GHS-PACDC	DX_LEGACY	ACTIVE	N	1900-01-01	9999-12-31	2015-07-10
Diagnosis: ICD10 Code, I00-I99, Define	I00-I99	Diseases of the circulatory system	207482	Acquired vascular malformation of liver (HCC)	ICD10CM	1	I77.0		Arteriovenous fistula, acquired	IMO	GHS-PACDC	DX_LEGACY	ACTIVE	N	1900-01-01	9999-12-31	2015-07-10
Diagnosis: ICD10 Code, I00-I99, Define	I00-I99	Diseases of the circulatory system	502276	Extrinsic compression of artery (HCC)	ICD10CM	1	I77.1		Stricture of artery	IMO	GHS-PACDC	IMO_2015_R	ACTIVE	N	1900-01-01	9999-12-31	2015-07-10
Diagnosis: ICD10 Code, I00-I99, Define	I00-I99	Diseases of the circulatory system	332317	Intragraft arterial stenosis of transplanted pancreas (HCC)	ICD10CM	5	I77.1	T82.868A,T86.898,Y83.0,T86.899	Stricture of artery	IMO	GHS-PACDC	DX_LEGACY	ACTIVE	N	1900-01-01	2017-10-14	2015-07-10
Diagnosis: ICD10 Code, I00-I99, Define	I00-I99	Diseases of the circulatory system	251538	SMA stenosis (HCC)	ICD10CM	1	I77.1		Stricture of artery	IMO	GHS-PACDC	DX_LEGACY	ACTIVE	N	1900-01-01	9999-12-31	2015-07-10
Diagnosis: ICD10 Code, I00-I99, Define	I00-I99	Diseases of the circulatory system	386906	Tortuous aorta (HCC)	ICD10CM	1	I77.1		Stricture of artery	IMO	GHS-PACDC	DX_LEGACY	ACTIVE	N	1900-01-01	9999-12-31	2015-07-10
Diagnosis: ICD10 Code, I00-I99, Define	I00-I99	Diseases of the circulatory system	316579	Right iliac artery stenosis (HCC)	ICD10CM	1	I77.1		Stricture of artery	IMO	GHS-PACDC	DX_LEGACY	ACTIVE	N	1900-01-01	9999-12-31	2015-07-10
Diagnosis: ICD10 Code, I00-I99, Define	I00-I99	Diseases of the circulatory system	232111	Kinking of renal artery (HCC)	ICD10CM	1	I77.1		Stricture of artery	IMO	GHS-PACDC	DX_LEGACY	ACTIVE	N	1900-01-01	9999-12-31	2015-07-10
Diagnosis: ICD10 Code, I00-I99, Define	I00-I99	Diseases of the circulatory system	232893	Stenosis of left brachiocephalic artery (HCC)	ICD10CM	1	I77.1		Stricture of artery	IMO	GHS-PACDC	DX_LEGACY	ACTIVE	N	1900-01-01	9999-12-31	2015-07-10
Diagnosis: ICD10 Code, I00-I99, Define	I00-I99	Diseases of the circulatory system	56729	Stricture, artery (HCC)	ICD10CM	1	I77.1		Stricture of artery	IMO	GHS-PACDC	DX_LEGACY	ACTIVE	N	1900-01-01	9999-12-31	2015-07-10
Diagnosis: ICD10 Code, I00-I99, Define	I00-I99	Diseases of the circulatory system	329807	Rupture of iliac artery (HCC)	ICD10CM	1	I77.2		Rupture of artery	IMO	GHS-PACDC	DX_LEGACY	ACTIVE	N	1900-01-01	9999-12-31	2015-07-10
Diagnosis: ICD10 Code, I00-I99, Define	I00-I99	Diseases of the circulatory system	228937	Aorto-enteric fistula (HCC)	ICD10CM	1	I77.2		Rupture of artery	IMO	GHS-PACDC	DX_LEGACY	ACTIVE	N	2016-04-10	9999-12-31	2015-07-10
Diagnosis: ICD10 Code, I00-I99, Define	I00-I99	Diseases of the circulatory system	229431	Hepatic artery fistula (HCC)	ICD10CM	1	I77.2		Rupture of artery	IMO	GHS-PACDC	DX_LEGACY	ACTIVE	N	1900-01-01	9999-12-31	2015-07-10
Diagnosis: ICD10 Code, I00-I99, Define	I00-I99	Diseases of the circulatory system	228593	Fibromuscular dysplasia (HCC)	ICD10CM	1	I77.3		Arterial fibromuscular dysplasia	IMO	GHS-PACDC	DX_LEGACY	ACTIVE	N	1900-01-01	9999-12-31	2015-07-10
Diagnosis: ICD10 Code, I00-I99, Define	I00-I99	Diseases of the circulatory system	525404	Fibromuscular dysplasia of cervicocranial artery (HCC)	ICD10CM	1	I77.3		Arterial fibromuscular dysplasia	IMO	GHS-PACDC	IMO_2015_R	ACTIVE	N	1900-01-01	9999-12-31	2015-07-10
Diagnosis: ICD10 Code, I00-I99, Define	I00-I99	Diseases of the circulatory system	341671	Acute intracranial arteriopathy associated with fibromuscular dysplasia (HCC)	ICD10CM	1	I77.3		Arterial fibromuscular dysplasia	IMO	GHS-PACDC	DX_LEGACY	ACTIVE	N	1900-01-01	9999-12-31	2015-07-10
Diagnosis: ICD10 Code, I00-I99, Define	I00-I99	Diseases of the circulatory system	97012	Celiac axis compression syndrome (HCC)	ICD10CM	1	I77.4		Celiac artery compression syndrome	IMO	GHS-PACDC	DX_LEGACY	ACTIVE	N	1900-01-01	9999-12-31	2015-07-10
Diagnosis: ICD10 Code, I00-I99, Define	I00-I99	Diseases of the circulatory system	5256	Necrosis of artery (HCC)	ICD10CM	1	I77.5		Necrosis of artery	IMO	GHS-PACDC	DX_LEGACY	ACTIVE	N	1900-01-01	9999-12-31	2015-07-10
Diagnosis: ICD10 Code, I00-I99, Define	I00-I99	Diseases of the circulatory system	67622	Endarteritis infective (HCC)	ICD10CM	1	I77.6		Arteritis, unspecified	IMO	GHS-PACDC	DX_LEGACY	ACTIVE	N	1900-01-01	9999-12-31	2015-07-10
Diagnosis: ICD10 Code, I00-I99, Define	I00-I99	Diseases of the circulatory system	226730	Central nervous system vasculitis (HCC)	ICD10CM	1	I77.6		Arteritis, unspecified	IMO	GHS-PACDC	DX_LEGACY	ACTIVE	N	1900-01-01	9999-12-31	2015-07-10
Diagnosis: ICD10 Code, I00-I99, Define	I00-I99	Diseases of the circulatory system	226735	Primary central nervous system vasculitis (HCC)	ICD10CM	1	I77.6		Arteritis, unspecified	IMO	GHS-PACDC	DX_LEGACY	ACTIVE	N	1900-01-01	9999-12-31	2015-07-10
Diagnosis: ICD10 Code, I00-I99, Define	I00-I99	Diseases of the circulatory system	514731	Vasculitis determined by biopsy of nerve (HCC)	ICD10CM	1	I77.6		Arteritis, unspecified	IMO	GHS-PACDC	IMO_2015_R	ACTIVE	N	1900-01-01	9999-12-31	2015-07-10
Diagnosis: ICD10 Code, I00-I99, Define	I00-I99	Diseases of the circulatory system	203176	Perinatal noninfectious intracranial vasculitis (HCC)	ICD10CM	2	I77.6	P96.89,I77.6	Arteritis, unspecified	IMO	GHS-PACDC	DX_LEGACY	ACTIVE	N	1900-01-01	9999-12-31	2015-07-10
Diagnosis: ICD10 Code, I00-I99, Define	I00-I99	Diseases of the circulatory system	202077	Acute noninfectious cervical arteritis (HCC)	ICD10CM	1	I77.6		Arteritis, unspecified	IMO	GHS-PACDC	DX_LEGACY	ACTIVE	N	1900-01-01	9999-12-31	2015-07-10
Diagnosis: ICD10 Code, I00-I99, Define	I00-I99	Diseases of the circulatory system	16018	Systemic vasculitis with associated relapsing polychondritis (HCC)	ICD10CM	1	I77.6		Arteritis, unspecified	IMO	GHS-PACDC	DX_LEGACY	ACTIVE	N	1900-01-01	9999-12-31	2015-07-10
Diagnosis: ICD10 Code, I00-I99, Define	I00-I99	Diseases of the circulatory system	537920	Dissection of carotid artery	ICD10CM	1	I77.71		Dissection of carotid artery	IMO	GHS-PACDC	IMO_2015_R	ACTIVE	Y	1900-01-01	9999-12-31	2015-07-10
Diagnosis: ICD10 Code, I00-I99, Define	I00-I99	Diseases of the circulatory system	16205	Dissection of iliac artery (HCC)	ICD10CM	1	I77.72		Dissection of iliac artery	IMO	GHS-PACDC	DX_LEGACY	ACTIVE	N	1900-01-01	9999-12-31	2015-07-10
Diagnosis: ICD10 Code, I00-I99, Define	I00-I99	Diseases of the circulatory system	537921	Dissection of iliac artery	ICD10CM	1	I77.72		Dissection of iliac artery	IMO	GHS-PACDC	IMO_2015_R	ACTIVE	Y	1900-01-01	9999-12-31	2015-07-10
Diagnosis: ICD10 Code, I00-I99, Define	I00-I99	Diseases of the circulatory system	386025	Dissecting aneurysm of basilar artery (HCC)	ICD10CM	1	I77.75		Dissection of other precerebral arteries	IMO	GHS-PACDC	DX_LEGACY	ACTIVE	N	2016-09-17	9999-12-31	2016-09-30
Diagnosis: ICD10 Code, I00-I99, Define	I00-I99	Diseases of the circulatory system	1258299	Dissection of artery of lower extremity (HCC)	ICD10CM	1	I77.77		Dissection of artery of lower extremity	IMO	GHS-PACDC	IMO_2017_R	ACTIVE	N	1900-01-01	9999-12-31	2016-09-30
Diagnosis: ICD10 Code, I00-I99, Define	I00-I99	Diseases of the circulatory system	318218	Celiac artery dissection (HCC)	ICD10CM	1	I77.79		Dissection of other specified artery	IMO	GHS-PACDC	DX_LEGACY	ACTIVE	N	1900-01-01	9999-12-31	2015-07-10
Diagnosis: ICD10 Code, I00-I99, Define	I00-I99	Diseases of the circulatory system	136099	Arterial dissection (HCC)	ICD10CM	1	I77.79		Dissection of other specified artery	IMO	GHS-PACDC	DX_LEGACY	ACTIVE	N	1900-01-01	2016-10-14	2015-07-10
Diagnosis: ICD10 Code, I00-I99, Define	I00-I99	Diseases of the circulatory system	231624	Hepatic artery dissection (HCC)	ICD10CM	1	I77.79		Dissection of other specified artery	IMO	GHS-PACDC	DX_LEGACY	ACTIVE	N	1900-01-01	9999-12-31	2015-07-10
Diagnosis: ICD10 Code, I00-I99, Define	I00-I99	Diseases of the circulatory system	586970	Thoracic aortic ectasia	ICD10CM	1	I77.810		Thoracic aortic ectasia	IMO	GHS-PACDC	IMO_2015_R	ACTIVE	Y	1900-01-01	9999-12-31	2015-07-10
Diagnosis: ICD10 Code, I00-I99, Define	I00-I99	Diseases of the circulatory system	154007	Dilatation of thoracic aorta (HCC)	ICD10CM	1	I77.810		Thoracic aortic ectasia	IMO	GHS-PACDC	DX_LEGACY	ACTIVE	N	2016-04-10	9999-12-31	2015-07-10
Diagnosis: ICD10 Code, I00-I99, Define	I00-I99	Diseases of the circulatory system	253048	Ectatic abdominal aorta (HCC)	ICD10CM	1	I77.811		Abdominal aortic ectasia	IMO	GHS-PACDC	DX_LEGACY	ACTIVE	N	1900-01-01	9999-12-31	2015-07-10
Diagnosis: ICD10 Code, I00-I99, Define	I00-I99	Diseases of the circulatory system	18700	Aortic ectasia, abdominal (HCC)	ICD10CM	1	I77.811		Abdominal aortic ectasia	IMO	GHS-PACDC	DX_LEGACY	ACTIVE	N	1900-01-01	9999-12-31	2015-07-10
Diagnosis: ICD10 Code, I00-I99, Define	I00-I99	Diseases of the circulatory system	111080	Maladie de Degos (HCC)	ICD10CM	1	I77.89		Other specified disorders of arteries and arterioles	IMO	GHS-PACDC	DX_LEGACY	ACTIVE	N	1900-01-01	9999-12-31	2015-07-10
Diagnosis: ICD10 Code, I00-I99, Define	I00-I99	Diseases of the circulatory system	246222	Leaking from artery of pancreas (HCC)	ICD10CM	1	I77.89		Other specified disorders of arteries and arterioles	IMO	GHS-PACDC	DX_LEGACY	ACTIVE	N	1900-01-01	9999-12-31	2015-07-10
Diagnosis: ICD10 Code, I00-I99, Define	I00-I99	Diseases of the circulatory system	65640	Degos' disease (HCC)	ICD10CM	1	I77.89		Other specified disorders of arteries and arterioles	IMO	GHS-PACDC	DX_LEGACY	ACTIVE	N	1900-01-01	9999-12-31	2015-07-10
Diagnosis: ICD10 Code, I00-I99, Define	I00-I99	Diseases of the circulatory system	1274680	Livedo reticularis and cerebrovascular accident (CVA) syndrome (HCC)	ICD10CM	1	I77.89		Other specified disorders of arteries and arterioles	IMO	GHS-PACDC	IMO_2017_R	ACTIVE	N	2017-09-17	9999-12-31	2017-10-30
Diagnosis: ICD10 Code, I00-I99, Define	I00-I99	Diseases of the circulatory system	232103	Irregularity of native hepatic artery (HCC)	ICD10CM	1	I77.89		Other specified disorders of arteries and arterioles	IMO	GHS-PACDC	DX_LEGACY	ACTIVE	N	1900-01-01	9999-12-31	2015-07-10
Diagnosis: ICD10 Code, I00-I99, Define	I00-I99	Diseases of the circulatory system	98165	Dysplasia, fibromuscular, artery, renal (HCC)	ICD10CM	1	I77.89		Other specified disorders of arteries and arterioles	IMO	GHS-PACDC	DX_LEGACY	ACTIVE	N	1900-01-01	9999-12-31	2015-07-10
Diagnosis: ICD10 Code, I00-I99, Define	I00-I99	Diseases of the circulatory system	202370	Chronic cervical arteriopathy associated with infection (HCC)	ICD10CM	1	I77.9	I77.9,B99.9	Disorder of arteries and arterioles, unspecified	IMO	GHS-PACDC	DX_LEGACY	ACTIVE	N	1900-01-01	9999-12-31	2015-07-10
Diagnosis: ICD10 Code, I00-I99, Define	I00-I99	Diseases of the circulatory system	342352	Arteriopathy, cervical, assoc w/ surg rep of aortic arch anomaly, perinatal (HCC)	ICD10CM	2	I77.9	P96.89,I77.9,Z98.89	Disorder of arteries and arterioles, unspecified	IMO	GHS-PACDC	DX_LEGACY	ACTIVE	N	1900-01-01	2016-05-07	2015-07-10
Diagnosis: ICD10 Code, I00-I99, Define	I00-I99	Diseases of the circulatory system	341869	Chronic cervical arteriopathy associated with surgical repair of aortic arch anomaly (HCC)	ICD10CM	1	I77.9	I77.9,Z98.89	Disorder of arteries and arterioles, unspecified	IMO	GHS-PACDC	DX_LEGACY	ACTIVE	N	1900-01-01	2016-10-14	2015-07-10
Diagnosis: ICD10 Code, I00-I99, Define	I00-I99	Diseases of the circulatory system	342330	Arteriopathy, intracranial, focal arteriopathy, post-varicella, perinatal (HCC)	ICD10CM	3	I77.9	P96.89,B01.89,I77.9	Disorder of arteries and arterioles, unspecified	IMO	GHS-PACDC	DX_LEGACY	ACTIVE	N	1900-01-01	9999-12-31	2015-07-10
Diagnosis: ICD10 Code, I00-I99, Define	I00-I99	Diseases of the circulatory system	202958	Perinatal cervical arteriopathy (HCC)	ICD10CM	2	I77.9	P96.89,I77.9	Disorder of arteries and arterioles, unspecified	IMO	GHS-PACDC	DX_LEGACY	ACTIVE	N	1900-01-01	9999-12-31	2015-07-10
Diagnosis: ICD10 Code, I00-I99, Define	I00-I99	Diseases of the circulatory system	15993	Vascular complications of renal artery (HCC)	ICD10CM	1	I77.9		Disorder of arteries and arterioles, unspecified	IMO	GHS-PACDC	DX_LEGACY	ACTIVE	Y	1900-01-01	9999-12-31	2017-04-14
Diagnosis: ICD10 Code, I00-I99, Define	I00-I99	Diseases of the circulatory system	203197	Arteriopathy, cervical, associated with infection, perinatal (HCC)	ICD10CM	2	I77.9	P96.89,I77.9,B99.9	Disorder of arteries and arterioles, unspecified	IMO	GHS-PACDC	DX_LEGACY	ACTIVE	N	1900-01-01	9999-12-31	2015-07-10
Diagnosis: ICD10 Code, I00-I99, Define	I00-I99	Diseases of the circulatory system	202558	Chronic idiopathic focal intracranial arteriopathy (HCC)	ICD10CM	1	I77.9		Disorder of arteries and arterioles, unspecified	IMO	GHS-PACDC	DX_LEGACY	ACTIVE	N	1900-01-01	9999-12-31	2015-07-10
Diagnosis: ICD10 Code, I00-I99, Define	I00-I99	Diseases of the circulatory system	202552	Acute intracranial arteriopathy associated with hemoglobinopathy (HCC)	ICD10CM	1	I77.9	I77.9,D58.2	Disorder of arteries and arterioles, unspecified	IMO	GHS-PACDC	DX_LEGACY	ACTIVE	N	1900-01-01	9999-12-31	2015-07-10
Diagnosis: ICD10 Code, I00-I99, Define	I00-I99	Diseases of the circulatory system	1355923	Autosomal recessive cerebral arteriopathy with subcortical infarcts and leukoencephalopathy (H	ICD10CM	1	I77.9	I77.9,I63.9,G93.49	Disorder of arteries and arterioles, unspecified	IMO	GHS-PACDC	IMO_2018_R	ACTIVE	N	1900-01-01	9999-12-31	2017-10-30
Diagnosis: ICD10 Code, I00-I99, Define	I00-I99	Diseases of the circulatory system	342352	Arteriopathy, cervical, assoc w/ surg rep of aortic arch anomaly, perinatal (HCC)	ICD10CM	2	I77.9	P96.89,I77.9,Z87.74	Disorder of arteries and arterioles, unspecified	IMO	GHS-PACDC	DX_LEGACY	ACTIVE	N	2016-04-10	9999-12-31	2015-07-10
Diagnosis: ICD10 Code, I00-I99, Define	I00-I99	Diseases of the circulatory system	1354213	Hereditary hemorrhagic telangiectasia type 2 (HCC)	ICD10CM	1	I78.0		Hereditary hemorrhagic telangiectasia	IMO	GHS-PACDC	IMO_2018_R	ACTIVE	N	1900-01-01	9999-12-31	2017-10-30
Diagnosis: ICD10 Code, I00-I99, Define	I00-I99	Diseases of the circulatory system	1355541	Familial capillaro-venous leptomeningeal angiomatosis (HCC)	ICD10CM	1	I78.0		Hereditary hemorrhagic telangiectasia	IMO	GHS-PACDC	IMO_2018_R	ACTIVE	N	1900-01-01	9999-12-31	2017-10-30
Diagnosis: ICD10 Code, I00-I99, Define	I00-I99	Diseases of the circulatory system	515784	SMAD4-related hereditary hemorrhagic telangiectasia syndrome (HCC)	ICD10CM	1	I78.0		Hereditary hemorrhagic telangiectasia	IMO	GHS-PACDC	IMO_2015_R	ACTIVE	N	1900-01-01	9999-12-31	2015-07-10
Diagnosis: ICD10 Code, I00-I99, Define	I00-I99	Diseases of the circulatory system	533050	Familial cutaneous telangiectasia with predisposition to malignant neoplasm (HCC)	ICD10CM	1	I78.0	I78.0,C80.1	Hereditary hemorrhagic telangiectasia	IMO	GHS-PACDC	IMO_2015_R	ACTIVE	N	1900-01-01	2017-04-27	2015-07-10
Diagnosis: ICD10 Code, I00-I99, Define	I00-I99	Diseases of the circulatory system	22162	Spider angioma of skin	ICD10CM	1	I78.1		Nevus, non-neoplastic	IMO	GHS-PACDC	DX_LEGACY	ACTIVE	N	1900-01-01	9999-12-31	2015-07-10
Diagnosis: ICD10 Code, I00-I99, Define	I00-I99	Diseases of the circulatory system	1260206	Nevoid telangiectasia, congenital	ICD10CM	1	I78.1		Nevus, non-neoplastic	IMO	GHS-PACDC	IMO_2017_R	ACTIVE	N	1900-01-01	9999-12-31	2016-09-30
Diagnosis: ICD10 Code, I00-I99, Define	I00-I99	Diseases of the circulatory system	100898	Non-neoplastic nevus	ICD10CM	1	I78.1		Nevus, non-neoplastic	IMO	GHS-PACDC	DX_LEGACY	ACTIVE	N	1900-01-01	9999-12-31	2015-07-10
Diagnosis: ICD10 Code, I00-I99, Define	I00-I99	Diseases of the circulatory system	258518	Telangiectasia of face	ICD10CM	1	I78.1		Nevus, non-neoplastic	IMO	GHS-PACDC	DX_LEGACY	ACTIVE	N	1900-01-01	9999-12-31	2015-07-10
Diagnosis: ICD10 Code, I00-I99, Define	I00-I99	Diseases of the circulatory system	22157	Angioma serpiginosum of skin	ICD10CM	1	I78.1		Nevus, non-neoplastic	IMO	GHS-PACDC	DX_LEGACY	ACTIVE	N	1900-01-01	2016-05-07	2015-07-10
Diagnosis: ICD10 Code, I00-I99, Define	I00-I99	Diseases of the circulatory system	255457	Symptomatic spider angioma	ICD10CM	1	I78.1		Nevus, non-neoplastic	IMO	GHS-PACDC	DX_LEGACY	ACTIVE	N	1900-01-01	9999-12-31	2015-07-10
Diagnosis: ICD10 Code, I00-I99, Define	I00-I99	Diseases of the circulatory system	22165	Stellar nevus of skin	ICD10CM	1	I78.1		Nevus, non-neoplastic	IMO	GHS-PACDC	DX_LEGACY	ACTIVE	N	1900-01-01	9999-12-31	2015-07-10
Diagnosis: ICD10 Code, I00-I99, Define	I00-I99	Diseases of the circulatory system	66274	Increased capillary permeability	ICD10CM	1	I78.8		Other diseases of capillaries	IMO	GHS-PACDC	DX_LEGACY	ACTIVE	N	1900-01-01	9999-12-31	2015-07-10
Diagnosis: ICD10 Code, I00-I99, Define	I00-I99	Diseases of the circulatory system	262363	Telangiectasia of skin	ICD10CM	1	I78.9		Disease of capillaries, unspecified	IMO	GHS-PACDC	DX_LEGACY	ACTIVE	N	1900-01-01	2016-05-07	2015-07-10
Diagnosis: ICD10 Code, I00-I99, Define	I00-I99	Diseases of the circulatory system	606811	Aneurysm of aorta in diseases classified elsewhere	ICD10CM	1	I79.0		Aneurysm of aorta in diseases classified elsewhere	IMO	GHS-PACDC	IMO_2015_R	ACTIVE	Y	1900-01-01	9999-12-31	2015-07-10
Diagnosis: ICD10 Code, I00-I99, Define	I00-I99	Diseases of the circulatory system	233160	Type 2 diabetes with peripheral circulatory disorder, controlled (HCC)	ICD10CM	2	I79.8	E11.51,I79.8	Other disorders of arteries, arterioles and capillaries in c	IMO	GHS-PACDC	DX_LEGACY	ACTIVE	N	1900-01-01	2016-05-07	2015-07-10
Diagnosis: ICD10 Code, I00-I99, Define	I00-I99	Diseases of the circulatory system	862564	Saphenous vein phlebitis, unspecified laterality	ICD10CM	1	I80.00		Phlebitis and thrombophlebitis of superficial vessels of u	IMO	GHS-PACDC	IMO_2015_R	ACTIVE	N	1900-01-01	9999-12-31	2015-07-10
Diagnosis: ICD10 Code, I00-I99, Define	I00-I99	Diseases of the circulatory system	784822	Superficial phlebitis of leg, unspecified laterality	ICD10CM	1	I80.00		Phlebitis and thrombophlebitis of superficial vessels of u	IMO	GHS-PACDC	IMO_2015_R	ACTIVE	N	1900-01-01	9999-12-31	2015-07-10
Diagnosis: ICD10 Code, I00-I99, Define	I00-I99	Diseases of the circulatory system	102457	Superficial thrombophlebitis of leg	ICD10CM	1	I80.00		Phlebitis and thrombophlebitis of superficial vessels of u	IMO	GHS-PACDC	DX_LEGACY	ACTIVE	N	1900-01-01	9999-12-31	2015-07-10
Diagnosis: ICD10 Code, I00-I99, Define	I00-I99	Diseases of the circulatory system	752643	Phlebitis of superficial veins of lower extremity, left	ICD10CM	1	I80.02		Phlebitis and thrombophlebitis of superficial vessels of l	IMO	GHS-PACDC	IMO_2015_R	ACTIVE	N	1900-01-01	9999-12-31	2015-07-10
Diagnosis: ICD10 Code, I00-I99, Define	I00-I99	Diseases of the circulatory system	502869	Chronic superficial phlebitis of left lower extremity	ICD10CM	1	I80.02		Phlebitis and thrombophlebitis of superficial vessels of l	IMO	GHS-PACDC	IMO_2015_R	ACTIVE	N	1900-01-01	9999-12-31	2015-07-10

Diagnosis: ICD10 Code, I00-I99, Define	I00-I99	Diseases of the circulatory system	478480	Superficial phlebitis and thrombophlebitis of left leg	ICD10CM	1	I80.02		Phlebitis and thrombophlebitis of superficial vessels of l	IMO	GHS-PACDC	IMO_2015_R	ACTIVE	N	1900-01-01	9999-12-31	2015-07-10
Diagnosis: ICD10 Code, I00-I99, Define	I00-I99	Diseases of the circulatory system	479714	Phlebitis of left saphenous vein	ICD10CM	1	I80.02		Phlebitis and thrombophlebitis of superficial vessels of l	IMO	GHS-PACDC	IMO_2015_R	ACTIVE	N	1900-01-01	9999-12-31	2015-07-10
Diagnosis: ICD10 Code, I00-I99, Define	I00-I99	Diseases of the circulatory system	937856	Phlebitis of femoropopliteal vein, bilateral	ICD10CM	1	I80.03		Phlebitis and thrombophlebitis of superficial vessels of l	IMO	GHS-PACDC	IMO_2015_R	ACTIVE	N	1900-01-01	9999-12-31	2015-07-10
Diagnosis: ICD10 Code, I00-I99, Define	I00-I99	Diseases of the circulatory system	67598	Femoral vein thrombophlebitis (HCC)	ICD10CM	1	I80.10		Phlebitis and thrombophlebitis of unspecified femoral v	IMO	GHS-PACDC	DX_LEGACY	ACTIVE	N	1900-01-01	9999-12-31	2015-07-10
Diagnosis: ICD10 Code, I00-I99, Define	I00-I99	Diseases of the circulatory system	1185856	Phlebitis and thrombophlebitis of unspecified femoral vein (HCC)	ICD10CM	1	I80.10		Phlebitis and thrombophlebitis of unspecified femoral v	IMO	GHS-PACDC	IMO_2016_R	ACTIVE	N	1900-01-01	9999-12-31	2016-06-02
Diagnosis: ICD10 Code, I00-I99, Define	I00-I99	Diseases of the circulatory system	722798	Thrombophlebitis of deep femoral vein, unspecified laterality (HCC)	ICD10CM	1	I80.10		Phlebitis and thrombophlebitis of unspecified femoral v	IMO	GHS-PACDC	IMO_2015_R	ACTIVE	N	1900-01-01	9999-12-31	2015-07-10
Diagnosis: ICD10 Code, I00-I99, Define	I00-I99	Diseases of the circulatory system	812537	Phlebitis of the femoral vein, unspecified laterality (HCC)	ICD10CM	1	I80.10		Phlebitis and thrombophlebitis of unspecified femoral v	IMO	GHS-PACDC	IMO_2015_R	ACTIVE	N	1900-01-01	9999-12-31	2015-07-10
Diagnosis: ICD10 Code, I00-I99, Define	I00-I99	Diseases of the circulatory system	244534	Phlebitis of femoral vein of right leg (HCC)	ICD10CM	1	I80.11		Phlebitis and thrombophlebitis of right femoral vein	IMO	GHS-PACDC	DX_LEGACY	ACTIVE	N	1900-01-01	9999-12-31	2015-07-10
Diagnosis: ICD10 Code, I00-I99, Define	I00-I99	Diseases of the circulatory system	245377	Thrombophlebitis of leg, right, deep, femoral vein (HCC)	ICD10CM	1	I80.11		Phlebitis and thrombophlebitis of right femoral vein	IMO	GHS-PACDC	DX_LEGACY	ACTIVE	N	1900-01-01	9999-12-31	2015-07-10
Diagnosis: ICD10 Code, I00-I99, Define	I00-I99	Diseases of the circulatory system	751770	Thrombophlebitis femoral vein, right (HCC)	ICD10CM	1	I80.11		Phlebitis and thrombophlebitis of right femoral vein	IMO	GHS-PACDC	IMO_2015_R	ACTIVE	N	1900-01-01	9999-12-31	2015-07-10
Diagnosis: ICD10 Code, I00-I99, Define	I00-I99	Diseases of the circulatory system	845271	Phlebitis and thrombophlebitis of femoral vein, left (HCC)	ICD10CM	1	I80.12		Phlebitis and thrombophlebitis of left femoral vein	IMO	GHS-PACDC	IMO_2015_R	ACTIVE	N	1900-01-01	9999-12-31	2015-07-10
Diagnosis: ICD10 Code, I00-I99, Define	I00-I99	Diseases of the circulatory system	939639	Thrombophlebitis of deep femoral vein, bilateral (HCC)	ICD10CM	1	I80.13		Phlebitis and thrombophlebitis of femoral vein, bilateral	IMO	GHS-PACDC	IMO_2015_R	ACTIVE	N	1900-01-01	9999-12-31	2015-07-10
Diagnosis: ICD10 Code, I00-I99, Define	I00-I99	Diseases of the circulatory system	518760	Phlebitis of deep vein of right thigh (HCC)	ICD10CM	1	I80.201		Phlebitis and thrombophlebitis of unspecified deep vess	IMO	GHS-PACDC	IMO_2015_R	ACTIVE	N	1900-01-01	9999-12-31	2015-07-10
Diagnosis: ICD10 Code, I00-I99, Define	I00-I99	Diseases of the circulatory system	448561	Thrombophlebitis of deep veins of right lower extremity (HCC)	ICD10CM	1	I80.201		Phlebitis and thrombophlebitis of unspecified deep vess	IMO	GHS-PACDC	IMO_2015_R	ACTIVE	N	1900-01-01	9999-12-31	2015-07-10
Diagnosis: ICD10 Code, I00-I99, Define	I00-I99	Diseases of the circulatory system	1092693	Deep vein thrombophlebitis of lower leg, left (HCC)	ICD10CM	1	I80.202		Phlebitis and thrombophlebitis of unspecified deep vess	IMO	GHS-PACDC	IMO_2015_R	ACTIVE	N	1900-01-01	9999-12-31	2015-07-10
Diagnosis: ICD10 Code, I00-I99, Define	I00-I99	Diseases of the circulatory system	516437	Thrombophlebitis of deep vein of left calf (HCC)	ICD10CM	1	I80.202		Phlebitis and thrombophlebitis of unspecified deep vess	IMO	GHS-PACDC	IMO_2015_R	ACTIVE	N	2017-03-31	9999-12-31	2017-04-14
Diagnosis: ICD10 Code, I00-I99, Define	I00-I99	Diseases of the circulatory system	1268830	Phlebitis of deep vein of left extremity	ICD10CM	1	I80.202		Phlebitis and thrombophlebitis of unspecified deep vess	IMO	GHS-PACDC	IMO_2017_R	DELETED	Y	1900-01-01	2017-04-27	2016-09-30
Diagnosis: ICD10 Code, I00-I99, Define	I00-I99	Diseases of the circulatory system	1091803	Deep vein phlebitis of lower leg, left (HCC)	ICD10CM	1	I80.202		Phlebitis and thrombophlebitis of unspecified deep vess	IMO	GHS-PACDC	IMO_2015_R	ACTIVE	N	1900-01-01	9999-12-31	2015-07-10
Diagnosis: ICD10 Code, I00-I99, Define	I00-I99	Diseases of the circulatory system	239557	Deep phlebitis of left leg (HCC)	ICD10CM	1	I80.202		Phlebitis and thrombophlebitis of unspecified deep vess	IMO	GHS-PACDC	DX_LEGACY	ACTIVE	N	1900-01-01	9999-12-31	2015-07-10
Diagnosis: ICD10 Code, I00-I99, Define	I00-I99	Diseases of the circulatory system	1050028	Phlebitis of deep vein, unspecified laterality (HCC)	ICD10CM	1	I80.209		Phlebitis and thrombophlebitis of unspecified deep vess	IMO	GHS-PACDC	IMO_2015_R	ACTIVE	N	1900-01-01	9999-12-31	2015-07-10
Diagnosis: ICD10 Code, I00-I99, Define	I00-I99	Diseases of the circulatory system	826995	Phlebitis of deep veins of lower extremity, unspecified laterality (HCC)	ICD10CM	1	I80.209		Phlebitis and thrombophlebitis of unspecified deep vess	IMO	GHS-PACDC	IMO_2015_R	ACTIVE	N	1900-01-01	9999-12-31	2015-07-10
Diagnosis: ICD10 Code, I00-I99, Define	I00-I99	Diseases of the circulatory system	731486	Iliac thrombophlebitis, right (HCC)	ICD10CM	1	I80.211		Phlebitis and thrombophlebitis of right iliac vein	IMO	GHS-PACDC	IMO_2015_R	ACTIVE	N	1900-01-01	9999-12-31	2015-07-10
Diagnosis: ICD10 Code, I00-I99, Define	I00-I99	Diseases of the circulatory system	949584	Thrombophlebitis of common iliac vein, left (HCC)	ICD10CM	1	I80.212		Phlebitis and thrombophlebitis of left iliac vein	IMO	GHS-PACDC	IMO_2015_R	ACTIVE	N	1900-01-01	9999-12-31	2015-07-10
Diagnosis: ICD10 Code, I00-I99, Define	I00-I99	Diseases of the circulatory system	472856	Thrombophlebitis of left internal iliac vein (HCC)	ICD10CM	1	I80.212		Phlebitis and thrombophlebitis of left iliac vein	IMO	GHS-PACDC	IMO_2015_R	ACTIVE	N	1900-01-01	9999-12-31	2015-07-10
Diagnosis: ICD10 Code, I00-I99, Define	I00-I99	Diseases of the circulatory system	472554	Phlebitis of left common iliac vein (HCC)	ICD10CM	1	I80.212		Phlebitis and thrombophlebitis of left iliac vein	IMO	GHS-PACDC	IMO_2015_R	ACTIVE	N	1900-01-01	9999-12-31	2015-07-10
Diagnosis: ICD10 Code, I00-I99, Define	I00-I99	Diseases of the circulatory system	947463	Phlebitis of external iliac vein, left (HCC)	ICD10CM	1	I80.212		Phlebitis and thrombophlebitis of left iliac vein	IMO	GHS-PACDC	IMO_2015_R	ACTIVE	N	1900-01-01	9999-12-31	2015-07-10
Diagnosis: ICD10 Code, I00-I99, Define	I00-I99	Diseases of the circulatory system	951445	Phlebitis of external iliac vein, bilateral (HCC)	ICD10CM	1	I80.213		Phlebitis and thrombophlebitis of iliac vein, bilateral	IMO	GHS-PACDC	IMO_2015_R	ACTIVE	N	1900-01-01	9999-12-31	2015-07-10
Diagnosis: ICD10 Code, I00-I99, Define	I00-I99	Diseases of the circulatory system	946064	Thrombophlebitis of external iliac vein, bilateral (HCC)	ICD10CM	1	I80.213		Phlebitis and thrombophlebitis of iliac vein, bilateral	IMO	GHS-PACDC	IMO_2015_R	ACTIVE	N	1900-01-01	9999-12-31	2015-07-10
Diagnosis: ICD10 Code, I00-I99, Define	I00-I99	Diseases of the circulatory system	633407	Phlebitis and thrombophlebitis of iliac vein, bilateral (HCC)	ICD10CM	1	I80.213		Phlebitis and thrombophlebitis of iliac vein, bilateral	IMO	GHS-PACDC	IMO_2015_R	ACTIVE	N	1900-01-01	9999-12-31	2015-07-10
Diagnosis: ICD10 Code, I00-I99, Define	I00-I99	Diseases of the circulatory system	1356453	Phlebitis or thrombophlebitis of both iliac veins (HCC)	ICD10CM	1	I80.213		Phlebitis and thrombophlebitis of iliac vein, bilateral	IMO	GHS-PACDC	IMO_2018_R	ACTIVE	N	1900-01-01	9999-12-31	2017-10-30
Diagnosis: ICD10 Code, I00-I99, Define	I00-I99	Diseases of the circulatory system	408151	Thrombophlebitis of external iliac vein (HCC)	ICD10CM	1	I80.219		Phlebitis and thrombophlebitis of unspecified iliac vein	IMO	GHS-PACDC	DX_LEGACY	ACTIVE	N	1900-01-01	9999-12-31	2015-07-10
Diagnosis: ICD10 Code, I00-I99, Define	I00-I99	Diseases of the circulatory system	22233	Thrombophlebitis of iliac vein (HCC)	ICD10CM	1	I80.219		Phlebitis and thrombophlebitis of unspecified iliac vein	IMO	GHS-PACDC	DX_LEGACY	ACTIVE	N	1900-01-01	9999-12-31	2015-07-10
Diagnosis: ICD10 Code, I00-I99, Define	I00-I99	Diseases of the circulatory system	943794	Thrombophlebitis of common iliac vein, unspecified laterality (HCC)	ICD10CM	1	I80.219		Phlebitis and thrombophlebitis of unspecified iliac vein	IMO	GHS-PACDC	IMO_2015_R	ACTIVE	N	1900-01-01	9999-12-31	2015-07-10
Diagnosis: ICD10 Code, I00-I99, Define	I00-I99	Diseases of the circulatory system	595935	Phlebitis and thrombophlebitis of right popliteal vein	ICD10CM	1	I80.221		Phlebitis and thrombophlebitis of right popliteal vein	IMO	GHS-PACDC	IMO_2015_R	ACTIVE	Y	1900-01-01	9999-12-31	2015-07-10
Diagnosis: ICD10 Code, I00-I99, Define	I00-I99	Diseases of the circulatory system	937277	Phlebitis of popliteal vein, bilateral (HCC)	ICD10CM	1	I80.223		Phlebitis and thrombophlebitis of popliteal vein, bilatera	IMO	GHS-PACDC	IMO_2015_R	ACTIVE	N	1900-01-01	9999-12-31	2015-07-10
Diagnosis: ICD10 Code, I00-I99, Define	I00-I99	Diseases of the circulatory system	596962	Phlebitis and thrombophlebitis of unspecified popliteal vein	ICD10CM	1	I80.229		Phlebitis and thrombophlebitis of unspecified popliteal	IMO	GHS-PACDC	IMO_2015_R	ACTIVE	Y	1900-01-01	9999-12-31	2015-07-10
Diagnosis: ICD10 Code, I00-I99, Define	I00-I99	Diseases of the circulatory system	759719	Thrombophlebitis of popliteal vein, unspecified laterality (HCC)	ICD10CM	1	I80.229		Phlebitis and thrombophlebitis of unspecified popliteal	IMO	GHS-PACDC	IMO_2015_R	ACTIVE	N	1900-01-01	9999-12-31	2015-07-10
Diagnosis: ICD10 Code, I00-I99, Define	I00-I99	Diseases of the circulatory system	733157	Tibial vein thrombosis, right (HCC)	ICD10CM	1	I80.231		Phlebitis and thrombophlebitis of right tibial vein	IMO	GHS-PACDC	IMO_2015_R	ACTIVE	N	1900-01-01	9999-12-31	2015-07-10
Diagnosis: ICD10 Code, I00-I99, Define	I00-I99	Diseases of the circulatory system	946894	Thrombophlebitis of posterior tibial vein, right (HCC)	ICD10CM	1	I80.231		Phlebitis and thrombophlebitis of right tibial vein	IMO	GHS-PACDC	IMO_2015_R	ACTIVE	N	1900-01-01	9999-12-31	2015-07-10
Diagnosis: ICD10 Code, I00-I99, Define	I00-I99	Diseases of the circulatory system	946231	Phlebitis of anterior tibial vein, left (HCC)	ICD10CM	1	I80.232		Phlebitis and thrombophlebitis of left tibial vein	IMO	GHS-PACDC	IMO_2015_R	ACTIVE	N	1900-01-01	9999-12-31	2015-07-10
Diagnosis: ICD10 Code, I00-I99, Define	I00-I99	Diseases of the circulatory system	473477	Phlebitis of left anterior tibial vein (HCC)	ICD10CM	1	I80.232		Phlebitis and thrombophlebitis of left tibial vein	IMO	GHS-PACDC	IMO_2015_R	ACTIVE	N	1900-01-01	9999-12-31	2015-07-10
Diagnosis: ICD10 Code, I00-I99, Define	I00-I99	Diseases of the circulatory system	948574	Phlebitis of posterior tibial vein, left (HCC)	ICD10CM	1	I80.232		Phlebitis and thrombophlebitis of left tibial vein	IMO	GHS-PACDC	IMO_2015_R	ACTIVE	N	1900-01-01	9999-12-31	2015-07-10
Diagnosis: ICD10 Code, I00-I99, Define	I00-I99	Diseases of the circulatory system	596915	Phlebitis and thrombophlebitis of left tibial vein	ICD10CM	1	I80.232		Phlebitis and thrombophlebitis of left tibial vein	IMO	GHS-PACDC	IMO_2015_R	ACTIVE	Y	1900-01-01	9999-12-31	2015-07-10
Diagnosis: ICD10 Code, I00-I99, Define	I00-I99	Diseases of the circulatory system	950415	Thrombophlebitis of anterior tibial vein, bilateral (HCC)	ICD10CM	1	I80.233		Phlebitis and thrombophlebitis of tibial vein, bilateral	IMO	GHS-PACDC	IMO_2015_R	ACTIVE	N	1900-01-01	9999-12-31	2015-07-10
Diagnosis: ICD10 Code, I00-I99, Define	I00-I99	Diseases of the circulatory system	941764	Thrombophlebitis of dorsalis pedis vein, bilateral (HCC)	ICD10CM	1	I80.293		Phlebitis and thrombophlebitis of other deep vessels of	IMO	GHS-PACDC	IMO_2015_R	ACTIVE	N	1900-01-01	9999-12-31	2015-07-10
Diagnosis: ICD10 Code, I00-I99, Define	I00-I99	Diseases of the circulatory system	1365898	Phlebitis and thombophlb of deep vessels of low extrm, bi (HCC)	ICD10CM	1	I80.293		Phlebitis and thrombophlebitis of other deep vessels of	IMO	GHS-PACDC	IMO_2018_R	ACTIVE	N	1900-01-01	9999-12-31	2017-10-30
Diagnosis: ICD10 Code, I00-I99, Define	I00-I99	Diseases of the circulatory system	517216	Thrombophlebitis of deep vein of both calves (HCC)	ICD10CM	1	I80.293		Phlebitis and thrombophlebitis of other deep vessels of	IMO	GHS-PACDC	IMO_2015_R	ACTIVE	N	1900-01-01	2017-04-27	2015-07-10
Diagnosis: ICD10 Code, I00-I99, Define	I00-I99	Diseases of the circulatory system	236535	Thrombophlebitis of leg (HCC)	ICD10CM	1	I80.299		Phlebitis and thrombophlebitis of other deep vessels of	IMO	GHS-PACDC	DX_LEGACY	ACTIVE	N	2016-04-10	9999-12-31	2015-07-10
Diagnosis: ICD10 Code, I00-I99, Define	I00-I99	Diseases of the circulatory system	245381	Thrombophlebitis of leg, right (HCC)	ICD10CM	1	I80.3		Phlebitis and thrombophlebitis of lower extremities, uns	IMO	GHS-PACDC	DX_LEGACY	ACTIVE	N	1900-01-01	2016-05-07	2015-07-10
Diagnosis: ICD10 Code, I00-I99, Define	I00-I99	Diseases of the circulatory system	244538	Phlebitis of right leg	ICD10CM	1	I80.3		Phlebitis and thrombophlebitis of lower extremities, uns	IMO	GHS-PACDC	DX_LEGACY	ACTIVE	N	1900-01-01	9999-12-31	2015-07-10
Diagnosis: ICD10 Code, I00-I99, Define	I00-I99	Diseases of the circulatory system	1132907	Deep vein thrombosis (DVT) of radial vein (HCC)	ICD10CM	1	I80.8		Phlebitis and thrombophlebitis of other sites	IMO	GHS-PACDC	IMO_2016_R	ACTIVE	N	1900-01-01	2016-10-14	2016-06-02
Diagnosis: ICD10 Code, I00-I99, Define	I00-I99	Diseases of the circulatory system	481054	Phlebitis of superficial vein of upper extremity	ICD10CM	1	I80.8		Phlebitis and thrombophlebitis of other sites	IMO	GHS-PACDC	IMO_2015_R	ACTIVE	N	1900-01-01	9999-12-31	2015-07-10
Diagnosis: ICD10 Code, I00-I99, Define	I00-I99	Diseases of the circulatory system	523366	Thrombophlebitis of right upper extremity	ICD10CM	1	I80.8		Phlebitis and thrombophlebitis of other sites	IMO	GHS-PACDC	IMO_2015_R	ACTIVE	N	1900-01-01	9999-12-31	2015-07-10
Diagnosis: ICD10 Code, I00-I99, Define	I00-I99	Diseases of the circulatory system	421952	Phlebitis and thrombophlebitis of other sites	ICD10CM	1	I80.8		Phlebitis and thrombophlebitis of other sites	IMO	GHS-PACDC	IMO_2015_R	DELETED	Y	1900-01-01	9999-12-31	2015-07-10
Diagnosis: ICD10 Code, I00-I99, Define	I00-I99	Diseases of the circulatory system	1107248	Superficial thrombophlebitis of right upper extremity	ICD10CM	1	I80.8		Phlebitis and thrombophlebitis of other sites	IMO	GHS-PACDC	IMO_2016_R	ACTIVE	N	1900-01-01	9999-12-31	2016-06-02
Diagnosis: ICD10 Code, I00-I99, Define	I00-I99	Diseases of the circulatory system	385796	DVT of axillary vein, acute left (HCC)	ICD10CM	1	I80.8		Phlebitis and thrombophlebitis of other sites	IMO	GHS-PACDC	DX_LEGACY	ACTIVE	N	1900-01-01	2016-05-07	2015-07-10
Diagnosis: ICD10 Code, I00-I99, Define	I00-I99	Diseases of the circulatory system	1107229	Superficial thrombophlebitis of left upper extremity	ICD10CM	1	I80.8		Phlebitis and thrombophlebitis of other sites	IMO	GHS-PACDC	IMO_2016_R	ACTIVE	N	1900-01-01	9999-12-31	2016-06-02
Diagnosis: ICD10 Code, I00-I99, Define	I00-I99	Diseases of the circulatory system	1241527	Superficial thrombophlebitis of upper extremity, unspecified laterality	ICD10CM	1	I80.8		Phlebitis and thrombophlebitis of other sites	IMO	GHS-PACDC	IMO_2016_R	ACTIVE	N	1900-01-01	9999-12-31	2016-06-02
Diagnosis: ICD10 Code, I00-I99, Define	I00-I99	Diseases of the circulatory system	520964	Superficial thrombophlebitis during pregnancy in third trimester	ICD10CM	2	I80.9	O22.23,I80.9	Phlebitis and thrombophlebitis of unspecified site	IMO	GHS-PACDC	IMO_2015_R	ACTIVE	N	1900-01-01	2016-10-14	2015-07-10
Diagnosis: ICD10 Code, I00-I99, Define	I00-I99	Diseases of the circulatory system	527080	Thrombosis affecting pregnancy in second trimester, antepartum	ICD10CM	2	I80.9	O22.22,I80.9	Phlebitis and thrombophlebitis of unspecified site	IMO	GHS-PACDC	IMO_2015_R	ACTIVE	N	1900-01-01	2017-10-14	2015-07-10
Diagnosis: ICD10 Code, I00-I99, Define	I00-I99	Diseases of the circulatory system	649525	Thrombosis/phlebitis, obstetric, second trimester	ICD10CM	2	I80.9	O22.22,I80.9	Phlebitis and thrombophlebitis of unspecified site	IMO	GHS-PACDC	IMO_2015_R	DELETED	Y	1900-01-01	9999-12-31	2015-07-10
Diagnosis: ICD10 Code, I00-I99, Define	I00-I99	Diseases of the circulatory system	954226	Thrombophlebitis during pregnancy, second trimester	ICD10CM	2	I80.9	O22.22,I80.9	Phlebitis and thrombophlebitis of unspecified site	IMO	GHS-PACDC	IMO_2015_R	ACTIVE	N	1900-01-01	2016-10-14	2015-07-10
Diagnosis: ICD10 Code, I00-I99, Define	I00-I99	Diseases of the circulatory system	687445	Thrombophlebitis, obstetric, superficial, third trimester	ICD10CM	2	I80.9	O22.23,I80.9	Phlebitis and thrombophlebitis of unspecified site	IMO	GHS-PACDC	IMO_2015_R	DELETED	Y	1900-01-01	9999-12-31	2015-07-10
Diagnosis: ICD10 Code, I00-I99, Define	I00-I99	Diseases of the circulatory system	5276	Phlebitis and thrombophlebitis	ICD10CM	1	I80.9		Phlebitis and thrombophlebitis of unspecified site	IMO	GHS-PACDC	DX_LEGACY	ACTIVE	N	1900-01-01	9999-12-31	2015-07-10
Diagnosis: ICD10 Code, I00-I99, Define	I00-I99	Diseases of the circulatory system	22210	Endophlebitis	ICD10CM	1	I80.9		Phlebitis and thrombophlebitis of unspecified site	IMO	GHS-PACDC	DX_LEGACY	ACTIVE	N	1900-01-01	9999-12-31	2015-07-10
Diagnosis: ICD10 Code, I00-I99, Define	I00-I99	Diseases of the circulatory system	49350	Thrombosis, hepatic vein (HCC)	ICD10CM	1	I82.0		Budd-Chiari syndrome	IMO	GHS-PACDC	DX_LEGACY	ACTIVE	N	1900-01-01	9999-12-31	2015-07-10
Diagnosis: ICD10 Code, I00-I99, Define	I00-I99	Diseases of the circulatory system	533710	Budd-Chiari syndrome	ICD10CM	1	I82.0		Budd-Chiari syndrome	IMO	GHS-PACDC	IMO_2015_R	ACTIVE	Y	1900-01-01	9999-12-31	2015-07-10
Diagnosis: ICD10 Code, I00-I99, Define	I00-I99	Diseases of the circulatory system	533717	Thrombophlebitis migrans	ICD10CM	1	I82.1		Thrombophlebitis migrans	IMO	GHS-PACDC	IMO_2015_R	ACTIVE	Y	1900-01-01	9999-12-31	2015-07-10
Diagnosis: ICD10 Code, I00-I99, Define	I00-I99	Diseases of the circulatory system	587951	Chronic embolism and thrombosis of superior vena cava	ICD10CM	1	I82.211		Chronic embolism and thrombosis of superior vena cava	IMO	GHS-PACDC	IMO_2015_R	ACTIVE	Y	1900-01-01	9999-12-31	2015-07-10
Diagnosis: ICD10 Code, I00-I99, Define	I00-I99	Diseases of the circulatory system	1124211	Neoplasm of right kidney with thrombus of inferior vena cava (HCC)	ICD10CM	2	I82.220	D49.5,I82.220	Acute embolism and thrombosis of inferior vena cava	IMO	GHS-PACDC	IMO_2016_R	ACTIVE	N	1900-01-01	2016-10-14	2016-06-02
Diagnosis: ICD10 Code, I00-I99, Define	I00-I99	Diseases of the circulatory system	589693	Acute embolism and thrombosis of inferior vena cava	ICD10CM	1	I82.220		Acute embolism and thrombosis of inferior vena cava	IMO	GHS-PACDC	IMO_2015_R	ACTIVE	Y	1900-01-01	9999-12-31	2015-07-10
Diagnosis: ICD10 Code, I00-I99, Define	I00-I99	Diseases of the circulatory system	1132514	Acute embolism from vein of thorax (HCC)	ICD10CM	1	I82.290		Acute embolism and thrombosis of other thoracic veins	IMO	GHS-PACDC	IMO_2016_R	ACTIVE	N	1900-01-01	9999-12-31	2016-06-02
Diagnosis: ICD10 Code, I00-I99, Define	I00-I99	Diseases of the circulatory system	213905	Thrombosis of superior vena cava (HCC)	ICD10CM	1	I82.290		Acute embolism and thrombosis of other thoracic veins	IMO	GHS-PACDC	DX_LEGACY	ACTIVE	N	1900-01-01	2016-05-07	2015-07-10
Diagnosis: ICD10 Code, I00-I99, Define	I00-I99	Diseases of the circulatory system	215797	Acute thrombosis of brachiocephalic (innominate) vein (HCC)	ICD10CM	1	I82.290		Acute embolism and thrombosis of other thoracic veins	IMO	GHS-PACDC	DX_LEGACY	ACTIVE	N	1900-01-01	9999-12-31	2015-07-10
Diagnosis: ICD10 Code, I00-I99, Define	I00-I99	Diseases of the circulatory system	215309	Acute embolism of thoracic vein	ICD10CM	1	I82.290		Acute embolism and thrombosis of other thoracic veins	IMO	GHS-PACDC	DX_LEGACY	DELETED	Y	1900-01-01	9999-12-31	2015-07-10
Diagnosis: ICD10 Code, I00-I99, Define	I00-I99	Diseases of the circulatory system	1132513	Chronic embolism from vein of thorax (HCC)	ICD10CM	1	I82.291		Chronic embolism and thrombosis of other thoracic vein	IMO	GHS-PACDC	IMO_2016_R	ACTIVE	N	1900-01-01	9999-12-31	2016-06-02
Diagnosis: ICD10 Code, I00-I99, Define	I00-I99	Diseases of the circulatory system	769682	Recurrent deep vein thrombosis, right (HCC)	ICD10CM	1	I82.401		Acute embolism and thrombosis of unspecified deep vei	IMO	GHS-PACDC	IMO_2015_R	ACTIVE	N	1900-01-01	9999-12-31	2015-07-10
Diagnosis: ICD10 Code, I00-I99, Define	I00-I99	Diseases of the circulatory system	1083735	Deep vein thrombosis of lower extremity related to air travel, left (HCC)	ICD10CM	1	I82.402		Acute embolism and thrombosis of unspecified deep vei	IMO	GHS-PACDC	IMO_2015_R	ACTIVE	N	1900-01-01	9999-12-31	2015-07-10
Diagnosis: ICD10 Code, I00-I99, Define	I00-I99	Diseases of the circulatory system	949182	Suspected DVT (deep vein thrombosis), left (HCC)	ICD10CM	1	I82.402		Acute embolism and thrombosis of unspecified deep vei	IMO	GHS-PACDC	IMO_2015_R	ACTIVE	N	1900-01-01	9999-12-31	2015-07-10
Diagnosis: ICD10 Code, I00-I99, Define	I00-I99	Diseases of the circulatory system	373788	Acute deep vein thrombosis of left lower extremity (HCC)	ICD10CM	1	I82.402		Acute embolism and thrombosis of unspecified deep vei	IMO	GHS-PACDC	DX_LEGACY	ACTIVE	N	1900-01-01	9999-12-31	2015-07-10
Diagnosis: ICD10 Code, I00-I99, Define	I00-I99	Diseases of the circulatory system	851840	Acute deep venous thrombosis, left	ICD10CM	1	I82.402		Acute embolism and thrombosis of unspecified deep vei	IMO	GHS-PACDC	IMO_2015_R	DELETED	Y	1900-01-01	9999-12-31	2015-07-10
Diagnosis: ICD10 Code, I00-I99, Define	I00-I99	Diseases of the circulatory system	1134350	Encounter for follow-up of acute deep vein thrombosis (DVT) of left lower extremity	ICD10CM	1	I82.402		Acute embolism and thrombosis of unspecified deep vei	IMO	GHS-PACDC	IMO_2016_R	ACTIVE	N	1900-01-01	2017-04-27	2016-06-02
Diagnosis: ICD10 Code, I00-I99, Define	I00-I99	Diseases of the circulatory system	835085	Deep vein thrombosis, left	ICD10CM	1	I82.402		Acute embolism and thrombosis of unspecified deep vei	IMO	GHS-PACDC	IMO_2015_R	DELETED	Y	1900-01-01	9999-12-31	2015-07-10
Diagnosis: ICD10 Code, I00-I99, Define	I00-I99	Diseases of the circulatory system	801925	Acute DVT (deep venous thrombosis), left	ICD10CM	1	I82.402		Acute embolism and thrombosis of unspecified deep vei	IMO	GHS-PACDC	IMO_2015_R	DELETED	Y	1900-01-01	9999-12-31	2015-07-10
Diagnosis: ICD10 Code, I00-I99, Define	I00-I99	Diseases of the circulatory system	820156	Recurrent acute deep vein thrombosis of lower extremity, left (HCC)	ICD10CM	1	I82.402		Acute embolism and thrombosis of unspecified deep vei	IMO	GHS-PACDC	IMO_2015_R	ACTIVE	N	1900-01-01	9999-12-31	2015-07-10
Diagnosis: ICD10 Code, I00-I99, Define	I00-I99	Diseases of the circulatory system	949605	Deep vein thrombosis of leg related to air travel, left (HCC)	ICD10CM	1	I82.402		Acute embolism and thrombosis of unspecified deep vei	IMO	GHS-PACDC	IMO_2015_R	ACTIVE	N	1900-01-01	9999-12-31	2015-07-10
Diagnosis: ICD10 Code, I00-I99, Define	I00-I99	Diseases of the circulatory system	753032	Progression of deep vein thrombosis, left (HCC)	ICD10CM	1	I82.402		Acute embolism and thrombosis of unspecified deep vei	IMO	GHS-PACDC	IMO_2015_R	ACTIVE	N	1900-01-01	9999-12-31	2015-07-10
Diagnosis: ICD10 Code, I00-I99, Define	I00-I99	Diseases of the circulatory system	458445	Deep vein thrombosis of left leg related to intravenous drug use (HCC)	ICD10CM	1	I82.402		Acute embolism and thrombosis of unspecified deep vei	IMO	GHS-PACDC	IMO_2015_R	ACTIVE	N	1900-01-01	9999-12-31	2015-07-10
Diagnosis: ICD10 Code, I00-I99, Define	I00-I99	Diseases of the circulatory system	466822	Recurrent acute deep vein thrombosis of both lower extremities (HCC)	ICD10CM	1	I82.403		Acute embolism and thrombosis of unspecified deep vei	IMO	GHS-PACDC	IMO_2015_R	ACTIVE	N	1900-01-01	9999-12-31	2015-07-10
Diagnosis: ICD10 Code, I00-I99, Define	I00-I99	Diseases of the circulatory system	448654	Encounter for follow-up of deep vein thrombosis of both lower extremities	ICD10CM	1	I82.403		Acute embolism and thrombosis of unspecified deep vei	IMO	GHS-PACDC	IMO_2015_R	ACTIVE	N	1900-01-01	2017-04-27	2015-07-10
Diagnosis: ICD10 Code, I00-I99, Define	I00-I99	Diseases of the circulatory system	1184507	Acute embolism and thrombosis of unspecified deep veins of lower extremity, bilateral (HCC)	ICD10CM	1	I82.403		Acute embolism and thrombosis of unspecified deep vei	IMO	GHS-PACDC	IMO_2016_R	ACTIVE	N	1900-01-01	9999-12-31	2016-06-02
Diagnosis: ICD10 Code, I00-I99, Define	I00-I99	Diseases of the circulatory system	317156	Bilateral deep vein thromboses (HCC)	ICD10CM	1	I82.403		Acute embolism and thrombosis of unspecified deep vei	IMO	GHS-PACDC	DX_LEGACY	ACTIVE	N	1900-01-01	9999-12-31	2015-07-10
Diagnosis: ICD10 Code, I00-I99, Define	I00-I99	Diseases of the circulatory system	1132961	Recurrent acute deep vein thrombosis (DVT) of both lower extremities (HCC)	ICD10CM	1	I82.403		Acute embolism and thrombosis of unspecified deep vei	IMO	GHS-PACDC	IMO_2016_R	ACTIVE	N	1900-01-01	9999-12-31	2016-06-02
Diagnosis: ICD10 Code, I00-I99, Define	I00-I99	Diseases of the circulatory system	940421	Thromboembolism of deep lower extremity veins, bilateral (HCC)	ICD10CM	1	I82.403		Acute embolism and thrombosis of unspecified deep vei	IMO	GHS-PACDC	IMO_2015_R	ACTIVE	N	1900-01-01	9999-12-31	2015-07-10
Diagnosis: ICD10 Code, I00-I99, Define	I00-I99	Diseases of the circulatory system	377292	Encounter for monitoring enoxaparin therapy for acute deep vein thrombosis of lower extremit	ICD10CM	3	I82.409	Z51.81,Z79.01,I82.409	Acute embolism and thrombosis of unspecified deep vei	IMO	GHS-PACDC	DX_LEGACY	ACTIVE	N	1900-01-01	9999-12-31	2015-07-10

Diagnosis: ICD10 Code, I00-I99, Define	I00-I99	Diseases of the circulatory system	674021	Maternal DVT (deep vein thrombosis), current pregnancy, unspecified trimester (HCC)	ICD10CM	2	I82.409	O22.30,I82.409	Acute embolism and thrombosis of unspecified deep vei	IMO	GHS-PACDC	IMO_2015_R	ACTIVE	N	2016-04-10	9999-12-31	2015-07-10
Diagnosis: ICD10 Code, I00-I99, Define	I00-I99	Diseases of the circulatory system	373613	DVT, recurrent, lower extremity, acute (HCC)	ICD10CM	1	I82.409		Acute embolism and thrombosis of unspecified deep vei	IMO	GHS-PACDC	DX_LEGACY	ACTIVE	N	1900-01-01	9999-12-31	2015-07-10
Diagnosis: ICD10 Code, I00-I99, Define	I00-I99	Diseases of the circulatory system	1415569	Postoperative acute deep vein thrombosis (DVT) of lower extremity, initial encounter (HCC)	ICD10CM	2	I82.409	T81.72XA,I82.409	Acute embolism and thrombosis of unspecified deep vei	IMO	GHS-PACDC	IMO_2018_R	ACTIVE	N	1900-01-01	9999-12-31	2017-10-30
Diagnosis: ICD10 Code, I00-I99, Define	I00-I99	Diseases of the circulatory system	718638	Postoperative acute deep vein thrombosis of lower extremity, sequela	ICD10CM	2	I82.409	T81.72XS,I82.409	Acute embolism and thrombosis of unspecified deep vei	IMO	GHS-PACDC	IMO_2015_R	ACTIVE	N	2017-09-17	9999-12-31	2017-10-30
Diagnosis: ICD10 Code, I00-I99, Define	I00-I99	Diseases of the circulatory system	210703	Thrombophlebitis, deep, antepartum (HCC)	ICD10CM	2	I82.409	O22.30,I82.409	Acute embolism and thrombosis of unspecified deep vei	IMO	GHS-PACDC	DX_LEGACY	ACTIVE	N	1900-01-01	9999-12-31	2015-07-10
Diagnosis: ICD10 Code, I00-I99, Define	I00-I99	Diseases of the circulatory system	1132782	Acute deep vein thrombosis (DVT) of lower extremity (HCC)	ICD10CM	1	I82.409		Acute embolism and thrombosis of unspecified deep vei	IMO	GHS-PACDC	IMO_2016_R	ACTIVE	N	1900-01-01	9999-12-31	2016-06-02
Diagnosis: ICD10 Code, I00-I99, Define	I00-I99	Diseases of the circulatory system	483758	Deep vein thrombosis associated with coronary artery bypass graft surgery (HCC)	ICD10CM	1	I82.409	I82.409,I25.810	Acute embolism and thrombosis of unspecified deep vei	IMO	GHS-PACDC	IMO_2015_R	ACTIVE	N	1900-01-01	2016-05-07	2015-07-10
Diagnosis: ICD10 Code, I00-I99, Define	I00-I99	Diseases of the circulatory system	176454	Maternal deep vein thrombosis, current pregnancy (HCC)	ICD10CM	2	I82.409	O22.30,I82.409	Acute embolism and thrombosis of unspecified deep vei	IMO	GHS-PACDC	DX_LEGACY	ACTIVE	N	1900-01-01	9999-12-31	2015-07-10
Diagnosis: ICD10 Code, I00-I99, Define	I00-I99	Diseases of the circulatory system	254843	Posttraumatic deep vein thrombosis (HCC)	ICD10CM	1	I82.409		Acute embolism and thrombosis of unspecified deep vei	IMO	GHS-PACDC	DX_LEGACY	ACTIVE	N	1900-01-01	9999-12-31	2015-07-10
Diagnosis: ICD10 Code, I00-I99, Define	I00-I99	Diseases of the circulatory system	292557	Deep phlebothrombosis in pregnancy in first trimester	ICD10CM	2	I82.409	O22.31,I82.409	Acute embolism and thrombosis of unspecified deep vei	IMO	GHS-PACDC	DX_LEGACY	ACTIVE	N	1900-01-01	2016-10-14	2015-07-10
Diagnosis: ICD10 Code, I00-I99, Define	I00-I99	Diseases of the circulatory system	718732	Postoperative acute deep vein thrombosis of lower extremity, subsequent encounter	ICD10CM	2	I82.409	T81.72XD,I82.409	Acute embolism and thrombosis of unspecified deep vei	IMO	GHS-PACDC	IMO_2015_R	ACTIVE	N	2017-09-17	9999-12-31	2017-10-30
Diagnosis: ICD10 Code, I00-I99, Define	I00-I99	Diseases of the circulatory system	7649	Deep phlebothrombosis, antepartum, unspecified as to episode of care	ICD10CM	2	I82.409	O22.30,I82.409	Acute embolism and thrombosis of unspecified deep vei	IMO	GHS-PACDC	DX_LEGACY	ACTIVE	Y	1900-01-01	9999-12-31	2015-07-10
Diagnosis: ICD10 Code, I00-I99, Define	I00-I99	Diseases of the circulatory system	676125	Deep vein thrombosis complicating pregnancy, unspecified trimester	ICD10CM	2	I82.409	O22.30,I82.409	Acute embolism and thrombosis of unspecified deep vei	IMO	GHS-PACDC	IMO_2015_R	ACTIVE	N	2017-09-17	9999-12-31	2017-10-30
Diagnosis: ICD10 Code, I00-I99, Define	I00-I99	Diseases of the circulatory system	613676	Acute embolism and thrombosis of femoral vein	ICD10CM	1	I82.41		Acute embolism and thrombosis of femoral vein	IMO	GHS-PACDC	IMO_2015_R	ACTIVE	Y	1900-01-01	9999-12-31	2015-07-10
Diagnosis: ICD10 Code, I00-I99, Define	I00-I99	Diseases of the circulatory system	471769	Deep venous thrombosis of right femoral vein (HCC)	ICD10CM	1	I82.411		Acute embolism and thrombosis of right femoral vein	IMO	GHS-PACDC	IMO_2015_R	ACTIVE	N	1900-01-01	9999-12-31	2015-07-10
Diagnosis: ICD10 Code, I00-I99, Define	I00-I99	Diseases of the circulatory system	479418	Deep venous thrombosis of right profunda femoris vein (HCC)	ICD10CM	1	I82.411		Acute embolism and thrombosis of right femoral vein	IMO	GHS-PACDC	IMO_2015_R	ACTIVE	N	1900-01-01	9999-12-31	2015-07-10
Diagnosis: ICD10 Code, I00-I99, Define	I00-I99	Diseases of the circulatory system	228602	Thrombosis of left femoral vein (HCC)	ICD10CM	1	I82.412		Acute embolism and thrombosis of left femoral vein	IMO	GHS-PACDC	DX_LEGACY	ACTIVE	N	1900-01-01	9999-12-31	2015-07-10
Diagnosis: ICD10 Code, I00-I99, Define	I00-I99	Diseases of the circulatory system	1132900	Deep vein thrombosis (DVT) of femoral vein of both lower extremities (HCC)	ICD10CM	1	I82.413		Acute embolism and thrombosis of femoral vein, bilater	IMO	GHS-PACDC	IMO_2016_R	ACTIVE	N	1900-01-01	9999-12-31	2016-06-02
Diagnosis: ICD10 Code, I00-I99, Define	I00-I99	Diseases of the circulatory system	532617	Venous thromboembolism (VTE) of femoral vein (HCC)	ICD10CM	1	I82.419		Acute embolism and thrombosis of unspecified femoral	IMO	GHS-PACDC	IMO_2015_R	ACTIVE	N	1900-01-01	9999-12-31	2015-07-10
Diagnosis: ICD10 Code, I00-I99, Define	I00-I99	Diseases of the circulatory system	1225828	Acute deep vein thrombosis (DVT) of femoral vein, unspecified laterality (HCC)	ICD10CM	1	I82.419		Acute embolism and thrombosis of unspecified femoral	IMO	GHS-PACDC	IMO_2016_R	ACTIVE	N	1900-01-01	9999-12-31	2016-06-02
Diagnosis: ICD10 Code, I00-I99, Define	I00-I99	Diseases of the circulatory system	612052	Acute embolism and thrombosis of iliac vein	ICD10CM	1	I82.42		Acute embolism and thrombosis of iliac vein	IMO	GHS-PACDC	IMO_2015_R	ACTIVE	Y	1900-01-01	9999-12-31	2015-07-10
Diagnosis: ICD10 Code, I00-I99, Define	I00-I99	Diseases of the circulatory system	821428	Ileofemoral deep vein thrombosis, right (HCC)	ICD10CM	1	I82.421		Acute embolism and thrombosis of right iliac vein	IMO	GHS-PACDC	IMO_2015_R	ACTIVE	N	1900-01-01	9999-12-31	2015-07-10
Diagnosis: ICD10 Code, I00-I99, Define	I00-I99	Diseases of the circulatory system	314615	Thrombosis of right iliac vein (HCC)	ICD10CM	1	I82.421		Acute embolism and thrombosis of right iliac vein	IMO	GHS-PACDC	DX_LEGACY	ACTIVE	N	1900-01-01	9999-12-31	2015-07-10
Diagnosis: ICD10 Code, I00-I99, Define	I00-I99	Diseases of the circulatory system	1127778	Deep vein thrombosis of iliac vein of left lower extremity (HCC)	ICD10CM	1	I82.422		Acute embolism and thrombosis of left iliac vein	IMO	GHS-PACDC	IMO_2016_R	ACTIVE	N	1900-01-01	9999-12-31	2016-06-02
Diagnosis: ICD10 Code, I00-I99, Define	I00-I99	Diseases of the circulatory system	303068	Acute deep vein thrombosis of left ileofemoral vein (HCC)	ICD10CM	1	I82.422		Acute embolism and thrombosis of left iliac vein	IMO	GHS-PACDC	DX_LEGACY	ACTIVE	N	1900-01-01	9999-12-31	2015-07-10
Diagnosis: ICD10 Code, I00-I99, Define	I00-I99	Diseases of the circulatory system	1184790	Acute embolism and thrombosis of left iliac vein (HCC)	ICD10CM	1	I82.422		Acute embolism and thrombosis of left iliac vein	IMO	GHS-PACDC	IMO_2016_R	ACTIVE	N	1900-01-01	9999-12-31	2016-06-02
Diagnosis: ICD10 Code, I00-I99, Define	I00-I99	Diseases of the circulatory system	1127501	Acute bilateral deep vein thrombosis (DVT) of iliac veins of lower extremities (HCC)	ICD10CM	1	I82.423		Acute embolism and thrombosis of iliac vein, bilateral	IMO	GHS-PACDC	IMO_2016_R	ACTIVE	N	1900-01-01	9999-12-31	2016-06-02
Diagnosis: ICD10 Code, I00-I99, Define	I00-I99	Diseases of the circulatory system	1223807	Acute deep vein thrombosis (DVT) of iliac vein, unspecified laterality (HCC)	ICD10CM	1	I82.429		Acute embolism and thrombosis of unspecified iliac vein	IMO	GHS-PACDC	IMO_2016_R	ACTIVE	N	1900-01-01	9999-12-31	2016-06-02
Diagnosis: ICD10 Code, I00-I99, Define	I00-I99	Diseases of the circulatory system	141230	Deep vein thrombosis of iliac vein (HCC)	ICD10CM	1	I82.429		Acute embolism and thrombosis of unspecified iliac vein	IMO	GHS-PACDC	DX_LEGACY	ACTIVE	N	1900-01-01	9999-12-31	2015-07-10
Diagnosis: ICD10 Code, I00-I99, Define	I00-I99	Diseases of the circulatory system	516477	Thromboembolism of right popilteal vein	ICD10CM	1	I82.431		Acute embolism and thrombosis of right popliteal vein	IMO	GHS-PACDC	IMO_2015_R	DELETED	Y	1900-01-01	9999-12-31	2015-07-10
Diagnosis: ICD10 Code, I00-I99, Define	I00-I99	Diseases of the circulatory system	473316	Femoropopliteal vein thrombosis of left lower extremity (HCC)	ICD10CM	2	I82.432	I82.412,I82.432	Acute embolism and thrombosis of left popliteal vein	IMO	GHS-PACDC	IMO_2015_R	ACTIVE	N	1900-01-01	9999-12-31	2015-07-10
Diagnosis: ICD10 Code, I00-I99, Define	I00-I99	Diseases of the circulatory system	1181779	Acute embolism and thrombosis of left popliteal vein (HCC)	ICD10CM	1	I82.432		Acute embolism and thrombosis of left popliteal vein	IMO	GHS-PACDC	IMO_2016_R	ACTIVE	N	1900-01-01	9999-12-31	2016-06-02
Diagnosis: ICD10 Code, I00-I99, Define	I00-I99	Diseases of the circulatory system	928442	Femoropopliteal vein thrombosis, bilateral (HCC)	ICD10CM	2	I82.433	I82.413,I82.433	Acute embolism and thrombosis of popliteal vein, bilate	IMO	GHS-PACDC	IMO_2015_R	ACTIVE	N	1900-01-01	9999-12-31	2015-07-10
Diagnosis: ICD10 Code, I00-I99, Define	I00-I99	Diseases of the circulatory system	930855	Popliteal vein thrombosis, bilateral (HCC)	ICD10CM	1	I82.433		Acute embolism and thrombosis of popliteal vein, bilate	IMO	GHS-PACDC	IMO_2015_R	ACTIVE	N	1900-01-01	9999-12-31	2015-07-10
Diagnosis: ICD10 Code, I00-I99, Define	I00-I99	Diseases of the circulatory system	1132906	Deep vein thrombosis (DVT) of popliteal vein (HCC)	ICD10CM	1	I82.439		Acute embolism and thrombosis of unspecified poplitea	IMO	GHS-PACDC	IMO_2016_R	ACTIVE	N	1900-01-01	9999-12-31	2016-06-02
Diagnosis: ICD10 Code, I00-I99, Define	I00-I99	Diseases of the circulatory system	251479	DVT of popliteal vein (HCC)	ICD10CM	1	I82.439		Acute embolism and thrombosis of unspecified poplitea	IMO	GHS-PACDC	DX_LEGACY	ACTIVE	N	1900-01-01	9999-12-31	2015-07-10
Diagnosis: ICD10 Code, I00-I99, Define	I00-I99	Diseases of the circulatory system	522499	Acute DVT of right tibial vein (HCC)	ICD10CM	1	I82.441		Acute embolism and thrombosis of right tibial vein	IMO	GHS-PACDC	IMO_2015_R	ACTIVE	N	1900-01-01	9999-12-31	2015-07-10
Diagnosis: ICD10 Code, I00-I99, Define	I00-I99	Diseases of the circulatory system	273871	Embolism and thrombosis of tibial vein (HCC)	ICD10CM	1	I82.449		Acute embolism and thrombosis of unspecified tibial vei	IMO	GHS-PACDC	DX_LEGACY	ACTIVE	N	1900-01-01	9999-12-31	2015-07-10
Diagnosis: ICD10 Code, I00-I99, Define	I00-I99	Diseases of the circulatory system	821476	Embolism and thrombosis of tibial vein, unspecified laterality (HCC)	ICD10CM	1	I82.449		Acute embolism and thrombosis of unspecified tibial vei	IMO	GHS-PACDC	IMO_2015_R	ACTIVE	N	1900-01-01	9999-12-31	2015-07-10
Diagnosis: ICD10 Code, I00-I99, Define	I00-I99	Diseases of the circulatory system	1182383	Acute embolism and thrombosis of unspecified tibial vein (HCC)	ICD10CM	1	I82.449		Acute embolism and thrombosis of unspecified tibial vei	IMO	GHS-PACDC	IMO_2016_R	ACTIVE	N	1900-01-01	9999-12-31	2016-06-02
Diagnosis: ICD10 Code, I00-I99, Define	I00-I99	Diseases of the circulatory system	141262	Deep venous thrombosis of peroneal vein (HCC)	ICD10CM	1	I82.499		Acute embolism and thrombosis of other specified deep	IMO	GHS-PACDC	DX_LEGACY	ACTIVE	N	1900-01-01	9999-12-31	2015-07-10
Diagnosis: ICD10 Code, I00-I99, Define	I00-I99	Diseases of the circulatory system	141263	Deep vein thrombosis of peroneal vein (HCC)	ICD10CM	1	I82.499		Acute embolism and thrombosis of other specified deep	IMO	GHS-PACDC	DX_LEGACY	ACTIVE	N	1900-01-01	9999-12-31	2015-07-10
Diagnosis: ICD10 Code, I00-I99, Define	I00-I99	Diseases of the circulatory system	738869	Acute deep vein thrombosis of peroneal vein, unspecified laterality (HCC)	ICD10CM	1	I82.499		Acute embolism and thrombosis of other specified deep	IMO	GHS-PACDC	IMO_2015_R	ACTIVE	N	1900-01-01	9999-12-31	2015-07-10
Diagnosis: ICD10 Code, I00-I99, Define	I00-I99	Diseases of the circulatory system	1224458	Deep vein thrombosis (DVT) of proximal vein of right lower extremity, unspecified chronicity (H	ICD10CM	1	I82.4Y1		Acute embolism and thrombosis of unspecified deep vei	IMO	GHS-PACDC	IMO_2016_R	ACTIVE	N	1900-01-01	9999-12-31	2016-06-02
Diagnosis: ICD10 Code, I00-I99, Define	I00-I99	Diseases of the circulatory system	1081805	Embolism from thrombosis of vein of thigh, right (HCC)	ICD10CM	1	I82.4Y1		Acute embolism and thrombosis of unspecified deep vei	IMO	GHS-PACDC	IMO_2015_R	ACTIVE	N	1900-01-01	9999-12-31	2015-07-10
Diagnosis: ICD10 Code, I00-I99, Define	I00-I99	Diseases of the circulatory system	477418	Acute venous embolism and thrombosis of deep vessels of proximal end of right lower extremit	ICD10CM	1	I82.4Y1		Acute embolism and thrombosis of unspecified deep vei	IMO	GHS-PACDC	IMO_2015_R	ACTIVE	N	1900-01-01	9999-12-31	2015-07-10
Diagnosis: ICD10 Code, I00-I99, Define	I00-I99	Diseases of the circulatory system	499726	Venous embolism and thrombosis of deep vessels of left proximal lower extremity (HCC)	ICD10CM	1	I82.4Y2		Acute embolism and thrombosis of unspecified deep vei	IMO	GHS-PACDC	IMO_2015_R	ACTIVE	N	1900-01-01	9999-12-31	2015-07-10
Diagnosis: ICD10 Code, I00-I99, Define	I00-I99	Diseases of the circulatory system	473486	Acute venous embolism and thrombosis of deep vessels of proximal end of left lower extremity	ICD10CM	1	I82.4Y2		Acute embolism and thrombosis of unspecified deep vei	IMO	GHS-PACDC	IMO_2015_R	ACTIVE	N	1900-01-01	9999-12-31	2015-07-10
Diagnosis: ICD10 Code, I00-I99, Define	I00-I99	Diseases of the circulatory system	728303	Acute thromboembolism of deep veins of proximal leg, left (HCC)	ICD10CM	1	I82.4Y2		Acute embolism and thrombosis of unspecified deep vei	IMO	GHS-PACDC	IMO_2015_R	ACTIVE	N	1900-01-01	9999-12-31	2015-07-10
Diagnosis: ICD10 Code, I00-I99, Define	I00-I99	Diseases of the circulatory system	596724	Acute embolism and thrombosis of unspecified deep veins of left proximal lower extremity	ICD10CM	1	I82.4Y2		Acute embolism and thrombosis of unspecified deep vei	IMO	GHS-PACDC	IMO_2015_R	ACTIVE	Y	1900-01-01	9999-12-31	2015-07-10
Diagnosis: ICD10 Code, I00-I99, Define	I00-I99	Diseases of the circulatory system	898021	DVT, lower extremity, proximal, acute, left (HCC)	ICD10CM	1	I82.4Y2		Acute embolism and thrombosis of unspecified deep vei	IMO	GHS-PACDC	IMO_2015_R	ACTIVE	N	1900-01-01	9999-12-31	2015-07-10
Diagnosis: ICD10 Code, I00-I99, Define	I00-I99	Diseases of the circulatory system	345028	Acute embolism and thrombosis of deep vein of both proximal lower extremities (HCC)	ICD10CM	1	I82.4Y3		Acute embolism and thrombosis of unspecified deep vei	IMO	GHS-PACDC	DX_LEGACY	ACTIVE	N	1900-01-01	9999-12-31	2015-07-10
Diagnosis: ICD10 Code, I00-I99, Define	I00-I99	Diseases of the circulatory system	475594	Venous embolism and thrombosis of deep vessels of distal end of right lower extremity (HCC)	ICD10CM	1	I82.4Z1		Acute embolism and thrombosis of unspecified deep vei	IMO	GHS-PACDC	IMO_2015_R	ACTIVE	N	1900-01-01	9999-12-31	2015-07-10
Diagnosis: ICD10 Code, I00-I99, Define	I00-I99	Diseases of the circulatory system	1084985	Acute deep vein thrombosis of distal end of lower extremity, right (HCC)	ICD10CM	1	I82.4Z1		Acute embolism and thrombosis of unspecified deep vei	IMO	GHS-PACDC	IMO_2015_R	ACTIVE	N	1900-01-01	9999-12-31	2015-07-10
Diagnosis: ICD10 Code, I00-I99, Define	I00-I99	Diseases of the circulatory system	812149	Acute venous embolism and thrombosis of deep vessels of distal lower extremity, left (HCC)	ICD10CM	1	I82.4Z2		Acute embolism and thrombosis of unspecified deep vei	IMO	GHS-PACDC	IMO_2015_R	ACTIVE	N	1900-01-01	9999-12-31	2015-07-10
Diagnosis: ICD10 Code, I00-I99, Define	I00-I99	Diseases of the circulatory system	600527	Acute embolism and thrombosis of unspecified deep veins of left distal lower extremity	ICD10CM	1	I82.4Z2		Acute embolism and thrombosis of unspecified deep vei	IMO	GHS-PACDC	IMO_2015_R	ACTIVE	Y	1900-01-01	9999-12-31	2015-07-10
Diagnosis: ICD10 Code, I00-I99, Define	I00-I99	Diseases of the circulatory system	375306	Acute embolism and thrombosis of deep vein of both distal legs (HCC)	ICD10CM	1	I82.4Z3		Acute embolism and thrombosis of unspecified deep vei	IMO	GHS-PACDC	DX_LEGACY	ACTIVE	N	1900-01-01	9999-12-31	2015-07-10
Diagnosis: ICD10 Code, I00-I99, Define	I00-I99	Diseases of the circulatory system	939455	Deep venous thrombosis of calf, bilateral	ICD10CM	1	I82.4Z3		Acute embolism and thrombosis of unspecified deep vei	IMO	GHS-PACDC	IMO_2015_R	DELETED	Y	1900-01-01	9999-12-31	2015-07-10
Diagnosis: ICD10 Code, I00-I99, Define	I00-I99	Diseases of the circulatory system	213912	Acute venous embolism and thrombosis of deep vessels of distal lower extremity (HCC)	ICD10CM	1	I82.4Z9		Acute embolism and thrombosis of unspecified deep vei	IMO	GHS-PACDC	DX_LEGACY	ACTIVE	N	1900-01-01	9999-12-31	2015-07-10
Diagnosis: ICD10 Code, I00-I99, Define	I00-I99	Diseases of the circulatory system	1223687	Acute deep vein thrombosis (DVT) of distal vein of lower extremity, unspecified laterality (HCC)	ICD10CM	1	I82.4Z9		Acute embolism and thrombosis of unspecified deep vei	IMO	GHS-PACDC	IMO_2016_R	ACTIVE	N	1900-01-01	9999-12-31	2016-06-02
Diagnosis: ICD10 Code, I00-I99, Define	I00-I99	Diseases of the circulatory system	869946	DVT, lower extremity, distal, unspecified laterality	ICD10CM	1	I82.4Z9		Acute embolism and thrombosis of unspecified deep vei	IMO	GHS-PACDC	IMO_2015_R	DELETED	Y	1900-01-01	9999-12-31	2015-07-10
Diagnosis: ICD10 Code, I00-I99, Define	I00-I99	Diseases of the circulatory system	778089	Acute thromboembolism of deep veins of distal leg, unspecified laterality (HCC)	ICD10CM	1	I82.4Z9		Acute embolism and thrombosis of unspecified deep vei	IMO	GHS-PACDC	IMO_2015_R	ACTIVE	N	1900-01-01	9999-12-31	2015-07-10
Diagnosis: ICD10 Code, I00-I99, Define	I00-I99	Diseases of the circulatory system	342798	Venous embolism and thrombosis of deep vessels of distal lower extremity (HCC)	ICD10CM	1	I82.4Z9		Acute embolism and thrombosis of unspecified deep vei	IMO	GHS-PACDC	DX_LEGACY	ACTIVE	N	1900-01-01	9999-12-31	2015-07-10
Diagnosis: ICD10 Code, I00-I99, Define	I00-I99	Diseases of the circulatory system	516045	Acute deep vein thrombosis of distal lower extremity (HCC)	ICD10CM	1	I82.4Z9		Acute embolism and thrombosis of unspecified deep vei	IMO	GHS-PACDC	IMO_2015_R	ACTIVE	N	1900-01-01	9999-12-31	2015-07-10
Diagnosis: ICD10 Code, I00-I99, Define	I00-I99	Diseases of the circulatory system	215741	Acute deep vein thrombosis of calf (HCC)	ICD10CM	1	I82.4Z9		Acute embolism and thrombosis of unspecified deep vei	IMO	GHS-PACDC	DX_LEGACY	ACTIVE	N	1900-01-01	9999-12-31	2015-07-10
Diagnosis: ICD10 Code, I00-I99, Define	I00-I99	Diseases of the circulatory system	490212	Chronic thromboembolism of deep vein of right lower extremity (HCC)	ICD10CM	1	I82.501		Chronic embolism and thrombosis of unspecified deep v	IMO	GHS-PACDC	IMO_2015_R	ACTIVE	N	1900-01-01	9999-12-31	2015-07-10
Diagnosis: ICD10 Code, I00-I99, Define	I00-I99	Diseases of the circulatory system	791622	Chronic deep vein thrombosis of leg, left	ICD10CM	1	I82.502		Chronic embolism and thrombosis of unspecified deep v	IMO	GHS-PACDC	IMO_2015_R	DELETED	Y	1900-01-01	9999-12-31	2015-07-10
Diagnosis: ICD10 Code, I00-I99, Define	I00-I99	Diseases of the circulatory system	929526	Chronic deep vein thrombosis of lower extremity, bilateral	ICD10CM	1	I82.503		Chronic embolism and thrombosis of unspecified deep v	IMO	GHS-PACDC	IMO_2015_R	DELETED	Y	1900-01-01	9999-12-31	2015-07-10
Diagnosis: ICD10 Code, I00-I99, Define	I00-I99	Diseases of the circulatory system	277153	Chronic embolism and thrombosis of deep vein of lower extremity	ICD10CM	1	I82.509		Chronic embolism and thrombosis of unspecified deep v	IMO	GHS-PACDC	DX_LEGACY	DELETED	Y	1900-01-01	9999-12-31	2015-07-10
Diagnosis: ICD10 Code, I00-I99, Define	I00-I99	Diseases of the circulatory system	303858	Chronic deep venous thrombosis of lower extremity due to CABG (HCC)	ICD10CM	2	I82.509	T82.868A,I82.509	Chronic embolism and thrombosis of unspecified deep v	IMO	GHS-PACDC	DX_LEGACY	ACTIVE	N	1900-01-01	2016-10-14	2015-07-10
Diagnosis: ICD10 Code, I00-I99, Define	I00-I99	Diseases of the circulatory system	1132930	Chronic deep vein thrombosis (DVT) of femoral vein of both lower extremities (HCC)	ICD10CM	1	I82.513		Chronic embolism and thrombosis of femoral vein, bilat	IMO	GHS-PACDC	IMO_2016_R	ACTIVE	N	1900-01-01	9999-12-31	2016-06-02
Diagnosis: ICD10 Code, I00-I99, Define	I00-I99	Diseases of the circulatory system	476022	Chronic thromboembolism of both femoral veins (HCC)	ICD10CM	1	I82.513		Chronic embolism and thrombosis of femoral vein, bilat	IMO	GHS-PACDC	IMO_2015_R	ACTIVE	N	1900-01-01	9999-12-31	2015-07-10
Diagnosis: ICD10 Code, I00-I99, Define	I00-I99	Diseases of the circulatory system	1223942	Chronic deep vein thrombosis (DVT) of femoral vein, unspecified laterality (HCC)	ICD10CM	1	I82.519		Chronic embolism and thrombosis of unspecified femor	IMO	GHS-PACDC	IMO_2016_R	ACTIVE	N	1900-01-01	9999-12-31	2016-06-02
Diagnosis: ICD10 Code, I00-I99, Define	I00-I99	Diseases of the circulatory system	269851	Chronic embolism and thrombosis of right iliac vein (HCC)	ICD10CM	1	I82.521		Chronic embolism and thrombosis of right iliac vein	IMO	GHS-PACDC	DX_LEGACY	ACTIVE	N	1900-01-01	9999-12-31	2015-07-10
Diagnosis: ICD10 Code, I00-I99, Define	I00-I99	Diseases of the circulatory system	303686	Chronic deep vein thrombosis of left ileofemoral vein (HCC)	ICD10CM	1	I82.522		Chronic embolism and thrombosis of left iliac vein	IMO	GHS-PACDC	DX_LEGACY	ACTIVE	N	1900-01-01	9999-12-31	2015-07-10
Diagnosis: ICD10 Code, I00-I99, Define	I00-I99	Diseases of the circulatory system	1132963	Chronic deep vein thrombosis (DVT) of iliac vein of left lower extremity (HCC)	ICD10CM	1	I82.522		Chronic embolism and thrombosis of left iliac vein	IMO	GHS-PACDC	IMO_2016_R	ACTIVE	N	1900-01-01	9999-12-31	2016-06-02
Diagnosis: ICD10 Code, I00-I99, Define	I00-I99	Diseases of the circulatory system	314621	Chronic iliac vein thrombosis (HCC)	ICD10CM	1	I82.529		Chronic embolism and thrombosis of unspecified iliac ve	IMO	GHS-PACDC	DX_LEGACY	ACTIVE	N	2017-09-17	9999-12-31	2017-10-30
Diagnosis: ICD10 Code, I00-I99, Define	I00-I99	Diseases of the circulatory system	749820	Chronic thromboembolism of popliteal vein, left (HCC)	ICD10CM	1	I82.532		Chronic embolism and thrombosis of left popliteal vein	IMO	GHS-PACDC	IMO_2015_R	ACTIVE	N	1900-01-01	9999-12-31	2015-07-10
Diagnosis: ICD10 Code, I00-I99, Define	I00-I99	Diseases of the circulatory system	722300	Chronic deep vein thrombosis of popliteal vein, unspecified laterality	ICD10CM	1	I82.539		Chronic embolism and thrombosis of unspecified poplite	IMO	GHS-PACDC	IMO_2015_R	DELETED	Y	1900-01-01	9999-12-31	2015-07-10
Diagnosis: ICD10 Code, I00-I99, Define	I00-I99	Diseases of the circulatory system	596270	Chronic embolism and thrombosis of other specified deep vein of lower extremity, bilateral	ICD10CM	1	I82.593		Chronic embolism and thrombosis of other specified de	IMO	GHS-PACDC	IMO_2015_R	ACTIVE	Y	1900-01-01	9999-12-31	2015-07-10
Diagnosis: ICD10 Code, I00-I99, Define	I00-I99	Diseases of the circulatory system	722265	Chronic embolism and thrombosis of deep vein of proximal lower extremity, right (HCC)	ICD10CM	1	I82.5Y1		Chronic embolism and thrombosis of unspecified deep v	IMO	GHS-PACDC	IMO_2015_R	ACTIVE	N	1900-01-01	9999-12-31	2015-07-10
Diagnosis: ICD10 Code, I00-I99, Define	I00-I99	Diseases of the circulatory system	1132834	Chronic deep vein thrombosis (DVT) of proximal vein of right lower extremity (HCC)	ICD10CM	1	I82.5Y1		Chronic embolism and thrombosis of unspecified deep v	IMO	GHS-PACDC	IMO_2016_R	ACTIVE	N	1900-01-01	9999-12-31	2016-06-02
Diagnosis: ICD10 Code, I00-I99, Define	I00-I99	Diseases of the circulatory system	731018	Chronic thromboembolism of deep veins of proximal leg, left (HCC)	ICD10CM	1	I82.5Y2		Chronic embolism and thrombosis of unspecified deep v	IMO	GHS-PACDC	IMO_2015_R	ACTIVE	N	1900-01-01	9999-12-31	2015-07-10
Diagnosis: ICD10 Code, I00-I99, Define	I00-I99	Diseases of the circulatory system	1366726	Chr emblsm and thombos unsp deep vn of left prox low extrm (HCC)	ICD10CM	1	I82.5Y2		Chronic embolism and thrombosis of unspecified deep v	IMO	GHS-PACDC	IMO_2018_R	ACTIVE	N	1900-01-01	9999-12-31	2017-10-30
Diagnosis: ICD10 Code, I00-I99, Define	I00-I99	Diseases of the circulatory system	724819	Chronic thromboembolism of deep veins of upper leg, left (HCC)	ICD10CM	1	I82.5Y2		Chronic embolism and thrombosis of unspecified deep v	IMO	GHS-PACDC	IMO_2015_R	ACTIVE	N	1900-01-01	9999-12-31	2015-07-10
Diagnosis: ICD10 Code, I00-I99, Define	I00-I99	Diseases of the circulatory system	761710	Chronic embolism and thrombosis of deep vein of proximal leg, left (HCC)	ICD10CM	1	I82.5Y2		Chronic embolism and thrombosis of unspecified deep v	IMO	GHS-PACDC	IMO_2015_R	ACTIVE	N	1900-01-01	9999-12-31	2015-07-10
Diagnosis: ICD10 Code, I00-I99, Define	I00-I99	Diseases of the circulatory system	215749	Chronic deep vein thrombosis of proximal leg (HCC)	ICD10CM	1	I82.5Y9		Chronic embolism and thrombosis of unspecified deep v	IMO	GHS-PACDC	DX_LEGACY	ACTIVE	N	1900-01-01	9999-12-31	2015-07-10
Diagnosis: ICD10 Code, I00-I99, Define	I00-I99	Diseases of the circulatory system	355221	Chronic embolism and thrombosis of deep vein of proximal lower extremity (HCC)	ICD10CM	1	I82.5Y9		Chronic embolism and thrombosis of unspecified deep v	IMO	GHS-PACDC	DX_LEGACY	ACTIVE	N	1900-01-01	9999-12-31	2015-07-10
Diagnosis: ICD10 Code, I00-I99, Define	I00-I99	Diseases of the circulatory system	472748	Chronic venous embolism and thrombosis of deep vessels of distal end of right lower extremity	ICD10CM	1	I82.5Z1		Chronic embolism and thrombosis of unspecified deep v	IMO	GHS-PACDC	IMO_2015_R	ACTIVE	N	1900-01-01	9999-12-31	2015-07-10
Diagnosis: ICD10 Code, I00-I99, Define	I00-I99	Diseases of the circulatory system	832298	Chronic deep vein thrombosis of lower leg, left	ICD10CM	1	I82.5Z2		Chronic embolism and thrombosis of unspecified deep v	IMO	GHS-PACDC	IMO_2015_R	DELETED	Y	1900-01-01	9999-12-31	2015-07-10
Diagnosis: ICD10 Code, I00-I99, Define	I00-I99	Diseases of the circulatory system	828995	Chronic deep vein thrombosis of calf, left	ICD10CM	1	I82.5Z2		Chronic embolism and thrombosis of unspecified deep v	IMO	GHS-PACDC	IMO_2015_R	DELETED	Y	1900-01-01	9999-12-31	2015-07-10
Diagnosis: ICD10 Code, I00-I99, Define	I00-I99	Diseases of the circulatory system	1127713	Chronic deep vein thrombosis of distal vein of left lower extremity (HCC)	ICD10CM	1	I82.5Z2		Chronic embolism and thrombosis of unspecified deep v	IMO	GHS-PACDC	IMO_2016_R	ACTIVE	N	1900-01-01	9999-12-31	2016-06-02
Diagnosis: ICD10 Code, I00-I99, Define	I00-I99	Diseases of the circulatory system	1365566	Chr emblsm and thombos unsp deep veins of dist low extrm, bi (HCC)	ICD10CM	1	I82.5Z3		Chronic embolism and thrombosis of unspecified deep v	IMO	GHS-PACDC	IMO_2018_R	ACTIVE	N	1900-01-01	9999-12-31	2017-10-30
Diagnosis: ICD10 Code, I00-I99, Define	I00-I99	Diseases of the circulatory system	219270	Chronic venous embolism and thrombosis of deep vessels of distal lower extremity (HCC)	ICD10CM	1	I82.5Z9		Chronic embolism and thrombosis of unspecified deep v	IMO	GHS-PACDC	DX_LEGACY	ACTIVE	N	1900-01-01	9999-12-31	2015-07-10
Diagnosis: ICD10 Code, I00-I99, Define	I00-I99	Diseases of the circulatory system	1132490	Acute embolism from vein of upper extremity	ICD10CM	1	I82.609		Acute embolism and thrombosis of unspecified veins of	IMO	GHS-PACDC	IMO_2016_R	ACTIVE	N	1900-01-01	9999-12-31	2016-06-02

Diagnosis: ICD10 Code, I00-I99, Define	I00-I99	Diseases of the circulatory system	947117	Blood clot of vein in shoulder area, unspecified laterality	ICD10CM	1	I82.609		Acute embolism and thrombosis of unspecified veins of	IMO	GHS-PACDC	IMO_2015_R	ACTIVE	N	1900-01-01	9999-12-31	2015-07-10
Diagnosis: ICD10 Code, I00-I99, Define	I00-I99	Diseases of the circulatory system	215314	Acute embolism of upper extremity vein	ICD10CM	1	I82.609		Acute embolism and thrombosis of unspecified veins of	IMO	GHS-PACDC	DX_LEGACY	DELETED	Y	1900-01-01	9999-12-31	2015-07-10
Diagnosis: ICD10 Code, I00-I99, Define	I00-I99	Diseases of the circulatory system	219107	Acute venous embolism and thrombosis of upper extremity	ICD10CM	1	I82.609		Acute embolism and thrombosis of unspecified veins of	IMO	GHS-PACDC	DX_LEGACY	ACTIVE	N	1900-01-01	9999-12-31	2015-07-10
Diagnosis: ICD10 Code, I00-I99, Define	I00-I99	Diseases of the circulatory system	215771	Acute thrombosis of arm	ICD10CM	1	I82.609		Acute embolism and thrombosis of unspecified veins of	IMO	GHS-PACDC	DX_LEGACY	ACTIVE	N	1900-01-01	9999-12-31	2015-07-10
Diagnosis: ICD10 Code, I00-I99, Define	I00-I99	Diseases of the circulatory system	868262	Acute venous embolism and thrombosis of superficial veins of upper extremity, right	ICD10CM	1	I82.611		Acute embolism and thrombosis of superficial veins of ri	IMO	GHS-PACDC	IMO_2015_R	ACTIVE	N	1900-01-01	9999-12-31	2015-07-10
Diagnosis: ICD10 Code, I00-I99, Define	I00-I99	Diseases of the circulatory system	354829	Acute embolism and thrombosis of superficial vein of right upper extremity	ICD10CM	1	I82.611		Acute embolism and thrombosis of superficial veins of ri	IMO	GHS-PACDC	DX_LEGACY	ACTIVE	N	1900-01-01	9999-12-31	2015-07-10
Diagnosis: ICD10 Code, I00-I99, Define	I00-I99	Diseases of the circulatory system	749954	Acute embolism of superficial veins of upper extremity, right	ICD10CM	1	I82.611		Acute embolism and thrombosis of superficial veins of ri	IMO	GHS-PACDC	IMO_2015_R	DELETED	Y	1900-01-01	9999-12-31	2015-07-10
Diagnosis: ICD10 Code, I00-I99, Define	I00-I99	Diseases of the circulatory system	478750	Acute embolism and thrombosis of left cephalic vein	ICD10CM	1	I82.612		Acute embolism and thrombosis of superficial veins of le	IMO	GHS-PACDC	IMO_2015_R	ACTIVE	N	1900-01-01	9999-12-31	2015-07-10
Diagnosis: ICD10 Code, I00-I99, Define	I00-I99	Diseases of the circulatory system	457954	Venous embolism and thrombosis of superficial veins of left upper extremity	ICD10CM	1	I82.612		Acute embolism and thrombosis of superficial veins of le	IMO	GHS-PACDC	IMO_2015_R	ACTIVE	N	1900-01-01	9999-12-31	2015-07-10
Diagnosis: ICD10 Code, I00-I99, Define	I00-I99	Diseases of the circulatory system	928701	Superficial venous thrombosis of upper extremity, bilateral	ICD10CM	1	I82.613		Acute embolism and thrombosis of superficial veins of u	IMO	GHS-PACDC	IMO_2015_R	ACTIVE	N	1900-01-01	9999-12-31	2015-07-10
Diagnosis: ICD10 Code, I00-I99, Define	I00-I99	Diseases of the circulatory system	925616	Superficial venous thrombosis of arm, bilateral	ICD10CM	1	I82.613		Acute embolism and thrombosis of superficial veins of u	IMO	GHS-PACDC	IMO_2015_R	ACTIVE	N	1900-01-01	9999-12-31	2015-07-10
Diagnosis: ICD10 Code, I00-I99, Define	I00-I99	Diseases of the circulatory system	925956	Thrombosis superficial vein, arm, acute, bilateral	ICD10CM	1	I82.613		Acute embolism and thrombosis of superficial veins of u	IMO	GHS-PACDC	IMO_2015_R	ACTIVE	N	1900-01-01	9999-12-31	2015-07-10
Diagnosis: ICD10 Code, I00-I99, Define	I00-I99	Diseases of the circulatory system	737690	Acute antecubital vein embolism, unspecified laterality	ICD10CM	1	I82.619		Acute embolism and thrombosis of superficial veins of u	IMO	GHS-PACDC	IMO_2015_R	ACTIVE	N	1900-01-01	9999-12-31	2015-07-10
Diagnosis: ICD10 Code, I00-I99, Define	I00-I99	Diseases of the circulatory system	723621	Acute cephalic vein embolism, unspecified laterality	ICD10CM	1	I82.619		Acute embolism and thrombosis of superficial veins of u	IMO	GHS-PACDC	IMO_2015_R	ACTIVE	N	1900-01-01	9999-12-31	2015-07-10
Diagnosis: ICD10 Code, I00-I99, Define	I00-I99	Diseases of the circulatory system	215217	Acute antecubital vein embolism	ICD10CM	1	I82.619		Acute embolism and thrombosis of superficial veins of u	IMO	GHS-PACDC	DX_LEGACY	ACTIVE	N	1900-01-01	9999-12-31	2015-07-10
Diagnosis: ICD10 Code, I00-I99, Define	I00-I99	Diseases of the circulatory system	743936	Thrombosis superficial vein, arm, acute, unspecified laterality	ICD10CM	1	I82.619		Acute embolism and thrombosis of superficial veins of u	IMO	GHS-PACDC	IMO_2015_R	ACTIVE	N	1900-01-01	9999-12-31	2015-07-10
Diagnosis: ICD10 Code, I00-I99, Define	I00-I99	Diseases of the circulatory system	213925	Basilic vein thrombosis	ICD10CM	1	I82.619		Acute embolism and thrombosis of superficial veins of u	IMO	GHS-PACDC	DX_LEGACY	ACTIVE	N	2016-04-10	9999-12-31	2015-07-10
Diagnosis: ICD10 Code, I00-I99, Define	I00-I99	Diseases of the circulatory system	1132486	Acute embolism from superficial vein of upper extremity	ICD10CM	1	I82.619		Acute embolism and thrombosis of superficial veins of u	IMO	GHS-PACDC	IMO_2016_R	ACTIVE	N	1900-01-01	9999-12-31	2016-06-02
Diagnosis: ICD10 Code, I00-I99, Define	I00-I99	Diseases of the circulatory system	790617	Acute thrombosis of superficial veins of upper extremity, unspecified laterality	ICD10CM	1	I82.619		Acute embolism and thrombosis of superficial veins of u	IMO	GHS-PACDC	IMO_2015_R	ACTIVE	N	1900-01-01	9999-12-31	2015-07-10
Diagnosis: ICD10 Code, I00-I99, Define	I00-I99	Diseases of the circulatory system	213942	Acute venous embolism and thrombosis of cephalic vein	ICD10CM	1	I82.619		Acute embolism and thrombosis of superficial veins of u	IMO	GHS-PACDC	DX_LEGACY	ACTIVE	N	1900-01-01	9999-12-31	2015-07-10
Diagnosis: ICD10 Code, I00-I99, Define	I00-I99	Diseases of the circulatory system	215316	Acute embolism of superficial veins of upper extremity	ICD10CM	1	I82.619		Acute embolism and thrombosis of superficial veins of u	IMO	GHS-PACDC	DX_LEGACY	DELETED	Y	1900-01-01	9999-12-31	2015-07-10
Diagnosis: ICD10 Code, I00-I99, Define	I00-I99	Diseases of the circulatory system	740831	Acute thrombosis of radial vein, right	ICD10CM	1	I82.621		Acute embolism and thrombosis of deep veins of right u	IMO	GHS-PACDC	IMO_2015_R	DELETED	Y	1900-01-01	9999-12-31	2015-07-10
Diagnosis: ICD10 Code, I00-I99, Define	I00-I99	Diseases of the circulatory system	808567	Acute embolism of deep veins of upper extremity, right	ICD10CM	1	I82.621		Acute embolism and thrombosis of deep veins of right u	IMO	GHS-PACDC	IMO_2015_R	DELETED	Y	1900-01-01	9999-12-31	2015-07-10
Diagnosis: ICD10 Code, I00-I99, Define	I00-I99	Diseases of the circulatory system	1128830	Deep vein thrombosis (DVT) of right radial vein (HCC)	ICD10CM	1	I82.621		Acute embolism and thrombosis of deep veins of right u	IMO	GHS-PACDC	IMO_2016_R	ACTIVE	N	1900-01-01	9999-12-31	2016-06-02
Diagnosis: ICD10 Code, I00-I99, Define	I00-I99	Diseases of the circulatory system	876336	Acute embolism of radial vein, right	ICD10CM	1	I82.621		Acute embolism and thrombosis of deep veins of right u	IMO	GHS-PACDC	IMO_2015_R	DELETED	Y	1900-01-01	9999-12-31	2015-07-10
Diagnosis: ICD10 Code, I00-I99, Define	I00-I99	Diseases of the circulatory system	1127743	Acute deep vein thrombosis of brachial vein of right upper extremity (HCC)	ICD10CM	1	I82.621		Acute embolism and thrombosis of deep veins of right u	IMO	GHS-PACDC	IMO_2016_R	ACTIVE	N	1900-01-01	9999-12-31	2016-06-02
Diagnosis: ICD10 Code, I00-I99, Define	I00-I99	Diseases of the circulatory system	233627	Brachial vein thrombosis, left (HCC)	ICD10CM	1	I82.622		Acute embolism and thrombosis of deep veins of left up	IMO	GHS-PACDC	DX_LEGACY	ACTIVE	N	1900-01-01	9999-12-31	2015-07-10
Diagnosis: ICD10 Code, I00-I99, Define	I00-I99	Diseases of the circulatory system	763097	Acute ulnar vein thrombosis, left	ICD10CM	1	I82.622		Acute embolism and thrombosis of deep veins of left up	IMO	GHS-PACDC	IMO_2015_R	DELETED	Y	1900-01-01	9999-12-31	2015-07-10
Diagnosis: ICD10 Code, I00-I99, Define	I00-I99	Diseases of the circulatory system	1225871	Deep vein thrombosis (DVT) of brachial vein of left upper extremity, unspecified chronicity (HCC	ICD10CM	1	I82.622		Acute embolism and thrombosis of deep veins of left up	IMO	GHS-PACDC	IMO_2016_R	ACTIVE	N	1900-01-01	9999-12-31	2016-06-02
Diagnosis: ICD10 Code, I00-I99, Define	I00-I99	Diseases of the circulatory system	478959	Acute embolism and thrombosis of left brachial vein (HCC)	ICD10CM	1	I82.622		Acute embolism and thrombosis of deep veins of left up	IMO	GHS-PACDC	IMO_2015_R	ACTIVE	N	1900-01-01	9999-12-31	2015-07-10
Diagnosis: ICD10 Code, I00-I99, Define	I00-I99	Diseases of the circulatory system	859149	Venous embolism and thrombosis of radial vein, left (HCC)	ICD10CM	1	I82.622		Acute embolism and thrombosis of deep veins of left up	IMO	GHS-PACDC	IMO_2015_R	ACTIVE	N	1900-01-01	9999-12-31	2015-07-10
Diagnosis: ICD10 Code, I00-I99, Define	I00-I99	Diseases of the circulatory system	736247	Venous embolism and thrombosis of deep veins of upper extremity, left (HCC)	ICD10CM	1	I82.622		Acute embolism and thrombosis of deep veins of left up	IMO	GHS-PACDC	IMO_2015_R	ACTIVE	N	1900-01-01	9999-12-31	2015-07-10
Diagnosis: ICD10 Code, I00-I99, Define	I00-I99	Diseases of the circulatory system	478562	Acute embolism and thrombosis of left ulnar vein (HCC)	ICD10CM	1	I82.622		Acute embolism and thrombosis of deep veins of left up	IMO	GHS-PACDC	IMO_2015_R	ACTIVE	N	1900-01-01	9999-12-31	2015-07-10
Diagnosis: ICD10 Code, I00-I99, Define	I00-I99	Diseases of the circulatory system	1132480	Acute embolism from left ulnar vein (HCC)	ICD10CM	1	I82.622		Acute embolism and thrombosis of deep veins of left up	IMO	GHS-PACDC	IMO_2016_R	ACTIVE	N	1900-01-01	9999-12-31	2016-06-02
Diagnosis: ICD10 Code, I00-I99, Define	I00-I99	Diseases of the circulatory system	739463	Deep venous thrombosis of arm, left	ICD10CM	1	I82.622		Acute embolism and thrombosis of deep veins of left up	IMO	GHS-PACDC	IMO_2015_R	DELETED	Y	1900-01-01	9999-12-31	2015-07-10
Diagnosis: ICD10 Code, I00-I99, Define	I00-I99	Diseases of the circulatory system	456374	Acute thrombosis of deep vein of left upper extremity (HCC)	ICD10CM	1	I82.622		Acute embolism and thrombosis of deep veins of left up	IMO	GHS-PACDC	IMO_2015_R	ACTIVE	N	1900-01-01	9999-12-31	2015-07-10
Diagnosis: ICD10 Code, I00-I99, Define	I00-I99	Diseases of the circulatory system	1230810	Acute deep vein thrombosis (DVT) of left upper extremity, unspecified vein (HCC)	ICD10CM	1	I82.622		Acute embolism and thrombosis of deep veins of left up	IMO	GHS-PACDC	IMO_2016_R	ACTIVE	N	1900-01-01	9999-12-31	2016-06-02
Diagnosis: ICD10 Code, I00-I99, Define	I00-I99	Diseases of the circulatory system	1128832	Deep vein thrombosis (DVT) of left radial vein (HCC)	ICD10CM	1	I82.622		Acute embolism and thrombosis of deep veins of left up	IMO	GHS-PACDC	IMO_2016_R	ACTIVE	N	1900-01-01	9999-12-31	2016-06-02
Diagnosis: ICD10 Code, I00-I99, Define	I00-I99	Diseases of the circulatory system	939453	Acute embolism of deep veins of upper extremity, bilateral	ICD10CM	1	I82.623		Acute embolism and thrombosis of deep veins of upper	IMO	GHS-PACDC	IMO_2015_R	DELETED	Y	1900-01-01	9999-12-31	2015-07-10
Diagnosis: ICD10 Code, I00-I99, Define	I00-I99	Diseases of the circulatory system	1179586	Acute embolism and thrombosis of deep veins of upper extremity, bilateral (HCC)	ICD10CM	1	I82.623		Acute embolism and thrombosis of deep veins of upper	IMO	GHS-PACDC	IMO_2016_R	ACTIVE	N	1900-01-01	9999-12-31	2016-06-02
Diagnosis: ICD10 Code, I00-I99, Define	I00-I99	Diseases of the circulatory system	927738	Deep venous thrombosis of upper extremity, bilateral	ICD10CM	1	I82.623		Acute embolism and thrombosis of deep veins of upper	IMO	GHS-PACDC	IMO_2015_R	DELETED	Y	1900-01-01	9999-12-31	2015-07-10
Diagnosis: ICD10 Code, I00-I99, Define	I00-I99	Diseases of the circulatory system	261447	Acute thrombosis of deep vein of upper extremity (HCC)	ICD10CM	1	I82.629		Acute embolism and thrombosis of deep veins of unspe	IMO	GHS-PACDC	DX_LEGACY	ACTIVE	N	1900-01-01	9999-12-31	2015-07-10
Diagnosis: ICD10 Code, I00-I99, Define	I00-I99	Diseases of the circulatory system	1132762	Acute deep vein thrombosis (DVT) of brachial vein (HCC)	ICD10CM	1	I82.629		Acute embolism and thrombosis of deep veins of unspe	IMO	GHS-PACDC	IMO_2016_R	ACTIVE	N	1900-01-01	9999-12-31	2016-06-02
Diagnosis: ICD10 Code, I00-I99, Define	I00-I99	Diseases of the circulatory system	1085163	Acute deep venous thrombosis of upper extremity as complication of procedure, initial encount	ICD10CM	2	I82.629	T81.72XA,I82.629	Acute embolism and thrombosis of deep veins of unspe	IMO	GHS-PACDC	IMO_2015_R	ACTIVE	N	2016-09-17	2017-04-27	2016-09-30
Diagnosis: ICD10 Code, I00-I99, Define	I00-I99	Diseases of the circulatory system	1132790	Acute deep vein thrombosis (DVT) of radial vein (HCC)	ICD10CM	1	I82.629		Acute embolism and thrombosis of deep veins of unspe	IMO	GHS-PACDC	IMO_2016_R	ACTIVE	N	1900-01-01	9999-12-31	2016-06-02
Diagnosis: ICD10 Code, I00-I99, Define	I00-I99	Diseases of the circulatory system	595690	Chronic embolism and thrombosis of unspecified veins of left upper extremity	ICD10CM	1	I82.702		Chronic embolism and thrombosis of unspecified veins c	IMO	GHS-PACDC	IMO_2015_R	ACTIVE	Y	1900-01-01	9999-12-31	2015-07-10
Diagnosis: ICD10 Code, I00-I99, Define	I00-I99	Diseases of the circulatory system	1365788	Chronic embolism and thombos unsp veins of l up extrem	ICD10CM	1	I82.702		Chronic embolism and thrombosis of unspecified veins c	IMO	GHS-PACDC	IMO_2018_R	ACTIVE	N	1900-01-01	9999-12-31	2017-10-30
Diagnosis: ICD10 Code, I00-I99, Define	I00-I99	Diseases of the circulatory system	930872	Chronic thrombosis of arm, bilateral	ICD10CM	1	I82.703		Chronic embolism and thrombosis of unspecified veins c	IMO	GHS-PACDC	IMO_2015_R	ACTIVE	N	1900-01-01	9999-12-31	2015-07-10
Diagnosis: ICD10 Code, I00-I99, Define	I00-I99	Diseases of the circulatory system	868945	Chronic embolism and thrombosis of vein of upper extremity, unspecified laterality	ICD10CM	1	I82.709		Chronic embolism and thrombosis of unspecified veins c	IMO	GHS-PACDC	IMO_2015_R	ACTIVE	N	1900-01-01	9999-12-31	2015-07-10
Diagnosis: ICD10 Code, I00-I99, Define	I00-I99	Diseases of the circulatory system	474450	Chronic venous embolism and thrombosis of right basilic vein	ICD10CM	1	I82.711		Chronic embolism and thrombosis of superficial veins of	IMO	GHS-PACDC	IMO_2015_R	ACTIVE	N	1900-01-01	9999-12-31	2015-07-10
Diagnosis: ICD10 Code, I00-I99, Define	I00-I99	Diseases of the circulatory system	478349	Chronic embolism and thrombosis of left cephalic vein	ICD10CM	1	I82.712		Chronic embolism and thrombosis of superficial veins of	IMO	GHS-PACDC	IMO_2015_R	ACTIVE	N	1900-01-01	9999-12-31	2015-07-10
Diagnosis: ICD10 Code, I00-I99, Define	I00-I99	Diseases of the circulatory system	1132500	Chronic embolism from superficial vein of upper extremity	ICD10CM	1	I82.719		Chronic embolism and thrombosis of superficial veins of	IMO	GHS-PACDC	IMO_2016_R	ACTIVE	N	1900-01-01	9999-12-31	2016-06-02
Diagnosis: ICD10 Code, I00-I99, Define	I00-I99	Diseases of the circulatory system	213929	Chronic venous embolism and thrombosis of cephalic vein	ICD10CM	1	I82.719		Chronic embolism and thrombosis of superficial veins of	IMO	GHS-PACDC	DX_LEGACY	ACTIVE	N	1900-01-01	9999-12-31	2015-07-10
Diagnosis: ICD10 Code, I00-I99, Define	I00-I99	Diseases of the circulatory system	1132854	Chronic deep vein thrombosis (DVT) of ulnar vein of right upper extremity (HCC)	ICD10CM	1	I82.721		Chronic embolism and thrombosis of deep veins of right	IMO	GHS-PACDC	IMO_2016_R	ACTIVE	N	1900-01-01	9999-12-31	2016-06-02
Diagnosis: ICD10 Code, I00-I99, Define	I00-I99	Diseases of the circulatory system	473971	Chronic thrombosis of right brachial vein (HCC)	ICD10CM	1	I82.721		Chronic embolism and thrombosis of deep veins of right	IMO	GHS-PACDC	IMO_2015_R	ACTIVE	N	1900-01-01	9999-12-31	2015-07-10
Diagnosis: ICD10 Code, I00-I99, Define	I00-I99	Diseases of the circulatory system	477143	Chronic embolism of right radial vein	ICD10CM	1	I82.721		Chronic embolism and thrombosis of deep veins of right	IMO	GHS-PACDC	IMO_2015_R	DELETED	Y	1900-01-01	9999-12-31	2015-07-10
Diagnosis: ICD10 Code, I00-I99, Define	I00-I99	Diseases of the circulatory system	303098	Chronic deep vein thrombosis of left upper extremity (HCC)	ICD10CM	1	I82.722		Chronic embolism and thrombosis of deep veins of left u	IMO	GHS-PACDC	DX_LEGACY	ACTIVE	N	1900-01-01	9999-12-31	2015-07-10
Diagnosis: ICD10 Code, I00-I99, Define	I00-I99	Diseases of the circulatory system	925915	Chronic thrombosis of brachial vein, bilateral	ICD10CM	1	I82.723		Chronic embolism and thrombosis of deep veins of uppe	IMO	GHS-PACDC	IMO_2015_R	DELETED	Y	1900-01-01	9999-12-31	2015-07-10
Diagnosis: ICD10 Code, I00-I99, Define	I00-I99	Diseases of the circulatory system	474721	Chronic embolism of both brachial veins	ICD10CM	1	I82.723		Chronic embolism and thrombosis of deep veins of uppe	IMO	GHS-PACDC	IMO_2015_R	DELETED	Y	1900-01-01	2016-05-07	2015-07-10
Diagnosis: ICD10 Code, I00-I99, Define	I00-I99	Diseases of the circulatory system	598755	Chronic embolism and thrombosis of deep veins of unspecified upper extremity	ICD10CM	1	I82.729		Chronic embolism and thrombosis of deep veins of unsp	IMO	GHS-PACDC	IMO_2015_R	ACTIVE	Y	1900-01-01	9999-12-31	2015-07-10
Diagnosis: ICD10 Code, I00-I99, Define	I00-I99	Diseases of the circulatory system	379152	Acute and chronic deep vein thrombosis of upper extremity	ICD10CM	2	I82.729	I82.629,I82.729	Chronic embolism and thrombosis of deep veins of unsp	IMO	GHS-PACDC	DX_LEGACY	DELETED	Y	1900-01-01	2016-05-07	2015-07-10
Diagnosis: ICD10 Code, I00-I99, Define	I00-I99	Diseases of the circulatory system	1224040	Chronic deep vein thrombosis (DVT) of brachial vein, unspecified laterality (HCC)	ICD10CM	1	I82.729		Chronic embolism and thrombosis of deep veins of unsp	IMO	GHS-PACDC	IMO_2016_R	ACTIVE	N	1900-01-01	9999-12-31	2016-06-02
Diagnosis: ICD10 Code, I00-I99, Define	I00-I99	Diseases of the circulatory system	690649	Chronic deep venous thrombosis of upper extremity due to CABG, initial encounter (HCC)	ICD10CM	2	I82.729	T81.72XA,I82.729,Z95.1	Chronic embolism and thrombosis of deep veins of unsp	IMO	GHS-PACDC	IMO_2015_R	ACTIVE	N	2016-09-17	9999-12-31	2016-09-30
Diagnosis: ICD10 Code, I00-I99, Define	I00-I99	Diseases of the circulatory system	1132530	Embolism from right lesser saphenous vein	ICD10CM	1	I82.811		Embolism and thrombosis of superficial veins of right lo	IMO	GHS-PACDC	IMO_2016_R	ACTIVE	N	1900-01-01	9999-12-31	2016-06-02
Diagnosis: ICD10 Code, I00-I99, Define	I00-I99	Diseases of the circulatory system	835067	Embolism and thrombosis of superficial veins, right	ICD10CM	1	I82.811		Embolism and thrombosis of superficial veins of right lo	IMO	GHS-PACDC	IMO_2015_R	DELETED	Y	1900-01-01	9999-12-31	2015-07-10
Diagnosis: ICD10 Code, I00-I99, Define	I00-I99	Diseases of the circulatory system	722951	Venous embolism and thrombosis of superficial vessels of lower extremity, left	ICD10CM	1	I82.812		Embolism and thrombosis of superficial veins of left low	IMO	GHS-PACDC	IMO_2015_R	ACTIVE	N	1900-01-01	9999-12-31	2015-07-10
Diagnosis: ICD10 Code, I00-I99, Define	I00-I99	Diseases of the circulatory system	816578	Superficial thrombosis of lower extremity, left	ICD10CM	1	I82.812		Embolism and thrombosis of superficial veins of left low	IMO	GHS-PACDC	IMO_2015_R	ACTIVE	N	1900-01-01	9999-12-31	2015-07-10
Diagnosis: ICD10 Code, I00-I99, Define	I00-I99	Diseases of the circulatory system	1365882	Embolism and thombos of superfic veins of low extrm, bi	ICD10CM	1	I82.813		Embolism and thrombosis of superficial veins of lower e	IMO	GHS-PACDC	IMO_2018_R	ACTIVE	N	1900-01-01	9999-12-31	2017-10-30
Diagnosis: ICD10 Code, I00-I99, Define	I00-I99	Diseases of the circulatory system	373367	Chronic superficial venous thrombosis of lower extremity	ICD10CM	1	I82.819		Embolism and thrombosis of superficial veins of unspeci	IMO	GHS-PACDC	DX_LEGACY	ACTIVE	N	1900-01-01	9999-12-31	2015-07-10
Diagnosis: ICD10 Code, I00-I99, Define	I00-I99	Diseases of the circulatory system	1366150	Embolism and thrombosis of superficial vn unsp low extrm	ICD10CM	1	I82.819		Embolism and thrombosis of superficial veins of unspeci	IMO	GHS-PACDC	IMO_2018_R	ACTIVE	N	1900-01-01	9999-12-31	2017-10-30
Diagnosis: ICD10 Code, I00-I99, Define	I00-I99	Diseases of the circulatory system	215265	Lesser saphenous vein embolism	ICD10CM	1	I82.819		Embolism and thrombosis of superficial veins of unspeci	IMO	GHS-PACDC	DX_LEGACY	ACTIVE	N	1900-01-01	9999-12-31	2015-07-10
Diagnosis: ICD10 Code, I00-I99, Define	I00-I99	Diseases of the circulatory system	744606	Greater saphenous vein embolism, unspecified laterality	ICD10CM	1	I82.819		Embolism and thrombosis of superficial veins of unspeci	IMO	GHS-PACDC	IMO_2015_R	ACTIVE	N	1900-01-01	9999-12-31	2015-07-10
Diagnosis: ICD10 Code, I00-I99, Define	I00-I99	Diseases of the circulatory system	729169	Acute superficial venous thrombosis of lower extremity, unspecified laterality	ICD10CM	1	I82.819		Embolism and thrombosis of superficial veins of unspeci	IMO	GHS-PACDC	IMO_2015_R	ACTIVE	N	1900-01-01	9999-12-31	2015-07-10
Diagnosis: ICD10 Code, I00-I99, Define	I00-I99	Diseases of the circulatory system	479738	Thrombosis of right internal and external jugular veins (HCC)	ICD10CM	2	I82.890	I82.C11,I82.890	Acute embolism and thrombosis of other specified veins	IMO	GHS-PACDC	IMO_2015_R	ACTIVE	N	1900-01-01	9999-12-31	2015-07-10
Diagnosis: ICD10 Code, I00-I99, Define	I00-I99	Diseases of the circulatory system	1132822	Chronic deep vein thrombosis (DVT) of pelvic vein	ICD10CM	1	I82.891		Chronic embolism and thrombosis of other specified vei	IMO	GHS-PACDC	IMO_2016_R	ACTIVE	N	1900-01-01	9999-12-31	2016-06-02
Diagnosis: ICD10 Code, I00-I99, Define	I00-I99	Diseases of the circulatory system	411438	Deep venous thrombosis of pelvic vein	ICD10CM	1	I82.90		Acute embolism and thrombosis of unspecified vein	IMO	GHS-PACDC	DX_LEGACY	ACTIVE	N	1900-01-01	9999-12-31	2015-07-10
Diagnosis: ICD10 Code, I00-I99, Define	I00-I99	Diseases of the circulatory system	116040	Venous thrombosis after immobility	ICD10CM	1	I82.90		Acute embolism and thrombosis of unspecified vein	IMO	GHS-PACDC	DX_LEGACY	ACTIVE	N	1900-01-01	9999-12-31	2015-07-10
Diagnosis: ICD10 Code, I00-I99, Define	I00-I99	Diseases of the circulatory system	532603	Discharged to home with confirmed venous thromboembolism (VTE)	ICD10CM	1	I82.90		Acute embolism and thrombosis of unspecified vein	IMO	GHS-PACDC	IMO_2015_R	ACTIVE	N	1900-01-01	9999-12-31	2015-07-10
Diagnosis: ICD10 Code, I00-I99, Define	I00-I99	Diseases of the circulatory system	527018	Thrombosis affecting pregnancy, childbirth, or puerperium	ICD10CM	2	I82.90	O22.20,I82.90	Acute embolism and thrombosis of unspecified vein	IMO	GHS-PACDC	IMO_2015_R	DELETED	Y	1900-01-01	2016-10-14	2015-07-10
Diagnosis: ICD10 Code, I00-I99, Define	I00-I99	Diseases of the circulatory system	502933	Thrombosis of superficial vessel	ICD10CM	1	I82.90		Acute embolism and thrombosis of unspecified vein	IMO	GHS-PACDC	IMO_2015_R	ACTIVE	N	1900-01-01	9999-12-31	2015-07-10
Diagnosis: ICD10 Code, I00-I99, Define	I00-I99	Diseases of the circulatory system	482715	Prophylactic use of unfractionated heparin for venous thromboembolism	ICD10CM	1	I82.90		Acute embolism and thrombosis of unspecified vein	IMO	GHS-PACDC	IMO_2015_R	ACTIVE	N	1900-01-01	9999-12-31	2015-07-10
Diagnosis: ICD10 Code, I00-I99, Define	I00-I99	Diseases of the circulatory system	1127712	Deep vein thrombosis (DVT) of non-extremity vein	ICD10CM	1	I82.90		Acute embolism and thrombosis of unspecified vein	IMO	GHS-PACDC	IMO_2016_R	ACTIVE	N	1900-01-01	9999-12-31	2016-06-02
Diagnosis: ICD10 Code, I00-I99, Define	I00-I99	Diseases of the circulatory system	38709	Ball thrombus	ICD10CM	1	I82.90		Acute embolism and thrombosis of unspecified vein	IMO	GHS-PACDC	DX_LEGACY	ACTIVE	N	1900-01-01	9999-12-31	2015-07-10
Diagnosis: ICD10 Code, I00-I99, Define	I00-I99	Diseases of the circulatory system	214029	Thrombosis complicating pregnancy and/or puerperium	ICD10CM	3	I82.90	O22.20,I82.91,I82.90	Acute embolism and thrombosis of unspecified vein	IMO	GHS-PACDC	DX_LEGACY	DELETED	Y	2016-04-10	9999-12-31	2015-07-10
Diagnosis: ICD10 Code, I00-I99, Define	I00-I99	Diseases of the circulatory system	492540	Venous thromboembolism in patient 41 to 60 years of age	ICD10CM	1	I82.91		Chronic embolism and thrombosis of unspecified vein	IMO	GHS-PACDC	IMO_2015_R	ACTIVE	N	1900-01-01	9999-12-31	2015-07-10
Diagnosis: ICD10 Code, I00-I99, Define	I00-I99	Diseases of the circulatory system	385796	DVT of axillary vein, acute left (HCC)	ICD10CM	1	I82.A12		Acute embolism and thrombosis of left axillary vein	IMO	GHS-PACDC	DX_LEGACY	ACTIVE	N	2016-04-10	9999-12-31	2015-07-10
Diagnosis: ICD10 Code, I00-I99, Define	I00-I99	Diseases of the circulatory system	1329163	Traumatic thrombosis of axillary vein, left (HCC)	ICD10CM	1	I82.A12		Acute embolism and thrombosis of left axillary vein	IMO	GHS-PACDC	IMO_2017_R	ACTIVE	N	1900-01-01	9999-12-31	2017-04-14
Diagnosis: ICD10 Code, I00-I99, Define	I00-I99	Diseases of the circulatory system	477042	Venous embolism and thrombosis of left axillary veins (HCC)	ICD10CM	1	I82.A12		Acute embolism and thrombosis of left axillary vein	IMO	GHS-PACDC	IMO_2015_R	ACTIVE	N	1900-01-01	9999-12-31	2015-07-10
Diagnosis: ICD10 Code, I00-I99, Define	I00-I99	Diseases of the circulatory system	1225900	Acute deep vein thrombosis (DVT) of axillary vein, unspecified laterality (HCC)	ICD10CM	1	I82.A19		Acute embolism and thrombosis of unspecified axillary v	IMO	GHS-PACDC	IMO_2016_R	ACTIVE	N	1900-01-01	9999-12-31	2016-06-02
Diagnosis: ICD10 Code, I00-I99, Define	I00-I99	Diseases of the circulatory system	215724	Axillary vein thrombosis (HCC)	ICD10CM	1	I82.A19		Acute embolism and thrombosis of unspecified axillary v	IMO	GHS-PACDC	DX_LEGACY	ACTIVE	N	2016-04-10	9999-12-31	2015-07-10
Diagnosis: ICD10 Code, I00-I99, Define	I00-I99	Diseases of the circulatory system	809928	Chronic venous embolism and thrombosis of axillary veins, left (HCC)	ICD10CM	1	I82.A22		Chronic embolism and thrombosis of left axillary vein	IMO	GHS-PACDC	IMO_2015_R	ACTIVE	N	1900-01-01	9999-12-31	2015-07-10
Diagnosis: ICD10 Code, I00-I99, Define	I00-I99	Diseases of the circulatory system	776772	Axillary vein thromboembolism, chronic, unspecified laterality (HCC)	ICD10CM	1	I82.A29		Chronic embolism and thrombosis of unspecified axillary	IMO	GHS-PACDC	IMO_2015_R	ACTIVE	N	1900-01-01	9999-12-31	2015-07-10
Diagnosis: ICD10 Code, I00-I99, Define	I00-I99	Diseases of the circulatory system	1132811	Chronic deep vein thrombosis (DVT) of axillary vein (HCC)	ICD10CM	1	I82.A29		Chronic embolism and thrombosis of unspecified axillary	IMO	GHS-PACDC	IMO_2016_R	ACTIVE	N	1900-01-01	9999-12-31	2016-06-02
Diagnosis: ICD10 Code, I00-I99, Define	I00-I99	Diseases of the circulatory system	808563	Acute subclavian vein thrombosis, right (HCC)	ICD10CM	1	I82.B11		Acute embolism and thrombosis of right subclavian vein	IMO	GHS-PACDC	IMO_2015_R	ACTIVE	N	1900-01-01	9999-12-31	2015-07-10
Diagnosis: ICD10 Code, I00-I99, Define	I00-I99	Diseases of the circulatory system	589697	Acute embolism and thrombosis of left subclavian vein	ICD10CM	1	I82.B12		Acute embolism and thrombosis of left subclavian vein	IMO	GHS-PACDC	IMO_2015_R	ACTIVE	Y	1900-01-01	9999-12-31	2015-07-10

Diagnosis: ICD10 Code, I00-I99, Define	I00-I99	Diseases of the circulatory system	887692	Acute subclavian vein thrombosis, left (HCC)	ICD10CM	1	I82.B12		Acute embolism and thrombosis of left subclavian vein	IMO	GHS-PACDC	IMO_2015_R	ACTIVE	N	1900-01-01	9999-12-31	2015-07-10
Diagnosis: ICD10 Code, I00-I99, Define	I00-I99	Diseases of the circulatory system	302972	Acute thrombosis of left subclavian vein (HCC)	ICD10CM	1	I82.B12		Acute embolism and thrombosis of left subclavian vein	IMO	GHS-PACDC	DX_LEGACY	ACTIVE	N	1900-01-01	9999-12-31	2015-07-10
Diagnosis: ICD10 Code, I00-I99, Define	I00-I99	Diseases of the circulatory system	925225	Subclavian vein thromboembolism, acute, bilateral (HCC)	ICD10CM	1	I82.B13		Acute embolism and thrombosis of subclavian vein, bilat	IMO	GHS-PACDC	IMO_2015_R	ACTIVE	N	1900-01-01	9999-12-31	2015-07-10
Diagnosis: ICD10 Code, I00-I99, Define	I00-I99	Diseases of the circulatory system	294679	Acute embolism and thrombosis of subclavian vein, bilateral (HCC)	ICD10CM	1	I82.B13		Acute embolism and thrombosis of subclavian vein, bilat	IMO	GHS-PACDC	DX_LEGACY	ACTIVE	N	1900-01-01	9999-12-31	2015-07-10
Diagnosis: ICD10 Code, I00-I99, Define	I00-I99	Diseases of the circulatory system	476032	Thrombosis of both subclavian veins (HCC)	ICD10CM	1	I82.B13		Acute embolism and thrombosis of subclavian vein, bilat	IMO	GHS-PACDC	IMO_2015_R	ACTIVE	N	1900-01-01	9999-12-31	2015-07-10
Diagnosis: ICD10 Code, I00-I99, Define	I00-I99	Diseases of the circulatory system	599358	Acute embolism and thrombosis of unspecified subclavian vein	ICD10CM	1	I82.B19		Acute embolism and thrombosis of unspecified subclavi	IMO	GHS-PACDC	IMO_2015_R	ACTIVE	Y	1900-01-01	9999-12-31	2015-07-10
Diagnosis: ICD10 Code, I00-I99, Define	I00-I99	Diseases of the circulatory system	809253	Internal jugular (IJ) vein thromboembolism, acute, right (HCC)	ICD10CM	1	I82.C11		Acute embolism and thrombosis of right internal jugular	IMO	GHS-PACDC	IMO_2015_R	ACTIVE	N	1900-01-01	9999-12-31	2015-07-10
Diagnosis: ICD10 Code, I00-I99, Define	I00-I99	Diseases of the circulatory system	779231	Acute embolism and thrombosis of internal jugular vein, right (HCC)	ICD10CM	1	I82.C11		Acute embolism and thrombosis of right internal jugular	IMO	GHS-PACDC	IMO_2015_R	ACTIVE	N	1900-01-01	9999-12-31	2015-07-10
Diagnosis: ICD10 Code, I00-I99, Define	I00-I99	Diseases of the circulatory system	864397	Acute venous embolism and thrombosis of internal jugular veins, left (HCC)	ICD10CM	1	I82.C12		Acute embolism and thrombosis of left internal jugular v	IMO	GHS-PACDC	IMO_2015_R	ACTIVE	N	1900-01-01	9999-12-31	2015-07-10
Diagnosis: ICD10 Code, I00-I99, Define	I00-I99	Diseases of the circulatory system	475464	Thrombosis of both internal jugular veins (HCC)	ICD10CM	1	I82.C13		Acute embolism and thrombosis of internal jugular vein,	IMO	GHS-PACDC	IMO_2015_R	ACTIVE	N	1900-01-01	9999-12-31	2015-07-10
Diagnosis: ICD10 Code, I00-I99, Define	I00-I99	Diseases of the circulatory system	215424	Venous embolism and thrombosis of internal jugular veins (HCC)	ICD10CM	1	I82.C19		Acute embolism and thrombosis of unspecified internal	IMO	GHS-PACDC	DX_LEGACY	ACTIVE	N	1900-01-01	9999-12-31	2015-07-10
Diagnosis: ICD10 Code, I00-I99, Define	I00-I99	Diseases of the circulatory system	269845	Chronic embolism and thrombosis of left internal jugular vein (HCC)	ICD10CM	1	I82.C22		Chronic embolism and thrombosis of left internal jugula	IMO	GHS-PACDC	DX_LEGACY	ACTIVE	N	1900-01-01	9999-12-31	2015-07-10
Diagnosis: ICD10 Code, I00-I99, Define	I00-I99	Diseases of the circulatory system	476375	Chronic embolism of both internal jugular veins	ICD10CM	1	I82.C23		Chronic embolism and thrombosis of internal jugular vei	IMO	GHS-PACDC	IMO_2015_R	DELETED	Y	1900-01-01	2016-05-07	2015-07-10
Diagnosis: ICD10 Code, I00-I99, Define	I00-I99	Diseases of the circulatory system	17983	Internal jugular (IJ) vein thromboembolism, chronic (HCC)	ICD10CM	1	I82.C29		Chronic embolism and thrombosis of unspecified interna	IMO	GHS-PACDC	DX_LEGACY	ACTIVE	N	1900-01-01	9999-12-31	2015-07-10
Diagnosis: ICD10 Code, I00-I99, Define	I00-I99	Diseases of the circulatory system	1427705	Venous stasis ulcer of thigh, unspecified laterality, unspecified ulcer stage, unspecified whether	ICD10CM	1	I83.001	I83.001,L97.109	Varicose veins of unspecified lower extremity with ulcer	IMO	GHS-PACDC	IMO_2018_R	ACTIVE	N	1900-01-01	9999-12-31	2017-10-30
Diagnosis: ICD10 Code, I00-I99, Define	I00-I99	Diseases of the circulatory system	1320830	Venous stasis ulcer of thigh with necrosis of muscle with varicose veins (HCC)	ICD10CM	1	I83.001	I83.001,L97.103	Varicose veins of unspecified lower extremity with ulcer	IMO	GHS-PACDC	IMO_2017_R	ACTIVE	N	1900-01-01	9999-12-31	2017-04-14
Diagnosis: ICD10 Code, I00-I99, Define	I00-I99	Diseases of the circulatory system	1426942	Venous stasis ulcer of thigh with muscle involvement without evidence of necrosis, unspecified	ICD10CM	1	I83.001	I83.001,L97.105	Varicose veins of unspecified lower extremity with ulcer	IMO	GHS-PACDC	IMO_2018_R	ACTIVE	N	1900-01-01	9999-12-31	2017-10-30
Diagnosis: ICD10 Code, I00-I99, Define	I00-I99	Diseases of the circulatory system	1426941	Venous stasis ulcer of thigh with bone involvement without evidence of necrosis, unspecified la	ICD10CM	1	I83.001	I83.001,L97.106	Varicose veins of unspecified lower extremity with ulcer	IMO	GHS-PACDC	IMO_2018_R	ACTIVE	N	1900-01-01	9999-12-31	2017-10-30
Diagnosis: ICD10 Code, I00-I99, Define	I00-I99	Diseases of the circulatory system	276446	Varicose veins of lower extremity with ulcer of thigh (HCC)	ICD10CM	1	I83.001		Varicose veins of unspecified lower extremity with ulcer	IMO	GHS-PACDC	DX_LEGACY	ACTIVE	N	1900-01-01	9999-12-31	2015-07-10
Diagnosis: ICD10 Code, I00-I99, Define	I00-I99	Diseases of the circulatory system	1427675	Venous stasis ulcer of calf limited to breakdown of skin, unspecified laterality, unspecified whet	ICD10CM	1	I83.002	I83.002,L97.201	Varicose veins of unspecified lower extremity with ulcer	IMO	GHS-PACDC	IMO_2018_R	ACTIVE	N	1900-01-01	9999-12-31	2017-10-30
Diagnosis: ICD10 Code, I00-I99, Define	I00-I99	Diseases of the circulatory system	1355208	Venous stasis ulcer of calf with bone involvement without evidence of necrosis with varicose ve	ICD10CM	1	I83.002	I83.002,L97.206	Varicose veins of unspecified lower extremity with ulcer	IMO	GHS-PACDC	IMO_2018_R	ACTIVE	N	1900-01-01	9999-12-31	2017-10-30
Diagnosis: ICD10 Code, I00-I99, Define	I00-I99	Diseases of the circulatory system	1426923	Venous stasis ulcer of ankle with muscle involvement without evidence of necrosis, unspecified	ICD10CM	1	I83.003	I83.003,L97.305	Varicose veins of unspecified lower extremity with ulcer	IMO	GHS-PACDC	IMO_2018_R	ACTIVE	N	1900-01-01	9999-12-31	2017-10-30
Diagnosis: ICD10 Code, I00-I99, Define	I00-I99	Diseases of the circulatory system	1355335	Venous stasis ulcer of ankle with bone involvement without evidence of necrosis (HCC)	ICD10CM	1	I83.003	I83.003,L97.306	Varicose veins of unspecified lower extremity with ulcer	IMO	GHS-PACDC	IMO_2018_R	ACTIVE	N	1900-01-01	9999-12-31	2017-10-30
Diagnosis: ICD10 Code, I00-I99, Define	I00-I99	Diseases of the circulatory system	1427681	Venous stasis ulcer of heel with necrosis of bone, unspecified laterality, unspecified whether va	ICD10CM	1	I83.004	I83.004,L97.404	Varicose veins of unspecified lower extremity with ulcer	IMO	GHS-PACDC	IMO_2018_R	ACTIVE	N	1900-01-01	9999-12-31	2017-10-30
Diagnosis: ICD10 Code, I00-I99, Define	I00-I99	Diseases of the circulatory system	1321067	Venous stasis ulcer of heel with fat layer exposed with varicose veins (HCC)	ICD10CM	1	I83.004	I83.004,L97.402	Varicose veins of unspecified lower extremity with ulcer	IMO	GHS-PACDC	IMO_2017_R	ACTIVE	N	1900-01-01	9999-12-31	2017-04-14
Diagnosis: ICD10 Code, I00-I99, Define	I00-I99	Diseases of the circulatory system	1427573	Venous stasis ulcer of midfoot with varicose veins, unspecified laterality, unspecified ulcer stage	ICD10CM	1	I83.004	I83.004,L97.409	Varicose veins of unspecified lower extremity with ulcer	IMO	GHS-PACDC	IMO_2018_R	ACTIVE	N	1900-01-01	9999-12-31	2017-10-30
Diagnosis: ICD10 Code, I00-I99, Define	I00-I99	Diseases of the circulatory system	1426851	Venous stasis ulcer of heel with other ulcer severity with varicose veins, unspecified laterality (H	ICD10CM	1	I83.004	I83.004,L97.408	Varicose veins of unspecified lower extremity with ulcer	IMO	GHS-PACDC	IMO_2018_R	ACTIVE	N	1900-01-01	9999-12-31	2017-10-30
Diagnosis: ICD10 Code, I00-I99, Define	I00-I99	Diseases of the circulatory system	1427682	Venous stasis ulcer of heel with necrosis of muscle, unspecified laterality, unspecified whether v	ICD10CM	1	I83.004	I83.004,L97.403	Varicose veins of unspecified lower extremity with ulcer	IMO	GHS-PACDC	IMO_2018_R	ACTIVE	N	1900-01-01	9999-12-31	2017-10-30
Diagnosis: ICD10 Code, I00-I99, Define	I00-I99	Diseases of the circulatory system	1426861	Venous stasis ulcer of other part of foot with muscle involvement without evidence of necrosis v	ICD10CM	1	I83.005	I83.005,L97.505	Varicose veins of unspecified lower extremity with ulcer	IMO	GHS-PACDC	IMO_2018_R	ACTIVE	N	1900-01-01	9999-12-31	2017-10-30
Diagnosis: ICD10 Code, I00-I99, Define	I00-I99	Diseases of the circulatory system	641709	Varicos vn unsp lower extremity w ulcer oth part of foot	ICD10CM	1	I83.005		Varicose veins of unspecified lower extremity with ulcer	IMO	GHS-PACDC	IMO_2015_R	DELETED	Y	1900-01-01	9999-12-31	2015-07-10
Diagnosis: ICD10 Code, I00-I99, Define	I00-I99	Diseases of the circulatory system	1183180	Varicose veins of unspecified lower extremity with ulcer other part of lower leg (HCC)	ICD10CM	1	I83.008		Varicose veins of unspecified lower extremity with ulcer	IMO	GHS-PACDC	IMO_2016_R	ACTIVE	N	1900-01-01	9999-12-31	2016-06-02
Diagnosis: ICD10 Code, I00-I99, Define	I00-I99	Diseases of the circulatory system	1427692	Venous stasis ulcer of other part of lower leg with fat layer exposed, unspecified laterality, unsp	ICD10CM	1	I83.008	I83.008,L97.802	Varicose veins of unspecified lower extremity with ulcer	IMO	GHS-PACDC	IMO_2018_R	ACTIVE	N	1900-01-01	9999-12-31	2017-10-30
Diagnosis: ICD10 Code, I00-I99, Define	I00-I99	Diseases of the circulatory system	1427704	Venous stasis ulcer of other part of lower leg, unspecified laterality, unspecified ulcer stage, uns	ICD10CM	1	I83.008	I83.008,L97.809	Varicose veins of unspecified lower extremity with ulcer	IMO	GHS-PACDC	IMO_2018_R	ACTIVE	N	1900-01-01	9999-12-31	2017-10-30
Diagnosis: ICD10 Code, I00-I99, Define	I00-I99	Diseases of the circulatory system	1259701	Skin ulcer due to varicose veins (HCC)	ICD10CM	1	I83.009	I83.009,L98.499	Varicose veins of unspecified lower extremity with ulcer	IMO	GHS-PACDC	IMO_2017_R	ACTIVE	N	1900-01-01	2017-10-14	2016-09-30
Diagnosis: ICD10 Code, I00-I99, Define	I00-I99	Diseases of the circulatory system	145923	Varicose veins of lower extremity with stasis ulcer (HCC)	ICD10CM	1	I83.009	I83.009,L97.909	Varicose veins of unspecified lower extremity with ulcer	IMO	GHS-PACDC	DX_LEGACY	ACTIVE	N	1900-01-01	2016-05-07	2015-07-10
Diagnosis: ICD10 Code, I00-I99, Define	I00-I99	Diseases of the circulatory system	1114662	Venous stasis ulcer with edema of lower leg (HCC)	ICD10CM	1	I83.009	I83.009,R60.9	Varicose veins of unspecified lower extremity with ulcer	IMO	GHS-PACDC	IMO_2016_R	ACTIVE	N	1900-01-01	9999-12-31	2016-06-02
Diagnosis: ICD10 Code, I00-I99, Define	I00-I99	Diseases of the circulatory system	329561	Ulcerated leg varices (HCC)	ICD10CM	1	I83.009		Varicose veins of unspecified lower extremity with ulcer	IMO	GHS-PACDC	DX_LEGACY	ACTIVE	N	1900-01-01	9999-12-31	2015-07-10
Diagnosis: ICD10 Code, I00-I99, Define	I00-I99	Diseases of the circulatory system	1427733	Venous stasis ulcer with necrosis of muscle, unspecified site, unspecified whether varicose veins	ICD10CM	1	I83.009	I83.009,L97.903	Varicose veins of unspecified lower extremity with ulcer	IMO	GHS-PACDC	IMO_2018_R	ACTIVE	N	1900-01-01	9999-12-31	2017-10-30
Diagnosis: ICD10 Code, I00-I99, Define	I00-I99	Diseases of the circulatory system	94120	Leg varicosity w/ ulcer (HCC)	ICD10CM	1	I83.009	I83.009,L97.909	Varicose veins of unspecified lower extremity with ulcer	IMO	GHS-PACDC	DX_LEGACY	ACTIVE	N	1900-01-01	2016-05-07	2015-07-10
Diagnosis: ICD10 Code, I00-I99, Define	I00-I99	Diseases of the circulatory system	1320883	Venous stasis ulcer of right thigh with varicose veins (HCC)	ICD10CM	1	I83.011	I83.011,L97.119	Varicose veins of right lower extremity with ulcer of thig	IMO	GHS-PACDC	IMO_2017_R	ACTIVE	N	1900-01-01	9999-12-31	2017-04-14
Diagnosis: ICD10 Code, I00-I99, Define	I00-I99	Diseases of the circulatory system	801909	Varicose veins of lower extremity with ulcer of thigh, right (HCC)	ICD10CM	1	I83.011	I83.011,I83.012,I83.013,I83.014,I	Varicose veins of right lower extremity with ulcer of thig	IMO	GHS-PACDC	IMO_2015_R	ACTIVE	N	1900-01-01	2016-05-07	2015-07-10
Diagnosis: ICD10 Code, I00-I99, Define	I00-I99	Diseases of the circulatory system	866694	Stasis ulcer, right (HCC)	ICD10CM	1	I83.011	I83.011,I83.012,I83.013,I83.014,I	Varicose veins of right lower extremity with ulcer of thig	IMO	GHS-PACDC	IMO_2015_R	ACTIVE	N	1900-01-01	2016-05-07	2015-07-10
Diagnosis: ICD10 Code, I00-I99, Define	I00-I99	Diseases of the circulatory system	795871	Ulcerated leg varices, right (HCC)	ICD10CM	2	I83.012	I83.011,I83.012,I83.013,I83.014,I	Varicose veins of right lower extremity with ulcer of calf	IMO	GHS-PACDC	IMO_2015_R	ACTIVE	N	1900-01-01	2016-05-07	2015-07-10
Diagnosis: ICD10 Code, I00-I99, Define	I00-I99	Diseases of the circulatory system	839544	Stasis leg ulcer, right (HCC)	ICD10CM	2	I83.012	I83.011,I83.012,I83.013,I83.014,I	Varicose veins of right lower extremity with ulcer of calf	IMO	GHS-PACDC	IMO_2015_R	ACTIVE	N	1900-01-01	2016-05-07	2015-07-10
Diagnosis: ICD10 Code, I00-I99, Define	I00-I99	Diseases of the circulatory system	1427717	Venous stasis ulcer of right calf, unspecified ulcer stage, unspecified whether varicose veins pres	ICD10CM	1	I83.012	I83.012,L97.219	Varicose veins of right lower extremity with ulcer of calf	IMO	GHS-PACDC	IMO_2018_R	ACTIVE	N	1900-01-01	9999-12-31	2017-10-30
Diagnosis: ICD10 Code, I00-I99, Define	I00-I99	Diseases of the circulatory system	1320888	Venous stasis ulcer of right calf with fat layer exposed (HCC)	ICD10CM	1	I83.012	I83.012,L97.212	Varicose veins of right lower extremity with ulcer of calf	IMO	GHS-PACDC	IMO_2017_R	ACTIVE	N	1900-01-01	9999-12-31	2017-04-14
Diagnosis: ICD10 Code, I00-I99, Define	I00-I99	Diseases of the circulatory system	759521	Varicose veins of lower extremity with ulcer of calf, right (HCC)	ICD10CM	2	I83.012	I83.011,I83.012,I83.013,I83.014,I	Varicose veins of right lower extremity with ulcer of calf	IMO	GHS-PACDC	IMO_2015_R	ACTIVE	N	1900-01-01	2016-05-07	2015-07-10
Diagnosis: ICD10 Code, I00-I99, Define	I00-I99	Diseases of the circulatory system	740811	Ulcerated varicose veins of leg, right (HCC)	ICD10CM	2	I83.012	I83.011,I83.012,I83.013,I83.014,I	Varicose veins of right lower extremity with ulcer of calf	IMO	GHS-PACDC	IMO_2015_R	ACTIVE	N	1900-01-01	2016-05-07	2015-07-10
Diagnosis: ICD10 Code, I00-I99, Define	I00-I99	Diseases of the circulatory system	1320950	Venous stasis ulcer of right ankle with necrosis of muscle with varicose veins (HCC)	ICD10CM	1	I83.013	I83.013,L97.313	Varicose veins of right lower extremity with ulcer of ank	IMO	GHS-PACDC	IMO_2017_R	ACTIVE	N	1900-01-01	9999-12-31	2017-04-14
Diagnosis: ICD10 Code, I00-I99, Define	I00-I99	Diseases of the circulatory system	1427654	Venous stasis ulcer of right ankle with necrosis of muscle, unspecified whether varicose veins pr	ICD10CM	1	I83.013	I83.013,L97.313	Varicose veins of right lower extremity with ulcer of ank	IMO	GHS-PACDC	IMO_2018_R	ACTIVE	N	1900-01-01	9999-12-31	2017-10-30
Diagnosis: ICD10 Code, I00-I99, Define	I00-I99	Diseases of the circulatory system	1321058	Venous stasis ulcer of right ankle with fat layer exposed with varicose veins (HCC)	ICD10CM	1	I83.013	I83.013,L97.312	Varicose veins of right lower extremity with ulcer of ank	IMO	GHS-PACDC	IMO_2017_R	ACTIVE	N	1900-01-01	9999-12-31	2017-04-14
Diagnosis: ICD10 Code, I00-I99, Define	I00-I99	Diseases of the circulatory system	447928	Stasis ulcer of right ankle (HCC)	ICD10CM	1	I83.013		Varicose veins of right lower extremity with ulcer of ank	IMO	GHS-PACDC	IMO_2015_R	ACTIVE	N	1900-01-01	9999-12-31	2015-07-10
Diagnosis: ICD10 Code, I00-I99, Define	I00-I99	Diseases of the circulatory system	1320796	Venous stasis ulcer of right midfoot with necrosis of muscle with varicose veins (HCC)	ICD10CM	1	I83.014	I83.014,L97.413	Varicose veins of right lower extremity with ulcer of hee	IMO	GHS-PACDC	IMO_2017_R	ACTIVE	N	1900-01-01	9999-12-31	2017-04-14
Diagnosis: ICD10 Code, I00-I99, Define	I00-I99	Diseases of the circulatory system	866694	Stasis ulcer, right (HCC)	ICD10CM	4	I83.014	I83.011,I83.012,I83.013,I83.014,I	Varicose veins of right lower extremity with ulcer of hee	IMO	GHS-PACDC	IMO_2015_R	ACTIVE	N	1900-01-01	2016-05-07	2015-07-10
Diagnosis: ICD10 Code, I00-I99, Define	I00-I99	Diseases of the circulatory system	762936	Varicose veins of lower extremity with ulcer of ankle, right (HCC)	ICD10CM	4	I83.014	I83.011,I83.012,I83.013,I83.014,I	Varicose veins of right lower extremity with ulcer of hee	IMO	GHS-PACDC	IMO_2015_R	ACTIVE	N	1900-01-01	2016-05-07	2015-07-10
Diagnosis: ICD10 Code, I00-I99, Define	I00-I99	Diseases of the circulatory system	747784	Ulcer of varicose vein of leg, right (HCC)	ICD10CM	4	I83.014	I83.011,I83.012,I83.013,I83.014,I	Varicose veins of right lower extremity with ulcer of hee	IMO	GHS-PACDC	IMO_2015_R	ACTIVE	N	1900-01-01	2016-05-07	2015-07-10
Diagnosis: ICD10 Code, I00-I99, Define	I00-I99	Diseases of the circulatory system	740818	Varicose veins of lower extremity with stasis ulcer, right (HCC)	ICD10CM	5	I83.015	I83.011,I83.012,I83.013,I83.014,I	Varicose veins of right lower extremity with ulcer other	IMO	GHS-PACDC	IMO_2015_R	ACTIVE	N	1900-01-01	2016-05-07	2015-07-10
Diagnosis: ICD10 Code, I00-I99, Define	I00-I99	Diseases of the circulatory system	1427639	Venous stasis ulcer of other part of right foot limited to breakdown of skin, unspecified whether	ICD10CM	1	I83.015	I83.015,L97.511	Varicose veins of right lower extremity with ulcer other	IMO	GHS-PACDC	IMO_2018_R	ACTIVE	N	1900-01-01	9999-12-31	2017-10-30
Diagnosis: ICD10 Code, I00-I99, Define	I00-I99	Diseases of the circulatory system	871804	Varicose veins of lower extremities with ulcer, right (HCC)	ICD10CM	1	I83.019		Varicose veins of right lower extremity with ulcer of uns	IMO	GHS-PACDC	IMO_2015_R	ACTIVE	N	2016-04-10	9999-12-31	2015-07-10
Diagnosis: ICD10 Code, I00-I99, Define	I00-I99	Diseases of the circulatory system	774869	Gravitational ulcer, right (HCC)	ICD10CM	7	I83.019	I83.011,I83.012,I83.013,I83.014,I	Varicose veins of right lower extremity with ulcer of uns	IMO	GHS-PACDC	IMO_2015_R	ACTIVE	N	1900-01-01	2016-05-07	2015-07-10
Diagnosis: ICD10 Code, I00-I99, Define	I00-I99	Diseases of the circulatory system	834546	Varicose veins with ulcer, right (HCC)	ICD10CM	1	I83.019		Varicose veins of right lower extremity with ulcer of uns	IMO	GHS-PACDC	IMO_2015_R	ACTIVE	N	2016-04-10	9999-12-31	2015-07-10
Diagnosis: ICD10 Code, I00-I99, Define	I00-I99	Diseases of the circulatory system	867213	Varicose ulceration, right (HCC)	ICD10CM	7	I83.019	I83.011,I83.012,I83.013,I83.014,I	Varicose veins of right lower extremity with ulcer of uns	IMO	GHS-PACDC	IMO_2015_R	ACTIVE	N	1900-01-01	2016-05-07	2015-07-10
Diagnosis: ICD10 Code, I00-I99, Define	I00-I99	Diseases of the circulatory system	1427627	Venous stasis ulcer of left thigh limited to breakdown of skin, unspecified whether varicose vein	ICD10CM	1	I83.021	I83.021,L97.121	Varicose veins of left lower extremity with ulcer of thigh	IMO	GHS-PACDC	IMO_2018_R	ACTIVE	N	1900-01-01	9999-12-31	2017-10-30
Diagnosis: ICD10 Code, I00-I99, Define	I00-I99	Diseases of the circulatory system	719154	Varicose veins with ulcer, left (HCC)	ICD10CM	1	I83.021	I83.021,I83.022,I83.023,I83.024,I	Varicose veins of left lower extremity with ulcer of thigh	IMO	GHS-PACDC	IMO_2015_R	ACTIVE	N	1900-01-01	2016-05-07	2015-07-10
Diagnosis: ICD10 Code, I00-I99, Define	I00-I99	Diseases of the circulatory system	1427629	Venous stasis ulcer of left thigh with necrosis of bone, unspecified whether varicose veins prese	ICD10CM	1	I83.021	I83.021,L97.124	Varicose veins of left lower extremity with ulcer of thigh	IMO	GHS-PACDC	IMO_2018_R	ACTIVE	N	1900-01-01	9999-12-31	2017-10-30
Diagnosis: ICD10 Code, I00-I99, Define	I00-I99	Diseases of the circulatory system	1427429	Venous stasis ulcer of left calf with other ulcer severity with varicose veins (HCC)	ICD10CM	1	I83.022	I83.022,L97.228	Varicose veins of left lower extremity with ulcer of calf	IMO	GHS-PACDC	IMO_2018_R	ACTIVE	N	1900-01-01	9999-12-31	2017-10-30
Diagnosis: ICD10 Code, I00-I99, Define	I00-I99	Diseases of the circulatory system	1355214	Venous stasis ulcer of left calf with bone involvement without evidence of necrosis with varicose	ICD10CM	1	I83.022	I83.022,L97.226	Varicose veins of left lower extremity with ulcer of calf	IMO	GHS-PACDC	IMO_2018_R	ACTIVE	N	1900-01-01	9999-12-31	2017-10-30
Diagnosis: ICD10 Code, I00-I99, Define	I00-I99	Diseases of the circulatory system	719019	Ulcerated varicose veins of leg, left (HCC)	ICD10CM	2	I83.022	I83.021,I83.022,I83.023,I83.024,I	Varicose veins of left lower extremity with ulcer of calf	IMO	GHS-PACDC	IMO_2015_R	ACTIVE	N	1900-01-01	2016-05-07	2015-07-10
Diagnosis: ICD10 Code, I00-I99, Define	I00-I99	Diseases of the circulatory system	773537	Varicose veins of lower extremity with stasis ulcer, left (HCC)	ICD10CM	2	I83.022	I83.021,I83.022,I83.023,I83.024,I	Varicose veins of left lower extremity with ulcer of calf	IMO	GHS-PACDC	IMO_2015_R	ACTIVE	N	1900-01-01	2016-05-07	2015-07-10
Diagnosis: ICD10 Code, I00-I99, Define	I00-I99	Diseases of the circulatory system	719154	Varicose veins with ulcer, left (HCC)	ICD10CM	2	I83.022	I83.021,I83.022,I83.023,I83.024,I	Varicose veins of left lower extremity with ulcer of calf	IMO	GHS-PACDC	IMO_2015_R	ACTIVE	N	1900-01-01	2016-05-07	2015-07-10
Diagnosis: ICD10 Code, I00-I99, Define	I00-I99	Diseases of the circulatory system	1320849	Venous stasis ulcer of left ankle limited to breakdown of skin with varicose veins (HCC)	ICD10CM	1	I83.023	I83.023,L97.321	Varicose veins of left lower extremity with ulcer of ankle	IMO	GHS-PACDC	IMO_2017_R	ACTIVE	N	1900-01-01	9999-12-31	2017-04-14
Diagnosis: ICD10 Code, I00-I99, Define	I00-I99	Diseases of the circulatory system	882468	Stasis ulcer, left (HCC)	ICD10CM	3	I83.023	I83.021,I83.022,I83.023,I83.024,I	Varicose veins of left lower extremity with ulcer of ankle	IMO	GHS-PACDC	IMO_2015_R	ACTIVE	N	1900-01-01	2016-05-07	2015-07-10
Diagnosis: ICD10 Code, I00-I99, Define	I00-I99	Diseases of the circulatory system	877201	Venous stasis ulcer of ankle, left (HCC)	ICD10CM	1	I83.023		Varicose veins of left lower extremity with ulcer of ankle	IMO	GHS-PACDC	IMO_2015_R	ACTIVE	N	2016-04-10	9999-12-31	2015-07-10
Diagnosis: ICD10 Code, I00-I99, Define	I00-I99	Diseases of the circulatory system	1320753	Venous stasis ulcer of left midfoot with varicose veins (HCC)	ICD10CM	1	I83.024	I83.024,L97.429	Varicose veins of left lower extremity with ulcer of heel	IMO	GHS-PACDC	IMO_2017_R	ACTIVE	N	1900-01-01	9999-12-31	2017-04-14
Diagnosis: ICD10 Code, I00-I99, Define	I00-I99	Diseases of the circulatory system	1320893	Venous stasis ulcer of left midfoot with necrosis of muscle (HCC)	ICD10CM	1	I83.024	I83.024,L97.423	Varicose veins of left lower extremity with ulcer of heel	IMO	GHS-PACDC	IMO_2017_R	ACTIVE	N	1900-01-01	9999-12-31	2017-04-14
Diagnosis: ICD10 Code, I00-I99, Define	I00-I99	Diseases of the circulatory system	719154	Varicose veins with ulcer, left (HCC)	ICD10CM	4	I83.024	I83.021,I83.022,I83.023,I83.024,I	Varicose veins of left lower extremity with ulcer of heel	IMO	GHS-PACDC	IMO_2015_R	ACTIVE	N	1900-01-01	2016-05-07	2015-07-10
Diagnosis: ICD10 Code, I00-I99, Define	I00-I99	Diseases of the circulatory system	811744	Varicose ulcer of lower extremity, left (HCC)	ICD10CM	4	I83.024	I83.021,I83.022,I83.023,I83.024,I	Varicose veins of left lower extremity with ulcer of heel	IMO	GHS-PACDC	IMO_2015_R	ACTIVE	N	1900-01-01	2016-05-07	2015-07-10
Diagnosis: ICD10 Code, I00-I99, Define	I00-I99	Diseases of the circulatory system	1321048	Venous stasis ulcer of left heel with varicose veins (HCC)	ICD10CM	1	I83.024	I83.024,L97.429	Varicose veins of left lower extremity with ulcer of heel	IMO	GHS-PACDC	IMO_2017_R	ACTIVE	N	1900-01-01	9999-12-31	2017-04-14
Diagnosis: ICD10 Code, I00-I99, Define	I00-I99	Diseases of the circulatory system	1427621	Venous stasis ulcer of left heel with necrosis of bone, unspecified whether varicose veins preser	ICD10CM	1	I83.024	I83.024,L97.424	Varicose veins of left lower extremity with ulcer of heel	IMO	GHS-PACDC	IMO_2018_R	ACTIVE	N	1900-01-01	9999-12-31	2017-10-30
Diagnosis: ICD10 Code, I00-I99, Define	I00-I99	Diseases of the circulatory system	882330	Gravitational ulcer, left	ICD10CM	4	I83.024	I83.021,I83.022,I83.023,I83.024,I	Varicose veins of left lower extremity with ulcer of heel	IMO	GHS-PACDC	IMO_2015_R	DELETED	Y	1900-01-01	2016-05-07	2015-07-10
Diagnosis: ICD10 Code, I00-I99, Define	I00-I99	Diseases of the circulatory system	822926	Varicose ulcer, left (HCC)	ICD10CM	4	I83.024	I83.021,I83.022,I83.023,I83.024,I	Varicose veins of left lower extremity with ulcer of heel	IMO	GHS-PACDC	IMO_2015_R	ACTIVE	N	1900-01-01	2016-05-07	2015-07-10
Diagnosis: ICD10 Code, I00-I99, Define	I00-I99	Diseases of the circulatory system	1426884	Venous stasis ulcer of left heel with muscle involvement without evidence of necrosis, unspecifi	ICD10CM	1	I83.024	I83.024,L97.425	Varicose veins of left lower extremity with ulcer of heel	IMO	GHS-PACDC	IMO_2018_R	ACTIVE	N	1900-01-01	9999-12-31	2017-10-30
Diagnosis: ICD10 Code, I00-I99, Define	I00-I99	Diseases of the circulatory system	741003	Ulcer, venous stasis, left	ICD10CM	4	I83.024	I83.021,I83.022,I83.023,I83.024,I	Varicose veins of left lower extremity with ulcer of heel	IMO	GHS-PACDC	IMO_2015_R	DELETED	Y	1900-01-01	2016-05-07	2015-07-10
Diagnosis: ICD10 Code, I00-I99, Define	I00-I99	Diseases of the circulatory system	822926	Varicose ulcer, left (HCC)	ICD10CM	5	I83.025	I83.021,I83.022,I83.023,I83.024,I	Varicose veins of left lower extremity with ulcer other p	IMO	GHS-PACDC	IMO_2015_R	ACTIVE	N	1900-01-01	2016-05-07	2015-07-10
Diagnosis: ICD10 Code, I00-I99, Define	I00-I99	Diseases of the circulatory system	779559	Varicose veins of lower extremity with ulcer of thigh, left (HCC)	ICD10CM	6	I83.028	I83.021,I83.022,I83.023,I83.024,I	Varicose veins of left lower extremity with ulcer other p	IMO	GHS-PACDC	IMO_2015_R	ACTIVE	N	1900-01-01	2016-05-07	2015-07-10
Diagnosis: ICD10 Code, I00-I99, Define	I00-I99	Diseases of the circulatory system	746594	Ulcer of varicose vein of leg, left (HCC)	ICD10CM	6	I83.028	I83.021,I83.022,I83.023,I83.024,I	Varicose veins of left lower extremity with ulcer other p	IMO	GHS-PACDC	IMO_2015_R	ACTIVE	N	1900-01-01	2016-05-07	2015-07-10
Diagnosis: ICD10 Code, I00-I99, Define	I00-I99	Diseases of the circulatory system	599576	Varicose veins of left lower extremity with ulcer other part of lower leg	ICD10CM	1	I83.028		Varicose veins of left lower extremity with ulcer other p	IMO	GHS-PACDC	IMO_2015_R	ACTIVE	Y	1900-01-01	9999-12-31	2015-07-10
Diagnosis: ICD10 Code, I00-I99, Define	I00-I99	Diseases of the circulatory system	822926	Varicose ulcer, left (HCC)	ICD10CM	1	I83.029		Varicose veins of left lower extremity with ulcer of unsp	IMO	GHS-PACDC	IMO_2015_R	ACTIVE	N	2016-04-10	9999-12-31	2015-07-10
Diagnosis: ICD10 Code, I00-I99, Define	I00-I99	Diseases of the circulatory system	844956	Venous stasis ulcer, left	ICD10CM	1	I83.029		Varicose veins of left lower extremity with ulcer of unsp	IMO	GHS-PACDC	IMO_2015_R	DELETED	Y	2016-04-10	2017-04-27	2015-07-10
Diagnosis: ICD10 Code, I00-I99, Define	I00-I99	Diseases of the circulatory system	502157	Chronic venous stasis dermatitis	ICD10CM	1	I83.10		Varicose veins of unspecified lower extremity with infla	IMO	GHS-PACDC	IMO_2015_R	ACTIVE	N	1900-01-01	2017-04-27	2015-07-10
Diagnosis: ICD10 Code, I00-I99, Define	I00-I99	Diseases of the circulatory system	806684	Varicose veins with inflammation, right	ICD10CM	1	I83.11		Varicose veins of right lower extremity with inflammatio	IMO	GHS-PACDC	IMO_2015_R	ACTIVE	N	1900-01-01	9999-12-31	2015-07-10
Diagnosis: ICD10 Code, I00-I99, Define	I00-I99	Diseases of the circulatory system	502152	Chronic venous stasis dermatitis of both lower extremities	ICD10CM	1	I83.11	I83.11,I83.12	Varicose veins of right lower extremity with inflammatio	IMO	GHS-PACDC	IMO_2015_R	ACTIVE	N	1900-01-01	2017-04-27	2015-07-10
Diagnosis: ICD10 Code, I00-I99, Define	I00-I99	Diseases of the circulatory system	502149	Acute venous stasis dermatitis of both lower extremities	ICD10CM	2	I83.12	I83.11,I83.12	Varicose veins of left lower extremity with inflammation	IMO	GHS-PACDC	IMO_2015_R	ACTIVE	N	1900-01-01	2017-04-27	2015-07-10
Diagnosis: ICD10 Code, I00-I99, Define	I00-I99	Diseases of the circulatory system	369369	Varicose veins of leg with both ulcer of thigh and inflammation (HCC)	ICD10CM	1	I83.201		Varicose veins of unspecified lower extremity with both	IMO	GHS-PACDC	DX_LEGACY	ACTIVE	N	1900-01-01	9999-12-31	2015-07-10
Diagnosis: ICD10 Code, I00-I99, Define	I00-I99	Diseases of the circulatory system	354811	Varicose veins of lower extremity with both ulcer of thigh and inflammation (HCC)	ICD10CM	1	I83.201		Varicose veins of unspecified lower extremity with both	IMO	GHS-PACDC	DX_LEGACY	ACTIVE	N	1900-01-01	9999-12-31	2015-07-10

Diagnosis: ICD10 Code, I00-I99, Define	I00-I99	Diseases of the circulatory system	1186451	Varicose veins of unspecified lower extremity with both ulcer of calf and inflammation (HCC)	ICD10CM	1	I83.202		Varicose veins of unspecified lower extremity with both	IMO	GHS-PACDC	IMO_2016_R	ACTIVE	N	1900-01-01	9999-12-31	2016-06-02
Diagnosis: ICD10 Code, I00-I99, Define	I00-I99	Diseases of the circulatory system	861660	Varicose veins of lower extremity with both ulcer and inflammation, right (HCC)	ICD10CM	1	I83.211	I83.211,I83.212,I83.213,I83.214,I	Varicose veins of right lower extremity with both ulcer o	IMO	GHS-PACDC	IMO_2015_R	ACTIVE	N	1900-01-01	2016-05-07	2015-07-10
Diagnosis: ICD10 Code, I00-I99, Define	I00-I99	Diseases of the circulatory system	719149	Varicose veins, lower extremity, with inflammation, ulcerated, right (HCC)	ICD10CM	1	I83.211	I83.211,I83.212,I83.213,I83.214,I	Varicose veins of right lower extremity with both ulcer o	IMO	GHS-PACDC	IMO_2015_R	ACTIVE	N	1900-01-01	2016-05-07	2015-07-10
Diagnosis: ICD10 Code, I00-I99, Define	I00-I99	Diseases of the circulatory system	730118	Varicose veins of lower extremity with both ulcer of ankle and inflammation, right (HCC)	ICD10CM	3	I83.213	I83.211,I83.212,I83.213,I83.214,I	Varicose veins of right lower extremity with both ulcer o	IMO	GHS-PACDC	IMO_2015_R	ACTIVE	N	1900-01-01	2016-05-07	2015-07-10
Diagnosis: ICD10 Code, I00-I99, Define	I00-I99	Diseases of the circulatory system	746902	Varicose veins of leg with both ulcer of thigh and inflammation, right (HCC)	ICD10CM	4	I83.214	I83.211,I83.212,I83.213,I83.214,I	Varicose veins of right lower extremity with both ulcer o	IMO	GHS-PACDC	IMO_2015_R	ACTIVE	N	1900-01-01	2016-05-07	2015-07-10
Diagnosis: ICD10 Code, I00-I99, Define	I00-I99	Diseases of the circulatory system	773490	Varicose veins of leg with both ulcer of calf and inflammation, right (HCC)	ICD10CM	5	I83.215	I83.211,I83.212,I83.213,I83.214,I	Varicose veins of right lower extremity with both ulcer o	IMO	GHS-PACDC	IMO_2015_R	ACTIVE	N	1900-01-01	2016-05-07	2015-07-10
Diagnosis: ICD10 Code, I00-I99, Define	I00-I99	Diseases of the circulatory system	833802	Varicose veins with ulcer and inflammation, right (HCC)	ICD10CM	5	I83.215	I83.211,I83.212,I83.213,I83.214,I	Varicose veins of right lower extremity with both ulcer o	IMO	GHS-PACDC	IMO_2015_R	ACTIVE	N	1900-01-01	2016-05-07	2015-07-10
Diagnosis: ICD10 Code, I00-I99, Define	I00-I99	Diseases of the circulatory system	1181033	Varicose veins of right lower extremity with both ulcer other part of foot and inflammation (HC	ICD10CM	1	I83.215		Varicose veins of right lower extremity with both ulcer o	IMO	GHS-PACDC	IMO_2016_R	ACTIVE	N	1900-01-01	9999-12-31	2016-06-02
Diagnosis: ICD10 Code, I00-I99, Define	I00-I99	Diseases of the circulatory system	746902	Varicose veins of leg with both ulcer of thigh and inflammation, right (HCC)	ICD10CM	5	I83.215	I83.211,I83.212,I83.213,I83.214,I	Varicose veins of right lower extremity with both ulcer o	IMO	GHS-PACDC	IMO_2015_R	ACTIVE	N	1900-01-01	2016-05-07	2015-07-10
Diagnosis: ICD10 Code, I00-I99, Define	I00-I99	Diseases of the circulatory system	811752	Varicose veins of legs with ulcer and inflammation, right	ICD10CM	5	I83.215	I83.211,I83.212,I83.213,I83.214,I	Varicose veins of right lower extremity with both ulcer o	IMO	GHS-PACDC	IMO_2015_R	DELETED	Y	1900-01-01	9999-12-31	2015-07-10
Diagnosis: ICD10 Code, I00-I99, Define	I00-I99	Diseases of the circulatory system	806507	Varicose veins of lower extremity with ulcer and inflammation, right	ICD10CM	5	I83.215	I83.211,I83.212,I83.213,I83.214,I	Varicose veins of right lower extremity with both ulcer o	IMO	GHS-PACDC	IMO_2015_R	DELETED	Y	1900-01-01	9999-12-31	2015-07-10
Diagnosis: ICD10 Code, I00-I99, Define	I00-I99	Diseases of the circulatory system	757235	Varicose veins of lower extremity with both ulcer of calf and inflammation, right (HCC)	ICD10CM	5	I83.215	I83.211,I83.212,I83.213,I83.214,I	Varicose veins of right lower extremity with both ulcer o	IMO	GHS-PACDC	IMO_2015_R	ACTIVE	N	1900-01-01	2016-05-07	2015-07-10
Diagnosis: ICD10 Code, I00-I99, Define	I00-I99	Diseases of the circulatory system	888008	Infected stasis ulcer, right (HCC)	ICD10CM	6	I83.218	I83.211,I83.212,I83.213,I83.214,I	Varicose veins of right lower extremity with both ulcer o	IMO	GHS-PACDC	IMO_2015_R	ACTIVE	N	1900-01-01	2016-05-07	2015-07-10
Diagnosis: ICD10 Code, I00-I99, Define	I00-I99	Diseases of the circulatory system	806507	Varicose veins of lower extremity with ulcer and inflammation, right	ICD10CM	6	I83.218	I83.211,I83.212,I83.213,I83.214,I	Varicose veins of right lower extremity with both ulcer o	IMO	GHS-PACDC	IMO_2015_R	DELETED	Y	1900-01-01	9999-12-31	2015-07-10
Diagnosis: ICD10 Code, I00-I99, Define	I00-I99	Diseases of the circulatory system	878101	Inflamed leg varices with ulceration, right (HCC)	ICD10CM	1	I83.219		Varicose veins of right lower extremity with both ulcer o	IMO	GHS-PACDC	IMO_2015_R	ACTIVE	N	2016-04-10	9999-12-31	2015-07-10
Diagnosis: ICD10 Code, I00-I99, Define	I00-I99	Diseases of the circulatory system	773490	Varicose veins of leg with both ulcer of calf and inflammation, right (HCC)	ICD10CM	7	I83.219	I83.211,I83.212,I83.213,I83.214,I	Varicose veins of right lower extremity with both ulcer o	IMO	GHS-PACDC	IMO_2015_R	ACTIVE	N	1900-01-01	2016-05-07	2015-07-10
Diagnosis: ICD10 Code, I00-I99, Define	I00-I99	Diseases of the circulatory system	850392	Varicose veins of lower extremity with ulcer and inflammation, left	ICD10CM	1	I83.221	I83.221,I83.222,I83.223,I83.224,I	Varicose veins of left lower extremity with both ulcer of	IMO	GHS-PACDC	IMO_2015_R	DELETED	Y	1900-01-01	9999-12-31	2015-07-10
Diagnosis: ICD10 Code, I00-I99, Define	I00-I99	Diseases of the circulatory system	790515	Varicose veins of lower extremity with both ulcer and inflammation, left (HCC)	ICD10CM	3	I83.223	I83.221,I83.222,I83.223,I83.224,I	Varicose veins of left lower extremity with both ulcer of	IMO	GHS-PACDC	IMO_2015_R	ACTIVE	N	1900-01-01	2016-05-07	2015-07-10
Diagnosis: ICD10 Code, I00-I99, Define	I00-I99	Diseases of the circulatory system	877201	Venous stasis ulcer of ankle, left (HCC)	ICD10CM	3	I83.223	I83.221,I83.222,I83.223,I83.224,I	Varicose veins of left lower extremity with both ulcer of	IMO	GHS-PACDC	IMO_2015_R	ACTIVE	N	1900-01-01	2016-05-07	2015-07-10
Diagnosis: ICD10 Code, I00-I99, Define	I00-I99	Diseases of the circulatory system	758072	Varicose veins of lower extremity with both ulcer of thigh and inflammation, left (HCC)	ICD10CM	4	I83.224	I83.221,I83.222,I83.223,I83.224,I	Varicose veins of left lower extremity with both ulcer of	IMO	GHS-PACDC	IMO_2015_R	ACTIVE	N	1900-01-01	2016-05-07	2015-07-10
Diagnosis: ICD10 Code, I00-I99, Define	I00-I99	Diseases of the circulatory system	751597	Varicose veins of lower extremities with both ulcer and inflammation, left	ICD10CM	5	I83.225	I83.221,I83.222,I83.223,I83.224,I	Varicose veins of left lower extremity with both ulcer ot	IMO	GHS-PACDC	IMO_2015_R	DELETED	Y	1900-01-01	9999-12-31	2015-07-10
Diagnosis: ICD10 Code, I00-I99, Define	I00-I99	Diseases of the circulatory system	769113	Venous ulcer of ankle, left (HCC)	ICD10CM	5	I83.225	I83.221,I83.222,I83.223,I83.224,I	Varicose veins of left lower extremity with both ulcer ot	IMO	GHS-PACDC	IMO_2015_R	ACTIVE	N	1900-01-01	2016-05-07	2015-07-10
Diagnosis: ICD10 Code, I00-I99, Define	I00-I99	Diseases of the circulatory system	790515	Varicose veins of lower extremity with both ulcer and inflammation, left (HCC)	ICD10CM	5	I83.225	I83.221,I83.222,I83.223,I83.224,I	Varicose veins of left lower extremity with both ulcer ot	IMO	GHS-PACDC	IMO_2015_R	ACTIVE	N	1900-01-01	2016-05-07	2015-07-10
Diagnosis: ICD10 Code, I00-I99, Define	I00-I99	Diseases of the circulatory system	779386	Stasis ulcer of ankle, left (HCC)	ICD10CM	5	I83.225	I83.221,I83.222,I83.223,I83.224,I	Varicose veins of left lower extremity with both ulcer ot	IMO	GHS-PACDC	IMO_2015_R	ACTIVE	N	1900-01-01	2016-05-07	2015-07-10
Diagnosis: ICD10 Code, I00-I99, Define	I00-I99	Diseases of the circulatory system	861681	Varicose veins of lower extremity with both ulcer of calf and inflammation, left (HCC)	ICD10CM	5	I83.225	I83.221,I83.222,I83.223,I83.224,I	Varicose veins of left lower extremity with both ulcer ot	IMO	GHS-PACDC	IMO_2015_R	ACTIVE	N	1900-01-01	2016-05-07	2015-07-10
Diagnosis: ICD10 Code, I00-I99, Define	I00-I99	Diseases of the circulatory system	757067	Varicose veins of legs with ulcer and inflammation, left	ICD10CM	7	I83.229	I83.221,I83.222,I83.223,I83.224,I	Varicose veins of left lower extremity with both ulcer of	IMO	GHS-PACDC	IMO_2015_R	DELETED	Y	1900-01-01	9999-12-31	2015-07-10
Diagnosis: ICD10 Code, I00-I99, Define	I00-I99	Diseases of the circulatory system	283273	Varicose veins of left lower extremity with pain	ICD10CM	1	I83.812		Varicose veins of left lower extremity with pain	IMO	GHS-PACDC	DX_LEGACY	ACTIVE	N	1900-01-01	9999-12-31	2015-07-10
Diagnosis: ICD10 Code, I00-I99, Define	I00-I99	Diseases of the circulatory system	840444	Varicose veins of leg with complications, right	ICD10CM	1	I83.891		Varicose veins of right lower extremity with other compl	IMO	GHS-PACDC	IMO_2015_R	DELETED	Y	1900-01-01	9999-12-31	2015-07-10
Diagnosis: ICD10 Code, I00-I99, Define	I00-I99	Diseases of the circulatory system	1081483	Varicose veins of lower extremity with edema, right	ICD10CM	1	I83.891		Varicose veins of right lower extremity with other compl	IMO	GHS-PACDC	IMO_2015_R	ACTIVE	N	1900-01-01	9999-12-31	2015-07-10
Diagnosis: ICD10 Code, I00-I99, Define	I00-I99	Diseases of the circulatory system	596655	Varicose veins of left lower extremities with other complications	ICD10CM	1	I83.892		Varicose veins of left lower extremity with other complic	IMO	GHS-PACDC	IMO_2015_R	ACTIVE	Y	1900-01-01	9999-12-31	2015-07-10
Diagnosis: ICD10 Code, I00-I99, Define	I00-I99	Diseases of the circulatory system	934169	Bleeding from varicose vein, bilateral	ICD10CM	1	I83.893		Varicose veins of bilateral lower extremities with other c	IMO	GHS-PACDC	IMO_2015_R	ACTIVE	N	1900-01-01	9999-12-31	2015-07-10
Diagnosis: ICD10 Code, I00-I99, Define	I00-I99	Diseases of the circulatory system	511873	Varicose veins with swelling	ICD10CM	1	I83.899		Varicose veins of unspecified lower extremity with other	IMO	GHS-PACDC	IMO_2015_R	ACTIVE	N	1900-01-01	9999-12-31	2015-07-10
Diagnosis: ICD10 Code, I00-I99, Define	I00-I99	Diseases of the circulatory system	1427284	Varicose veins of unspecified lower extremity with other complications (CODE)	ICD10CM	1	I83.899		Varicose veins of unspecified lower extremity with other	IMO	GHS-PACDC	IMO_2018_R	ACTIVE	N	1900-01-01	9999-12-31	2017-10-30
Diagnosis: ICD10 Code, I00-I99, Define	I00-I99	Diseases of the circulatory system	766236	Varicose veins of lower limb without ulcer or inflammation, unspecified laterality	ICD10CM	1	I83.90		Asymptomatic varicose veins of unspecified lower extre	IMO	GHS-PACDC	IMO_2015_R	ACTIVE	N	1900-01-01	9999-12-31	2015-07-10
Diagnosis: ICD10 Code, I00-I99, Define	I00-I99	Diseases of the circulatory system	102929	Varicose veins of lower extremities without ulcer or inflammation	ICD10CM	1	I83.90		Asymptomatic varicose veins of unspecified lower extre	IMO	GHS-PACDC	DX_LEGACY	ACTIVE	N	1900-01-01	9999-12-31	2015-07-10
Diagnosis: ICD10 Code, I00-I99, Define	I00-I99	Diseases of the circulatory system	191265	Varicosed vein	ICD10CM	1	I83.90		Asymptomatic varicose veins of unspecified lower extre	IMO	GHS-PACDC	DX_LEGACY	DELETED	Y	2017-09-17	9999-12-31	2017-10-30
Diagnosis: ICD10 Code, I00-I99, Define	I00-I99	Diseases of the circulatory system	5289	Varicose vein of leg	ICD10CM	1	I83.90		Asymptomatic varicose veins of unspecified lower extre	IMO	GHS-PACDC	DX_LEGACY	ACTIVE	N	2016-09-17	9999-12-31	2016-09-30
Diagnosis: ICD10 Code, I00-I99, Define	I00-I99	Diseases of the circulatory system	1302459	Asymptomatic superficial varicose vein of lower extremity	ICD10CM	1	I83.90		Asymptomatic varicose veins of unspecified lower extre	IMO	GHS-PACDC	IMO_2017_R	ACTIVE	N	1900-01-01	9999-12-31	2017-04-14
Diagnosis: ICD10 Code, I00-I99, Define	I00-I99	Diseases of the circulatory system	246848	Ruptured varicosity	ICD10CM	1	I83.90		Asymptomatic varicose veins of unspecified lower extre	IMO	GHS-PACDC	DX_LEGACY	ACTIVE	N	2016-04-10	9999-12-31	2015-07-10
Diagnosis: ICD10 Code, I00-I99, Define	I00-I99	Diseases of the circulatory system	1092753	Uncomplicated varicose veins, unspecified laterality	ICD10CM	1	I83.90		Asymptomatic varicose veins of unspecified lower extre	IMO	GHS-PACDC	IMO_2015_R	ACTIVE	N	1900-01-01	9999-12-31	2015-07-10
Diagnosis: ICD10 Code, I00-I99, Define	I00-I99	Diseases of the circulatory system	267961	Asymptomatic varicose veins of left lower extremity	ICD10CM	1	I83.92		Asymptomatic varicose veins of left lower extremity	IMO	GHS-PACDC	DX_LEGACY	ACTIVE	N	1900-01-01	9999-12-31	2015-07-10
Diagnosis: ICD10 Code, I00-I99, Define	I00-I99	Diseases of the circulatory system	884809	Asymptomatic varicose veins of lower extremity, left	ICD10CM	1	I83.92		Asymptomatic varicose veins of left lower extremity	IMO	GHS-PACDC	IMO_2015_R	ACTIVE	N	1900-01-01	9999-12-31	2015-07-10
Diagnosis: ICD10 Code, I00-I99, Define	I00-I99	Diseases of the circulatory system	1089829	Reticular venous varices, bilateral	ICD10CM	1	I83.93		Asymptomatic varicose veins of bilateral lower extremiti	IMO	GHS-PACDC	IMO_2015_R	ACTIVE	N	1900-01-01	9999-12-31	2015-07-10
Diagnosis: ICD10 Code, I00-I99, Define	I00-I99	Diseases of the circulatory system	325759	Leg varices	ICD10CM	1	I83.93		Asymptomatic varicose veins of bilateral lower extremiti	IMO	GHS-PACDC	DX_LEGACY	ACTIVE	N	1900-01-01	2016-10-14	2015-07-10
Diagnosis: ICD10 Code, I00-I99, Define	I00-I99	Diseases of the circulatory system	533899	Esophageal varices without bleeding	ICD10CM	1	I85.00		Esophageal varices without bleeding	IMO	GHS-PACDC	IMO_2015_R	ACTIVE	Y	1900-01-01	9999-12-31	2015-07-10
Diagnosis: ICD10 Code, I00-I99, Define	I00-I99	Diseases of the circulatory system	130956	Esophageal and gastric varices (HCC)	ICD10CM	1	I85.00	I85.00,I86.4	Esophageal varices without bleeding	IMO	GHS-PACDC	DX_LEGACY	ACTIVE	N	1900-01-01	9999-12-31	2015-07-10
Diagnosis: ICD10 Code, I00-I99, Define	I00-I99	Diseases of the circulatory system	1348322	BOV (bleeding ooesophageal varices) (HCC)	ICD10CM	1	I85.01		Esophageal varices with bleeding	IMO	GHS-PACDC	IMO_2018_R	ACTIVE	N	1900-01-01	9999-12-31	2017-10-30
Diagnosis: ICD10 Code, I00-I99, Define	I00-I99	Diseases of the circulatory system	1108995	Idiopathic esophageal varices with bleeding (HCC)	ICD10CM	1	I85.01		Esophageal varices with bleeding	IMO	GHS-PACDC	IMO_2016_R	ACTIVE	N	1900-01-01	9999-12-31	2016-06-02
Diagnosis: ICD10 Code, I00-I99, Define	I00-I99	Diseases of the circulatory system	25263	Bleeding esophageal varices (HCC)	ICD10CM	1	I85.01		Esophageal varices with bleeding	IMO	GHS-PACDC	DX_LEGACY	ACTIVE	N	1900-01-01	9999-12-31	2015-07-10
Diagnosis: ICD10 Code, I00-I99, Define	I00-I99	Diseases of the circulatory system	202868	Bilateral varicoceles	ICD10CM	1	I86.1		Scrotal varices	IMO	GHS-PACDC	DX_LEGACY	ACTIVE	N	1900-01-01	9999-12-31	2015-07-10
Diagnosis: ICD10 Code, I00-I99, Define	I00-I99	Diseases of the circulatory system	102042	Scrotal varices - varicocele	ICD10CM	1	I86.1		Scrotal varices	IMO	GHS-PACDC	DX_LEGACY	DELETED	Y	1900-01-01	9999-12-31	2015-07-10
Diagnosis: ICD10 Code, I00-I99, Define	I00-I99	Diseases of the circulatory system	5309	Pelvic varices	ICD10CM	1	I86.2		Pelvic varices	IMO	GHS-PACDC	DX_LEGACY	ACTIVE	N	1900-01-01	9999-12-31	2015-07-10
Diagnosis: ICD10 Code, I00-I99, Define	I00-I99	Diseases of the circulatory system	112898	Perineal varices	ICD10CM	1	I86.3		Vulval varices	IMO	GHS-PACDC	DX_LEGACY	ACTIVE	N	1900-01-01	9999-12-31	2015-07-10
Diagnosis: ICD10 Code, I00-I99, Define	I00-I99	Diseases of the circulatory system	255856	Varicose veins of vulva and perineum	ICD10CM	1	I86.3		Vulval varices	IMO	GHS-PACDC	DX_LEGACY	ACTIVE	N	1900-01-01	9999-12-31	2015-07-10
Diagnosis: ICD10 Code, I00-I99, Define	I00-I99	Diseases of the circulatory system	115995	Varix, gastric	ICD10CM	1	I86.4		Gastric varices	IMO	GHS-PACDC	DX_LEGACY	ACTIVE	N	1900-01-01	9999-12-31	2015-07-10
Diagnosis: ICD10 Code, I00-I99, Define	I00-I99	Diseases of the circulatory system	58110	Varicose veins	ICD10CM	1	I86.8		Varicose veins of other specified sites	IMO	GHS-PACDC	DX_LEGACY	DELETED	Y	1900-01-01	2017-10-14	2015-07-10
Diagnosis: ICD10 Code, I00-I99, Define	I00-I99	Diseases of the circulatory system	328752	Peristomal varices	ICD10CM	1	I86.8		Varicose veins of other specified sites	IMO	GHS-PACDC	DX_LEGACY	ACTIVE	N	1900-01-01	9999-12-31	2015-07-10
Diagnosis: ICD10 Code, I00-I99, Define	I00-I99	Diseases of the circulatory system	22176	Medusa's head	ICD10CM	1	I86.8		Varicose veins of other specified sites	IMO	GHS-PACDC	DX_LEGACY	ACTIVE	N	1900-01-01	9999-12-31	2015-07-10
Diagnosis: ICD10 Code, I00-I99, Define	I00-I99	Diseases of the circulatory system	22175	Cirsomphalos	ICD10CM	1	I86.8		Varicose veins of other specified sites	IMO	GHS-PACDC	DX_LEGACY	ACTIVE	N	1900-01-01	9999-12-31	2015-07-10
Diagnosis: ICD10 Code, I00-I99, Define	I00-I99	Diseases of the circulatory system	615309	Other disorders of veins	ICD10CM	1	I87		Other disorders of veins	IMO	GHS-PACDC	IMO_2015_R	ACTIVE	Y	1900-01-01	9999-12-31	2015-07-10
Diagnosis: ICD10 Code, I00-I99, Define	I00-I99	Diseases of the circulatory system	599840	Postthrombotic syndrome without complications of right lower extremity	ICD10CM	1	I87.001		Postthrombotic syndrome without complications of righ	IMO	GHS-PACDC	IMO_2015_R	ACTIVE	Y	1900-01-01	9999-12-31	2015-07-10
Diagnosis: ICD10 Code, I00-I99, Define	I00-I99	Diseases of the circulatory system	1366327	Postthrom syndrome w/o complications of bilateral low extrm	ICD10CM	1	I87.003		Postthrombotic syndrome without complications of bila	IMO	GHS-PACDC	IMO_2018_R	ACTIVE	N	1900-01-01	9999-12-31	2017-10-30
Diagnosis: ICD10 Code, I00-I99, Define	I00-I99	Diseases of the circulatory system	1260384	Ulcer of left lower extremity due to postphlebitic syndrome (HCC)	ICD10CM	1	I87.012	I87.012,L97.909	Postthrombotic syndrome with ulcer of left lower extre	IMO	GHS-PACDC	IMO_2017_R	ACTIVE	N	1900-01-01	2017-04-27	2016-09-30
Diagnosis: ICD10 Code, I00-I99, Define	I00-I99	Diseases of the circulatory system	1260384	Ulcer of left lower extremity due to postphlebitic syndrome (HCC)	ICD10CM	1	I87.012	I87.012,L97.929	Postthrombotic syndrome with ulcer of left lower extre	IMO	GHS-PACDC	IMO_2017_R	ACTIVE	N	2017-03-31	9999-12-31	2017-04-14
Diagnosis: ICD10 Code, I00-I99, Define	I00-I99	Diseases of the circulatory system	136161	Postphlebitic ulcer (HCC)	ICD10CM	1	I87.019		Postthrombotic syndrome with ulcer of unspecified low	IMO	GHS-PACDC	DX_LEGACY	ACTIVE	N	1900-01-01	9999-12-31	2015-07-10
Diagnosis: ICD10 Code, I00-I99, Define	I00-I99	Diseases of the circulatory system	1183784	Postthrombotic syndrome with inflammation of unspecified lower extremity	ICD10CM	1	I87.029		Postthrombotic syndrome with inflammation of unspeci	IMO	GHS-PACDC	IMO_2016_R	ACTIVE	N	1900-01-01	9999-12-31	2016-06-02
Diagnosis: ICD10 Code, I00-I99, Define	I00-I99	Diseases of the circulatory system	755741	Chronic venous hypertension due to deep vein thrombosis, left	ICD10CM	1	I87.092		Postthrombotic syndrome with other complications of le	IMO	GHS-PACDC	IMO_2015_R	ACTIVE	N	1900-01-01	9999-12-31	2015-07-10
Diagnosis: ICD10 Code, I00-I99, Define	I00-I99	Diseases of the circulatory system	874722	Complicated postphlebitic syndrome, unspecified laterality	ICD10CM	1	I87.099		Postthrombotic syndrome with other complications of u	IMO	GHS-PACDC	IMO_2015_R	ACTIVE	N	1900-01-01	9999-12-31	2015-07-10
Diagnosis: ICD10 Code, I00-I99, Define	I00-I99	Diseases of the circulatory system	594747	Postthrombotic syndrome with other complications of unspecified lower extremity	ICD10CM	1	I87.099		Postthrombotic syndrome with other complications of u	IMO	GHS-PACDC	IMO_2015_R	ACTIVE	Y	1900-01-01	9999-12-31	2015-07-10
Diagnosis: ICD10 Code, I00-I99, Define	I00-I99	Diseases of the circulatory system	230623	Inferior vena caval stenosis	ICD10CM	1	I87.1		Compression of vein	IMO	GHS-PACDC	DX_LEGACY	ACTIVE	N	1900-01-01	9999-12-31	2015-07-10
Diagnosis: ICD10 Code, I00-I99, Define	I00-I99	Diseases of the circulatory system	332321	Intragraft venous stenosis of transplanted pancreas (HCC)	ICD10CM	4	I87.1	T82.868A,T86.899,Y83.0,I87.1	Compression of vein	IMO	GHS-PACDC	DX_LEGACY	ACTIVE	N	1900-01-01	2017-10-14	2015-07-10
Diagnosis: ICD10 Code, I00-I99, Define	I00-I99	Diseases of the circulatory system	709254	Intragraft venous stenosis of transplanted pancreas, subsequent encounter	ICD10CM	3	I87.1	T82.868D,T86.899,I87.1,Y83.0	Compression of vein	IMO	GHS-PACDC	IMO_2015_R	ACTIVE	N	2017-03-31	9999-12-31	2017-04-14
Diagnosis: ICD10 Code, I00-I99, Define	I00-I99	Diseases of the circulatory system	234723	External compression of portal vein	ICD10CM	1	I87.1		Compression of vein	IMO	GHS-PACDC	DX_LEGACY	ACTIVE	N	1900-01-01	9999-12-31	2015-07-10
Diagnosis: ICD10 Code, I00-I99, Define	I00-I99	Diseases of the circulatory system	230036	Stenosis of right jugular vein	ICD10CM	1	I87.1		Compression of vein	IMO	GHS-PACDC	DX_LEGACY	ACTIVE	N	1900-01-01	9999-12-31	2015-07-10
Diagnosis: ICD10 Code, I00-I99, Define	I00-I99	Diseases of the circulatory system	232972	Superior vena caval stricture	ICD10CM	1	I87.1		Compression of vein	IMO	GHS-PACDC	DX_LEGACY	ACTIVE	N	1900-01-01	9999-12-31	2015-07-10
Diagnosis: ICD10 Code, I00-I99, Define	I00-I99	Diseases of the circulatory system	305316	Portal vein stenosis of transplanted liver (HCC)	ICD10CM	2	I87.1	T86.49,I87.1	Compression of vein	IMO	GHS-PACDC	DX_LEGACY	ACTIVE	N	1900-01-01	9999-12-31	2015-07-10
Diagnosis: ICD10 Code, I00-I99, Define	I00-I99	Diseases of the circulatory system	305368	Pancreas vein stenosis of pancreas transplant	ICD10CM	2	I87.1	T86.898,I87.1	Compression of vein	IMO	GHS-PACDC	DX_LEGACY	ACTIVE	N	1900-01-01	9999-12-31	2015-07-10
Diagnosis: ICD10 Code, I00-I99, Define	I00-I99	Diseases of the circulatory system	709363	Intragraft venous stenosis of transplanted pancreas, sequela	ICD10CM	3	I87.1	T82.868S,T86.899,I87.1,Y83.0	Compression of vein	IMO	GHS-PACDC	IMO_2015_R	ACTIVE	N	2017-03-31	9999-12-31	2017-04-14
Diagnosis: ICD10 Code, I00-I99, Define	I00-I99	Diseases of the circulatory system	332072	Venous obstruction	ICD10CM	1	I87.1		Compression of vein	IMO	GHS-PACDC	DX_LEGACY	ACTIVE	N	1900-01-01	9999-12-31	2015-07-10
Diagnosis: ICD10 Code, I00-I99, Define	I00-I99	Diseases of the circulatory system	415805	Brachial vein stenosis	ICD10CM	1	I87.1		Compression of vein	IMO	GHS-PACDC	DX_LEGACY	ACTIVE	N	1900-01-01	9999-12-31	2015-07-10
Diagnosis: ICD10 Code, I00-I99, Define	I00-I99	Diseases of the circulatory system	249515	Subclavian vein obstruction with collaterals	ICD10CM	1	I87.1		Compression of vein	IMO	GHS-PACDC	DX_LEGACY	ACTIVE	N	2017-03-31	9999-12-31	2017-04-14
Diagnosis: ICD10 Code, I00-I99, Define	I00-I99	Diseases of the circulatory system	230710	Subclavian vein stenosis	ICD10CM	1	I87.1		Compression of vein	IMO	GHS-PACDC	DX_LEGACY	ACTIVE	N	1900-01-01	9999-12-31	2015-07-10
Diagnosis: ICD10 Code, I00-I99, Define	I00-I99	Diseases of the circulatory system	1321095	Venous stasis ulcer of heel with fat layer exposed without varicose veins	ICD10CM	1	I87.2		Venous insufficiency (chronic) (peripheral)	IMO	GHS-PACDC	IMO_2017_R	ACTIVE	N	1900-01-01	2017-10-14	2017-04-14
Diagnosis: ICD10 Code, I00-I99, Define	I00-I99	Diseases of the circulatory system	500346	Venous insufficiency of both lower extremities	ICD10CM	1	I87.2		Venous insufficiency (chronic) (peripheral)	IMO	GHS-PACDC	IMO_2015_R	ACTIVE	N	1900-01-01	9999-12-31	2015-07-10
Diagnosis: ICD10 Code, I00-I99, Define	I00-I99	Diseases of the circulatory system	1404708	Venous stasis ulcer of other part of left foot with necrosis of muscle without varicose veins (HC	ICD10CM	1	I87.2	I87.2,L97.523	Venous insufficiency (chronic) (peripheral)	IMO	GHS-PACDC	IMO_2018_R	ACTIVE	N	1900-01-01	9999-12-31	2017-10-30
Diagnosis: ICD10 Code, I00-I99, Define	I00-I99	Diseases of the circulatory system	1320801	Venous stasis ulcer of midfoot with fat layer exposed without varicose veins (HCC)	ICD10CM	1	I87.2	I87.2,L97.402	Venous insufficiency (chronic) (peripheral)	IMO	GHS-PACDC	IMO_2017_R	ACTIVE	N	2017-09-17	9999-12-31	2017-10-30
Diagnosis: ICD10 Code, I00-I99, Define	I00-I99	Diseases of the circulatory system	1320822	Venous stasis ulcer of midfoot without varicose veins	ICD10CM	1	I87.2		Venous insufficiency (chronic) (peripheral)	IMO	GHS-PACDC	IMO_2017_R	ACTIVE	N	1900-01-01	2017-10-14	2017-04-14
Diagnosis: ICD10 Code, I00-I99, Define	I00-I99	Diseases of the circulatory system	1426844	Venous stasis ulcer of calf with muscle involvement without evidence of necrosis without varico	ICD10CM	1	I87.2	I87.2,L97.205	Venous insufficiency (chronic) (peripheral)	IMO	GHS-PACDC	IMO_2018_R	ACTIVE	N	1900-01-01	9999-12-31	2017-10-30
Diagnosis: ICD10 Code, I00-I99, Define	I00-I99	Diseases of the circulatory system	1321085	Venous stasis ulcer of left heel with fat layer exposed without varicose veins	ICD10CM	1	I87.2		Venous insufficiency (chronic) (peripheral)	IMO	GHS-PACDC	IMO_2017_R	ACTIVE	N	1900-01-01	2017-10-14	2017-04-14
Diagnosis: ICD10 Code, I00-I99, Define	I00-I99	Diseases of the circulatory system	1427385	Venous stasis ulcer of other part of right lower leg with muscle involvement without evidence o	ICD10CM	1	I87.2	I87.2,L97.815	Venous insufficiency (chronic) (peripheral)	IMO	GHS-PACDC	IMO_2018_R	ACTIVE	N	1900-01-01	9999-12-31	2017-10-30
Diagnosis: ICD10 Code, I00-I99, Define	I00-I99	Diseases of the circulatory system	1427552	Venous stasis ulcer of other part of lower leg limited to breakdown of skin without varicose veir	ICD10CM	1	I87.2	I87.2,L97.801	Venous insufficiency (chronic) (peripheral)	IMO	GHS-PACDC	IMO_2018_R	ACTIVE	N	1900-01-01	9999-12-31	2017-10-30
Diagnosis: ICD10 Code, I00-I99, Define	I00-I99	Diseases of the circulatory system	1427391	Venous stasis ulcer of other part of left lower leg with bone involvement without evidence of ne	ICD10CM	1	I87.2	I87.2,L97.826	Venous insufficiency (chronic) (peripheral)	IMO	GHS-PACDC	IMO_2018_R	ACTIVE	N	1900-01-01	9999-12-31	2017-10-30
Diagnosis: ICD10 Code, I00-I99, Define	I00-I99	Diseases of the circulatory system	1355372	Venous stasis ulcer of left thigh with bone involvement without evidence of necrosis without va	ICD10CM	1	I87.2	I87.2,L97.126	Venous insufficiency (chronic) (peripheral)	IMO	GHS-PACDC	IMO_2018_R	ACTIVE	N	1900-01-01	9999-12-31	2017-10-30
Diagnosis: ICD10 Code, I00-I99, Define	I00-I99	Diseases of the circulatory system	1320778	Venous stasis ulcer of left heel with necrosis of bone without varicose veins	ICD10CM	1	I87.2	I87.2,L97.424	Venous insufficiency (chronic) (peripheral)	IMO	GHS-PACDC	IMO_2017_R	ACTIVE	N	2017-09-17	9999-12-31	2017-10-30
Diagnosis: ICD10 Code, I00-I99, Define	I00-I99	Diseases of the circulatory system	1427454	Venous stasis ulcer of other part of right foot with bone involvement without evidence of necro	ICD10CM	1	I87.2	I87.2,L97.516	Venous insufficiency (chronic) (peripheral)	IMO	GHS-PACDC	IMO_2018_R	ACTIVE	N	1900-01-01	9999-12-31	2017-10-30

Diagnosis: ICD10 Code, I00-I99, Define	I00-I99	Diseases of the circulatory system	499444	Venous ulcer of right lower extremity without varicose veins	ICD10CM	1	I87.2		Venous insufficiency (chronic) (peripheral)	IMO	GHS-PACDC	IMO_2015_R	ACTIVE	N	1900-01-01	2017-10-14	2015-07-10
Diagnosis: ICD10 Code, I00-I99, Define	I00-I99	Diseases of the circulatory system	1427469	Venous stasis ulcer of other site with muscle involvement without evidence of necrosis without	ICD10CM	1	I87.2	I87.2,L97.805	Venous insufficiency (chronic) (peripheral)	IMO	GHS-PACDC	IMO_2018_R	ACTIVE	N	1900-01-01	9999-12-31	2017-10-30
Diagnosis: ICD10 Code, I00-I99, Define	I00-I99	Diseases of the circulatory system	1427462	Venous stasis ulcer of right thigh with other ulcer severity without varicose veins	ICD10CM	1	I87.2	I87.2,L97.118	Venous insufficiency (chronic) (peripheral)	IMO	GHS-PACDC	IMO_2018_R	ACTIVE	N	1900-01-01	9999-12-31	2017-10-30
Diagnosis: ICD10 Code, I00-I99, Define	I00-I99	Diseases of the circulatory system	1320912	Venous stasis ulcer of thigh with necrosis of muscle without varicose veins	ICD10CM	1	I87.2	I87.2,L97.103	Venous insufficiency (chronic) (peripheral)	IMO	GHS-PACDC	IMO_2017_R	ACTIVE	N	2017-09-17	9999-12-31	2017-10-30
Diagnosis: ICD10 Code, I00-I99, Define	I00-I99	Diseases of the circulatory system	1427728	Venous stasis ulcer with necrosis of muscle without varicose veins, unspecified site (HCC)	ICD10CM	1	I87.2	I87.2,L97.903	Venous insufficiency (chronic) (peripheral)	IMO	GHS-PACDC	IMO_2018_R	ACTIVE	N	1900-01-01	9999-12-31	2017-10-30
Diagnosis: ICD10 Code, I00-I99, Define	I00-I99	Diseases of the circulatory system	1427554	Venous stasis ulcer of other part of lower leg with fat layer exposed without varicose veins, uns	ICD10CM	1	I87.2	I87.2,L97.802	Venous insufficiency (chronic) (peripheral)	IMO	GHS-PACDC	IMO_2018_R	ACTIVE	N	1900-01-01	9999-12-31	2017-10-30
Diagnosis: ICD10 Code, I00-I99, Define	I00-I99	Diseases of the circulatory system	1404677	Venous stasis ulcer of other part of left foot with necrosis of bone without varicose veins (HCC)	ICD10CM	1	I87.2	I87.2,L97.524	Venous insufficiency (chronic) (peripheral)	IMO	GHS-PACDC	IMO_2018_R	ACTIVE	N	1900-01-01	9999-12-31	2017-10-30
Diagnosis: ICD10 Code, I00-I99, Define	I00-I99	Diseases of the circulatory system	355287	Idiopathic chronic venous hypertension of right lower extremity without complications	ICD10CM	1	I87.301		Chronic venous hypertension (idiopathic) without compl	IMO	GHS-PACDC	DX_LEGACY	ACTIVE	N	1900-01-01	9999-12-31	2015-07-10
Diagnosis: ICD10 Code, I00-I99, Define	I00-I99	Diseases of the circulatory system	798422	Venous hypertension, chronic, right	ICD10CM	1	I87.301		Chronic venous hypertension (idiopathic) without compl	IMO	GHS-PACDC	IMO_2015_R	ACTIVE	N	1900-01-01	9999-12-31	2015-07-10
Diagnosis: ICD10 Code, I00-I99, Define	I00-I99	Diseases of the circulatory system	303513	Chronic venous hypertension involving left side	ICD10CM	1	I87.302		Chronic venous hypertension (idiopathic) without compl	IMO	GHS-PACDC	DX_LEGACY	ACTIVE	N	1900-01-01	9999-12-31	2015-07-10
Diagnosis: ICD10 Code, I00-I99, Define	I00-I99	Diseases of the circulatory system	755388	Asymptomatic chronic venous hypertension, left	ICD10CM	1	I87.302		Chronic venous hypertension (idiopathic) without compl	IMO	GHS-PACDC	IMO_2015_R	ACTIVE	N	1900-01-01	9999-12-31	2015-07-10
Diagnosis: ICD10 Code, I00-I99, Define	I00-I99	Diseases of the circulatory system	456828	Venous hypertension of both lower extremities	ICD10CM	1	I87.303		Chronic venous hypertension (idiopathic) without compl	IMO	GHS-PACDC	IMO_2015_R	ACTIVE	N	1900-01-01	9999-12-31	2015-07-10
Diagnosis: ICD10 Code, I00-I99, Define	I00-I99	Diseases of the circulatory system	926429	Asymptomatic chronic venous hypertension, bilateral	ICD10CM	1	I87.303		Chronic venous hypertension (idiopathic) without compl	IMO	GHS-PACDC	IMO_2015_R	ACTIVE	N	1900-01-01	9999-12-31	2015-07-10
Diagnosis: ICD10 Code, I00-I99, Define	I00-I99	Diseases of the circulatory system	374469	Idiopathic chronic venous hypertension of both legs w/o complications	ICD10CM	1	I87.303		Chronic venous hypertension (idiopathic) without compl	IMO	GHS-PACDC	DX_LEGACY	ACTIVE	N	1900-01-01	9999-12-31	2015-07-10
Diagnosis: ICD10 Code, I00-I99, Define	I00-I99	Diseases of the circulatory system	597542	Chronic venous hypertension (idiopathic) with ulcer of right lower extremity	ICD10CM	1	I87.311		Chronic venous hypertension (idiopathic) with ulcer of r	IMO	GHS-PACDC	IMO_2015_R	ACTIVE	Y	1900-01-01	9999-12-31	2015-07-10
Diagnosis: ICD10 Code, I00-I99, Define	I00-I99	Diseases of the circulatory system	1181284	Chronic venous hypertension (idiopathic) with ulcer of right lower extremity (HCC)	ICD10CM	1	I87.311		Chronic venous hypertension (idiopathic) with ulcer of r	IMO	GHS-PACDC	IMO_2016_R	ACTIVE	N	1900-01-01	9999-12-31	2016-06-02
Diagnosis: ICD10 Code, I00-I99, Define	I00-I99	Diseases of the circulatory system	472364	Stasis ulcer of left lower extremity (HCC)	ICD10CM	1	I87.312		Chronic venous hypertension (idiopathic) with ulcer of le	IMO	GHS-PACDC	IMO_2015_R	ACTIVE	N	1900-01-01	2016-05-07	2015-07-10
Diagnosis: ICD10 Code, I00-I99, Define	I00-I99	Diseases of the circulatory system	489777	Venous stasis ulcer of left lower extremity (HCC)	ICD10CM	1	I87.312		Chronic venous hypertension (idiopathic) with ulcer of le	IMO	GHS-PACDC	IMO_2015_R	ACTIVE	N	1900-01-01	2016-05-07	2015-07-10
Diagnosis: ICD10 Code, I00-I99, Define	I00-I99	Diseases of the circulatory system	819790	Idiopathic chronic venous hypertension of lower extremity with ulcer, left (HCC)	ICD10CM	1	I87.312		Chronic venous hypertension (idiopathic) with ulcer of le	IMO	GHS-PACDC	IMO_2015_R	ACTIVE	N	1900-01-01	9999-12-31	2015-07-10
Diagnosis: ICD10 Code, I00-I99, Define	I00-I99	Diseases of the circulatory system	1365351	Chronic venous hypertension w ulcer of l low extrem (HCC)	ICD10CM	1	I87.312		Chronic venous hypertension (idiopathic) with ulcer of le	IMO	GHS-PACDC	IMO_2018_R	ACTIVE	N	1900-01-01	9999-12-31	2017-10-30
Diagnosis: ICD10 Code, I00-I99, Define	I00-I99	Diseases of the circulatory system	790028	Chronic venous hypertension w ulceration, left (HCC)	ICD10CM	1	I87.312		Chronic venous hypertension (idiopathic) with ulcer of le	IMO	GHS-PACDC	IMO_2015_R	ACTIVE	N	1900-01-01	9999-12-31	2015-07-10
Diagnosis: ICD10 Code, I00-I99, Define	I00-I99	Diseases of the circulatory system	819980	Stasis edema with inflammation, right	ICD10CM	1	I87.321		Chronic venous hypertension (idiopathic) with inflamma	IMO	GHS-PACDC	IMO_2015_R	ACTIVE	N	1900-01-01	9999-12-31	2015-07-10
Diagnosis: ICD10 Code, I00-I99, Define	I00-I99	Diseases of the circulatory system	937667	Venous hypertension, chronic, with inflammation, bilateral	ICD10CM	1	I87.323		Chronic venous hypertension (idiopathic) with inflamma	IMO	GHS-PACDC	IMO_2015_R	ACTIVE	N	1900-01-01	9999-12-31	2015-07-10
Diagnosis: ICD10 Code, I00-I99, Define	I00-I99	Diseases of the circulatory system	1366220	Chronic venous htn w inflammation of bilateral low extrm	ICD10CM	1	I87.323		Chronic venous hypertension (idiopathic) with inflamma	IMO	GHS-PACDC	IMO_2018_R	ACTIVE	N	1900-01-01	9999-12-31	2017-10-30
Diagnosis: ICD10 Code, I00-I99, Define	I00-I99	Diseases of the circulatory system	136180	Stasis edema with inflammation	ICD10CM	1	I87.329		Chronic venous hypertension (idiopathic) with inflamma	IMO	GHS-PACDC	DX_LEGACY	ACTIVE	N	1900-01-01	9999-12-31	2015-07-10
Diagnosis: ICD10 Code, I00-I99, Define	I00-I99	Diseases of the circulatory system	1125112	Stasis dermatitis of left lower extremity with venous ulcer due to chronic peripheral venous hyp	ICD10CM	1	I87.332		Chronic venous hypertension (idiopathic) with ulcer and	IMO	GHS-PACDC	IMO_2016_R	ACTIVE	N	1900-01-01	9999-12-31	2016-06-02
Diagnosis: ICD10 Code, I00-I99, Define	I00-I99	Diseases of the circulatory system	925084	Idiopathic chronic venous hypertension of leg w/ulcer and inflammation, bilateral (HCC)	ICD10CM	1	I87.333		Chronic venous hypertension (idiopathic) with ulcer and	IMO	GHS-PACDC	IMO_2015_R	ACTIVE	N	1900-01-01	9999-12-31	2015-07-10
Diagnosis: ICD10 Code, I00-I99, Define	I00-I99	Diseases of the circulatory system	833361	Venous hypertension, chronic, with ulcer and inflammation, unspecified laterality (HCC)	ICD10CM	1	I87.339		Chronic venous hypertension (idiopathic) with ulcer and	IMO	GHS-PACDC	IMO_2015_R	ACTIVE	N	1900-01-01	9999-12-31	2015-07-10
Diagnosis: ICD10 Code, I00-I99, Define	I00-I99	Diseases of the circulatory system	380258	Major systemic-pulmonary collateral artry w/unknown distribution zone	ICD10CM	1	I87.8		Other specified disorders of veins	IMO	GHS-PACDC	DX_LEGACY	ACTIVE	N	1900-01-01	9999-12-31	2015-07-10
Diagnosis: ICD10 Code, I00-I99, Define	I00-I99	Diseases of the circulatory system	328360	Venoocclusive disease	ICD10CM	1	I87.8		Other specified disorders of veins	IMO	GHS-PACDC	DX_LEGACY	ACTIVE	N	1900-01-01	9999-12-31	2015-07-10
Diagnosis: ICD10 Code, I00-I99, Define	I00-I99	Diseases of the circulatory system	258106	Poor venous access	ICD10CM	1	I87.8		Other specified disorders of veins	IMO	GHS-PACDC	DX_LEGACY	ACTIVE	N	1900-01-01	9999-12-31	2015-07-10
Diagnosis: ICD10 Code, I00-I99, Define	I00-I99	Diseases of the circulatory system	1192444	Other specified disorders of veins	ICD10CM	1	I87.8		Other specified disorders of veins	IMO	GHS-PACDC	IMO_2016_R	DELETED	Y	1900-01-01	9999-12-31	2016-06-02
Diagnosis: ICD10 Code, I00-I99, Define	I00-I99	Diseases of the circulatory system	116042	Venous vascular disease or syndrome	ICD10CM	1	I87.9		Disorder of vein, unspecified	IMO	GHS-PACDC	DX_LEGACY	ACTIVE	N	1900-01-01	9999-12-31	2015-07-10
Diagnosis: ICD10 Code, I00-I99, Define	I00-I99	Diseases of the circulatory system	51759	Lymphangiectasis	ICD10CM	1	I89.0		Lymphedema, not elsewhere classified	IMO	GHS-PACDC	DX_LEGACY	ACTIVE	N	1900-01-01	9999-12-31	2015-07-10
Diagnosis: ICD10 Code, I00-I99, Define	I00-I99	Diseases of the circulatory system	378055	Meige disease (lymphedema praecox)	ICD10CM	1	I89.0		Lymphedema, not elsewhere classified	IMO	GHS-PACDC	DX_LEGACY	ACTIVE	N	1900-01-01	9999-12-31	2015-07-10
Diagnosis: ICD10 Code, I00-I99, Define	I00-I99	Diseases of the circulatory system	1274542	Secondary intestinal lymphangiectasis	ICD10CM	1	I89.0		Lymphedema, not elsewhere classified	IMO	GHS-PACDC	IMO_2017_R	ACTIVE	N	1900-01-01	9999-12-31	2016-09-30
Diagnosis: ICD10 Code, I00-I99, Define	I00-I99	Diseases of the circulatory system	1132863	Lymphedema of left arm	ICD10CM	1	I89.0		Lymphedema, not elsewhere classified	IMO	GHS-PACDC	IMO_2016_R	ACTIVE	N	1900-01-01	9999-12-31	2016-06-02
Diagnosis: ICD10 Code, I00-I99, Define	I00-I99	Diseases of the circulatory system	38723	Acquired lymphedema	ICD10CM	1	I89.0		Lymphedema, not elsewhere classified	IMO	GHS-PACDC	DX_LEGACY	ACTIVE	N	1900-01-01	9999-12-31	2015-07-10
Diagnosis: ICD10 Code, I00-I99, Define	I00-I99	Diseases of the circulatory system	237963	Pelvic lymphangitis	ICD10CM	1	I89.1		Lymphangitis	IMO	GHS-PACDC	DX_LEGACY	ACTIVE	N	1900-01-01	9999-12-31	2015-07-10
Diagnosis: ICD10 Code, I00-I99, Define	I00-I99	Diseases of the circulatory system	689078	Lymphocele of upper extremity secondary to dialysis shunt, subsequent encounter	ICD10CM	2	I89.8	T82.898D,I89.8	Other specified noninfective disorders of lymphatic vess	IMO	GHS-PACDC	IMO_2015_R	ACTIVE	N	1900-01-01	9999-12-31	2015-07-10
Diagnosis: ICD10 Code, I00-I99, Define	I00-I99	Diseases of the circulatory system	1366953	Oth noninfective disorders of lymphatic vessels and nodes	ICD10CM	1	I89.8		Other specified noninfective disorders of lymphatic vess	IMO	GHS-PACDC	IMO_2018_R	ACTIVE	N	1900-01-01	9999-12-31	2017-10-30
Diagnosis: ICD10 Code, I00-I99, Define	I00-I99	Diseases of the circulatory system	229018	Arm lymphocele, left	ICD10CM	1	I89.8		Other specified noninfective disorders of lymphatic vess	IMO	GHS-PACDC	DX_LEGACY	ACTIVE	N	1900-01-01	9999-12-31	2015-07-10
Diagnosis: ICD10 Code, I00-I99, Define	I00-I99	Diseases of the circulatory system	253582	Inguinal lymphocyst	ICD10CM	1	I89.8		Other specified noninfective disorders of lymphatic vess	IMO	GHS-PACDC	DX_LEGACY	ACTIVE	N	1900-01-01	9999-12-31	2015-07-10
Diagnosis: ICD10 Code, I00-I99, Define	I00-I99	Diseases of the circulatory system	51775	Lymphatic cyst	ICD10CM	1	I89.8		Other specified noninfective disorders of lymphatic vess	IMO	GHS-PACDC	DX_LEGACY	ACTIVE	N	1900-01-01	9999-12-31	2015-07-10
Diagnosis: ICD10 Code, I00-I99, Define	I00-I99	Diseases of the circulatory system	163070	Disorder of lymphatic vessel	ICD10CM	1	I89.9		Noninfective disorder of lymphatic vessels and lymph nc	IMO	GHS-PACDC	DX_LEGACY	ACTIVE	N	1900-01-01	9999-12-31	2015-07-10
Diagnosis: ICD10 Code, I00-I99, Define	I00-I99	Diseases of the circulatory system	148944	Disorder of lymphatic system	ICD10CM	1	I89.9		Noninfective disorder of lymphatic vessels and lymph nc	IMO	GHS-PACDC	DX_LEGACY	ACTIVE	N	1900-01-01	9999-12-31	2015-07-10
Diagnosis: ICD10 Code, I00-I99, Define	I00-I99	Diseases of the circulatory system	50093	Hypotension, postural	ICD10CM	1	I95.1		Orthostatic hypotension	IMO	GHS-PACDC	DX_LEGACY	ACTIVE	N	1900-01-01	9999-12-31	2015-07-10
Diagnosis: ICD10 Code, I00-I99, Define	I00-I99	Diseases of the circulatory system	5319	Orthostatic hypotension	ICD10CM	1	I95.1		Orthostatic hypotension	IMO	GHS-PACDC	DX_LEGACY	ACTIVE	N	1900-01-01	9999-12-31	2015-07-10
Diagnosis: ICD10 Code, I00-I99, Define	I00-I99	Diseases of the circulatory system	173699	Postural orthostatic tachycardia syndrome	ICD10CM	2	I95.1	R00.0,I95.1	Orthostatic hypotension	IMO	GHS-PACDC	DX_LEGACY	ACTIVE	N	1900-01-01	9999-12-31	2015-07-10
Diagnosis: ICD10 Code, I00-I99, Define	I00-I99	Diseases of the circulatory system	32457	Hypoadrenergic postural hypotension (HCC)	ICD10CM	2	I95.1	E27.49,I95.1	Orthostatic hypotension	IMO	GHS-PACDC	DX_LEGACY	ACTIVE	N	1900-01-01	9999-12-31	2015-07-10
Diagnosis: ICD10 Code, I00-I99, Define	I00-I99	Diseases of the circulatory system	229304	Zenapax hypotension	ICD10CM	1	I95.2	I95.2,T45.1X5A	Hypotension due to drugs	IMO	GHS-PACDC	DX_LEGACY	ACTIVE	N	2017-09-17	9999-12-31	2017-10-30
Diagnosis: ICD10 Code, I00-I99, Define	I00-I99	Diseases of the circulatory system	162626	Hypotension due to drugs	ICD10CM	1	I95.2		Hypotension due to drugs	IMO	GHS-PACDC	DX_LEGACY	ACTIVE	N	1900-01-01	9999-12-31	2015-07-10
Diagnosis: ICD10 Code, I00-I99, Define	I00-I99	Diseases of the circulatory system	1260028	Hypotension due to medication	ICD10CM	1	I95.2		Hypotension due to drugs	IMO	GHS-PACDC	IMO_2017_R	ACTIVE	N	1900-01-01	9999-12-31	2016-09-30
Diagnosis: ICD10 Code, I00-I99, Define	I00-I99	Diseases of the circulatory system	163073	Hemodialysis-associated hypotension	ICD10CM	1	I95.3		Hypotension of hemodialysis	IMO	GHS-PACDC	DX_LEGACY	ACTIVE	N	1900-01-01	9999-12-31	2015-07-10
Diagnosis: ICD10 Code, I00-I99, Define	I00-I99	Diseases of the circulatory system	5322	Hypotension	ICD10CM	1	I95.9		Hypotension, unspecified	IMO	GHS-PACDC	DX_LEGACY	ACTIVE	N	1900-01-01	9999-12-31	2015-07-10
Diagnosis: ICD10 Code, I00-I99, Define	I00-I99	Diseases of the circulatory system	67805	Low blood pressure	ICD10CM	1	I95.9		Hypotension, unspecified	IMO	GHS-PACDC	DX_LEGACY	ACTIVE	N	1900-01-01	9999-12-31	2015-07-10
Diagnosis: ICD10 Code, I00-I99, Define	I00-I99	Diseases of the circulatory system	373603	Transient hypotension	ICD10CM	1	I95.9		Hypotension, unspecified	IMO	GHS-PACDC	DX_LEGACY	ACTIVE	N	2016-04-10	9999-12-31	2015-07-10
Diagnosis: ICD10 Code, I00-I99, Define	I00-I99	Diseases of the circulatory system	257542	Ischemic necrosis of foot (HCC)	ICD10CM	1	I96		Gangrene, not elsewhere classified	IMO	GHS-PACDC	DX_LEGACY	ACTIVE	N	1900-01-01	9999-12-31	2015-07-10
Diagnosis: ICD10 Code, I00-I99, Define	I00-I99	Diseases of the circulatory system	839466	Necrotic hand wound, right, subsequent encounter (HCC)	ICD10CM	2	I96	S61.401D,I96	Gangrene, not elsewhere classified	IMO	GHS-PACDC	IMO_2015_R	ACTIVE	N	2017-03-31	9999-12-31	2017-04-14
Diagnosis: ICD10 Code, I00-I99, Define	I00-I99	Diseases of the circulatory system	717906	Skin flap necrosis, subsequent encounter	ICD10CM	2	I96	T88.8XXD,I96	Gangrene, not elsewhere classified	IMO	GHS-PACDC	IMO_2015_R	DELETED	Y	1900-01-01	9999-12-31	2015-07-10
Diagnosis: ICD10 Code, I00-I99, Define	I00-I99	Diseases of the circulatory system	324449	External ear necrosis	ICD10CM	1	I96		Gangrene, not elsewhere classified	IMO	GHS-PACDC	DX_LEGACY	ACTIVE	N	2017-09-17	9999-12-31	2017-10-30
Diagnosis: ICD10 Code, I00-I99, Define	I00-I99	Diseases of the circulatory system	462572	Progressive bacterial synergistic gangrene with necrosis of muscle (HCC)	ICD10CM	2	I96	L98.493,I96	Gangrene, not elsewhere classified	IMO	GHS-PACDC	IMO_2015_R	ACTIVE	N	1900-01-01	9999-12-31	2015-07-10
Diagnosis: ICD10 Code, I00-I99, Define	I00-I99	Diseases of the circulatory system	665653	Necrosis of surgical wound, initial encounter (HCC)	ICD10CM	2	I96	T81.89XA,I96	Gangrene, not elsewhere classified	IMO	GHS-PACDC	IMO_2015_R	ACTIVE	N	1900-01-01	9999-12-31	2015-07-10
Diagnosis: ICD10 Code, I00-I99, Define	I00-I99	Diseases of the circulatory system	758389	Necrotic hand wound, right, initial encounter (HCC)	ICD10CM	2	I96	S61.401A,I96	Gangrene, not elsewhere classified	IMO	GHS-PACDC	IMO_2015_R	ACTIVE	N	2017-03-31	9999-12-31	2017-04-14
Diagnosis: ICD10 Code, I00-I99, Define	I00-I99	Diseases of the circulatory system	931021	Trash foot, bilateral (HCC)	ICD10CM	2	I96	I75.023,I96	Gangrene, not elsewhere classified	IMO	GHS-PACDC	IMO_2015_R	ACTIVE	N	1900-01-01	2016-05-07	2015-07-10
Diagnosis: ICD10 Code, I00-I99, Define	I00-I99	Diseases of the circulatory system	257296	Gangrene of digit	ICD10CM	1	I96		Gangrene, not elsewhere classified	IMO	GHS-PACDC	DX_LEGACY	DELETED	Y	1900-01-01	9999-12-31	2015-07-10
Diagnosis: ICD10 Code, I00-I99, Define	I00-I99	Diseases of the circulatory system	720936	Pressure ulcer with gangrene, stage III	ICD10CM	2	I96	L89.93,I96	Gangrene, not elsewhere classified	IMO	GHS-PACDC	IMO_2015_R	DELETED	Y	2016-04-10	9999-12-31	2015-07-10
Diagnosis: ICD10 Code, I00-I99, Define	I00-I99	Diseases of the circulatory system	1350512	Gangrene of toe of left foot (HCC)	ICD10CM	1	I96		Gangrene, not elsewhere classified	IMO	GHS-PACDC	IMO_2018_R	ACTIVE	N	1900-01-01	9999-12-31	2017-10-30
Diagnosis: ICD10 Code, I00-I99, Define	I00-I99	Diseases of the circulatory system	462582	Tropical phagedenic ulcer with necrosis of bone (HCC)	ICD10CM	2	I96	L98.494,I96	Gangrene, not elsewhere classified	IMO	GHS-PACDC	IMO_2015_R	ACTIVE	N	1900-01-01	2017-10-14	2015-07-10
Diagnosis: ICD10 Code, I00-I99, Define	I00-I99	Diseases of the circulatory system	889107	Pressure sore with gangrene, stage I	ICD10CM	2	I96	L89.91,I96	Gangrene, not elsewhere classified	IMO	GHS-PACDC	IMO_2015_R	DELETED	Y	2016-04-10	9999-12-31	2015-07-10
Diagnosis: ICD10 Code, I00-I99, Define	I00-I99	Diseases of the circulatory system	794211	Pressure ulcer with gangrene, stage II	ICD10CM	2	I96	L89.92,I96	Gangrene, not elsewhere classified	IMO	GHS-PACDC	IMO_2015_R	DELETED	Y	2016-04-10	9999-12-31	2015-07-10
Diagnosis: ICD10 Code, I00-I99, Define	I00-I99	Diseases of the circulatory system	720630	Necrotic hand wound, right, sequela (HCC)	ICD10CM	2	I96	S61.401S,I96	Gangrene, not elsewhere classified	IMO	GHS-PACDC	IMO_2015_R	ACTIVE	N	2017-03-31	9999-12-31	2017-04-14
Diagnosis: ICD10 Code, I00-I99, Define	I00-I99	Diseases of the circulatory system	527786	Epidermal necrosis present on biopsy of skin (HCC)	ICD10CM	1	I96		Gangrene, not elsewhere classified	IMO	GHS-PACDC	IMO_2015_R	ACTIVE	N	1900-01-01	9999-12-31	2015-07-10
Diagnosis: ICD10 Code, I00-I99, Define	I00-I99	Diseases of the circulatory system	493578	Necrosis of muscle due to infection by anaerobic bacteria (HCC)	ICD10CM	1	I96	I96,B96.89	Gangrene, not elsewhere classified	IMO	GHS-PACDC	IMO_2015_R	ACTIVE	N	1900-01-01	9999-12-31	2015-07-10
Diagnosis: ICD10 Code, I00-I99, Define	I00-I99	Diseases of the circulatory system	991809	Necrotic wound of right hand, sequela (HCC)	ICD10CM	2	I96	S61.401S,I96	Gangrene, not elsewhere classified	IMO	GHS-PACDC	IMO_2015_R	ACTIVE	N	2017-03-31	9999-12-31	2017-04-14
Diagnosis: ICD10 Code, I00-I99, Define	I00-I99	Diseases of the circulatory system	269387	Postprocedural cardiac arrest following cardiac surgery	ICD10CM	1	I97.120		Postprocedural cardiac arrest following cardiac surgery	IMO	GHS-PACDC	DX_LEGACY	ACTIVE	N	1900-01-01	9999-12-31	2015-07-10
Diagnosis: ICD10 Code, I00-I99, Define	I00-I99	Diseases of the circulatory system	588220	Postprocedural heart failure following other surgery	ICD10CM	1	I97.131		Postprocedural heart failure following other surgery	IMO	GHS-PACDC	IMO_2015_R	ACTIVE	Y	1900-01-01	9999-12-31	2015-07-10
Diagnosis: ICD10 Code, I00-I99, Define	I00-I99	Diseases of the circulatory system	1179748	Other postprocedural cardiac functional disturbances following cardiac surgery	ICD10CM	1	I97.190		Other postprocedural cardiac functional disturbances fo	IMO	GHS-PACDC	IMO_2016_R	ACTIVE	N	1900-01-01	9999-12-31	2016-06-02
Diagnosis: ICD10 Code, I00-I99, Define	I00-I99	Diseases of the circulatory system	523943	Intraoperative hemorrhage involving circulatory system complicating cardiac bypass	ICD10CM	1	I97.411		Intraoperative hemorrhage and hematoma of a circulatc	IMO	GHS-PACDC	IMO_2015_R	ACTIVE	N	1900-01-01	9999-12-31	2015-07-10
Diagnosis: ICD10 Code, I00-I99, Define	I00-I99	Diseases of the circulatory system	597262	Intraoperative hemorrhage and hematoma of a circulatory system organ or structure complicati	ICD10CM	1	I97.418		Intraoperative hemorrhage and hematoma of a circulatc	IMO	GHS-PACDC	IMO_2015_R	ACTIVE	Y	1900-01-01	9999-12-31	2015-07-10
Diagnosis: ICD10 Code, I00-I99, Define	I00-I99	Diseases of the circulatory system	1184334	Accidental puncture and laceration of a circulatory system organ or structure during a circulator	ICD10CM	1	I97.51		Accidental puncture and laceration of a circulatory syste	IMO	GHS-PACDC	IMO_2016_R	ACTIVE	N	1900-01-01	9999-12-31	2016-06-02
Diagnosis: ICD10 Code, I00-I99, Define	I00-I99	Diseases of the circulatory system	597960	Postprocedural hemorrhage of a circulatory system organ or structure following cardiac bypass	ICD10CM	1	I97.611		Postprocedural hemorrhage of a circulatory system orga	IMO	GHS-PACDC	IMO_2015_R	ACTIVE	Y	1900-01-01	9999-12-31	2015-07-10
Diagnosis: ICD10 Code, I00-I99, Define	I00-I99	Diseases of the circulatory system	232320	Percutaneous transluminal coronary angioplasty bleed	ICD10CM	1	I97.618		Postprocedural hemorrhage of a circulatory system orga	IMO	GHS-PACDC	DX_LEGACY	ACTIVE	N	2016-04-10	9999-12-31	2015-07-10
Diagnosis: ICD10 Code, I00-I99, Define	I00-I99	Diseases of the circulatory system	524132	Postoperative hematoma involving circulatory system	ICD10CM	1	I97.62		Postprocedural hemorrhage, hematoma and seroma of	IMO	GHS-PACDC	IMO_2015_R	DELETED	Y	1900-01-01	2016-10-14	2015-07-10
Diagnosis: ICD10 Code, I00-I99, Define	I00-I99	Diseases of the circulatory system	246211	Hemorrhage of surgical wound following transplant	ICD10CM	1	I97.62		Postprocedural hemorrhage, hematoma and seroma of	IMO	GHS-PACDC	DX_LEGACY	DELETED	Y	1900-01-01	2016-10-14	2015-07-10
Diagnosis: ICD10 Code, I00-I99, Define	I00-I99	Diseases of the circulatory system	1290547	Postoperative seroma involving circulatory system after other circulatory system procedure	ICD10CM	1	I97.648		Postprocedural seroma of a circulatory system organ or	IMO	GHS-PACDC	IMO_2017_R	ACTIVE	N	1900-01-01	9999-12-31	2016-09-30
Diagnosis: ICD10 Code, I00-I99, Define	I00-I99	Diseases of the circulatory system	589373	Postprocedural cerebrovascular infarction following other surgery	ICD10CM	1	I97.821		Postprocedural cerebrovascular infarction following oth	IMO	GHS-PACDC	IMO_2015_R	ACTIVE	Y	1900-01-01	9999-12-31	2015-07-10
Diagnosis: ICD10 Code, I00-I99, Define	I00-I99	Diseases of the circulatory system	304073	Postoperative AV block	ICD10CM	1	I97.89	I97.89,I44.30	Other postprocedural complications and disorders of th	IMO	GHS-PACDC	DX_LEGACY	ACTIVE	N	1900-01-01	9999-12-31	2015-07-10
Diagnosis: ICD10 Code, I00-I99, Define	I00-I99	Diseases of the circulatory system	1107563	Postoperative surgical complication involving circulatory system associated with non-circulatory	ICD10CM	1	I97.89		Other postprocedural complications and disorders of th	IMO	GHS-PACDC	IMO_2016_R	ACTIVE	N	1900-01-01	9999-12-31	2016-06-02
Diagnosis: ICD10 Code, I00-I99, Define	I00-I99	Diseases of the circulatory system	1107550	Postoperative surgical complication involving circulatory system associated with non-cardiac pr	ICD10CM	1	I97.89		Other postprocedural complications and disorders of th	IMO	GHS-PACDC	IMO_2016_R	ACTIVE	N	1900-01-01	9999-12-31	2016-06-02
Diagnosis: ICD10 Code, I00-I99, Define	I00-I99	Diseases of the circulatory system	411782	Postoperative myocardial infarction (HCC)	ICD10CM	1	I97.89	I97.89,I21.3	Other postprocedural complications and disorders of th	IMO	GHS-PACDC	DX_LEGACY	ACTIVE	N	1900-01-01	2017-10-14	2015-07-10
Diagnosis: ICD10 Code, I00-I99, Define	I00-I99	Diseases of the circulatory system	687067	Intraoperative ischemia of hand, sequela	ICD10CM	2	I99.8	T81.89XS,I99.8	Other disorder of circulatory system	IMO	GHS-PACDC	IMO_2015_R	ACTIVE	N	1900-01-01	9999-12-31	2015-07-10
Diagnosis: ICD10 Code, I00-I99, Define	I00-I99	Diseases of the circulatory system	503360	Ischemia of left BKA site (HCC)	ICD10CM	2	I99.8	T87.89,I99.8	Other disorder of circulatory system	IMO	GHS-PACDC	IMO_2015_R	ACTIVE	N	2017-03-31	9999-12-31	2017-04-14
Diagnosis: ICD10 Code, I00-I99, Define	I00-I99	Diseases of the circulatory system	519711	Intermediate hyperglycemia with multiple microvascular complications	ICD10CM	2	I99.8	R73.9,I99.8	Other disorder of circulatory system	IMO	GHS-PACDC	IMO_2015_R	ACTIVE	N	1900-01-01	9999-12-31	2015-07-10
Diagnosis: ICD10 Code, I00-I99, Define	I00-I99	Diseases of the circulatory system	324591	Extremity ischemia	ICD10CM	1	I99.8		Other disorder of circulatory system	IMO	GHS-PACDC	DX_LEGACY	ACTIVE	N	1900-01-01	9999-12-31	2015-07-10

Diagnosis: ICD10 Code, I00-I99, Define	I00-I99	Diseases of the circulatory system	1125211	Ischemia of right upper extremity	ICD10CM	1	I99.8		Other disorder of circulatory system	IMO	GHS-PACDC	IMO_2016_R	ACTIVE	N	1900-01-01	9999-12-31	2016-06-02
Diagnosis: ICD10 Code, I00-I99, Define	I00-I99	Diseases of the circulatory system	1107541	Surgical complication involving circulatory system associated with non-cardiac procedure	ICD10CM	1	I99.8		Other disorder of circulatory system	IMO	GHS-PACDC	IMO_2016_R	ACTIVE	N	1900-01-01	9999-12-31	2016-06-02
Diagnosis: ICD10 Code, I00-I99, Define	I00-I99	Diseases of the circulatory system	503352	Ischemia of right BKA site (HCC)	ICD10CM	2	I99.8	T87.89,I99.8	Other disorder of circulatory system	IMO	GHS-PACDC	IMO_2015_R	ACTIVE	N	2017-03-31	9999-12-31	2017-04-14
Diagnosis: ICD10 Code, I00-I99, Define	I00-I99	Diseases of the circulatory system	123481	Disorder of the heart and blood vessels	ICD10CM	1	I99.9		Unspecified disorder of circulatory system	IMO	GHS-PACDC	DX_LEGACY	ACTIVE	N	1900-01-01	2016-05-07	2015-07-10
Diagnosis: ICD10 Code, I00-I99, Define	I00-I99	Diseases of the circulatory system	1099071	Venous complication NOS-deliver	ICD10CM	2	I99.9	O87.9,I99.9	Unspecified disorder of circulatory system	IMO	GHS-PACDC	IMO_2016_R	ACTIVE	N	1900-01-01	9999-12-31	2016-06-02
Diagnosis: ICD10 Code, I00-I99, Define	I00-I99	Diseases of the circulatory system	420926	Impaired circulatory function	ICD10CM	1	I99.9		Unspecified disorder of circulatory system	IMO	GHS-PACDC	DX_LEGACY	ACTIVE	N	1900-01-01	9999-12-31	2015-07-10
Diagnosis: ICD10 Code, I00-I99, Define	I00-I99	Diseases of the circulatory system	58130	Vascular problem	ICD10CM	1	I99.9		Unspecified disorder of circulatory system	IMO	GHS-PACDC	DX_LEGACY	ACTIVE	N	1900-01-01	9999-12-31	2015-07-10
Diagnosis: ICD10 Code, J00-J99 Define	J00-J99	Diseases of the respiratory system	1351773	Acute noninfective rhinitis	ICD10CM	1	J00		Acute nasopharyngitis [common cold]	IMO	GHS-PACDC	IMO_2018_R	ACTIVE	N	1900-01-01	9999-12-31	2017-10-30
Diagnosis: ICD10 Code, J00-J99 Define	J00-J99	Diseases of the respiratory system	37098	Acute rhinitis	ICD10CM	1	J00		Acute nasopharyngitis [common cold]	IMO	GHS-PACDC	DX_LEGACY	ACTIVE	N	1900-01-01	9999-12-31	2015-07-10
Diagnosis: ICD10 Code, J00-J99 Define	J00-J99	Diseases of the respiratory system	21004	Subacute bronchitis	ICD10CM	1	J00		Acute nasopharyngitis [common cold]	IMO	GHS-PACDC	DX_LEGACY	ACTIVE	N	1900-01-01	2016-05-07	2015-07-10
Diagnosis: ICD10 Code, J00-J99 Define	J00-J99	Diseases of the respiratory system	237338	Sinusitis, acute ethmoidal	ICD10CM	1	J01.20		Acute ethmoidal sinusitis, unspecified	IMO	GHS-PACDC	DX_LEGACY	ACTIVE	N	1900-01-01	9999-12-31	2015-07-10
Diagnosis: ICD10 Code, J00-J99 Define	J00-J99	Diseases of the respiratory system	20884	Acute suppurative inflammation of nasal sinus	ICD10CM	1	J01.90		Acute sinusitis, unspecified	IMO	GHS-PACDC	DX_LEGACY	ACTIVE	N	1900-01-01	9999-12-31	2015-07-10
Diagnosis: ICD10 Code, J00-J99 Define	J00-J99	Diseases of the respiratory system	56134	Epidemic sore throat	ICD10CM	1	J02.0		Streptococcal pharyngitis	IMO	GHS-PACDC	DX_LEGACY	ACTIVE	N	1900-01-01	9999-12-31	2015-07-10
Diagnosis: ICD10 Code, J00-J99 Define	J00-J99	Diseases of the respiratory system	372405	Streptococcal tonsillopharyngitis	ICD10CM	1	J02.0	J02.0,J03.00	Streptococcal pharyngitis	IMO	GHS-PACDC	DX_LEGACY	ACTIVE	N	1900-01-01	2017-04-27	2015-07-10
Diagnosis: ICD10 Code, J00-J99 Define	J00-J99	Diseases of the respiratory system	36197	Streptococcal angina	ICD10CM	1	J02.0		Streptococcal pharyngitis	IMO	GHS-PACDC	DX_LEGACY	ACTIVE	N	1900-01-01	9999-12-31	2015-07-10
Diagnosis: ICD10 Code, J00-J99 Define	J00-J99	Diseases of the respiratory system	36195	Streptococcal sore throat	ICD10CM	1	J02.0		Streptococcal pharyngitis	IMO	GHS-PACDC	DX_LEGACY	ACTIVE	N	1900-01-01	9999-12-31	2015-07-10
Diagnosis: ICD10 Code, J00-J99 Define	J00-J99	Diseases of the respiratory system	384857	Periodic fever, aphthous stomatitis, pharyngitis, adenitis (PFAPA) syndrome (HCC)	ICD10CM	4	J02.9	R50.81,K12.0,I88.9,J02.9	Acute pharyngitis, unspecified	IMO	GHS-PACDC	DX_LEGACY	ACTIVE	N	1900-01-01	2016-05-07	2015-07-10
Diagnosis: ICD10 Code, J00-J99 Define	J00-J99	Diseases of the respiratory system	528230	Respiratory syncytial virus (RSV) pharyngitis	ICD10CM	1	J02.9		Acute pharyngitis, unspecified	IMO	GHS-PACDC	IMO_2015_R	ACTIVE	N	1900-01-01	9999-12-31	2015-07-10
Diagnosis: ICD10 Code, J00-J99 Define	J00-J99	Diseases of the respiratory system	20934	Gangrenous pharyngitis	ICD10CM	1	J02.9		Acute pharyngitis, unspecified	IMO	GHS-PACDC	DX_LEGACY	ACTIVE	N	1900-01-01	9999-12-31	2015-07-10
Diagnosis: ICD10 Code, J00-J99 Define	J00-J99	Diseases of the respiratory system	1097628	Acute pharyngitis, unspecified pharyngitis type	ICD10CM	1	J02.9		Acute pharyngitis, unspecified	IMO	GHS-PACDC	IMO_2015_R	DELETED	Y	1900-01-01	9999-12-31	2015-07-10
Diagnosis: ICD10 Code, J00-J99 Define	J00-J99	Diseases of the respiratory system	384858	PFAPA syndrome (HCC)	ICD10CM	1	J02.9	J02.9,R50.81,K12.0,I88.9	Acute pharyngitis, unspecified	IMO	GHS-PACDC	DX_LEGACY	ACTIVE	N	2016-04-10	2016-10-14	2015-07-10
Diagnosis: ICD10 Code, J00-J99 Define	J00-J99	Diseases of the respiratory system	410313	Acute bacterial tonsillitis	ICD10CM	1	J03.80	J03.80,B96.89	Acute tonsillitis due to other specified organisms	IMO	GHS-PACDC	DX_LEGACY	ACTIVE	N	2016-04-10	9999-12-31	2015-07-10
Diagnosis: ICD10 Code, J00-J99 Define	J00-J99	Diseases of the respiratory system	592980	Acute recurrent tonsillitis due to other specified organisms	ICD10CM	1	J03.81		Acute recurrent tonsillitis due to other specified organis	IMO	GHS-PACDC	IMO_2015_R	ACTIVE	Y	1900-01-01	9999-12-31	2015-07-10
Diagnosis: ICD10 Code, J00-J99 Define	J00-J99	Diseases of the respiratory system	73528	Acute follicular tonsillitis	ICD10CM	1	J03.90		Acute tonsillitis, unspecified	IMO	GHS-PACDC	DX_LEGACY	ACTIVE	N	1900-01-01	9999-12-31	2015-07-10
Diagnosis: ICD10 Code, J00-J99 Define	J00-J99	Diseases of the respiratory system	33405	Suppurative tonsillitis	ICD10CM	1	J03.90		Acute tonsillitis, unspecified	IMO	GHS-PACDC	DX_LEGACY	ACTIVE	N	1900-01-01	9999-12-31	2015-07-10
Diagnosis: ICD10 Code, J00-J99 Define	J00-J99	Diseases of the respiratory system	33406	Septic tonsillitis	ICD10CM	1	J03.90		Acute tonsillitis, unspecified	IMO	GHS-PACDC	DX_LEGACY	ACTIVE	N	1900-01-01	9999-12-31	2015-07-10
Diagnosis: ICD10 Code, J00-J99 Define	J00-J99	Diseases of the respiratory system	206840	Recurrent acute tonsillitis	ICD10CM	1	J03.91		Acute recurrent tonsillitis, unspecified	IMO	GHS-PACDC	DX_LEGACY	ACTIVE	N	1900-01-01	9999-12-31	2015-07-10
Diagnosis: ICD10 Code, J00-J99 Define	J00-J99	Diseases of the respiratory system	250229	Acute recurrent tonsillitis	ICD10CM	1	J03.91		Acute recurrent tonsillitis, unspecified	IMO	GHS-PACDC	DX_LEGACY	ACTIVE	N	1900-01-01	9999-12-31	2015-07-10
Diagnosis: ICD10 Code, J00-J99 Define	J00-J99	Diseases of the respiratory system	235620	Laryngitis without obstruction, acute	ICD10CM	1	J04.0		Acute laryngitis	IMO	GHS-PACDC	DX_LEGACY	ACTIVE	N	1900-01-01	9999-12-31	2015-07-10
Diagnosis: ICD10 Code, J00-J99 Define	J00-J99	Diseases of the respiratory system	235622	Laryngitis with obstruction, acute	ICD10CM	1	J04.0		Acute laryngitis	IMO	GHS-PACDC	DX_LEGACY	ACTIVE	N	1900-01-01	2016-05-07	2015-07-10
Diagnosis: ICD10 Code, J00-J99 Define	J00-J99	Diseases of the respiratory system	1111238	Acute tracheitis without airway obstruction	ICD10CM	1	J04.10		Acute tracheitis without obstruction	IMO	GHS-PACDC	IMO_2016_R	ACTIVE	N	1900-01-01	9999-12-31	2016-06-02
Diagnosis: ICD10 Code, J00-J99 Define	J00-J99	Diseases of the respiratory system	532902	Acute supraglottitis with epiglottitis	ICD10CM	1	J04.30	J04.30,J05.10	Supraglottitis, unspecified, without obstruction	IMO	GHS-PACDC	IMO_2015_R	ACTIVE	N	1900-01-01	9999-12-31	2015-07-10
Diagnosis: ICD10 Code, J00-J99 Define	J00-J99	Diseases of the respiratory system	170026	Acute Haemophilus influenzae laryngitis with obstruction	ICD10CM	1	J05.0	J05.0,B96.3	Acute obstructive laryngitis [croup]	IMO	GHS-PACDC	DX_LEGACY	ACTIVE	N	2016-04-10	9999-12-31	2015-07-10
Diagnosis: ICD10 Code, J00-J99 Define	J00-J99	Diseases of the respiratory system	1311989	Croup in child	ICD10CM	1	J05.0		Acute obstructive laryngitis [croup]	IMO	GHS-PACDC	IMO_2017_R	ACTIVE	N	1900-01-01	9999-12-31	2017-04-14
Diagnosis: ICD10 Code, J00-J99 Define	J00-J99	Diseases of the respiratory system	170026	Acute Haemophilus influenzae laryngitis with obstruction	ICD10CM	1	J05.0		Acute obstructive laryngitis [croup]	IMO	GHS-PACDC	DX_LEGACY	ACTIVE	N	1900-01-01	2016-05-07	2015-07-10
Diagnosis: ICD10 Code, J00-J99 Define	J00-J99	Diseases of the respiratory system	156563	Acute epiglottiditis	ICD10CM	1	J05.10		Acute epiglottitis without obstruction	IMO	GHS-PACDC	DX_LEGACY	ACTIVE	N	1900-01-01	9999-12-31	2015-07-10
Diagnosis: ICD10 Code, J00-J99 Define	J00-J99	Diseases of the respiratory system	1111256	Acute epiglottitis without airway obstruction	ICD10CM	1	J05.10		Acute epiglottitis without obstruction	IMO	GHS-PACDC	IMO_2016_R	ACTIVE	N	1900-01-01	9999-12-31	2016-06-02
Diagnosis: ICD10 Code, J00-J99 Define	J00-J99	Diseases of the respiratory system	36543	Haemophilus influenzae epiglottitis	ICD10CM	1	J05.10	J05.10,A49.2	Acute epiglottitis without obstruction	IMO	GHS-PACDC	DX_LEGACY	ACTIVE	N	1900-01-01	9999-12-31	2015-07-10
Diagnosis: ICD10 Code, J00-J99 Define	J00-J99	Diseases of the respiratory system	502982	Asthma occurring only with upper respiratory infection	ICD10CM	2	J06.9	J45.909,J06.9	Acute upper respiratory infection, unspecified	IMO	GHS-PACDC	IMO_2015_R	ACTIVE	N	1900-01-01	9999-12-31	2015-07-10
Diagnosis: ICD10 Code, J00-J99 Define	J00-J99	Diseases of the respiratory system	102882	URTI (acute upper respiratory infection)	ICD10CM	1	J06.9		Acute upper respiratory infection, unspecified	IMO	GHS-PACDC	DX_LEGACY	ACTIVE	N	1900-01-01	9999-12-31	2015-07-10
Diagnosis: ICD10 Code, J00-J99 Define	J00-J99	Diseases of the respiratory system	259315	Viral upper respiratory illness	ICD10CM	1	J06.9		Acute upper respiratory infection, unspecified	IMO	GHS-PACDC	DX_LEGACY	ACTIVE	N	1900-01-01	2016-05-07	2015-07-10
Diagnosis: ICD10 Code, J00-J99 Define	J00-J99	Diseases of the respiratory system	417584	Bacterial upper respiratory infection	ICD10CM	1	J06.9	J06.9,B96.89	Acute upper respiratory infection, unspecified	IMO	GHS-PACDC	DX_LEGACY	ACTIVE	N	2016-04-10	9999-12-31	2015-07-10
Diagnosis: ICD10 Code, J00-J99 Define	J00-J99	Diseases of the respiratory system	110069	Infection of the upper respiratory tract	ICD10CM	1	J06.9		Acute upper respiratory infection, unspecified	IMO	GHS-PACDC	DX_LEGACY	ACTIVE	N	1900-01-01	9999-12-31	2015-07-10
Diagnosis: ICD10 Code, J00-J99 Define	J00-J99	Diseases of the respiratory system	1273943	Fungal infection of upper respiratory tract	ICD10CM	1	J06.9	J06.9,B49	Acute upper respiratory infection, unspecified	IMO	GHS-PACDC	IMO_2017_R	ACTIVE	N	1900-01-01	9999-12-31	2016-09-30
Diagnosis: ICD10 Code, J00-J99 Define	J00-J99	Diseases of the respiratory system	1107928	Pneumonia of both lungs due to influenza A virus	ICD10CM	1	J09.X1		Influenza due to identified novel influenza A virus with p	IMO	GHS-PACDC	IMO_2016_R	ACTIVE	N	2017-09-17	9999-12-31	2017-10-30
Diagnosis: ICD10 Code, J00-J99 Define	J00-J99	Diseases of the respiratory system	426663	Pneumonia due to influenza A virus	ICD10CM	1	J09.X1		Influenza due to identified novel influenza A virus with p	IMO	GHS-PACDC	IMO_2015_R	ACTIVE	N	2016-04-10	9999-12-31	2015-07-10
Diagnosis: ICD10 Code, J00-J99 Define	J00-J99	Diseases of the respiratory system	313677	Novel influenza A pharyngitis	ICD10CM	1	J09.X2		Influenza due to identified novel influenza A virus with c	IMO	GHS-PACDC	DX_LEGACY	ACTIVE	N	2016-09-17	9999-12-31	2016-09-30
Diagnosis: ICD10 Code, J00-J99 Define	J00-J99	Diseases of the respiratory system	248163	Acute respiratory infection due to avian influenza virus	ICD10CM	1	J09.X2		Influenza due to identified novel influenza A virus with c	IMO	GHS-PACDC	DX_LEGACY	ACTIVE	N	1900-01-01	9999-12-31	2015-07-10
Diagnosis: ICD10 Code, J00-J99 Define	J00-J99	Diseases of the respiratory system	313680	Novel influenza A with involvement of gastrointestinal tract	ICD10CM	1	J09.X3		Influenza due to identified novel influenza A virus with g	IMO	GHS-PACDC	DX_LEGACY	ACTIVE	N	2016-09-17	9999-12-31	2016-09-30
Diagnosis: ICD10 Code, J00-J99 Define	J00-J99	Diseases of the respiratory system	270754	Influenza due to identified avian influenza virus with myocarditis	ICD10CM	1	J09.X9	J09.X9,I41	Influenza due to identified novel influenza A virus with c	IMO	GHS-PACDC	DX_LEGACY	ACTIVE	N	1900-01-01	9999-12-31	2015-07-10
Diagnosis: ICD10 Code, J00-J99 Define	J00-J99	Diseases of the respiratory system	248180	Novel 2009 influenza H1N1	ICD10CM	1	J10.1		Influenza due to other identified influenza virus with oth	IMO	GHS-PACDC	DX_LEGACY	ACTIVE	N	1900-01-01	9999-12-31	2015-07-10
Diagnosis: ICD10 Code, J00-J99 Define	J00-J99	Diseases of the respiratory system	606775	Influenza due to other identified influenza virus with other respiratory manifestations	ICD10CM	1	J10.1		Influenza due to other identified influenza virus with oth	IMO	GHS-PACDC	IMO_2015_R	ACTIVE	Y	1900-01-01	9999-12-31	2015-07-10
Diagnosis: ICD10 Code, J00-J99 Define	J00-J99	Diseases of the respiratory system	1114047	Acute respiratory infection due to influenza A subtype H1N1 virus	ICD10CM	1	J10.1		Influenza due to other identified influenza virus with oth	IMO	GHS-PACDC	IMO_2016_R	ACTIVE	N	1900-01-01	9999-12-31	2016-06-02
Diagnosis: ICD10 Code, J00-J99 Define	J00-J99	Diseases of the respiratory system	1114409	Encephalopathy due to influenza A subtype H1N1 virus	ICD10CM	1	J10.81		Influenza due to other identified influenza virus with en	IMO	GHS-PACDC	IMO_2016_R	ACTIVE	N	1900-01-01	9999-12-31	2016-06-02
Diagnosis: ICD10 Code, J00-J99 Define	J00-J99	Diseases of the respiratory system	375305	Influenza due to identified novel h1n1 influenza virus w/otitis media	ICD10CM	1	J10.83		Influenza due to other identified influenza virus with oti	IMO	GHS-PACDC	DX_LEGACY	ACTIVE	N	1900-01-01	9999-12-31	2015-07-10
Diagnosis: ICD10 Code, J00-J99 Define	J00-J99	Diseases of the respiratory system	591734	Influenza due to other identified influenza virus with otitis media	ICD10CM	1	J10.83		Influenza due to other identified influenza virus with oti	IMO	GHS-PACDC	IMO_2015_R	ACTIVE	Y	1900-01-01	9999-12-31	2015-07-10
Diagnosis: ICD10 Code, J00-J99 Define	J00-J99	Diseases of the respiratory system	613494	Influenza due to unidentified influenza virus	ICD10CM	1	J11		Influenza due to unidentified influenza virus	IMO	GHS-PACDC	IMO_2015_R	ACTIVE	Y	1900-01-01	9999-12-31	2015-07-10
Diagnosis: ICD10 Code, J00-J99 Define	J00-J99	Diseases of the respiratory system	1107928	Pneumonia of both lungs due to influenza A virus	ICD10CM	1	J11.00		Influenza due to unidentified influenza virus with unspe	IMO	GHS-PACDC	IMO_2016_R	ACTIVE	N	1900-01-01	2017-10-14	2016-06-02
Diagnosis: ICD10 Code, J00-J99 Define	J00-J99	Diseases of the respiratory system	99531	Influenza and pneumonia	ICD10CM	1	J11.00		Influenza due to unidentified influenza virus with unspe	IMO	GHS-PACDC	DX_LEGACY	ACTIVE	N	1900-01-01	9999-12-31	2015-07-10
Diagnosis: ICD10 Code, J00-J99 Define	J00-J99	Diseases of the respiratory system	270766	Influenza due to unidentified influenza virus with specified pneumonia	ICD10CM	1	J11.08		Influenza due to unidentified influenza virus with specifi	IMO	GHS-PACDC	DX_LEGACY	ACTIVE	N	1900-01-01	9999-12-31	2015-07-10
Diagnosis: ICD10 Code, J00-J99 Define	J00-J99	Diseases of the respiratory system	91091	Influenza with respiratory manifestations	ICD10CM	1	J11.1		Influenza due to unidentified influenza virus with other	IMO	GHS-PACDC	DX_LEGACY	DELETED	Y	2016-04-10	9999-12-31	2015-07-10
Diagnosis: ICD10 Code, J00-J99 Define	J00-J99	Diseases of the respiratory system	315330	Influenza-like illness	ICD10CM	1	J11.1		Influenza due to unidentified influenza virus with other	IMO	GHS-PACDC	DX_LEGACY	ACTIVE	N	1900-01-01	2016-10-14	2015-07-10
Diagnosis: ICD10 Code, J00-J99 Define	J00-J99	Diseases of the respiratory system	1181356	Influenza due to unidentified influenza virus with other respiratory manifestations	ICD10CM	1	J11.1		Influenza due to unidentified influenza virus with other	IMO	GHS-PACDC	IMO_2016_R	ACTIVE	N	1900-01-01	9999-12-31	2016-06-02
Diagnosis: ICD10 Code, J00-J99 Define	J00-J99	Diseases of the respiratory system	248531	Influenzal bronchitis	ICD10CM	1	J11.1		Influenza due to unidentified influenza virus with other	IMO	GHS-PACDC	DX_LEGACY	ACTIVE	N	1900-01-01	9999-12-31	2015-07-10
Diagnosis: ICD10 Code, J00-J99 Define	J00-J99	Diseases of the respiratory system	351609	Influenza due to unidentified influenza virus with gastrointestinal manifestation	ICD10CM	1	J11.2		Influenza due to unidentified influenza virus with gastro	IMO	GHS-PACDC	DX_LEGACY	ACTIVE	N	1900-01-01	9999-12-31	2015-07-10
Diagnosis: ICD10 Code, J00-J99 Define	J00-J99	Diseases of the respiratory system	110154	Influenza with gastrointestinal tract involvement	ICD10CM	1	J11.2		Influenza due to unidentified influenza virus with gastro	IMO	GHS-PACDC	DX_LEGACY	ACTIVE	N	1900-01-01	9999-12-31	2015-07-10
Diagnosis: ICD10 Code, J00-J99 Define	J00-J99	Diseases of the respiratory system	1183349	Influenza due to unidentified influenza virus with other manifestations	ICD10CM	1	J11.89		Influenza due to unidentified influenza virus with other	IMO	GHS-PACDC	IMO_2016_R	ACTIVE	N	1900-01-01	9999-12-31	2016-06-02
Diagnosis: ICD10 Code, J00-J99 Define	J00-J99	Diseases of the respiratory system	248783	Cardiomyopathy due to influenza (HCC)	ICD10CM	1	J11.89	J11.89,I43	Influenza due to unidentified influenza virus with other	IMO	GHS-PACDC	DX_LEGACY	ACTIVE	N	1900-01-01	9999-12-31	2015-07-10
Diagnosis: ICD10 Code, J00-J99 Define	J00-J99	Diseases of the respiratory system	615769	Viral pneumonia, not elsewhere classified	ICD10CM	1	J12		Viral pneumonia, not elsewhere classified	IMO	GHS-PACDC	IMO_2015_R	ACTIVE	Y	1900-01-01	9999-12-31	2015-07-10
Diagnosis: ICD10 Code, J00-J99 Define	J00-J99	Diseases of the respiratory system	426494	Pneumonia due to respiratory syncytial virus (RSV)	ICD10CM	1	J12.1		Respiratory syncytial virus pneumonia	IMO	GHS-PACDC	IMO_2015_R	ACTIVE	N	1900-01-01	9999-12-31	2015-07-10
Diagnosis: ICD10 Code, J00-J99 Define	J00-J99	Diseases of the respiratory system	137158	SARS virus pneumonia	ICD10CM	1	J12.81		Pneumonia due to SARS-associated coronavirus	IMO	GHS-PACDC	DX_LEGACY	ACTIVE	N	1900-01-01	9999-12-31	2015-07-10
Diagnosis: ICD10 Code, J00-J99 Define	J00-J99	Diseases of the respiratory system	137159	Severe acute respiratory syndrome	ICD10CM	1	J12.81		Pneumonia due to SARS-associated coronavirus	IMO	GHS-PACDC	DX_LEGACY	ACTIVE	N	1900-01-01	2017-10-14	2015-07-10
Diagnosis: ICD10 Code, J00-J99 Define	J00-J99	Diseases of the respiratory system	1114593	Bronchopneumonia due to virus	ICD10CM	1	J12.9		Viral pneumonia, unspecified	IMO	GHS-PACDC	IMO_2016_R	ACTIVE	N	1900-01-01	9999-12-31	2016-06-02
Diagnosis: ICD10 Code, J00-J99 Define	J00-J99	Diseases of the respiratory system	54499	Viral pneumonitis	ICD10CM	1	J12.9		Viral pneumonia, unspecified	IMO	GHS-PACDC	DX_LEGACY	ACTIVE	N	1900-01-01	9999-12-31	2015-07-10
Diagnosis: ICD10 Code, J00-J99 Define	J00-J99	Diseases of the respiratory system	1271114	Community acquired pneumonia due to Streptococcus pneumoniae (HCC)	ICD10CM	1	J13		Pneumonia due to Streptococcus pneumoniae	IMO	GHS-PACDC	IMO_2017_R	ACTIVE	N	1900-01-01	9999-12-31	2016-09-30
Diagnosis: ICD10 Code, J00-J99 Define	J00-J99	Diseases of the respiratory system	1107914	Pneumonia of right lung due to Haemophilus influenzae (HCC)	ICD10CM	1	J14		Pneumonia due to Hemophilus influenzae	IMO	GHS-PACDC	IMO_2016_R	ACTIVE	N	1900-01-01	9999-12-31	2016-06-02
Diagnosis: ICD10 Code, J00-J99 Define	J00-J99	Diseases of the respiratory system	1107941	Pneumonia of left upper lobe due to Haemophilus influenzae (HCC)	ICD10CM	1	J14		Pneumonia due to Hemophilus influenzae	IMO	GHS-PACDC	IMO_2016_R	ACTIVE	N	1900-01-01	9999-12-31	2016-06-02
Diagnosis: ICD10 Code, J00-J99 Define	J00-J99	Diseases of the respiratory system	1237407	Pneumonia due to Klebsiella pneumoniae, unspecified laterality, unspecified part of lung (HCC)	ICD10CM	1	J15.0		Pneumonia due to Klebsiella pneumoniae	IMO	GHS-PACDC	IMO_2016_R	ACTIVE	N	1900-01-01	9999-12-31	2016-06-02
Diagnosis: ICD10 Code, J00-J99 Define	J00-J99	Diseases of the respiratory system	1271110	Community acquired pneumonia due to Klebsiella pneumoniae (HCC)	ICD10CM	1	J15.0		Pneumonia due to Klebsiella pneumoniae	IMO	GHS-PACDC	IMO_2017_R	ACTIVE	N	1900-01-01	9999-12-31	2016-09-30
Diagnosis: ICD10 Code, J00-J99 Define	J00-J99	Diseases of the respiratory system	1238798	Pneumonia of lower lobe due to Klebsiella pneumoniae, unspecified laterality (HCC)	ICD10CM	1	J15.0		Pneumonia due to Klebsiella pneumoniae	IMO	GHS-PACDC	IMO_2016_R	ACTIVE	N	1900-01-01	9999-12-31	2016-06-02
Diagnosis: ICD10 Code, J00-J99 Define	J00-J99	Diseases of the respiratory system	1107892	Pneumonia of left upper lobe due to Pseudomonas species (HCC)	ICD10CM	1	J15.1		Pneumonia due to Pseudomonas	IMO	GHS-PACDC	IMO_2016_R	ACTIVE	N	1900-01-01	9999-12-31	2016-06-02
Diagnosis: ICD10 Code, J00-J99 Define	J00-J99	Diseases of the respiratory system	36587	Pneumonia due to Pseudomonas (HCC)	ICD10CM	1	J15.1		Pneumonia due to Pseudomonas	IMO	GHS-PACDC	DX_LEGACY	ACTIVE	N	1900-01-01	9999-12-31	2015-07-10
Diagnosis: ICD10 Code, J00-J99 Define	J00-J99	Diseases of the respiratory system	387939	Staphylococcal pneumonia, left (HCC)	ICD10CM	1	J15.211		Pneumonia due to Methicillin susceptible Staphylococcu	IMO	GHS-PACDC	DX_LEGACY	ACTIVE	N	1900-01-01	9999-12-31	2015-07-10
Diagnosis: ICD10 Code, J00-J99 Define	J00-J99	Diseases of the respiratory system	1271111	Community acquired pneumonia due to methicillin resistant Staphylococcus aureus (MRSA) (HC	ICD10CM	1	J15.212		Pneumonia due to Methicillin resistant Staphylococcus a	IMO	GHS-PACDC	IMO_2017_R	ACTIVE	N	1900-01-01	9999-12-31	2016-09-30
Diagnosis: ICD10 Code, J00-J99 Define	J00-J99	Diseases of the respiratory system	17693	MRSA pneumonia (HCC)	ICD10CM	1	J15.212		Pneumonia due to Methicillin resistant Staphylococcus a	IMO	GHS-PACDC	DX_LEGACY	ACTIVE	N	1900-01-01	9999-12-31	2015-07-10
Diagnosis: ICD10 Code, J00-J99 Define	J00-J99	Diseases of the respiratory system	1237806	Pneumonia due to methicillin resistant Staphylococcus aureus, unspecified laterality, unspecifie	ICD10CM	1	J15.212		Pneumonia due to Methicillin resistant Staphylococcus a	IMO	GHS-PACDC	IMO_2016_R	ACTIVE	N	1900-01-01	9999-12-31	2016-06-02
Diagnosis: ICD10 Code, J00-J99 Define	J00-J99	Diseases of the respiratory system	1107996	Pneumonia of both lungs due to group B Streptococcus (HCC)	ICD10CM	1	J15.3		Pneumonia due to streptococcus, group B	IMO	GHS-PACDC	IMO_2016_R	ACTIVE	N	1900-01-01	9999-12-31	2016-06-02
Diagnosis: ICD10 Code, J00-J99 Define	J00-J99	Diseases of the respiratory system	1137912	Pneumonia due to other streptococci (HCC)	ICD10CM	1	J15.4		Pneumonia due to other streptococci	IMO	GHS-PACDC	IMO_2016_R	ACTIVE	N	1900-01-01	9999-12-31	2016-06-02
Diagnosis: ICD10 Code, J00-J99 Define	J00-J99	Diseases of the respiratory system	1289611	Pneumonia of upper lobe due to Escherichia coli, unspecified laterality (HCC)	ICD10CM	1	J15.5		Pneumonia due to Escherichia coli	IMO	GHS-PACDC	IMO_2017_R	ACTIVE	N	1900-01-01	9999-12-31	2016-09-30
Diagnosis: ICD10 Code, J00-J99 Define	J00-J99	Diseases of the respiratory system	1132400	Bilateral lower lobe pneumonia due to Escherichia coli (HCC)	ICD10CM	1	J15.5		Pneumonia due to Escherichia coli	IMO	GHS-PACDC	IMO_2016_R	ACTIVE	N	1900-01-01	9999-12-31	2016-06-02
Diagnosis: ICD10 Code, J00-J99 Define	J00-J99	Diseases of the respiratory system	1289584	Pneumonia due to Escherichia coli, unspecified laterality, unspecified part of lung (HCC)	ICD10CM	1	J15.5		Pneumonia due to Escherichia coli	IMO	GHS-PACDC	IMO_2017_R	ACTIVE	N	1900-01-01	9999-12-31	2016-09-30
Diagnosis: ICD10 Code, J00-J99 Define	J00-J99	Diseases of the respiratory system	1289614	Pneumonia of both lungs due to other aerobic Gram-negative bacteria, unspecified part of lung	ICD10CM	1	J15.6		Pneumonia due to other Gram-negative bacteria	IMO	GHS-PACDC	IMO_2017_R	ACTIVE	N	1900-01-01	9999-12-31	2016-09-30
Diagnosis: ICD10 Code, J00-J99 Define	J00-J99	Diseases of the respiratory system	91088	Pneumonia due to Gram-negative bacteria (HCC)	ICD10CM	1	J15.6		Pneumonia due to other Gram-negative bacteria	IMO	GHS-PACDC	DX_LEGACY	ACTIVE	N	2016-04-10	9999-12-31	2015-07-10
Diagnosis: ICD10 Code, J00-J99 Define	J00-J99	Diseases of the respiratory system	1283003	Pneumonia of right lower lobe due to other aerobic Gram-negative bacteria (HCC)	ICD10CM	1	J15.6		Pneumonia due to other Gram-negative bacteria	IMO	GHS-PACDC	IMO_2017_R	ACTIVE	N	1900-01-01	9999-12-31	2016-09-30
Diagnosis: ICD10 Code, J00-J99 Define	J00-J99	Diseases of the respiratory system	5442	Pneumonia due to other Gram-negative bacteria	ICD10CM	1	J15.6		Pneumonia due to other Gram-negative bacteria	IMO	GHS-PACDC	DX_LEGACY	ACTIVE	Y	2017-09-17	9999-12-31	2017-10-30

Diagnosis: ICD10 Code, J00-J99 Define	J00-J99	Diseases of the respiratory system	1114075	Bronchopneumonia due to Mycoplasma pneumoniae	ICD10CM	1	J15.7		Pneumonia due to Mycoplasma pneumoniae	IMO	GHS-PACDC	IMO_2016_R	ACTIVE	N	1900-01-01	9999-12-31	2016-06-02
Diagnosis: ICD10 Code, J00-J99 Define	J00-J99	Diseases of the respiratory system	380513	Actinomycotic pneumonia (HCC)	ICD10CM	1	J15.8		Pneumonia due to other specified bacteria	IMO	GHS-PACDC	DX_LEGACY	ACTIVE	N	1900-01-01	2017-10-14	2015-07-10
Diagnosis: ICD10 Code, J00-J99 Define	J00-J99	Diseases of the respiratory system	535586	Pneumonia due to other specified bacteria	ICD10CM	1	J15.8		Pneumonia due to other specified bacteria	IMO	GHS-PACDC	IMO_2015_R	ACTIVE	Y	1900-01-01	9999-12-31	2015-07-10
Diagnosis: ICD10 Code, J00-J99 Define	J00-J99	Diseases of the respiratory system	1114305	Bronchopneumonia due to bacteria	ICD10CM	1	J15.9		Unspecified bacterial pneumonia	IMO	GHS-PACDC	IMO_2016_R	ACTIVE	N	1900-01-01	9999-12-31	2016-06-02
Diagnosis: ICD10 Code, J00-J99 Define	J00-J99	Diseases of the respiratory system	167738	Community acquired bacterial pneumonia	ICD10CM	1	J15.9		Unspecified bacterial pneumonia	IMO	GHS-PACDC	DX_LEGACY	ACTIVE	N	1900-01-01	9999-12-31	2015-07-10
Diagnosis: ICD10 Code, J00-J99 Define	J00-J99	Diseases of the respiratory system	385731	Acquired immunodeficiency syndrome (AIDS) with bacterial pneumonia (HCC)	ICD10CM	2	J15.9	B20,J15.9	Unspecified bacterial pneumonia	IMO	GHS-PACDC	DX_LEGACY	ACTIVE	N	1900-01-01	2017-10-14	2015-07-10
Diagnosis: ICD10 Code, J00-J99 Define	J00-J99	Diseases of the respiratory system	1239312	Pneumonia of upper lobe due to Chlamydia species, unspecified laterality	ICD10CM	1	J16.0		Chlamydial pneumonia	IMO	GHS-PACDC	IMO_2016_R	ACTIVE	N	1900-01-01	9999-12-31	2016-06-02
Diagnosis: ICD10 Code, J00-J99 Define	J00-J99	Diseases of the respiratory system	1347604	Pneumonia due to other specified organism	ICD10CM	1	J16.8		Pneumonia due to other specified infectious organisms	IMO	GHS-PACDC	IMO_2018_R	DELETED	Y	1900-01-01	9999-12-31	2017-10-30
Diagnosis: ICD10 Code, J00-J99 Define	J00-J99	Diseases of the respiratory system	55231	Bronchopneumonia, hiberno-vernal	ICD10CM	2	J17	A78,J17	Pneumonia in diseases classified elsewhere	IMO	GHS-PACDC	DX_LEGACY	ACTIVE	N	2016-04-10	9999-12-31	2015-07-10
Diagnosis: ICD10 Code, J00-J99 Define	J00-J99	Diseases of the respiratory system	147568	Pneumonia in infectious disease	ICD10CM	2	J17	B99.9,J17	Pneumonia in diseases classified elsewhere	IMO	GHS-PACDC	DX_LEGACY	ACTIVE	N	2016-04-10	2016-10-14	2015-07-10
Diagnosis: ICD10 Code, J00-J99 Define	J00-J99	Diseases of the respiratory system	1104512	Pneumonia due to Schistosoma haematobium	ICD10CM	2	J17	B65.0,J17	Pneumonia in diseases classified elsewhere	IMO	GHS-PACDC	IMO_2016_R	DELETED	Y	1900-01-01	9999-12-31	2016-06-02
Diagnosis: ICD10 Code, J00-J99 Define	J00-J99	Diseases of the respiratory system	1347755	Pneumonia in other systemic mycoses	ICD10CM	2	J17	B49,J17	Pneumonia in diseases classified elsewhere	IMO	GHS-PACDC	IMO_2018_R	DELETED	Y	1900-01-01	9999-12-31	2017-10-30
Diagnosis: ICD10 Code, J00-J99 Define	J00-J99	Diseases of the respiratory system	496635	Pneumonia of lower lobe of lung (HCC)	ICD10CM	1	J18.1		Lobar pneumonia, unspecified organism	IMO	GHS-PACDC	IMO_2015_R	ACTIVE	N	2017-03-31	9999-12-31	2017-04-14
Diagnosis: ICD10 Code, J00-J99 Define	J00-J99	Diseases of the respiratory system	1107993	Pneumonia of right lower lobe due to infectious organism (HCC)	ICD10CM	1	J18.1		Lobar pneumonia, unspecified organism	IMO	GHS-PACDC	IMO_2016_R	ACTIVE	N	2017-03-31	9999-12-31	2017-04-14
Diagnosis: ICD10 Code, J00-J99 Define	J00-J99	Diseases of the respiratory system	1355716	Community acquired pneumonia of right upper lobe of lung (HCC)	ICD10CM	1	J18.1		Lobar pneumonia, unspecified organism	IMO	GHS-PACDC	IMO_2018_R	ACTIVE	N	1900-01-01	9999-12-31	2017-10-30
Diagnosis: ICD10 Code, J00-J99 Define	J00-J99	Diseases of the respiratory system	21357	Passive pneumonia	ICD10CM	1	J18.2		Hypostatic pneumonia, unspecified organism	IMO	GHS-PACDC	DX_LEGACY	ACTIVE	N	1900-01-01	9999-12-31	2015-07-10
Diagnosis: ICD10 Code, J00-J99 Define	J00-J99	Diseases of the respiratory system	1107895	Pneumonia of right upper lobe due to infectious organism (HCC)	ICD10CM	1	J18.9		Pneumonia, unspecified organism	IMO	GHS-PACDC	IMO_2016_R	ACTIVE	N	1900-01-01	2017-04-27	2016-06-02
Diagnosis: ICD10 Code, J00-J99 Define	J00-J99	Diseases of the respiratory system	326616	Obstructive pneumonia	ICD10CM	1	J18.9		Pneumonia, unspecified organism	IMO	GHS-PACDC	DX_LEGACY	ACTIVE	N	1900-01-01	9999-12-31	2015-07-10
Diagnosis: ICD10 Code, J00-J99 Define	J00-J99	Diseases of the respiratory system	223655	Pneumonia due to infectious agent	ICD10CM	1	J18.9		Pneumonia, unspecified organism	IMO	GHS-PACDC	DX_LEGACY	ACTIVE	N	2016-09-17	9999-12-31	2016-09-30
Diagnosis: ICD10 Code, J00-J99 Define	J00-J99	Diseases of the respiratory system	385031	Acute ulcerative gastroenteritis complicating pneumonia (HCC)	ICD10CM	2	J18.9	K51.90,J18.9	Pneumonia, unspecified organism	IMO	GHS-PACDC	DX_LEGACY	ACTIVE	N	1900-01-01	9999-12-31	2015-07-10
Diagnosis: ICD10 Code, J00-J99 Define	J00-J99	Diseases of the respiratory system	1427810	Community acquired pneumonia of left lung, unspecified part of lung	ICD10CM	1	J18.9		Pneumonia, unspecified organism	IMO	GHS-PACDC	IMO_2018_R	ACTIVE	N	1900-01-01	9999-12-31	2017-10-30
Diagnosis: ICD10 Code, J00-J99 Define	J00-J99	Diseases of the respiratory system	493376	Pneumonia with cavity of lung	ICD10CM	1	J18.9	J18.9,J98.4	Pneumonia, unspecified organism	IMO	GHS-PACDC	IMO_2015_R	ACTIVE	N	1900-01-01	9999-12-31	2015-07-10
Diagnosis: ICD10 Code, J00-J99 Define	J00-J99	Diseases of the respiratory system	380516	Basal pneumonia	ICD10CM	1	J18.9		Pneumonia, unspecified organism	IMO	GHS-PACDC	DX_LEGACY	ACTIVE	N	1900-01-01	9999-12-31	2015-07-10
Diagnosis: ICD10 Code, J00-J99 Define	J00-J99	Diseases of the respiratory system	190353	Postop pneumonia	ICD10CM	2	J18.9	J95.88,J18.9	Pneumonia, unspecified organism	IMO	GHS-PACDC	DX_LEGACY	ACTIVE	N	1900-01-01	2016-05-07	2015-07-10
Diagnosis: ICD10 Code, J00-J99 Define	J00-J99	Diseases of the respiratory system	1239578	Pneumonia of lower lobe due to infectious organism, unspecified laterality (HCC)	ICD10CM	1	J18.9		Pneumonia, unspecified organism	IMO	GHS-PACDC	IMO_2016_R	ACTIVE	N	1900-01-01	2017-04-27	2016-06-02
Diagnosis: ICD10 Code, J00-J99 Define	J00-J99	Diseases of the respiratory system	1107925	Pneumonia of left lower lobe due to infectious organism (HCC)	ICD10CM	1	J18.9		Pneumonia, unspecified organism	IMO	GHS-PACDC	IMO_2016_R	ACTIVE	N	1900-01-01	2017-04-27	2016-06-02
Diagnosis: ICD10 Code, J00-J99 Define	J00-J99	Diseases of the respiratory system	202870	Right upper lobe pneumonia (HCC)	ICD10CM	1	J18.9		Pneumonia, unspecified organism	IMO	GHS-PACDC	DX_LEGACY	ACTIVE	N	1900-01-01	2017-04-27	2015-07-10
Diagnosis: ICD10 Code, J00-J99 Define	J00-J99	Diseases of the respiratory system	182833	CAP (community acquired pneumonia)	ICD10CM	1	J18.9		Pneumonia, unspecified organism	IMO	GHS-PACDC	DX_LEGACY	ACTIVE	N	1900-01-01	9999-12-31	2015-07-10
Diagnosis: ICD10 Code, J00-J99 Define	J00-J99	Diseases of the respiratory system	1039644	Pneumonia involving right lung, unspecified part of lung	ICD10CM	1	J18.9		Pneumonia, unspecified organism	IMO	GHS-PACDC	IMO_2015_R	DELETED	Y	1900-01-01	9999-12-31	2015-07-10
Diagnosis: ICD10 Code, J20 Define	J00-J99	Diseases of the respiratory system	246489	Acute bronchitis, viral	ICD10CM	1	J20.8		Acute bronchitis due to other specified organisms	IMO	GHS-PACDC	DX_LEGACY	ACTIVE	N	1900-01-01	9999-12-31	2015-07-10
Diagnosis: ICD10 Code, J00-J99 Define	J00-J99	Diseases of the respiratory system	246489	Acute bronchitis, viral	ICD10CM	1	J20.8		Acute bronchitis due to other specified organisms	IMO	GHS-PACDC	DX_LEGACY	ACTIVE	N	1900-01-01	9999-12-31	2015-07-10
Diagnosis: ICD10 Code, J20 Define	J00-J99	Diseases of the respiratory system	237147	Bronchitis, acute, with bronchospasm	ICD10CM	1	J20.9		Acute bronchitis, unspecified	IMO	GHS-PACDC	DX_LEGACY	ACTIVE	N	1900-01-01	9999-12-31	2015-07-10
Diagnosis: ICD10 Code, J20 Define	J00-J99	Diseases of the respiratory system	242921	Acute bronchitis with asthma	ICD10CM	1	J20.9	J20.9,J45.909	Acute bronchitis, unspecified	IMO	GHS-PACDC	DX_LEGACY	ACTIVE	N	1900-01-01	9999-12-31	2015-07-10
Diagnosis: ICD10 Code, J00-J99 Define	J00-J99	Diseases of the respiratory system	237147	Bronchitis, acute, with bronchospasm	ICD10CM	1	J20.9		Acute bronchitis, unspecified	IMO	GHS-PACDC	DX_LEGACY	ACTIVE	N	1900-01-01	9999-12-31	2015-07-10
Diagnosis: ICD10 Code, J00-J99 Define	J00-J99	Diseases of the respiratory system	242921	Acute bronchitis with asthma	ICD10CM	1	J20.9	J20.9,J45.909	Acute bronchitis, unspecified	IMO	GHS-PACDC	DX_LEGACY	ACTIVE	N	1900-01-01	9999-12-31	2015-07-10
Diagnosis: ICD10 Code, J00-J99 Define	J00-J99	Diseases of the respiratory system	269821	Acute bronchiolitis due to human metapneumovirus	ICD10CM	1	J21.1		Acute bronchiolitis due to human metapneumovirus	IMO	GHS-PACDC	DX_LEGACY	ACTIVE	N	1900-01-01	9999-12-31	2015-07-10
Diagnosis: ICD10 Code, J00-J99 Define	J00-J99	Diseases of the respiratory system	21021	Capillary pneumonia	ICD10CM	1	J21.9		Acute bronchiolitis, unspecified	IMO	GHS-PACDC	DX_LEGACY	ACTIVE	N	1900-01-01	9999-12-31	2015-07-10
Diagnosis: ICD10 Code, J00-J99 Define	J00-J99	Diseases of the respiratory system	21028	Subacute obliterative bronchiolitis	ICD10CM	1	J21.9		Acute bronchiolitis, unspecified	IMO	GHS-PACDC	DX_LEGACY	ACTIVE	N	1900-01-01	9999-12-31	2015-07-10
Diagnosis: ICD10 Code, J00-J99 Define	J00-J99	Diseases of the respiratory system	36698	Adenoviral bronchiolitis	ICD10CM	1	J21.9		Acute bronchiolitis, unspecified	IMO	GHS-PACDC	DX_LEGACY	ACTIVE	N	1900-01-01	2016-05-07	2015-07-10
Diagnosis: ICD10 Code, J00-J99 Define	J00-J99	Diseases of the respiratory system	116137	VMR (vasomotor rhinitis)	ICD10CM	1	J30.0		Vasomotor rhinitis	IMO	GHS-PACDC	DX_LEGACY	ACTIVE	N	1900-01-01	9999-12-31	2015-07-10
Diagnosis: ICD10 Code, J00-J99 Define	J00-J99	Diseases of the respiratory system	1320342	Acute allergic rhinitis due to pollen	ICD10CM	1	J30.1		Allergic rhinitis due to pollen	IMO	GHS-PACDC	IMO_2017_R	ACTIVE	N	1900-01-01	9999-12-31	2017-04-14
Diagnosis: ICD10 Code, J00-J99 Define	J00-J99	Diseases of the respiratory system	1273961	Allergy to wall pellitory pollen	ICD10CM	1	J30.1		Allergic rhinitis due to pollen	IMO	GHS-PACDC	IMO_2017_R	ACTIVE	N	1900-01-01	9999-12-31	2016-09-30
Diagnosis: ICD10 Code, J00-J99 Define	J00-J99	Diseases of the respiratory system	1132243	Non-seasonal allergic rhinitis due to food	ICD10CM	1	J30.5		Allergic rhinitis due to food	IMO	GHS-PACDC	IMO_2016_R	ACTIVE	N	2016-09-17	9999-12-31	2016-09-30
Diagnosis: ICD10 Code, J00-J99 Define	J00-J99	Diseases of the respiratory system	1272471	Non-seasonal allergic rhinitis due to animal hair and dander	ICD10CM	1	J30.81		Allergic rhinitis due to animal (cat) (dog) hair and dande	IMO	GHS-PACDC	IMO_2017_R	ACTIVE	N	1900-01-01	9999-12-31	2016-09-30
Diagnosis: ICD10 Code, J00-J99 Define	J00-J99	Diseases of the respiratory system	1320371	Chronic allergic rhinitis due to animal hair and dander	ICD10CM	1	J30.81		Allergic rhinitis due to animal (cat) (dog) hair and dande	IMO	GHS-PACDC	IMO_2017_R	ACTIVE	N	1900-01-01	9999-12-31	2017-04-14
Diagnosis: ICD10 Code, J00-J99 Define	J00-J99	Diseases of the respiratory system	1320299	Acute allergic rhinitis due to animal hair and dander	ICD10CM	1	J30.81		Allergic rhinitis due to animal (cat) (dog) hair and dande	IMO	GHS-PACDC	IMO_2017_R	ACTIVE	N	1900-01-01	9999-12-31	2017-04-14
Diagnosis: ICD10 Code, J00-J99 Define	J00-J99	Diseases of the respiratory system	141272	Allergy to animal dander	ICD10CM	1	J30.81		Allergic rhinitis due to animal (cat) (dog) hair and dande	IMO	GHS-PACDC	DX_LEGACY	ACTIVE	N	1900-01-01	9999-12-31	2015-07-10
Diagnosis: ICD10 Code, J00-J99 Define	J00-J99	Diseases of the respiratory system	417073	Allergy to animals	ICD10CM	1	J30.81		Allergic rhinitis due to animal (cat) (dog) hair and dande	IMO	GHS-PACDC	DX_LEGACY	ACTIVE	N	1900-01-01	9999-12-31	2015-07-10
Diagnosis: ICD10 Code, J00-J99 Define	J00-J99	Diseases of the respiratory system	526703	Allergic rhinitis due to dust mite	ICD10CM	1	J30.89		Other allergic rhinitis	IMO	GHS-PACDC	IMO_2015_R	ACTIVE	N	1900-01-01	9999-12-31	2015-07-10
Diagnosis: ICD10 Code, J00-J99 Define	J00-J99	Diseases of the respiratory system	331723	Smoke hypersensitivity	ICD10CM	1	J30.89		Other allergic rhinitis	IMO	GHS-PACDC	DX_LEGACY	ACTIVE	N	1900-01-01	9999-12-31	2015-07-10
Diagnosis: ICD10 Code, J00-J99 Define	J00-J99	Diseases of the respiratory system	38050	Severe chronic rhinitis	ICD10CM	1	J31.0		Chronic rhinitis	IMO	GHS-PACDC	DX_LEGACY	ACTIVE	N	1900-01-01	9999-12-31	2015-07-10
Diagnosis: ICD10 Code, J00-J99 Define	J00-J99	Diseases of the respiratory system	38049	Ozaena	ICD10CM	1	J31.0		Chronic rhinitis	IMO	GHS-PACDC	DX_LEGACY	ACTIVE	N	1900-01-01	9999-12-31	2015-07-10
Diagnosis: ICD10 Code, J00-J99 Define	J00-J99	Diseases of the respiratory system	105510	Chronic atrophic rhinitis	ICD10CM	1	J31.0		Chronic rhinitis	IMO	GHS-PACDC	DX_LEGACY	ACTIVE	N	1900-01-01	9999-12-31	2015-07-10
Diagnosis: ICD10 Code, J00-J99 Define	J00-J99	Diseases of the respiratory system	252549	Chronic hypertrophic rhinitis	ICD10CM	1	J31.0		Chronic rhinitis	IMO	GHS-PACDC	DX_LEGACY	ACTIVE	N	1900-01-01	9999-12-31	2015-07-10
Diagnosis: ICD10 Code, J00-J99 Define	J00-J99	Diseases of the respiratory system	55739	Rhinitis, atrophic	ICD10CM	1	J31.0		Chronic rhinitis	IMO	GHS-PACDC	DX_LEGACY	ACTIVE	N	1900-01-01	9999-12-31	2015-07-10
Diagnosis: ICD10 Code, J00-J99 Define	J00-J99	Diseases of the respiratory system	243234	Rhinitis, purulent, chronic	ICD10CM	1	J31.0		Chronic rhinitis	IMO	GHS-PACDC	DX_LEGACY	ACTIVE	N	1900-01-01	9999-12-31	2015-07-10
Diagnosis: ICD10 Code, J00-J99 Define	J00-J99	Diseases of the respiratory system	5369	Chronic nasopharyngitis	ICD10CM	1	J31.1		Chronic nasopharyngitis	IMO	GHS-PACDC	DX_LEGACY	ACTIVE	N	1900-01-01	9999-12-31	2015-07-10
Diagnosis: ICD10 Code, J00-J99 Define	J00-J99	Diseases of the respiratory system	20941	Hypertrophic pharyngitis	ICD10CM	1	J31.2		Chronic pharyngitis	IMO	GHS-PACDC	DX_LEGACY	ACTIVE	N	1900-01-01	9999-12-31	2015-07-10
Diagnosis: ICD10 Code, J00-J99 Define	J00-J99	Diseases of the respiratory system	226902	Chronic pharyngitis and nasopharyngitis	ICD10CM	2	J31.2	J31.1,J31.2	Chronic pharyngitis	IMO	GHS-PACDC	DX_LEGACY	ACTIVE	N	1900-01-01	9999-12-31	2015-07-10
Diagnosis: ICD10 Code, J00-J99 Define	J00-J99	Diseases of the respiratory system	5372	Chronic frontal sinusitis	ICD10CM	1	J32.1		Chronic frontal sinusitis	IMO	GHS-PACDC	DX_LEGACY	ACTIVE	N	1900-01-01	9999-12-31	2015-07-10
Diagnosis: ICD10 Code, J00-J99 Define	J00-J99	Diseases of the respiratory system	56445	Sphenoidal sinusitis	ICD10CM	1	J32.3		Chronic sphenoidal sinusitis	IMO	GHS-PACDC	DX_LEGACY	ACTIVE	N	1900-01-01	9999-12-31	2015-07-10
Diagnosis: ICD10 Code, J00-J99 Define	J00-J99	Diseases of the respiratory system	533612	Chronic sphenoidal sinusitis	ICD10CM	1	J32.3		Chronic sphenoidal sinusitis	IMO	GHS-PACDC	IMO_2015_R	ACTIVE	Y	1900-01-01	9999-12-31	2015-07-10
Diagnosis: ICD10 Code, J00-J99 Define	J00-J99	Diseases of the respiratory system	1097679	Sinusitis, unspecified chronicity, unspecified location	ICD10CM	1	J32.9		Chronic sinusitis, unspecified	IMO	GHS-PACDC	IMO_2015_R	ACTIVE	N	1900-01-01	9999-12-31	2015-07-10
Diagnosis: ICD10 Code, J00-J99 Define	J00-J99	Diseases of the respiratory system	182108	Sinusitis due to Moraxella catarrhalis	ICD10CM	1	J32.9	J32.9,B96.89	Chronic sinusitis, unspecified	IMO	GHS-PACDC	DX_LEGACY	ACTIVE	N	2016-09-17	9999-12-31	2016-09-30
Diagnosis: ICD10 Code, J00-J99 Define	J00-J99	Diseases of the respiratory system	52964	Polyp, nasal, cavity	ICD10CM	1	J33.0		Polyp of nasal cavity	IMO	GHS-PACDC	DX_LEGACY	ACTIVE	N	1900-01-01	9999-12-31	2015-07-10
Diagnosis: ICD10 Code, J00-J99 Define	J00-J99	Diseases of the respiratory system	231423	Nasal polyp, benign	ICD10CM	1	J33.0		Polyp of nasal cavity	IMO	GHS-PACDC	DX_LEGACY	ACTIVE	N	1900-01-01	9999-12-31	2015-07-10
Diagnosis: ICD10 Code, J00-J99 Define	J00-J99	Diseases of the respiratory system	20878	Woakes' syndrome	ICD10CM	1	J33.1		Polypoid sinus degeneration	IMO	GHS-PACDC	DX_LEGACY	ACTIVE	N	1900-01-01	9999-12-31	2015-07-10
Diagnosis: ICD10 Code, J00-J99 Define	J00-J99	Diseases of the respiratory system	20900	Polyp of ethmoidal sinus	ICD10CM	1	J33.8		Other polyp of sinus	IMO	GHS-PACDC	DX_LEGACY	ACTIVE	N	1900-01-01	9999-12-31	2015-07-10
Diagnosis: ICD10 Code, J00-J99 Define	J00-J99	Diseases of the respiratory system	73551	Sphenoidal polyp of sinus	ICD10CM	1	J33.8		Other polyp of sinus	IMO	GHS-PACDC	DX_LEGACY	ACTIVE	N	1900-01-01	9999-12-31	2015-07-10
Diagnosis: ICD10 Code, J00-J99 Define	J00-J99	Diseases of the respiratory system	113337	Polyp, sinus sphenoidal	ICD10CM	1	J33.8		Other polyp of sinus	IMO	GHS-PACDC	DX_LEGACY	ACTIVE	N	1900-01-01	9999-12-31	2015-07-10
Diagnosis: ICD10 Code, J00-J99 Define	J00-J99	Diseases of the respiratory system	136625	Triad asthma	ICD10CM	2	J33.9	J45.998,J33.9	Nasal polyp, unspecified	IMO	GHS-PACDC	DX_LEGACY	ACTIVE	N	1900-01-01	2016-05-07	2015-07-10
Diagnosis: ICD10 Code, J00-J99 Define	J00-J99	Diseases of the respiratory system	151926	Ulcer of nasal septum	ICD10CM	1	J34.0		Abscess, furuncle and carbuncle of nose	IMO	GHS-PACDC	DX_LEGACY	ACTIVE	N	2016-04-10	9999-12-31	2015-07-10
Diagnosis: ICD10 Code, J00-J99 Define	J00-J99	Diseases of the respiratory system	372697	Cellulitis of nasal tip	ICD10CM	1	J34.0		Abscess, furuncle and carbuncle of nose	IMO	GHS-PACDC	DX_LEGACY	ACTIVE	N	2016-04-10	9999-12-31	2015-07-10
Diagnosis: ICD10 Code, J00-J99 Define	J00-J99	Diseases of the respiratory system	252477	Cellulitis of nostril	ICD10CM	1	J34.0		Abscess, furuncle and carbuncle of nose	IMO	GHS-PACDC	DX_LEGACY	ACTIVE	N	2016-04-10	9999-12-31	2015-07-10
Diagnosis: ICD10 Code, J00-J99 Define	J00-J99	Diseases of the respiratory system	209608	Nasal septal abscess	ICD10CM	1	J34.0		Abscess, furuncle and carbuncle of nose	IMO	GHS-PACDC	DX_LEGACY	ACTIVE	N	1900-01-01	9999-12-31	2015-07-10
Diagnosis: ICD10 Code, J00-J99 Define	J00-J99	Diseases of the respiratory system	415846	Intranasal ulcers	ICD10CM	1	J34.0		Abscess, furuncle and carbuncle of nose	IMO	GHS-PACDC	DX_LEGACY	ACTIVE	N	1900-01-01	9999-12-31	2015-07-10
Diagnosis: ICD10 Code, J00-J99 Define	J00-J99	Diseases of the respiratory system	414295	Abscess of nasal cavity	ICD10CM	1	J34.0		Abscess, furuncle and carbuncle of nose	IMO	GHS-PACDC	DX_LEGACY	ACTIVE	N	1900-01-01	9999-12-31	2015-07-10
Diagnosis: ICD10 Code, J00-J99 Define	J00-J99	Diseases of the respiratory system	232264	Sphenoid cyst, left	ICD10CM	1	J34.1		Cyst and mucocele of nose and nasal sinus	IMO	GHS-PACDC	DX_LEGACY	ACTIVE	N	1900-01-01	9999-12-31	2015-07-10
Diagnosis: ICD10 Code, J00-J99 Define	J00-J99	Diseases of the respiratory system	254176	Mucocele of frontal sinus	ICD10CM	1	J34.1		Cyst and mucocele of nose and nasal sinus	IMO	GHS-PACDC	DX_LEGACY	ACTIVE	N	1900-01-01	9999-12-31	2015-07-10
Diagnosis: ICD10 Code, J00-J99 Define	J00-J99	Diseases of the respiratory system	1137905	Cyst and mucocele of nose and nasal sinus	ICD10CM	1	J34.1		Cyst and mucocele of nose and nasal sinus	IMO	GHS-PACDC	IMO_2016_R	DELETED	Y	1900-01-01	9999-12-31	2016-06-02
Diagnosis: ICD10 Code, J00-J99 Define	J00-J99	Diseases of the respiratory system	232267	Cyst of sphenoid sinus	ICD10CM	1	J34.1		Cyst and mucocele of nose and nasal sinus	IMO	GHS-PACDC	DX_LEGACY	ACTIVE	N	1900-01-01	9999-12-31	2015-07-10
Diagnosis: ICD10 Code, J00-J99 Define	J00-J99	Diseases of the respiratory system	501602	Deviated nasal bone	ICD10CM	1	J34.2		Deviated nasal septum	IMO	GHS-PACDC	IMO_2015_R	ACTIVE	N	2016-04-10	9999-12-31	2015-07-10
Diagnosis: ICD10 Code, J00-J99 Define	J00-J99	Diseases of the respiratory system	331132	Nasal septal spur	ICD10CM	1	J34.89		Other specified disorders of nose and nasal sinuses	IMO	GHS-PACDC	DX_LEGACY	ACTIVE	N	1900-01-01	9999-12-31	2015-07-10
Diagnosis: ICD10 Code, J00-J99 Define	J00-J99	Diseases of the respiratory system	151925	Nasal septal ulcer	ICD10CM	1	J34.89		Other specified disorders of nose and nasal sinuses	IMO	GHS-PACDC	DX_LEGACY	ACTIVE	N	1900-01-01	2016-05-07	2015-07-10
Diagnosis: ICD10 Code, J00-J99 Define	J00-J99	Diseases of the respiratory system	328256	Dry nares	ICD10CM	1	J34.89		Other specified disorders of nose and nasal sinuses	IMO	GHS-PACDC	DX_LEGACY	ACTIVE	N	1900-01-01	9999-12-31	2015-07-10
Diagnosis: ICD10 Code, J00-J99 Define	J00-J99	Diseases of the respiratory system	367091	Silent sinus syndrome	ICD10CM	1	J34.89		Other specified disorders of nose and nasal sinuses	IMO	GHS-PACDC	DX_LEGACY	ACTIVE	N	1900-01-01	9999-12-31	2015-07-10
Diagnosis: ICD10 Code, J00-J99 Define	J00-J99	Diseases of the respiratory system	327503	Columella nasi atrophy	ICD10CM	1	J34.89		Other specified disorders of nose and nasal sinuses	IMO	GHS-PACDC	DX_LEGACY	ACTIVE	N	1900-01-01	2016-10-14	2015-07-10
Diagnosis: ICD10 Code, J00-J99 Define	J00-J99	Diseases of the respiratory system	327149	Frontal sinus pain	ICD10CM	1	J34.89		Other specified disorders of nose and nasal sinuses	IMO	GHS-PACDC	DX_LEGACY	ACTIVE	N	1900-01-01	9999-12-31	2015-07-10
Diagnosis: ICD10 Code, J00-J99 Define	J00-J99	Diseases of the respiratory system	416635	Nasal discharge with watery eyes	ICD10CM	1	J34.89	J34.89,H04.209	Other specified disorders of nose and nasal sinuses	IMO	GHS-PACDC	DX_LEGACY	ACTIVE	N	1900-01-01	9999-12-31	2015-07-10
Diagnosis: ICD10 Code, J00-J99 Define	J00-J99	Diseases of the respiratory system	372571	Internal nasal lesion	ICD10CM	1	J34.89		Other specified disorders of nose and nasal sinuses	IMO	GHS-PACDC	DX_LEGACY	ACTIVE	N	1900-01-01	9999-12-31	2015-07-10
Diagnosis: ICD10 Code, J00-J99 Define	J00-J99	Diseases of the respiratory system	332368	Nasal sore	ICD10CM	1	J34.89		Other specified disorders of nose and nasal sinuses	IMO	GHS-PACDC	DX_LEGACY	ACTIVE	N	1900-01-01	9999-12-31	2015-07-10
Diagnosis: ICD10 Code, J00-J99 Define	J00-J99	Diseases of the respiratory system	212274	ENT disease	ICD10CM	2	J34.9	H93.90,J34.9,J39.2	Unspecified disorder of nose and nasal sinuses	IMO	GHS-PACDC	DX_LEGACY	ACTIVE	N	2016-09-17	9999-12-31	2016-09-30
Diagnosis: ICD10 Code, J00-J99 Define	J00-J99	Diseases of the respiratory system	602848	Unspecified disorder of nose and nasal sinuses	ICD10CM	1	J34.9		Unspecified disorder of nose and nasal sinuses	IMO	GHS-PACDC	IMO_2015_R	ACTIVE	Y	1900-01-01	9999-12-31	2015-07-10
Diagnosis: ICD10 Code, J00-J99 Define	J00-J99	Diseases of the respiratory system	246901	Paranasal sinus disease	ICD10CM	1	J34.9		Unspecified disorder of nose and nasal sinuses	IMO	GHS-PACDC	DX_LEGACY	ACTIVE	N	1900-01-01	9999-12-31	2015-07-10
Diagnosis: ICD10 Code, J00-J99 Define	J00-J99	Diseases of the respiratory system	263827	Swollen tonsil	ICD10CM	1	J35.1		Hypertrophy of tonsils	IMO	GHS-PACDC	DX_LEGACY	ACTIVE	N	1900-01-01	9999-12-31	2015-07-10

Diagnosis: ICD10 Code, J00-J99 Define	J00-J99	Diseases of the respiratory system	156559	Enlarged tonsils	ICD10CM	1	J35.1		Hypertrophy of tonsils	IMO	GHS-PACDC	DX_LEGACY	ACTIVE	N	1900-01-01	9999-12-31	2015-07-10
Diagnosis: ICD10 Code, J00-J99 Define	J00-J99	Diseases of the respiratory system	265116	Large tonsils	ICD10CM	1	J35.1		Hypertrophy of tonsils	IMO	GHS-PACDC	DX_LEGACY	ACTIVE	N	1900-01-01	9999-12-31	2015-07-10
Diagnosis: ICD10 Code, J00-J99 Define	J00-J99	Diseases of the respiratory system	62128	Vegetation, adenoid	ICD10CM	1	J35.2		Hypertrophy of adenoids	IMO	GHS-PACDC	DX_LEGACY	ACTIVE	N	1900-01-01	2016-05-07	2015-07-10
Diagnosis: ICD10 Code, J00-J99 Define	J00-J99	Diseases of the respiratory system	534636	Hypertrophy of adenoids	ICD10CM	1	J35.2		Hypertrophy of adenoids	IMO	GHS-PACDC	IMO_2015_R	ACTIVE	Y	1900-01-01	9999-12-31	2015-07-10
Diagnosis: ICD10 Code, J00-J99 Define	J00-J99	Diseases of the respiratory system	102655	Tonsillar and adenoid hypertrophy	ICD10CM	1	J35.3		Hypertrophy of tonsils with hypertrophy of adenoids	IMO	GHS-PACDC	DX_LEGACY	ACTIVE	N	1900-01-01	9999-12-31	2015-07-10
Diagnosis: ICD10 Code, J00-J99 Define	J00-J99	Diseases of the respiratory system	254559	Obstructive tonsils and adenoids	ICD10CM	1	J35.8		Other chronic diseases of tonsils and adenoids	IMO	GHS-PACDC	DX_LEGACY	ACTIVE	N	1900-01-01	9999-12-31	2015-07-10
Diagnosis: ICD10 Code, J00-J99 Define	J00-J99	Diseases of the respiratory system	1428196	Other chronic diseases of tonsils and adenoids (CODE)	ICD10CM	1	J35.8		Other chronic diseases of tonsils and adenoids	IMO	GHS-PACDC	IMO_2018_R	ACTIVE	N	1900-01-01	9999-12-31	2017-10-30
Diagnosis: ICD10 Code, J00-J99 Define	J00-J99	Diseases of the respiratory system	252176	Asymmetry of tonsils	ICD10CM	1	J35.8		Other chronic diseases of tonsils and adenoids	IMO	GHS-PACDC	DX_LEGACY	ACTIVE	N	1900-01-01	9999-12-31	2015-07-10
Diagnosis: ICD10 Code, J00-J99 Define	J00-J99	Diseases of the respiratory system	73557	Ulcer, tonsil	ICD10CM	1	J35.8		Other chronic diseases of tonsils and adenoids	IMO	GHS-PACDC	DX_LEGACY	ACTIVE	N	1900-01-01	9999-12-31	2015-07-10
Diagnosis: ICD10 Code, J00-J99 Define	J00-J99	Diseases of the respiratory system	316529	Tonsil ulcer	ICD10CM	1	J35.8		Other chronic diseases of tonsils and adenoids	IMO	GHS-PACDC	DX_LEGACY	ACTIVE	N	1900-01-01	9999-12-31	2015-07-10
Diagnosis: ICD10 Code, J00-J99 Define	J00-J99	Diseases of the respiratory system	255460	Symptomatic tonsillar crypt	ICD10CM	1	J35.8		Other chronic diseases of tonsils and adenoids	IMO	GHS-PACDC	DX_LEGACY	ACTIVE	N	1900-01-01	9999-12-31	2015-07-10
Diagnosis: ICD10 Code, J00-J99 Define	J00-J99	Diseases of the respiratory system	256858	Cellulitis of tonsil	ICD10CM	1	J36		Peritonsillar abscess	IMO	GHS-PACDC	DX_LEGACY	ACTIVE	N	1900-01-01	9999-12-31	2015-07-10
Diagnosis: ICD10 Code, J00-J99 Define	J00-J99	Diseases of the respiratory system	81587	Abscess, intratonsillar	ICD10CM	1	J36		Peritonsillar abscess	IMO	GHS-PACDC	DX_LEGACY	ACTIVE	N	1900-01-01	9999-12-31	2015-07-10
Diagnosis: ICD10 Code, J00-J99 Define	J00-J99	Diseases of the respiratory system	20971	Paralysis of glottis	ICD10CM	1	J38.00		Paralysis of vocal cords and larynx, unspecified	IMO	GHS-PACDC	DX_LEGACY	ACTIVE	N	1900-01-01	9999-12-31	2015-07-10
Diagnosis: ICD10 Code, J00-J99 Define	J00-J99	Diseases of the respiratory system	205354	Congenital vocal cord paralysis	ICD10CM	1	J38.00		Paralysis of vocal cords and larynx, unspecified	IMO	GHS-PACDC	DX_LEGACY	ACTIVE	N	1900-01-01	9999-12-31	2015-07-10
Diagnosis: ICD10 Code, J00-J99 Define	J00-J99	Diseases of the respiratory system	324247	Vocal cord weakness	ICD10CM	1	J38.00		Paralysis of vocal cords and larynx, unspecified	IMO	GHS-PACDC	DX_LEGACY	ACTIVE	N	1900-01-01	9999-12-31	2015-07-10
Diagnosis: ICD10 Code, J00-J99 Define	J00-J99	Diseases of the respiratory system	516338	Gerhardt syndrome	ICD10CM	1	J38.00		Paralysis of vocal cords and larynx, unspecified	IMO	GHS-PACDC	IMO_2015_R	ACTIVE	N	1900-01-01	9999-12-31	2015-07-10
Diagnosis: ICD10 Code, J00-J99 Define	J00-J99	Diseases of the respiratory system	77223	Unilateral complete paralysis of vocal cords or larynx	ICD10CM	1	J38.01		Paralysis of vocal cords and larynx, unilateral	IMO	GHS-PACDC	DX_LEGACY	ACTIVE	Y	1900-01-01	9999-12-31	2015-07-10
Diagnosis: ICD10 Code, J00-J99 Define	J00-J99	Diseases of the respiratory system	75927	Bilateral partial paralysis of vocal cords or larynx	ICD10CM	1	J38.02		Paralysis of vocal cords and larynx, bilateral	IMO	GHS-PACDC	DX_LEGACY	ACTIVE	N	1900-01-01	9999-12-31	2015-07-10
Diagnosis: ICD10 Code, J00-J99 Define	J00-J99	Diseases of the respiratory system	1192845	Paralysis of vocal cords and larynx, bilateral	ICD10CM	1	J38.02		Paralysis of vocal cords and larynx, bilateral	IMO	GHS-PACDC	IMO_2016_R	DELETED	Y	1900-01-01	9999-12-31	2016-06-02
Diagnosis: ICD10 Code, J00-J99 Define	J00-J99	Diseases of the respiratory system	20968	Laryngeal nodule	ICD10CM	1	J38.1		Polyp of vocal cord and larynx	IMO	GHS-PACDC	DX_LEGACY	ACTIVE	N	1900-01-01	9999-12-31	2015-07-10
Diagnosis: ICD10 Code, J00-J99 Define	J00-J99	Diseases of the respiratory system	202020	Sulcus vocalis of vocal cord	ICD10CM	1	J38.3		Other diseases of vocal cords	IMO	GHS-PACDC	DX_LEGACY	ACTIVE	N	1900-01-01	9999-12-31	2015-07-10
Diagnosis: ICD10 Code, J00-J99 Define	J00-J99	Diseases of the respiratory system	490392	Dysplasia of vocal cord	ICD10CM	1	J38.3		Other diseases of vocal cords	IMO	GHS-PACDC	IMO_2015_R	ACTIVE	N	1900-01-01	9999-12-31	2015-07-10
Diagnosis: ICD10 Code, J00-J99 Define	J00-J99	Diseases of the respiratory system	20982	Leukoplakia of vocal cords	ICD10CM	1	J38.3		Other diseases of vocal cords	IMO	GHS-PACDC	DX_LEGACY	ACTIVE	N	1900-01-01	9999-12-31	2015-07-10
Diagnosis: ICD10 Code, J00-J99 Define	J00-J99	Diseases of the respiratory system	253874	Lesion of vocal fold	ICD10CM	1	J38.3		Other diseases of vocal cords	IMO	GHS-PACDC	DX_LEGACY	ACTIVE	N	1900-01-01	9999-12-31	2015-07-10
Diagnosis: ICD10 Code, J00-J99 Define	J00-J99	Diseases of the respiratory system	20979	Abscess of vocal cords	ICD10CM	1	J38.3		Other diseases of vocal cords	IMO	GHS-PACDC	DX_LEGACY	ACTIVE	N	1900-01-01	9999-12-31	2015-07-10
Diagnosis: ICD10 Code, J00-J99 Define	J00-J99	Diseases of the respiratory system	213648	Spasmodic dysphonia	ICD10CM	1	J38.3		Other diseases of vocal cords	IMO	GHS-PACDC	DX_LEGACY	ACTIVE	N	1900-01-01	9999-12-31	2015-07-10
Diagnosis: ICD10 Code, J00-J99 Define	J00-J99	Diseases of the respiratory system	59953	Tongue edema	ICD10CM	1	J38.4		Edema of larynx	IMO	GHS-PACDC	DX_LEGACY	ACTIVE	N	1900-01-01	2016-05-07	2015-07-10
Diagnosis: ICD10 Code, J00-J99 Define	J00-J99	Diseases of the respiratory system	229294	Vocal cord edema	ICD10CM	1	J38.4		Edema of larynx	IMO	GHS-PACDC	DX_LEGACY	ACTIVE	N	1900-01-01	9999-12-31	2015-07-10
Diagnosis: ICD10 Code, J00-J99 Define	J00-J99	Diseases of the respiratory system	171247	Accumulated fluid in larynx	ICD10CM	1	J38.4		Edema of larynx	IMO	GHS-PACDC	DX_LEGACY	ACTIVE	N	1900-01-01	9999-12-31	2015-07-10
Diagnosis: ICD10 Code, J00-J99 Define	J00-J99	Diseases of the respiratory system	112227	Obstructed, larynx	ICD10CM	1	J38.6		Stenosis of larynx	IMO	GHS-PACDC	DX_LEGACY	ACTIVE	N	2016-04-10	9999-12-31	2015-07-10
Diagnosis: ICD10 Code, J00-J99 Define	J00-J99	Diseases of the respiratory system	1367616	Diseases of vocal cords and larynx, not elsewhere classified	ICD10CM	2	J38.7	J38.3,J38.7	Other diseases of larynx	IMO	GHS-PACDC	IMO_2018_R	ACTIVE	N	1900-01-01	9999-12-31	2017-10-30
Diagnosis: ICD10 Code, J00-J99 Define	J00-J99	Diseases of the respiratory system	110625	Laryngeal perichondritis	ICD10CM	1	J38.7		Other diseases of larynx	IMO	GHS-PACDC	DX_LEGACY	ACTIVE	N	1900-01-01	9999-12-31	2015-07-10
Diagnosis: ICD10 Code, J00-J99 Define	J00-J99	Diseases of the respiratory system	21452	Abductor spastic dysphonia	ICD10CM	1	J38.7		Other diseases of larynx	IMO	GHS-PACDC	DX_LEGACY	ACTIVE	N	1900-01-01	2016-05-07	2015-07-10
Diagnosis: ICD10 Code, J00-J99 Define	J00-J99	Diseases of the respiratory system	101410	Pharynx or nasopharynx cellulitis	ICD10CM	1	J39.1		Other abscess of pharynx	IMO	GHS-PACDC	DX_LEGACY	ACTIVE	N	2016-04-10	9999-12-31	2015-07-10
Diagnosis: ICD10 Code, J00-J99 Define	J00-J99	Diseases of the respiratory system	1101113	Disorder of hypopharynx	ICD10CM	1	J39.2		Other diseases of pharynx	IMO	GHS-PACDC	IMO_2016_R	ACTIVE	N	1900-01-01	9999-12-31	2016-06-02
Diagnosis: ICD10 Code, J00-J99 Define	J00-J99	Diseases of the respiratory system	218130	Pharyngeal or nasopharyngeal edema	ICD10CM	1	J39.2		Other diseases of pharynx	IMO	GHS-PACDC	DX_LEGACY	ACTIVE	N	1900-01-01	9999-12-31	2015-07-10
Diagnosis: ICD10 Code, J00-J99 Define	J00-J99	Diseases of the respiratory system	253682	Pharyngeal irritation	ICD10CM	1	J39.2		Other diseases of pharynx	IMO	GHS-PACDC	DX_LEGACY	ACTIVE	N	1900-01-01	9999-12-31	2015-07-10
Diagnosis: ICD10 Code, J00-J99 Define	J00-J99	Diseases of the respiratory system	493050	Hypopharyngeal cyst	ICD10CM	1	J39.2		Other diseases of pharynx	IMO	GHS-PACDC	IMO_2015_R	ACTIVE	N	1900-01-01	9999-12-31	2015-07-10
Diagnosis: ICD10 Code, J00-J99 Define	J00-J99	Diseases of the respiratory system	54334	Pharyngeal disease	ICD10CM	1	J39.2		Other diseases of pharynx	IMO	GHS-PACDC	DX_LEGACY	ACTIVE	N	1900-01-01	9999-12-31	2015-07-10
Diagnosis: ICD10 Code, J00-J99 Define	J00-J99	Diseases of the respiratory system	20927	Cellulitis of nasopharynx	ICD10CM	1	J39.2		Other diseases of pharynx	IMO	GHS-PACDC	DX_LEGACY	ACTIVE	N	1900-01-01	9999-12-31	2015-07-10
Diagnosis: ICD10 Code, J00-J99 Define	J00-J99	Diseases of the respiratory system	204350	Mass of nasopharynx	ICD10CM	1	J39.2		Other diseases of pharynx	IMO	GHS-PACDC	DX_LEGACY	ACTIVE	N	1900-01-01	9999-12-31	2015-07-10
Diagnosis: ICD10 Code, J00-J99 Define	J00-J99	Diseases of the respiratory system	73562	Abscess of pharynx or nasopharynx	ICD10CM	1	J39.2		Other diseases of pharynx	IMO	GHS-PACDC	DX_LEGACY	ACTIVE	N	1900-01-01	9999-12-31	2015-07-10
Diagnosis: ICD10 Code, J00-J99 Define	J00-J99	Diseases of the respiratory system	107451	Edema pharynx	ICD10CM	1	J39.2		Other diseases of pharynx	IMO	GHS-PACDC	DX_LEGACY	ACTIVE	N	1900-01-01	9999-12-31	2015-07-10
Diagnosis: ICD10 Code, J00-J99 Define	J00-J99	Diseases of the respiratory system	5420	Upper respiratory tract hypersensitivity reaction	ICD10CM	1	J39.3		Upper respiratory tract hypersensitivity reaction, site un	IMO	GHS-PACDC	DX_LEGACY	ACTIVE	N	1900-01-01	9999-12-31	2015-07-10
Diagnosis: ICD10 Code, J00-J99 Define	J00-J99	Diseases of the respiratory system	257816	Tracheal mass	ICD10CM	1	J39.8		Other specified diseases of upper respiratory tract	IMO	GHS-PACDC	DX_LEGACY	ACTIVE	N	2016-04-10	9999-12-31	2015-07-10
Diagnosis: ICD10 Code, J00-J99 Define	J00-J99	Diseases of the respiratory system	223408	Disorder of trachea or bronchus	ICD10CM	1	J39.8	J39.8,J98.09	Other specified diseases of upper respiratory tract	IMO	GHS-PACDC	DX_LEGACY	ACTIVE	N	1900-01-01	9999-12-31	2015-07-10
Diagnosis: ICD10 Code, J00-J99 Define	J00-J99	Diseases of the respiratory system	493216	Stenosis of tracheal stoma	ICD10CM	1	J39.8		Other specified diseases of upper respiratory tract	IMO	GHS-PACDC	IMO_2015_R	ACTIVE	N	1900-01-01	9999-12-31	2015-07-10
Diagnosis: ICD10 Code, J00-J99 Define	J00-J99	Diseases of the respiratory system	20863	Disease of upper respiratory tract	ICD10CM	1	J39.9		Disease of upper respiratory tract, unspecified	IMO	GHS-PACDC	DX_LEGACY	ACTIVE	N	1900-01-01	9999-12-31	2015-07-10
Diagnosis: ICD10 Code, J00-J99 Define	J00-J99	Diseases of the respiratory system	21009	Chronic catarrhal bronchitis (HCC)	ICD10CM	1	J41.0		Simple chronic bronchitis	IMO	GHS-PACDC	DX_LEGACY	ACTIVE	N	1900-01-01	9999-12-31	2015-07-10
Diagnosis: ICD10 Code, J00-J99 Define	J00-J99	Diseases of the respiratory system	62172	Bronchitis, chronic, mucopurulent (HCC)	ICD10CM	1	J41.1		Mucopurulent chronic bronchitis	IMO	GHS-PACDC	DX_LEGACY	ACTIVE	N	1900-01-01	9999-12-31	2015-07-10
Diagnosis: ICD10 Code, J00-J99 Define	J00-J99	Diseases of the respiratory system	533633	Mucopurulent chronic bronchitis	ICD10CM	1	J41.1		Mucopurulent chronic bronchitis	IMO	GHS-PACDC	IMO_2015_R	ACTIVE	Y	1900-01-01	9999-12-31	2015-07-10
Diagnosis: ICD10 Code, J00-J99 Define	J00-J99	Diseases of the respiratory system	538481	Mixed simple and mucopurulent chronic bronchitis	ICD10CM	1	J41.8		Mixed simple and mucopurulent chronic bronchitis	IMO	GHS-PACDC	IMO_2015_R	ACTIVE	Y	1900-01-01	9999-12-31	2015-07-10
Diagnosis: ICD10 Code, J00-J99 Define	J00-J99	Diseases of the respiratory system	68503	Unilateral emphysema syndrome (HCC)	ICD10CM	1	J43.0		Unilateral pulmonary emphysema [MacLeod's syndrome	IMO	GHS-PACDC	DX_LEGACY	ACTIVE	N	1900-01-01	9999-12-31	2015-07-10
Diagnosis: ICD10 Code, J00-J99 Define	J00-J99	Diseases of the respiratory system	266766	Unilateral pulmonary emphysema (MacLeod's syndrome) (HCC)	ICD10CM	1	J43.0		Unilateral pulmonary emphysema [MacLeod's syndrome	IMO	GHS-PACDC	DX_LEGACY	ACTIVE	N	1900-01-01	9999-12-31	2015-07-10
Diagnosis: ICD10 Code, J00-J99 Define	J00-J99	Diseases of the respiratory system	21063	Panlobular emphysema (HCC)	ICD10CM	1	J43.1		Panlobular emphysema	IMO	GHS-PACDC	DX_LEGACY	ACTIVE	N	1900-01-01	9999-12-31	2015-07-10
Diagnosis: ICD10 Code, J00-J99 Define	J00-J99	Diseases of the respiratory system	318002	Nocturnal hypoxemia due to emphysema (HCC)	ICD10CM	1	J43.9	J43.9,G47.36	Emphysema, unspecified	IMO	GHS-PACDC	DX_LEGACY	ACTIVE	N	1900-01-01	9999-12-31	2015-07-10
Diagnosis: ICD10 Code, J00-J99 Define	J00-J99	Diseases of the respiratory system	166036	Emphysematous COPD (HCC)	ICD10CM	1	J43.9		Emphysema, unspecified	IMO	GHS-PACDC	DX_LEGACY	ACTIVE	N	1900-01-01	9999-12-31	2015-07-10
Diagnosis: ICD10 Code, J00-J99 Define	J00-J99	Diseases of the respiratory system	21064	Vesicular emphysema (HCC)	ICD10CM	1	J43.9		Emphysema, unspecified	IMO	GHS-PACDC	DX_LEGACY	ACTIVE	N	2017-09-17	9999-12-31	2017-10-30
Diagnosis: ICD10 Code, J00-J99 Define	J00-J99	Diseases of the respiratory system	21074	Ruptured emphysematous bleb of lung (HCC)	ICD10CM	1	J43.9		Emphysema, unspecified	IMO	GHS-PACDC	DX_LEGACY	ACTIVE	N	2016-04-10	9999-12-31	2015-07-10
Diagnosis: ICD10 Code, J00-J99 Define	J00-J99	Diseases of the respiratory system	615461	Other chronic obstructive pulmonary disease	ICD10CM	1	J44		Other chronic obstructive pulmonary disease	IMO	GHS-PACDC	IMO_2015_R	ACTIVE	Y	1900-01-01	9999-12-31	2015-07-10
Diagnosis: ICD10 Code, J00-J99 Define	J00-J99	Diseases of the respiratory system	538582	Chronic obstructive pulmonary disease with acute lower respiratory infection	ICD10CM	1	J44.0		Chronic obstructive pulmonary disease with acute lower	IMO	GHS-PACDC	IMO_2015_R	ACTIVE	Y	1900-01-01	9999-12-31	2015-07-10
Diagnosis: ICD10 Code, J00-J99 Define	J00-J99	Diseases of the respiratory system	412677	Acute infective exacerbation of chronic obstructive airway disease (HCC)	ICD10CM	1	J44.1		Chronic obstructive pulmonary disease with (acute) exac	IMO	GHS-PACDC	DX_LEGACY	ACTIVE	N	1900-01-01	9999-12-31	2015-07-10
Diagnosis: ICD10 Code, J00-J99 Define	J00-J99	Diseases of the respiratory system	1176883	Chronic obstructive pulmonary disease with (acute) exacerbation (HCC)	ICD10CM	1	J44.1		Chronic obstructive pulmonary disease with (acute) exac	IMO	GHS-PACDC	IMO_2016_R	ACTIVE	N	1900-01-01	9999-12-31	2016-06-02
Diagnosis: ICD10 Code, J00-J99 Define	J00-J99	Diseases of the respiratory system	303814	Asthma, chronic obstructive, with acute exacerbation (HCC)	ICD10CM	1	J44.1	J44.1,J45.901	Chronic obstructive pulmonary disease with (acute) exac	IMO	GHS-PACDC	DX_LEGACY	ACTIVE	N	2016-04-10	9999-12-31	2015-07-10
Diagnosis: ICD10 Code, J00-J99 Define	J00-J99	Diseases of the respiratory system	1124226	Chronic obstructive pulmonary disease with bronchial hyperresponsiveness (HCC)	ICD10CM	1	J44.1		Chronic obstructive pulmonary disease with (acute) exac	IMO	GHS-PACDC	IMO_2016_R	ACTIVE	N	1900-01-01	9999-12-31	2016-06-02
Diagnosis: ICD10 Code, J00-J99 Define	J00-J99	Diseases of the respiratory system	16780	Acute bronchitis with COPD (HCC)	ICD10CM	1	J44.1		Chronic obstructive pulmonary disease with (acute) exac	IMO	GHS-PACDC	DX_LEGACY	ACTIVE	N	1900-01-01	2016-05-07	2015-07-10
Diagnosis: ICD10 Code, J00-J99 Define	J00-J99	Diseases of the respiratory system	960773	Asthma with irreversible airway obstruction, unspecified asthma severity, with status asthmatic	ICD10CM	2	J44.9	J45.902,J44.9	Chronic obstructive pulmonary disease, unspecified	IMO	GHS-PACDC	IMO_2015_R	ACTIVE	N	2017-09-17	9999-12-31	2017-10-30
Diagnosis: ICD10 Code, J00-J99 Define	J00-J99	Diseases of the respiratory system	1102473	COPD without exacerbation (HCC)	ICD10CM	1	J44.9		Chronic obstructive pulmonary disease, unspecified	IMO	GHS-PACDC	IMO_2016_R	ACTIVE	N	1900-01-01	9999-12-31	2016-06-02
Diagnosis: ICD10 Code, J00-J99 Define	J00-J99	Diseases of the respiratory system	137160	Bronchitis with emphysema	ICD10CM	1	J44.9		Chronic obstructive pulmonary disease, unspecified	IMO	GHS-PACDC	DX_LEGACY	DELETED	Y	1900-01-01	2016-05-07	2015-07-10
Diagnosis: ICD10 Code, J00-J99 Define	J00-J99	Diseases of the respiratory system	211748	Asthma with COPD with exacerbation (HCC)	ICD10CM	1	J44.9		Chronic obstructive pulmonary disease, unspecified	IMO	GHS-PACDC	DX_LEGACY	ACTIVE	N	1900-01-01	2016-05-07	2015-07-10
Diagnosis: ICD10 Code, J00-J99 Define	J00-J99	Diseases of the respiratory system	1304406	Stage 2 moderate chronic obstructive pulmonary disease by Global Initiative for Chronic Obstru	ICD10CM	1	J44.9		Chronic obstructive pulmonary disease, unspecified	IMO	GHS-PACDC	IMO_2017_R	ACTIVE	N	1900-01-01	9999-12-31	2017-04-14
Diagnosis: ICD10 Code, J00-J99 Define	J00-J99	Diseases of the respiratory system	249789	Asthma with COPD (HCC)	ICD10CM	1	J44.9		Chronic obstructive pulmonary disease, unspecified	IMO	GHS-PACDC	DX_LEGACY	ACTIVE	N	2017-09-17	9999-12-31	2017-10-30
Diagnosis: ICD10 Code, J00-J99 Define	J00-J99	Diseases of the respiratory system	464055	Mild intermittent asthma with irreversible airway obstruction with acute exacerbation	ICD10CM	2	J44.9	J45.21,J44.9	Chronic obstructive pulmonary disease, unspecified	IMO	GHS-PACDC	IMO_2015_R	ACTIVE	N	2017-09-17	9999-12-31	2017-10-30
Diagnosis: ICD10 Code, J00-J99 Define	J00-J99	Diseases of the respiratory system	137161	Bronchitis with chronic airway obstruction (HCC)	ICD10CM	1	J44.9		Chronic obstructive pulmonary disease, unspecified	IMO	GHS-PACDC	DX_LEGACY	ACTIVE	N	1900-01-01	9999-12-31	2015-07-10
Diagnosis: ICD10 Code, J00-J99 Define	J00-J99	Diseases of the respiratory system	18866	COPD, severe (HCC)	ICD10CM	1	J44.9		Chronic obstructive pulmonary disease, unspecified	IMO	GHS-PACDC	DX_LEGACY	ACTIVE	N	1900-01-01	9999-12-31	2015-07-10
Diagnosis: ICD10 Code, J00-J99 Define	J00-J99	Diseases of the respiratory system	1107686	COPD suggested by initial evaluation (HCC)	ICD10CM	1	J44.9		Chronic obstructive pulmonary disease, unspecified	IMO	GHS-PACDC	IMO_2016_R	ACTIVE	N	1900-01-01	9999-12-31	2016-06-02
Diagnosis: ICD10 Code, J00-J99 Define	J00-J99	Diseases of the respiratory system	159977	Chronic airway obstruction, not elsewhere classified	ICD10CM	1	J44.9		Chronic obstructive pulmonary disease, unspecified	IMO	GHS-PACDC	DX_LEGACY	ACTIVE	Y	1900-01-01	9999-12-31	2015-07-10
Diagnosis: ICD10 Code, J00-J99 Define	J00-J99	Diseases of the respiratory system	955194	Hay asthma, mild intermittent, uncomplicated	ICD10CM	1	J45.20		Mild intermittent asthma, uncomplicated	IMO	GHS-PACDC	IMO_2015_R	ACTIVE	N	1900-01-01	9999-12-31	2015-07-10
Diagnosis: ICD10 Code, J00-J99 Define	J00-J99	Diseases of the respiratory system	459348	Mild intermittent rapid onset asthma without complication	ICD10CM	1	J45.20		Mild intermittent asthma, uncomplicated	IMO	GHS-PACDC	IMO_2015_R	ACTIVE	N	1900-01-01	9999-12-31	2015-07-10
Diagnosis: ICD10 Code, J00-J99 Define	J00-J99	Diseases of the respiratory system	955760	Asthma, non-allergic, with acute exacerbation	ICD10CM	1	J45.21		Mild intermittent asthma with (acute) exacerbation	IMO	GHS-PACDC	IMO_2015_R	DELETED	Y	1900-01-01	9999-12-31	2015-07-10
Diagnosis: ICD10 Code, J00-J99 Define	J00-J99	Diseases of the respiratory system	1106101	Mild intermittent non-allergic asthma with acute exacerbation	ICD10CM	1	J45.21		Mild intermittent asthma with (acute) exacerbation	IMO	GHS-PACDC	IMO_2016_R	ACTIVE	N	1900-01-01	9999-12-31	2016-06-02
Diagnosis: ICD10 Code, J00-J99 Define	J00-J99	Diseases of the respiratory system	482665	Atopic asthma with acute exacerbation	ICD10CM	1	J45.21		Mild intermittent asthma with (acute) exacerbation	IMO	GHS-PACDC	IMO_2015_R	ACTIVE	N	1900-01-01	2016-10-14	2015-07-10
Diagnosis: ICD10 Code, J00-J99 Define	J00-J99	Diseases of the respiratory system	466284	Poorly controlled intermittent asthma with acute exacerbation	ICD10CM	1	J45.21		Mild intermittent asthma with (acute) exacerbation	IMO	GHS-PACDC	IMO_2015_R	ACTIVE	N	1900-01-01	9999-12-31	2015-07-10
Diagnosis: ICD10 Code, J00-J99 Define	J00-J99	Diseases of the respiratory system	459682	Mild intermittent reactive airway disease with acute exacerbation	ICD10CM	1	J45.21		Mild intermittent asthma with (acute) exacerbation	IMO	GHS-PACDC	IMO_2015_R	ACTIVE	N	1900-01-01	9999-12-31	2015-07-10
Diagnosis: ICD10 Code, J00-J99 Define	J00-J99	Diseases of the respiratory system	957021	Asthma in adult, mild intermittent, with acute exacerbation	ICD10CM	1	J45.21		Mild intermittent asthma with (acute) exacerbation	IMO	GHS-PACDC	IMO_2015_R	ACTIVE	N	1900-01-01	9999-12-31	2015-07-10
Diagnosis: ICD10 Code, J00-J99 Define	J00-J99	Diseases of the respiratory system	962027	Extrinsic asthma, mild intermittent, with acute exacerbation	ICD10CM	1	J45.21		Mild intermittent asthma with (acute) exacerbation	IMO	GHS-PACDC	IMO_2015_R	ACTIVE	N	1900-01-01	9999-12-31	2015-07-10
Diagnosis: ICD10 Code, J00-J99 Define	J00-J99	Diseases of the respiratory system	1349671	Intermittent asthma with acute exacerbation	ICD10CM	1	J45.21		Mild intermittent asthma with (acute) exacerbation	IMO	GHS-PACDC	IMO_2018_R	ACTIVE	N	1900-01-01	9999-12-31	2017-10-30
Diagnosis: ICD10 Code, J00-J99 Define	J00-J99	Diseases of the respiratory system	957458	Asthma exacerbation, non-allergic, mild intermittent	ICD10CM	1	J45.21		Mild intermittent asthma with (acute) exacerbation	IMO	GHS-PACDC	IMO_2015_R	ACTIVE	N	1900-01-01	9999-12-31	2015-07-10
Diagnosis: ICD10 Code, J00-J99 Define	J00-J99	Diseases of the respiratory system	956770	Acute asthmatic bronchitis, mild intermittent	ICD10CM	1	J45.21		Mild intermittent asthma with (acute) exacerbation	IMO	GHS-PACDC	IMO_2015_R	DELETED	Y	1900-01-01	9999-12-31	2015-07-10
Diagnosis: ICD10 Code, J00-J99 Define	J00-J99	Diseases of the respiratory system	458866	Mild intermittent mixed asthma with status asthmaticus	ICD10CM	1	J45.22		Mild intermittent asthma with status asthmaticus	IMO	GHS-PACDC	IMO_2015_R	ACTIVE	N	1900-01-01	9999-12-31	2015-07-10
Diagnosis: ICD10 Code, J00-J99 Define	J00-J99	Diseases of the respiratory system	268900	Mild intermittent asthma with status asthmaticus	ICD10CM	1	J45.22		Mild intermittent asthma with status asthmaticus	IMO	GHS-PACDC	DX_LEGACY	ACTIVE	N	1900-01-01	9999-12-31	2015-07-10
Diagnosis: ICD10 Code, J00-J99 Define	J00-J99	Diseases of the respiratory system	260752	Well controlled mild persistent asthma	ICD10CM	1	J45.30		Mild persistent asthma, uncomplicated	IMO	GHS-PACDC	DX_LEGACY	ACTIVE	N	1900-01-01	9999-12-31	2015-07-10
Diagnosis: ICD10 Code, J00-J99 Define	J00-J99	Diseases of the respiratory system	963134	Extrinsic asthma, mild persistent, uncomplicated	ICD10CM	1	J45.30		Mild persistent asthma, uncomplicated	IMO	GHS-PACDC	IMO_2015_R	ACTIVE	N	1900-01-01	9999-12-31	2015-07-10
Diagnosis: ICD10 Code, J00-J99 Define	J00-J99	Diseases of the respiratory system	1114356	Well controlled mild persistent allergic asthma	ICD10CM	1	J45.30		Mild persistent asthma, uncomplicated	IMO	GHS-PACDC	IMO_2016_R	ACTIVE	N	1900-01-01	9999-12-31	2016-06-02

Diagnosis: ICD10 Code, J00-J99 Define	J00-J99	Diseases of the respiratory system	957181	AB (asthmatic bronchitis), mild persistent, uncomplicated	ICD10CM	1	J45.30		Mild persistent asthma, uncomplicated	IMO	GHS-PACDC	IMO_2015_R	ACTIVE	N	1900-01-01	9999-12-31	2015-07-10
Diagnosis: ICD10 Code, J00-J99 Define	J00-J99	Diseases of the respiratory system	1131454	Mild persistent asthma dependent on systemic steroids without complication	ICD10CM	1	J45.30	J45.30,Z79.52	Mild persistent asthma, uncomplicated	IMO	GHS-PACDC	IMO_2016_R	ACTIVE	N	1900-01-01	9999-12-31	2016-06-02
Diagnosis: ICD10 Code, J00-J99 Define	J00-J99	Diseases of the respiratory system	1131326	Mild persistent asthma not dependent on systemic steroids	ICD10CM	1	J45.30		Mild persistent asthma, uncomplicated	IMO	GHS-PACDC	IMO_2016_R	ACTIVE	N	1900-01-01	9999-12-31	2016-06-02
Diagnosis: ICD10 Code, J00-J99 Define	J00-J99	Diseases of the respiratory system	260751	Mild persistent asthma, well controlled	ICD10CM	1	J45.30		Mild persistent asthma, uncomplicated	IMO	GHS-PACDC	DX_LEGACY	ACTIVE	N	1900-01-01	9999-12-31	2015-07-10
Diagnosis: ICD10 Code, J00-J99 Define	J00-J99	Diseases of the respiratory system	1131416	Mild persistent asthma refractory to systemic steroids	ICD10CM	1	J45.30	J45.30,Z79.52	Mild persistent asthma, uncomplicated	IMO	GHS-PACDC	IMO_2016_R	ACTIVE	N	1900-01-01	9999-12-31	2016-06-02
Diagnosis: ICD10 Code, J00-J99 Define	J00-J99	Diseases of the respiratory system	958915	Bronchitis, allergic, mild persistent, uncomplicated	ICD10CM	1	J45.30		Mild persistent asthma, uncomplicated	IMO	GHS-PACDC	IMO_2015_R	ACTIVE	N	1900-01-01	9999-12-31	2015-07-10
Diagnosis: ICD10 Code, J00-J99 Define	J00-J99	Diseases of the respiratory system	489760	Mild persistent asthma with acute bronchitis and acute exacerbation	ICD10CM	2	J45.31	J20.9,J45.31	Mild persistent asthma with (acute) exacerbation	IMO	GHS-PACDC	IMO_2015_R	ACTIVE	N	1900-01-01	9999-12-31	2015-07-10
Diagnosis: ICD10 Code, J00-J99 Define	J00-J99	Diseases of the respiratory system	1186564	Mild persistent asthma with (acute) exacerbation	ICD10CM	1	J45.31		Mild persistent asthma with (acute) exacerbation	IMO	GHS-PACDC	IMO_2016_R	ACTIVE	N	1900-01-01	9999-12-31	2016-06-02
Diagnosis: ICD10 Code, J00-J99 Define	J00-J99	Diseases of the respiratory system	956739	Mixed asthma, mild persistent, with acute exacerbation	ICD10CM	1	J45.31		Mild persistent asthma with (acute) exacerbation	IMO	GHS-PACDC	IMO_2015_R	ACTIVE	N	1900-01-01	9999-12-31	2015-07-10
Diagnosis: ICD10 Code, J00-J99 Define	J00-J99	Diseases of the respiratory system	454720	Mild persistent intrinsic asthma without status asthmaticus with acute exacerbation	ICD10CM	1	J45.31		Mild persistent asthma with (acute) exacerbation	IMO	GHS-PACDC	IMO_2015_R	ACTIVE	N	1900-01-01	9999-12-31	2015-07-10
Diagnosis: ICD10 Code, J00-J99 Define	J00-J99	Diseases of the respiratory system	450345	Not well controlled mild persistent asthma with acute exacerbation	ICD10CM	1	J45.31		Mild persistent asthma with (acute) exacerbation	IMO	GHS-PACDC	IMO_2015_R	ACTIVE	N	1900-01-01	9999-12-31	2015-07-10
Diagnosis: ICD10 Code, J00-J99 Define	J00-J99	Diseases of the respiratory system	458934	Mild persistent allergic asthma with acute exacerbation	ICD10CM	1	J45.31		Mild persistent asthma with (acute) exacerbation	IMO	GHS-PACDC	IMO_2015_R	ACTIVE	N	1900-01-01	9999-12-31	2015-07-10
Diagnosis: ICD10 Code, J00-J99 Define	J00-J99	Diseases of the respiratory system	958309	Extrinsic asthma, mild persistent, with status asthmaticus	ICD10CM	1	J45.32		Mild persistent asthma with status asthmaticus	IMO	GHS-PACDC	IMO_2015_R	ACTIVE	N	1900-01-01	9999-12-31	2015-07-10
Diagnosis: ICD10 Code, J00-J99 Define	J00-J99	Diseases of the respiratory system	956420	Extrinsic allergic asthma, mild persistent, with status asthmaticus	ICD10CM	1	J45.32		Mild persistent asthma with status asthmaticus	IMO	GHS-PACDC	IMO_2015_R	ACTIVE	N	1900-01-01	9999-12-31	2015-07-10
Diagnosis: ICD10 Code, J00-J99 Define	J00-J99	Diseases of the respiratory system	960028	Asthma, extrinsic with status asthmaticus, mild persistent	ICD10CM	1	J45.32		Mild persistent asthma with status asthmaticus	IMO	GHS-PACDC	IMO_2015_R	ACTIVE	N	1900-01-01	9999-12-31	2015-07-10
Diagnosis: ICD10 Code, J00-J99 Define	J00-J99	Diseases of the respiratory system	963537	Asthma in pediatric patient, mild persistent, with status asthmaticus	ICD10CM	1	J45.32		Mild persistent asthma with status asthmaticus	IMO	GHS-PACDC	IMO_2015_R	ACTIVE	N	1900-01-01	9999-12-31	2015-07-10
Diagnosis: ICD10 Code, J00-J99 Define	J00-J99	Diseases of the respiratory system	963981	Asthma, extrinsic, with status asthmaticus, mild persistent	ICD10CM	1	J45.32		Mild persistent asthma with status asthmaticus	IMO	GHS-PACDC	IMO_2015_R	ACTIVE	N	1900-01-01	9999-12-31	2015-07-10
Diagnosis: ICD10 Code, J00-J99 Define	J00-J99	Diseases of the respiratory system	958672	Occupational asthma, mild persistent, with status asthmaticus	ICD10CM	1	J45.32	J45.32,Z57.9	Mild persistent asthma with status asthmaticus	IMO	GHS-PACDC	IMO_2015_R	ACTIVE	N	1900-01-01	9999-12-31	2015-07-10
Diagnosis: ICD10 Code, J00-J99 Define	J00-J99	Diseases of the respiratory system	453541	Moderate persistent reactive airway disease without complication	ICD10CM	1	J45.40		Moderate persistent asthma, uncomplicated	IMO	GHS-PACDC	IMO_2015_R	ACTIVE	N	1900-01-01	9999-12-31	2015-07-10
Diagnosis: ICD10 Code, J00-J99 Define	J00-J99	Diseases of the respiratory system	955550	Uncomplicated asthma, moderate persistent	ICD10CM	1	J45.40		Moderate persistent asthma, uncomplicated	IMO	GHS-PACDC	IMO_2015_R	DELETED	Y	1900-01-01	9999-12-31	2015-07-10
Diagnosis: ICD10 Code, J00-J99 Define	J00-J99	Diseases of the respiratory system	963066	Hay fever with asthma, moderate persistent, uncomplicated	ICD10CM	1	J45.40		Moderate persistent asthma, uncomplicated	IMO	GHS-PACDC	IMO_2015_R	ACTIVE	N	1900-01-01	9999-12-31	2015-07-10
Diagnosis: ICD10 Code, J00-J99 Define	J00-J99	Diseases of the respiratory system	959072	Reactive airway disease with wheezing, moderate persistent, uncomplicated	ICD10CM	1	J45.40		Moderate persistent asthma, uncomplicated	IMO	GHS-PACDC	IMO_2015_R	ACTIVE	N	1900-01-01	9999-12-31	2015-07-10
Diagnosis: ICD10 Code, J00-J99 Define	J00-J99	Diseases of the respiratory system	958844	Asthma, late onset, moderate persistent, uncomplicated	ICD10CM	1	J45.40		Moderate persistent asthma, uncomplicated	IMO	GHS-PACDC	IMO_2015_R	ACTIVE	N	1900-01-01	9999-12-31	2015-07-10
Diagnosis: ICD10 Code, J00-J99 Define	J00-J99	Diseases of the respiratory system	960543	Asthma in pediatric patient, moderate persistent, uncomplicated	ICD10CM	1	J45.40		Moderate persistent asthma, uncomplicated	IMO	GHS-PACDC	IMO_2015_R	ACTIVE	N	1900-01-01	9999-12-31	2015-07-10
Diagnosis: ICD10 Code, J00-J99 Define	J00-J99	Diseases of the respiratory system	470723	Moderate persistent cold-induced asthma without complication	ICD10CM	1	J45.40		Moderate persistent asthma, uncomplicated	IMO	GHS-PACDC	IMO_2015_R	ACTIVE	N	1900-01-01	9999-12-31	2015-07-10
Diagnosis: ICD10 Code, J00-J99 Define	J00-J99	Diseases of the respiratory system	459273	Moderate persistent late onset asthma without complication	ICD10CM	1	J45.40		Moderate persistent asthma, uncomplicated	IMO	GHS-PACDC	IMO_2015_R	ACTIVE	N	1900-01-01	9999-12-31	2015-07-10
Diagnosis: ICD10 Code, J00-J99 Define	J00-J99	Diseases of the respiratory system	454118	Moderate persistent extrinsic asthma without status asthmaticus with acute exacerbation	ICD10CM	1	J45.41		Moderate persistent asthma with (acute) exacerbation	IMO	GHS-PACDC	IMO_2015_R	ACTIVE	N	1900-01-01	9999-12-31	2015-07-10
Diagnosis: ICD10 Code, J00-J99 Define	J00-J99	Diseases of the respiratory system	961147	Rapid onset asthma, moderate persistent, with acute exacerbation	ICD10CM	1	J45.41		Moderate persistent asthma with (acute) exacerbation	IMO	GHS-PACDC	IMO_2015_R	ACTIVE	N	1900-01-01	9999-12-31	2015-07-10
Diagnosis: ICD10 Code, J00-J99 Define	J00-J99	Diseases of the respiratory system	960937	Extrinsic asthma with exacerbation, moderate persistent	ICD10CM	1	J45.41		Moderate persistent asthma with (acute) exacerbation	IMO	GHS-PACDC	IMO_2015_R	ACTIVE	N	1900-01-01	9999-12-31	2015-07-10
Diagnosis: ICD10 Code, J00-J99 Define	J00-J99	Diseases of the respiratory system	958913	Asthma with hay fever, moderate persistent, with acute exacerbation	ICD10CM	1	J45.41		Moderate persistent asthma with (acute) exacerbation	IMO	GHS-PACDC	IMO_2015_R	ACTIVE	N	1900-01-01	9999-12-31	2015-07-10
Diagnosis: ICD10 Code, J00-J99 Define	J00-J99	Diseases of the respiratory system	489767	Moderate persistent asthma with allergic rhinitis without status asthmaticus with acute exacerb	ICD10CM	1	J45.41		Moderate persistent asthma with (acute) exacerbation	IMO	GHS-PACDC	IMO_2015_R	ACTIVE	N	1900-01-01	9999-12-31	2015-07-10
Diagnosis: ICD10 Code, J00-J99 Define	J00-J99	Diseases of the respiratory system	955651	Rhinitis, allergic, with asthma, without status asthmaticus, moderate persistent, with acute exac	ICD10CM	1	J45.41		Moderate persistent asthma with (acute) exacerbation	IMO	GHS-PACDC	IMO_2015_R	ACTIVE	N	1900-01-01	9999-12-31	2015-07-10
Diagnosis: ICD10 Code, J00-J99 Define	J00-J99	Diseases of the respiratory system	957967	RAD (reactive airway disease), moderate persistent, with acute exacerbation	ICD10CM	1	J45.41		Moderate persistent asthma with (acute) exacerbation	IMO	GHS-PACDC	IMO_2015_R	ACTIVE	N	1900-01-01	9999-12-31	2015-07-10
Diagnosis: ICD10 Code, J00-J99 Define	J00-J99	Diseases of the respiratory system	1268798	Allergic rhinitis with moderate persistent asthma in acute exacerbation, without status asthmati	ICD10CM	1	J45.41		Moderate persistent asthma with (acute) exacerbation	IMO	GHS-PACDC	IMO_2017_R	ACTIVE	N	1900-01-01	9999-12-31	2016-09-30
Diagnosis: ICD10 Code, J00-J99 Define	J00-J99	Diseases of the respiratory system	956785	Reactive airway disease, moderate persistent, with acute exacerbation	ICD10CM	1	J45.41		Moderate persistent asthma with (acute) exacerbation	IMO	GHS-PACDC	IMO_2015_R	ACTIVE	N	1900-01-01	9999-12-31	2015-07-10
Diagnosis: ICD10 Code, J00-J99 Define	J00-J99	Diseases of the respiratory system	959600	Bronchial asthma, moderate persistent, with acute exacerbation	ICD10CM	1	J45.41		Moderate persistent asthma with (acute) exacerbation	IMO	GHS-PACDC	IMO_2015_R	DELETED	Y	1900-01-01	9999-12-31	2015-07-10
Diagnosis: ICD10 Code, J00-J99 Define	J00-J99	Diseases of the respiratory system	467371	Moderate persistent asthmatic bronchitis with acute exacerbation	ICD10CM	1	J45.41		Moderate persistent asthma with (acute) exacerbation	IMO	GHS-PACDC	IMO_2015_R	ACTIVE	N	1900-01-01	9999-12-31	2015-07-10
Diagnosis: ICD10 Code, J00-J99 Define	J00-J99	Diseases of the respiratory system	955883	Asthma exacerbation attacks, moderate persistent	ICD10CM	1	J45.41		Moderate persistent asthma with (acute) exacerbation	IMO	GHS-PACDC	IMO_2015_R	DELETED	Y	1900-01-01	9999-12-31	2015-07-10
Diagnosis: ICD10 Code, J00-J99 Define	J00-J99	Diseases of the respiratory system	961348	Asthma, exogenous, moderate persistent, with acute exacerbation	ICD10CM	1	J45.41		Moderate persistent asthma with (acute) exacerbation	IMO	GHS-PACDC	IMO_2015_R	ACTIVE	N	1900-01-01	9999-12-31	2015-07-10
Diagnosis: ICD10 Code, J00-J99 Define	J00-J99	Diseases of the respiratory system	1131281	Moderate persistent asthma dependent on systemic steroids with acute exacerbation	ICD10CM	1	J45.41	J45.41,Z79.52	Moderate persistent asthma with (acute) exacerbation	IMO	GHS-PACDC	IMO_2016_R	ACTIVE	N	1900-01-01	9999-12-31	2016-06-02
Diagnosis: ICD10 Code, J00-J99 Define	J00-J99	Diseases of the respiratory system	959228	Atopic asthma, moderate persistent, with status asthmaticus	ICD10CM	1	J45.42		Moderate persistent asthma with status asthmaticus	IMO	GHS-PACDC	IMO_2015_R	ACTIVE	N	1900-01-01	9999-12-31	2015-07-10
Diagnosis: ICD10 Code, J00-J99 Define	J00-J99	Diseases of the respiratory system	963012	Asthma, extrinsic, with status asthmaticus, moderate persistent	ICD10CM	1	J45.42		Moderate persistent asthma with status asthmaticus	IMO	GHS-PACDC	IMO_2015_R	ACTIVE	N	1900-01-01	9999-12-31	2015-07-10
Diagnosis: ICD10 Code, J00-J99 Define	J00-J99	Diseases of the respiratory system	954209	Steroid-dependent asthma, moderate persistent, with status asthmaticus	ICD10CM	1	J45.42		Moderate persistent asthma with status asthmaticus	IMO	GHS-PACDC	IMO_2015_R	ACTIVE	N	1900-01-01	9999-12-31	2015-07-10
Diagnosis: ICD10 Code, J00-J99 Define	J00-J99	Diseases of the respiratory system	460780	Moderate persistent asthma with status asthmaticus in adult	ICD10CM	1	J45.42		Moderate persistent asthma with status asthmaticus	IMO	GHS-PACDC	IMO_2015_R	ACTIVE	N	1900-01-01	9999-12-31	2015-07-10
Diagnosis: ICD10 Code, J00-J99 Define	J00-J99	Diseases of the respiratory system	955740	Asthma, moderate persistent, with status asthmaticus	ICD10CM	1	J45.42		Moderate persistent asthma with status asthmaticus	IMO	GHS-PACDC	IMO_2015_R	DELETED	Y	1900-01-01	9999-12-31	2015-07-10
Diagnosis: ICD10 Code, J00-J99 Define	J00-J99	Diseases of the respiratory system	954706	Asthma, very poorly controlled, moderate persistent, with status asthmaticus	ICD10CM	1	J45.42		Moderate persistent asthma with status asthmaticus	IMO	GHS-PACDC	IMO_2015_R	ACTIVE	N	1900-01-01	9999-12-31	2015-07-10
Diagnosis: ICD10 Code, J00-J99 Define	J00-J99	Diseases of the respiratory system	463953	Moderate persistent late onset asthma with status asthmaticus	ICD10CM	1	J45.42		Moderate persistent asthma with status asthmaticus	IMO	GHS-PACDC	IMO_2015_R	ACTIVE	N	1900-01-01	9999-12-31	2015-07-10
Diagnosis: ICD10 Code, J00-J99 Define	J00-J99	Diseases of the respiratory system	451266	Severe persistent allergic asthma without complication	ICD10CM	1	J45.50		Severe persistent asthma, uncomplicated	IMO	GHS-PACDC	IMO_2015_R	ACTIVE	N	1900-01-01	9999-12-31	2015-07-10
Diagnosis: ICD10 Code, J00-J99 Define	J00-J99	Diseases of the respiratory system	954410	Asthma with irreversible airway obstruction, severe persistent, uncomplicated (HCC)	ICD10CM	1	J45.50	J45.50,J44.9	Severe persistent asthma, uncomplicated	IMO	GHS-PACDC	IMO_2015_R	ACTIVE	N	2016-04-10	9999-12-31	2015-07-10
Diagnosis: ICD10 Code, J00-J99 Define	J00-J99	Diseases of the respiratory system	958182	Hay asthma, severe persistent, uncomplicated	ICD10CM	1	J45.50		Severe persistent asthma, uncomplicated	IMO	GHS-PACDC	IMO_2015_R	ACTIVE	N	1900-01-01	9999-12-31	2015-07-10
Diagnosis: ICD10 Code, J00-J99 Define	J00-J99	Diseases of the respiratory system	963064	Asthma, extrinsic, severe persistent, uncomplicated	ICD10CM	1	J45.50		Severe persistent asthma, uncomplicated	IMO	GHS-PACDC	IMO_2015_R	ACTIVE	N	1900-01-01	9999-12-31	2015-07-10
Diagnosis: ICD10 Code, J00-J99 Define	J00-J99	Diseases of the respiratory system	455226	Severe persistent intrinsic asthma without status asthmaticus without complication	ICD10CM	1	J45.50		Severe persistent asthma, uncomplicated	IMO	GHS-PACDC	IMO_2015_R	ACTIVE	N	1900-01-01	9999-12-31	2015-07-10
Diagnosis: ICD10 Code, J00-J99 Define	J00-J99	Diseases of the respiratory system	1106375	Severe persistent asthma with intensive monitoring	ICD10CM	1	J45.50		Severe persistent asthma, uncomplicated	IMO	GHS-PACDC	IMO_2016_R	ACTIVE	N	1900-01-01	9999-12-31	2016-06-02
Diagnosis: ICD10 Code, J00-J99 Define	J00-J99	Diseases of the respiratory system	956389	Allergy-induced asthma, severe persistent, uncomplicated	ICD10CM	1	J45.50		Severe persistent asthma, uncomplicated	IMO	GHS-PACDC	IMO_2015_R	ACTIVE	N	1900-01-01	9999-12-31	2015-07-10
Diagnosis: ICD10 Code, J00-J99 Define	J00-J99	Diseases of the respiratory system	452533	Severe persistent extrinsic asthma without complication	ICD10CM	1	J45.50		Severe persistent asthma, uncomplicated	IMO	GHS-PACDC	IMO_2015_R	ACTIVE	N	1900-01-01	9999-12-31	2015-07-10
Diagnosis: ICD10 Code, J00-J99 Define	J00-J99	Diseases of the respiratory system	961921	RAD (reactive airway disease), severe persistent, with acute exacerbation	ICD10CM	1	J45.51		Severe persistent asthma with (acute) exacerbation	IMO	GHS-PACDC	IMO_2015_R	ACTIVE	N	1900-01-01	9999-12-31	2015-07-10
Diagnosis: ICD10 Code, J00-J99 Define	J00-J99	Diseases of the respiratory system	469294	Severe persistent chronic asthma with acute exacerbation	ICD10CM	1	J45.51		Severe persistent asthma with (acute) exacerbation	IMO	GHS-PACDC	IMO_2015_R	ACTIVE	N	1900-01-01	9999-12-31	2015-07-10
Diagnosis: ICD10 Code, J00-J99 Define	J00-J99	Diseases of the respiratory system	958692	Acute asthma exacerbation, severe persistent	ICD10CM	1	J45.51		Severe persistent asthma with (acute) exacerbation	IMO	GHS-PACDC	IMO_2015_R	DELETED	Y	1900-01-01	9999-12-31	2015-07-10
Diagnosis: ICD10 Code, J00-J99 Define	J00-J99	Diseases of the respiratory system	280444	Severe persistent asthma with acute exacerbation	ICD10CM	1	J45.51		Severe persistent asthma with (acute) exacerbation	IMO	GHS-PACDC	DX_LEGACY	ACTIVE	N	1900-01-01	9999-12-31	2015-07-10
Diagnosis: ICD10 Code, J00-J99 Define	J00-J99	Diseases of the respiratory system	1115059	Acute severe exacerbation of severe persistent asthma with allergic rhinitis	ICD10CM	1	J45.51		Severe persistent asthma with (acute) exacerbation	IMO	GHS-PACDC	IMO_2016_R	ACTIVE	N	1900-01-01	9999-12-31	2016-06-02
Diagnosis: ICD10 Code, J00-J99 Define	J00-J99	Diseases of the respiratory system	960614	Allergic atopic asthma, severe persistent, with acute exacerbation	ICD10CM	1	J45.51		Severe persistent asthma with (acute) exacerbation	IMO	GHS-PACDC	IMO_2015_R	ACTIVE	N	1900-01-01	9999-12-31	2015-07-10
Diagnosis: ICD10 Code, J00-J99 Define	J00-J99	Diseases of the respiratory system	958195	Severe persistent asthma, poorly-controlled, with acute exacerbation	ICD10CM	1	J45.51		Severe persistent asthma with (acute) exacerbation	IMO	GHS-PACDC	IMO_2015_R	ACTIVE	N	1900-01-01	9999-12-31	2015-07-10
Diagnosis: ICD10 Code, J00-J99 Define	J00-J99	Diseases of the respiratory system	466239	Severe persistent reactive airway disease with wheezing with status asthmaticus	ICD10CM	1	J45.52		Severe persistent asthma with status asthmaticus	IMO	GHS-PACDC	IMO_2015_R	ACTIVE	N	1900-01-01	9999-12-31	2015-07-10
Diagnosis: ICD10 Code, J00-J99 Define	J00-J99	Diseases of the respiratory system	452919	Severe persistent allergic asthma with status asthmaticus	ICD10CM	1	J45.52		Severe persistent asthma with status asthmaticus	IMO	GHS-PACDC	IMO_2015_R	ACTIVE	N	1900-01-01	9999-12-31	2015-07-10
Diagnosis: ICD10 Code, J00-J99 Define	J00-J99	Diseases of the respiratory system	954881	Atopic asthma, severe persistent, with status asthmaticus	ICD10CM	1	J45.52		Severe persistent asthma with status asthmaticus	IMO	GHS-PACDC	IMO_2015_R	ACTIVE	N	1900-01-01	9999-12-31	2015-07-10
Diagnosis: ICD10 Code, J00-J99 Define	J00-J99	Diseases of the respiratory system	450763	Poorly controlled severe persistent asthma with status asthmaticus	ICD10CM	1	J45.52		Severe persistent asthma with status asthmaticus	IMO	GHS-PACDC	IMO_2015_R	ACTIVE	N	1900-01-01	9999-12-31	2015-07-10
Diagnosis: ICD10 Code, J00-J99 Define	J00-J99	Diseases of the respiratory system	457600	Severe persistent late onset asthma with status asthmaticus	ICD10CM	1	J45.52		Severe persistent asthma with status asthmaticus	IMO	GHS-PACDC	IMO_2015_R	ACTIVE	N	1900-01-01	9999-12-31	2015-07-10
Diagnosis: ICD10 Code, J00-J99 Define	J00-J99	Diseases of the respiratory system	961667	Hay asthma, severe persistent, with status asthmaticus	ICD10CM	1	J45.52		Severe persistent asthma with status asthmaticus	IMO	GHS-PACDC	IMO_2015_R	ACTIVE	N	1900-01-01	9999-12-31	2015-07-10
Diagnosis: ICD10 Code, J00-J99 Define	J00-J99	Diseases of the respiratory system	957557	Asthma, very poorly controlled, severe persistent, with status asthmaticus	ICD10CM	1	J45.52		Severe persistent asthma with status asthmaticus	IMO	GHS-PACDC	IMO_2015_R	ACTIVE	N	1900-01-01	9999-12-31	2015-07-10
Diagnosis: ICD10 Code, J00-J99 Define	J00-J99	Diseases of the respiratory system	466026	Severe persistent childhood asthma with status asthmaticus	ICD10CM	1	J45.52		Severe persistent asthma with status asthmaticus	IMO	GHS-PACDC	IMO_2015_R	ACTIVE	N	1900-01-01	9999-12-31	2015-07-10
Diagnosis: ICD10 Code, J00-J99 Define	J00-J99	Diseases of the respiratory system	954416	Extrinsic allergic asthma, severe persistent, with status asthmaticus	ICD10CM	1	J45.52		Severe persistent asthma with status asthmaticus	IMO	GHS-PACDC	IMO_2015_R	ACTIVE	N	1900-01-01	9999-12-31	2015-07-10
Diagnosis: ICD10 Code, J00-J99 Define	J00-J99	Diseases of the respiratory system	1427098	Mild asthma with acute exacerbation, unspecified whether persistent	ICD10CM	1	J45.901		Unspecified asthma with (acute) exacerbation	IMO	GHS-PACDC	IMO_2018_R	ACTIVE	N	1900-01-01	9999-12-31	2017-10-30
Diagnosis: ICD10 Code, J00-J99 Define	J00-J99	Diseases of the respiratory system	328273	Asthma exacerbation attacks	ICD10CM	1	J45.901		Unspecified asthma with (acute) exacerbation	IMO	GHS-PACDC	DX_LEGACY	ACTIVE	N	1900-01-01	9999-12-31	2015-07-10
Diagnosis: ICD10 Code, J00-J99 Define	J00-J99	Diseases of the respiratory system	1354940	Moderate asthma with exacerbation	ICD10CM	1	J45.901		Unspecified asthma with (acute) exacerbation	IMO	GHS-PACDC	IMO_2018_R	ACTIVE	N	1900-01-01	9999-12-31	2017-10-30
Diagnosis: ICD10 Code, J00-J99 Define	J00-J99	Diseases of the respiratory system	961389	Asthma exacerbation attacks, unspecified asthma severity	ICD10CM	1	J45.901		Unspecified asthma with (acute) exacerbation	IMO	GHS-PACDC	IMO_2015_R	DELETED	Y	1900-01-01	9999-12-31	2015-07-10
Diagnosis: ICD10 Code, J00-J99 Define	J00-J99	Diseases of the respiratory system	137170	Asthma with exacerbation	ICD10CM	1	J45.901		Unspecified asthma with (acute) exacerbation	IMO	GHS-PACDC	DX_LEGACY	ACTIVE	N	1900-01-01	9999-12-31	2015-07-10
Diagnosis: ICD10 Code, J00-J99 Define	J00-J99	Diseases of the respiratory system	957832	Non-IgE mediated allergic asthma, unspecified asthma severity, with acute exacerbation	ICD10CM	1	J45.901		Unspecified asthma with (acute) exacerbation	IMO	GHS-PACDC	IMO_2015_R	ACTIVE	N	1900-01-01	9999-12-31	2015-07-10
Diagnosis: ICD10 Code, J00-J99 Define	J00-J99	Diseases of the respiratory system	958164	Asthma, intrinsic with exacerbation, unspecified asthma severity	ICD10CM	1	J45.901		Unspecified asthma with (acute) exacerbation	IMO	GHS-PACDC	IMO_2015_R	ACTIVE	N	1900-01-01	9999-12-31	2015-07-10
Diagnosis: ICD10 Code, J00-J99 Define	J00-J99	Diseases of the respiratory system	1257727	Allergic asthma with severe asthma attack	ICD10CM	1	J45.901		Unspecified asthma with (acute) exacerbation	IMO	GHS-PACDC	IMO_2017_R	ACTIVE	N	1900-01-01	9999-12-31	2016-09-30
Diagnosis: ICD10 Code, J00-J99 Define	J00-J99	Diseases of the respiratory system	301638	Acute exacerbation of COPD with asthma (HCC)	ICD10CM	2	J45.901	J44.1,J45.901	Unspecified asthma with (acute) exacerbation	IMO	GHS-PACDC	DX_LEGACY	ACTIVE	N	2016-04-10	9999-12-31	2015-07-10
Diagnosis: ICD10 Code, J00-J99 Define	J00-J99	Diseases of the respiratory system	256662	Asthmatic bronchitis with exacerbation	ICD10CM	1	J45.901		Unspecified asthma with (acute) exacerbation	IMO	GHS-PACDC	DX_LEGACY	ACTIVE	N	1900-01-01	9999-12-31	2015-07-10
Diagnosis: ICD10 Code, J00-J99 Define	J00-J99	Diseases of the respiratory system	504515	Allergic rhinitis with acute exacerbation of asthma without status asthmaticus	ICD10CM	1	J45.901		Unspecified asthma with (acute) exacerbation	IMO	GHS-PACDC	IMO_2015_R	ACTIVE	N	1900-01-01	9999-12-31	2015-07-10
Diagnosis: ICD10 Code, J00-J99 Define	J00-J99	Diseases of the respiratory system	957747	Asthma with allergic rhinitis, unspecified asthma severity, with acute exacerbation	ICD10CM	1	J45.901		Unspecified asthma with (acute) exacerbation	IMO	GHS-PACDC	IMO_2015_R	ACTIVE	N	1900-01-01	9999-12-31	2015-07-10
Diagnosis: ICD10 Code, J00-J99 Define	J00-J99	Diseases of the respiratory system	1272850	Exacerbation of asthma associated with volcanic smog exposure	ICD10CM	1	J45.901	J45.901,Z77.110	Unspecified asthma with (acute) exacerbation	IMO	GHS-PACDC	IMO_2017_R	ACTIVE	N	2017-03-31	9999-12-31	2017-04-14
Diagnosis: ICD10 Code, J00-J99 Define	J00-J99	Diseases of the respiratory system	482308	Allergic atopic asthma with acute exacerbation	ICD10CM	1	J45.901		Unspecified asthma with (acute) exacerbation	IMO	GHS-PACDC	IMO_2015_R	ACTIVE	N	1900-01-01	9999-12-31	2015-07-10
Diagnosis: ICD10 Code, J00-J99 Define	J00-J99	Diseases of the respiratory system	1427212	Non-IgE mediated allergic asthma with status asthmaticus, unspecified asthma severity, unspeci	ICD10CM	1	J45.902		Unspecified asthma with status asthmaticus	IMO	GHS-PACDC	IMO_2018_R	ACTIVE	N	1900-01-01	9999-12-31	2017-10-30
Diagnosis: ICD10 Code, J00-J99 Define	J00-J99	Diseases of the respiratory system	483214	Not well controlled asthma with status asthmaticus	ICD10CM	1	J45.902		Unspecified asthma with status asthmaticus	IMO	GHS-PACDC	IMO_2015_R	ACTIVE	N	1900-01-01	9999-12-31	2015-07-10
Diagnosis: ICD10 Code, J00-J99 Define	J00-J99	Diseases of the respiratory system	482507	Chronic asthma with status asthmaticus	ICD10CM	1	J45.902		Unspecified asthma with status asthmaticus	IMO	GHS-PACDC	IMO_2015_R	ACTIVE	N	1900-01-01	9999-12-31	2015-07-10
Diagnosis: ICD10 Code, J00-J99 Define	J00-J99	Diseases of the respiratory system	961522	Asthma with hay fever, unspecified asthma severity, with status asthmaticus	ICD10CM	1	J45.902		Unspecified asthma with status asthmaticus	IMO	GHS-PACDC	IMO_2015_R	ACTIVE	N	1900-01-01	9999-12-31	2015-07-10
Diagnosis: ICD10 Code, J00-J99 Define	J00-J99	Diseases of the respiratory system	236643	COPD with asthma and status asthmaticus (HCC)	ICD10CM	2	J45.902	J44.9,J45.902	Unspecified asthma with status asthmaticus	IMO	GHS-PACDC	DX_LEGACY	ACTIVE	N	2016-04-10	9999-12-31	2015-07-10
Diagnosis: ICD10 Code, J00-J99 Define	J00-J99	Diseases of the respiratory system	957148	RAD (reactive airway disease), unspecified asthma severity, with status asthmaticus	ICD10CM	1	J45.902		Unspecified asthma with status asthmaticus	IMO	GHS-PACDC	IMO_2015_R	ACTIVE	N	1900-01-01	9999-12-31	2015-07-10
Diagnosis: ICD10 Code, J00-J99 Define	J00-J99	Diseases of the respiratory system	1427186	Cold-induced asthma with status asthmaticus, unspecified asthma severity, unspecified whether	ICD10CM	1	J45.902		Unspecified asthma with status asthmaticus	IMO	GHS-PACDC	IMO_2018_R	ACTIVE	N	1900-01-01	9999-12-31	2017-10-30
Diagnosis: ICD10 Code, J00-J99 Define	J00-J99	Diseases of the respiratory system	960981	Late onset asthma, unspecified asthma severity, with status asthmaticus	ICD10CM	1	J45.902		Unspecified asthma with status asthmaticus	IMO	GHS-PACDC	IMO_2015_R	ACTIVE	N	1900-01-01	9999-12-31	2015-07-10
Diagnosis: ICD10 Code, J00-J99 Define	J00-J99	Diseases of the respiratory system	303812	Asthma, chronic obstructive, with status asthmaticus (HCC)	ICD10CM	2	J45.902	J44.9,J45.902	Unspecified asthma with status asthmaticus	IMO	GHS-PACDC	DX_LEGACY	ACTIVE	N	2016-04-10	9999-12-31	2015-07-10
Diagnosis: ICD10 Code, J00-J99 Define	J00-J99	Diseases of the respiratory system	955470	Occasional asthma, unspecified asthma severity, with status asthmaticus	ICD10CM	1	J45.902		Unspecified asthma with status asthmaticus	IMO	GHS-PACDC	IMO_2015_R	ACTIVE	N	1900-01-01	9999-12-31	2015-07-10

Diagnosis: ICD10 Code, J00-J99 Define	J00-J99	Diseases of the respiratory system	483455	Reactive airway disease with wheezing with status asthmaticus	ICD10CM	1	J45.902	J45.902,R06.2	Unspecified asthma with status asthmaticus	IMO	GHS-PACDC	IMO_2015_R	ACTIVE	N	1900-01-01	2016-10-14	2015-07-10
Diagnosis: ICD10 Code, J00-J99 Define	J00-J99	Diseases of the respiratory system	483180	Steroid-dependent asthma with status asthmaticus	ICD10CM	1	J45.902		Unspecified asthma with status asthmaticus	IMO	GHS-PACDC	IMO_2015_R	ACTIVE	N	1900-01-01	9999-12-31	2015-07-10
Diagnosis: ICD10 Code, J00-J99 Define	J00-J99	Diseases of the respiratory system	1349686	Mild asthma with status asthmaticus	ICD10CM	1	J45.902		Unspecified asthma with status asthmaticus	IMO	GHS-PACDC	IMO_2018_R	ACTIVE	N	1900-01-01	9999-12-31	2017-10-30
Diagnosis: ICD10 Code, J00-J99 Define	J00-J99	Diseases of the respiratory system	482626	Asthma with status asthmaticus in adult	ICD10CM	1	J45.902		Unspecified asthma with status asthmaticus	IMO	GHS-PACDC	IMO_2015_R	ACTIVE	N	1900-01-01	9999-12-31	2015-07-10
Diagnosis: ICD10 Code, J00-J99 Define	J00-J99	Diseases of the respiratory system	954667	Asthma with allergic rhinitis, unspecified asthma severity, with status asthmaticus	ICD10CM	1	J45.902		Unspecified asthma with status asthmaticus	IMO	GHS-PACDC	IMO_2015_R	ACTIVE	N	1900-01-01	9999-12-31	2015-07-10
Diagnosis: ICD10 Code, J00-J99 Define	J00-J99	Diseases of the respiratory system	962570	Allergy-induced asthma, unspecified asthma severity, uncomplicated	ICD10CM	1	J45.909		Unspecified asthma, uncomplicated	IMO	GHS-PACDC	IMO_2015_R	ACTIVE	N	1900-01-01	9999-12-31	2015-07-10
Diagnosis: ICD10 Code, J00-J99 Define	J00-J99	Diseases of the respiratory system	123501	Rapid onset asthma	ICD10CM	1	J45.909		Unspecified asthma, uncomplicated	IMO	GHS-PACDC	DX_LEGACY	ACTIVE	N	1900-01-01	9999-12-31	2015-07-10
Diagnosis: ICD10 Code, J00-J99 Define	J00-J99	Diseases of the respiratory system	957572	Asthma, stable, unspecified asthma severity	ICD10CM	1	J45.909		Unspecified asthma, uncomplicated	IMO	GHS-PACDC	IMO_2015_R	ACTIVE	N	1900-01-01	9999-12-31	2015-07-10
Diagnosis: ICD10 Code, J00-J99 Define	J00-J99	Diseases of the respiratory system	414246	Asthma in adult	ICD10CM	1	J45.909		Unspecified asthma, uncomplicated	IMO	GHS-PACDC	DX_LEGACY	ACTIVE	N	1900-01-01	9999-12-31	2015-07-10
Diagnosis: ICD10 Code, J00-J99 Define	J00-J99	Diseases of the respiratory system	236644	Asthma with bronchitis	ICD10CM	1	J45.909		Unspecified asthma, uncomplicated	IMO	GHS-PACDC	DX_LEGACY	ACTIVE	N	1900-01-01	9999-12-31	2015-07-10
Diagnosis: ICD10 Code, J00-J99 Define	J00-J99	Diseases of the respiratory system	1427202	Extrinsic asthma without status asthmaticus, unspecified asthma severity, unspecified whether	ICD10CM	1	J45.909		Unspecified asthma, uncomplicated	IMO	GHS-PACDC	IMO_2018_R	ACTIVE	N	1900-01-01	9999-12-31	2017-10-30
Diagnosis: ICD10 Code, J00-J99 Define	J00-J99	Diseases of the respiratory system	1202677	Asthma, non-allergic, unspecified asthma severity, uncomplicated	ICD10CM	1	J45.909		Unspecified asthma, uncomplicated	IMO	GHS-PACDC	IMO_2016_R	DELETED	Y	1900-01-01	9999-12-31	2016-06-02
Diagnosis: ICD10 Code, J00-J99 Define	J00-J99	Diseases of the respiratory system	505494	Asthma without status asthmaticus or acute exacerbation	ICD10CM	1	J45.909		Unspecified asthma, uncomplicated	IMO	GHS-PACDC	IMO_2015_R	ACTIVE	N	1900-01-01	9999-12-31	2015-07-10
Diagnosis: ICD10 Code, J00-J99 Define	J00-J99	Diseases of the respiratory system	137185	Unspecified asthma	ICD10CM	1	J45.909		Unspecified asthma, uncomplicated	IMO	GHS-PACDC	DX_LEGACY	ACTIVE	Y	1900-01-01	9999-12-31	2017-04-14
Diagnosis: ICD10 Code, J00-J99 Define	J00-J99	Diseases of the respiratory system	960296	Hay fever with asthma, unspecified asthma severity, uncomplicated	ICD10CM	1	J45.909		Unspecified asthma, uncomplicated	IMO	GHS-PACDC	IMO_2015_R	ACTIVE	N	1900-01-01	9999-12-31	2015-07-10
Diagnosis: ICD10 Code, J00-J99 Define	J00-J99	Diseases of the respiratory system	407608	Non-IgE mediated allergic asthma	ICD10CM	1	J45.909		Unspecified asthma, uncomplicated	IMO	GHS-PACDC	DX_LEGACY	ACTIVE	N	1900-01-01	9999-12-31	2015-07-10
Diagnosis: ICD10 Code, J00-J99 Define	J00-J99	Diseases of the respiratory system	1427217	Occasional asthma without complication, unspecified asthma severity, unspecified whether pers	ICD10CM	1	J45.909		Unspecified asthma, uncomplicated	IMO	GHS-PACDC	IMO_2018_R	ACTIVE	N	1900-01-01	9999-12-31	2017-10-30
Diagnosis: ICD10 Code, J00-J99 Define	J00-J99	Diseases of the respiratory system	1131951	Asthma not dependent on systemic steroids	ICD10CM	1	J45.909		Unspecified asthma, uncomplicated	IMO	GHS-PACDC	IMO_2016_R	ACTIVE	N	1900-01-01	9999-12-31	2016-06-02
Diagnosis: ICD10 Code, J00-J99 Define	J00-J99	Diseases of the respiratory system	429879	Nocturnal asthma symptoms nightly	ICD10CM	1	J45.998		Other asthma	IMO	GHS-PACDC	IMO_2015_R	ACTIVE	N	1900-01-01	9999-12-31	2015-07-10
Diagnosis: ICD10 Code, J00-J99 Define	J00-J99	Diseases of the respiratory system	159192	Printers' asthma	ICD10CM	1	J45.998		Other asthma	IMO	GHS-PACDC	DX_LEGACY	ACTIVE	N	1900-01-01	9999-12-31	2015-07-10
Diagnosis: ICD10 Code, J00-J99 Define	J00-J99	Diseases of the respiratory system	1427106	Mild asthma, unspecified whether complicated, unspecified whether persistent	ICD10CM	1	J45.998		Other asthma	IMO	GHS-PACDC	IMO_2018_R	ACTIVE	N	1900-01-01	9999-12-31	2017-10-30
Diagnosis: ICD10 Code, J00-J99 Define	J00-J99	Diseases of the respiratory system	133614	Nocturnal asthma	ICD10CM	1	J45.998		Other asthma	IMO	GHS-PACDC	DX_LEGACY	ACTIVE	N	1900-01-01	9999-12-31	2015-07-10
Diagnosis: ICD10 Code, J00-J99 Define	J00-J99	Diseases of the respiratory system	145883	Mounier-Kuhn syndrome with bronchiectasis with acute exacerbation (HCC)	ICD10CM	1	J47.1		Bronchiectasis with (acute) exacerbation	IMO	GHS-PACDC	DX_LEGACY	ACTIVE	N	1900-01-01	9999-12-31	2015-07-10
Diagnosis: ICD10 Code, J00-J99 Define	J00-J99	Diseases of the respiratory system	418329	Toxin-induced bronchiectasis (HCC)	ICD10CM	1	J47.9		Bronchiectasis, uncomplicated	IMO	GHS-PACDC	DX_LEGACY	ACTIVE	N	1900-01-01	9999-12-31	2015-07-10
Diagnosis: ICD10 Code, J00-J99 Define	J00-J99	Diseases of the respiratory system	132428	Bronchiolectasis (HCC)	ICD10CM	1	J47.9		Bronchiectasis, uncomplicated	IMO	GHS-PACDC	DX_LEGACY	ACTIVE	N	1900-01-01	9999-12-31	2015-07-10
Diagnosis: ICD10 Code, J00-J99 Define	J00-J99	Diseases of the respiratory system	95185	Black lung (HCC)	ICD10CM	1	J60		Coalworker's pneumoconiosis	IMO	GHS-PACDC	DX_LEGACY	ACTIVE	N	1900-01-01	9999-12-31	2015-07-10
Diagnosis: ICD10 Code, J00-J99 Define	J00-J99	Diseases of the respiratory system	113232	Pneumoconiosis due to talc dust (HCC)	ICD10CM	1	J62.0		Pneumoconiosis due to talc dust	IMO	GHS-PACDC	DX_LEGACY	ACTIVE	N	1900-01-01	9999-12-31	2015-07-10
Diagnosis: ICD10 Code, J00-J99 Define	J00-J99	Diseases of the respiratory system	21136	Siderosilicosis (HCC)	ICD10CM	1	J62.8		Pneumoconiosis due to other dust containing silica	IMO	GHS-PACDC	DX_LEGACY	ACTIVE	N	1900-01-01	9999-12-31	2015-07-10
Diagnosis: ICD10 Code, J00-J99 Define	J00-J99	Diseases of the respiratory system	21142	Hematite pneumoconiosis (HCC)	ICD10CM	1	J62.8		Pneumoconiosis due to other dust containing silica	IMO	GHS-PACDC	DX_LEGACY	ACTIVE	N	1900-01-01	9999-12-31	2015-07-10
Diagnosis: ICD10 Code, J00-J99 Define	J00-J99	Diseases of the respiratory system	109179	Hematite Miner's lung disease (HCC)	ICD10CM	1	J62.8		Pneumoconiosis due to other dust containing silica	IMO	GHS-PACDC	DX_LEGACY	ACTIVE	N	1900-01-01	9999-12-31	2015-07-10
Diagnosis: ICD10 Code, J00-J99 Define	J00-J99	Diseases of the respiratory system	21141	Hematite miners' lung disease (HCC)	ICD10CM	1	J62.8		Pneumoconiosis due to other dust containing silica	IMO	GHS-PACDC	DX_LEGACY	ACTIVE	N	1900-01-01	9999-12-31	2015-07-10
Diagnosis: ICD10 Code, J00-J99 Define	J00-J99	Diseases of the respiratory system	417819	Mica pneumoconiosis (HCC)	ICD10CM	1	J62.8		Pneumoconiosis due to other dust containing silica	IMO	GHS-PACDC	DX_LEGACY	ACTIVE	N	2016-04-10	9999-12-31	2015-07-10
Diagnosis: ICD10 Code, J00-J99 Define	J00-J99	Diseases of the respiratory system	91095	Pneumoconiosis due to silica or silicates (HCC)	ICD10CM	1	J62.8		Pneumoconiosis due to other dust containing silica	IMO	GHS-PACDC	DX_LEGACY	ACTIVE	N	1900-01-01	9999-12-31	2015-07-10
Diagnosis: ICD10 Code, J00-J99 Define	J00-J99	Diseases of the respiratory system	81711	Bauxite fume pneumoconiosis (HCC)	ICD10CM	1	J63.1		Bauxite fibrosis (of lung)	IMO	GHS-PACDC	DX_LEGACY	ACTIVE	N	1900-01-01	9999-12-31	2015-07-10
Diagnosis: ICD10 Code, J00-J99 Define	J00-J99	Diseases of the respiratory system	81736	Tin pneumoconiosis (HCC)	ICD10CM	1	J63.5		Stannosis	IMO	GHS-PACDC	DX_LEGACY	ACTIVE	N	1900-01-01	9999-12-31	2015-07-10
Diagnosis: ICD10 Code, J00-J99 Define	J00-J99	Diseases of the respiratory system	536679	Stannosis	ICD10CM	1	J63.5		Stannosis	IMO	GHS-PACDC	IMO_2015_R	ACTIVE	Y	1900-01-01	9999-12-31	2015-07-10
Diagnosis: ICD10 Code, J00-J99 Define	J00-J99	Diseases of the respiratory system	21177	Cobaltosis (HCC)	ICD10CM	1	J63.6		Pneumoconiosis due to other specified inorganic dusts	IMO	GHS-PACDC	DX_LEGACY	ACTIVE	N	1900-01-01	9999-12-31	2015-07-10
Diagnosis: ICD10 Code, J00-J99 Define	J00-J99	Diseases of the respiratory system	21145	Benign pneumoconiosis (HCC)	ICD10CM	1	J64		Unspecified pneumoconiosis	IMO	GHS-PACDC	DX_LEGACY	ACTIVE	N	1900-01-01	9999-12-31	2015-07-10
Diagnosis: ICD10 Code, J00-J99 Define	J00-J99	Diseases of the respiratory system	417627	Complicated pneumoconiosis (HCC)	ICD10CM	1	J64		Unspecified pneumoconiosis	IMO	GHS-PACDC	DX_LEGACY	ACTIVE	N	1900-01-01	9999-12-31	2015-07-10
Diagnosis: ICD10 Code, J00-J99 Define	J00-J99	Diseases of the respiratory system	21193	Flax-dressers' disease (HCC)	ICD10CM	1	J66.1		Flax-dressers' disease	IMO	GHS-PACDC	DX_LEGACY	ACTIVE	N	1900-01-01	9999-12-31	2015-07-10
Diagnosis: ICD10 Code, J00-J99 Define	J00-J99	Diseases of the respiratory system	21194	Strippers' disease (HCC)	ICD10CM	1	J66.1		Flax-dressers' disease	IMO	GHS-PACDC	DX_LEGACY	ACTIVE	N	1900-01-01	9999-12-31	2015-07-10
Diagnosis: ICD10 Code, J00-J99 Define	J00-J99	Diseases of the respiratory system	533638	Flax-dressers' disease	ICD10CM	1	J66.1		Flax-dressers' disease	IMO	GHS-PACDC	IMO_2015_R	ACTIVE	Y	1900-01-01	9999-12-31	2015-07-10
Diagnosis: ICD10 Code, J00-J99 Define	J00-J99	Diseases of the respiratory system	73601	Cannabinosis (HCC)	ICD10CM	1	J66.2		Cannabinosis	IMO	GHS-PACDC	DX_LEGACY	ACTIVE	N	1900-01-01	9999-12-31	2015-07-10
Diagnosis: ICD10 Code, J00-J99 Define	J00-J99	Diseases of the respiratory system	114489	Sisal-worker's disease (HCC)	ICD10CM	1	J66.8		Airway disease due to other specific organic dusts	IMO	GHS-PACDC	DX_LEGACY	ACTIVE	N	1900-01-01	9999-12-31	2015-07-10
Diagnosis: ICD10 Code, J00-J99 Define	J00-J99	Diseases of the respiratory system	21253	Cork-handlers' disease (HCC)	ICD10CM	1	J67.3		Suberosis	IMO	GHS-PACDC	DX_LEGACY	ACTIVE	N	1900-01-01	9999-12-31	2015-07-10
Diagnosis: ICD10 Code, J00-J99 Define	J00-J99	Diseases of the respiratory system	97528	Cork-handlers lung (HCC)	ICD10CM	1	J67.3		Suberosis	IMO	GHS-PACDC	DX_LEGACY	ACTIVE	N	1900-01-01	9999-12-31	2015-07-10
Diagnosis: ICD10 Code, J00-J99 Define	J00-J99	Diseases of the respiratory system	21256	Alveolitis due to aspergillus clavatus and fumigatus (HCC)	ICD10CM	1	J67.4		Maltworker's lung	IMO	GHS-PACDC	DX_LEGACY	ACTIVE	N	1900-01-01	9999-12-31	2015-07-10
Diagnosis: ICD10 Code, J00-J99 Define	J00-J99	Diseases of the respiratory system	21258	Malt house workers' cough (HCC)	ICD10CM	1	J67.4		Maltworker's lung	IMO	GHS-PACDC	DX_LEGACY	ACTIVE	N	1900-01-01	9999-12-31	2015-07-10
Diagnosis: ICD10 Code, J00-J99 Define	J00-J99	Diseases of the respiratory system	5494	Maple-bark strippers' lung (HCC)	ICD10CM	1	J67.6		Maple-bark-stripper's lung	IMO	GHS-PACDC	DX_LEGACY	ACTIVE	N	1900-01-01	9999-12-31	2015-07-10
Diagnosis: ICD10 Code, J00-J99 Define	J00-J99	Diseases of the respiratory system	62188	Humidifier and air-conditioner pneumonitis (HCC)	ICD10CM	1	J67.7		Air conditioner and humidifier lung	IMO	GHS-PACDC	DX_LEGACY	ACTIVE	N	1900-01-01	9999-12-31	2015-07-10
Diagnosis: ICD10 Code, J00-J99 Define	J00-J99	Diseases of the respiratory system	21285	Hairspray thesaurosis (HCC)	ICD10CM	1	J67.8		Hypersensitivity pneumonitis due to other organic dusts	IMO	GHS-PACDC	DX_LEGACY	ACTIVE	N	1900-01-01	9999-12-31	2015-07-10
Diagnosis: ICD10 Code, J00-J99 Define	J00-J99	Diseases of the respiratory system	5497	Allergic alveolitis and pneumonitis (HCC)	ICD10CM	1	J67.9		Hypersensitivity pneumonitis due to unspecified organic	IMO	GHS-PACDC	DX_LEGACY	ACTIVE	N	1900-01-01	9999-12-31	2015-07-10
Diagnosis: ICD10 Code, J00-J99 Define	J00-J99	Diseases of the respiratory system	77874	Extrinsic allergic alveolitis (HCC)	ICD10CM	1	J67.9		Hypersensitivity pneumonitis due to unspecified organic	IMO	GHS-PACDC	DX_LEGACY	ACTIVE	N	1900-01-01	9999-12-31	2015-07-10
Diagnosis: ICD10 Code, J00-J99 Define	J00-J99	Diseases of the respiratory system	42571	Alveolitis, allergic (HCC)	ICD10CM	1	J67.9		Hypersensitivity pneumonitis due to unspecified organic	IMO	GHS-PACDC	DX_LEGACY	ACTIVE	N	1900-01-01	9999-12-31	2015-07-10
Diagnosis: ICD10 Code, J00-J99 Define	J00-J99	Diseases of the respiratory system	123647	Acute chemical pneumonitis (HCC)	ICD10CM	1	J68.0		Bronchitis and pneumonitis due to chemicals, gases, fum	IMO	GHS-PACDC	DX_LEGACY	ACTIVE	N	1900-01-01	9999-12-31	2015-07-10
Diagnosis: ICD10 Code, J00-J99 Define	J00-J99	Diseases of the respiratory system	21217	Chemical pneumonia (HCC)	ICD10CM	1	J68.0		Bronchitis and pneumonitis due to chemicals, gases, fum	IMO	GHS-PACDC	DX_LEGACY	ACTIVE	N	1900-01-01	9999-12-31	2015-07-10
Diagnosis: ICD10 Code, J00-J99 Define	J00-J99	Diseases of the respiratory system	1366391	Oth ac & subac resp cond d/t chemicals, gas, fumes & vapors (HCC)	ICD10CM	1	J68.3		Other acute and subacute respiratory conditions due to	IMO	GHS-PACDC	IMO_2018_R	ACTIVE	N	1900-01-01	9999-12-31	2017-10-30
Diagnosis: ICD10 Code, J00-J99 Define	J00-J99	Diseases of the respiratory system	224996	Respiratory condition, acute/subacute, due to fumes and vapors	ICD10CM	1	J68.3	J68.3,T59.91XA	Other acute and subacute respiratory conditions due to	IMO	GHS-PACDC	DX_LEGACY	DELETED	Y	1900-01-01	2016-05-07	2015-07-10
Diagnosis: ICD10 Code, J00-J99 Define	J00-J99	Diseases of the respiratory system	235788	Bronchitis due to fumes or vapors, chronic (HCC)	ICD10CM	1	J68.4		Chronic respiratory conditions due to chemicals, gases, f	IMO	GHS-PACDC	DX_LEGACY	ACTIVE	N	1900-01-01	9999-12-31	2015-07-10
Diagnosis: ICD10 Code, J00-J99 Define	J00-J99	Diseases of the respiratory system	1237246	Aspiration pneumonia of left upper lobe, unspecified aspiration pneumonia type (HCC)	ICD10CM	1	J69.0		Pneumonitis due to inhalation of food and vomit	IMO	GHS-PACDC	IMO_2016_R	ACTIVE	N	1900-01-01	9999-12-31	2016-06-02
Diagnosis: ICD10 Code, J00-J99 Define	J00-J99	Diseases of the respiratory system	147183	Pneumonitis due to liquids (HCC)	ICD10CM	1	J69.0		Pneumonitis due to inhalation of food and vomit	IMO	GHS-PACDC	DX_LEGACY	ACTIVE	N	1900-01-01	2016-05-07	2015-07-10
Diagnosis: ICD10 Code, J00-J99 Define	J00-J99	Diseases of the respiratory system	1125250	Aspiration pneumonia of upper lobe due to milk (HCC)	ICD10CM	1	J69.0		Pneumonitis due to inhalation of food and vomit	IMO	GHS-PACDC	IMO_2016_R	ACTIVE	N	1900-01-01	9999-12-31	2016-06-02
Diagnosis: ICD10 Code, J00-J99 Define	J00-J99	Diseases of the respiratory system	1123883	Aspiration pneumonia of right lung due to milk (HCC)	ICD10CM	1	J69.0		Pneumonitis due to inhalation of food and vomit	IMO	GHS-PACDC	IMO_2016_R	ACTIVE	N	1900-01-01	9999-12-31	2016-06-02
Diagnosis: ICD10 Code, J00-J99 Define	J00-J99	Diseases of the respiratory system	1125256	Aspiration pneumonia of upper lobe due to gastric secretions (HCC)	ICD10CM	1	J69.0		Pneumonitis due to inhalation of food and vomit	IMO	GHS-PACDC	IMO_2016_R	ACTIVE	N	1900-01-01	9999-12-31	2016-06-02
Diagnosis: ICD10 Code, J00-J99 Define	J00-J99	Diseases of the respiratory system	1125257	Aspiration pneumonia of lower lobe due to milk (HCC)	ICD10CM	1	J69.0		Pneumonitis due to inhalation of food and vomit	IMO	GHS-PACDC	IMO_2016_R	ACTIVE	N	1900-01-01	9999-12-31	2016-06-02
Diagnosis: ICD10 Code, J00-J99 Define	J00-J99	Diseases of the respiratory system	116151	Vomit inhalation pneumonitis (HCC)	ICD10CM	1	J69.0		Pneumonitis due to inhalation of food and vomit	IMO	GHS-PACDC	DX_LEGACY	ACTIVE	N	1900-01-01	9999-12-31	2015-07-10
Diagnosis: ICD10 Code, J00-J99 Define	J00-J99	Diseases of the respiratory system	1237020	Aspiration pneumonia of upper lobe, unspecified aspiration pneumonia type, unspecified latera	ICD10CM	1	J69.0		Pneumonitis due to inhalation of food and vomit	IMO	GHS-PACDC	IMO_2016_R	ACTIVE	N	1900-01-01	9999-12-31	2016-06-02
Diagnosis: ICD10 Code, J00-J99 Define	J00-J99	Diseases of the respiratory system	5514	Pneumonitis due to inhalation of essences (HCC)	ICD10CM	1	J69.1		Pneumonitis due to inhalation of oils and essences	IMO	GHS-PACDC	DX_LEGACY	ACTIVE	N	1900-01-01	9999-12-31	2015-07-10
Diagnosis: ICD10 Code, J00-J99 Define	J00-J99	Diseases of the respiratory system	157422	Pneumonitis due to solids and liquids (HCC)	ICD10CM	1	J69.8		Pneumonitis due to inhalation of other solids and liquids	IMO	GHS-PACDC	DX_LEGACY	ACTIVE	N	1900-01-01	9999-12-31	2015-07-10
Diagnosis: ICD10 Code, J00-J99 Define	J00-J99	Diseases of the respiratory system	169748	Chronic drug-induced interstitial lung disorders (HCC)	ICD10CM	1	J70.3		Chronic drug-induced interstitial lung disorders	IMO	GHS-PACDC	DX_LEGACY	ACTIVE	N	1900-01-01	9999-12-31	2015-07-10
Diagnosis: ICD10 Code, J00-J99 Define	J00-J99	Diseases of the respiratory system	1058659	Pulmonary drug toxicity, accidental or unintentional, initial encounter	ICD10CM	2	J70.8	T50.901A,J70.8	Respiratory conditions due to other specified external a	IMO	GHS-PACDC	IMO_2015_R	ACTIVE	N	1900-01-01	2016-10-14	2015-07-10
Diagnosis: ICD10 Code, J00-J99 Define	J00-J99	Diseases of the respiratory system	327654	Pulmonary toxicity caused by Carmustine	ICD10CM	1	J70.8	J70.8,T45.1X5A	Respiratory conditions due to other specified external a	IMO	GHS-PACDC	DX_LEGACY	ACTIVE	N	1900-01-01	2016-10-14	2015-07-10
Diagnosis: ICD10 Code, J00-J99 Define	J00-J99	Diseases of the respiratory system	201721	Respiratory conditions due to external agent (HCC)	ICD10CM	1	J70.9		Respiratory conditions due to unspecified external agen	IMO	GHS-PACDC	DX_LEGACY	ACTIVE	N	1900-01-01	9999-12-31	2015-07-10
Diagnosis: ICD10 Code, J00-J99 Define	J00-J99	Diseases of the respiratory system	306377	Postimmersion-submersion syndrome (HCC)	ICD10CM	1	J80		Acute respiratory distress syndrome	IMO	GHS-PACDC	DX_LEGACY	ACTIVE	N	1900-01-01	9999-12-31	2015-07-10
Diagnosis: ICD10 Code, J00-J99 Define	J00-J99	Diseases of the respiratory system	537827	Acute respiratory distress syndrome	ICD10CM	1	J80		Acute respiratory distress syndrome	IMO	GHS-PACDC	IMO_2015_R	ACTIVE	Y	1900-01-01	9999-12-31	2015-07-10
Diagnosis: ICD10 Code, J00-J99 Define	J00-J99	Diseases of the respiratory system	306423	Adult RDS (HCC)	ICD10CM	1	J80		Acute respiratory distress syndrome	IMO	GHS-PACDC	DX_LEGACY	ACTIVE	N	1900-01-01	9999-12-31	2015-07-10
Diagnosis: ICD10 Code, J00-J99 Define	J00-J99	Diseases of the respiratory system	236629	Lung edema, acute (HCC)	ICD10CM	1	J81.0		Acute pulmonary edema	IMO	GHS-PACDC	DX_LEGACY	ACTIVE	N	1900-01-01	9999-12-31	2015-07-10
Diagnosis: ICD10 Code, J00-J99 Define	J00-J99	Diseases of the respiratory system	96102	Acute pulmonary edema syndrome (HCC)	ICD10CM	1	J81.0		Acute pulmonary edema	IMO	GHS-PACDC	DX_LEGACY	ACTIVE	N	1900-01-01	9999-12-31	2015-07-10
Diagnosis: ICD10 Code, J00-J99 Define	J00-J99	Diseases of the respiratory system	5536	Pulmonary congestion and hypostasis	ICD10CM	1	J81.1		Chronic pulmonary edema	IMO	GHS-PACDC	DX_LEGACY	ACTIVE	N	1900-01-01	9999-12-31	2015-07-10
Diagnosis: ICD10 Code, J00-J99 Define	J00-J99	Diseases of the respiratory system	233183	Immunosuppressive-induced fluid in lung	ICD10CM	1	J81.1		Chronic pulmonary edema	IMO	GHS-PACDC	DX_LEGACY	ACTIVE	N	1900-01-01	2017-10-14	2015-07-10
Diagnosis: ICD10 Code, J00-J99 Define	J00-J99	Diseases of the respiratory system	21327	Allergic pneumonia (HCC)	ICD10CM	1	J82		Pulmonary eosinophilia, not elsewhere classified	IMO	GHS-PACDC	DX_LEGACY	ACTIVE	N	1900-01-01	9999-12-31	2015-07-10
Diagnosis: ICD10 Code, J00-J99 Define	J00-J99	Diseases of the respiratory system	248704	Brown lung (HCC)	ICD10CM	2	J84.03	E83.10,J84.03	Idiopathic pulmonary hemosiderosis	IMO	GHS-PACDC	DX_LEGACY	ACTIVE	N	1900-01-01	2016-10-14	2015-07-10
Diagnosis: ICD10 Code, J00-J99 Define	J00-J99	Diseases of the respiratory system	248712	IPH (idiopathic pulmonary hemosiderosis) (HCC)	ICD10CM	2	J84.03	E83.10,J84.03	Idiopathic pulmonary hemosiderosis	IMO	GHS-PACDC	DX_LEGACY	ACTIVE	N	1900-01-01	2016-10-14	2015-07-10
Diagnosis: ICD10 Code, J00-J99 Define	J00-J99	Diseases of the respiratory system	312771	Alveolar capillary dysplasia (HCC)	ICD10CM	1	J84.09		Other alveolar and parieto-alveolar conditions	IMO	GHS-PACDC	DX_LEGACY	ACTIVE	N	1900-01-01	9999-12-31	2015-07-10
Diagnosis: ICD10 Code, J00-J99 Define	J00-J99	Diseases of the respiratory system	266988	Alveolar and parieto-alveolar condition (HCC)	ICD10CM	1	J84.09		Other alveolar and parieto-alveolar conditions	IMO	GHS-PACDC	DX_LEGACY	ACTIVE	N	1900-01-01	9999-12-31	2015-07-10
Diagnosis: ICD10 Code, J00-J99 Define	J00-J99	Diseases of the respiratory system	182938	Fibrosis, pulmonary, interstitial, diffuse (HCC)	ICD10CM	1	J84.10		Pulmonary fibrosis, unspecified	IMO	GHS-PACDC	DX_LEGACY	ACTIVE	N	1900-01-01	9999-12-31	2015-07-10
Diagnosis: ICD10 Code, J00-J99 Define	J00-J99	Diseases of the respiratory system	408658	Pulmonary tuberose sclerosis (HCC)	ICD10CM	1	J84.10		Pulmonary fibrosis, unspecified	IMO	GHS-PACDC	DX_LEGACY	ACTIVE	N	1900-01-01	9999-12-31	2015-07-10
Diagnosis: ICD10 Code, J00-J99 Define	J00-J99	Diseases of the respiratory system	21371	Peribronchial fibrosis of lung (HCC)	ICD10CM	1	J84.10		Pulmonary fibrosis, unspecified	IMO	GHS-PACDC	DX_LEGACY	ACTIVE	N	1900-01-01	9999-12-31	2015-07-10
Diagnosis: ICD10 Code, J00-J99 Define	J00-J99	Diseases of the respiratory system	80194	Lung fibrosis (HCC)	ICD10CM	1	J84.10		Pulmonary fibrosis, unspecified	IMO	GHS-PACDC	DX_LEGACY	ACTIVE	N	1900-01-01	9999-12-31	2015-07-10
Diagnosis: ICD10 Code, J00-J99 Define	J00-J99	Diseases of the respiratory system	314064	Fibrosing alveolitis (HCC)	ICD10CM	1	J84.112		Idiopathic pulmonary fibrosis	IMO	GHS-PACDC	DX_LEGACY	ACTIVE	N	1900-01-01	9999-12-31	2015-07-10
Diagnosis: ICD10 Code, J00-J99 Define	J00-J99	Diseases of the respiratory system	18971	Cryptogenic organizing pneumonia (HCC)	ICD10CM	1	J84.116		Cryptogenic organizing pneumonia	IMO	GHS-PACDC	DX_LEGACY	ACTIVE	N	1900-01-01	9999-12-31	2015-07-10
Diagnosis: ICD10 Code, J00-J99 Define	J00-J99	Diseases of the respiratory system	306439	Alveolar capillary block (HCC)	ICD10CM	1	J84.17		Other interstitial pulmonary diseases with fibrosis in dis	IMO	GHS-PACDC	DX_LEGACY	ACTIVE	N	1900-01-01	2016-10-14	2015-07-10
Diagnosis: ICD10 Code, J00-J99 Define	J00-J99	Diseases of the respiratory system	18976	Pulmonary interstitial glycogenosis (HCC)	ICD10CM	1	J84.842		Pulmonary interstitial glycogenosis	IMO	GHS-PACDC	DX_LEGACY	ACTIVE	N	1900-01-01	9999-12-31	2015-07-10
Diagnosis: ICD10 Code, J00-J99 Define	J00-J99	Diseases of the respiratory system	312742	Interstitial lung diseases of childhood (HCC)	ICD10CM	1	J84.848		Other interstitial lung diseases of childhood	IMO	GHS-PACDC	DX_LEGACY	ACTIVE	N	1900-01-01	9999-12-31	2015-07-10
Diagnosis: ICD10 Code, J00-J99 Define	J00-J99	Diseases of the respiratory system	609079	Other specified interstitial pulmonary diseases	ICD10CM	1	J84.89		Other specified interstitial pulmonary diseases	IMO	GHS-PACDC	IMO_2015_R	ACTIVE	Y	1900-01-01	9999-12-31	2015-07-10

Diagnosis: ICD10 Code, J00-J99 Define	J00-J99	Diseases of the respiratory system	156637	Nonspecific interstitial pneumonia (HCC)	ICD10CM	1	J84.89		Other specified interstitial pulmonary diseases	IMO	GHS-PACDC	DX_LEGACY	ACTIVE	N	1900-01-01	9999-12-31	2015-07-10
Diagnosis: ICD10 Code, J00-J99 Define	J00-J99	Diseases of the respiratory system	81478	Chronic interstitial pneumonia (HCC)	ICD10CM	1	J84.9		Interstitial pulmonary disease, unspecified	IMO	GHS-PACDC	DX_LEGACY	ACTIVE	N	1900-01-01	2016-05-07	2015-07-10
Diagnosis: ICD10 Code, J00-J99 Define	J00-J99	Diseases of the respiratory system	21331	Parietoalveolar pneumopathy (HCC)	ICD10CM	1	J84.9		Interstitial pulmonary disease, unspecified	IMO	GHS-PACDC	DX_LEGACY	ACTIVE	N	1900-01-01	2016-05-07	2015-07-10
Diagnosis: ICD10 Code, J00-J99 Define	J00-J99	Diseases of the respiratory system	686830	Cyclophosphamide-induced pulmonary interstitial fibrosis, sequela	ICD10CM	2	J84.9	T45.1X5S,J84.9	Interstitial pulmonary disease, unspecified	IMO	GHS-PACDC	IMO_2015_R	DELETED	Y	1900-01-01	9999-12-31	2015-07-10
Diagnosis: ICD10 Code, J00-J99 Define	J00-J99	Diseases of the respiratory system	65330	Pneumonia, interstitial (HCC)	ICD10CM	1	J84.9		Interstitial pulmonary disease, unspecified	IMO	GHS-PACDC	DX_LEGACY	ACTIVE	N	1900-01-01	9999-12-31	2015-07-10
Diagnosis: ICD10 Code, J00-J99 Define	J00-J99	Diseases of the respiratory system	21348	Necrotic pneumonia (HCC)	ICD10CM	1	J85.0		Gangrene and necrosis of lung	IMO	GHS-PACDC	DX_LEGACY	ACTIVE	N	1900-01-01	9999-12-31	2015-07-10
Diagnosis: ICD10 Code, J00-J99 Define	J00-J99	Diseases of the respiratory system	1109932	Abscess of left lung with pneumonia (HCC)	ICD10CM	1	J85.1		Abscess of lung with pneumonia	IMO	GHS-PACDC	IMO_2016_R	ACTIVE	N	1900-01-01	9999-12-31	2016-06-02
Diagnosis: ICD10 Code, J00-J99 Define	J00-J99	Diseases of the respiratory system	57485	Esophagotracheal fistula (HCC)	ICD10CM	1	J86.0		Pyothorax with fistula	IMO	GHS-PACDC	DX_LEGACY	ACTIVE	N	2016-04-10	9999-12-31	2015-07-10
Diagnosis: ICD10 Code, J00-J99 Define	J00-J99	Diseases of the respiratory system	113793	Pyothorax with bronchocutaneous fistula (HCC)	ICD10CM	1	J86.0		Pyothorax with fistula	IMO	GHS-PACDC	DX_LEGACY	ACTIVE	N	1900-01-01	9999-12-31	2015-07-10
Diagnosis: ICD10 Code, J00-J99 Define	J00-J99	Diseases of the respiratory system	67279	Chest wall fistula (HCC)	ICD10CM	1	J86.0		Pyothorax with fistula	IMO	GHS-PACDC	DX_LEGACY	ACTIVE	N	1900-01-01	9999-12-31	2015-07-10
Diagnosis: ICD10 Code, J00-J99 Define	J00-J99	Diseases of the respiratory system	251888	Tracheoesophageal fistula, acquired (HCC)	ICD10CM	1	J86.0		Pyothorax with fistula	IMO	GHS-PACDC	DX_LEGACY	ACTIVE	N	2016-04-10	9999-12-31	2015-07-10
Diagnosis: ICD10 Code, J00-J99 Define	J00-J99	Diseases of the respiratory system	101744	Pyothorax with fistula (HCC)	ICD10CM	1	J86.0		Pyothorax with fistula	IMO	GHS-PACDC	DX_LEGACY	ACTIVE	N	1900-01-01	9999-12-31	2015-07-10
Diagnosis: ICD10 Code, J00-J99 Define	J00-J99	Diseases of the respiratory system	46579	Pleuritis, purulent (HCC)	ICD10CM	1	J86.9		Pyothorax without fistula	IMO	GHS-PACDC	DX_LEGACY	ACTIVE	N	1900-01-01	9999-12-31	2015-07-10
Diagnosis: ICD10 Code, J00-J99 Define	J00-J99	Diseases of the respiratory system	1260708	Empyema of pleural space without fistula (HCC)	ICD10CM	1	J86.9		Pyothorax without fistula	IMO	GHS-PACDC	IMO_2017_R	ACTIVE	N	1900-01-01	9999-12-31	2016-09-30
Diagnosis: ICD10 Code, J00-J99 Define	J00-J99	Diseases of the respiratory system	21407	Fibrinopurulent pleurisy (HCC)	ICD10CM	1	J86.9		Pyothorax without fistula	IMO	GHS-PACDC	DX_LEGACY	ACTIVE	N	1900-01-01	9999-12-31	2015-07-10
Diagnosis: ICD10 Code, J00-J99 Define	J00-J99	Diseases of the respiratory system	1260705	Empyema of pleural space (HCC)	ICD10CM	1	J86.9		Pyothorax without fistula	IMO	GHS-PACDC	IMO_2017_R	ACTIVE	N	1900-01-01	9999-12-31	2016-09-30
Diagnosis: ICD10 Code, J00-J99 Define	J00-J99	Diseases of the respiratory system	208836	Fluid in pleural cavity associated with pancreatitis	ICD10CM	1	J90	J90,K85.90	Pleural effusion, not elsewhere classified	IMO	GHS-PACDC	DX_LEGACY	ACTIVE	N	2016-09-17	9999-12-31	2016-09-30
Diagnosis: ICD10 Code, J00-J99 Define	J00-J99	Diseases of the respiratory system	328694	Recurrent right pleural effusion	ICD10CM	1	J90		Pleural effusion, not elsewhere classified	IMO	GHS-PACDC	DX_LEGACY	ACTIVE	N	1900-01-01	9999-12-31	2015-07-10
Diagnosis: ICD10 Code, J00-J99 Define	J00-J99	Diseases of the respiratory system	774233	Ovarian fibroma with ascites and pleural effusion, unspecified laterality	ICD10CM	2	J90	D27.9,J90,R18.8	Pleural effusion, not elsewhere classified	IMO	GHS-PACDC	IMO_2015_R	ACTIVE	N	2016-04-10	9999-12-31	2015-07-10
Diagnosis: ICD10 Code, J00-J99 Define	J00-J99	Diseases of the respiratory system	189563	Pleural effusion associated with pancreatitis	ICD10CM	1	J90	J90,K85.90	Pleural effusion, not elsewhere classified	IMO	GHS-PACDC	DX_LEGACY	ACTIVE	N	2016-09-17	9999-12-31	2016-09-30
Diagnosis: ICD10 Code, J00-J99 Define	J00-J99	Diseases of the respiratory system	36204	Streptococcal pleurisy with effusion	ICD10CM	1	J90		Pleural effusion, not elsewhere classified	IMO	GHS-PACDC	DX_LEGACY	ACTIVE	N	1900-01-01	9999-12-31	2015-07-10
Diagnosis: ICD10 Code, J00-J99 Define	J00-J99	Diseases of the respiratory system	373297	Loculated pleural effusion	ICD10CM	1	J90		Pleural effusion, not elsewhere classified	IMO	GHS-PACDC	DX_LEGACY	ACTIVE	N	1900-01-01	9999-12-31	2015-07-10
Diagnosis: ICD10 Code, J00-J99 Define	J00-J99	Diseases of the respiratory system	741221	Meigs syndrome, unspecified laterality	ICD10CM	3	J90	D27.9,R18.8,J90	Pleural effusion, not elsewhere classified	IMO	GHS-PACDC	IMO_2015_R	ACTIVE	N	2016-04-10	9999-12-31	2015-07-10
Diagnosis: ICD10 Code, J00-J99 Define	J00-J99	Diseases of the respiratory system	208836	Fluid in pleural cavity associated with pancreatitis	ICD10CM	2	J91.8	K85.9,J91.8	Pleural effusion in other conditions classified elsewhere	IMO	GHS-PACDC	DX_LEGACY	ACTIVE	N	2016-04-10	2016-10-14	2015-07-10
Diagnosis: ICD10 Code, J00-J99 Define	J00-J99	Diseases of the respiratory system	1124163	Calcified pleural plaque due to asbestos exposure	ICD10CM	1	J92.0		Pleural plaque with presence of asbestos	IMO	GHS-PACDC	IMO_2016_R	ACTIVE	N	1900-01-01	9999-12-31	2016-06-02
Diagnosis: ICD10 Code, J00-J99 Define	J00-J99	Diseases of the respiratory system	113208	Pleural fibrosis	ICD10CM	1	J92.9		Pleural plaque without asbestos	IMO	GHS-PACDC	DX_LEGACY	ACTIVE	N	2016-04-10	9999-12-31	2015-07-10
Diagnosis: ICD10 Code, J00-J99 Define	J00-J99	Diseases of the respiratory system	533651	Spontaneous tension pneumothorax	ICD10CM	1	J93.0		Spontaneous tension pneumothorax	IMO	GHS-PACDC	IMO_2015_R	ACTIVE	Y	1900-01-01	9999-12-31	2015-07-10
Diagnosis: ICD10 Code, J00-J99 Define	J00-J99	Diseases of the respiratory system	18960	Primary spontaneous pneumothorax	ICD10CM	1	J93.11		Primary spontaneous pneumothorax	IMO	GHS-PACDC	DX_LEGACY	ACTIVE	N	1900-01-01	9999-12-31	2015-07-10
Diagnosis: ICD10 Code, J00-J99 Define	J00-J99	Diseases of the respiratory system	306472	Acute pneumothorax	ICD10CM	1	J93.83		Other pneumothorax	IMO	GHS-PACDC	DX_LEGACY	ACTIVE	N	1900-01-01	9999-12-31	2015-07-10
Diagnosis: ICD10 Code, J00-J99 Define	J00-J99	Diseases of the respiratory system	325985	Pneumothorax, open	ICD10CM	1	J93.83		Other pneumothorax	IMO	GHS-PACDC	DX_LEGACY	ACTIVE	N	1900-01-01	9999-12-31	2015-07-10
Diagnosis: ICD10 Code, J00-J99 Define	J00-J99	Diseases of the respiratory system	325984	Pneumothorax, closed	ICD10CM	1	J93.9		Pneumothorax, unspecified	IMO	GHS-PACDC	DX_LEGACY	ACTIVE	N	1900-01-01	9999-12-31	2015-07-10
Diagnosis: ICD10 Code, J00-J99 Define	J00-J99	Diseases of the respiratory system	539452	Pneumothorax, unspecified	ICD10CM	1	J93.9		Pneumothorax, unspecified	IMO	GHS-PACDC	IMO_2015_R	ACTIVE	Y	1900-01-01	9999-12-31	2015-07-10
Diagnosis: ICD10 Code, J00-J99 Define	J00-J99	Diseases of the respiratory system	21390	Thickening of pleura	ICD10CM	1	J94.1		Fibrothorax	IMO	GHS-PACDC	DX_LEGACY	ACTIVE	N	1900-01-01	2016-05-07	2015-07-10
Diagnosis: ICD10 Code, J00-J99 Define	J00-J99	Diseases of the respiratory system	229424	Hemopneumothorax, right	ICD10CM	1	J94.2		Hemothorax	IMO	GHS-PACDC	DX_LEGACY	ACTIVE	N	1900-01-01	9999-12-31	2015-07-10
Diagnosis: ICD10 Code, J00-J99 Define	J00-J99	Diseases of the respiratory system	189565	Pleural effusion associated with pulmonary infection	ICD10CM	1	J94.8		Other specified pleural conditions	IMO	GHS-PACDC	DX_LEGACY	ACTIVE	N	1900-01-01	2016-05-07	2015-07-10
Diagnosis: ICD10 Code, J00-J99 Define	J00-J99	Diseases of the respiratory system	182951	Fetal pleural effusion	ICD10CM	1	J94.8		Other specified pleural conditions	IMO	GHS-PACDC	DX_LEGACY	DELETED	Y	1900-01-01	2016-05-07	2015-07-10
Diagnosis: ICD10 Code, J00-J99 Define	J00-J99	Diseases of the respiratory system	166501	Pleural scarring	ICD10CM	1	J94.8		Other specified pleural conditions	IMO	GHS-PACDC	DX_LEGACY	ACTIVE	N	2016-04-10	9999-12-31	2015-07-10
Diagnosis: ICD10 Code, J00-J99 Define	J00-J99	Diseases of the respiratory system	162815	Disorder of pleura	ICD10CM	1	J94.9		Pleural condition, unspecified	IMO	GHS-PACDC	DX_LEGACY	ACTIVE	N	1900-01-01	9999-12-31	2015-07-10
Diagnosis: ICD10 Code, J00-J99 Define	J00-J99	Diseases of the respiratory system	1138071	Pleural condition, unspecified	ICD10CM	1	J94.9		Pleural condition, unspecified	IMO	GHS-PACDC	IMO_2016_R	DELETED	Y	1900-01-01	9999-12-31	2016-06-02
Diagnosis: ICD10 Code, J00-J99 Define	J00-J99	Diseases of the respiratory system	93267	Tracheostomy complication, unspecified	ICD10CM	1	J95.00		Unspecified tracheostomy complication	IMO	GHS-PACDC	DX_LEGACY	ACTIVE	Y	1900-01-01	9999-12-31	2015-07-10
Diagnosis: ICD10 Code, J00-J99 Define	J00-J99	Diseases of the respiratory system	20999	Tracheal stenosis following tracheostomy (HCC)	ICD10CM	1	J95.02		Infection of tracheostomy stoma	IMO	GHS-PACDC	DX_LEGACY	ACTIVE	N	1900-01-01	2016-05-07	2015-07-10
Diagnosis: ICD10 Code, J00-J99 Define	J00-J99	Diseases of the respiratory system	15454	Other tracheostomy complication (HCC)	ICD10CM	1	J95.09		Other tracheostomy complication	IMO	GHS-PACDC	DX_LEGACY	ACTIVE	N	1900-01-01	9999-12-31	2015-07-10
Diagnosis: ICD10 Code, J00-J99 Define	J00-J99	Diseases of the respiratory system	594352	Acute pulmonary insufficiency following thoracic surgery	ICD10CM	1	J95.1		Acute pulmonary insufficiency following thoracic surgery	IMO	GHS-PACDC	IMO_2015_R	ACTIVE	Y	1900-01-01	9999-12-31	2015-07-10
Diagnosis: ICD10 Code, J00-J99 Define	J00-J99	Diseases of the respiratory system	603971	Chemical pneumonitis due to anesthesia	ICD10CM	1	J95.4		Chemical pneumonitis due to anesthesia	IMO	GHS-PACDC	IMO_2015_R	ACTIVE	Y	1900-01-01	9999-12-31	2015-07-10
Diagnosis: ICD10 Code, J00-J99 Define	J00-J99	Diseases of the respiratory system	523897	Intraoperative hematoma involving respiratory system complicating non-respiratory system pro	ICD10CM	1	J95.62		Intraoperative hemorrhage and hematoma of a respirat	IMO	GHS-PACDC	IMO_2015_R	ACTIVE	N	1900-01-01	9999-12-31	2015-07-10
Diagnosis: ICD10 Code, J00-J99 Define	J00-J99	Diseases of the respiratory system	5531	Iatrogenic pneumothorax	ICD10CM	1	J95.811		Postprocedural pneumothorax	IMO	GHS-PACDC	DX_LEGACY	ACTIVE	N	1900-01-01	9999-12-31	2015-07-10
Diagnosis: ICD10 Code, J00-J99 Define	J00-J99	Diseases of the respiratory system	589625	Postprocedural respiratory failure	ICD10CM	1	J95.82		Postprocedural respiratory failure	IMO	GHS-PACDC	IMO_2015_R	ACTIVE	Y	1900-01-01	9999-12-31	2015-07-10
Diagnosis: ICD10 Code, J00-J99 Define	J00-J99	Diseases of the respiratory system	494122	Acute on chronic postprocedural respiratory failure (HCC)	ICD10CM	1	J95.822		Acute and chronic postprocedural respiratory failure	IMO	GHS-PACDC	IMO_2015_R	ACTIVE	N	1900-01-01	9999-12-31	2015-07-10
Diagnosis: ICD10 Code, J00-J99 Define	J00-J99	Diseases of the respiratory system	1351527	Traumatic hemorrhage of trachea in newborn after procedure on lower respiratory tract	ICD10CM	2	J95.830	P96.89,J95.830	Postprocedural hemorrhage of a respiratory system orga	IMO	GHS-PACDC	IMO_2018_R	ACTIVE	N	1900-01-01	9999-12-31	2017-10-30
Diagnosis: ICD10 Code, J00-J99 Define	J00-J99	Diseases of the respiratory system	603539	Postprocedural hemorrhage of a respiratory system organ or structure following a respiratory s	ICD10CM	1	J95.830		Postprocedural hemorrhage of a respiratory system orga	IMO	GHS-PACDC	IMO_2015_R	ACTIVE	Y	1900-01-01	9999-12-31	2015-07-10
Diagnosis: ICD10 Code, J00-J99 Define	J00-J99	Diseases of the respiratory system	524155	Postoperative hemorrhage involving respiratory system following non-respiratory system proce	ICD10CM	1	J95.831		Postprocedural hemorrhage of a respiratory system orga	IMO	GHS-PACDC	IMO_2015_R	ACTIVE	N	2016-04-10	9999-12-31	2015-07-10
Diagnosis: ICD10 Code, J00-J99 Define	J00-J99	Diseases of the respiratory system	144918	Mech comp respirator (HCC)	ICD10CM	1	J95.850		Mechanical complication of respirator	IMO	GHS-PACDC	DX_LEGACY	ACTIVE	N	1900-01-01	9999-12-31	2015-07-10
Diagnosis: ICD10 Code, J00-J99 Define	J00-J99	Diseases of the respiratory system	1275362	Postprocedural hematoma of a respiratory system organ or structure following a respiratory sys	ICD10CM	1	J95.860		Postprocedural hematoma of a respiratory system organ	IMO	GHS-PACDC	IMO_2017_R	ACTIVE	Y	1900-01-01	9999-12-31	2017-04-14
Diagnosis: ICD10 Code, J00-J99 Define	J00-J99	Diseases of the respiratory system	1293275	Postprocedural seroma of a respiratory system organ or structure following a respiratory system	ICD10CM	1	J95.862		Postprocedural seroma of a respiratory system organ or	IMO	GHS-PACDC	IMO_2017_R	ACTIVE	N	1900-01-01	9999-12-31	2016-09-30
Diagnosis: ICD10 Code, J00-J99 Define	J00-J99	Diseases of the respiratory system	1293266	Postprocedural seroma of a respiratory system organ or structure following other procedure	ICD10CM	1	J95.863		Postprocedural seroma of a respiratory system organ or	IMO	GHS-PACDC	IMO_2017_R	ACTIVE	N	1900-01-01	9999-12-31	2016-09-30
Diagnosis: ICD10 Code, J00-J99 Define	J00-J99	Diseases of the respiratory system	1240231	Postoperative surgical complication involving respiratory system associated with respiratory sys	ICD10CM	1	J95.89		Other postprocedural complications and disorders of res	IMO	GHS-PACDC	IMO_2016_R	ACTIVE	N	1900-01-01	9999-12-31	2016-06-02
Diagnosis: ICD10 Code, J00-J99 Define	J00-J99	Diseases of the respiratory system	1238055	Postoperative surgical complication involving respiratory system associated with non-respirator	ICD10CM	1	J95.89		Other postprocedural complications and disorders of res	IMO	GHS-PACDC	IMO_2016_R	ACTIVE	N	1900-01-01	9999-12-31	2016-06-02
Diagnosis: ICD10 Code, J00-J99 Define	J00-J99	Diseases of the respiratory system	306424	Respiratory insufficiency following shock, trauma, or surgery	ICD10CM	1	J95.89		Other postprocedural complications and disorders of res	IMO	GHS-PACDC	DX_LEGACY	DELETED	Y	2016-04-10	2016-10-14	2015-07-10
Diagnosis: ICD10 Code, J00-J99 Define	J00-J99	Diseases of the respiratory system	379491	Insufficient treatment with nasal CPAP	ICD10CM	1	J95.89		Other postprocedural complications and disorders of res	IMO	GHS-PACDC	DX_LEGACY	ACTIVE	N	1900-01-01	2016-10-14	2015-07-10
Diagnosis: ICD10 Code, J00-J99 Define	J00-J99	Diseases of the respiratory system	615427	Respiratory failure, not elsewhere classified	ICD10CM	1	J96		Respiratory failure, not elsewhere classified	IMO	GHS-PACDC	IMO_2015_R	ACTIVE	Y	1900-01-01	9999-12-31	2015-07-10
Diagnosis: ICD10 Code, J00-J99 Define	J00-J99	Diseases of the respiratory system	505011	Acute respiratory failure with hypoxemia (HCC)	ICD10CM	1	J96.01		Acute respiratory failure with hypoxia	IMO	GHS-PACDC	IMO_2015_R	ACTIVE	N	1900-01-01	9999-12-31	2015-07-10
Diagnosis: ICD10 Code, J00-J99 Define	J00-J99	Diseases of the respiratory system	496469	Acute respiratory failure with hypoxia and hypercarbia (HCC)	ICD10CM	1	J96.01	J96.01,J96.02	Acute respiratory failure with hypoxia	IMO	GHS-PACDC	IMO_2015_R	ACTIVE	N	2016-04-10	9999-12-31	2015-07-10
Diagnosis: ICD10 Code, J00-J99 Define	J00-J99	Diseases of the respiratory system	1111024	Chronic respiratory failure with hypoxia and hypercapnia (HCC)	ICD10CM	1	J96.11	J96.11,J96.12	Chronic respiratory failure with hypoxia	IMO	GHS-PACDC	IMO_2016_R	ACTIVE	N	2016-09-17	9999-12-31	2016-09-30
Diagnosis: ICD10 Code, J00-J99 Define	J00-J99	Diseases of the respiratory system	1111024	Chronic respiratory failure with hypoxia and hypercapnia (HCC)	ICD10CM	2	J96.12	J96.11,J96.12	Chronic respiratory failure with hypercapnia	IMO	GHS-PACDC	IMO_2016_R	ACTIVE	N	2016-09-17	9999-12-31	2016-09-30
Diagnosis: ICD10 Code, J00-J99 Define	J00-J99	Diseases of the respiratory system	1106165	Chronic type 2 respiratory failure (HCC)	ICD10CM	1	J96.12		Chronic respiratory failure with hypercapnia	IMO	GHS-PACDC	IMO_2016_R	ACTIVE	N	1900-01-01	9999-12-31	2016-06-02
Diagnosis: ICD10 Code, J00-J99 Define	J00-J99	Diseases of the respiratory system	167151	Respiratory failure, acute neuromuscular (HCC)	ICD10CM	1	J96.90	J96.90,G70.9	Respiratory failure, unspecified, unspecified whether wi	IMO	GHS-PACDC	DX_LEGACY	ACTIVE	N	1900-01-01	9999-12-31	2015-07-10
Diagnosis: ICD10 Code, J00-J99 Define	J00-J99	Diseases of the respiratory system	1260547	Respiratory failure after trauma (HCC)	ICD10CM	1	J96.90		Respiratory failure, unspecified, unspecified whether wi	IMO	GHS-PACDC	IMO_2017_R	ACTIVE	N	2017-09-17	9999-12-31	2017-10-30
Diagnosis: ICD10 Code, J00-J99 Define	J00-J99	Diseases of the respiratory system	1180208	Respiratory failure, unspecified, unspecified whether with hypoxia or hypercapnia (HCC)	ICD10CM	1	J96.90		Respiratory failure, unspecified, unspecified whether wi	IMO	GHS-PACDC	IMO_2016_R	ACTIVE	N	1900-01-01	9999-12-31	2016-06-02
Diagnosis: ICD10 Code, J00-J99 Define	J00-J99	Diseases of the respiratory system	201347	Sleep-related respiratory failure (HCC)	ICD10CM	1	J96.90		Respiratory failure, unspecified, unspecified whether wi	IMO	GHS-PACDC	DX_LEGACY	ACTIVE	N	1900-01-01	9999-12-31	2015-07-10
Diagnosis: ICD10 Code, J00-J99 Define	J00-J99	Diseases of the respiratory system	147667	Type I respiratory failure (HCC)	ICD10CM	1	J96.90		Respiratory failure, unspecified, unspecified whether wi	IMO	GHS-PACDC	DX_LEGACY	ACTIVE	N	1900-01-01	9999-12-31	2015-07-10
Diagnosis: ICD10 Code, J00-J99 Define	J00-J99	Diseases of the respiratory system	596276	Respiratory failure, unspecified, unspecified whether with hypoxia or hypercapnia	ICD10CM	1	J96.90		Respiratory failure, unspecified, unspecified whether wi	IMO	GHS-PACDC	IMO_2015_R	ACTIVE	Y	1900-01-01	9999-12-31	2015-07-10
Diagnosis: ICD10 Code, J00-J99 Define	J00-J99	Diseases of the respiratory system	147666	Type 2 respiratory failure (HCC)	ICD10CM	1	J96.92		Respiratory failure, unspecified with hypercapnia	IMO	GHS-PACDC	DX_LEGACY	ACTIVE	N	2016-04-10	9999-12-31	2015-07-10
Diagnosis: ICD10 Code, J00-J99 Define	J00-J99	Diseases of the respiratory system	1114501	Hypercapnic respiratory failure (HCC)	ICD10CM	1	J96.92		Respiratory failure, unspecified with hypercapnia	IMO	GHS-PACDC	IMO_2016_R	ACTIVE	N	1900-01-01	9999-12-31	2016-06-02
Diagnosis: ICD10 Code, J00-J99 Define	J00-J99	Diseases of the respiratory system	379187	Cough due to bronchospasm	ICD10CM	1	J98.01		Acute bronchospasm	IMO	GHS-PACDC	DX_LEGACY	ACTIVE	N	1900-01-01	9999-12-31	2015-07-10
Diagnosis: ICD10 Code, J00-J99 Define	J00-J99	Diseases of the respiratory system	229085	Bronchial stenosis, left	ICD10CM	1	J98.09		Other diseases of bronchus, not elsewhere classified	IMO	GHS-PACDC	DX_LEGACY	ACTIVE	N	1900-01-01	9999-12-31	2015-07-10
Diagnosis: ICD10 Code, J00-J99 Define	J00-J99	Diseases of the respiratory system	230464	Bronchial stenosis, right	ICD10CM	1	J98.09		Other diseases of bronchus, not elsewhere classified	IMO	GHS-PACDC	DX_LEGACY	ACTIVE	N	1900-01-01	9999-12-31	2015-07-10
Diagnosis: ICD10 Code, J00-J99 Define	J00-J99	Diseases of the respiratory system	514586	Lithoptysis	ICD10CM	1	J98.09		Other diseases of bronchus, not elsewhere classified	IMO	GHS-PACDC	IMO_2015_R	ACTIVE	N	1900-01-01	9999-12-31	2015-07-10
Diagnosis: ICD10 Code, J00-J99 Define	J00-J99	Diseases of the respiratory system	229035	Atelectasis of left lung	ICD10CM	1	J98.11		Atelectasis	IMO	GHS-PACDC	DX_LEGACY	ACTIVE	N	1900-01-01	9999-12-31	2015-07-10
Diagnosis: ICD10 Code, J00-J99 Define	J00-J99	Diseases of the respiratory system	503049	Mild bibasilar atelectasis	ICD10CM	1	J98.11		Atelectasis	IMO	GHS-PACDC	IMO_2015_R	ACTIVE	N	1900-01-01	9999-12-31	2015-07-10
Diagnosis: ICD10 Code, J00-J99 Define	J00-J99	Diseases of the respiratory system	229038	Atelectasis of right lung	ICD10CM	1	J98.11		Atelectasis	IMO	GHS-PACDC	DX_LEGACY	ACTIVE	N	1900-01-01	9999-12-31	2015-07-10
Diagnosis: ICD10 Code, J00-J99 Define	J00-J99	Diseases of the respiratory system	21324	Brock syndrome	ICD10CM	1	J98.19		Other pulmonary collapse	IMO	GHS-PACDC	DX_LEGACY	ACTIVE	N	1900-01-01	9999-12-31	2015-07-10
Diagnosis: ICD10 Code, J00-J99 Define	J00-J99	Diseases of the respiratory system	255558	Trapped lung	ICD10CM	1	J98.19		Other pulmonary collapse	IMO	GHS-PACDC	DX_LEGACY	ACTIVE	N	1900-01-01	9999-12-31	2015-07-10
Diagnosis: ICD10 Code, J00-J99 Define	J00-J99	Diseases of the respiratory system	254535	Nontraumatic subcutaneous emphysema (HCC)	ICD10CM	1	J98.2		Interstitial emphysema	IMO	GHS-PACDC	DX_LEGACY	ACTIVE	N	1900-01-01	9999-12-31	2015-07-10
Diagnosis: ICD10 Code, J00-J99 Define	J00-J99	Diseases of the respiratory system	327068	Cavitating mass in right lower lung lobe	ICD10CM	1	J98.4		Other disorders of lung	IMO	GHS-PACDC	DX_LEGACY	ACTIVE	N	1900-01-01	9999-12-31	2015-07-10
Diagnosis: ICD10 Code, J00-J99 Define	J00-J99	Diseases of the respiratory system	44119	Bronchogenic cyst	ICD10CM	1	J98.4		Other disorders of lung	IMO	GHS-PACDC	DX_LEGACY	ACTIVE	N	2016-04-10	9999-12-31	2015-07-10
Diagnosis: ICD10 Code, J00-J99 Define	J00-J99	Diseases of the respiratory system	1114318	Restrictive lung mechanics due to neuromuscular disease (HCC)	ICD10CM	1	J98.4	J98.4,G70.9	Other disorders of lung	IMO	GHS-PACDC	IMO_2016_R	ACTIVE	N	1900-01-01	9999-12-31	2016-06-02
Diagnosis: ICD10 Code, J00-J99 Define	J00-J99	Diseases of the respiratory system	254965	Pulmonary toxicity due to bleomycin	ICD10CM	1	J98.4	J98.4,T45.1X5A	Other disorders of lung	IMO	GHS-PACDC	DX_LEGACY	ACTIVE	N	2016-09-17	9999-12-31	2016-09-30
Diagnosis: ICD10 Code, J00-J99 Define	J00-J99	Diseases of the respiratory system	258771	Sleep-related hypoventilation due to pulmonary parenchymal pathology	ICD10CM	1	J98.4	J98.4,G47.36	Other disorders of lung	IMO	GHS-PACDC	DX_LEGACY	ACTIVE	N	1900-01-01	9999-12-31	2015-07-10
Diagnosis: ICD10 Code, J00-J99 Define	J00-J99	Diseases of the respiratory system	208791	Narrowed leaky pulmonary heart valve	ICD10CM	1	J98.4	J98.4,Q25.6	Other disorders of lung	IMO	GHS-PACDC	DX_LEGACY	ACTIVE	N	1900-01-01	9999-12-31	2015-07-10
Diagnosis: ICD10 Code, J00-J99 Define	J00-J99	Diseases of the respiratory system	1052894	Pulmonary drug toxicity, accidental or unintentional, sequela	ICD10CM	2	J98.4	T50.901S,J98.4	Other disorders of lung	IMO	GHS-PACDC	IMO_2015_R	ACTIVE	N	2016-09-17	9999-12-31	2016-09-30
Diagnosis: ICD10 Code, J00-J99 Define	J00-J99	Diseases of the respiratory system	105625	Ciliary dyskinesia	ICD10CM	1	J98.4		Other disorders of lung	IMO	GHS-PACDC	DX_LEGACY	ACTIVE	N	1900-01-01	9999-12-31	2015-07-10
Diagnosis: ICD10 Code, J00-J99 Define	J00-J99	Diseases of the respiratory system	110147	Inflammatory pseudotumors, lung	ICD10CM	1	J98.4		Other disorders of lung	IMO	GHS-PACDC	DX_LEGACY	ACTIVE	N	1900-01-01	9999-12-31	2015-07-10
Diagnosis: ICD10 Code, J00-J99 Define	J00-J99	Diseases of the respiratory system	501830	Lung cyst	ICD10CM	1	J98.4		Other disorders of lung	IMO	GHS-PACDC	IMO_2015_R	ACTIVE	N	1900-01-01	9999-12-31	2015-07-10
Diagnosis: ICD10 Code, J00-J99 Define	J00-J99	Diseases of the respiratory system	111300	Mediastinal fibrosis	ICD10CM	1	J98.5		Diseases of mediastinum, not elsewhere classified	IMO	GHS-PACDC	DX_LEGACY	ACTIVE	N	1900-01-01	2016-10-14	2015-07-10
Diagnosis: ICD10 Code, J00-J99 Define	J00-J99	Diseases of the respiratory system	5563	Mediastinitis	ICD10CM	1	J98.5		Diseases of mediastinum, not elsewhere classified	IMO	GHS-PACDC	DX_LEGACY	ACTIVE	N	1900-01-01	2016-10-14	2015-07-10

Diagnosis: ICD10 Code, J00-J99 Define	J00-J99	Diseases of the respiratory system	205497	Scarring of mediastinum	ICD10CM	1	J98.59		Other diseases of mediastinum, not elsewhere classified	IMO	GHS-PACDC	DX_LEGACY	ACTIVE	N	2016-09-17	9999-12-31	2016-09-30
Diagnosis: ICD10 Code, J00-J99 Define	J00-J99	Diseases of the respiratory system	107518	Embarrassed airway	ICD10CM	1	J98.8		Other specified respiratory disorders	IMO	GHS-PACDC	DX_LEGACY	ACTIVE	N	1900-01-01	9999-12-31	2015-07-10
Diagnosis: ICD10 Code, J00-J99 Define	J00-J99	Diseases of the respiratory system	258751	Viral respiratory illness	ICD10CM	1	J98.8	J98.8,B97.89	Other specified respiratory disorders	IMO	GHS-PACDC	DX_LEGACY	ACTIVE	N	2016-04-10	9999-12-31	2015-07-10
Diagnosis: ICD10 Code, J00-J99 Define	J00-J99	Diseases of the respiratory system	258750	Viral respiratory infection	ICD10CM	1	J98.8	J98.8,B97.89	Other specified respiratory disorders	IMO	GHS-PACDC	DX_LEGACY	ACTIVE	N	2016-04-10	9999-12-31	2015-07-10
Diagnosis: ICD10 Code, J00-J99 Define	J00-J99	Diseases of the respiratory system	147073	Reversible airways disease	ICD10CM	1	J98.8		Other specified respiratory disorders	IMO	GHS-PACDC	DX_LEGACY	ACTIVE	N	1900-01-01	9999-12-31	2015-07-10
Diagnosis: ICD10 Code, J00-J99 Define	J00-J99	Diseases of the respiratory system	417582	Bacterial respiratory infection	ICD10CM	1	J98.8		Other specified respiratory disorders	IMO	GHS-PACDC	DX_LEGACY	ACTIVE	N	1900-01-01	9999-12-31	2015-07-10
Diagnosis: ICD10 Code, J00-J99 Define	J00-J99	Diseases of the respiratory system	1107609	Surgical complication involving respiratory system associated with thoracic procedure	ICD10CM	1	J98.9		Respiratory disorder, unspecified	IMO	GHS-PACDC	IMO_2016_R	ACTIVE	N	1900-01-01	9999-12-31	2016-06-02
Diagnosis: ICD10 Code, J00-J99 Define	J00-J99	Diseases of the respiratory system	1107536	Surgical complication involving respiratory system associated with respiratory system procedure	ICD10CM	1	J98.9		Respiratory disorder, unspecified	IMO	GHS-PACDC	IMO_2016_R	ACTIVE	N	1900-01-01	9999-12-31	2016-06-02
Diagnosis: ICD10 Code, J00-J99 Define	J00-J99	Diseases of the respiratory system	1084722	Allergic disorder of respiratory system, initial encounter	ICD10CM	2	J98.9	T78.40XA,J98.9	Respiratory disorder, unspecified	IMO	GHS-PACDC	IMO_2015_R	ACTIVE	N	2016-09-17	9999-12-31	2016-09-30
Diagnosis: ICD10 Code, J00-J99 Define	J00-J99	Diseases of the respiratory system	379578	Respiratory illness with fever	ICD10CM	1	J98.9	J98.9,R50.9	Respiratory disorder, unspecified	IMO	GHS-PACDC	DX_LEGACY	ACTIVE	N	2016-04-10	9999-12-31	2015-07-10
Diagnosis: ICD10 Code, J00-J99 Define	J00-J99	Diseases of the respiratory system	422974	Respiratory complications	ICD10CM	1	J98.9		Respiratory disorder, unspecified	IMO	GHS-PACDC	IMO_2015_R	DELETED	Y	1900-01-01	9999-12-31	2015-07-10
Diagnosis: ICD10 Code, J00-J99 Define	J00-J99	Diseases of the respiratory system	1274197	Diphtheria of respiratory system (HCC)	ICD10CM	2	J99	A36.89,J99	Respiratory disorders in diseases classified elsewhere	IMO	GHS-PACDC	IMO_2017_R	ACTIVE	N	1900-01-01	9999-12-31	2016-09-30

EDG to ICD codes

Chart Category	edg	icd10	icd10_desc
Diagnosis: ICD10 Code, J00-J99 Define	103525	J00	Acute nasopharyngitis [common cold]
Diagnosis: ICD10 Code, J00-J99 Define	105768	J00	Acute nasopharyngitis [common cold]
Diagnosis: ICD10 Code, J00-J99 Define	111838	J00	Acute nasopharyngitis [common cold]
Diagnosis: ICD10 Code, J00-J99 Define	111839	J00	Acute nasopharyngitis [common cold]
Diagnosis: ICD10 Code, J00-J99 Define	1137866	J00	Acute nasopharyngitis [common cold]
Diagnosis: ICD10 Code, J00-J99 Define	123795	J00	Acute nasopharyngitis [common cold]
Diagnosis: ICD10 Code, J00-J99 Define	125620	J00	Acute nasopharyngitis [common cold]
Diagnosis: ICD10 Code, J00-J99 Define	1404672	J00	Acute nasopharyngitis [common cold]
Diagnosis: ICD10 Code, J00-J99 Define	1425367	J00	Acute nasopharyngitis [common cold]
Diagnosis: ICD10 Code, J00-J99 Define	20923	J00	Acute nasopharyngitis [common cold]
Diagnosis: ICD10 Code, J00-J99 Define	20994	J00	Acute nasopharyngitis [common cold]
Diagnosis: ICD10 Code, J00-J99 Define	21003	J00	Acute nasopharyngitis [common cold]
Diagnosis: ICD10 Code, J00-J99 Define	21004	J00	Acute nasopharyngitis [common cold]
Diagnosis: ICD10 Code, J00-J99 Define	236425	J00	Acute nasopharyngitis [common cold]
Diagnosis: ICD10 Code, J00-J99 Define	242934	J00	Acute nasopharyngitis [common cold]
Diagnosis: ICD10 Code, J00-J99 Define	242935	J00	Acute nasopharyngitis [common cold]
Diagnosis: ICD10 Code, J00-J99 Define	242936	J00	Acute nasopharyngitis [common cold]
Diagnosis: ICD10 Code, J00-J99 Define	256484	J00	Acute nasopharyngitis [common cold]
Diagnosis: ICD10 Code, J00-J99 Define	256485	J00	Acute nasopharyngitis [common cold]
Diagnosis: ICD10 Code, J00-J99 Define	265346	J00	Acute nasopharyngitis [common cold]
Diagnosis: ICD10 Code, J00-J99 Define	36117	J00	Acute nasopharyngitis [common cold]
Diagnosis: ICD10 Code, J00-J99 Define	37095	J00	Acute nasopharyngitis [common cold]
Diagnosis: ICD10 Code, J00-J99 Define	37096	J00	Acute nasopharyngitis [common cold]
Diagnosis: ICD10 Code, J00-J99 Define	37098	J00	Acute nasopharyngitis [common cold]
Diagnosis: ICD10 Code, J00-J99 Define	37099	J00	Acute nasopharyngitis [common cold]
Diagnosis: ICD10 Code, J00-J99 Define	417056	J00	Acute nasopharyngitis [common cold]
Diagnosis: ICD10 Code, J00-J99 Define	5331	J00	Acute nasopharyngitis [common cold]
Diagnosis: ICD10 Code, J00-J99 Define	5461	J00	Acute nasopharyngitis [common cold]
Diagnosis: ICD10 Code, J00-J99 Define	73516	J00	Acute nasopharyngitis [common cold]
Diagnosis: ICD10 Code, J00-J99 Define	73517	J00	Acute nasopharyngitis [common cold]
Diagnosis: ICD10 Code, J00-J99 Define	75809	J00	Acute nasopharyngitis [common cold]
Diagnosis: ICD10 Code, J00-J99 Define	78389	J00	Acute nasopharyngitis [common cold]
Diagnosis: ICD10 Code, J00-J99 Define	78394	J00	Acute nasopharyngitis [common cold]
Diagnosis: ICD10 Code, J00-J99 Define	79758	J00	Acute nasopharyngitis [common cold]
Diagnosis: ICD10 Code, J00-J99 Define	80710	J00	Acute nasopharyngitis [common cold]
Diagnosis: ICD10 Code, J00-J99 Define	80874	J00	Acute nasopharyngitis [common cold]
Diagnosis: ICD10 Code, J00-J99 Define	81322	J00	Acute nasopharyngitis [common cold]
Diagnosis: ICD10 Code, J00-J99 Define	504748	J01.80	Other acute sinusitis
Diagnosis: ICD10 Code, J00-J99 Define	134625	J02.8	Acute pharyngitis due to other specified organisms
Diagnosis: ICD10 Code, J00-J99 Define	163324	J02.8	Acute pharyngitis due to other specified organisms
Diagnosis: ICD10 Code, J00-J99 Define	215159	J02.8	Acute pharyngitis due to other specified organisms
Diagnosis: ICD10 Code, J00-J99 Define	36695	J02.8	Acute pharyngitis due to other specified organisms
Diagnosis: ICD10 Code, J00-J99 Define	73522	J02.8	Acute pharyngitis due to other specified organisms
Diagnosis: ICD10 Code, J00-J99 Define	1097628	J02.9	Acute pharyngitis, unspecified
Diagnosis: ICD10 Code, J00-J99 Define	1111267	J02.9	Acute pharyngitis, unspecified
Diagnosis: ICD10 Code, J00-J99 Define	1127838	J02.9	Acute pharyngitis, unspecified
Diagnosis: ICD10 Code, J00-J99 Define	1137623	J02.9	Acute pharyngitis, unspecified
Diagnosis: ICD10 Code, J00-J99 Define	1224981	J02.9	Acute pharyngitis, unspecified
Diagnosis: ICD10 Code, J00-J99 Define	1247180	J02.9	Acute pharyngitis, unspecified
Diagnosis: ICD10 Code, J00-J99 Define	134055	J02.9	Acute pharyngitis, unspecified
Diagnosis: ICD10 Code, J00-J99 Define	134625	J02.9	Acute pharyngitis, unspecified
Diagnosis: ICD10 Code, J00-J99 Define	163324	J02.9	Acute pharyngitis, unspecified
Diagnosis: ICD10 Code, J00-J99 Define	193538	J02.9	Acute pharyngitis, unspecified
Diagnosis: ICD10 Code, J00-J99 Define	20933	J02.9	Acute pharyngitis, unspecified
Diagnosis: ICD10 Code, J00-J99 Define	20935	J02.9	Acute pharyngitis, unspecified
Diagnosis: ICD10 Code, J00-J99 Define	20937	J02.9	Acute pharyngitis, unspecified
Diagnosis: ICD10 Code, J00-J99 Define	215159	J02.9	Acute pharyngitis, unspecified
Diagnosis: ICD10 Code, J00-J99 Define	228489	J02.9	Acute pharyngitis, unspecified
Diagnosis: ICD10 Code, J00-J99 Define	258215	J02.9	Acute pharyngitis, unspecified
Diagnosis: ICD10 Code, J00-J99 Define	266027	J02.9	Acute pharyngitis, unspecified
Diagnosis: ICD10 Code, J00-J99 Define	300945	J02.9	Acute pharyngitis, unspecified
Diagnosis: ICD10 Code, J00-J99 Define	36695	J02.9	Acute pharyngitis, unspecified
Diagnosis: ICD10 Code, J00-J99 Define	405737	J02.9	Acute pharyngitis, unspecified
Diagnosis: ICD10 Code, J00-J99 Define	408585	J02.9	Acute pharyngitis, unspecified
Diagnosis: ICD10 Code, J00-J99 Define	416559	J02.9	Acute pharyngitis, unspecified
Diagnosis: ICD10 Code, J00-J99 Define	528230	J02.9	Acute pharyngitis, unspecified
Diagnosis: ICD10 Code, J00-J99 Define	5339	J02.9	Acute pharyngitis, unspecified
Diagnosis: ICD10 Code, J00-J99 Define	73520	J02.9	Acute pharyngitis, unspecified
Diagnosis: ICD10 Code, J00-J99 Define	73521	J02.9	Acute pharyngitis, unspecified
Diagnosis: ICD10 Code, J00-J99 Define	73522	J02.9	Acute pharyngitis, unspecified
Diagnosis: ICD10 Code, J00-J99 Define	73525	J02.9	Acute pharyngitis, unspecified
Diagnosis: ICD10 Code, J00-J99 Define	73526	J02.9	Acute pharyngitis, unspecified
Diagnosis: ICD10 Code, J00-J99 Define	73527	J02.9	Acute pharyngitis, unspecified
Diagnosis: ICD10 Code, J00-J99 Define	79272	J02.9	Acute pharyngitis, unspecified
Diagnosis: ICD10 Code, J00-J99 Define	79997	J02.9	Acute pharyngitis, unspecified
Diagnosis: ICD10 Code, J00-J99 Define	79998	J02.9	Acute pharyngitis, unspecified
Diagnosis: ICD10 Code, J00-J99 Define	81631	J02.9	Acute pharyngitis, unspecified
Diagnosis: ICD10 Code, J00-J99 Define	81636	J02.9	Acute pharyngitis, unspecified
Diagnosis: ICD10 Code, J00-J99 Define	95863	J02.9	Acute pharyngitis, unspecified
Diagnosis: ICD10 Code, J00-J99 Define	36680	J03.80	Acute tonsillitis due to other specified organisms

Diagnosis: ICD10 Code, J00-J99 Define	73536	J03.80	Acute tonsillitis due to other specified organisms
Diagnosis: ICD10 Code, J00-J99 Define	245674	J03.90	Acute tonsillitis, unspecified
Diagnosis: ICD10 Code, J00-J99 Define	367300	J03.90	Acute tonsillitis, unspecified
Diagnosis: ICD10 Code, J00-J99 Define	73536	J03.90	Acute tonsillitis, unspecified
Diagnosis: ICD10 Code, J00-J99 Define	135075	J04.0	Acute laryngitis
Diagnosis: ICD10 Code, J00-J99 Define	135076	J04.0	Acute laryngitis
Diagnosis: ICD10 Code, J00-J99 Define	135077	J04.0	Acute laryngitis
Diagnosis: ICD10 Code, J00-J99 Define	135084	J04.0	Acute laryngitis
Diagnosis: ICD10 Code, J00-J99 Define	135088	J04.0	Acute laryngitis
Diagnosis: ICD10 Code, J00-J99 Define	135090	J04.0	Acute laryngitis
Diagnosis: ICD10 Code, J00-J99 Define	135095	J04.0	Acute laryngitis
Diagnosis: ICD10 Code, J00-J99 Define	135096	J04.0	Acute laryngitis
Diagnosis: ICD10 Code, J00-J99 Define	15943	J04.0	Acute laryngitis
Diagnosis: ICD10 Code, J00-J99 Define	15944	J04.0	Acute laryngitis
Diagnosis: ICD10 Code, J00-J99 Define	205673	J04.0	Acute laryngitis
Diagnosis: ICD10 Code, J00-J99 Define	235620	J04.0	Acute laryngitis
Diagnosis: ICD10 Code, J00-J99 Define	265348	J04.0	Acute laryngitis
Diagnosis: ICD10 Code, J00-J99 Define	330573	J04.0	Acute laryngitis
Diagnosis: ICD10 Code, J00-J99 Define	417058	J04.0	Acute laryngitis
Diagnosis: ICD10 Code, J00-J99 Define	417203	J04.0	Acute laryngitis
Diagnosis: ICD10 Code, J00-J99 Define	1111238	J04.10	Acute tracheitis without obstruction
Diagnosis: ICD10 Code, J00-J99 Define	123645	J04.10	Acute tracheitis without obstruction
Diagnosis: ICD10 Code, J00-J99 Define	123704	J04.10	Acute tracheitis without obstruction
Diagnosis: ICD10 Code, J00-J99 Define	135570	J04.10	Acute tracheitis without obstruction
Diagnosis: ICD10 Code, J00-J99 Define	182112	J04.10	Acute tracheitis without obstruction
Diagnosis: ICD10 Code, J00-J99 Define	182113	J04.10	Acute tracheitis without obstruction
Diagnosis: ICD10 Code, J00-J99 Define	20989	J04.10	Acute tracheitis without obstruction
Diagnosis: ICD10 Code, J00-J99 Define	20991	J04.10	Acute tracheitis without obstruction
Diagnosis: ICD10 Code, J00-J99 Define	330784	J04.10	Acute tracheitis without obstruction
Diagnosis: ICD10 Code, J00-J99 Define	36681	J04.10	Acute tracheitis without obstruction
Diagnosis: ICD10 Code, J00-J99 Define	5344	J04.10	Acute tracheitis without obstruction
Diagnosis: ICD10 Code, J00-J99 Define	57481	J04.10	Acute tracheitis without obstruction
Diagnosis: ICD10 Code, J00-J99 Define	102682	J04.11	Acute tracheitis with obstruction
Diagnosis: ICD10 Code, J00-J99 Define	1111201	J04.11	Acute tracheitis with obstruction
Diagnosis: ICD10 Code, J00-J99 Define	5345	J04.11	Acute tracheitis with obstruction
Diagnosis: ICD10 Code, J00-J99 Define	135560	J04.2	Acute laryngotracheitis
Diagnosis: ICD10 Code, J00-J99 Define	149350	J04.2	Acute laryngotracheitis
Diagnosis: ICD10 Code, J00-J99 Define	20958	J04.2	Acute laryngotracheitis
Diagnosis: ICD10 Code, J00-J99 Define	20959	J04.2	Acute laryngotracheitis
Diagnosis: ICD10 Code, J00-J99 Define	231260	J04.2	Acute laryngotracheitis
Diagnosis: ICD10 Code, J00-J99 Define	235621	J04.2	Acute laryngotracheitis
Diagnosis: ICD10 Code, J00-J99 Define	259511	J04.2	Acute laryngotracheitis
Diagnosis: ICD10 Code, J00-J99 Define	50877	J04.2	Acute laryngotracheitis
Diagnosis: ICD10 Code, J00-J99 Define	5347	J04.2	Acute laryngotracheitis
Diagnosis: ICD10 Code, J00-J99 Define	5348	J04.2	Acute laryngotracheitis
Diagnosis: ICD10 Code, J00-J99 Define	62109	J04.2	Acute laryngotracheitis
Diagnosis: ICD10 Code, J00-J99 Define	1111229	J04.30	Supraglottitis, unspecified, without obstruction
Diagnosis: ICD10 Code, J00-J99 Define	135106	J04.30	Supraglottitis, unspecified, without obstruction
Diagnosis: ICD10 Code, J00-J99 Define	242300	J04.30	Supraglottitis, unspecified, without obstruction
Diagnosis: ICD10 Code, J00-J99 Define	252006	J04.30	Supraglottitis, unspecified, without obstruction
Diagnosis: ICD10 Code, J00-J99 Define	532902	J04.30	Supraglottitis, unspecified, without obstruction
Diagnosis: ICD10 Code, J00-J99 Define	490641	J04.31	Supraglottitis, unspecified, with obstruction
Diagnosis: ICD10 Code, J00-J99 Define	124581	J05.0	Acute obstructive laryngitis [croup]
Diagnosis: ICD10 Code, J00-J99 Define	15944	J05.0	Acute obstructive laryngitis [croup]
Diagnosis: ICD10 Code, J00-J99 Define	415879	J05.0	Acute obstructive laryngitis [croup]
Diagnosis: ICD10 Code, J00-J99 Define	5348	J05.0	Acute obstructive laryngitis [croup]
Diagnosis: ICD10 Code, J00-J99 Define	62109	J05.0	Acute obstructive laryngitis [croup]
Diagnosis: ICD10 Code, J00-J99 Define	1273216	J06.9	Acute upper respiratory infection, unspecified
Diagnosis: ICD10 Code, J00-J99 Define	143434	J06.9	Acute upper respiratory infection, unspecified
Diagnosis: ICD10 Code, J00-J99 Define	143436	J06.9	Acute upper respiratory infection, unspecified
Diagnosis: ICD10 Code, J00-J99 Define	143438	J06.9	Acute upper respiratory infection, unspecified
Diagnosis: ICD10 Code, J00-J99 Define	162846	J06.9	Acute upper respiratory infection, unspecified
Diagnosis: ICD10 Code, J00-J99 Define	259315	J06.9	Acute upper respiratory infection, unspecified
Diagnosis: ICD10 Code, J00-J99 Define	259317	J06.9	Acute upper respiratory infection, unspecified
Diagnosis: ICD10 Code, J00-J99 Define	300950	J06.9	Acute upper respiratory infection, unspecified
Diagnosis: ICD10 Code, J00-J99 Define	300951	J06.9	Acute upper respiratory infection, unspecified
Diagnosis: ICD10 Code, J00-J99 Define	405696	J06.9	Acute upper respiratory infection, unspecified
Diagnosis: ICD10 Code, J00-J99 Define	423568	J06.9	Acute upper respiratory infection, unspecified
Diagnosis: ICD10 Code, J00-J99 Define	1114377	J09.X9	Influenza due to identified novel influenza A virus with other manifestations
Diagnosis: ICD10 Code, J00-J99 Define	110153	J11.08	Influenza due to unidentified influenza virus with specified pneumonia
Diagnosis: ICD10 Code, J00-J99 Define	110157	J11.08	Influenza due to unidentified influenza virus with specified pneumonia
Diagnosis: ICD10 Code, J00-J99 Define	36925	J11.08	Influenza due to unidentified influenza virus with specified pneumonia
Diagnosis: ICD10 Code, J00-J99 Define	248905	J11.1	Influenza due to unidentified influenza virus with other respiratory manifestations
Diagnosis: ICD10 Code, J00-J99 Define	248908	J11.1	Influenza due to unidentified influenza virus with other respiratory manifestations
Diagnosis: ICD10 Code, J00-J99 Define	315330	J11.1	Influenza due to unidentified influenza virus with other respiratory manifestations
Diagnosis: ICD10 Code, J00-J99 Define	1192545	J12.89	Other viral pneumonia
Diagnosis: ICD10 Code, J00-J99 Define	5426	J12.89	Other viral pneumonia
Diagnosis: ICD10 Code, J00-J99 Define	5426	J12.9	Viral pneumonia, unspecified
Diagnosis: ICD10 Code, J00-J99 Define	1107869	J18.1	Lobar pneumonia, unspecified organism
Diagnosis: ICD10 Code, J00-J99 Define	1107895	J18.1	Lobar pneumonia, unspecified organism
Diagnosis: ICD10 Code, J00-J99 Define	1107925	J18.1	Lobar pneumonia, unspecified organism
Diagnosis: ICD10 Code, J00-J99 Define	1107950	J18.1	Lobar pneumonia, unspecified organism
Diagnosis: ICD10 Code, J00-J99 Define	1107993	J18.1	Lobar pneumonia, unspecified organism
Diagnosis: ICD10 Code, J00-J99 Define	1108023	J18.1	Lobar pneumonia, unspecified organism
Diagnosis: ICD10 Code, J00-J99 Define	1239578	J18.1	Lobar pneumonia, unspecified organism
Diagnosis: ICD10 Code, J00-J99 Define	1239588	J18.1	Lobar pneumonia, unspecified organism
Diagnosis: ICD10 Code, J00-J99 Define	202870	J18.1	Lobar pneumonia, unspecified organism
Diagnosis: ICD10 Code, J00-J99 Define	202871	J18.1	Lobar pneumonia, unspecified organism
Diagnosis: ICD10 Code, J00-J99 Define	202872	J18.1	Lobar pneumonia, unspecified organism
Diagnosis: ICD10 Code, J00-J99 Define	202873	J18.1	Lobar pneumonia, unspecified organism
Diagnosis: ICD10 Code, J00-J99 Define	300897	J18.1	Lobar pneumonia, unspecified organism
Diagnosis: ICD10 Code, J00-J99 Define	300930	J18.1	Lobar pneumonia, unspecified organism
Diagnosis: ICD10 Code, J00-J99 Define	300931	J18.1	Lobar pneumonia, unspecified organism
Diagnosis: ICD10 Code, J00-J99 Define	300934	J18.1	Lobar pneumonia, unspecified organism
Diagnosis: ICD10 Code, J00-J99 Define	328293	J18.1	Lobar pneumonia, unspecified organism

Diagnosis: ICD10 Code, J00-J99 Define	496635	J18.1	Lobar pneumonia, unspecified organism
Diagnosis: ICD10 Code, J00-J99 Define	641272	J18.8	Other pneumonia, unspecified organism
Diagnosis: ICD10 Code, J00-J99 Define	1039558	J18.9	Pneumonia, unspecified organism
Diagnosis: ICD10 Code, J00-J99 Define	1039644	J18.9	Pneumonia, unspecified organism
Diagnosis: ICD10 Code, J00-J99 Define	1090222	J18.9	Pneumonia, unspecified organism
Diagnosis: ICD10 Code, J00-J99 Define	1090236	J18.9	Pneumonia, unspecified organism
Diagnosis: ICD10 Code, J00-J99 Define	1107855	J18.9	Pneumonia, unspecified organism
Diagnosis: ICD10 Code, J00-J99 Define	1107869	J18.9	Pneumonia, unspecified organism
Diagnosis: ICD10 Code, J00-J99 Define	1107895	J18.9	Pneumonia, unspecified organism
Diagnosis: ICD10 Code, J00-J99 Define	1107925	J18.9	Pneumonia, unspecified organism
Diagnosis: ICD10 Code, J00-J99 Define	1107937	J18.9	Pneumonia, unspecified organism
Diagnosis: ICD10 Code, J00-J99 Define	1107944	J18.9	Pneumonia, unspecified organism
Diagnosis: ICD10 Code, J00-J99 Define	1107950	J18.9	Pneumonia, unspecified organism
Diagnosis: ICD10 Code, J00-J99 Define	1107973	J18.9	Pneumonia, unspecified organism
Diagnosis: ICD10 Code, J00-J99 Define	1107993	J18.9	Pneumonia, unspecified organism
Diagnosis: ICD10 Code, J00-J99 Define	1107994	J18.9	Pneumonia, unspecified organism
Diagnosis: ICD10 Code, J00-J99 Define	1108023	J18.9	Pneumonia, unspecified organism
Diagnosis: ICD10 Code, J00-J99 Define	1113767	J18.9	Pneumonia, unspecified organism
Diagnosis: ICD10 Code, J00-J99 Define	1134919	J18.9	Pneumonia, unspecified organism
Diagnosis: ICD10 Code, J00-J99 Define	1186711	J18.9	Pneumonia, unspecified organism
Diagnosis: ICD10 Code, J00-J99 Define	1234918	J18.9	Pneumonia, unspecified organism
Diagnosis: ICD10 Code, J00-J99 Define	1237270	J18.9	Pneumonia, unspecified organism
Diagnosis: ICD10 Code, J00-J99 Define	1238805	J18.9	Pneumonia, unspecified organism
Diagnosis: ICD10 Code, J00-J99 Define	1239578	J18.9	Pneumonia, unspecified organism
Diagnosis: ICD10 Code, J00-J99 Define	1239588	J18.9	Pneumonia, unspecified organism
Diagnosis: ICD10 Code, J00-J99 Define	1240737	J18.9	Pneumonia, unspecified organism
Diagnosis: ICD10 Code, J00-J99 Define	1264332	J18.9	Pneumonia, unspecified organism
Diagnosis: ICD10 Code, J00-J99 Define	133451	J18.9	Pneumonia, unspecified organism
Diagnosis: ICD10 Code, J00-J99 Define	1348013	J18.9	Pneumonia, unspecified organism
Diagnosis: ICD10 Code, J00-J99 Define	1355715	J18.9	Pneumonia, unspecified organism
Diagnosis: ICD10 Code, J00-J99 Define	1427809	J18.9	Pneumonia, unspecified organism
Diagnosis: ICD10 Code, J00-J99 Define	1427810	J18.9	Pneumonia, unspecified organism
Diagnosis: ICD10 Code, J00-J99 Define	1427811	J18.9	Pneumonia, unspecified organism
Diagnosis: ICD10 Code, J00-J99 Define	147568	J18.9	Pneumonia, unspecified organism
Diagnosis: ICD10 Code, J00-J99 Define	147709	J18.9	Pneumonia, unspecified organism
Diagnosis: ICD10 Code, J00-J99 Define	147710	J18.9	Pneumonia, unspecified organism
Diagnosis: ICD10 Code, J00-J99 Define	147711	J18.9	Pneumonia, unspecified organism
Diagnosis: ICD10 Code, J00-J99 Define	15788	J18.9	Pneumonia, unspecified organism
Diagnosis: ICD10 Code, J00-J99 Define	15789	J18.9	Pneumonia, unspecified organism
Diagnosis: ICD10 Code, J00-J99 Define	159273	J18.9	Pneumonia, unspecified organism
Diagnosis: ICD10 Code, J00-J99 Define	159274	J18.9	Pneumonia, unspecified organism
Diagnosis: ICD10 Code, J00-J99 Define	159279	J18.9	Pneumonia, unspecified organism
Diagnosis: ICD10 Code, J00-J99 Define	159686	J18.9	Pneumonia, unspecified organism
Diagnosis: ICD10 Code, J00-J99 Define	182833	J18.9	Pneumonia, unspecified organism
Diagnosis: ICD10 Code, J00-J99 Define	189564	J18.9	Pneumonia, unspecified organism
Diagnosis: ICD10 Code, J00-J99 Define	202869	J18.9	Pneumonia, unspecified organism
Diagnosis: ICD10 Code, J00-J99 Define	202870	J18.9	Pneumonia, unspecified organism
Diagnosis: ICD10 Code, J00-J99 Define	202871	J18.9	Pneumonia, unspecified organism
Diagnosis: ICD10 Code, J00-J99 Define	202872	J18.9	Pneumonia, unspecified organism
Diagnosis: ICD10 Code, J00-J99 Define	202873	J18.9	Pneumonia, unspecified organism
Diagnosis: ICD10 Code, J00-J99 Define	205642	J18.9	Pneumonia, unspecified organism
Diagnosis: ICD10 Code, J00-J99 Define	21039	J18.9	Pneumonia, unspecified organism
Diagnosis: ICD10 Code, J00-J99 Define	222413	J18.9	Pneumonia, unspecified organism
Diagnosis: ICD10 Code, J00-J99 Define	223655	J18.9	Pneumonia, unspecified organism
Diagnosis: ICD10 Code, J00-J99 Define	250136	J18.9	Pneumonia, unspecified organism
Diagnosis: ICD10 Code, J00-J99 Define	258083	J18.9	Pneumonia, unspecified organism
Diagnosis: ICD10 Code, J00-J99 Define	258128	J18.9	Pneumonia, unspecified organism
Diagnosis: ICD10 Code, J00-J99 Define	300897	J18.9	Pneumonia, unspecified organism
Diagnosis: ICD10 Code, J00-J99 Define	300930	J18.9	Pneumonia, unspecified organism
Diagnosis: ICD10 Code, J00-J99 Define	300931	J18.9	Pneumonia, unspecified organism
Diagnosis: ICD10 Code, J00-J99 Define	300934	J18.9	Pneumonia, unspecified organism
Diagnosis: ICD10 Code, J00-J99 Define	302420	J18.9	Pneumonia, unspecified organism
Diagnosis: ICD10 Code, J00-J99 Define	302423	J18.9	Pneumonia, unspecified organism
Diagnosis: ICD10 Code, J00-J99 Define	302424	J18.9	Pneumonia, unspecified organism
Diagnosis: ICD10 Code, J00-J99 Define	326616	J18.9	Pneumonia, unspecified organism
Diagnosis: ICD10 Code, J00-J99 Define	326618	J18.9	Pneumonia, unspecified organism
Diagnosis: ICD10 Code, J00-J99 Define	328293	J18.9	Pneumonia, unspecified organism
Diagnosis: ICD10 Code, J00-J99 Define	330140	J18.9	Pneumonia, unspecified organism
Diagnosis: ICD10 Code, J00-J99 Define	379401	J18.9	Pneumonia, unspecified organism
Diagnosis: ICD10 Code, J00-J99 Define	379402	J18.9	Pneumonia, unspecified organism
Diagnosis: ICD10 Code, J00-J99 Define	380516	J18.9	Pneumonia, unspecified organism
Diagnosis: ICD10 Code, J00-J99 Define	387290	J18.9	Pneumonia, unspecified organism
Diagnosis: ICD10 Code, J00-J99 Define	387291	J18.9	Pneumonia, unspecified organism
Diagnosis: ICD10 Code, J00-J99 Define	408591	J18.9	Pneumonia, unspecified organism
Diagnosis: ICD10 Code, J00-J99 Define	408594	J18.9	Pneumonia, unspecified organism
Diagnosis: ICD10 Code, J00-J99 Define	408595	J18.9	Pneumonia, unspecified organism
Diagnosis: ICD10 Code, J00-J99 Define	416560	J18.9	Pneumonia, unspecified organism
Diagnosis: ICD10 Code, J00-J99 Define	425482	J18.9	Pneumonia, unspecified organism
Diagnosis: ICD10 Code, J00-J99 Define	493374	J18.9	Pneumonia, unspecified organism
Diagnosis: ICD10 Code, J00-J99 Define	493376	J18.9	Pneumonia, unspecified organism
Diagnosis: ICD10 Code, J00-J99 Define	496635	J18.9	Pneumonia, unspecified organism
Diagnosis: ICD10 Code, J00-J99 Define	516054	J18.9	Pneumonia, unspecified organism
Diagnosis: ICD10 Code, J00-J99 Define	518564	J18.9	Pneumonia, unspecified organism
Diagnosis: ICD10 Code, J00-J99 Define	523559	J18.9	Pneumonia, unspecified organism
Diagnosis: ICD10 Code, J00-J99 Define	5447	J18.9	Pneumonia, unspecified organism
Diagnosis: ICD10 Code, J00-J99 Define	5456	J18.9	Pneumonia, unspecified organism
Diagnosis: ICD10 Code, J00-J99 Define	80043	J18.9	Pneumonia, unspecified organism
Diagnosis: ICD10 Code, J00-J99 Define	81508	J18.9	Pneumonia, unspecified organism
Diagnosis: ICD10 Code, J00-J99 Define	95567	J18.9	Pneumonia, unspecified organism
Diagnosis: ICD10 Code, J00-J99 Define	95868	J18.9	Pneumonia, unspecified organism
Diagnosis: ICD10 Code, J20 Define	246489	J20.8	Acute bronchitis due to other specified organisms
Diagnosis: ICD10 Code, J20 Define	246490	J20.8	Acute bronchitis due to other specified organisms
Diagnosis: ICD10 Code, J20 Define	256484	J20.8	Acute bronchitis due to other specified organisms
Diagnosis: ICD10 Code, J20 Define	256485	J20.8	Acute bronchitis due to other specified organisms
Diagnosis: ICD10 Code, J20 Define	493182	J20.8	Acute bronchitis due to other specified organisms

Diagnosis: ICD10 Code, J20 Define	493190	J20.8	Acute bronchitis due to other specified organisms
Diagnosis: ICD10 Code, J20 Define	625168	J20.8	Acute bronchitis due to other specified organisms
Diagnosis: ICD10 Code, J20 Define	73544	J20.8	Acute bronchitis due to other specified organisms
Diagnosis: ICD10 Code, J20 Define	1039729	J20.9	Acute bronchitis, unspecified
Diagnosis: ICD10 Code, J20 Define	1137741	J20.9	Acute bronchitis, unspecified
Diagnosis: ICD10 Code, J20 Define	149076	J20.9	Acute bronchitis, unspecified
Diagnosis: ICD10 Code, J20 Define	149362	J20.9	Acute bronchitis, unspecified
Diagnosis: ICD10 Code, J20 Define	20994	J20.9	Acute bronchitis, unspecified
Diagnosis: ICD10 Code, J20 Define	21002	J20.9	Acute bronchitis, unspecified
Diagnosis: ICD10 Code, J20 Define	21003	J20.9	Acute bronchitis, unspecified
Diagnosis: ICD10 Code, J20 Define	21004	J20.9	Acute bronchitis, unspecified
Diagnosis: ICD10 Code, J20 Define	21005	J20.9	Acute bronchitis, unspecified
Diagnosis: ICD10 Code, J20 Define	225042	J20.9	Acute bronchitis, unspecified
Diagnosis: ICD10 Code, J20 Define	235605	J20.9	Acute bronchitis, unspecified
Diagnosis: ICD10 Code, J20 Define	236425	J20.9	Acute bronchitis, unspecified
Diagnosis: ICD10 Code, J20 Define	237092	J20.9	Acute bronchitis, unspecified
Diagnosis: ICD10 Code, J20 Define	237147	J20.9	Acute bronchitis, unspecified
Diagnosis: ICD10 Code, J20 Define	242921	J20.9	Acute bronchitis, unspecified
Diagnosis: ICD10 Code, J20 Define	242922	J20.9	Acute bronchitis, unspecified
Diagnosis: ICD10 Code, J20 Define	242934	J20.9	Acute bronchitis, unspecified
Diagnosis: ICD10 Code, J20 Define	242935	J20.9	Acute bronchitis, unspecified
Diagnosis: ICD10 Code, J20 Define	242936	J20.9	Acute bronchitis, unspecified
Diagnosis: ICD10 Code, J20 Define	245142	J20.9	Acute bronchitis, unspecified
Diagnosis: ICD10 Code, J20 Define	245143	J20.9	Acute bronchitis, unspecified
Diagnosis: ICD10 Code, J20 Define	265346	J20.9	Acute bronchitis, unspecified
Diagnosis: ICD10 Code, J20 Define	299967	J20.9	Acute bronchitis, unspecified
Diagnosis: ICD10 Code, J20 Define	301350	J20.9	Acute bronchitis, unspecified
Diagnosis: ICD10 Code, J20 Define	301351	J20.9	Acute bronchitis, unspecified
Diagnosis: ICD10 Code, J20 Define	301352	J20.9	Acute bronchitis, unspecified
Diagnosis: ICD10 Code, J20 Define	301353	J20.9	Acute bronchitis, unspecified
Diagnosis: ICD10 Code, J20 Define	313122	J20.9	Acute bronchitis, unspecified
Diagnosis: ICD10 Code, J20 Define	331554	J20.9	Acute bronchitis, unspecified
Diagnosis: ICD10 Code, J20 Define	357171	J20.9	Acute bronchitis, unspecified
Diagnosis: ICD10 Code, J20 Define	414917	J20.9	Acute bronchitis, unspecified
Diagnosis: ICD10 Code, J20 Define	482491	J20.9	Acute bronchitis, unspecified
Diagnosis: ICD10 Code, J20 Define	489760	J20.9	Acute bronchitis, unspecified
Diagnosis: ICD10 Code, J20 Define	489761	J20.9	Acute bronchitis, unspecified
Diagnosis: ICD10 Code, J20 Define	5358	J20.9	Acute bronchitis, unspecified
Diagnosis: ICD10 Code, J20 Define	5461	J20.9	Acute bronchitis, unspecified
Diagnosis: ICD10 Code, J20 Define	59614	J20.9	Acute bronchitis, unspecified
Diagnosis: ICD10 Code, J20 Define	59616	J20.9	Acute bronchitis, unspecified
Diagnosis: ICD10 Code, J20 Define	69784	J20.9	Acute bronchitis, unspecified
Diagnosis: ICD10 Code, J20 Define	69785	J20.9	Acute bronchitis, unspecified
Diagnosis: ICD10 Code, J00-J99 Define	327054	J21.8	Acute bronchiolitis due to other specified organisms
Diagnosis: ICD10 Code, J00-J99 Define	110924	J22	Unspecified acute lower respiratory infection
Diagnosis: ICD10 Code, J00-J99 Define	110925	J22	Unspecified acute lower respiratory infection
Diagnosis: ICD10 Code, J00-J99 Define	110929	J22	Unspecified acute lower respiratory infection
Diagnosis: ICD10 Code, J00-J99 Define	1176952	J22	Unspecified acute lower respiratory infection
Diagnosis: ICD10 Code, J00-J99 Define	183057	J22	Unspecified acute lower respiratory infection
Diagnosis: ICD10 Code, J00-J99 Define	183058	J22	Unspecified acute lower respiratory infection
Diagnosis: ICD10 Code, J00-J99 Define	20870	J22	Unspecified acute lower respiratory infection
Diagnosis: ICD10 Code, J00-J99 Define	20871	J22	Unspecified acute lower respiratory infection
Diagnosis: ICD10 Code, J00-J99 Define	20872	J22	Unspecified acute lower respiratory infection
Diagnosis: ICD10 Code, J00-J99 Define	234824	J22	Unspecified acute lower respiratory infection
Diagnosis: ICD10 Code, J00-J99 Define	262321	J22	Unspecified acute lower respiratory infection
Diagnosis: ICD10 Code, J00-J99 Define	338800	J22	Unspecified acute lower respiratory infection
Diagnosis: ICD10 Code, J00-J99 Define	421970	J22	Unspecified acute lower respiratory infection
Diagnosis: ICD10 Code, J00-J99 Define	5566	J22	Unspecified acute lower respiratory infection
Diagnosis: ICD10 Code, J00-J99 Define	327716	J32.9	Chronic sinusitis, unspecified
Diagnosis: ICD10 Code, J00-J99 Define	361342	J32.9	Chronic sinusitis, unspecified
Diagnosis: ICD10 Code, J00-J99 Define	80874	J34.89	Other specified disorders of nose and nasal sinuses
Diagnosis: ICD10 Code, J00-J99 Define	17201	J39.8	Other specified diseases of upper respiratory tract
Diagnosis: ICD10 Code, J00-J99 Define	147085	J40	Bronchitis, not specified as acute or chronic
Diagnosis: ICD10 Code, J00-J99 Define	149362	J40	Bronchitis, not specified as acute or chronic
Diagnosis: ICD10 Code, J00-J99 Define	15321	J40	Bronchitis, not specified as acute or chronic
Diagnosis: ICD10 Code, J00-J99 Define	182105	J40	Bronchitis, not specified as acute or chronic
Diagnosis: ICD10 Code, J00-J99 Define	208620	J40	Bronchitis, not specified as acute or chronic
Diagnosis: ICD10 Code, J00-J99 Define	21001	J40	Bronchitis, not specified as acute or chronic
Diagnosis: ICD10 Code, J00-J99 Define	258162	J40	Bronchitis, not specified as acute or chronic
Diagnosis: ICD10 Code, J00-J99 Define	331436	J40	Bronchitis, not specified as acute or chronic
Diagnosis: ICD10 Code, J00-J99 Define	331437	J40	Bronchitis, not specified as acute or chronic
Diagnosis: ICD10 Code, J00-J99 Define	380522	J40	Bronchitis, not specified as acute or chronic
Diagnosis: ICD10 Code, J00-J99 Define	385287	J40	Bronchitis, not specified as acute or chronic
Diagnosis: ICD10 Code, J00-J99 Define	412292	J40	Bronchitis, not specified as acute or chronic
Diagnosis: ICD10 Code, J00-J99 Define	420003	J40	Bronchitis, not specified as acute or chronic
Diagnosis: ICD10 Code, J00-J99 Define	420865	J40	Bronchitis, not specified as acute or chronic
Diagnosis: ICD10 Code, J00-J99 Define	69933	J40	Bronchitis, not specified as acute or chronic
Diagnosis: ICD10 Code, J00-J99 Define	73574	J40	Bronchitis, not specified as acute or chronic
Diagnosis: ICD10 Code, J00-J99 Define	73575	J40	Bronchitis, not specified as acute or chronic
Diagnosis: ICD10 Code, J00-J99 Define	75946	J40	Bronchitis, not specified as acute or chronic
Diagnosis: ICD10 Code, J00-J99 Define	79473	J40	Bronchitis, not specified as acute or chronic
Diagnosis: ICD10 Code, J00-J99 Define	1101940	J44.0	Chronic obstructive pulmonary disease with acute lower respiratory infection
Diagnosis: ICD10 Code, J00-J99 Define	1304957	J44.0	Chronic obstructive pulmonary disease with acute lower respiratory infection
Diagnosis: ICD10 Code, J00-J99 Define	1305126	J44.0	Chronic obstructive pulmonary disease with acute lower respiratory infection
Diagnosis: ICD10 Code, J00-J99 Define	1366597	J44.0	Chronic obstructive pulmonary disease with acute lower respiratory infection
Diagnosis: ICD10 Code, J00-J99 Define	164899	J44.0	Chronic obstructive pulmonary disease with acute lower respiratory infection
Diagnosis: ICD10 Code, J00-J99 Define	340146	J44.0	Chronic obstructive pulmonary disease with acute lower respiratory infection
Diagnosis: ICD10 Code, J00-J99 Define	136187	J44.1	Chronic obstructive pulmonary disease with (acute) exacerbation
Diagnosis: ICD10 Code, J00-J99 Define	136187	J44.9	Chronic obstructive pulmonary disease, unspecified
Diagnosis: ICD10 Code, J00-J99 Define	149363	J44.9	Chronic obstructive pulmonary disease, unspecified
Diagnosis: ICD10 Code, J00-J99 Define	1214845	J45.21	Mild intermittent asthma with (acute) exacerbation
Diagnosis: ICD10 Code, J00-J99 Define	1304196	J45.21	Mild intermittent asthma with (acute) exacerbation
Diagnosis: ICD10 Code, J00-J99 Define	489762	J45.51	Severe persistent asthma with (acute) exacerbation
Diagnosis: ICD10 Code, J00-J99 Define	408592	J69.0	Pneumonitis due to inhalation of food and vomit
Diagnosis: ICD10 Code, J00-J99 Define	1194661	J84.09	Other alveolar and parieto-alveolar conditions

Diagnosis: ICD10 Code, J00-J99 Define	5543	J84.09	Other alveolar and parieto-alveolar conditions
Diagnosis: ICD10 Code, J00-J99 Define	5544	J84.09	Other alveolar and parieto-alveolar conditions
Diagnosis: ICD10 Code, J00-J99 Define	254962	J84.10	Pulmonary fibrosis, unspecified
Diagnosis: ICD10 Code, J00-J99 Define	314062	J84.112	Idiopathic pulmonary fibrosis
Diagnosis: ICD10 Code, J00-J99 Define	314064	J84.112	Idiopathic pulmonary fibrosis
Diagnosis: ICD10 Code, J00-J99 Define	110803	J84.2	Lymphoid interstitial pneumonia
Diagnosis: ICD10 Code, J00-J99 Define	111003	J84.2	Lymphoid interstitial pneumonia
Diagnosis: ICD10 Code, J00-J99 Define	21341	J84.2	Lymphoid interstitial pneumonia
Diagnosis: ICD10 Code, J00-J99 Define	21342	J84.2	Lymphoid interstitial pneumonia
Diagnosis: ICD10 Code, J00-J99 Define	1192461	J84.89	Other specified interstitial pulmonary diseases
Diagnosis: ICD10 Code, J00-J99 Define	155851	J84.89	Other specified interstitial pulmonary diseases
Diagnosis: ICD10 Code, J00-J99 Define	155854	J84.89	Other specified interstitial pulmonary diseases
Diagnosis: ICD10 Code, J00-J99 Define	156637	J84.89	Other specified interstitial pulmonary diseases
Diagnosis: ICD10 Code, J00-J99 Define	156638	J84.89	Other specified interstitial pulmonary diseases
Diagnosis: ICD10 Code, J00-J99 Define	156639	J84.89	Other specified interstitial pulmonary diseases
Diagnosis: ICD10 Code, J00-J99 Define	156640	J84.89	Other specified interstitial pulmonary diseases
Diagnosis: ICD10 Code, J00-J99 Define	312728	J84.89	Other specified interstitial pulmonary diseases
Diagnosis: ICD10 Code, J00-J99 Define	312729	J84.89	Other specified interstitial pulmonary diseases
Diagnosis: ICD10 Code, J00-J99 Define	313717	J84.89	Other specified interstitial pulmonary diseases
Diagnosis: ICD10 Code, J00-J99 Define	313718	J84.89	Other specified interstitial pulmonary diseases
Diagnosis: ICD10 Code, J00-J99 Define	5543	J84.89	Other specified interstitial pulmonary diseases
Diagnosis: ICD10 Code, J00-J99 Define	1137823	J84.9	Interstitial pulmonary disease, unspecified
Diagnosis: ICD10 Code, J00-J99 Define	124761	J84.9	Interstitial pulmonary disease, unspecified
Diagnosis: ICD10 Code, J00-J99 Define	148518	J84.9	Interstitial pulmonary disease, unspecified
Diagnosis: ICD10 Code, J00-J99 Define	21334	J84.9	Interstitial pulmonary disease, unspecified
Diagnosis: ICD10 Code, J00-J99 Define	21339	J84.9	Interstitial pulmonary disease, unspecified
Diagnosis: ICD10 Code, J00-J99 Define	254961	J84.9	Interstitial pulmonary disease, unspecified
Diagnosis: ICD10 Code, J00-J99 Define	254962	J84.9	Interstitial pulmonary disease, unspecified
Diagnosis: ICD10 Code, J00-J99 Define	312718	J84.9	Interstitial pulmonary disease, unspecified
Diagnosis: ICD10 Code, J00-J99 Define	312719	J84.9	Interstitial pulmonary disease, unspecified
Diagnosis: ICD10 Code, J00-J99 Define	312721	J84.9	Interstitial pulmonary disease, unspecified
Diagnosis: ICD10 Code, J00-J99 Define	312726	J84.9	Interstitial pulmonary disease, unspecified
Diagnosis: ICD10 Code, J00-J99 Define	312727	J84.9	Interstitial pulmonary disease, unspecified
Diagnosis: ICD10 Code, J00-J99 Define	5544	J84.9	Interstitial pulmonary disease, unspecified
Diagnosis: ICD10 Code, J00-J99 Define	65330	J84.9	Interstitial pulmonary disease, unspecified
Diagnosis: ICD10 Code, J00-J99 Define	189564	J94.8	Other specified pleural conditions
Diagnosis: ICD10 Code, J00-J99 Define	373785	J98.01	Acute bronchospasm
Diagnosis: ICD10 Code, J00-J99 Define	110924	J98.8	Other specified respiratory disorders
Diagnosis: ICD10 Code, J00-J99 Define	110925	J98.8	Other specified respiratory disorders
Diagnosis: ICD10 Code, J00-J99 Define	110929	J98.8	Other specified respiratory disorders
Diagnosis: ICD10 Code, J00-J99 Define	114086	J98.8	Other specified respiratory disorders
Diagnosis: ICD10 Code, J00-J99 Define	1192484	J98.8	Other specified respiratory disorders
Diagnosis: ICD10 Code, J00-J99 Define	157387	J98.8	Other specified respiratory disorders
Diagnosis: ICD10 Code, J00-J99 Define	17802	J98.8	Other specified respiratory disorders
Diagnosis: ICD10 Code, J00-J99 Define	183057	J98.8	Other specified respiratory disorders
Diagnosis: ICD10 Code, J00-J99 Define	183058	J98.8	Other specified respiratory disorders
Diagnosis: ICD10 Code, J00-J99 Define	20870	J98.8	Other specified respiratory disorders
Diagnosis: ICD10 Code, J00-J99 Define	20871	J98.8	Other specified respiratory disorders
Diagnosis: ICD10 Code, J00-J99 Define	20872	J98.8	Other specified respiratory disorders
Diagnosis: ICD10 Code, J00-J99 Define	246922	J98.8	Other specified respiratory disorders
Diagnosis: ICD10 Code, J00-J99 Define	246923	J98.8	Other specified respiratory disorders
Diagnosis: ICD10 Code, J00-J99 Define	258750	J98.8	Other specified respiratory disorders
Diagnosis: ICD10 Code, J00-J99 Define	258751	J98.8	Other specified respiratory disorders
Diagnosis: ICD10 Code, J00-J99 Define	328733	J98.8	Other specified respiratory disorders
Diagnosis: ICD10 Code, J00-J99 Define	330691	J98.8	Other specified respiratory disorders
Diagnosis: ICD10 Code, J00-J99 Define	330692	J98.8	Other specified respiratory disorders
Diagnosis: ICD10 Code, J00-J99 Define	338800	J98.8	Other specified respiratory disorders
Diagnosis: ICD10 Code, J00-J99 Define	417563	J98.8	Other specified respiratory disorders
Diagnosis: ICD10 Code, J00-J99 Define	417582	J98.8	Other specified respiratory disorders
Diagnosis: ICD10 Code, J00-J99 Define	418375	J98.8	Other specified respiratory disorders
Diagnosis: ICD10 Code, J00-J99 Define	421970	J98.8	Other specified respiratory disorders
Diagnosis: ICD10 Code, J00-J99 Define	425769	J98.8	Other specified respiratory disorders
Diagnosis: ICD10 Code, J00-J99 Define	494015	J98.8	Other specified respiratory disorders
Diagnosis: ICD10 Code, J00-J99 Define	55528	J98.8	Other specified respiratory disorders
Diagnosis: ICD10 Code, J00-J99 Define	55529	J98.8	Other specified respiratory disorders
Diagnosis: ICD10 Code, J00-J99 Define	55530	J98.8	Other specified respiratory disorders
Diagnosis: ICD10 Code, J00-J99 Define	5566	J98.8	Other specified respiratory disorders
Diagnosis: ICD10 Code, J00-J99 Define	379577	J98.9	Respiratory disorder, unspecified
Diagnosis: ICD10 Code, J00-J99 Define	379578	J98.9	Respiratory disorder, unspecified
Diagnosis: ICD10 Code, J00-J99 Define	420391	J98.9	Respiratory disorder, unspecified
Diagnosis: ICD10 Code, J00-J99 Define	420392	J98.9	Respiratory disorder, unspecified

CPT codes

**Chart Category**	**PX_CD**	**PX_CD_N**	**PX_CD_TYPE**	**PX_PRIMARY_YN**
Billing CPT Code: 99284 -Define	99284	NON EMI	CPT4	0
Billing CPT Code: 71020 - Define	71020	CHEST 2V	CPT4	0
Billing CPT Code: 3008F - Define	3008F	BODY MA	CPT4	0
Billing CPT Code: 99283 - Define	99283	NON EMI	CPT4	0

LISTS FROM WHICH ABOVE WAS SELECTED

2021 ICD-10-CM Codes

A00-B99 Certain infectious and parasitic diseases
C00-D49 Neoplasms
D50-D89 Diseases of the blood and blood-forming organs and certain disorders involving the immune mechanism
E00-E89 Endocrine, nutritional and metabolic diseases
F01-F99 Mental, Behavioral and Neurodevelopmental disorders
G00-G99 Diseases of the nervous system
H00-H59 Diseases of the eye and adnexa
H60-H95 Diseases of the ear and mastoid process
I00-I99 Diseases of the circulatory system
J00-J99 Diseases of the respiratory system

LISTS FROM WHICH ABOVE WAS SELECTED

Eosinophils # (is this % or absolute?)	0.6
Drug ATC_S Define	0.6
Temperature values	0.6
Drugs for severe asthma	0.6
Admission values	0.7
Diagnosis of asthma	0.7
Diagnosis: ICD10 Code, F00-F99 Define	0.7
Temperature trends	0.8
Admission ED OR Preop IP GCMC	0.9
Drug (ATC_R03A). Adrenergic medicatons and inhalants	1.0

K00-K95 Diseases of the digestive system	Gender	1.0
L00-L99 Diseases of the skin and subcutaneous tissue	Billing CPT Code: 99284 -Define	1.1
M00-M99 Diseases of the musculoskeletal system and connective tissue	SpO2 values (high or low?)	1.1
N00-N99 Diseases of the genitourinary system	Billing CPT Code: 71020 - Define	1.2
O00-O9A Pregnancy, childbirth and the puerperium	Congenital Adrenal Hyperplasia	1.4
P00-P96 Certain conditions originating in the perinatal period	Diagnosis: ICD10 Code, I00-I99, Define	1.7
Q00-Q99 Congenital malformations, deformations and chromosomal abnormalities	Billing CPT Code: 3008F - Define	1.8
R00-R99 Symptoms, signs and abnormal clinical and laboratory findings, not elsewhere classified	Vaccination status	1.8
S00-T88 Injury, poisoning and certain other consequences of external causes	Drug (ATC_R). Drugs? Respiratory system	2.3
U00-U85 Codes for special purposes	Weight	2.5
V00-Y99 External causes of morbidity	Smoking	2.5
Z00-Z99 Factors influencing health status and contact with health ser	Billing CPT Code: 99283 - Define	2.5
	Drug ATC_J01, Antibacterial medication	2.9
	Diagnosis: ICD10 Code, J20 Define	2.9
	Age	3.3
	Drug (ATC_A), Medication for alimentary tract illness and metabolism	3.4
	Flu Registry	4.1
	Complications	6.4
	Membership	8.4
	Diagnosis: ICD10 Code, J00-J99 Define	8.7

Supplementary Table S3 (Feature contribution) Feature

Diagnosis: ICD10 Code, J00-J99 Define	Value
Membership	8.7
Complications	8.4
Flu Registry	6.4
Drug (ATC_A), Medication for alimentary tract illness a	4.1
Age	3.4
Drug ATC_J01, Antibacterial medication	3.3
Diagnosis: ICD10 Code, J20 Define	2.9
Weight	2.9
Smoking	2.5
Billing CPT Code: 99283 - Define	2.5
Drug (ATC_R). Drugs? Respiratory system	2.5
Billing CPT Code: 3008F - Define	2.3
Vaccination status	1.8
Diagnosis: ICD10 Code, I00-I99, Define	1.8
Congenital Adrenal Hyperplasia	1.7
Billing CPT Code: 71020 - Define	1.4
Billing CPT Code: 99284 -Define	1.2
SpO2 values (high or low?)	1.1
Drug (ATC_R03A). Adrenergic medicatons and inhalan	1.1
Gender	1.0
Admission ED OR Preop IP GCMC	1.0
Temperature trends	0.9
Admission values	0.8
Diagnosis of asthma	0.7
Diagnosis: ICD10 Code, F00-F99 Define	0.7
Eosinophils # (is this % or absolute?)	0.7
Drug ATC_S Define	0.6
Temperature values	0.6
Drugs for severe asthma	0.6
	0.6

Supplementary Table S3 (2021 ICD-10-CM Codes)

A00-B99 Certain infectious and parasitic diseases
C00-D49 Neoplasms
D50-D89 Diseases of the blood and blood-forming organs and certain disorders involving the immune mechanism
E00-E89 Endocrine, nutritional and metabolic diseases
F01-F99 Mental, Behavioral and Neurodevelopmental disorders
G00-G99 Diseases of the nervous system
H00-H59 Diseases of the eye and adnexa
H60-H95 Diseases of the ear and mastoid process
I00-I99 Diseases of the circulatory system
J00-J99 Diseases of the respiratory system
K00-K95 Diseases of the digestive system
L00-L99 Diseases of the skin and subcutaneous tissue
M00-M99 Diseases of the musculoskeletal system and connective tissue
N00-N99 Diseases of the genitourinary system
O00-O9A Pregnancy, childbirth and the puerperium
P00-P96 Certain conditions originating in the perinatal period
Q00-Q99 Congenital malformations, deformations and chromosomal abnormalities
R00-R99 Symptoms, signs and abnormal clinical and laboratory findings, not elsewhere classified
S00-T88 Injury, poisoning and certain other consequences of external causes
U00-U85 Codes for special purposes
V00-Y99 External causes of morbidity
Z00-Z99 Factors influencing health status and contact with health ser

Supplementary Table S4 (Tiers of Confidence for Influenza Diagnosis and Levels of Severity for Influenza-complications)

Outcomes	Cases				Controls
Severity Levels of Complications	**Tier 1<br>Influenza RT-PCR +<br>*AND* Influenza ICD code**	Tier 1<br>Influenza RT-PCR +	Tier 1 ICD-10 plus<br>Antiviral Medication	**Tier 2<br>ILI *OR* Respiratory disease**	**Tier 3<br>Mild *OR* No influenza**
**Level 1 Complication Tier 1 Death**	Laboratory Registry +/ ICD code	Laboratory Registry	Phenomic Registry	ILI Subgroup	Control group
**Level 2 Complication Tier 2 Hospitalization or ED visit**	Laboratory Registry +/ ICD code	Laboratory Registry	Phenomic Registry	ILI Subgroup	Control group
**Level 3 Complication Tier 3 Severe illness**	Laboratory Registry +/ ICD code	Laboratory Registry	Phenomic Registry	ILI Subgroup	Control group

Supplementary Table S5 [Steps in deployment for the rule set that informed data filtering for the model(s)]

1. When the ratio between the descender count and the ascender count is similar, only the ascender code is retained, due to the preference for using the more granular codes. For example, if a descender has 10,000 examples (and an ascender has 10,100 examples, the additional 100 examples represent only 1% of the data; therefore, the ascender was eliminated. The control threshold for this process was set to 5%.

2. When the ascender "behaves" differently from at least 2 descenders, such that aggregation of the descender into the ascender might be unreasonable, the ascender was removed. The occurs with different enough statistical properties of p_value and average lift. For example, when parameters equal [filter_child_pval_diff (default value is 1e-10) + filter_child_lift_ratio]. The default value was set to 0.05. When both the p_value difference between ascender and descender is below filter_child_pval_diff AND the difference in average lift is below filter_child_lift_ratio.

3. When a node had a descender that passed the above filters and a descender that did not pass and was removed,

if the aggregated sum of the removed examples is high, removal of ascender is considered. In other words, if the ascender contains descenders with different enough meanings, then it makes less sense to use them together. For example, if an ascender that has 10,000 examples and descenders with 8,000 examples were filtered, then the ascender is left with descenders that consist with 2,000 examples. In this scenario, it would make no sense to use a more granular ascender code that would result in 80% of examples being filtered.

Supplementary Table S6 (Data filtering that occurred according to a rule set, ordered in the following stepwise manner)

model_parameters	auc_test	auc_on_train
xgb: tree_method=auto; booster=gbtree; objective=binary:logistic; eta=0.100; alpha=0.000; lambda=0.100; gamma=0.100; max_depth=7; colsample_bytree=1.000; colsample_bylevel=0.800; min_child_weight=10; num_round=100; subsample=0.700	-0.7807	-0.81
xgb: tree_method=auto; booster=gbtree; objective=binary:logistic; eta=0.100; alpha=0.000; lambda=0.100; gamma=0.100; max_depth=6; colsample_bytree=1.000; colsample_bylevel=0.800; min_child_weight=10; num_round=100; subsample=0.700	-0.7805	-0.8
xgb: tree_method=auto; booster=gbtree; objective=binary:logistic; eta=0.100; alpha=0.000; lambda=0.100; gamma=0.100; max_depth=5; colsample_bytree=1.000; colsample_bylevel=0.800; min_child_weight=10; num_round=100; subsample=0.700	-0.7792	-0.79
xgb: tree_method=auto; booster=gbtree; objective=binary:logistic; eta=0.300; alpha=0.000; lambda=0.100; gamma=0.100; max_depth=4; colsample_bytree=1.000; colsample_bylevel=0.800; min_child_weight=10; num_round=100; subsample=0.700	-0.7781	-0.8
xgb: tree_method=auto; booster=gbtree; objective=binary:logistic; eta=0.100; alpha=0.000; lambda=0.100; gamma=0.100; max_depth=4; colsample_bytree=1.000; colsample_bylevel=0.800; min_child_weight=10; num_round=100; subsample=0.700	-0.7773	-0.78
xgb: tree_method=auto; booster=gbtree; objective=binary:logistic; eta=0.050; alpha=0.000; lambda=0.100; gamma=0.100; max_depth=7; colsample_bytree=1.000; colsample_bylevel=0.800; min_child_weight=10; num_round=100; subsample=0.700	-0.777	-0.8
xgb: tree_method=auto; booster=gbtree; objective=binary:logistic; eta=0.300; alpha=0.000; lambda=0.100; gamma=0.100; max_depth=5; colsample_bytree=1.000; colsample_bylevel=0.800; min_child_weight=10; num_round=100; subsample=0.700	-0.7763	-0.81
xgb: tree_method=auto; booster=gbtree; objective=binary:logistic; eta=0.050; alpha=0.000; lambda=0.100; gamma=0.100; max_depth=6; colsample_bytree=1.000; colsample_bylevel=0.800; min_child_weight=10; num_round=100; subsample=0.700	-0.7753	-0.79
xgb: tree_method=auto; booster=gbtree; objective=binary:logistic; eta=0.050; alpha=0.000; lambda=0.100; gamma=0.100; max_depth=5; colsample_bytree=1.000; colsample_bylevel=0.800; min_child_weight=10; num_round=100; subsample=0.700	-0.7731	-0.78
xgb: tree_method=auto; booster=gbtree; objective=binary:logistic; eta=0.300; alpha=0.000; lambda=0.100; gamma=0.100; max_depth=6; colsample_bytree=1.000; colsample_bylevel=0.800; min_child_weight=10; num_round=100; subsample=0.700	-0.7727	-0.82
xgb: tree_method=auto; booster=gbtree; objective=binary:logistic; eta=0.300; alpha=0.000; lambda=0.100; gamma=0.100; max_depth=7; colsample_bytree=1.000; colsample_bylevel=0.800; min_child_weight=10; num_round=100; subsample=0.700	-0.7699	-0.83
xgb: tree_method=auto; booster=gbtree; objective=binary:logistic; eta=0.050; alpha=0.000; lambda=0.100; gamma=0.100; max_depth=4; colsample_bytree=1.000; colsample_bylevel=0.800; min_child_weight=10; num_round=100; subsample=0.700	-0.7692	-0.77

Without Weights (on best cohort definition):

model_name	AUC_Mea	AUC_Mean_on_train
xgb:tree_method=auto;booster=gbtree;objective=binary:logistic;eta=0.100;alpha=0.000;lambda=0.100;gamma=0.100;max_depth=7;colsample_bytree=1.000;colsample_bylevel=0.800;min_child_weight=10;num_round=100;subsample=0.700	-0.7794	-0.81
xgb:tree_method=auto;booster=gbtree;objective=binary:logistic;eta=0.100;alpha=0.000;lambda=0.100;gamma=0.100;max_depth=6;colsample_bytree=1.000;colsample_bylevel=0.800;min_child_weight=10;num_round=100;subsample=0.700	-0.7792	-0.8
xgb:tree_method=auto;booster=gbtree;objective=binary:logistic;eta=0.100;alpha=0.000;lambda=0.100;gamma=0.100;max_depth=5;colsample_bytree=1.000;colsample_bylevel=0.800;min_child_weight=10;num_round=100;subsample=0.700	-0.7779	-0.79
xgb:tree_method=auto;booster=gbtree;objective=binary:logistic;eta=0.300;alpha=0.000;lambda=0.100;gamma=0.100;max_depth=4;colsample_bytree=1.000;colsample_bylevel=0.800;min_child_weight=10;num_round=100;subsample=0.700	-0.7758	-0.8
xgb:tree_method=auto;booster=gbtree;objective=binary:logistic;eta=0.050;alpha=0.000;lambda=0.100;gamma=0.100;max_depth=7;colsample_bytree=1.000;colsample_bylevel=0.800;min_child_weight=10;num_round=100;subsample=0.700	-0.7757	-0.8
xgb:tree_method=auto;booster=gbtree;objective=binary:logistic;eta=0.100;alpha=0.000;lambda=0.100;gamma=0.100;max_depth=4;colsample_bytree=1.000;colsample_bylevel=0.800;min_child_weight=10;num_round=100;subsample=0.700	-0.7755	-0.78
xgb:tree_method=auto;booster=gbtree;objective=binary:logistic;eta=0.300;alpha=0.000;lambda=0.100;gamma=0.100;max_depth=5;colsample_bytree=1.000;colsample_bylevel=0.800;min_child_weight=10;num_round=100;subsample=0.700	-0.7748	-0.81
xgb:tree_method=auto;booster=gbtree;objective=binary:logistic;eta=0.050;alpha=0.000;lambda=0.100;gamma=0.100;max_depth=6;colsample_bytree=1.000;colsample_bylevel=0.800;min_child_weight=10;num_round=100;subsample=0.700	-0.7741	-0.79
xgb:tree_method=auto;booster=gbtree;objective=binary:logistic;eta=0.050;alpha=0.000;lambda=0.100;gamma=0.100;max_depth=5;colsample_bytree=1.000;colsample_bylevel=0.800;min_child_weight=10;num_round=100;subsample=0.700	-0.7716	-0.78
xgb:tree_method=auto;booster=gbtree;objective=binary:logistic;eta=0.300;alpha=0.000;lambda=0.100;gamma=0.100;max_depth=6;colsample_bytree=1.000;colsample_bylevel=0.800;min_child_weight=10;num_round=100;subsample=0.700	-0.7714	-0.82
xgb:tree_method=auto;booster=gbtree;objective=binary:logistic;eta=0.050;alpha=0.000;lambda=0.100;gamma=0.100;max_depth=4;colsample_bytree=1.000;colsample_bylevel=0.800;min_child_weight=10;num_round=100;subsample=0.700	-0.7682	-0.77
xgb:tree_method=auto;booster=gbtree;objective=binary:logistic;eta=0.300;alpha=0.000;lambda=0.100;gamma=0.100;max_depth=7;colsample_bytree=1.000;colsample_bylevel=0.800;min_child_weight=10;num_round=100;subsample=0.700	-0.7676	-0.83

Supplementary Table S7 (XGBoost parameter tests and results)

Measurement	Logistic
AUC	0.687[0.685 - 0.690]
PPV@SENS_10	18.05[17.61 - 18.64]
PPV@SENS_15	16.13[15.74 - 16.56]
PPV@SENS_20	14.82[14.51 - 15.18]
PPV@SENS_25	14.04[13.82 - 14.31]
PPV@SENS_30	13.12[12.90 - 13.39]
PPV@SENS_35	12.36[12.17 - 12.58]
PPV@SENS_40	11.59[11.39 - 11.80]
PPV@SENS_45	10.75[10.57 - 10.95]
PPV@SENS_50	9.93[9.78 - 10.08]
PPV@SENS_60	8.81[8.70 - 8.93]
PPV@SENS_70	7.73[7.63 - 7.83]
PPV@SENS_80	6.68[6.60 - 6.77]
SENS@FPR_01	4.99[4.81 - 5.21]
SENS@FPR_05	17.69[17.38 - 18.04]
SENS@FPR_10	29.91[29.51 - 30.36]
SENS@FPR_15	39.29[38.88 - 39.77]
SENS@FPR_20	46.27[45.89 - 46.66]
SENS@FPR_30	58.59[58.23 - 58.98]
SENS@FPR_40	68.07[67.72 - 68.43]
SENS@FPR_50	75.89[75.56 - 76.21]

Supplementary Table S8 (Attributes of Full Gflu-Cx Flag Model and MES Minimal Model)

Gflu-Cx Flag Full Model				
**Demographic**	**Behavioural**	**Clinical**	**Vital Signs**	**Laboratory Test Results**
Membership coverage	Smoking	Diagnosis	Blood pressure	Flu RT PCR
Age		Medications	Temperture	Acetaminophen
Gender		Admissions	SpO2	Albumin
Race		Hospital transfers	FiO2	ALKP
ETHNICITY		admission reason		ALT
Weight		outpatient visits		Amylase
BMI		Procedures		aPTT
		Billing codes		AST
		Flu vaccination		B12
				Bands#
				Bands%
				Base_excess
				Basophils#
				Basophils%
				Bicarbonate
				Bilirubin
				Bilirubin_Direct
				BNP
				BUN
				C3
				C4
				Ca
				CA125
				CA199
				CEA
				Chloride
				Cholesterol
				Cholesterol_over_HDL
				CK
				CK_MB_INDEX
				Cl

CO2
Cortisol
Creatine_Kinase_MB
Creatinine
CRP
D_DIMER
Digoxin
eGFR
Eosinophils#
Eosinophils%
ESR
Ferritin
Fibrinogen
freeCalcium
FreeT3
FreeT4
FSH
GFR
GGT
Globulin
Glucose
Haptoglobin
HbA1C
HDL
Hematocrit
Hemoglobin
IgA
IgG
IgM
Immature_Granulocytes#
Immature_Granulocytes%
INR
Iron_Binding_Capacity
Iron_Fe

Lactate
LDH
LDL
Lipase
Lithium
LuteinisingHormone
Lymphocytes#
Lymphocytes%
MCH
MCHC
MCV
Metamyelocytes#
Metamyelocytes%
Mg
Monocytes#
Monocytes%
MPV
Myelocytes#
Myelocytes%
Na
Neutrophils#
Neutrophils%
NonHDLCholesterol
NRBC%
Osmolality
PCO2
pH
Phenytoin
Phosphore
Platelets
PO2
Potassium
Progesterone
Prolactin

Protein_Total
PSA
PTH
RBC
RDW
Reticulocytes#
Reticulocytes%
SaO2
SerumAnionGap
T4
Testosterone
Transferrin
Transferrin_Saturation_Index
Triglycerides
TroponinI
TroponinT
TSH
Uric_Acid
UrineAlbumin_over_Creatinine
Urine_Creatinine
Urine_Microalbumin
Urine_Osmolality
Urine_PH
Urine_Spec_Gravity
VitaminD_25
WBC

MES Flu Algomarker, Minimal

**Demographic**	**Behavioural**	**Clinical**
MEMBERSHIP	Smoking	Diagnosis
Sex/Gender		Medications
Age		Admissions
Weight		
BMI		

Supplementary Table S9 (Bias Analysis using a simple age cutoff to classify individuals > 65 yrs. as high-risk, by each attribute of interest.)

Group (% of total population)	Sensitivity [95% CI]	Effect of matching	Aged 65+ "Model" Sensitivity [95% CI]
Race[1]			
White (92.6%)	43.1 [42.4 - 43.9]	-	16.68 [16.08 - 17.36]
Black (5.3%)	38.8 [35.3 - 42.3]*	Mitigated after matching for age	3.86 [2.58 - 5.21]*
Asians (1%)	27.6 [16.7 - 39.8]*	Maintained after matching for age	9.27 [2.99 - 16.67]
Ethnicity			
Hispanic / LA (5.4%)	46.5 [43.3 - 50.0]	-	3.22 [2.11 - 4.52]
Non-Hispanic / LA (94%)	42.5 [41.7 - 43.3]*	Mitigated after matching for age	16.59 [15.92 - 17.25]*
Insurance Type[1]			
Medicaid[2] (36.1%)	49.5 [48.4 - 50.5]	-	2.79 [2.41 - 3.25]
Medicare (14.5%)	52.0 [50.4 - 53.7]*	Reversed after matching for age	70.59 [68.83 - 72.37]*
Commercial (48.5%)	28.1 [27.0 - 29.3]*	Maintained after matching for age	4.32 [3.75 - 4.98]*
Sex			
Female (53.3%)	45.0 [44.2 - 45.9]	-	16.63 [15.82 - 17.48]
Male (46.7%)	39.7 [38.5 - 41.0]*	Mitigated after matching for age and number of visits	15.12 [14.20 - 16.15]

*Significantly different at $p < 0.05$ compared to reference category, always listed first.

CI = Confidence Interval; LA = Latin American

[1]Other race and insurer categories exist but each compose less than 1% of the population.

[2]Patients enrolled in Medicaid at any point in the last 11 years were placed in this category, even if they later shifted insurance (e.g. aged into Medicare).

Supplementary Table S10 [Medical Subject Headings (MeSH) used in literature search]

**Inclusion MeSH Terms**		**Exclusion MeSH Terms**
All languages:		Pediatric
Influneza	Influenza, Human	Children
Vaccine	Influenza A Virus	SARS-CoV-2
Vaccinations	Orthomyxovirus Type A, Human	COVID19
Vaccine Hesitancy	Myxovirus influenzae-A hominis	
Risk-Stratification		
Machine Learning		
Artificial Intelligence		
Xgboost		
Clinical decision		
Support systems		
EHR		
Electronic Health Record		
Medical record		
Health informatics		

Supplementary Table S11 (Calculations used for impact analysis)

Category	Population 2020	% top-risk selected for intervention @ Geisinger	At-risk people identified	Lay terms
G-primary care	641,000	10%	64,100	64 thousand
Pennsylvania	13,002,700	10%	1,300,270	1.3 million
United States	331,449,300	10%	33,144,930	33 million
World-wide (Global)	7,800,000,000	10%	780,000,000	780 million

Supplementary Table 11

SuppT11 includes calculations and references for calculating burden and impact of the Geisinger Flu-Flag Model were it used in practice to identify those at high risk of post-influenza flu complications, in an attempt to reduce vaccine hesitancy.

Assumptions, 2020

1 billion = 1,000,000,000		
globe = 7.8 billion	7,794,798,739	https://www.macrotrends.net/countries/WLD/world/population-growth-rate#
US = 331.5 million	331,449,281	https://en.wikipedia.org/wiki/2020_United_States_census
Pennsylvania = 13 million	13,002,700	https://www.porh.psu.edu/quick-stats-2020-census-pennsylvania-data-changes/
